# High-throughput AR dimerization assay identifies androgen disrupting chemicals and metabolites

**DOI:** 10.3389/ftox.2023.1134783

**Published:** 2023-04-04

**Authors:** Evan C. Brown, Daniel R. Hallinger, Steven O. Simmons

**Affiliations:** ^1^ Oak Ridge Institute for Science Education Fellow, Research Triangle Park, NC, United States; ^2^ Rapid Assay Development Branch, Biomolecular and Computational Toxicology Division, Center for Computational Toxicology and Exposure, Office of Research and Development, U. S. Environmental Protection Agency, Research Triangle Park, NC, United States

**Keywords:** androgen receptor, high-throughput (HT) screening, endocrine, metabolism, CYP450, new approach methodologies (NAMs), agonist, antagonist

## Abstract

**Introduction:** Analysis of streamlined computational models used to predict androgen disrupting chemicals revealed that assays measuring androgen receptor (AR) cofactor recruitment/dimerization were particularly indispensable to high predictivity, especially for AR antagonists. As the original dimerization assays used to develop the minimal assay models are no longer available, new assays must be established and evaluated as suitable alternatives to assess chemicals beyond the original 1,800+ supported by the current data. Here we present the AR2 assay, which is a stable, cell-based method that uses an enzyme complementation approach.

**Methods:** Bipartite domains of the NanoLuc luciferase enzyme were fused to the human AR to quantitatively measure ligand-dependent AR homodimerization. 128 chemicals with known endocrine activity profiles including 43 AR reference chemicals were screened in agonist and antagonist modes and compared to the legacy assays. Test chemicals were rescreened in both modes using a retrofit method to incorporate robust cytochrome P450 (CYP) metabolism to assess CYP-mediated shifts in bioactivity.

**Results:** The AR2 assay is amenable to high-throughput screening with excellent robust Z’-factors (rZ’) for both agonist (0.94) and antagonist (0.85) modes. The AR2 assay successfully classified known agonists (balanced accuracy = 0.92) and antagonists (balanced accuracy = 0.79–0.88) as well as or better than the legacy assays with equal or higher estimated potencies. The subsequent reevaluation of the 128 chemicals tested in the presence of individual human CYP enzymes changed the activity calls for five compounds and shifted the estimated potencies for several others.

**Discussion:** This study shows the AR2 assay is well suited to replace the previous AR dimerization assays in a revised computational model to predict AR bioactivity for parent chemicals and their metabolites.

## Introduction

Endocrine disrupting chemicals (EDCs) are compounds that alter normal hormone signaling. Human exposure to EDCs can have profound and lasting effects, particularly during developmental life stages (Filer et al., 2014). In 1998, the US EPA’s Endocrine Disruptor Screening Program (EDSP) was established to “use validated methods for the screening and testing of chemicals to identify potential endocrine disruptors, determine adverse effects, dose-response, assess risk and ultimately manage risk under current laws” (U.S. EPA, 2002a). The current test battery relies upon a number of animal-based assays that have proven too time- and resource-intensive to meet the demands of the EDSP. Following the advances in biotechnology and computational modeling in the early 21^st^ century, EPA launched ToxCast, a toxicity forecasting project built upon high-throughput screening data of agency-relevant environmental chemicals. A key aim of the ToxCast program was to implement a two-tiered testing approach to pre-screen chemicals for their potential effect on three major endocrine signaling pathways: estrogen, androgen, and thyroid. This approach relies on quantifying endocrine disruption using *in vitro* high-throughput screening (HTS) assays and integrating results across wide-ranging chemical and biological endpoints using computational models (Thomas et al., 2019). The use of rapid, cost-effective approaches to screen and prioritize chemicals for formal EDSP testing facilitates faster risk assessment decisions to limit exposure to EDCs.

A subset of the ToxCast assays was used to construct a predictive model of estrogen receptor (ER) bioactivity (Browne et al., 2015; Judson et al., 2015). The ToxCast ER Model was validated using an internationally developed list of ER reference chemicals and is now used as a regulatory alternative to some EDSP tier 1 assays including the *in vivo* uterotrophic screening assay (Federal Register, 2002). A similar model has been constructed using ToxCast assay data for the androgen receptor (AR) with the goal to serve as an alternative to the *in vitro* AR binding assay and the *in vivo* EDSP tier 1 Hershberger screening assay. The data used to build both the ToxCast ER and AR models are derived from a battery of *in vitro* assays that measure four critical receptor pathway events: 1) ligand binding, 2) receptor dimerization and/or co-factor recruitment, 3) transactivation, and 4) cell proliferation and viability. While minimal (streamlined) models built on subsets of these HTS assays generally required at least one of each type, (Judson et al., 2017; Judson et al., 2020), receptor dimerization assays proved to be especially important for building highly predictive models, especially for AR antagonists. These dimerization assays measured the protein-protein interaction (PPI) between the receptor and itself (homodimer) or a co-activator protein (heterodimer). The ER and AR PPI assays used to build the original ER and AR models are no longer available through the original sources; therefore, establishing revised models to assess new chemicals will require the development of replacement assays. Ideally, replacement assays would: 1) provide predictive bioactivity data that maintains or improves the performance of the ER and AR computational models, 2) be of equal or superior throughput to the legacy assays, 3) undergo a thorough interlaboratory validation, and 4) not require cost-prohibitive reagents or detection equipment.

This study describes the development of a novel cell-based HTS assay that measures ligand-dependent AR homodimerization using the split-reporter Nanoluciferase Binary Technology (NanoBiT) (Dixon et al., 2016). The NanoBiT system is composed of two stable subunits, Large BiT (LgBit, 18 kDa) and Small BiT (SmBit, 1.3 kDa) that together, constitute a functional NanoLuc luciferase enzyme. Because the subunits have relatively low affinity for one another, the fusion of LgBit and SmBit to proteins of interest can be used to quantitatively measure PPI. Here, we developed a stable HepG2 cell line (HepG2-AR2) that constitutively overexpresses full-length human AR fused to either LgBit or SmBit to test 128 chemicals with known endocrine activity in both agonist and antagonist modes. These results were then compared to legacy ToxCast assays for AR co-factor recruitment to determine whether the AR2 assay is a suitable replacement assay capable of generating data for use in future computational modeling efforts.

It is well known that one of the major limitations of the HTS assays used in ToxCast is the inability to recapitulate the effects of *in vivo* xenobiotic metabolism (Tice et al., 2013). The latest ToxCast strategic plan proposed to address the metabolic deficiency by retrofitting existing HTS assays using two parallel approaches (Thomas et al., 2019). One of these approach utilizes the transfection of chemically modified mRNAs encoding human cytochrome P450 (CYP) enzymes into cells to induce intracellular CYP expression and functional activity (DeGroot et al., 2018). Here, we adapted this method to retrofit the AR2 assay for the same purpose, and present the results of a parallel reevaluation of the 128 test chemicals serially tested with each of ten different human CYP enzymes. These results showcase the amenability of the AR2 assay to the mRNA transfection method and, more importantly, demonstrate how HTS assays enhanced with metabolic activity can alter the bioactivity of tested chemicals.

## Materials and methods

### Chemicals

Dimethyl sulfoxide (DMSO), (17b)-17-hydroxy-17-methyl-estra-4,9,11-trien-3-one (R1881), bicalutamide (BICAL) and dichlone (DCLN) were purchased from Sigma-Aldrich (St. Louis, MO). The 128 chemical test set used to evaluate the AR2 assay, and their respective sources, are listed in Supplementary Table S1. All control and test compounds were solubilized in DMSO, plated in Echo-qualified source plates (Beckman Coulter; Brea, CA), and stored sealed with adhesive foil in a desiccator at −80°C prior to experiments. All compound plates were thawed and stored in a desiccator away from light at ambient temperature 24 h before and between experimental replicates.

### Plasmid constructs

A double-stranded oligonucleotide encoding a G_4_Sx2 linker (N-GGGGSGGGGS-C) was cloned into the pTRED plasmid (Simmons et al., 2011) between the SpeI and XhoI restriction sites. The LgBit NanoLuc domain (amino acids 1-158) was isolated by PCR from pNL1.1CMV-Nluc (Promega; Madison, WI) and cloned into pTRED in frame and upstream of the linker between the BamHI and SpeI sites (pLgBitN) or downstream between the XhoI and XbaI sites (pLgBitC). A double-stranded oligonucleotide encoding the SmBit NanoLuc domain (modified NanoLuc amino acids 159-170; N-LVTGYRLFEEIL-C) was cloned into pTRED in frame and upstream of the linker between the BamHI and SpeI sites (pSmBitN) or downstream between the XhoI and XbaI site (pSmBitC). Human cDNAs for the androgen receptor (GenBank: BC132975.1) and p160/steroid receptor coactivator-1 (SRC1; GenBank: BC111533.1) were purchased from Horizon Discovery (Lafayette, CO). cDNA encoding full-length AR and the receptor interacting domain (RID; amino acids 596-979) of SRC1 were isolated by PCR and cloned in frame into pLgBitC and pSmBitC between the BamHI and SpeI sites and into pLgBitN and pSmBitN between the XhoI and XbaI sites. This resulted in AR and SRC1 RID fused to either LgBit or SmBit in every possible combination and orientation. All recombinant plasmids were purified by cesium chloride-ethidium bromide gradient and sequence verified by fluorescent DNA capillary sequencing as previously described (Simmons et al., 2011).

### Cell culture

HepG2 human hepatocellular carcinoma cells (passage 86) were purchased from American Type Culture Collection (ATCC; Manassas, VA) and cultured in a humidified 37°C atmosphere containing 5% CO_2_ in a complete growth medium composed of high-glucose Dulbecco’s Modified Eagle Medium (DMEM) with L-glutamine and sodium pyruvate (Life Technologies; Grand Island, NY) supplemented with 10% qualified fetal bovine serum (Life Technologies), penicillin-streptomycin (100 U/mL-100 μg/mL final concentration; HyClone; Logan, UT), and 5 mM HEPES (Sigma-Aldrich). HepG2 cryostocks were preserved at passage 91. Wild-type HepG2 cells were recovered from cryopreservation and sub-cultured every 48–72 h at a density of 8-12 × 10^4^ cells/cm^2^ up to passage 121. For all experiments, cells were seeded into solid white 384-well microplates (Greiner Bio-One, Monroe, NC) pre-coated with collagen I (from rat tail; prepared at 50 μg/mL in 0.05 N acetic acid, Corning, Corning, NY) at 8,000 cells per well in 40 µL of assay medium composed of phenol red-free DMEM (Life Technologies; Grand Island, NY) supplemented with 4 mM glutamine and 2% charcoal-dextran stripped FBS (Atlanta Biologicals; Flowery Branch, GA) using a Certus Flex automated liquid dispensing system (Fritz Gyger AG, Switzerland) fitted with a 0.45/0.15 mm microvalve at 0.2 bar. Cells were counted with a Scepter 3.0 Handheld Automated Cell Counter (Millipore; Burlington, MA).

### Transient transfections

Purified plasmids encoding AR- and SRC1-LgBit and -SmBit fusions were transiently transfected into HepG2 cells (plated in 384-well plates as described) using Lipofectamine 3000™ (Thermo Fisher; Waltham, MA). Transfection mixes were prepared in 327 µL batches composed of 300 µL phenol red-free Opti-MEM™ (Life Technologies; Grand Island, NY), 3 µg each of LgBit and SmBit fusion plasmid (equimolar), 9 µL of Lipofectamine 3000™, and 12 µL of P3000 reagent per manufacturer’s protocol. Each well was transfected with 2.5 µL of DNA lipid nanoparticles dispensed using a Certus Flex fitted with a 0.45/0.15 mm microvalve at 0.06 bar. Transfected cells were incubated 56 h as previously described prior to administration of test chemicals.

### Lentiviral preparation and stable cell transduction

Lentiviral vectors for the AR-LgBitC and SmBitN-AR fusions were prepared and titered as previously described (Simmons et al., 2011). HepG2 cells (passage 98) were transduced with AR-LgBitC and SmBitN-AR lentiviral vectors at a multiplicity of infection (MOI) of 5 for both constructs (equimolar) to generate the polyclonal stable reporter cell line HepG2-AR2 (referred to hereafter as “AR2 cells”) which was subsequently cultured identically to parental HepG2 cells. AR2 cells cryostocks were preserved at passage 103. AR2 cell experiments used cells recovered from cryopreservation and sub-cultured every 48–72 h up to passage 133.

### AR2 assay

AR2 cells were seeded in 384-well plates (as described) and incubated 24 h prior to chemical exposure. For agonist mode experiments, cells were treated with DMSO (vehicle; eight replicate wells), R1881 (positive agonist control) titrated at 12 concentrations from 722 pM to 10 nM (final concentration) with four replicate wells at 10 nM (maximally effective concentration; MEC), or test chemicals titrated at 11 concentrations from 691 nM to 99.9 µM for 18 h. For antagonist mode experiments, all cells were treated with R1881 at a final concentration of 10 nM (to induce AR homodimerization) and co-treated with either DMSO (vehicle), bicalutamide (positive antagonist control) titrated at 12 concentrations from 722 nM to 99.8 µM (final concentration) with four replicate wells at 99.8 µM (MEC), or test chemicals titrated at 11 concentrations from 691 nM to 99.9 µM for 18 h. Dichlone titrated at 12 concentrations from 722 nM to 99.8 µM (final concentration) with four replicate wells at 99.8 µM (MEC) was included as a positive cytotoxicity control. Final DMSO concentrations were 0.1% for all wells. Assay plates were equilibrated to ambient temperature for 15 min before 2.5 µL of a NanoLuc substrate solution (18x) composed of 250 µM furimazine (AOBIOUS; Gloucester, MA), 290 mM NaCl, 5.5 mM EDTA, 27.5 mM HEPES and 0.275% Tween-20 was added to each well using a Certus Flex fitted with a 0.45/0.15 mm microvalve at 0.2 bar. Assay plates were read immediately on a CLARIOstar microplate reader (BMG Labtech; Cary, NC) using an endpoint luminescent protocol (top read) with an integration time of 0.08 s, unrestricted gain to maximize signal detection and a 384-well aperture spoon to minimize signal interference from neighboring wells. Following luciferase assay signal detection, 2.66 µL of an Alamar Blue solution (18x) composed of 120 µM resazurin sodium (Thermo Fisher) solubilized in 1x phosphate-buffered saline (pH 7.4) was added to each well using a Certus Flex fitted with a 0.45/0.15 mm microvalve at 0.2 bar. Assay plates were incubated for 2 hours in a humidified 37°C atmosphere containing 5% CO_2_ before reading on a CLARIOstar microplate reader using a fluorescence intensity protocol, single multichromatic setting with a 530 nm excitation filter (15 nm bandpass), a 590 nM emision filter (20 nm bandpass), a 558.8 nm dichroic setting, a restricted gain setting of 860 (0-4095), top optic read with a focal height of 7.8 mm, integrating 35 flashes per well.

### mRNA transfection

DNA plasmids serving as templates for *in vitro* transcription of beta-galactosidase (Bgal, used as a non-specific negative control), human cytochrome P450 enzymes CYP1A2, CYP2A6, CYP2B6, CYP2C8, CYP2C9, CYP2C19, CYP2D6, CYP2E1, CYP2J2, CYP3A4, and P450 oxidoreductase (POR) were previously described (DeGroot et al., 2018). A single point mutation was introduced by site-directed mutagenesis to make a single G to A substitution at the transcriptional start site within each pmRNA (TriLink Biotechnologies) plasmid harboring the 12 cDNAs to facilitate CleanCap® synthesis instead of the anti-reverse cap analog (ARCA) used previously. The mutant sequence was designated pmRNA2 and verified by fluorescent DNA capillary sequencing. Additionally, site-directed mutagenesis was used to revert the inactive mutant *CYP1A2* clone identified in the previous study to the wild-type sequence by introducing a single A to G point mutation that changed amino acid 81 from aspartic acid to glycine. Purified pmRNA2 plasmids were sent to TriLink Biotechnologies for the synthesis of chemically modified mRNA. The purified mRNA products were fully substituted with pseudouridine (ψ), contained a poly(A) tail, and were capped using CleanCap®. All mRNAs were processed *via* standard procedures including enzymatic treatment with DNase and phosphatase to remove the DNA template and the terminal 5’ triphosphate, respectively. Upon receipt, mRNA was aliquoted into single use volumes and stored at −80°C.

For all mRNA transfection experiments, mRNA:lipid complexes were prepared at 18 h post-seeding using Lipofectamine MessengerMAX (ThermoFisher) per the manufacturer’s recommendations. Briefly, MessengerMAX transfection reagent was diluted 1:20 into phenol red-free Opti-MEM™ and incubated for 10 min at room temperature. Separately, mRNA was diluted into an equivalent volume of phenol red-free Opti-MEM™ and then mixed with the diluted MessengerMax transfection reagent and incubated for an additional 5 minutes at ambient temperature. The mRNA lipid nanoparticles were dispensed at 5 µL per well using a Certus Flex fitted with a 0.45/0.15 mm microvalve at 0.06 bar. The 5 µL delivery constituted 50 ng total mRNA per well with 1% (500 pg) being POR mRNA for all CYP transfection groups. The Bgal group contained no POR mRNA. Cell plates were returned to a 37°C/5% CO_2_ humidified atmosphere and incubated 6 hours before controls or test chemicals were administered.

### Data analysis

Data were analyzed using R (version 4.1.0) and RStudio (version 1.4.1717). All data files and source code are made available at (https://github.com/SimmonsLabEPA/AR2-Assay-Method.git). Plate level statistics were derived for each assay plate using well-level raw relative light unit (RLU) data for all luminescent endpoints or relative fluorescence unit (RFU) data for the Alamar Blue cytotoxicity assay. For agonist mode, well-level normalized response (resp) was calculated as:

$$resp=\log2\left(\frac{RLU}{bval}\right)$$

where bval is the median RLU value for the two lowest concentrations across all test compounds.

For antagonist mode and cytotoxicity data, well-level normalized response (resp) was calculated as:

$$resp=100\;*\;\frac{\left(RLU-bval\right)}{\left(pval-bval\right)}$$

where pval is the median RLU value of the MEC positive control wells (minimum four wells).

These normalization methods yielded zero-centered values with positive responses suitable for concentration-response modeling using the tcpl package in R. Concentration-response curves were fit to a 3-parameter Hill model (bottom constrained to zero) using tcpl_lite R source code and plotted using the ggplot2 package. Active agonists were defined as those with a median response at any single concentration exceeding a 5*bmad threshold, where bmad is the median absolute deviation of normalized DMSO responses across the entire experiment. Active antagonists were defined as those with a median response at any single concentration exceeding a 3*bmad threshold and an antagonist half-maximal activity concentration (AC_50_) value at least 3.16 times (0.5 as log 10) lower than its cytotoxicity AC_50_ value (if also cytotoxic).

The rZ’ factor is a unitless metric that quantifies both the difference between the median vehicle and positive control responses (signal-to-background), but also the variability around both responses (Murray and Wigglesworth, 2016). rZ’ factors were calculated for each assay plate to evaluate assay performance using the following equation:

$$rZ^{\prime }=1-\frac{3*\left(pmad+dmad\right)}{abs\left(pmed-dmed\right)}$$

where pmad is the median absolute deviation (mad) RLU value of the MEC positive control wells, pmed is the median RLU value for of the MEC positive control wells, dmad is the mad of RLU values from DMSO-treated wells, and dmed is the median of RLU values from DMSO-treated wells.

Data for Odyssey Thera and University of Pittsburg AR co-factor recruitment (heterodimerization) assays were retrieved from the invitroDBv3.4 data base (released October 2021) on 5 May 2022 (U.S. EPA, 2002b).

## Results

### Construction of AR/SRC1 NanoBit fusions and identification of optimal reporter pair

**FIGURE 1:**
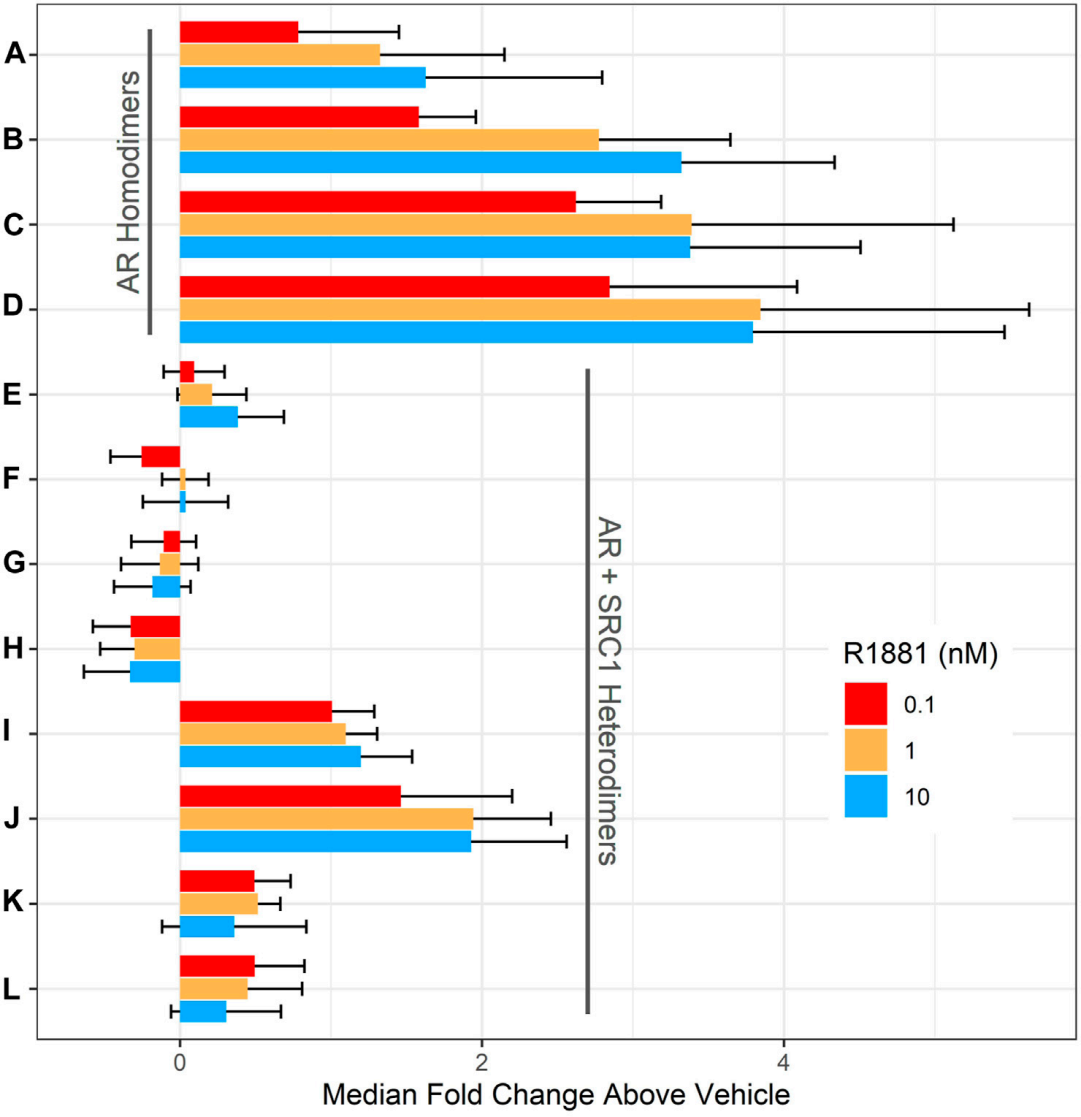
Evaluation of AR dimer pairs. HepG2 cells were transiently transfected with DNA plasmids encoding one LgBit and one SmBit fusion. The LgBit and SmBit fusion parter pairings **(A–L)** are listed in Table 1. 56 h after transfection, cells were treated with R1881 at 0.1 (red), 1 (orange), or 10 nM (blue; final concentration) for 18 h before NanoLuc luciferase activity was detected. Data are expressed as median fold change over DMSO-treated (vehicle) wells ± the median absolute deviation (mad) and represent four replicate wells for each group at each concentration tested in three independent experiments.

DNA encoding full-length human AR and the SRC1 receptor interaction domain were cloned in frame with either LgBit or SmBit in each possible orientation into a lentiviral transfer plasmid to facilitate subsequent stable cell line development. A complete set of all possible AR homodimer and AR-SRC1 heterodimer LgBit/SmBit pairs (Table 1. A–L) were transiently transfected into HepG2 cells (1:1 ratio of each pair) then treated with DMSO (vehicle control), 0.1, 1, or 10 nM R1881 (synthetic androgen control) for 18 h. Nanoluciferase activities were determined and are reported as median fold change above vehicle in Figure 1. Overall, nine of the 12 homo- and heterodimer pairs tested exhibited a positive response to R1881 treatment at each of the three tested concentrations. The AR homodimer pairs (A-D) exhibited a greater fold response to R1881 treatment than the AR-SRC1 heterodimer pairs (E-L). The homodimer pairs with N-terminal LgBit (C and D) exhibited the highest overall response to R1881, specifically at 1 and 10 nM. The homodimer pairs with C-terminal LgBit (A and B) exhibited a stepwise response across the tested range of R1881. The heterodimer pairs that included LgBit-SRC1 in all orientations (I-L) were modestly responsive to R1881 treatment, while the heterodimer pairs with SmBit-SRC1 (E-H) were largely unresponsive. This disparate pattern of response illustrates the importance of testing each combination and orientation to identify the optimal fusion partner pairing(s).

**TABLE 1:** Transfected NanoBit pairs.

Transfection group	LgBit (orientation^a^)	SmBit (orientation^a^)	Interaction	Cumulative rZ’
A	AR(C)	AR(C)	Homodimer	−4.94
B	AR(C)	AR(N)	Homodimer	0.08
C	AR(N)	AR(C)	Homodimer	−20.60
D	AR(N)	AR(N)	Homodimer	−85.70
E	AR(C)	SRC1(C)	Heterodimer	−0.50
F	AR(C)	SRC1(N)	Heterodimer	−1.22
G	AR(N)	SRC1(C)	Heterodimer	−24.90
H	AR(N)	SRC1(N)	Heterodimer	−11.40
I	SRC1(C)	AR(C)	Heterodimer	0.12
J	SRC1(N)	AR(C)	Heterodimer	−1.20
K	SRC1(C)	AR(N)	Heterodimer	−7.43
L	SRC1(N)	AR(N)	Heterodimer	−10.10

^a^ C = carboxy-terminal, N = amino-terminal.

The rZ’ factor is a unitless metric that quantifies both the difference between the median vehicle and positive control responses (signal-to-background), but also the variability around both responses (Murray and Wigglesworth, 2016). Assays suitable for high-throughput screening applications generally have rZ’ > 0.5. None of the homo- or heterodimeric pairs in these pre-optimized transient experiments had rZ’ > 0.5. This is likely due to overexpression of the fusion partners which promotes interaction and reporter activity in the absence of agonist thereby reducing the dynamic range. To identify a candidate pair for stable transgene integration, cumulative rZ’ factors (Table 1) were calculated for each pair by summing the rZ’ from each of the three tested R1881 concentrations to compare median dynamic range and variability across the tested R1881 concentrations to vehicle control. Pair B had the highest cumulative rZ’ factor of any AR homodimer pair. Pair I had a slightly higher cumulative rZ’ factor than pair B, but a lower fold change across all R1881 concentrations (Figure 1). The pair B plasmids encoding AR-LgBitC and SmBitN-AR were used to generate lentiviral vectors to establish a polyclonal stable AR dimerization reporter line, HepG2-AR2.

### Stable AR2 cells respond to agonists and antagonists

**FIGURE 2:**
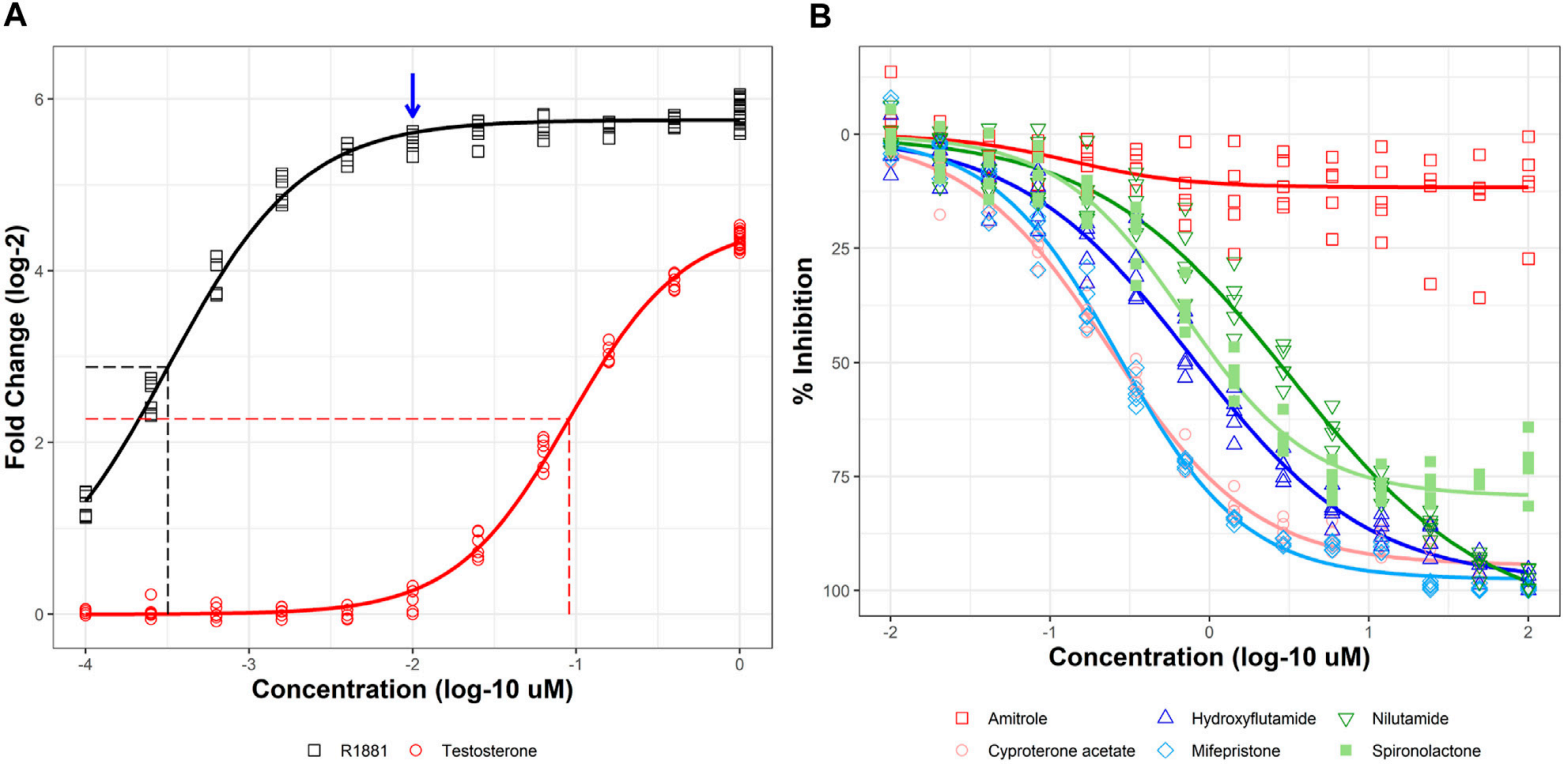
Agonist and antagonist responses in stable AR2 assay. Stable AR2 cells were treated with agonists at concentrations ranging from 100 pM to 1 µM **(A)** or co-treated with 10 nM R1881 and antagonists at concentrations ranging from 10 nM to 100 µM **(B)** for 18 h before NanoLuc luciferase activity detection. Micromolar concentrations are expressed as log 10 (*x*-axis). Responses are expressed as fold change (log 2) over DMSO vehicle for agonists and % inhibition (maximal response) for antagonists. Concentration-response curves were fit using tcpl_Lite. Horizontal dashed lines show half maximal response for agonists and vertical dashed lines mark the half maximal active concentration (AC_50_). The blue arrow is shown to denote the >95% effective R1881 concentration (10 nM) used to stimulate AR response for antagonist mode experiments. Data represent duplicate wells for each concentration tested in two independent experiments in panel A and individual wells from five independent experiments in panel **(B)**

AR2 cells were treated with a titrated concentration series of two known AR agonists, the synthetic androgen R1881 and testosterone for 18 h (Figure 2A; black and red lines, respectively). R1881 elicited a >50-fold response (5.75 shown as log-2) at concentrations above 10 nM and a half-maximal activity concentration (AC_50_) of 319 pM (vertical dotted black line). The lowest tested R1881 concentration (100 pM) produced a 2.41-fold response above vehicle control, suggesting lower concentrations were needed to observe a no effect level. Testosterone was not as efficacious as R1881, evoking only a 23-fold response (4.55 shown as log-2) and only at the highest tested concentration of 1 µM. Testosterone was also more than 200 times less potent than R1881 with an AC_50_ of 90.5 nM (vertical dotted red line). R1881 was selected as the positive control for agonist mode experiments whereby test chemicals were evaluated for their ability to promote AR homodimerization. R1881 at 10 nM (blue arrow) produced a >95% maximal response and was selected as the inducer for antagonist mode experiments where test chemicals are co-administered with R1881 and evaluated for their ability to disrupt AR homodimerization.

An example of antagonist mode testing is shown in Figure 2B. R1881 (10 nM) was co-administered with a titrated series of five known AR antagonists and a negative control for 18 h. Cyproterone acetate (pink), an antiandrogenic progestin, was the most potent AR antagonist with an AC_50_ of 240 nM. The steroidal antiprogestogen, mifepristone (light blue) was nearly as potent with an AC_50_ of 271 nM. Two other chemicals, hydroxyflutamide (dark blue) and nilutamide (dark green) elicited full antagonist responses (>90% inhibition) albeit at higher concentrations (AC_50_ values of 781 nM and 3.26 µM, respectively). The antihypertensive drug spironolactone (light green) partially inhibited AR dimerization with maximum efficacy of 79% at the highest test concentrations. Amitrole (red) was used as a negative control and did not significantly inhibit AR dimerization. Together, these results show that AR2 responds predictably to both agonists and antagonists.

### AR2 evaluation using 128 chemicals with known endocrine activities

**FIGURE 3:**
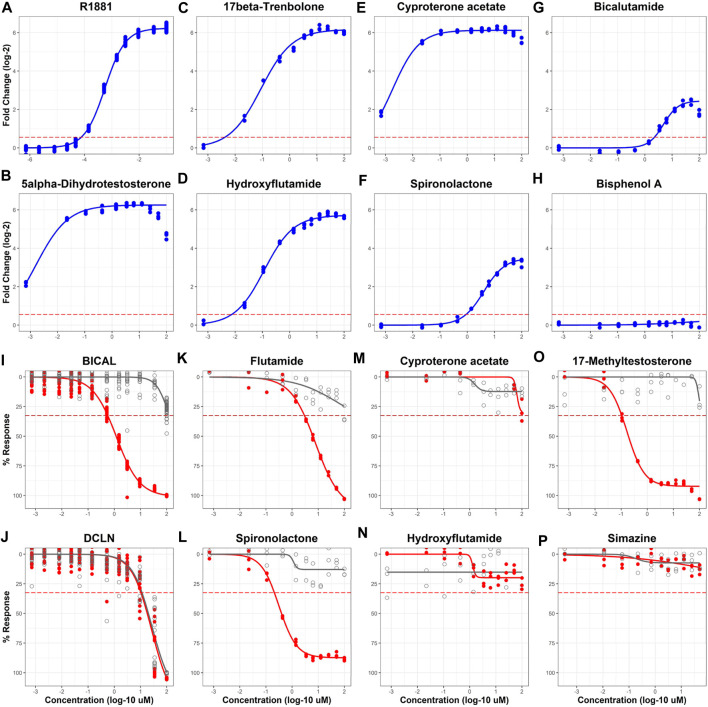
Sample agonist and antagonist mode concentration-response curves for 128 evaluation chemicals. AR2 cells were exposed to 128 unique chemicals at concentrations ranging from 691 pM to 99.9 µM for 18 h in both agonist [blue curves, **(A–H)**] and antagonist modes [red curves, **(I–P)**]. Micromolar concentrations are expressed as log 10 (*x*-axis). Responses are expressed as fold change (log 2) over DMSO vehicle for agonists **(A–H)** and % response (% maximal positive control) for antagonists **(I–P)**. Concentration-response curves were fit using tcpl_Lite. The red dotted lines represent the activity thresholds defined by the baseline median absolute deviation (bmad) for both agonists (5 x bmad) and antagonists (3 x bmad). Antagonist mode testing utilized a *post hoc* cell viability assay (I-P, grey) to identify responses confounded by cytotoxicity. Concentrations shown for cell viability reflect those for the 18-h exposure period and do not account for reagent addition during the 2-h reagent incubation. Data represent individual wells from three independent experiments. BICAL and DCLN are the designations for bicalutamide and dichlone controls. The agonist and antagonist response curves for all 128 chemicals can be found in Supplementary Figures S1, S2, respectively.

A set of 128 chemicals (Supplementary Table S1) used in previous studies to validate AR and ER assays was used to evaluate the performance of the AR2 assay in both agonist and antagonist modes (Figure 3; Supplementary Figures S1, S2). The 128-chemical set includes drugs with pro- and anti-androgen, estrogen, progestin, cortisol, and aromatase activities as well as pesticides and compounds used in various industrial processes. Of the 44 unique reference chemicals used by Kleinstreuer *et al.* to validate the Tox21 AR computational model (Kleinstreuer et al., 2017), 43 (excepting 4-tert-octylphenol) were included in the 128 chemicals tested in this study (Supplementary Table S1). Among those, there were 28 AR agonist and 26 AR antagonist reference chemicals included (11 overlapping chemicals). In agonist mode (Figures 3A–H; blue curves), test chemicals are administered and evaluated for their ability to stimulate AR homodimerization. The positive control R1881 induced a 75-fold response (Figure 3A; 6.23 shown as log-2) with an AC_50_ of 560 nM and a median assay plate rZ’ factor of 0.94. Thirty (23%) of the 128 test chemicals tested active (i.e., maximal median response >5*bmad) in agonist mode including all eight active AR agonist reference chemicals (100%) and all five androgenic drugs (100%) including 5α-dihydrotestosterone (Figure 3B) and 17β-trenbolone (Figure 3C). Interestingly, 10 of the 19 AR antagonist reference chemicals, including four of the six anti-androgen drugs, also tested active in agonist mode. Among these were hydroxyflutamide, cyproterone acetate, spironolactone, and the AR antagonist positive control used in this study, bicalutamide (Figures 3D–G). Only 2 of the 16 agonist reference inactive chemicals tested active: 17α-estradiol and prochloraz. The remaining 98 test chemicals tested negative as illustrated by bisphenol A (Figure 3H). The agonist mode dose-response curves for all 128 evaluation chemicals and assay controls are provided in Supplementary Figure S1 and the summary dose-response data is provided in Supplementary Table S2.

The antagonist mode of the AR2 assay utilizes a maximally effective concentration of R1881 agonist to stimulate AR homodimerization co-administered with test compounds. After the exposure period, test compounds are evaluated for their ability to disrupt R1881-mediated AR homodimerization, which is measured by a loss of assay signal. In contrast to the agonist mode which uses a gain of signal approach, the antagonist mode can be confounded by other influences like cytotoxicity that create a loss of assay signal independent of AR bioactivity. To delineate AR antagonists and cytotoxic compounds, the antagonist mode includes a *post hoc* fluorescent cell viability measurement taken after the initial luminescent signal read from the AR2 assay. An active test result in the antagonist mode requires that a chemical produce an AR2 maximal median response above the activity (inhibition) threshold (3*bmad) and have an AR2 assay AC_50_ value that is at least 0.5 log units (3.16-fold) lower that its cytotoxicity AC_50_ value. This is illustrated clearly in Figure 3I with the antagonist positive control, bicalutamide (BICAL) which elicited an AR inhibition response (red) at concentrations >100-fold (>2 log units) lower than its cytotoxicity (grey) whereas the cytotoxicity positive control, dichlone (DCLN; Figure 3J), produced an AR inhibition response that precisely coincided with its cytotoxicity. BICAL induced a maximal inhibitory response with an AC_50_ of 560 nM and a median assay plate rZ’ factor of 0.85. Of the 128 test chemicals, 72 (56%) tested active in antagonist mode. Sixteen of the 18 AR antagonist reference chemicals (88%) tested active in antagonist mode including flutamide (Figure 3K), spironolactone (Figure 3L), and all seven of the “weak” reference antagonists. The two AR reference antagonists that tested inactive, cyproterone acetate and hydroxyflutamide (Figures 3M, N), failed to produce an AR inhibition response above the threshold in this experiment but were active in the previous experiment (Figure 2B). Three of the seven reference antagonist inactive chemicals tested active including 17-methyltestosterone (Figure 3O) and each elicited a strong antagonist response (>80% inhibition) with AC_50_ values < 1 µM. Another 20 test chemicals produced antagonist responses that were confounded by cytotoxicity exemplified by DCLN and 36 were inactive such as simazine (Figure 3P). The antagonist mode dose-response curves for all 128 evaluation chemicals and assay controls are provided in Supplementary Figure S2 and the summary dose-response data is provided in Supplementary Table S3.

### Comparison of AR2 results to legacy ToxCast AR dimerization assays

The original AR computational model and subsequent minimal assay set models utilized AR dimerization ToxCast assays derived from two sources, Odyssey Thera (OT) and University of Pittsburgh (UPitt). The OT AR_ARSRC1 assay utilizes a reporter complementation approach fusing full-length human AR and the receptor interacting domain (RID) of SRC1 to bipartite domains of yellow fluorescent protein (YFP) in human HEK293T cells (U.S. EPA, 2022). Both agonists and antagonists reportedly drive AR-SRC1 heterodimerization and translocation to the nucleus (measured as the nuclear:cytoplasm ratio of reconstituted YFP signal), therefore there are no separate agonist/antagonist modes for the OT assay. The UPitt HCI_U2OS_AR_TIF2_Nucleoli assays use only the ligand-binding (LBD) and activation function 2 (AF2) domains of human AR, which comprise about ∼30% of the full-length receptor, fused to red fluorescent protein (RFP) and the RID of human SRC2 fused to green fluorescent protein (GFP) in human U-2OS cells (Hua et al., 2014). Response in the UPitt assay is reported as colocalized nuclear RFP and GFP signal, for both agonist and antagonist modes (using 20 nM DHT as the inducer). Unlike the AR2 assay which measures AR homodimerization using a luminescent endpoint, the OT and UPitt assays measure AR dimerization with SRC family members using a fluorescent imaging approach.

**FIGURE 4:**
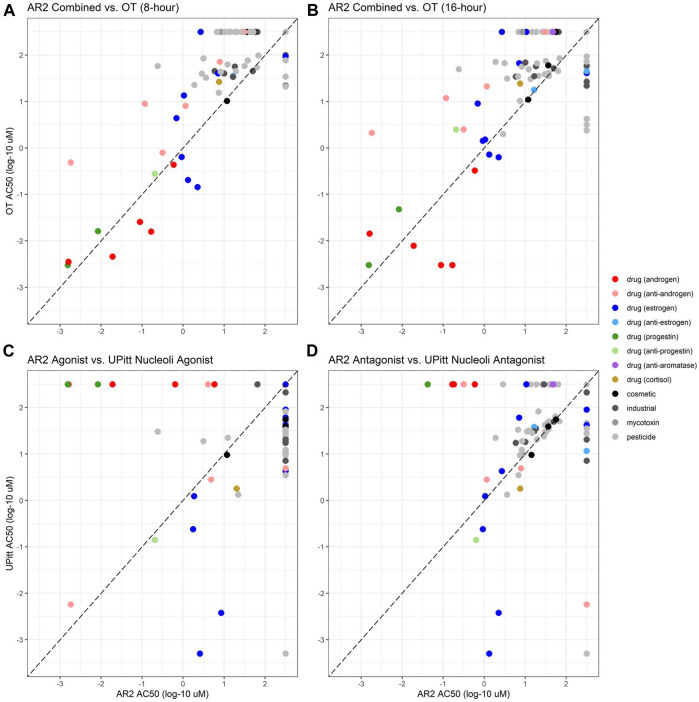
Comparison of AR2 bioactivity with legacy ToxCast assay data. Activity calls and estimated potencies (AC_50_) for chemicals tested in AR2 agonist and antagonist assay modes were compared to ToxCast data from the Odyssey Thera (OT, **(A, B)** and University of Pittsburgh **(C, D)** AR dimerization assays. Any active call and the lower AC_50_ value (if active in both modes) was used for AR2 to facilitate a comparison to the single mode OT assay run at both 8 **(A)** and 16 h **(B)**. AR2-UPitt comparisons match agonist mode **(C)** or antagonist mode data. AC_50_ values are micromolar concentrations expressed as log 10 for AR2 (*x*-axis) and OT/UPitt assays (*y*-axis). The diagonal dashed line represents equipotency in both assays. Chemicals above the diagonal were more potent in the AR2 assay and those below more potent in the OT/UPitt assays. Chemicals inactive in either assay were assigned an AC_50_ of 2.5 and are aligned along the top (inactive in OT/UPitt assays) and right (inactive in AR2) of each graph.

Of the 128 chemicals used to evaluate the performance of the AR2 assay, 104 had been previously tested in the OT assay using two exposure durations of eight and 16 h, whereas the AR2 assay used an 18-h exposure period. Since the OT assay does not have distinct agonist and antagonist modes, AR2 agonist and antagonist data were combined to facilitate a comparison of AR2 and OT assays at both timepoints (Figures 4A,B). The AR2 activity call and AC_50_ value from either the agonist or antagonist mode were used and the lower AC_50_ value was used for compounds active in both modes. Inactive chemicals were assigned an AC_50_ value of 2.5 for both assays. Of the 104 common chemicals, 45 were active at 8 hours and 58 at 16 h in the OT assay. Among these, 39 (87%) were active in the AR2 assay: 23 as agonists, 34 as antagonists with 18 testing active in both modes. The six chemicals missed by AR2 (4-(1,1,3,3-tetramethylbutyl)phenol, 4-dodecylphenol, chlorothalonil, folpet, raloxifene hydrochloride, and tetramethrin) were inactive as agonists, and all excepting tetramethrin had an active antagonist response confounded by cytotoxicity. As with AR2, the OT assay identified all eight AR reference agonists and all five androgenic drugs but with AC_50_ values lower than those estimated from AR2 (red circles below dashed line). The OT assay also identified 14 of the 17 (82%) AR reference antagonists while 15 (88%) were identified by AR2. Among the six anti-androgen drugs (pink circles), the OT assay correctly identified five including hydroxyflutamide and cyproterone acetate which were missed in the subsequent AR2 experiments, but misclassified finasteride. The antagonist AC_50_ values estimated from the OT assay were generally higher (lower potency) than those from AR2 (pink circles above dashed line).

The UPitt assays previously tested 101 of the 128 chemicals used to evaluate AR2 (and all within the set of 104 tested in the OT assay). The UPitt assays utilized a 2-h exposure and tested chemicals in both agonist and antagonist modes which allowed for a direct comparison with the AR2 assay for agonists (Figure 4C) and antagonists (Figure 4D). The UPitt assay identified 53 actives in agonist mode but failed to detect any of the four androgenic drugs and missed 7 of the 8 AR agonist reference chemicals, correctly identifying only cyproterone acetate, a weak reference agonist. These results are in stark contrast to those from the AR2 assay where all four androgenic drugs and all eight AR reference agonists were correctly identified (100%). In antagonist mode, the UPitt assay correctly identified 4 of 5 (80%) of the anti-androgenic drugs and 15 of 16 of the AR antagonist reference chemicals (94%), misclassifying only spironolactone, identical to the results from the AR2 assay except that AR2 misclassified cyproterone acetate. The poor performance of the UPitt assay in agonist mode obviates any comparison of relative potencies between the assays; however, in antagonist mode the AC_50_ values derived from AR2 were generally lower (median = 0.08 log units) than those from the UPitt assay with the notable exceptions of two estrogenic drugs (blue circles), 17α-estradiol and 17β-estradiol, which were active in the UPitt assay at concentrations more than 100-fold lower than in the AR2 assay. Tables 2, 3 show the assay comparisons for AR reference agonists and antagonists, respectively.

**TABLE 2:** Assay Comparison using Reference Agonists.

Chemical	Agonist reference	AR2 agonist	OT 8 hr	OT 16 hr	UPitt agonist
17beta-Trenbolone	Strong	1	1	1	NT
17-Methyltestosterone	Strong	1	1	1	0
Levonorgestrel	Strong	1	1	1	0
Norethindrone	Strong	1	1	1	0
Testosterone propionate	Strong	1	1	1	0
4-Androstene-3,17-dione	Moderate	1	1	1	0
5alpha-Dihydrotestosterone	Moderate	1	1	1	0
Cyproterone acetate	Weak	1	1	1	1
17alpha-Estradiol	Inactive	1	1	1	1
Atrazine	Inactive	0	0	0	0
Benomyl	Inactive	0	0	1	0
Carbendazim	Inactive	0	0	1	0
Cyfluthrin	Inactive	0	0	0	0
Cypermethrin	Inactive	0	0	0	0
Deltamethrin	Inactive	0	0	0	0
Fenarimol	Inactive	0	0	1	1
Finasteride	Inactive	0	0	0	1
Flutamide	Inactive	0	1	1	1
Fulvestrant	Inactive	0	0	0	0
o,p'-DDT	Inactive	0	1	1	1
Permethrin	Inactive	0	0	0	0
Prochloraz	Inactive	1	1	1	1
Tamoxifen	Inactive	0	1	1	1
Tetramethrin	Inactive	0	1	1	0
					
	True positive	8	8	8	1
	False positive	2	6	9	7
	False negative	0	0	0	6
	True negative	14	10	7	9
					
	Specificity	88%	63%	44%	56%
	Sensitivity	100%	100%	100%	14%
	Balanced accuracy	94%	81%	72%	35%

A total of 24 reference agonists (8 active, 20 inactive) were used to compare the AR2 assay to ToxCast assays from Odyssey Thera (OT) and the University of Pittsburgh (UPitt). The OT, assay was evaluated at two timepoints (8 and 16 h) and has a single protocol that detects both agonists and antagonists. Both the AR2 and UPitt, assays have a distinct agonist test mode, and both were evaluated at a single timepoint (18 and 2 h, respectively). NT, not tested, 0 = inactive, 1 = active.

**TABLE 3:** Assay Comparison using Reference Antagonists.

Chemical	Antagonist reference	AR2 antagonist	OT 8 hr	OT 16 hr	UPitt antagonist
Bicalutamide	Strong	1	1	1	1
Fenitrothion	Strong	1	1	1	1
Hydroxyflutamide	Strong	0	1	1	NT
Mifepristone	Strong	1	1	1	1
Spironolactone	Strong	1	1	1	0
Bisphenol A	Moderate	1	0	1	1
Cyproterone acetate	Moderate	0	1	1	1
Flutamide	Moderate	1	1	1	1
Linuron	Moderate	1	0	0	1
Prochloraz	Moderate	1	1	1	1
Fenarimol	Weak	1	0	1	1
Methoxychlor	Weak	1	0	1	1
o,p'-DDT	Weak	1	1	1	1
Procymidone	Weak	1	0	0	1
Propiconazole	Weak	1	1	1	1
Vinclozolin	Weak	1	1	1	1
Zearalenone	Weak	1	0	0	1
17-Methyltestosterone	Inactive	1	1	1	0
4-Androstene-3,17-dione	Inactive	1	1	1	0
Atrazine	Inactive	0	0	0	0
Deltamethrin	Inactive	0	0	0	0
Methomyl	Inactive	0	0	0	0
Simazine	Inactive	0	0	0	0
Testosterone propionate	Inactive	1	1	1	0
					
	True positive	15	11	14	15
	False positive	3	3	3	0
	False negative	2	6	3	1
	True negative	4	4	4	7
					
	Specificity	57%	57%	57%	100%
	Sensitivity	88%	65%	82%	94%
	Balanced accuracy	73%	61%	70%	97%

A total of 24 reference antagonists (21 active, 7 inactive) were used to compare the AR2 assay to ToxCast assays from Odyssey Thera (OT) and the University of Pittsburgh (UPitt). The OT, assay was evaluated at two timepoints (8 and 16 h) and has a single protocol that detects both agonists and antagonists. Both the AR2 and UPitt, assays have a distinct antagonist test mode, and both were evaluated at a single timepoint (18 and 2 h, respectively). NT, not tested, 0 = inactive, 1 = active.

### Metabolic retrofit of AR2 assay by mRNA transfection

**FIGURE 5:**
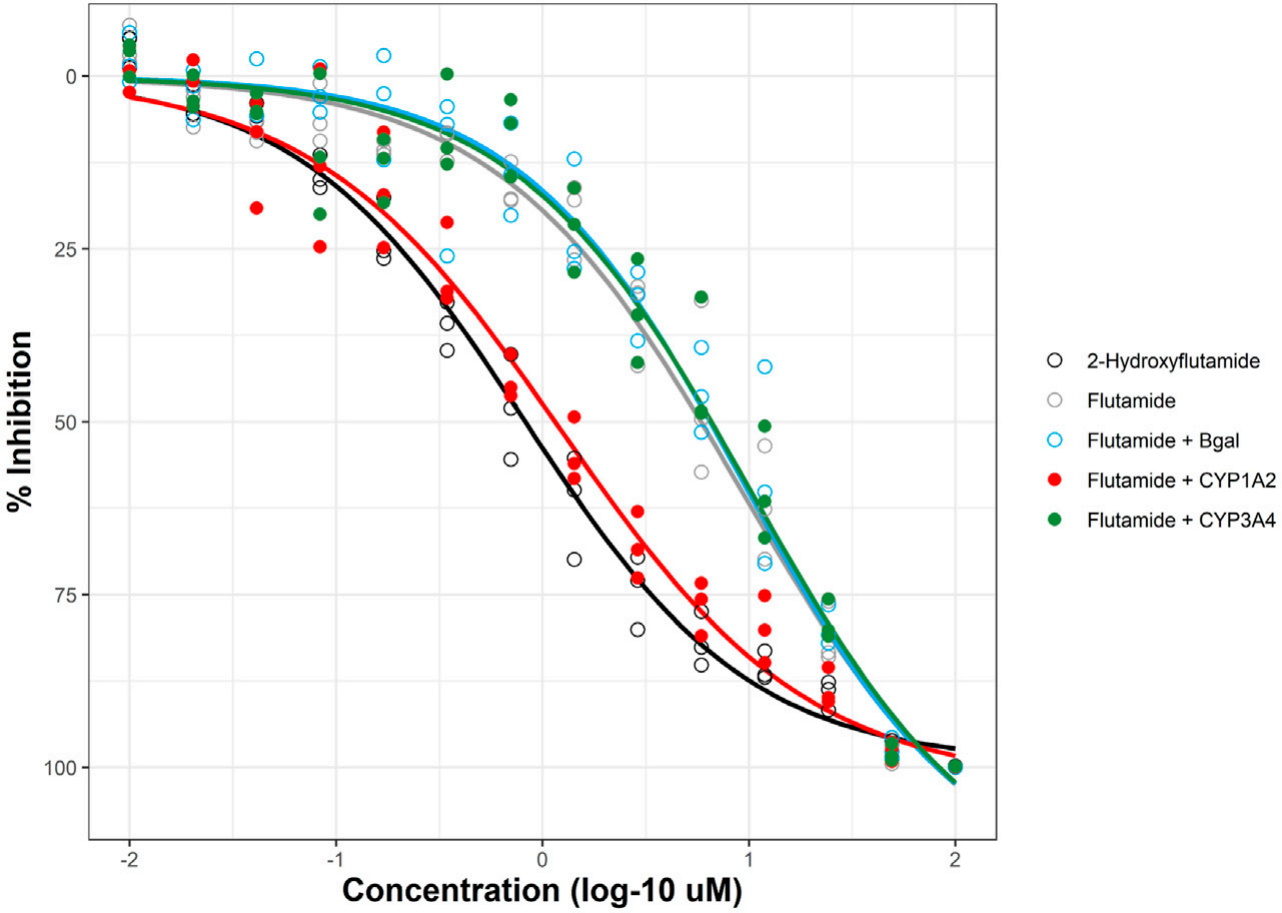
Metabolic retrofit of AR2 assay. AR2 cells were transfected with mRNAs encoding beta-galactosidase (Bgal, blue), human CYP1A2 (red), CYP3A4 (green), or no RNA (black and grey) 6 h prior to co-treatment with 10 nM R1881 and either flutamide or its anti-androgenic metabolite, 2-hydroxyflutamide (black) at concentrations ranging from 10 nM to 100 µM for an additional 18 h. Responses are expressed as % inhibition (of maximal response). Concentration-response curves were fit using tcpl_Lite. Data represent individual wells from three independent experiments.

The mRNA transfection method used in DeGroot *et al.* for HEK293T cells was adapted to retrofit the AR2 assay with CYP metabolic activity. The optimized mRNA transfection protocol using CleanCap® mRNA in this study for HepG2 cells resulted in a 100% increase in CYP activity compared to the previously published study using ARCA-capped mRNA in HEK293T (data not shown). AR2 cells were transfected 6 hours prior to chemical treatment to promote CYP expression and activity and then exposed for 18 h prior to endpoint detection. The previous study showed that maximal CYP activity was sustained beyond 18 h. The impact of CYP metabolism on an AR-active chemical can be clearly seen in Figure 5 with flutamide. In the absence of CYP metabolism, flutamide is an efficacious AR antagonist (Figure 5, grey; Figure 3K) with an AC_50_ of ∼9 μM; however, the clinical effectiveness of flutamide as an antiandrogenic drug depends on its metabolism *in vivo* to the more potent 2-hydroxyflutamide, primarily by CYP1A2 (Shet et al., 1997). A distinct metabolite (4-nitro-3-(trifluoromethyl)phenylamine) with AR antagonist potency similar to the flutamide parent is formed by CYP3A4 (Goda et al., 2006). Flutamide-treated AR2 cells transfected with Bgal (light blue) or CYP3A4 (green) elicited AR-inhibitive response to those of mock-transfected cells (grey) with AC_50_ values of 9.39, 10.2, and 9.21 µM, respectively. These results stand in stark contrast to mock-transfected AR2 cells treated with 2-hydroxyflutamide (black) with an AC_50_ value (811 nM), more than 10-fold lower than flutamide. Flutamide-treated AR2 cells transfected with CYP1A2 (red) produced an AC_50_ value of 1.22 µM, nearly 8-fold lower (more potent) than mock-, Bgal-, and CYP3A4-transfected cells. Based on the bioactivity of the 2-hydroxyflutamide control, these results suggest that more than 80% of the flutamide parent was metabolized to 2-hydroxyflutamide by the augmented CYP1A2 activity in AR2 cells.

**FIGURE 6:**
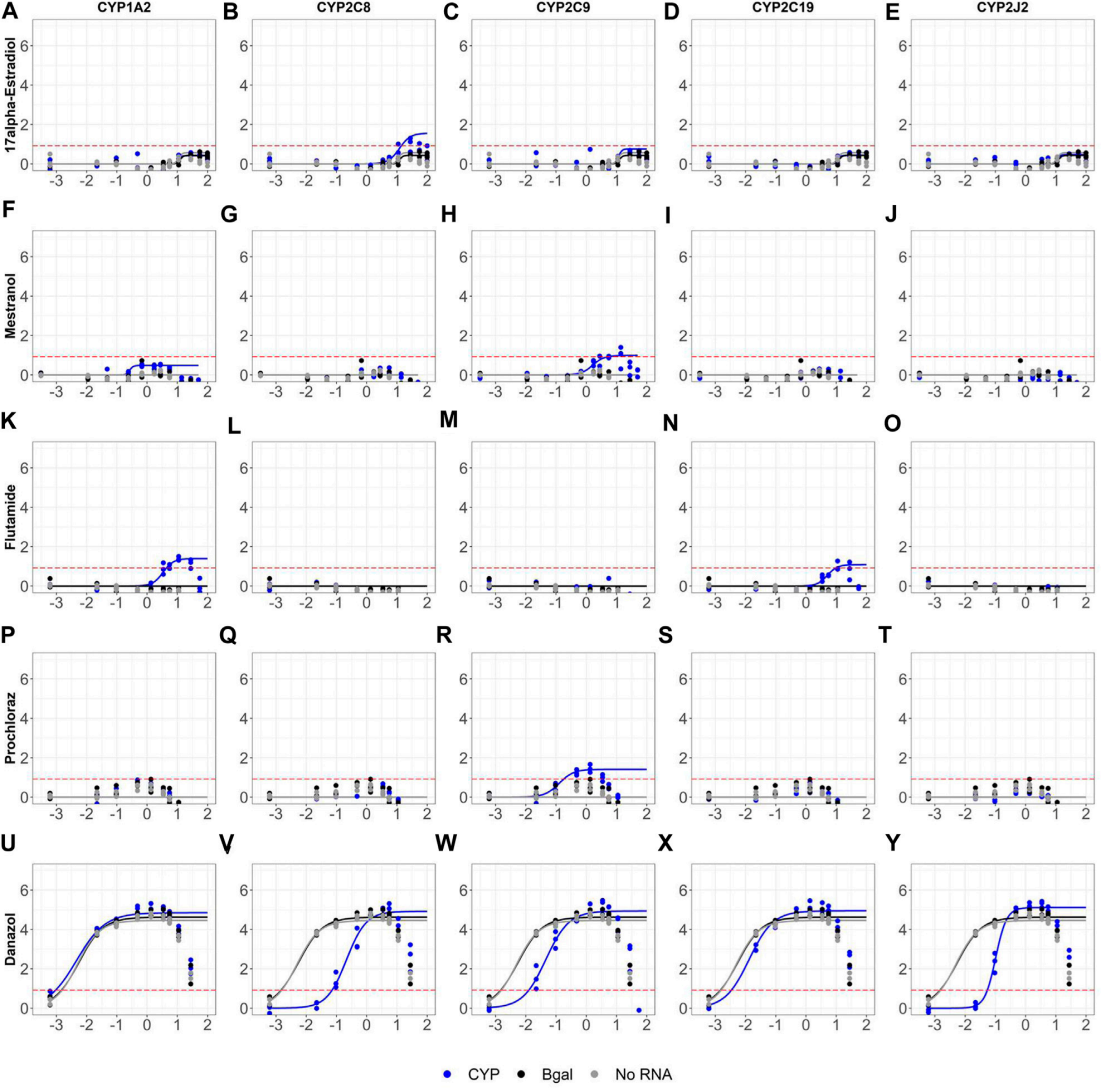
Sample retrofitted agonist concentration-response curves for 128 evaluation chemicals. AR2 cells were transfected with mRNAs encoding beta-galactosidase (black), one of ten human CYP enzymes (blue), or no RNA (grey) for 6 hours then exposed to 128 unique chemicals at concentrations ranging from 691 pM- 99.9 μM for 18 hours in agonist mode. Shown are the dose-response profiles for 17α-Estradiol **(A–E)**, Mestranol **(F–J)**, Flutamide **(K–O)**, Prochloraz **(P–T)** and Danazol **(U–Y)** in cells expressing one of five CYP enzymes: CYP1A2 **(A,F,K,P,U)**, CYP2C8 **(B,G,L,Q,V)**, CYP2C9 **(C,H,M,R,W)**, CYP2C19 **(D,I,N,S,X)** and CYP2J2 **(E,J,O,T,Y)**. Micromolar concentrations are expressed as log 10 (x-axis). Responses are expressed as fold change (log 2) over DMSO vehicle. Concentration-response curves were fit using tcpl_Lite. The red dotted lines represent the activity thresholds defined as 5 x bmad. Data represent individual wells from three independent experiments. The agonist response curves for all 128 chemicals tested across all ten CYP enzymes can be found in Supplementary Figure S3.

The optimized mRNA transfection protocol was used to retrofit the AR2 assay in both agonist and antagonist modes to retest the evaluation set of 128 chemicals across twelve parallel transfection “biogroups”: mock (no RNA), Bgal mRNA, and mRNA of ten human CYPs 1A2, 2A6, 2B6, 2C8, 2C9, 2C19, 2D6, 2E1, 2J2 and 3A4. The agonist mode dose-response curves for all 128 evaluation chemicals and assay controls across all ten CYP biogroups (with no RNA and Bgal graphed on each) are provided in Supplementary Figure S3 and the summary dose-response data is provided in Supplementary Table S4. Figure 6 highlights five chemicals across five CYP biogroups to illustrate the impact of CYP metabolism on AR agonist mode bioactivity. Four chemicals, including two estrogenic drugs, were bioactivated as more efficacious AR agonist metabolites were formed. 17α-estradiol produced a median AR agonist response 1.41-fold compared to vehicle (0.49 shown as log-2) across all biogroups (Figures 6A,C–E) except CYP2C8 (Figure 6B), where the maximal response increased to 2.17 (1.12 shown as log-2). Similarly, mestranol tested inactive across all biogroups (Figures 6F,G) with a median response of 1.12 compared to vehicle (0.16 shown as log-2), except CYP2C9 (Figure 6H) where the maximal response increased to 2.12 (1.08 shown as log-2). Flutamide was bioactivated by both CYP1A2 and CYP2C19 (Figures 6K,N). Like mestranol, the fungicide prochloraz was bioactivated by CYP2C9 (Figure 6R). These bioactivated compounds were the only four chemicals whose activity call changed with CYP metabolism. No active agonist chemicals were inactivated by CYP metabolism; however, several agonist chemicals exhibited CYP-specific reductions in potency. The androgenic drug danazol was a potent and efficacious AR agonist across biogroups (Figure 6U–Y) with a median AC_50_ value of 5.98 nM across all biogroups. CYPs 2C8, 2C9, and 2J2 considerably reduced the potency of danazol (Figure 6V-W, Y) increasing the AC_50_ values 9- to 35-fold to 214, 45.8, and 102 nM respectively.

**FIGURE 7:**
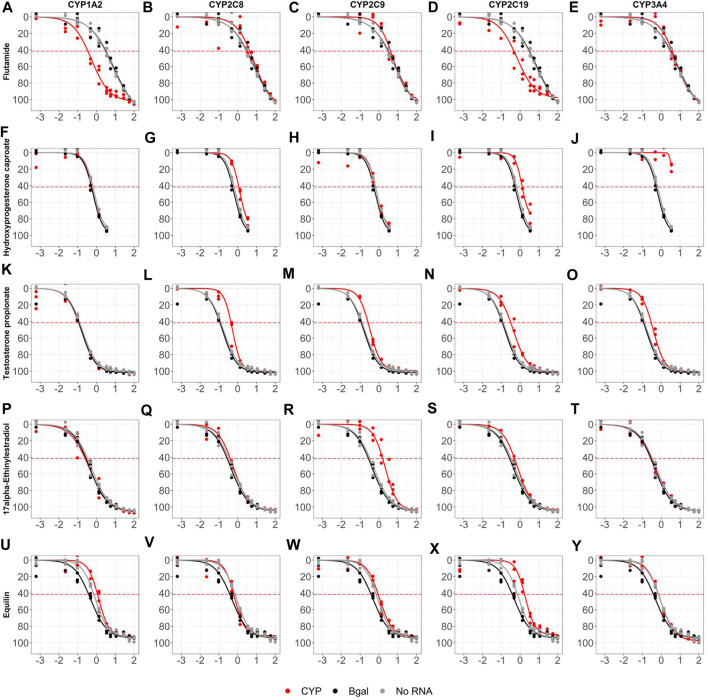
Sample retrofitted antagonist concentration-response curves for 128 evaluation chemicals. AR2 cells were transfected with mRNAs encoding beta-galactosidase (black), one of ten human CYP enzymes (red), or no RNA (grey) for 6 hours then exposed to 128 unique chemicals at concentrations ranging from 691 pM- 99.9 μM for 18 hours in antagonist mode. Shown are the dose-response profiles for Flutamide **(A–E)**, Hydroxyprogesterone caproate **(F–J)**, Testosterone propionate **(K–O)**, 17α-Ethinylestradiol **(P–T)** and Equilin **(U–Y)** in cells expressing one of five CYP enzymes: CYP1A2 **(A,F,K,P,U)**, CYP2C8 **(B,G,L,Q,V)**, CYP2C9 **(C,H,M,R,W)**, CYP2C19 **(D,I,N,S,X)** and CYP3A4 **(E,J,O,T,Y)**. Micromolar concentrations are expressed as log 10 (x-axis). Responses are expressed as % inhibition (% maximal positive control). Concentration-response curves were fit using tcpl_Lite. The red dotted lines represent the activity thresholds defined as 3 x bmad. Data represent individual wells from three independent experiments. The antagonist response curves for all 128 chemicals tested across all ten CYP enzymes can be found in Supplementary Figure S4.

The antagonist mode dose-response curves for all 128 evaluation chemicals and assay controls across all ten CYP biogroups (with no RNA and Bgal graphed on each) are provided in Supplementary Figure S4 and the summary dose-response data is provided in Supplementary Table S5. Figure 7 highlights five chemicals across five CYP biogroups to illustrate the impact of CYP metabolism on AR antagonist mode bioactivity. Flutamide was bioactivated by CYP1A2 (Figure 7A) replicating the results shown in Figure 5 and was also bioactivated in a nearly identical manner by CYP2C19 (Figure 7D). Nine chemicals had median maximal responses fall below the activity threshold in at least one biogroup (apparent inactivation), however most of these are not likely significant since the both the active and inactive biogroups are just above and below the threshold, respectively. The exception, hydroxyprogesterone caproate (Figures 7F–J) was a strong (90.9% median maximal response) and potent (median AC_50_ = 732 nM) AR antagonist in all but one biogroup, but its potency was reduced nearly 2-fold with CYP2C8 and CYP2C19 (Figures 7G,I), and CYP3A4 (Figure 7J) completely inactivated hydroxyprogesterone caproate as an AR antagonist dropping its maximal response to 14.7%. Similarly, 11 chemicals had median maximal response rise above the activity threshold in at least one biogroup (apparent bioactivation), but most of these are likely confounded by increased cytotoxicity and not statistically significant. The potencies of several AR antagonists were reduced in a CYP-specific manner. Testosterone propionate was a strong antagonist, but its AC_50_ value decreased more than 2-fold from a median of 169 nM across all biogroups to 529 nM with CYP2C8 (Figure 7L), 333 nM with CYP2C9 (Figure 7M), 440 nM with CYP2C19 (Figure 7N), and 380 nM with CYP3A4 (Figure 7O). 17α-ethinylestradiol was another potent antagonist (median AC_50_ = 509 nM) whose potency was reduced 4-fold by CYP2C9 (Figure 7R; AC_50_ = 2.15 µM). As a final example, CYP2C19 reduced the potency of equilin (Figure 7X).

## Discussion

There are thousands of commercially used chemicals for which endocrine disruption data does not exist, and the current EDSP battery of assays is too time- and resource-intensive to practicably solve this challenge. The ToxCast and Tox21 programs have implemented HTS assays and computational approaches to identify endocrine-active chemicals rapidly and inexpensively. The most successful project to date has been the ToxCast ER Model for bioactivity which integrates data from 18 ER HTS assays to predict ER agonist and antagonist activity in EDSP Tier 1 screening assays (Browne et al., 2015). In 2015, the EPA announced it would accept the model scores for more than 1,800 chemicals as an alternative to some EDSP Tier 1 assays (Federal Register, 2002). In parallel, an AR model using 11 HTS assays was developed as a possible alternative to additional EDSP Tier 1 assays (Kleinstreuer et al., 2017).

Subsequent efforts have examined whether data from a smaller subset of the original 11 HTS assays and three additional assays can be used without sacrificing model predictivity (Judson et al., 2020). Those efforts showed that as few as six assays for AR agonists and five for antagonists could be used and still achieve balanced accuracies (BA) of 95% or greater compared to the full 11-assay model. The highest-performing minimal agonist models used at least one cell-free receptor binding assay, a cofactor recruitment (dimerization and translocation) assay, a transcriptional activation reporter assay, and a cell proliferation assay. The highest-performing minimal antagonist models also used a cell-free receptor binding and a cofactor recruitment assay plus two or three antagonist-mode transcriptional deactivation assays with non-redundant reporter technologies. While not sufficiently predictive alone, the OT and UPitt dimerization assays proved to be the most indispensable to model predictivity evidenced by their inclusion in the highest-scoring models, especially for AR antagonists. Of the six models with BA > 95% which included only five assays, all six (100%) used the UPitt antagonist mode data and four (67%) also used the 16-h OT data. As the UPitt assay failed to detect any reference agonist and missed every androgenic drug, its exclusion in the top agonist models is unsurprising. However, the 16-h OT data was used in four of the six (67%) models with BA > 95% which included six assays.

While noting the importance of the dimerization assays, Judson *et al.* state that “not all of the AR assays are currently available through the original sources, so in order to implement subset models, one may need to find existing or develop new assays that have similar behavior to the ones selected for the optimal subsets.” They also state that one of the outstanding challenges to using *in vitro* data to predict *in vivo* outcomes is the lack of xenobiotic metabolism. Indeed, both the OT and UPitt assays are no longer available through their original sources, driving the need to develop a replacement AR PPI assay(s). Ideally, a replacement assay would: 1) have protocols for both agonist and antagonist modes, 2) provide AR bioactivity data that maintains or improves the predictivity of the computational model, 3) possess equal or superior throughput to the legacy assays, 4) be made widely accessible for interlaboratory validation, 5) not require cost-prohibitive reagents or detection equipment, and 6) have the potential to incorporate xenobiotic metabolism inexpensively and without sacrificing throughput. This study details the development of AR2, a stable cell-based AR homodimerization assay, and presents its evaluation using 128 chemicals including all but one of the 44 reference chemicals used to develop the AR computational model as well as a demonstration of its metabolic retrofit using an mRNA transfection approach.

The AR2 assay was executed in agonist and antagonist modes and data compared to that derived from the single mode OT and bimodal UPitt assays used to build the AR computational model. Binary classifications using 43 of the reference chemicals used to train the AR model can facilitate useful comparisons of AR2 assay performance to the OT and UPitt assays. There were 24 agonist reference chemicals in the evaluation set where eight were active (“strong”, “moderate” or “weak”), and twenty inactive. All 24 were tested in the AR2 and OT assays and all but one (17β-trenbolone) were tested in the UPitt assay. The AR2 agonist assay had the highest balanced accuracy (91.7%) compared to OT at eight (75.0%) and 16 h (62.5%). The UPitt agonist assay performed poorly (BA = 43.5%) largely due to its inability to detect active agonists (i.e., sensitivity). The AR2 agonist assay misclassified just 2 of the 24 reference agonists (17α-estradiol and prochloraz), and both were false positives with maximal activities nearly equal to the activity threshold.

While the high AR2 agonist BA is impressive, the antagonist performance is of greater interest since AR antagonist activity is more prevalent among xenobiotic chemicals. There were 24 antagonist reference chemicals in the evaluation set where 17 were active (“strong”, “moderate” or “weak”), and seven inactive. All 24 were tested in the AR2 and OT assays and all but one (hydroxyflutamide) were tested in the UPitt assay. The UPitt antagonist assay had the highest BA (95.7%), correctly classifying 15 of 16 reference antagonists and all seven inactive chemicals, while only misclassifying spironolactone as a false negative. The AR2 antagonist assay had a higher BA (79.2%) than did OT at eight (62.5%) or 16 h (75.0%). Although the AR2 antagonist assay performed well, the BA from this experiment suffered due to two factors. First, there were three reference inactive chemicals misclassified as false positives in the antagonist mode: 17-methyltestosterone, 4-androstene-3,17-dione, and testosterone propionate. This lowered the AR2 sensitivity to 57.1% (compared to 100% in UPitt antagonist assay) given there were a total of seven inactive chemicals. Secondly, there were two reference antagonists misclassified by AR2 in antagonist mode. In the larger 128-chemical evaluation, hydroxyflutamide (Figure 3N) failed to elicit an active response which contrasts the strong antagonist response observed in two independent experiments conducted earlier in this study (Figure 2B; Figure 5). It is unclear whether the UPitt antagonist assay would have correctly classified hydroxyflutamide since it was the only reference antagonist untested in that assay. Similarly, the AR2 antagonist assay misclassified cyproterone acetate in the evaluation screen (Figure 3M) although a strong response was observed in a previous experiment (Figure 2B). Since both compounds evoked a strong and potent antagonist response in the AR2 assay in the previous experiments where acoustic source plates were populated independently from those used in the larger screen, the inconsistent responses for both hydroxyflutamide and cyproterone acetate are likely due to chemical management failures and not any insufficiencies in the AR2 assay. This example does serve to highlight the reliance on test chemical fidelity in the interpretation of chemical bioactivity data. If both hydroxyflutamide and cyproterone acetate were recognized as active antagonists based on the results shown in Figure 2B and Figure 5, then the AR antagonist BA would improve from 79.2% to 87.5%. The reason three androgens listed as reference inactive chemicals tested active in antagonist mode is not clear. AR antagonists that act as agonists in the absence of competing androgens has been well documented (Wong et al., 1995; Culig et al., 1999); however, reports of partial AR agonists acting as antagonists are comparatively limited (Chen et al., 2004; Schlezinger et al., 2020).

For antagonist mode testing, a near maximally effective concentration (EC_95_) of strong androgen agonist (R1881) was co-administered with test compounds or controls to stimulate assay signal. The use of a strong agonist reference in antagonist mode testing is standard practice for *in vitro* AR assays. The University of Pittsburgh assays used 20 mM DHT for antagonist mode testing (Hua et al., 2014) while the Odyssey Thera (OT) did not have a dedicated antagonist mode. All three OECD-validated AR transcriptional activation (ARTA) reporter gene assays also use DHT (0.3–0.5 nM) to stimulate signal for attenuation in antagonist mode (https://www.oecd.org/env/test-no-458-stably-transfected-human-androgen-receptor-transcriptional-activation-assay-for-detection-of-androgenic-agonist-9789264264366-en.htm). Perhaps more important than the selection of agonist is the concentration of agonist used to stimulate assay signal in antagonist mode. Using a higher concentration such as the EC_95_ produces the highest signal-to-background but introduces the possibility that weaker or less potent antagonists might go undetected. Using a lower concentration of agonist may improve assay sensitivity to better detect weaker competitive inhibitors. Formal implementation of this assay for ToxCast testing will use two parallel agonist concentrations for this reason.

The AR2 assay will made available to external partners for an interlaboratory evaluation, and this process is critical to independently assess the utility, transferability, and reliability of any assay. Although the AR2 assay was conducted in a HTS 384-well format with automated liquid dispensing in this study, the excellent rZ’ factors for both agonist (0.94) and antagonist (0.84) modes suggest there is ample signal-to-background for further miniaturization to higher-throughput 1536-well format. The homogenous (add-only) protocol which also includes the mRNA transfection method to retrofit the assay with CYP activity is also preferable for HTS applications. Yet the AR2 assay does not require automated liquid dispensers and can be manually pipetted for lower-throughput, larger volume formats like 96-well applications and thus is highly transferrable. The AR2 assay requires no expensive detection reagents or equipment, only a microplate reader capable of luminescent and fluorescent detection which is relatively inexpensive compared to the imaging systems used by the legacy assays.

A two-part strategy to address the limitation of xenobiotic metabolism in *vitro* assays was recently described by Thomas *et al.* (U.S. EPA, 2002a) One part of that strategy utilizes mRNA transfection to induce CYP activity within cell-based assays to model target tissue metabolism. The feasibility of this approach was demonstrated using a cytotoxicity assay with mRNA-transfected HEK293T cells to produce CYP-specific shifts in bioactivity (DeGroot et al., 2018). This method was adapted for use in HepG2 cells with the AR2 assay. Since cells differ in their amenability to mRNA transfection, susceptibility to the cationic lipid reagents used and endogenous levels of POR expression, the optimization steps outlined in DeGroot *et al.* must be conducted when adapting a new cell background for this retrofit approach. Prior to this study, the protocol was optimized for maximal CYP activity in HepG2 cells. HepG2 were more tolerant of mRNA lipid particles than HEK293T such that twice as much mRNA could be delivered per well than in the previous study. HepG2 cells also require less co-transfected POR mRNA to maximize CYP activity, presumably because HepG2 cells endogenously express more POR than HEK293T. The increased mRNA delivery and reduced requirement for POR mRNA made HepG2 cells a more hospitable background for CYP expression than HEK293T and subsequently the better cell choice for the AR2 assay. Cell type continuity with the OT and UPitt assays which utilized HEK293T and U-2 OS cells, respectively, would have facilitated a more direct comparison of assay designs by removing cell type difference as a confounding issue; however, maximizing the potential for CYP expression and observation of metabolically induced bioactivity changes was the preeminent consideration in cell choice.

Several other significant changes were made to the original mRNA transfection protocol. A newer mRNA cap analog known as CleanCap® was evaluated and found to increase CYP activity over the ARCA-capped mRNA used previously (data not shown). Also, the methylcytosine (5 mC) substitution used previously was omitted during the CleanCap® mRNA synthesis for this study which alternatively used only a pseudouracil substitution. In the previous study, the CYP1A2 open reading frame (ORF) was found to harbor an unreported G to D mutation at amino acid 81 which was implicated in the lack of CYP1A2 activity observed using both a luminogenic substrate and phenacetin. Prior to this study, the CYP1A2 ORF was corrected to the canonical wild-type sequence by site-directed mutagenesis. The wild-type CYP1A2 used in this study performed as expected, converting >80% of the parent flutamide to 2-hydroxyflutamide over an 18-h exposure (Figure 5).

The impact of retrofitting the AR2 assay with CYP metabolism was restricted to only a few chemicals, most of which are highlighted in Figures 6, 7. This study only examined 128 test chemicals which were not selected based on their potential for CYP metabolism, but rather their known endocrine activities. Non-etheless, CYP metabolism did bioactivate four chemicals in agonist mode increasing the number of agonist actives from 30 to 33, a 10% increase. The discrepancy was 17α-estradiol which tested marginally active without metabolism and then inactive across all biogroups during the retrofitted screen except CYP2C8 which bioactivated it to a weak androgen (Figure 6B). Another estrogenic chemical, mestranol, tested negative in the OT and AR2 assays except when retrofit with CYP2C9 (Figure 6H). Mestranol is known to be converted by CYP2C9 to its active metabolite, ethinyl estradiol (Hu et al., 2015), but since ethinyl estradiol was not tested in this study, it is not known if this is the active metabolite of mestranol to induce AR activity. Prochloraz is an efficacious AR antagonist, but inactive as an agonist across all biogroups except CYP2C9 which bioactivated it to a marginally active agonist (Figure 6R). Danazol is an efficacious agonist (Figure 6U), but its potency was diminished by CYP2C8, CYP2C9 and CYP3A4 (Figure 6V-W, Y). CYP3A4 is known to metabolize danazol to the less androgenic 2-hydroxymethylethisterone. Danazol also reportedly inhibits both CYP2C8 and CYP2C9 (Al-Badr, 2022), but this study showed these two enzymes metabolized danazol and impacted its potency as an androgen. Still, no reported CYP2C8 or CYP2C9 metabolite(s) are found in the literature. Hydroxyprogesterone was a potent and efficacious agonist inactivated by CYP3A4 (Figure 7J) and is known to be primarily metabolized by CYP3A4 (Sharma et al., 2010). The examples in this study of CYP-shifted AR bioactivity are few, given the relatively small number of chemical by CYP interactions tested, but serve to highlight the importance of xenobiotic metabolism for *in vitro* toxicity assays.

The introduction of xenobiotic metabolism into cell-based assays does raise several unforeseen issues. The first is the paradox posed by testing chemicals solubilized in DMSO, a solvent known to inhibit CYP activity (Easterbrook et al., 2001). DMSO dissolves a wide range of chemicals from relatively polar to relatively non-polar compounds and thus is the solvent of choice for diverse chemical libraries like those used in ToxCast and Tox21. In this study, DMSO concentrations were kept at 0.1% to minimize (not eliminate) CYP inhibition, and this may be the most practical approach to address this issue; however, this target DMSO concentration limit requires stock solutions of 50 mM to achieve the normal 100 µM final test concentrations, and chemicals insoluble at this stock concentration may be beyond the range of testing to 100 µM with CYP metabolism. Testing chemicals at lower top concentrations is possible, but runs the risk of achieving a lower %CYP V_max_ (slower metabolism) as the substrate concentration falls relative to the K_m_.

Positive controls for CYP activity (independent of assay controls) are another unresolved technical challenge. One solution would be to identify chemicals known to be transformed by specific CYP enzymes to metabolites with altered bioactivity in an endpoint of interest (e.g., AR dimerization) like flutamide. However, there are few examples of such chemicals for most toxicity endpoints. An alternative approach would be to find CYP-metabolized chemicals that elicit a response that can be measured independently (or in parallel) to the endpoint of interest. Cytotoxicity may be the best candidate as it is a response available to all cell-based assays that can be measured using a pre- or *post hoc* multiplexed endpoint such as Alamar Blue in this study. Using cytotoxicity CYP-specific controls would obviate the need to search for pathway-specific CYP controls. Conversely, the assay controls used would ideally be unaffected by CYP metabolism. The AR2 controls (R1881, bicalutamide and dichlone) were all unimpacted by CYP metabolism.

Interpretation of retrofitted data is another complicated issue. Criteria are needed to clearly define when bioactivation or inactivation has occurred. Once defined, there is still the question of biological significance, i.e., should the bioactivation of an otherwise inactive chemical with a single CYP biogroup (e.g., mestranol with CYP2C9) change its activity designation? It should also be made plain that an absence of CYP-shifted bioactivity in a given assay should not be interpreted as evidence that a chemical is not CYP-metabolized. CYP-shifted bioactivities are only manifested after at least two criteria have been met: 1) the chemical is metabolized by a CYP enzyme, and 2) the metabolite(s) after any subsequent Phase II metabolism or other biochemical transformation elicits a disparate response to the parent chemical. Flutamide is known to be metabolized by CYP1A2 and also CYP2C19 to hydroxyflutamide which is much more anti-androgenic, but also 4-nitro-3-(trifluoromethyl)phenylamine (Flu-1) by CYP3A4 (Kang et al., 2008). It is highly likely flutamide is converted to Flu-1 by CYP3A4 in this study; however, Flu-1 may have similar anti-androgenic properties as flutamide and therefore no shift in AR bioactivity is observed.

A formal re-evaluation of the AR2 assay in addition to several OECD-approved AR transactivation assays (OECD iLibrary, 2002) will be the focus of a future study that re-optimizes the minimal assay composition of a new AR computational model. It is expected that the AR2 assay will serve as a valuable replacement for the OT and UPitt assays. Based on reference chemical classification, AR2 was superior to the OT assay at both timepoints in identifying both agonists and antagonists; moreover, the AR2 can be run in both agonist and antagonist modes while the OT assay is unimodal. The AR2 assay is far superior to the UPitt assay in identifying AR agonists and likely similar in their near-perfect detection of antagonists. The performance, availability, transferability, throughput, cost-effectiveness, and amenability to retrofit with CYP metabolism of the AR2 assay is ideally suited to support future *in vitro* endocrine testing.

## Data Availability

The datasets presented in this study can be found in online repositories. The names of the repository/repositories and accession number(s) can be found below: https://github.com/SimmonsLabEPA/AR2-Assay-Method.git.
