# Supplementary material for: High-throughput AR dimerization assay identifies androgen disrupting chemicals and metabolites
Source: Front Toxicol. 2023 Apr 4;5:1134783. doi: 10.3389/ftox.2023.1134783 (PMC10112521; doi:10.3389/ftox.2023.1134783)
Supplement: Supplementary file 1 [file DataSheet2.PDF]

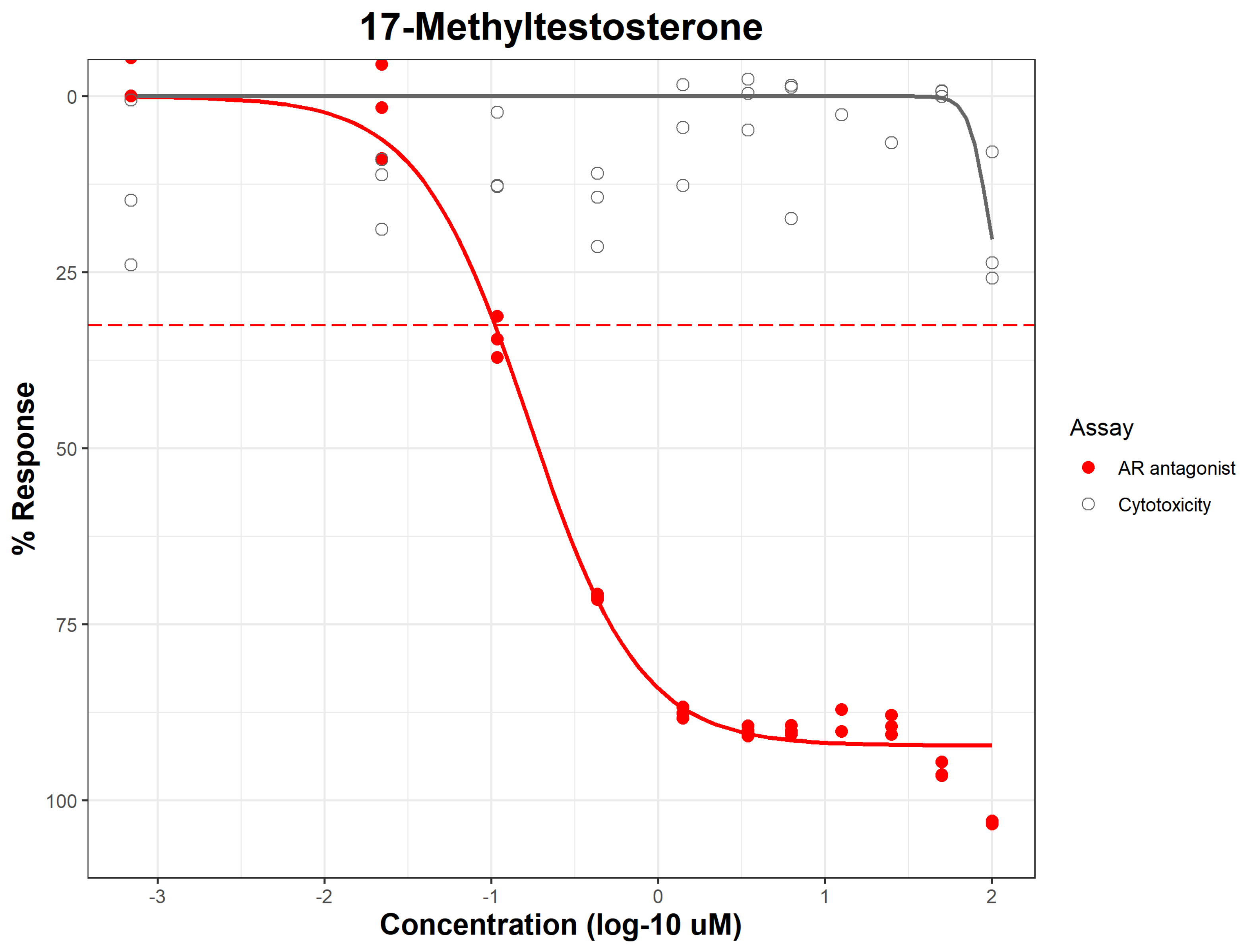
17-Methyltestosterone
% Response
0
25
50
75
100
-3
-2
-1
0
1
2
Concentration (log-10 uM)
Assay
AR antagonist
Cytotoxicity

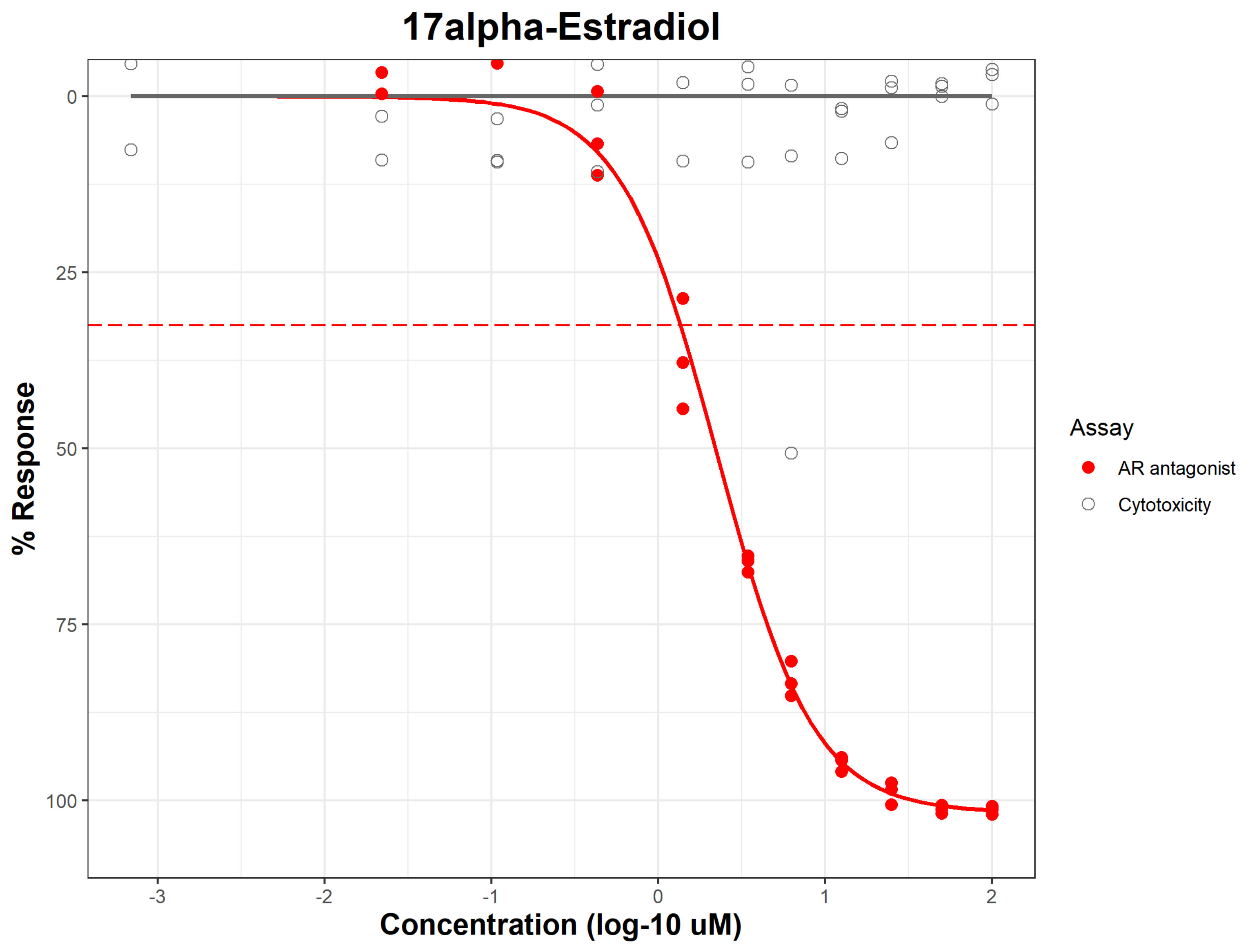

17alpha-Estradiol
% Response
0
25
50
75
100
-3
-2
-1
0
1
2
Concentration (log-10 uM)
Assay
AR antagonist
Cytotoxicity

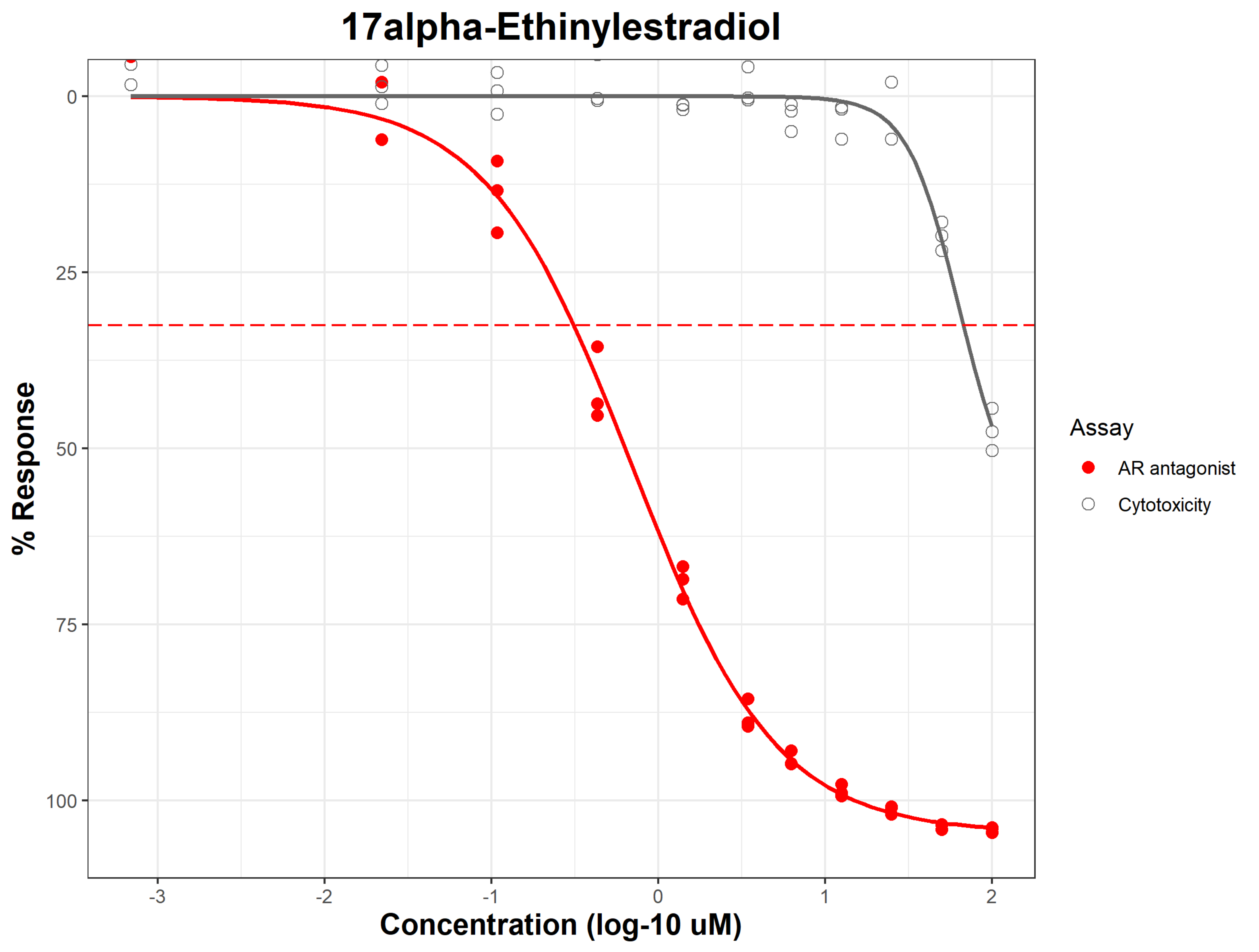

17alpha-Ethinylestradiol
% Response
0
25
50
75
100
Concentration (log-10 uM)
-3
-2
-1
0
1
2
Assay
AR antagonist
Cytotoxicity

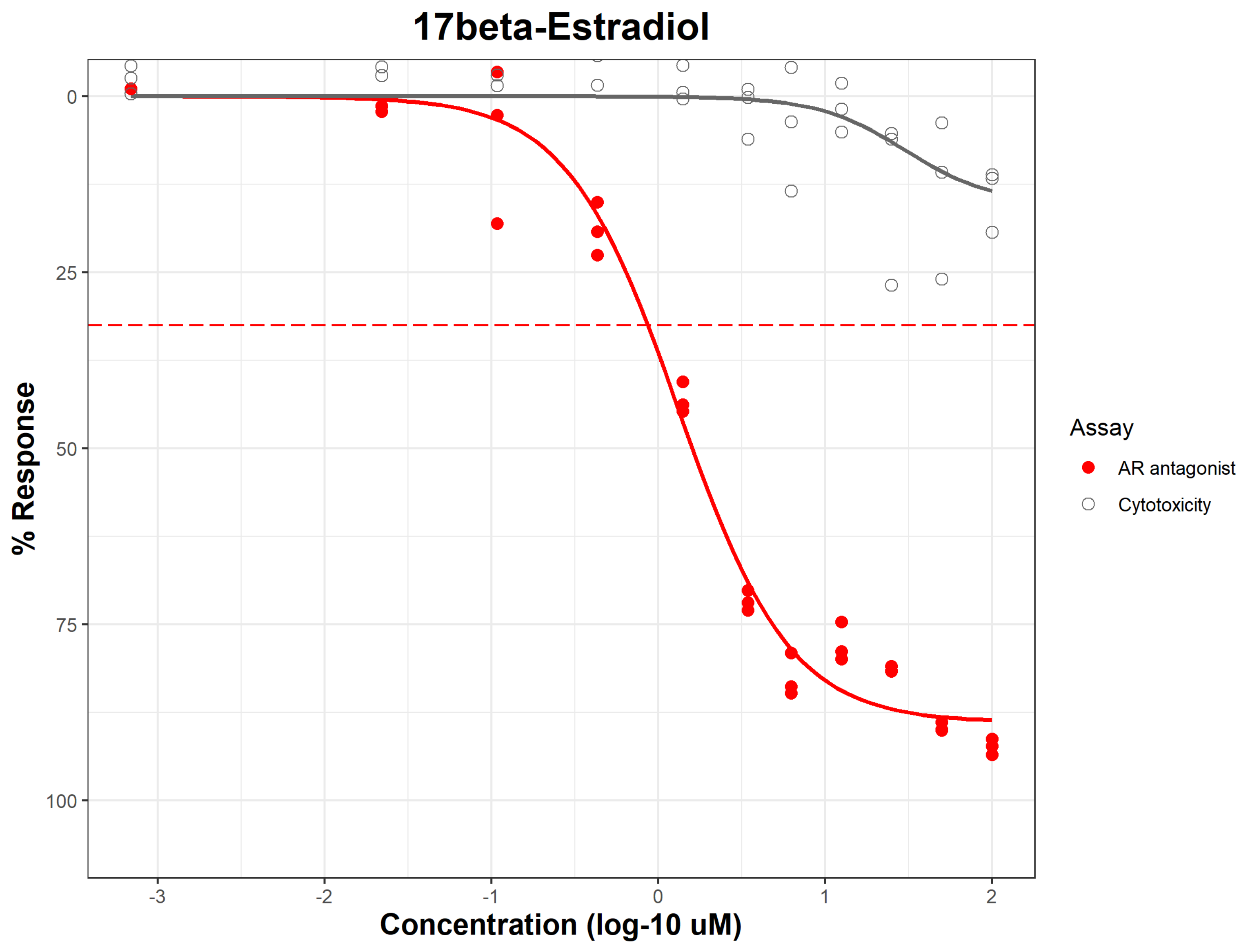
17beta-Estradiol
% Response
0
25
50
75
100
-3
-2
-1
0
1
2
Concentration (log-10 uM)
Assay
AR antagonist
Cytotoxicity

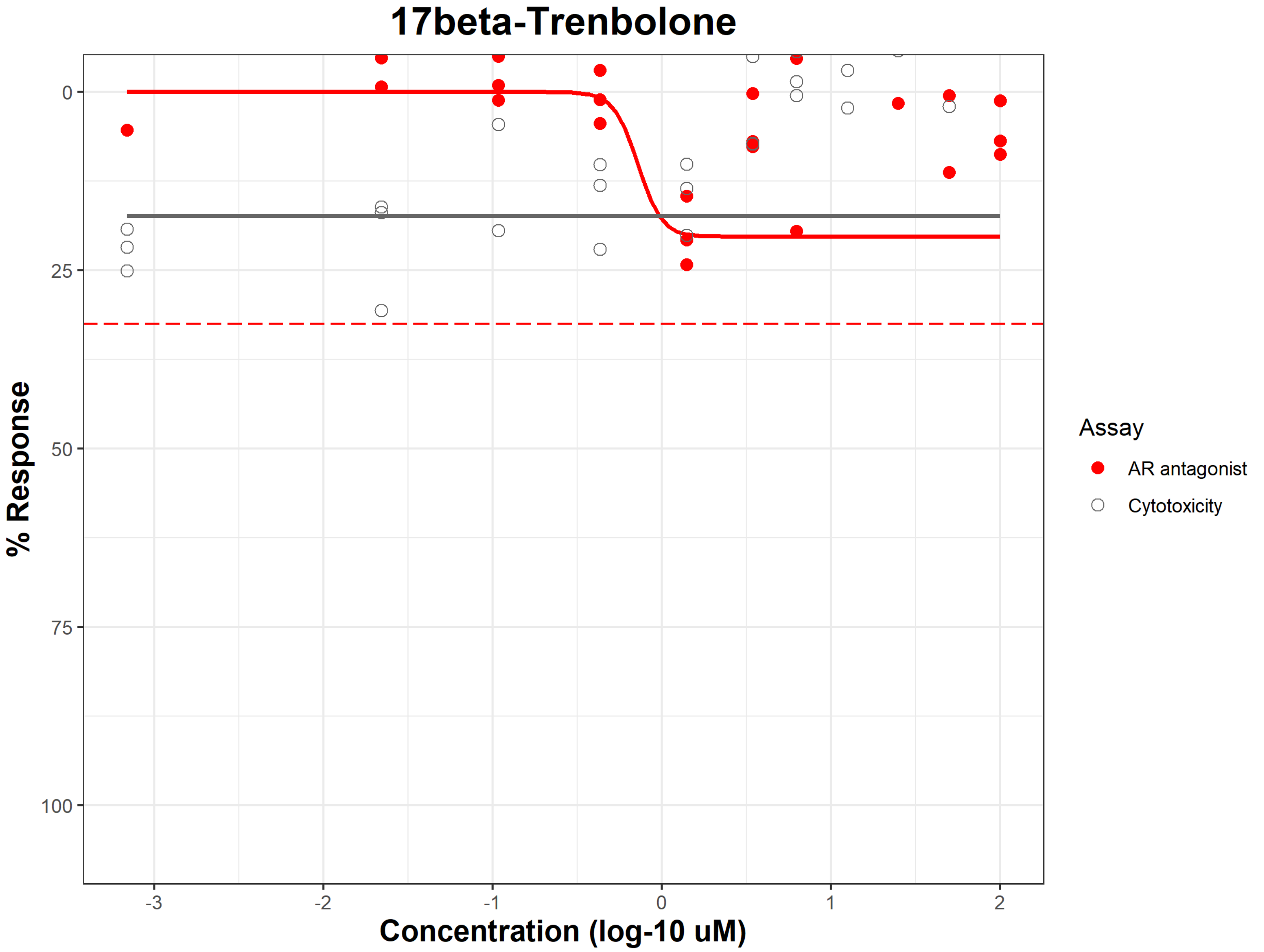

17beta-Trenbolone
% Response
0
25
50
75
100
-3
-2
-1
0
1
2
Concentration (log-10 uM)
Assay
AR antagonist
Cytotoxicity

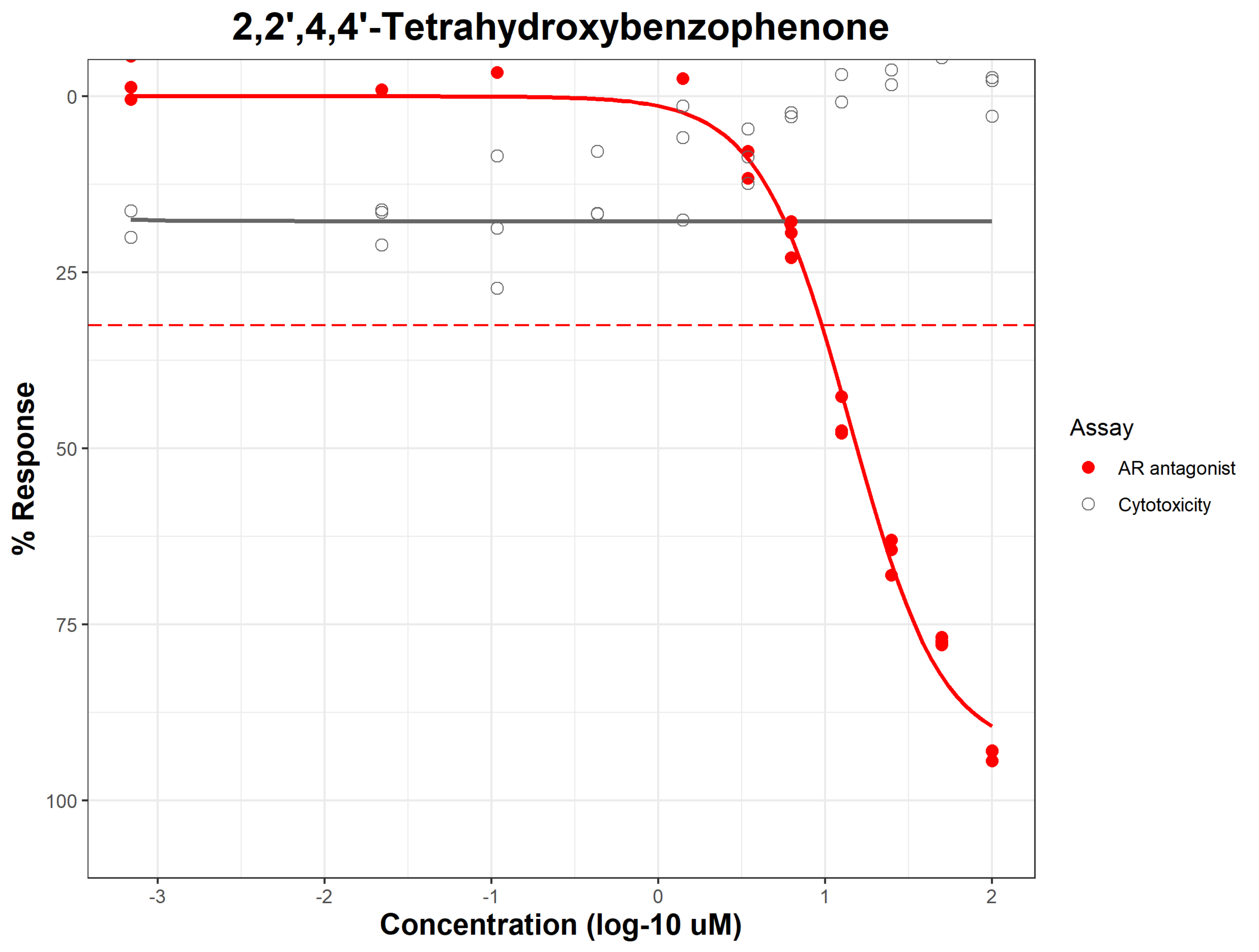

2,2',4,4'-Tetrahydroxybenzophenone
% Response
0
25
50
75
100
Concentration (log-10 uM)
-3
-2
-1
0
1
2
Assay
AR antagonist
Cytotoxicity

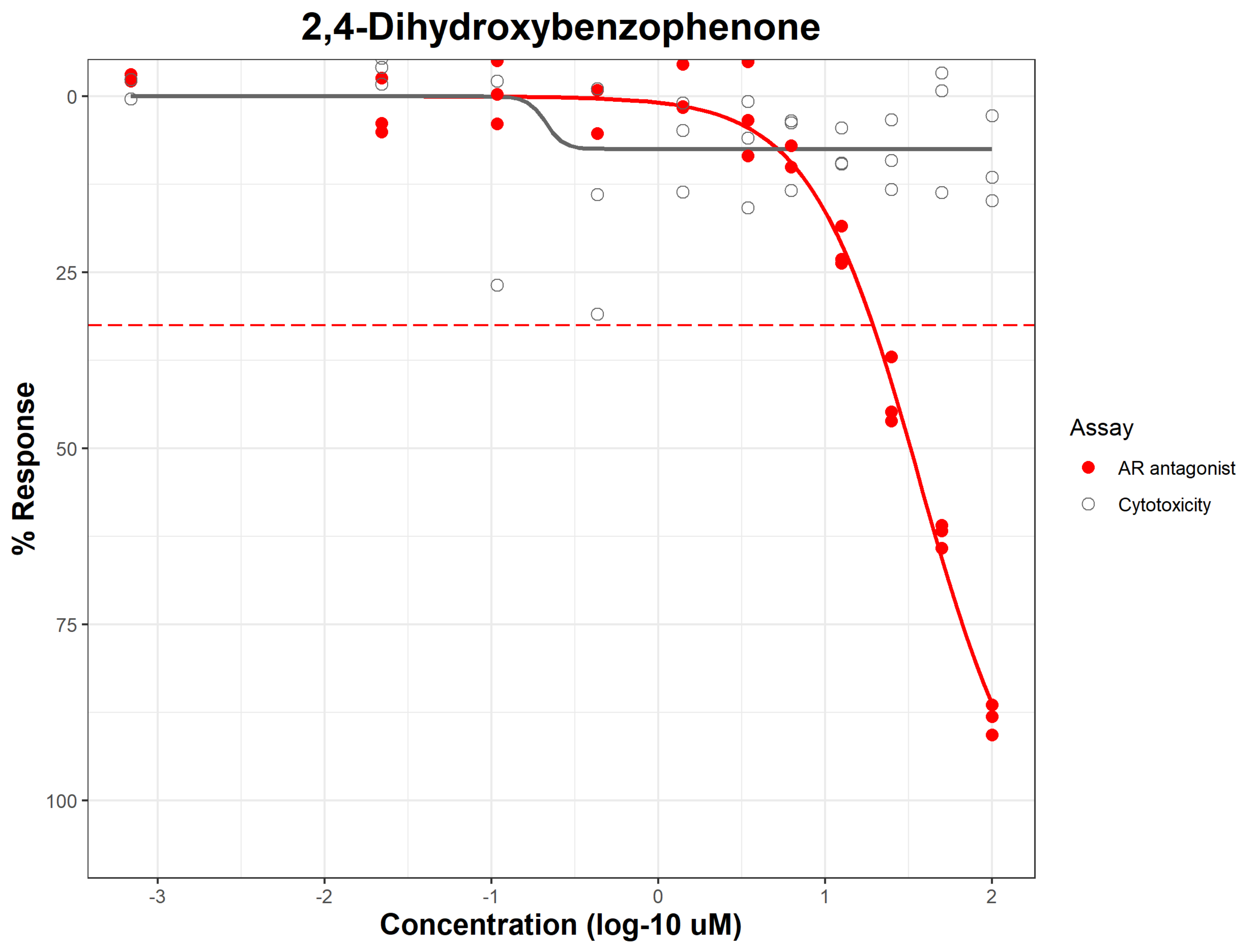

2,4-Dihydroxybenzophenone
% Response
0
25
50
75
100
-3
-2
-1
0
1
2
Concentration (log-10 uM)
Assay
AR antagonist
Cytotoxicity

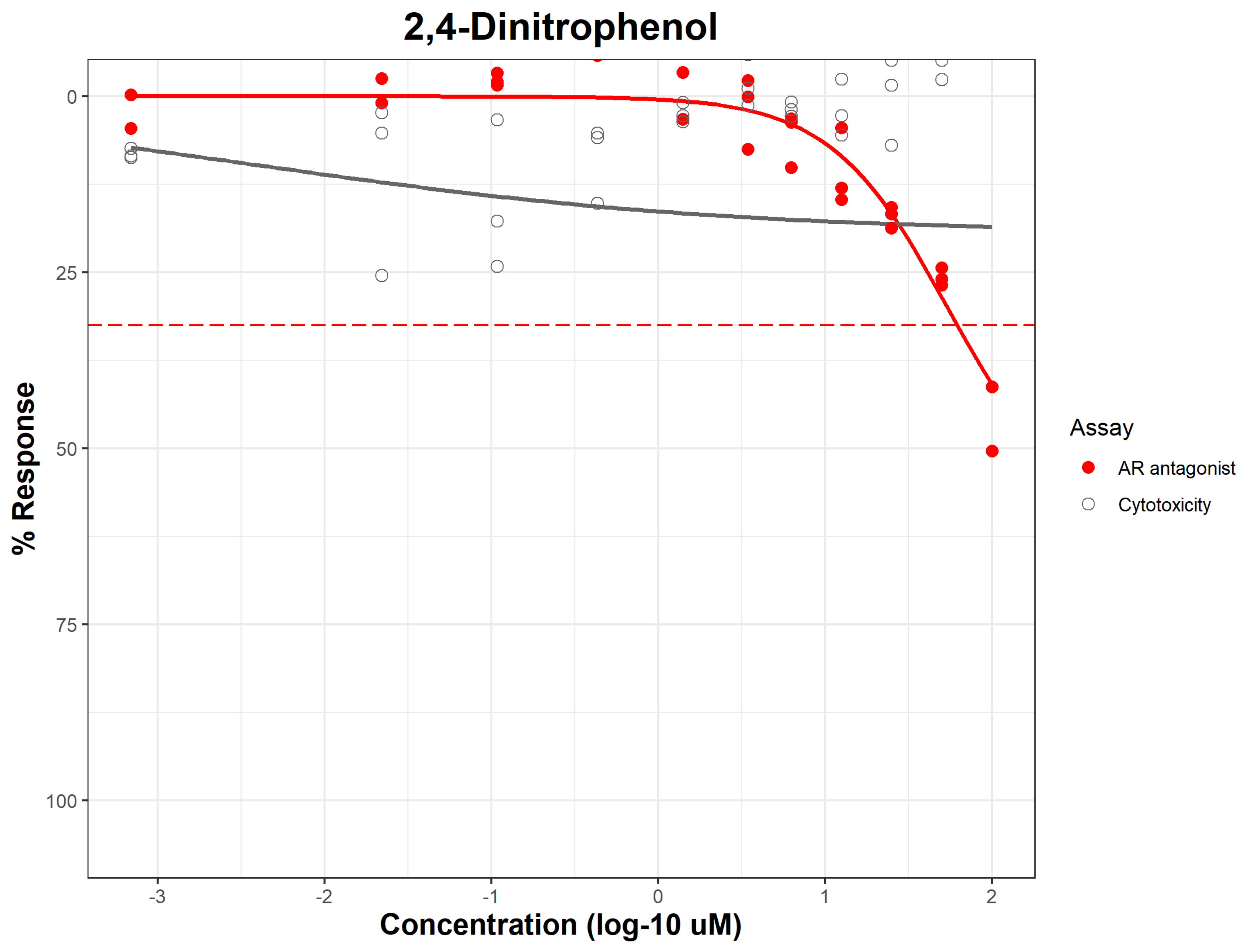
2,4-Dinitrophenol
% Response
0
25
50
75
100
-3
-2
-1
0
1
2
Concentration (log-10 uM)
Assay
AR antagonist
Cytotoxicity

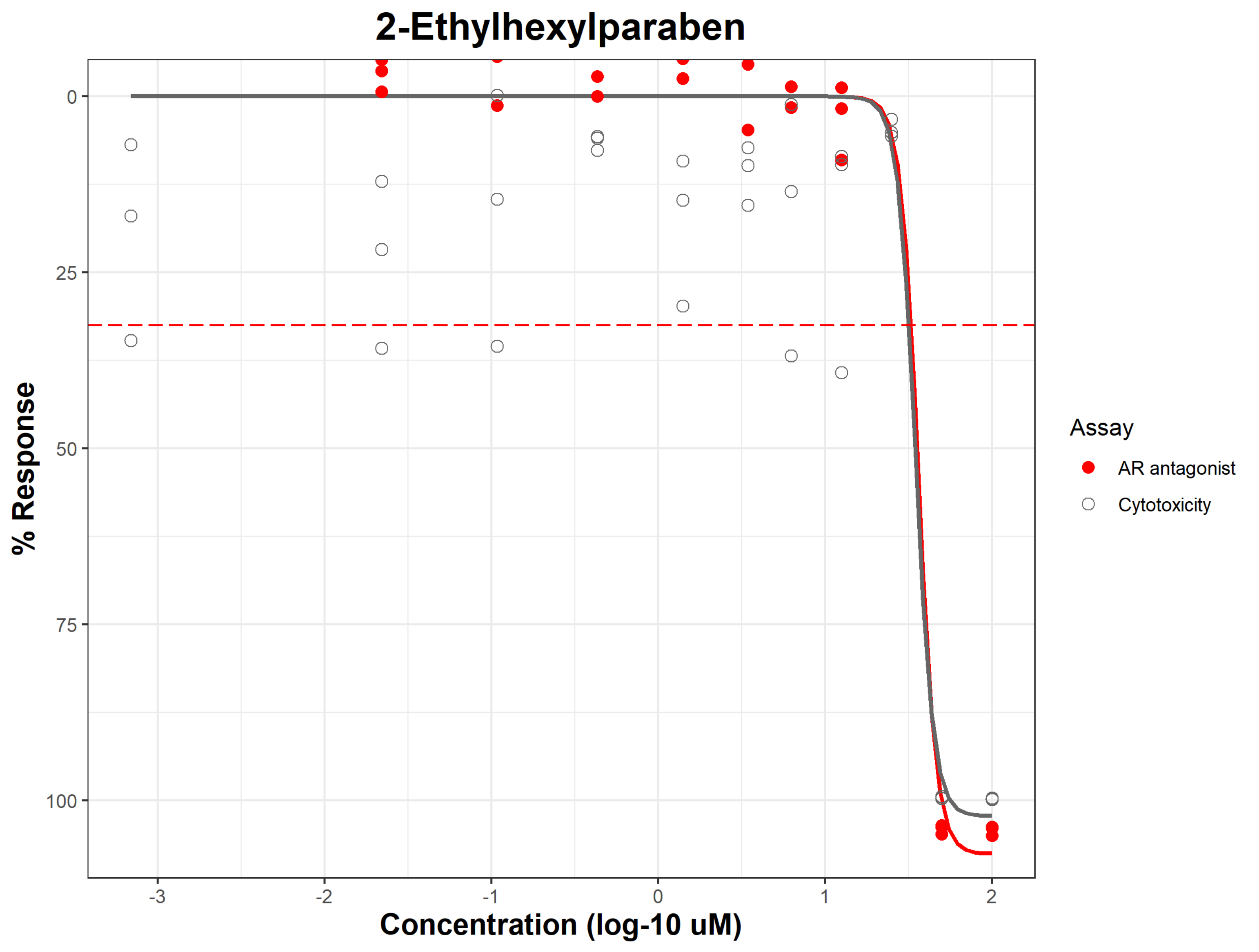

2-Ethylhexylparaben
% Response
0
25
50
75
100
-3
-2
-1
0
1
2
Concentration (log-10 uM)
Assay
AR antagonist
Cytotoxicity

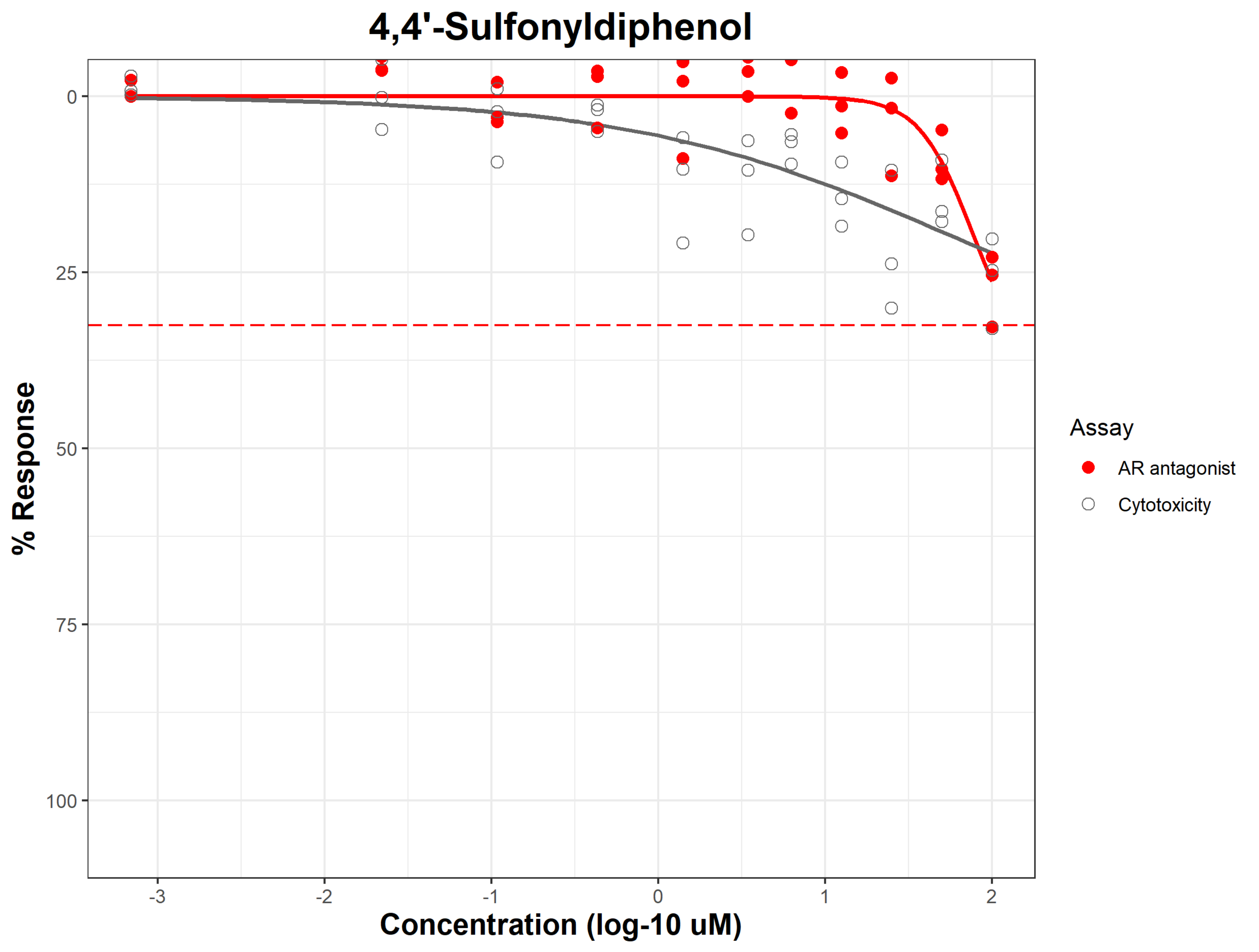

4,4'-Sulfonyldiphenol
% Response
0
25
50
75
100
-3
-2
-1
0
1
2
Concentration (log-10 uM)
Assay
AR antagonist
Cytotoxicity

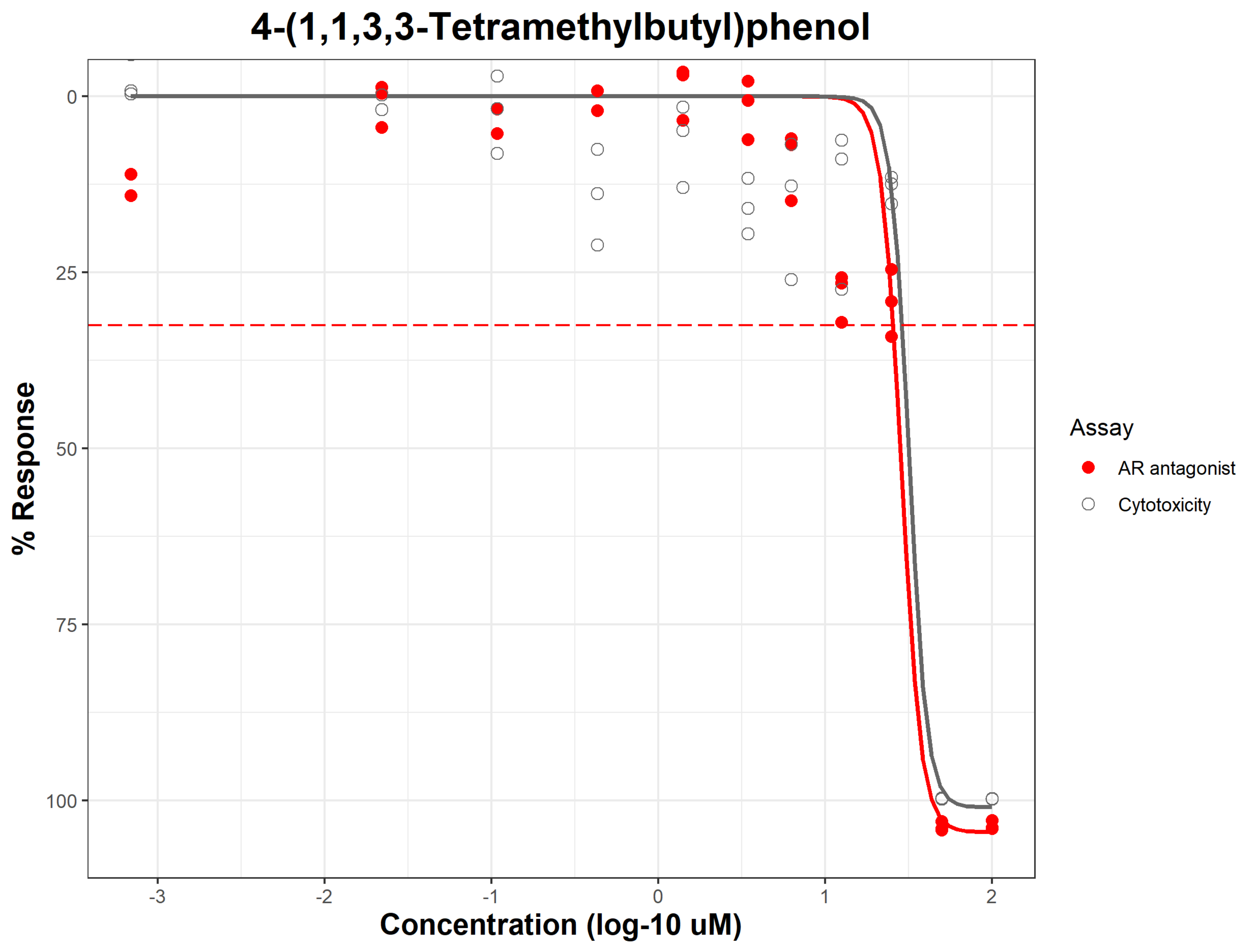
4-(1,1,3,3-Tetramethylbutyl)phenol
% Response
0
25
50
75
100
Concentration (log-10 uM)
-3
-2
-1
0
1
2
Assay
AR antagonist
Cytotoxicity

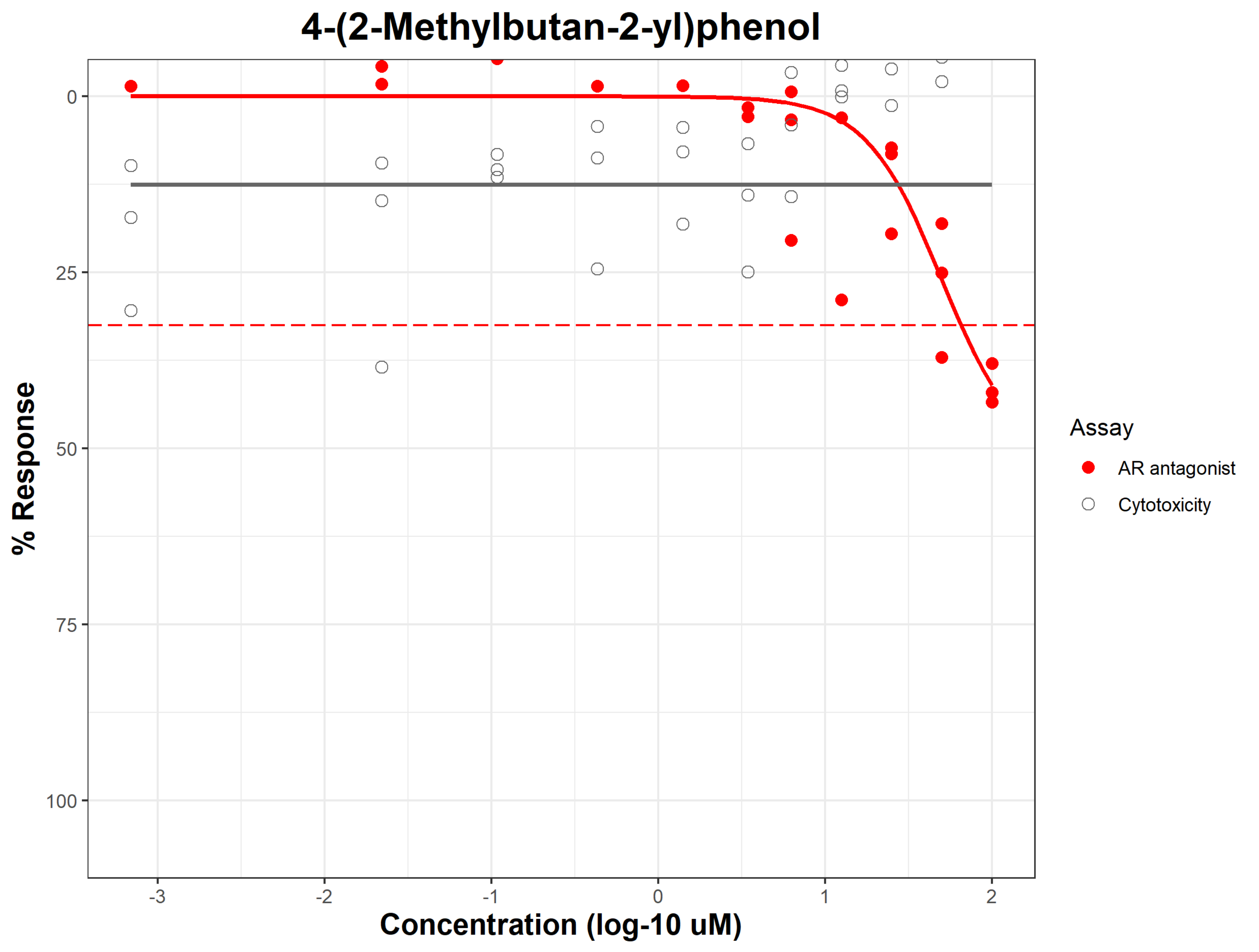
4-(2-Methylbutan-2-yl)phenol
% Response
0
25
50
75
100
Concentration (log-10 uM)
-3
-2
-1
0
1
2
Assay
AR antagonist
Cytotoxicity

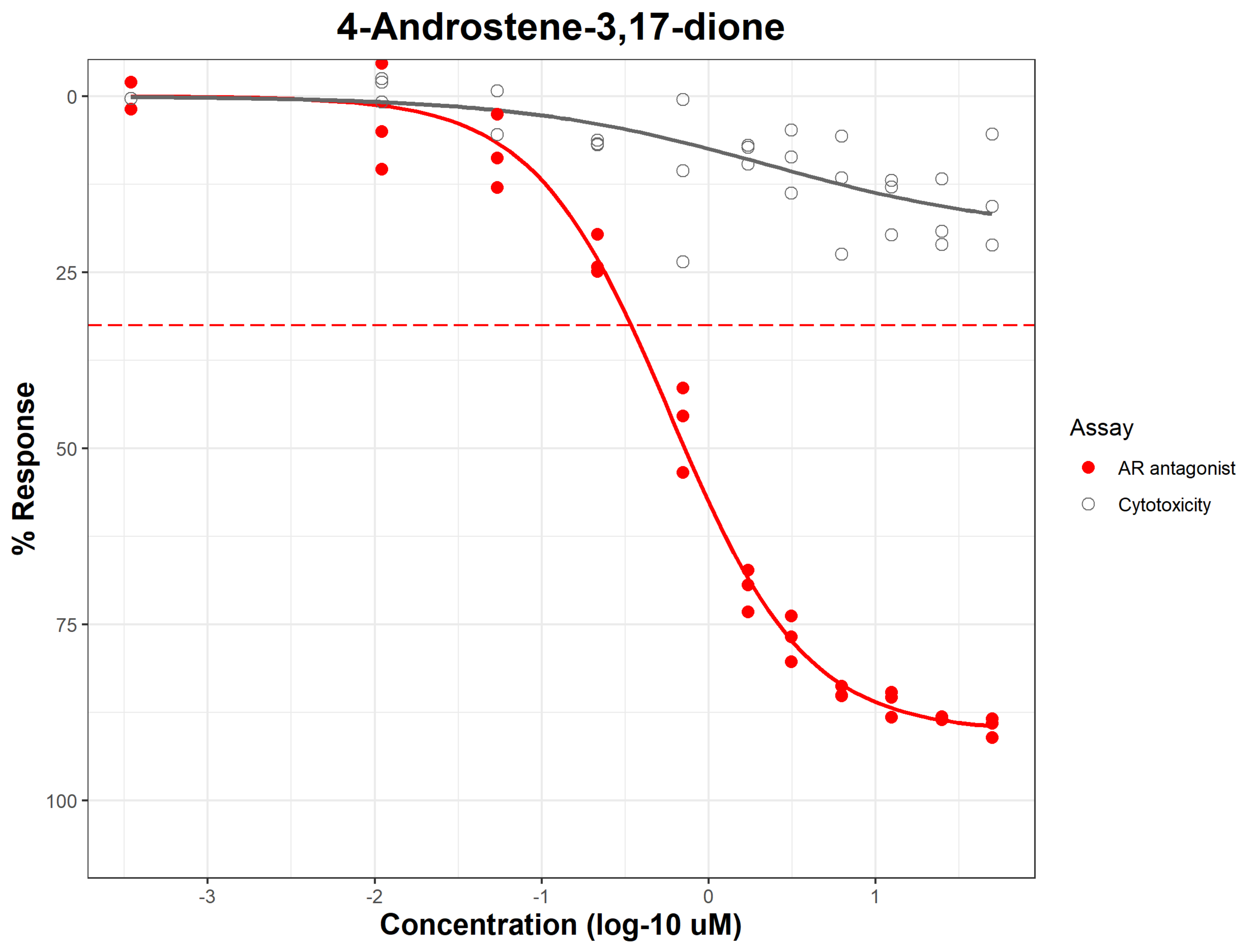

4-Androstene-3,17-dione
% Response
0
25
50
75
100
-3
-2
-1
0
1
Concentration (log-10 uM)
Assay
AR antagonist
Cytotoxicity

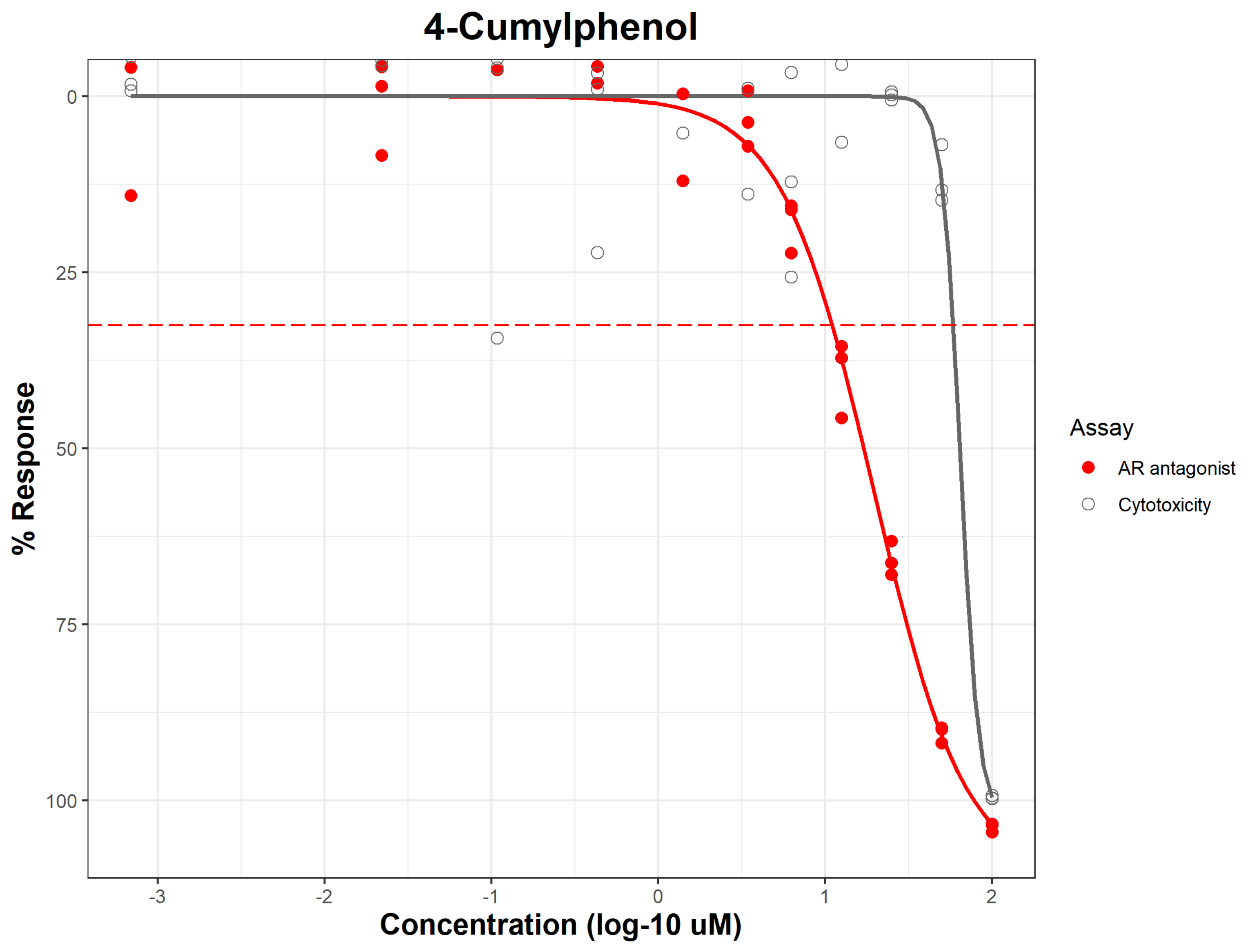

4-Cumylphenol
% Response
0
25
50
75
100
Concentration (log-10 uM)
-3
-2
-1
0
1
2
Assay
AR antagonist
Cytotoxicity

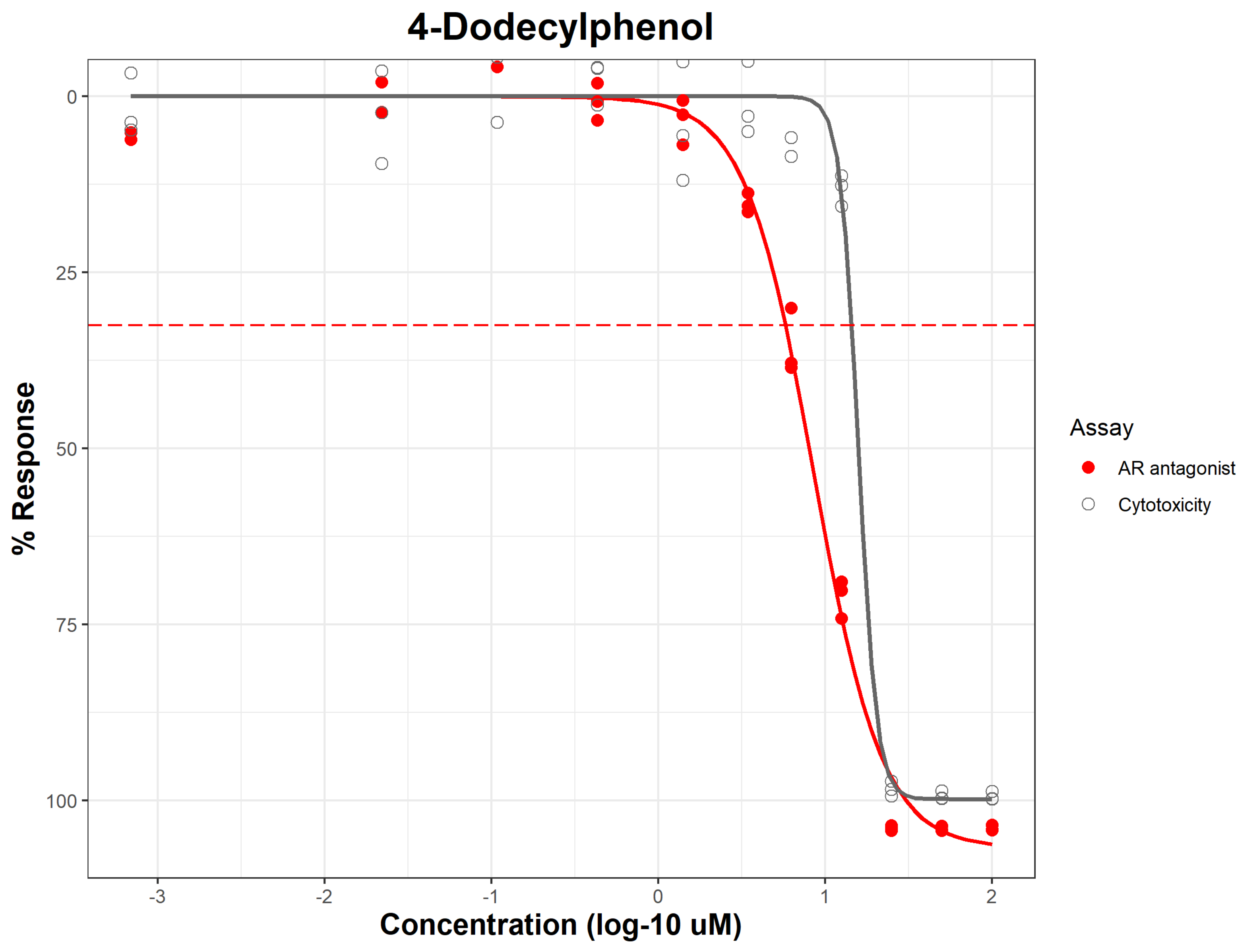

4-Dodecylphenol
% Response
0
25
50
75
100
-3
-2
-1
0
1
2
Concentration (log-10 uM)
Assay
AR antagonist
Cytotoxicity

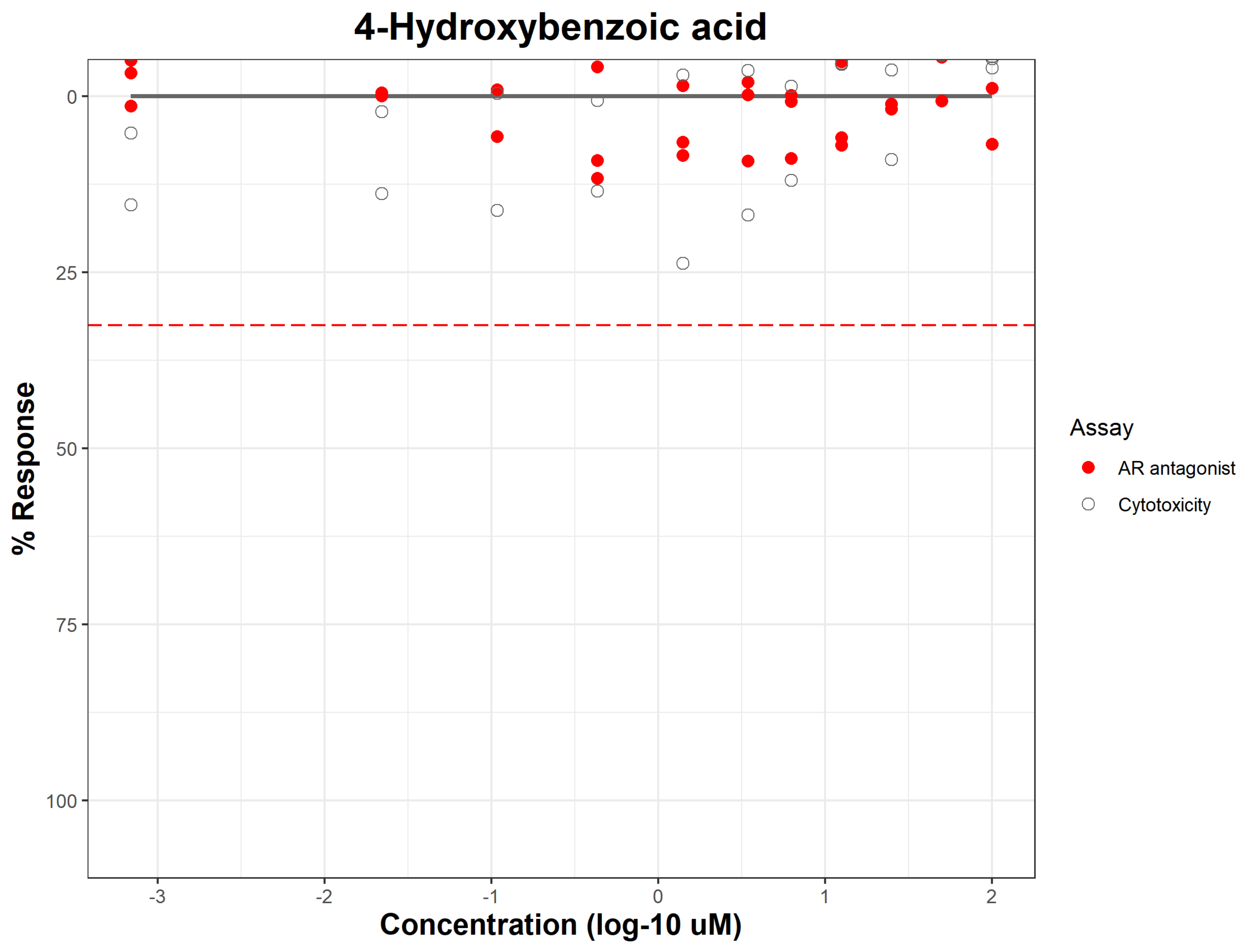
4-Hydroxybenzoic acid
% Response
0
25
50
75
100
-3
-2
-1
0
1
2
Concentration (log-10 uM)
Assay
AR antagonist
Cytotoxicity

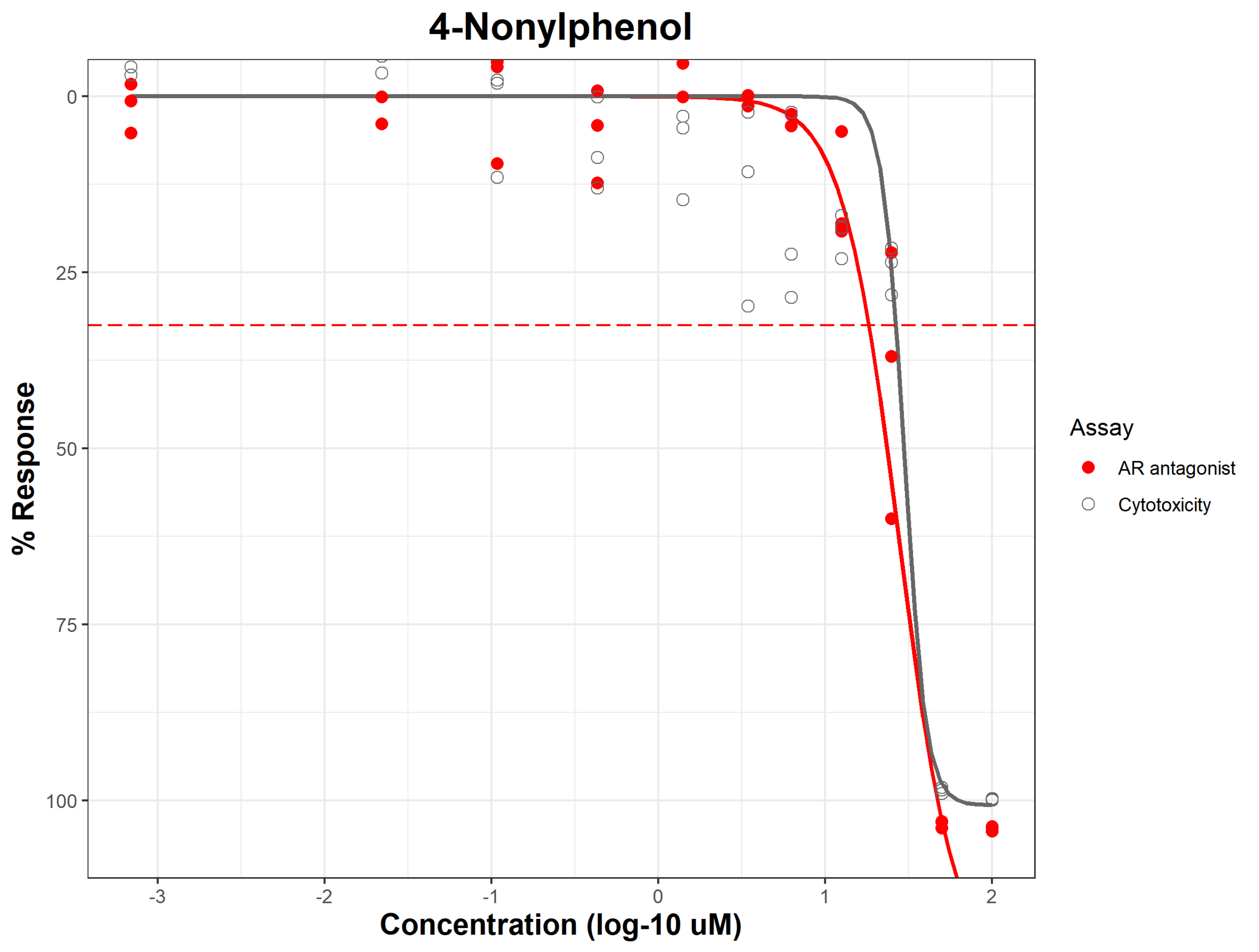

4-Nonylphenol
% Response
0
25
50
75
100
-3
-2
-1
0
1
2
Concentration (log-10 uM)
Assay
AR antagonist
Cytotoxicity

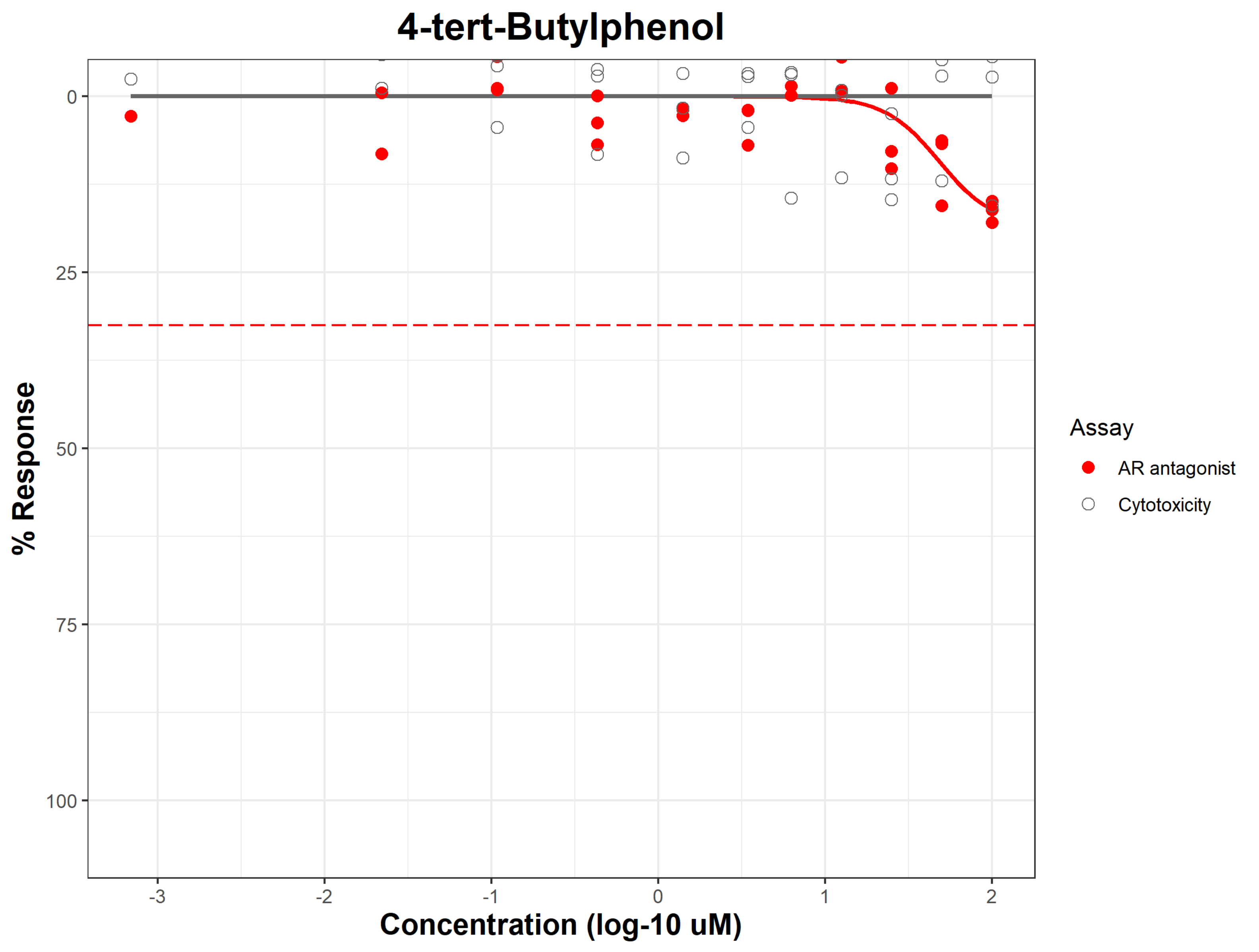

4-tert-Butylphenol
% Response
0
25
50
75
100
-3
-2
-1
0
1
2
Concentration (log-10 uM)
Assay
AR antagonist
Cytotoxicity

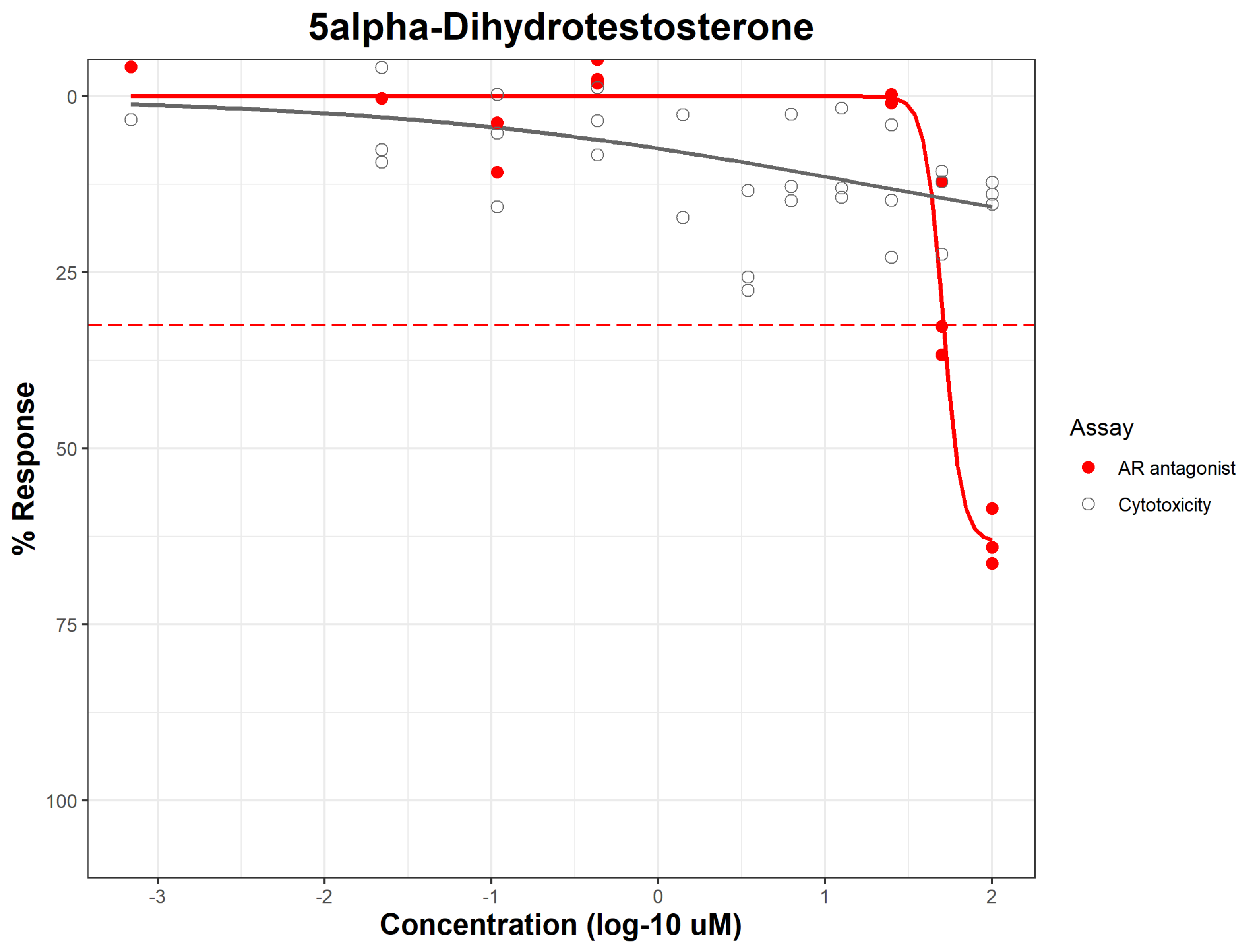

5alpha-Dihydrotestosterone
% Response
0
25
50
75
100
-3
-2
-1
0
1
2
Concentration (log-10 uM)
Assay
AR antagonist
Cytotoxicity

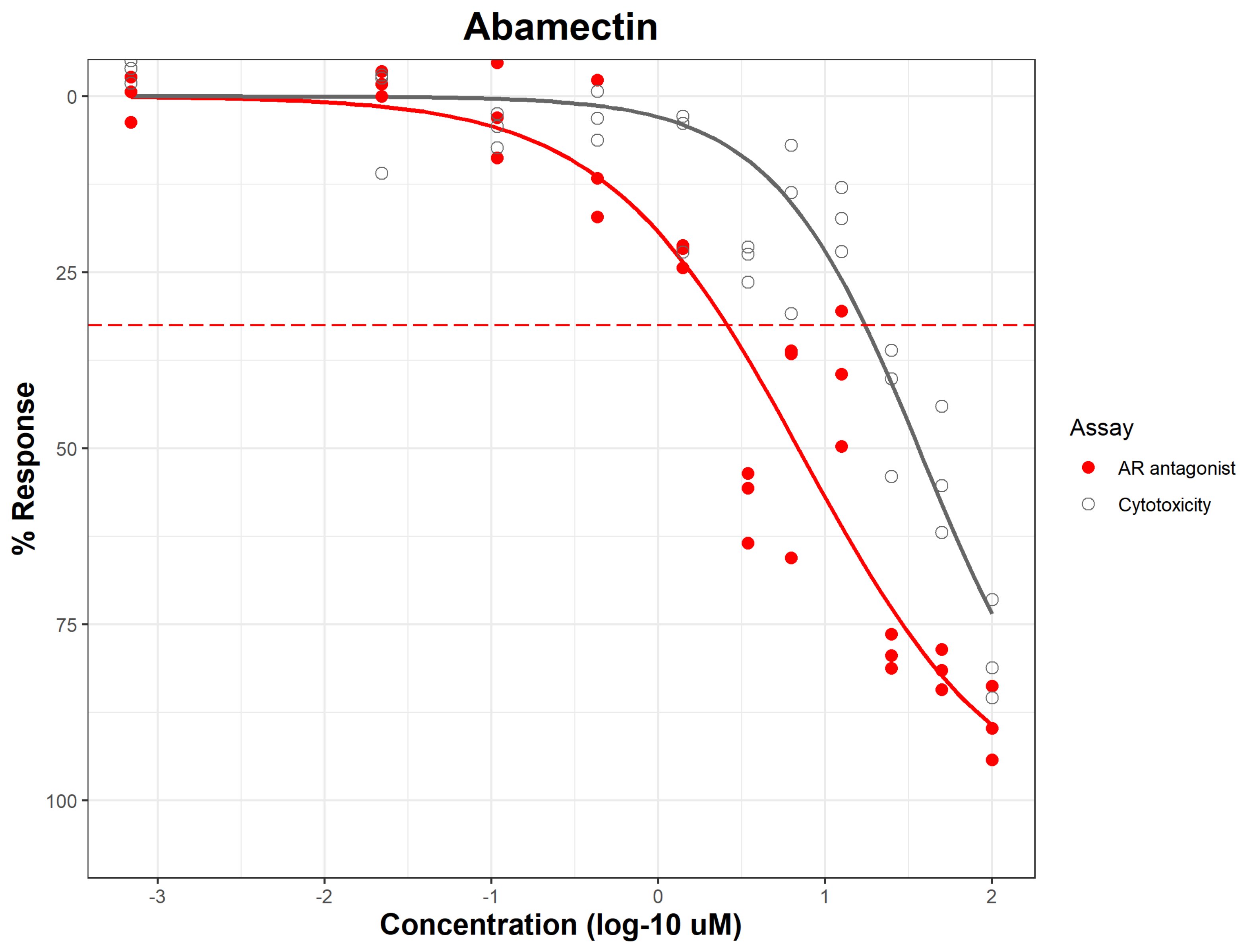

Abamectin
% Response
0
25
50
75
100
-3
-2
-1
0
1
2
Concentration (log-10 uM)
Assay
AR antagonist
Cytotoxicity

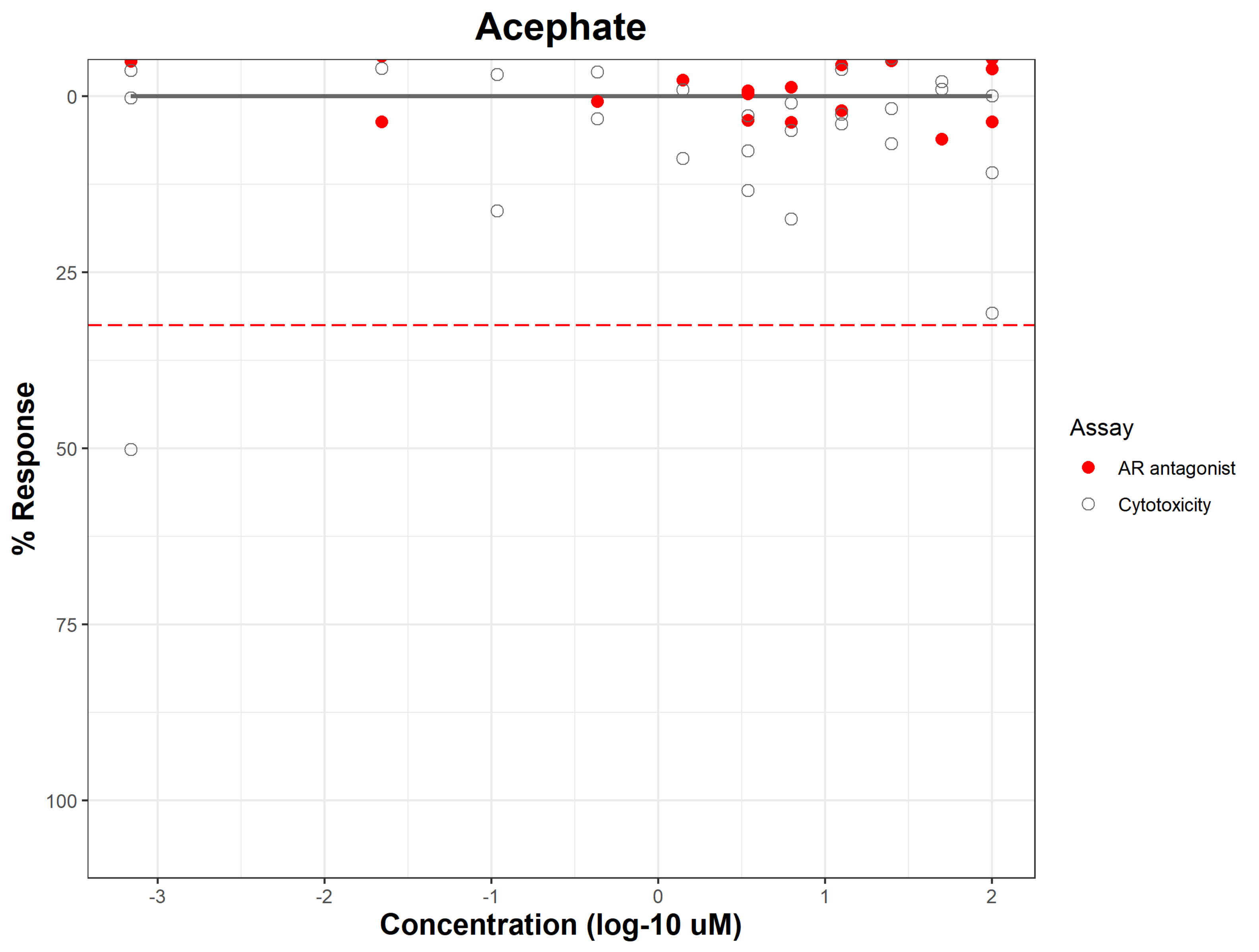
Acephate
% Response
0
25
50
75
100
-3
-2
-1
0
1
2
Concentration (log-10 uM)
Assay
AR antagonist
Cytotoxicity

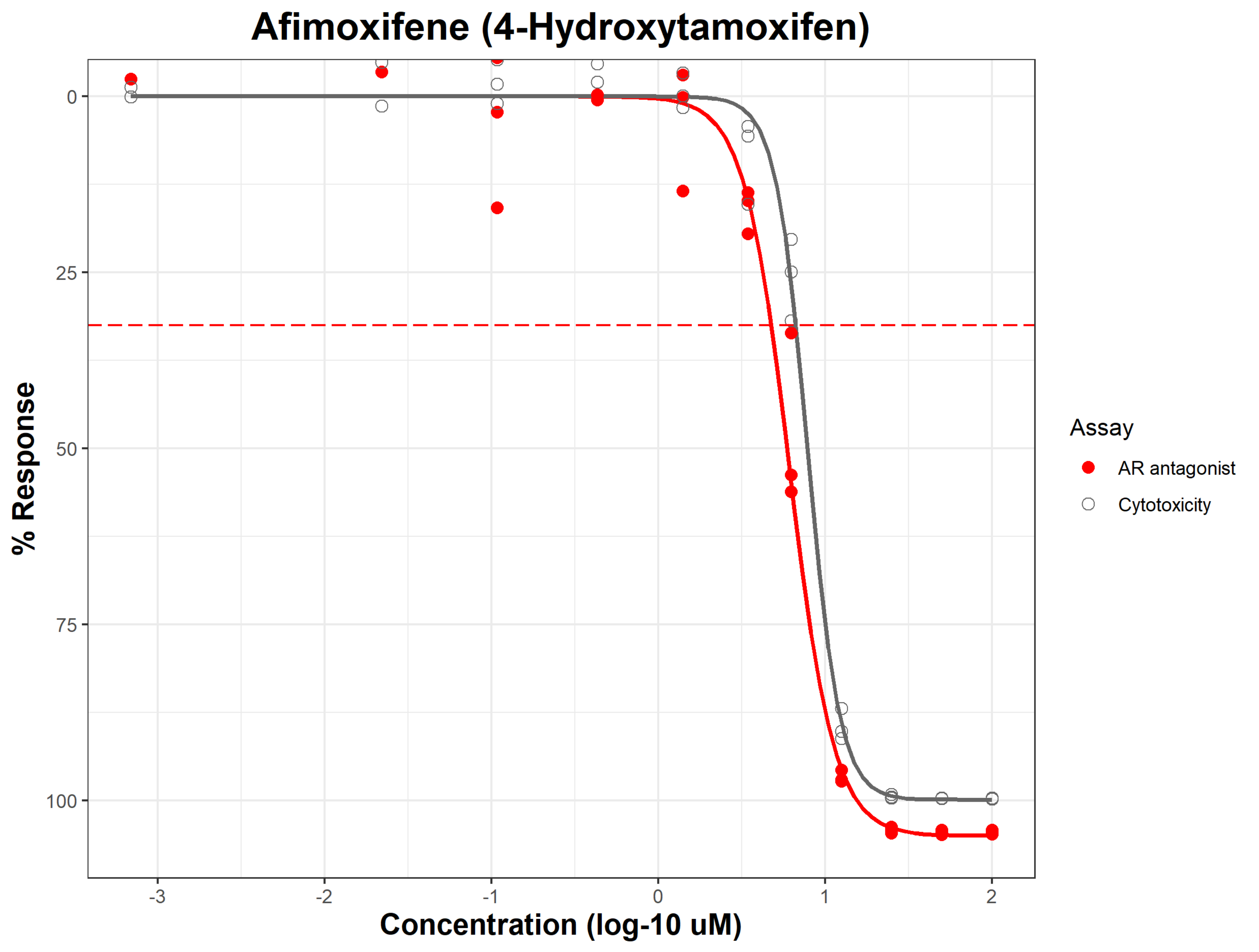

Afimoxifene (4-Hydroxytamoxifen)
% Response
0
25
50
75
100
-3
-2
-1
0
1
2
Concentration (log-10 uM)
Assay
AR antagonist
Cytotoxicity

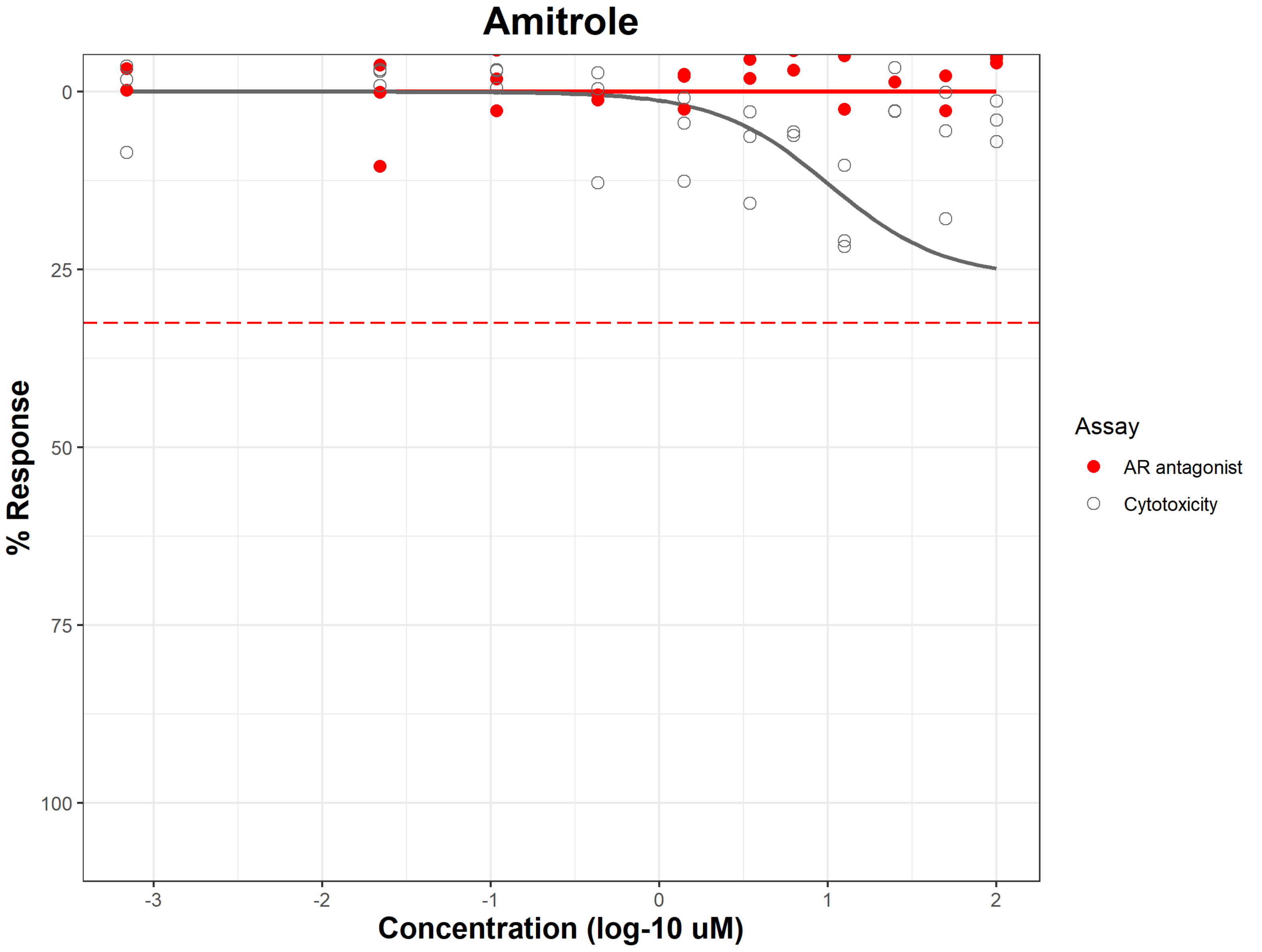

Amitrole
% Response
0
25
50
75
100
-3
-2
-1
0
1
2
Concentration (log-10 uM)
Assay
AR antagonist
Cytotoxicity

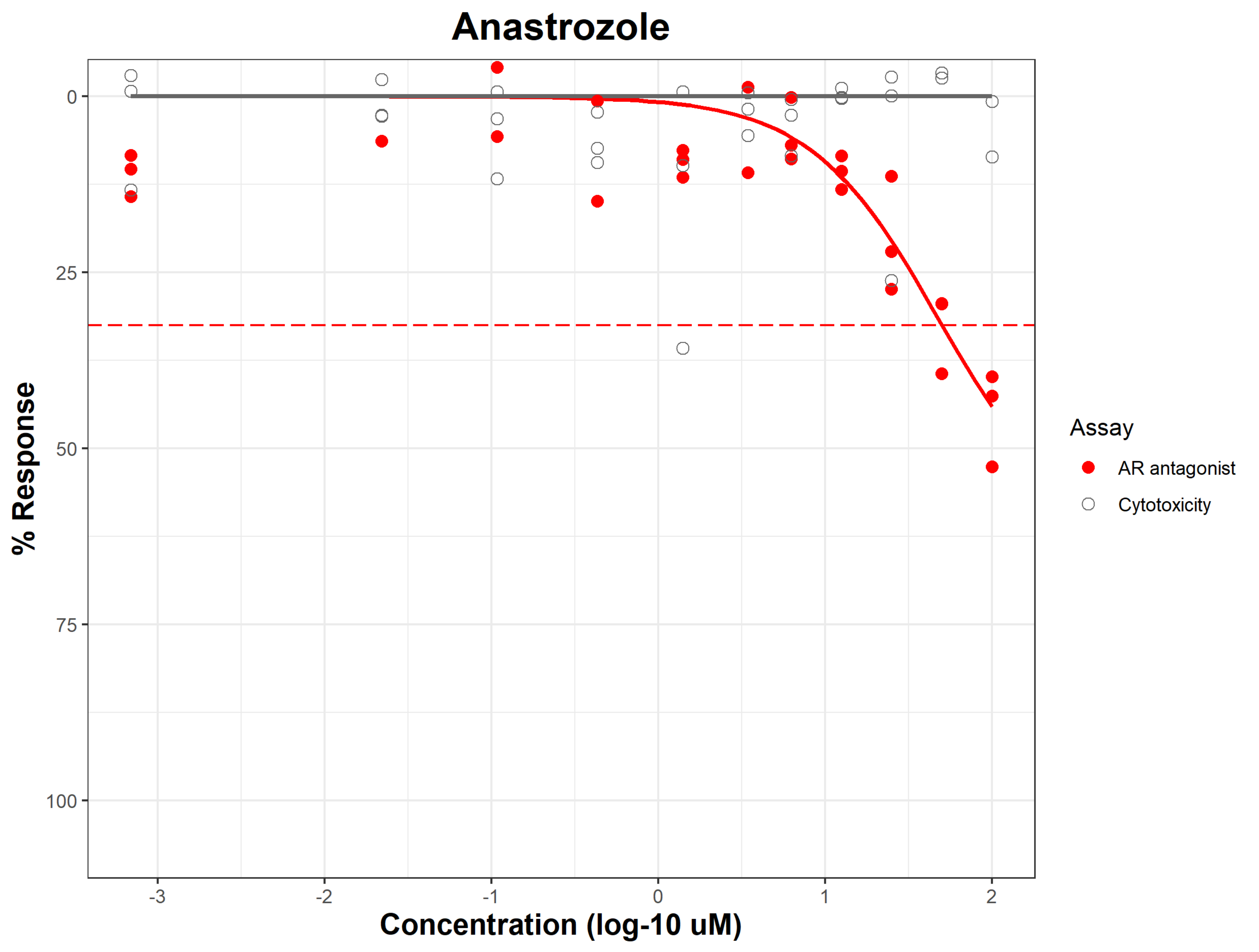

Anastrozole
% Response
0
25
50
75
100
-3
-2
-1
0
1
2
Concentration (log-10 uM)
Assay
AR antagonist
Cytotoxicity

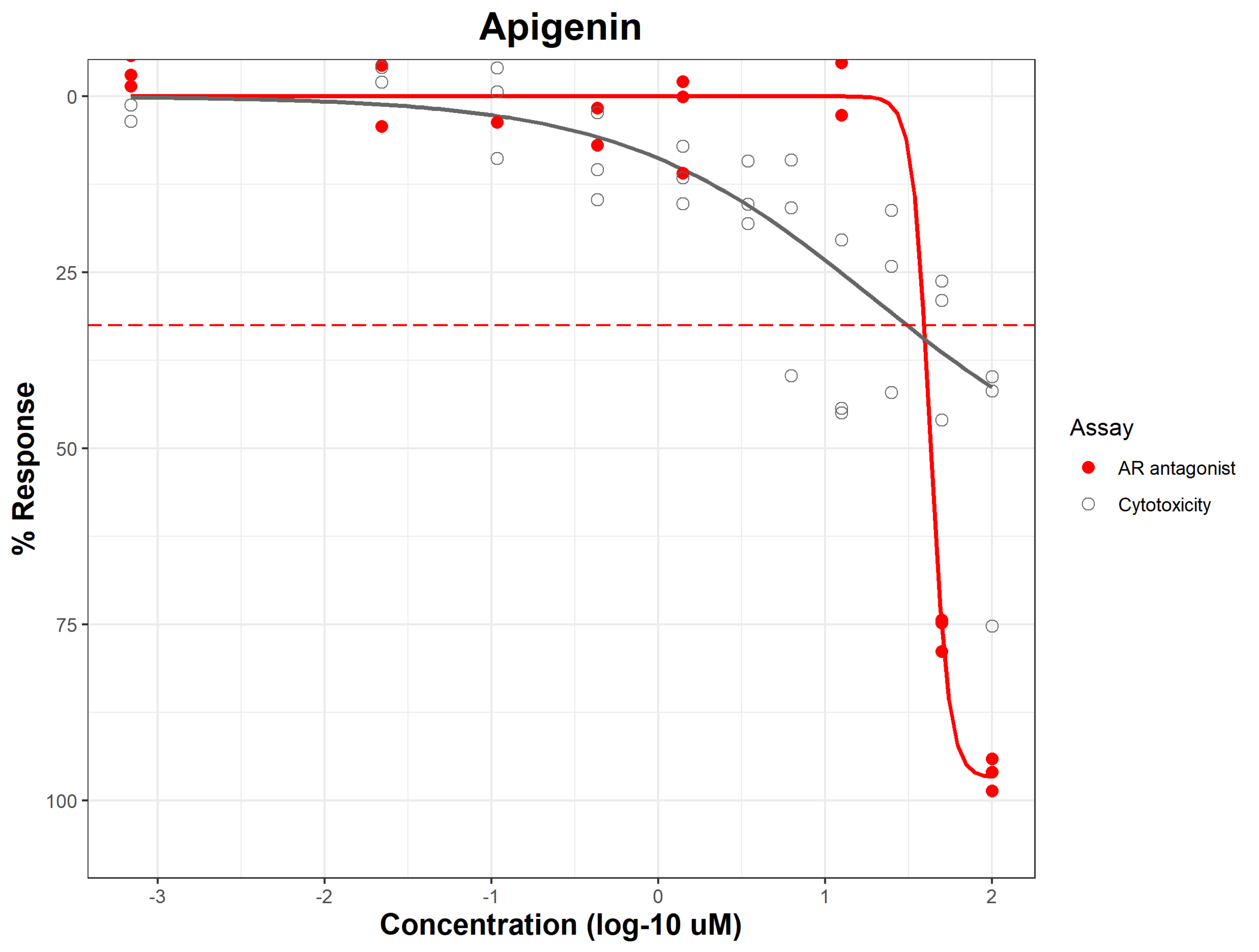

Apigenin
% Response
0
25
50
75
100
-3
-2
-1
0
1
2
Concentration (log-10 uM)
Assay
AR antagonist
Cytotoxicity

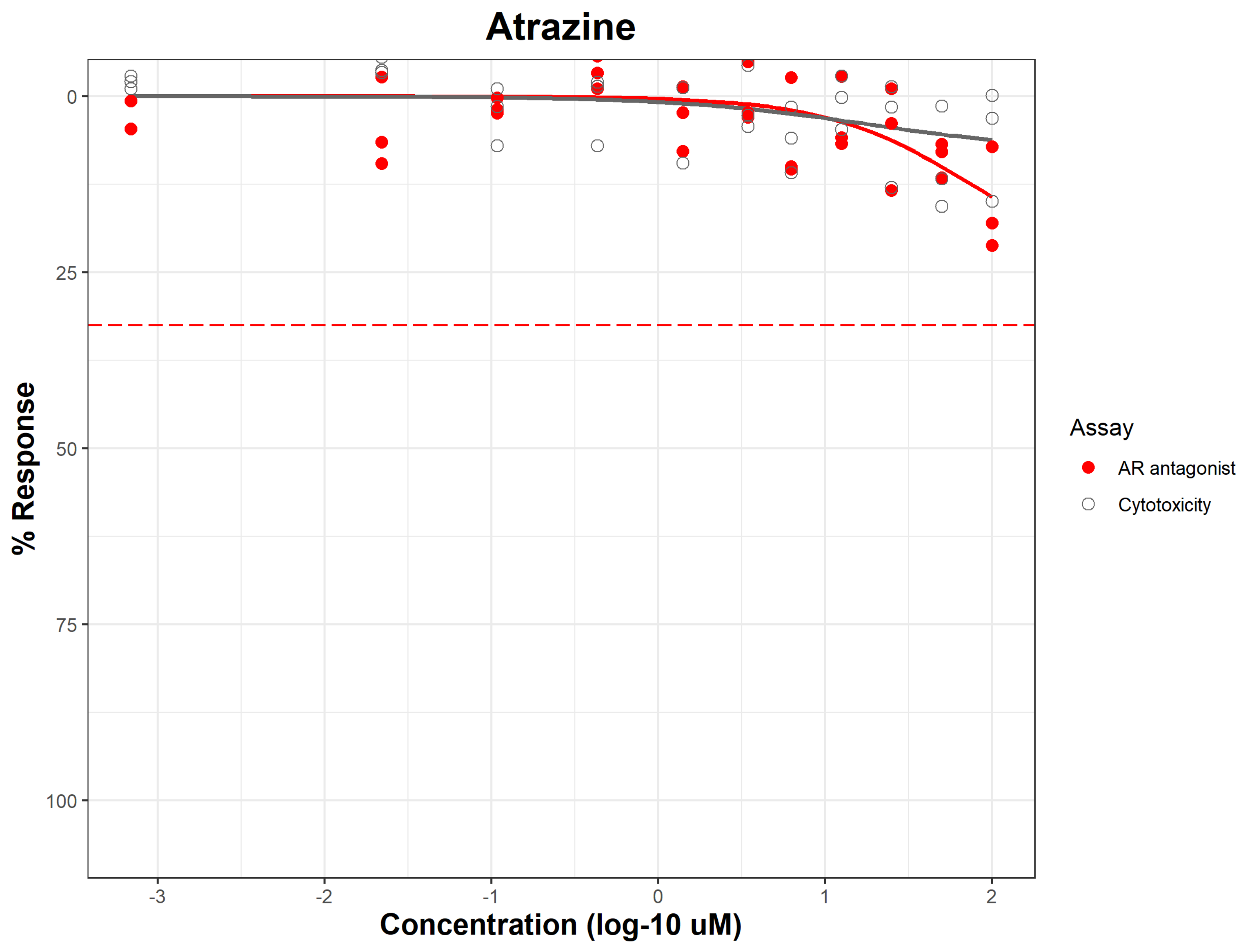

Atrazine
% Response
0
25
50
75
100
-3
-2
-1
0
1
2
Concentration (log-10 uM)
Assay
AR antagonist
Cytotoxicity

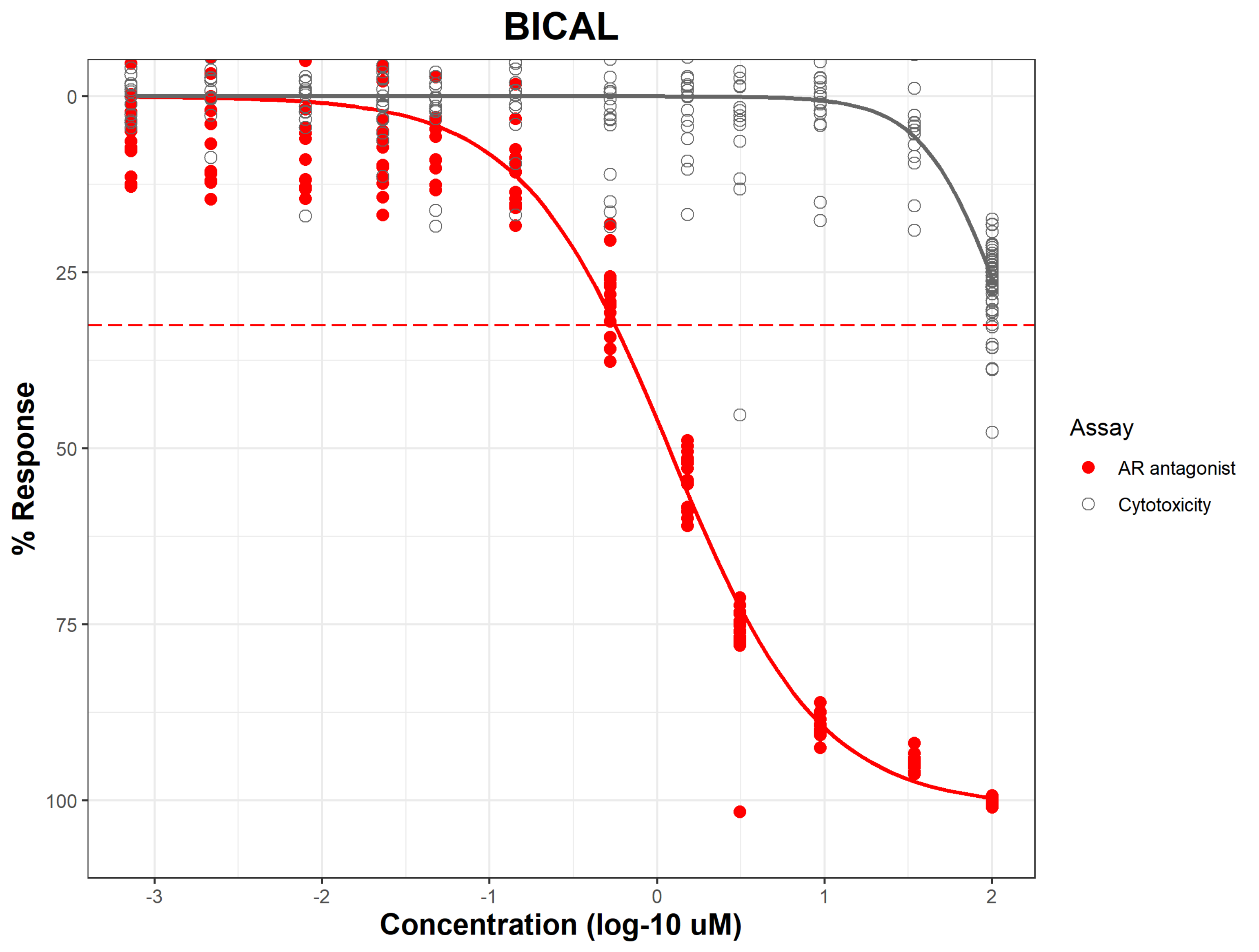
BICAL
% Response
0
25
50
75
100
Concentration (log-10 uM)
-3
-2
-1
0
1
2
Assay
AR antagonist
Cytotoxicity

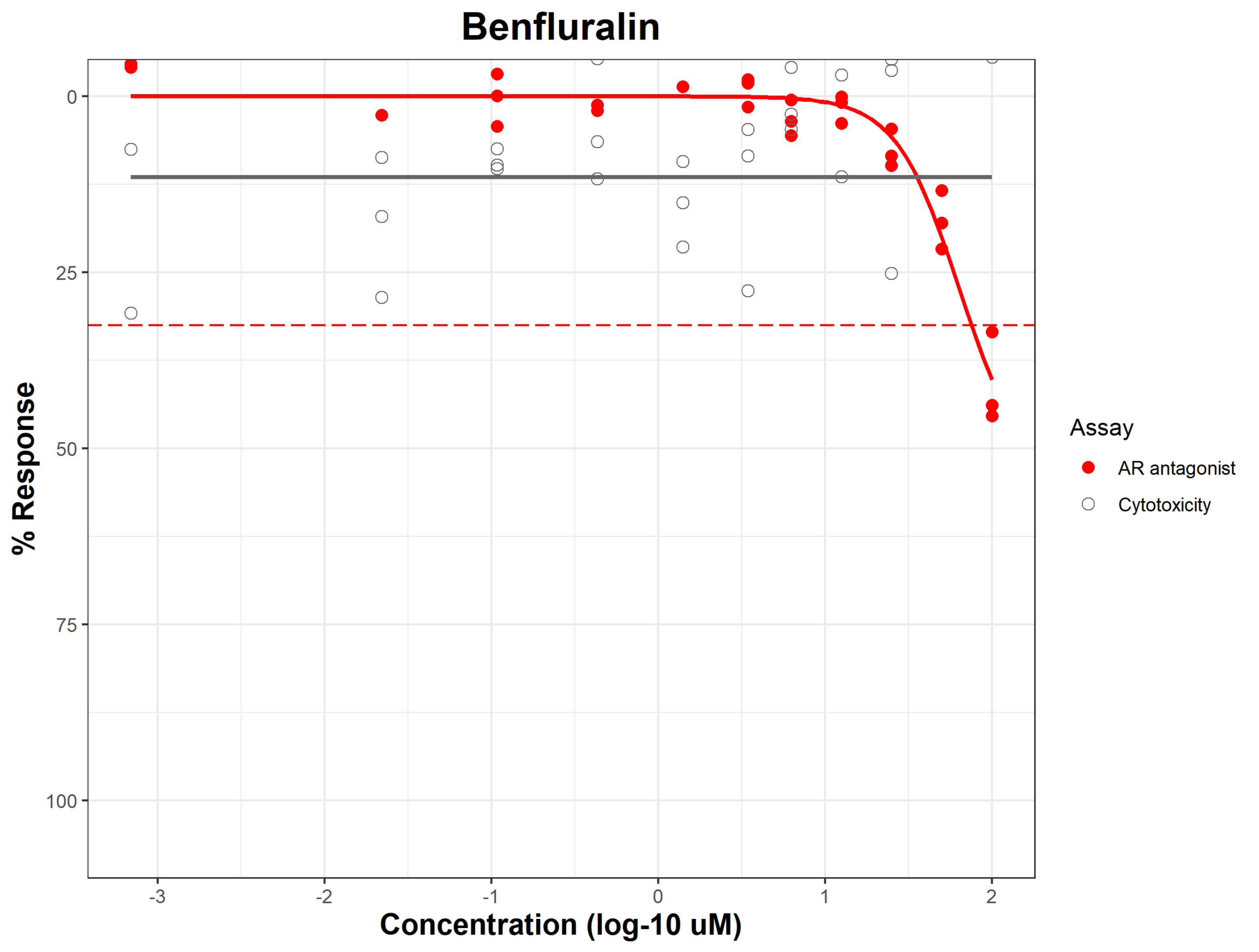

Benfluralin
% Response
0
25
50
75
100
-3
-2
-1
0
1
2
Concentration (log-10 uM)
Assay
AR antagonist
Cytotoxicity

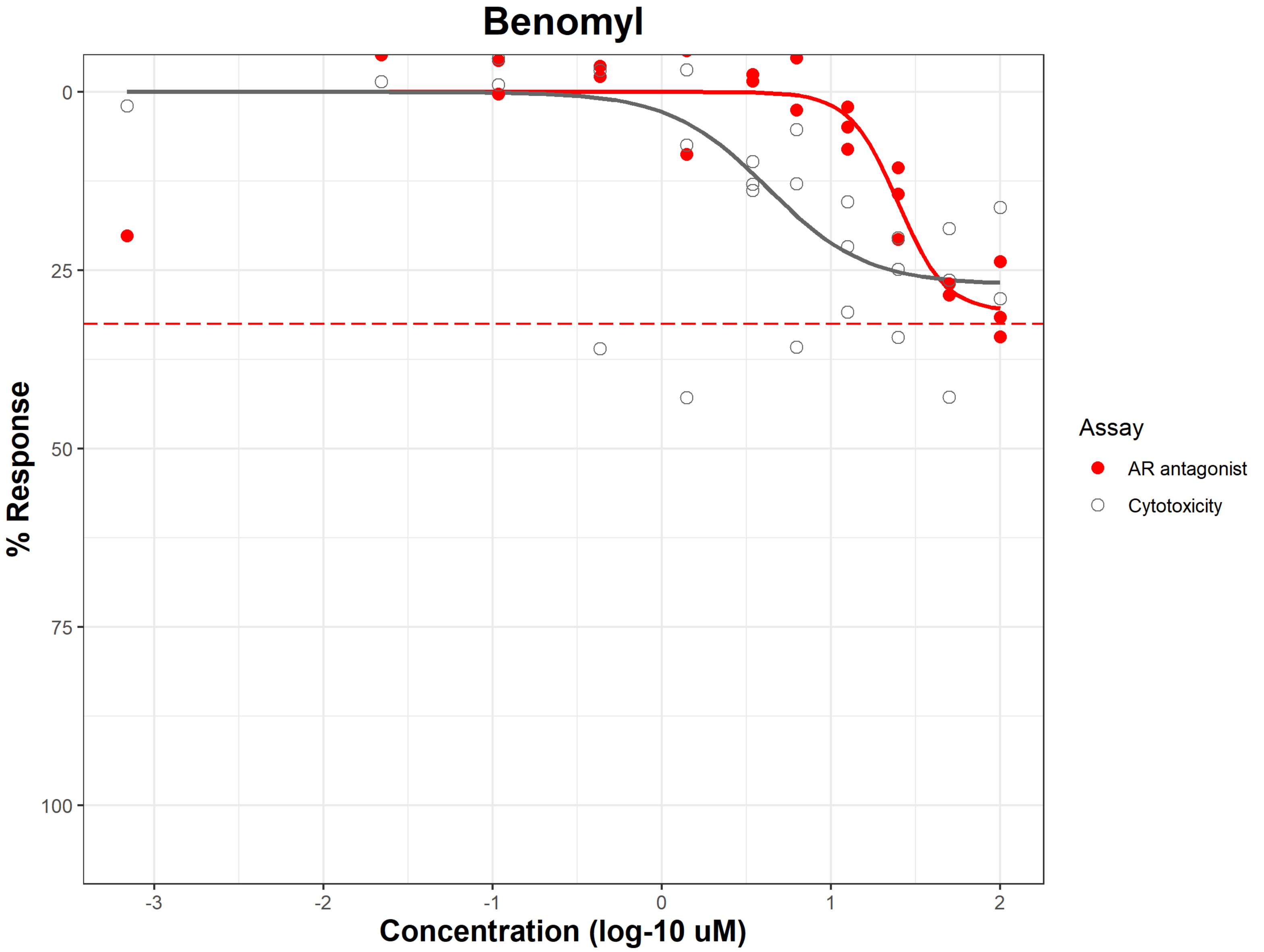

Benomyl
% Response
0
25
50
75
100
-3
-2
-1
0
1
2
Concentration (log-10 uM)
Assay
AR antagonist
Cytotoxicity

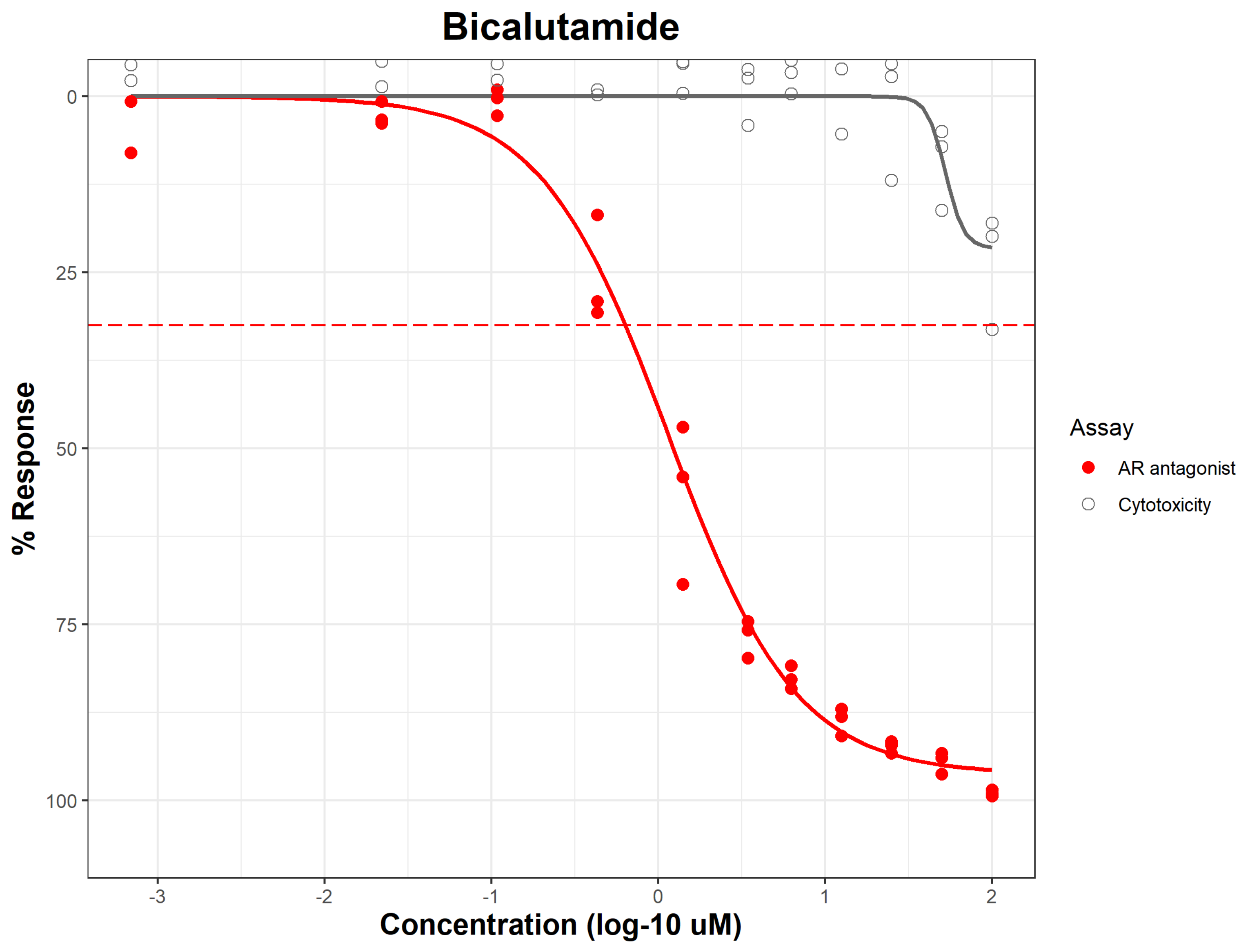
Bicalutamide
0
25
50
75
100
% Response
-3
-2
-1
0
1
2
Concentration (log-10 uM)
Assay
AR antagonist
Cytotoxicity

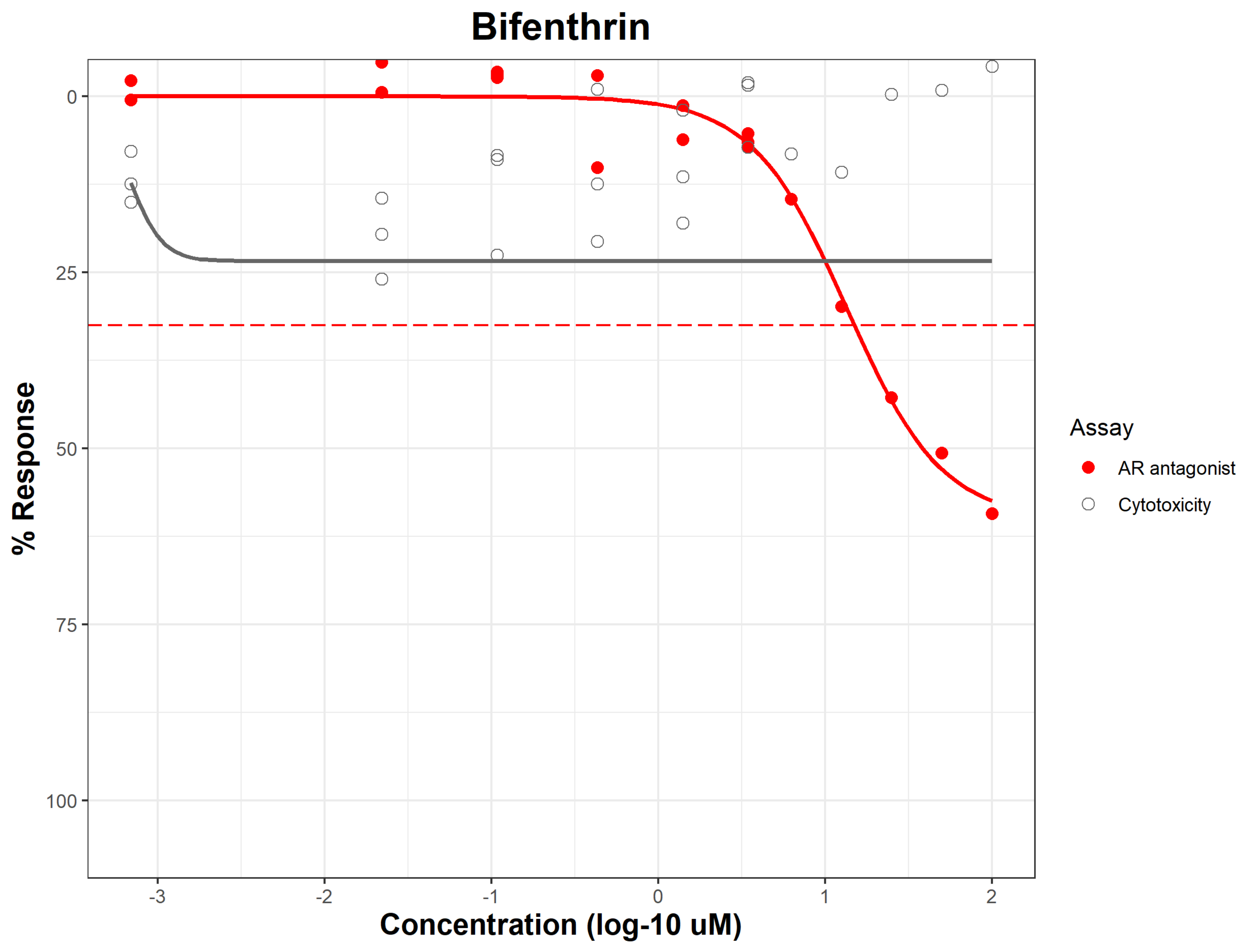
Bifenthrin
% Response
0
25
50
75
100
Concentration (log-10 uM)
-3
-2
-1
0
1
2
Assay
AR antagonist
Cytotoxicity

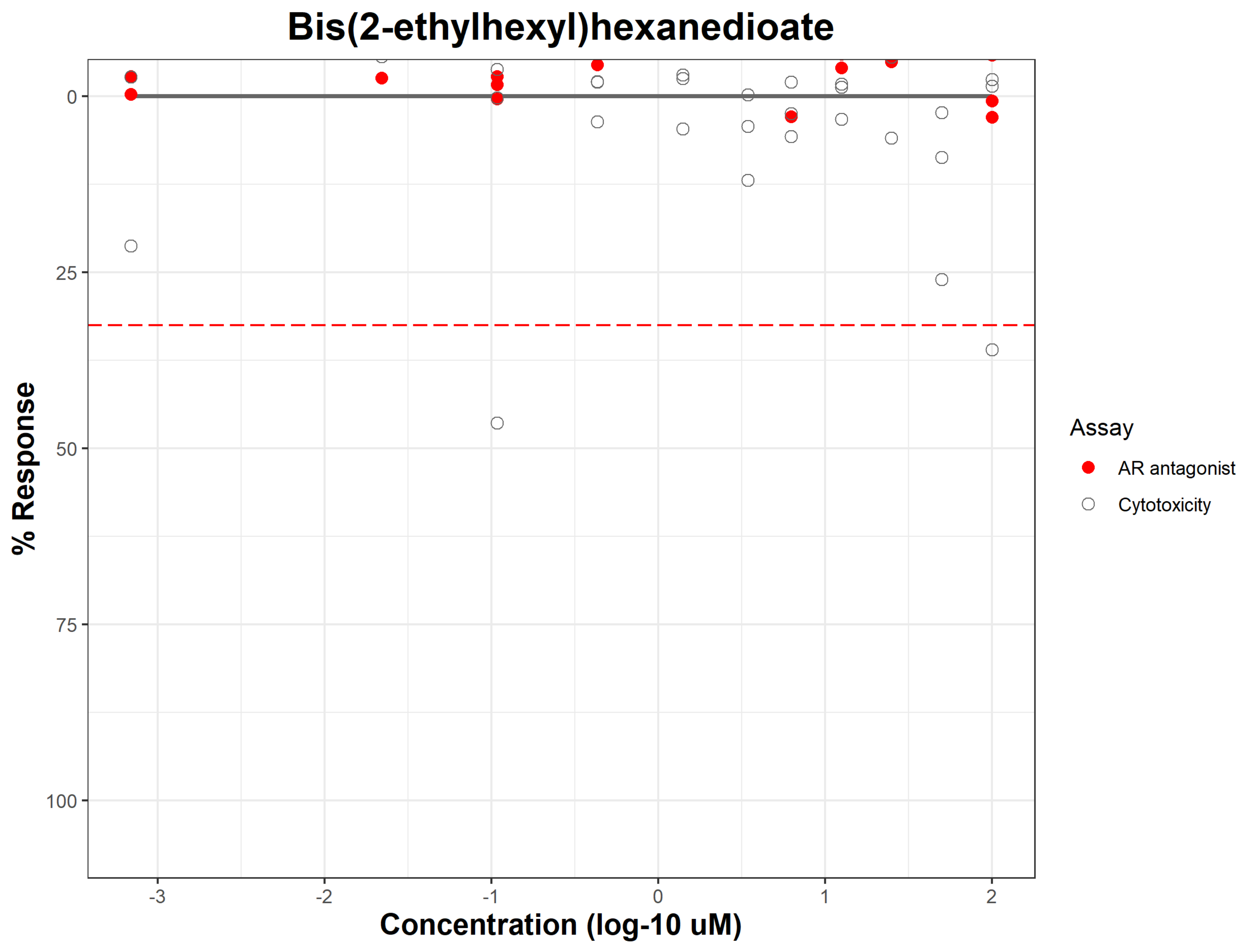
Bis(2-ethylhexyl)hexanedioate
% Response
Concentration (log-10 uM)
0
25
50
75
100
-3
-2
-1
0
1
2
Assay
AR antagonist
Cytotoxicity

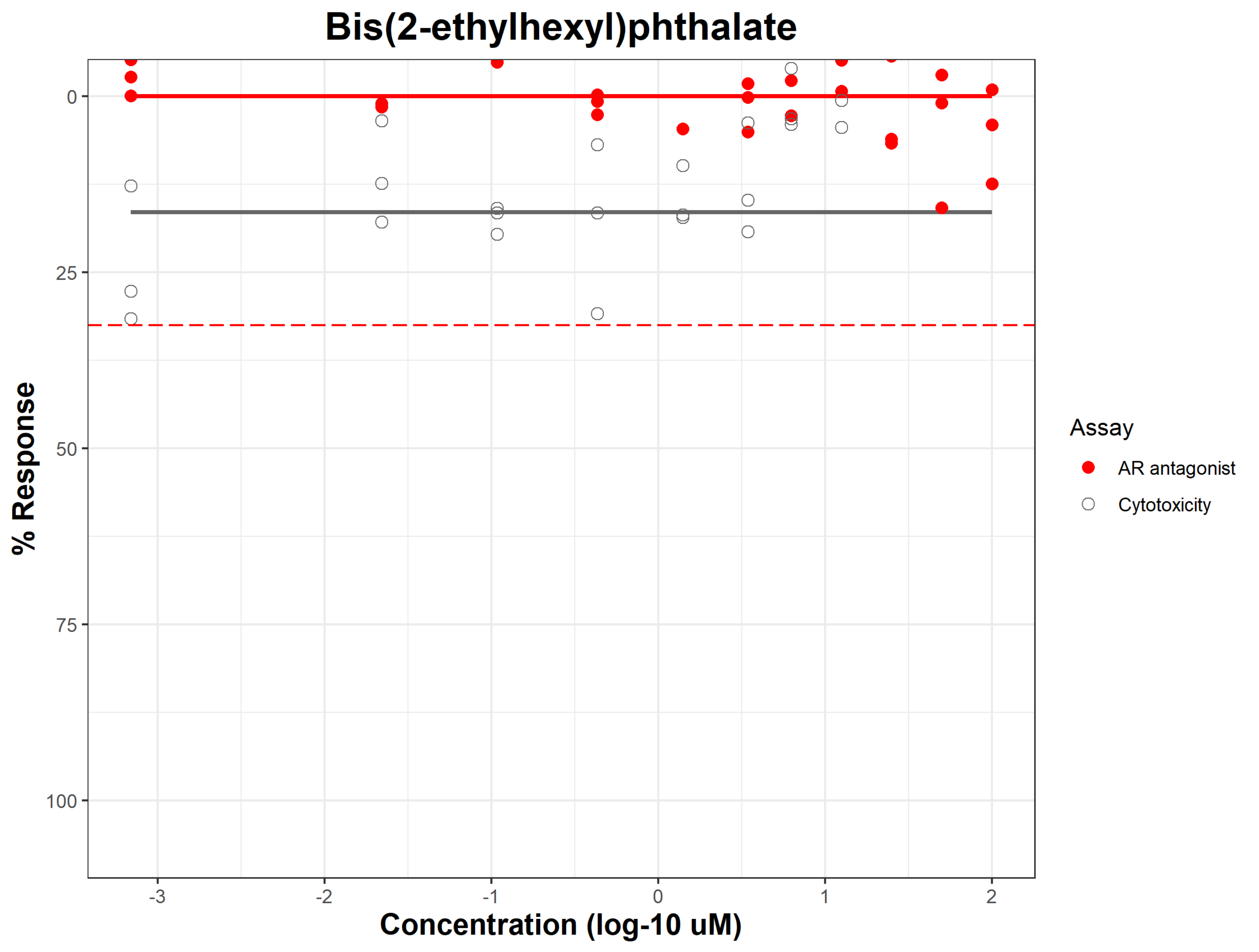

Bis(2-ethylhexyl)phthalate
% Response
0
25
50
75
100
-3
-2
-1
0
1
2
Concentration (log-10 uM)
Assay
AR antagonist
Cytotoxicity

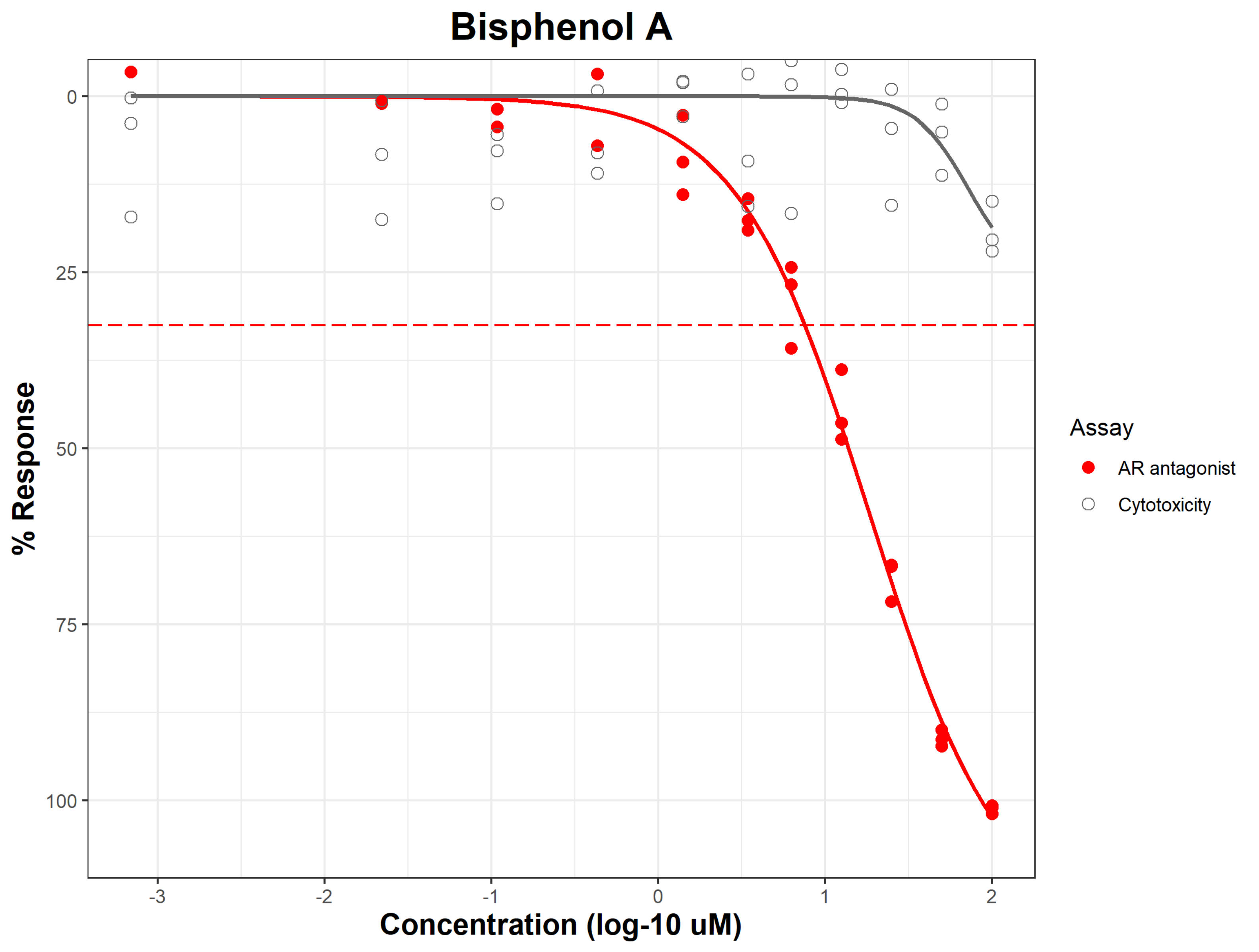

Bisphenol A
% Response
0
25
50
75
100
-3
-2
-1
0
1
2
Concentration (log-10 uM)
Assay
AR antagonist
Cytotoxicity

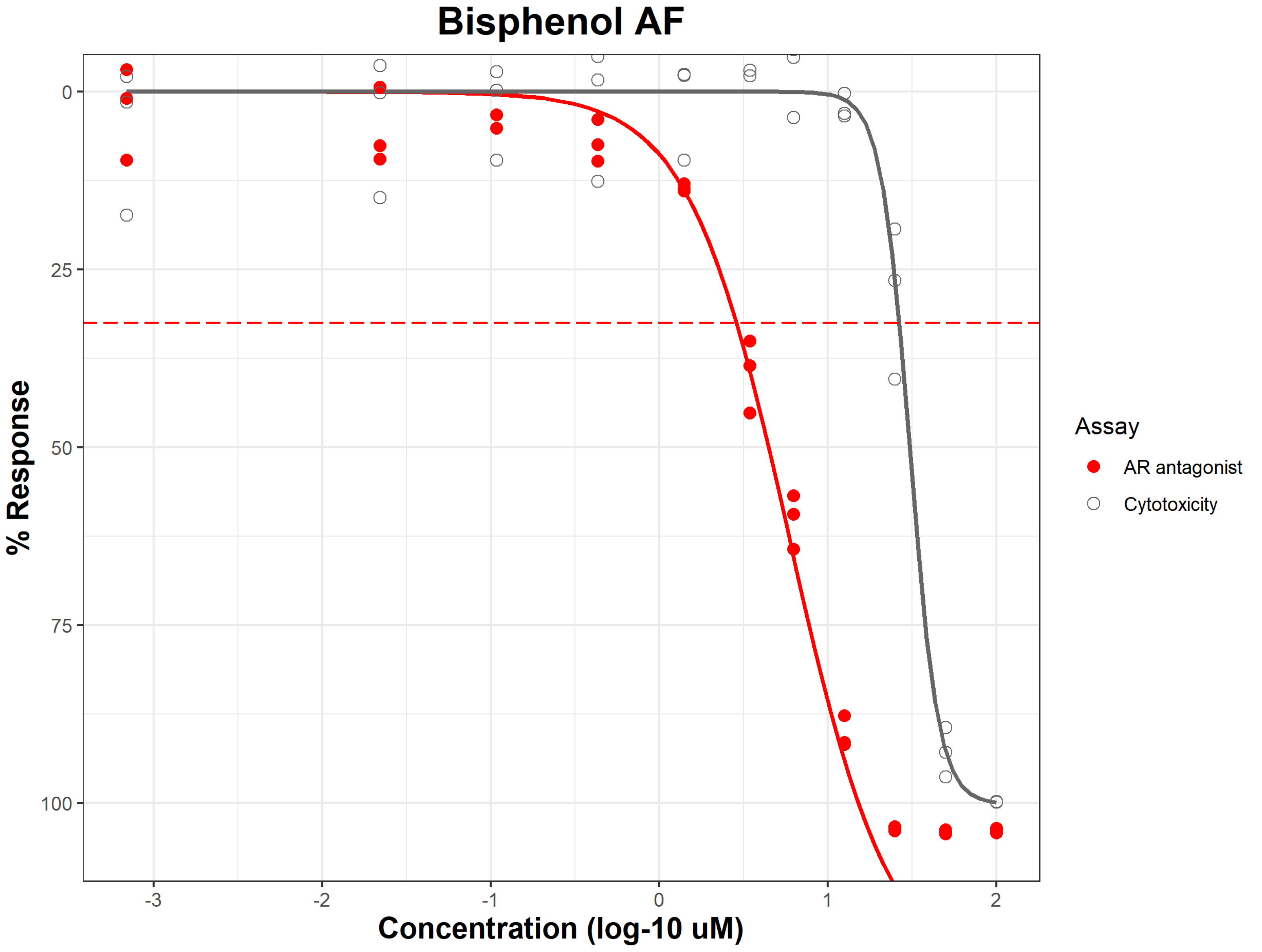

Bisphenol AF
% Response
0
25
50
75
100
-3
-2
-1
0
1
2
Concentration (log-10 uM)
Assay
AR antagonist
Cytotoxicity

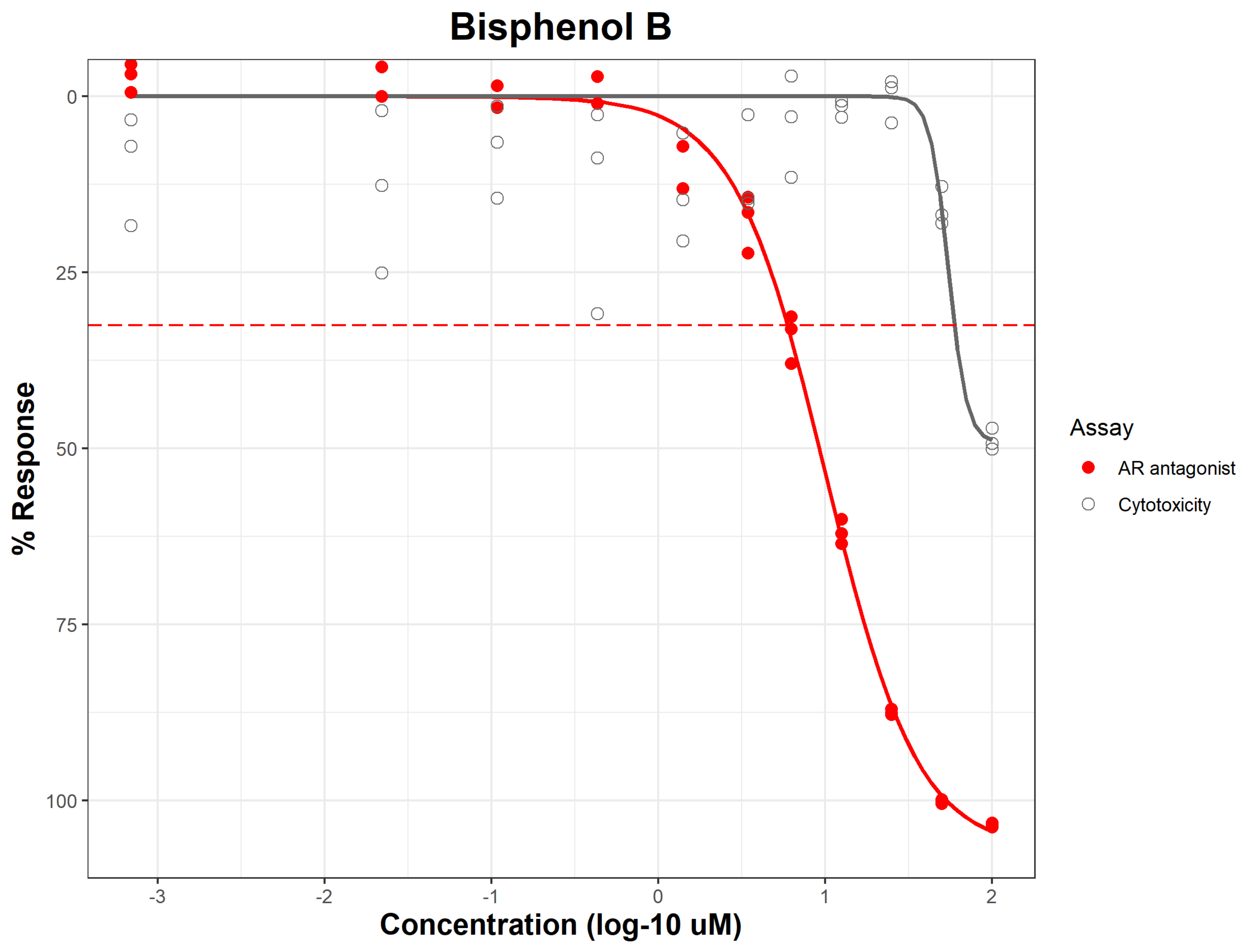

Bisphenol B
% Response
0
25
50
75
100
Concentration (log-10 uM)
-3
-2
-1
0
1
2
Assay
AR antagonist
Cytotoxicity

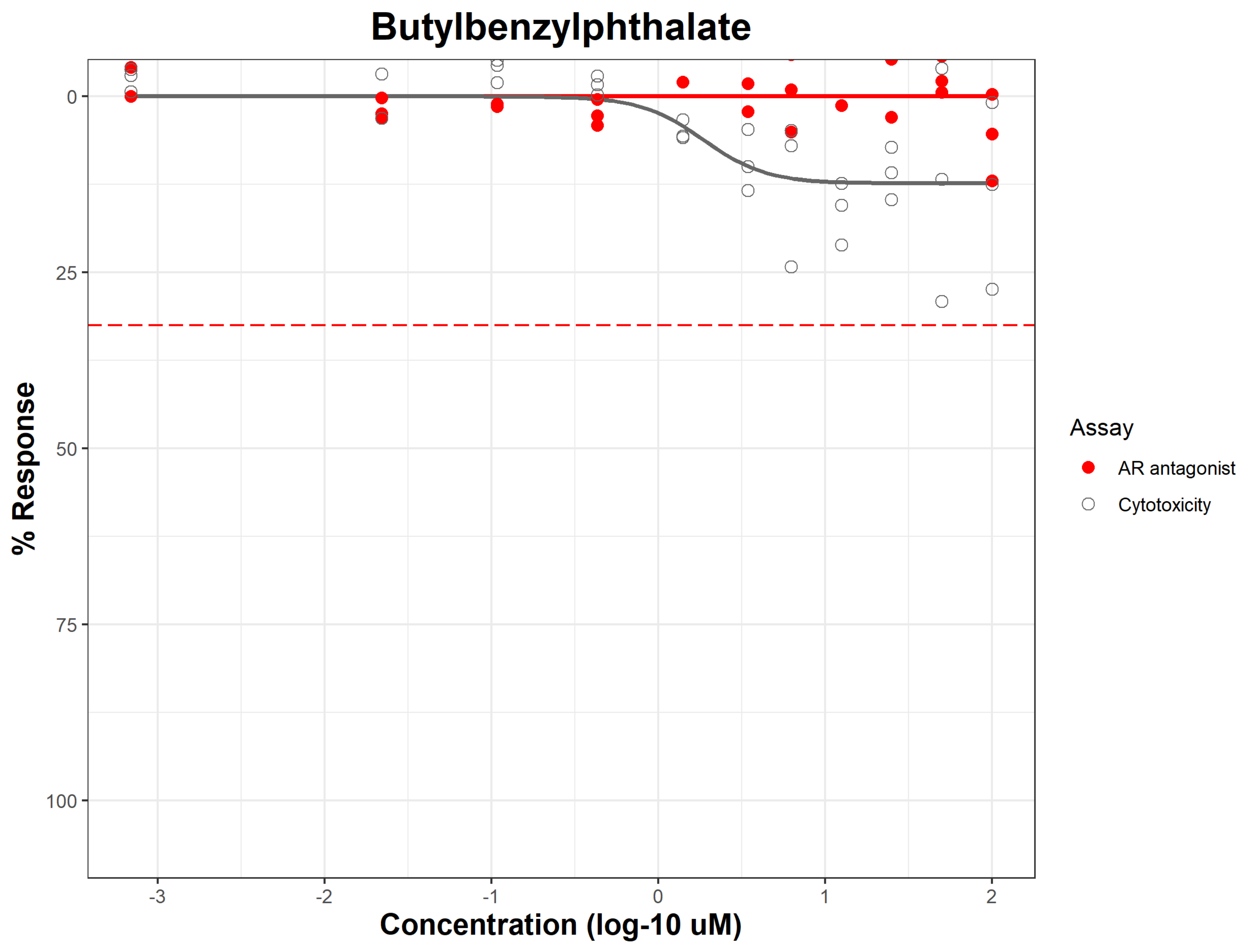

Butylbenzylphthalate
% Response
0
25
50
75
100
-3
-2
-1
0
1
2
Concentration (log-10 uM)
Assay
AR antagonist
Cytotoxicity

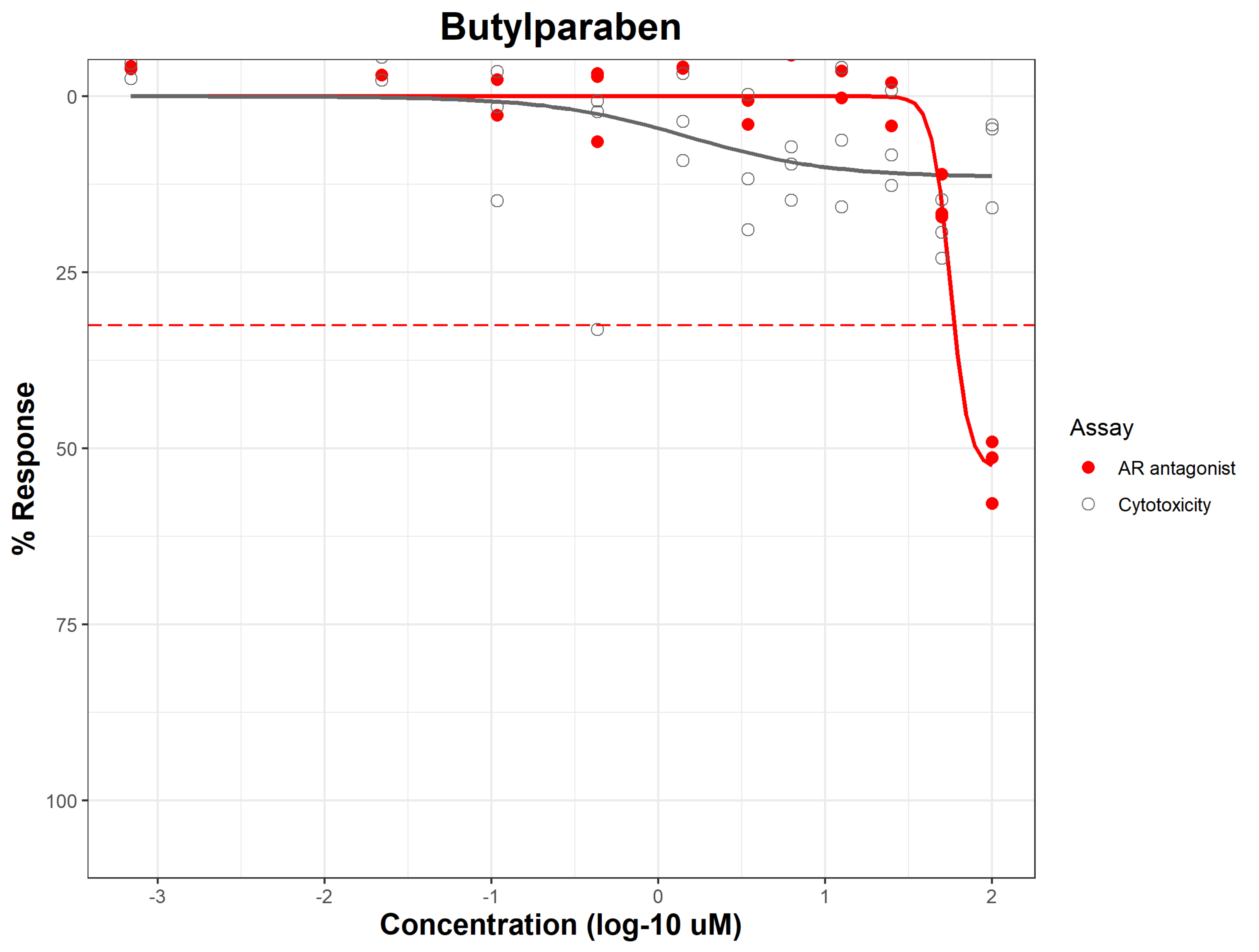

Butylparaben
% Response
0
25
50
75
100
-3
-2
-1
0
1
2
Concentration (log-10 uM)
Assay
AR antagonist
Cytotoxicity

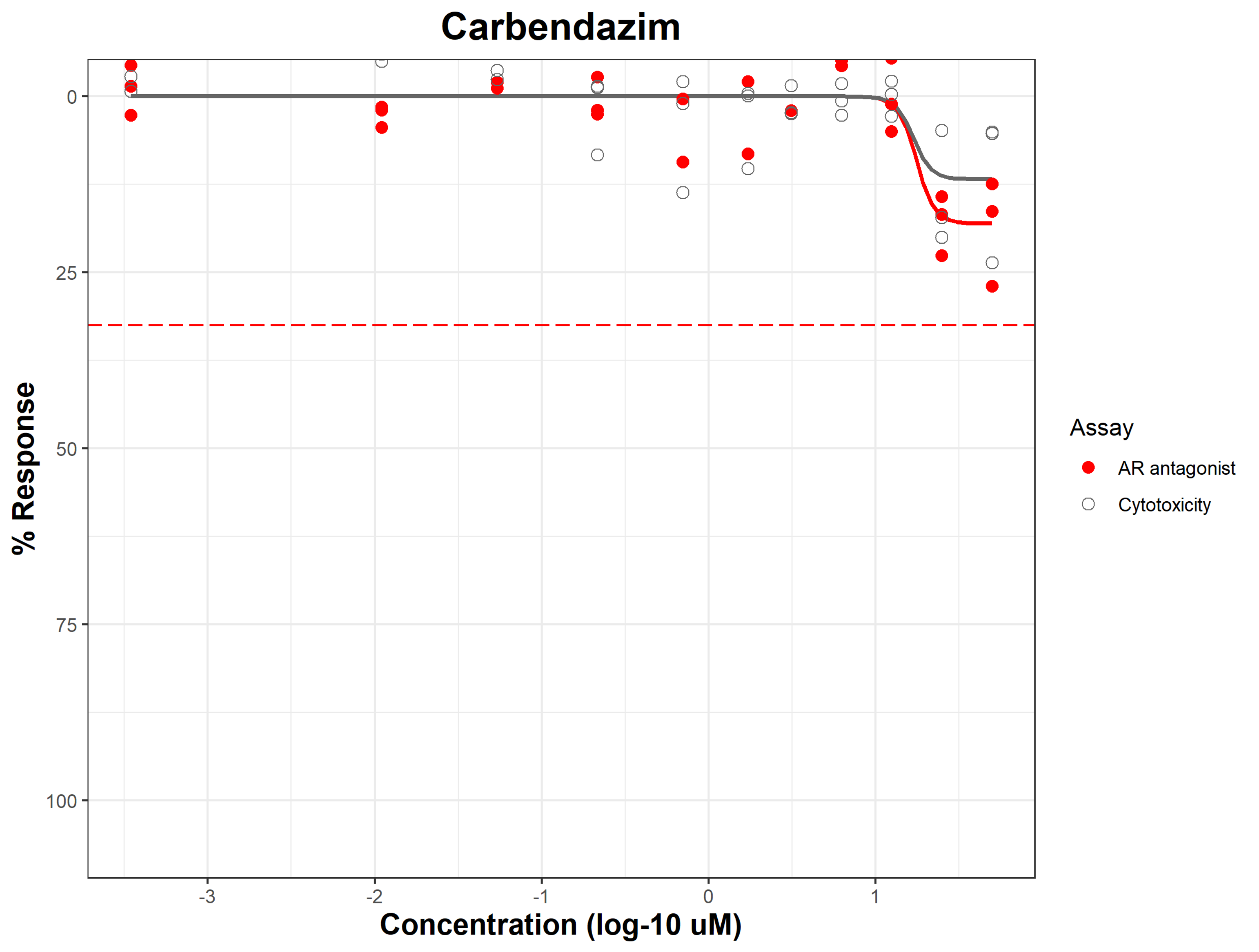

Carbendazim
% Response
0
25
50
75
100
-3
-2
-1
0
1
Concentration (log-10 uM)
Assay
AR antagonist
Cytotoxicity

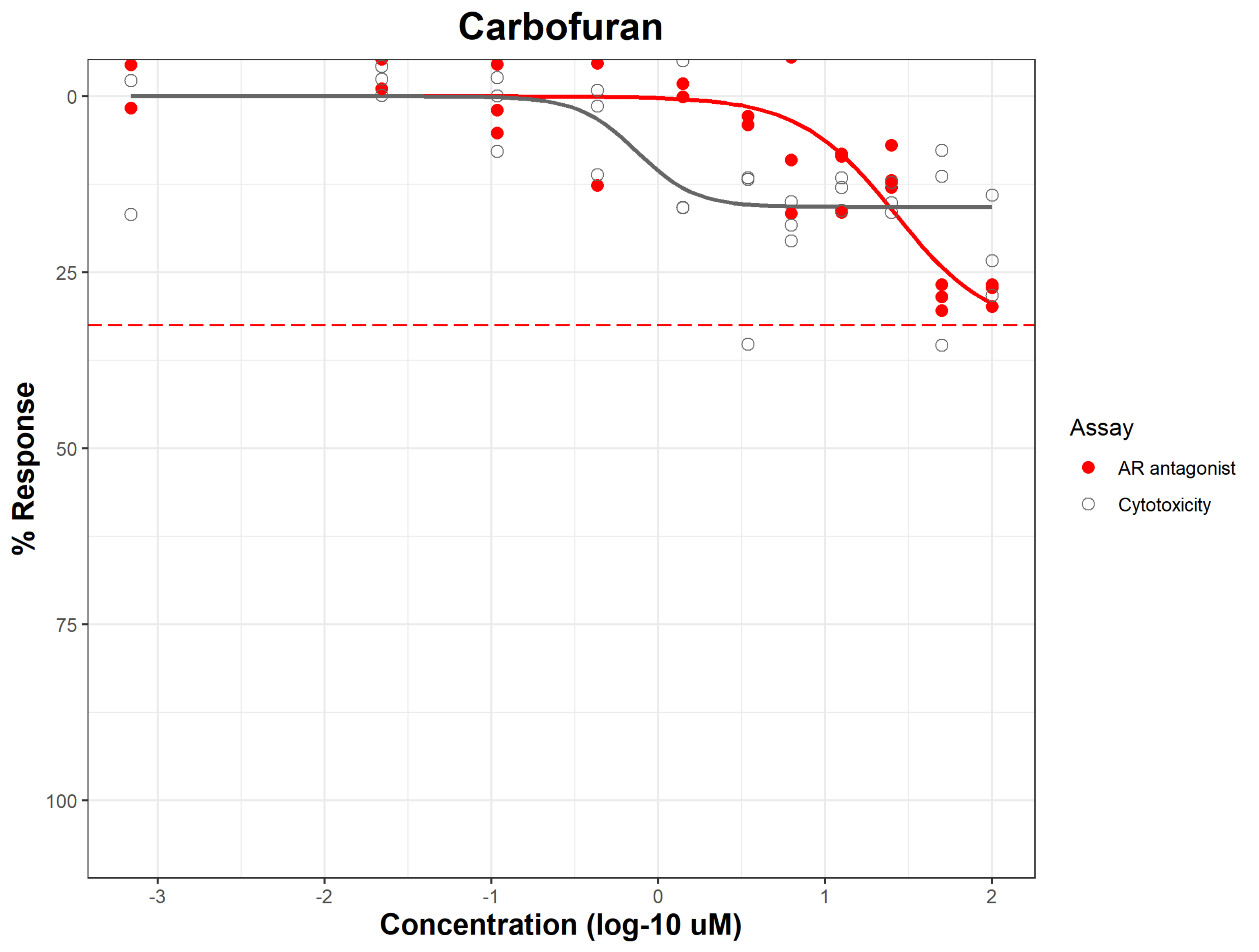

Carbofuran
0
25
50
75
100
% Response
-3
-2
-1
0
1
2
Concentration (log-10 uM)
Assay
AR antagonist
Cytotoxicity

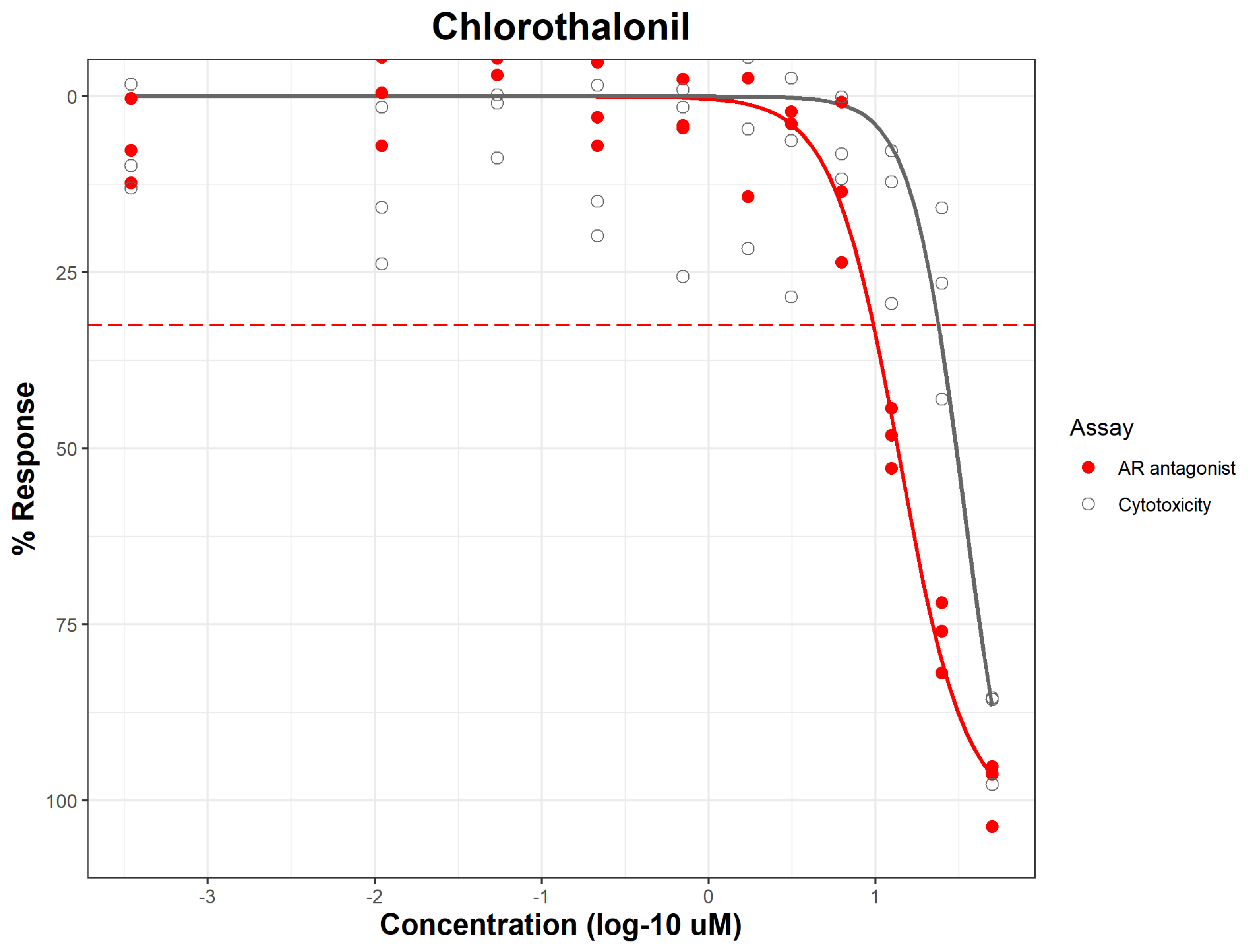

Chlorothalonil
% Response
0
25
50
75
100
-3
-2
-1
0
1
Concentration (log-10 uM)
Assay
AR antagonist
Cytotoxicity

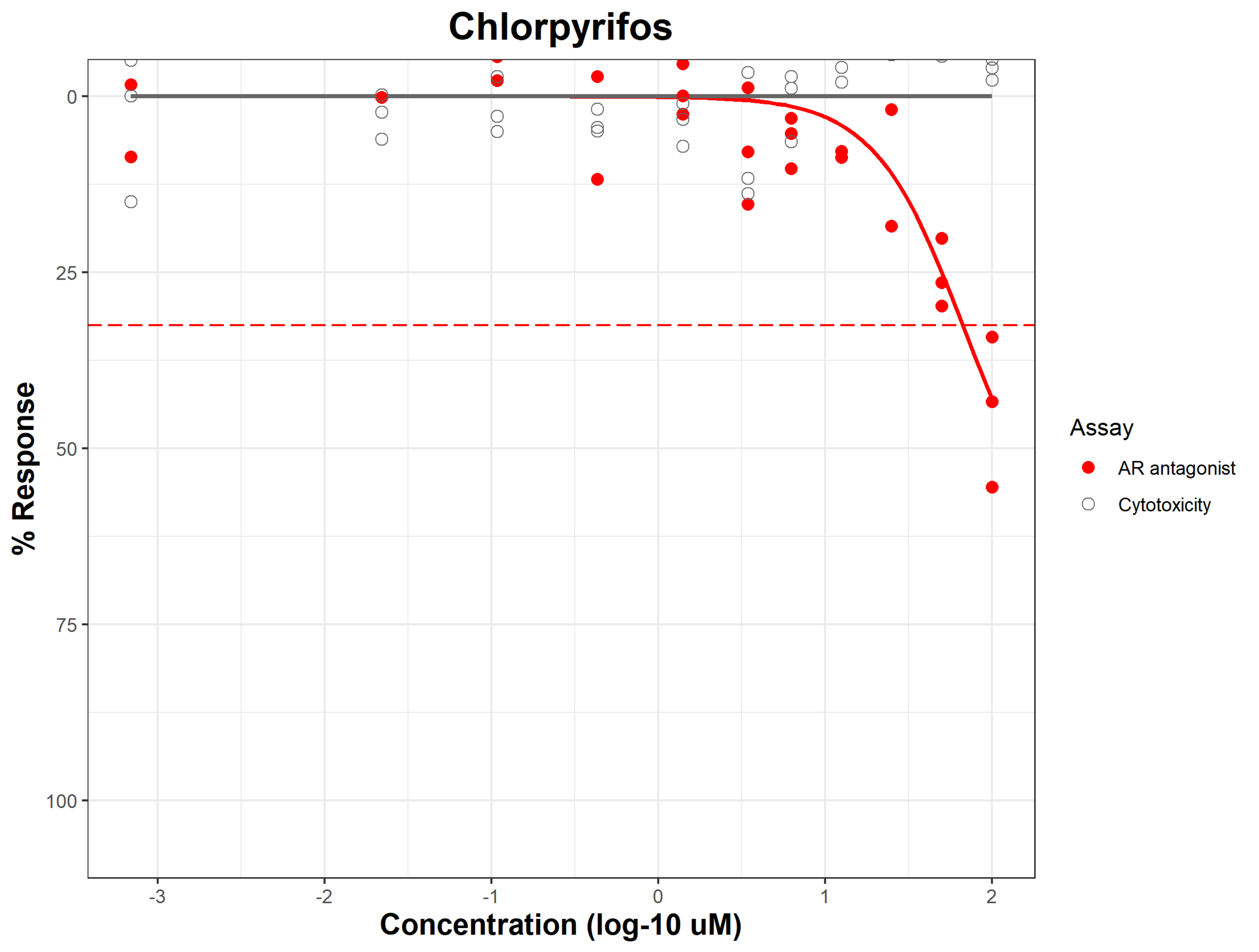

Chlorpyrifos
% Response
0
25
50
75
100
Concentration (log-10 uM)
-3
-2
-1
0
1
2
Assay
AR antagonist
Cytotoxicity

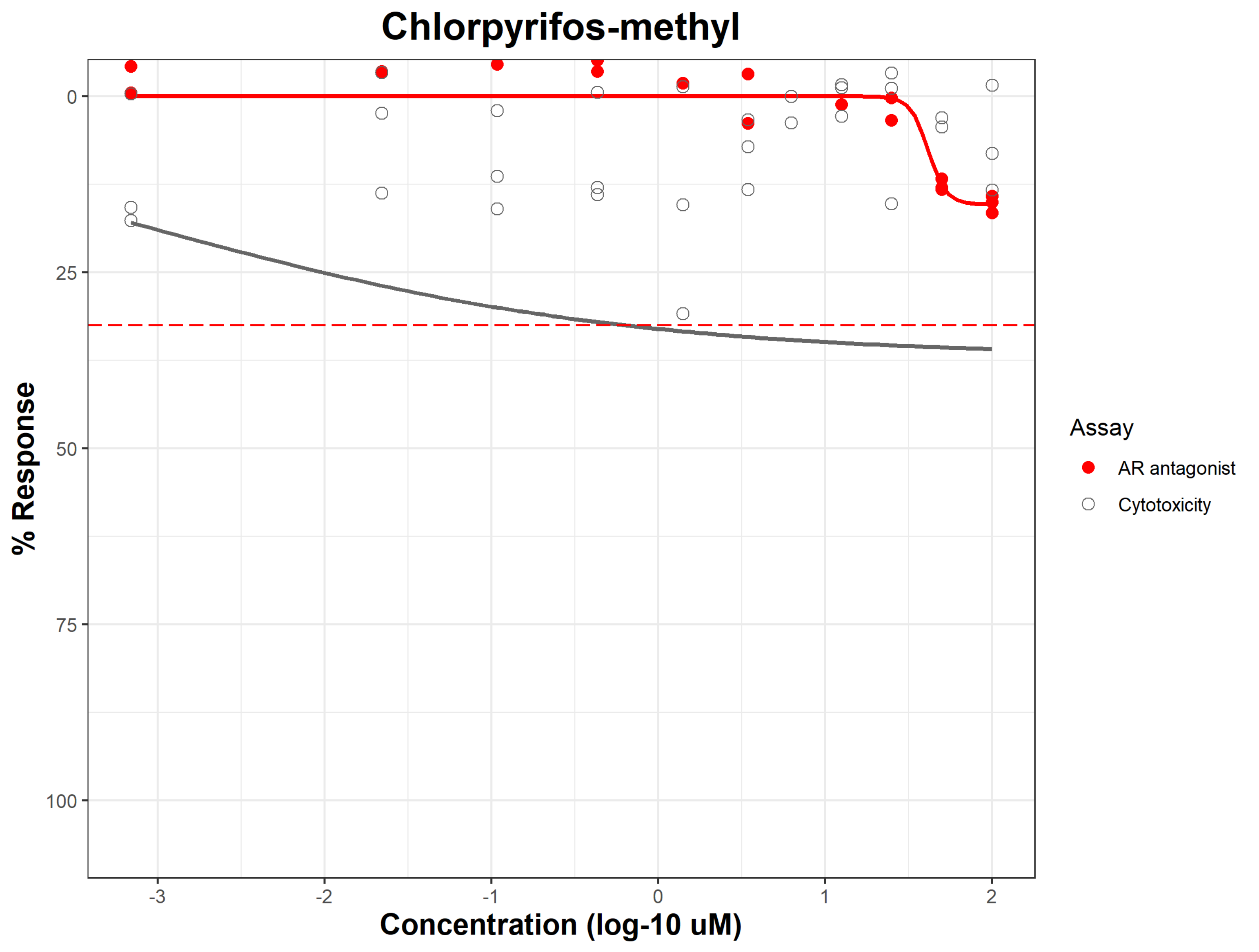

Chlorpyrifos-methyl
% Response
0
25
50
75
100
-3
-2
-1
0
1
2
Concentration (log-10 uM)
Assay
AR antagonist
Cytotoxicity

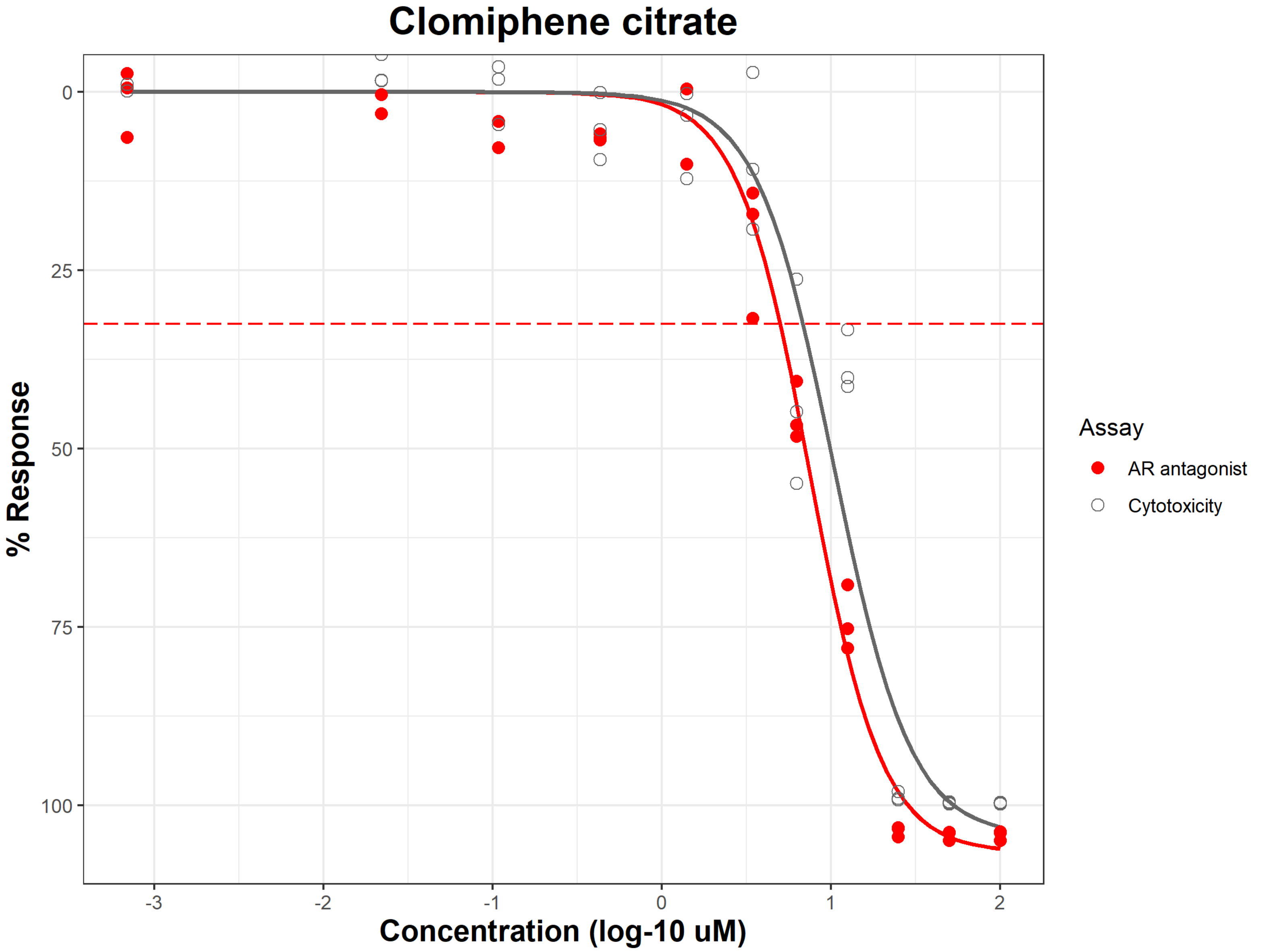

Clomiphene citrate
% Response
0
25
50
75
100
-3
-2
-1
0
1
2
Concentration (log-10 uM)
Assay
AR antagonist
Cytotoxicity

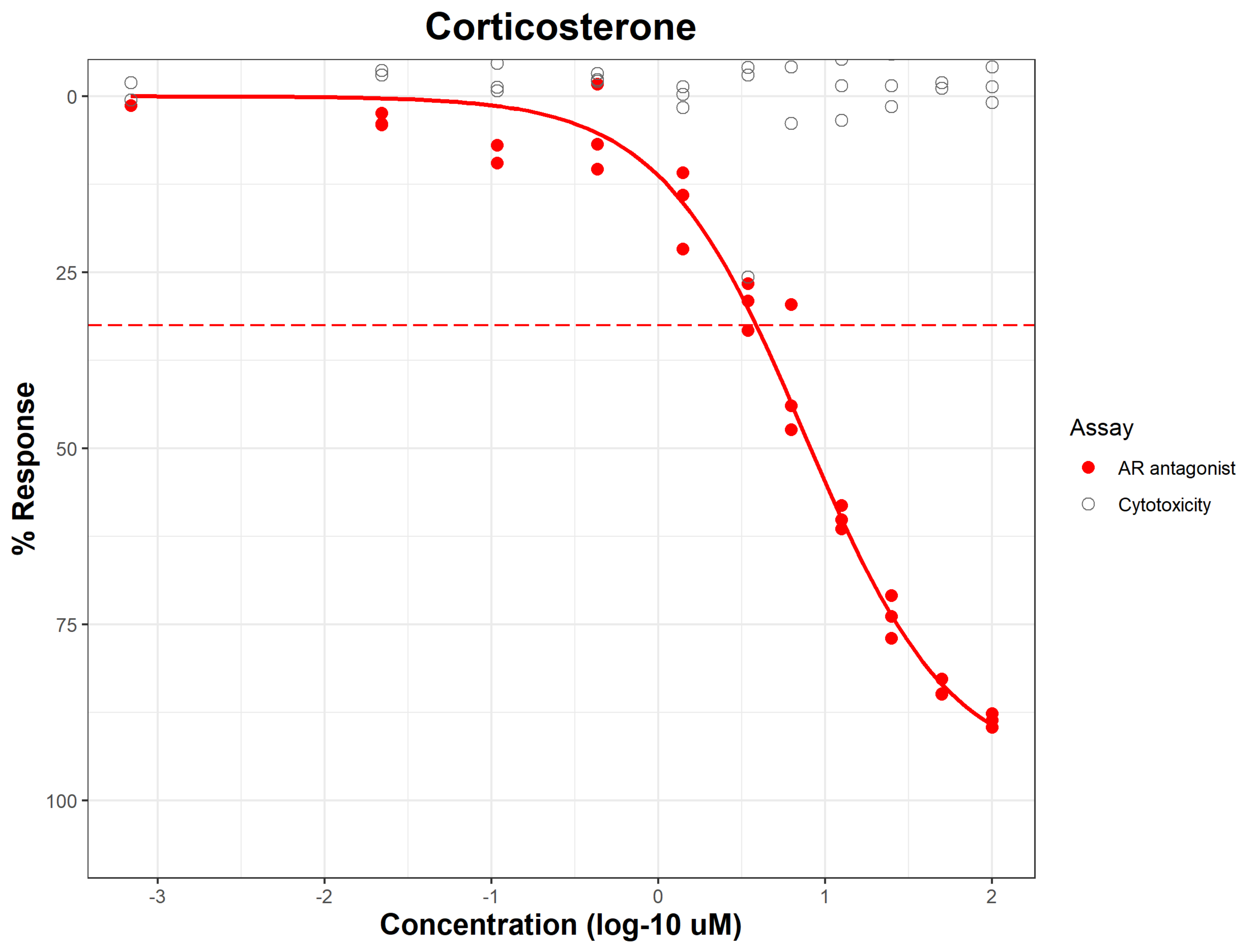

Corticosterone
% Response
0
25
50
75
100
-3
-2
-1
0
1
2
Concentration (log-10 uM)
Assay
AR antagonist
Cytotoxicity

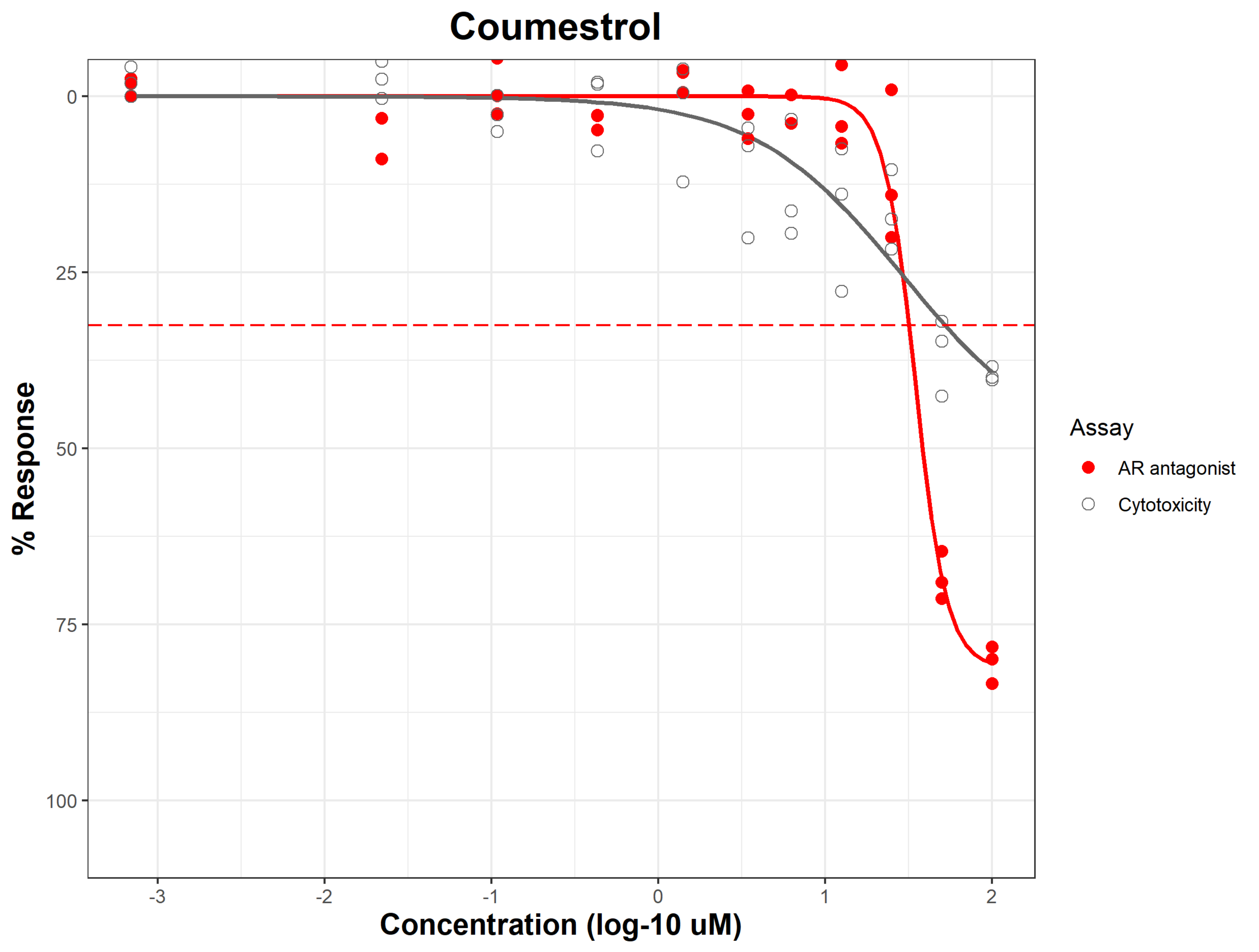

Coumestrol
% Response
0
25
50
75
100
-3
-2
-1
0
1
2
Concentration (log-10 uM)
Assay
AR antagonist
Cytotoxicity

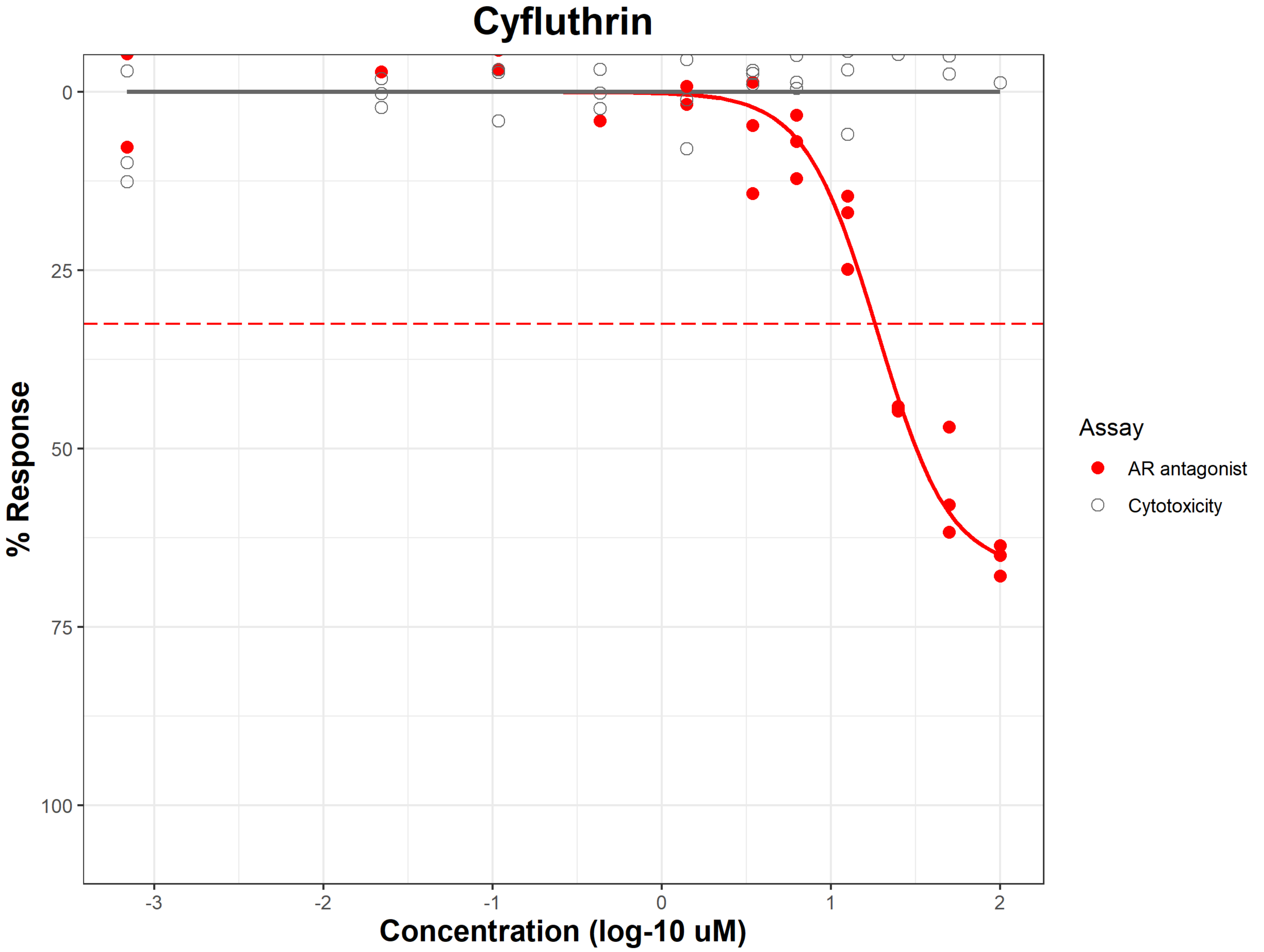

Cyfluthrin
% Response
0
25
50
75
100
-3
-2
-1
0
1
2
Concentration (log-10 uM)
Assay
AR antagonist
Cytotoxicity

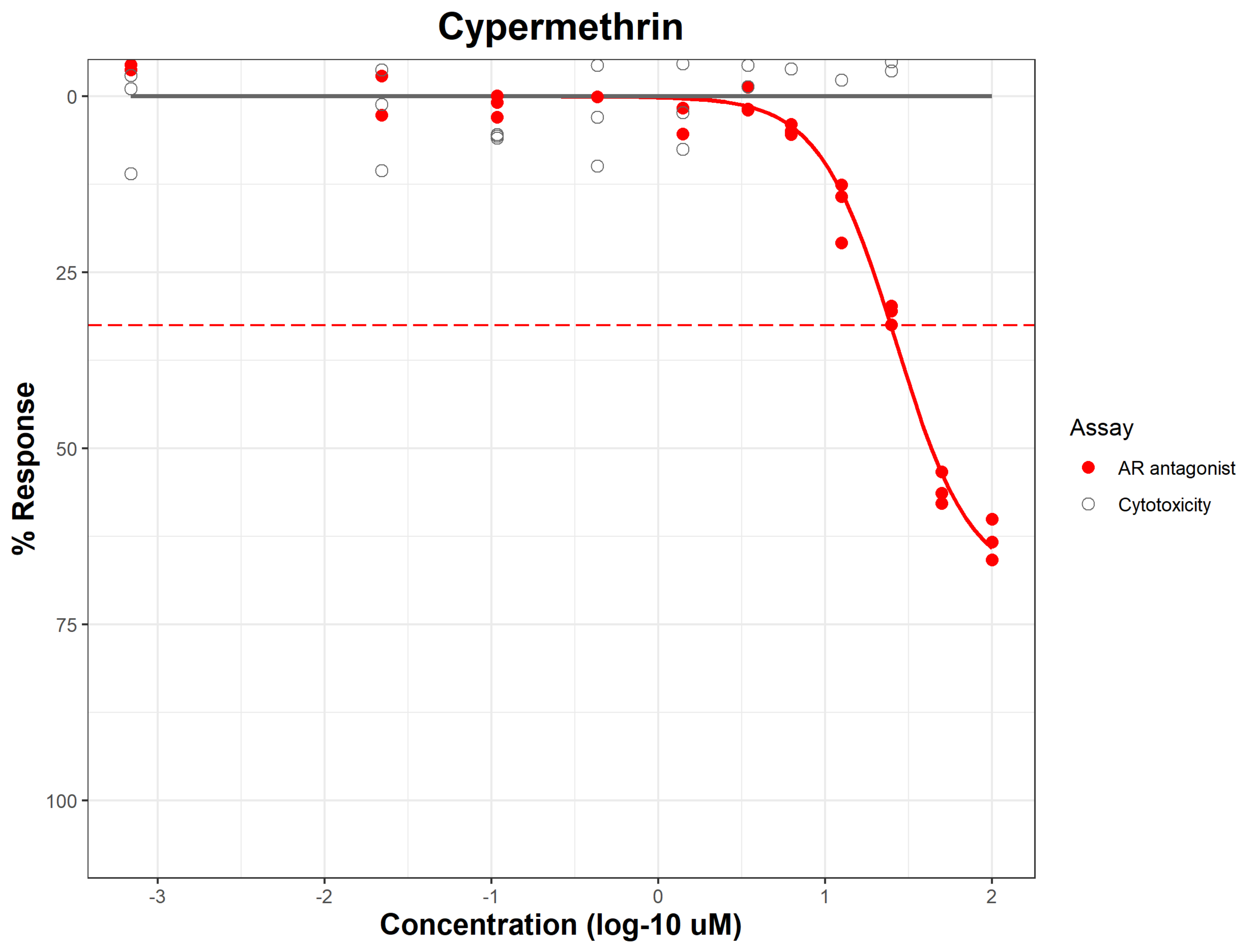

Cypermethrin
% Response
0
25
50
75
100
-3
-2
-1
0
1
2
Concentration (log-10 uM)
Assay
AR antagonist
Cytotoxicity

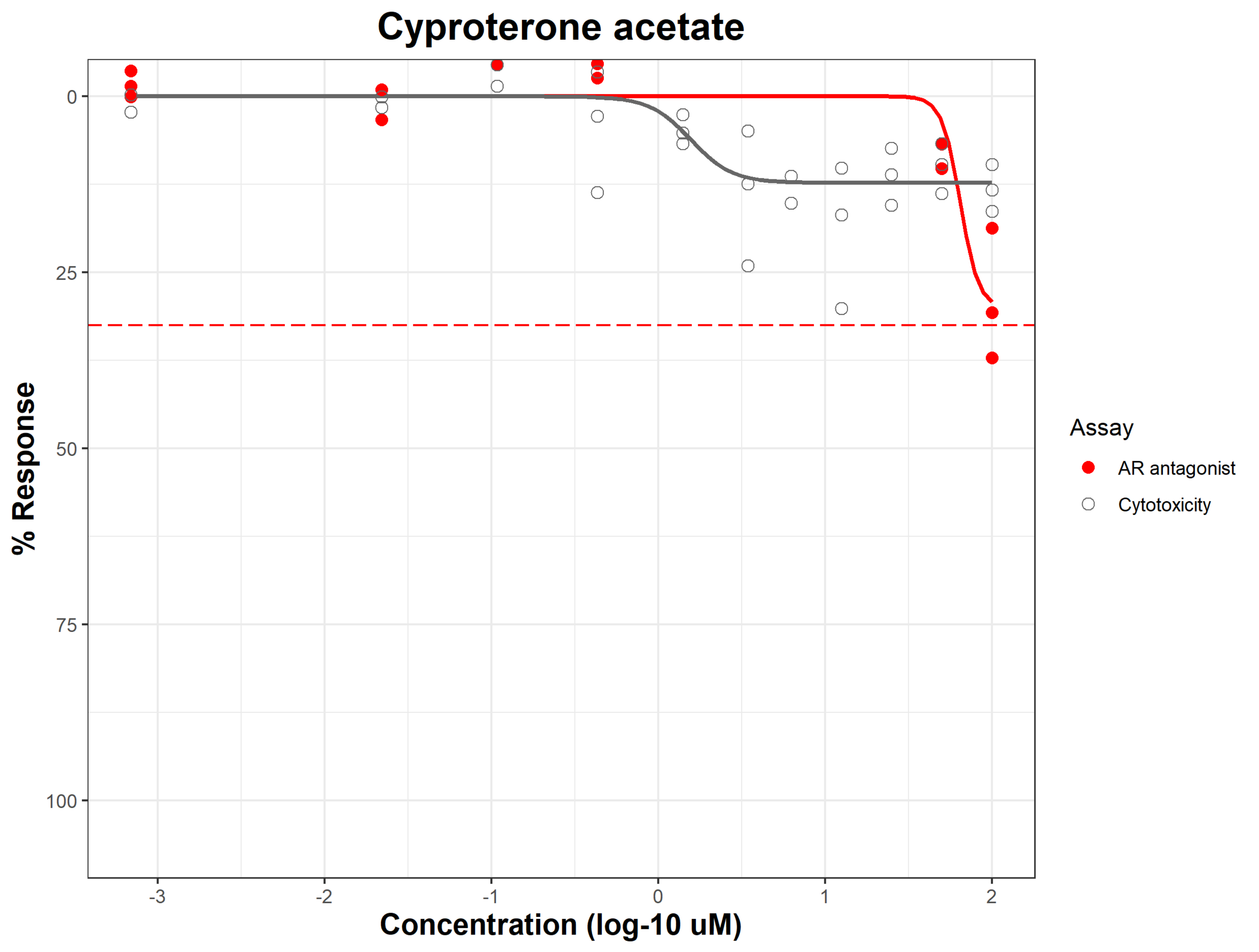
Cyproterone acetate
% Response
0
25
50
75
100
-3
-2
-1
0
1
2
Concentration (log-10 uM)
Assay
AR antagonist
Cytotoxicity

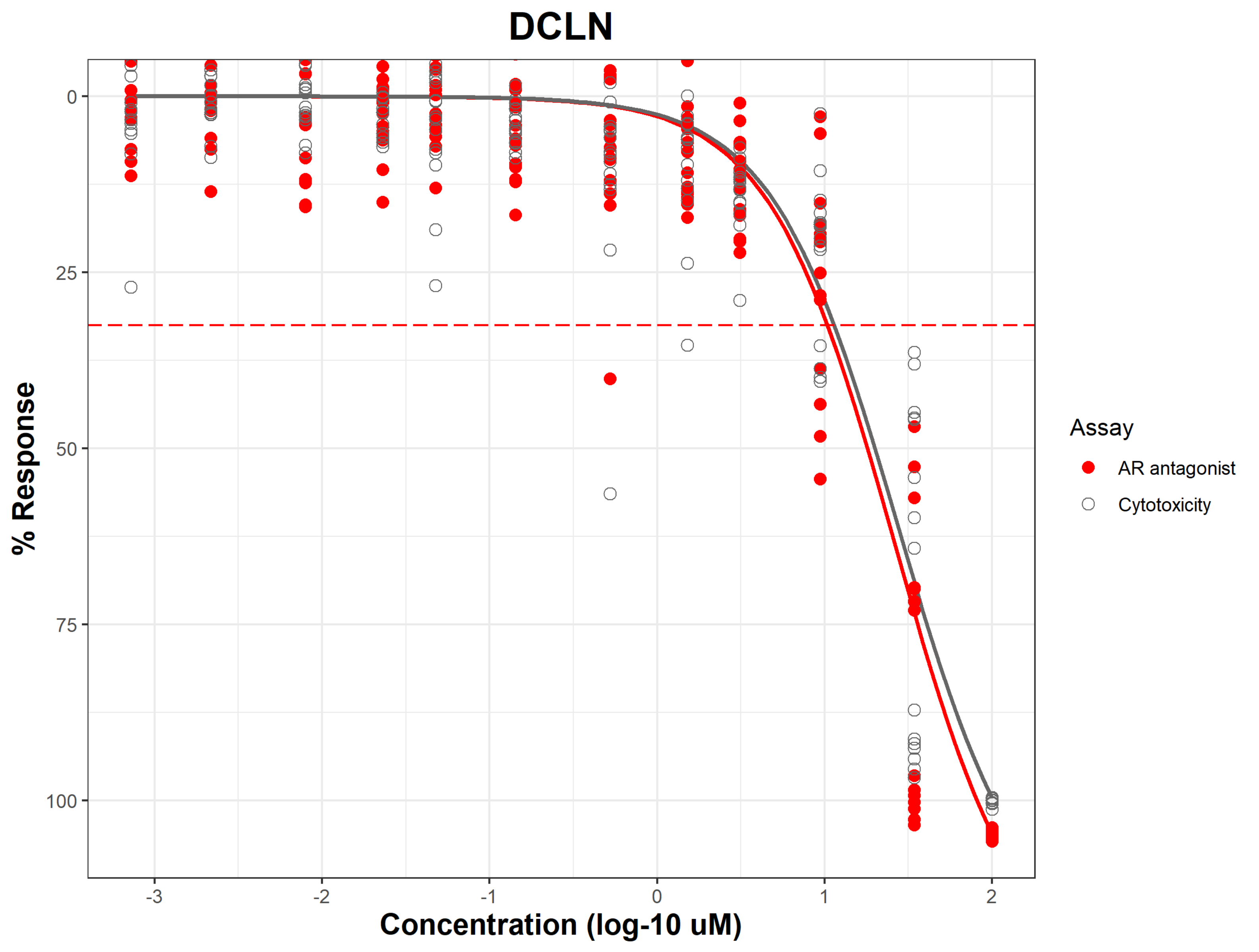

DCLN
% Response
0
25
50
75
100
Concentration (log-10 uM)
-3
-2
-1
0
1
2
Assay
AR antagonist
Cytotoxicity

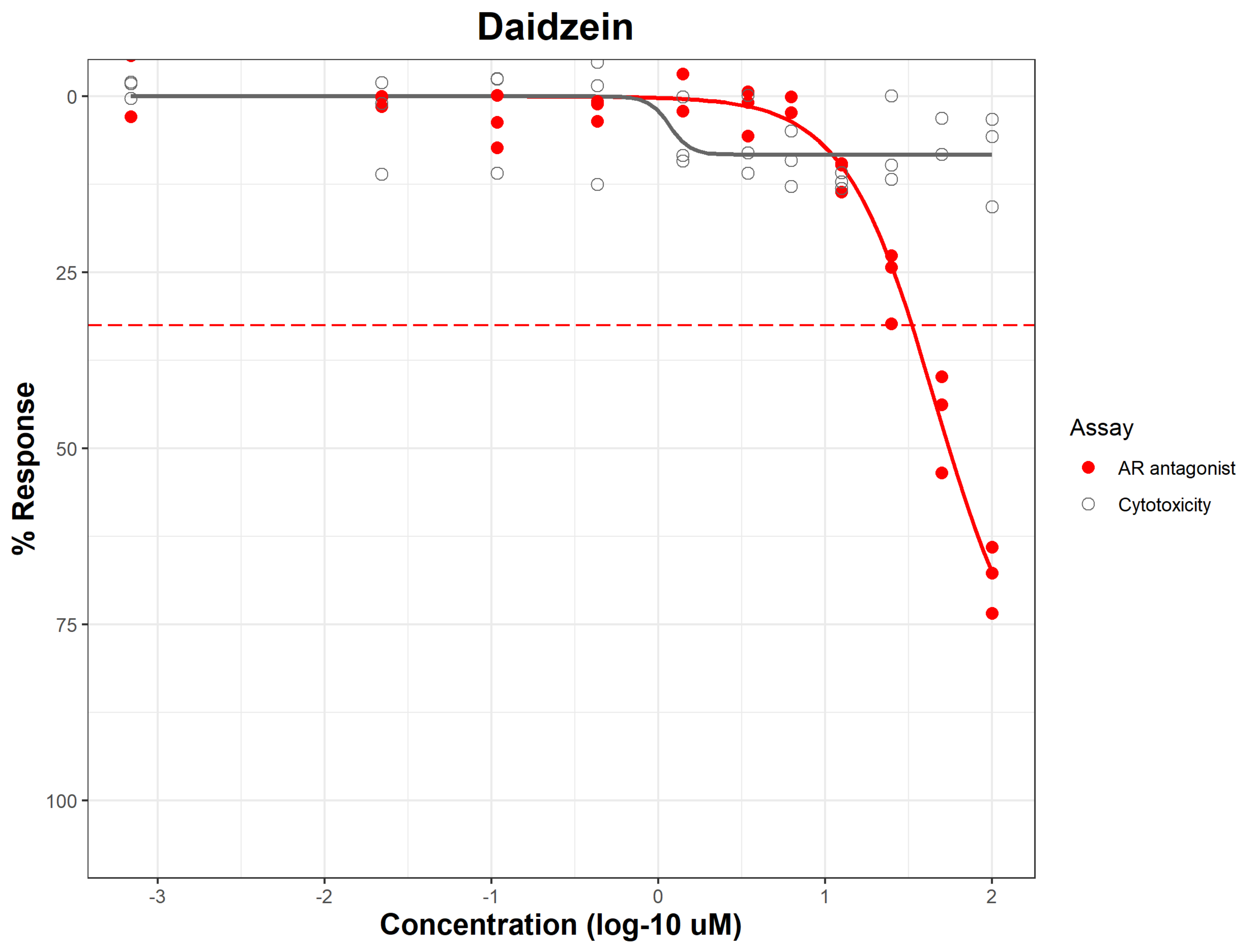

Daidzein
% Response
0
25
50
75
100
-3
-2
-1
0
1
2
Concentration (log-10 uM)
Assay
AR antagonist
Cytotoxicity

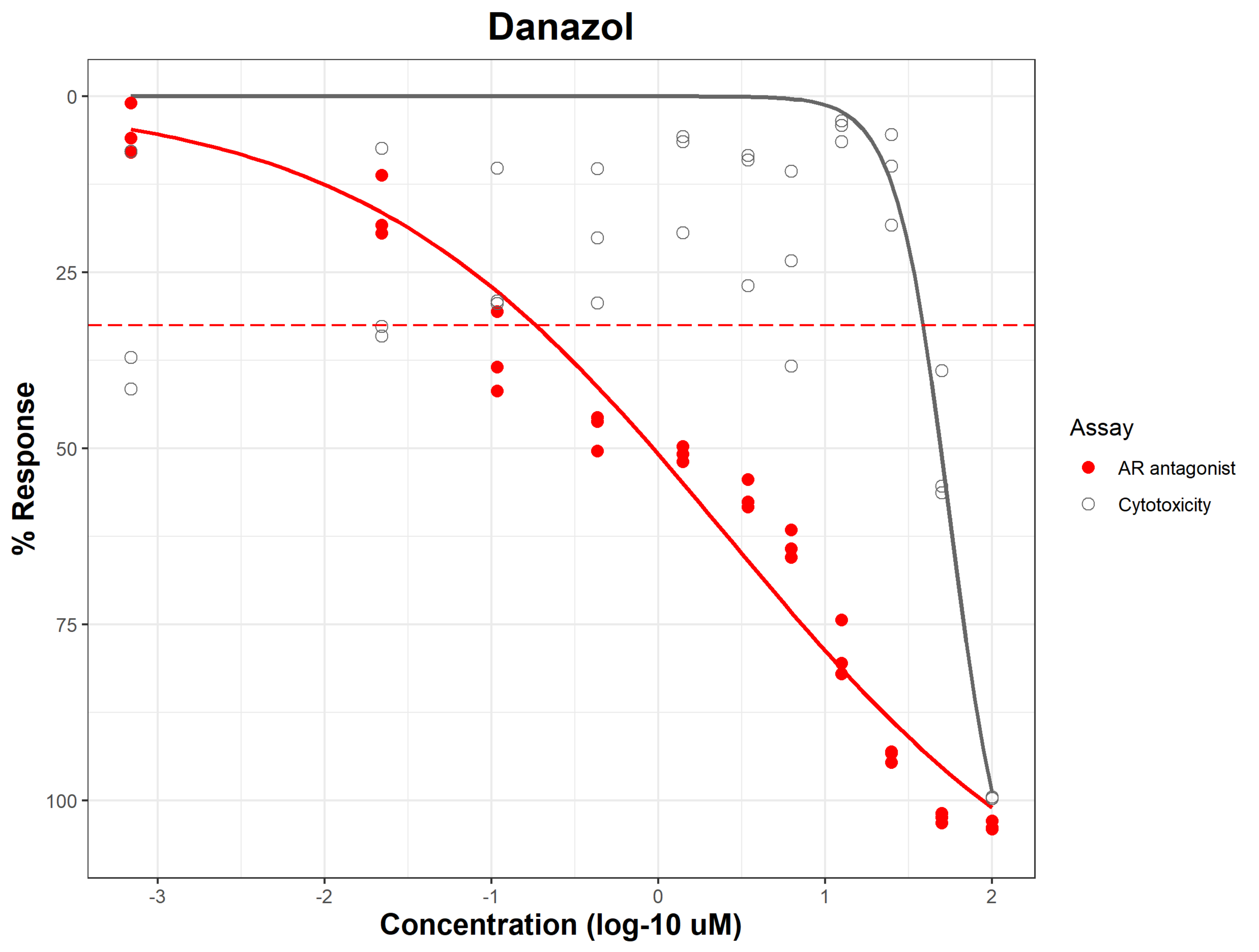

Danazol
% Response
0
25
50
75
100
-3
-2
-1
0
1
2
Concentration (log-10 uM)
Assay
AR antagonist
Cytotoxicity

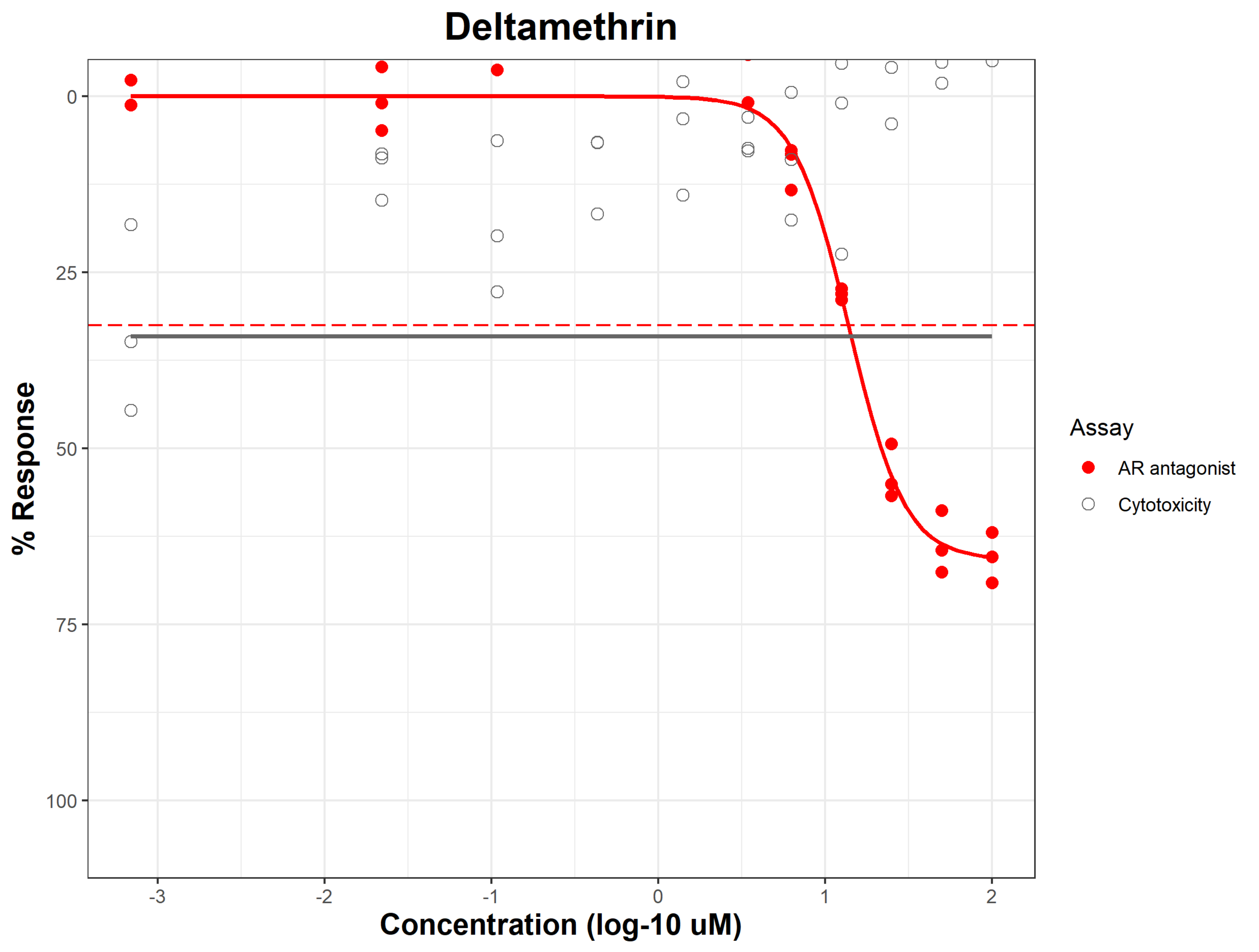
Deltamethrin
% Response
0
25
50
75
100
-3
-2
-1
0
1
2
Concentration (log-10 uM)
Assay
AR antagonist
Cytotoxicity

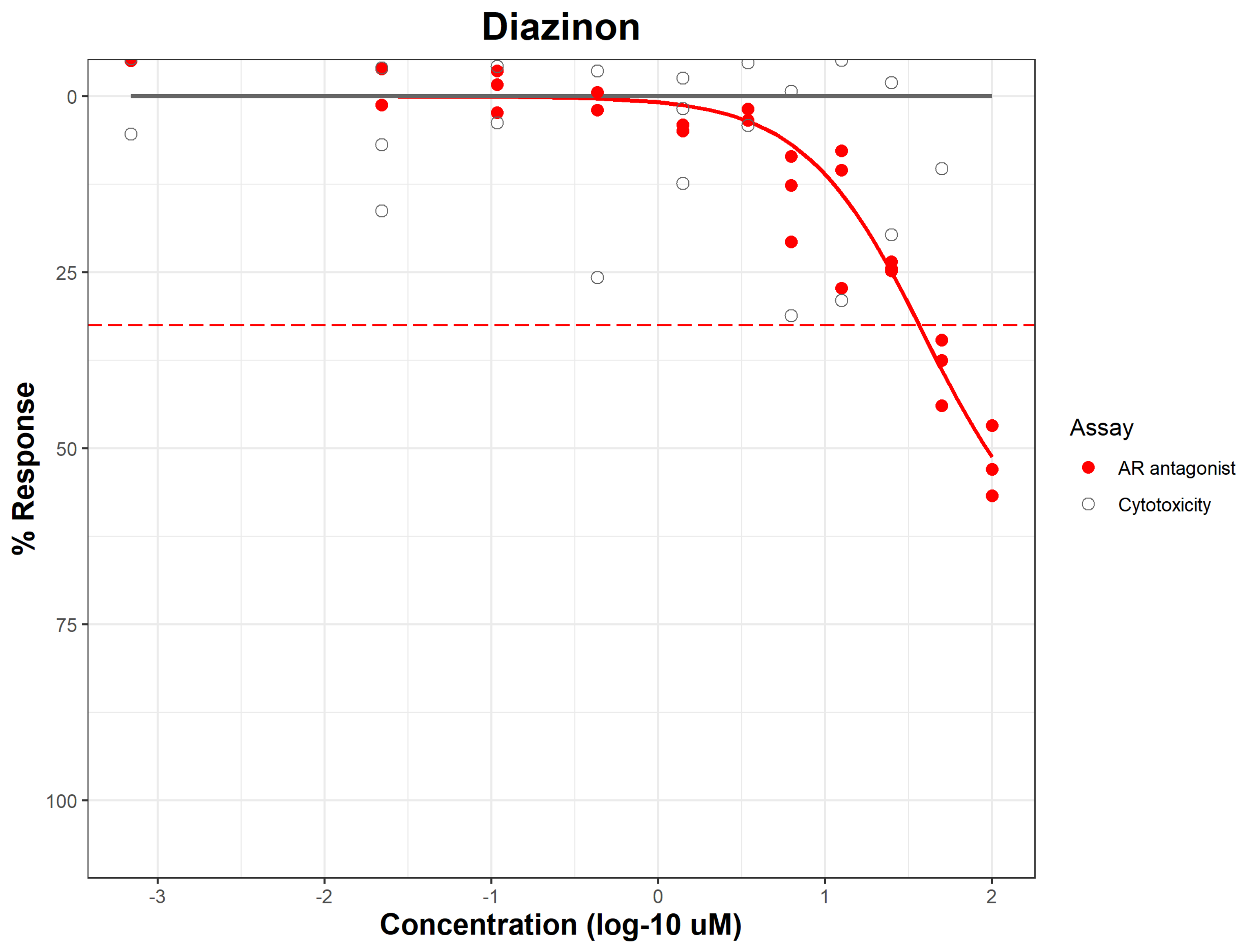
Diazinon
% Response
0
25
50
75
100
-3
-2
-1
0
1
2
Concentration (log-10 uM)
Assay
AR antagonist
Cytotoxicity

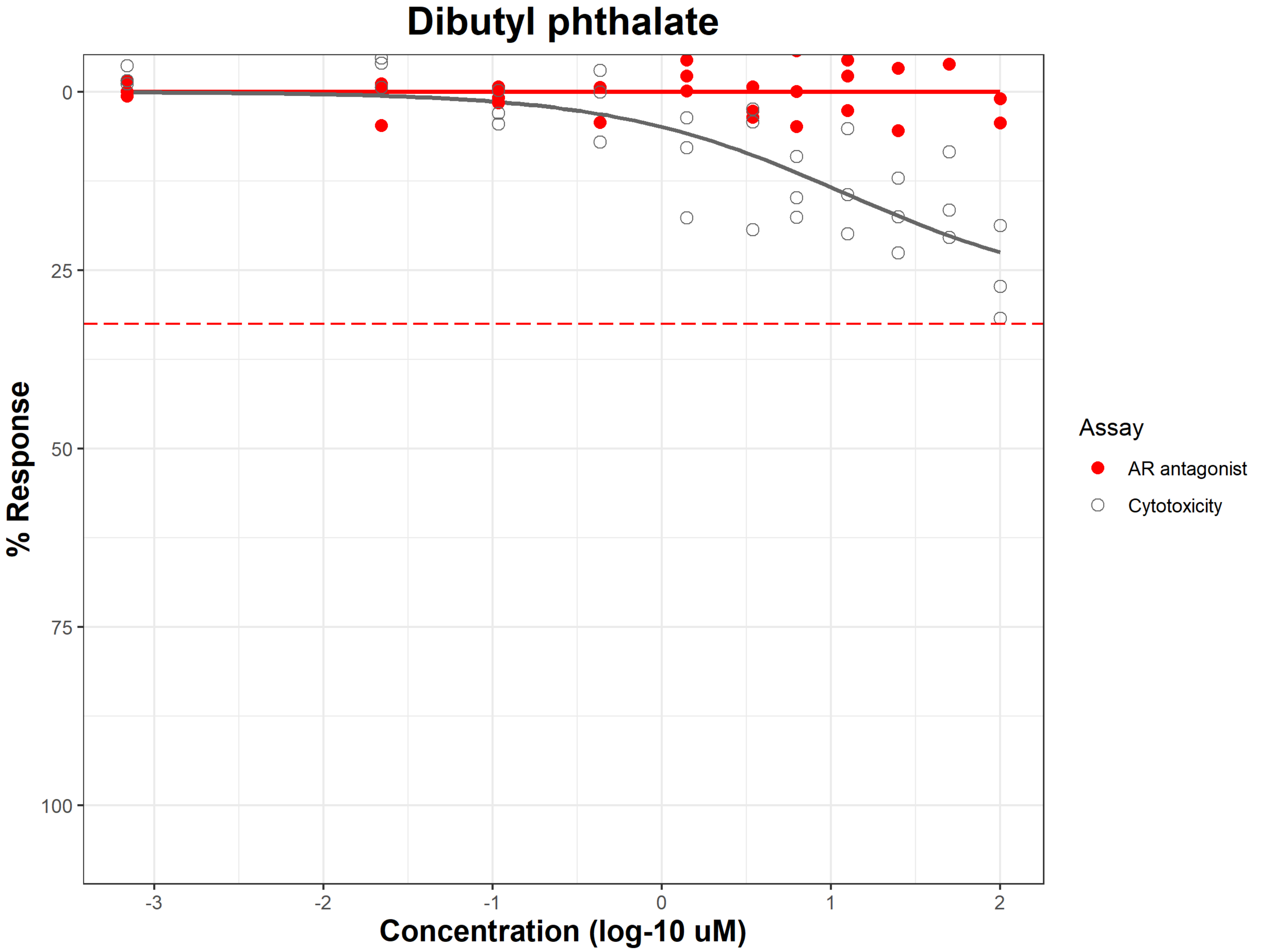

Dibutyl phthalate
% Response
0
25
50
75
100
-3
-2
-1
0
1
2
Concentration (log-10 uM)
Assay
AR antagonist
Cytotoxicity

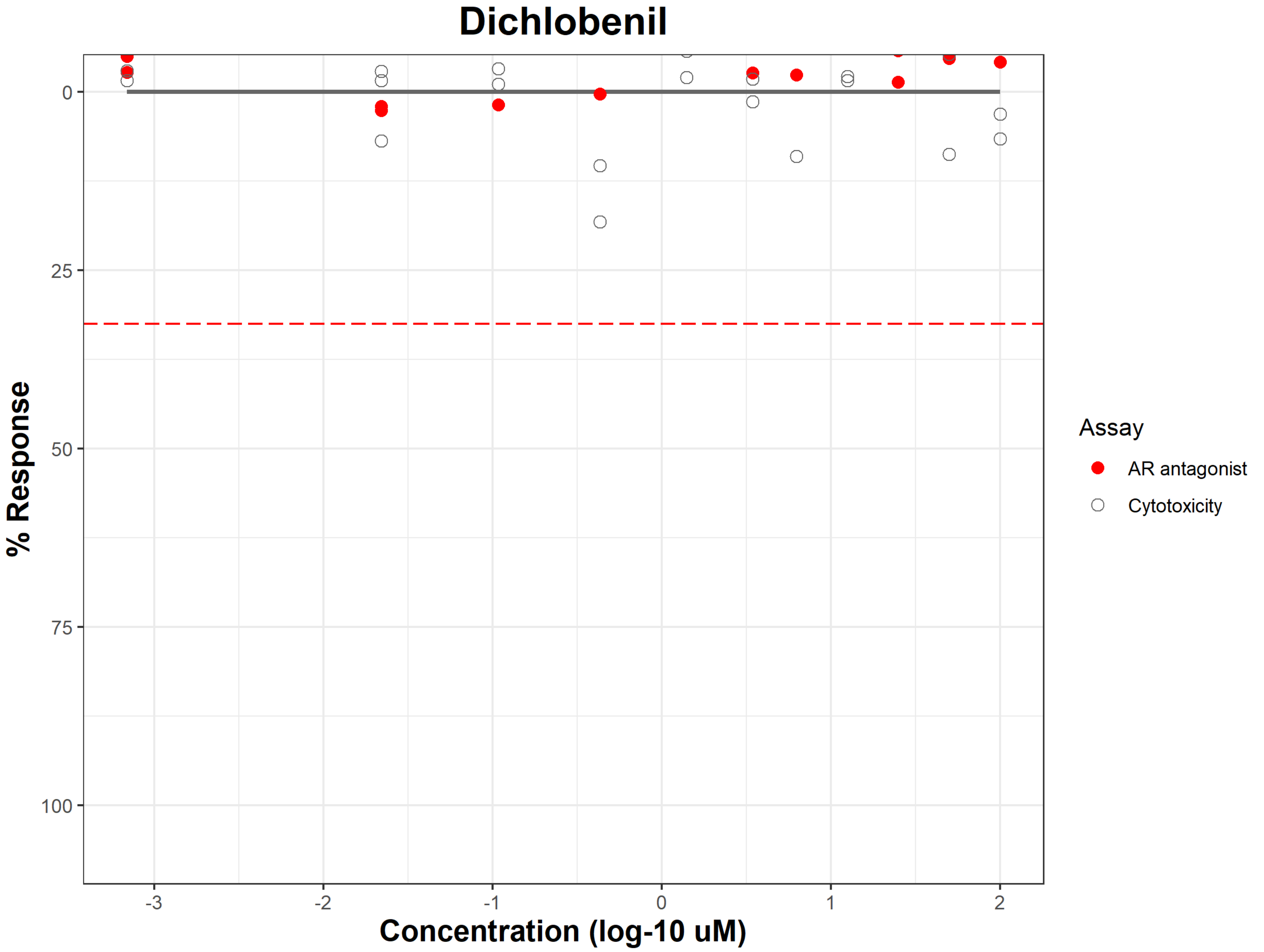
Dichlobenil
% Response
0
25
50
75
100
Concentration (log-10 uM)
-3
-2
-1
0
1
2
Assay
AR antagonist
Cytotoxicity

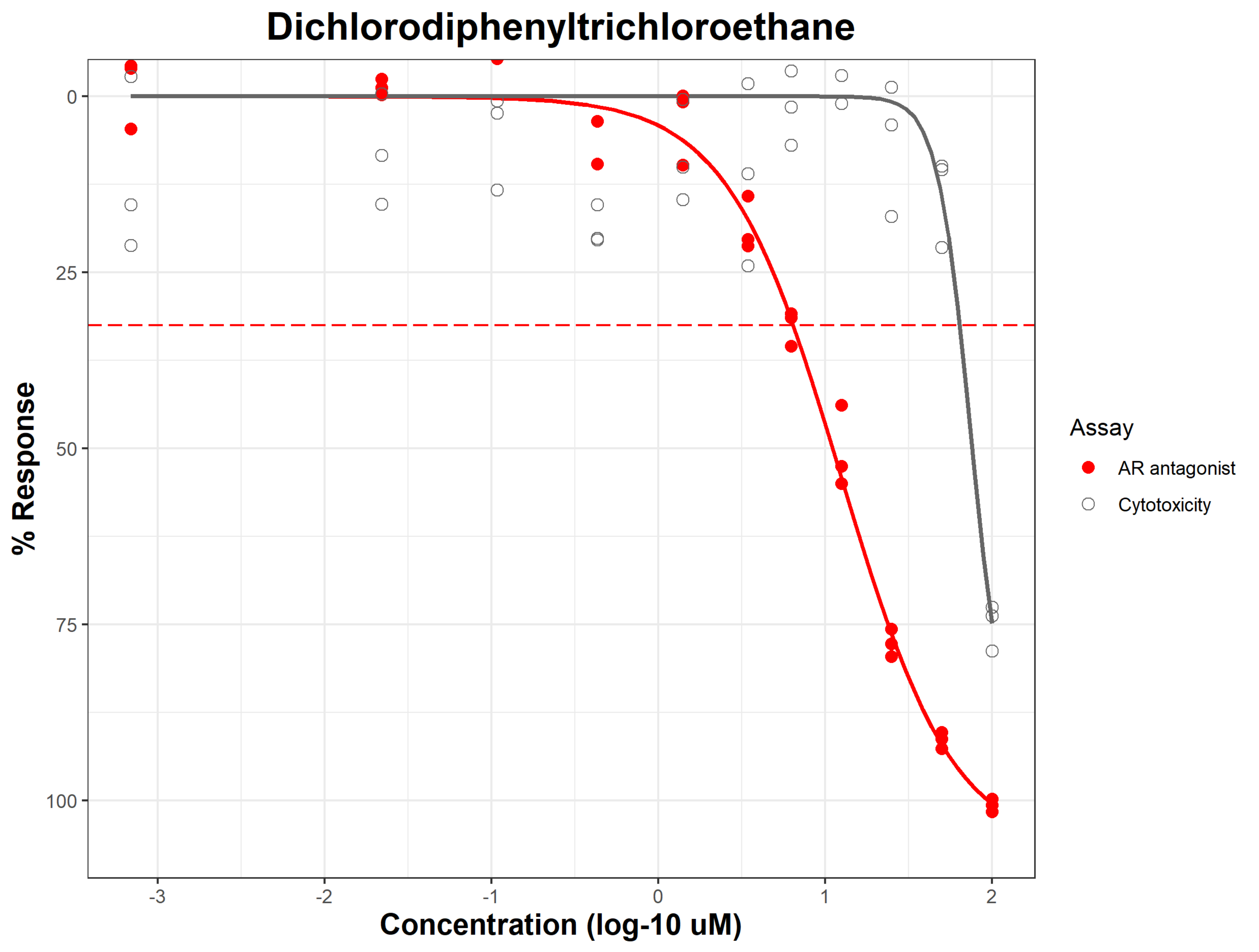

Dichlorodiphenyltrichloroethane
% Response
Concentration (log-10 uM)
0
25
50
75
100
-3
-2
-1
0
1
2
Assay
AR antagonist
Cytotoxicity

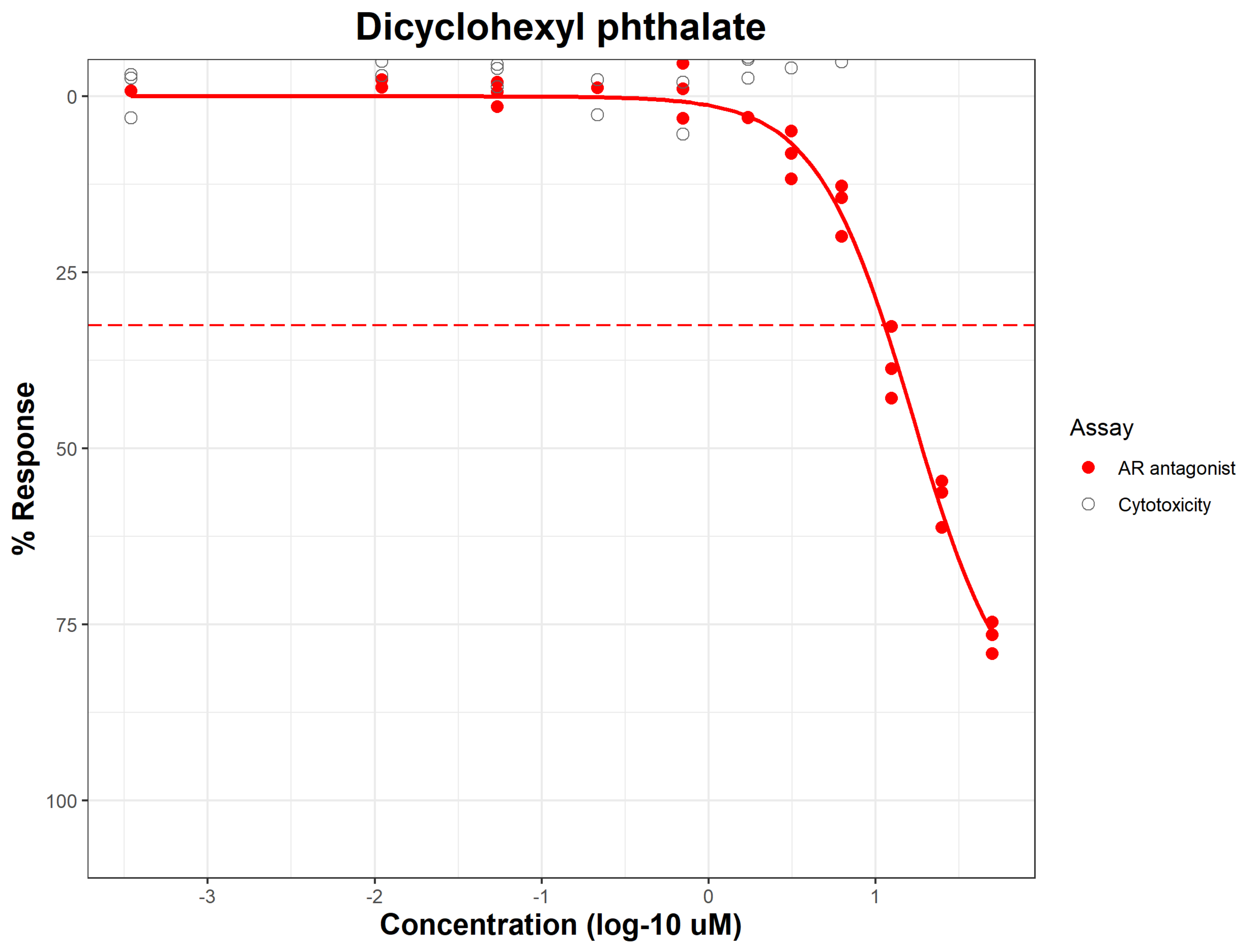
Dicyclohexyl phthalate
% Response
0
25
50
75
100
-3
-2
-1
0
1
Concentration (log-10 uM)
Assay
AR antagonist
Cytotoxicity

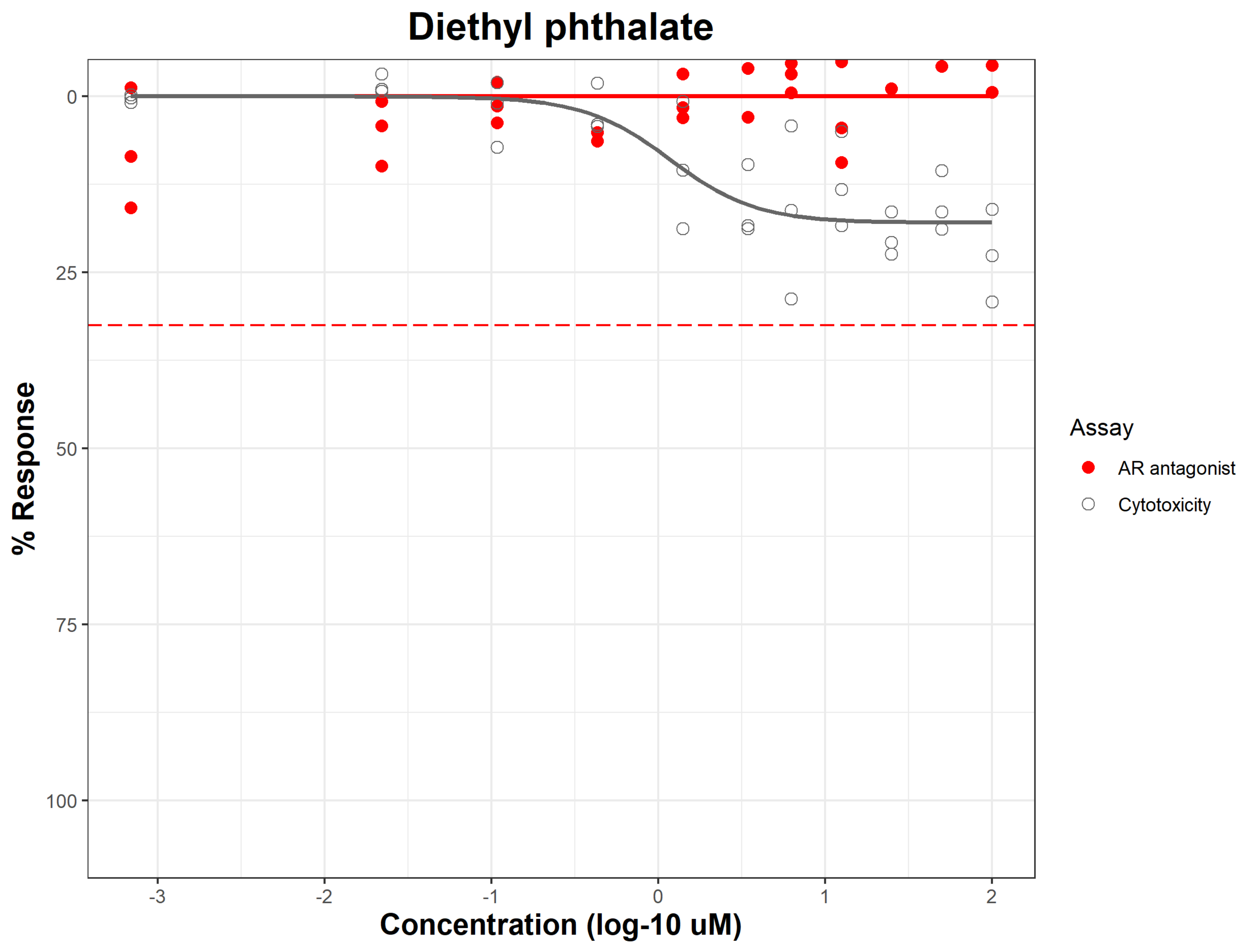
Diethyl phthalate
% Response
0
25
50
75
100
Concentration (log-10 uM)
-3
-2
-1
0
1
2
Assay
AR antagonist
Cytotoxicity

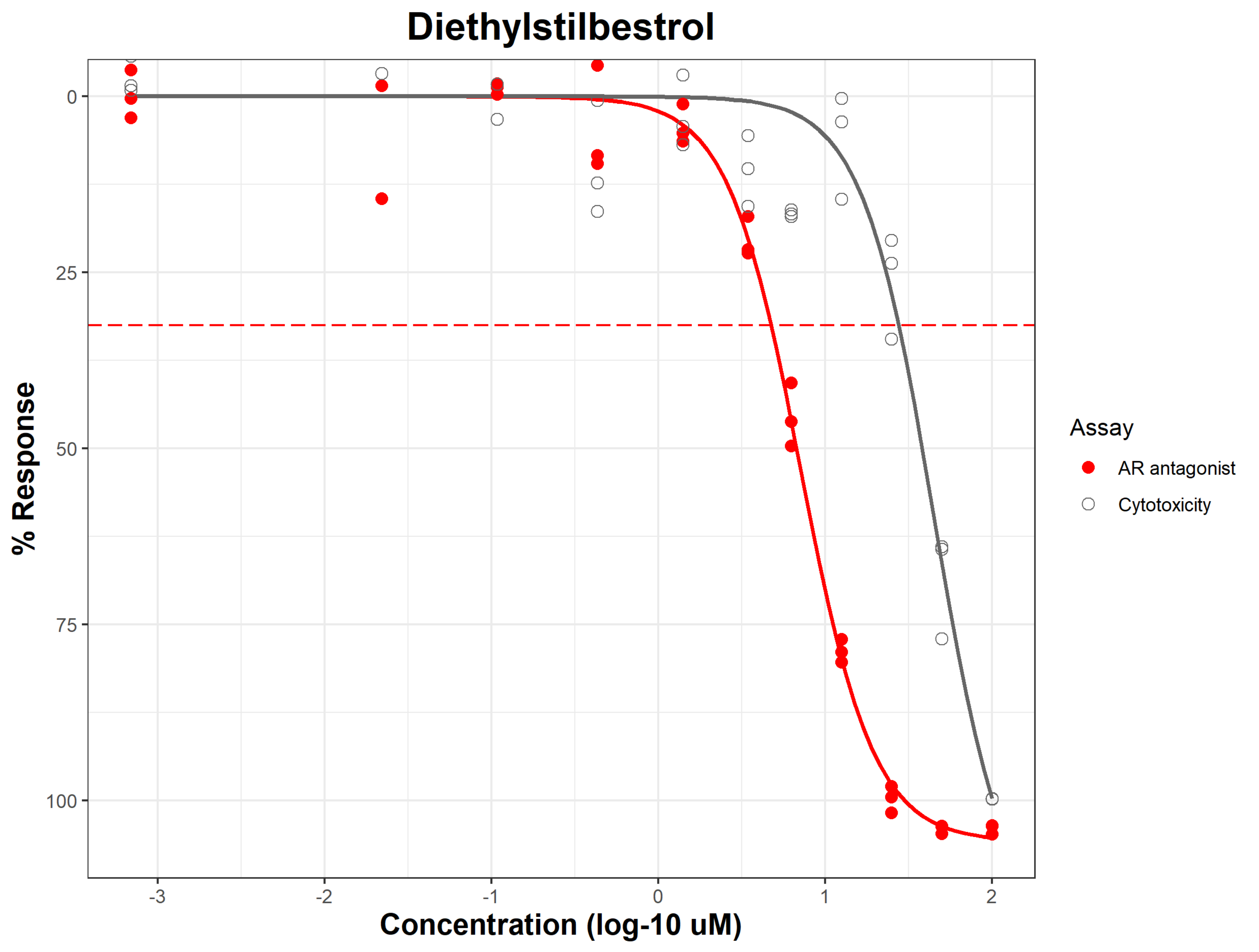

Diethylstilbestrol
% Response
0
25
50
75
100
Concentration (log-10 uM)
-3
-2
-1
0
1
2
Assay
AR antagonist
Cytotoxicity

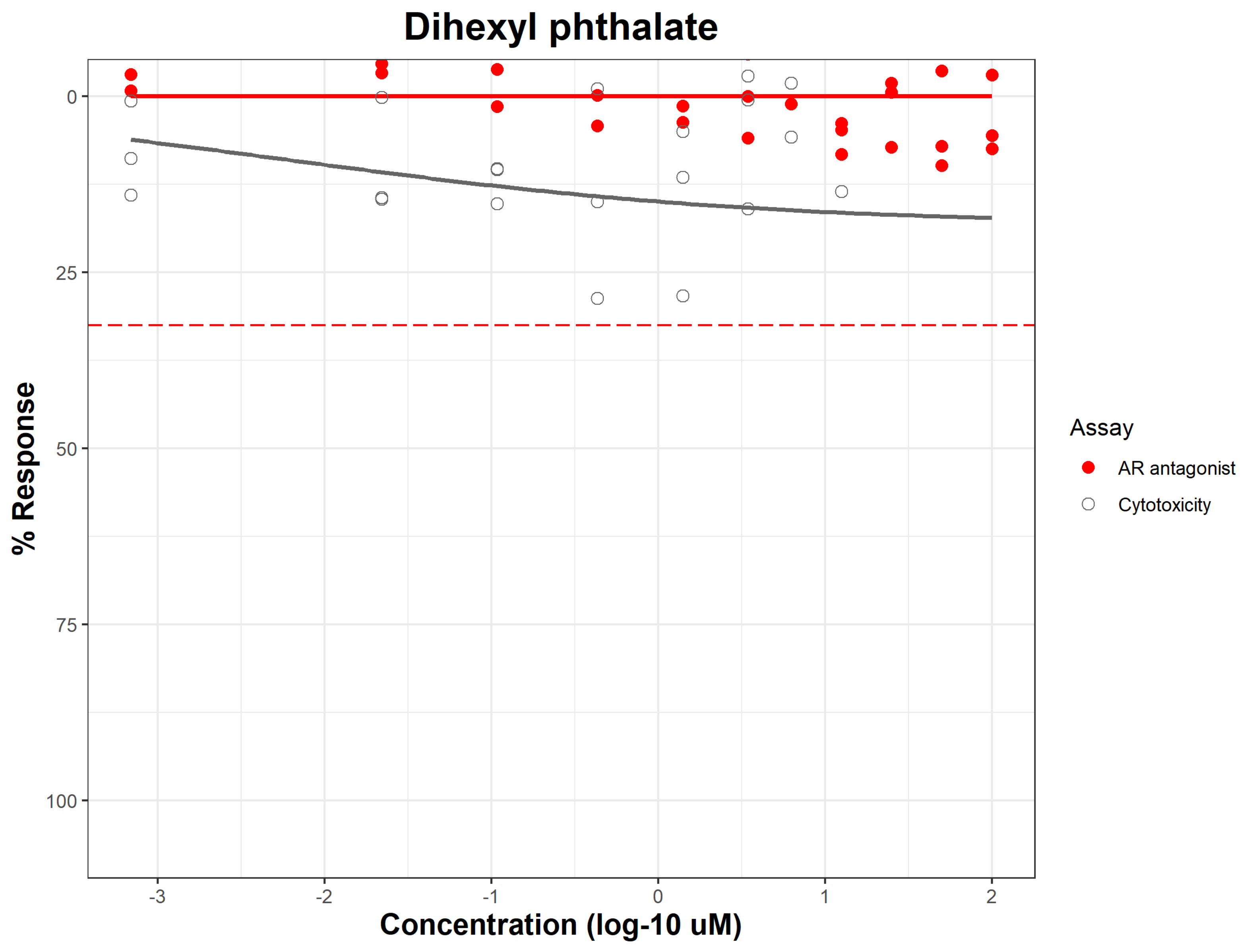

Dihexyl phthalate
% Response
0
25
50
75
100
-3
-2
-1
0
1
2
Concentration (log-10 uM)
Assay
AR antagonist
Cytotoxicity

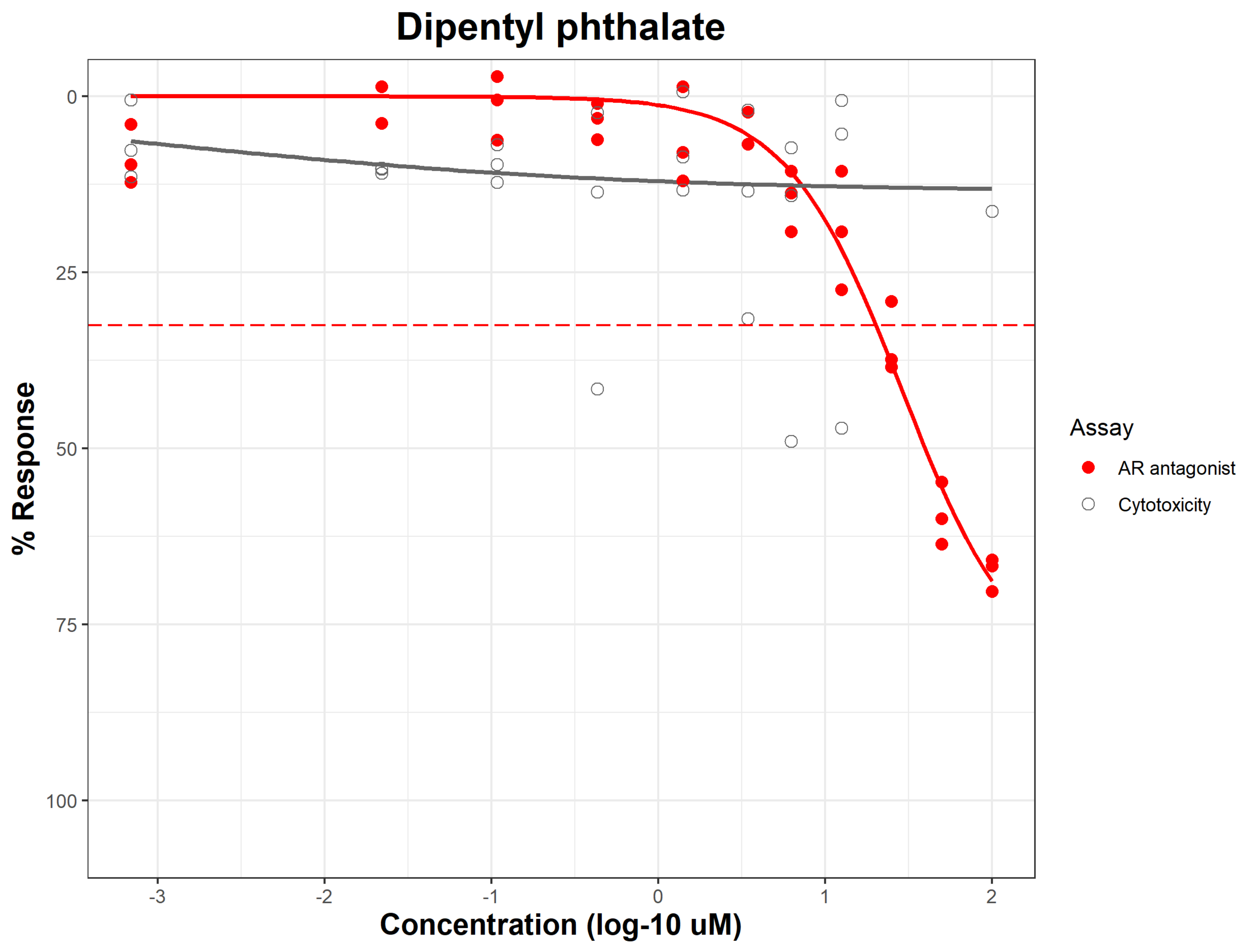

Dipentyl phthalate
% Response
0
25
50
75
100
-3
-2
-1
0
1
2
Concentration (log-10 uM)
Assay
AR antagonist
Cytotoxicity

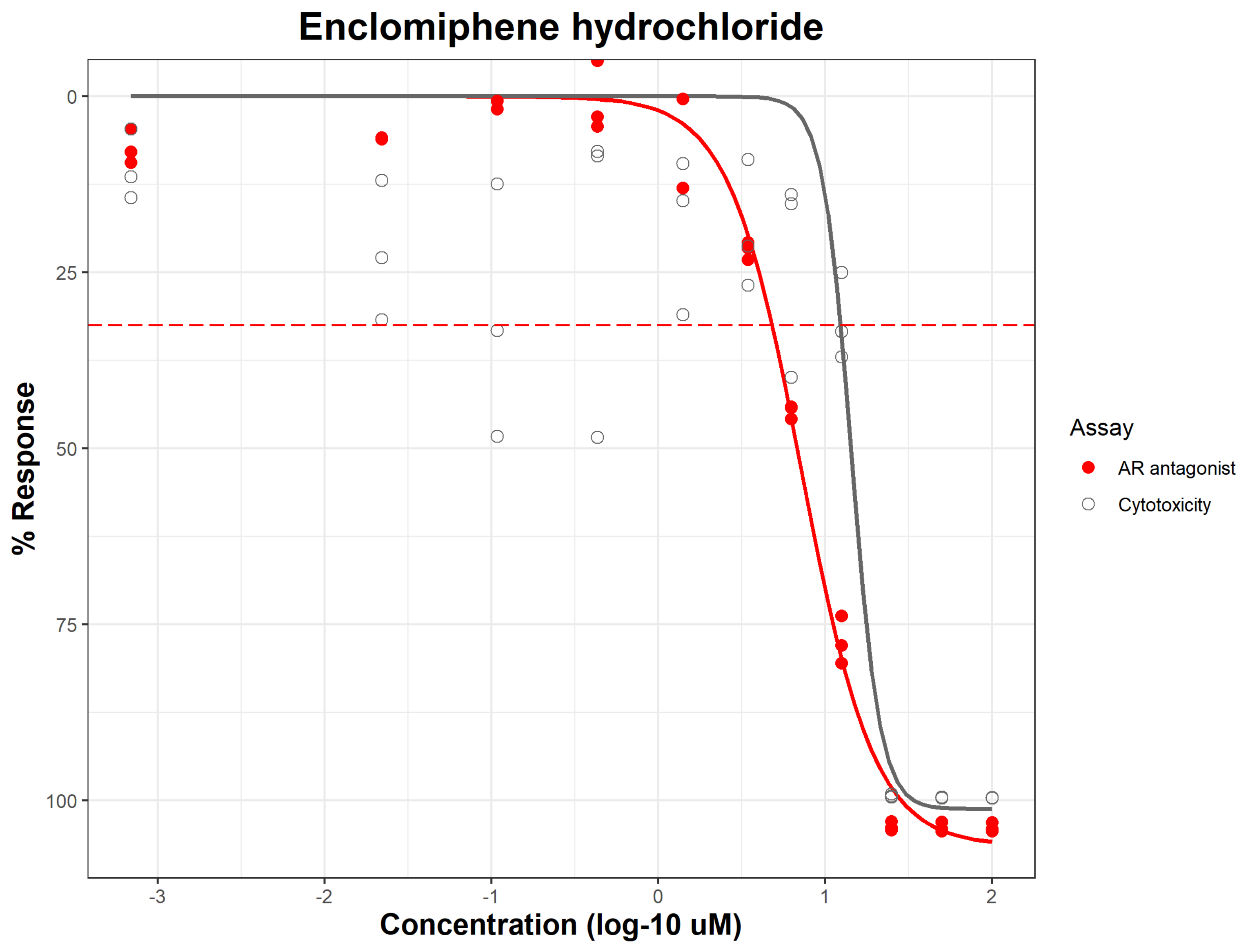
Enclomiphene hydrochloride
% Response
0
25
50
75
100
-3
-2
-1
0
1
2
Concentration (log-10 uM)
Assay
AR antagonist
Cytotoxicity

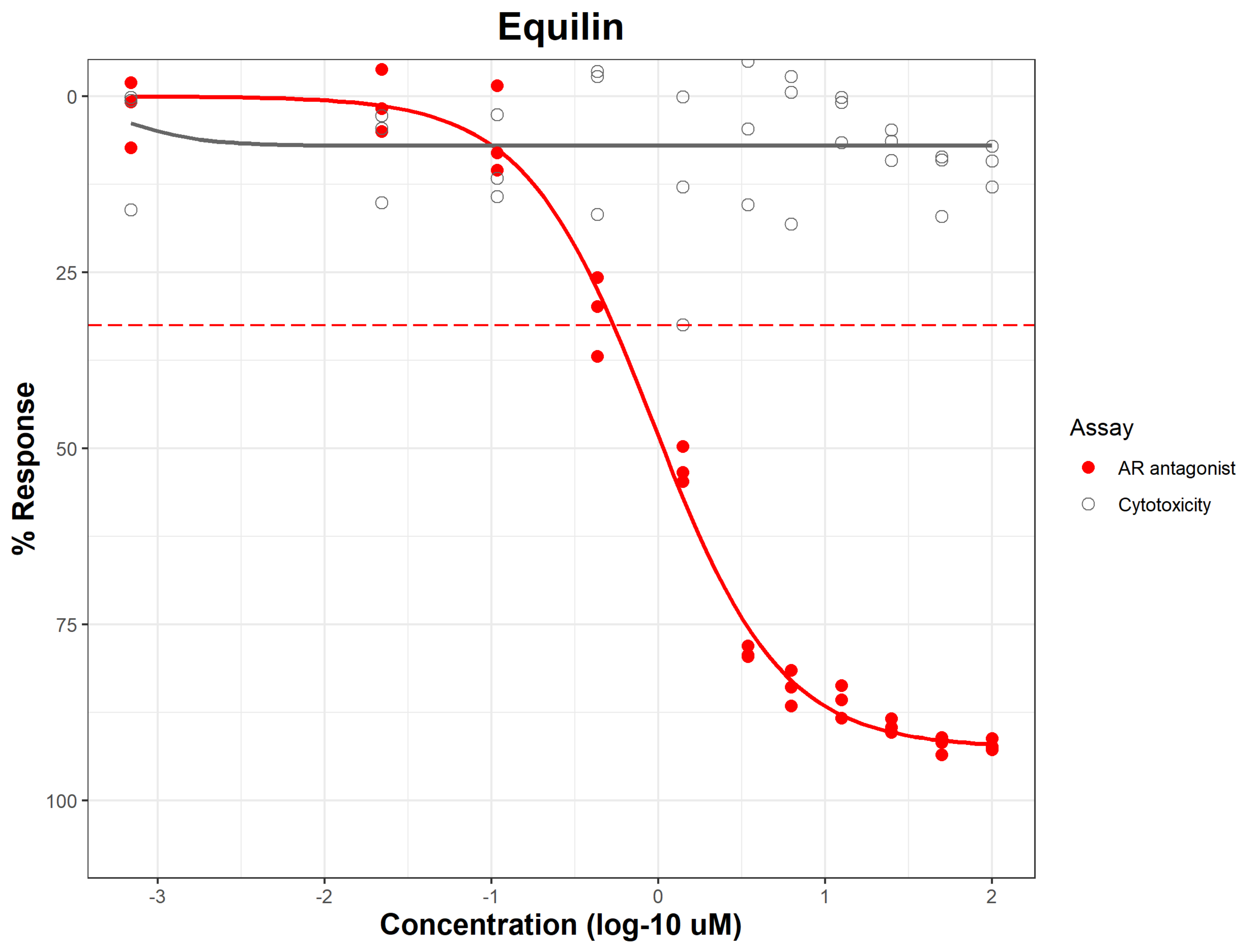

Equilin
% Response
0
25
50
75
100
-3
-2
-1
0
1
2
Concentration (log-10 uM)
Assay
AR antagonist
Cytotoxicity

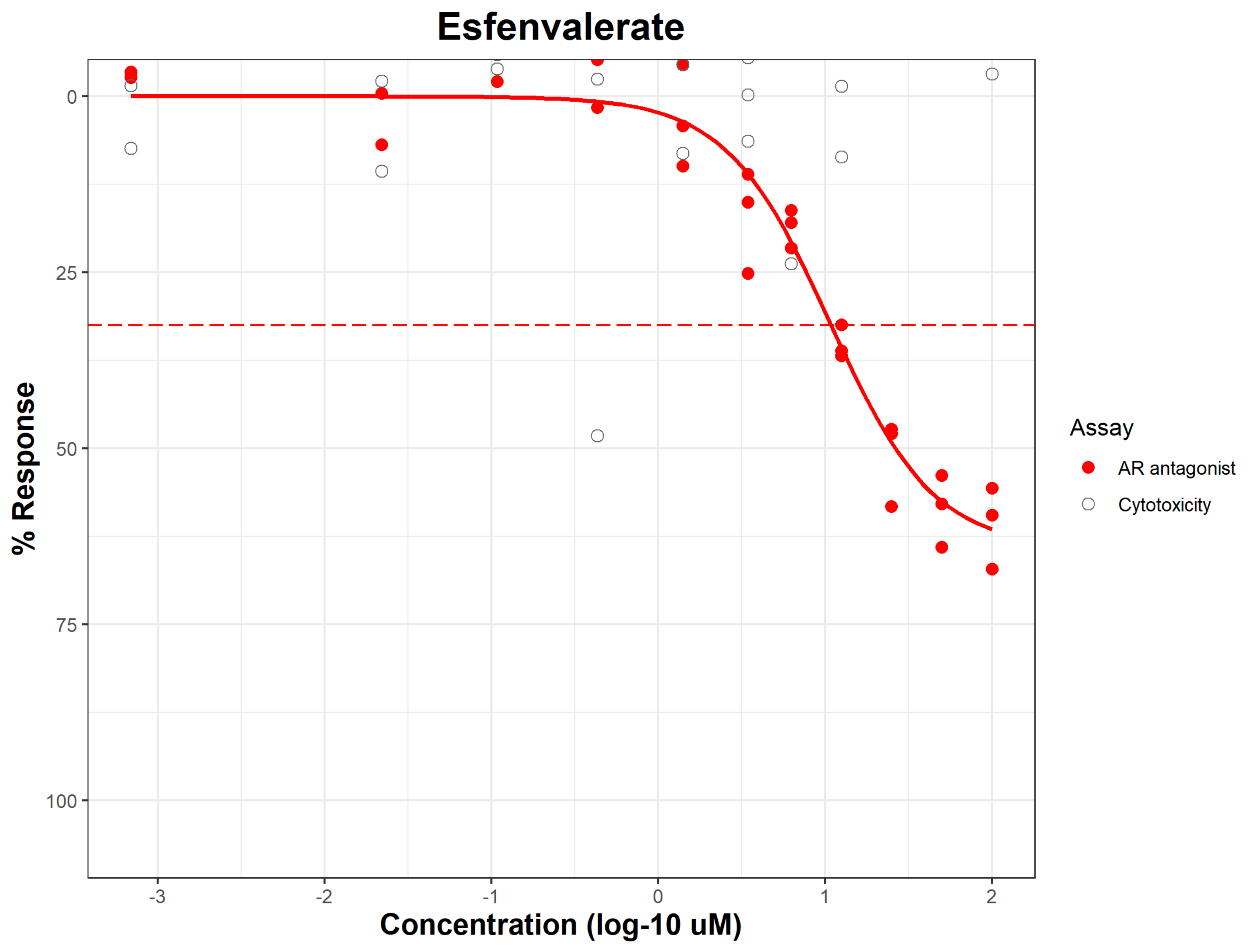

Esfenvalerate
% Response
0
25
50
75
100
-3
-2
-1
0
1
2
Concentration (log-10 uM)
Assay
AR antagonist
Cytotoxicity

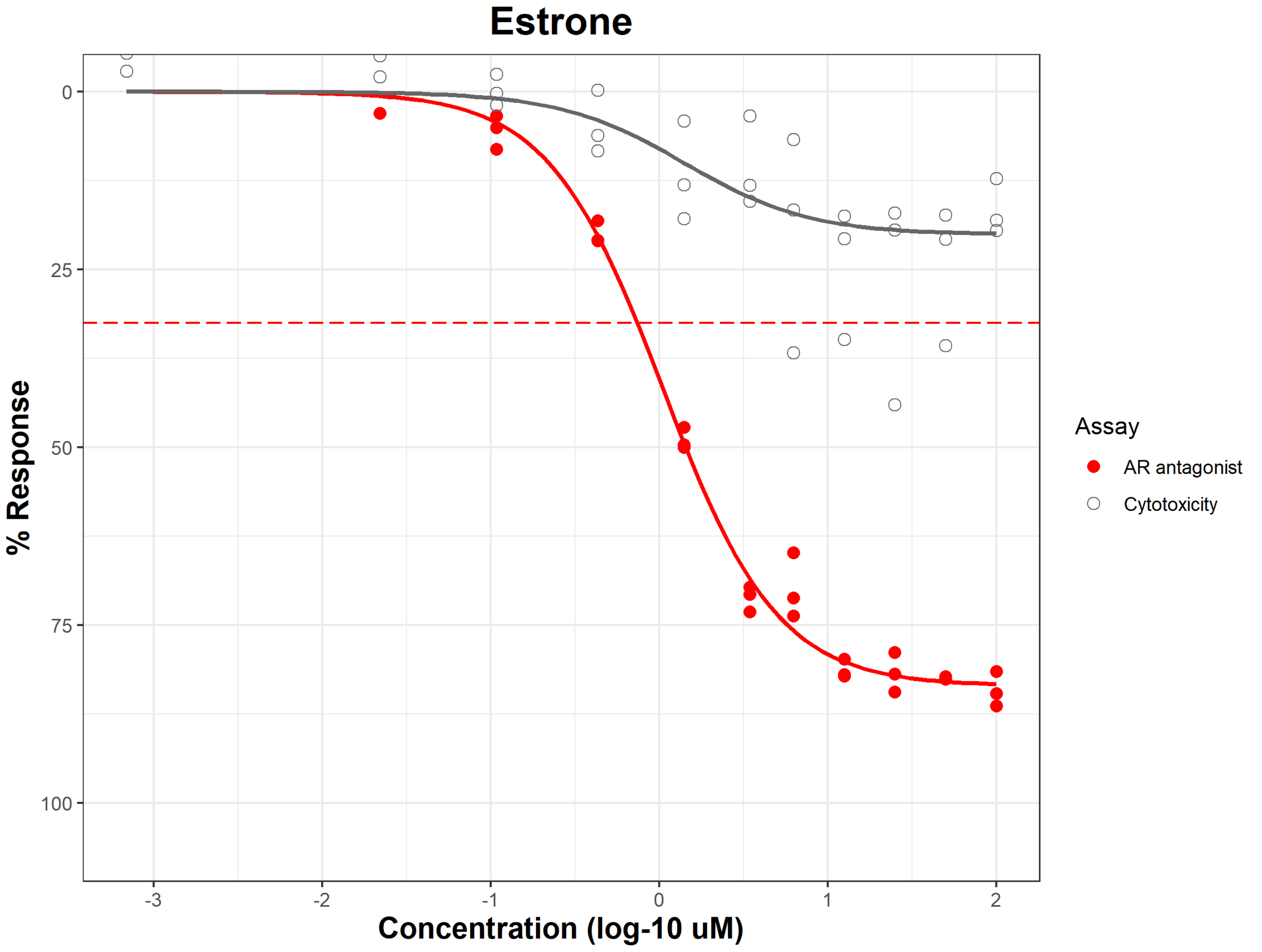
Estrone
% Response
0
25
50
75
100
-3
-2
-1
0
1
2
Concentration (log-10 uM)
Assay
AR antagonist
Cytotoxicity

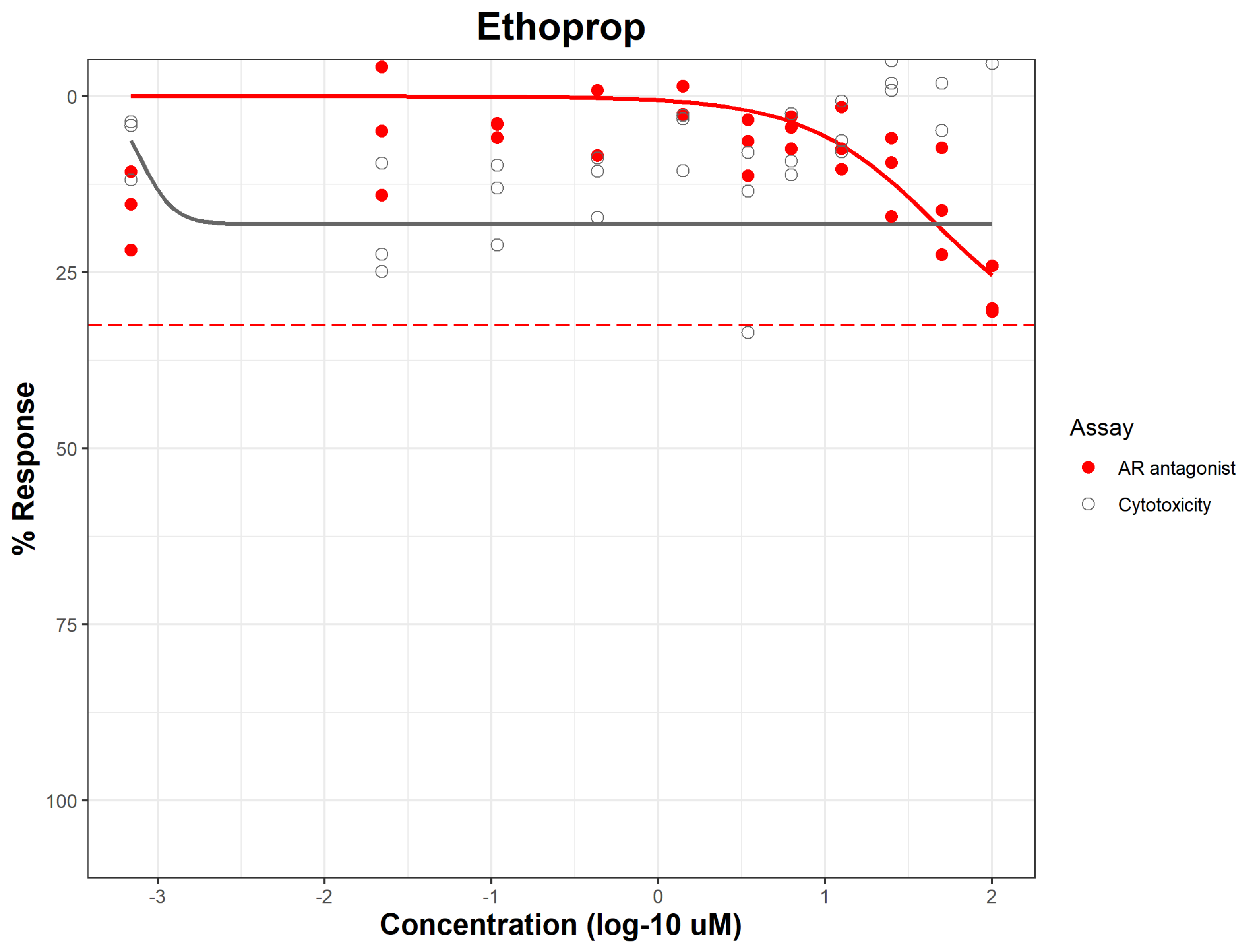
Ethoprop
% Response
0
25
50
75
100
-3
-2
-1
0
1
2
Concentration (log-10 uM)
Assay
AR antagonist
Cytotoxicity

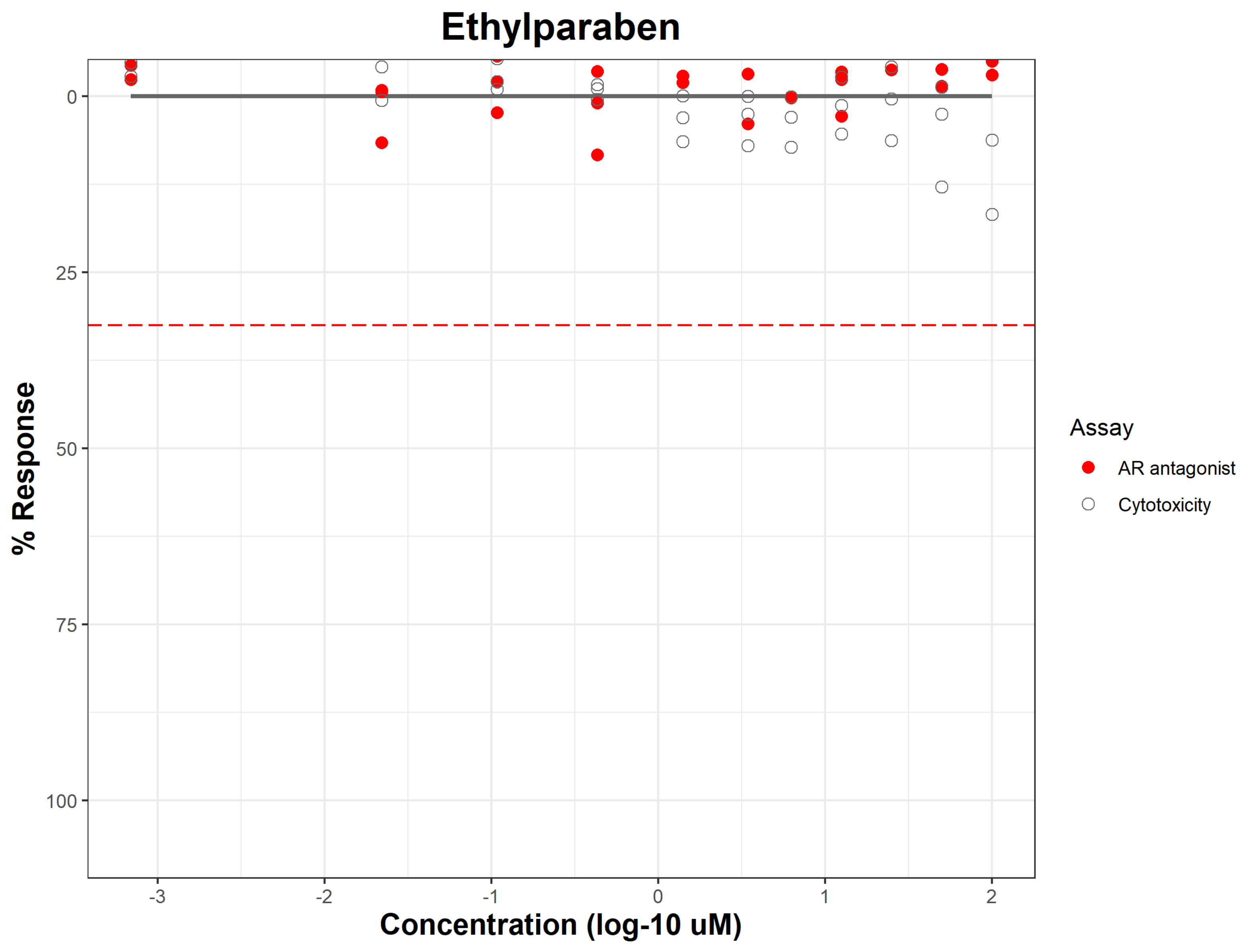

Ethylparaben
% Response
0
25
50
75
100
-3
-2
-1
0
1
2
Concentration (log-10 uM)
Assay
AR antagonist
Cytotoxicity

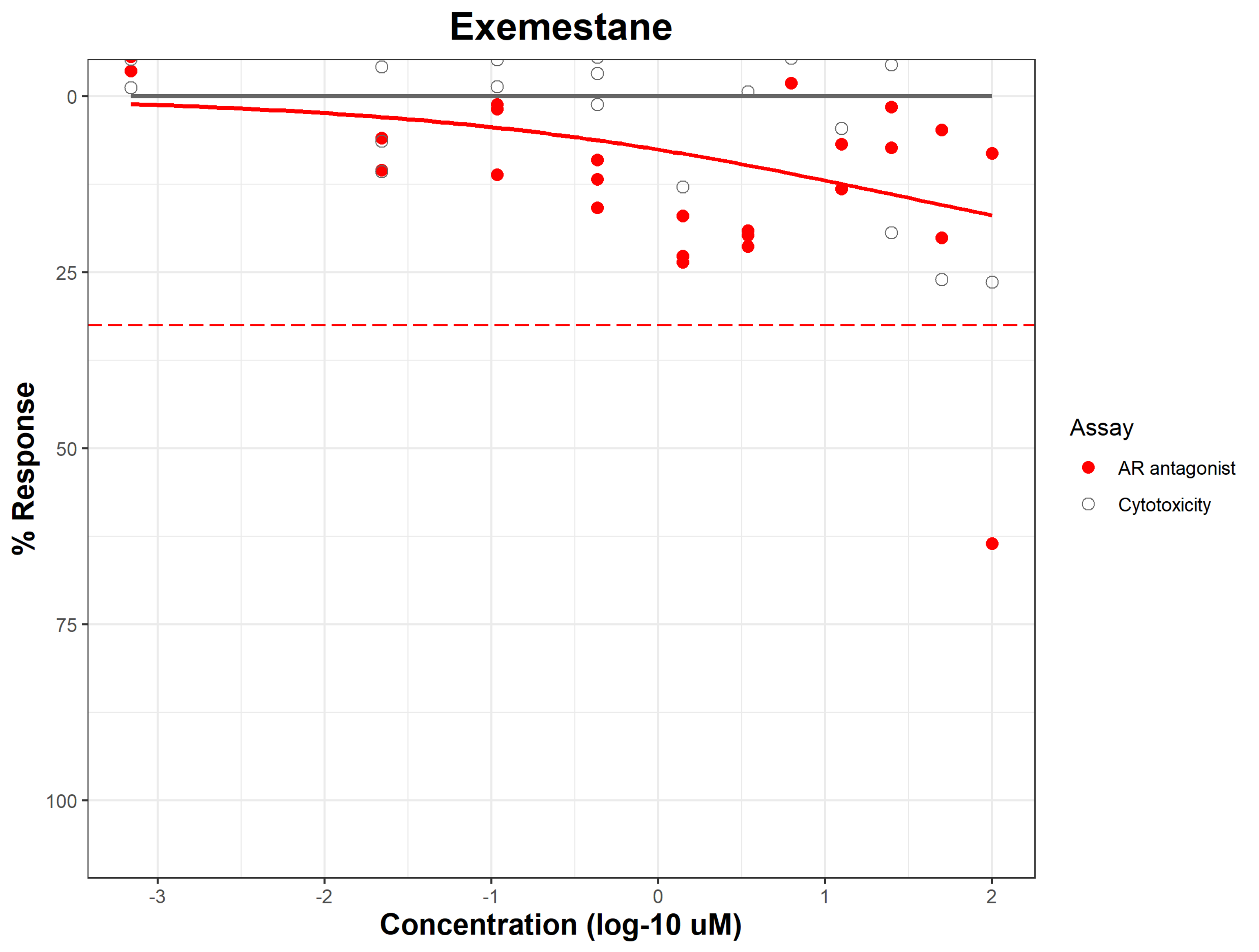

Exemestane
% Response
0
25
50
75
100
-3
-2
-1
0
1
2
Concentration (log-10 uM)
Assay
AR antagonist
Cytotoxicity

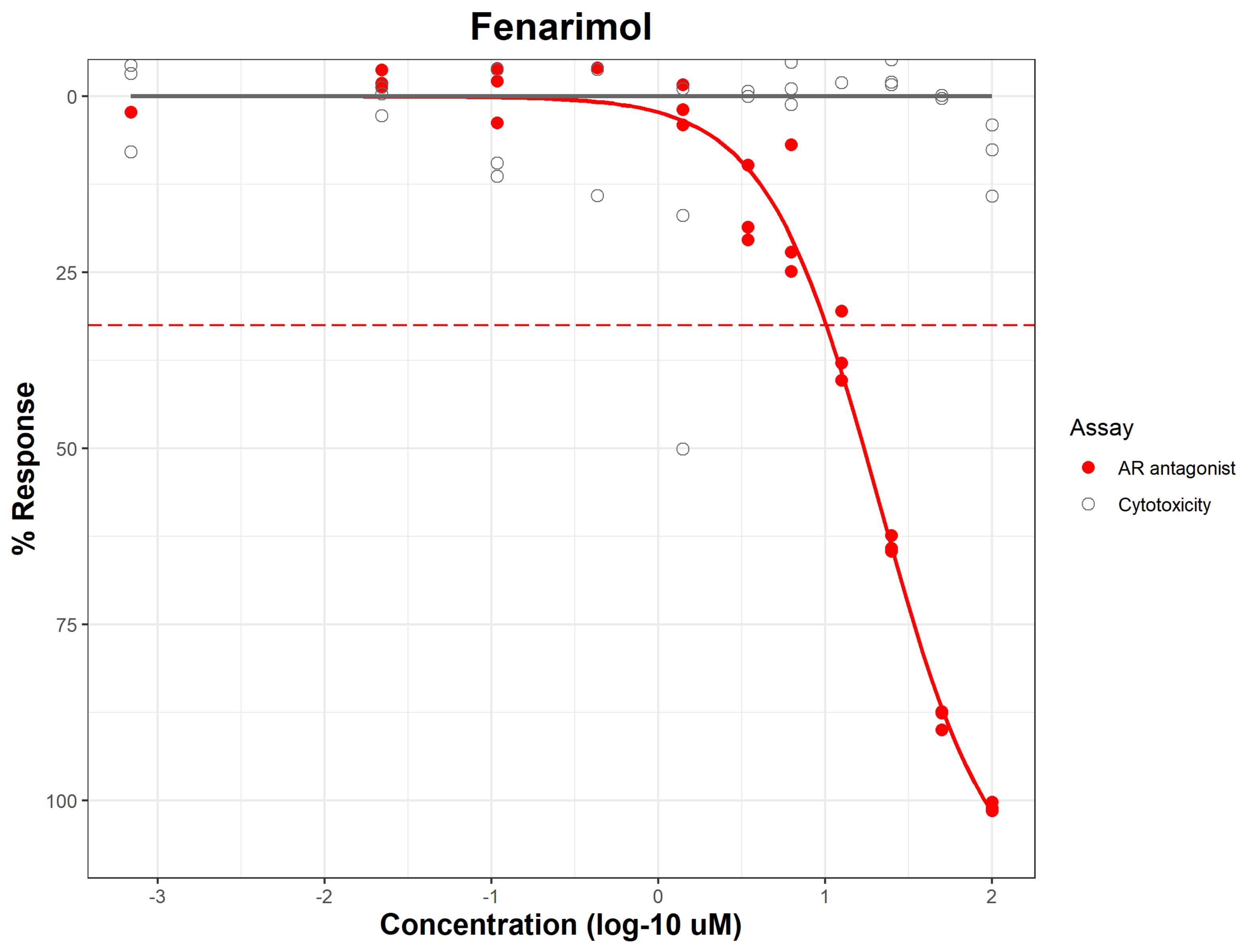
Fenarimol
% Response
0
25
50
75
100
-3
-2
-1
0
1
2
Concentration (log-10 uM)
Assay
AR antagonist
Cytotoxicity

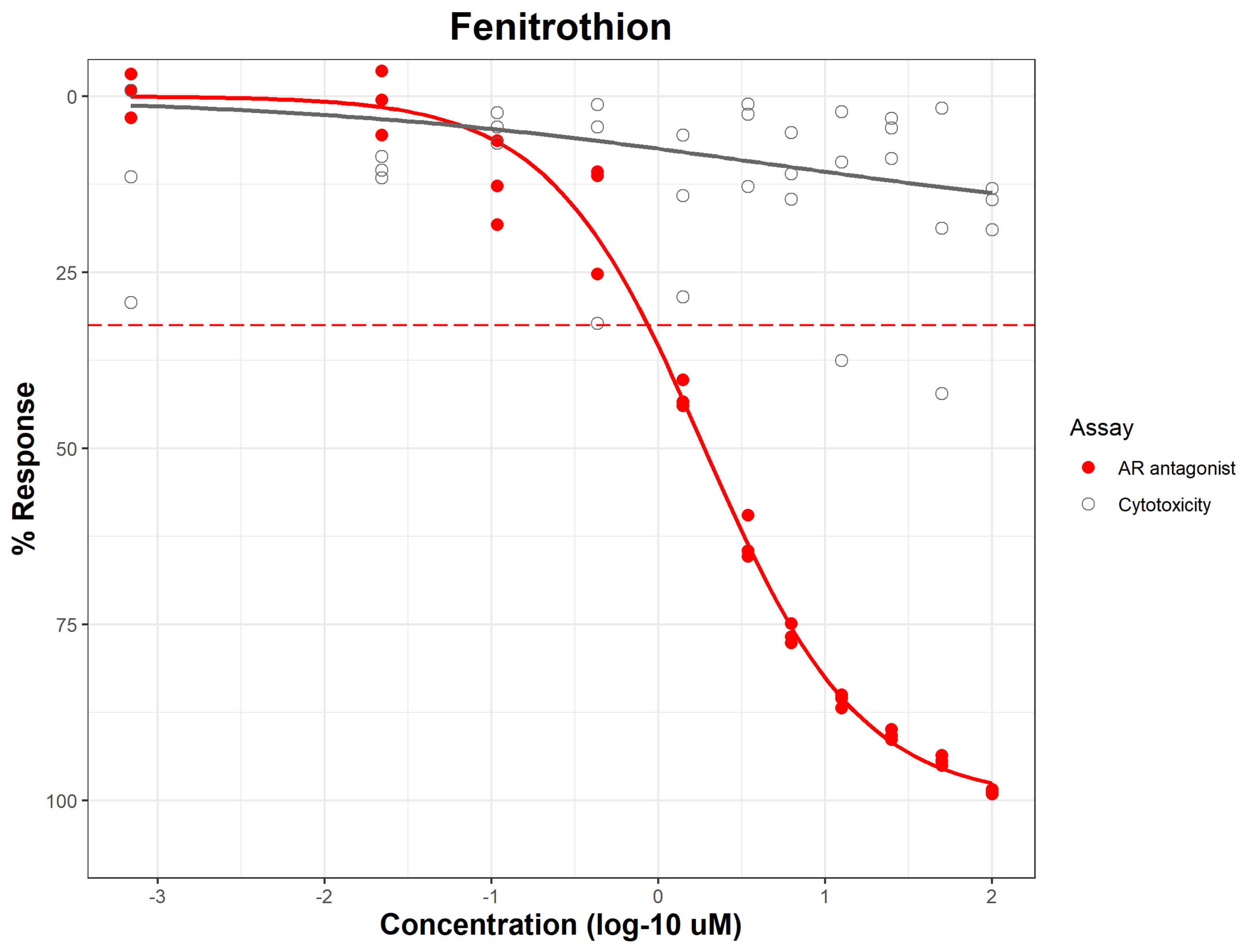

Fenitrothion
0
25
50
75
100
% Response
-3
-2
-1
0
1
2
Concentration (log-10 uM)
Assay
AR antagonist
Cytotoxicity

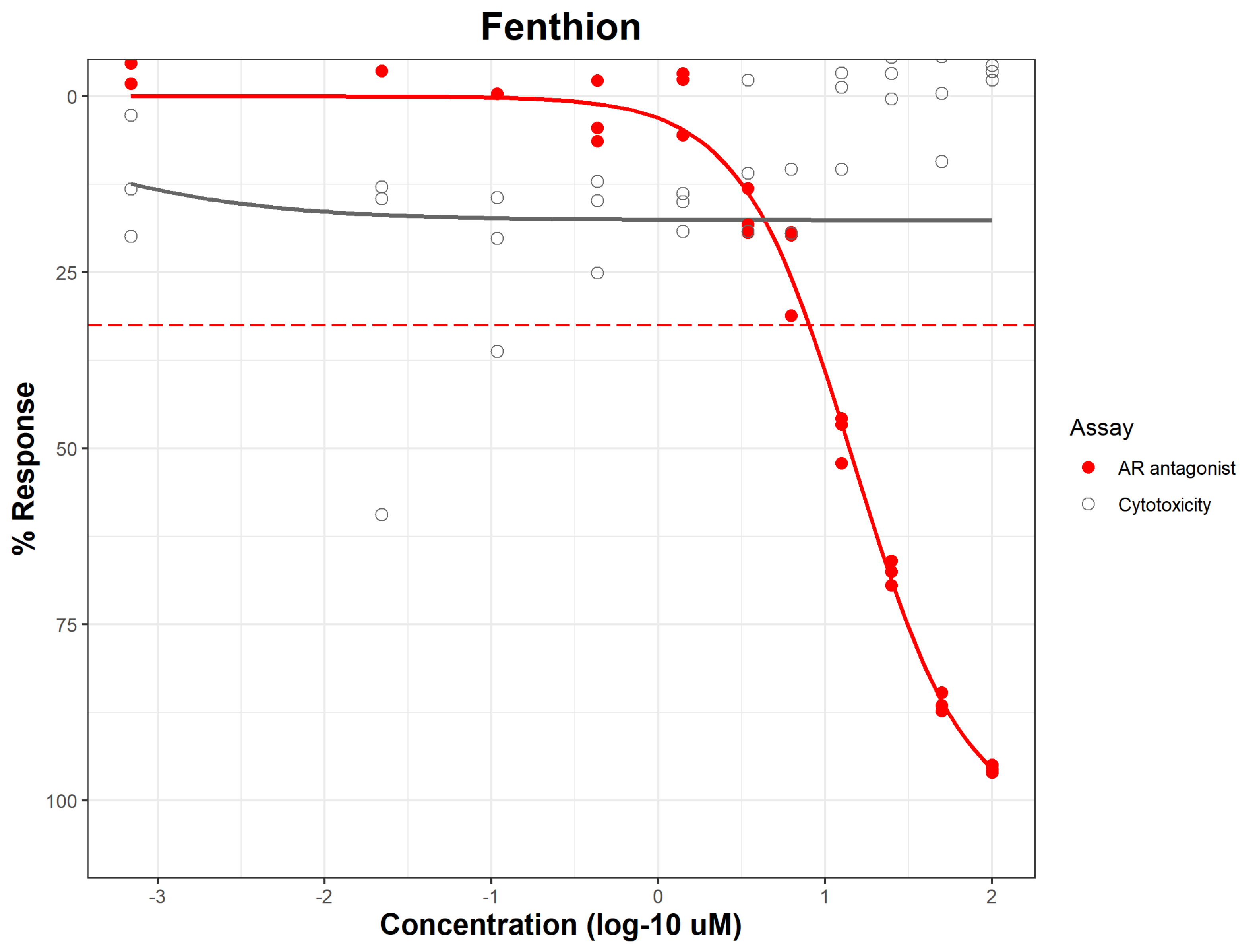

Fenthion
% Response
0
25
50
75
100
-3
-2
-1
0
1
2
Concentration (log-10 uM)
Assay
AR antagonist
Cytotoxicity

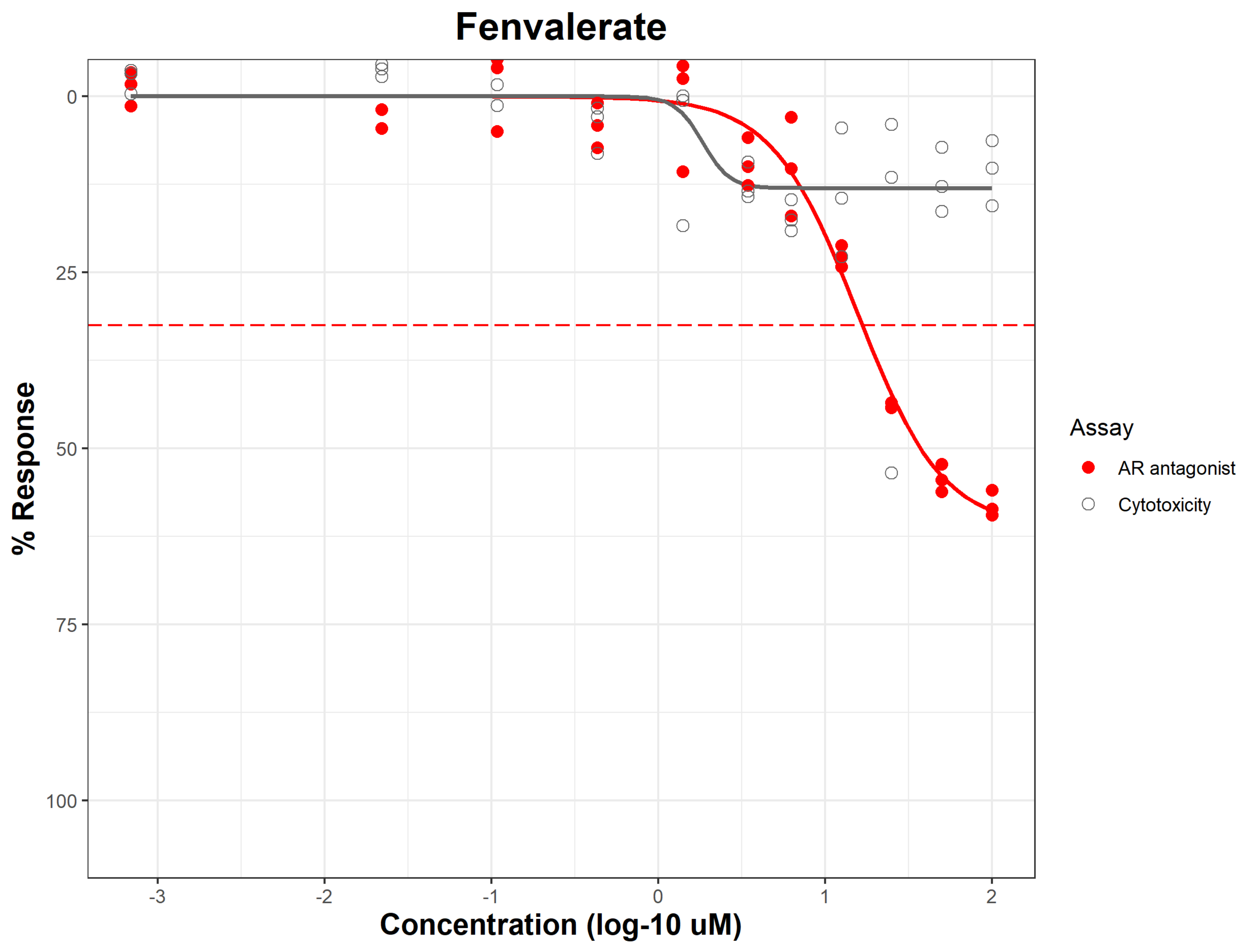

Fenvalerate
% Response
0
25
50
75
100
-3
-2
-1
0
1
2
Concentration (log-10 uM)
Assay
AR antagonist
Cytotoxicity

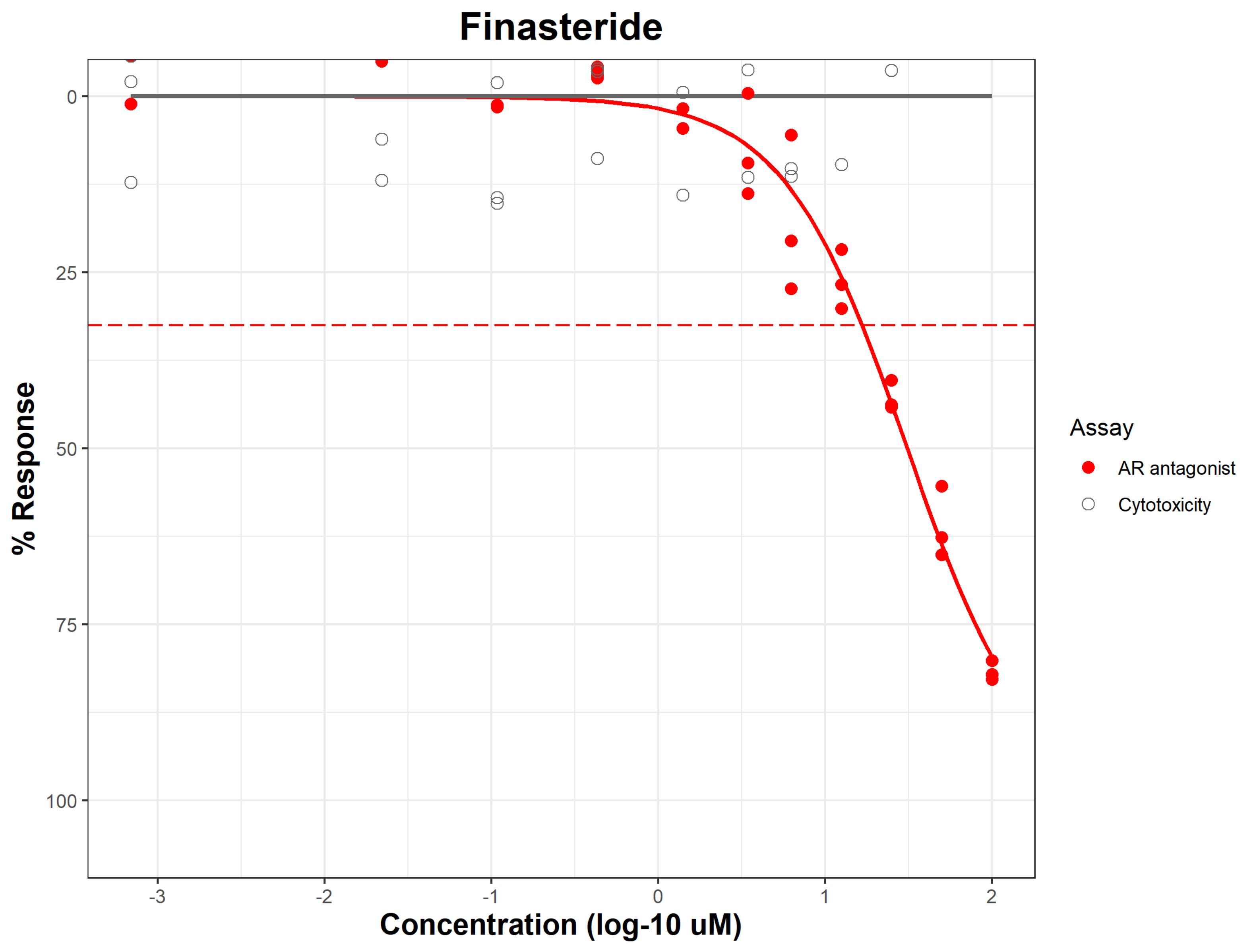
Finasteride
% Response
0
25
50
75
100
-3
-2
-1
0
1
2
Concentration (log-10 uM)
Assay
AR antagonist
Cytotoxicity

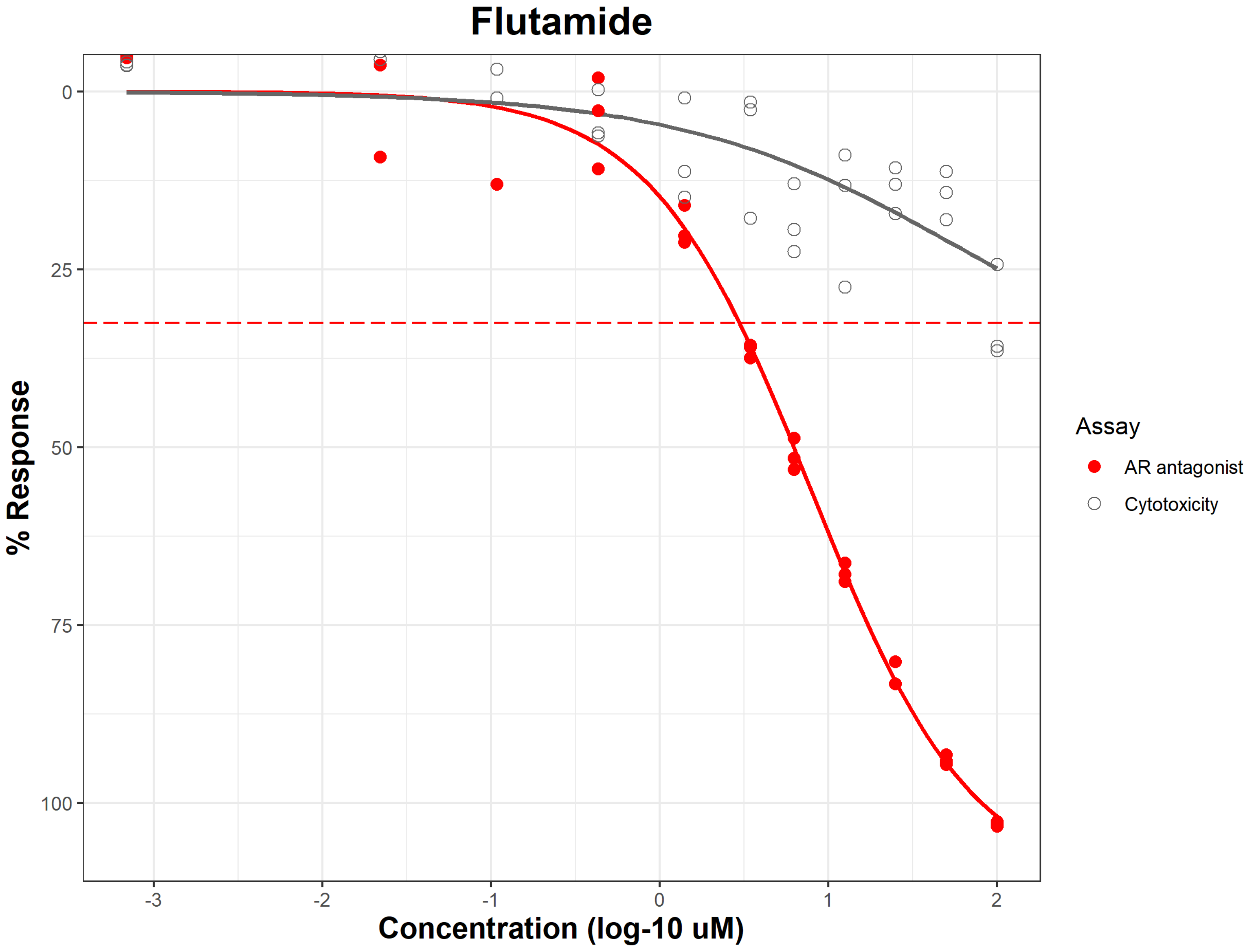
Flutamide
% Response
0
25
50
75
100
-3
-2
-1
0
1
2
Concentration (log-10 uM)
Assay
AR antagonist
Cytotoxicity

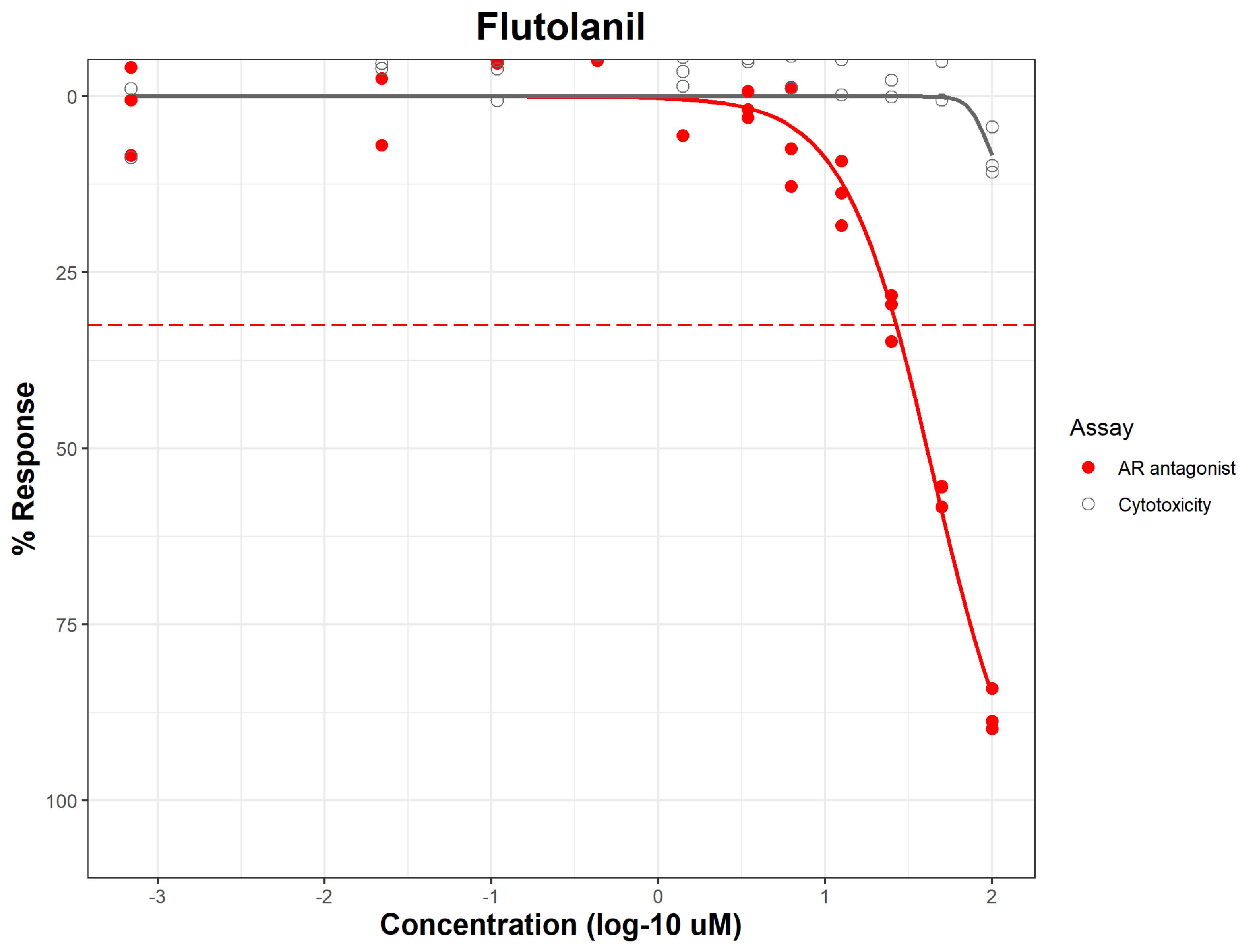

Flutolanil
% Response
0
25
50
75
100
-3
-2
-1
0
1
2
Concentration (log-10 uM)
Assay
AR antagonist
Cytotoxicity

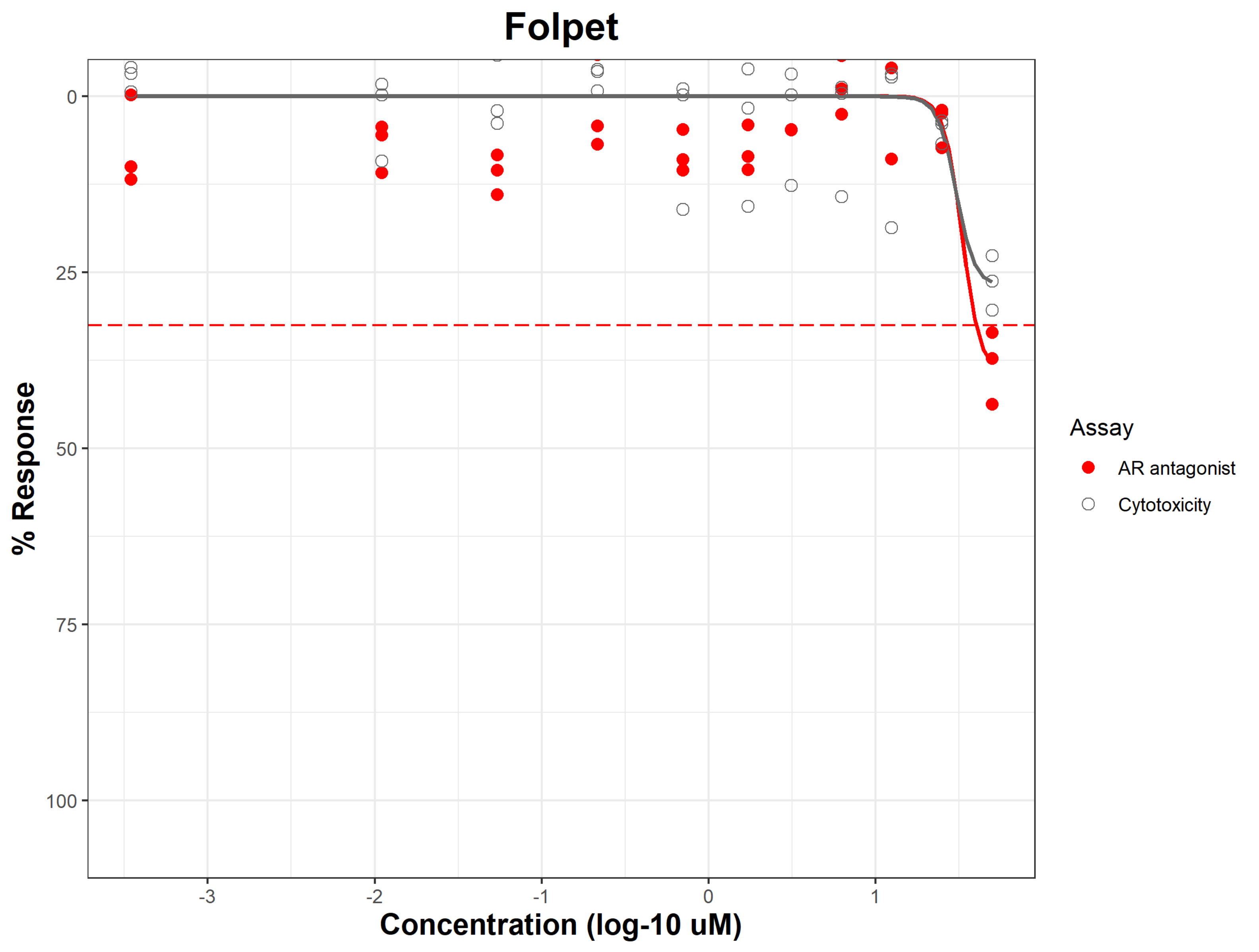

Folpet
% Response
0
25
50
75
100
-3
-2
-1
0
1
Concentration (log-10 uM)
Assay
AR antagonist
Cytotoxicity

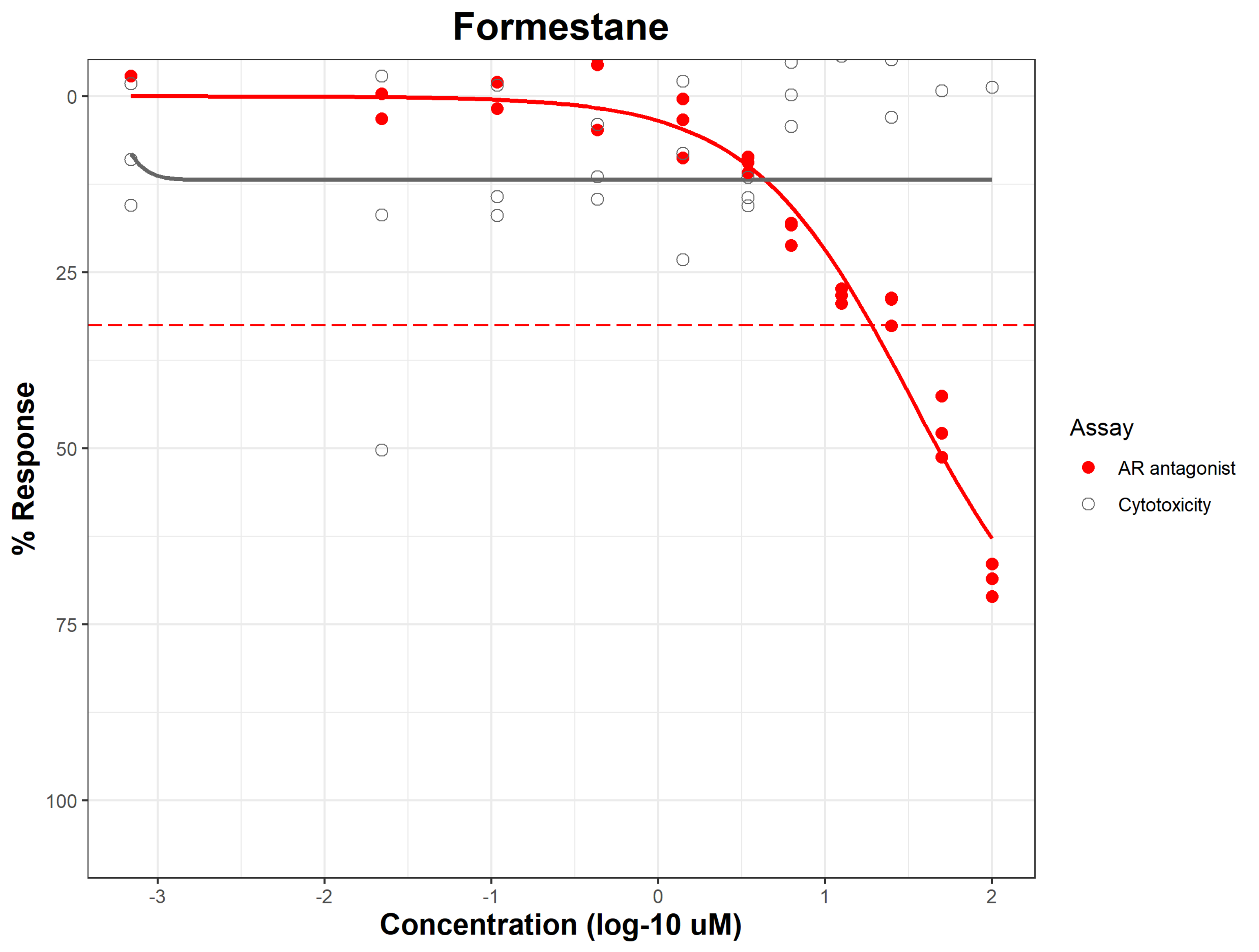

Formestane
% Response
0
25
50
75
100
-3
-2
-1
0
1
2
Concentration (log-10 uM)
Assay
AR antagonist
Cytotoxicity

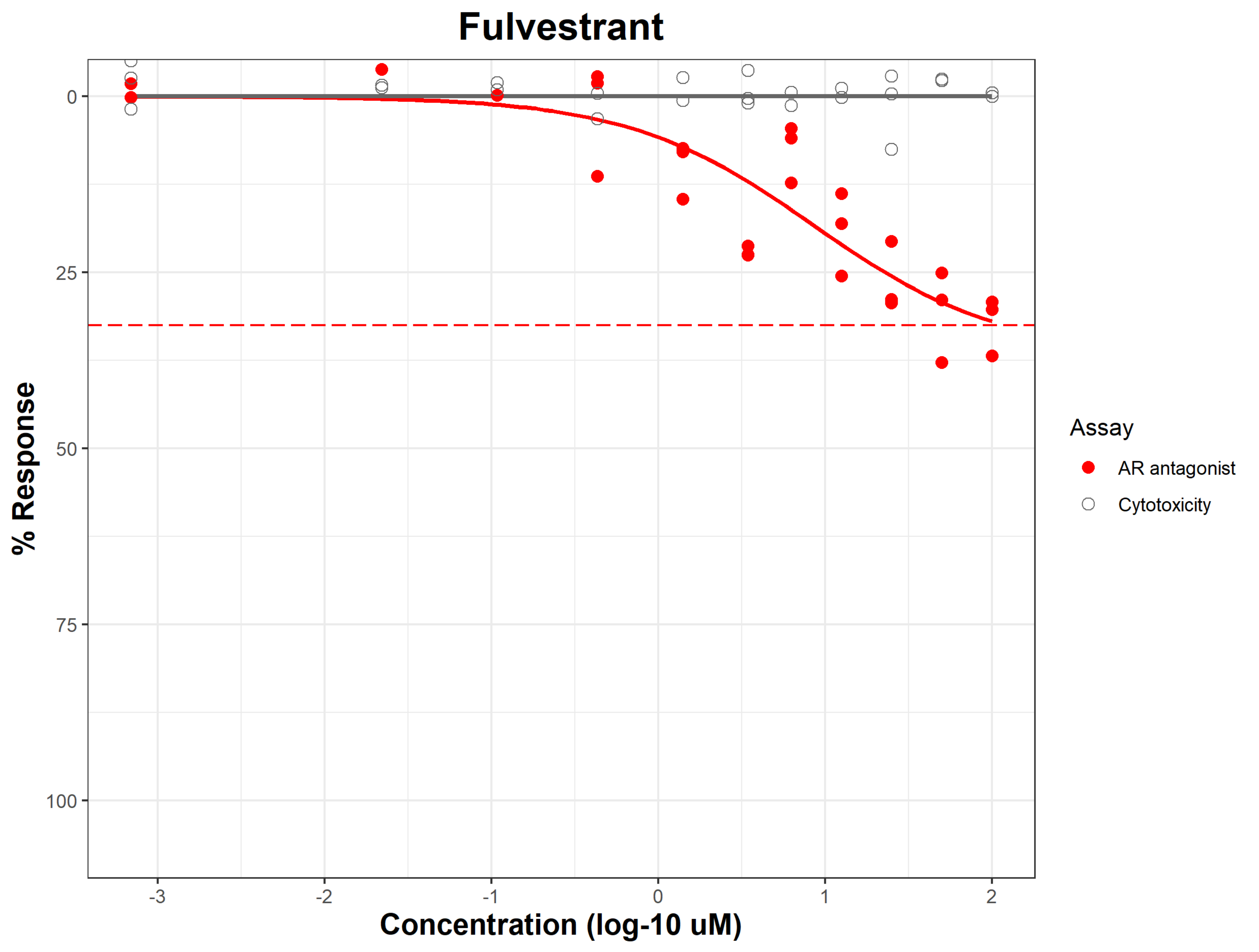
Fulvestrant
% Response
0
25
50
75
100
-3
-2
-1
0
1
2
Concentration (log-10 uM)
Assay
AR antagonist
Cytotoxicity

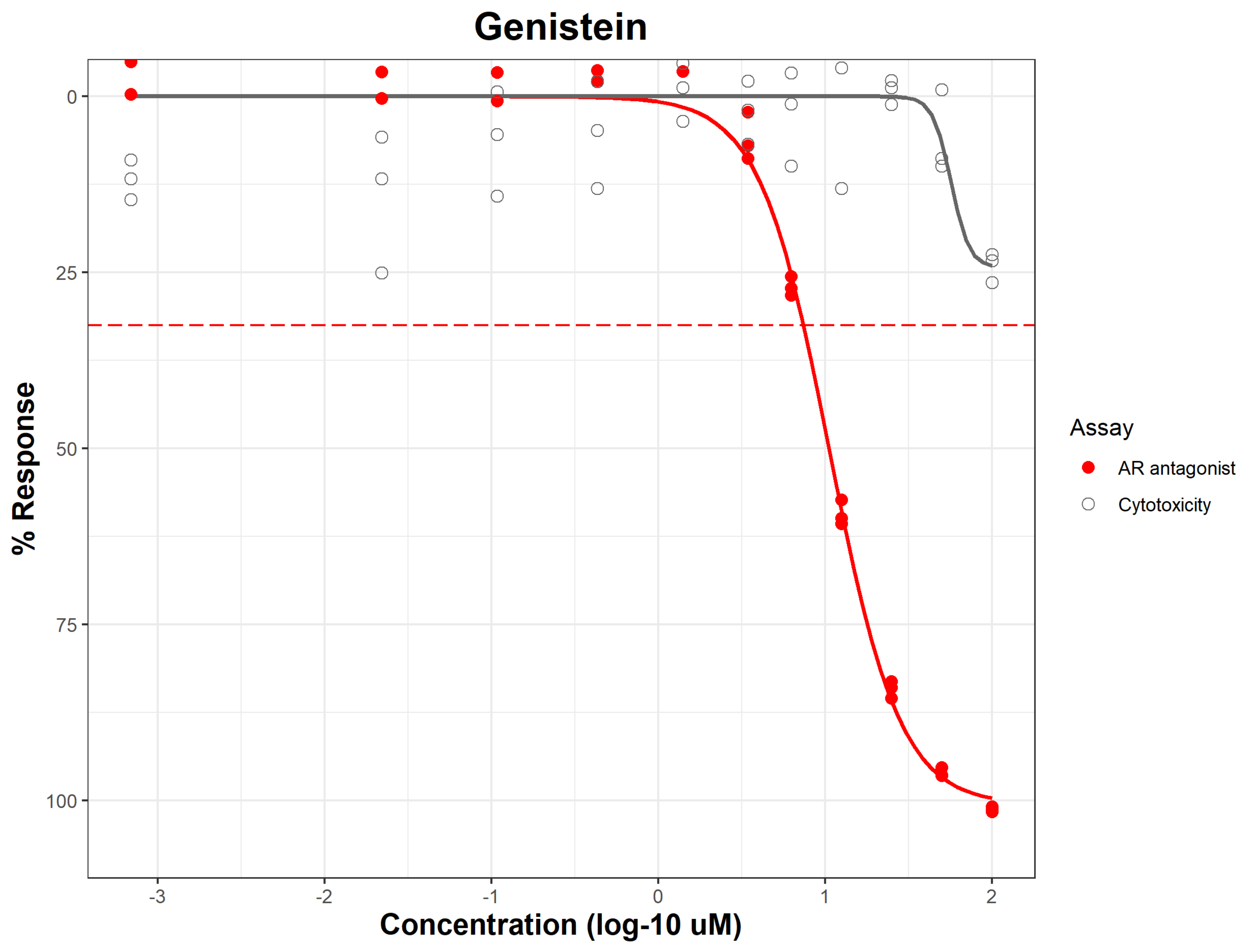

Genistein
% Response
0
25
50
75
100
-3
-2
-1
0
1
2
Concentration (log-10 uM)
Assay
AR antagonist
Cytotoxicity

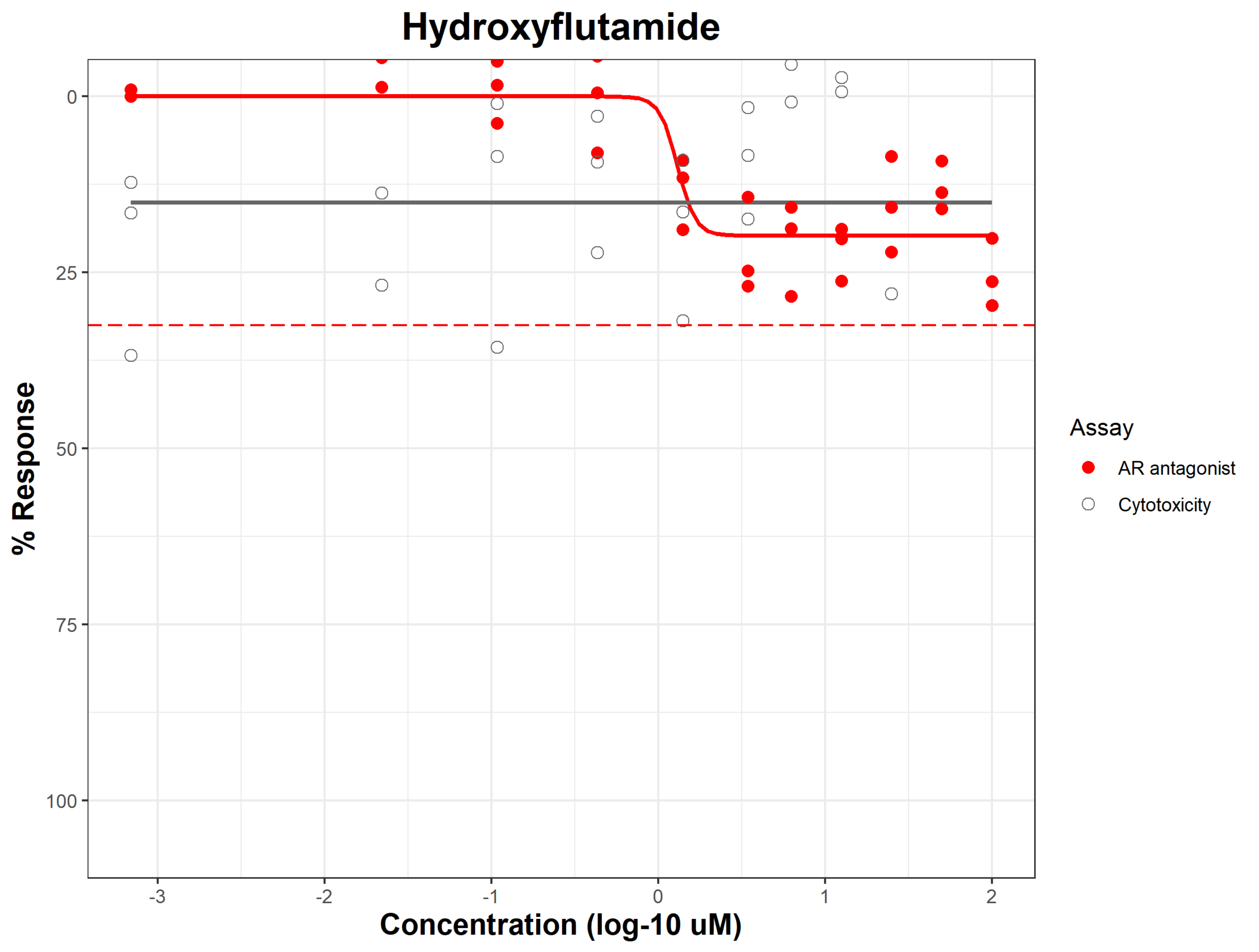
Hydroxyflutamide
% Response
0
25
50
75
100
-3
-2
-1
0
1
2
Concentration (log-10 uM)
Assay
AR antagonist
Cytotoxicity

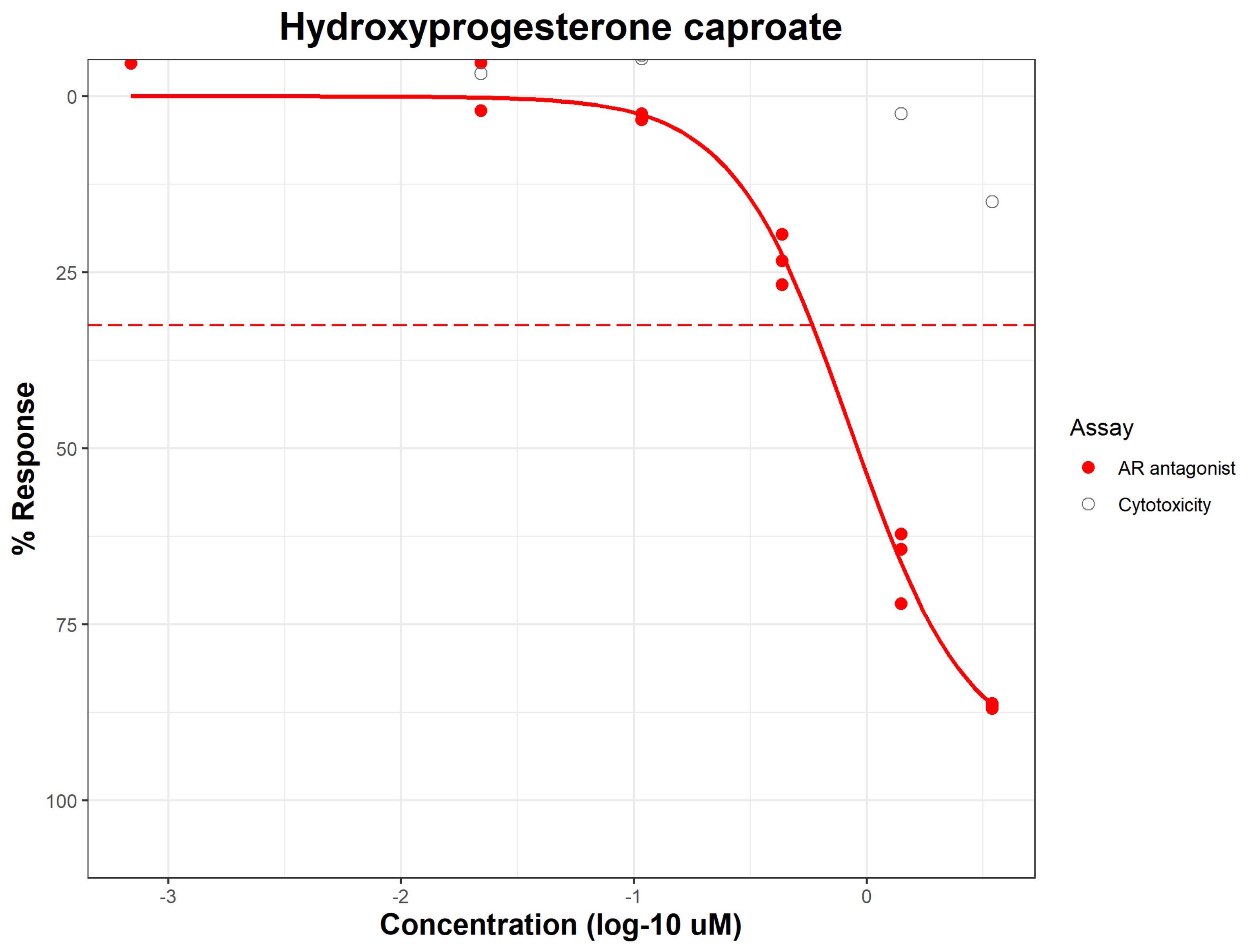

Hydroxyprogesterone caproate
% Response
0
25
50
75
100
-3
-2
-1
0
Concentration (log-10 uM)
Assay
AR antagonist
Cytotoxicity

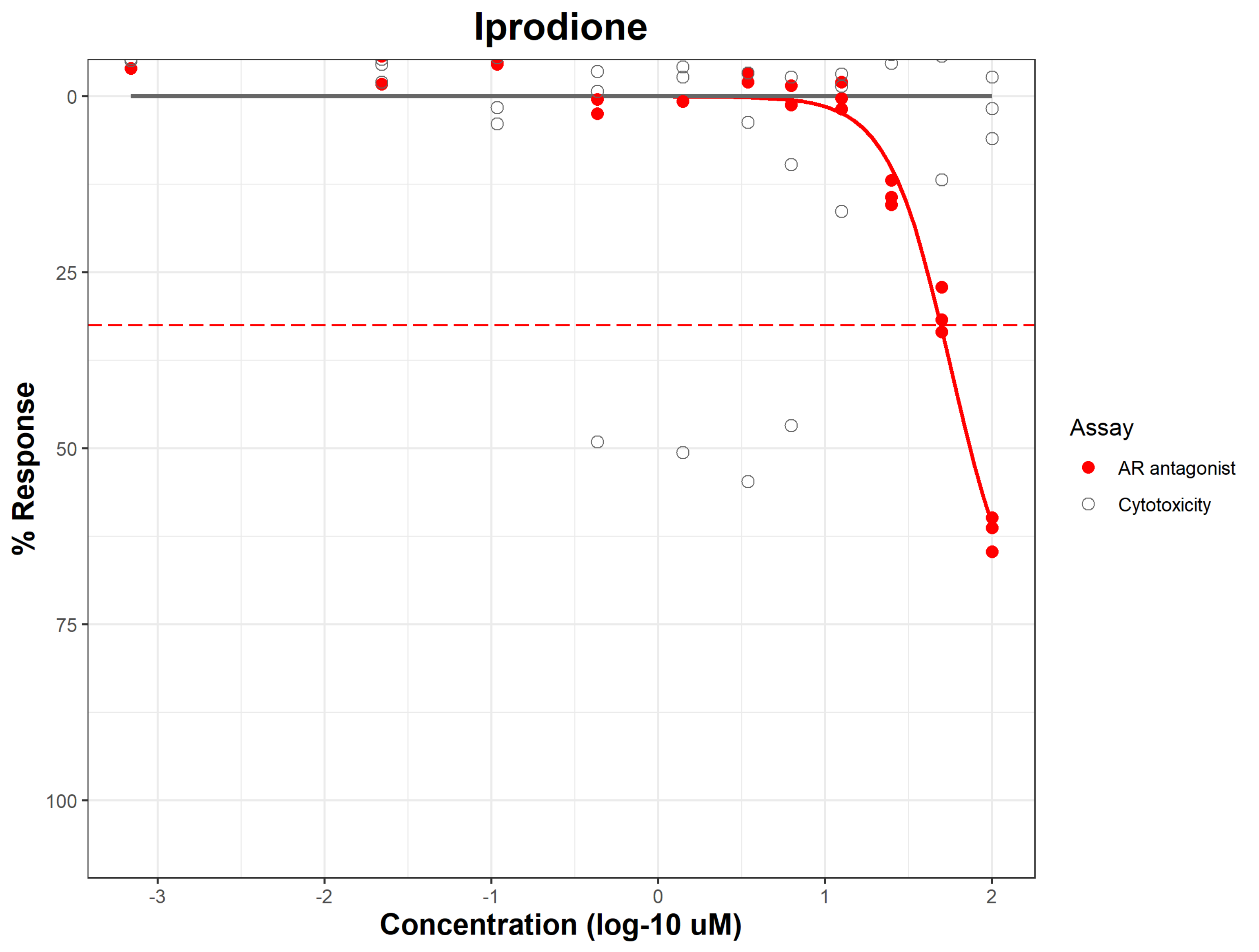

Iprodione
% Response
0
25
50
75
100
-3
-2
-1
0
1
2
Concentration (log-10 uM)
Assay
AR antagonist
Cytotoxicity

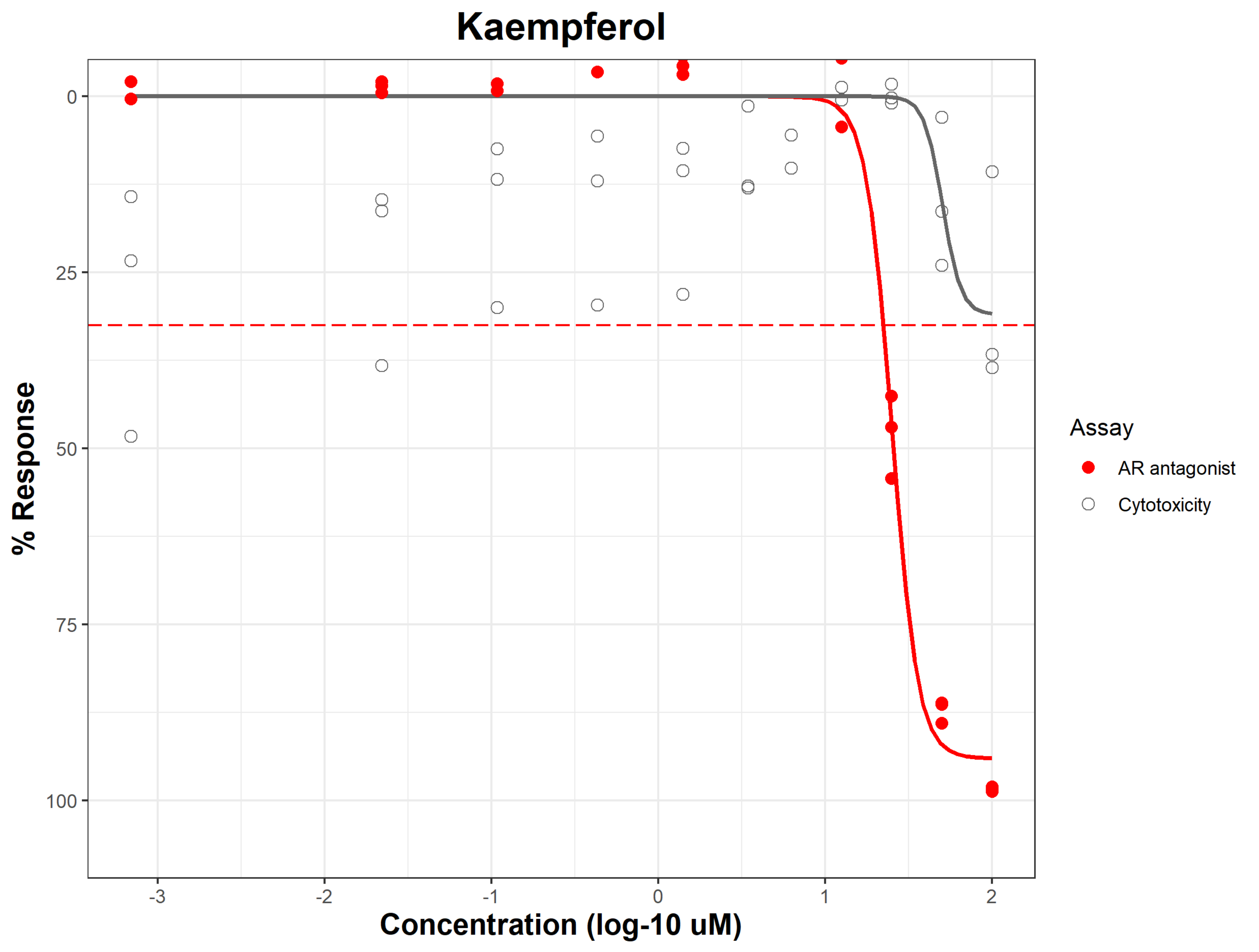

Kaempferol
% Response
0
25
50
75
100
-3
-2
-1
0
1
2
Concentration (log-10 uM)
Assay
AR antagonist
Cytotoxicity

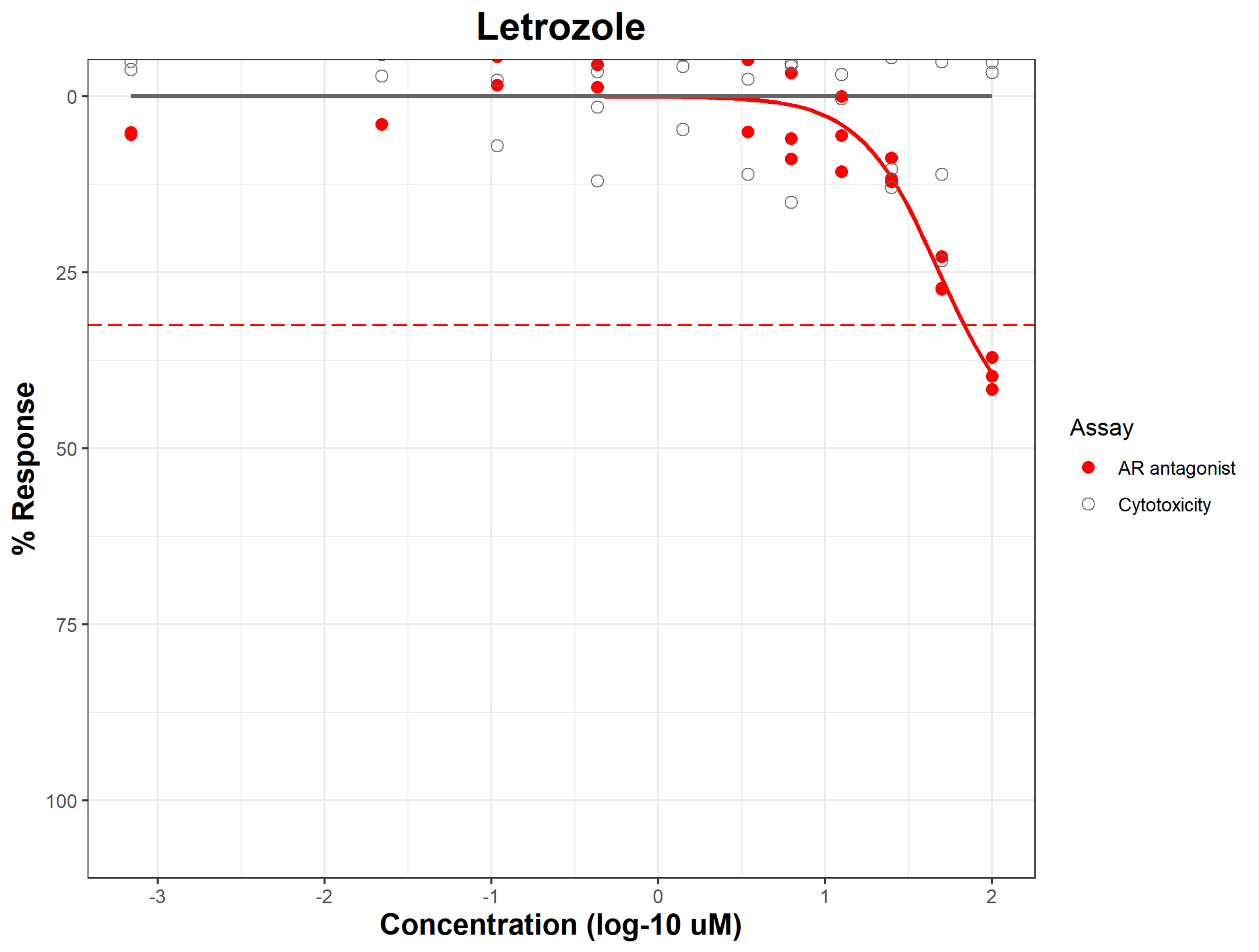

Letrozole
% Response
0
25
50
75
100
-3
-2
-1
0
1
2
Concentration (log-10 uM)
Assay
AR antagonist
Cytotoxicity

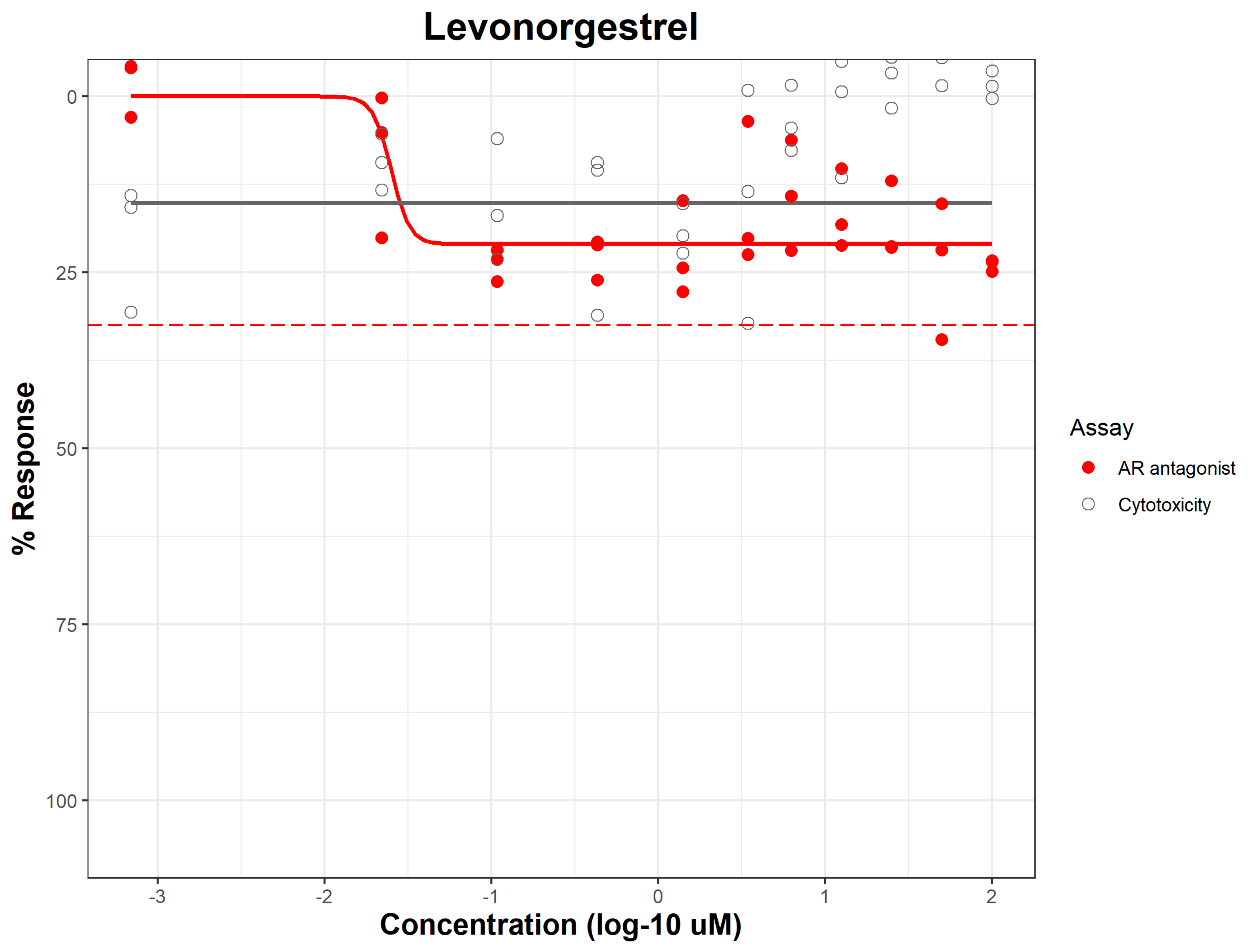
Levonorgestrel
% Response
0
25
50
75
100
-3
-2
-1
0
1
2
Concentration (log-10 uM)
Assay
AR antagonist
Cytotoxicity

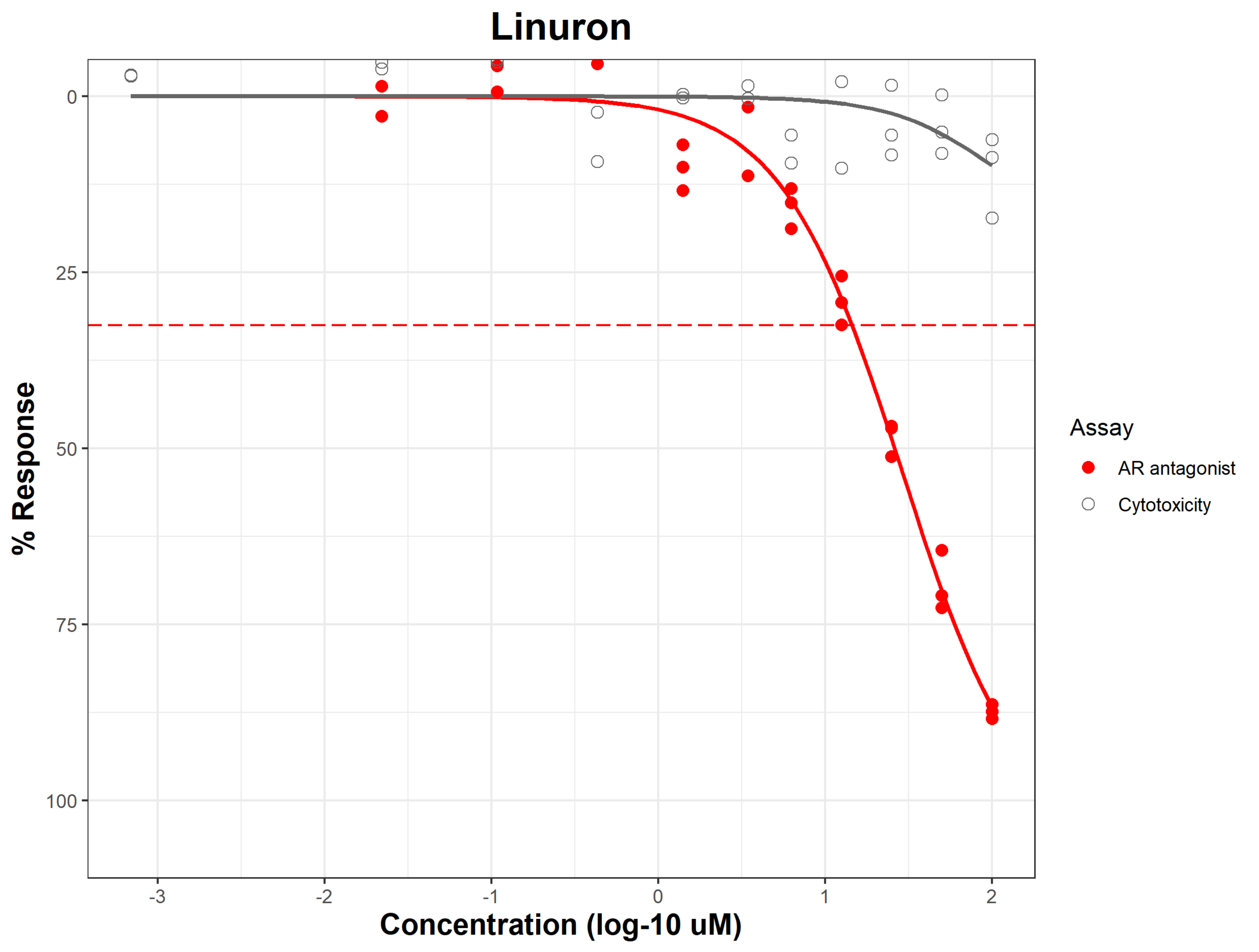

Linuron
% Response
0
25
50
75
100
-3
-2
-1
0
1
2
Concentration (log-10 uM)
Assay
AR antagonist
Cytotoxicity

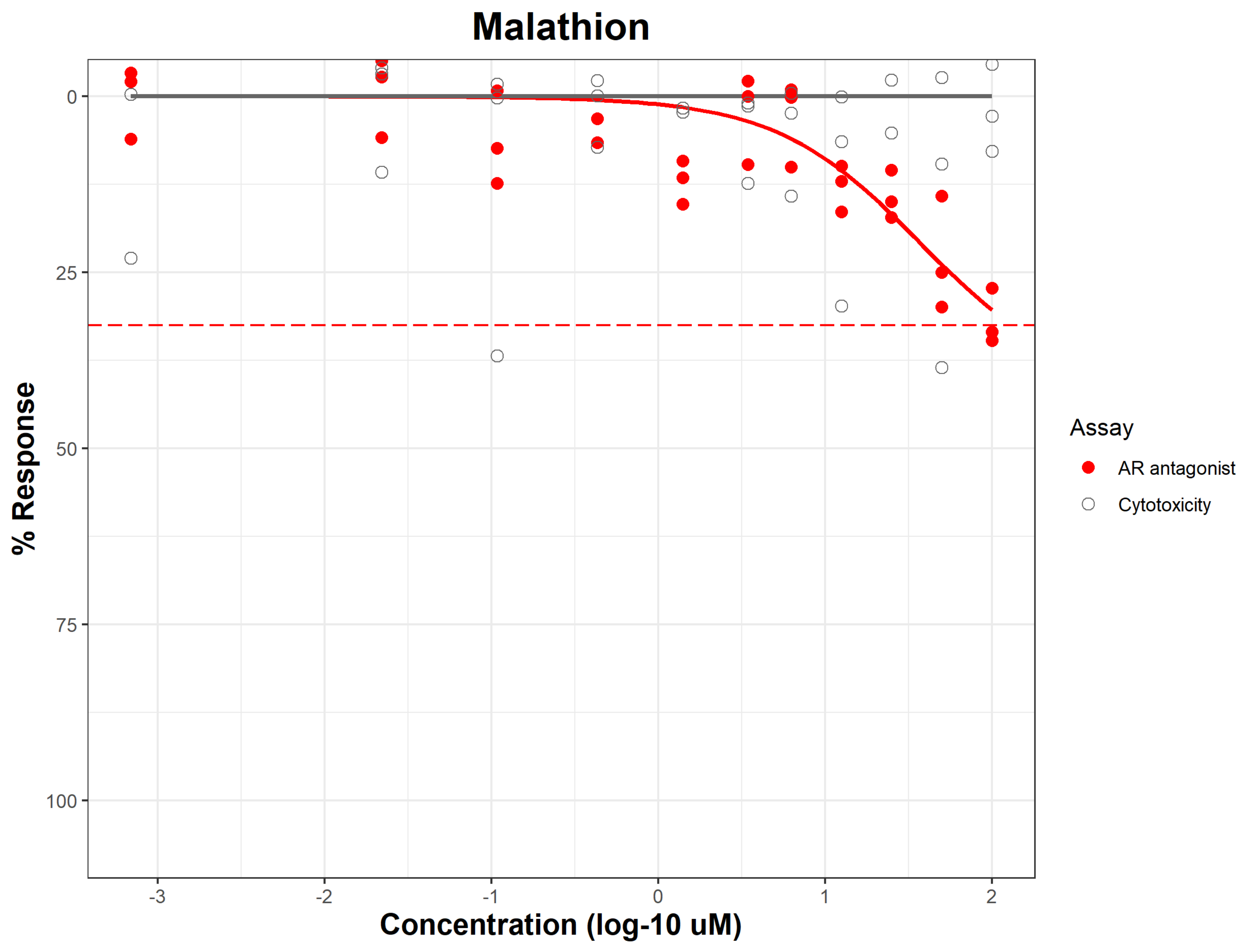

Malathion
% Response
0
25
50
75
100
-3
-2
-1
0
1
2
Concentration (log-10 uM)
Assay
AR antagonist
Cytotoxicity

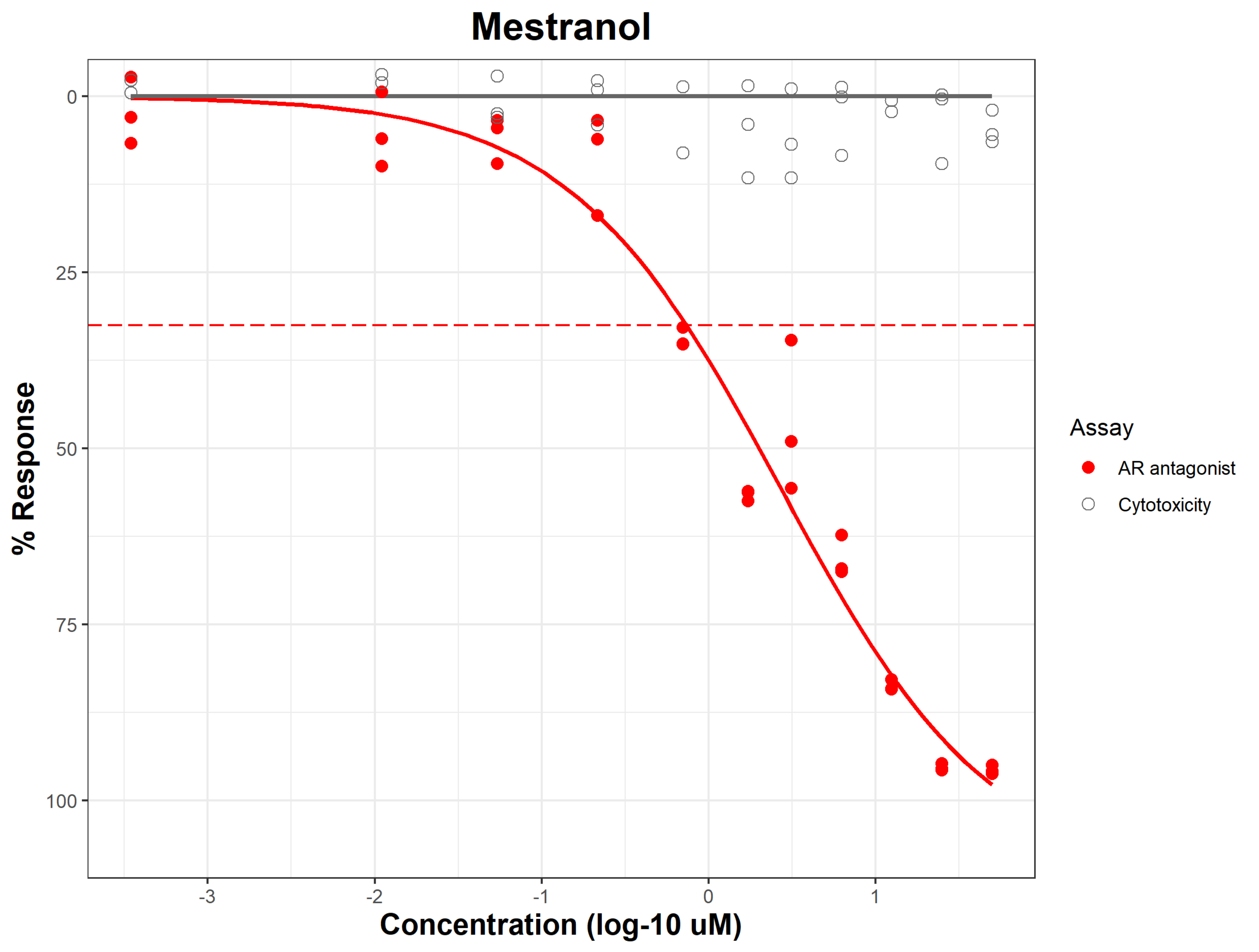

Mestranol
% Response
0
25
50
75
100
-3
-2
-1
0
1
Concentration (log-10 uM)
Assay
AR antagonist
Cytotoxicity

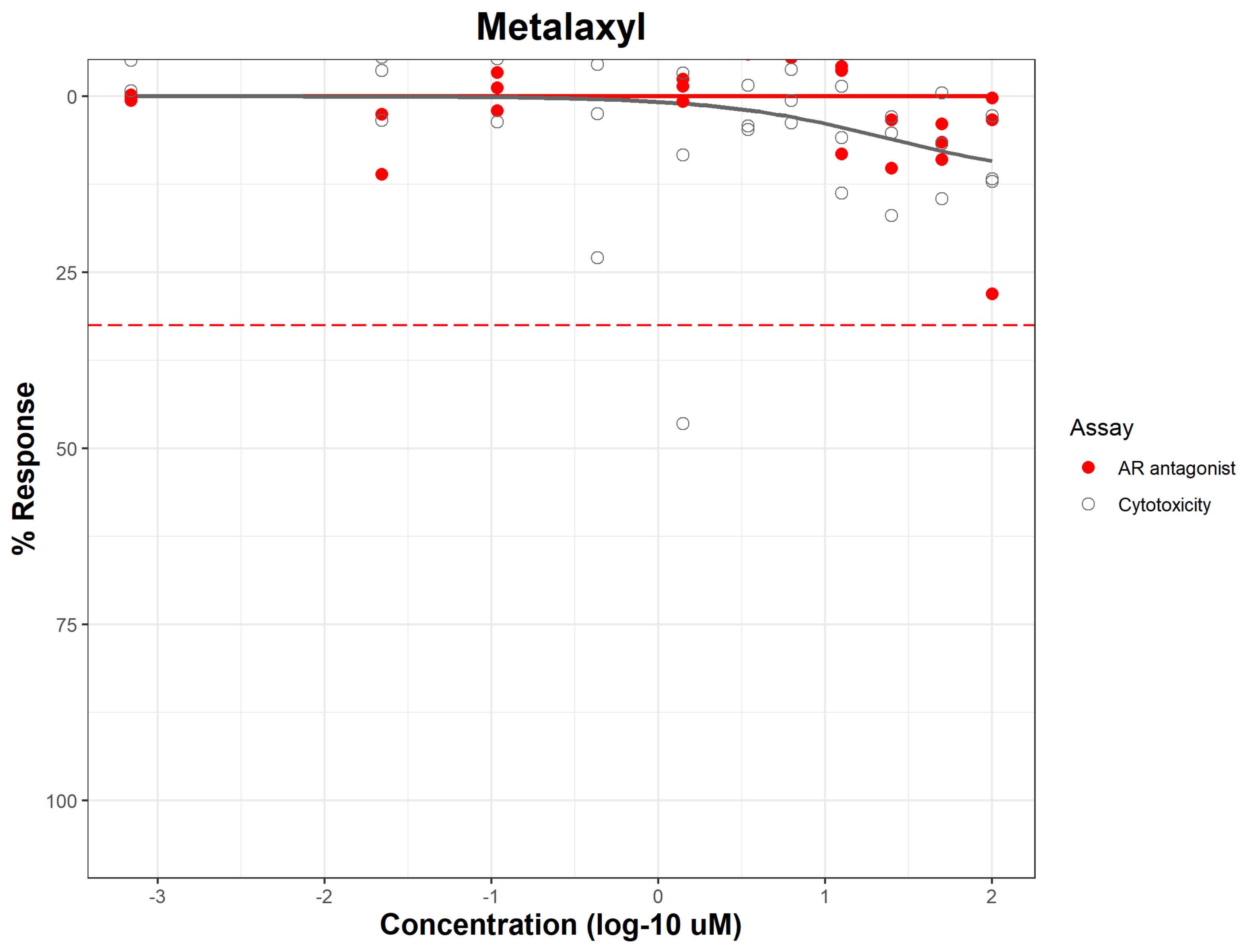

Metalaxyl
% Response
0
25
50
75
100
-3
-2
-1
0
1
2
Concentration (log-10 uM)
Assay
AR antagonist
Cytotoxicity

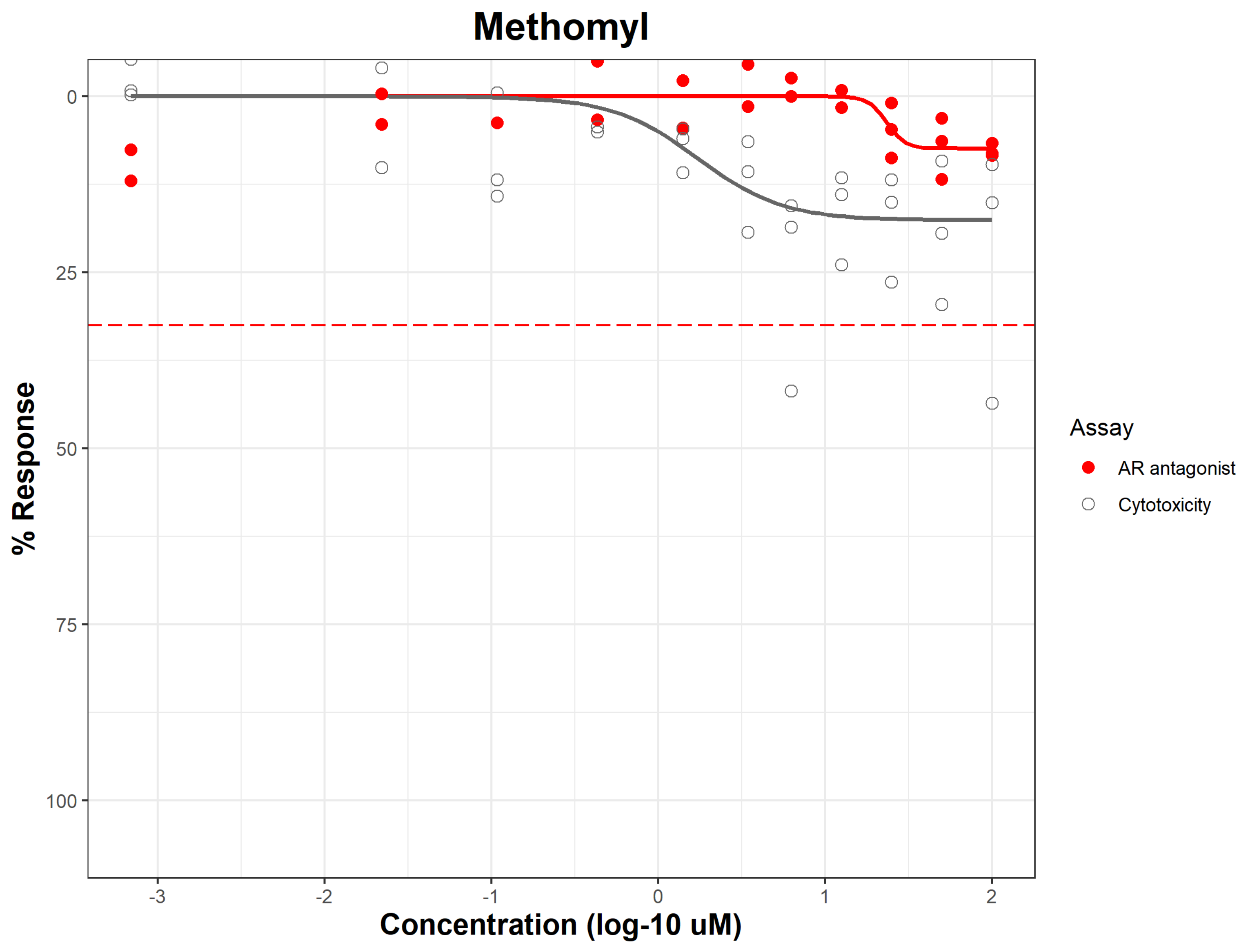

Methomyl
% Response
0
25
50
75
100
-3
-2
-1
0
1
2
Concentration (log-10 uM)
Assay
AR antagonist
Cytotoxicity

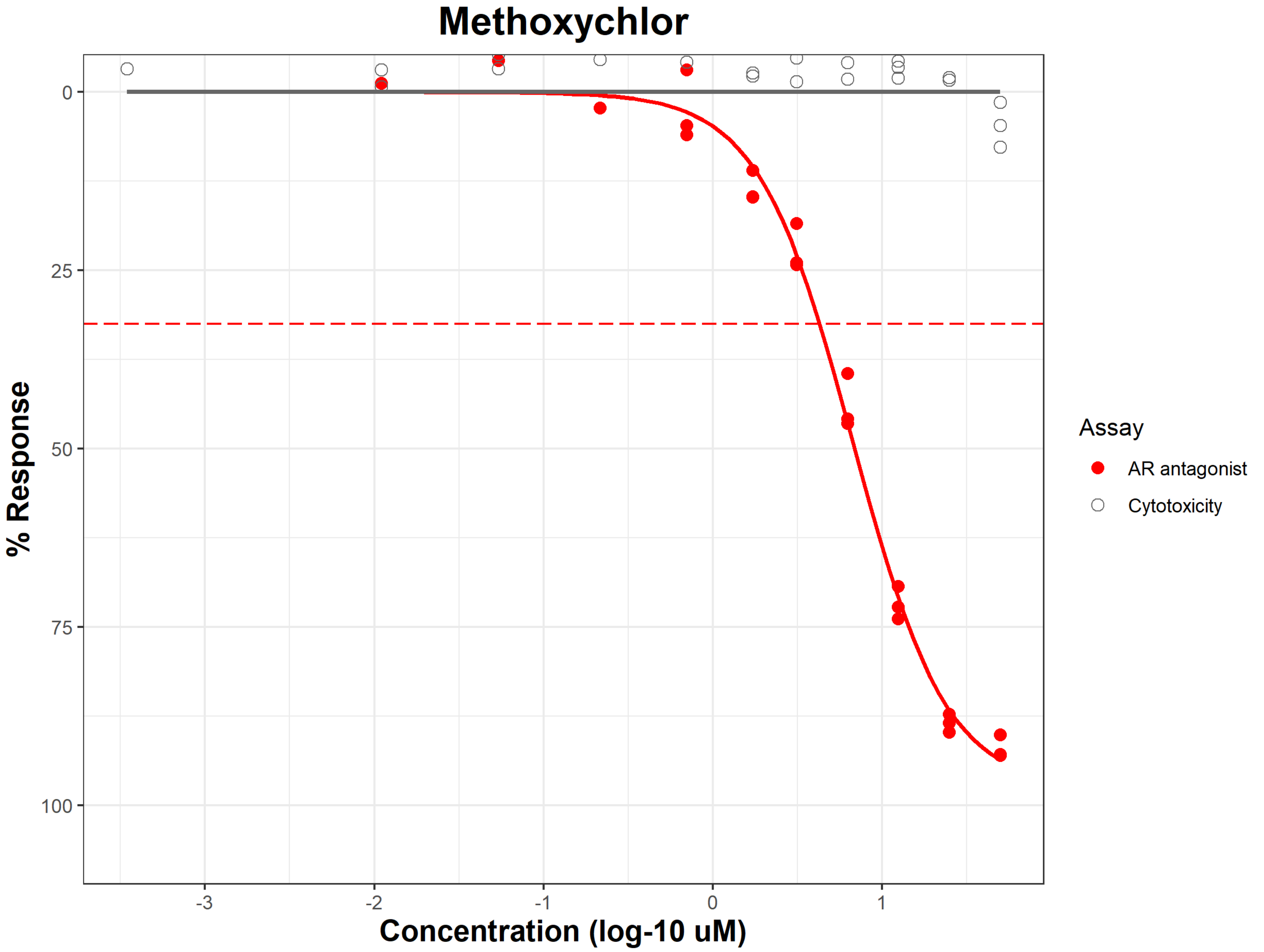

Methoxychlor
% Response
0
25
50
75
100
-3
-2
-1
0
1
Concentration (log-10 uM)
Assay
AR antagonist
Cytotoxicity

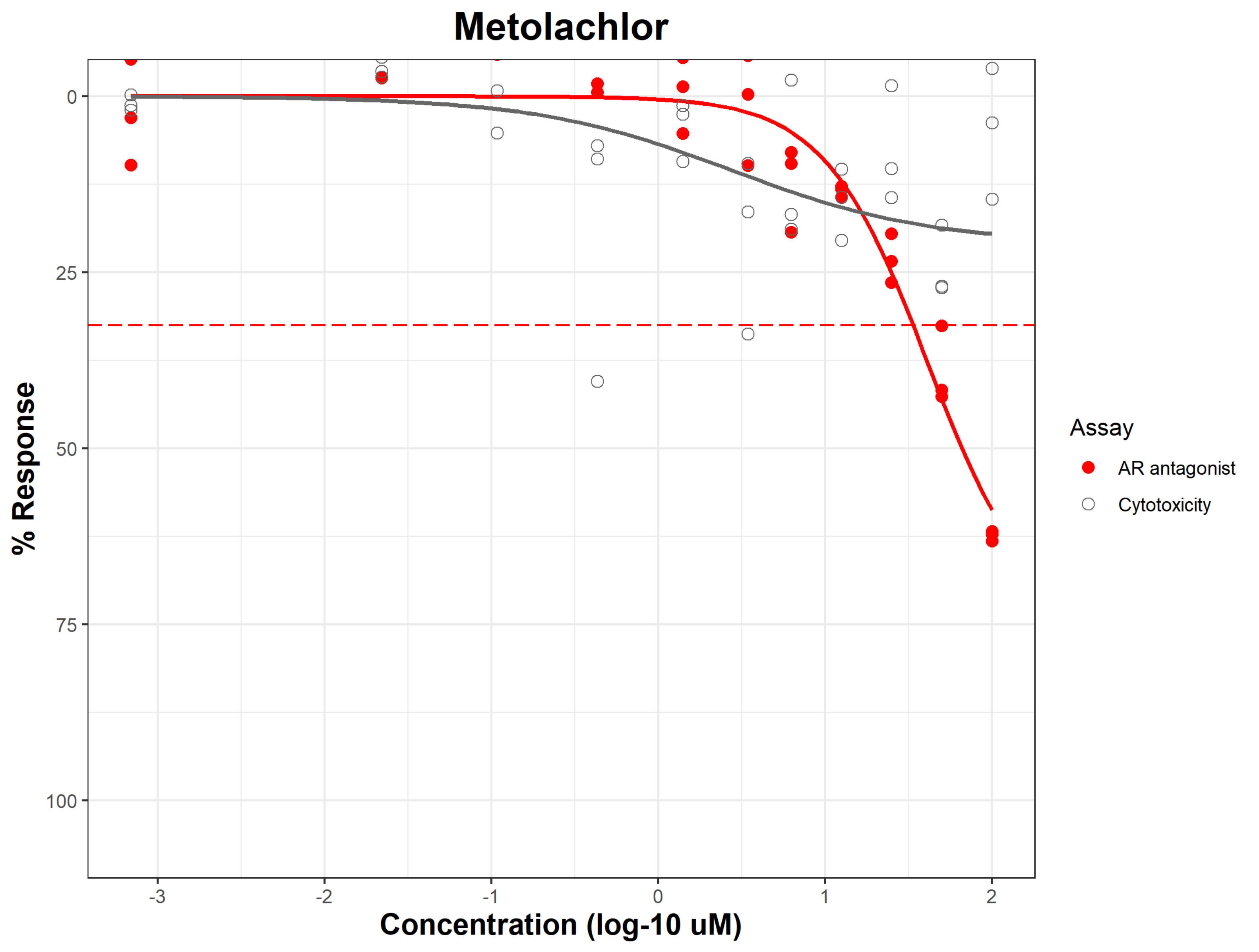

Metolachlor
% Response
0
25
50
75
100
-3
-2
-1
0
1
2
Concentration (log-10 uM)
Assay
AR antagonist
Cytotoxicity

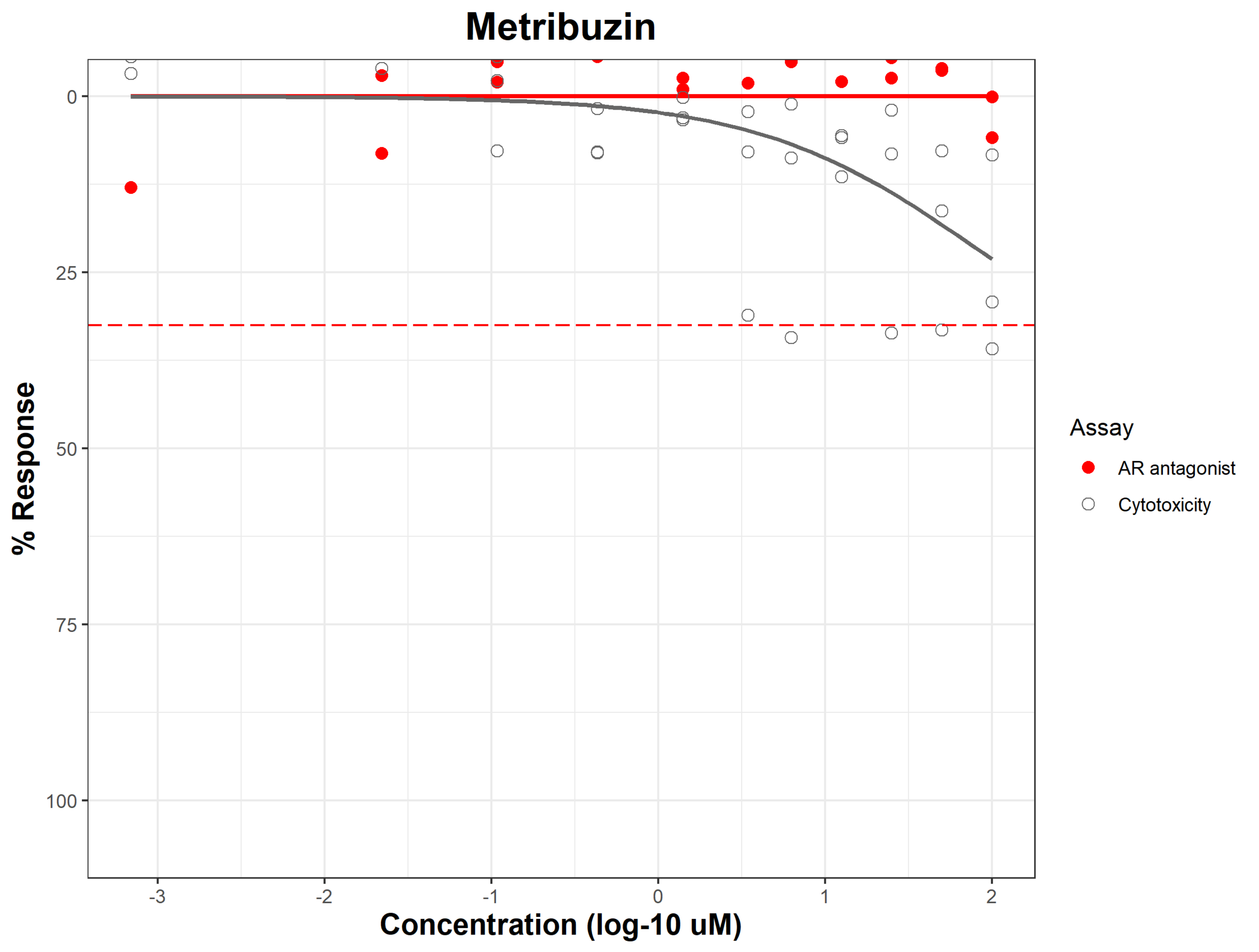

Metribuzin
% Response
0
25
50
75
100
-3
-2
-1
0
1
2
Concentration (log-10 uM)
Assay
AR antagonist
Cytotoxicity

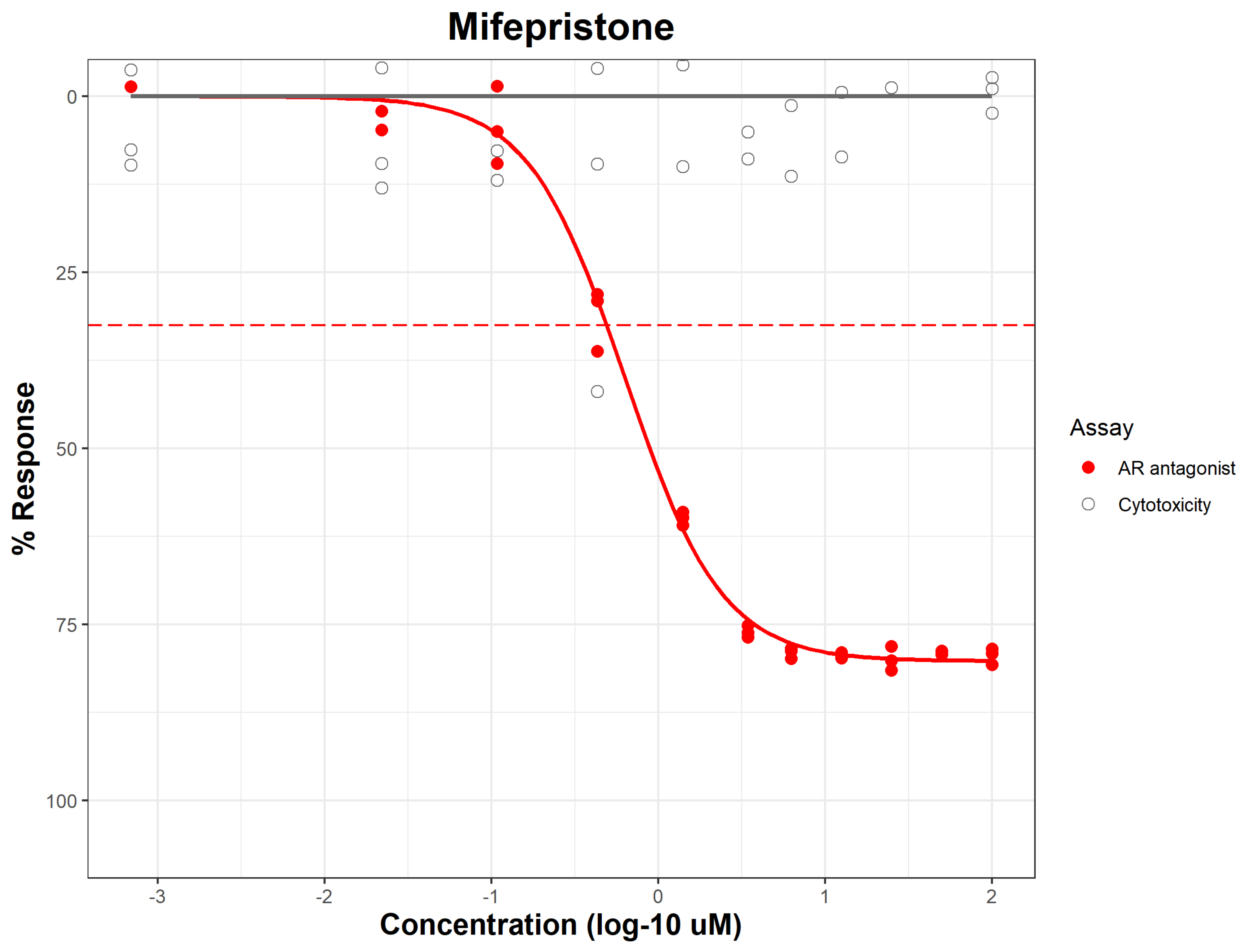

Mifepristone
% Response
0
25
50
75
100
-3
-2
-1
0
1
2
Concentration (log-10 uM)
Assay
AR antagonist
Cytotoxicity

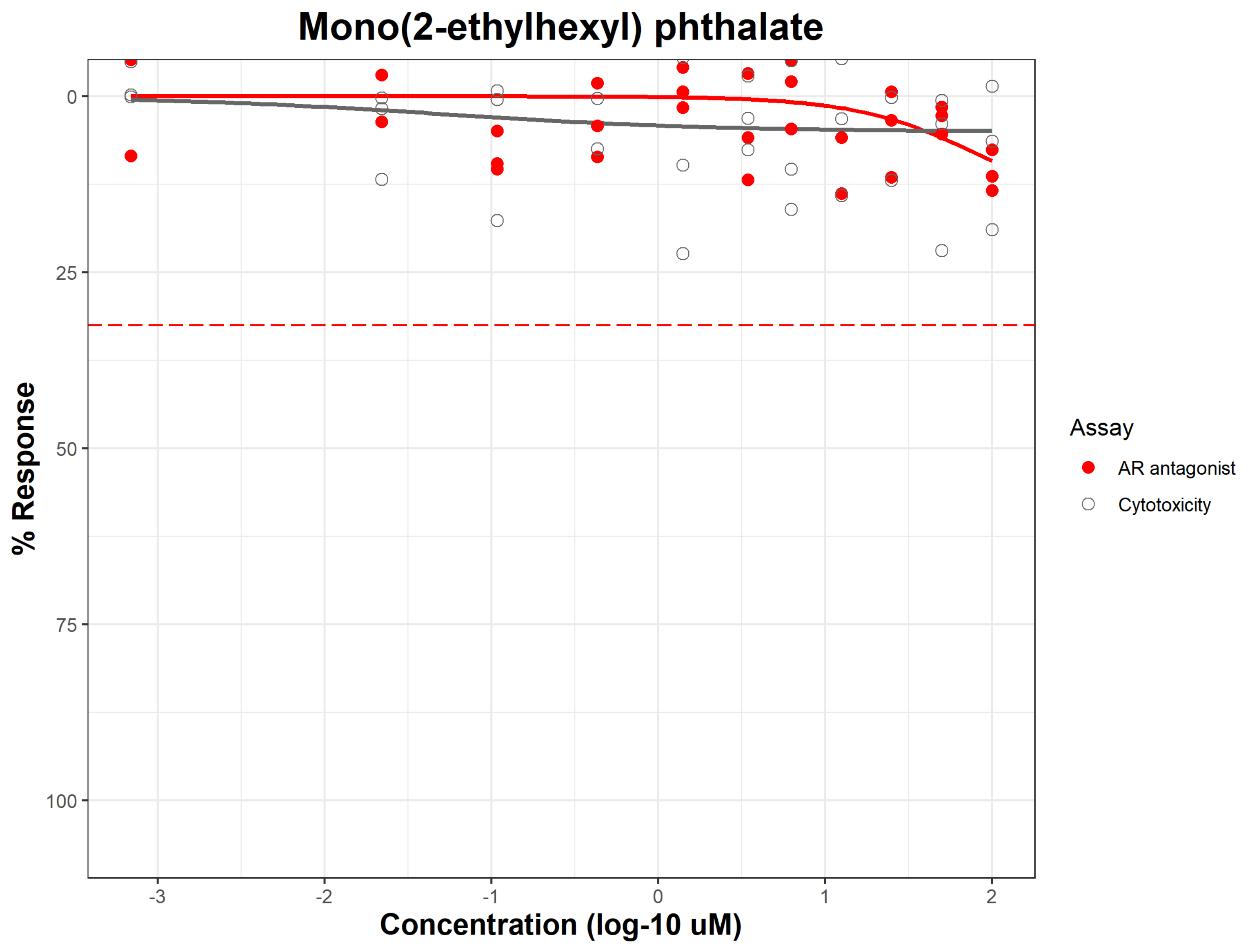
Mono(2-ethylhexyl) phthalate
% Response
0
25
50
75
100
-3
-2
-1
0
1
2
Concentration (log-10 uM)
Assay
AR antagonist
Cytotoxicity

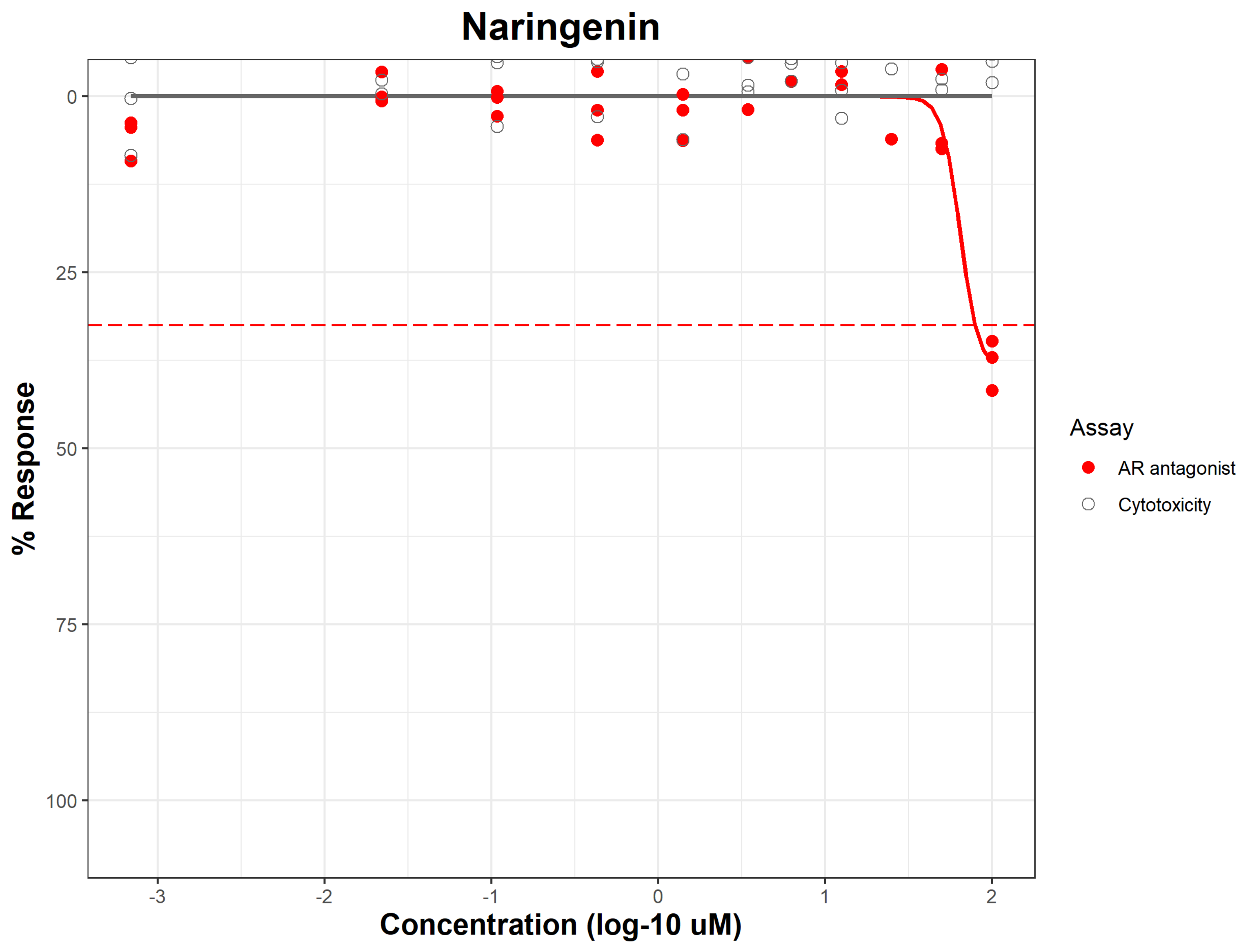

Naringenin
% Response
0
25
50
75
100
Concentration (log-10 uM)
-3
-2
-1
0
1
2
Assay
AR antagonist
Cytotoxicity

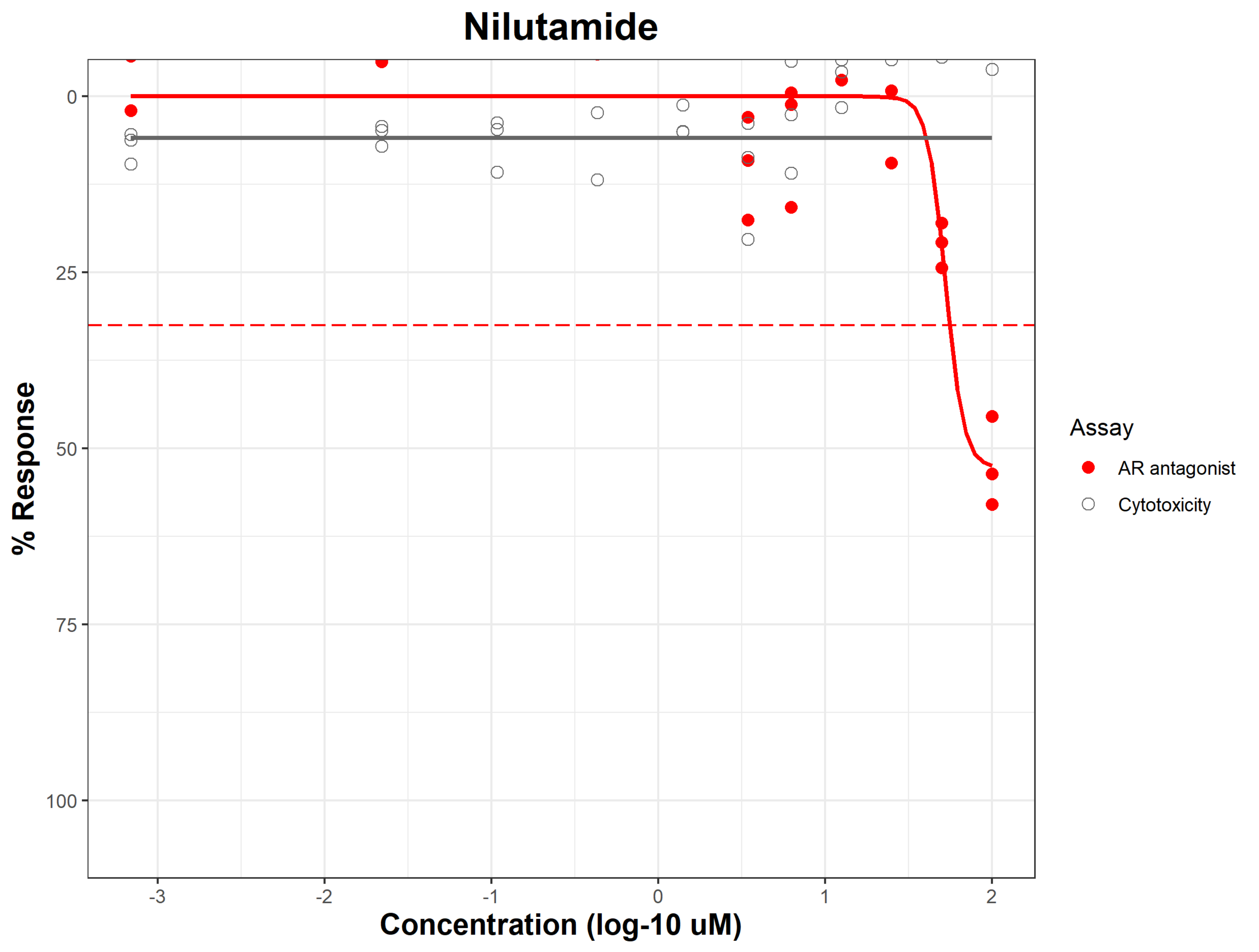

Nilutamide
% Response
0
25
50
75
100
-3
-2
-1
0
1
2
Concentration (log-10 uM)
Assay
AR antagonist
Cytotoxicity

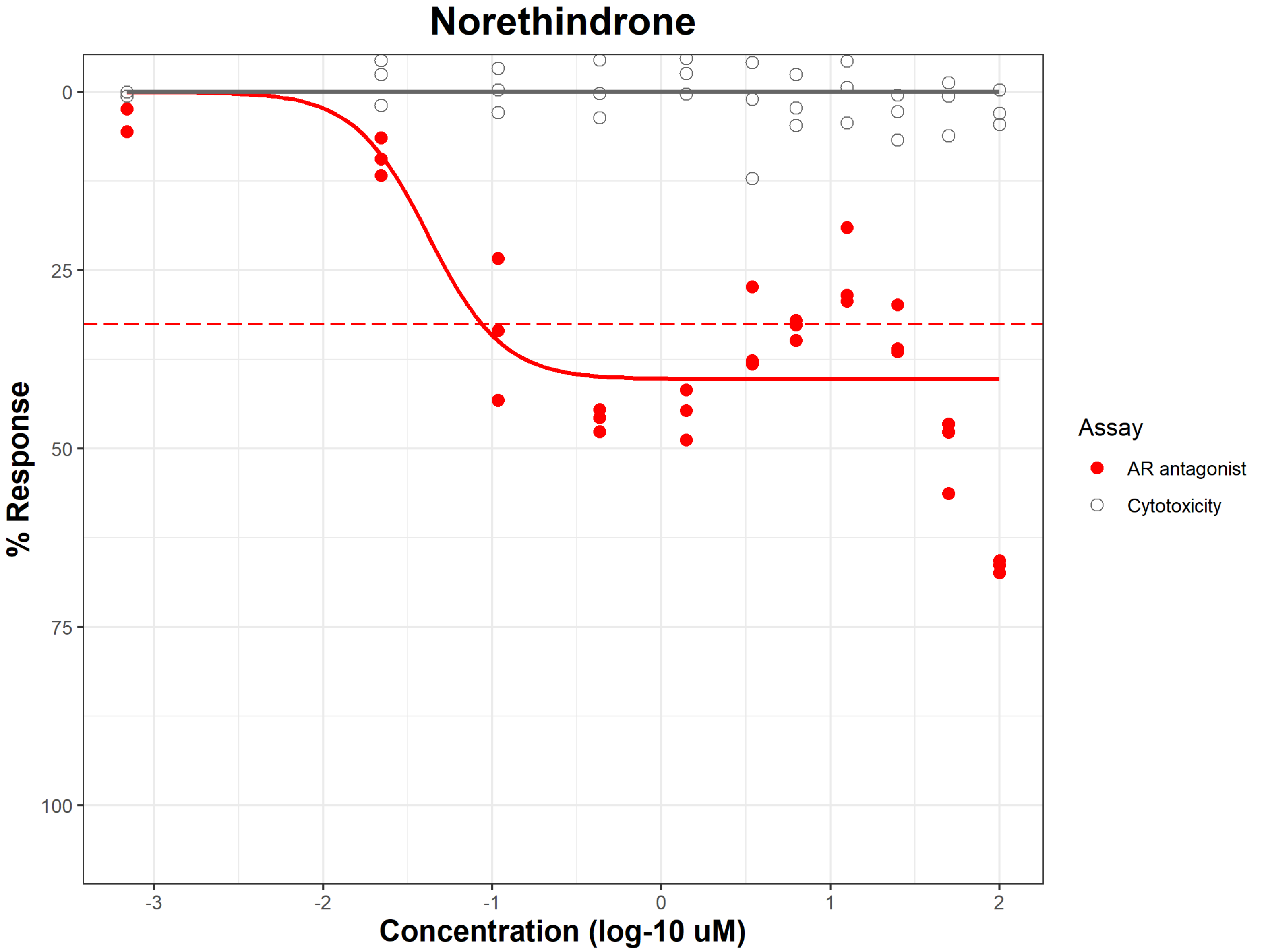

Norethindrone
% Response
0
25
50
75
100
Concentration (log-10 uM)
-3
-2
-1
0
1
2
Assay
AR antagonist
Cytotoxicity

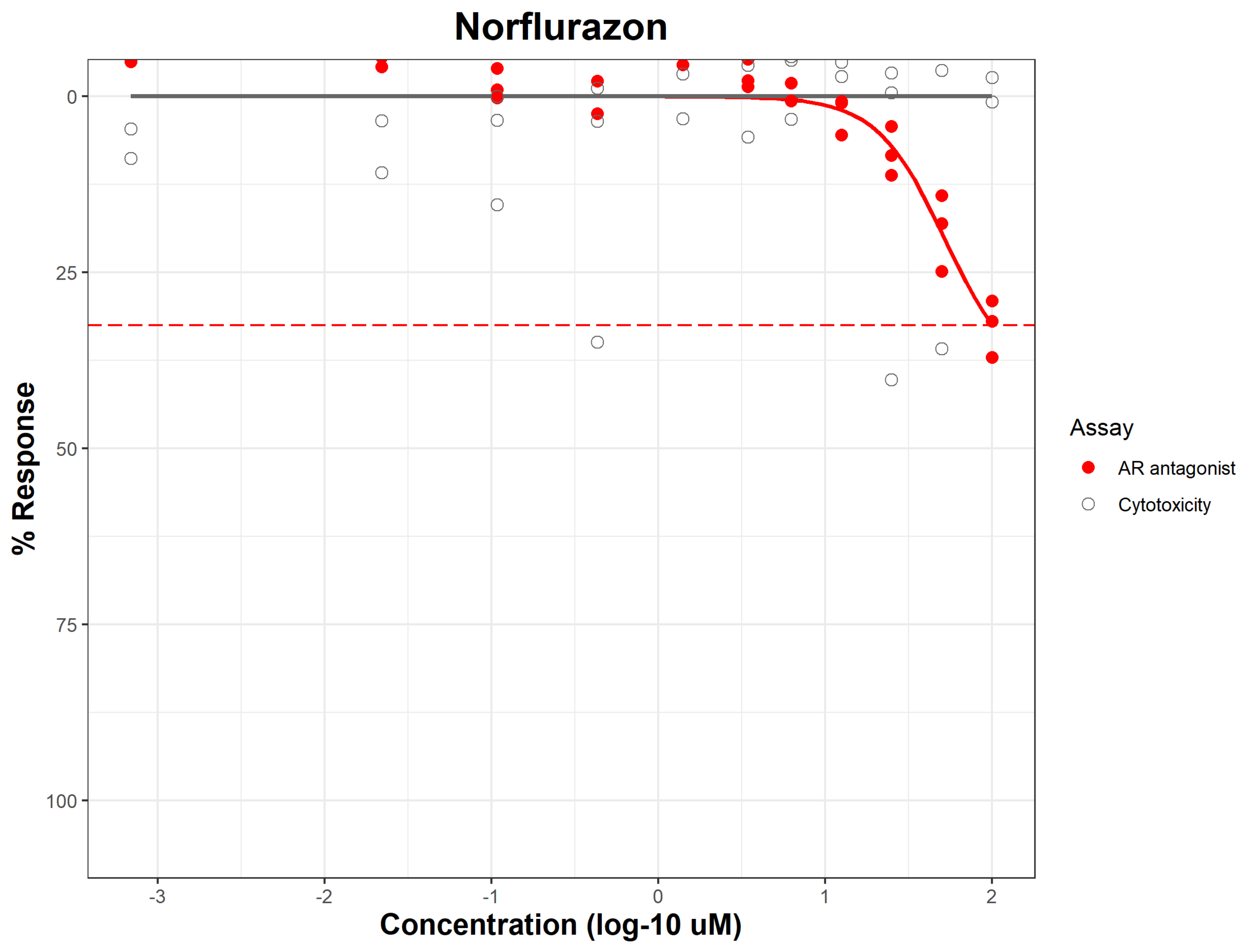

Norflurazon
% Response
0
25
50
75
100
-3
-2
-1
0
1
2
Concentration (log-10 uM)
Assay
AR antagonist
Cytotoxicity

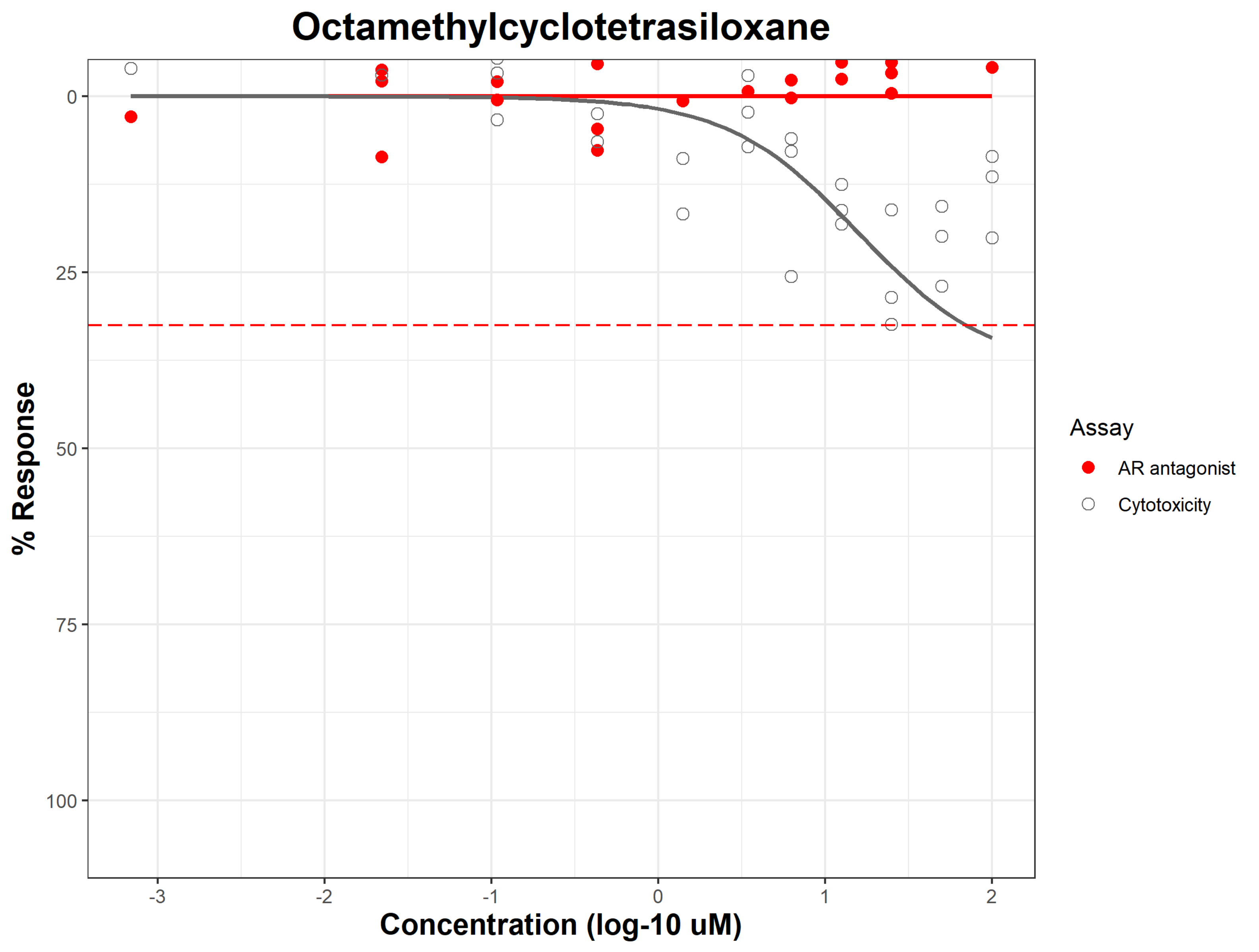

Octamethylcyclotetrasiloxane
% Response
0
25
50
75
100
-3
-2
-1
0
1
2
Concentration (log-10 uM)
Assay
AR antagonist
Cytotoxicity

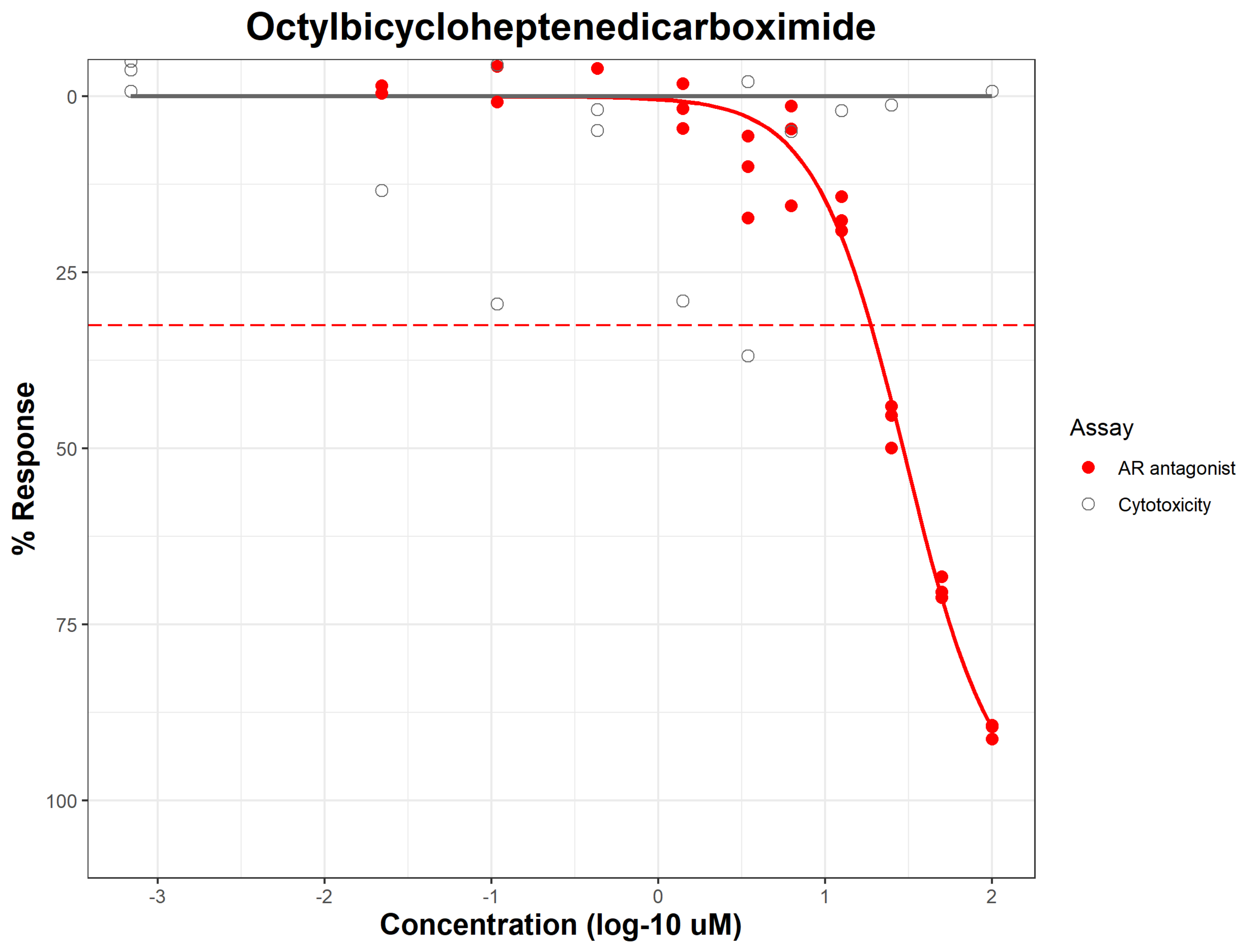

Octylbicycloheptenedicarboximide
% Response
0
25
50
75
100
Concentration (log-10 uM)
-3
-2
-1
0
1
2
Assay
AR antagonist
Cytotoxicity

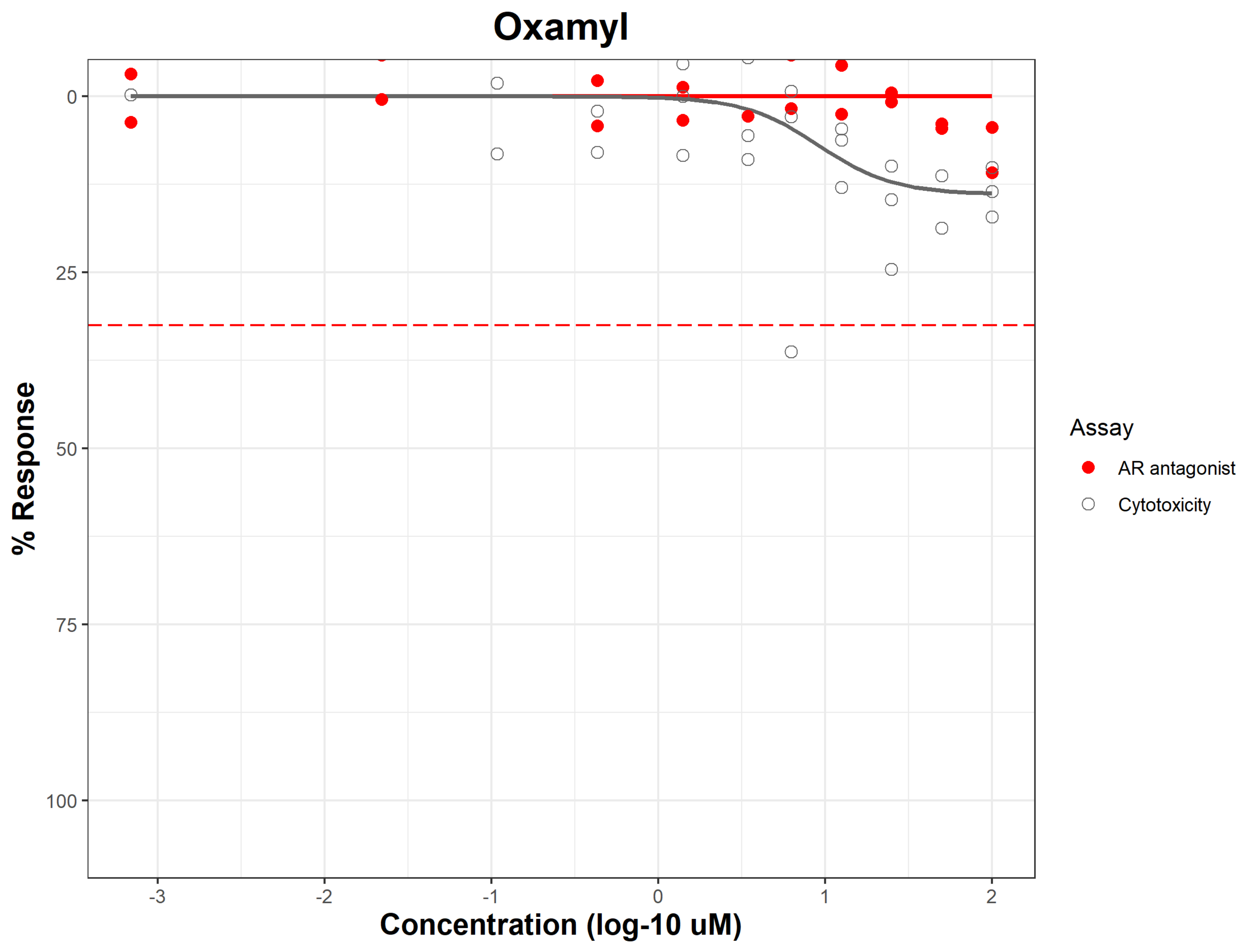

Oxamyl
% Response
0
25
50
75
100
-3
-2
-1
0
1
2
Concentration (log-10 uM)
Assay
AR antagonist
Cytotoxicity

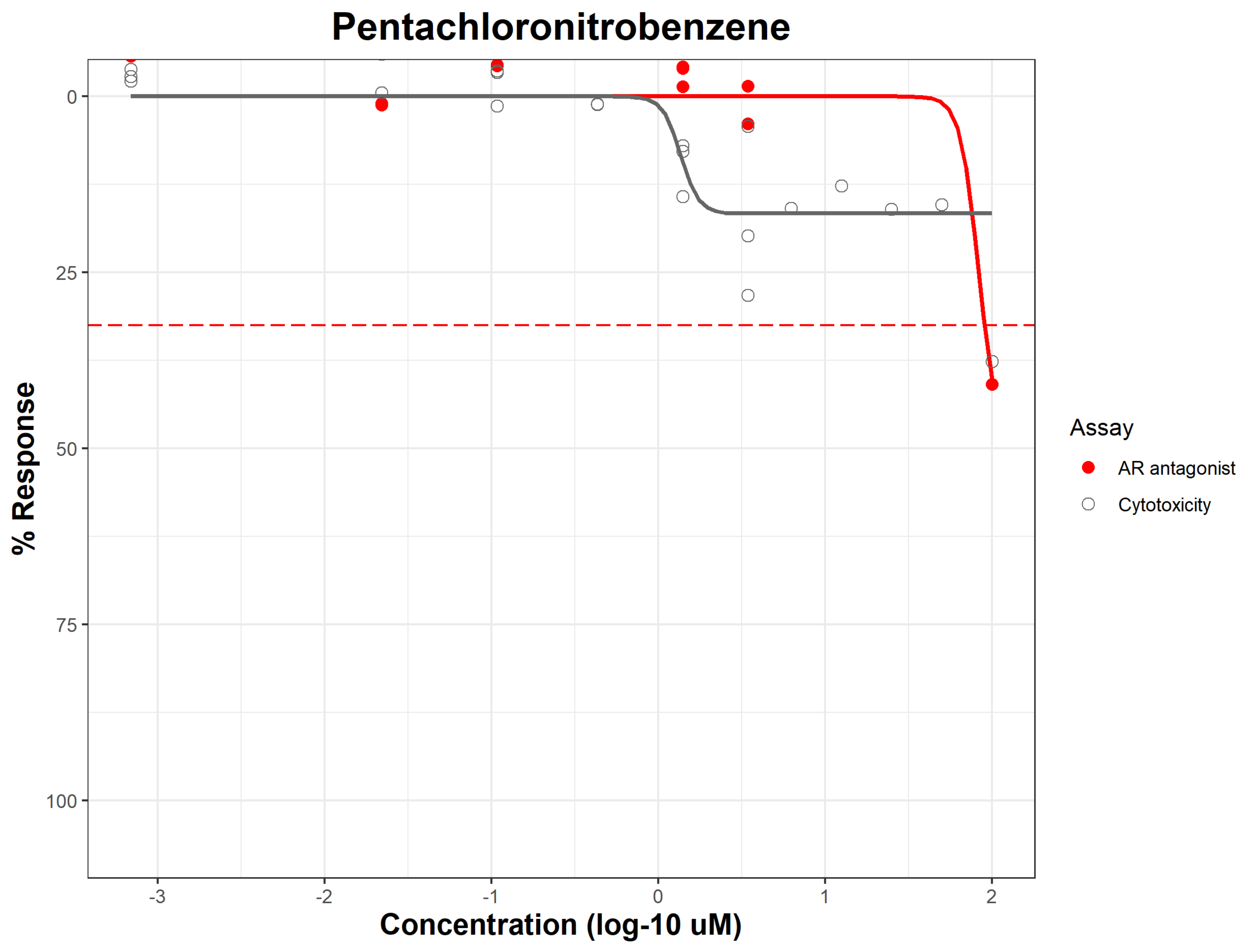

Pentachloronitrobenzene
% Response
0
25
50
75
100
-3
-2
-1
0
1
2
Concentration (log-10 uM)
Assay
AR antagonist
Cytotoxicity

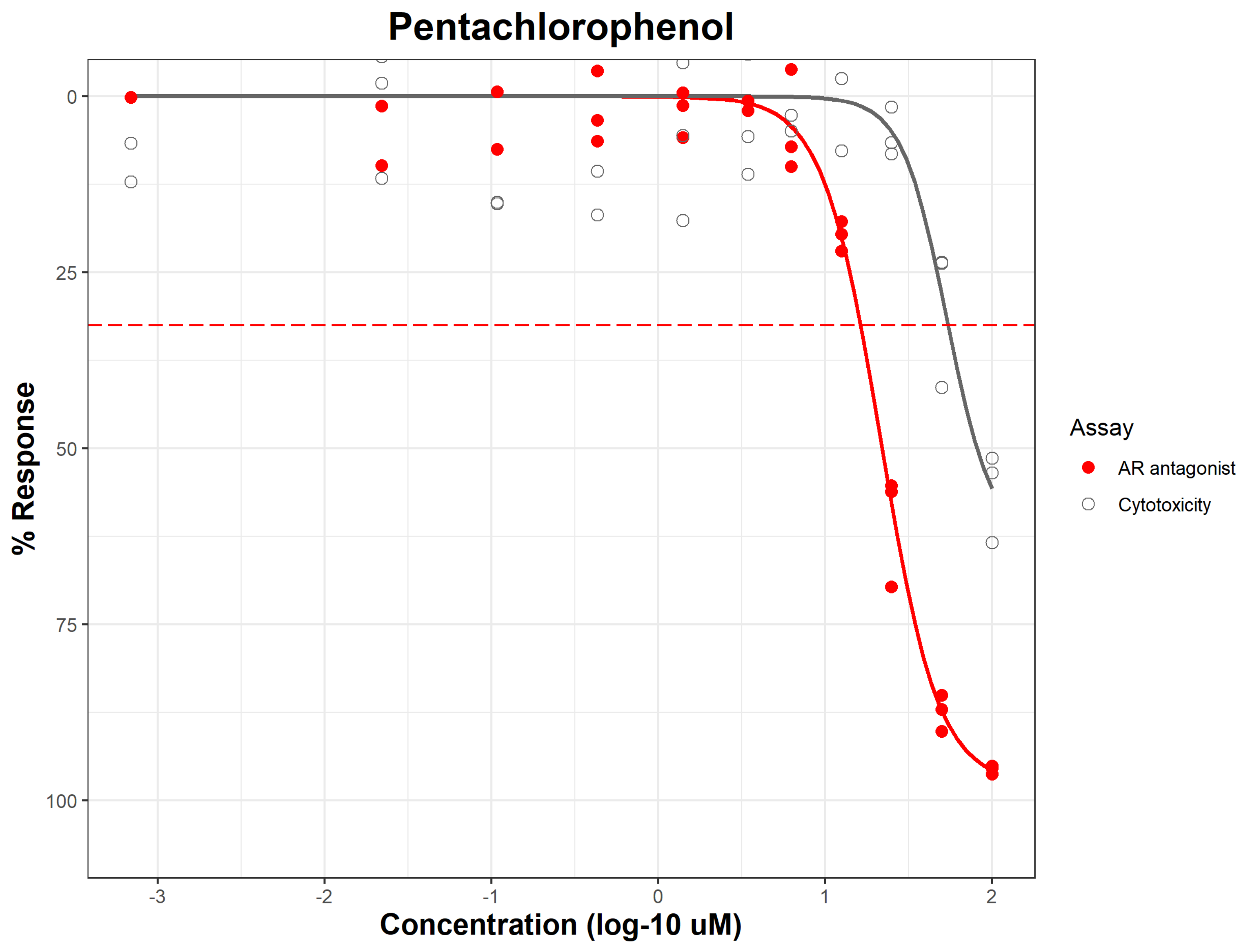

Pentachlorophenol
% Response
0
25
50
75
100
-3
-2
-1
0
1
2
Concentration (log-10 uM)
Assay
AR antagonist
Cytotoxicity

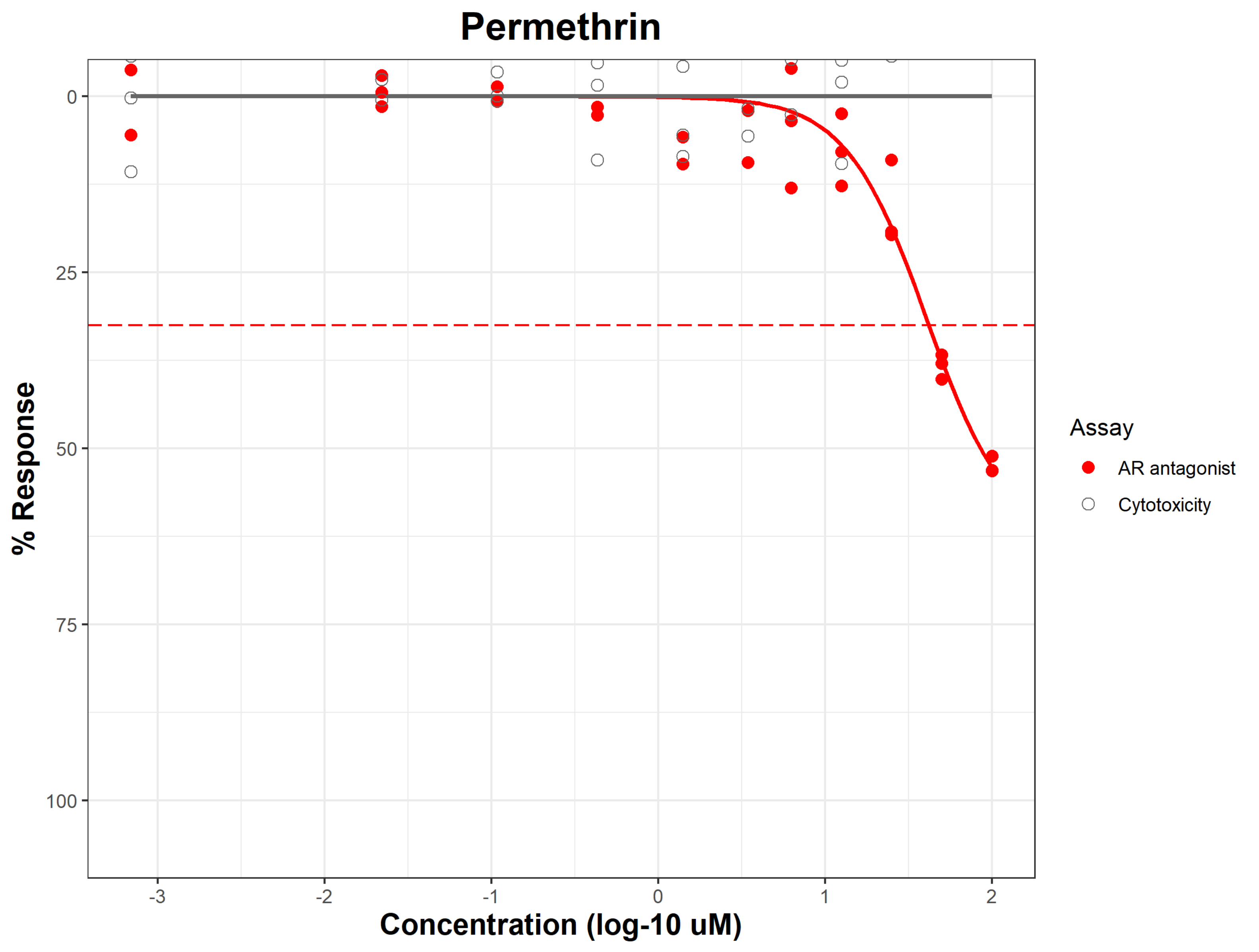

Permethrin
% Response
0
25
50
75
100
-3
-2
-1
0
1
2
Concentration (log-10 uM)
Assay
AR antagonist
Cytotoxicity

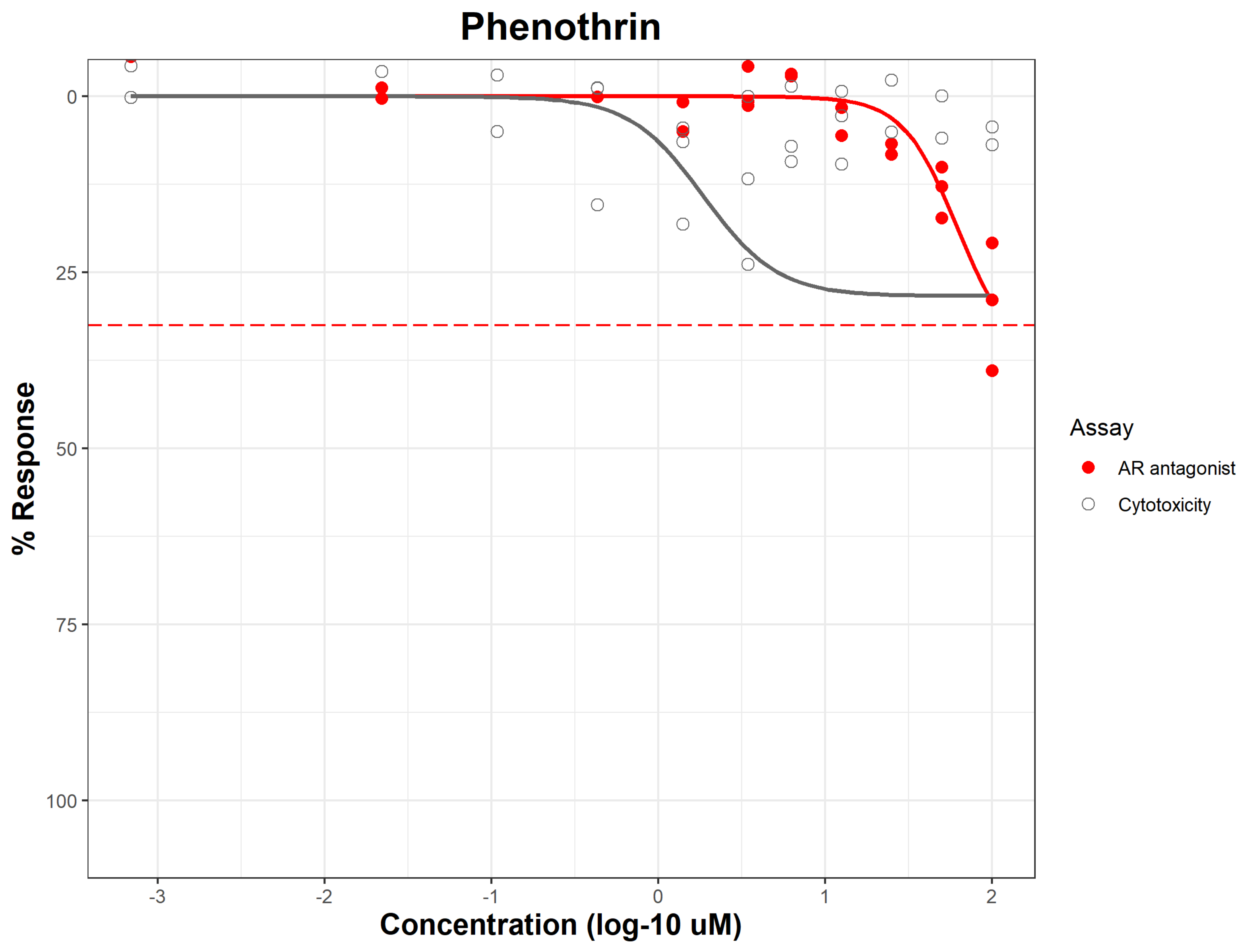

Phenothrin
% Response
Concentration (log-10 uM)
0
25
50
75
100
-3
-2
-1
0
1
2
Assay
AR antagonist
Cytotoxicity

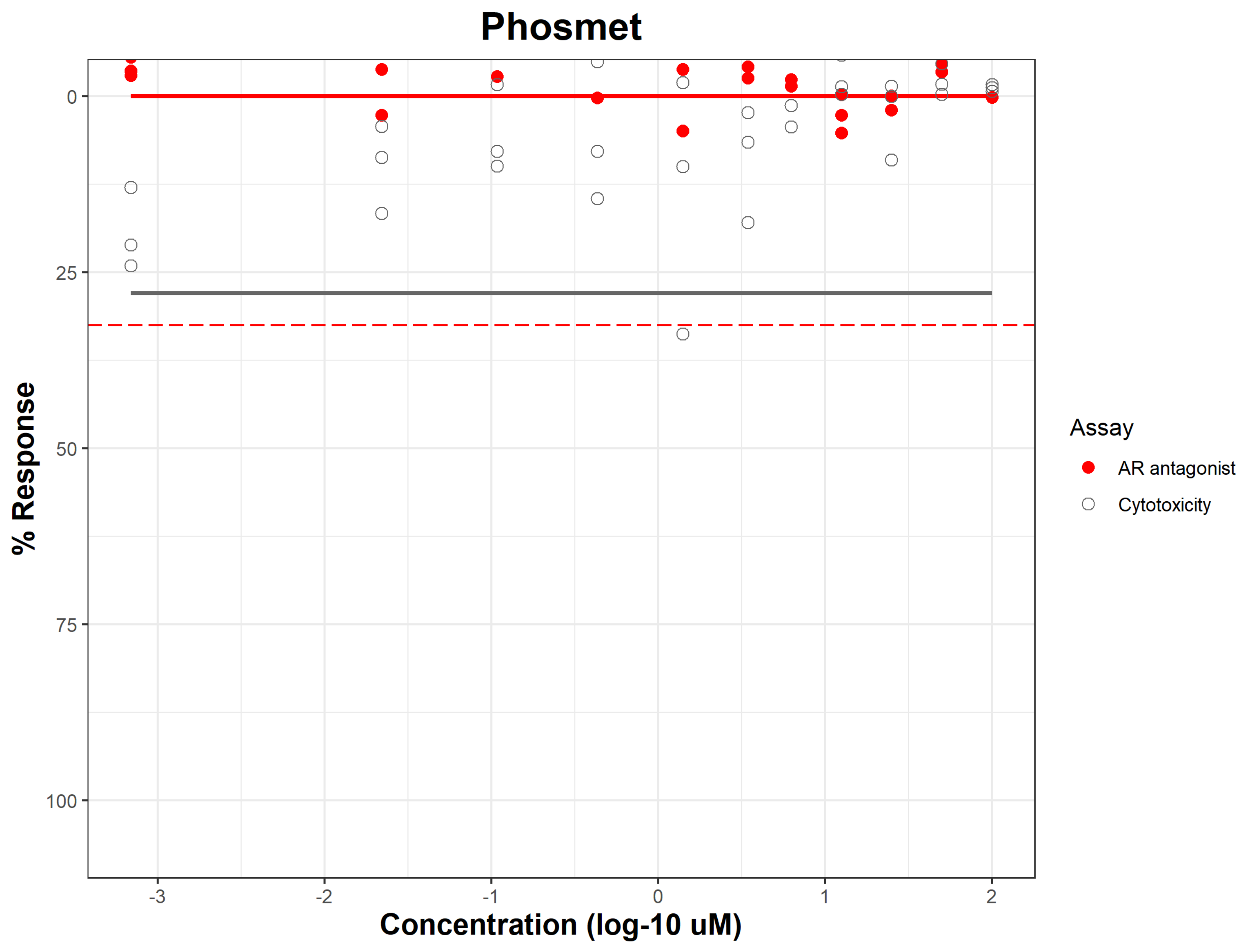

Phosmet
% Response
0
25
50
75
100
-3
-2
-1
0
1
2
Concentration (log-10 uM)
Assay
AR antagonist
Cytotoxicity

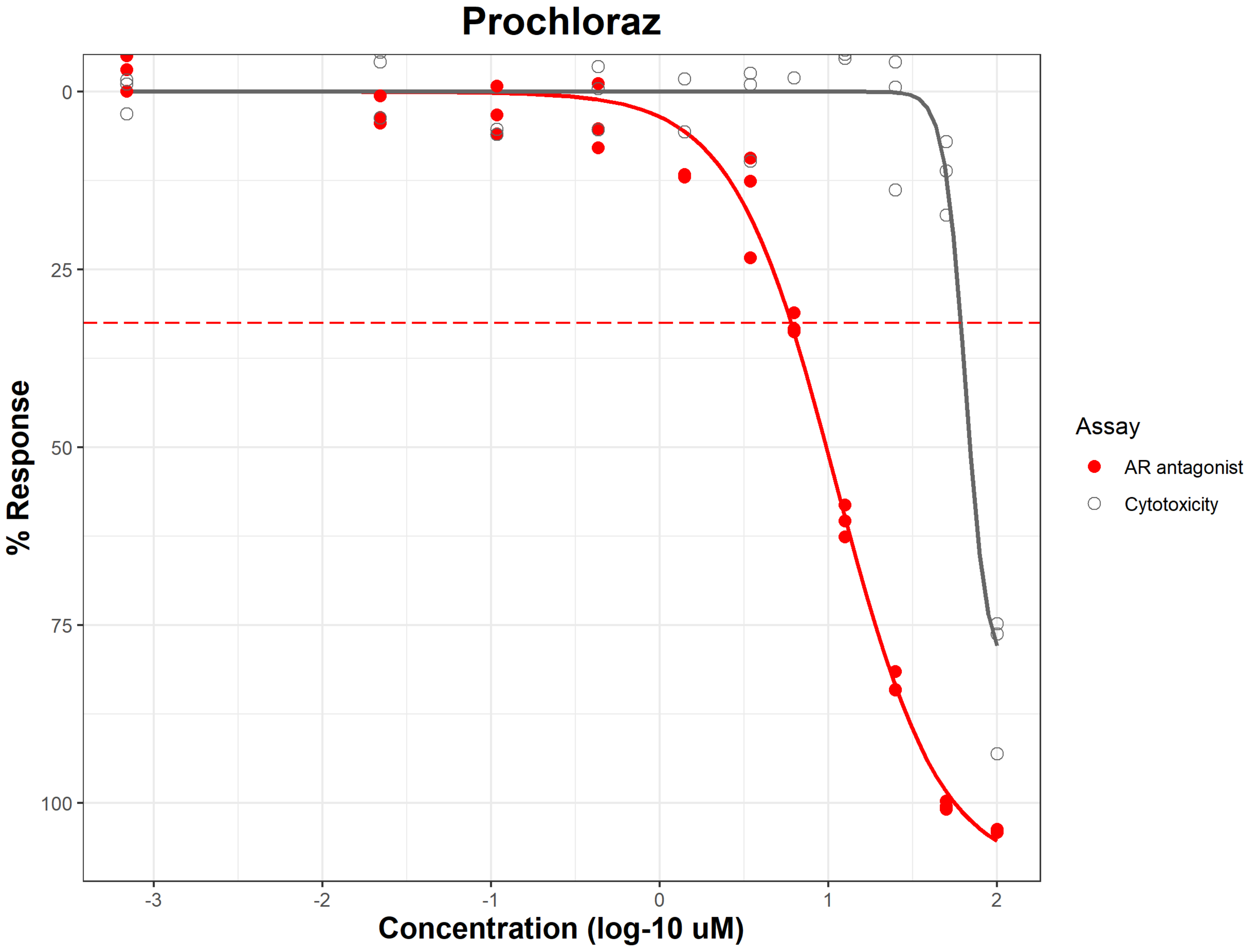

Prochloraz
% Response
0
25
50
75
100
-3
-2
-1
0
1
2
Concentration (log-10 uM)
Assay
AR antagonist
Cytotoxicity

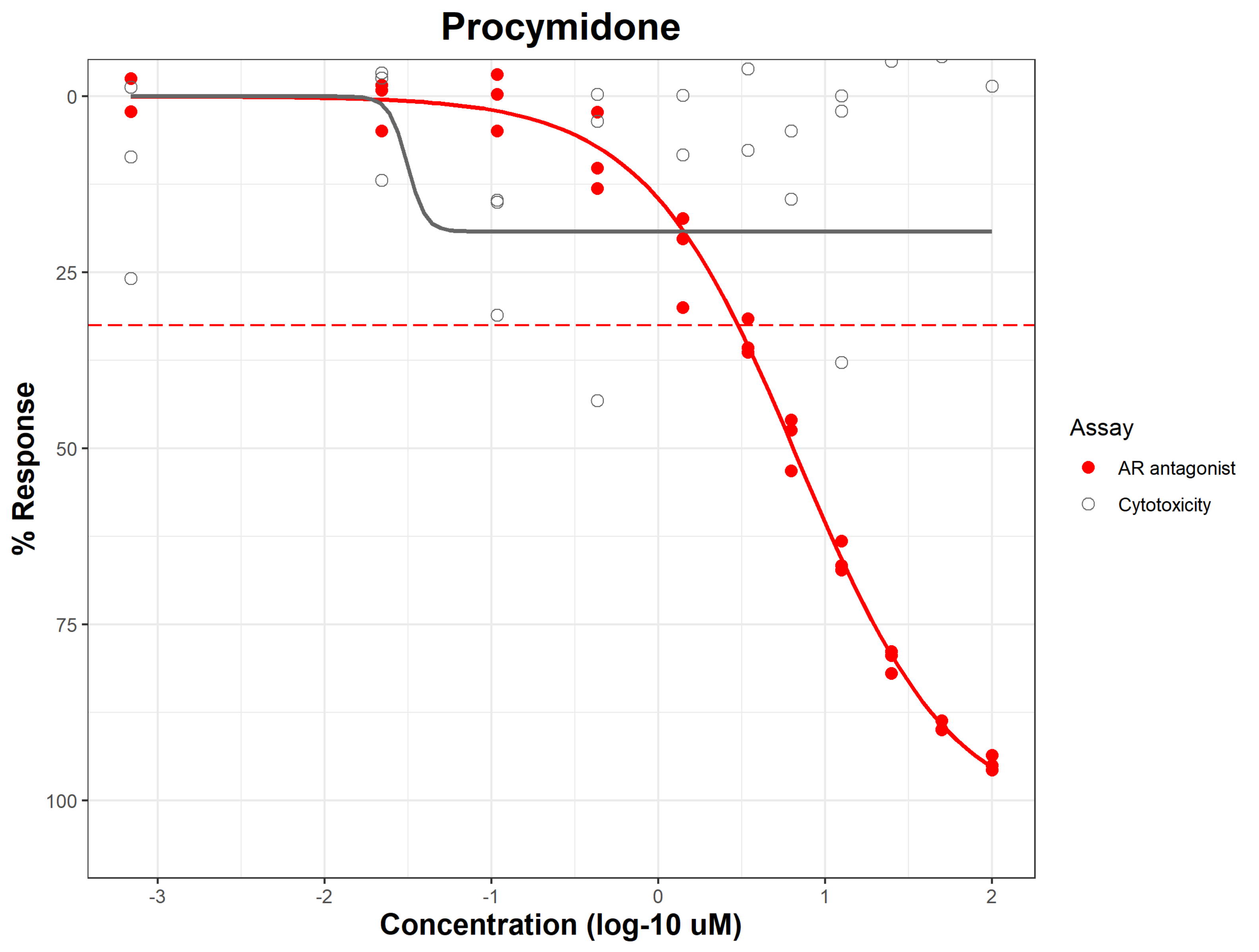

Procymidone
% Response
Concentration (log-10 uM)
0
25
50
75
100
-3
-2
-1
0
1
2
Assay
AR antagonist
Cytotoxicity

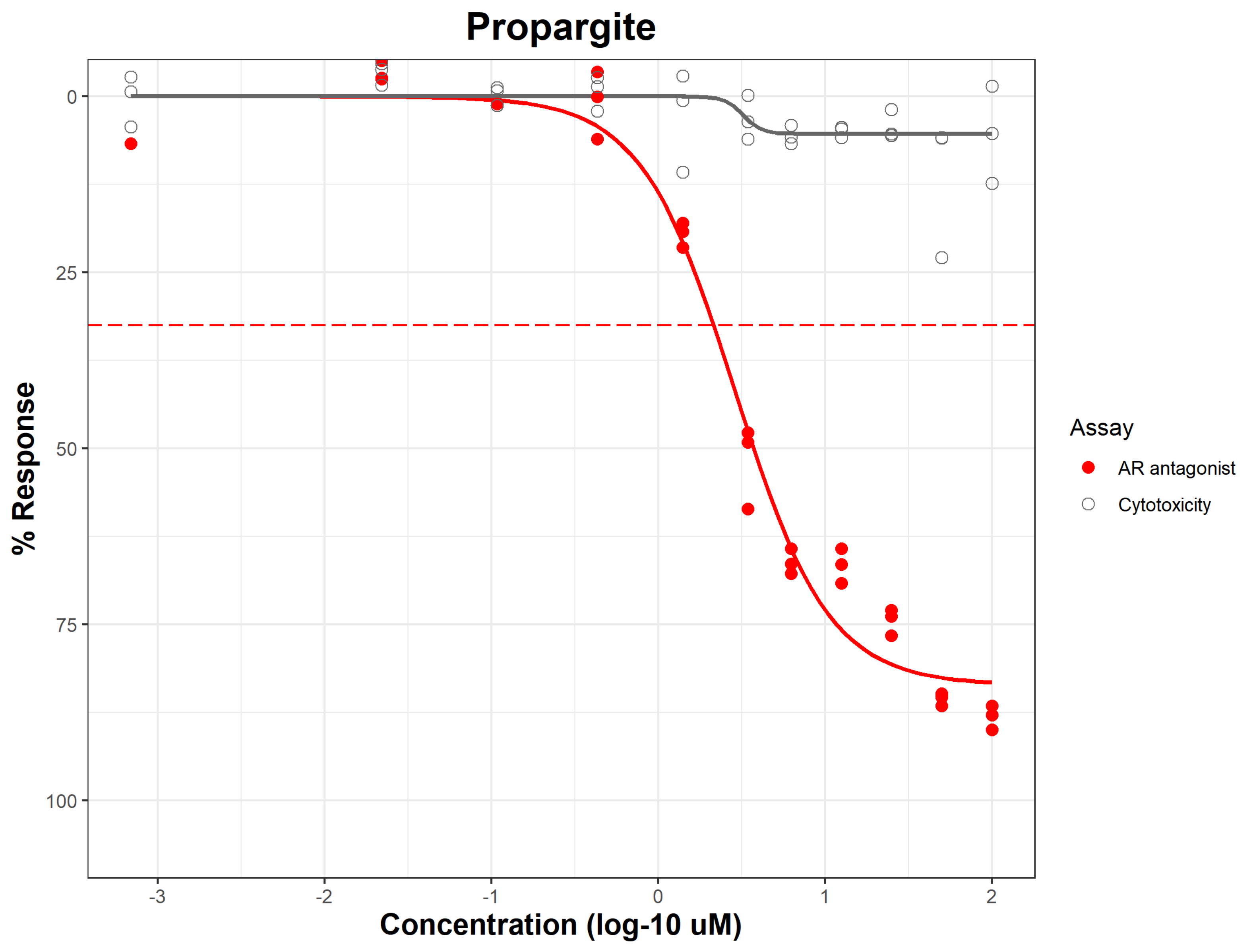

Propargite
% Response
0
25
50
75
100
-3
-2
-1
0
1
2
Concentration (log-10 uM)
Assay
AR antagonist
Cytotoxicity

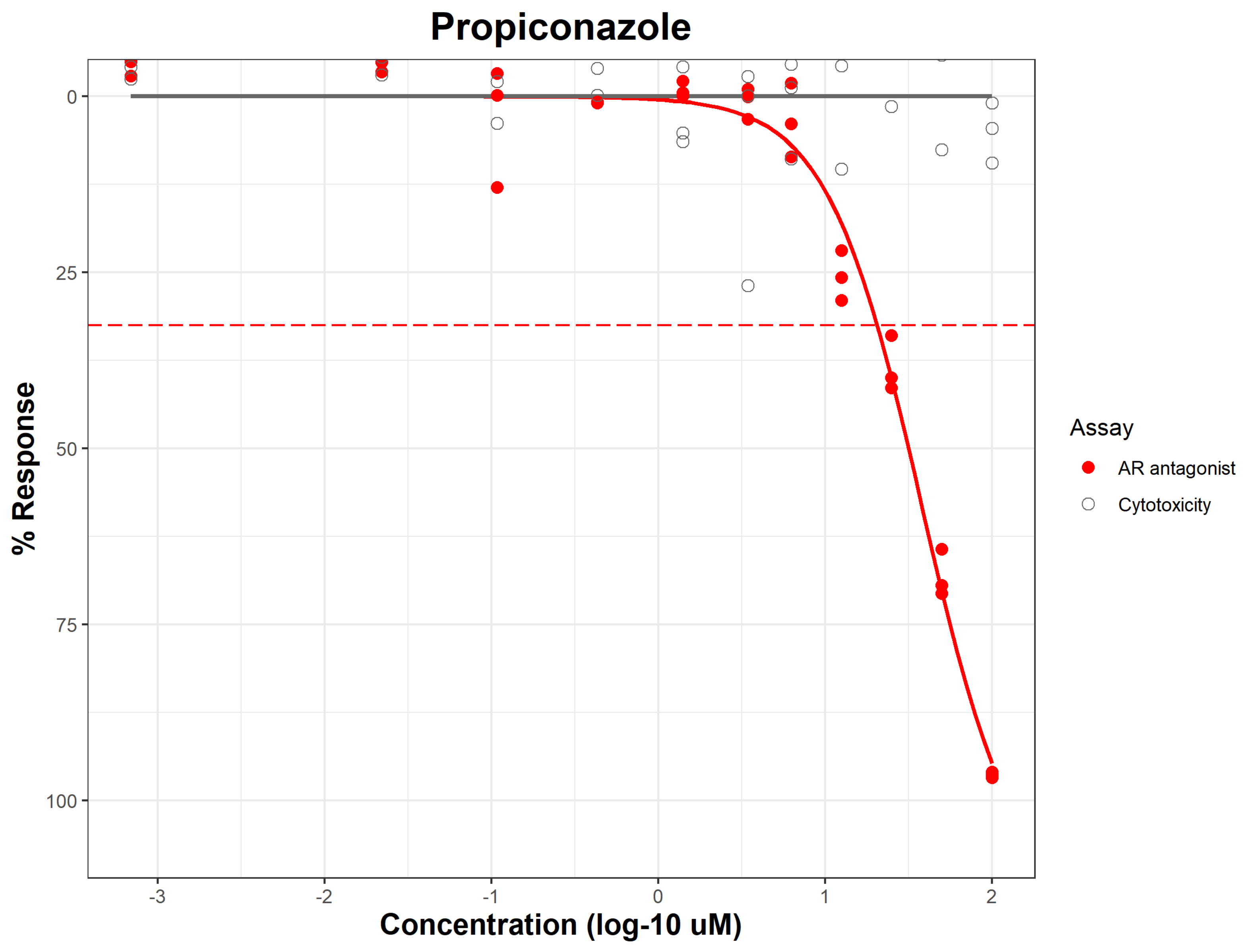
Propiconazole
% Response
0
25
50
75
100
-3
-2
-1
0
1
2
Concentration (log-10 uM)
Assay
AR antagonist
Cytotoxicity

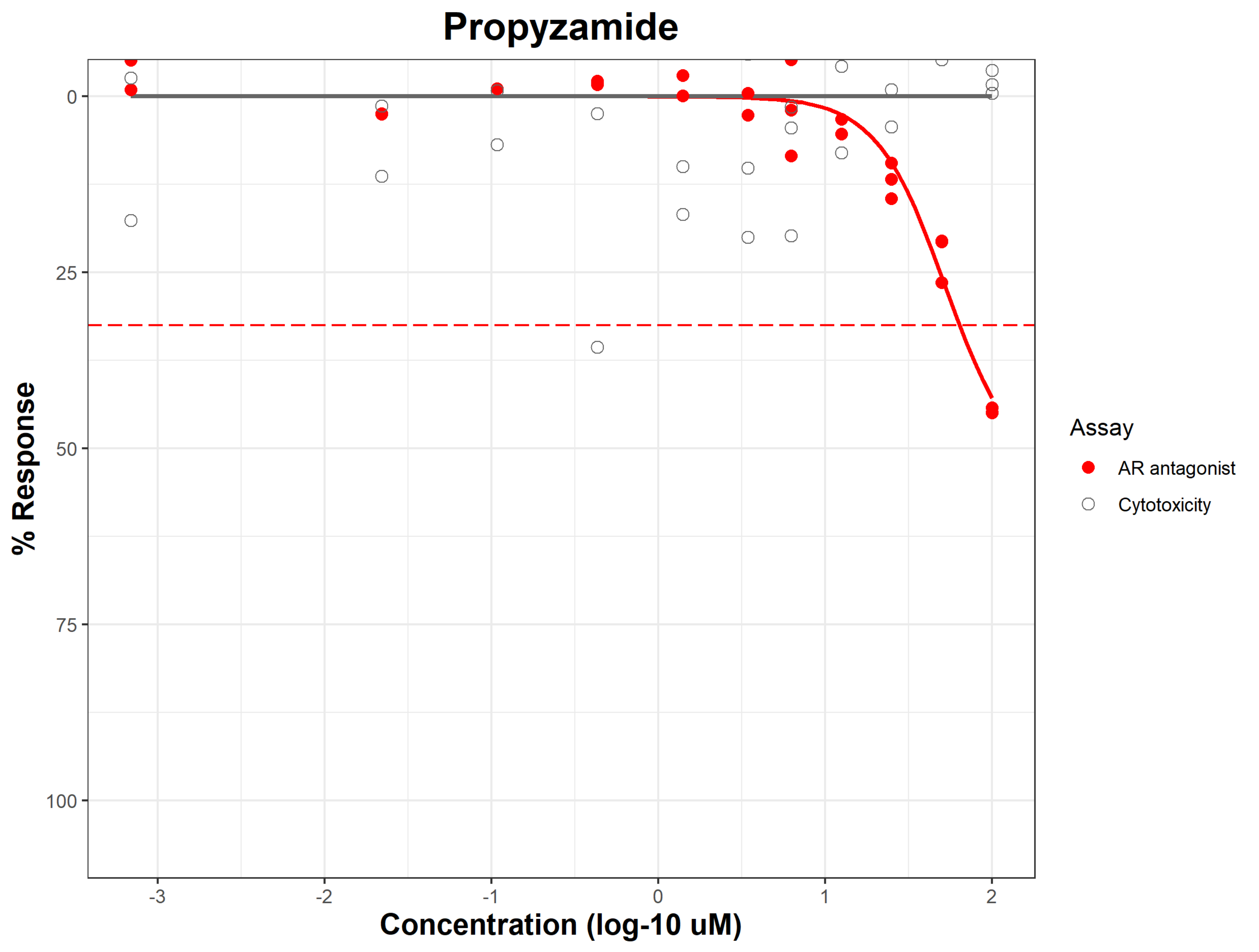

Propyzamide
% Response
Concentration (log-10 uM)
0
25
50
75
100
-3
-2
-1
0
1
2
Assay
AR antagonist
Cytotoxicity

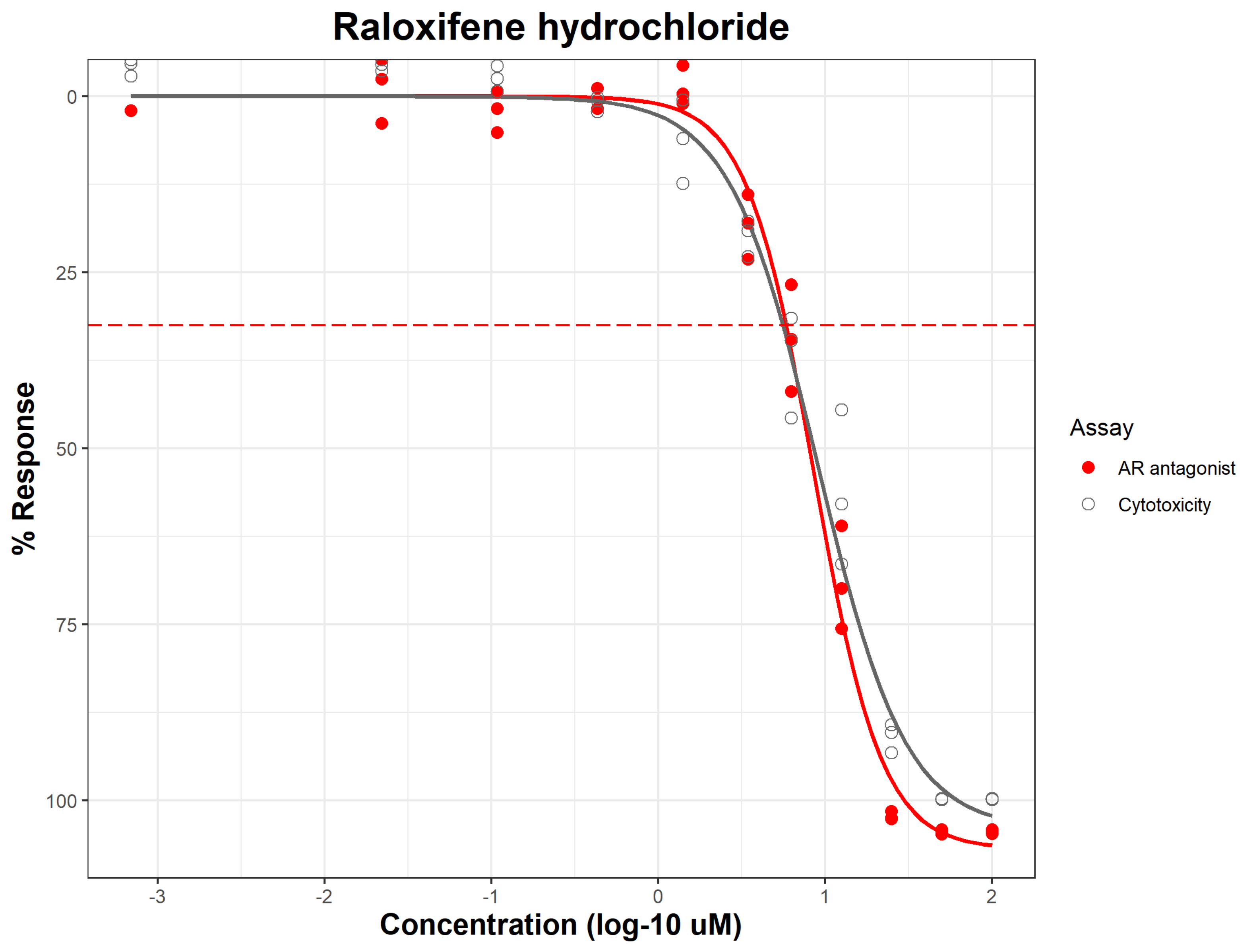

Raloxifene hydrochloride
% Response
0
25
50
75
100
-3
-2
-1
0
1
2
Concentration (log-10 uM)
Assay
AR antagonist
Cytotoxicity

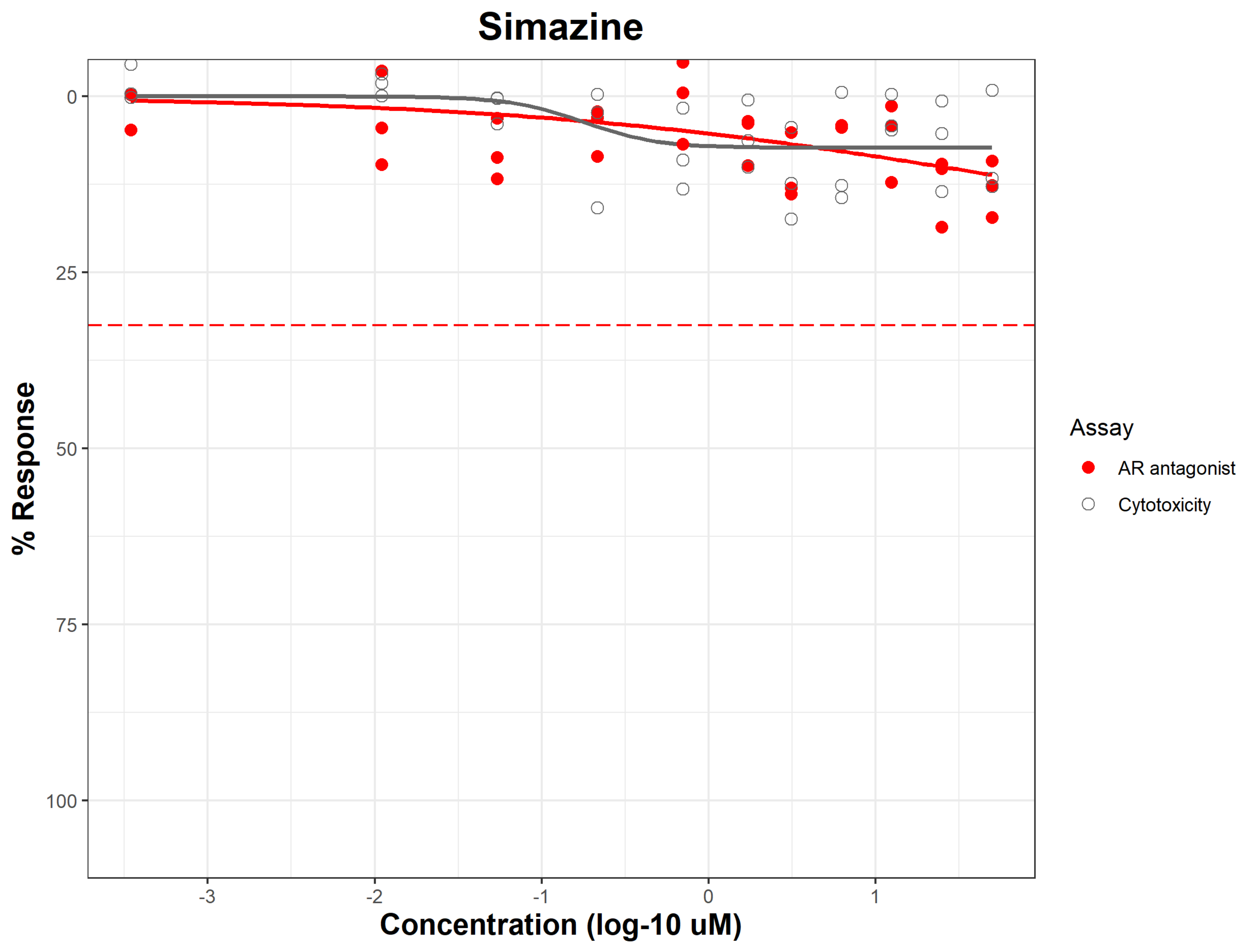

Simazine
% Response
0
25
50
75
100
-3
-2
-1
0
1
Concentration (log-10 uM)
Assay
AR antagonist
Cytotoxicity

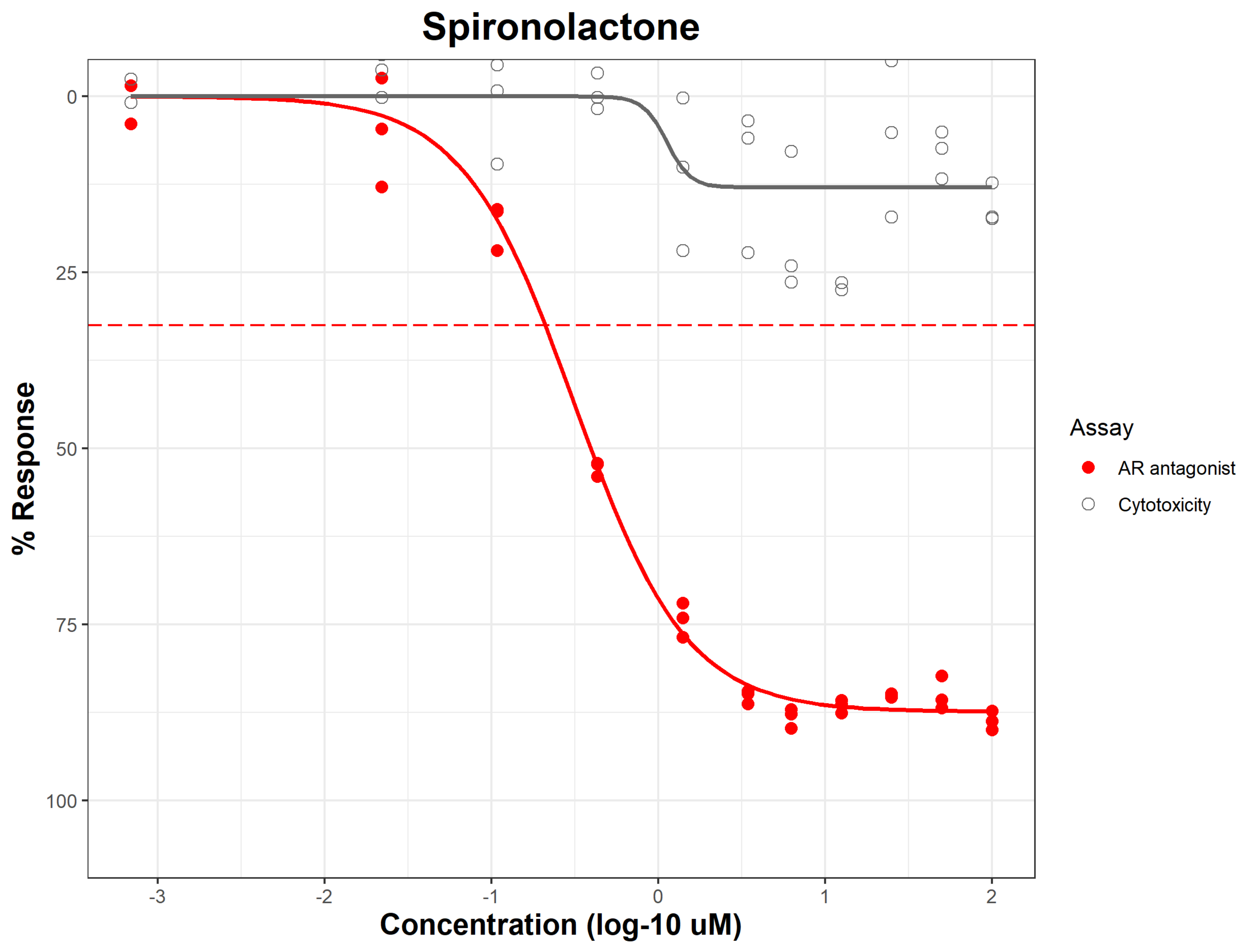

Spironolactone
% Response
Concentration (log-10 uM)
0
25
50
75
100
-3
-2
-1
0
1
2
Assay
AR antagonist
Cytotoxicity

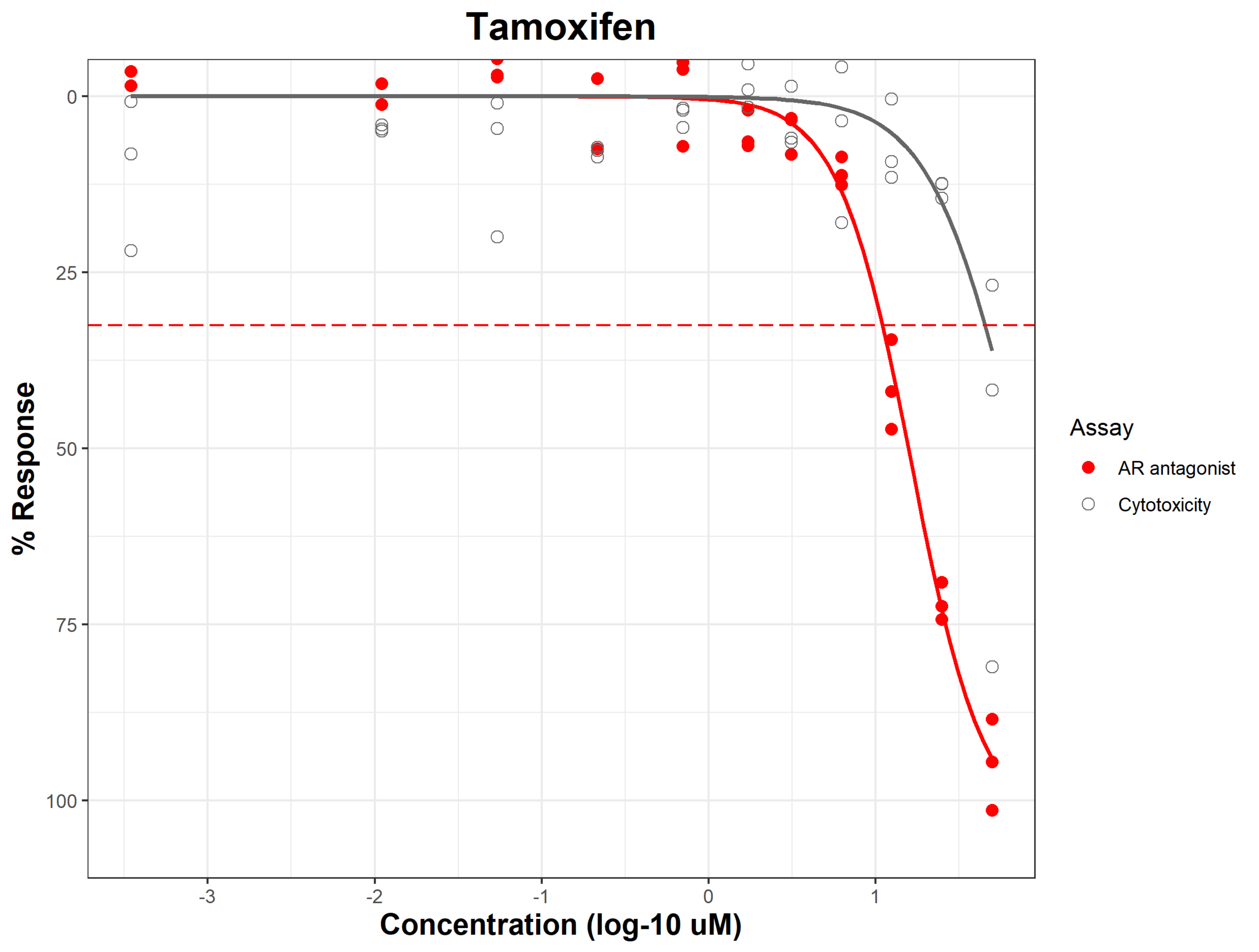

Tamoxifen
% Response
0
25
50
75
100
-3
-2
-1
0
1
Concentration (log-10 uM)
Assay
AR antagonist
Cytotoxicity

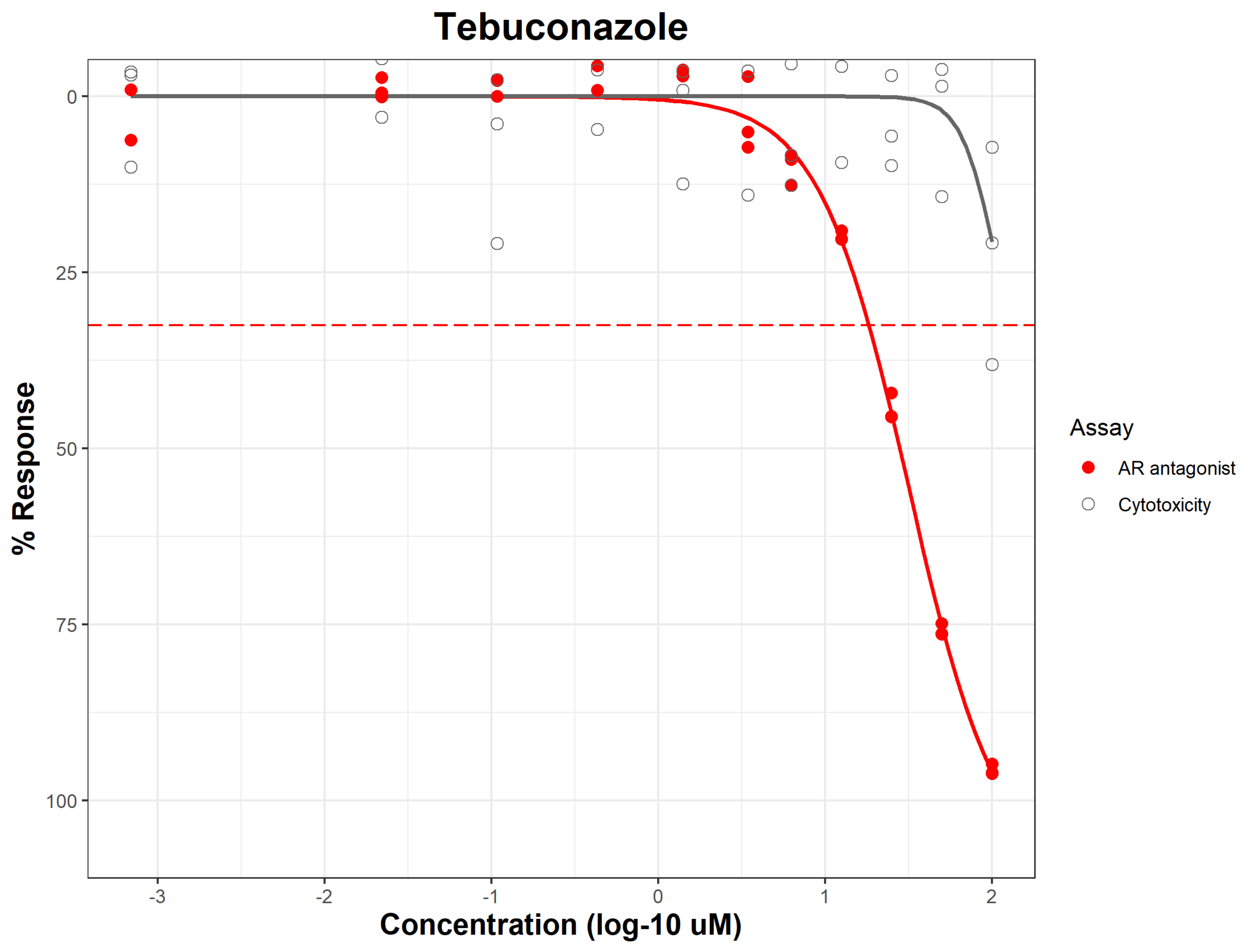
Tebuconazole
% Response
0
25
50
75
100
-3
-2
-1
0
1
2
Concentration (log-10 uM)
Assay
AR antagonist
Cytotoxicity

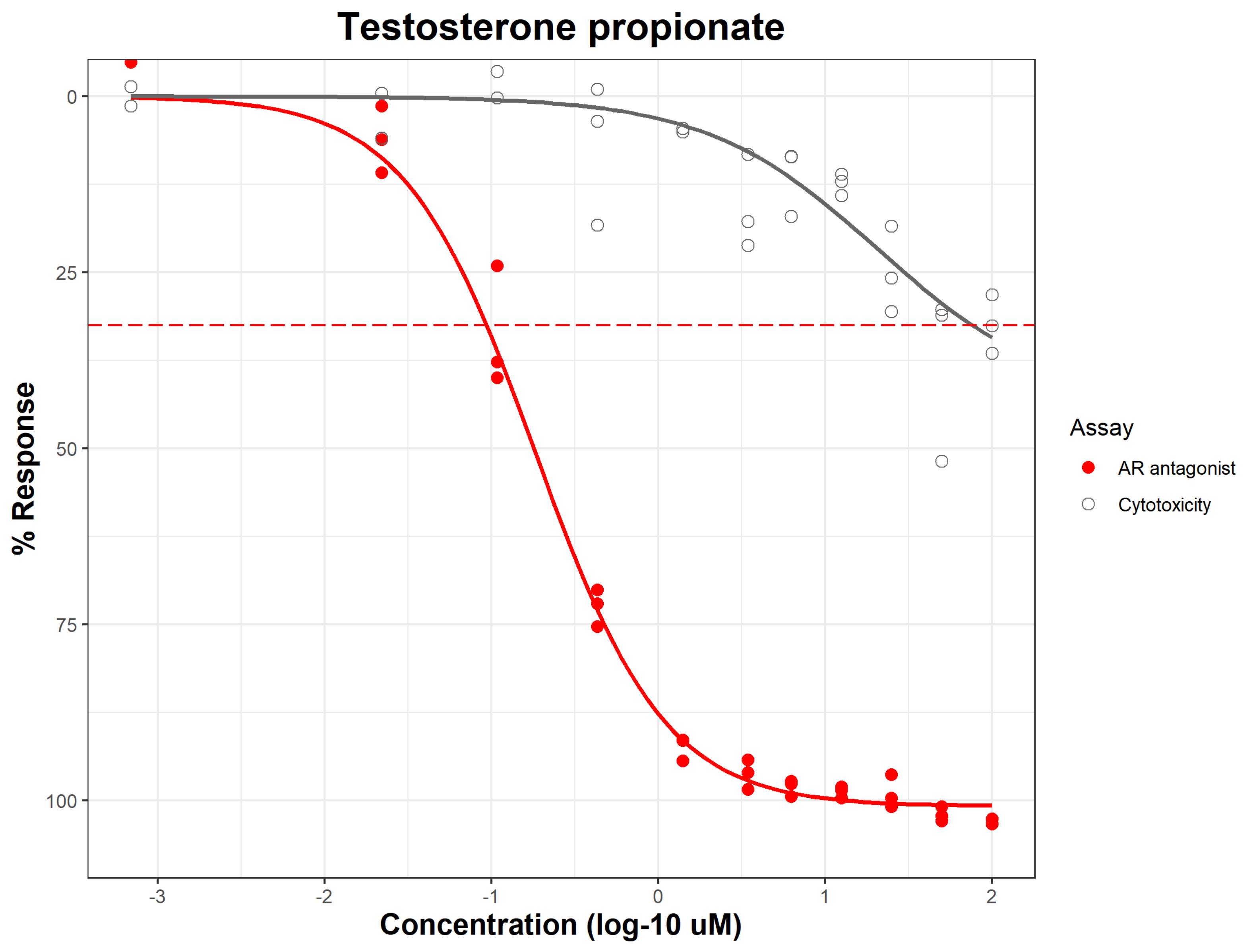
Testosterone propionate
% Response
0
25
50
75
100
-3
-2
-1
0
1
2
Concentration (log-10 uM)
Assay
AR antagonist
Cytotoxicity

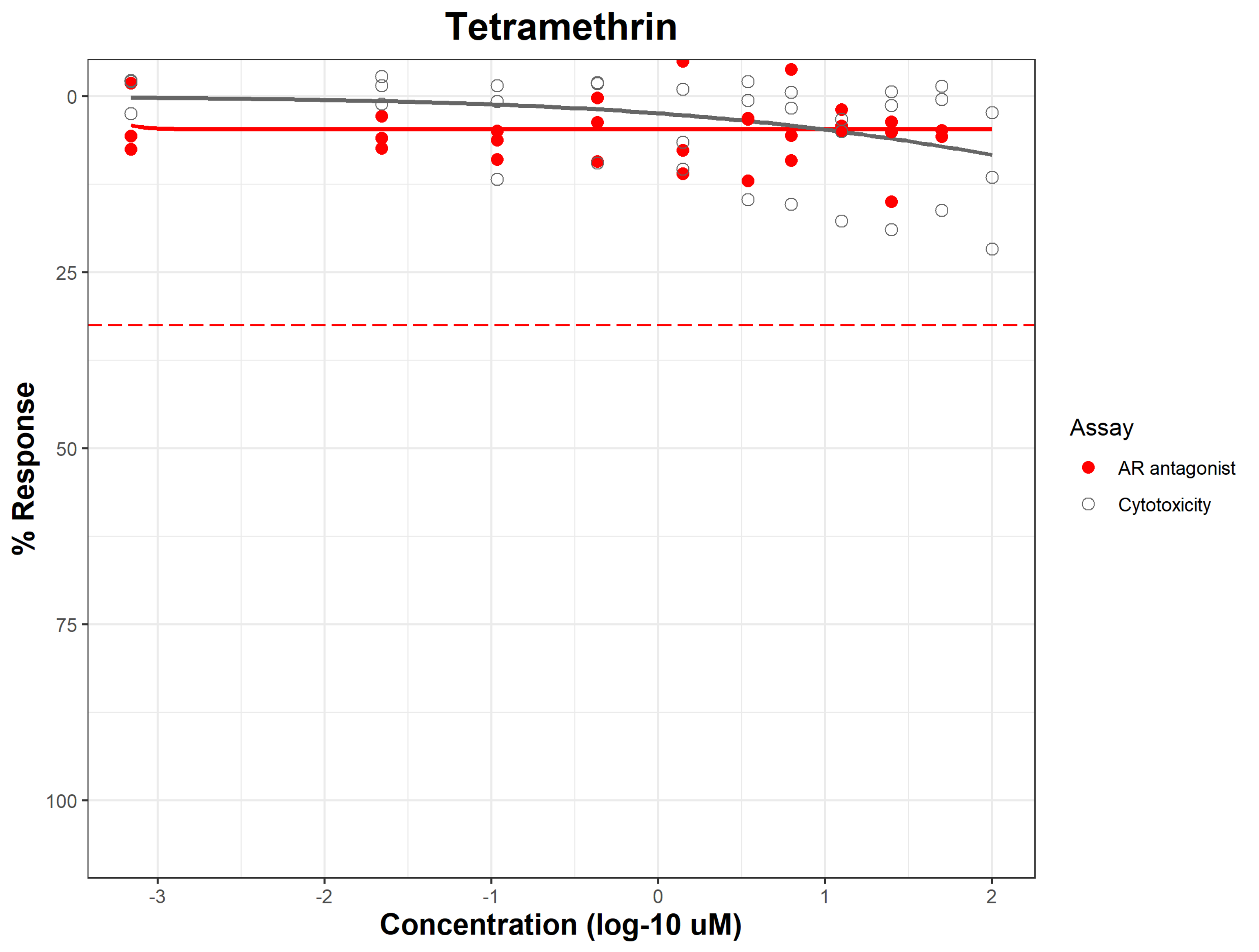

Tetramethrin
% Response
0
25
50
75
100
-3
-2
-1
0
1
2
Concentration (log-10 uM)
Assay
AR antagonist
Cytotoxicity

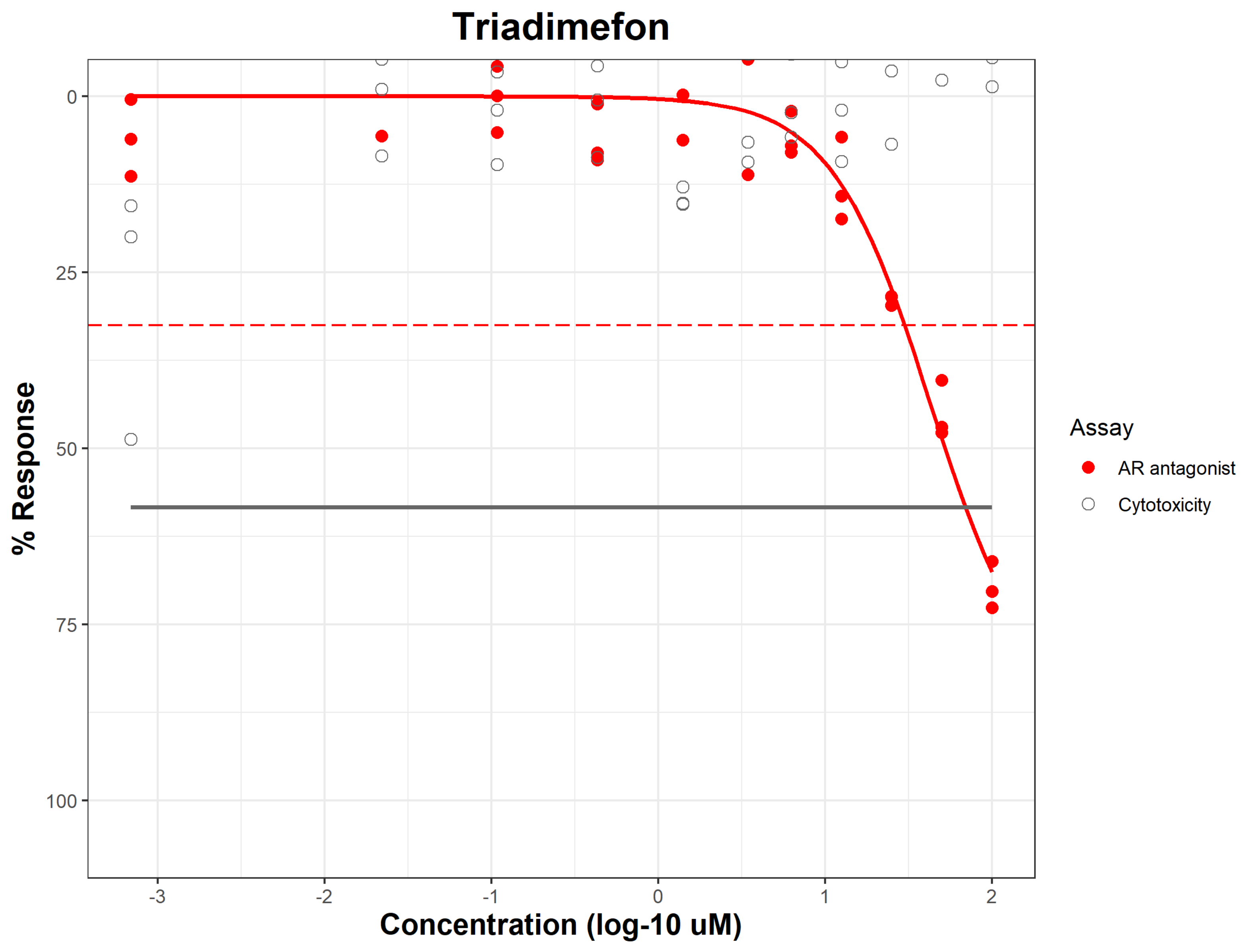

Triadimefon
% Response
0
25
50
75
100
Concentration (log-10 uM)
-3
-2
-1
0
1
2
Assay
AR antagonist
Cytotoxicity

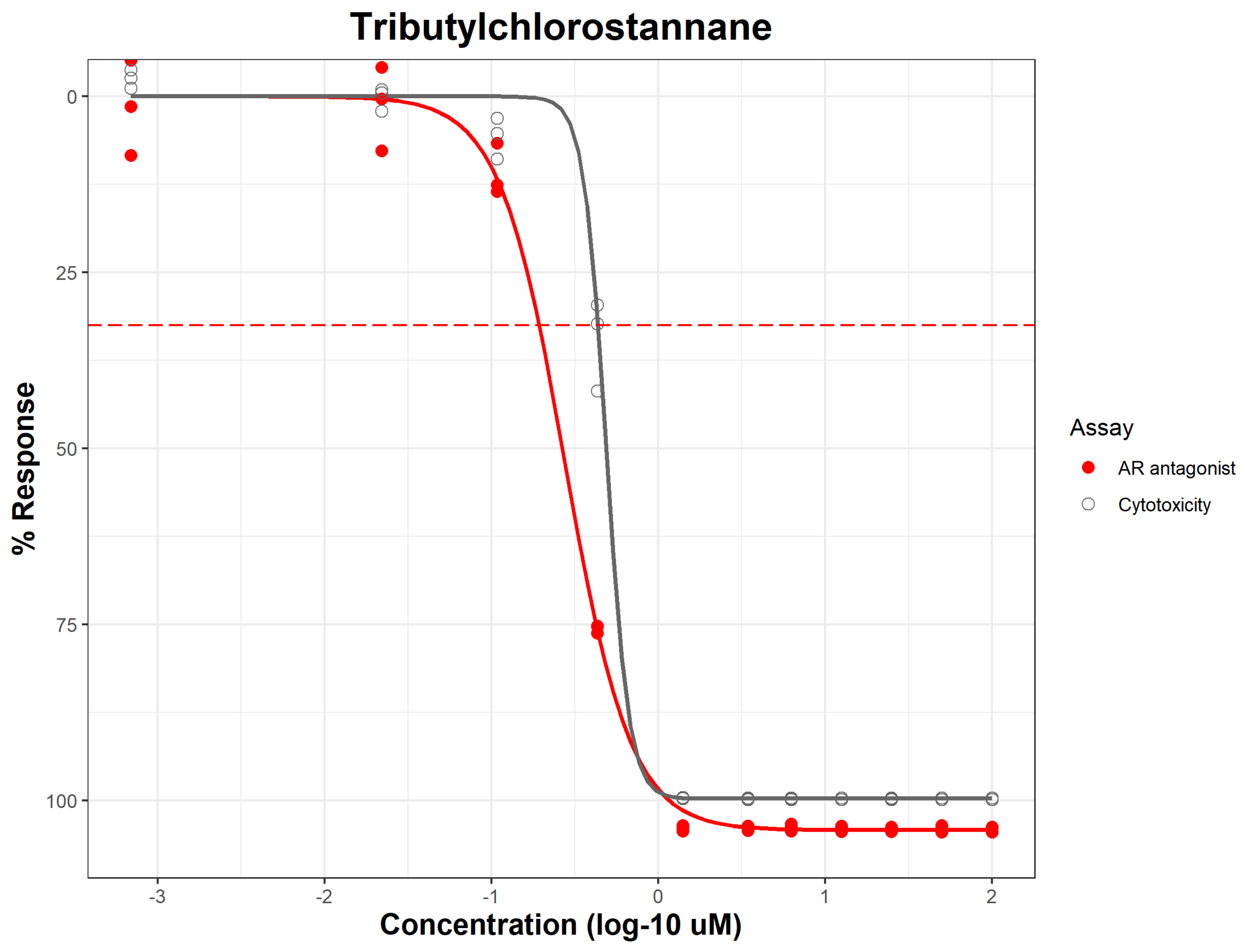

Tributylchlorostannane
% Response
0
25
50
75
100
-3
-2
-1
0
1
2
Concentration (log-10 uM)
Assay
AR antagonist
Cytotoxicity

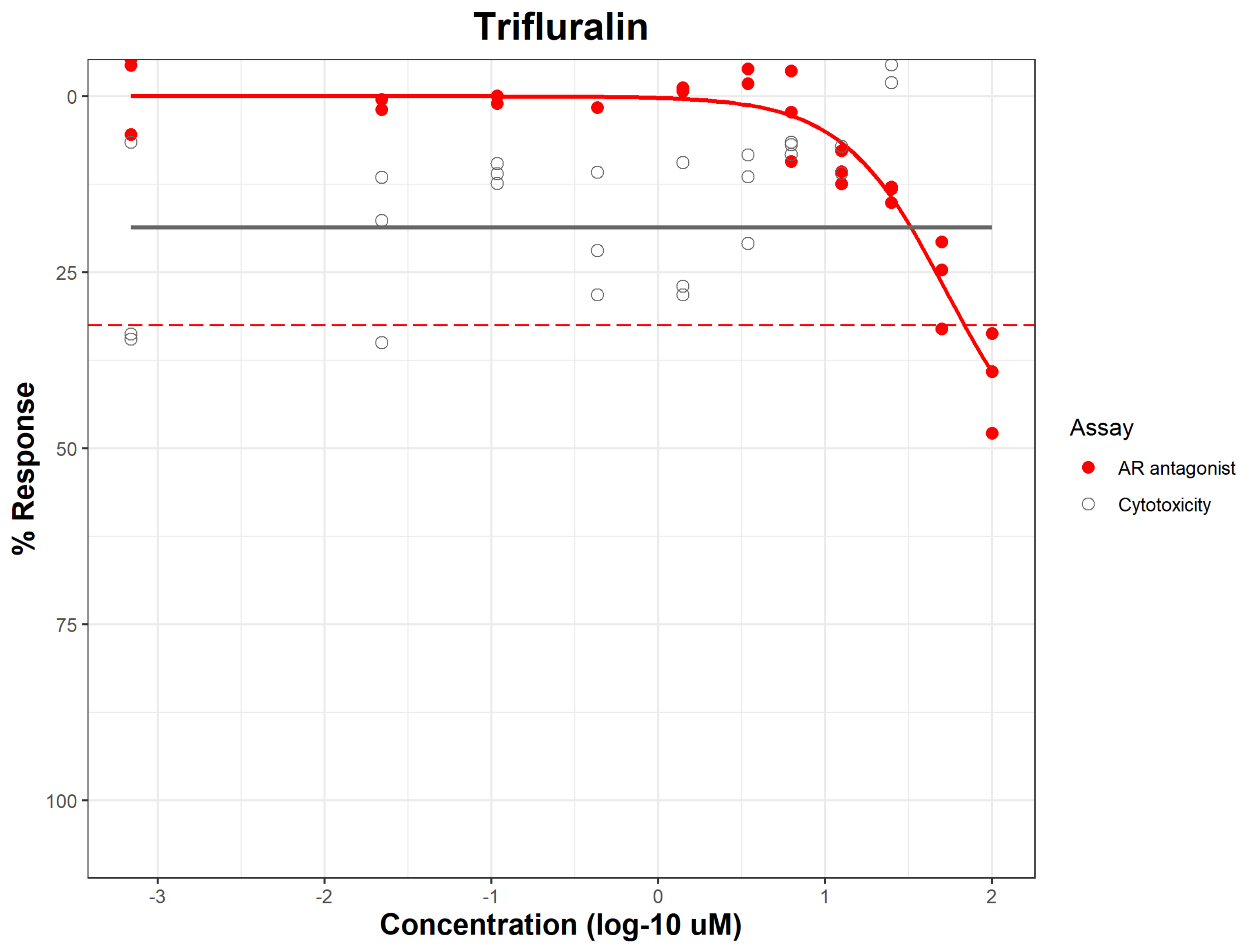

Trifluralin
% Response
0
25
50
75
100
Concentration (log-10 uM)
-3
-2
-1
0
1
2
Assay
AR antagonist
Cytotoxicity

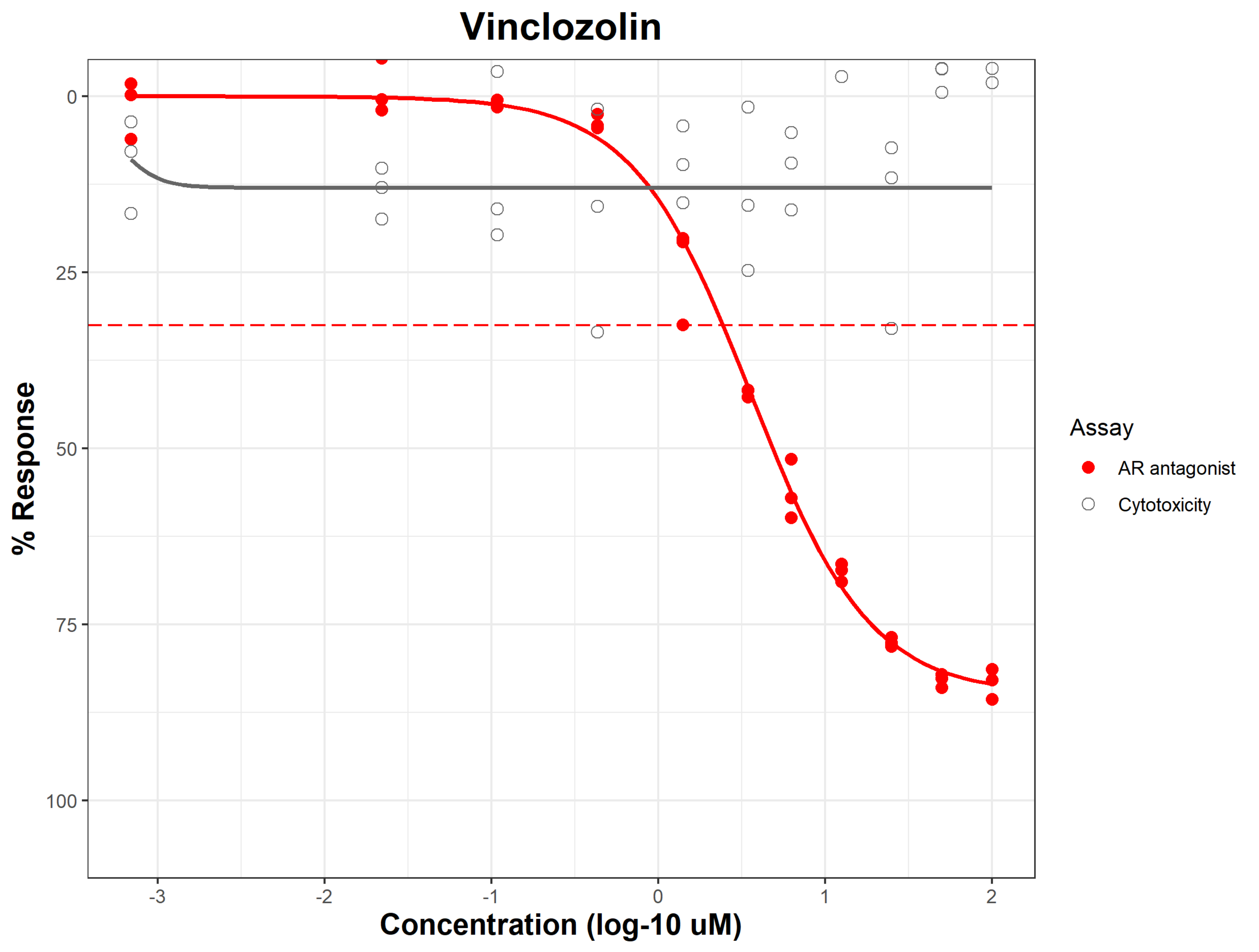

Vinclozolin
% Response
0
25
50
75
100
-3
-2
-1
0
1
2
Concentration (log-10 uM)
Assay
AR antagonist
Cytotoxicity

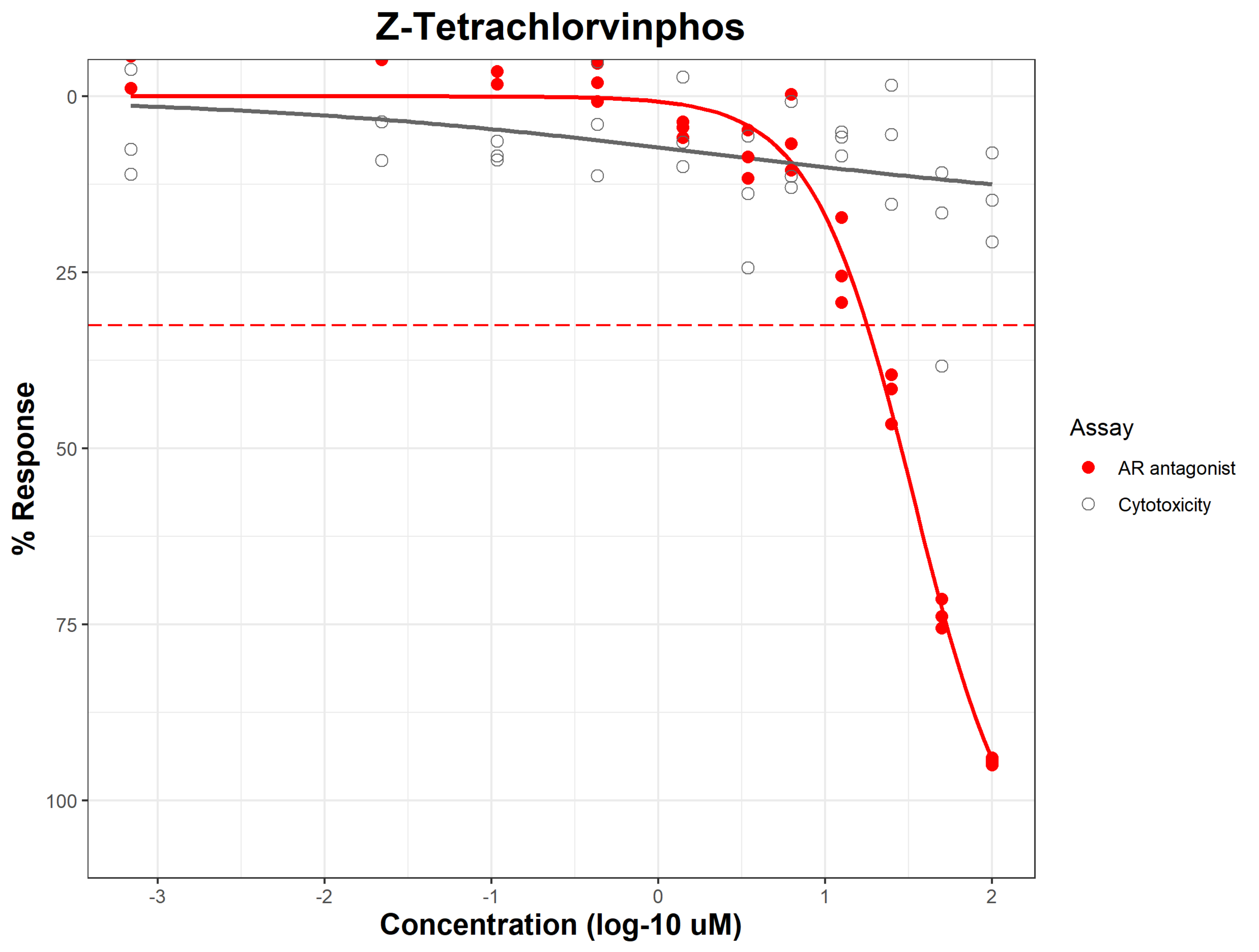

Z-Tetrachlorvinphos
% Response
0
25
50
75
100
-3
-2
-1
0
1
2
Concentration (log-10 uM)
Assay
AR antagonist
Cytotoxicity

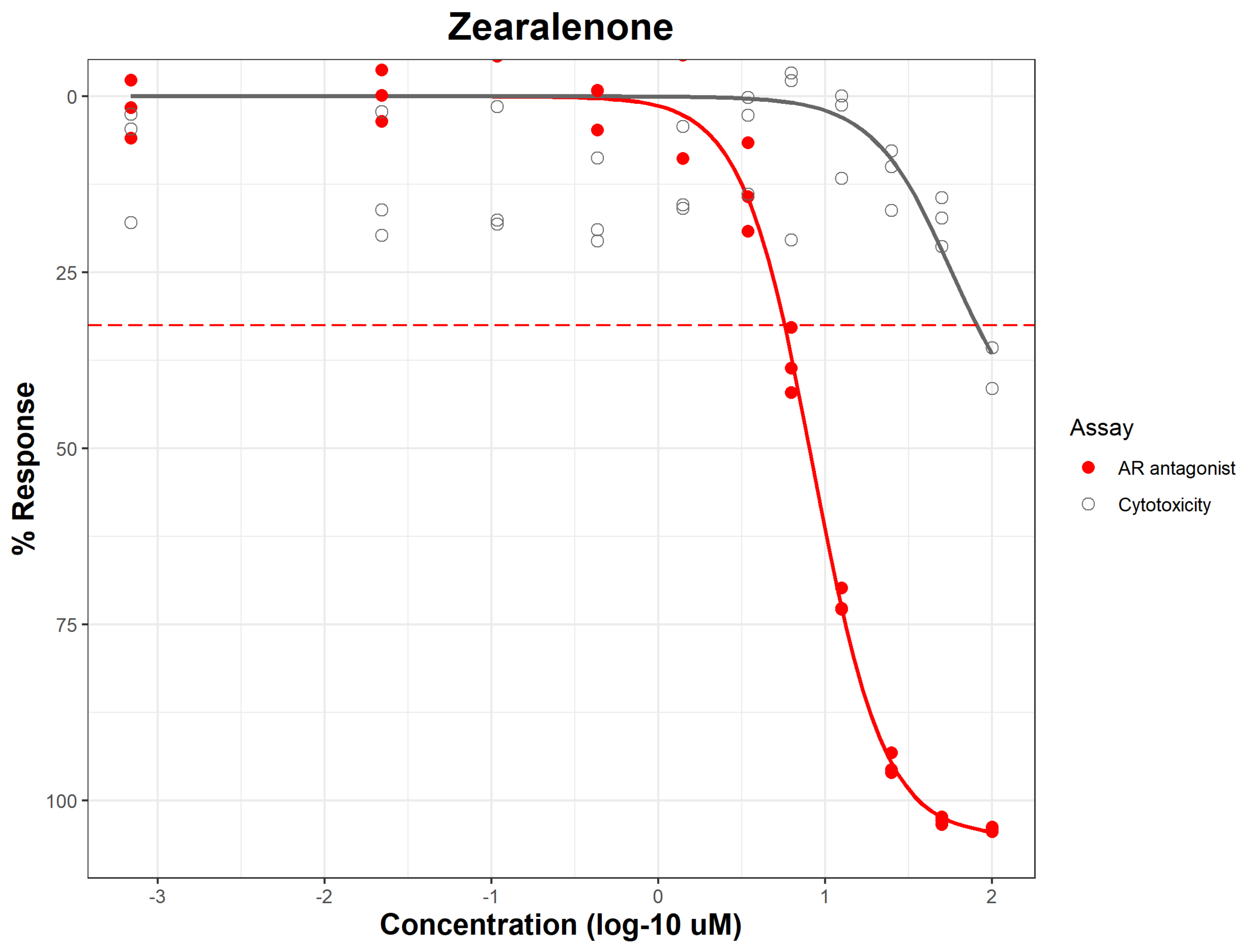

Zearalenone
% Response
0
25
50
75
100
-3
-2
-1
0
1
2
Concentration (log-10 uM)
Assay
AR antagonist
Cytotoxicity

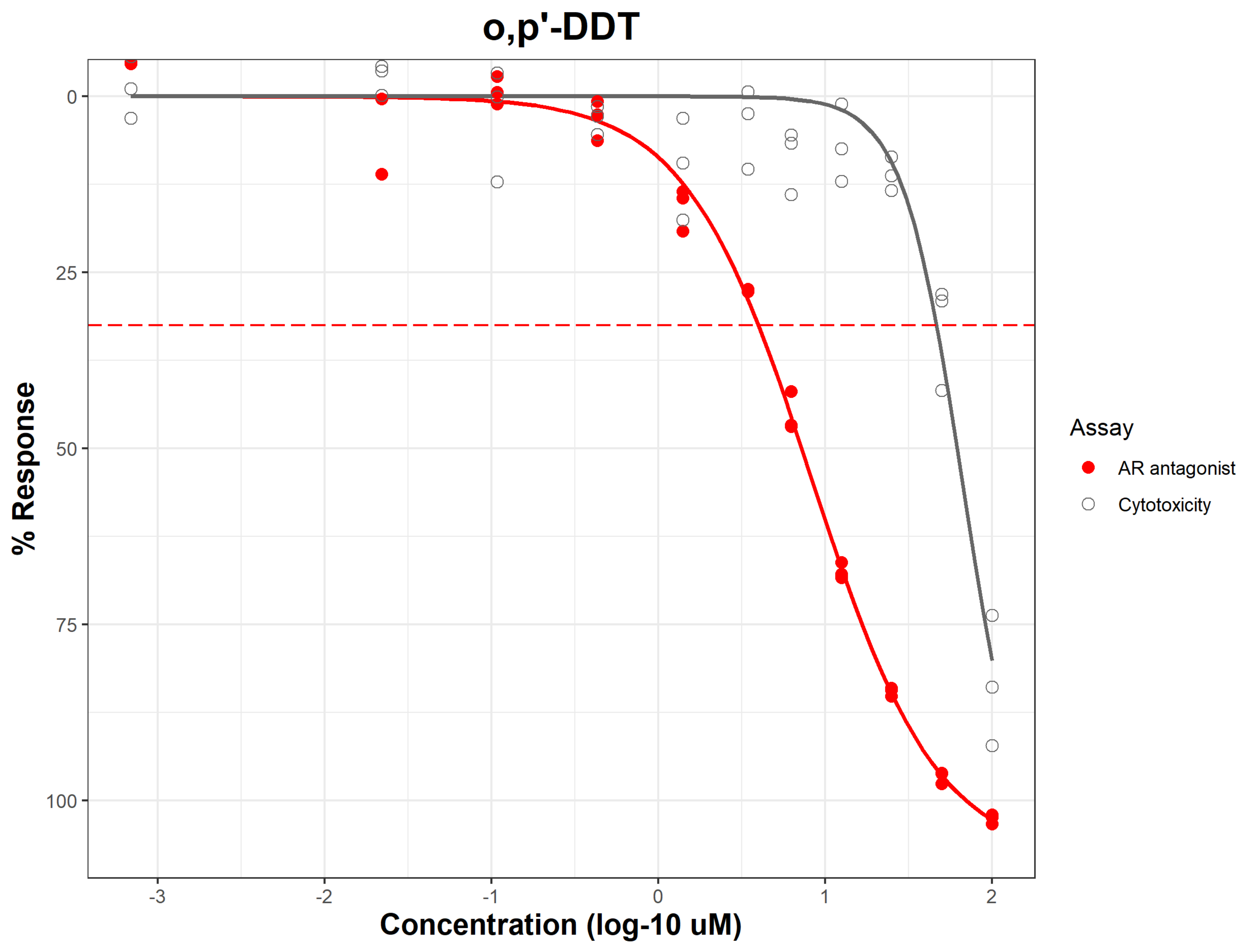

o,p'-DDT
% Response
0
25
50
75
100
-3
-2
-1
0
1
2
Concentration (log-10 uM)
Assay
AR antagonist
Cytotoxicity

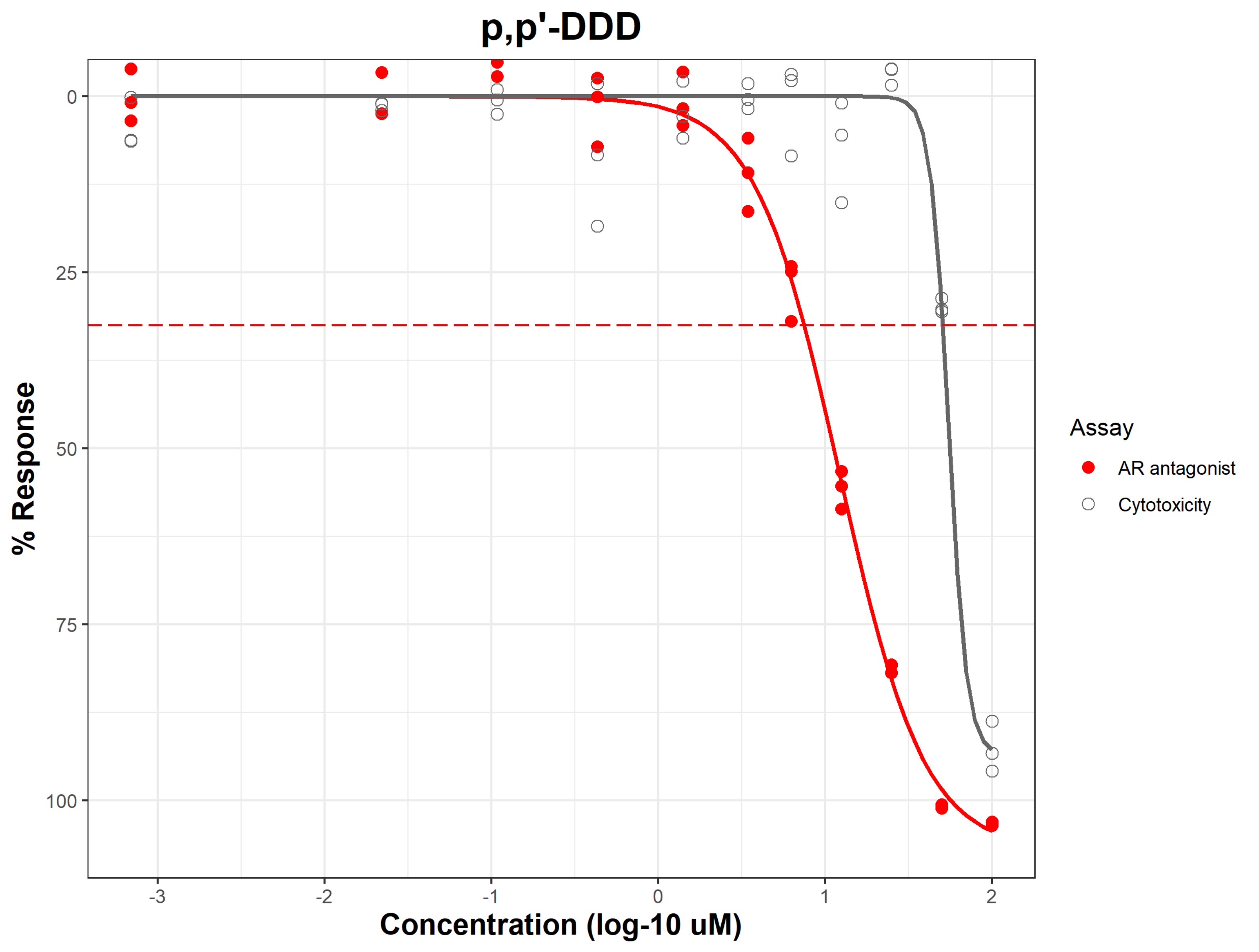

p,p'-DDD
% Response
0
25
50
75
100
-3
-2
-1
0
1
2
Concentration (log-10 uM)
Assay
AR antagonist
Cytotoxicity

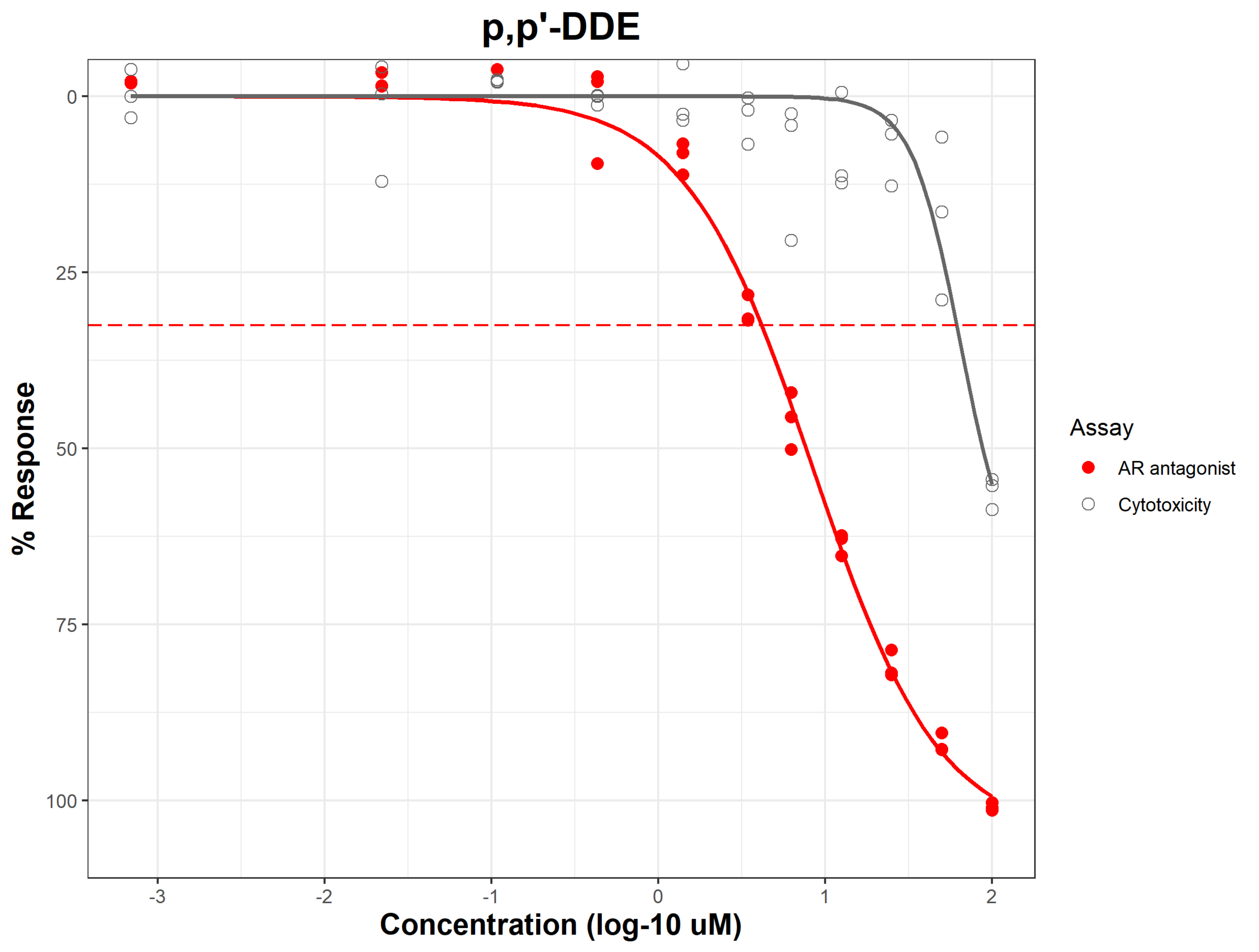
p,p'-DDE
% Response
0
25
50
75
100
-3
-2
-1
0
1
2
Concentration (log-10 uM)
Assay
AR antagonist
Cytotoxicity

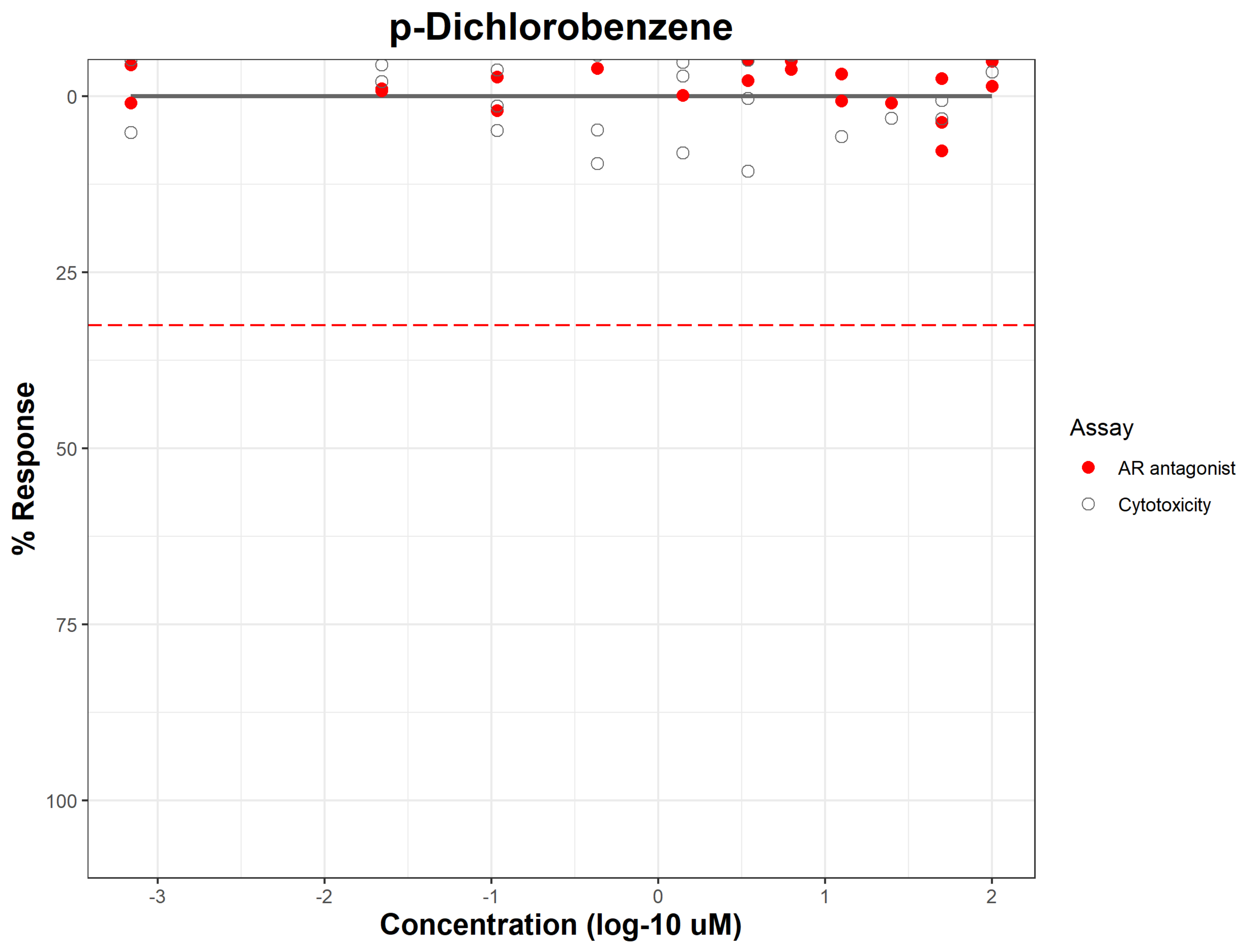

p-Dichlorobenzene
% Response
Concentration (log-10 uM)
0
25
50
75
100
-3
-2
-1
0
1
2
Assay
AR antagonist
Cytotoxicity
