# Supplementary material for: High-throughput AR dimerization assay identifies androgen disrupting chemicals and metabolites
Source: Front Toxicol. 2023 Apr 4;5:1134783. doi: 10.3389/ftox.2023.1134783 (PMC10112521; doi:10.3389/ftox.2023.1134783)
Supplement: Supplementary file 3 [file DataSheet4.PDF]

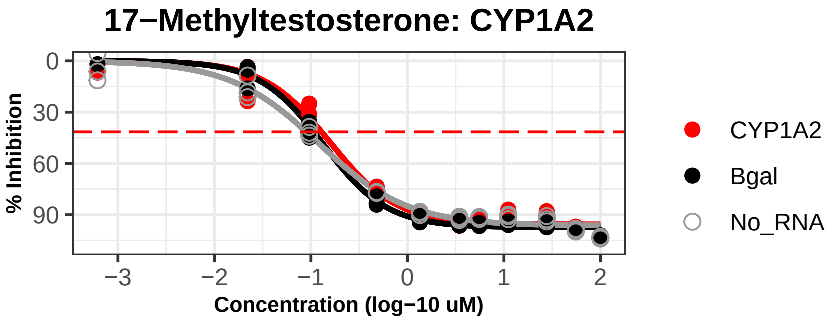
17−Methyltestosterone: CYP1A2
% Inhibition
Concentration (log−10 uM)
CYP1A2
Bgal
No_RNA

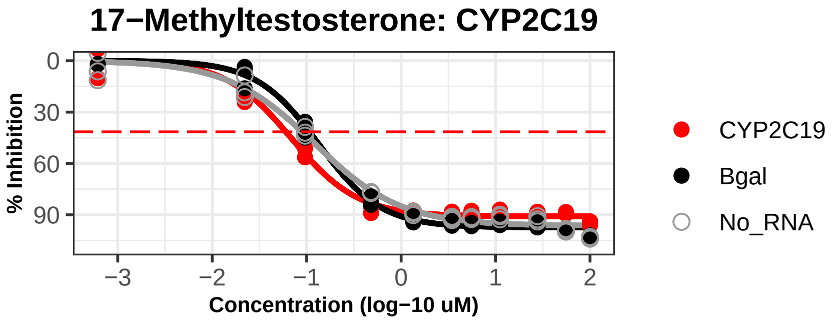
17−Methyltestosterone: CYP2C19
% Inhibition
Concentration (log−10 uM)
CYP2C19
Bgal
No_RNA

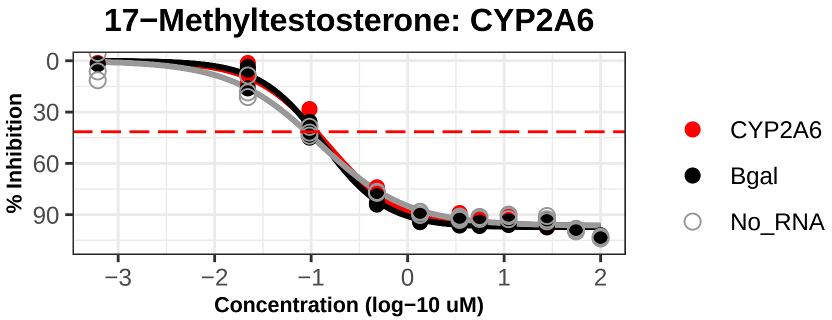
17−Methyltestosterone: CYP2A6
% Inhibition
Concentration (log−10 uM)
CYP2A6
Bgal
No_RNA

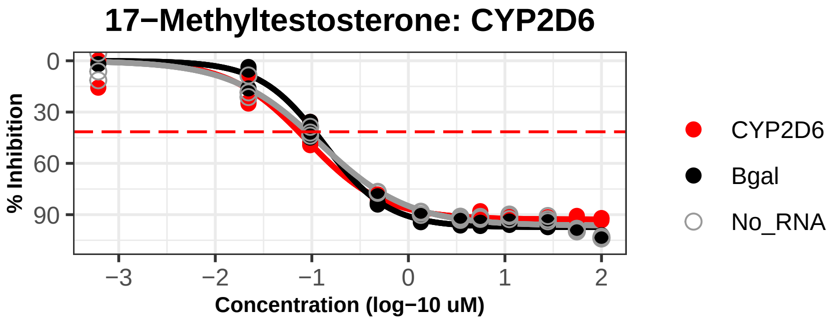
17−Methyltestosterone: CYP2D6
% Inhibition
Concentration (log−10 uM)
CYP2D6
Bgal
No_RNA

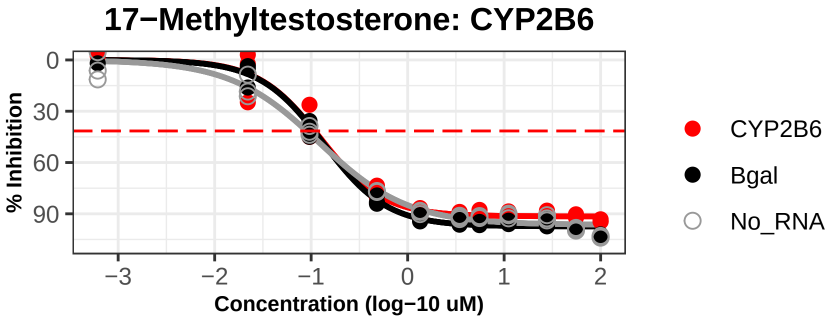
17−Methyltestosterone: CYP2B6
% Inhibition
Concentration (log−10 uM)
CYP2B6
Bgal
No_RNA

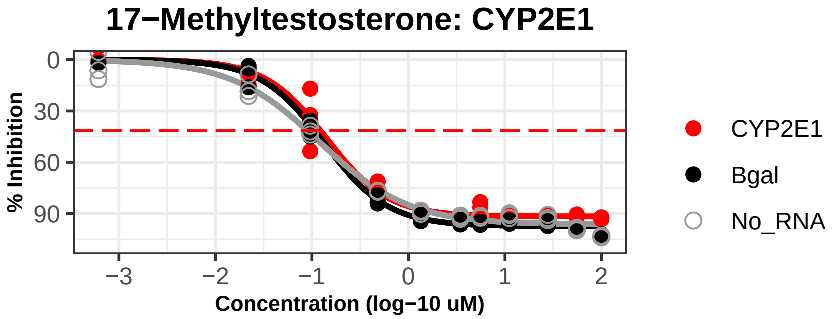
17−Methyltestosterone: CYP2E1
% Inhibition
Concentration (log−10 uM)
CYP2E1
Bgal
No_RNA

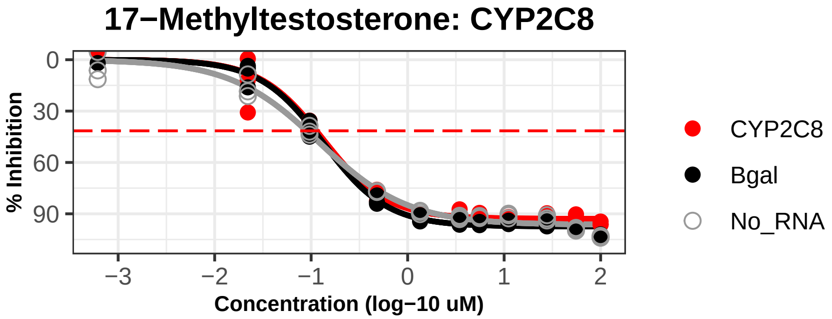
17−Methyltestosterone: CYP2C8
% Inhibition
Concentration (log−10 uM)
CYP2C8
Bgal
No_RNA

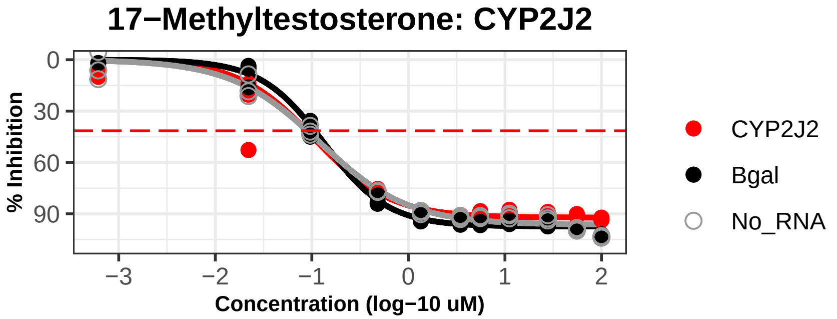
17−Methyltestosterone: CYP2J2
% Inhibition
Concentration (log−10 uM)
CYP2J2
Bgal
No_RNA

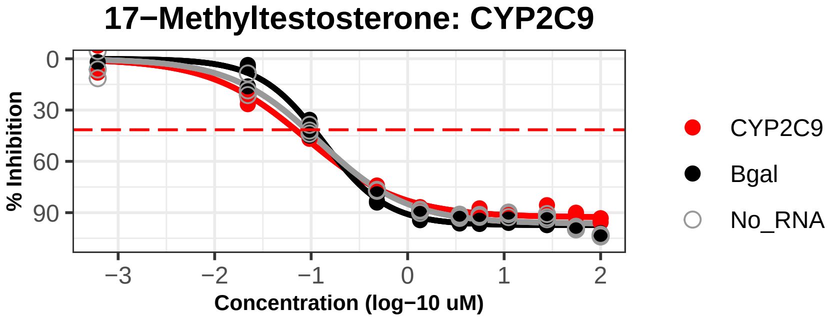
17−Methyltestosterone: CYP2C9
% Inhibition
Concentration (log−10 uM)
CYP2C9
Bgal
No_RNA

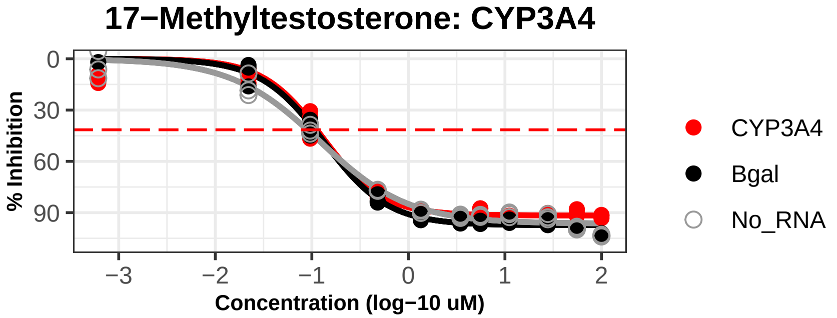
17−Methyltestosterone: CYP3A4
% Inhibition
Concentration (log−10 uM)
CYP3A4
Bgal
No_RNA

17alpha−Estradiol: CYP1A2

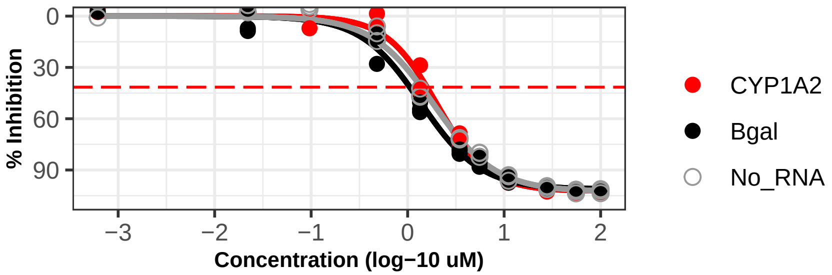

17alpha−Estradiol: CYP2C19

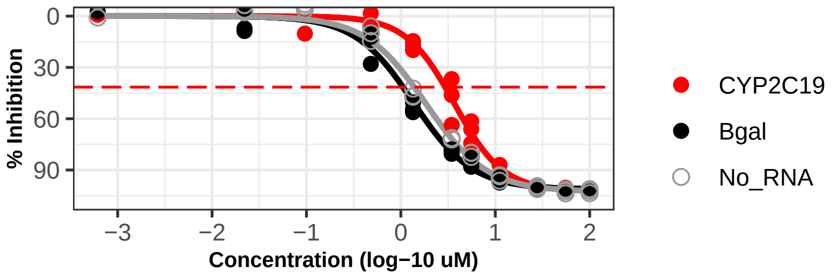

17alpha−Estradiol: CYP2A6

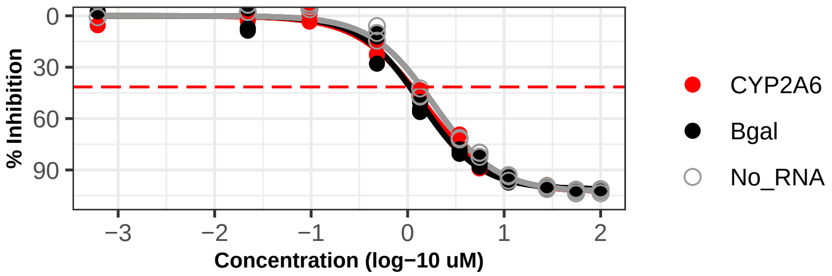

17alpha−Estradiol: CYP2D6

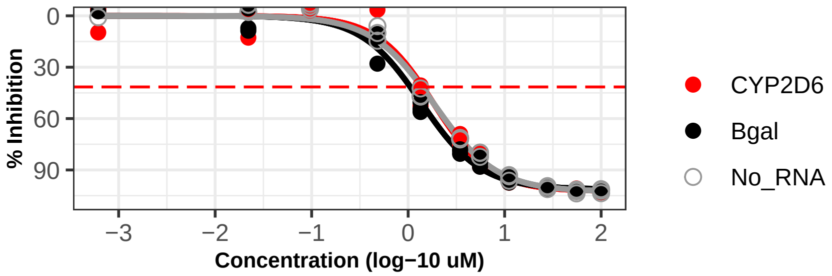

17alpha−Estradiol: CYP2B6

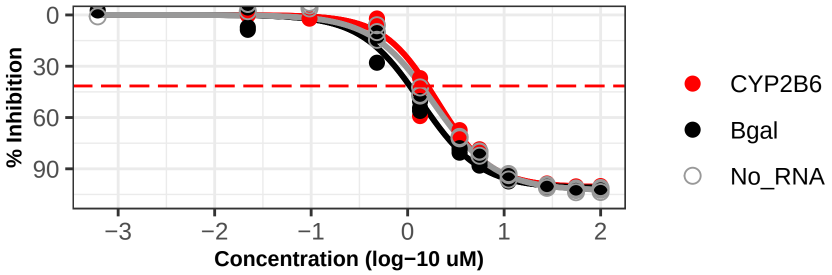

17alpha−Estradiol: CYP2E1

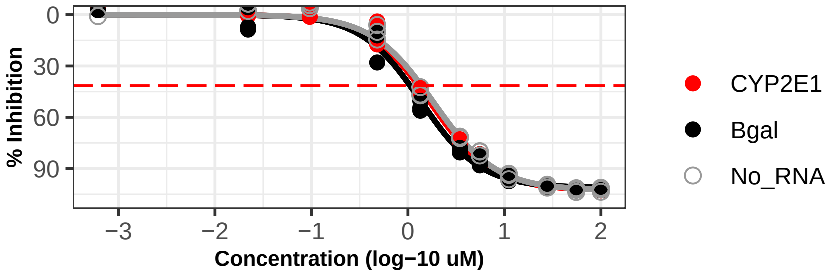

17alpha−Estradiol: CYP2C8

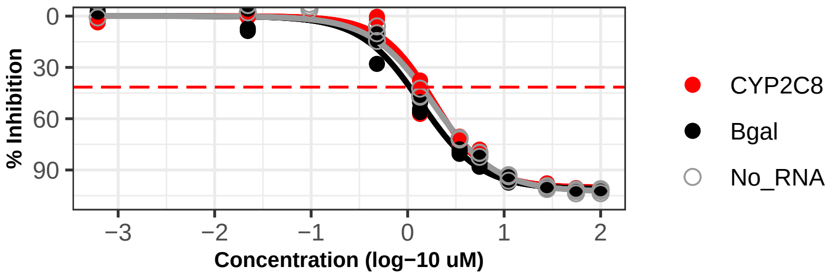

17alpha−Estradiol: CYP2J2

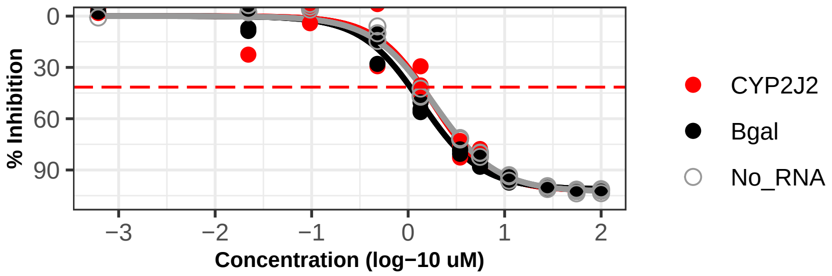

17alpha−Estradiol: CYP2C9

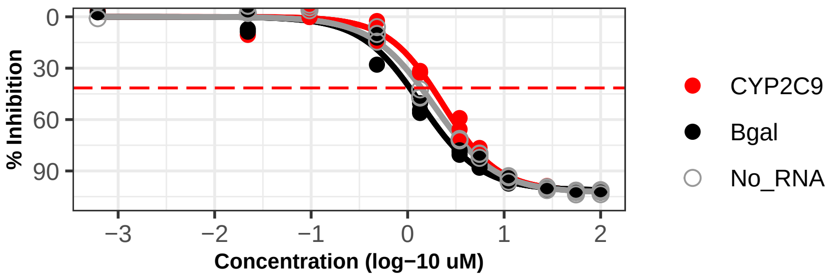

17alpha−Estradiol: CYP3A4

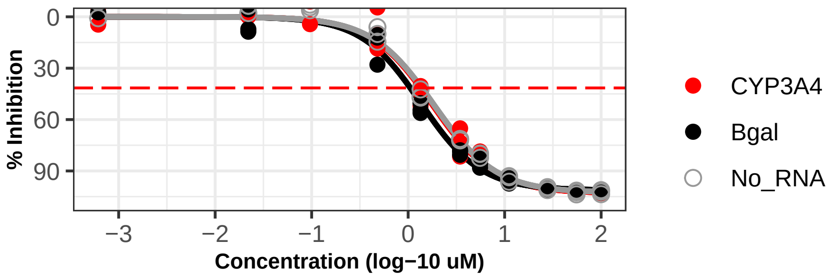

17alpha−Ethinylestradiol: CYP1A2

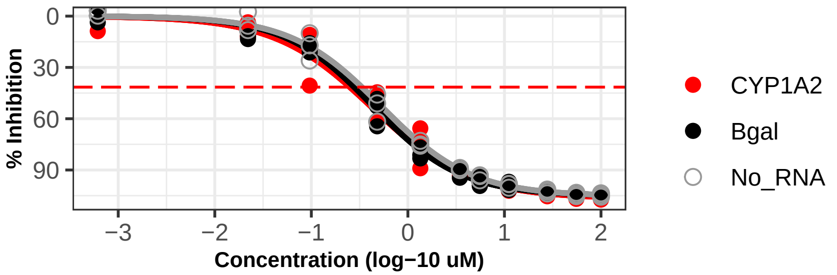

17alpha−Ethinylestradiol: CYP2C19

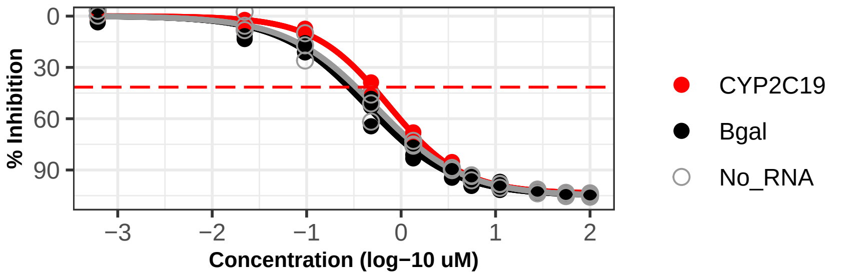

17alpha−Ethinylestradiol: CYP2A6

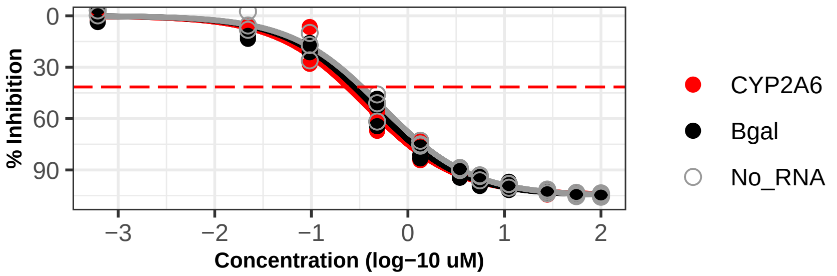

17alpha−Ethinylestradiol: CYP2D6

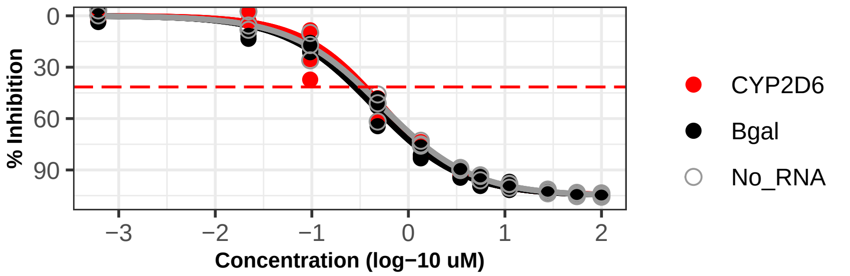

17alpha−Ethinylestradiol: CYP2B6

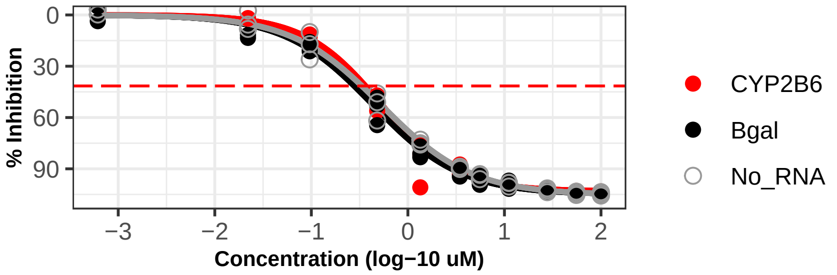

17alpha−Ethinylestradiol: CYP2E1

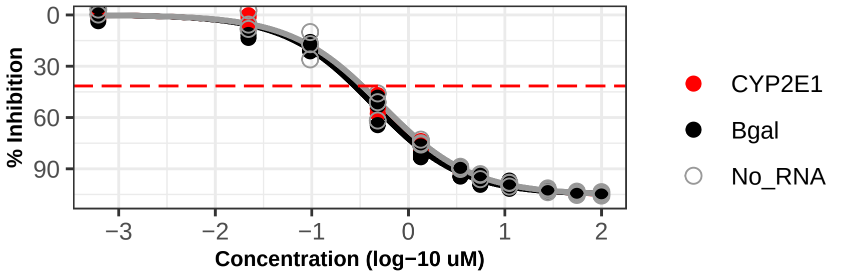

17alpha−Ethinylestradiol: CYP2C8

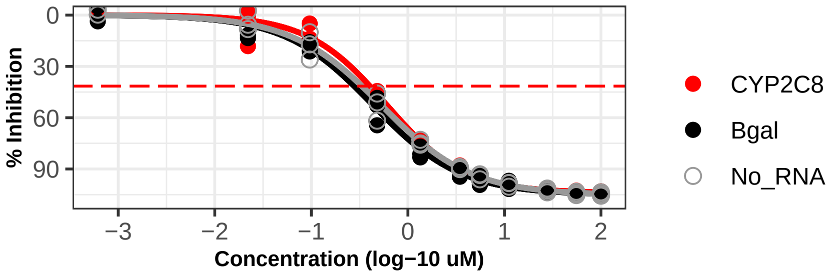

17alpha−Ethinylestradiol: CYP2J2

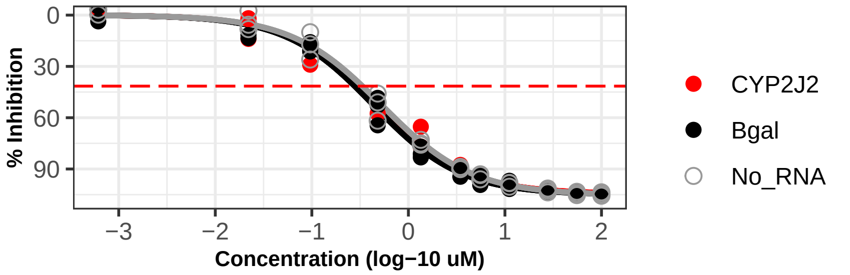

17alpha−Ethinylestradiol: CYP2C9

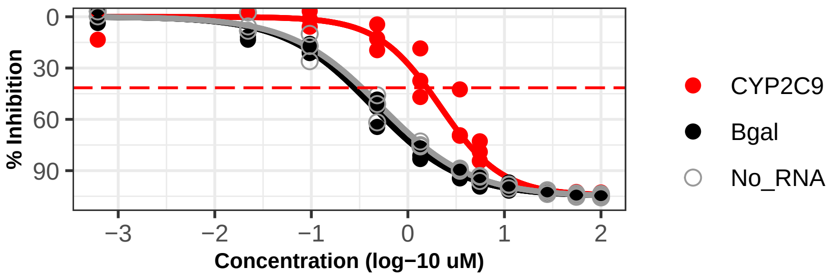

17alpha−Ethinylestradiol: CYP3A4

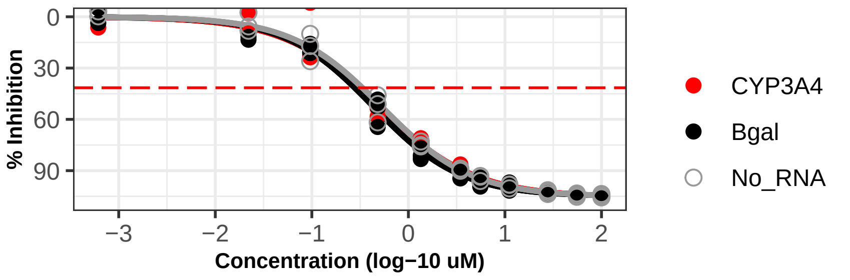

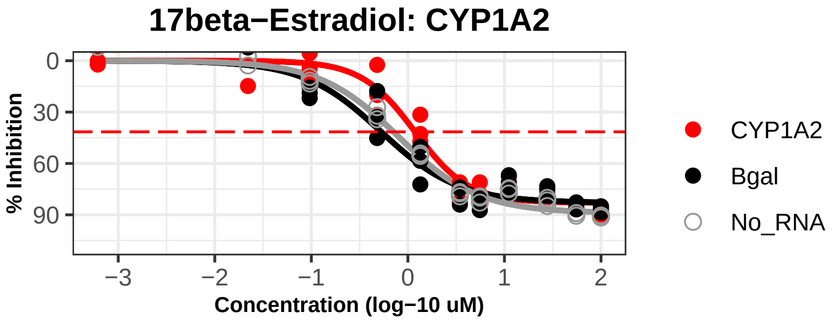
17beta−Estradiol: CYP1A2
% Inhibition
0
30
60
90
−3
−2
−1
0
1
2
Concentration (log−10 uM)
CYP1A2
Bgal
No_RNA

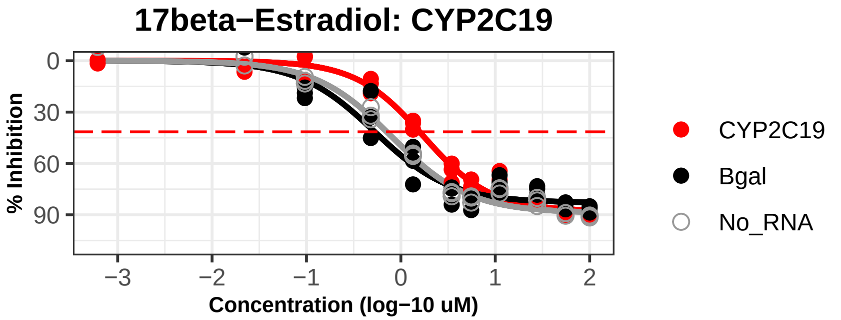
17beta−Estradiol: CYP2C19
% Inhibition
0
30
60
90
−3
−2
−1
0
1
2
Concentration (log−10 uM)
CYP2C19
Bgal
No_RNA

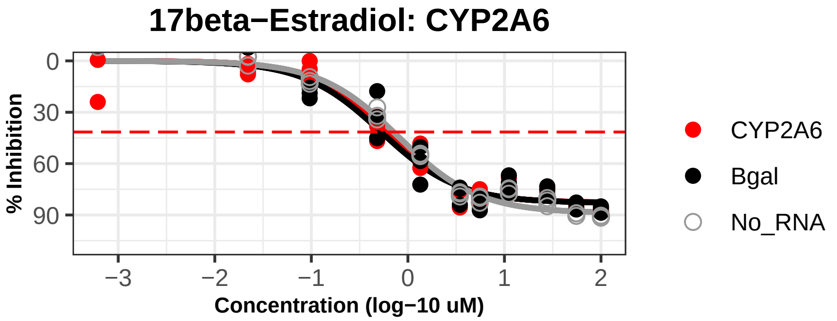
17beta−Estradiol: CYP2A6
% Inhibition
0
30
60
90
−3
−2
−1
0
1
2
Concentration (log−10 uM)
CYP2A6
Bgal
No_RNA

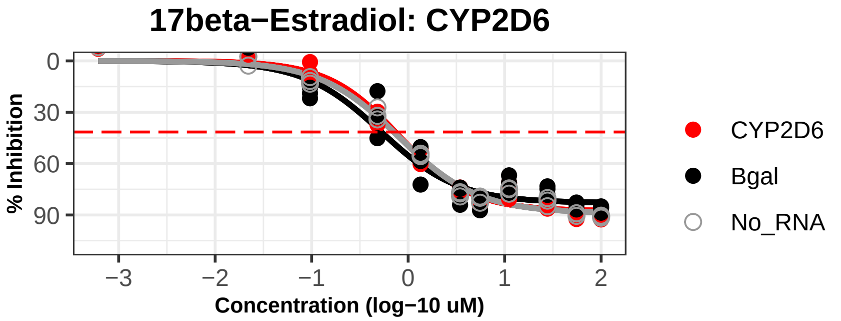
17beta−Estradiol: CYP2D6
% Inhibition
0
30
60
90
−3
−2
−1
0
1
2
Concentration (log−10 uM)
CYP2D6
Bgal
No_RNA

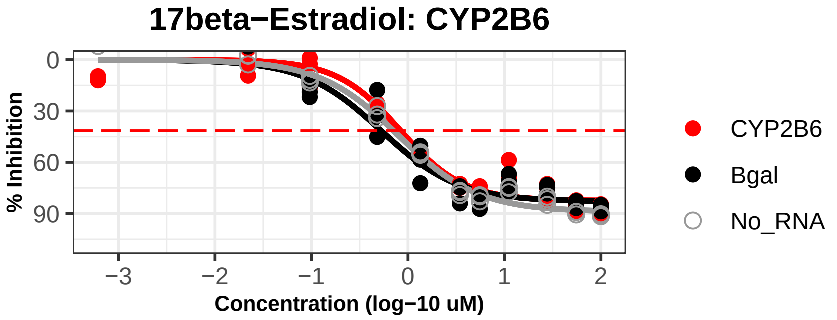
17beta−Estradiol: CYP2B6
% Inhibition
0
30
60
90
−3
−2
−1
0
1
2
Concentration (log−10 uM)
CYP2B6
Bgal
No_RNA

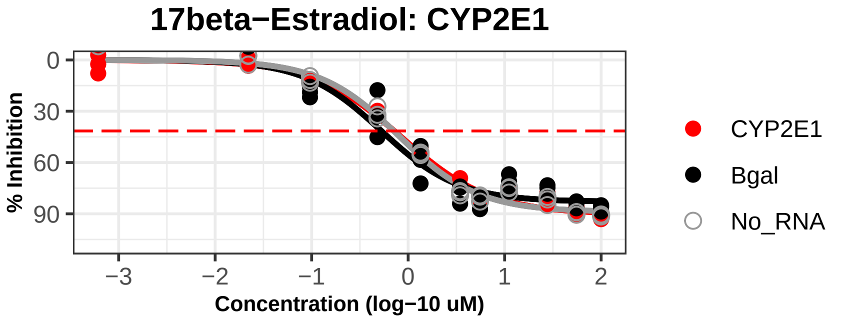
17beta−Estradiol: CYP2E1
% Inhibition
0
30
60
90
−3
−2
−1
0
1
2
Concentration (log−10 uM)
CYP2E1
Bgal
No_RNA

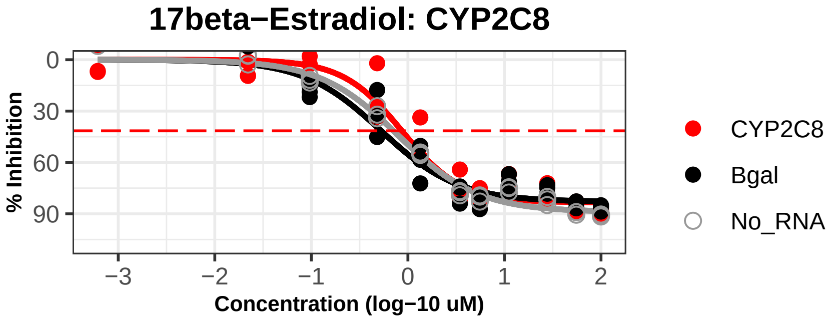
17beta−Estradiol: CYP2C8
% Inhibition
0
30
60
90
−3
−2
−1
0
1
2
Concentration (log−10 uM)
CYP2C8
Bgal
No_RNA

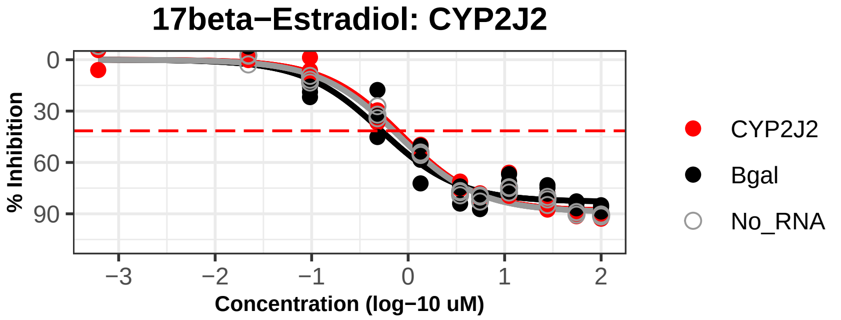
17beta−Estradiol: CYP2J2
% Inhibition
0
30
60
90
−3
−2
−1
0
1
2
Concentration (log−10 uM)
CYP2J2
Bgal
No_RNA

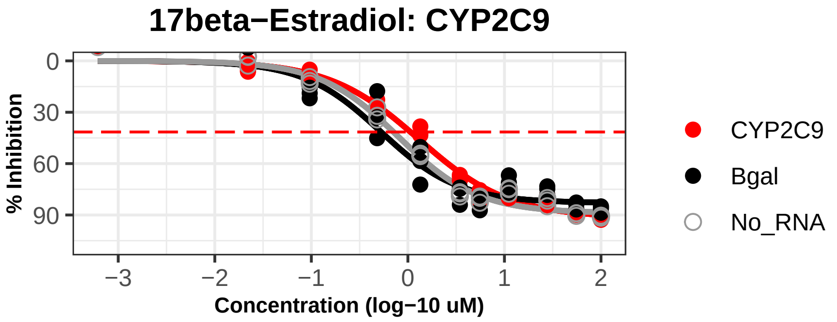
17beta−Estradiol: CYP2C9
% Inhibition
0
30
60
90
−3
−2
−1
0
1
2
Concentration (log−10 uM)
CYP2C9
Bgal
No_RNA

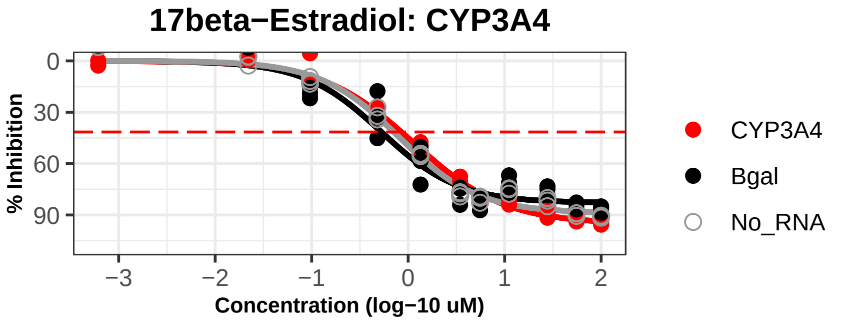
17beta−Estradiol: CYP3A4
% Inhibition
0
30
60
90
−3
−2
−1
0
1
2
Concentration (log−10 uM)
CYP3A4
Bgal
No_RNA

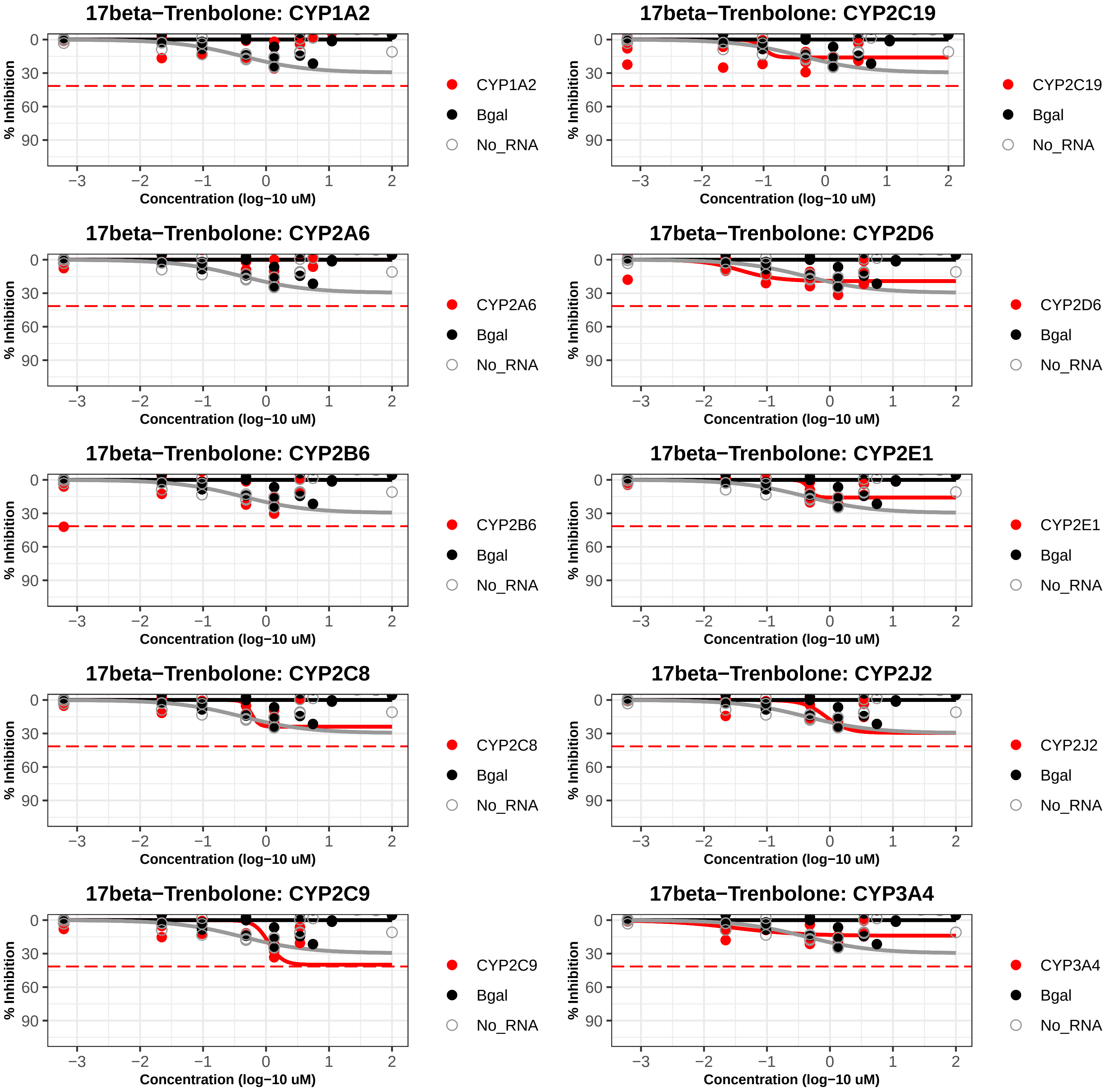
17beta−Trenbolone: CYP1A2
CYP1A2
Bgal
No_RNA
17beta−Trenbolone: CYP2C19
CYP2C19
Bgal
No_RNA
17beta−Trenbolone: CYP2A6
CYP2A6
Bgal
No_RNA
17beta−Trenbolone: CYP2D6
CYP2D6
Bgal
No_RNA
17beta−Trenbolone: CYP2B6
CYP2B6
Bgal
No_RNA
17beta−Trenbolone: CYP2E1
CYP2E1
Bgal
No_RNA
17beta−Trenbolone: CYP2C8
CYP2C8
Bgal
No_RNA
17beta−Trenbolone: CYP2J2
CYP2J2
Bgal
No_RNA
17beta−Trenbolone: CYP2C9
CYP2C9
Bgal
No_RNA
17beta−Trenbolone: CYP3A4
CYP3A4
Bgal
No_RNA
% Inhibition
0
30
60
90
Concentration (log−10 uM)
−3
−2
−1
0
1
2

2,2',4,4'−Tetrahydroxybenzophenone: CYP1A2

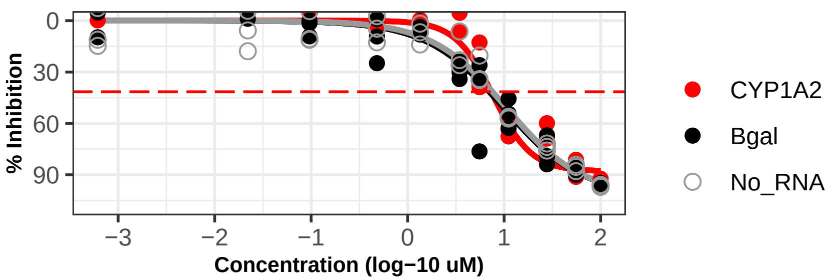

2,2',4,4'−Tetrahydroxybenzophenone: CYP2C19

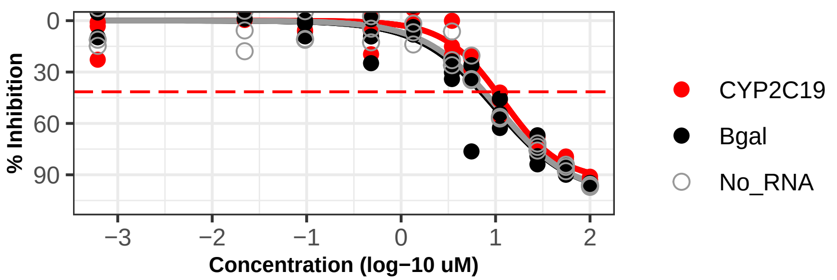

2,2',4,4'−Tetrahydroxybenzophenone: CYP2A6

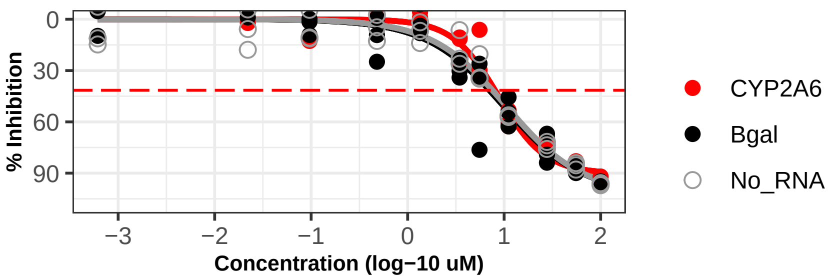

2,2',4,4'−Tetrahydroxybenzophenone: CYP2D6

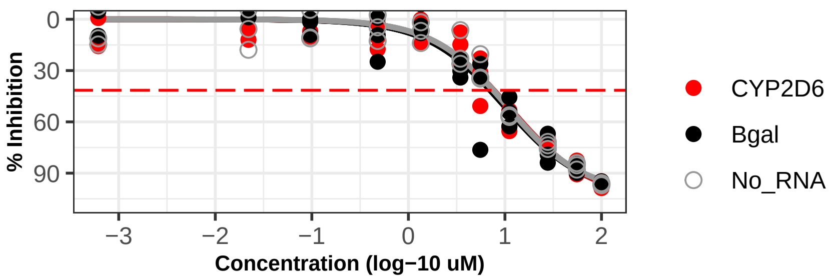

2,2',4,4'−Tetrahydroxybenzophenone: CYP2B6

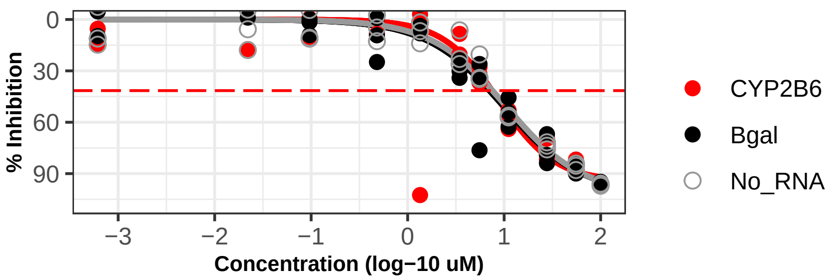

2,2',4,4'−Tetrahydroxybenzophenone: CYP2E1

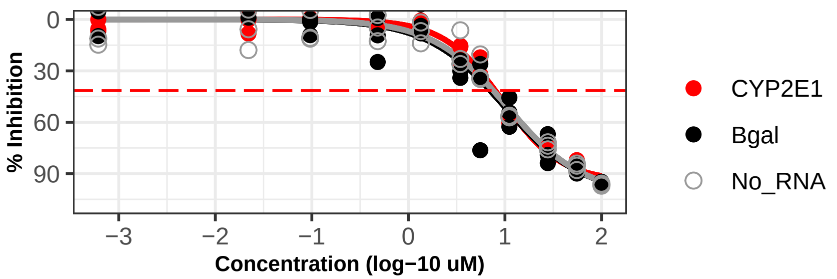

2,2',4,4'−Tetrahydroxybenzophenone: CYP2C8

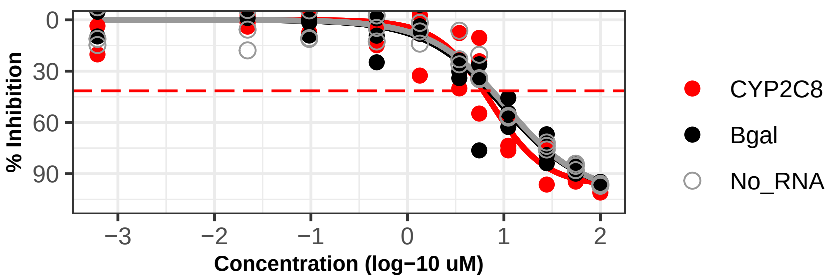

2,2',4,4'−Tetrahydroxybenzophenone: CYP2J2

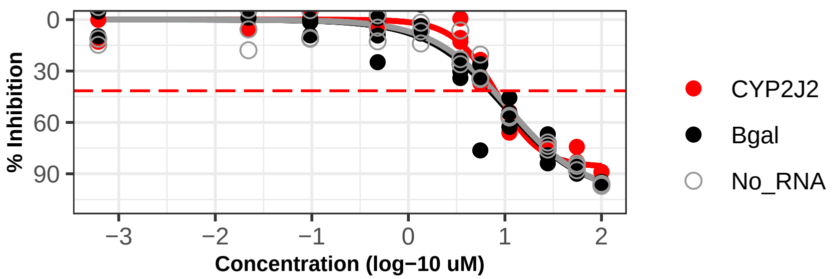

2,2',4,4'−Tetrahydroxybenzophenone: CYP2C9

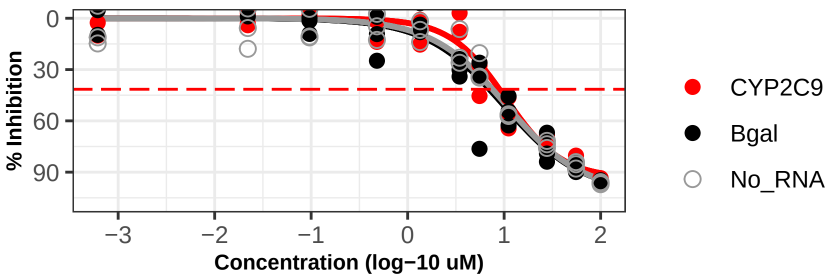

2,2',4,4'−Tetrahydroxybenzophenone: CYP3A4

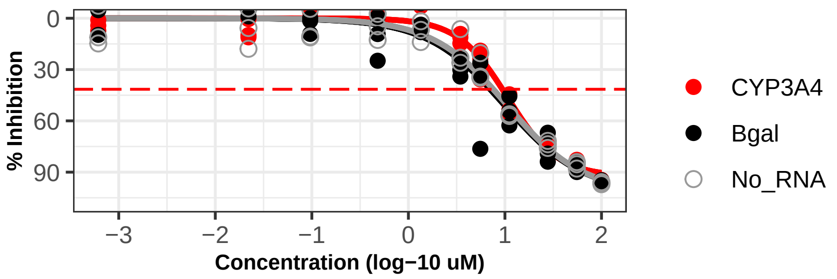

2,4−Dihydroxybenzophenone: CYP1A2

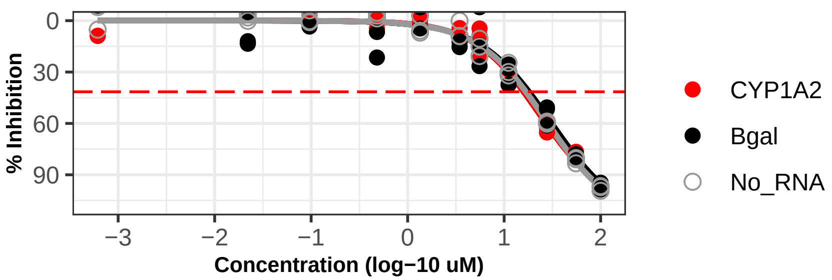

2,4−Dihydroxybenzophenone: CYP2C19

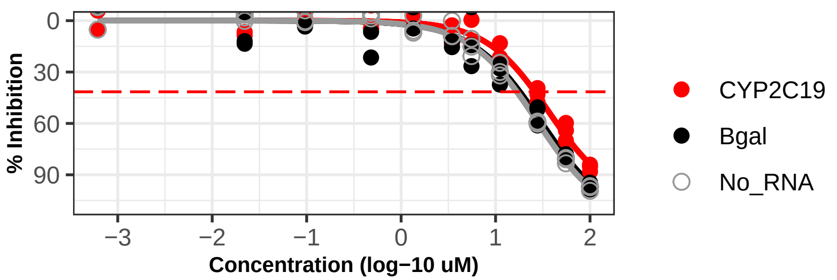

2,4−Dihydroxybenzophenone: CYP2A6

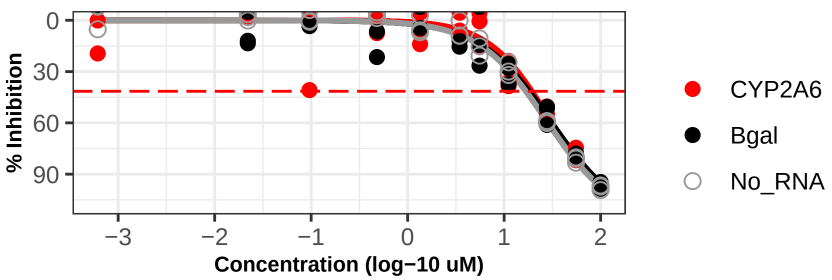

2,4−Dihydroxybenzophenone: CYP2D6

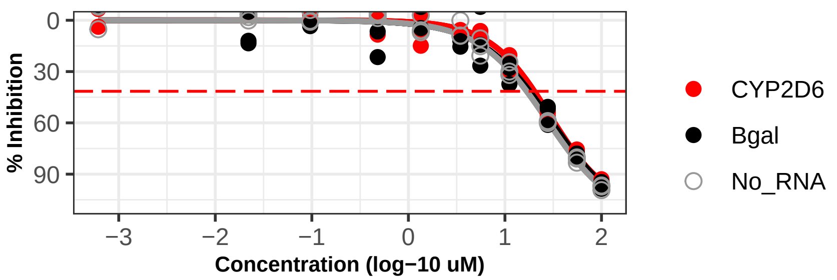

2,4−Dihydroxybenzophenone: CYP2B6

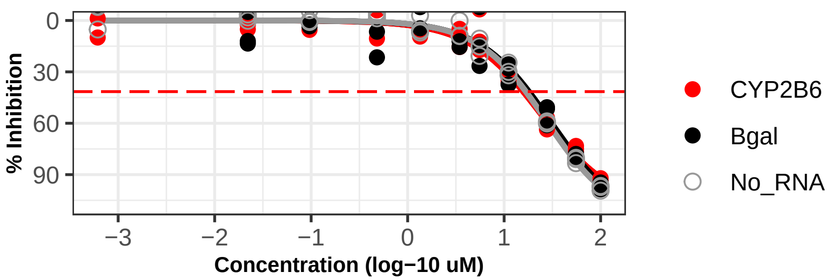

2,4−Dihydroxybenzophenone: CYP2E1

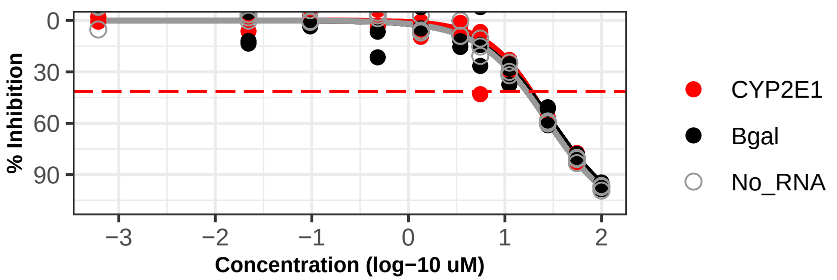

2,4−Dihydroxybenzophenone: CYP2C8

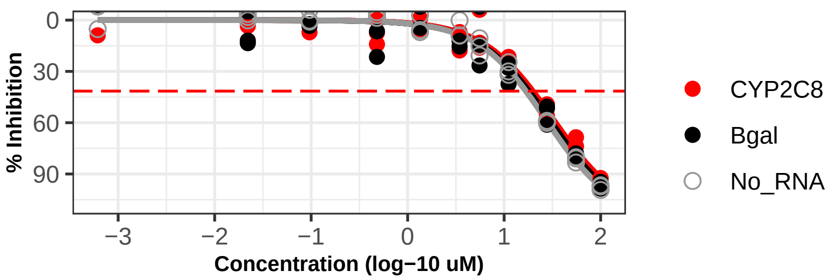

2,4−Dihydroxybenzophenone: CYP2J2

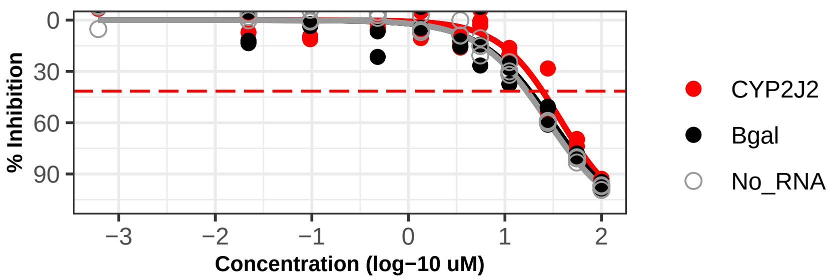

2,4−Dihydroxybenzophenone: CYP2C9

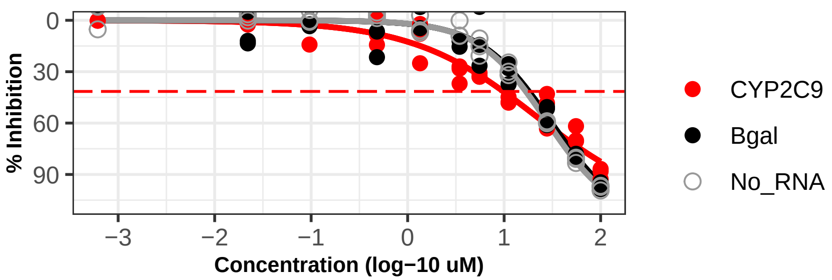

2,4−Dihydroxybenzophenone: CYP3A4

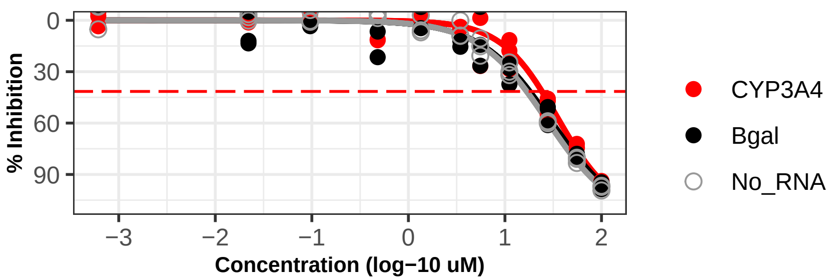

2,4−Dinitrophenol: CYP1A2

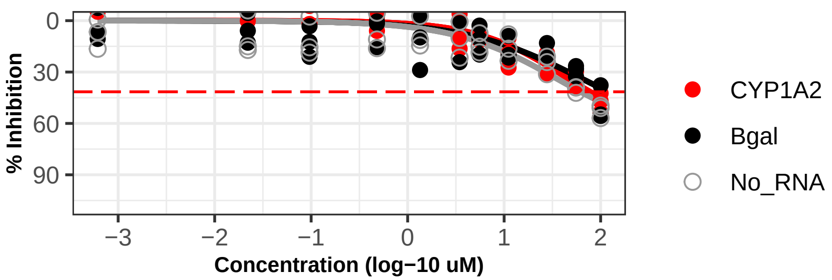

2,4−Dinitrophenol: CYP2C19

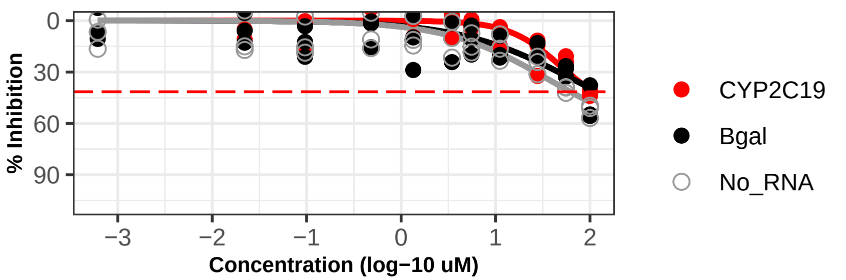

2,4−Dinitrophenol: CYP2A6

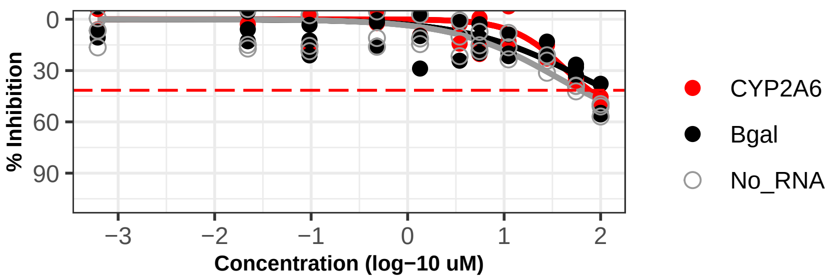

2,4−Dinitrophenol: CYP2D6

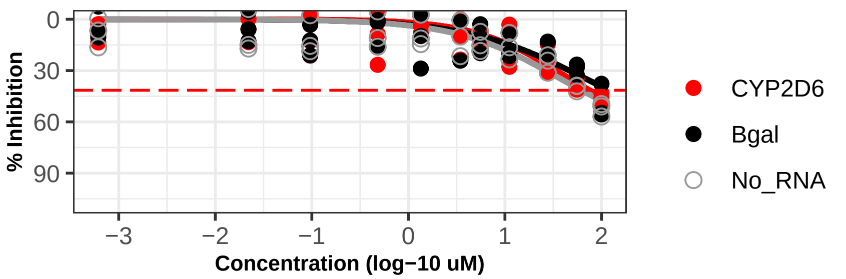

2,4−Dinitrophenol: CYP2B6

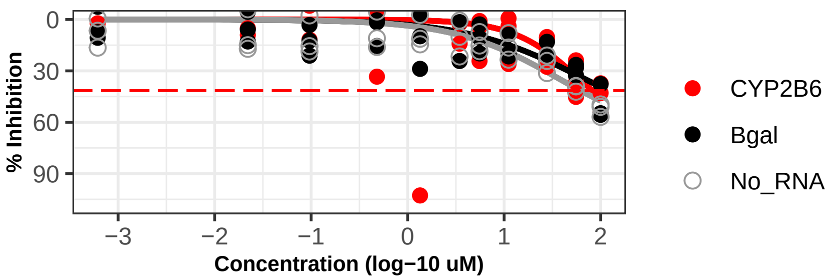

2,4−Dinitrophenol: CYP2E1

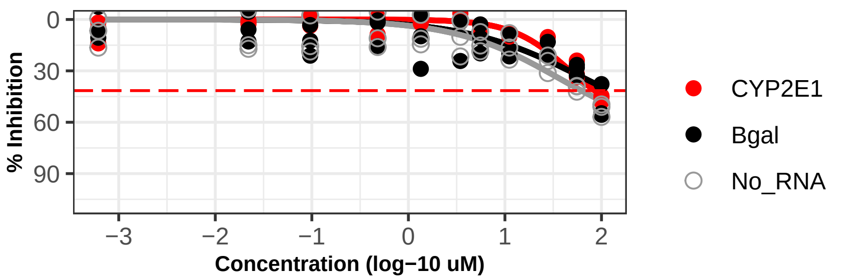

2,4−Dinitrophenol: CYP2C8

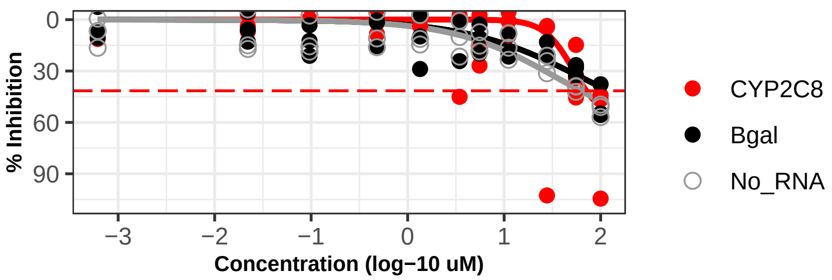

2,4−Dinitrophenol: CYP2J2

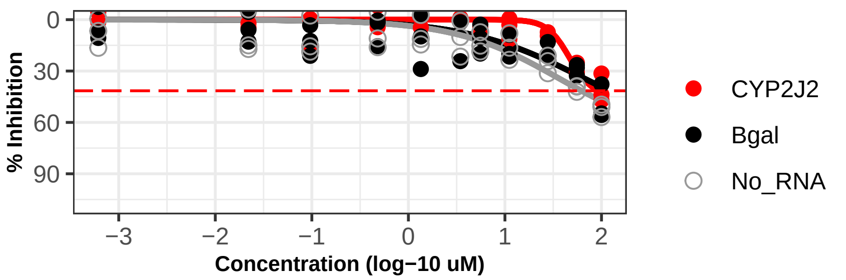

2,4−Dinitrophenol: CYP2C9

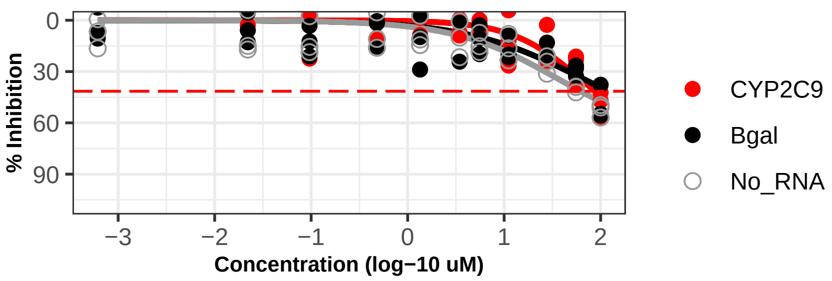

2,4−Dinitrophenol: CYP3A4

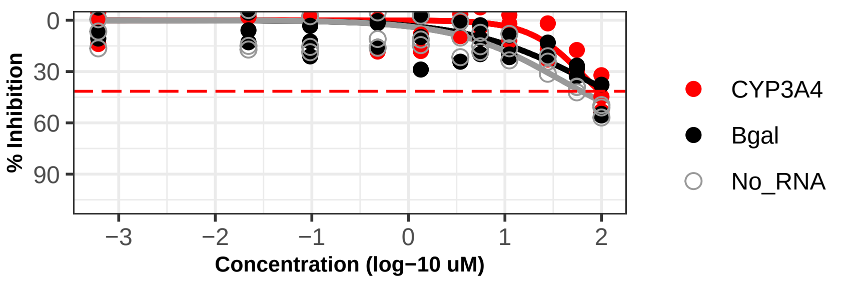

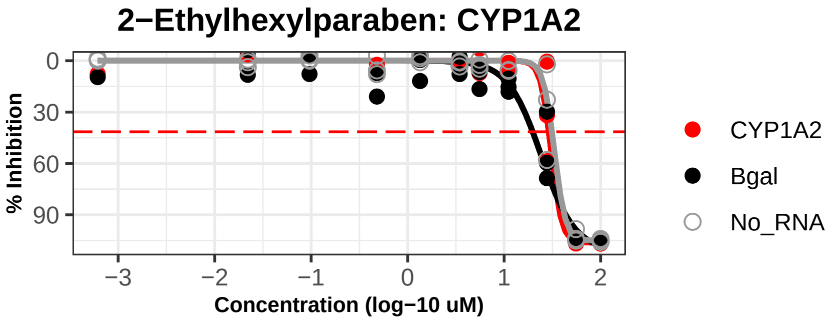
2−Ethylhexylparaben: CYP1A2
% Inhibition
Concentration (log−10 uM)
CYP1A2
Bgal
No_RNA

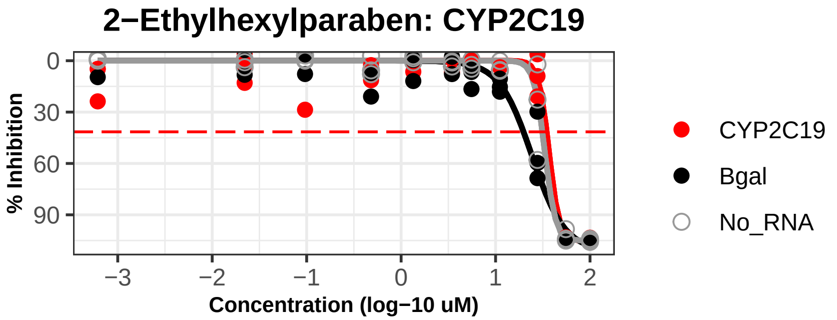
2−Ethylhexylparaben: CYP2C19
% Inhibition
Concentration (log−10 uM)
CYP2C19
Bgal
No_RNA

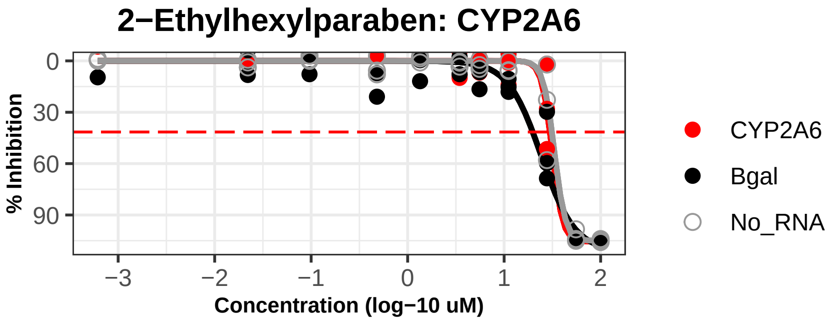
2−Ethylhexylparaben: CYP2A6
% Inhibition
Concentration (log−10 uM)
CYP2A6
Bgal
No_RNA

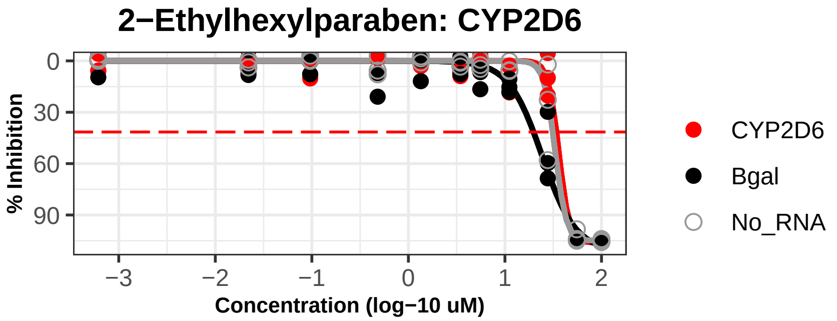
2−Ethylhexylparaben: CYP2D6
% Inhibition
Concentration (log−10 uM)
CYP2D6
Bgal
No_RNA

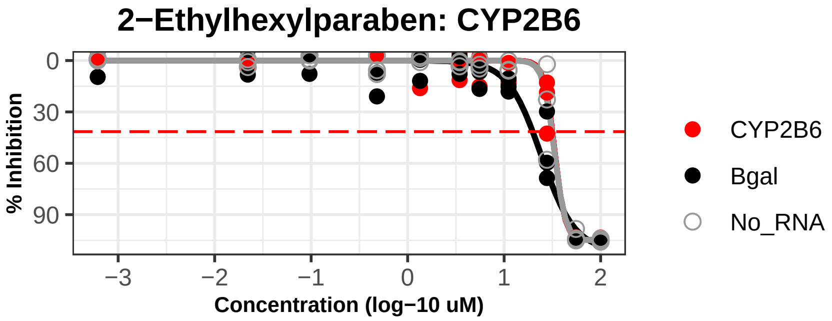
2−Ethylhexylparaben: CYP2B6
% Inhibition
Concentration (log−10 uM)
CYP2B6
Bgal
No_RNA

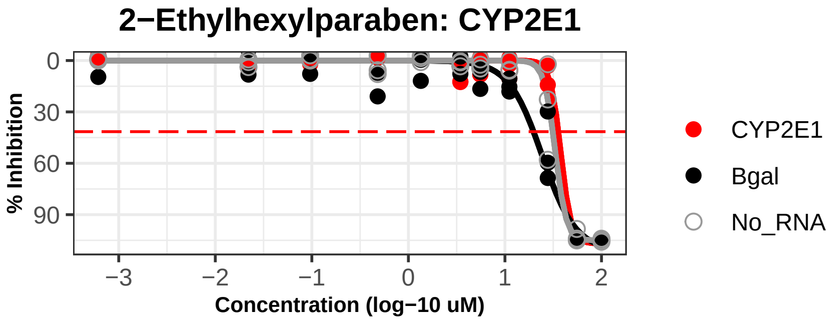
2−Ethylhexylparaben: CYP2E1
% Inhibition
Concentration (log−10 uM)
CYP2E1
Bgal
No_RNA

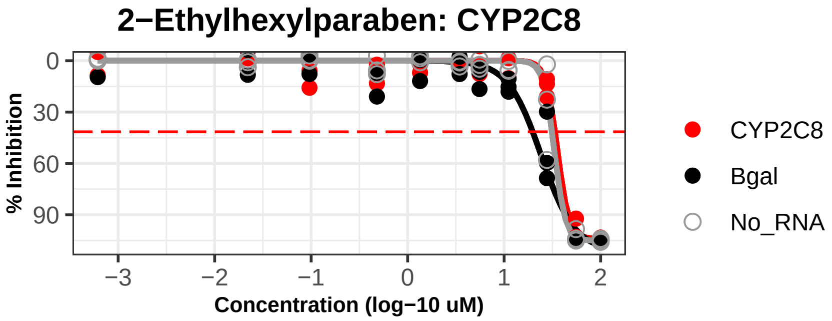
2−Ethylhexylparaben: CYP2C8
% Inhibition
Concentration (log−10 uM)
CYP2C8
Bgal
No_RNA

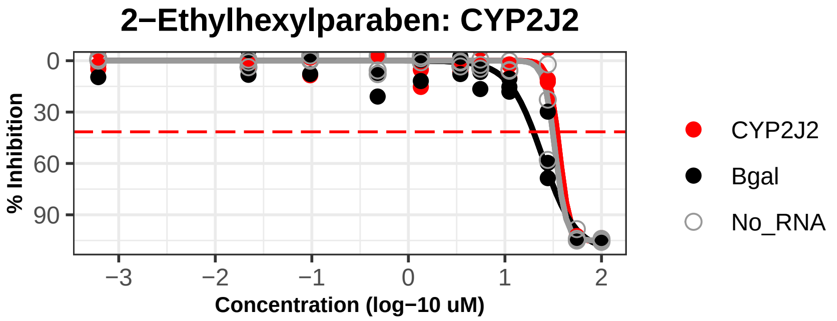
2−Ethylhexylparaben: CYP2J2
% Inhibition
Concentration (log−10 uM)
CYP2J2
Bgal
No_RNA

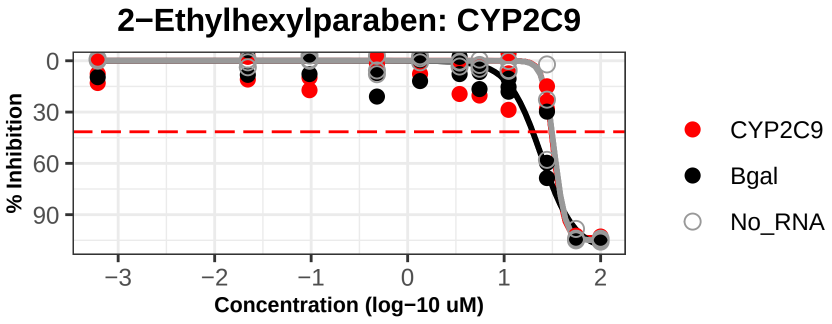
2−Ethylhexylparaben: CYP2C9
% Inhibition
Concentration (log−10 uM)
CYP2C9
Bgal
No_RNA

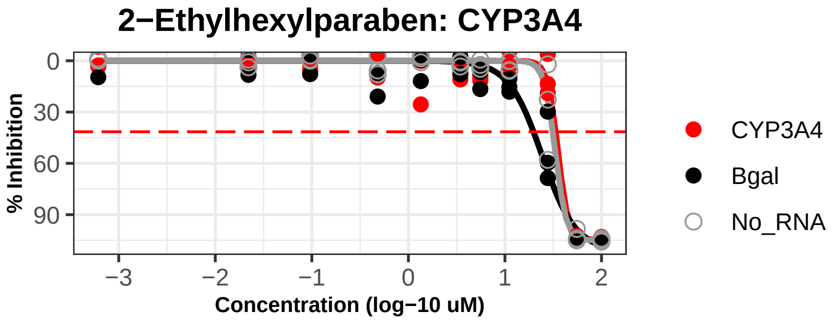
2−Ethylhexylparaben: CYP3A4
% Inhibition
Concentration (log−10 uM)
CYP3A4
Bgal
No_RNA

4,4'−Sulfonyldiphenol: CYP1A2

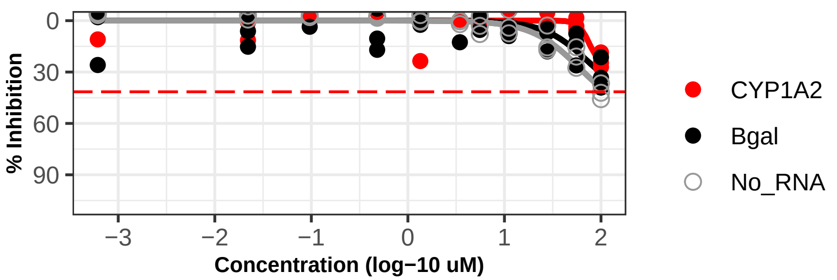

4,4'−Sulfonyldiphenol: CYP2C19

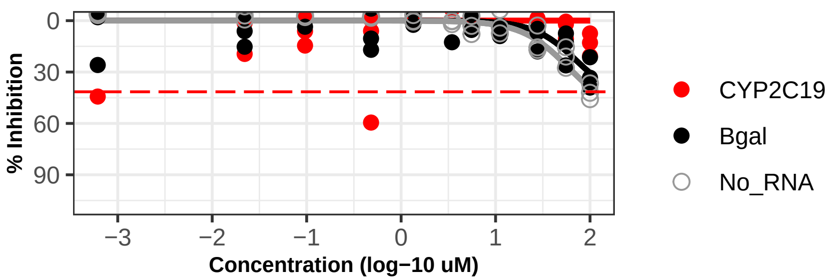

4,4'−Sulfonyldiphenol: CYP2A6

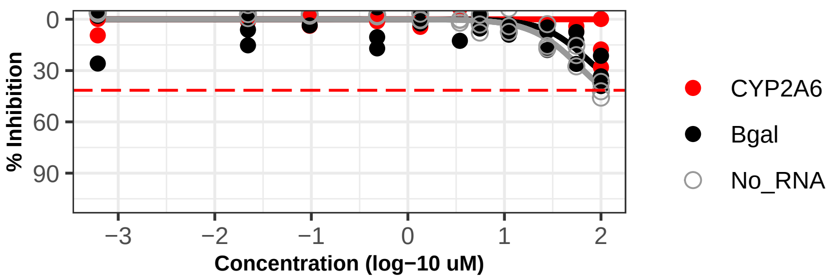

4,4'−Sulfonyldiphenol: CYP2D6

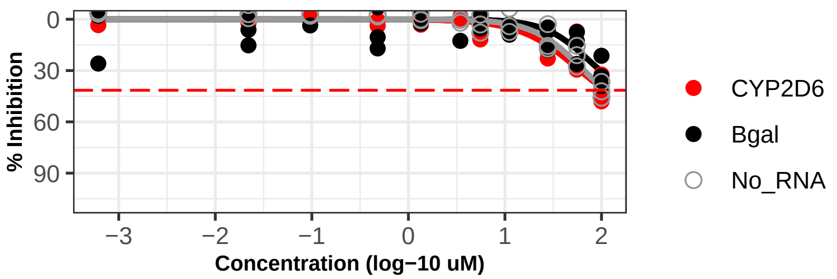

4,4'−Sulfonyldiphenol: CYP2B6

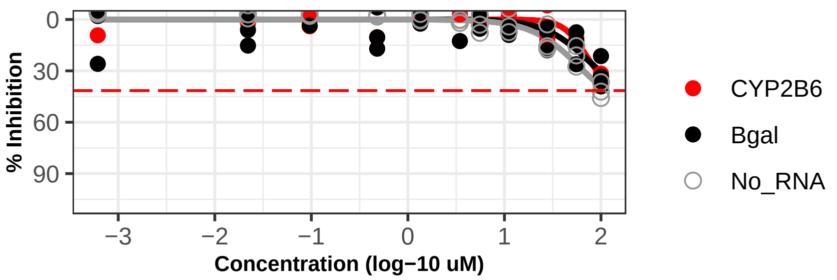

4,4'−Sulfonyldiphenol: CYP2E1

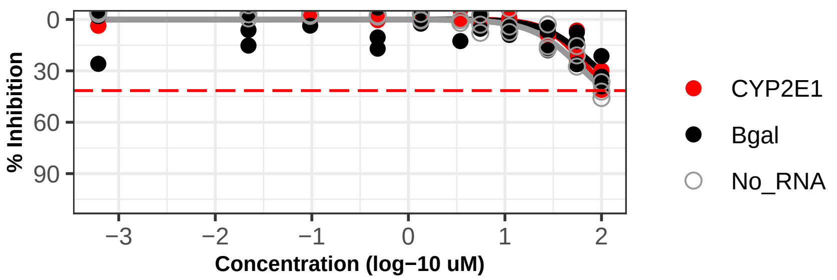

4,4'−Sulfonyldiphenol: CYP2C8

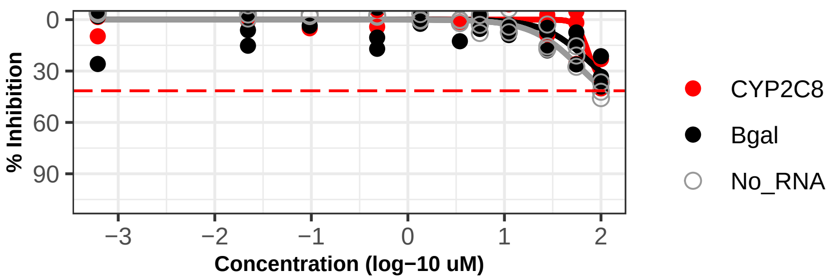

4,4'−Sulfonyldiphenol: CYP2J2

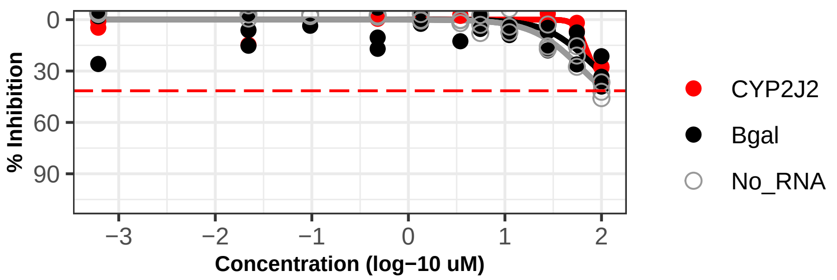

4,4'−Sulfonyldiphenol: CYP2C9

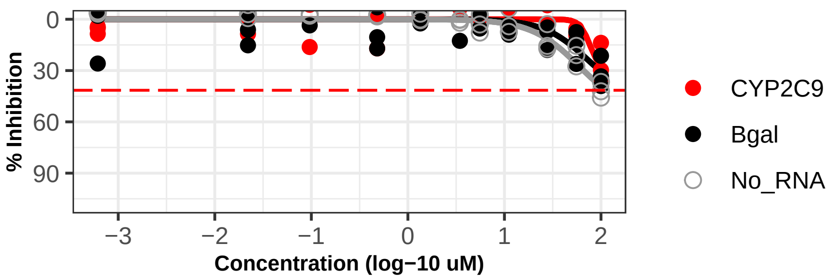

4,4'−Sulfonyldiphenol: CYP3A4

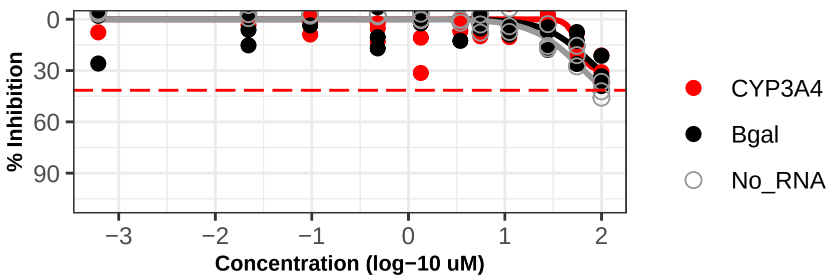

4−(1,1,3,3−Tetramethylbutyl)phenol: CYP1A2

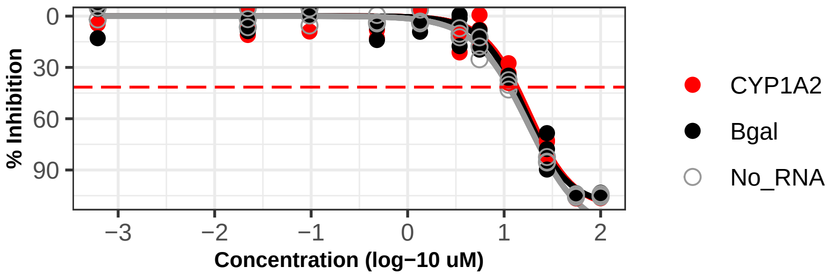

4−(1,1,3,3−Tetramethylbutyl)phenol: CYP2C19

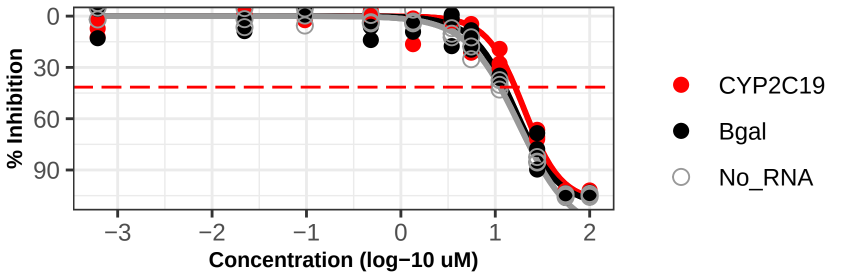

4−(1,1,3,3−Tetramethylbutyl)phenol: CYP2A6

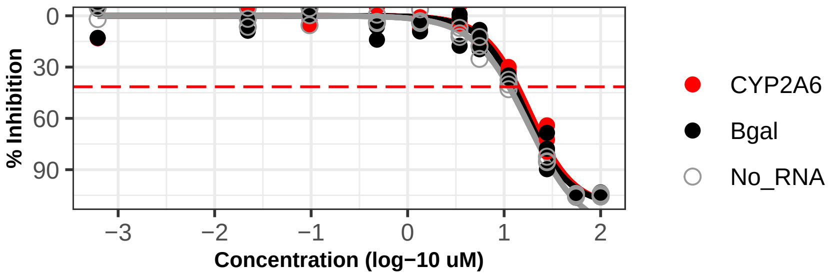

4−(1,1,3,3−Tetramethylbutyl)phenol: CYP2D6

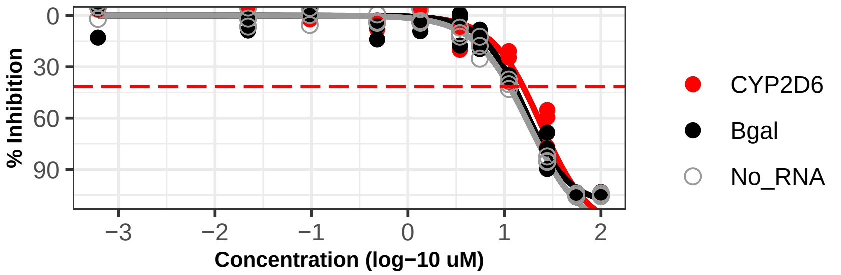

4−(1,1,3,3−Tetramethylbutyl)phenol: CYP2B6

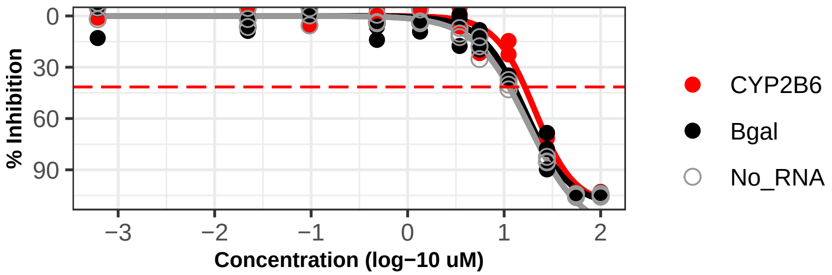

4−(1,1,3,3−Tetramethylbutyl)phenol: CYP2E1

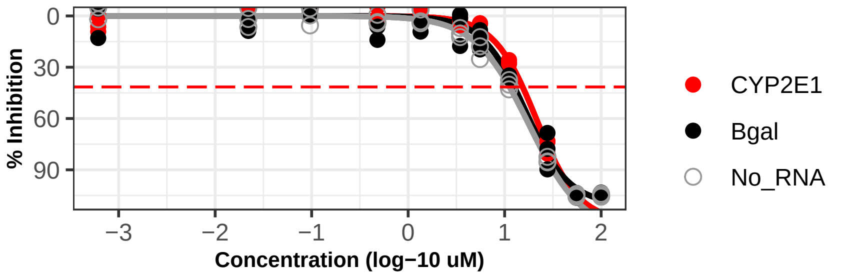

4−(1,1,3,3−Tetramethylbutyl)phenol: CYP2C8

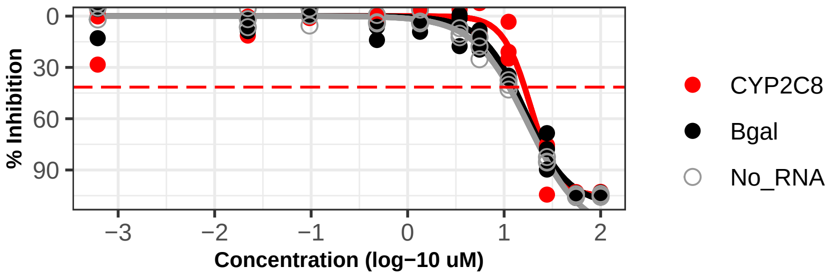

4−(1,1,3,3−Tetramethylbutyl)phenol: CYP2J2

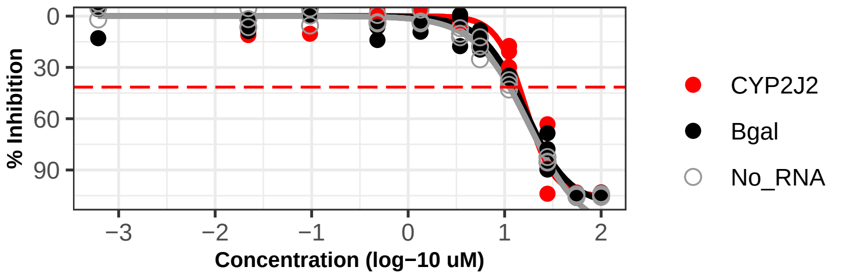

4−(1,1,3,3−Tetramethylbutyl)phenol: CYP2C9

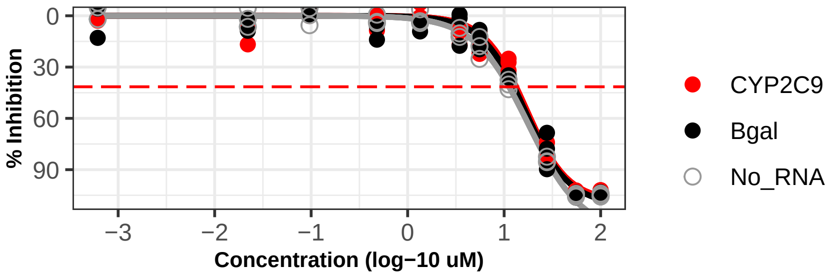

4−(1,1,3,3−Tetramethylbutyl)phenol: CYP3A4

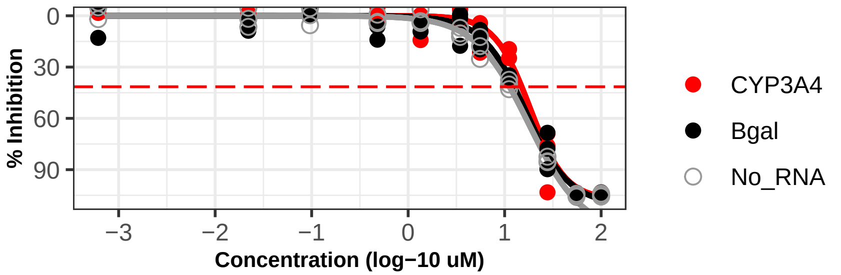

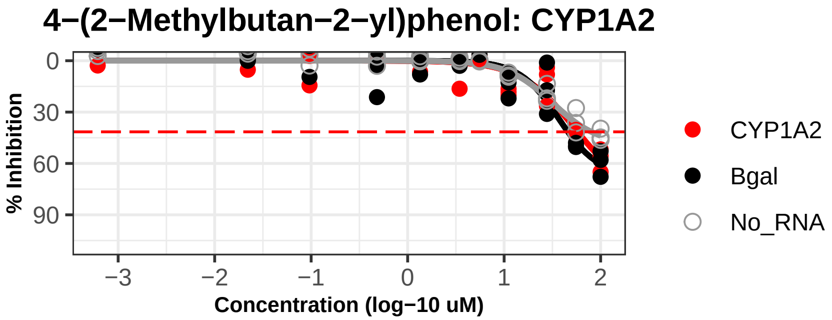
4−(2−Methylbutan−2−yl)phenol: CYP1A2
% Inhibition
0
30
60
90
−3
−2
−1
0
1
2
Concentration (log−10 uM)
CYP1A2
Bgal
No_RNA

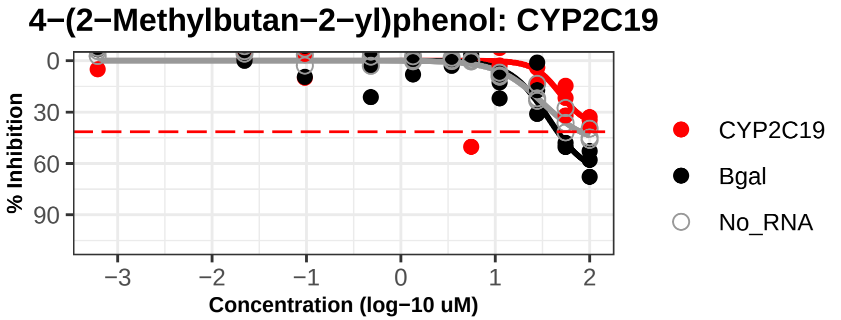
4−(2−Methylbutan−2−yl)phenol: CYP2C19
% Inhibition
0
30
60
90
−3
−2
−1
0
1
2
Concentration (log−10 uM)
CYP2C19
Bgal
No_RNA

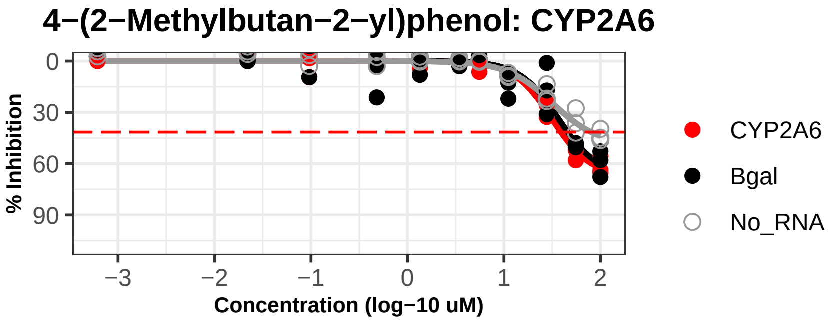
4−(2−Methylbutan−2−yl)phenol: CYP2A6
% Inhibition
0
30
60
90
−3
−2
−1
0
1
2
Concentration (log−10 uM)
CYP2A6
Bgal
No_RNA

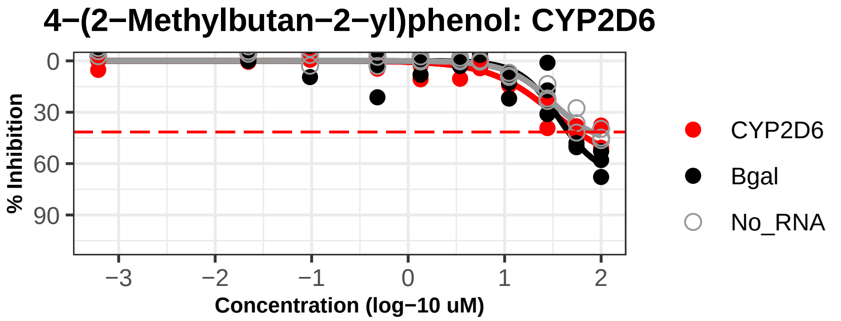
4−(2−Methylbutan−2−yl)phenol: CYP2D6
% Inhibition
0
30
60
90
−3
−2
−1
0
1
2
Concentration (log−10 uM)
CYP2D6
Bgal
No_RNA

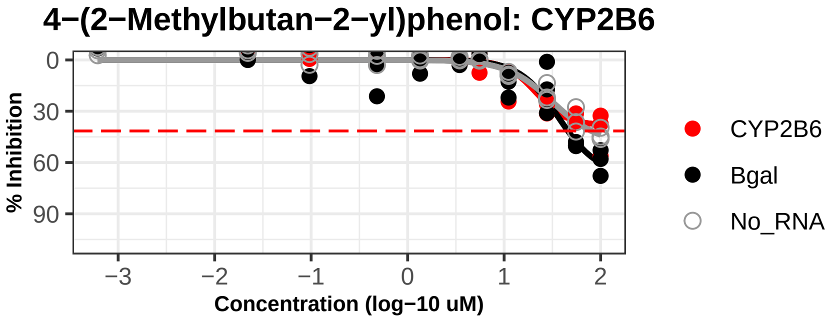
4−(2−Methylbutan−2−yl)phenol: CYP2B6
% Inhibition
0
30
60
90
−3
−2
−1
0
1
2
Concentration (log−10 uM)
CYP2B6
Bgal
No_RNA

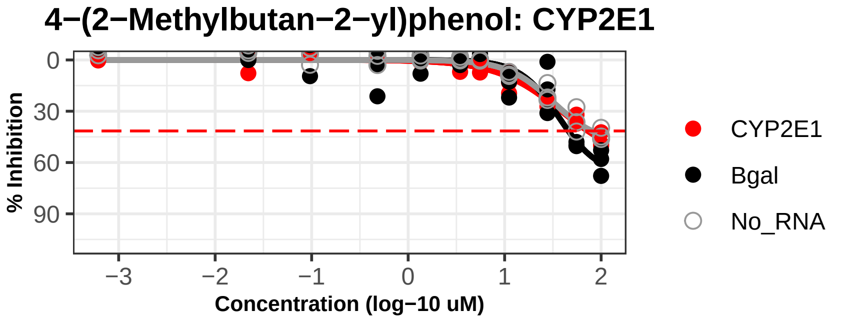
4−(2−Methylbutan−2−yl)phenol: CYP2E1
% Inhibition
0
30
60
90
−3
−2
−1
0
1
2
Concentration (log−10 uM)
CYP2E1
Bgal
No_RNA

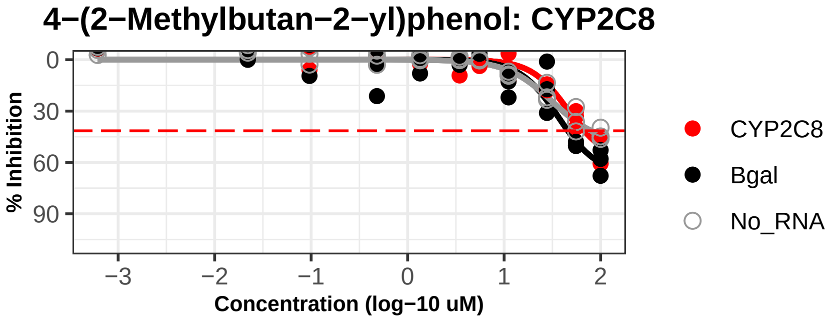
4−(2−Methylbutan−2−yl)phenol: CYP2C8
% Inhibition
0
30
60
90
−3
−2
−1
0
1
2
Concentration (log−10 uM)
CYP2C8
Bgal
No_RNA

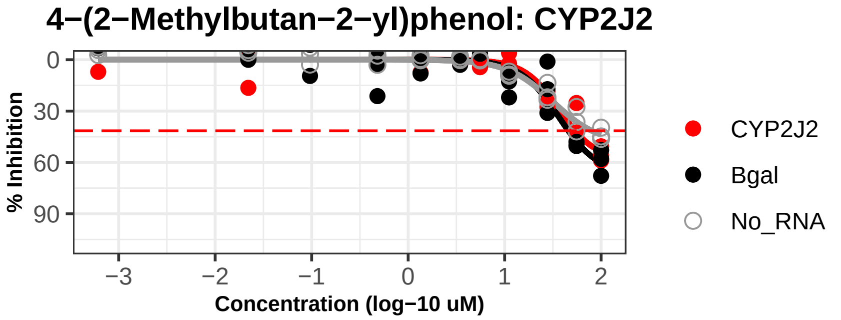
4−(2−Methylbutan−2−yl)phenol: CYP2J2
% Inhibition
0
30
60
90
−3
−2
−1
0
1
2
Concentration (log−10 uM)
CYP2J2
Bgal
No_RNA

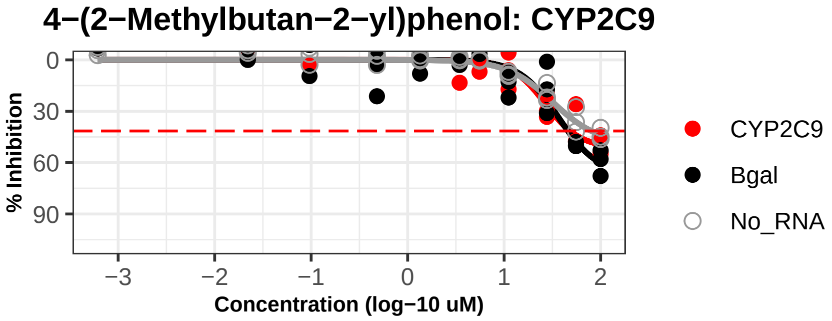
4−(2−Methylbutan−2−yl)phenol: CYP2C9
% Inhibition
0
30
60
90
−3
−2
−1
0
1
2
Concentration (log−10 uM)
CYP2C9
Bgal
No_RNA

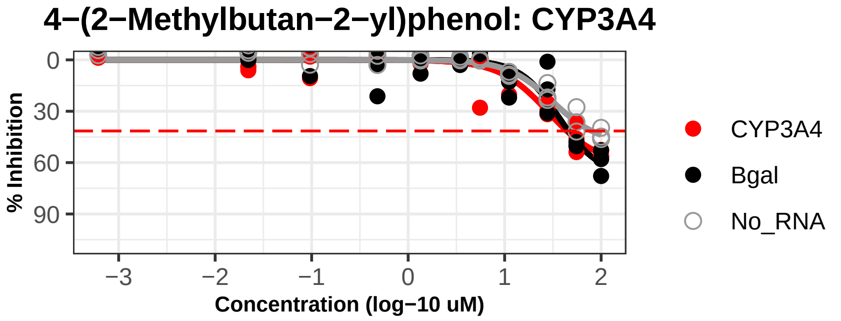
4−(2−Methylbutan−2−yl)phenol: CYP3A4
% Inhibition
0
30
60
90
−3
−2
−1
0
1
2
Concentration (log−10 uM)
CYP3A4
Bgal
No_RNA

4−Androstene−3,17−dione: CYP1A2

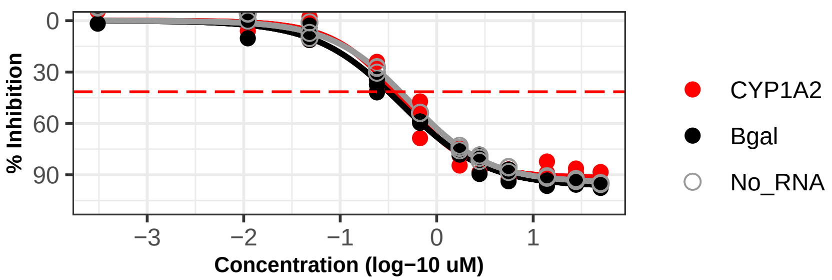

4−Androstene−3,17−dione: CYP2C19

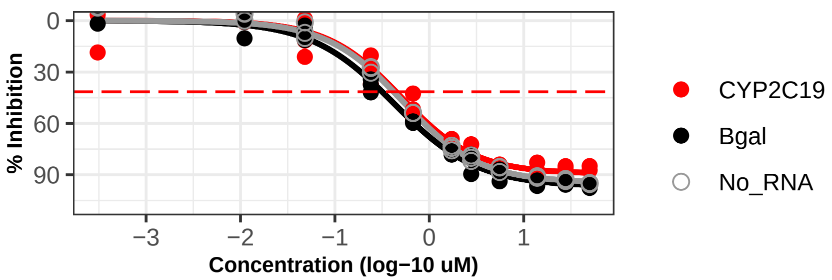

4−Androstene−3,17−dione: CYP2A6

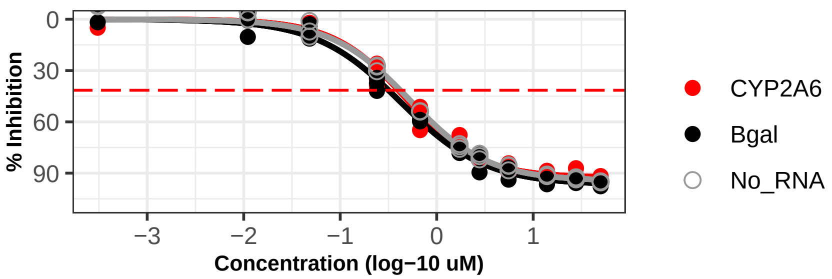

4−Androstene−3,17−dione: CYP2D6

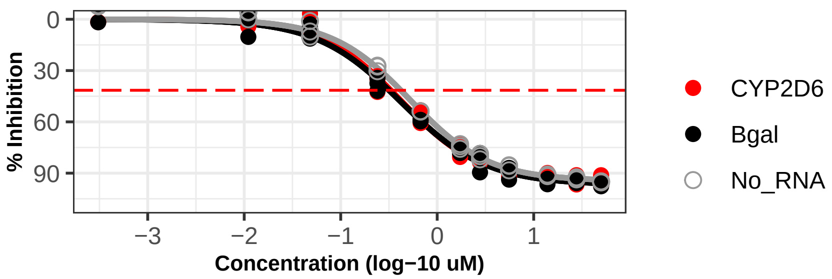

4−Androstene−3,17−dione: CYP2B6

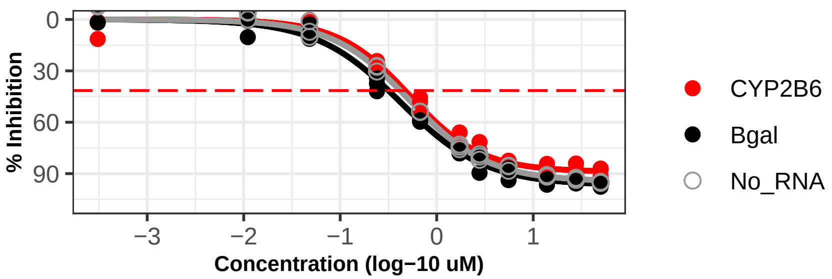

4−Androstene−3,17−dione: CYP2E1

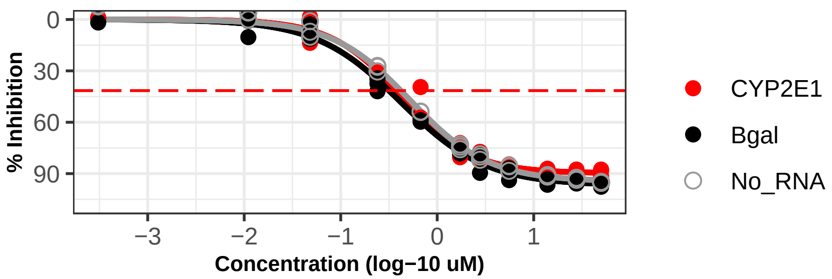

4−Androstene−3,17−dione: CYP2C8

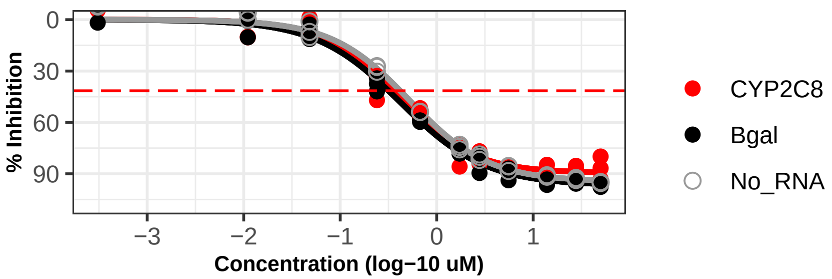

4−Androstene−3,17−dione: CYP2J2

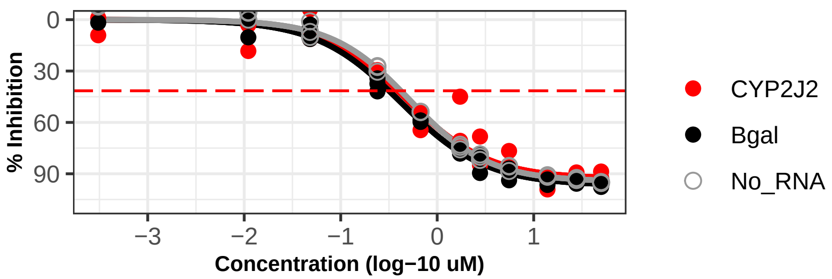

4−Androstene−3,17−dione: CYP2C9

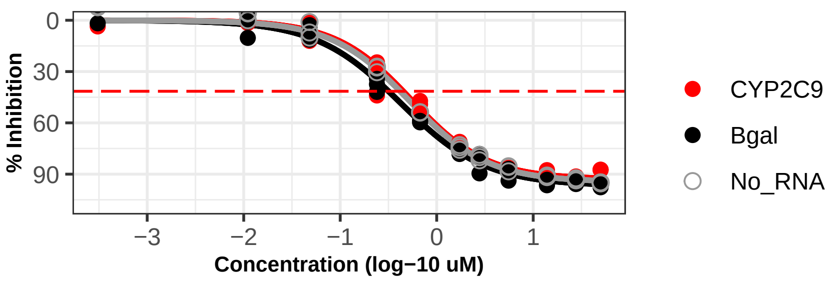

4−Androstene−3,17−dione: CYP3A4

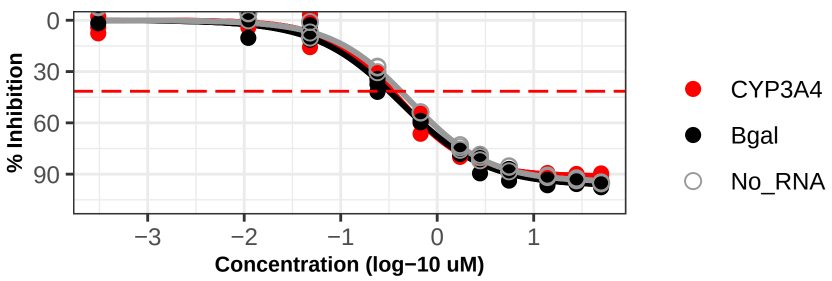

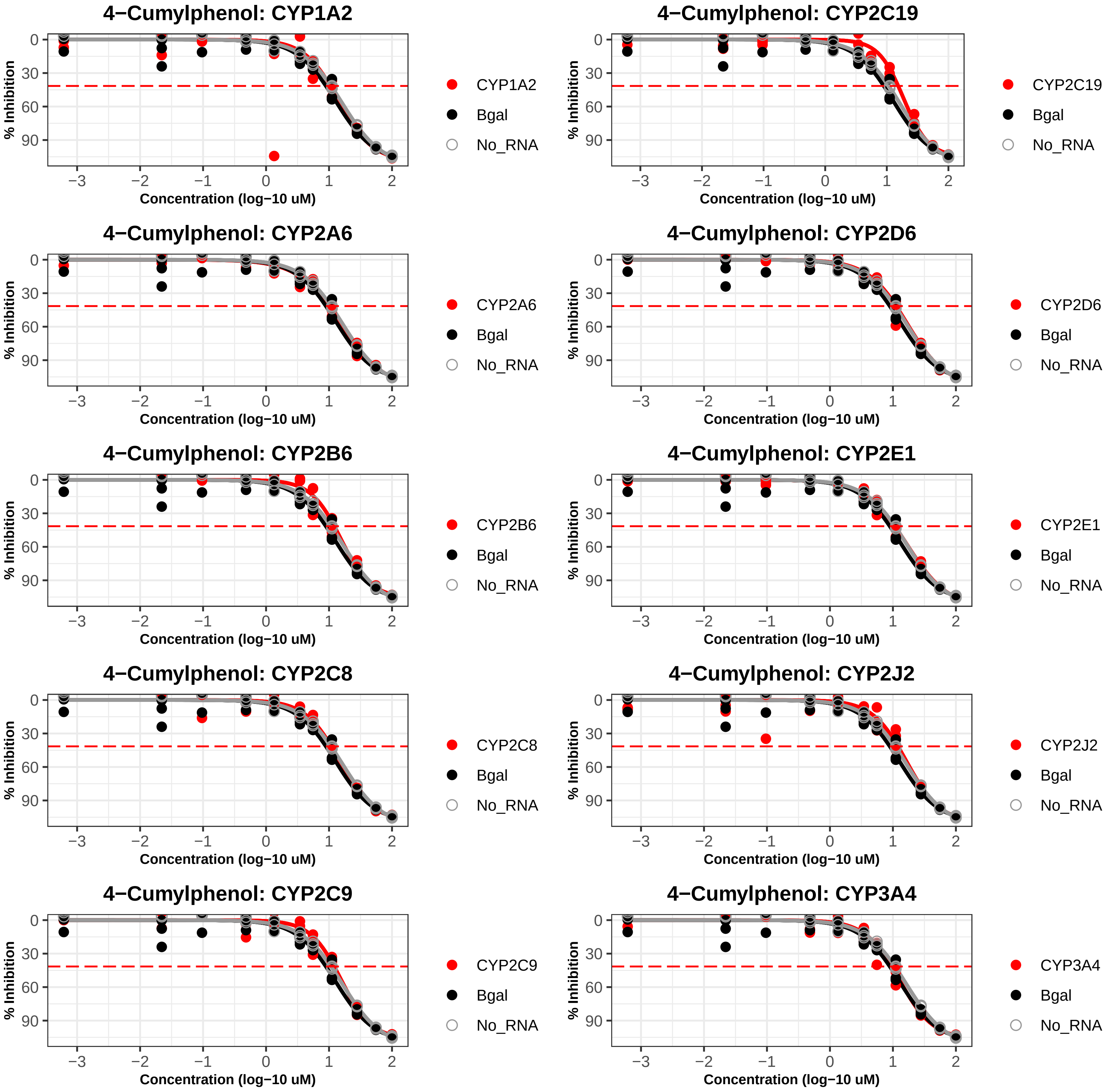
4−Cumylphenol: CYP1A2
% Inhibition
Concentration (log−10 uM)
CYP1A2
Bgal
No_RNA
4−Cumylphenol: CYP2C19
% Inhibition
Concentration (log−10 uM)
CYP2C19
Bgal
No_RNA
4−Cumylphenol: CYP2A6
% Inhibition
Concentration (log−10 uM)
CYP2A6
Bgal
No_RNA
4−Cumylphenol: CYP2D6
% Inhibition
Concentration (log−10 uM)
CYP2D6
Bgal
No_RNA
4−Cumylphenol: CYP2B6
% Inhibition
Concentration (log−10 uM)
CYP2B6
Bgal
No_RNA
4−Cumylphenol: CYP2E1
% Inhibition
Concentration (log−10 uM)
CYP2E1
Bgal
No_RNA
4−Cumylphenol: CYP2C8
% Inhibition
Concentration (log−10 uM)
CYP2C8
Bgal
No_RNA
4−Cumylphenol: CYP2J2
% Inhibition
Concentration (log−10 uM)
CYP2J2
Bgal
No_RNA
4−Cumylphenol: CYP2C9
% Inhibition
Concentration (log−10 uM)
CYP2C9
Bgal
No_RNA
4−Cumylphenol: CYP3A4
% Inhibition
Concentration (log−10 uM)
CYP3A4
Bgal
No_RNA

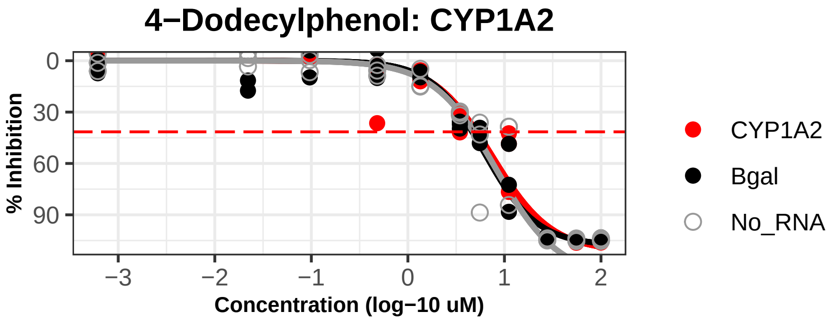
4−Dodecylphenol: CYP1A2
% Inhibition
Concentration (log−10 uM)
CYP1A2
Bgal
No_RNA

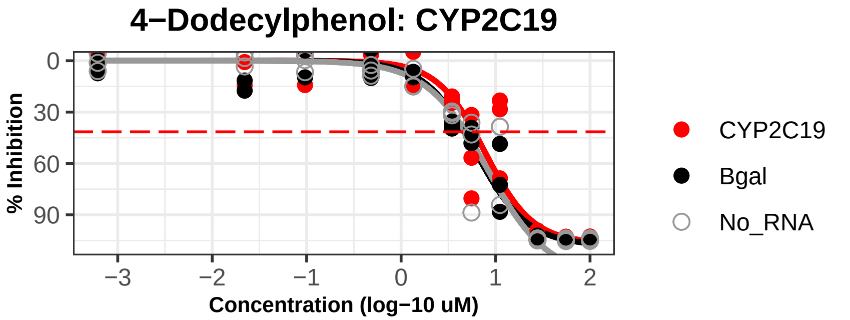
4−Dodecylphenol: CYP2C19
% Inhibition
Concentration (log−10 uM)
CYP2C19
Bgal
No_RNA

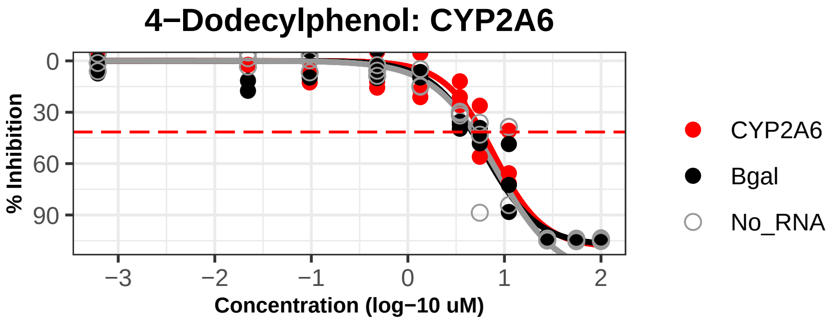
4−Dodecylphenol: CYP2A6
% Inhibition
Concentration (log−10 uM)
CYP2A6
Bgal
No_RNA

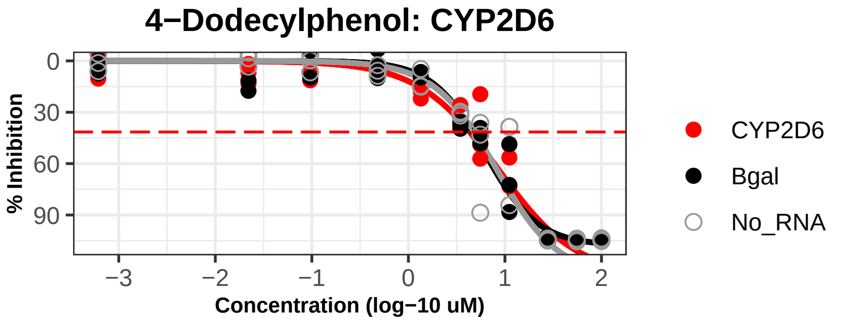
4−Dodecylphenol: CYP2D6
% Inhibition
Concentration (log−10 uM)
CYP2D6
Bgal
No_RNA

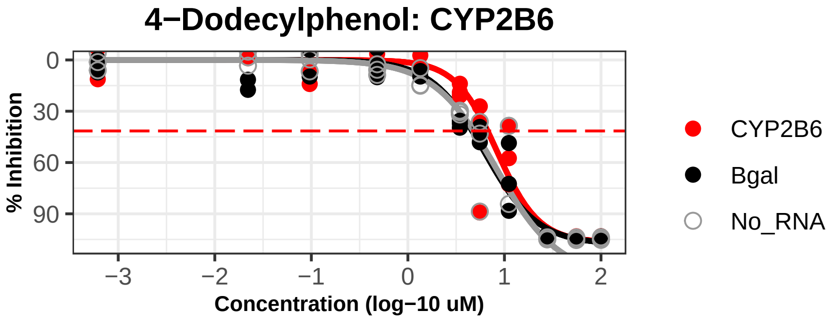
4−Dodecylphenol: CYP2B6
% Inhibition
Concentration (log−10 uM)
CYP2B6
Bgal
No_RNA

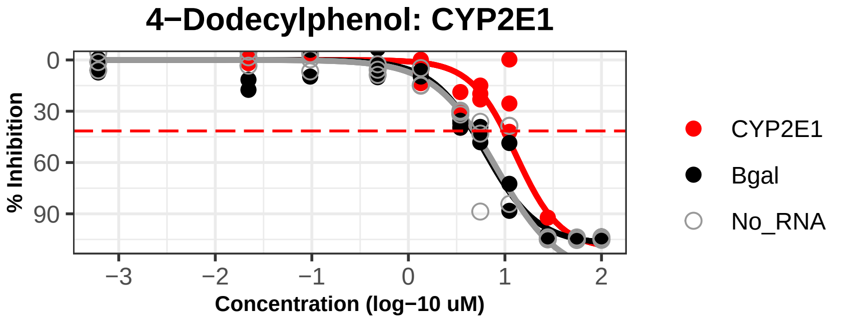
4−Dodecylphenol: CYP2E1
% Inhibition
Concentration (log−10 uM)
CYP2E1
Bgal
No_RNA

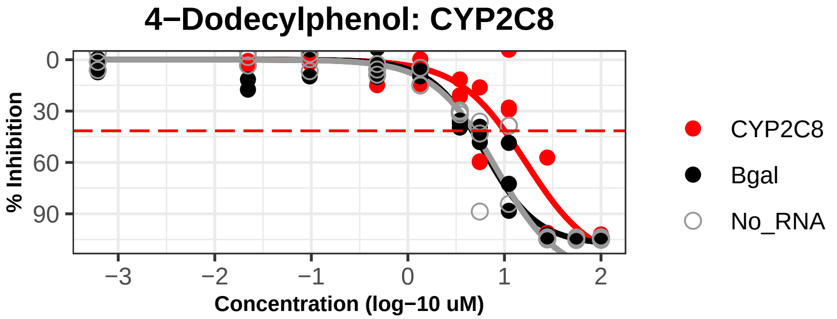
4−Dodecylphenol: CYP2C8
% Inhibition
Concentration (log−10 uM)
CYP2C8
Bgal
No_RNA

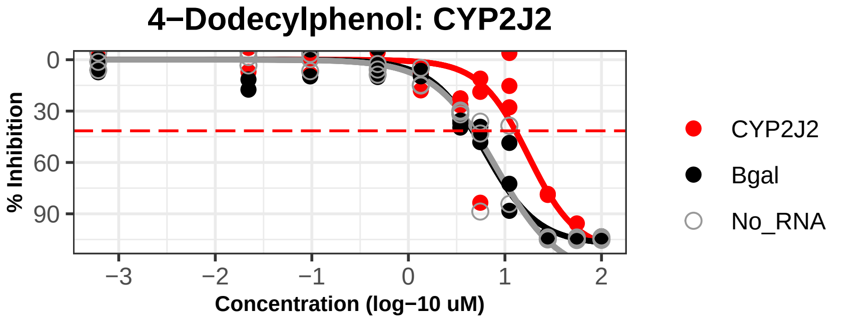
4−Dodecylphenol: CYP2J2
% Inhibition
Concentration (log−10 uM)
CYP2J2
Bgal
No_RNA

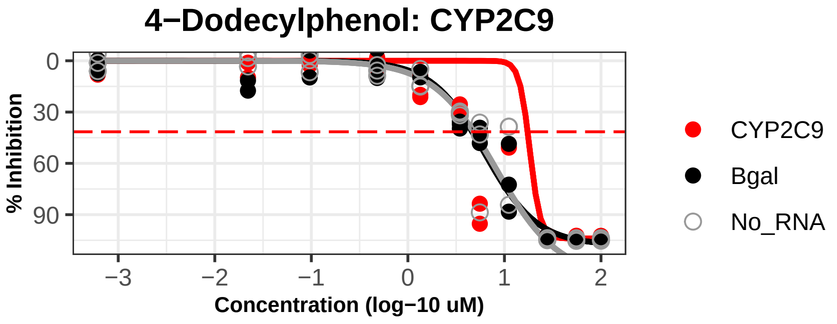
4−Dodecylphenol: CYP2C9
% Inhibition
Concentration (log−10 uM)
CYP2C9
Bgal
No_RNA

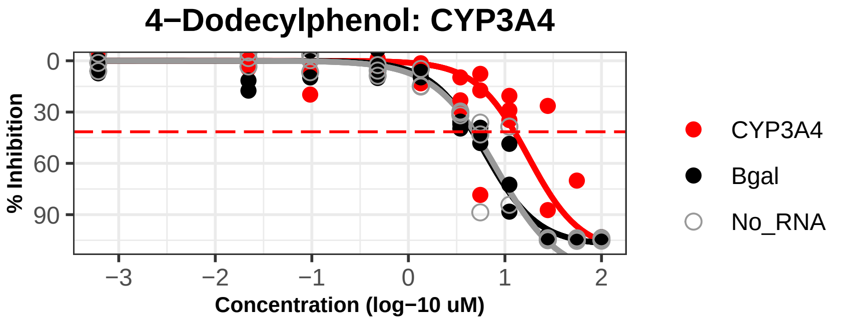
4−Dodecylphenol: CYP3A4
% Inhibition
Concentration (log−10 uM)
CYP3A4
Bgal
No_RNA

4−Hydroxybenzoic acid: CYP1A2

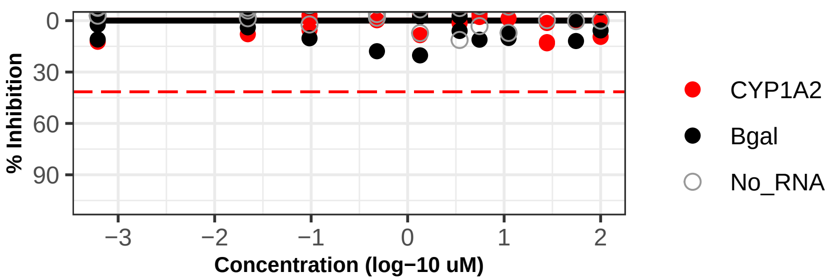

4−Hydroxybenzoic acid: CYP2C19

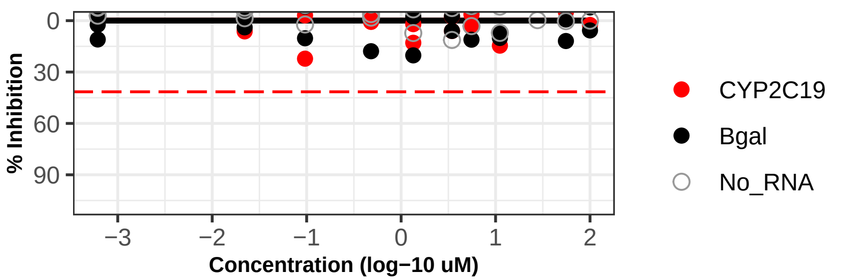

4−Hydroxybenzoic acid: CYP2A6

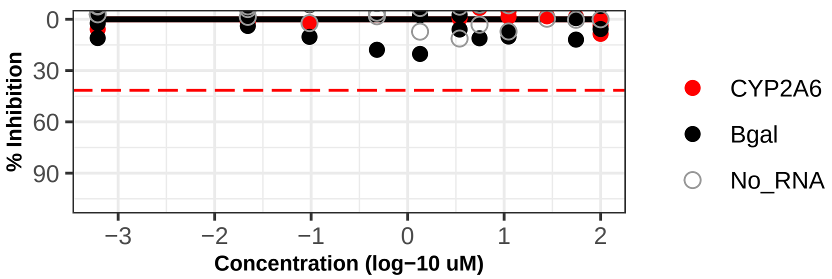

4−Hydroxybenzoic acid: CYP2D6

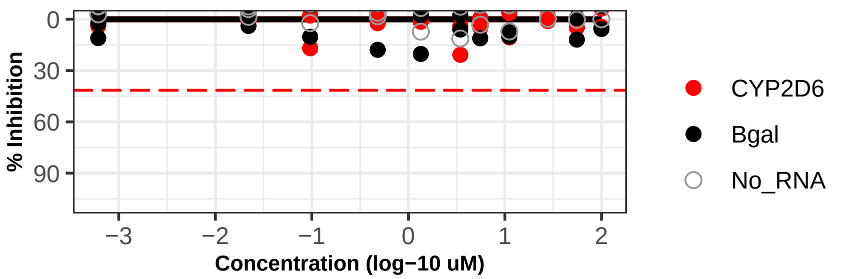

4−Hydroxybenzoic acid: CYP2B6

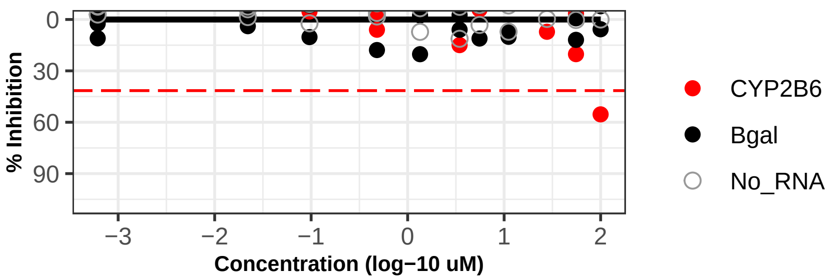

4−Hydroxybenzoic acid: CYP2E1

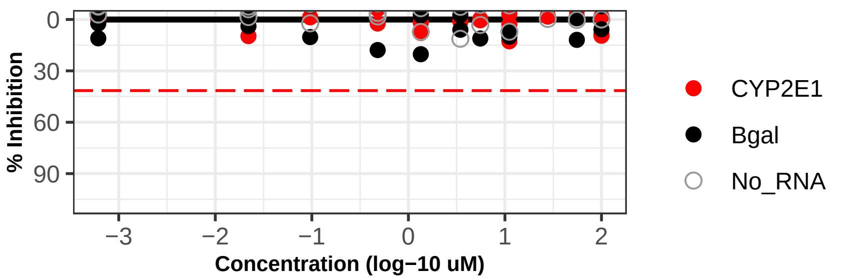

4−Hydroxybenzoic acid: CYP2C8

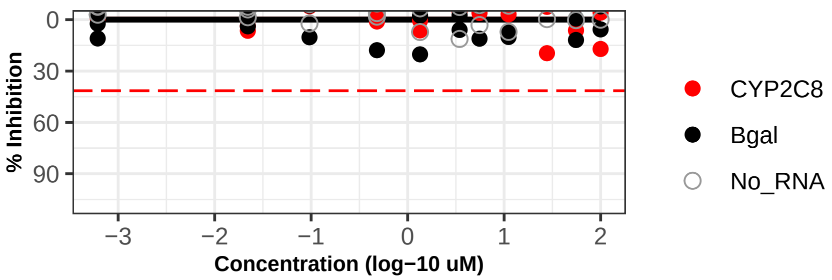

4−Hydroxybenzoic acid: CYP2J2

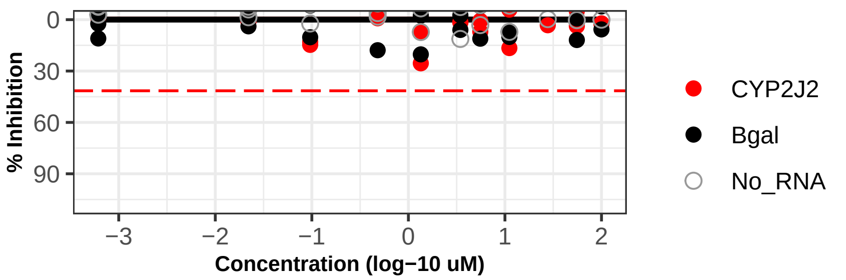

4−Hydroxybenzoic acid: CYP2C9

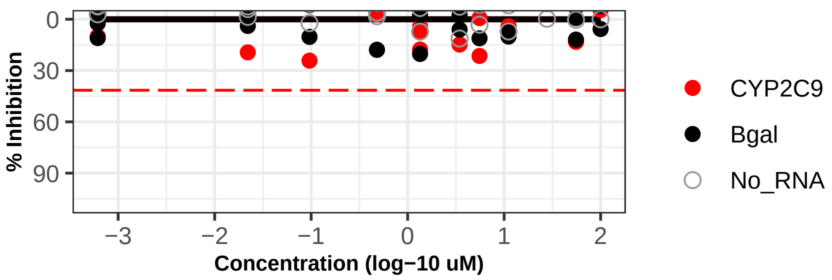

4−Hydroxybenzoic acid: CYP3A4

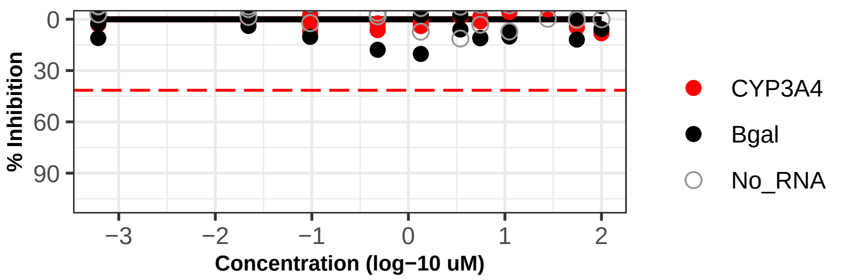

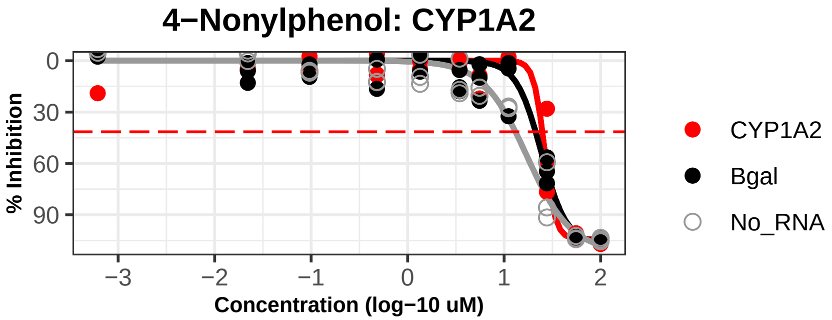
4−Nonylphenol: CYP1A2
% Inhibition
Concentration (log−10 uM)
CYP1A2
Bgal
No_RNA

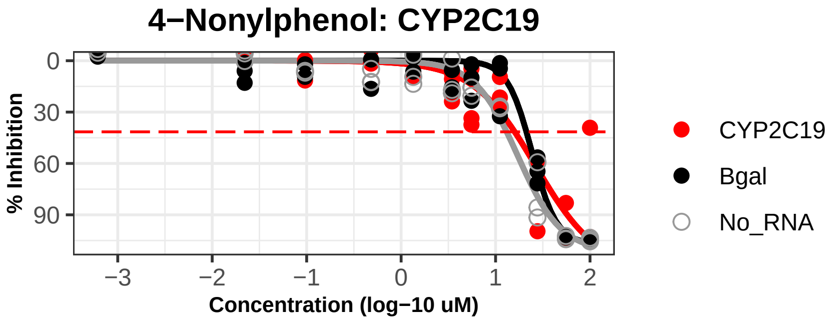
4−Nonylphenol: CYP2C19
% Inhibition
Concentration (log−10 uM)
CYP2C19
Bgal
No_RNA

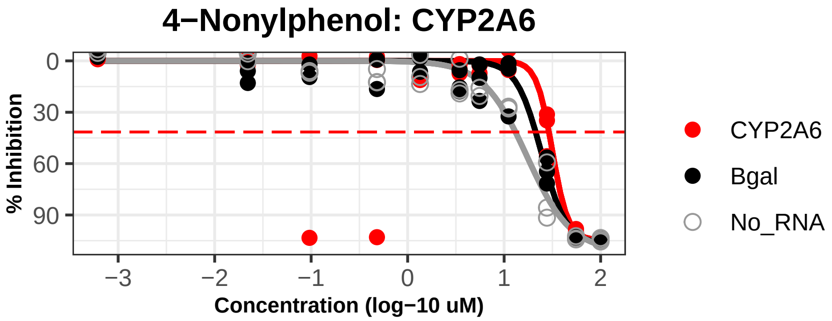
4−Nonylphenol: CYP2A6
% Inhibition
Concentration (log−10 uM)
CYP2A6
Bgal
No_RNA

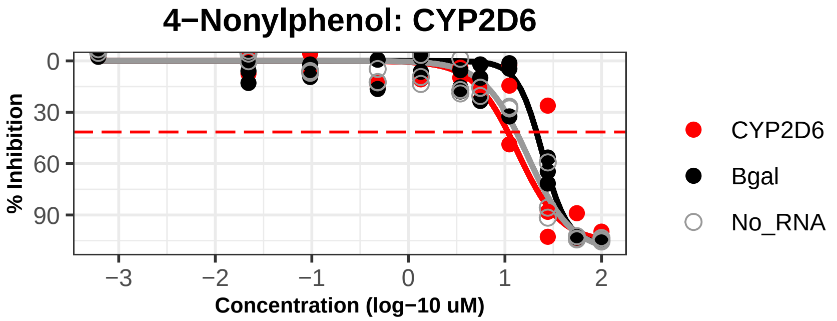
4−Nonylphenol: CYP2D6
% Inhibition
Concentration (log−10 uM)
CYP2D6
Bgal
No_RNA

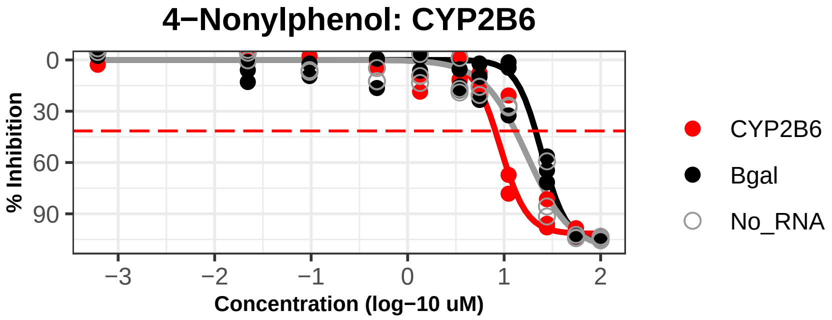
4−Nonylphenol: CYP2B6
% Inhibition
Concentration (log−10 uM)
CYP2B6
Bgal
No_RNA

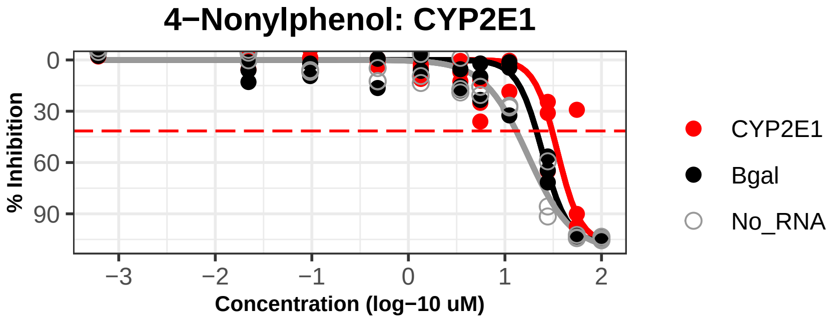
4−Nonylphenol: CYP2E1
% Inhibition
Concentration (log−10 uM)
CYP2E1
Bgal
No_RNA

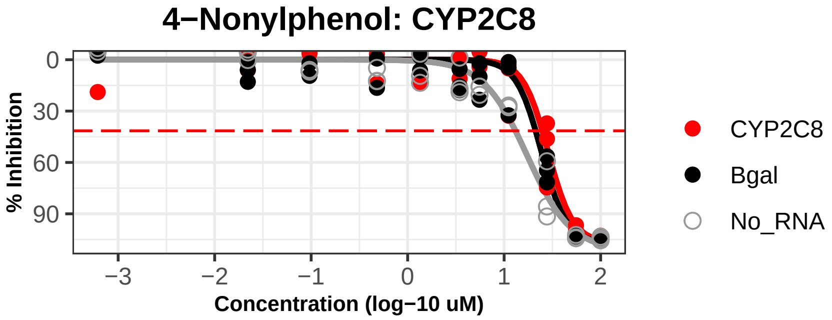
4−Nonylphenol: CYP2C8
% Inhibition
Concentration (log−10 uM)
CYP2C8
Bgal
No_RNA

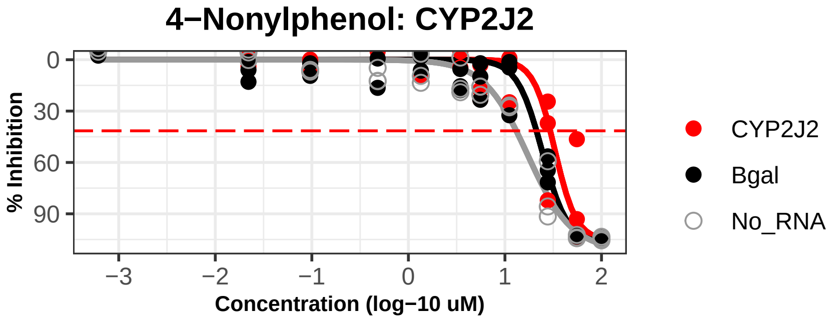
4−Nonylphenol: CYP2J2
% Inhibition
Concentration (log−10 uM)
CYP2J2
Bgal
No_RNA

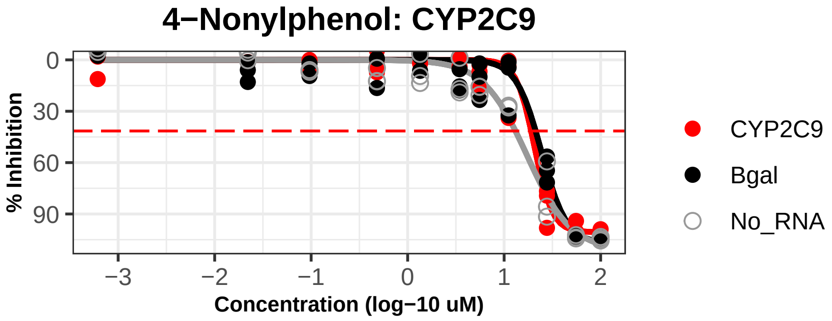
4−Nonylphenol: CYP2C9
% Inhibition
Concentration (log−10 uM)
CYP2C9
Bgal
No_RNA

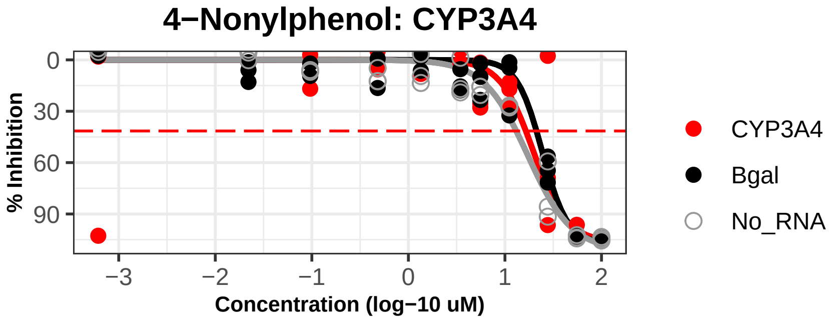
4−Nonylphenol: CYP3A4
% Inhibition
Concentration (log−10 uM)
CYP3A4
Bgal
No_RNA

4−tert−Butylphenol: CYP1A2

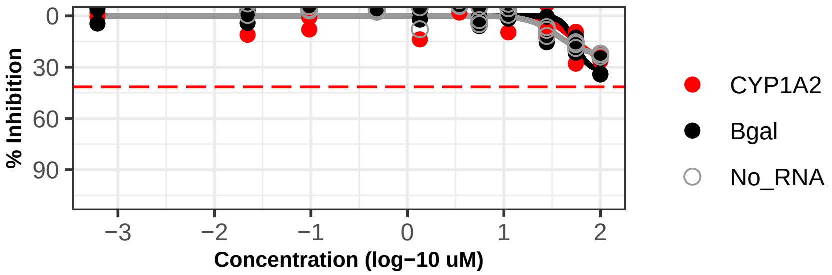

4−tert−Butylphenol: CYP2C19

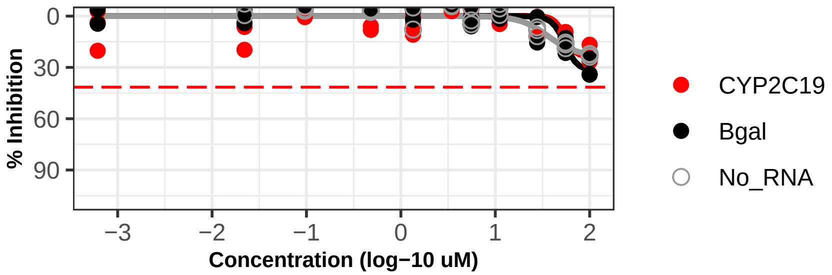

4−tert−Butylphenol: CYP2A6

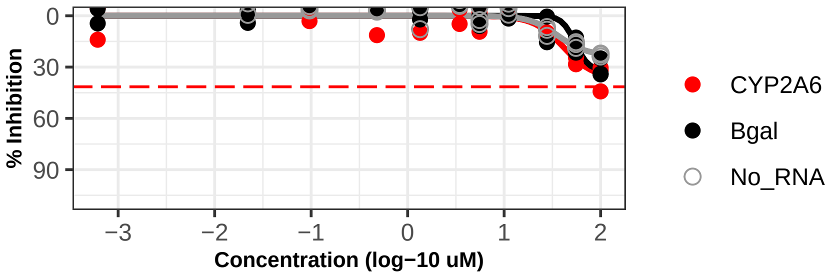

4−tert−Butylphenol: CYP2D6

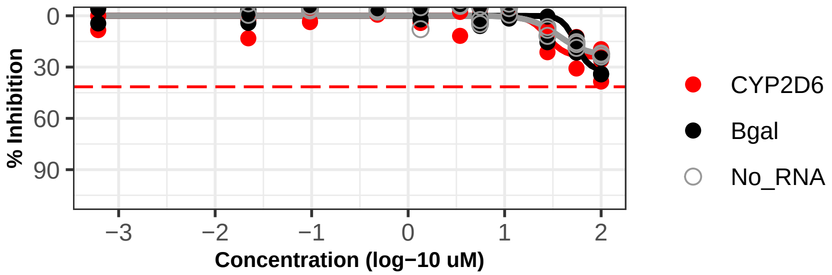

4−tert−Butylphenol: CYP2B6

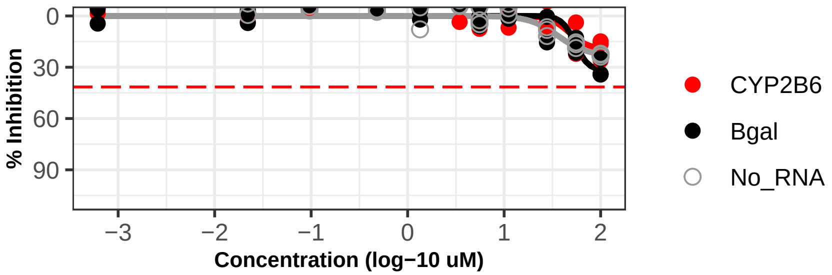

4−tert−Butylphenol: CYP2E1

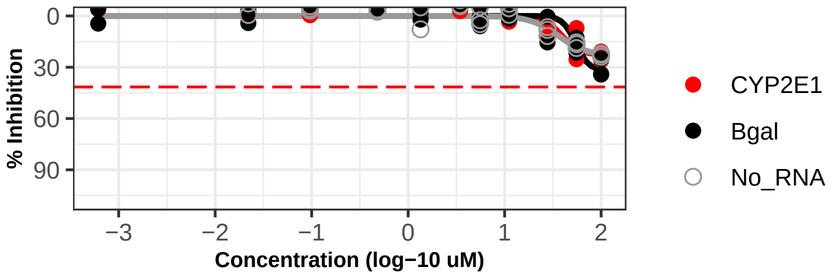

4−tert−Butylphenol: CYP2C8

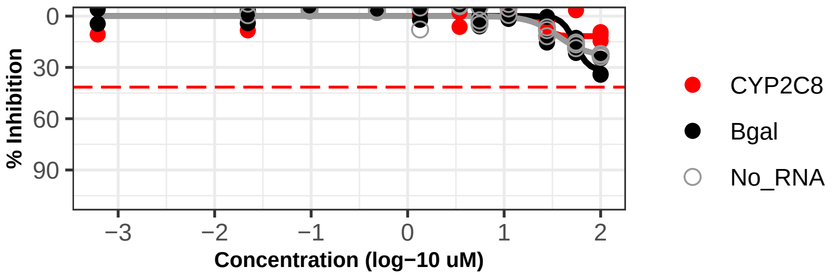

4−tert−Butylphenol: CYP2J2

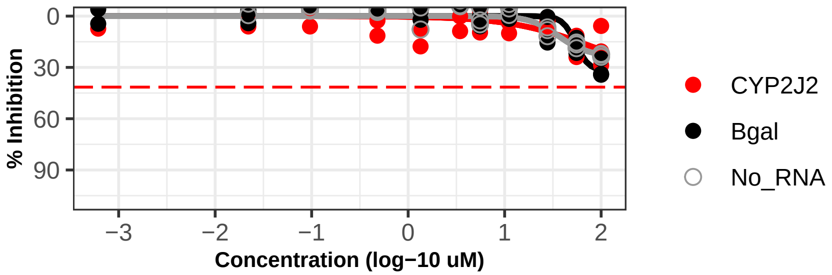

4−tert−Butylphenol: CYP2C9

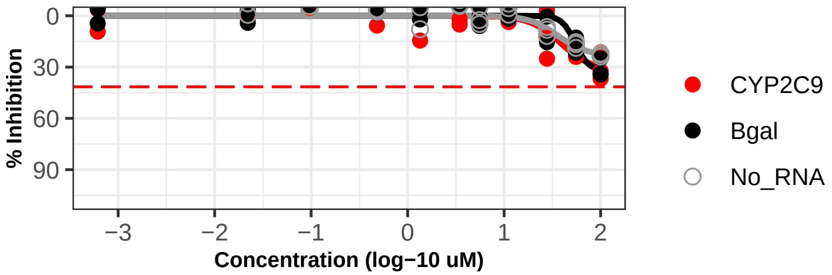

4−tert−Butylphenol: CYP3A4

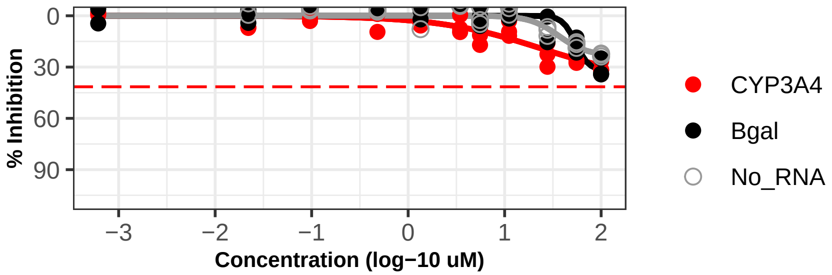

5alpha−Dihydrotestosterone: CYP1A2

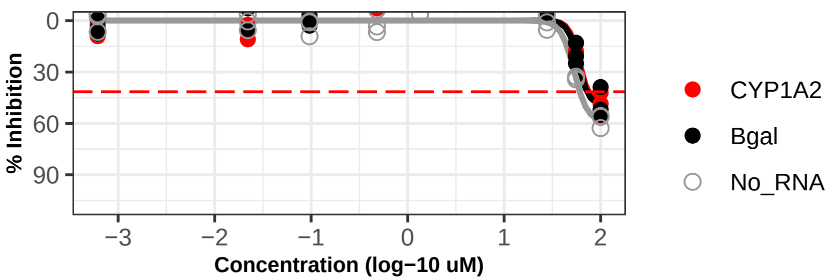

5alpha−Dihydrotestosterone: CYP2C19

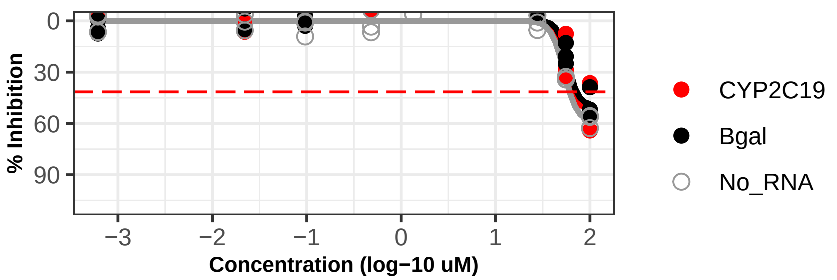

5alpha−Dihydrotestosterone: CYP2A6

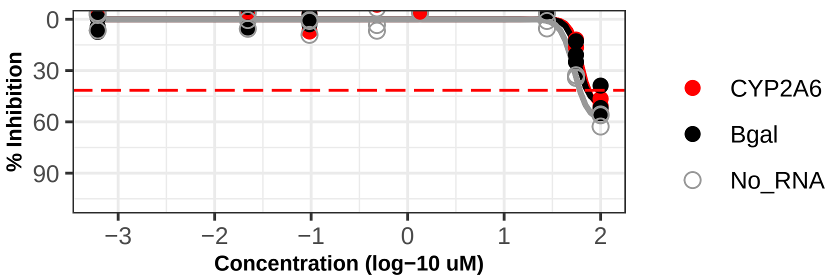

5alpha−Dihydrotestosterone: CYP2D6

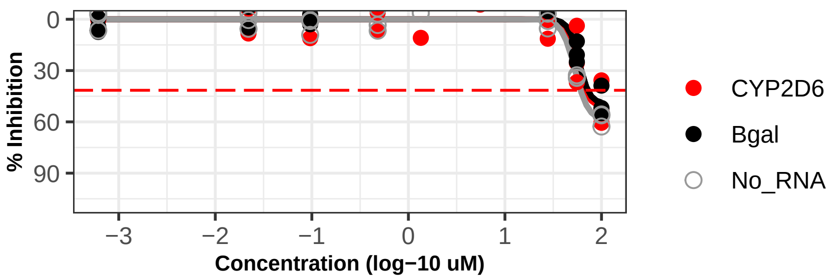

5alpha−Dihydrotestosterone: CYP2B6

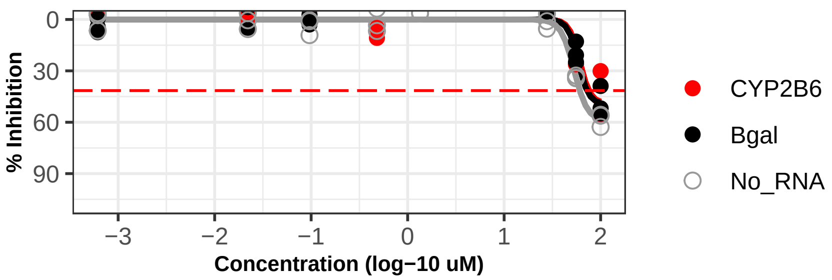

5alpha−Dihydrotestosterone: CYP2E1

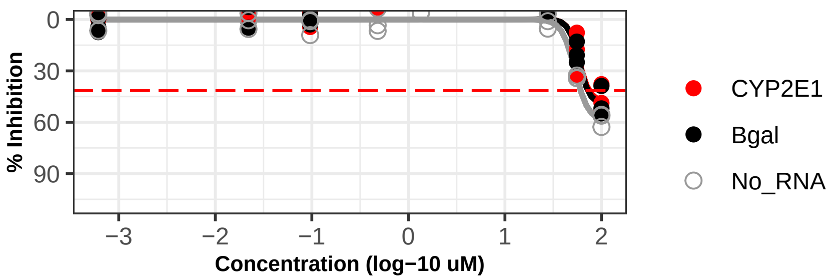

5alpha−Dihydrotestosterone: CYP2C8

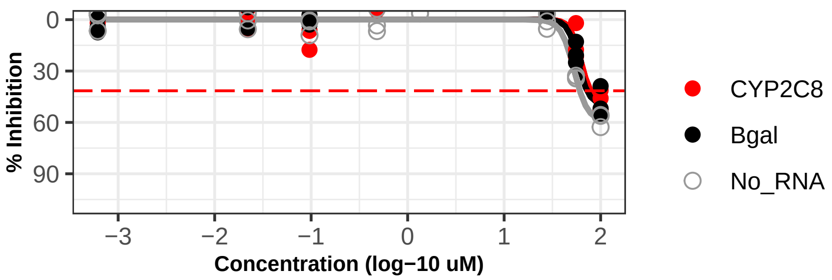

5alpha−Dihydrotestosterone: CYP2J2

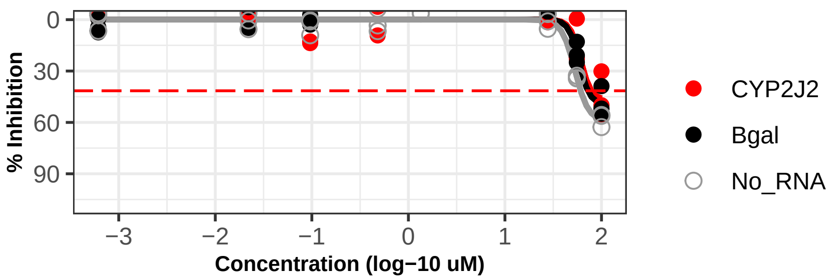

5alpha−Dihydrotestosterone: CYP2C9

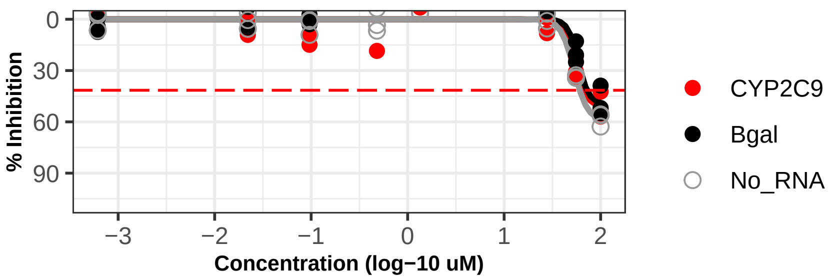

5alpha−Dihydrotestosterone: CYP3A4

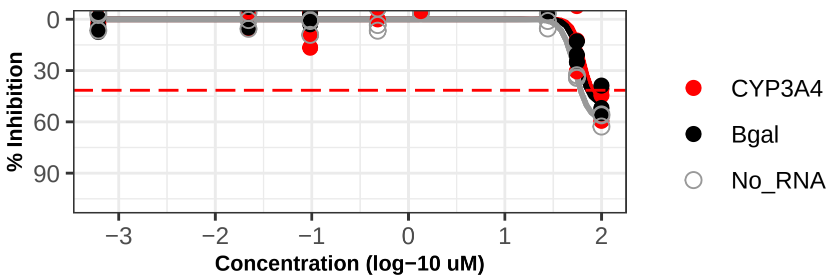

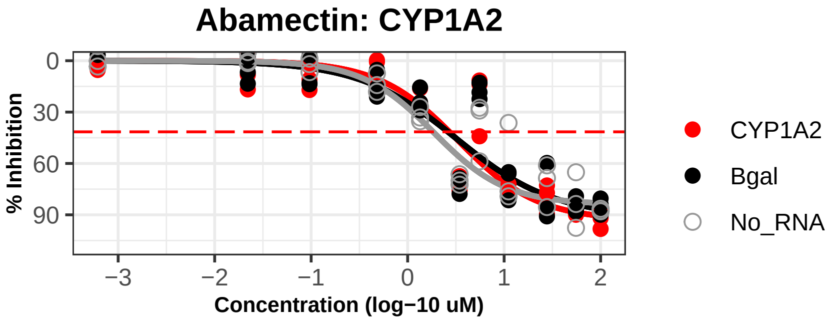
Abamectin: CYP1A2
% Inhibition
Concentration (log−10 uM)
CYP1A2
Bgal
No_RNA

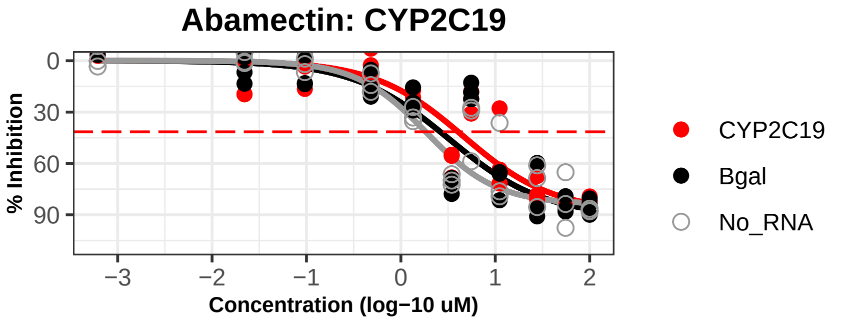
Abamectin: CYP2C19
% Inhibition
Concentration (log−10 uM)
CYP2C19
Bgal
No_RNA

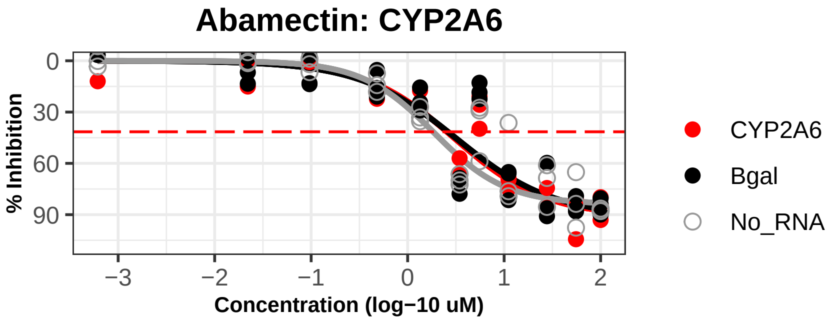
Abamectin: CYP2A6
% Inhibition
Concentration (log−10 uM)
CYP2A6
Bgal
No_RNA

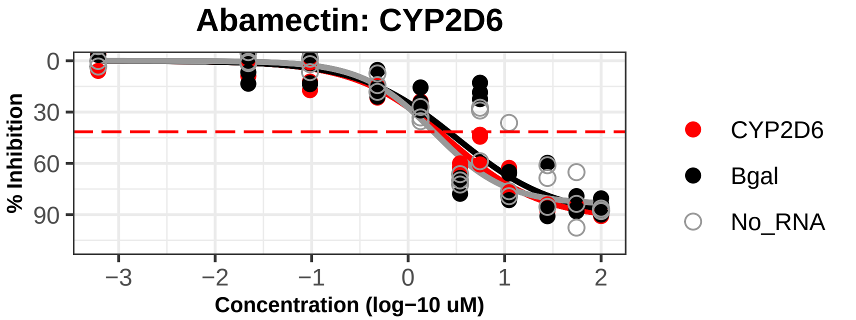
Abamectin: CYP2D6
% Inhibition
Concentration (log−10 uM)
CYP2D6
Bgal
No_RNA

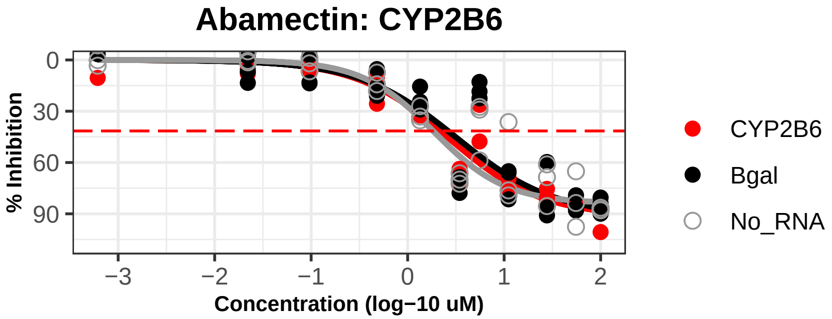
Abamectin: CYP2B6
% Inhibition
Concentration (log−10 uM)
CYP2B6
Bgal
No_RNA

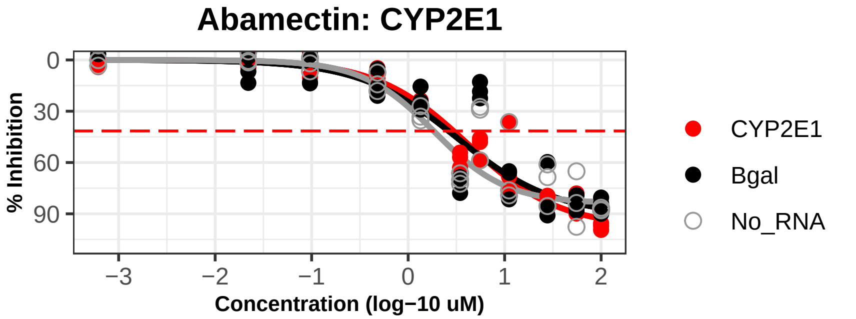
Abamectin: CYP2E1
% Inhibition
Concentration (log−10 uM)
CYP2E1
Bgal
No_RNA

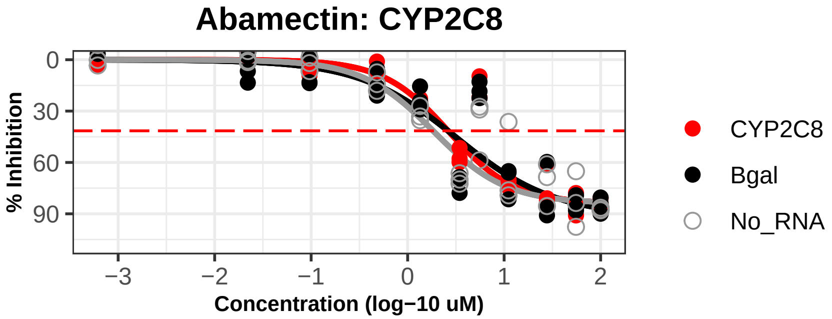
Abamectin: CYP2C8
% Inhibition
Concentration (log−10 uM)
CYP2C8
Bgal
No_RNA

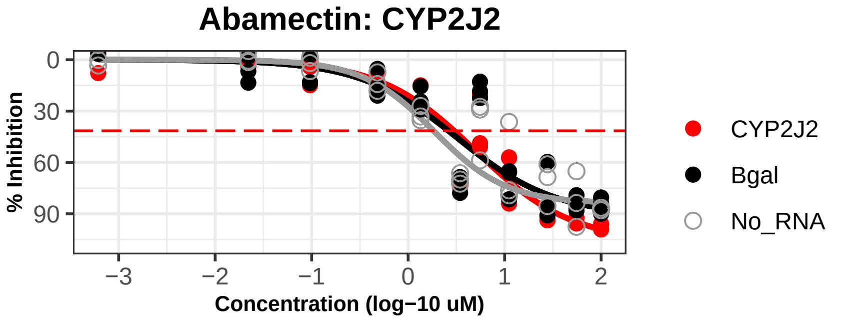
Abamectin: CYP2J2
% Inhibition
Concentration (log−10 uM)
CYP2J2
Bgal
No_RNA

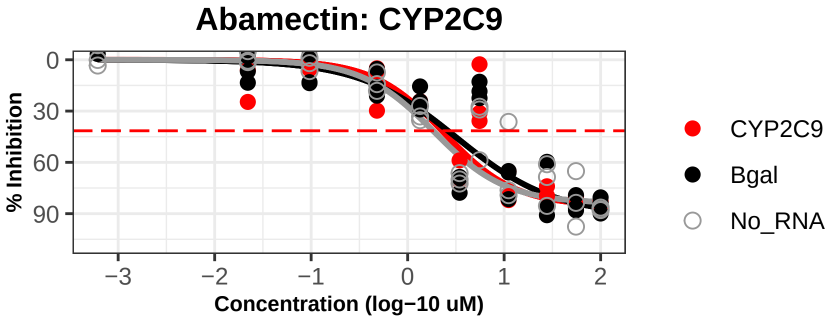
Abamectin: CYP2C9
% Inhibition
Concentration (log−10 uM)
CYP2C9
Bgal
No_RNA

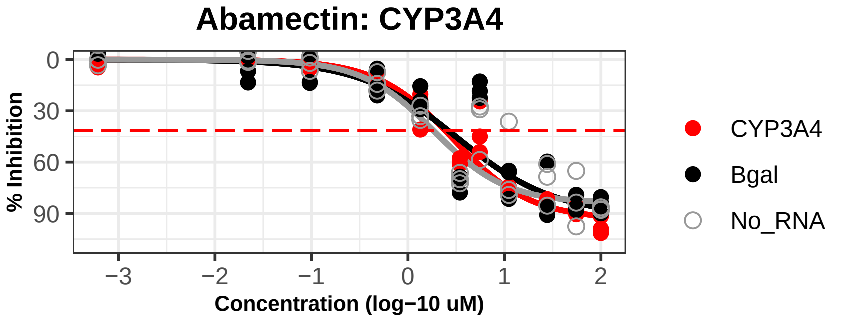
Abamectin: CYP3A4
% Inhibition
Concentration (log−10 uM)
CYP3A4
Bgal
No_RNA

Acephate: CYP1A2

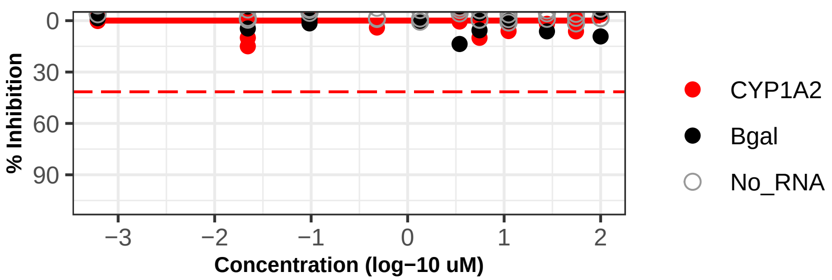

Acephate: CYP2C19

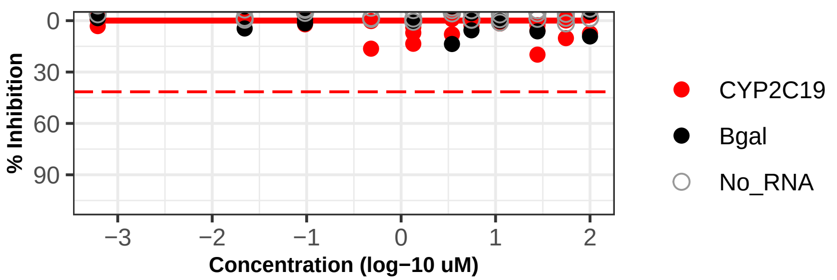

Acephate: CYP2A6

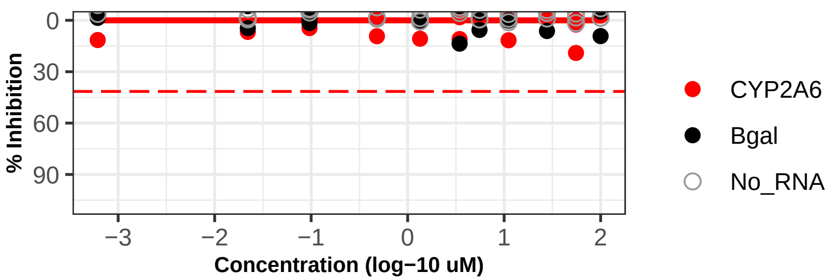

Acephate: CYP2D6

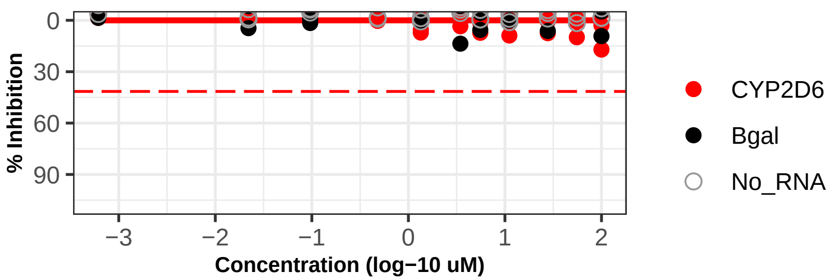

Acephate: CYP2B6

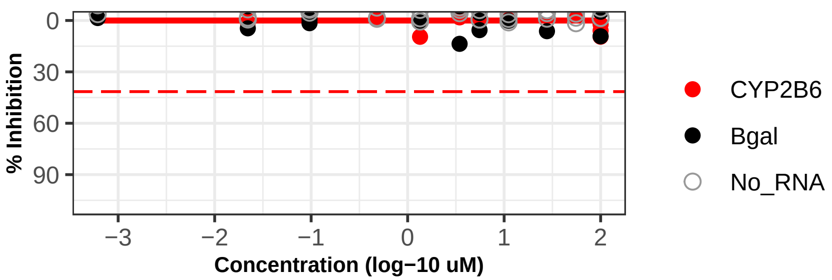

Acephate: CYP2E1

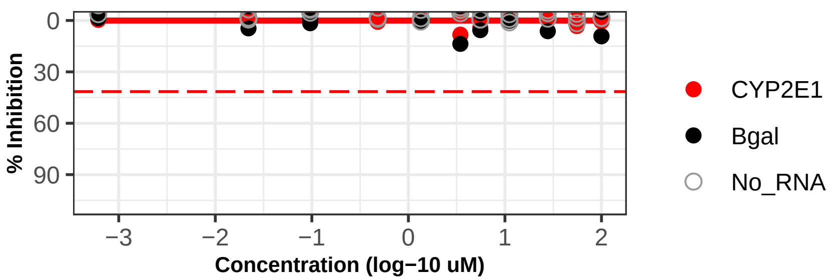

Acephate: CYP2C8

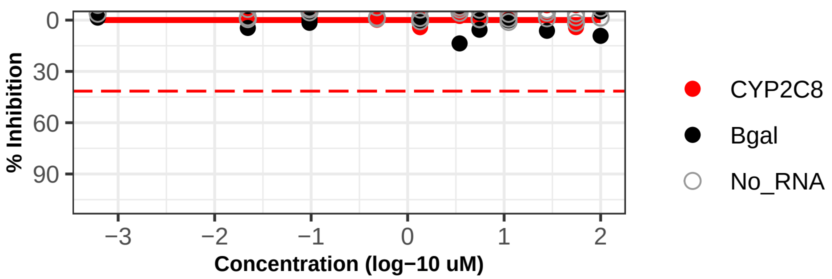

Acephate: CYP2J2

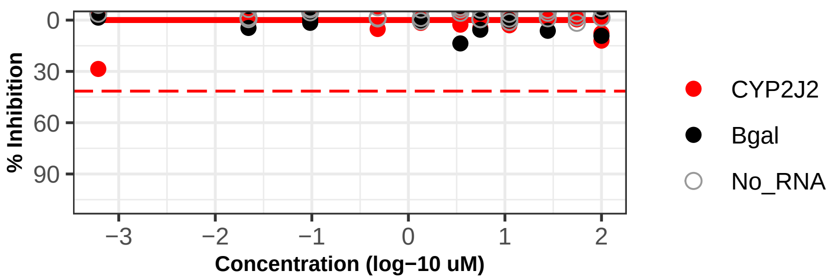

Acephate: CYP2C9

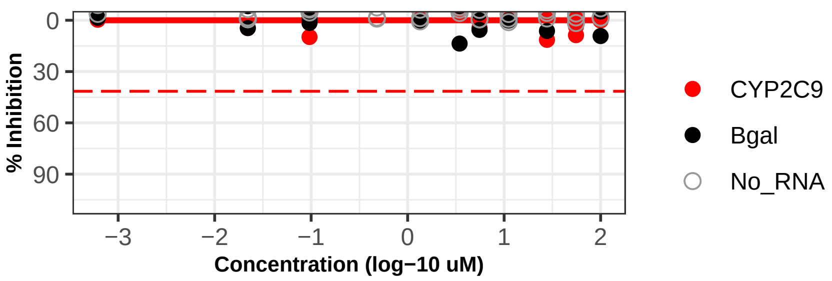

Acephate: CYP3A4

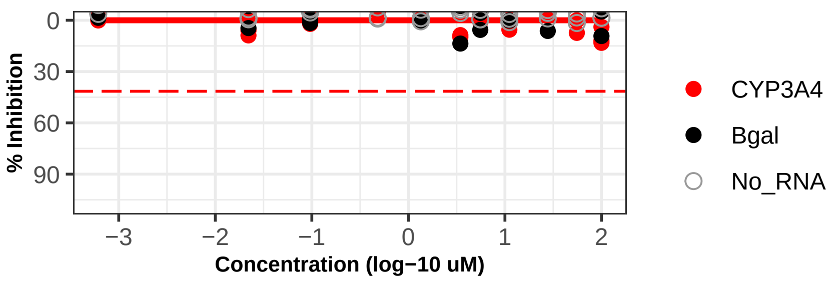

Afimoxifene (4−Hydroxytamoxifen): CYP1A2

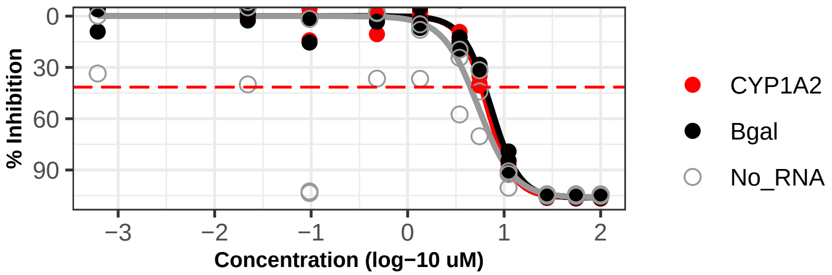

Afimoxifene (4−Hydroxytamoxifen): CYP2C19

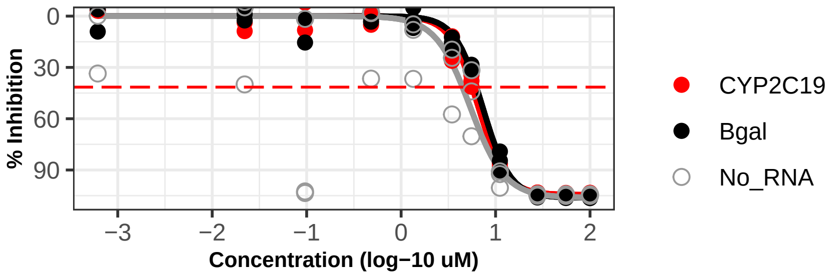

Afimoxifene (4−Hydroxytamoxifen): CYP2A6

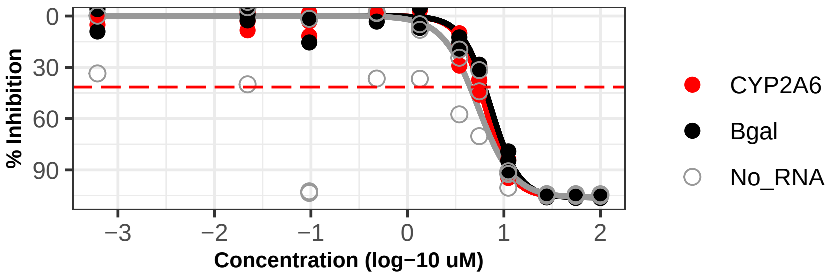

Afimoxifene (4−Hydroxytamoxifen): CYP2D6

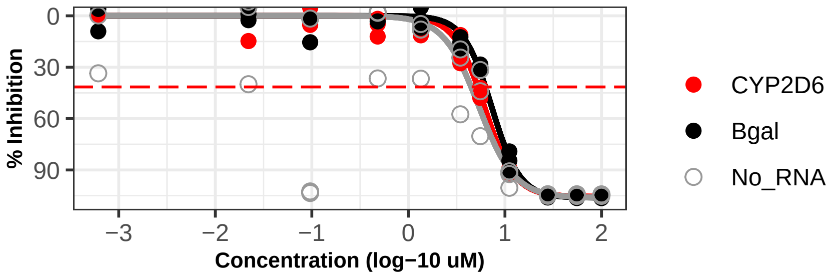

Afimoxifene (4−Hydroxytamoxifen): CYP2B6

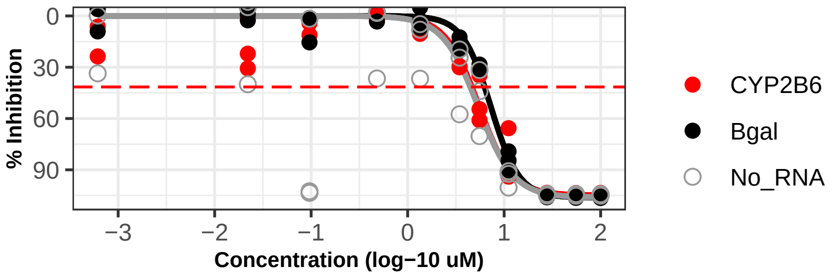

Afimoxifene (4−Hydroxytamoxifen): CYP2E1

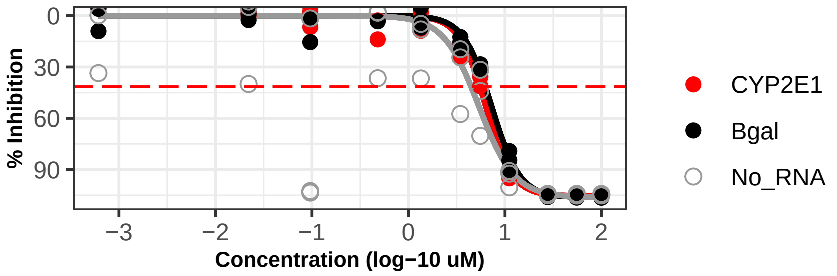

Afimoxifene (4−Hydroxytamoxifen): CYP2C8

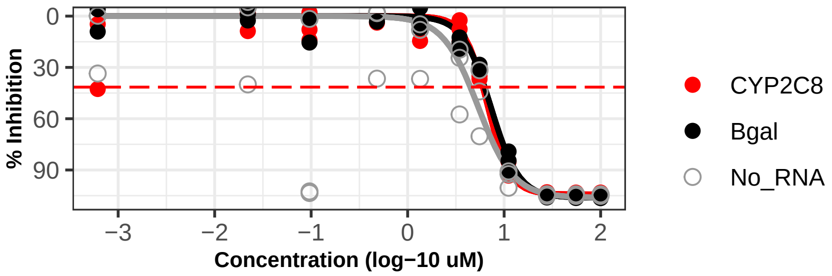

Afimoxifene (4−Hydroxytamoxifen): CYP2J2

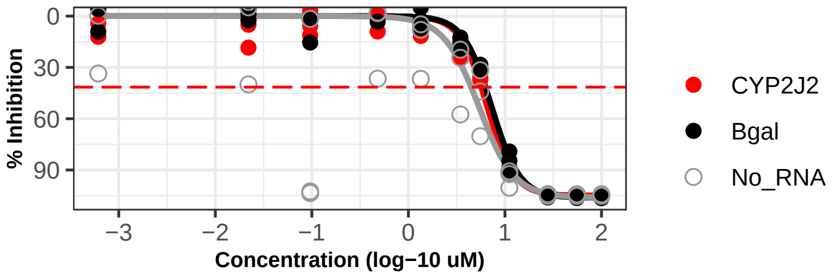

Afimoxifene (4−Hydroxytamoxifen): CYP2C9

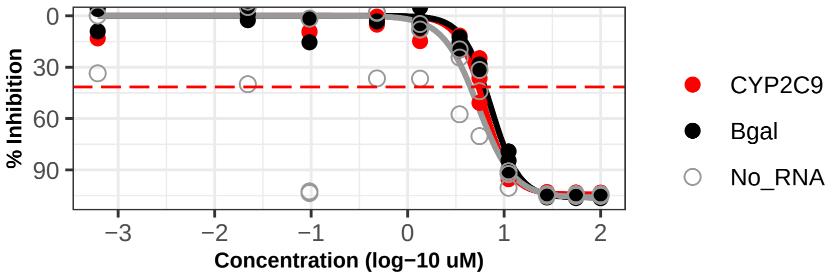

Afimoxifene (4−Hydroxytamoxifen): CYP3A4

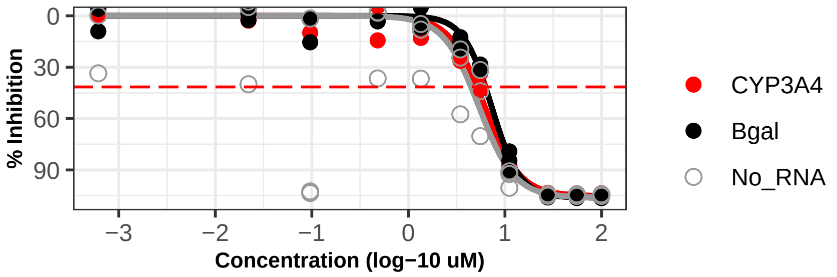

Amitrole: CYP1A2

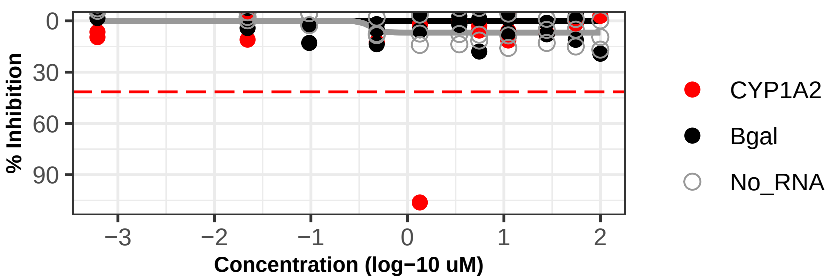

Amitrole: CYP2C19

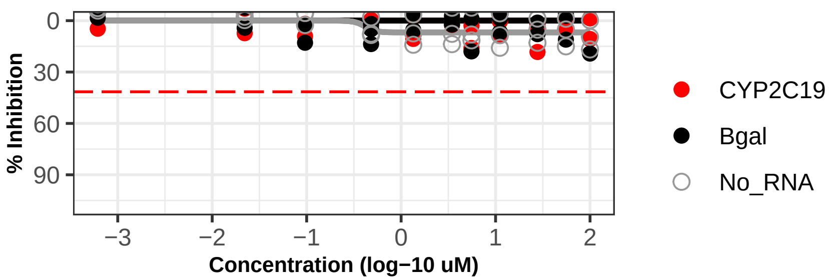

Amitrole: CYP2A6

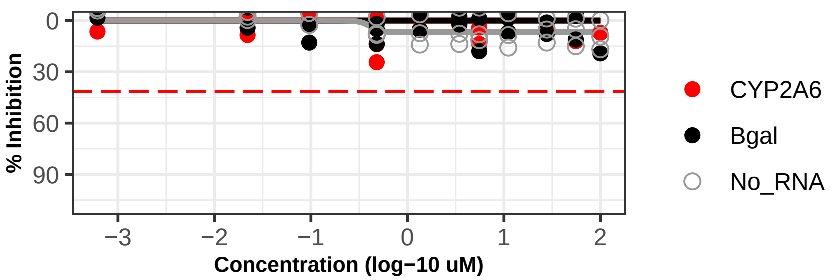

Amitrole: CYP2D6

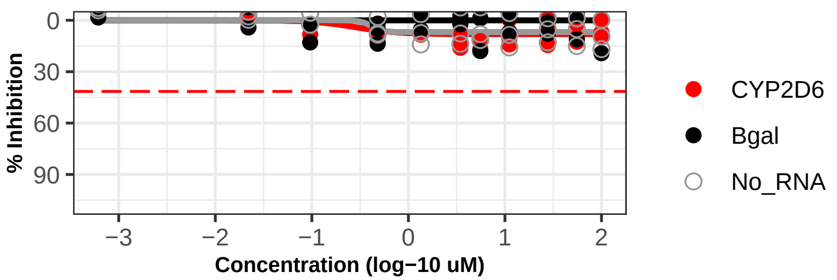

Amitrole: CYP2B6

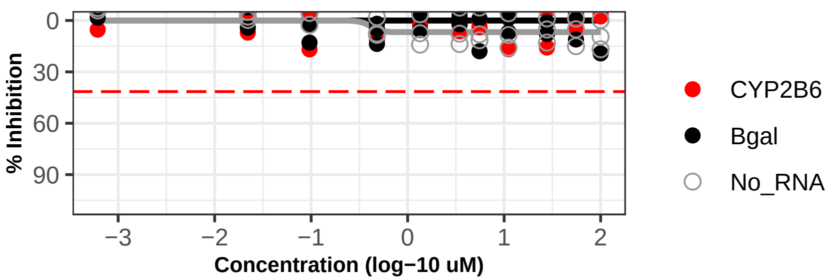

Amitrole: CYP2E1

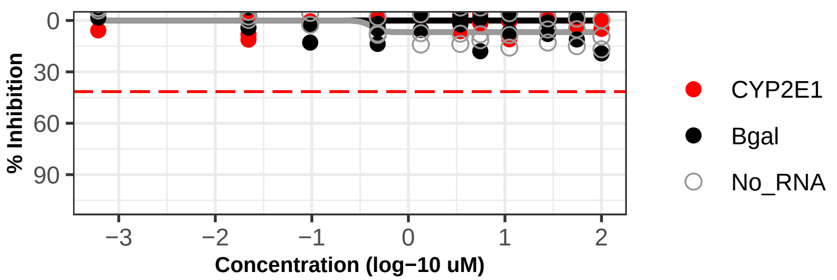

Amitrole: CYP2C8

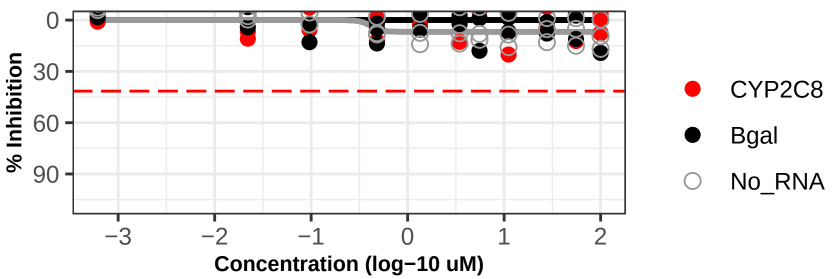

Amitrole: CYP2J2

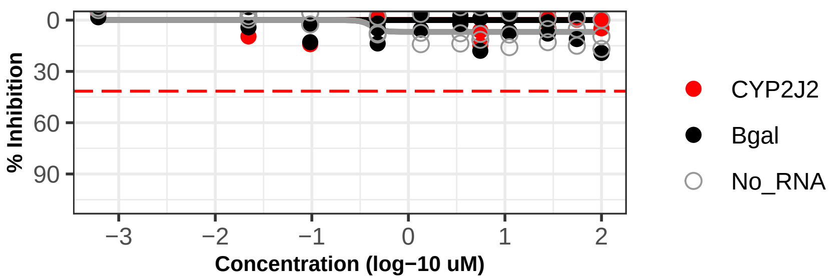

Amitrole: CYP2C9

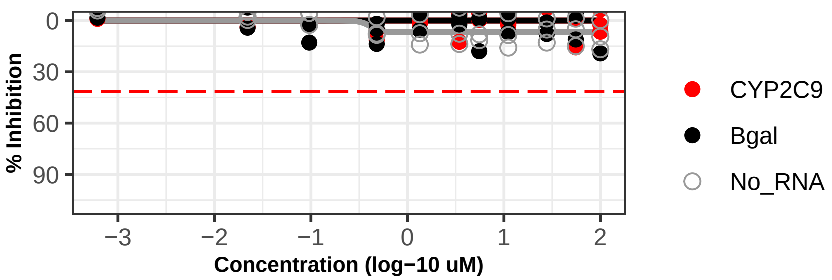

Amitrole: CYP3A4

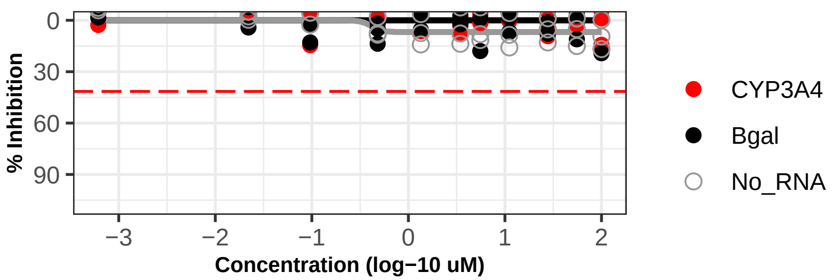

Anastrozole: CYP1A2

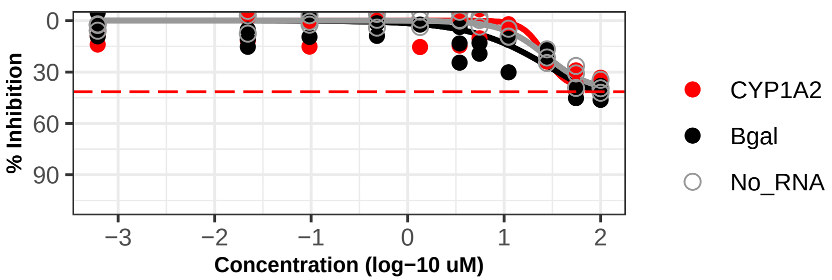

Anastrozole: CYP2C19

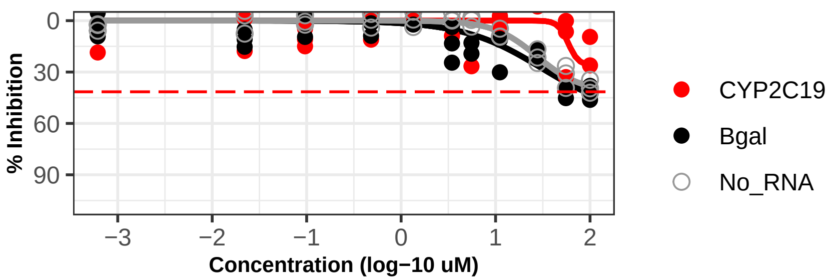

Anastrozole: CYP2A6

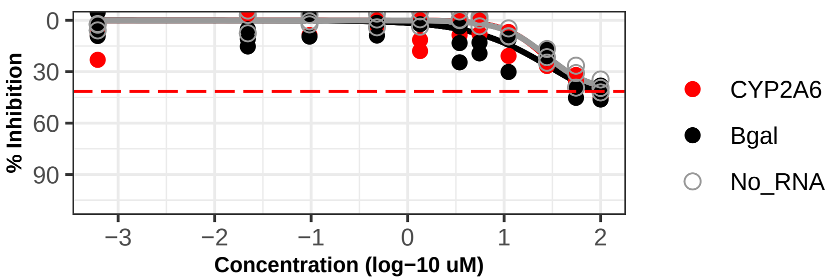

Anastrozole: CYP2D6

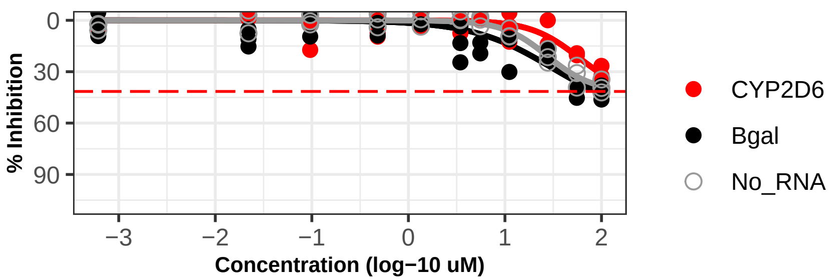

Anastrozole: CYP2B6

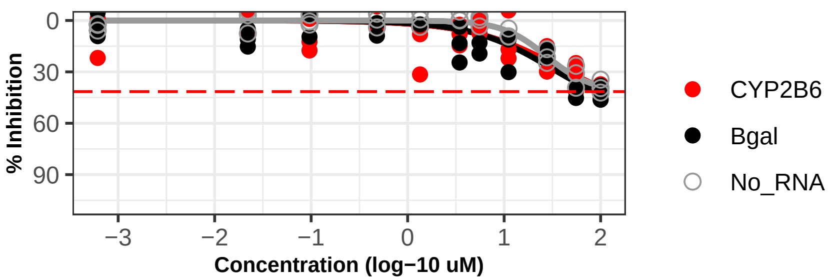

Anastrozole: CYP2E1

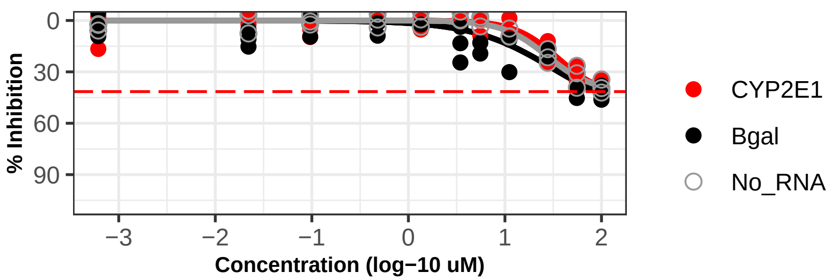

Anastrozole: CYP2C8

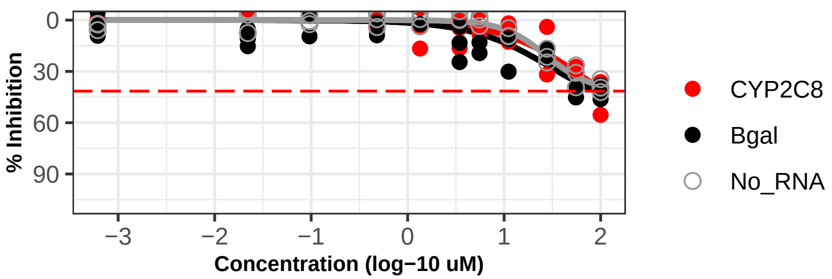

Anastrozole: CYP2J2

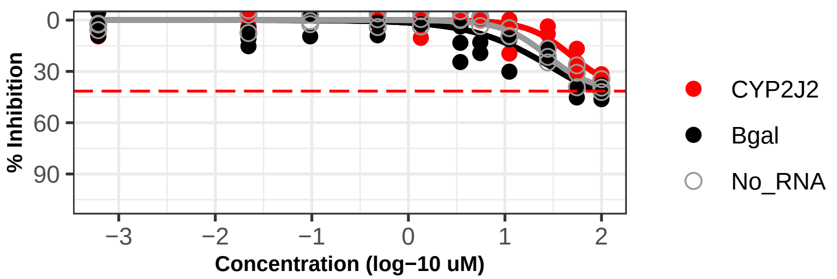

Anastrozole: CYP2C9

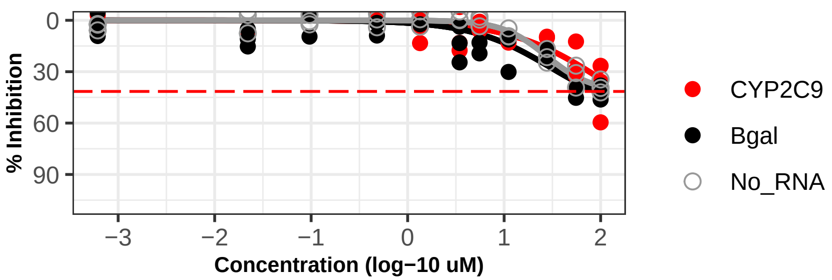

Anastrozole: CYP3A4

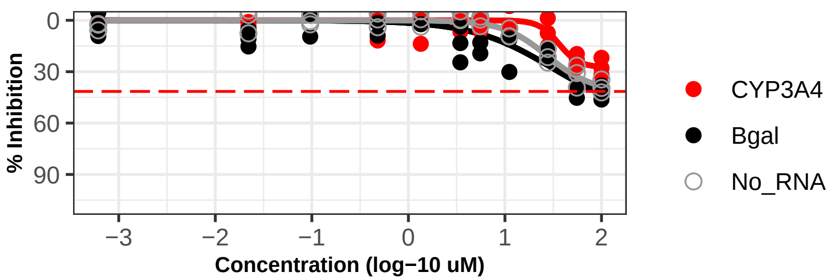

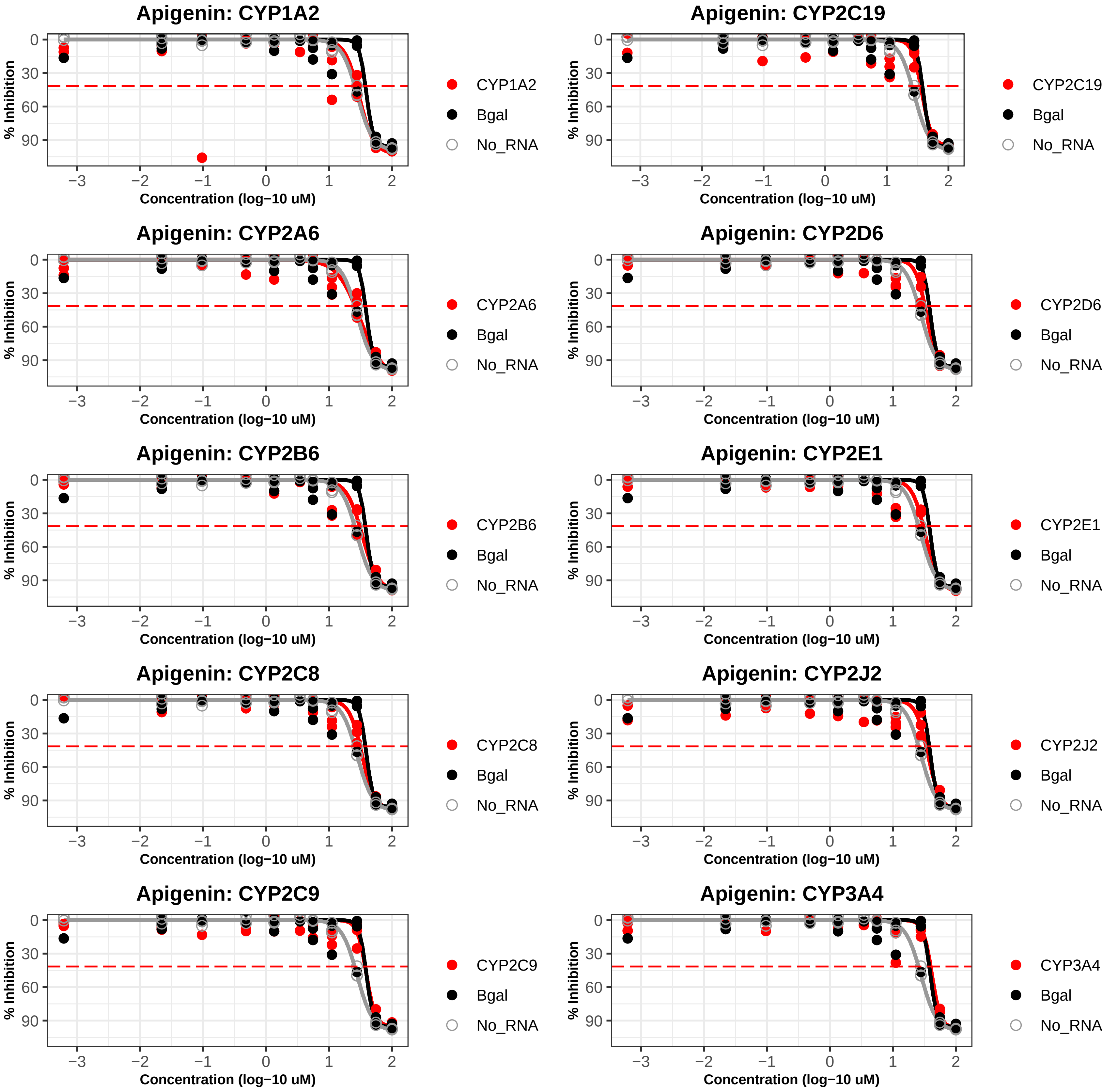
Apigenin: CYP1A2
Apigenin: CYP2C19
Apigenin: CYP2A6
Apigenin: CYP2D6
Apigenin: CYP2B6
Apigenin: CYP2E1
Apigenin: CYP2C8
Apigenin: CYP2J2
Apigenin: CYP2C9
Apigenin: CYP3A4
% Inhibition
Concentration (log−10 uM)
CYP1A2
CYP2C19
CYP2A6
CYP2D6
CYP2B6
CYP2E1
CYP2C8
CYP2J2
CYP2C9
CYP3A4
Bgal
No_RNA

Atrazine: CYP1A2

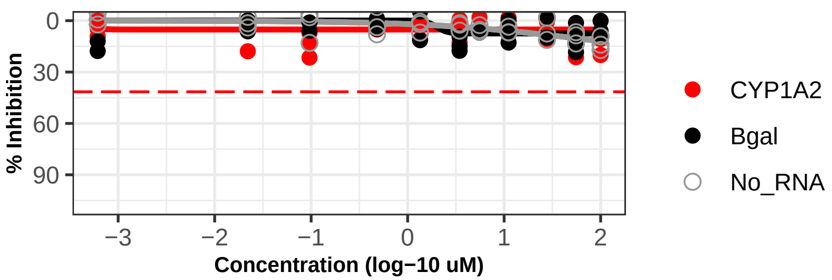

Atrazine: CYP2C19

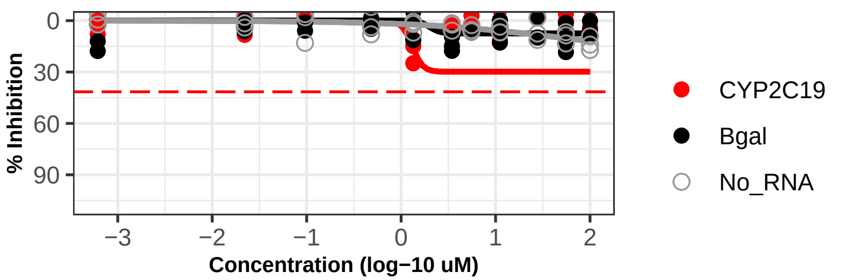

Atrazine: CYP2A6

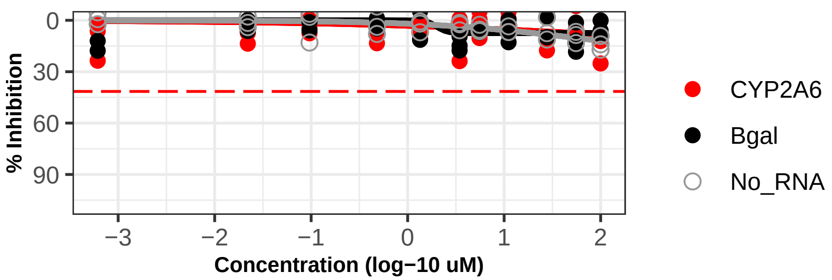

Atrazine: CYP2D6

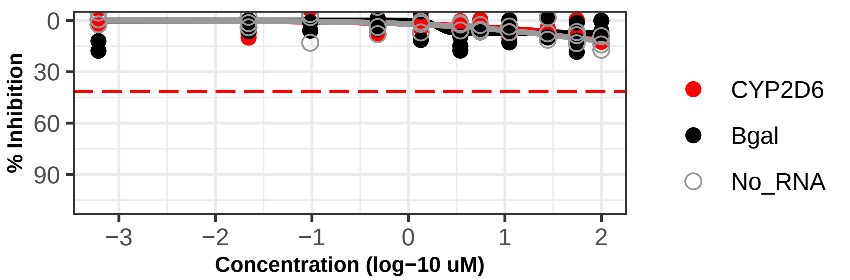

Atrazine: CYP2B6

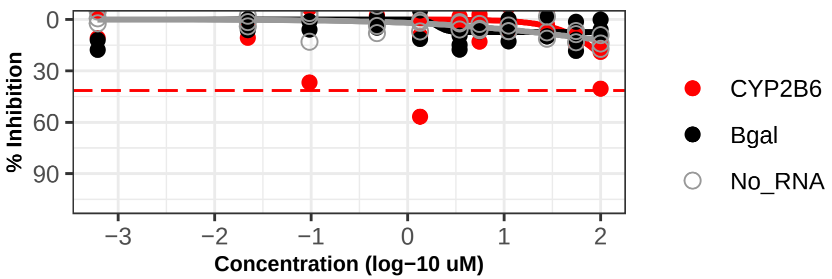

Atrazine: CYP2E1

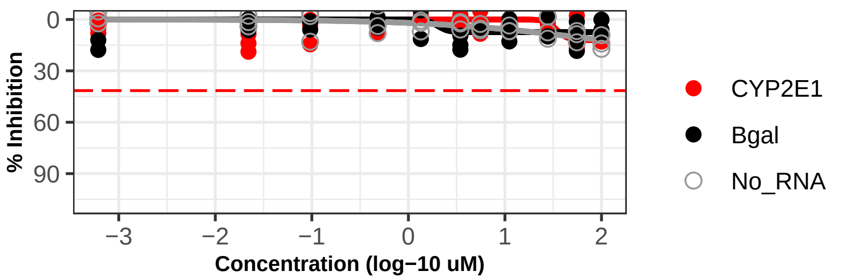

Atrazine: CYP2C8

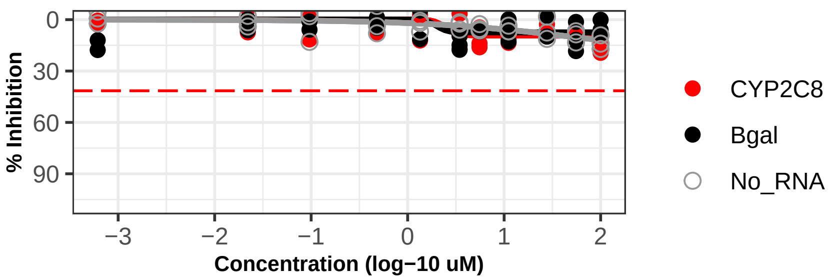

Atrazine: CYP2J2

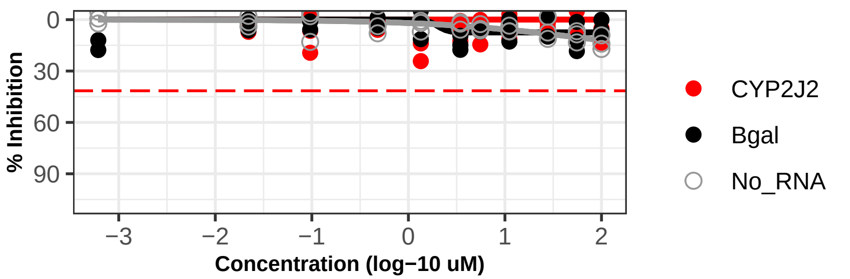

Atrazine: CYP2C9

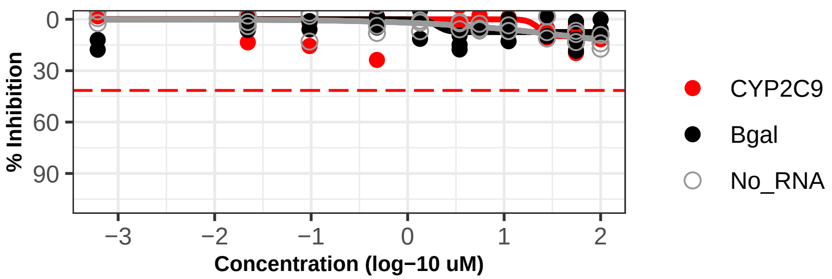

Atrazine: CYP3A4

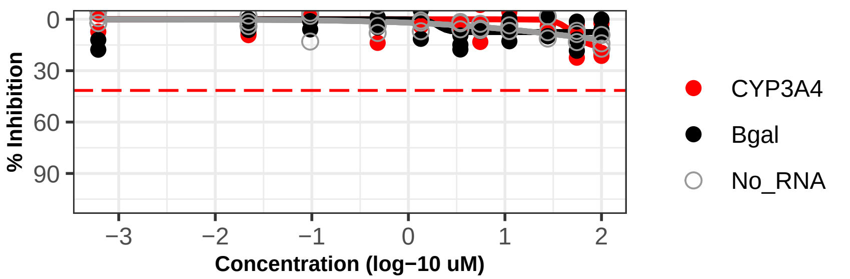

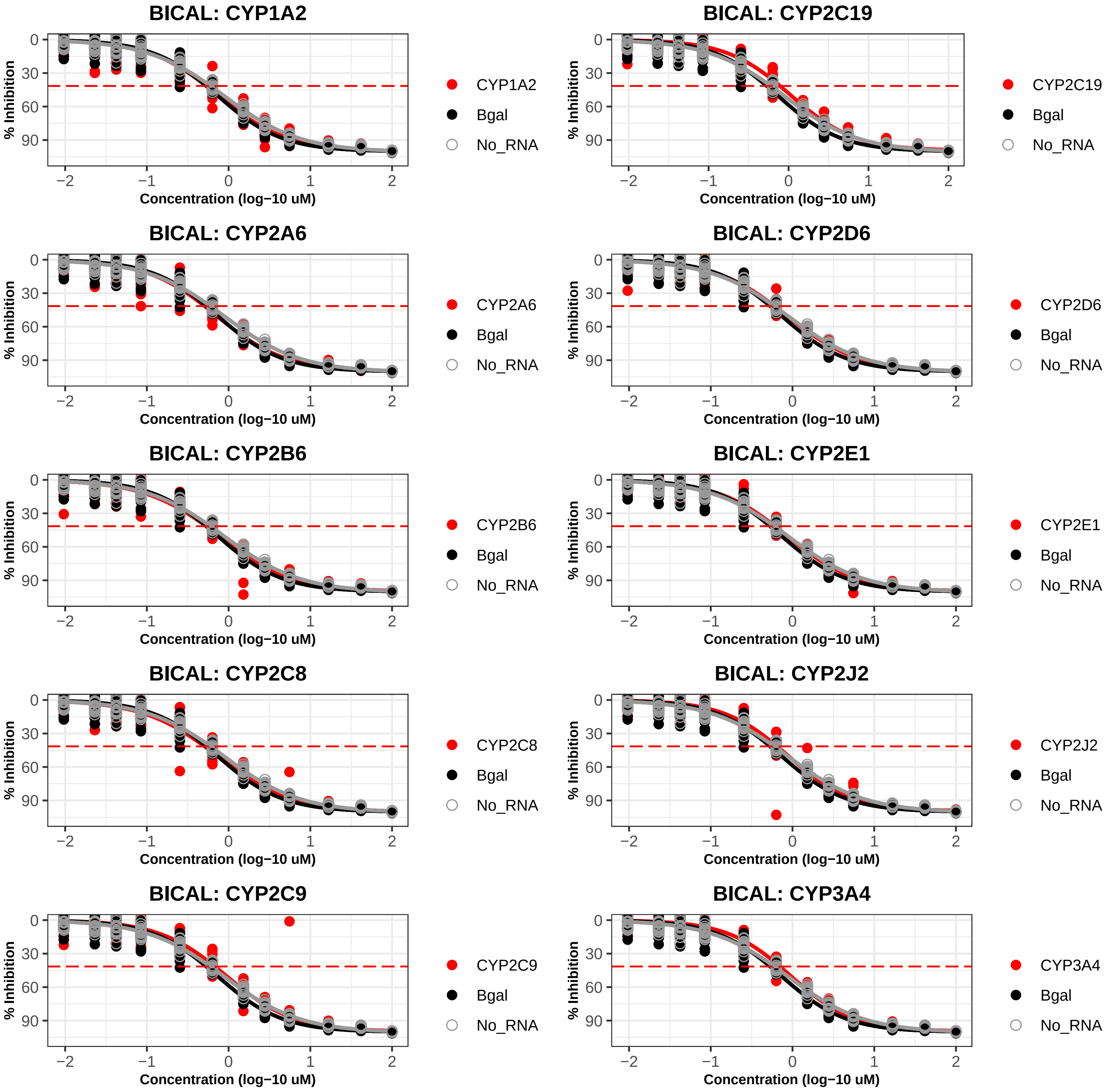
BICAL: CYP1A2
BICAL: CYP2C19
BICAL: CYP2A6
BICAL: CYP2D6
BICAL: CYP2B6
BICAL: CYP2E1
BICAL: CYP2C8
BICAL: CYP2J2
BICAL: CYP2C9
BICAL: CYP3A4
% Inhibition
Concentration (log−10 uM)
Bgal
No_RNA

Benfluralin: CYP1A2

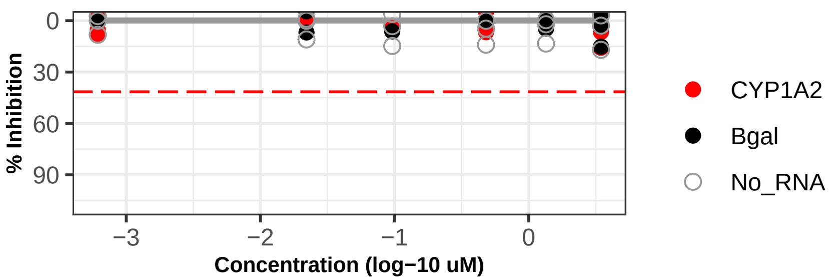

Benfluralin: CYP2C19

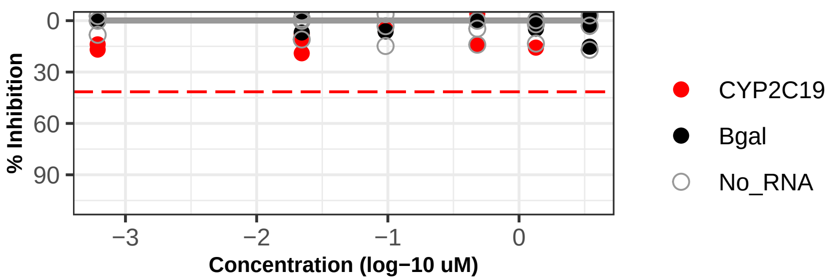

Benfluralin: CYP2A6

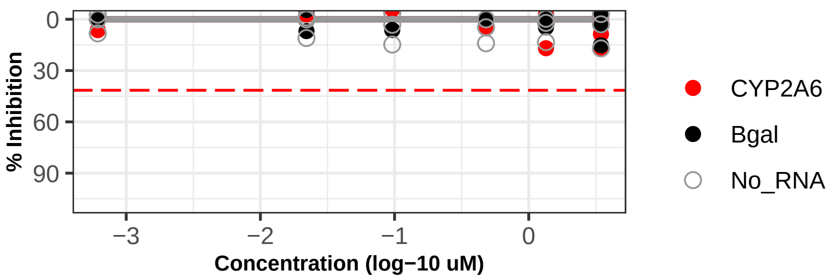

Benfluralin: CYP2D6

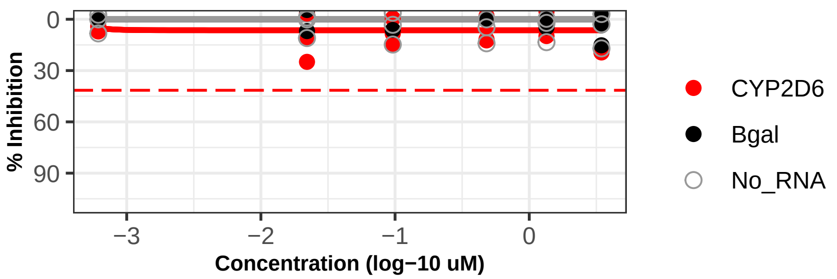

Benfluralin: CYP2B6

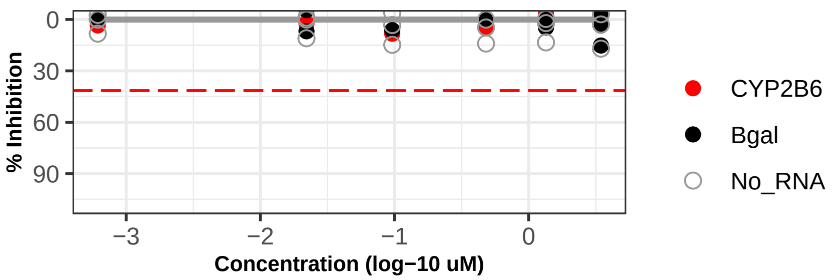

Benfluralin: CYP2E1

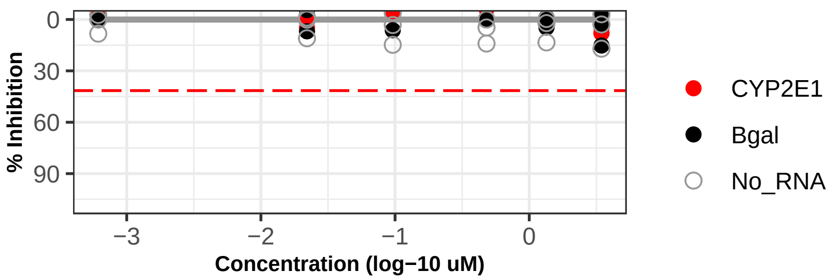

Benfluralin: CYP2C8

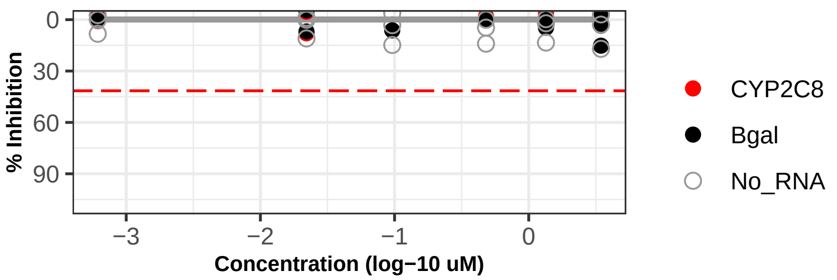

Benfluralin: CYP2J2

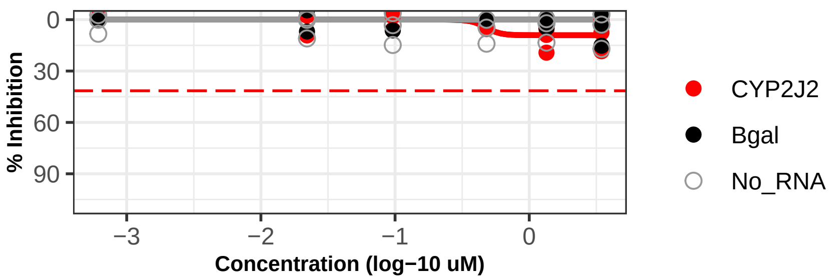

Benfluralin: CYP2C9

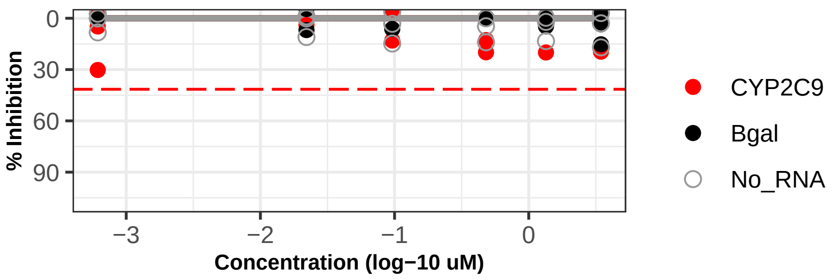

Benfluralin: CYP3A4

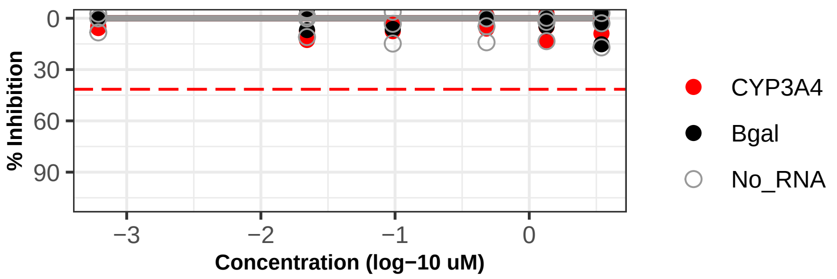

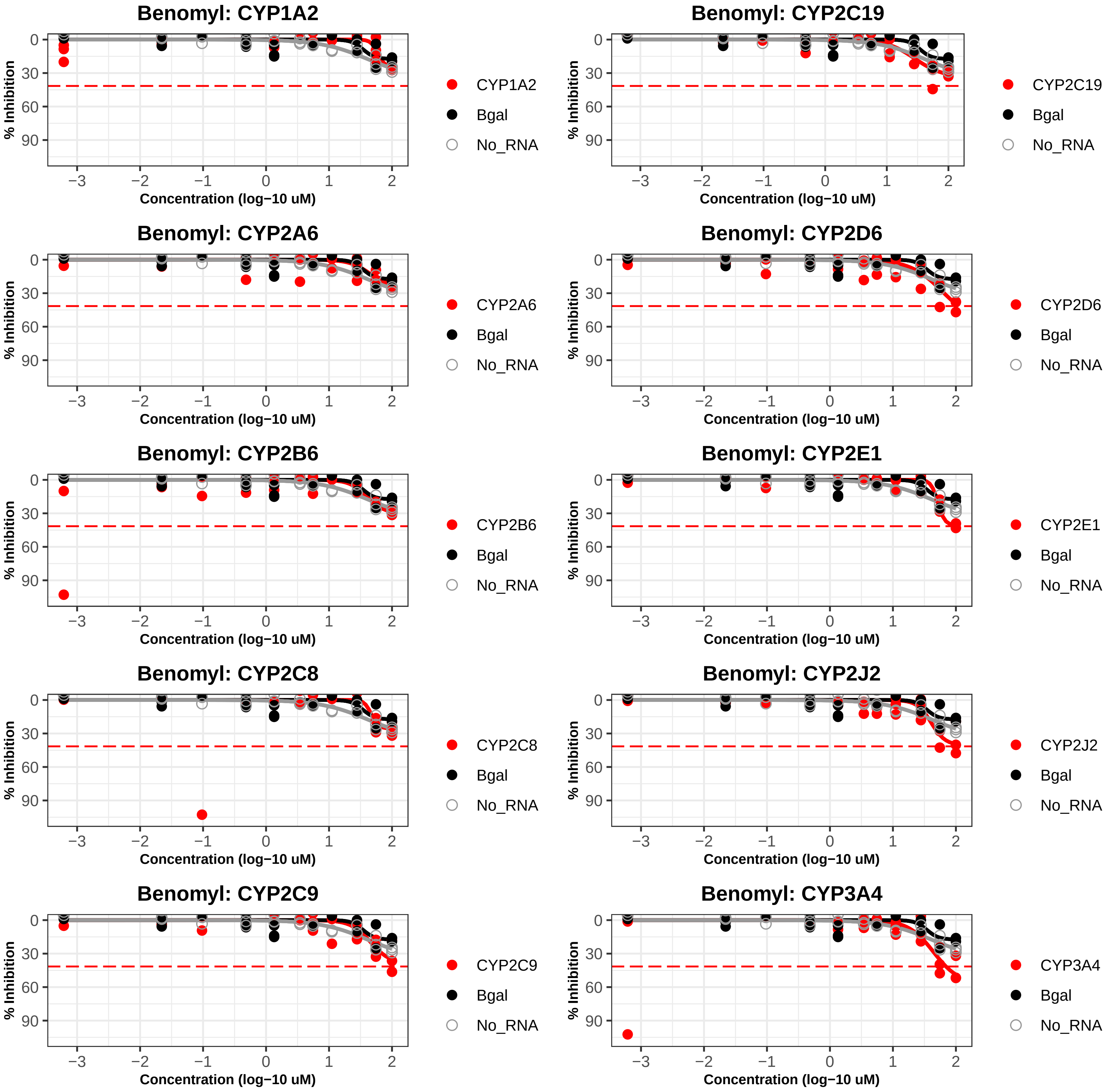
Benomyl: CYP1A2
% Inhibition
0
30
60
90
−3
−2
−1
0
1
2
Concentration (log−10 uM)
CYP1A2
Bgal
No_RNA
Benomyl: CYP2C19
% Inhibition
Concentration (log−10 uM)
CYP2C19
Bgal
No_RNA
Benomyl: CYP2A6
% Inhibition
Concentration (log−10 uM)
CYP2A6
Bgal
No_RNA
Benomyl: CYP2D6
% Inhibition
Concentration (log−10 uM)
CYP2D6
Bgal
No_RNA
Benomyl: CYP2B6
% Inhibition
Concentration (log−10 uM)
CYP2B6
Bgal
No_RNA
Benomyl: CYP2E1
% Inhibition
Concentration (log−10 uM)
CYP2E1
Bgal
No_RNA
Benomyl: CYP2C8
% Inhibition
Concentration (log−10 uM)
CYP2C8
Bgal
No_RNA
Benomyl: CYP2J2
% Inhibition
Concentration (log−10 uM)
CYP2J2
Bgal
No_RNA
Benomyl: CYP2C9
% Inhibition
Concentration (log−10 uM)
CYP2C9
Bgal
No_RNA
Benomyl: CYP3A4
% Inhibition
Concentration (log−10 uM)
CYP3A4
Bgal
No_RNA

Bicalutamide: CYP1A2

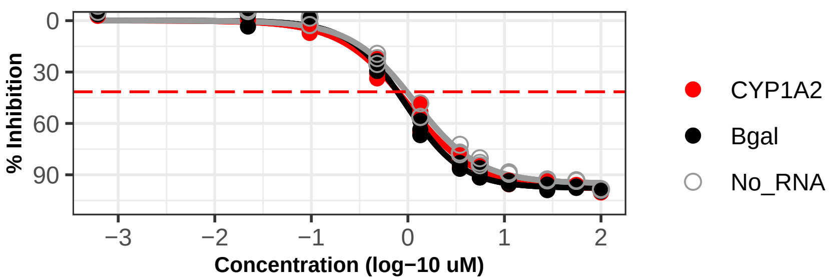

Bicalutamide: CYP2C19

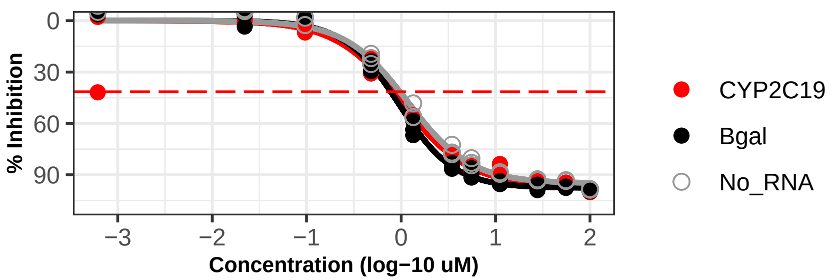

Bicalutamide: CYP2A6

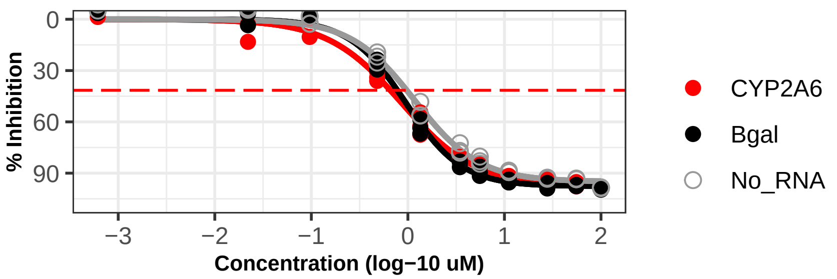

Bicalutamide: CYP2D6

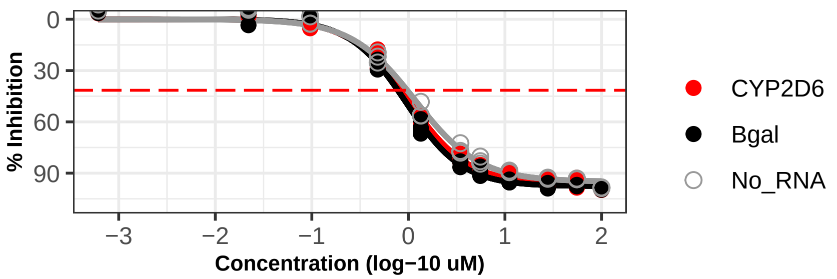

Bicalutamide: CYP2B6

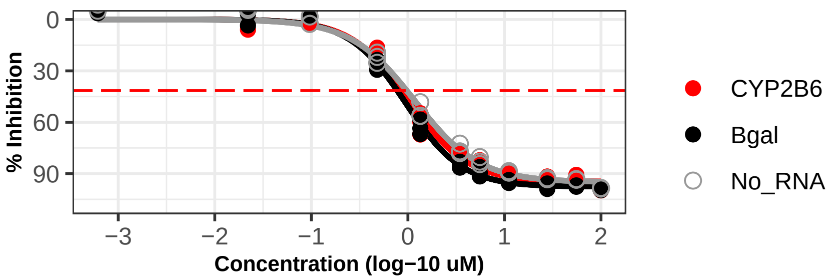

Bicalutamide: CYP2E1

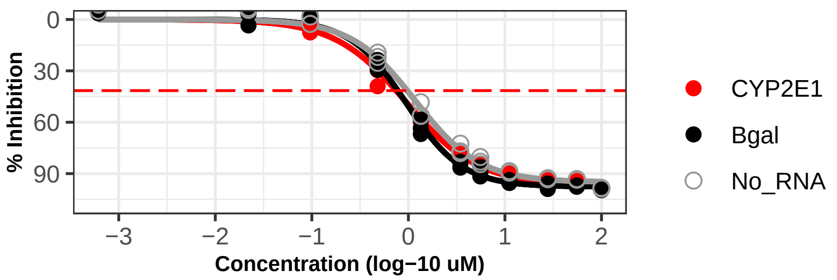

Bicalutamide: CYP2C8

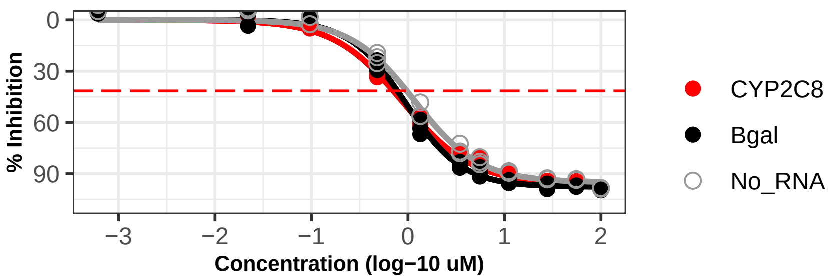

Bicalutamide: CYP2J2

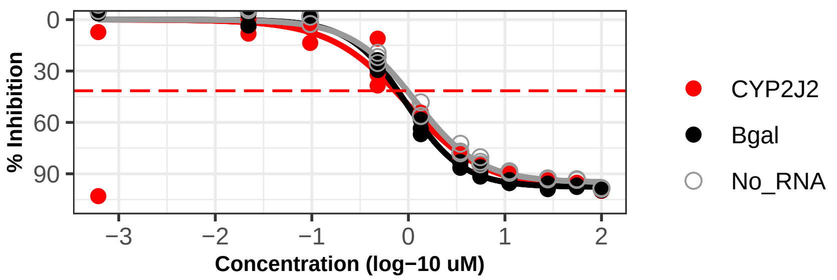

Bicalutamide: CYP2C9

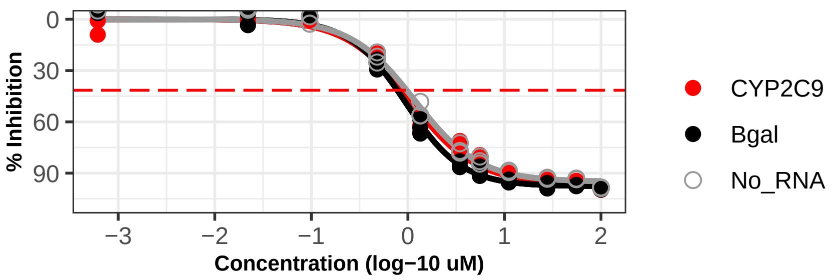

Bicalutamide: CYP3A4

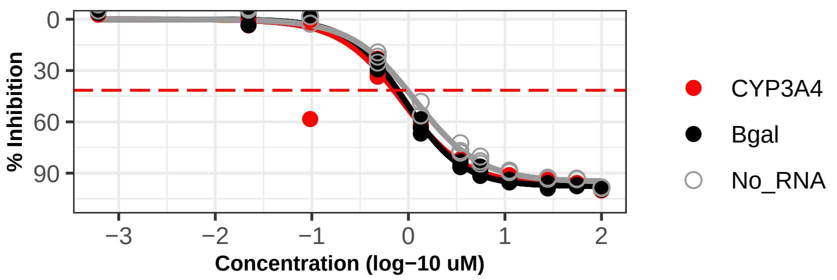

Bifenthrin: CYP1A2

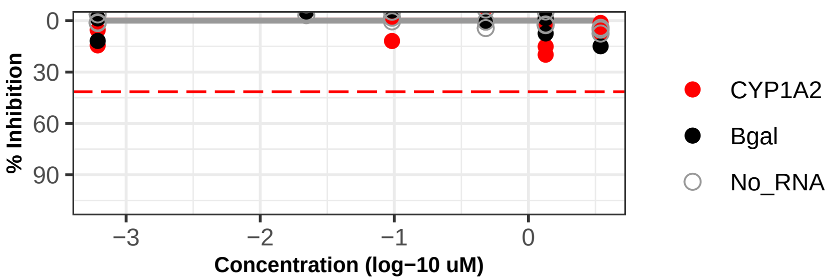

Bifenthrin: CYP2C19

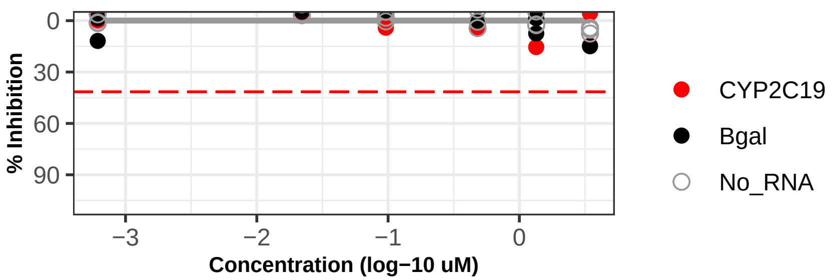

Bifenthrin: CYP2A6

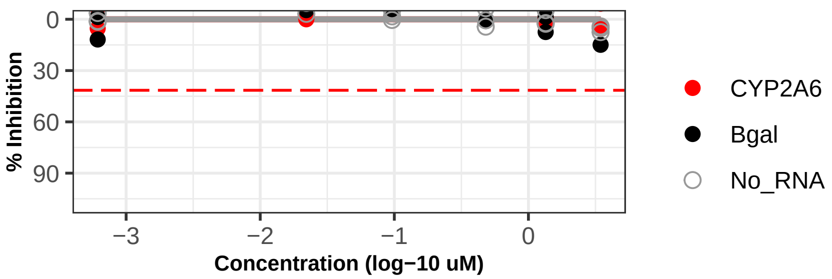

Bifenthrin: CYP2D6

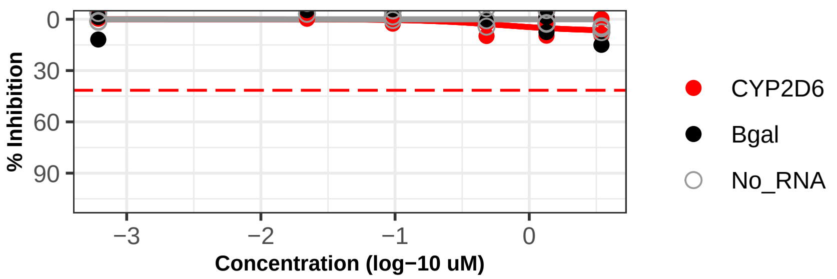

Bifenthrin: CYP2B6

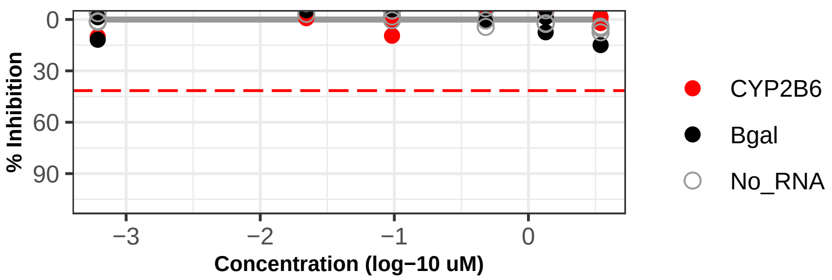

Bifenthrin: CYP2E1

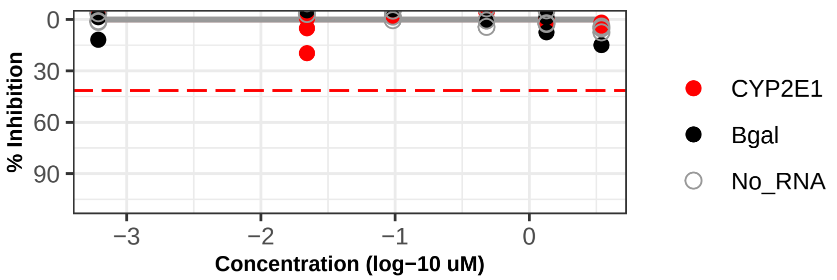

Bifenthrin: CYP2C8

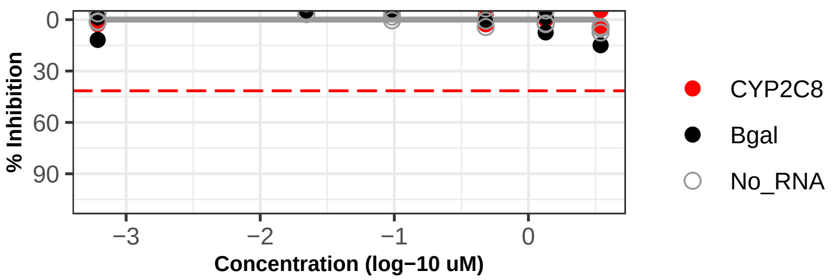

Bifenthrin: CYP2J2

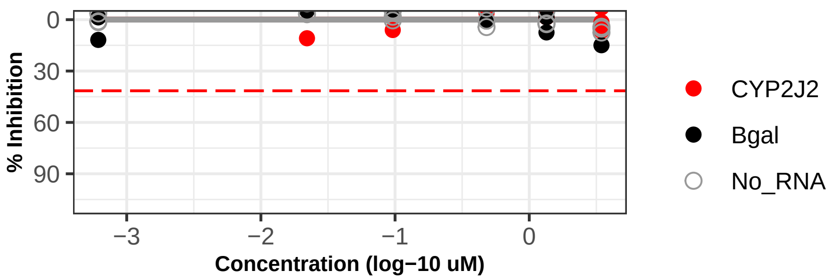

Bifenthrin: CYP2C9

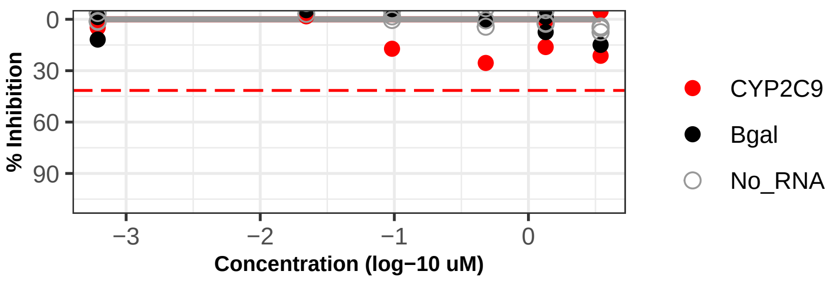

Bifenthrin: CYP3A4

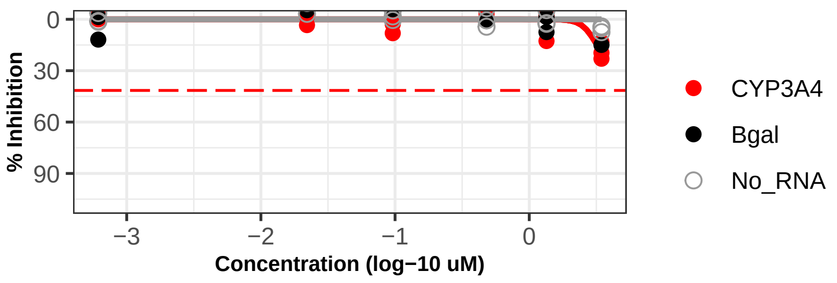

Bis(2−ethylhexyl)hexanedioate: CYP1A2

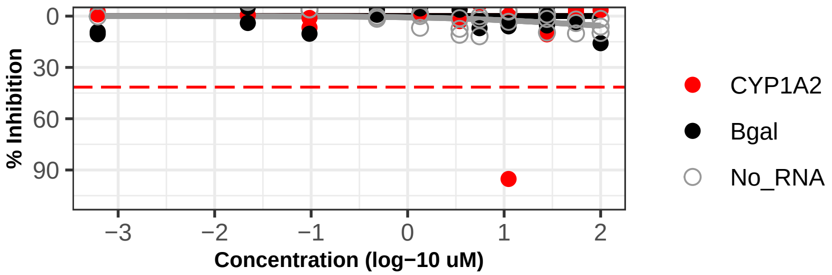

Bis(2−ethylhexyl)hexanedioate: CYP2C19

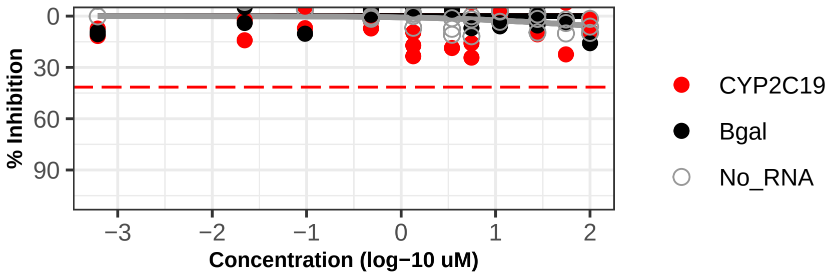

Bis(2−ethylhexyl)hexanedioate: CYP2A6

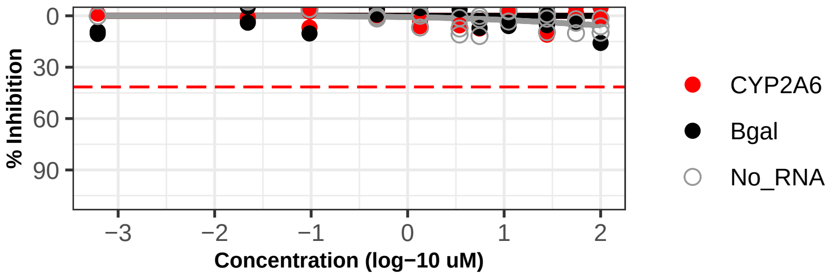

Bis(2−ethylhexyl)hexanedioate: CYP2D6

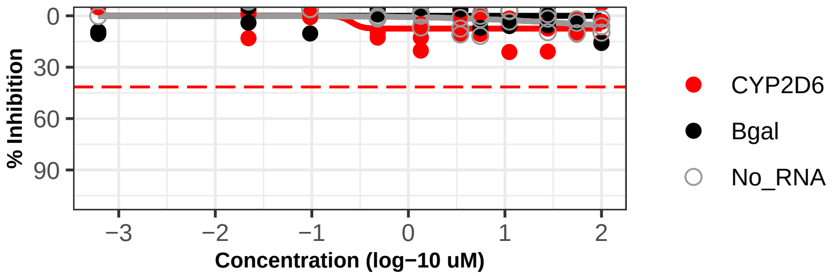

Bis(2−ethylhexyl)hexanedioate: CYP2B6

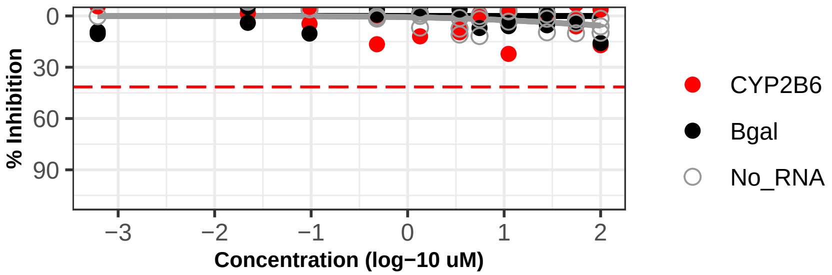

Bis(2−ethylhexyl)hexanedioate: CYP2E1

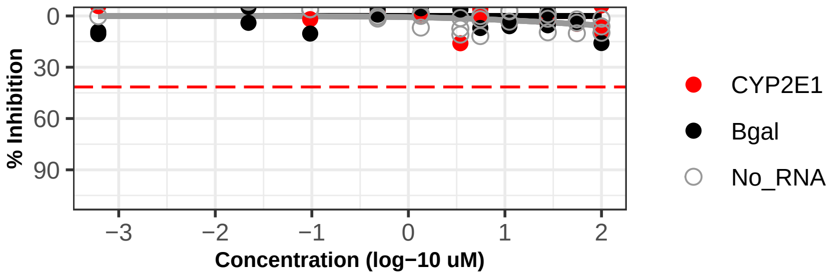

Bis(2−ethylhexyl)hexanedioate: CYP2C8

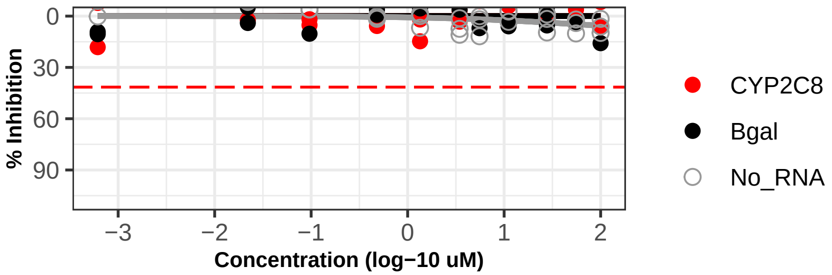

Bis(2−ethylhexyl)hexanedioate: CYP2J2

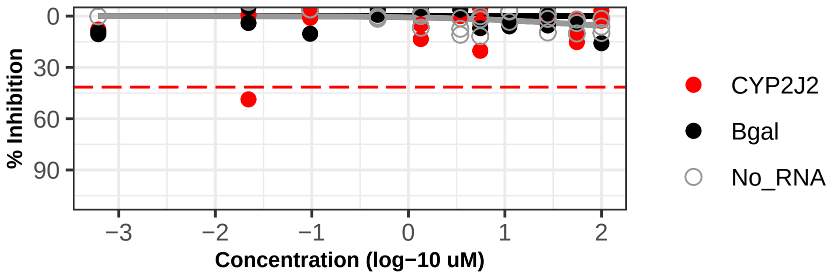

Bis(2−ethylhexyl)hexanedioate: CYP2C9

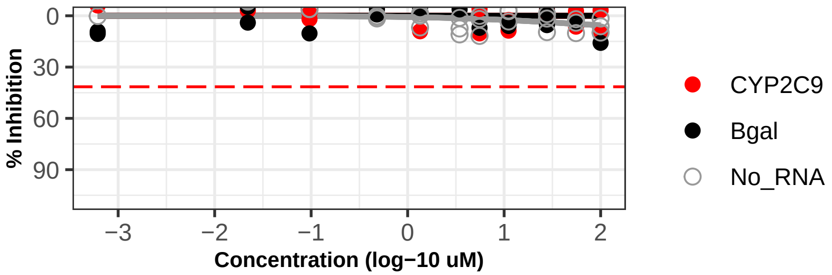

Bis(2−ethylhexyl)hexanedioate: CYP3A4

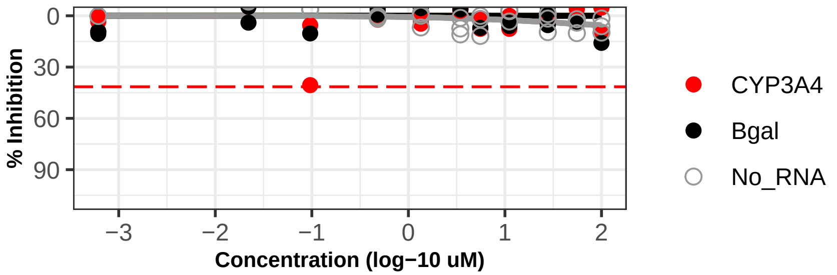

Bis(2−ethylhexyl)phthalate: CYP1A2

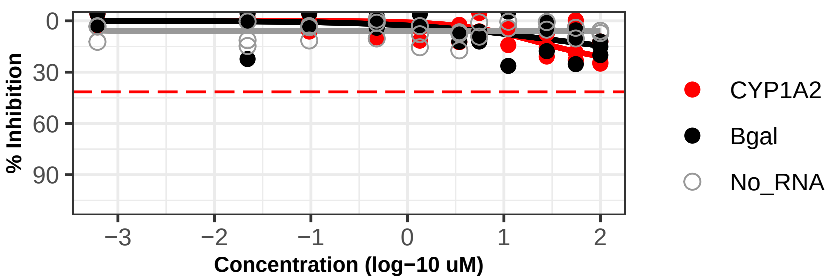

Bis(2−ethylhexyl)phthalate: CYP2C19

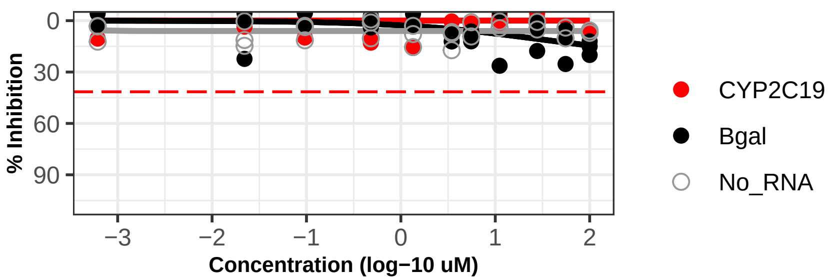

Bis(2−ethylhexyl)phthalate: CYP2A6

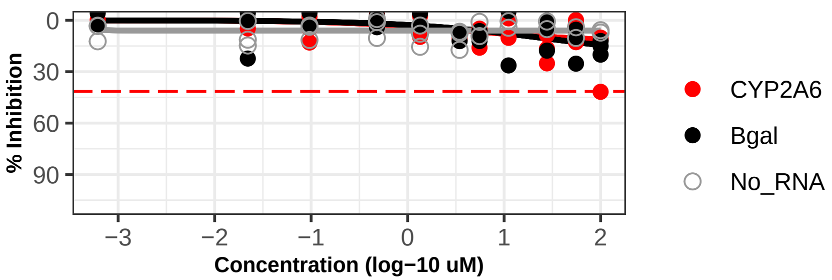

Bis(2−ethylhexyl)phthalate: CYP2D6

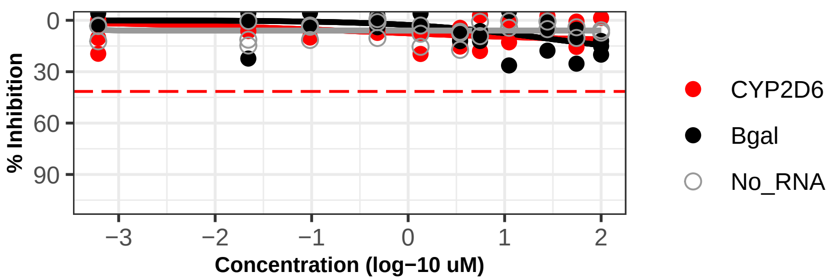

Bis(2−ethylhexyl)phthalate: CYP2B6

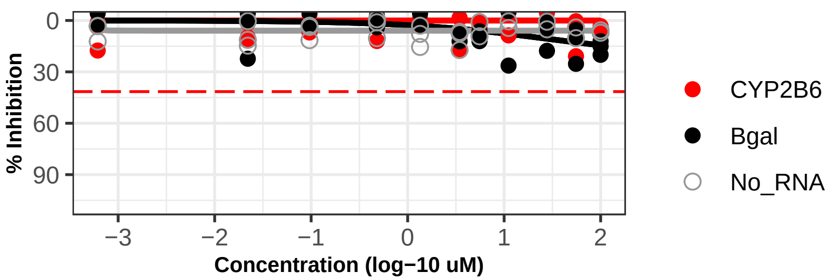

Bis(2−ethylhexyl)phthalate: CYP2E1

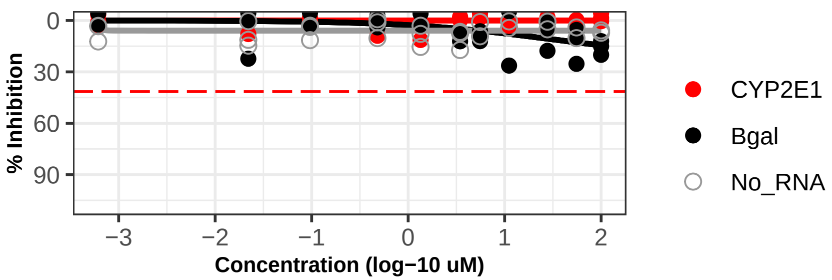

Bis(2−ethylhexyl)phthalate: CYP2C8

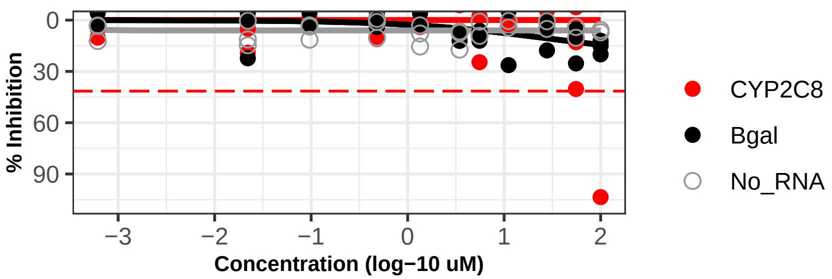

Bis(2−ethylhexyl)phthalate: CYP2J2

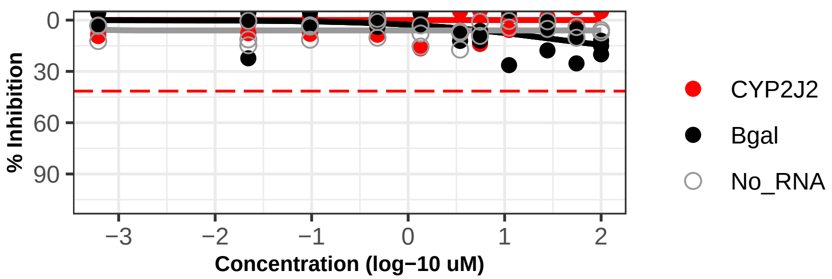

Bis(2−ethylhexyl)phthalate: CYP2C9

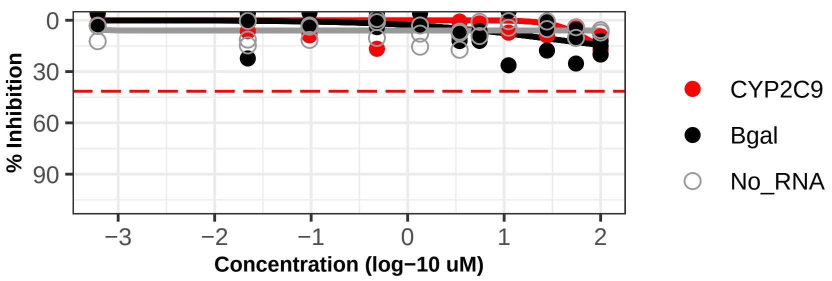

Bis(2−ethylhexyl)phthalate: CYP3A4

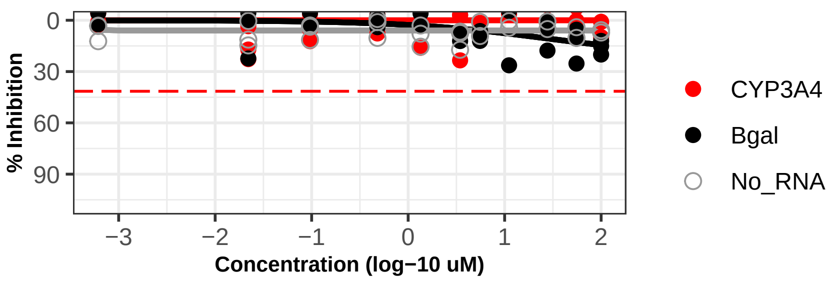

Bisphenol A: CYP1A2

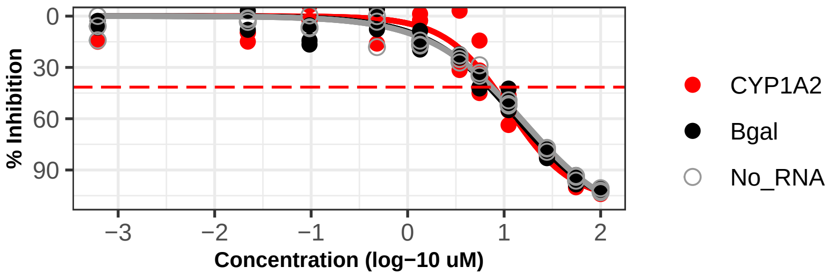

Bisphenol A: CYP2C19

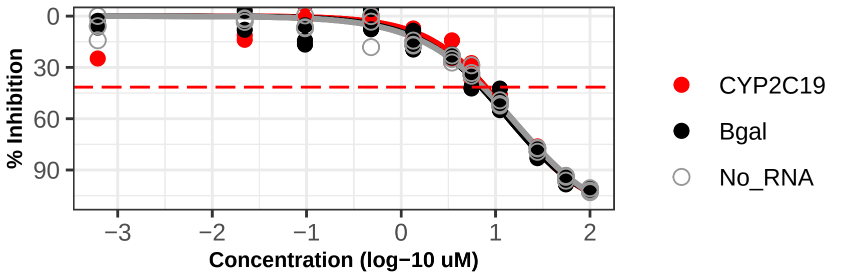

Bisphenol A: CYP2A6

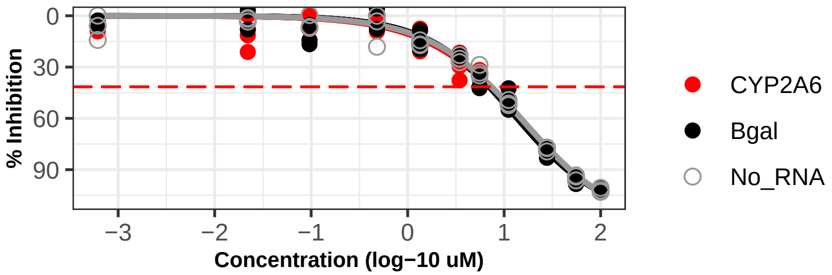

Bisphenol A: CYP2D6

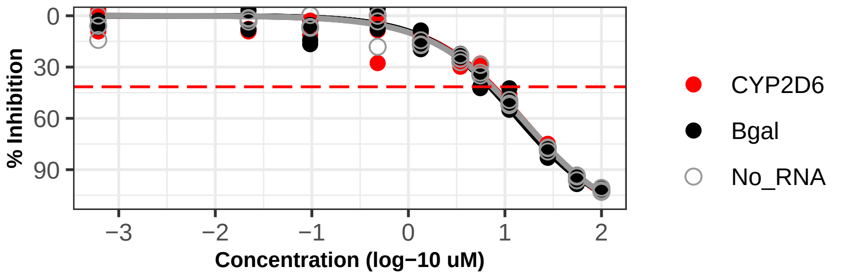

Bisphenol A: CYP2B6

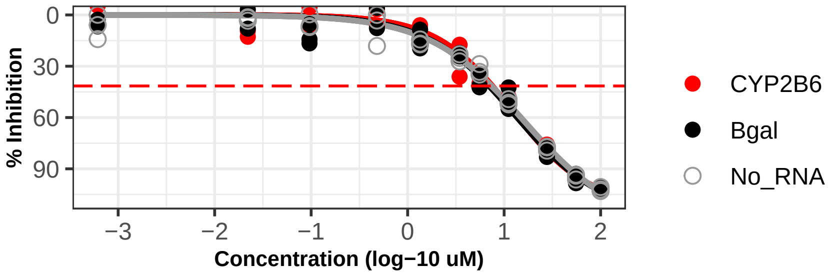

Bisphenol A: CYP2E1

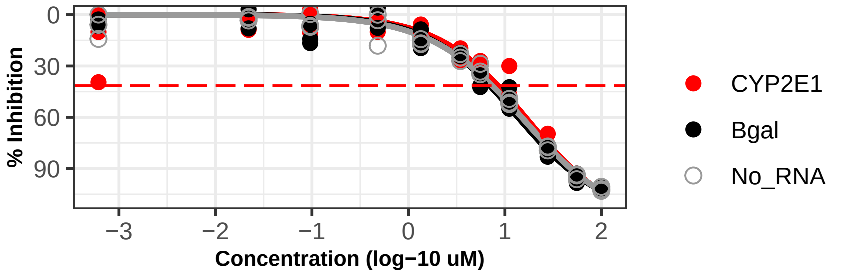

Bisphenol A: CYP2C8

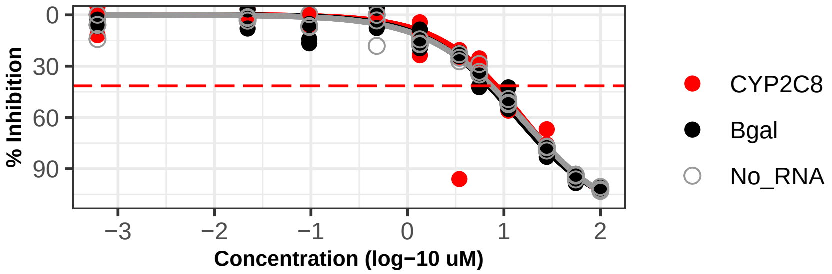

Bisphenol A: CYP2J2

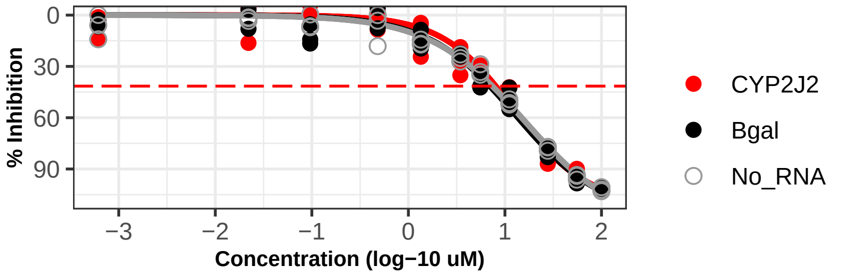

Bisphenol A: CYP2C9

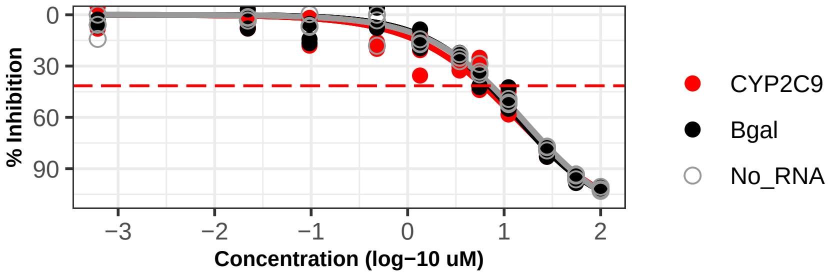

Bisphenol A: CYP3A4

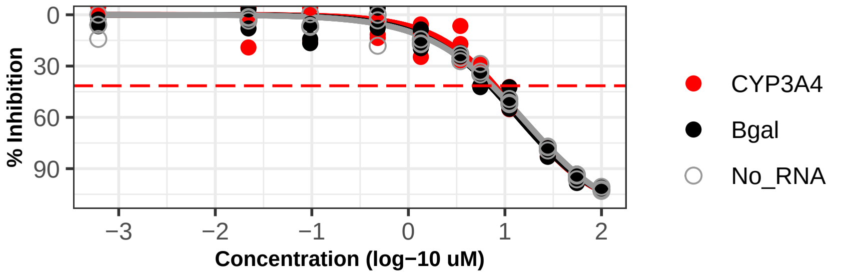

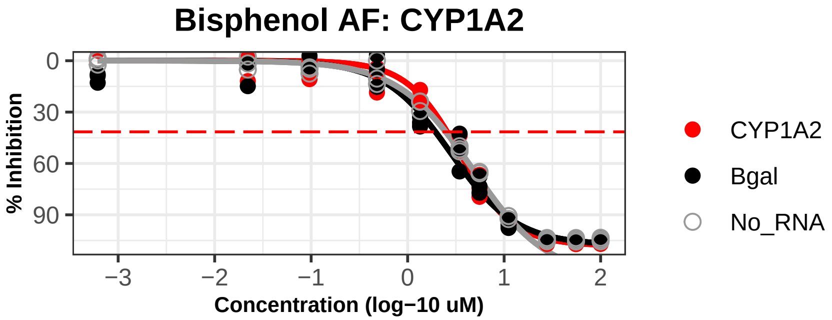
Bisphenol AF: CYP1A2
% Inhibition
0
30
60
90
−3
−2
−1
0
1
2
Concentration (log−10 uM)
CYP1A2
Bgal
No_RNA

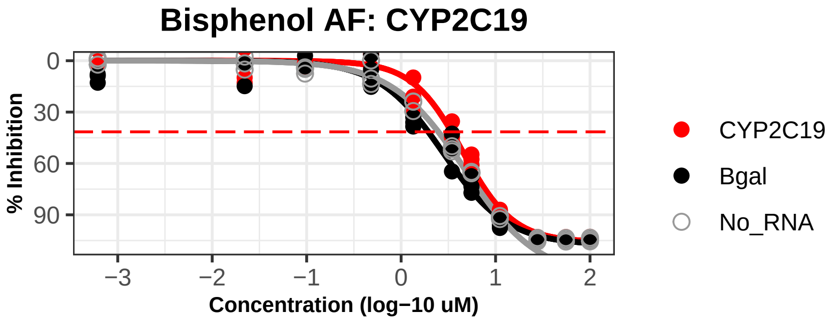
Bisphenol AF: CYP2C19
% Inhibition
0
30
60
90
−3
−2
−1
0
1
2
Concentration (log−10 uM)
CYP2C19
Bgal
No_RNA

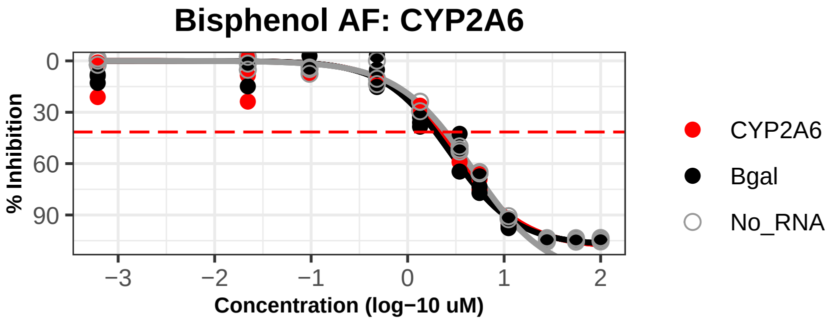
Bisphenol AF: CYP2A6
% Inhibition
0
30
60
90
−3
−2
−1
0
1
2
Concentration (log−10 uM)
CYP2A6
Bgal
No_RNA

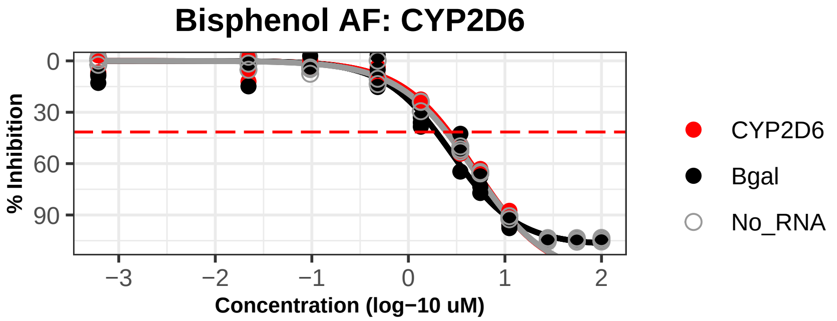
Bisphenol AF: CYP2D6
% Inhibition
0
30
60
90
−3
−2
−1
0
1
2
Concentration (log−10 uM)
CYP2D6
Bgal
No_RNA

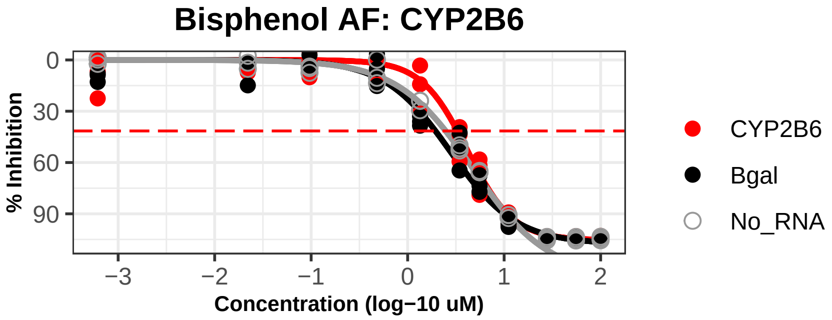
Bisphenol AF: CYP2B6
% Inhibition
0
30
60
90
−3
−2
−1
0
1
2
Concentration (log−10 uM)
CYP2B6
Bgal
No_RNA

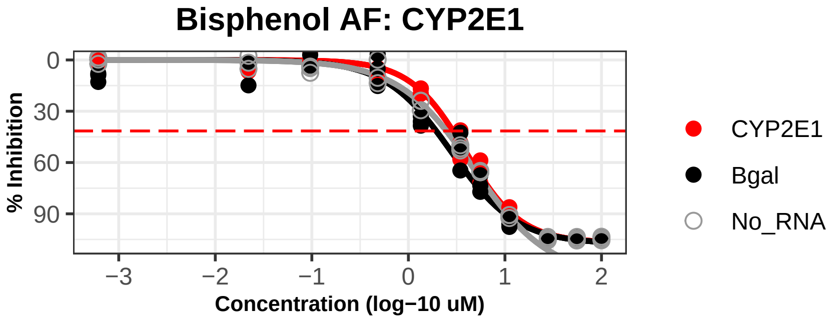
Bisphenol AF: CYP2E1
% Inhibition
0
30
60
90
−3
−2
−1
0
1
2
Concentration (log−10 uM)
CYP2E1
Bgal
No_RNA

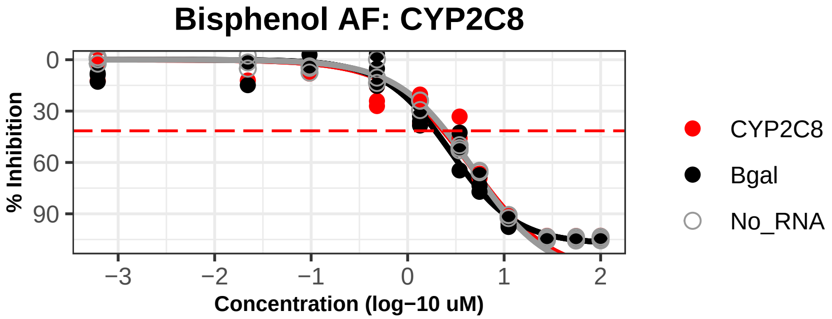
Bisphenol AF: CYP2C8
% Inhibition
0
30
60
90
−3
−2
−1
0
1
2
Concentration (log−10 uM)
CYP2C8
Bgal
No_RNA

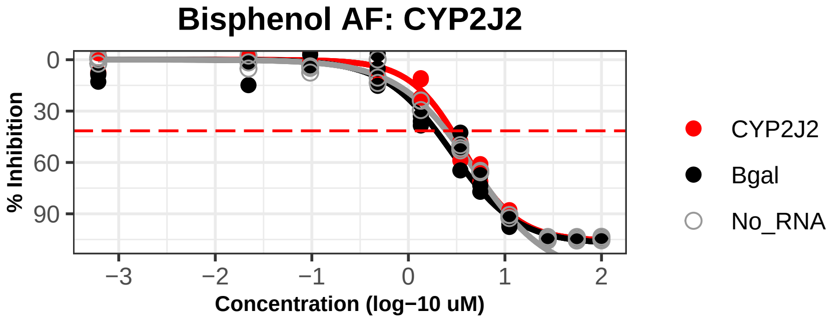
Bisphenol AF: CYP2J2
% Inhibition
0
30
60
90
−3
−2
−1
0
1
2
Concentration (log−10 uM)
CYP2J2
Bgal
No_RNA

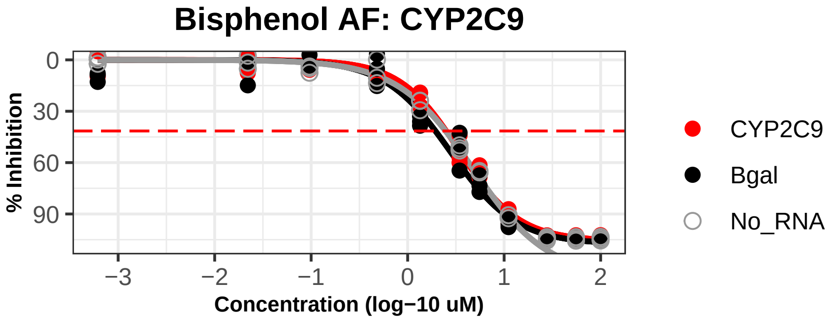
Bisphenol AF: CYP2C9
% Inhibition
0
30
60
90
−3
−2
−1
0
1
2
Concentration (log−10 uM)
CYP2C9
Bgal
No_RNA

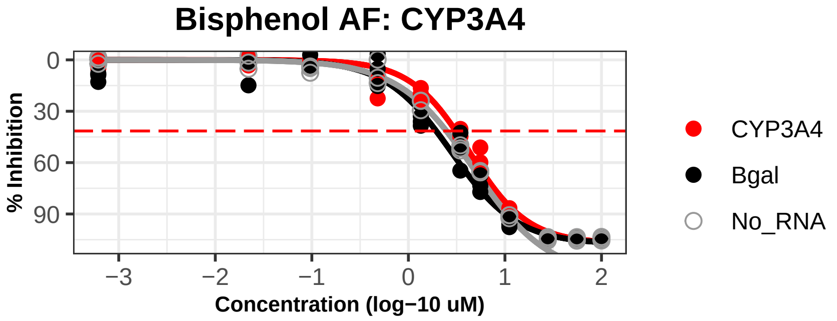
Bisphenol AF: CYP3A4
% Inhibition
0
30
60
90
−3
−2
−1
0
1
2
Concentration (log−10 uM)
CYP3A4
Bgal
No_RNA

Bisphenol B: CYP1A2

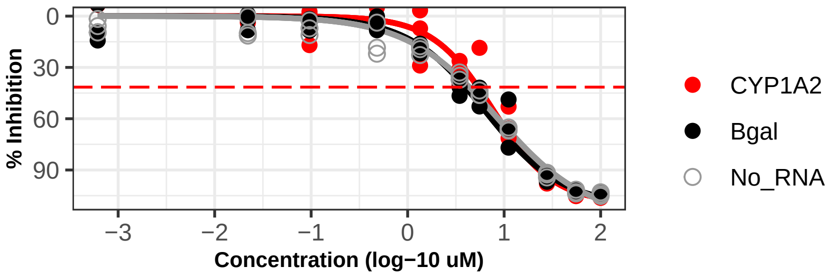

Bisphenol B: CYP2C19

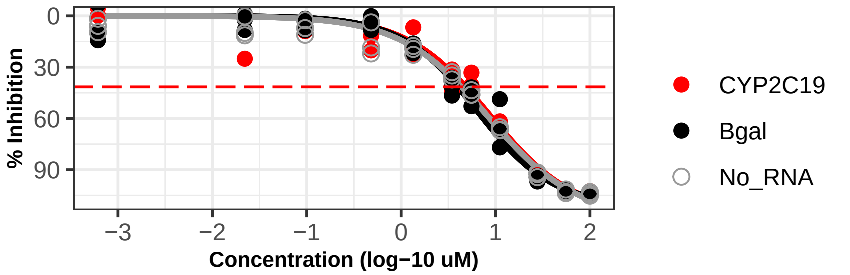

Bisphenol B: CYP2A6

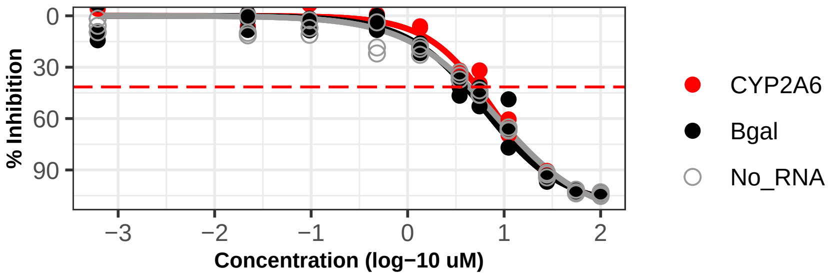

Bisphenol B: CYP2D6

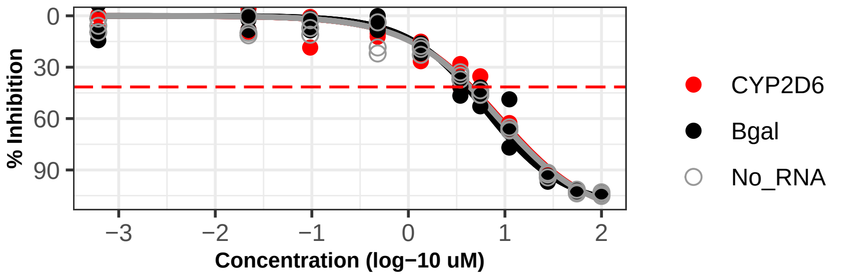

Bisphenol B: CYP2B6

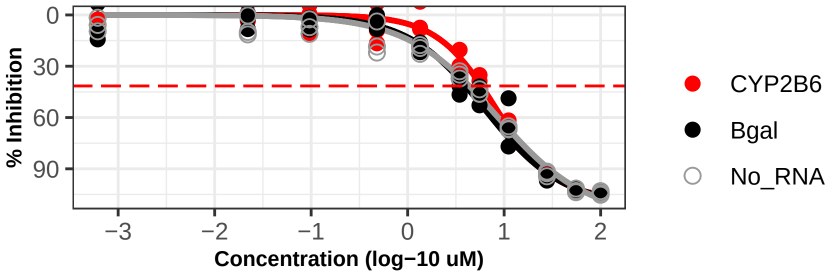

Bisphenol B: CYP2E1

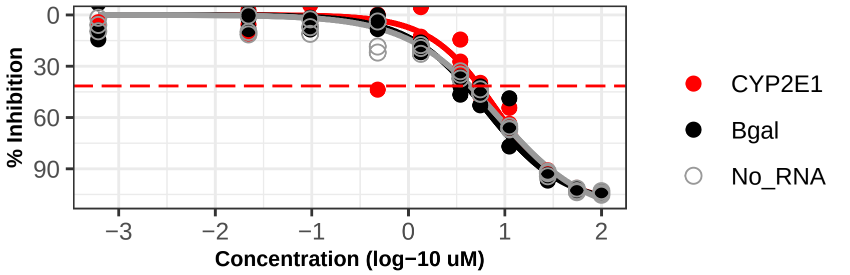

Bisphenol B: CYP2C8

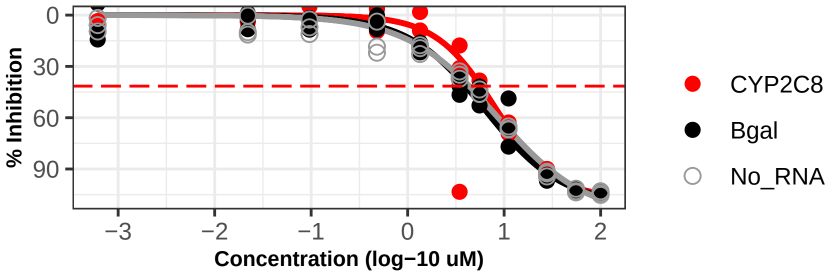

Bisphenol B: CYP2J2

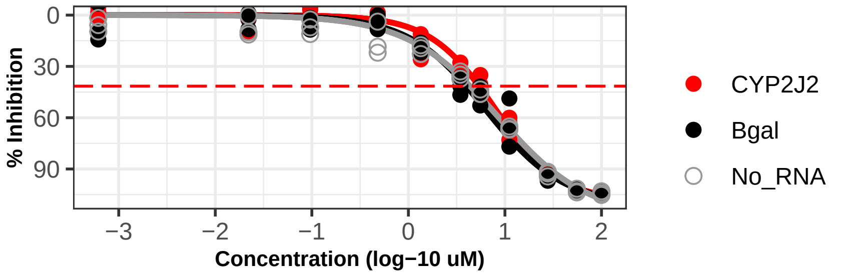

Bisphenol B: CYP2C9

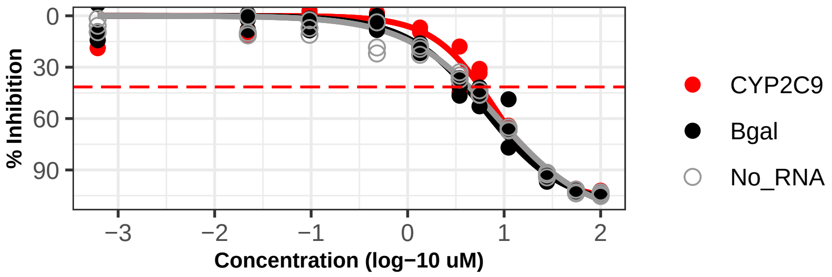

Bisphenol B: CYP3A4

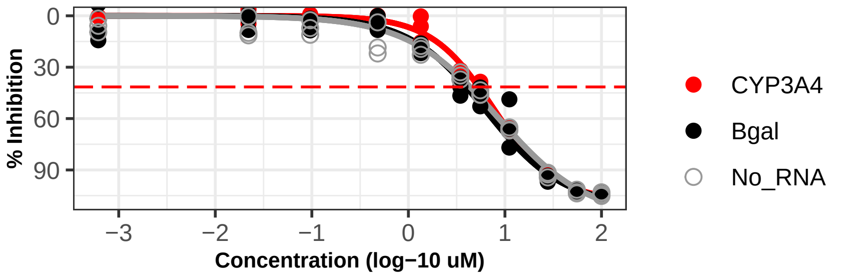

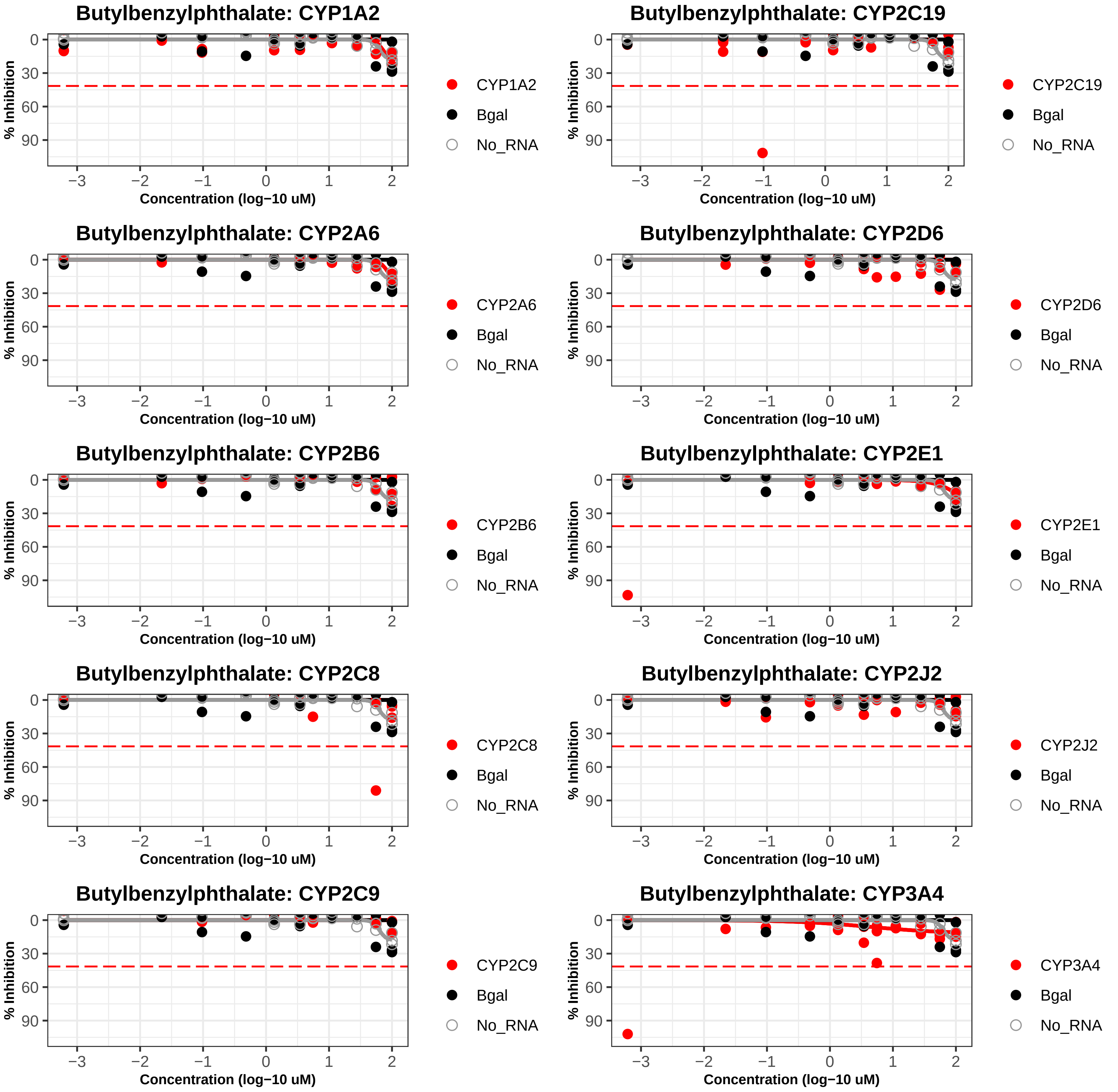
Butylbenzylphthalate: CYP1A2
Butylbenzylphthalate: CYP2C19
Butylbenzylphthalate: CYP2A6
Butylbenzylphthalate: CYP2D6
Butylbenzylphthalate: CYP2B6
Butylbenzylphthalate: CYP2E1
Butylbenzylphthalate: CYP2C8
Butylbenzylphthalate: CYP2J2
Butylbenzylphthalate: CYP2C9
Butylbenzylphthalate: CYP3A4
% Inhibition
Concentration (log−10 uM)
CYP1A2
CYP2C19
CYP2A6
CYP2D6
CYP2B6
CYP2E1
CYP2C8
CYP2J2
CYP2C9
CYP3A4
Bgal
No_RNA

Butylparaben: CYP1A2

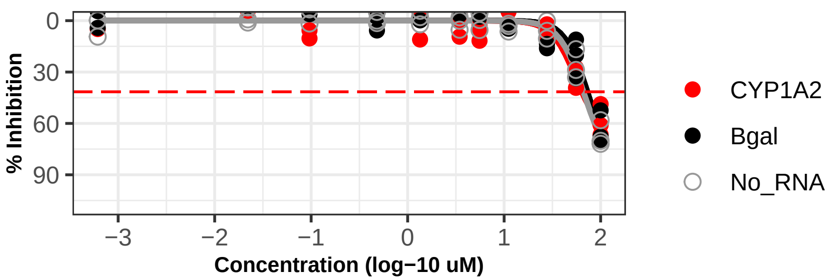

Butylparaben: CYP2A6

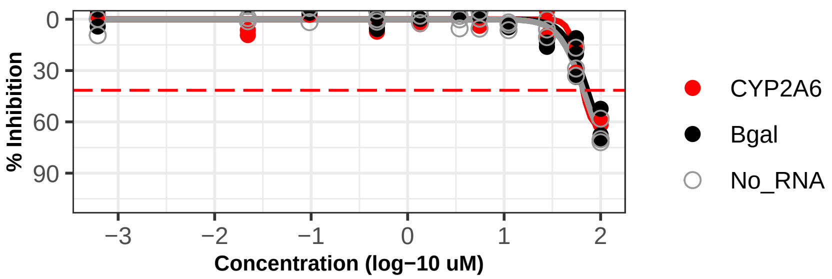

Butylparaben: CYP2B6

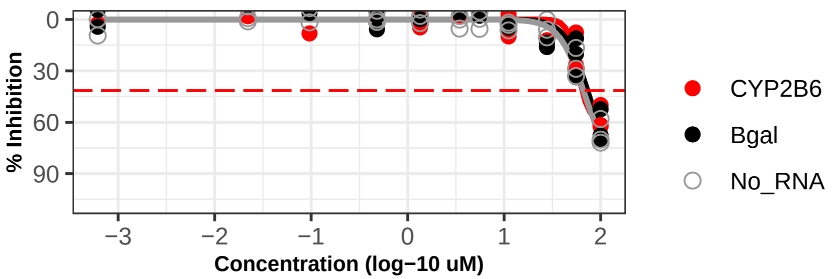

Butylparaben: CYP2C8

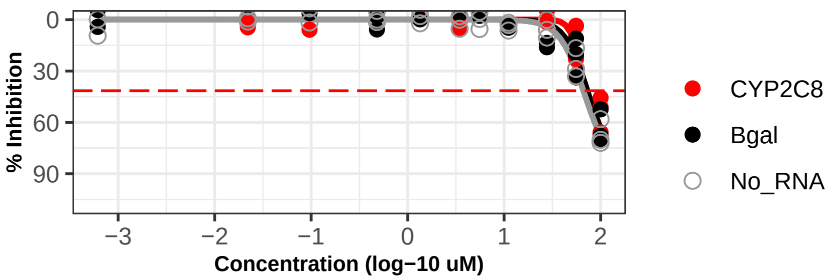

Butylparaben: CYP2C9

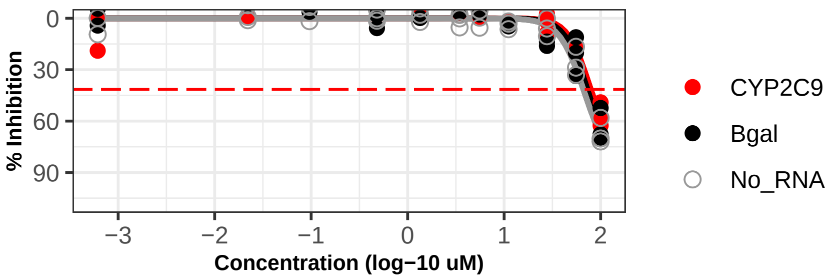

Butylparaben: CYP2C19

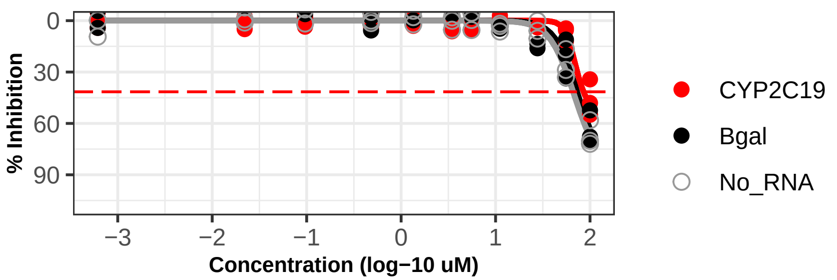

Butylparaben: CYP2D6

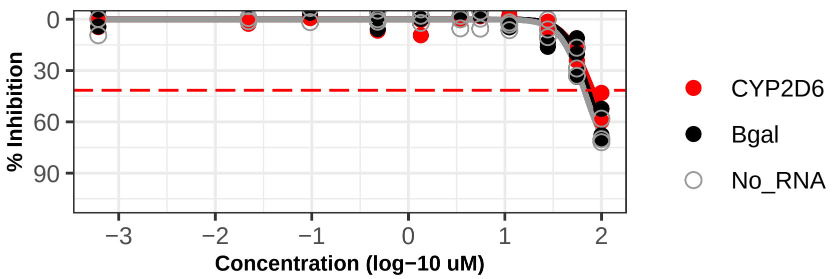

Butylparaben: CYP2E1

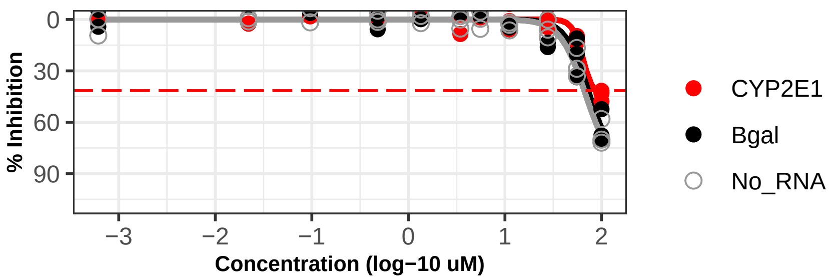

Butylparaben: CYP2J2

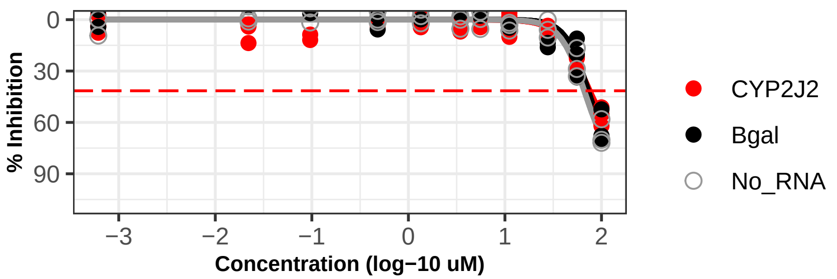

Butylparaben: CYP3A4

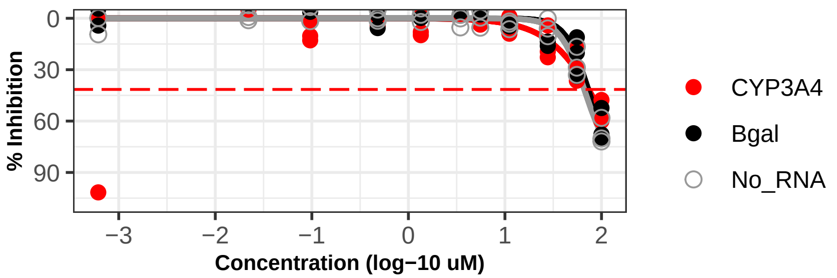

Carbendazim: CYP1A2

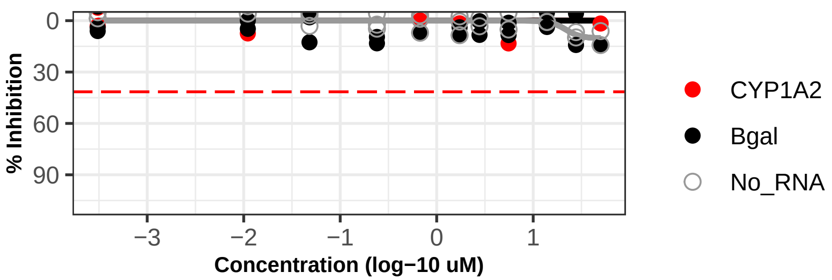

Carbendazim: CYP2C19

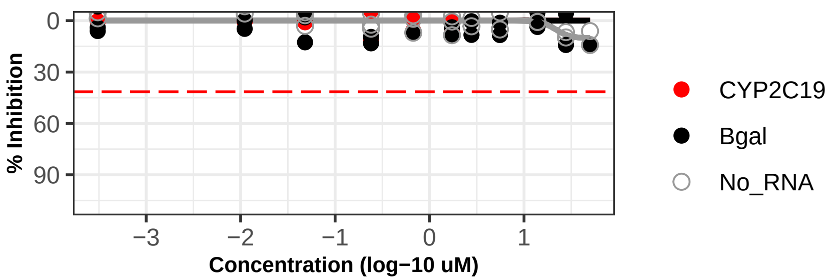

Carbendazim: CYP2A6

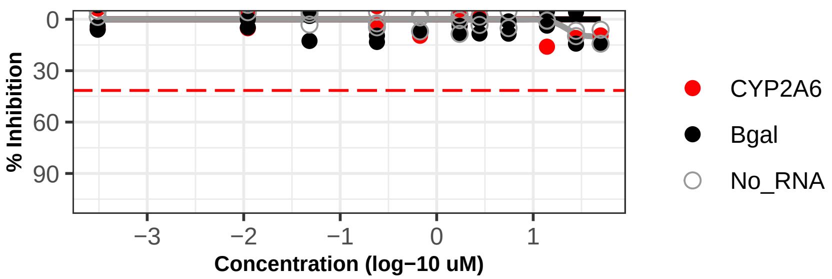

Carbendazim: CYP2D6

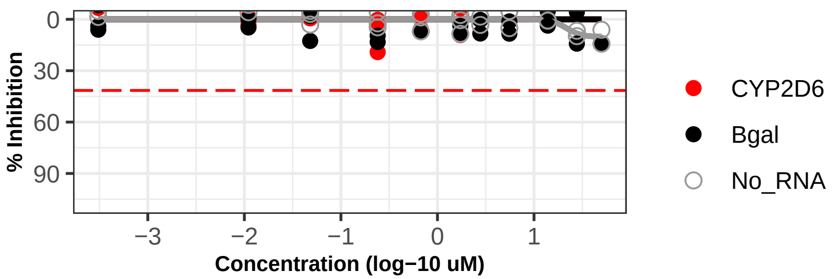

Carbendazim: CYP2B6

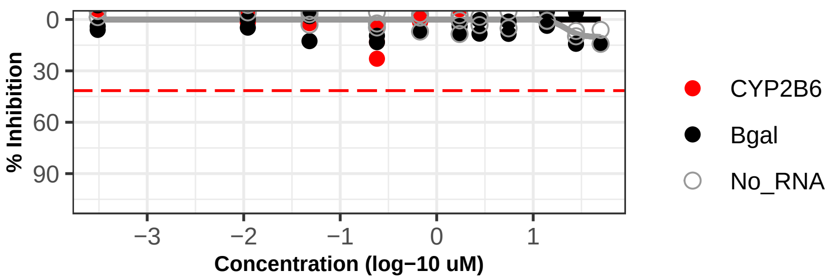

Carbendazim: CYP2E1

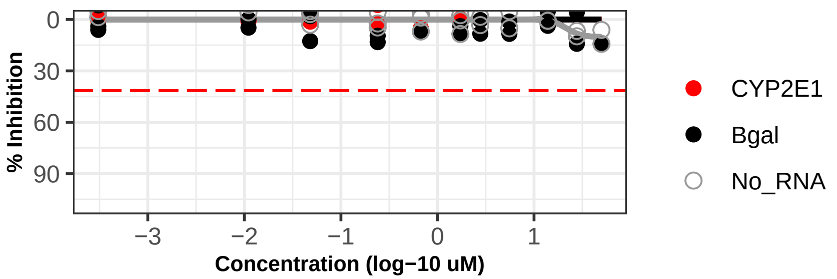

Carbendazim: CYP2C8

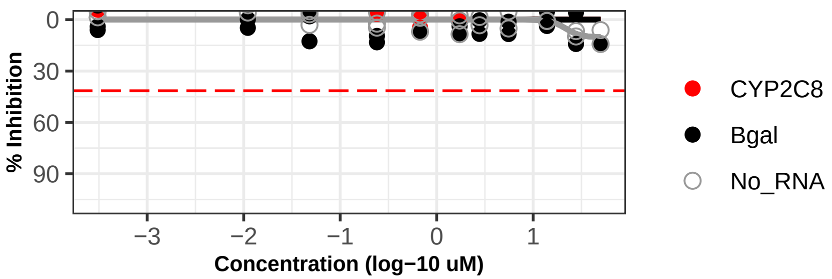

Carbendazim: CYP2J2

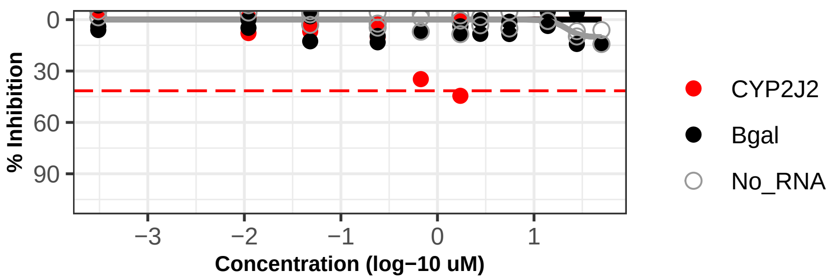

Carbendazim: CYP2C9

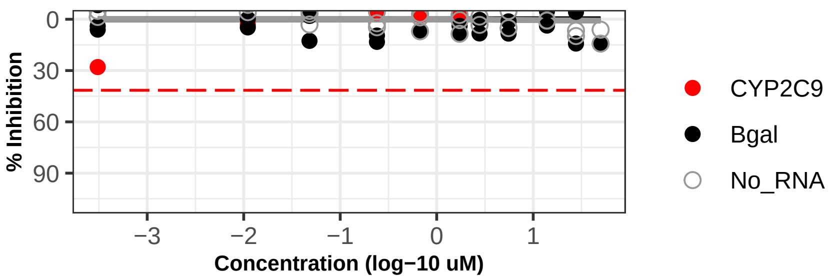

Carbendazim: CYP3A4

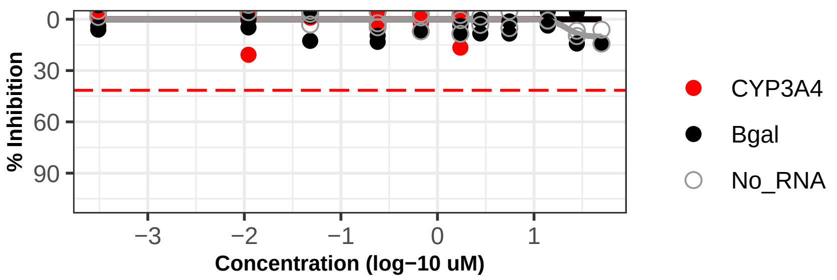

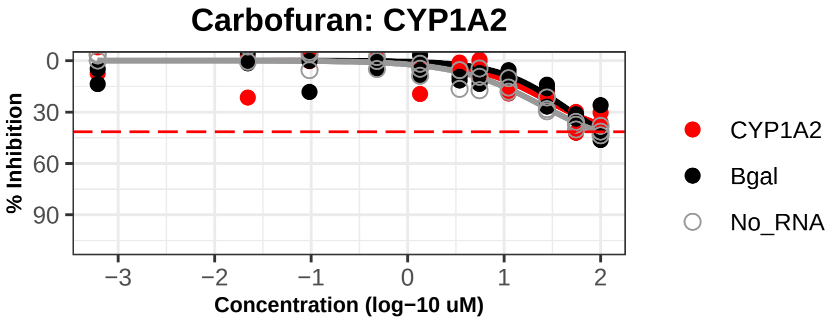
Carbofuran: CYP1A2
% Inhibition
0
30
60
90
−3
−2
−1
0
1
2
Concentration (log−10 uM)
CYP1A2
Bgal
No_RNA

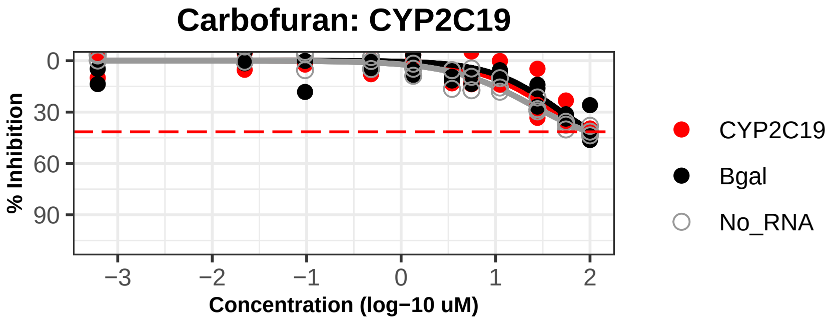
Carbofuran: CYP2C19
% Inhibition
0
30
60
90
−3
−2
−1
0
1
2
Concentration (log−10 uM)
CYP2C19
Bgal
No_RNA

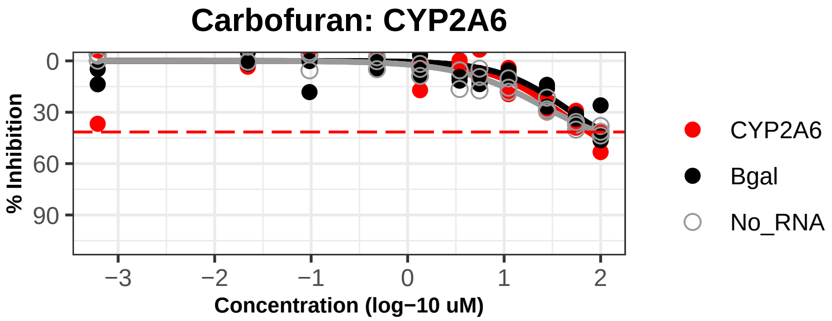
Carbofuran: CYP2A6
% Inhibition
0
30
60
90
−3
−2
−1
0
1
2
Concentration (log−10 uM)
CYP2A6
Bgal
No_RNA

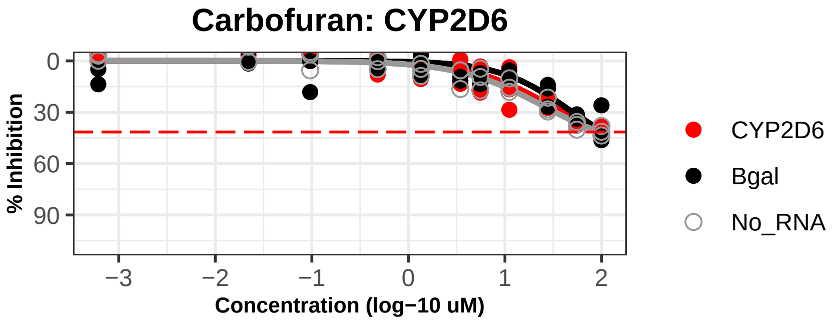
Carbofuran: CYP2D6
% Inhibition
0
30
60
90
−3
−2
−1
0
1
2
Concentration (log−10 uM)
CYP2D6
Bgal
No_RNA

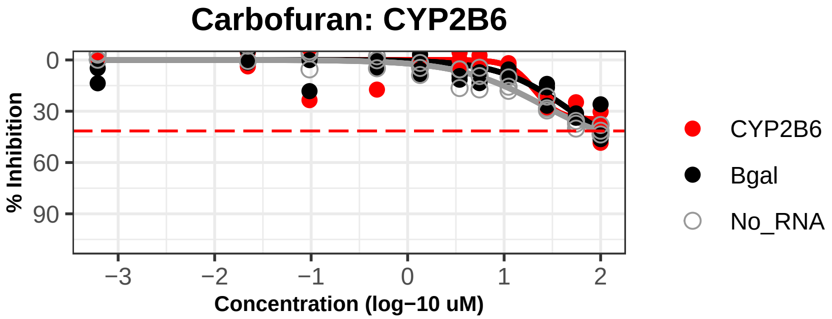
Carbofuran: CYP2B6
% Inhibition
0
30
60
90
−3
−2
−1
0
1
2
Concentration (log−10 uM)
CYP2B6
Bgal
No_RNA

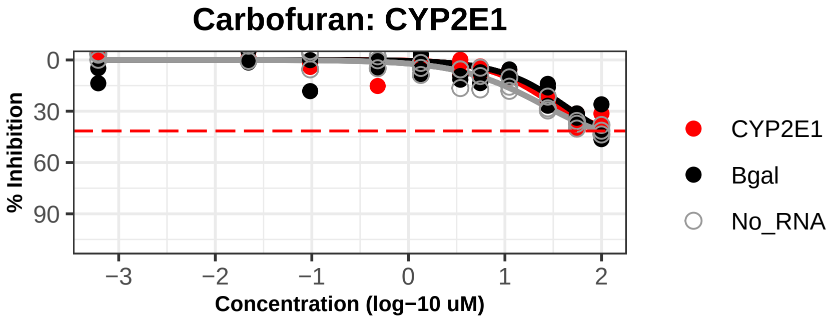
Carbofuran: CYP2E1
% Inhibition
0
30
60
90
−3
−2
−1
0
1
2
Concentration (log−10 uM)
CYP2E1
Bgal
No_RNA

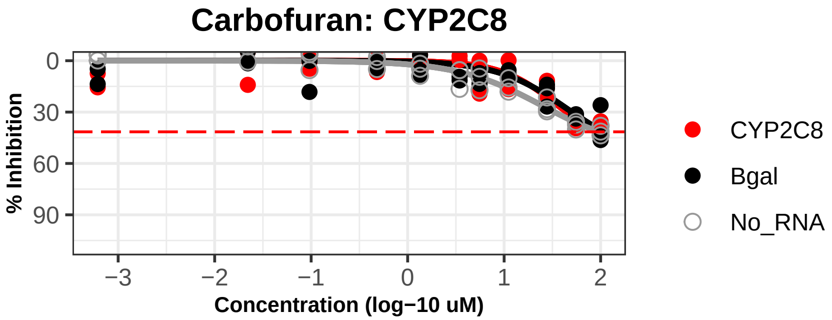
Carbofuran: CYP2C8
% Inhibition
0
30
60
90
−3
−2
−1
0
1
2
Concentration (log−10 uM)
CYP2C8
Bgal
No_RNA

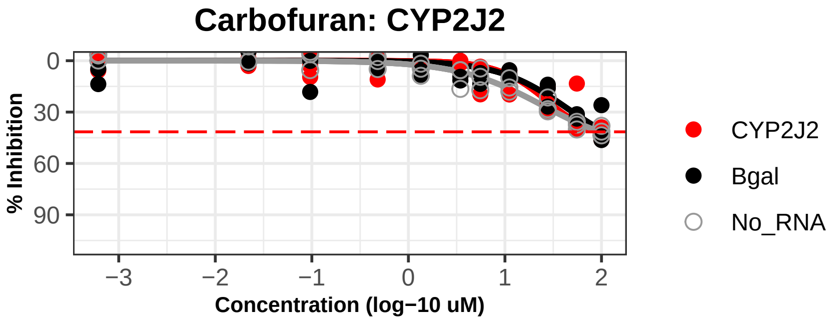
Carbofuran: CYP2J2
% Inhibition
0
30
60
90
−3
−2
−1
0
1
2
Concentration (log−10 uM)
CYP2J2
Bgal
No_RNA

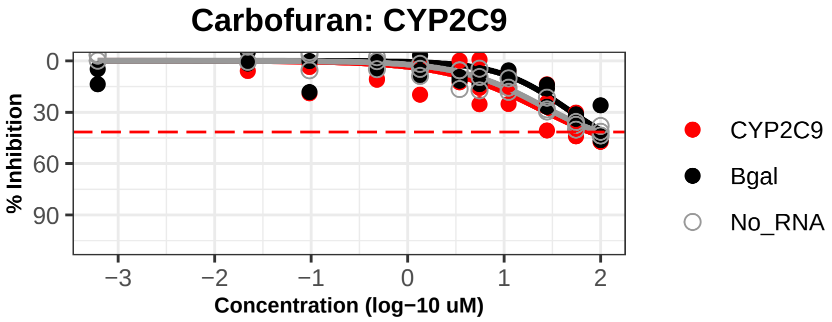
Carbofuran: CYP2C9
% Inhibition
0
30
60
90
−3
−2
−1
0
1
2
Concentration (log−10 uM)
CYP2C9
Bgal
No_RNA

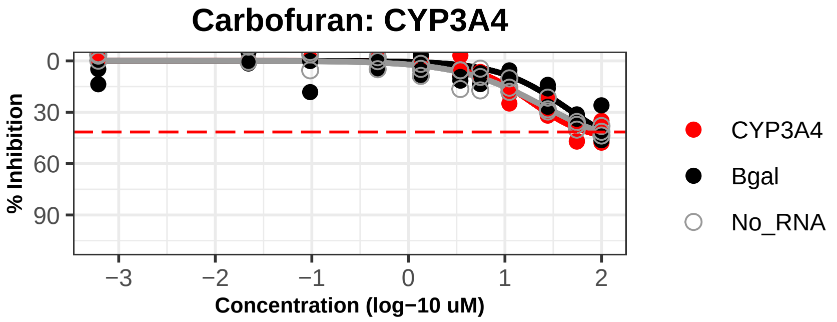
Carbofuran: CYP3A4
% Inhibition
0
30
60
90
−3
−2
−1
0
1
2
Concentration (log−10 uM)
CYP3A4
Bgal
No_RNA

Chlorothalonil: CYP1A2

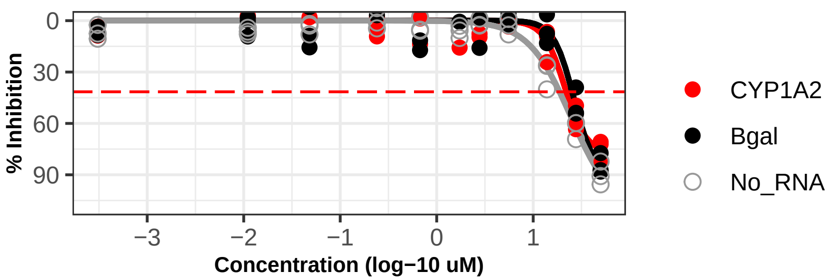

Chlorothalonil: CYP2C19

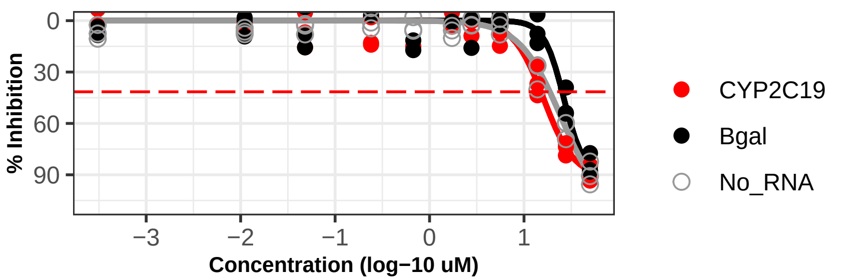

Chlorothalonil: CYP2A6

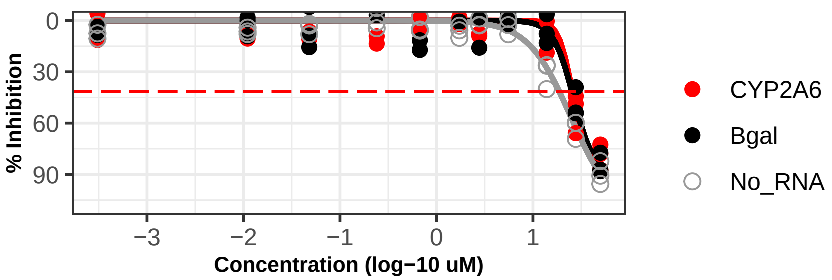

Chlorothalonil: CYP2D6

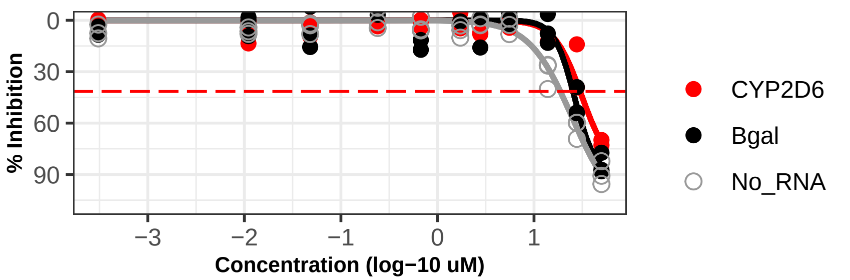

Chlorothalonil: CYP2B6

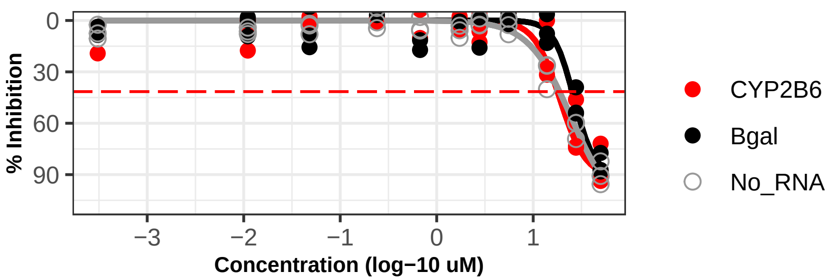

Chlorothalonil: CYP2E1

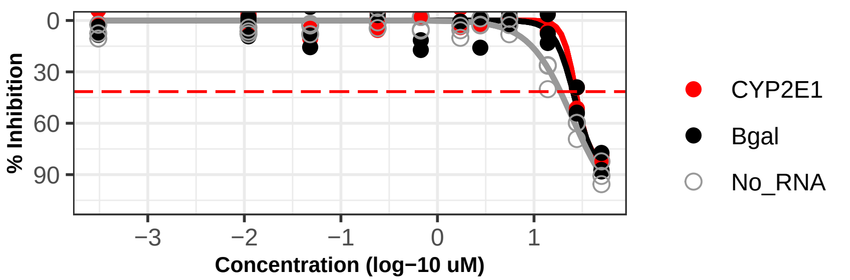

Chlorothalonil: CYP2C8

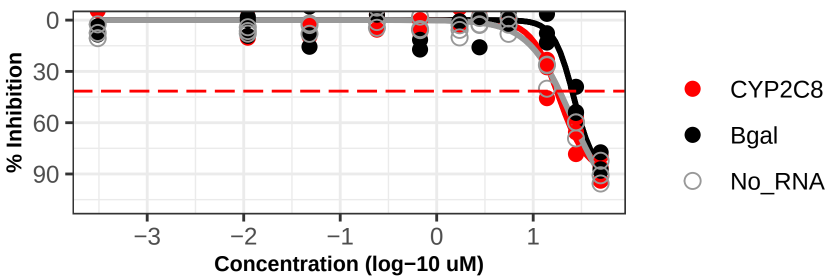

Chlorothalonil: CYP2J2

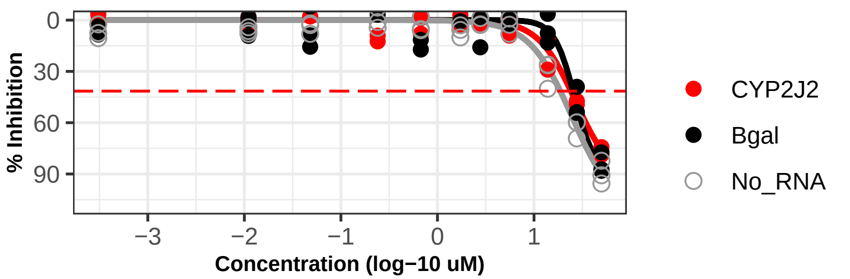

Chlorothalonil: CYP2C9

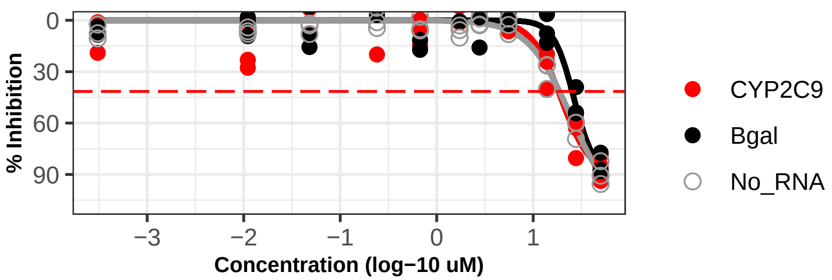

Chlorothalonil: CYP3A4

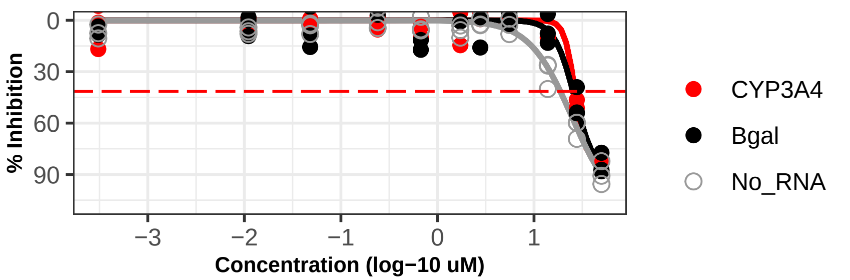

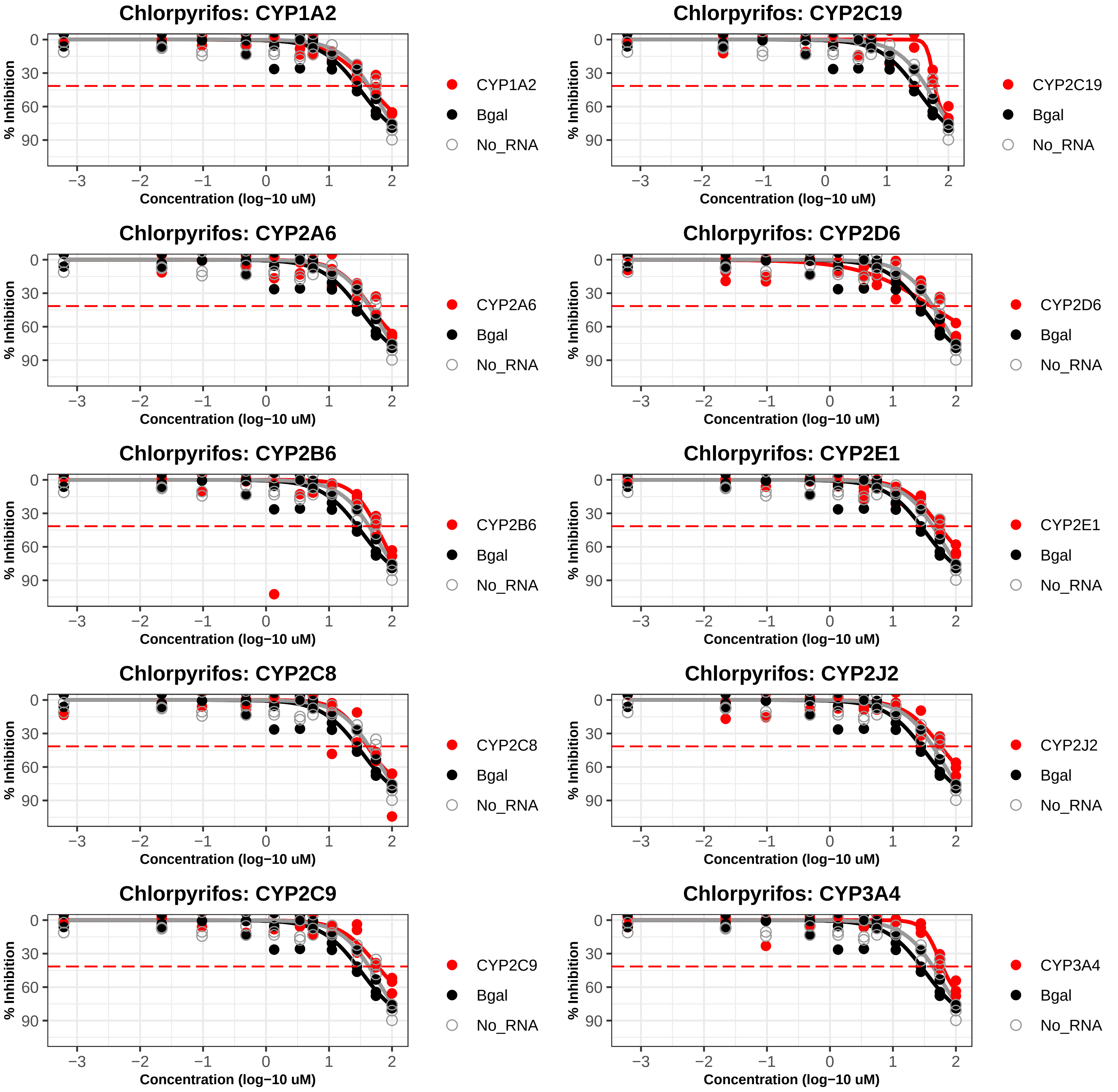
Chlorpyrifos: CYP1A2
Chlorpyrifos: CYP2C19
Chlorpyrifos: CYP2A6
Chlorpyrifos: CYP2D6
Chlorpyrifos: CYP2B6
Chlorpyrifos: CYP2E1
Chlorpyrifos: CYP2C8
Chlorpyrifos: CYP2J2
Chlorpyrifos: CYP2C9
Chlorpyrifos: CYP3A4
% Inhibition
0
30
60
90
Concentration (log−10 uM)
−3
−2
−1
0
1
2
CYP1A2
CYP2C19
CYP2A6
CYP2D6
CYP2B6
CYP2E1
CYP2C8
CYP2J2
CYP2C9
CYP3A4
Bgal
No_RNA

Chlorpyrifos−methyl: CYP1A2

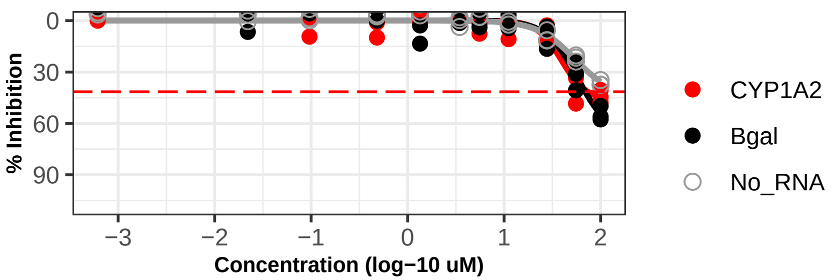

Chlorpyrifos−methyl: CYP2C19

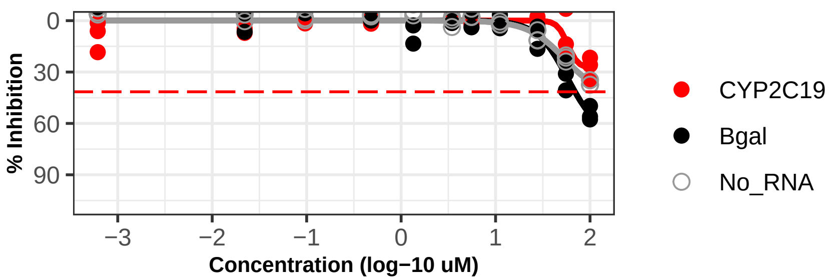

Chlorpyrifos−methyl: CYP2A6

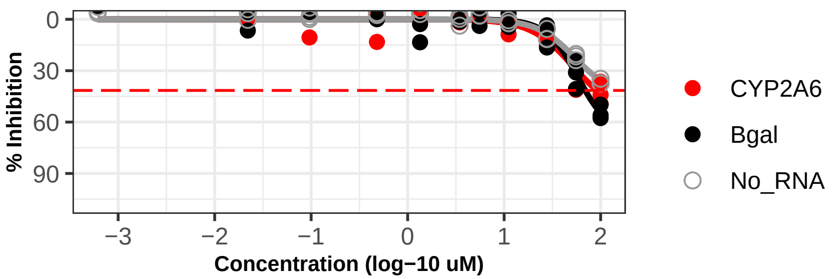

Chlorpyrifos−methyl: CYP2D6

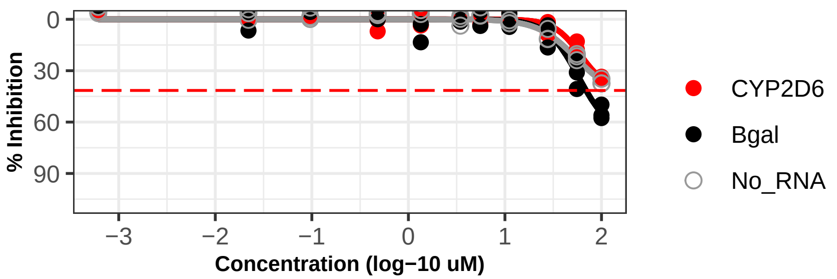

Chlorpyrifos−methyl: CYP2B6

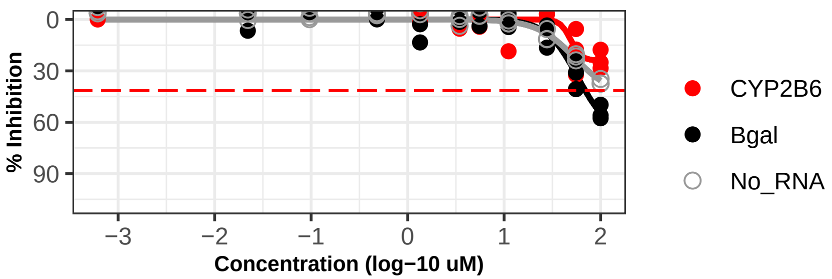

Chlorpyrifos−methyl: CYP2E1

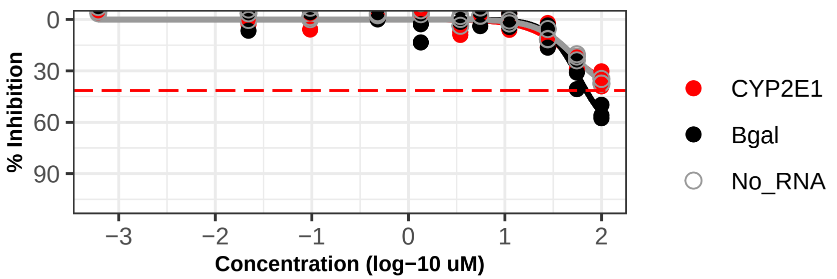

Chlorpyrifos−methyl: CYP2C8

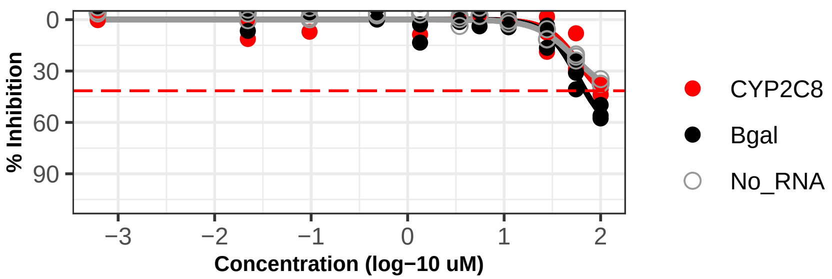

Chlorpyrifos−methyl: CYP2J2

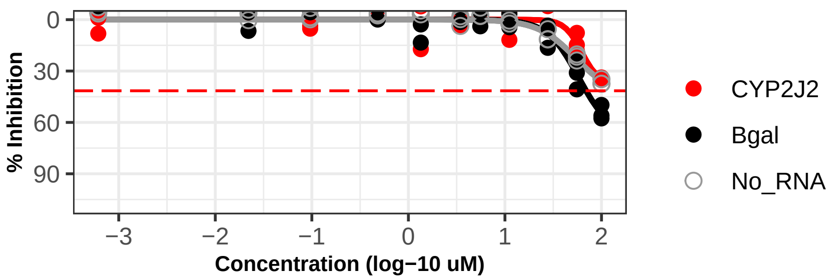

Chlorpyrifos−methyl: CYP2C9

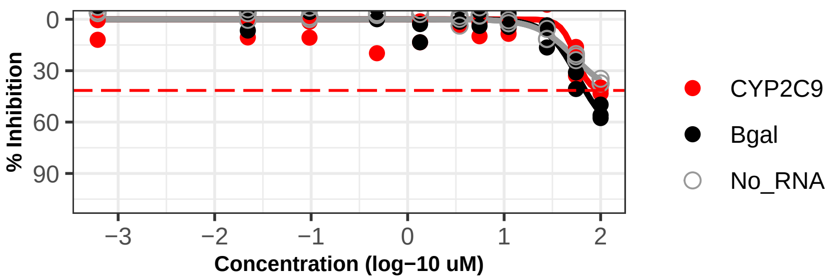

Chlorpyrifos−methyl: CYP3A4

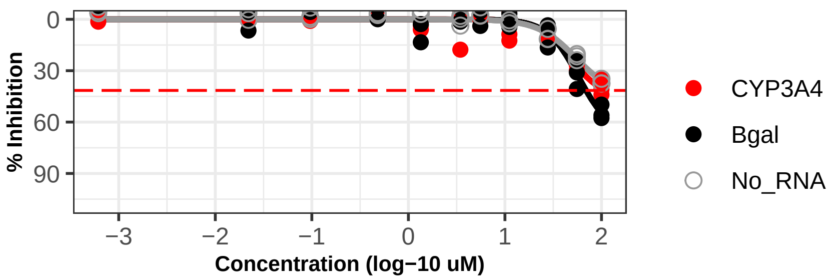

Clomiphene citrate: CYP1A2

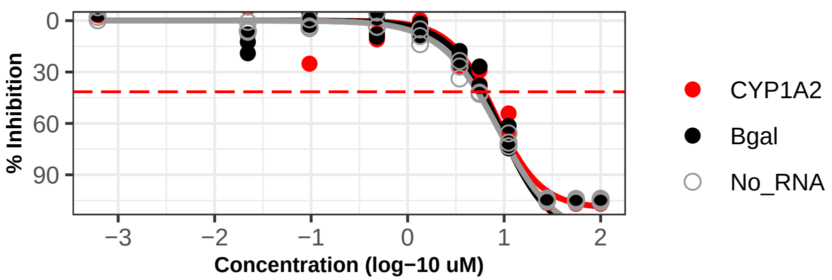

Clomiphene citrate: CYP2C19

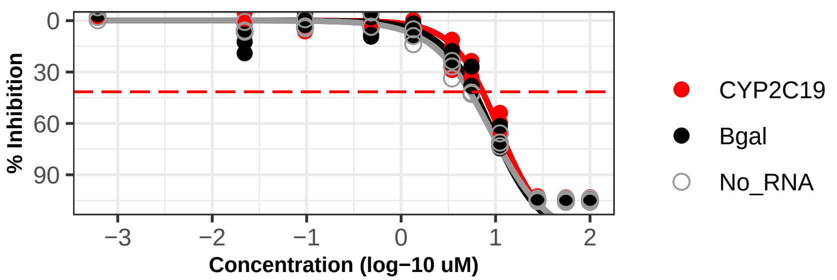

Clomiphene citrate: CYP2A6

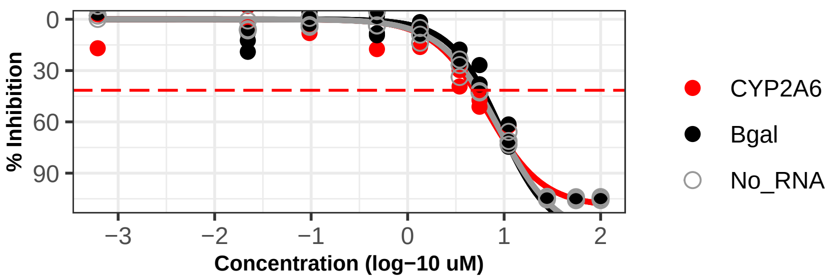

Clomiphene citrate: CYP2D6

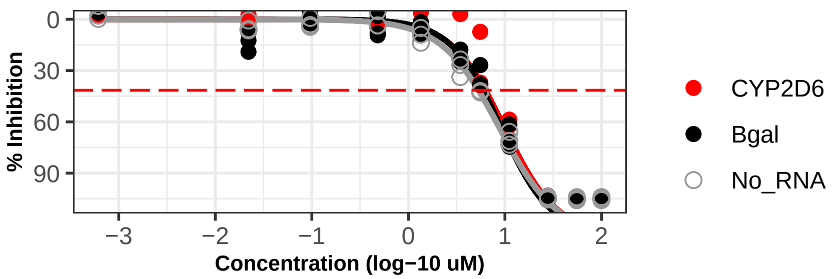

Clomiphene citrate: CYP2B6

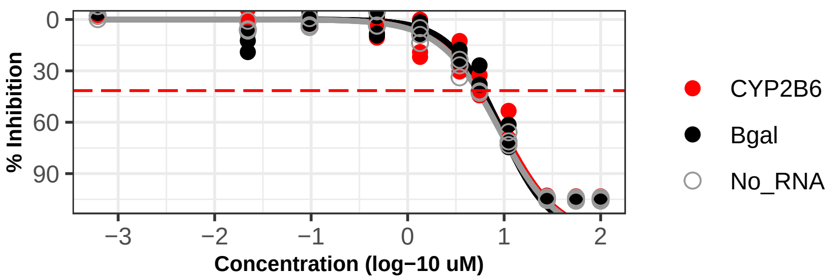

Clomiphene citrate: CYP2E1

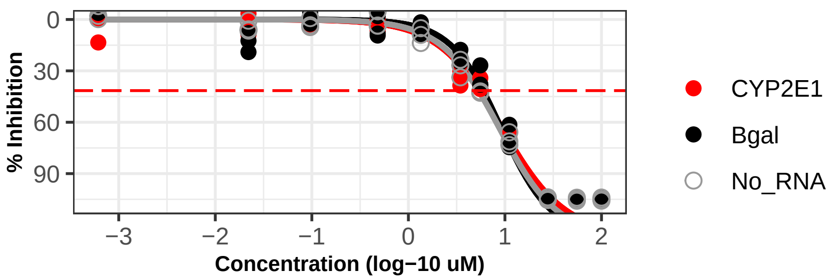

Clomiphene citrate: CYP2C8

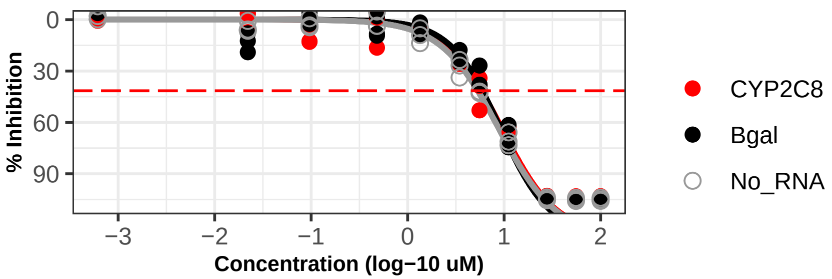

Clomiphene citrate: CYP2J2

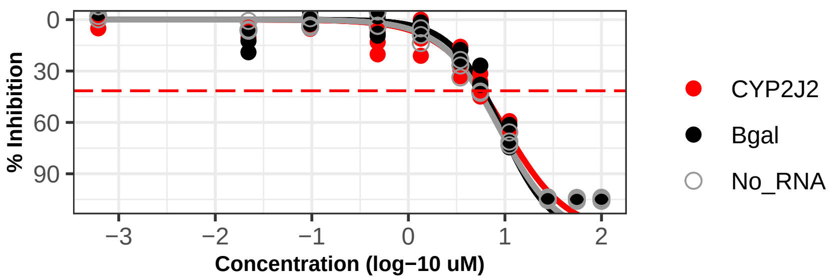

Clomiphene citrate: CYP2C9

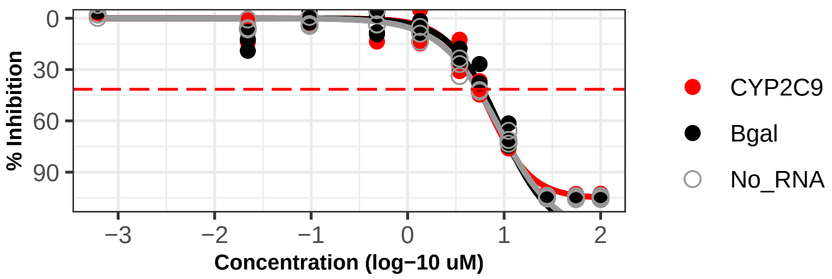

Clomiphene citrate: CYP3A4

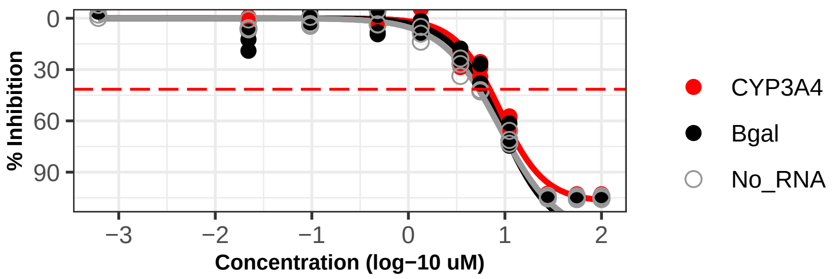

Corticosterone: CYP1A2

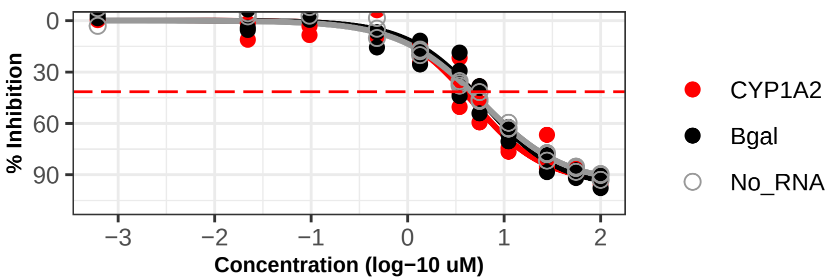

Corticosterone: CYP2C19

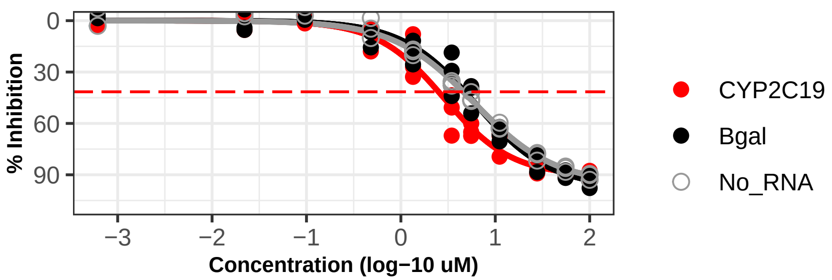

Corticosterone: CYP2A6

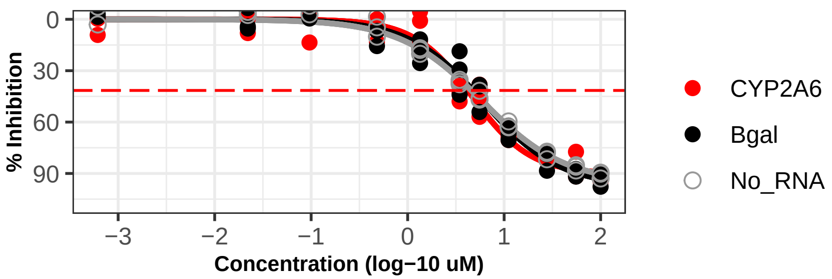

Corticosterone: CYP2D6

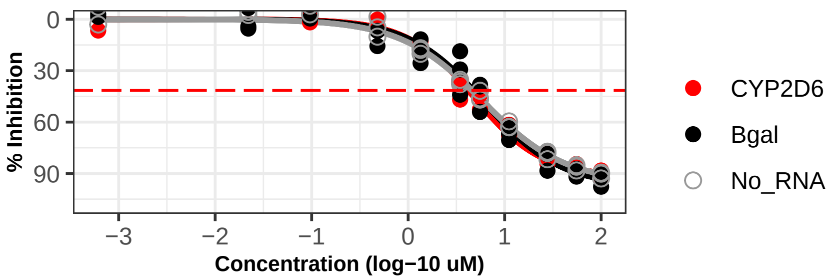

Corticosterone: CYP2B6

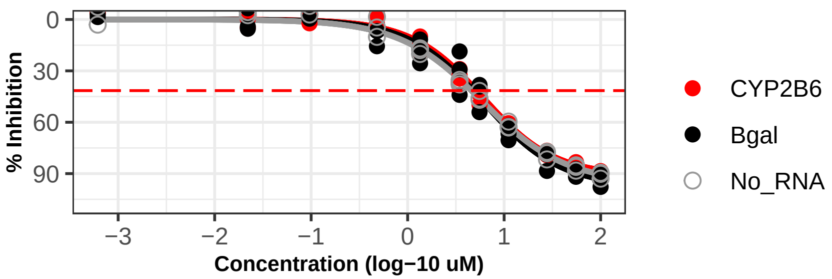

Corticosterone: CYP2E1

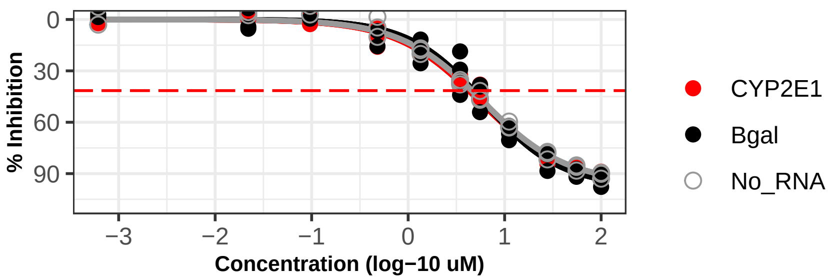

Corticosterone: CYP2C8

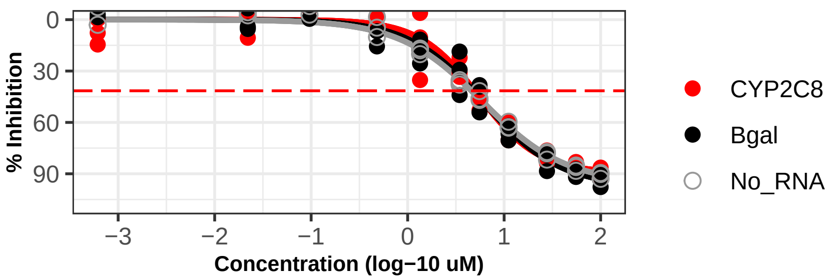

Corticosterone: CYP2J2

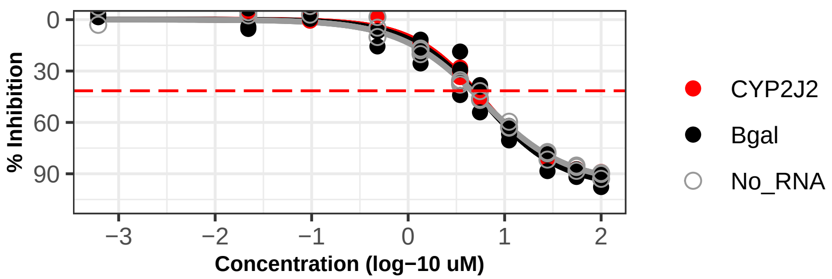

Corticosterone: CYP2C9

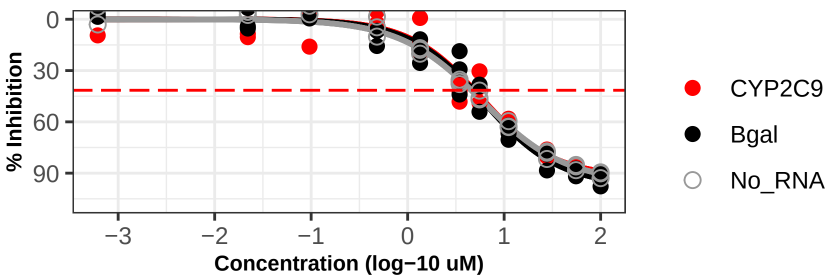

Corticosterone: CYP3A4

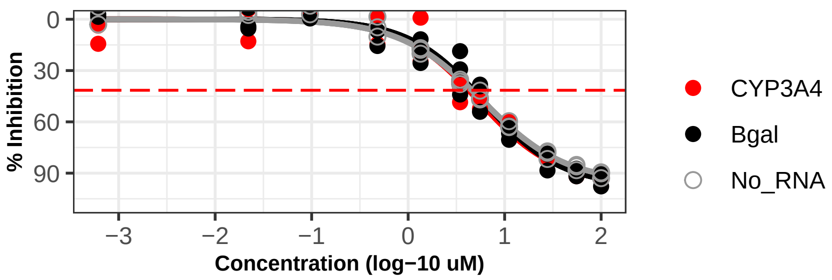

Coumestrol : CYP1A2

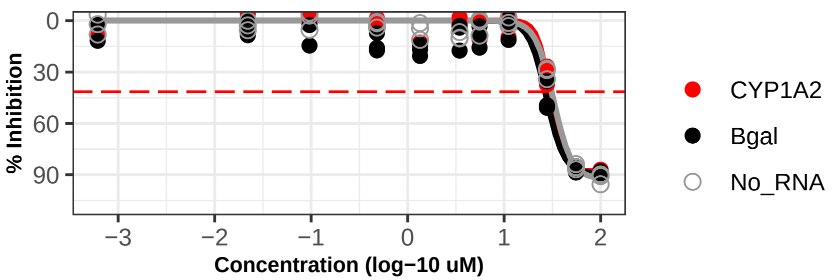

Coumestrol : CYP2C19

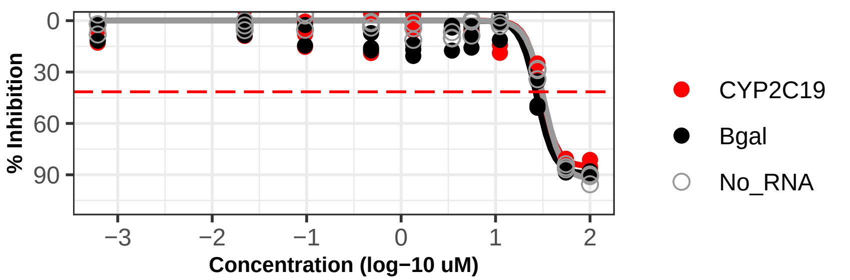

Coumestrol : CYP2A6

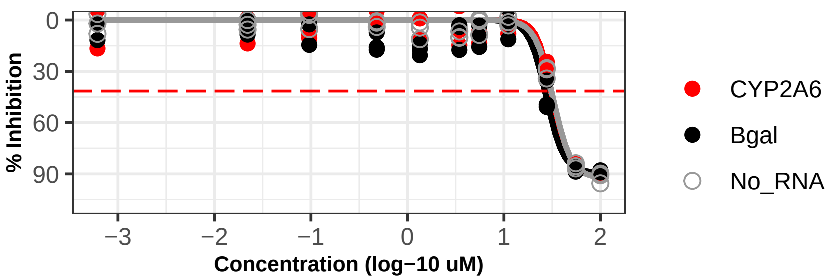

Coumestrol : CYP2D6

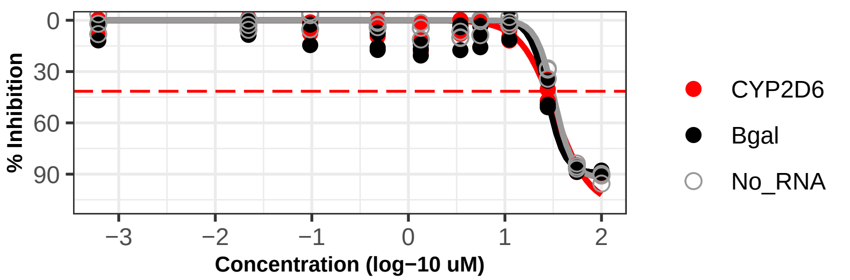

Coumestrol : CYP2B6

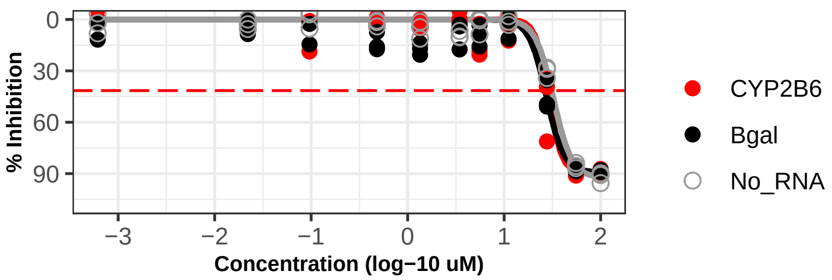

Coumestrol : CYP2E1

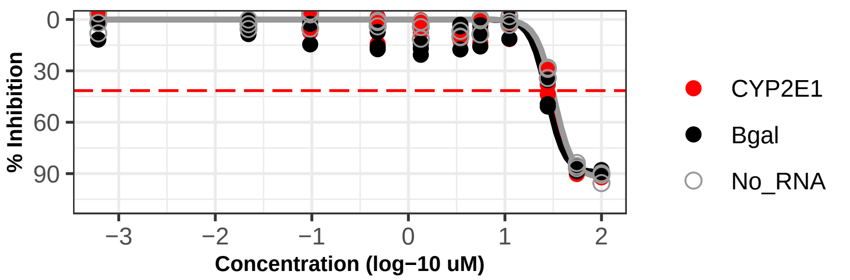

Coumestrol : CYP2C8

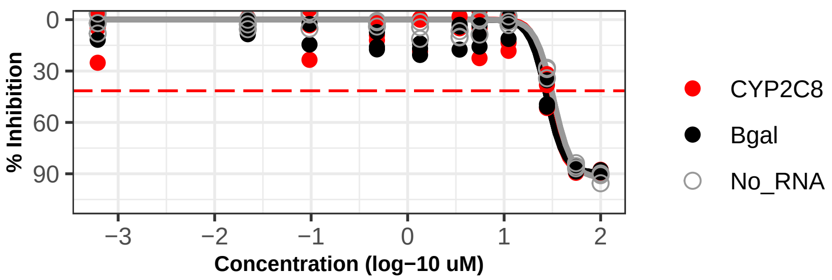

Coumestrol : CYP2J2

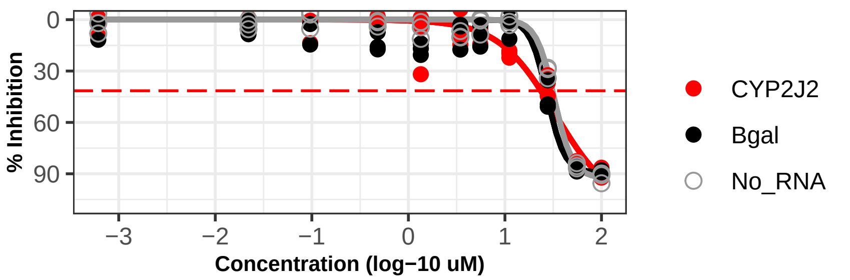

Coumestrol : CYP2C9

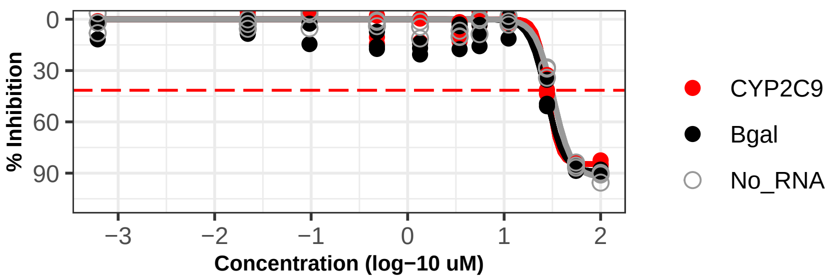

Coumestrol : CYP3A4

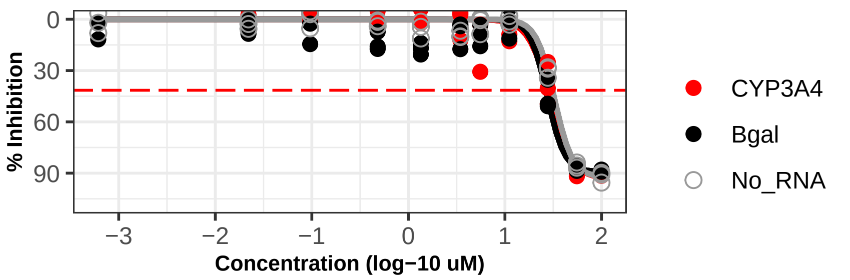

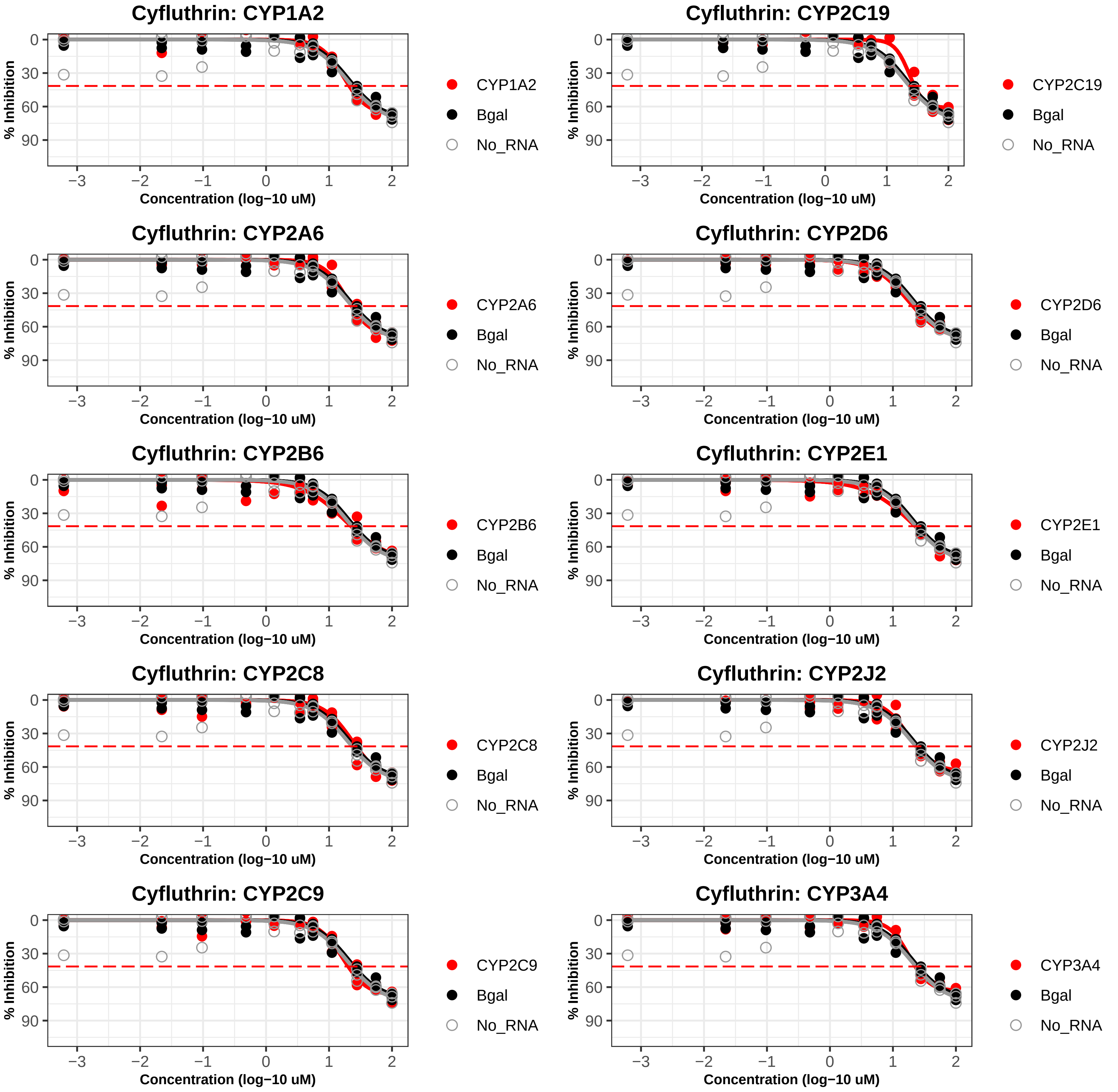
Cyfluthrin: CYP1A2
Cyfluthrin: CYP2C19
Cyfluthrin: CYP2A6
Cyfluthrin: CYP2D6
Cyfluthrin: CYP2B6
Cyfluthrin: CYP2E1
Cyfluthrin: CYP2C8
Cyfluthrin: CYP2J2
Cyfluthrin: CYP2C9
Cyfluthrin: CYP3A4
% Inhibition
Concentration (log−10 uM)
CYP1A2
CYP2C19
CYP2A6
CYP2D6
CYP2B6
CYP2E1
CYP2C8
CYP2J2
CYP2C9
CYP3A4
Bgal
No_RNA

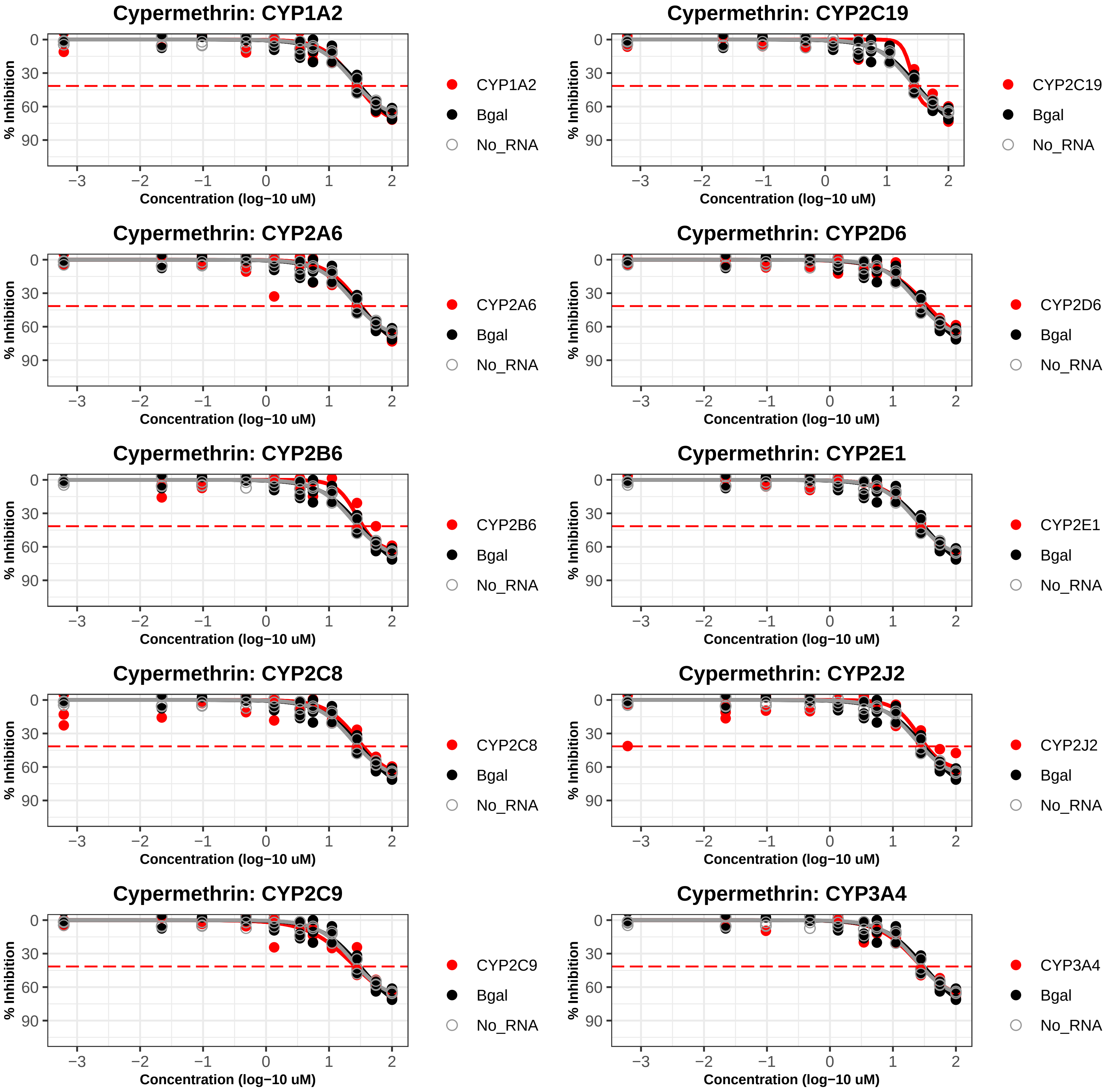
Cypermethrin: CYP1A2
CYP1A2
Bgal
No_RNA
Cypermethrin: CYP2C19
CYP2C19
Bgal
No_RNA
Cypermethrin: CYP2A6
CYP2A6
Bgal
No_RNA
Cypermethrin: CYP2D6
CYP2D6
Bgal
No_RNA
Cypermethrin: CYP2B6
CYP2B6
Bgal
No_RNA
Cypermethrin: CYP2E1
CYP2E1
Bgal
No_RNA
Cypermethrin: CYP2C8
CYP2C8
Bgal
No_RNA
Cypermethrin: CYP2J2
CYP2J2
Bgal
No_RNA
Cypermethrin: CYP2C9
CYP2C9
Bgal
No_RNA
Cypermethrin: CYP3A4
CYP3A4
Bgal
No_RNA
% Inhibition
0
30
60
90
Concentration (log−10 uM)
−3
−2
−1
0
1
2

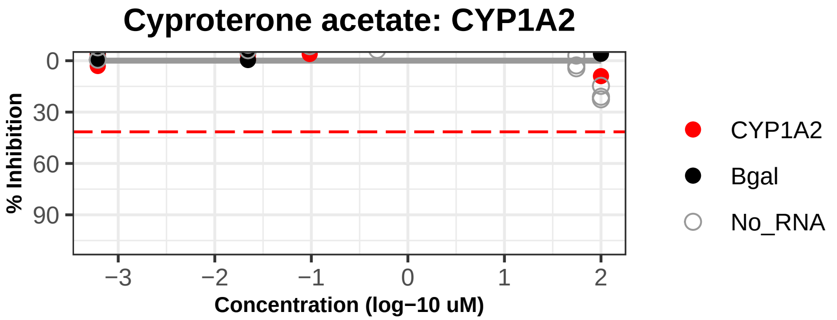
Cyproterone acetate: CYP1A2
% Inhibition
0
30
60
90
−3
−2
−1
0
1
2
Concentration (log−10 uM)
CYP1A2
Bgal
No_RNA

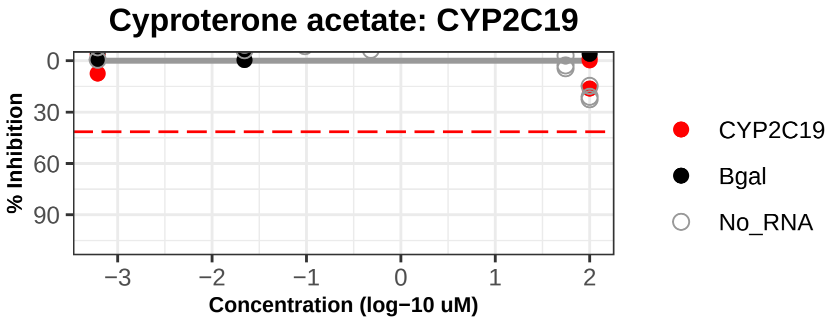
Cyproterone acetate: CYP2C19
% Inhibition
0
30
60
90
−3
−2
−1
0
1
2
Concentration (log−10 uM)
CYP2C19
Bgal
No_RNA

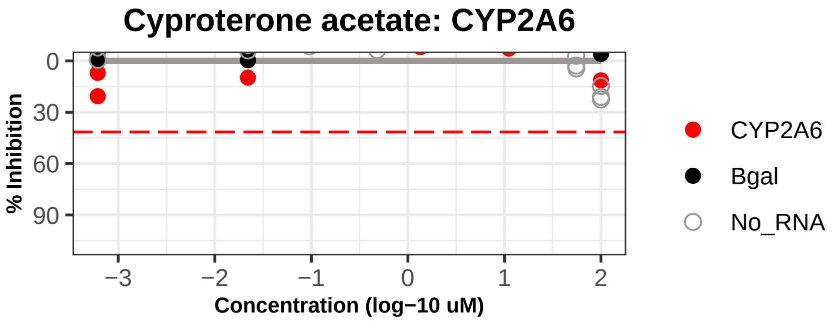
Cyproterone acetate: CYP2A6
% Inhibition
0
30
60
90
−3
−2
−1
0
1
2
Concentration (log−10 uM)
CYP2A6
Bgal
No_RNA

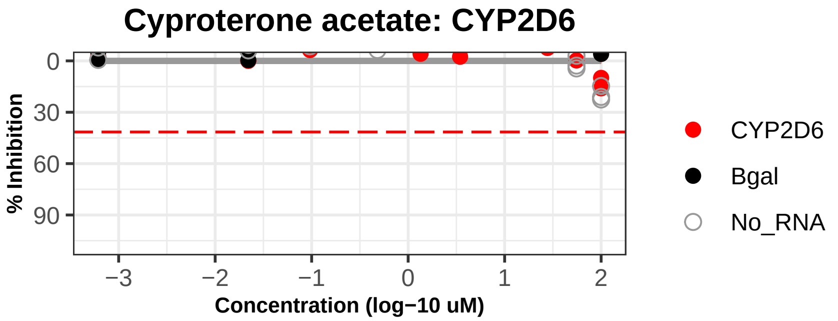
Cyproterone acetate: CYP2D6
% Inhibition
0
30
60
90
−3
−2
−1
0
1
2
Concentration (log−10 uM)
CYP2D6
Bgal
No_RNA

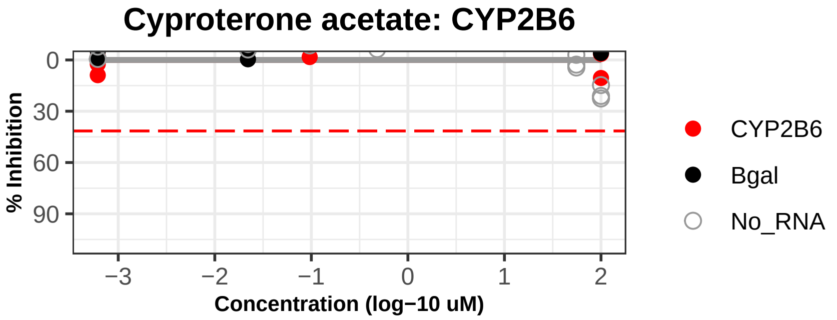
Cyproterone acetate: CYP2B6
% Inhibition
0
30
60
90
−3
−2
−1
0
1
2
Concentration (log−10 uM)
CYP2B6
Bgal
No_RNA

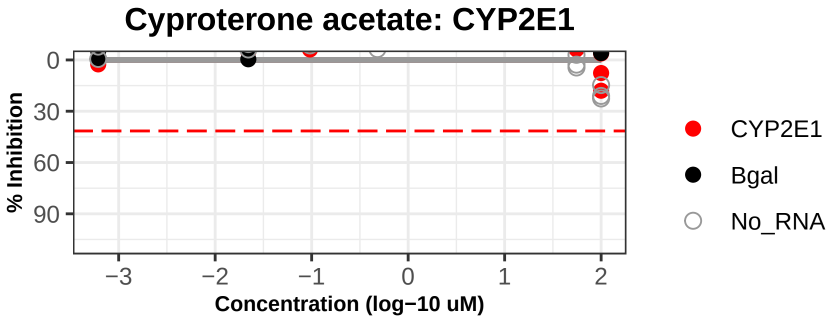
Cyproterone acetate: CYP2E1
% Inhibition
0
30
60
90
−3
−2
−1
0
1
2
Concentration (log−10 uM)
CYP2E1
Bgal
No_RNA

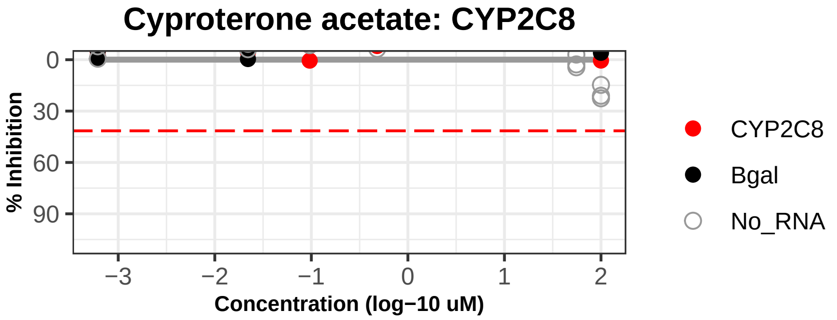
Cyproterone acetate: CYP2C8
% Inhibition
0
30
60
90
−3
−2
−1
0
1
2
Concentration (log−10 uM)
CYP2C8
Bgal
No_RNA

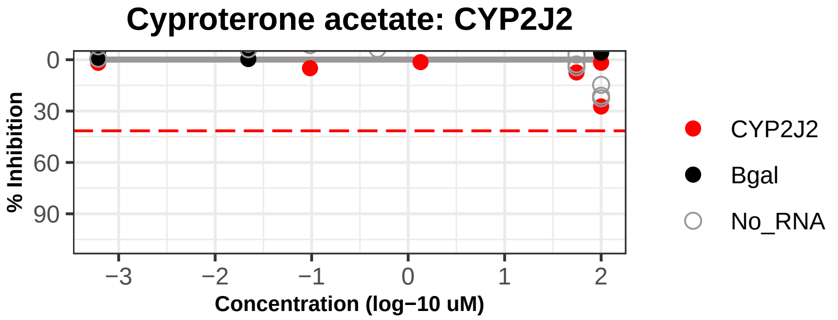
Cyproterone acetate: CYP2J2
% Inhibition
0
30
60
90
−3
−2
−1
0
1
2
Concentration (log−10 uM)
CYP2J2
Bgal
No_RNA

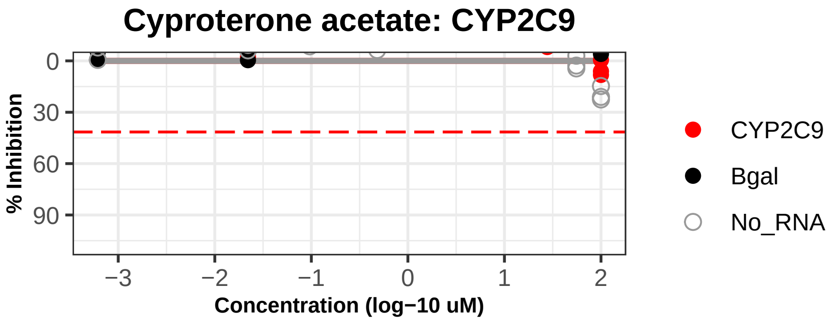
Cyproterone acetate: CYP2C9
% Inhibition
0
30
60
90
−3
−2
−1
0
1
2
Concentration (log−10 uM)
CYP2C9
Bgal
No_RNA

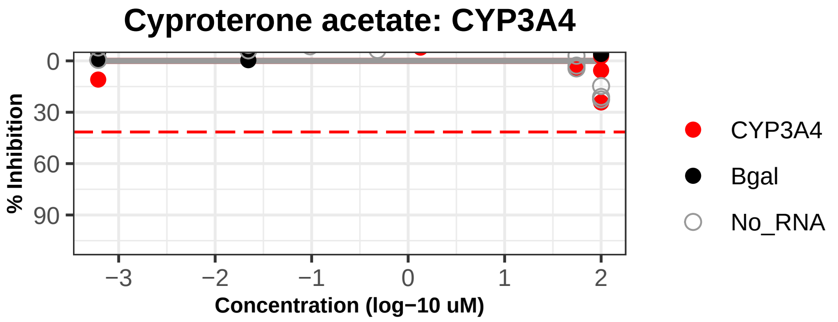
Cyproterone acetate: CYP3A4
% Inhibition
0
30
60
90
−3
−2
−1
0
1
2
Concentration (log−10 uM)
CYP3A4
Bgal
No_RNA

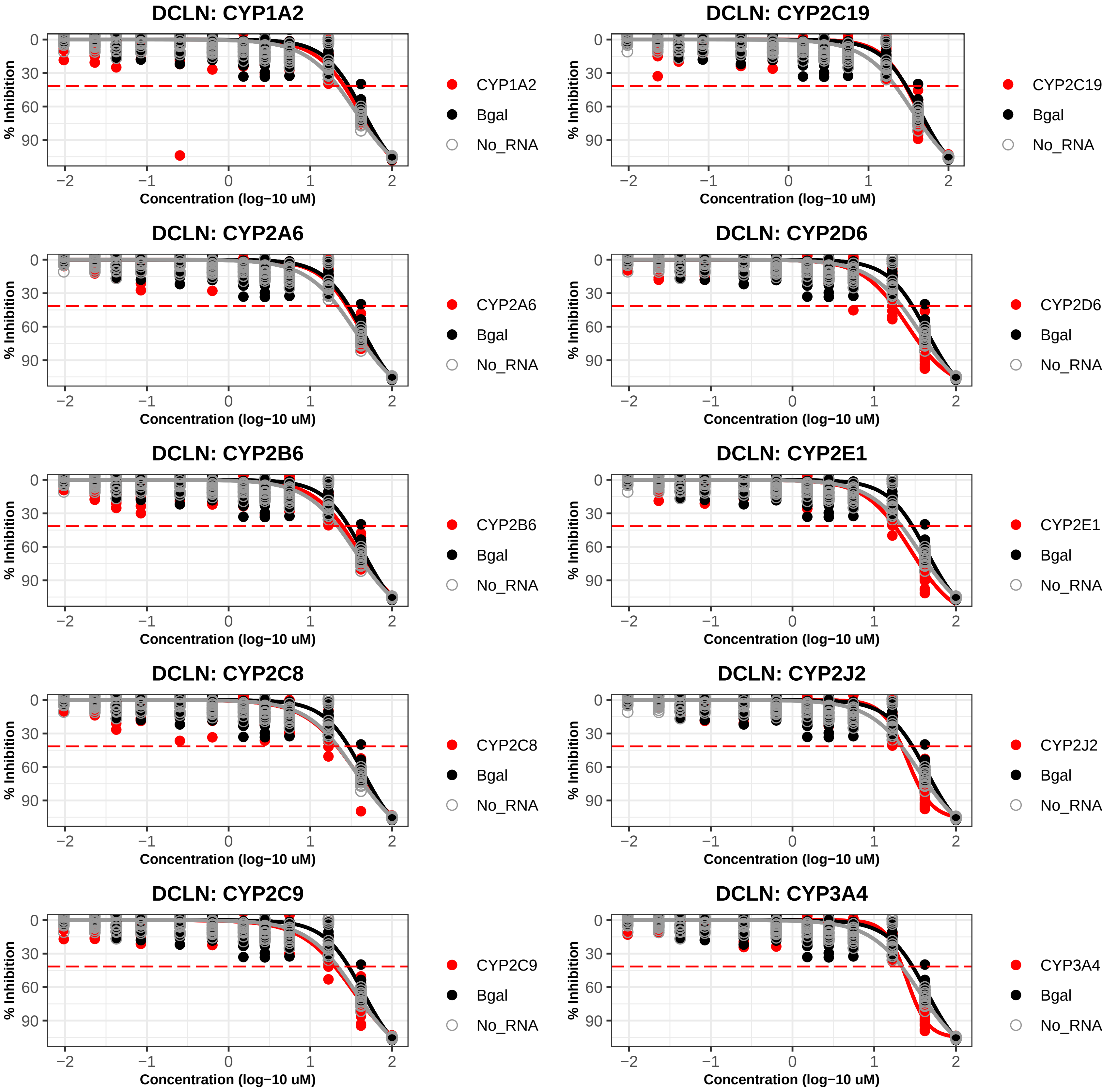
DCLN: CYP1A2
CYP1A2
Bgal
No_RNA
DCLN: CYP2C19
CYP2C19
Bgal
No_RNA
DCLN: CYP2A6
CYP2A6
Bgal
No_RNA
DCLN: CYP2D6
CYP2D6
Bgal
No_RNA
DCLN: CYP2B6
CYP2B6
Bgal
No_RNA
DCLN: CYP2E1
CYP2E1
Bgal
No_RNA
DCLN: CYP2C8
CYP2C8
Bgal
No_RNA
DCLN: CYP2J2
CYP2J2
Bgal
No_RNA
DCLN: CYP2C9
CYP2C9
Bgal
No_RNA
DCLN: CYP3A4
CYP3A4
Bgal
No_RNA
% Inhibition
0
30
60
90
Concentration (log−10 uM)
−2
−1
0
1
2

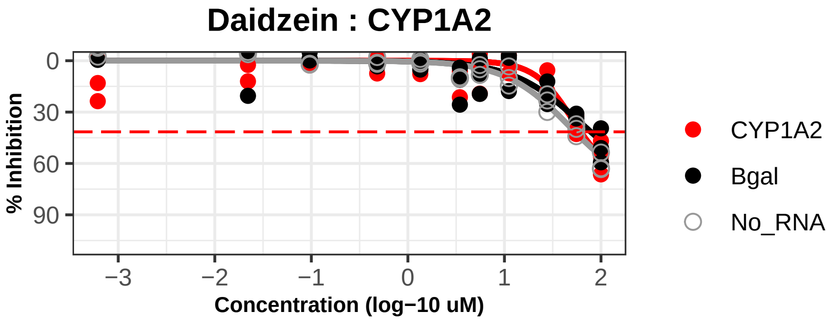
Daidzein : CYP1A2
% Inhibition
Concentration (log−10 uM)
CYP1A2
Bgal
No_RNA

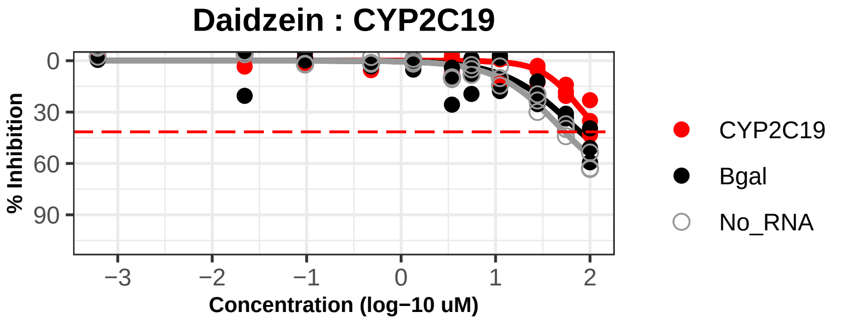
Daidzein : CYP2C19
% Inhibition
Concentration (log−10 uM)
CYP2C19
Bgal
No_RNA

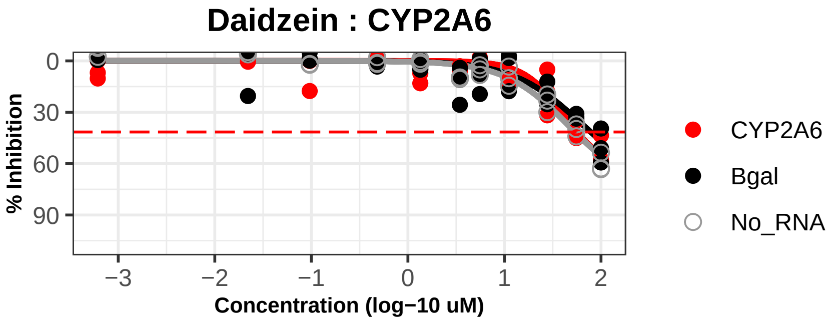
Daidzein : CYP2A6
% Inhibition
Concentration (log−10 uM)
CYP2A6
Bgal
No_RNA

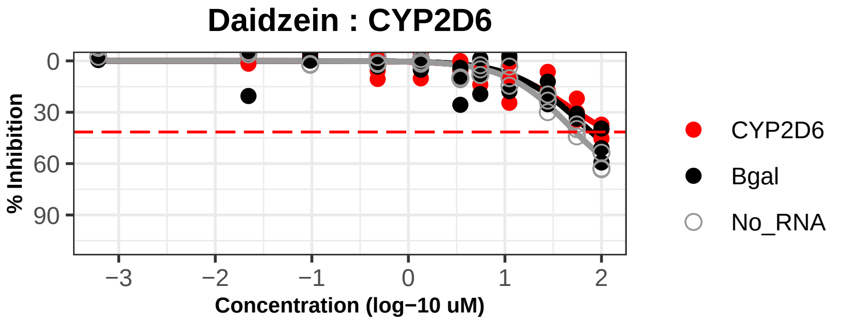
Daidzein : CYP2D6
% Inhibition
Concentration (log−10 uM)
CYP2D6
Bgal
No_RNA

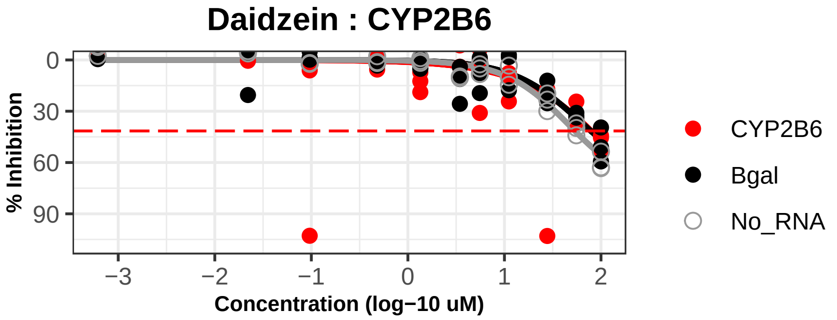
Daidzein : CYP2B6
% Inhibition
Concentration (log−10 uM)
CYP2B6
Bgal
No_RNA

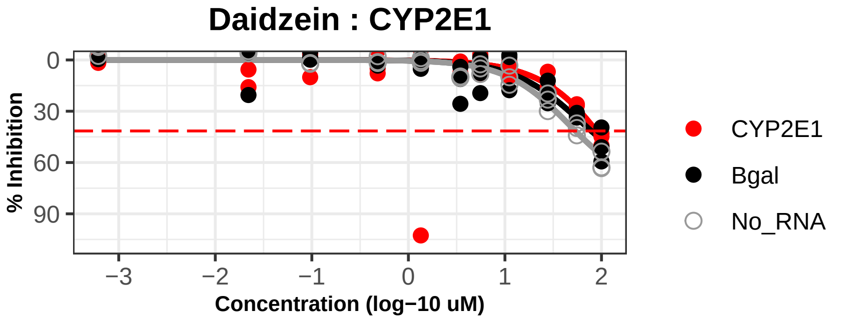
Daidzein : CYP2E1
% Inhibition
Concentration (log−10 uM)
CYP2E1
Bgal
No_RNA

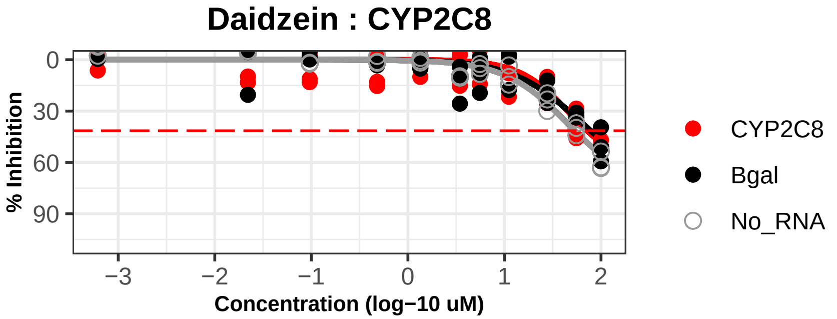
Daidzein : CYP2C8
% Inhibition
Concentration (log−10 uM)
CYP2C8
Bgal
No_RNA

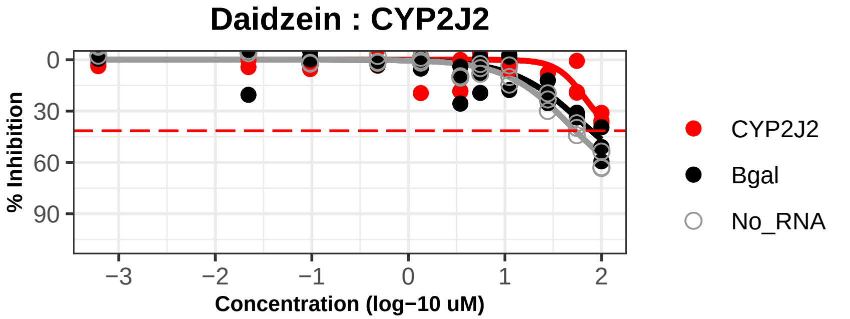
Daidzein : CYP2J2
% Inhibition
Concentration (log−10 uM)
CYP2J2
Bgal
No_RNA

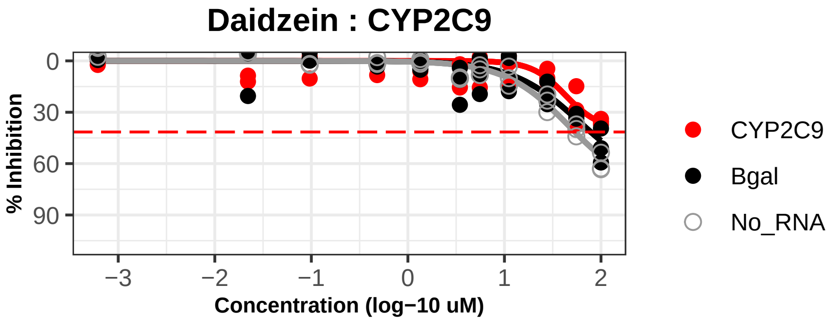
Daidzein : CYP2C9
% Inhibition
Concentration (log−10 uM)
CYP2C9
Bgal
No_RNA

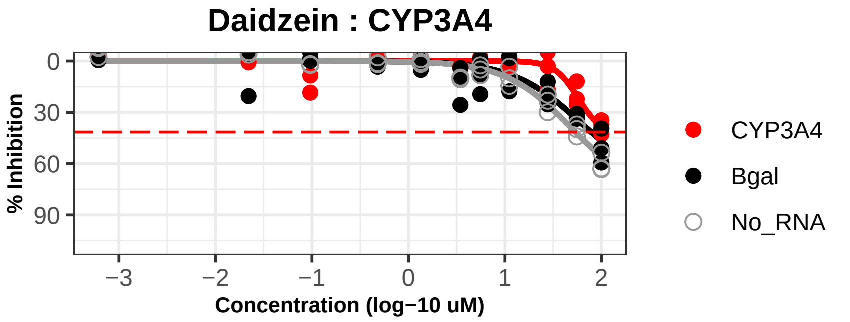
Daidzein : CYP3A4
% Inhibition
Concentration (log−10 uM)
CYP3A4
Bgal
No_RNA

Danazol: CYP1A2

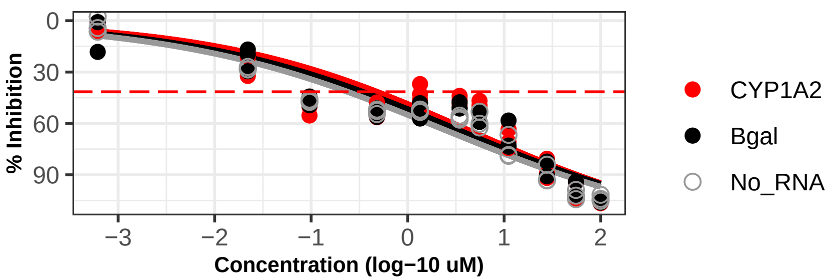

Danazol: CYP2C19

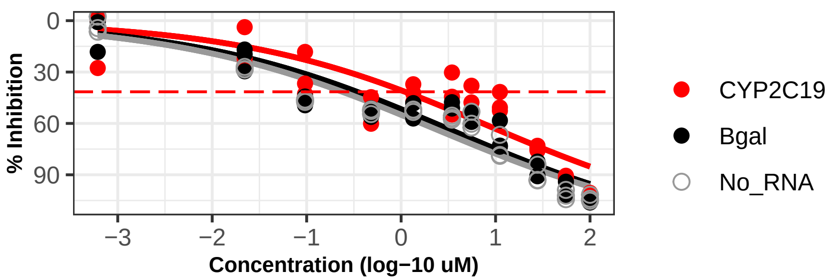

Danazol: CYP2A6

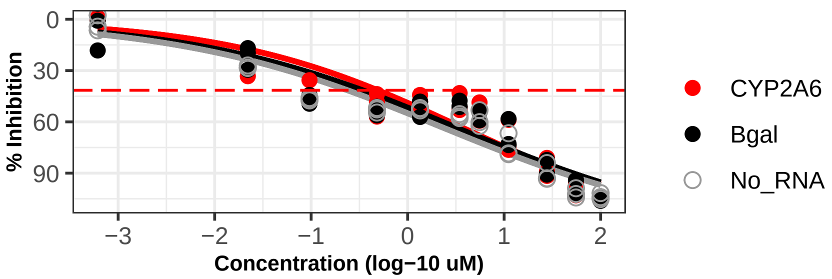

Danazol: CYP2D6

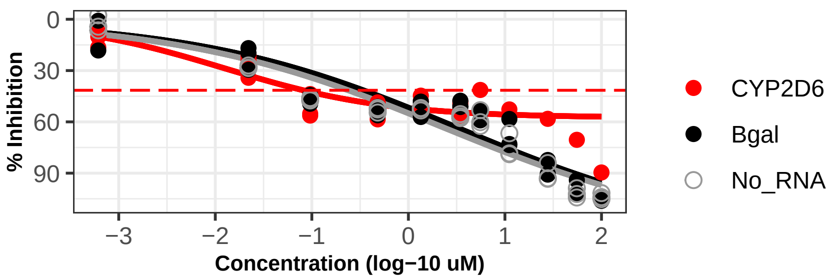

Danazol: CYP2B6

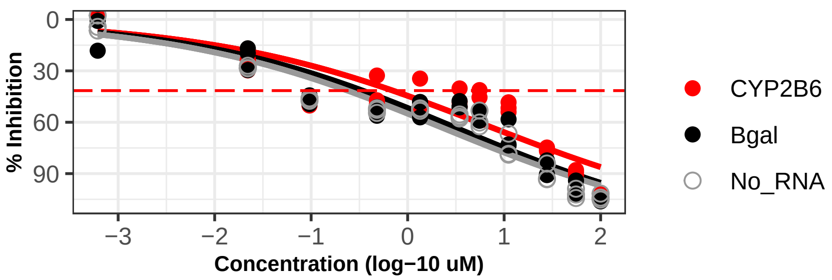

Danazol: CYP2E1

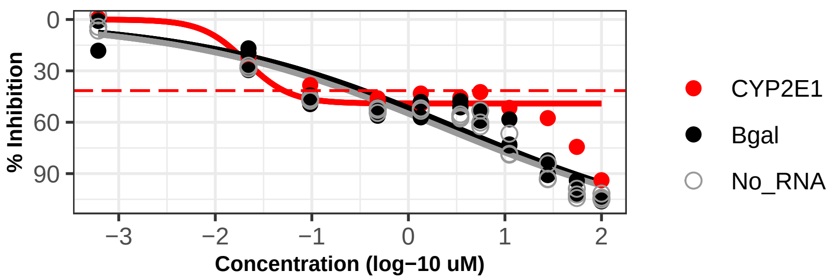

Danazol: CYP2C8

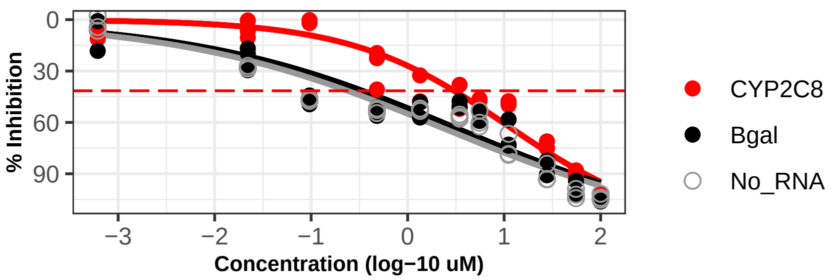

Danazol: CYP2J2

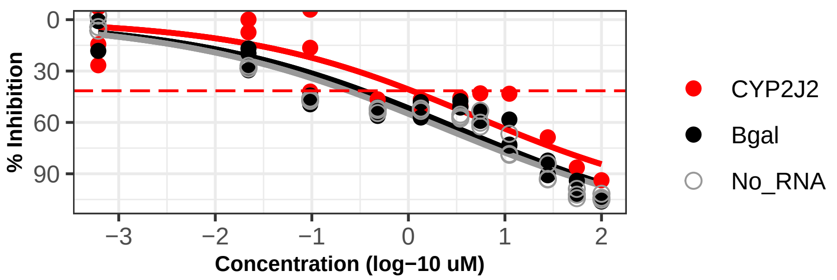

Danazol: CYP2C9

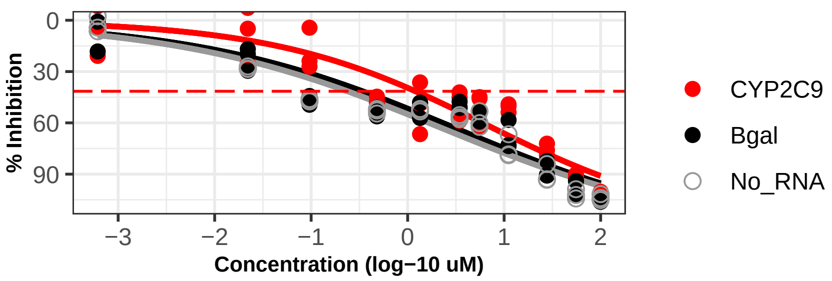

Danazol: CYP3A4

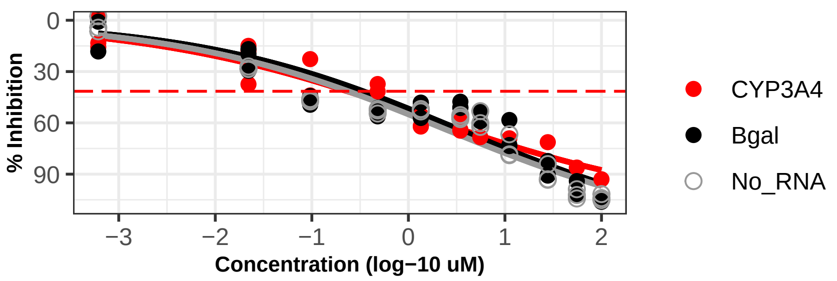

Deltamethrin: CYP1A2

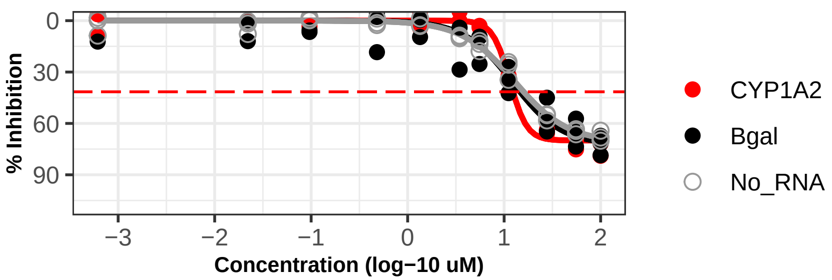

Deltamethrin: CYP2C19

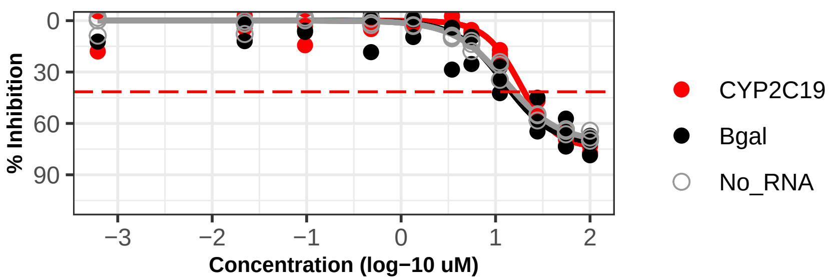

Deltamethrin: CYP2A6

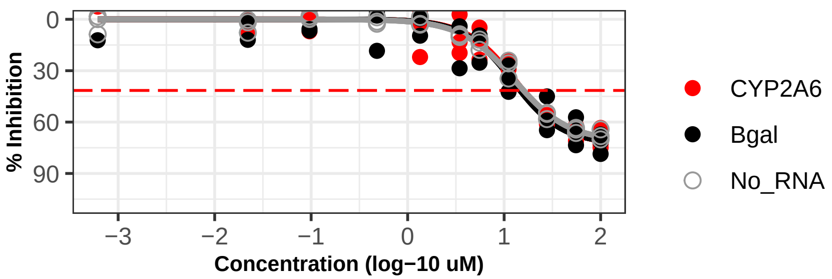

Deltamethrin: CYP2D6

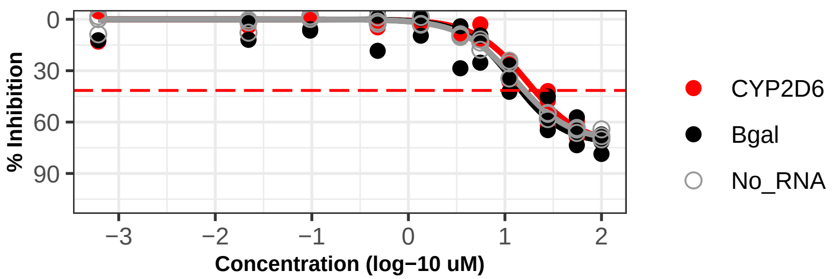

Deltamethrin: CYP2B6

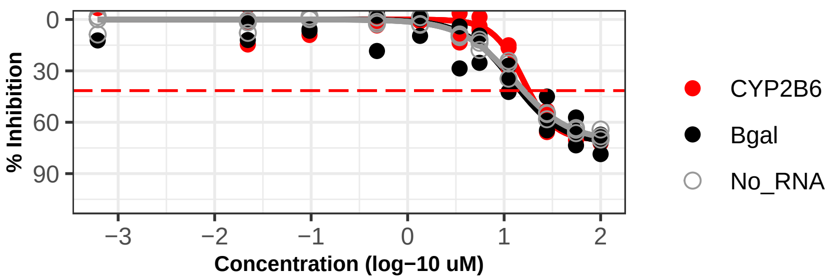

Deltamethrin: CYP2E1

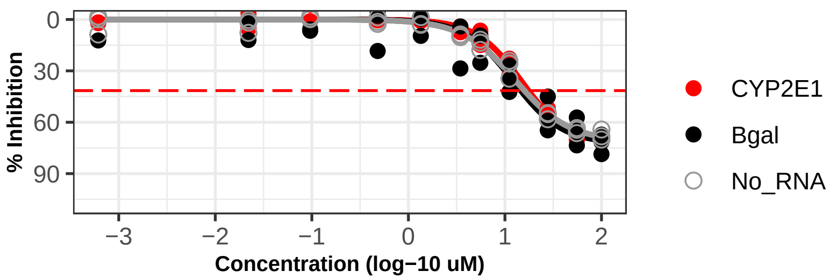

Deltamethrin: CYP2C8

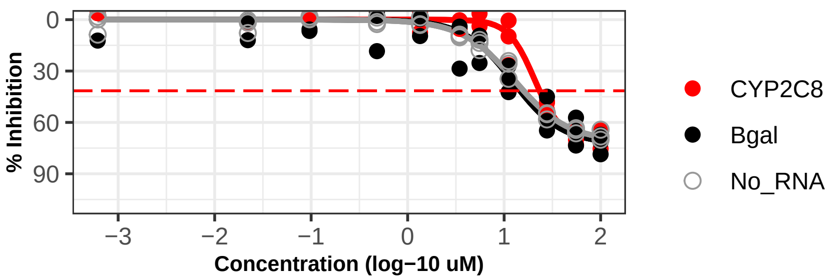

Deltamethrin: CYP2J2

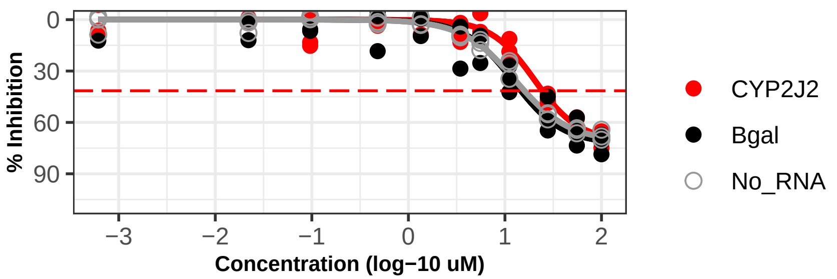

Deltamethrin: CYP2C9

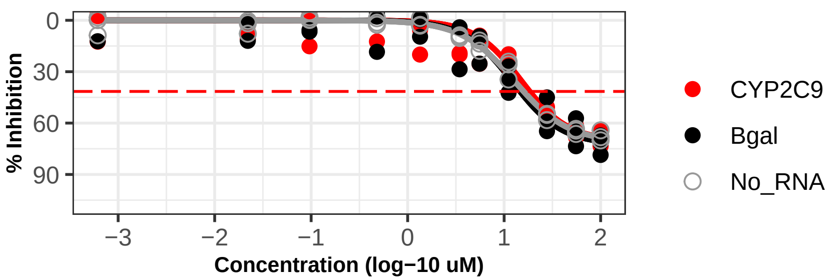

Deltamethrin: CYP3A4

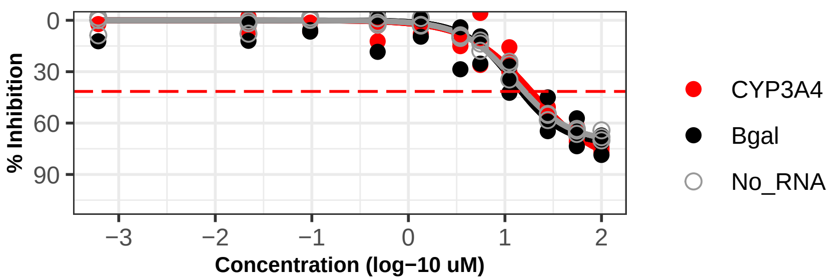

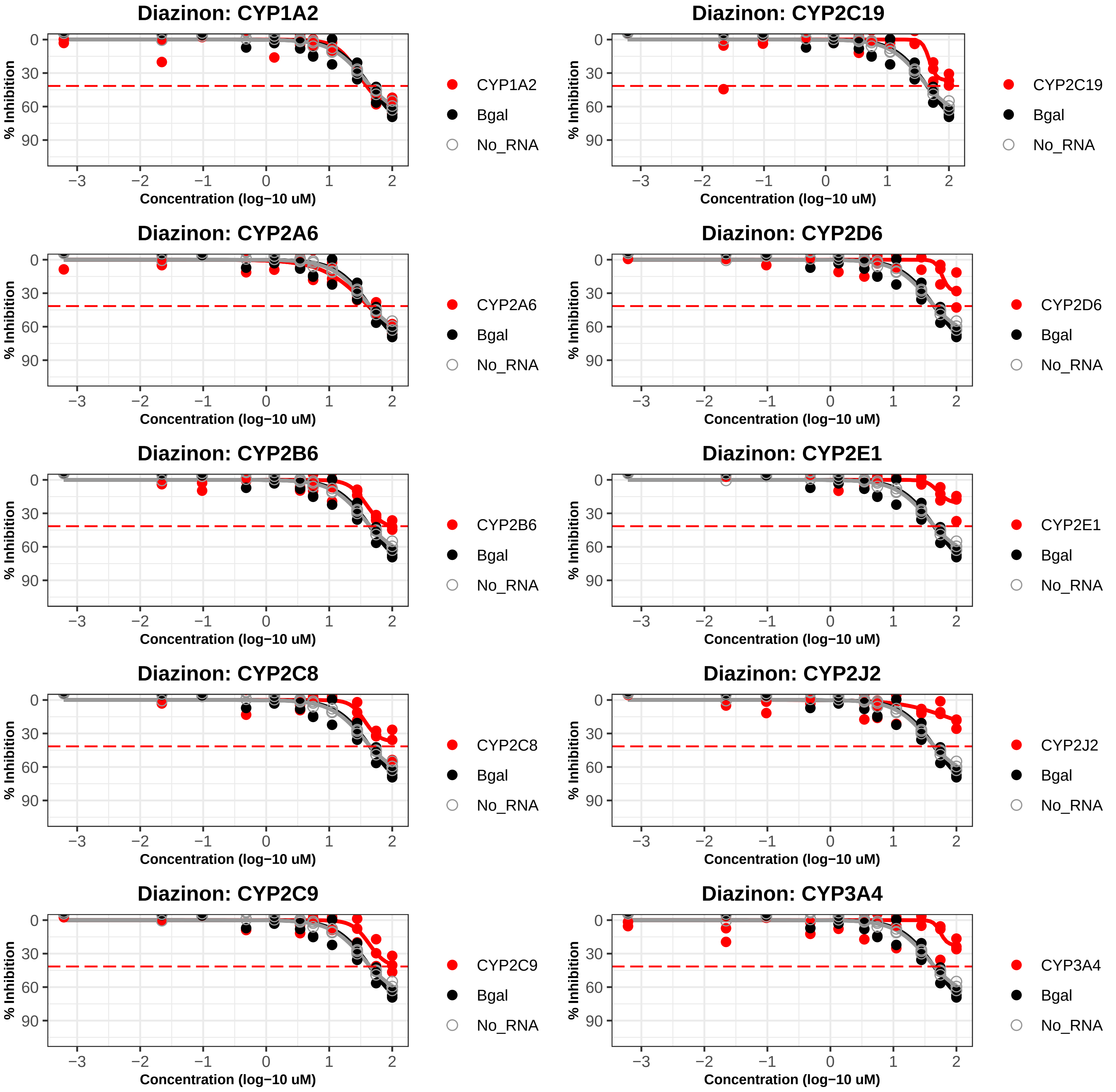
Diazinon: CYP1A2
Diazinon: CYP2C19
Diazinon: CYP2A6
Diazinon: CYP2D6
Diazinon: CYP2B6
Diazinon: CYP2E1
Diazinon: CYP2C8
Diazinon: CYP2J2
Diazinon: CYP2C9
Diazinon: CYP3A4
% Inhibition
0
30
60
90
Concentration (log−10 uM)
−3
−2
−1
0
1
2
CYP1A2
CYP2C19
CYP2A6
CYP2D6
CYP2B6
CYP2E1
CYP2C8
CYP2J2
CYP2C9
CYP3A4
Bgal
No_RNA

Dibutyl phthalate: CYP1A2

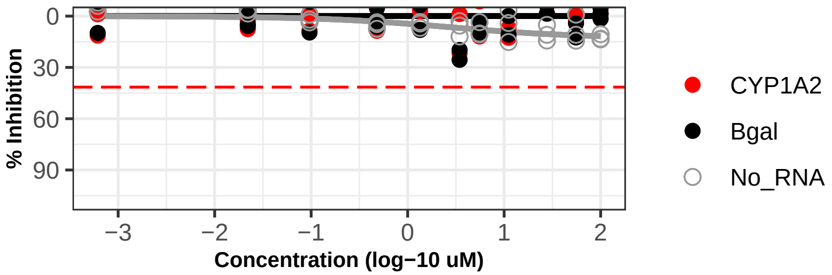

Dibutyl phthalate: CYP2C19

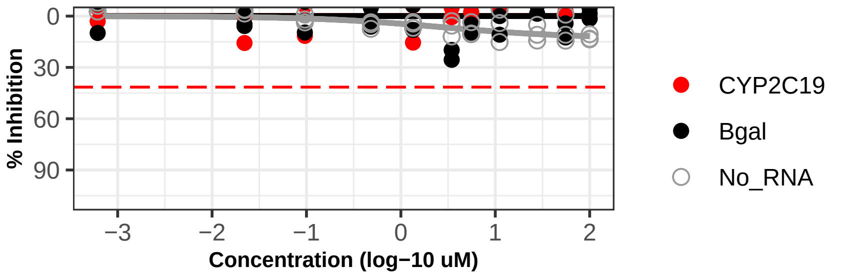

Dibutyl phthalate: CYP2A6

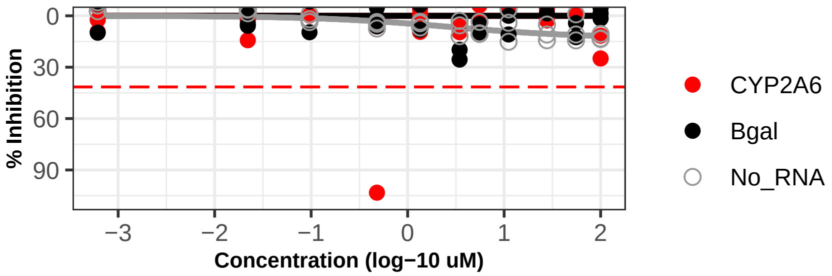

Dibutyl phthalate: CYP2D6

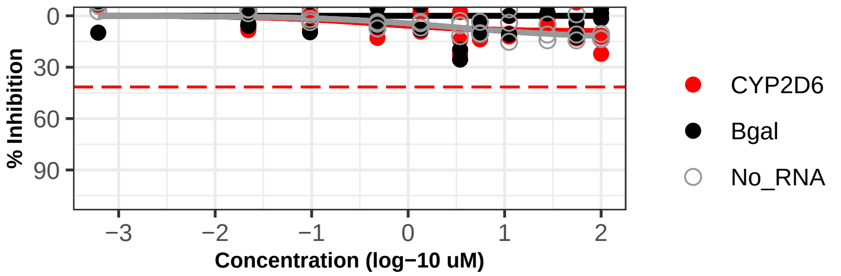

Dibutyl phthalate: CYP2B6

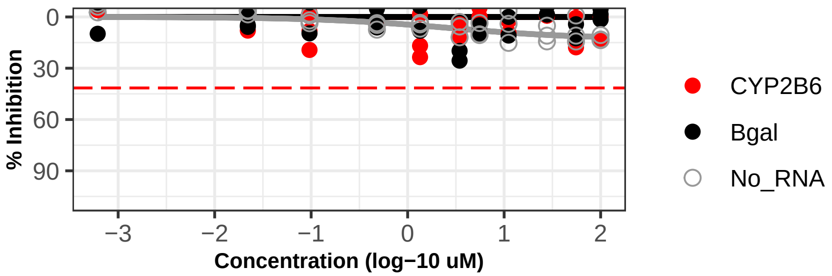

Dibutyl phthalate: CYP2E1

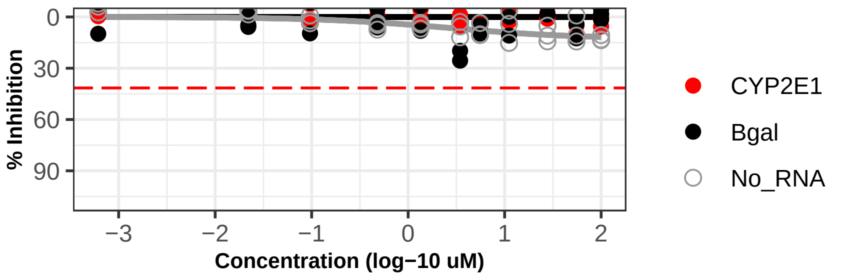

Dibutyl phthalate: CYP2C8

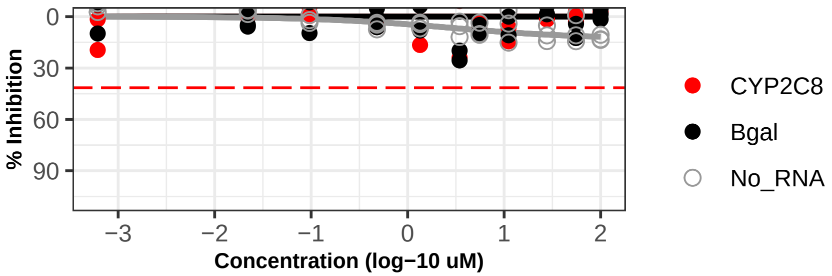

Dibutyl phthalate: CYP2J2

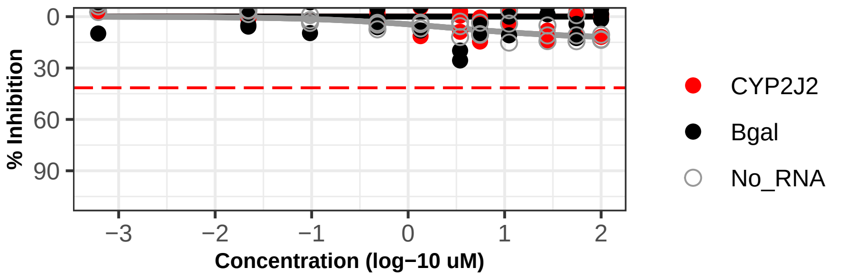

Dibutyl phthalate: CYP2C9

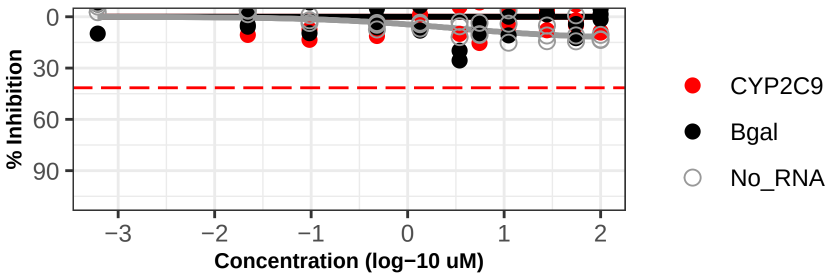

Dibutyl phthalate: CYP3A4

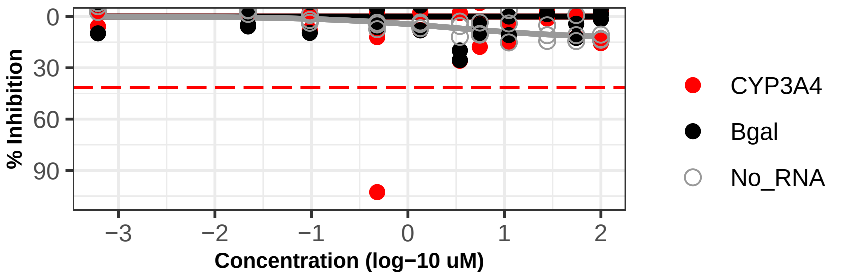

Dichlobenil: CYP1A2

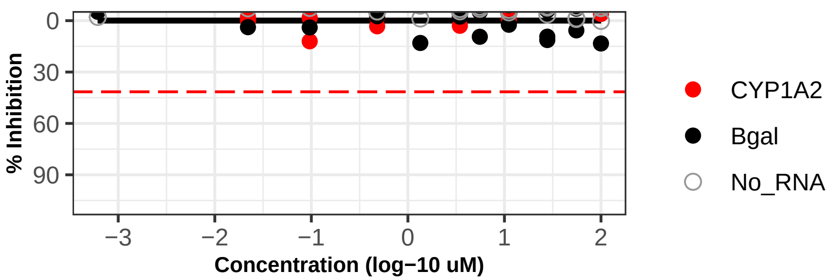

Dichlobenil: CYP2C19

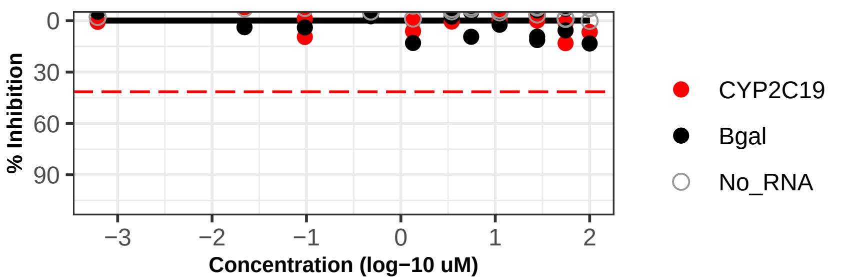

Dichlobenil: CYP2A6

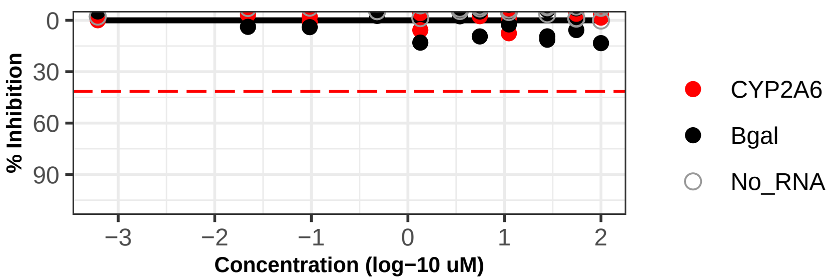

Dichlobenil: CYP2D6

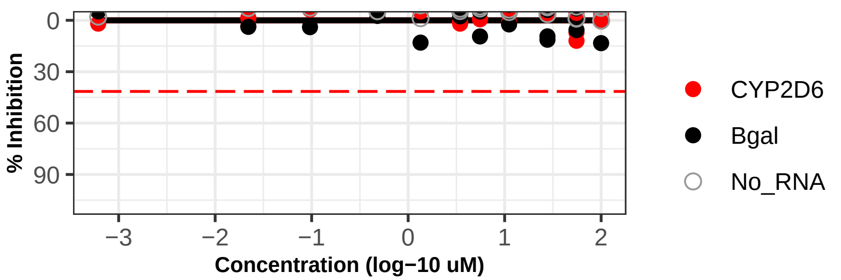

Dichlobenil: CYP2B6

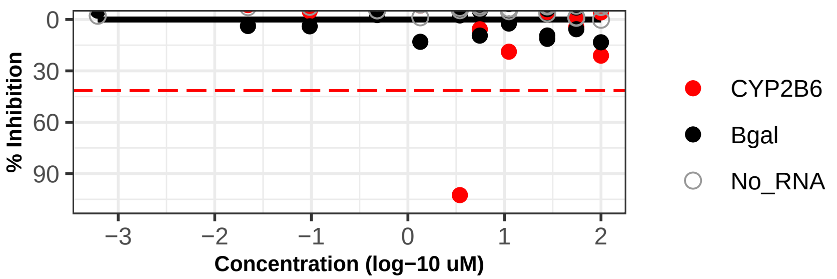

Dichlobenil: CYP2E1

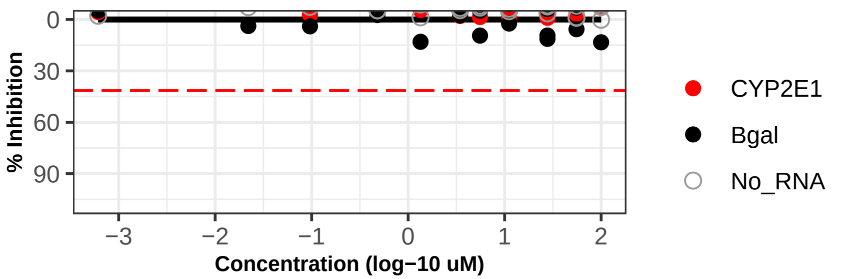

Dichlobenil: CYP2C8

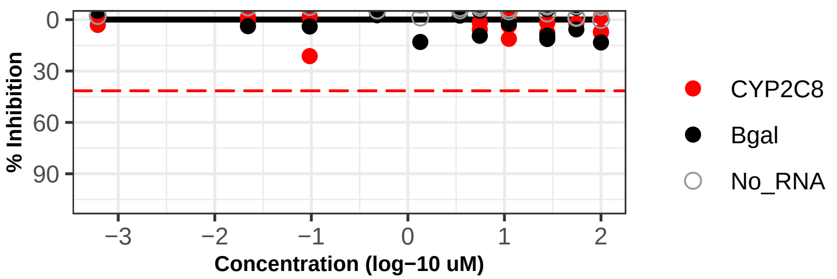

Dichlobenil: CYP2J2

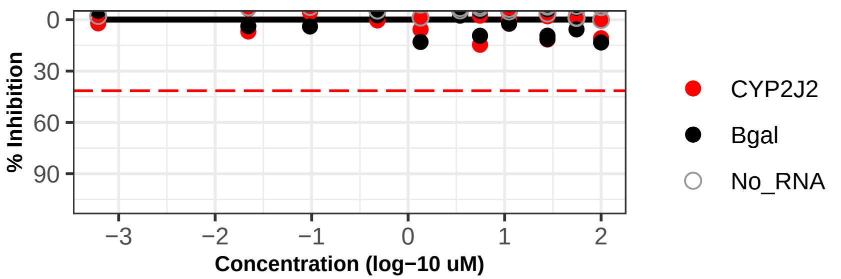

Dichlobenil: CYP2C9

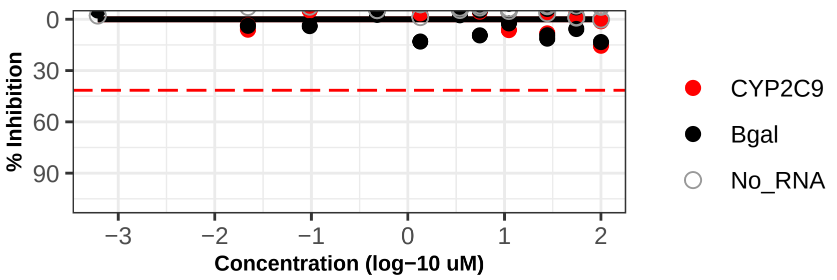

Dichlobenil: CYP3A4

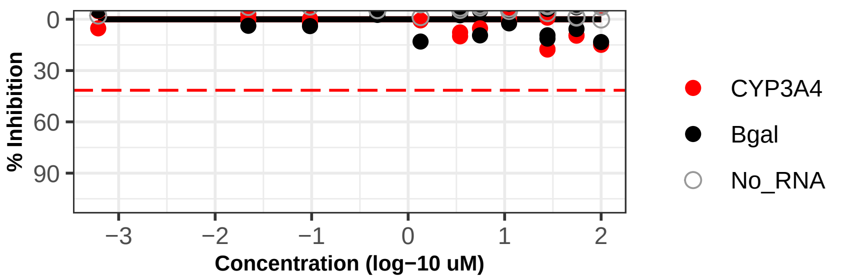

Dichlorodiphenyltrichloroethane: CYP1A2

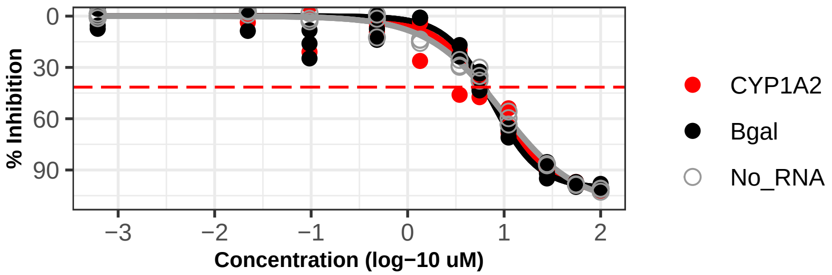

Dichlorodiphenyltrichloroethane: CYP2C19

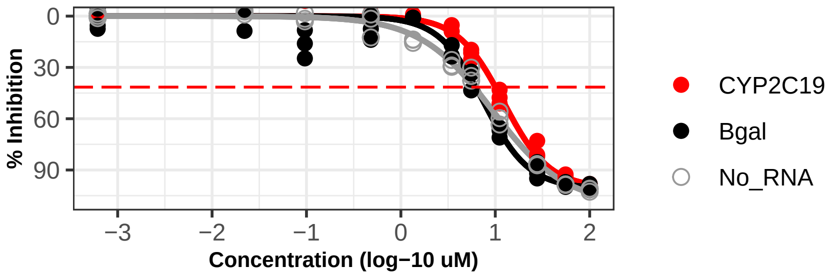

Dichlorodiphenyltrichloroethane: CYP2A6

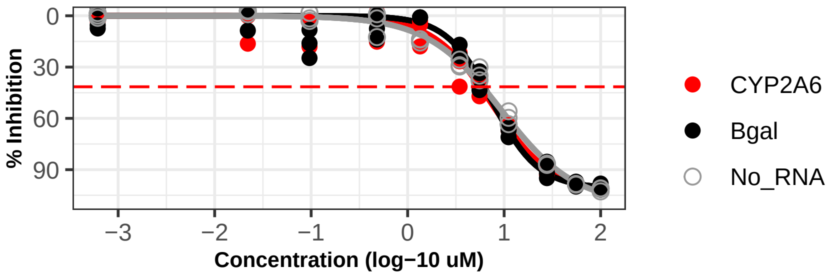

Dichlorodiphenyltrichloroethane: CYP2D6

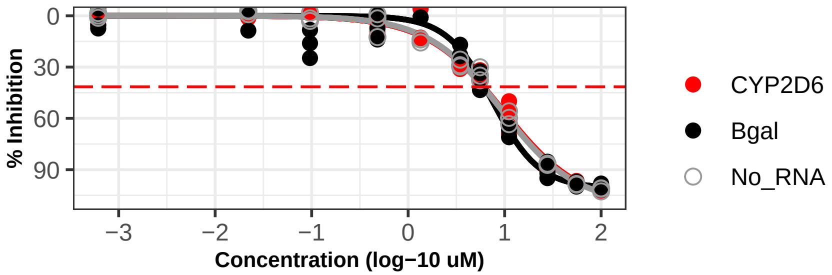

Dichlorodiphenyltrichloroethane: CYP2B6

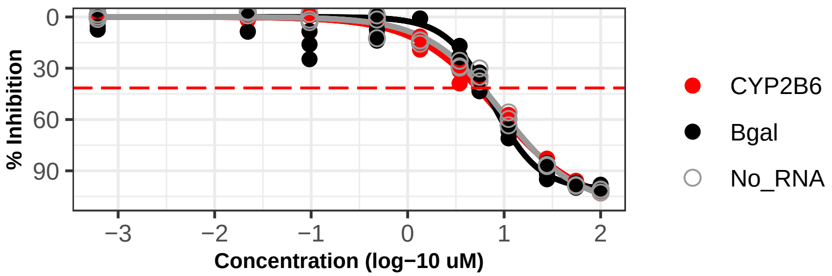

Dichlorodiphenyltrichloroethane: CYP2E1

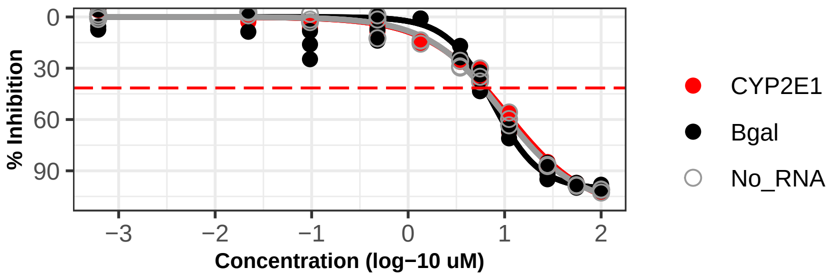

Dichlorodiphenyltrichloroethane: CYP2C8

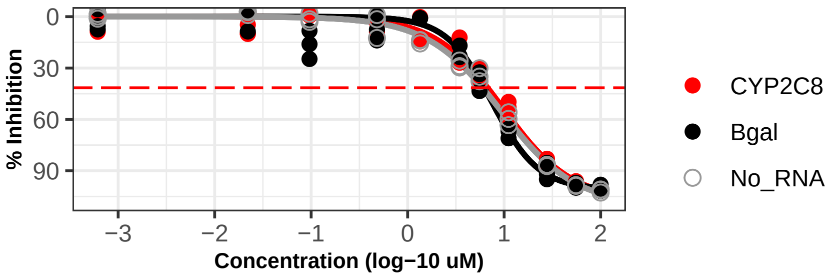

Dichlorodiphenyltrichloroethane: CYP2J2

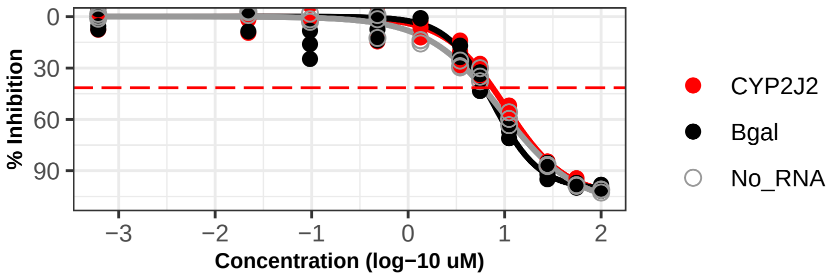

Dichlorodiphenyltrichloroethane: CYP2C9

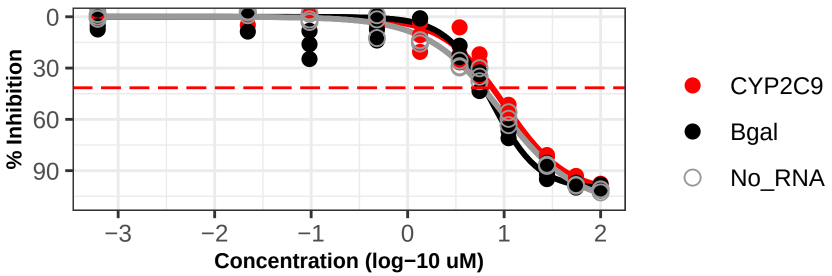

Dichlorodiphenyltrichloroethane: CYP3A4

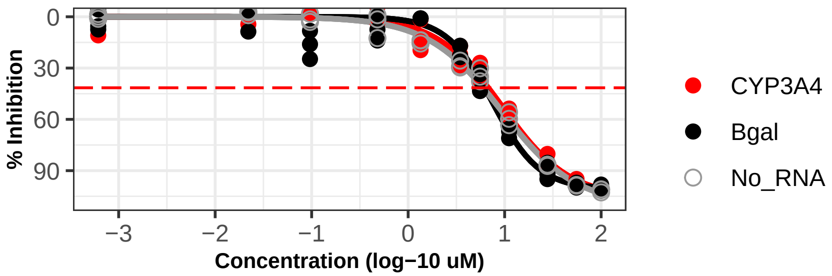

Dicyclohexyl phthalate: CYP1A2

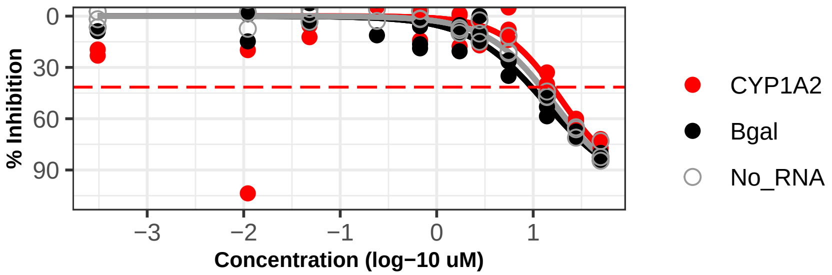

Dicyclohexyl phthalate: CYP2C19

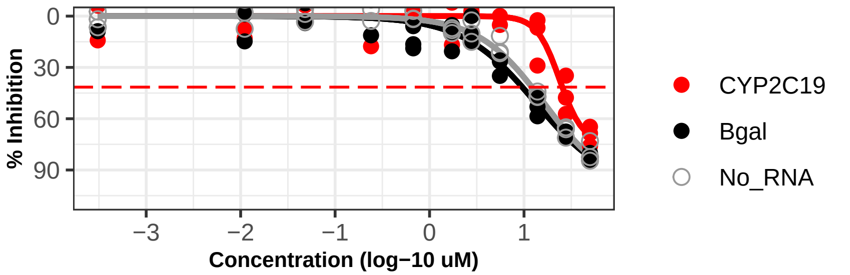

Dicyclohexyl phthalate: CYP2A6

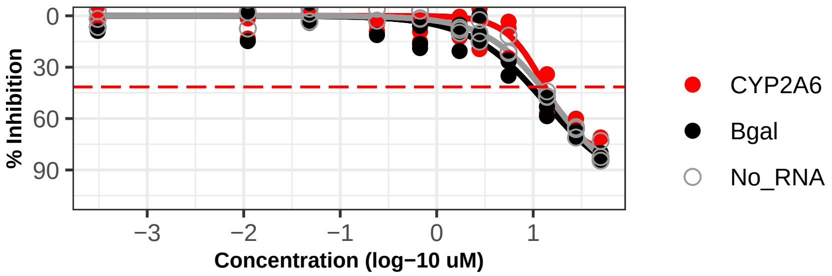

Dicyclohexyl phthalate: CYP2D6

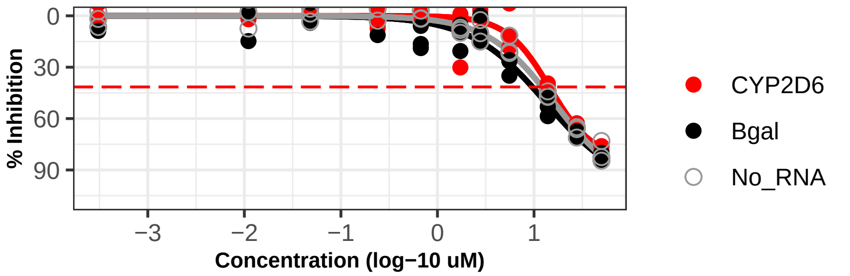

Dicyclohexyl phthalate: CYP2B6

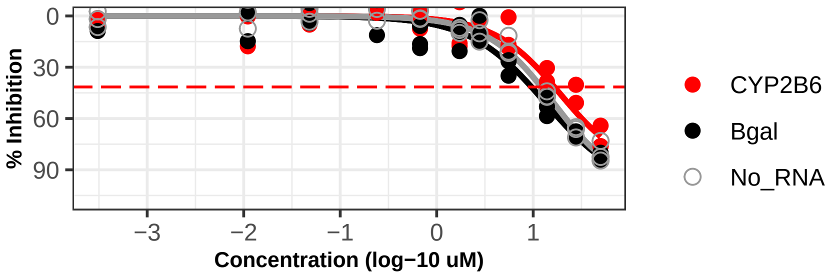

Dicyclohexyl phthalate: CYP2E1

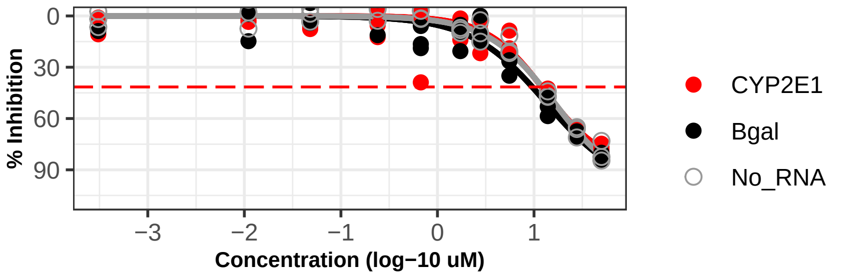

Dicyclohexyl phthalate: CYP2C8

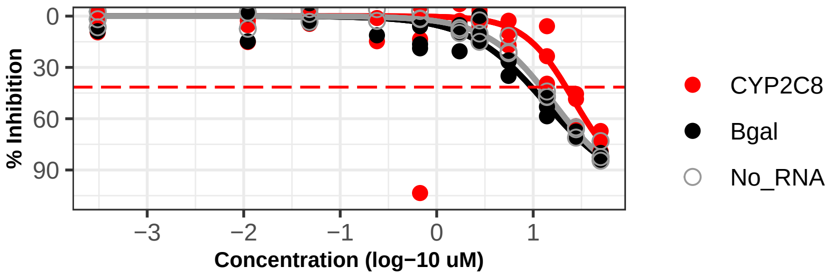

Dicyclohexyl phthalate: CYP2J2

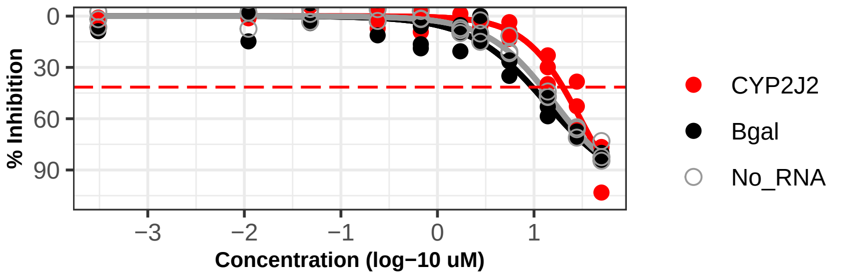

Dicyclohexyl phthalate: CYP2C9

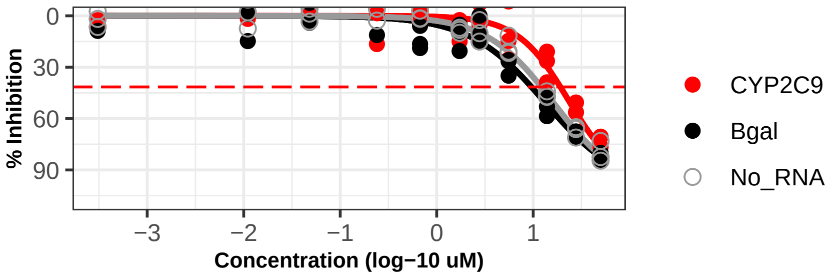

Dicyclohexyl phthalate: CYP3A4

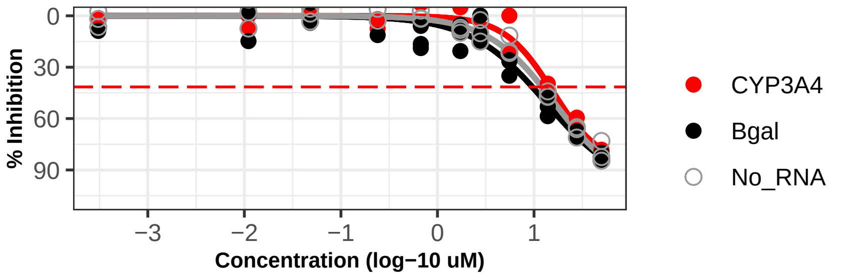

Diethyl phthalate: CYP1A2

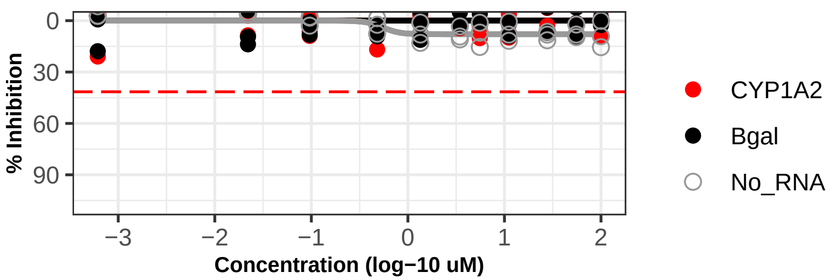

Diethyl phthalate: CYP2C19

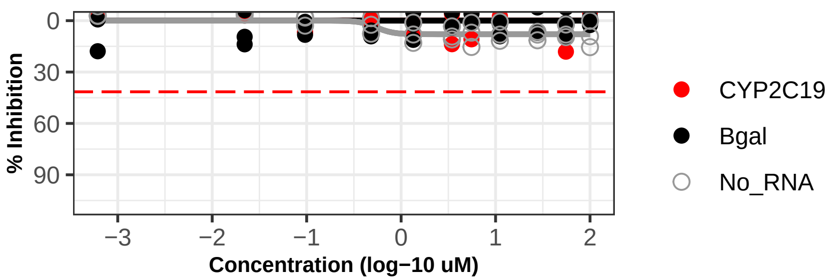

Diethyl phthalate: CYP2A6

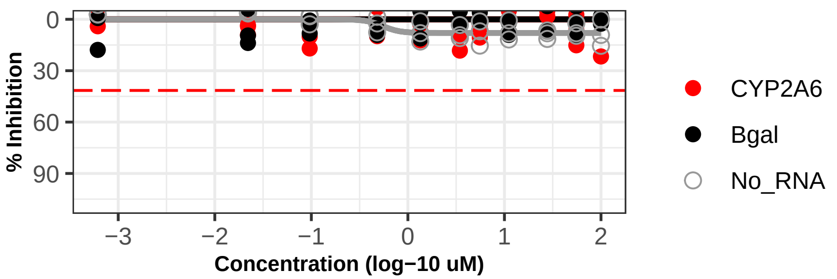

Diethyl phthalate: CYP2D6

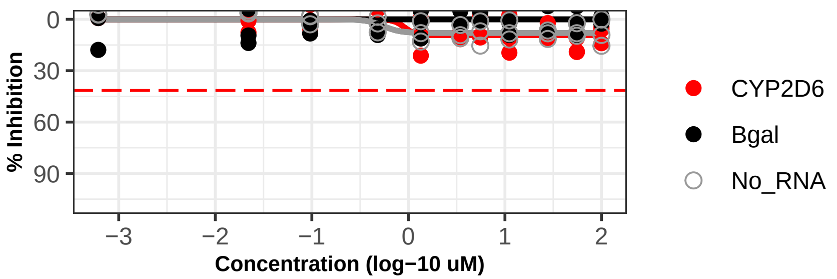

Diethyl phthalate: CYP2B6

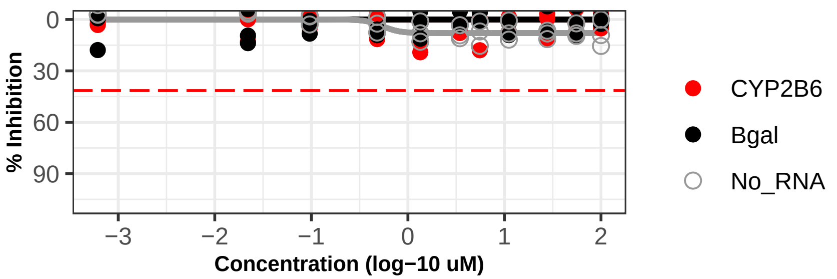

Diethyl phthalate: CYP2E1

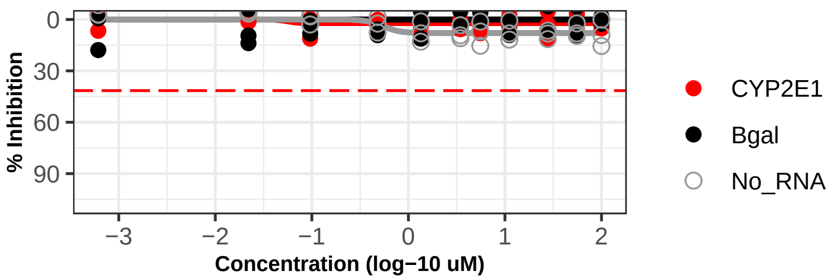

Diethyl phthalate: CYP2C8

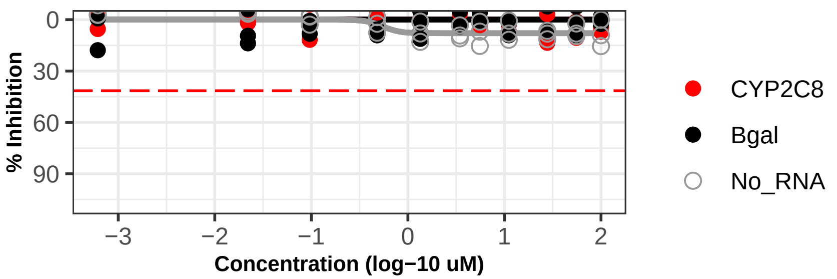

Diethyl phthalate: CYP2J2

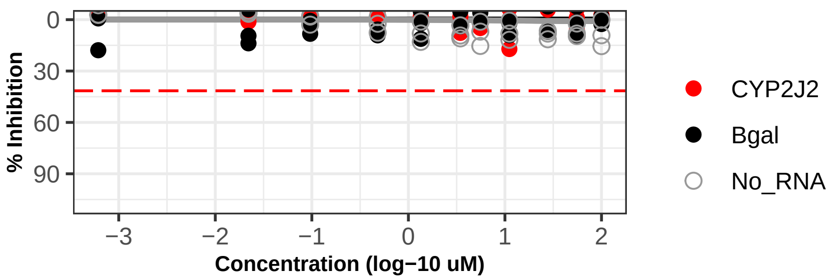

Diethyl phthalate: CYP2C9

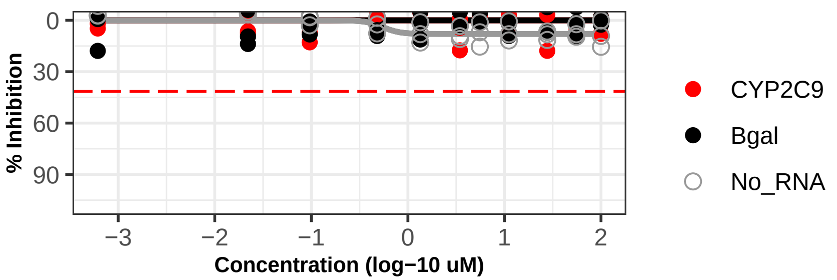

Diethyl phthalate: CYP3A4

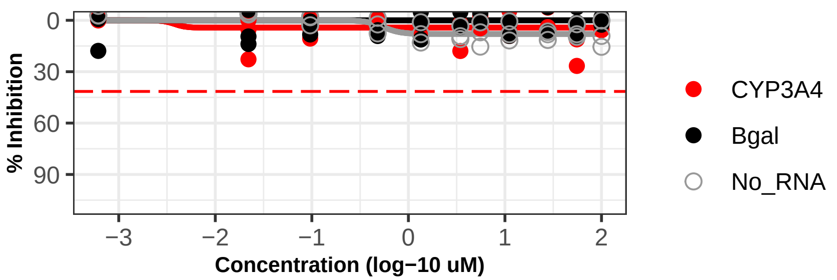

Diethylstilbestrol: CYP1A2

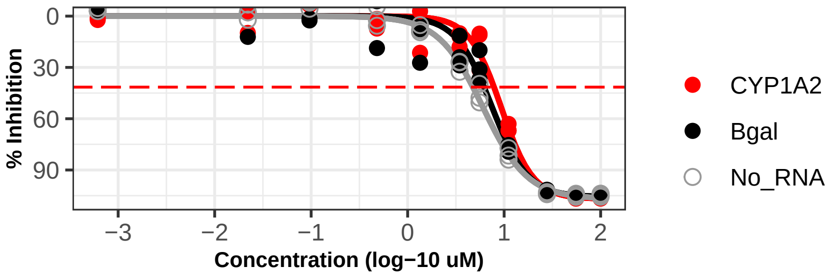

Diethylstilbestrol: CYP2C19

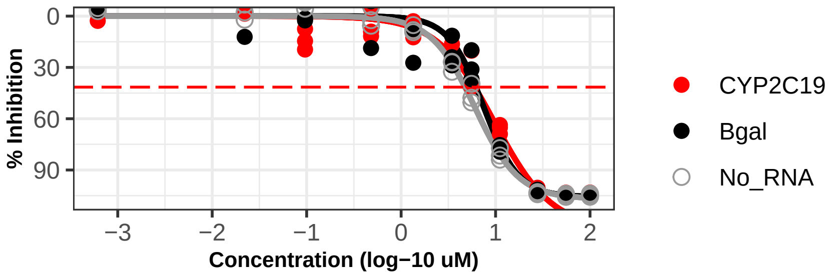

Diethylstilbestrol: CYP2A6

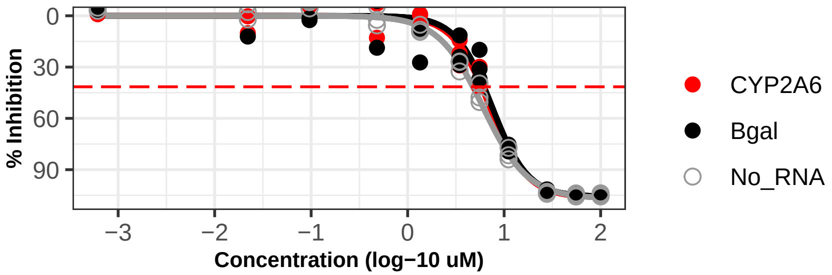

Diethylstilbestrol: CYP2D6

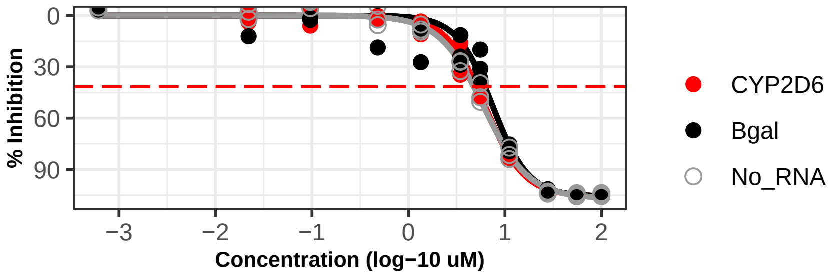

Diethylstilbestrol: CYP2B6

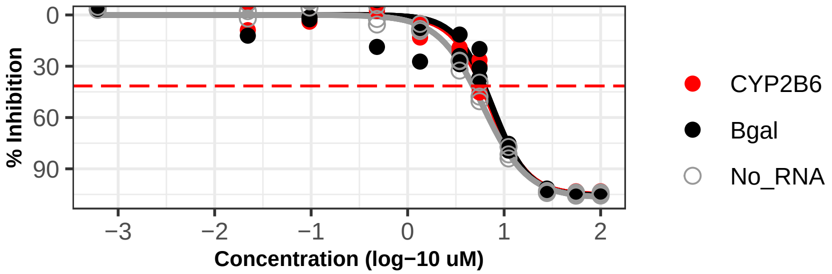

Diethylstilbestrol: CYP2E1

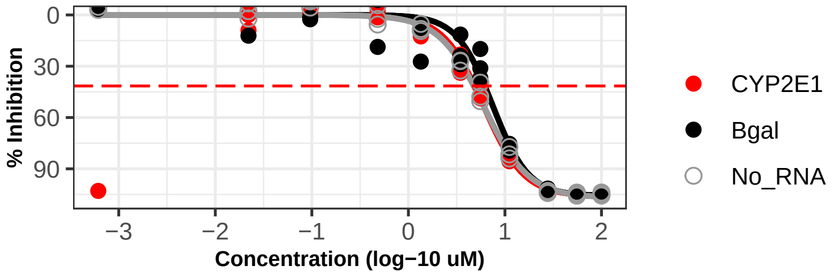

Diethylstilbestrol: CYP2C8

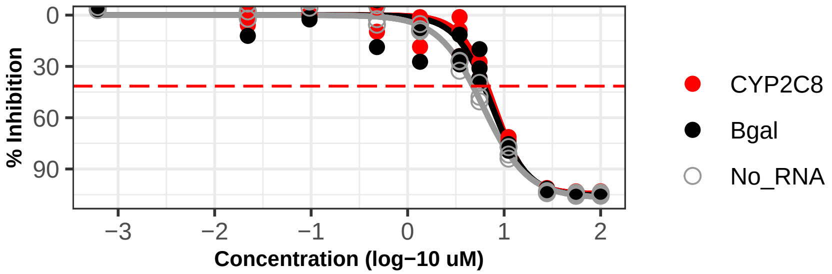

Diethylstilbestrol: CYP2J2

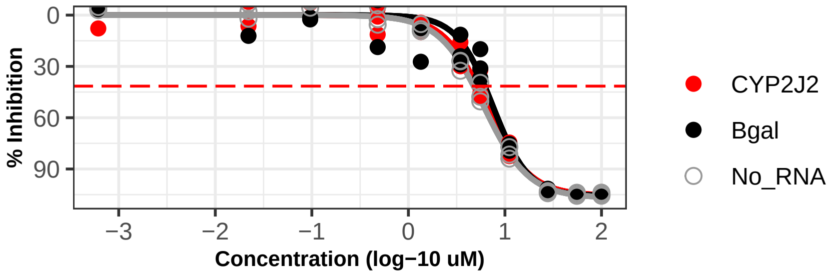

Diethylstilbestrol: CYP2C9

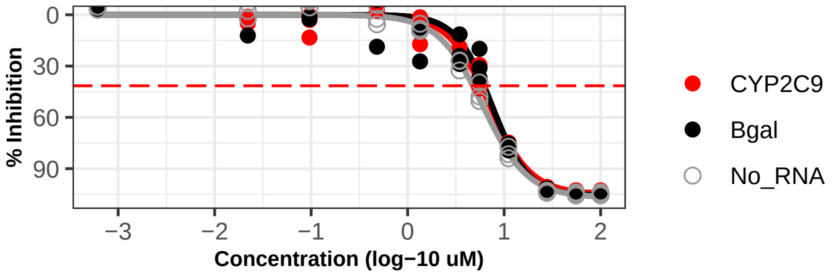

Diethylstilbestrol: CYP3A4

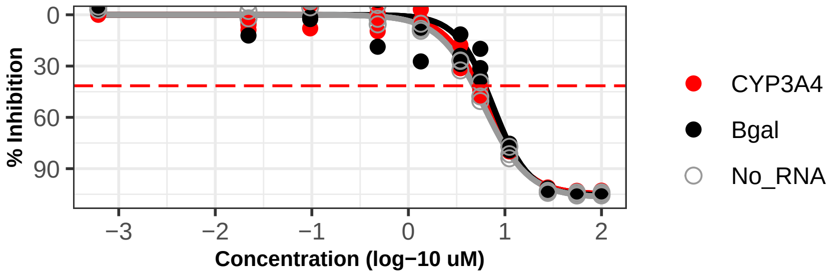

Dihexyl phthalate: CYP1A2

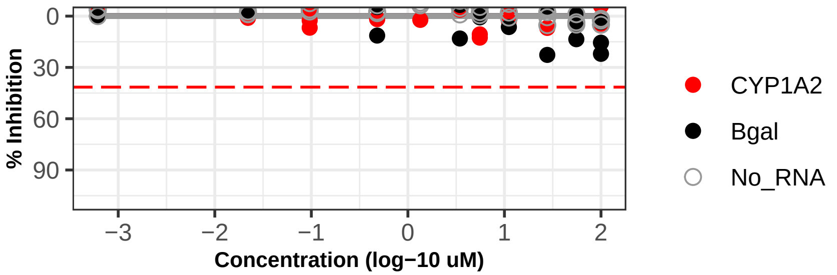

Dihexyl phthalate: CYP2C19

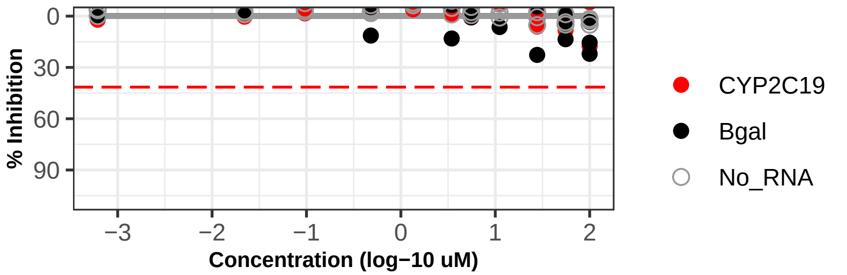

Dihexyl phthalate: CYP2A6

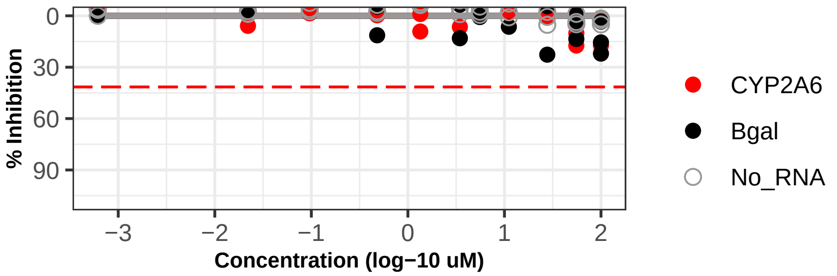

Dihexyl phthalate: CYP2D6

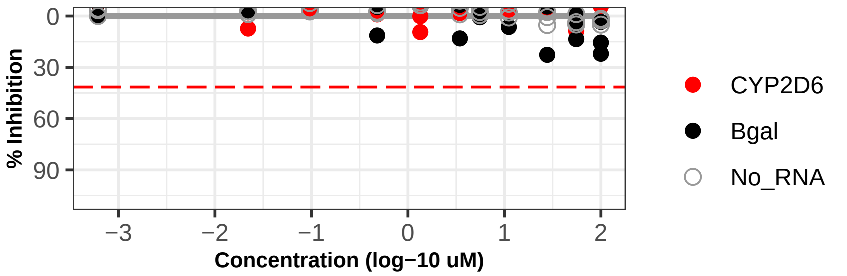

Dihexyl phthalate: CYP2B6

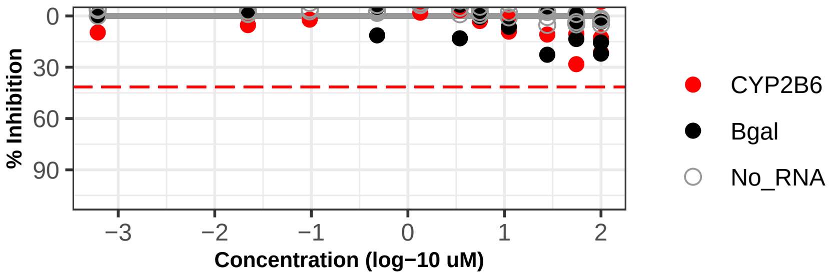

Dihexyl phthalate: CYP2E1

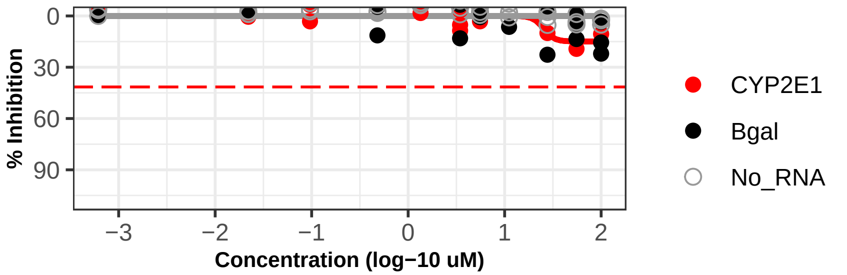

Dihexyl phthalate: CYP2C8

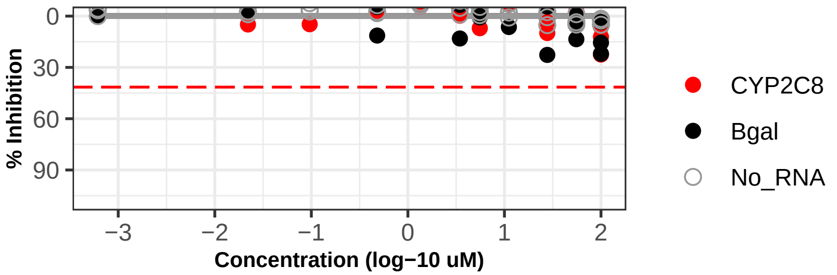

Dihexyl phthalate: CYP2J2

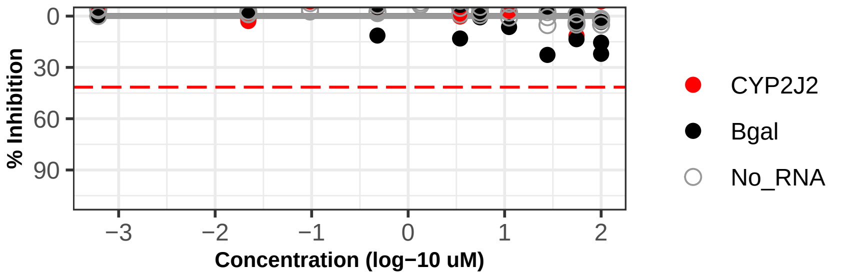

Dihexyl phthalate: CYP2C9

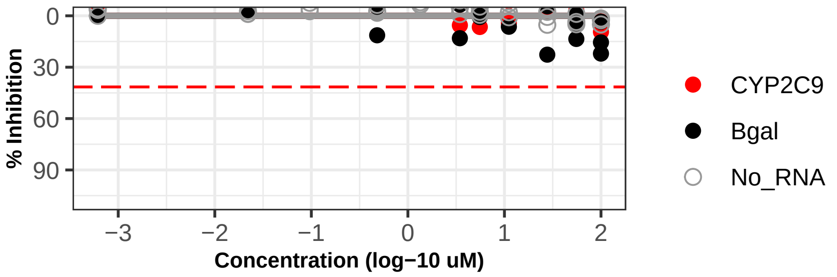

Dihexyl phthalate: CYP3A4

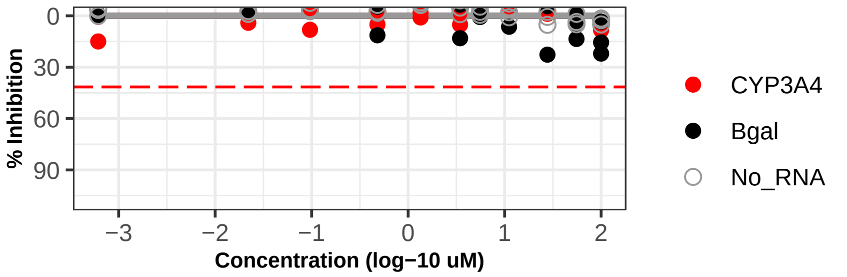

Dipentyl phthalate: CYP1A2

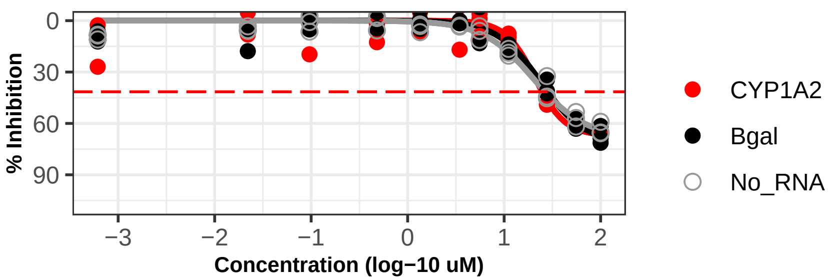

Dipentyl phthalate: CYP2C19

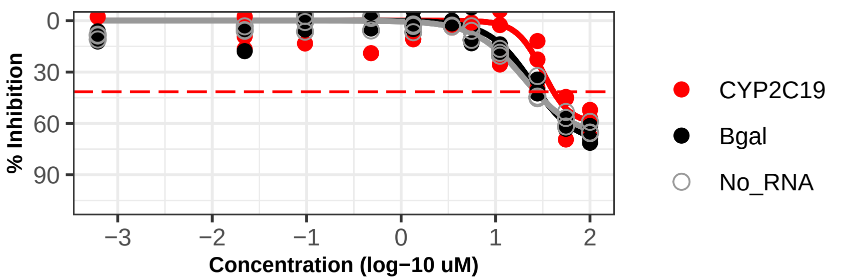

Dipentyl phthalate: CYP2A6

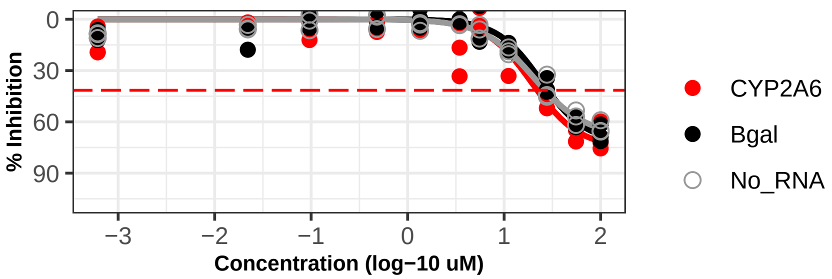

Dipentyl phthalate: CYP2D6

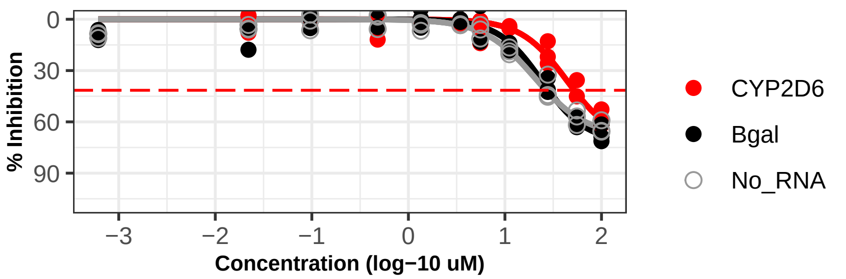

Dipentyl phthalate: CYP2B6

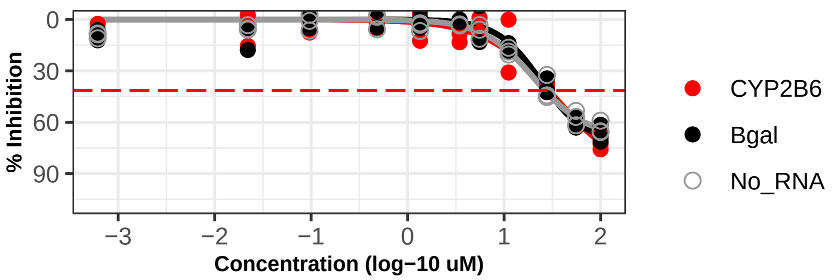

Dipentyl phthalate: CYP2E1

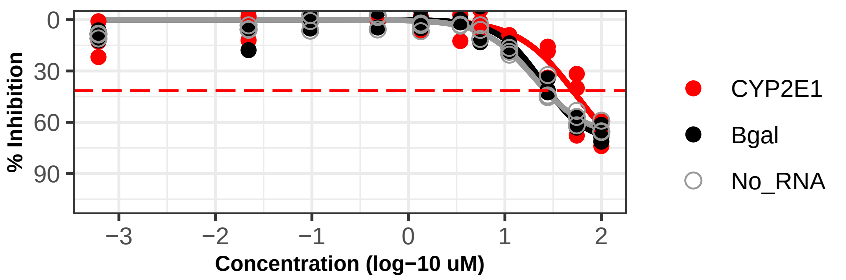

Dipentyl phthalate: CYP2C8

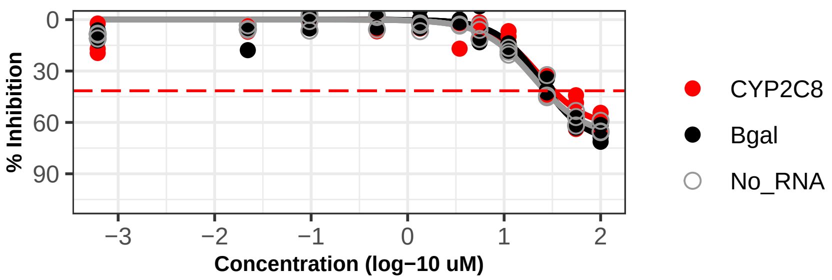

Dipentyl phthalate: CYP2J2

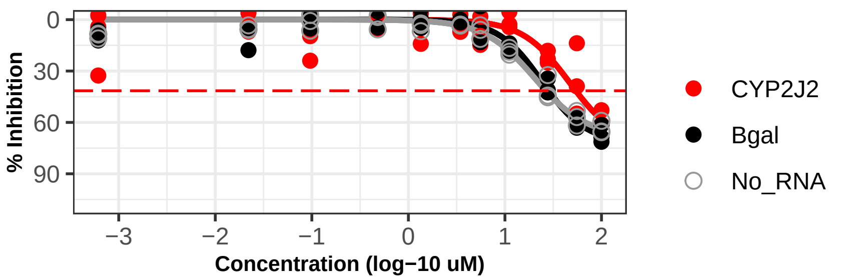

Dipentyl phthalate: CYP2C9

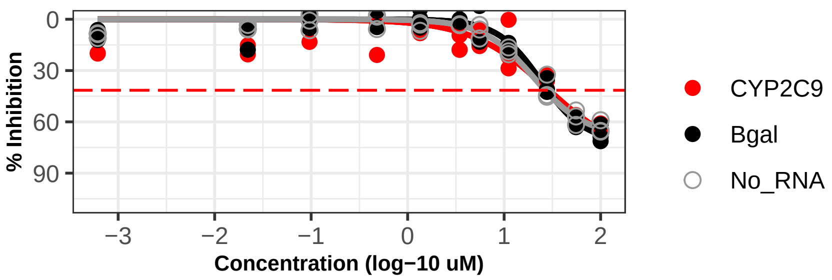

Dipentyl phthalate: CYP3A4

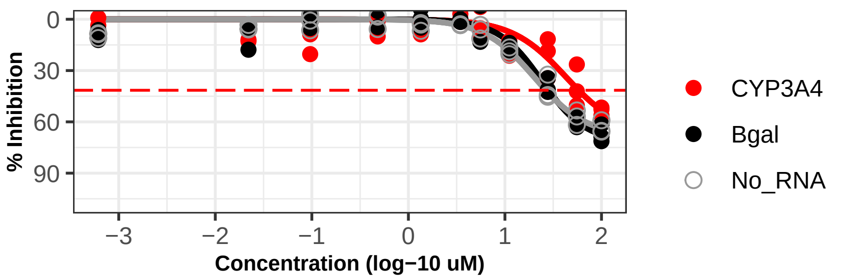

Enclomiphene hydrochloride: CYP1A2

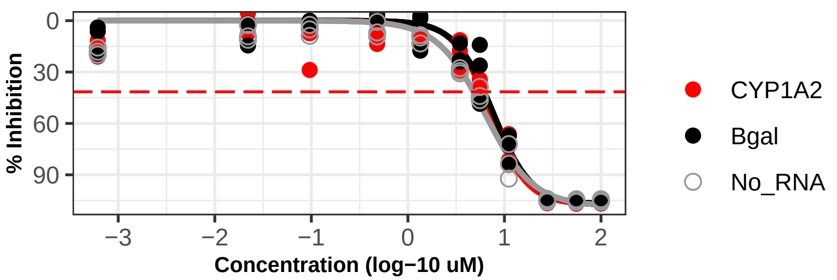

Enclomiphene hydrochloride: CYP2A6

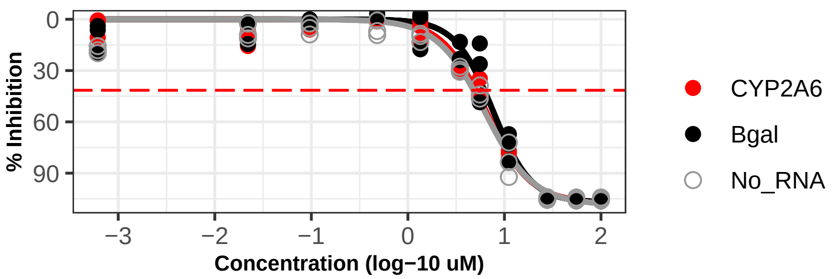

Enclomiphene hydrochloride: CYP2B6

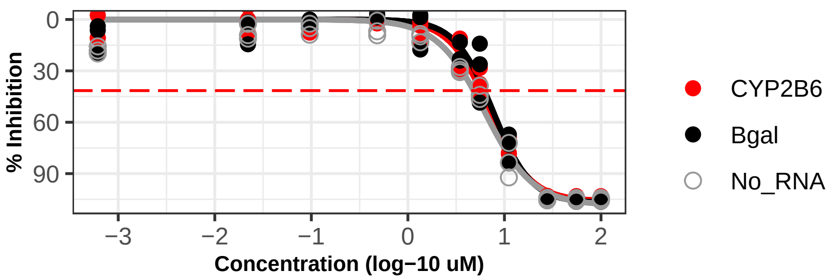

Enclomiphene hydrochloride: CYP2C8

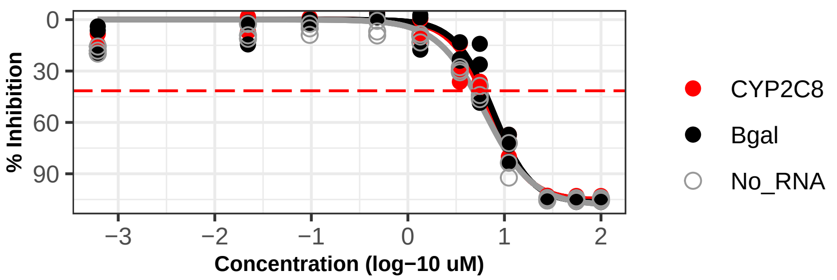

Enclomiphene hydrochloride: CYP2C9

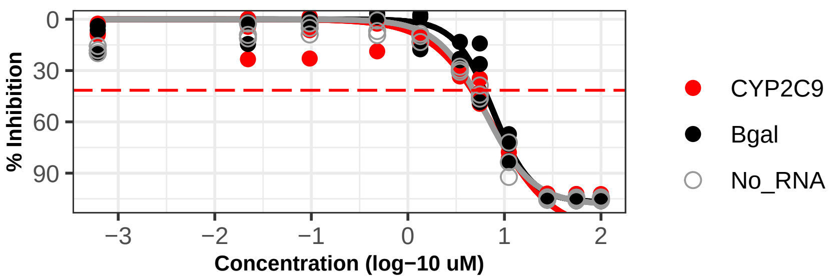

Enclomiphene hydrochloride: CYP2C19

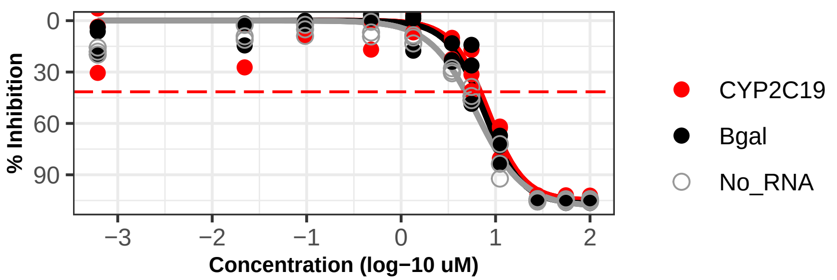

Enclomiphene hydrochloride: CYP2D6

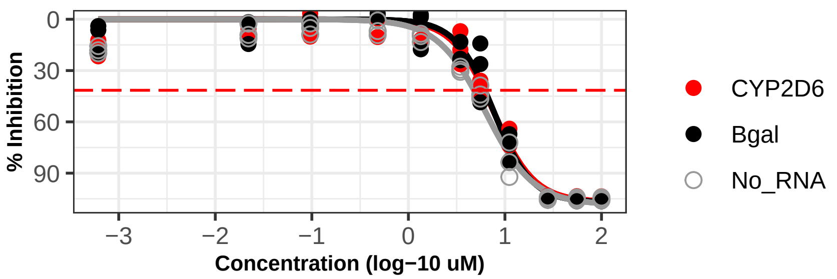

Enclomiphene hydrochloride: CYP2E1

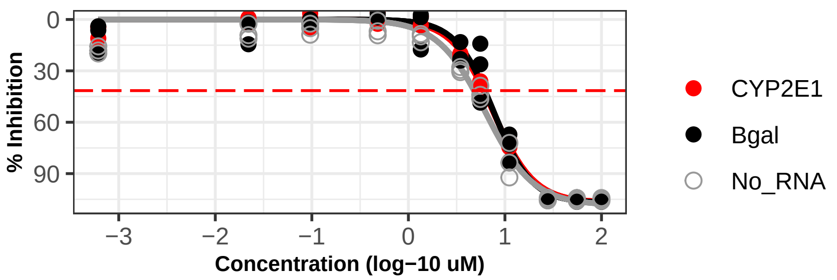

Enclomiphene hydrochloride: CYP2J2

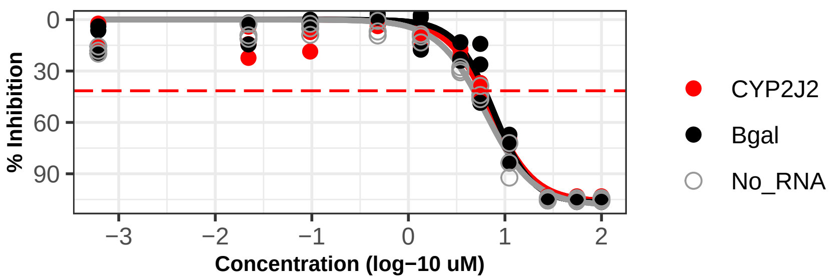

Enclomiphene hydrochloride: CYP3A4

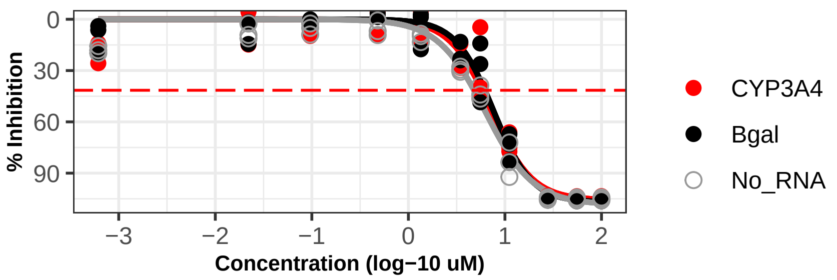

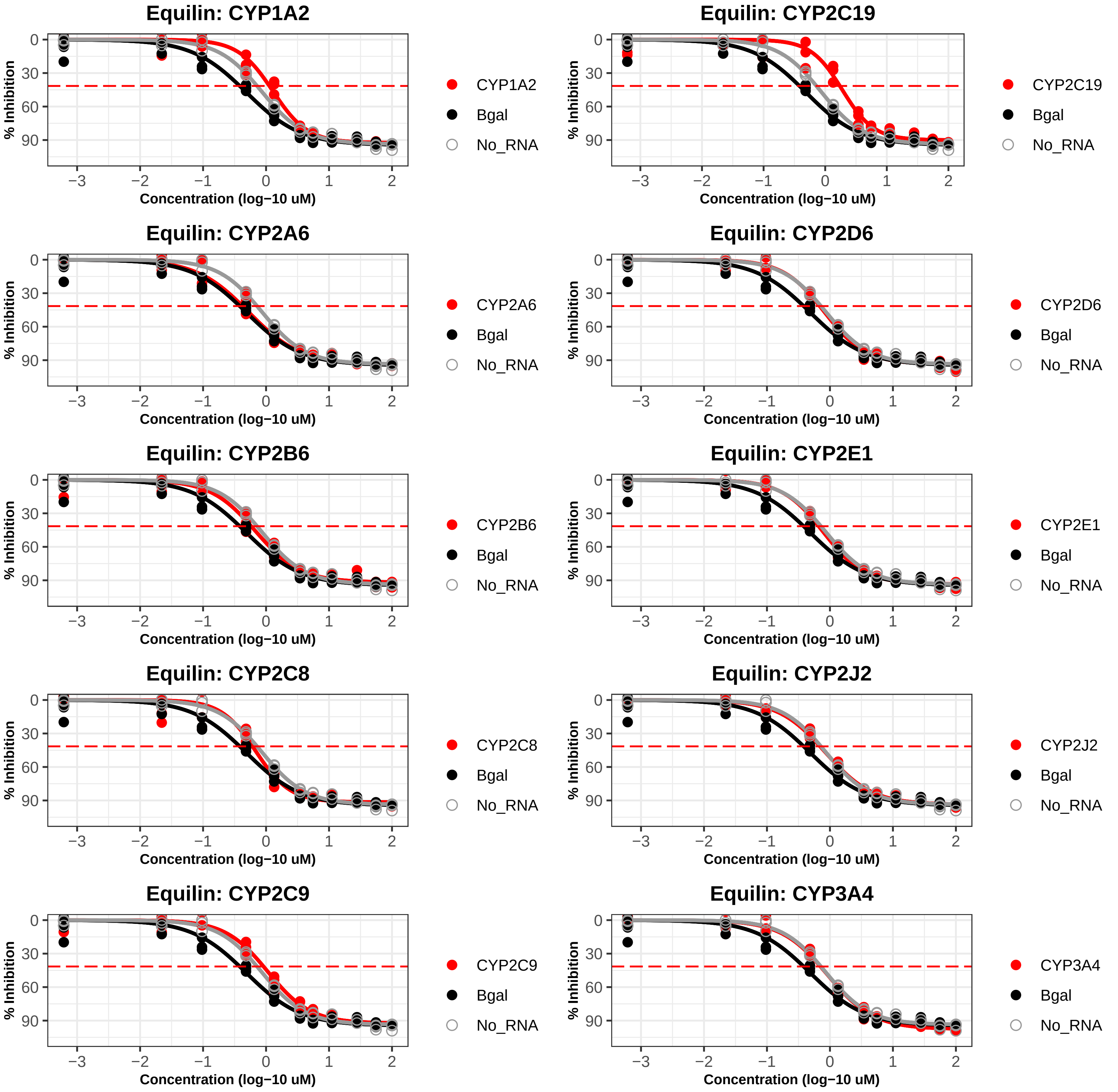
Equilin: CYP1A2
Equilin: CYP2C19
Equilin: CYP2A6
Equilin: CYP2D6
Equilin: CYP2B6
Equilin: CYP2E1
Equilin: CYP2C8
Equilin: CYP2J2
Equilin: CYP2C9
Equilin: CYP3A4
% Inhibition
Concentration (log−10 uM)
0
30
60
90
−3
−2
−1
0
1
2
CYP1A2
CYP2C19
CYP2A6
CYP2D6
CYP2B6
CYP2E1
CYP2C8
CYP2J2
CYP2C9
CYP3A4
Bgal
No_RNA

Esfenvalerate: CYP1A2

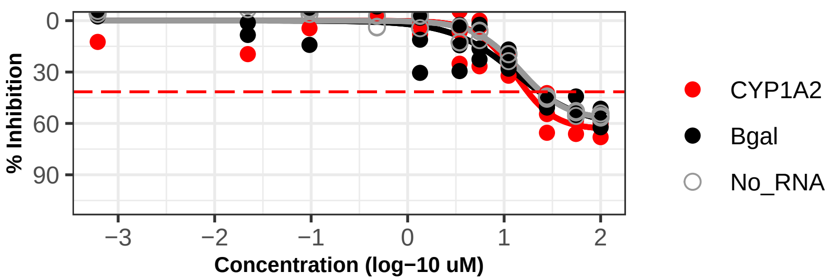

Esfenvalerate: CYP2C19

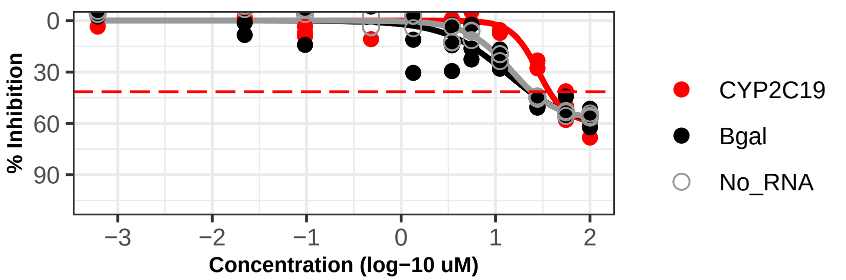

Esfenvalerate: CYP2A6

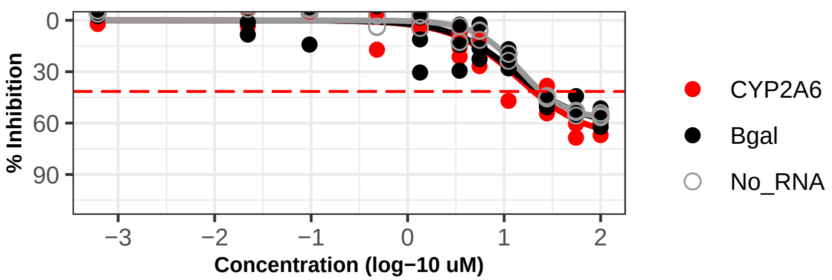

Esfenvalerate: CYP2D6

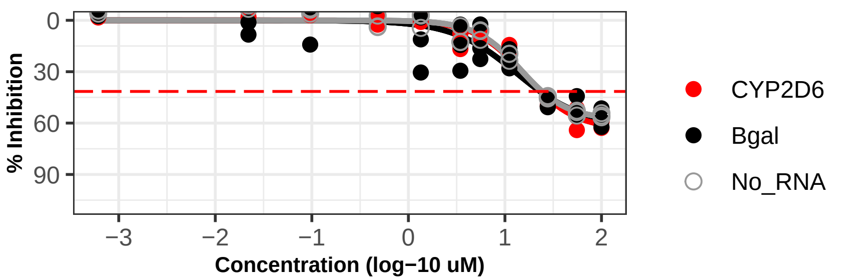

Esfenvalerate: CYP2B6

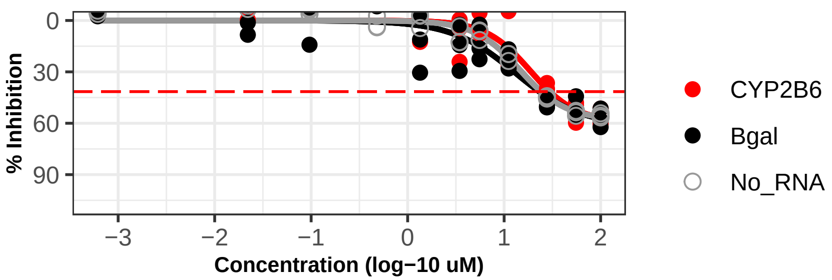

Esfenvalerate: CYP2E1

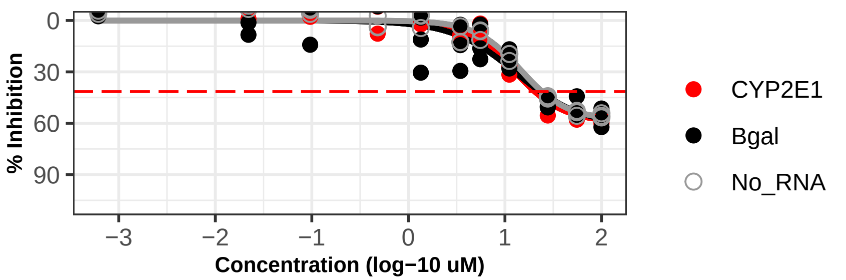

Esfenvalerate: CYP2C8

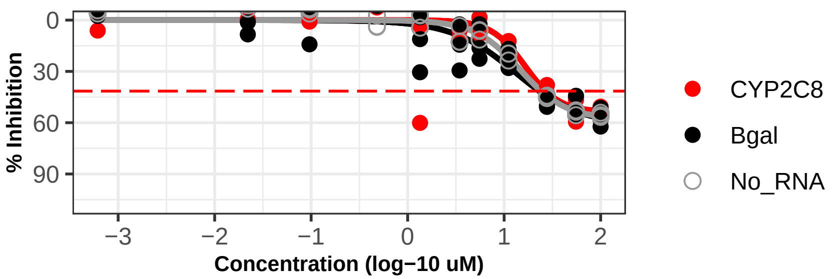

Esfenvalerate: CYP2J2

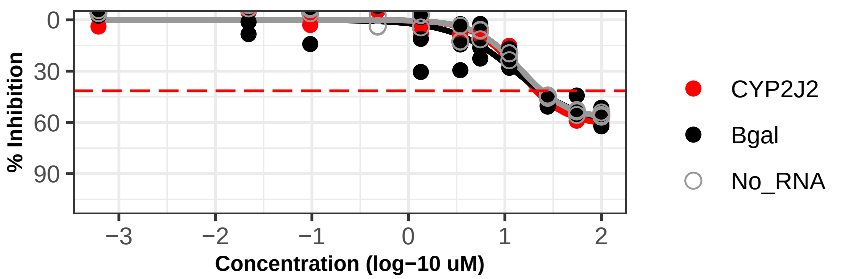

Esfenvalerate: CYP2C9

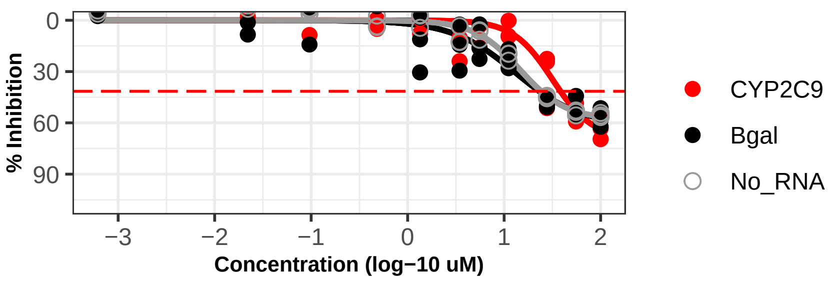

Esfenvalerate: CYP3A4

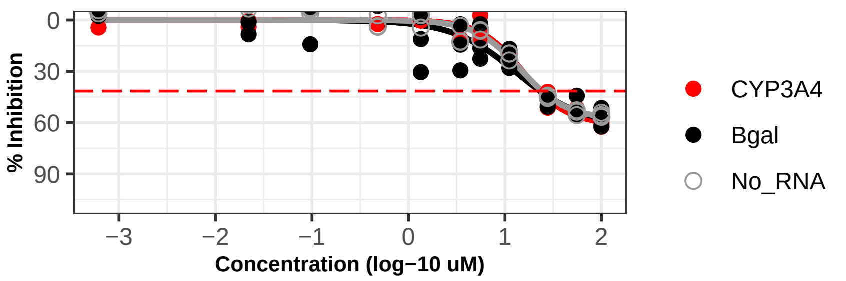

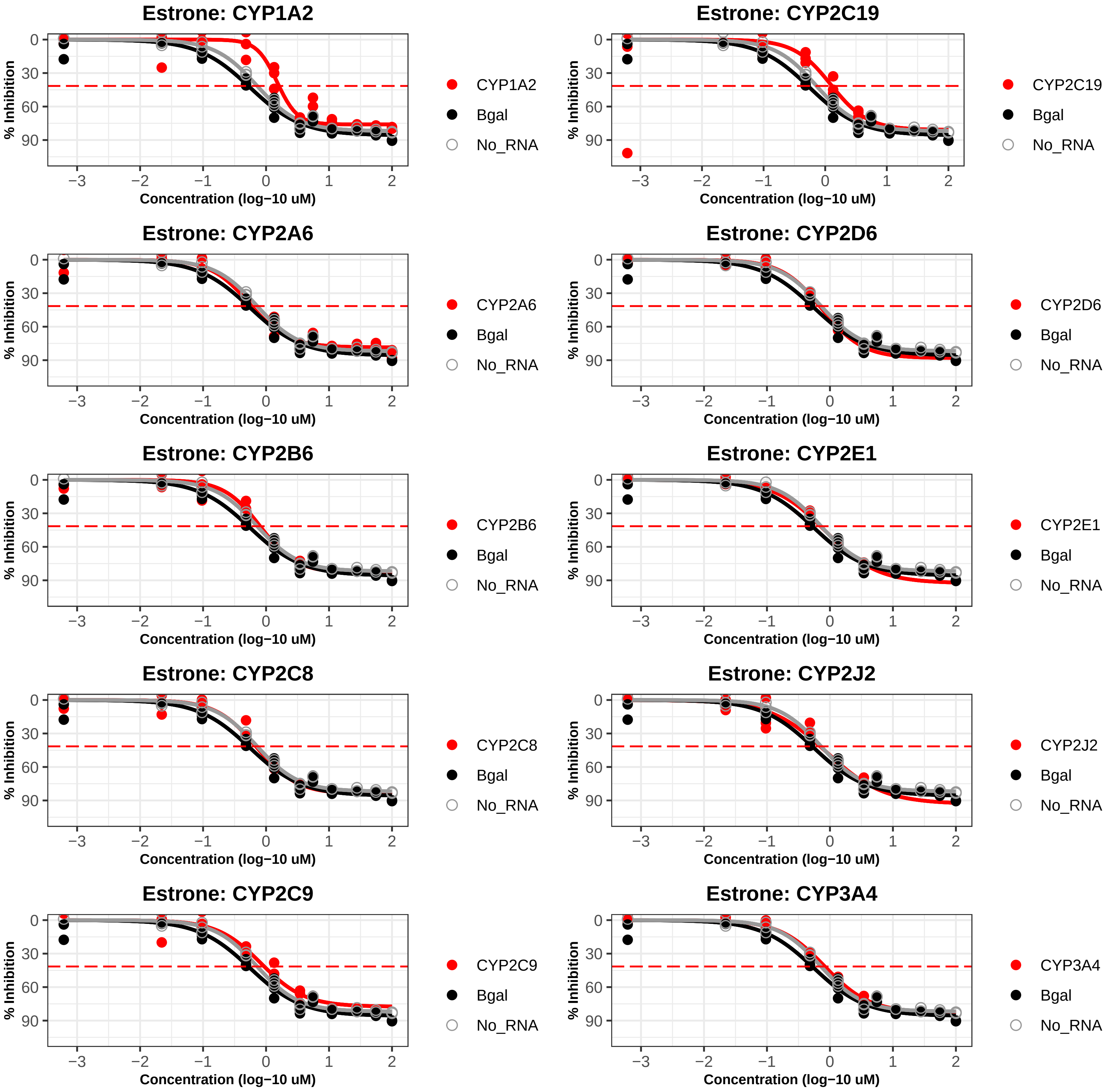
Estrone: CYP1A2
% Inhibition
Concentration (log−10 uM)
CYP1A2
Bgal
No_RNA
Estrone: CYP2C19
% Inhibition
Concentration (log−10 uM)
CYP2C19
Bgal
No_RNA
Estrone: CYP2A6
% Inhibition
Concentration (log−10 uM)
CYP2A6
Bgal
No_RNA
Estrone: CYP2D6
% Inhibition
Concentration (log−10 uM)
CYP2D6
Bgal
No_RNA
Estrone: CYP2B6
% Inhibition
Concentration (log−10 uM)
CYP2B6
Bgal
No_RNA
Estrone: CYP2E1
% Inhibition
Concentration (log−10 uM)
CYP2E1
Bgal
No_RNA
Estrone: CYP2C8
% Inhibition
Concentration (log−10 uM)
CYP2C8
Bgal
No_RNA
Estrone: CYP2J2
% Inhibition
Concentration (log−10 uM)
CYP2J2
Bgal
No_RNA
Estrone: CYP2C9
% Inhibition
Concentration (log−10 uM)
CYP2C9
Bgal
No_RNA
Estrone: CYP3A4
% Inhibition
Concentration (log−10 uM)
CYP3A4
Bgal
No_RNA

Ethoprop: CYP1A2

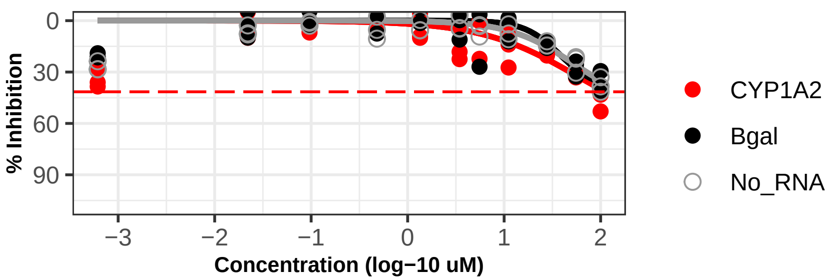

Ethoprop: CYP2C19

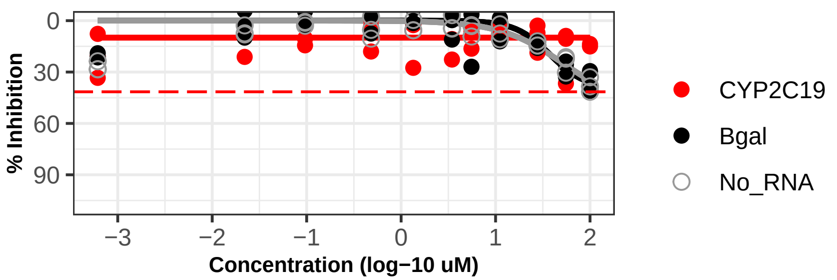

Ethoprop: CYP2A6

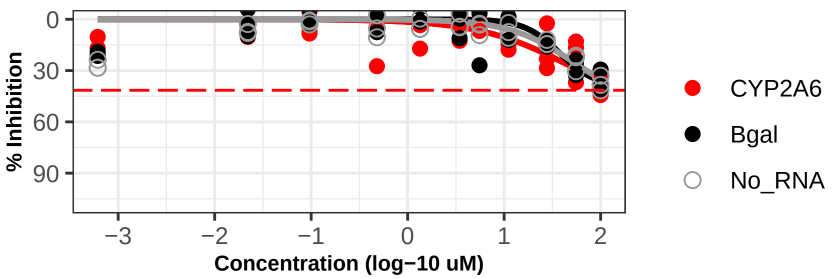

Ethoprop: CYP2D6

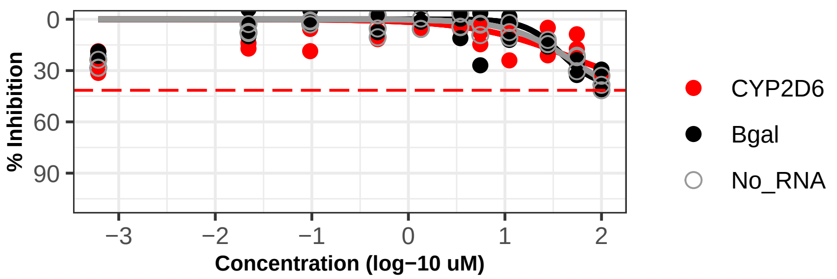

Ethoprop: CYP2B6

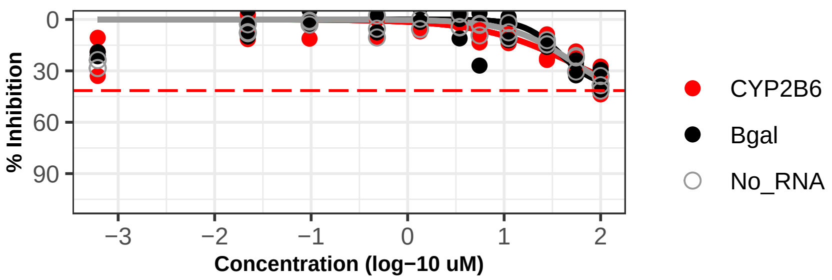

Ethoprop: CYP2E1

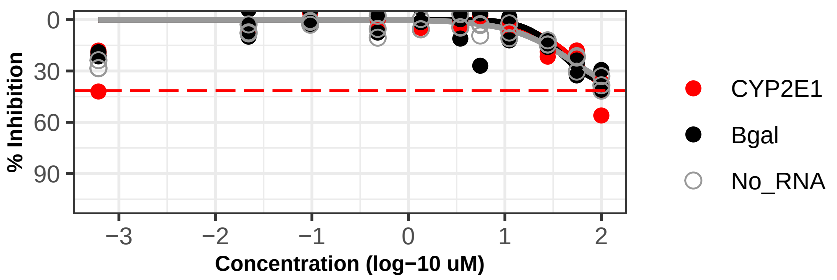

Ethoprop: CYP2C8

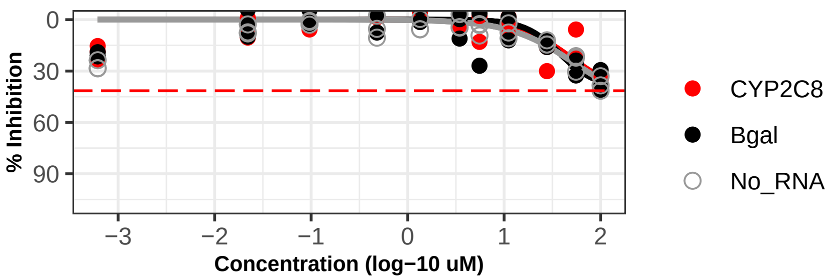

Ethoprop: CYP2J2

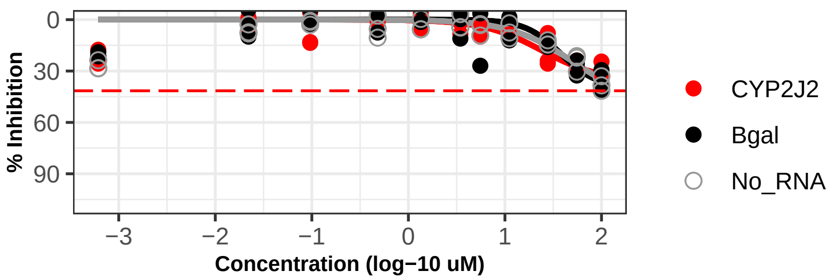

Ethoprop: CYP2C9

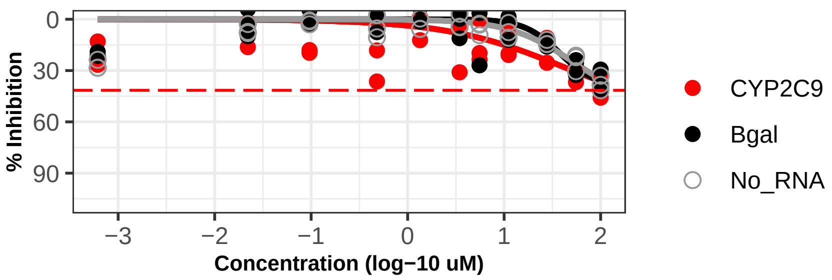

Ethoprop: CYP3A4

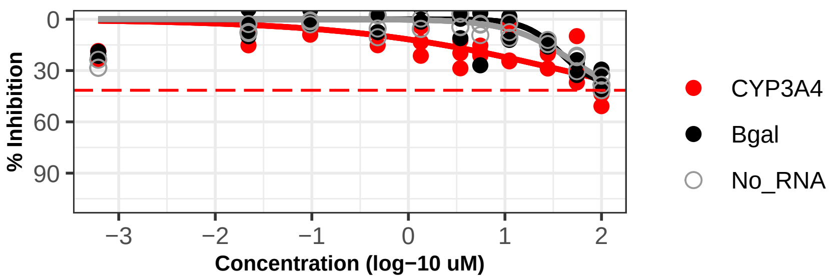

Ethylparaben: CYP1A2

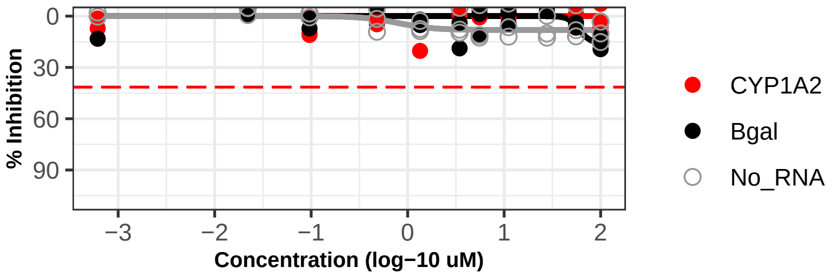

Ethylparaben: CYP2C19

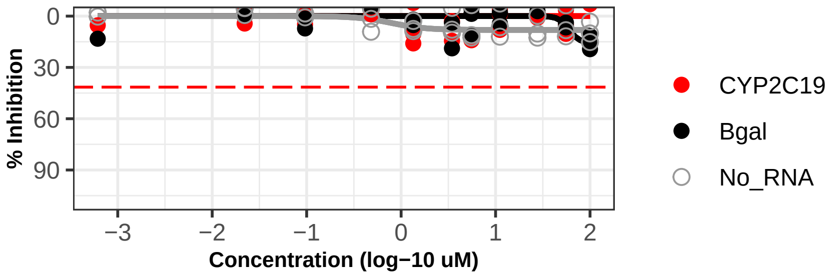

Ethylparaben: CYP2A6

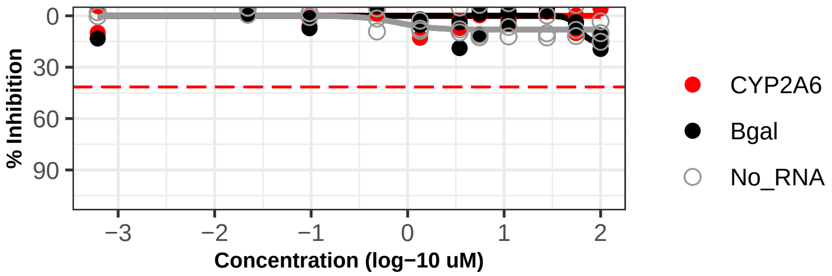

Ethylparaben: CYP2D6

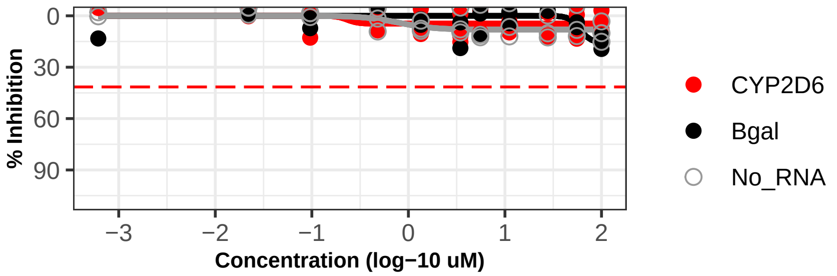

Ethylparaben: CYP2B6

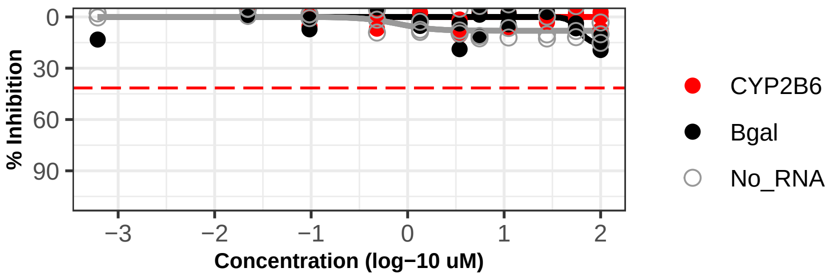

Ethylparaben: CYP2E1

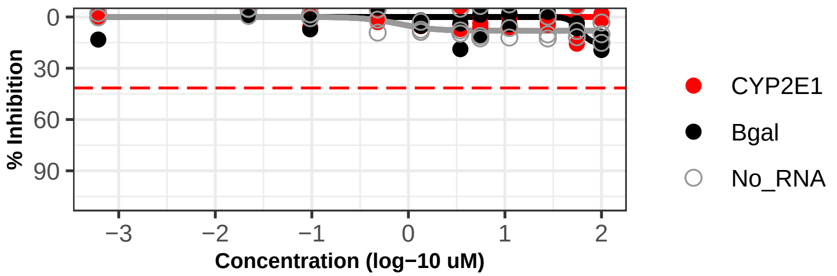

Ethylparaben: CYP2C8

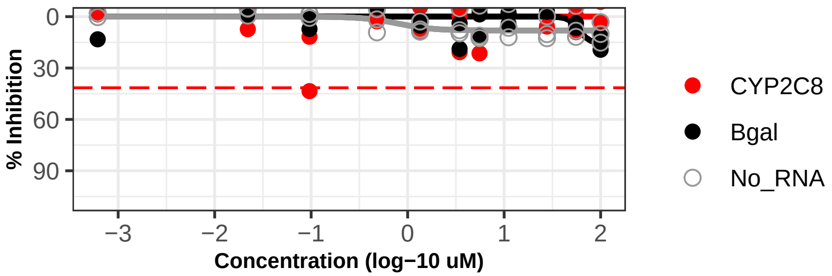

Ethylparaben: CYP2J2

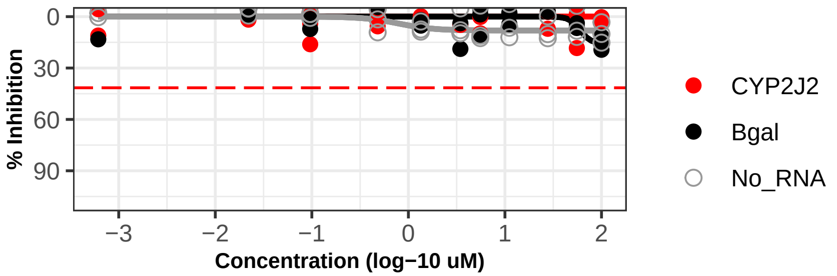

Ethylparaben: CYP2C9

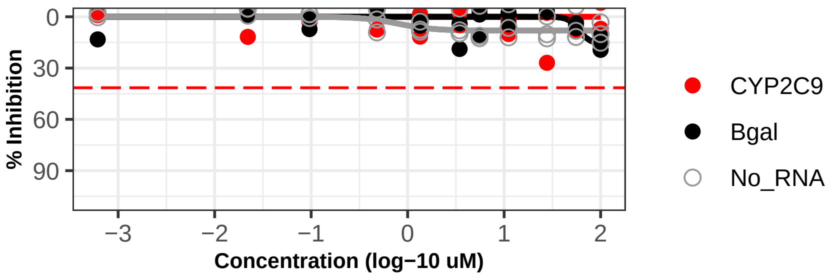

Ethylparaben: CYP3A4

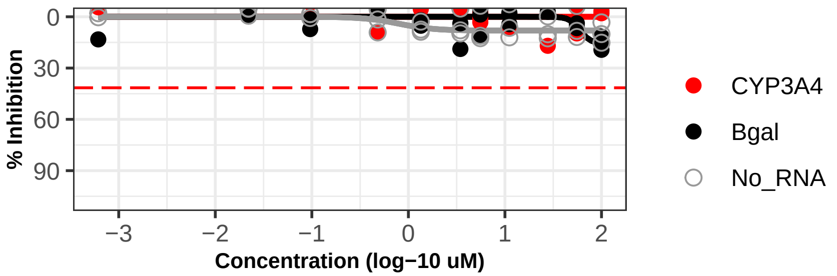

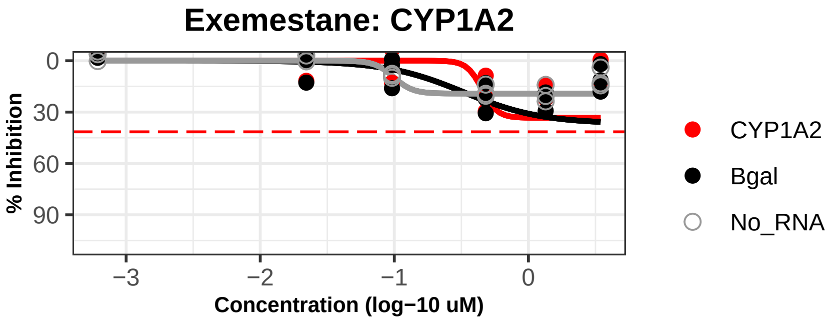
Exemestane: CYP1A2
% Inhibition
0
30
60
90
−3
−2
−1
0
Concentration (log−10 uM)
CYP1A2
Bgal
No_RNA

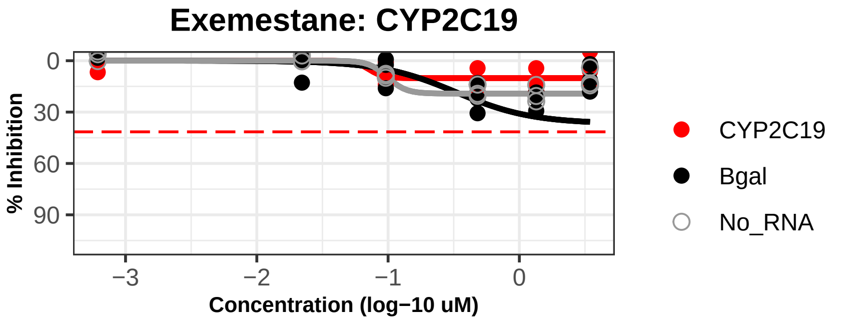
Exemestane: CYP2C19
% Inhibition
0
30
60
90
−3
−2
−1
0
Concentration (log−10 uM)
CYP2C19
Bgal
No_RNA

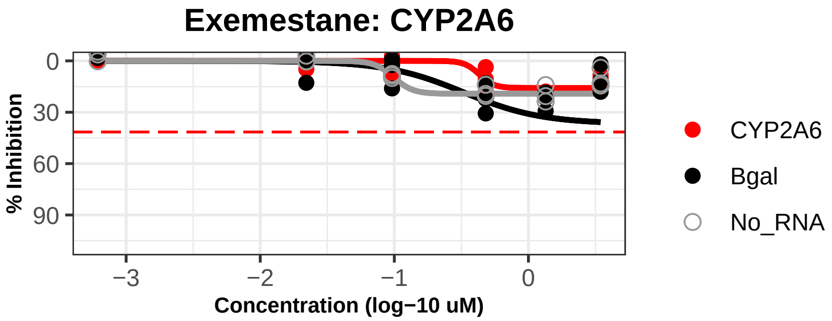
Exemestane: CYP2A6
% Inhibition
0
30
60
90
−3
−2
−1
0
Concentration (log−10 uM)
CYP2A6
Bgal
No_RNA

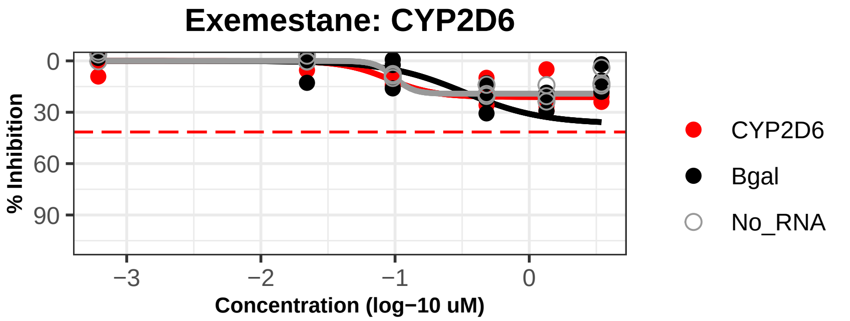
Exemestane: CYP2D6
% Inhibition
0
30
60
90
−3
−2
−1
0
Concentration (log−10 uM)
CYP2D6
Bgal
No_RNA

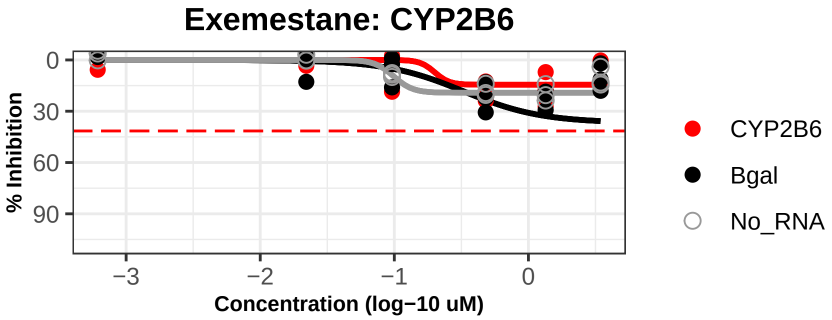
Exemestane: CYP2B6
% Inhibition
0
30
60
90
−3
−2
−1
0
Concentration (log−10 uM)
CYP2B6
Bgal
No_RNA

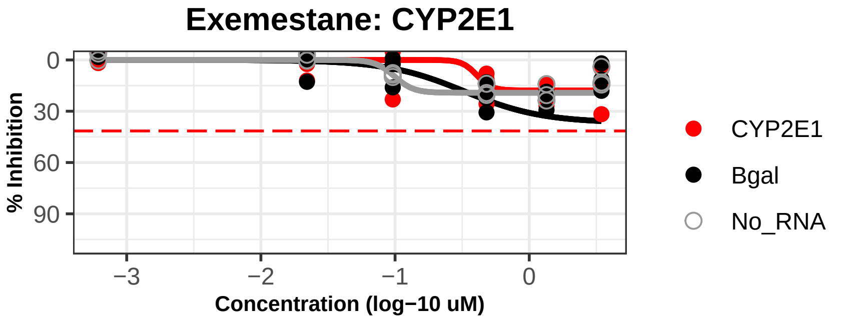
Exemestane: CYP2E1
% Inhibition
0
30
60
90
−3
−2
−1
0
Concentration (log−10 uM)
CYP2E1
Bgal
No_RNA

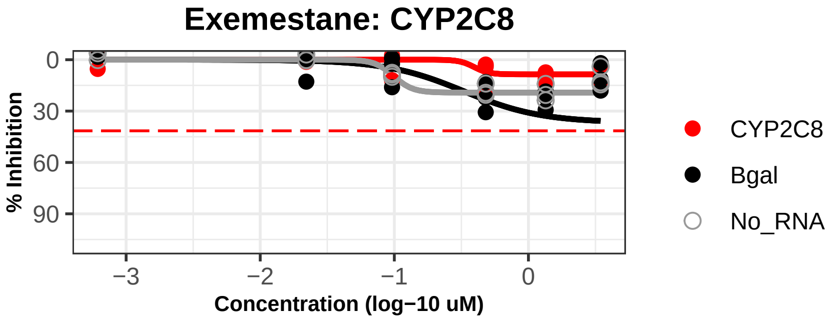
Exemestane: CYP2C8
% Inhibition
0
30
60
90
−3
−2
−1
0
Concentration (log−10 uM)
CYP2C8
Bgal
No_RNA

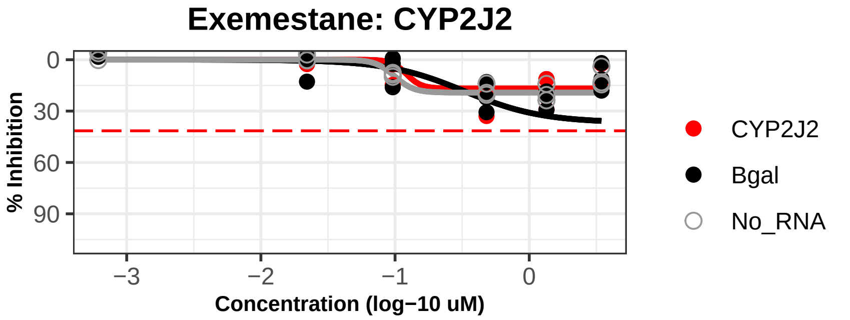
Exemestane: CYP2J2
% Inhibition
0
30
60
90
−3
−2
−1
0
Concentration (log−10 uM)
CYP2J2
Bgal
No_RNA

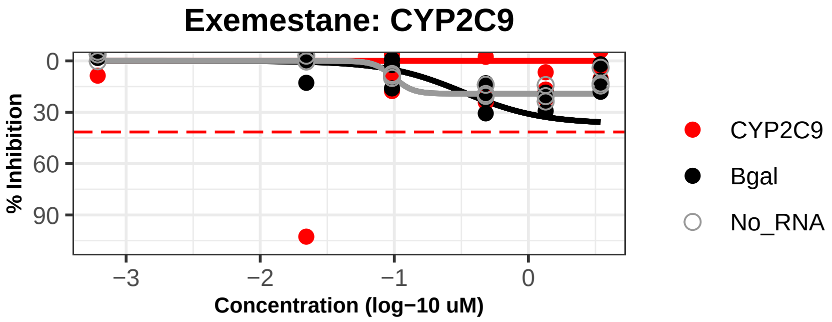
Exemestane: CYP2C9
% Inhibition
0
30
60
90
−3
−2
−1
0
Concentration (log−10 uM)
CYP2C9
Bgal
No_RNA

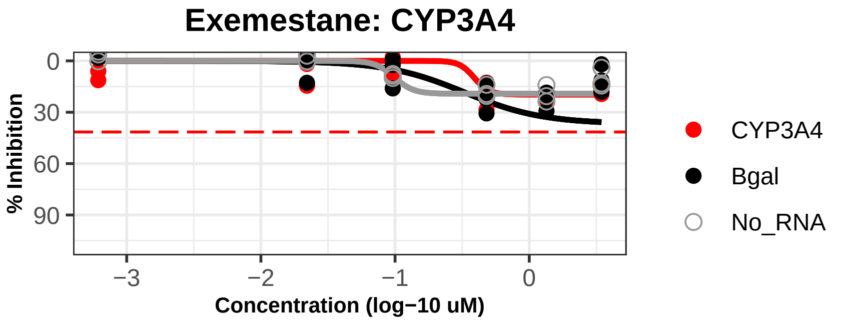
Exemestane: CYP3A4
% Inhibition
0
30
60
90
−3
−2
−1
0
Concentration (log−10 uM)
CYP3A4
Bgal
No_RNA

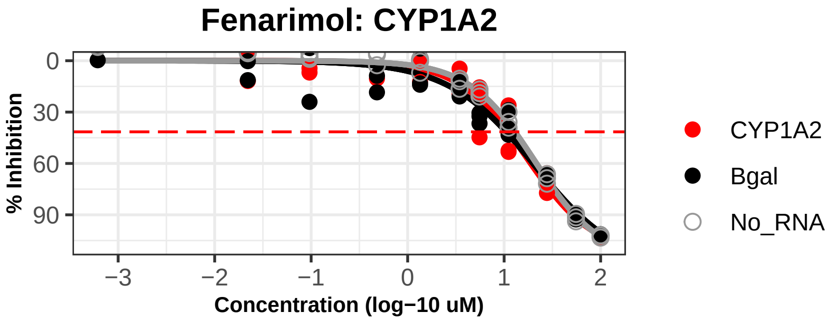
Fenarimol: CYP1A2
% Inhibition
0
30
60
90
−3
−2
−1
0
1
2
Concentration (log−10 uM)
CYP1A2
Bgal
No_RNA

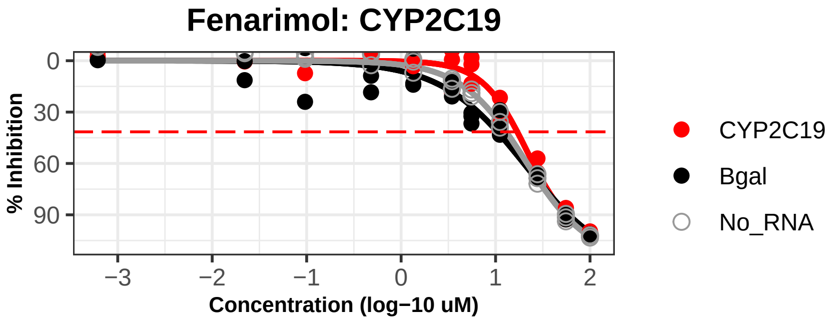
Fenarimol: CYP2C19
% Inhibition
0
30
60
90
−3
−2
−1
0
1
2
Concentration (log−10 uM)
CYP2C19
Bgal
No_RNA

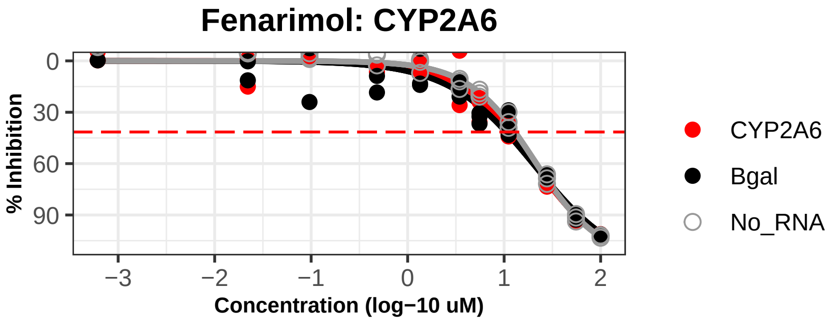
Fenarimol: CYP2A6
% Inhibition
0
30
60
90
−3
−2
−1
0
1
2
Concentration (log−10 uM)
CYP2A6
Bgal
No_RNA

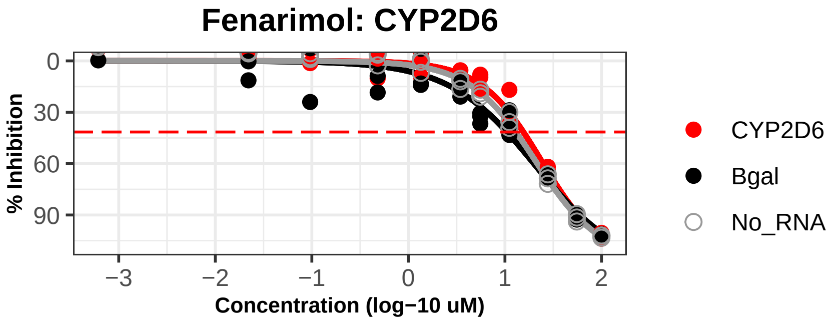
Fenarimol: CYP2D6
% Inhibition
0
30
60
90
−3
−2
−1
0
1
2
Concentration (log−10 uM)
CYP2D6
Bgal
No_RNA

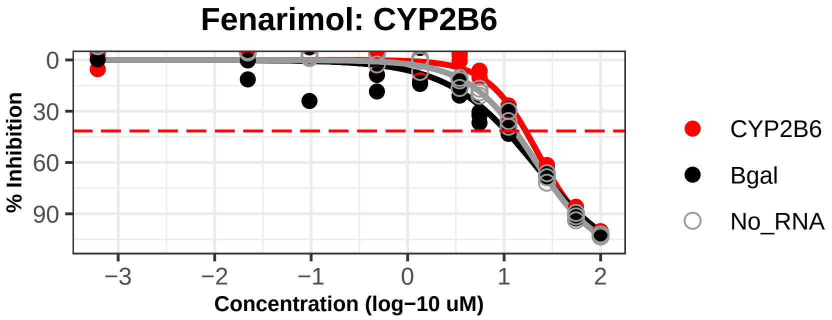
Fenarimol: CYP2B6
% Inhibition
0
30
60
90
−3
−2
−1
0
1
2
Concentration (log−10 uM)
CYP2B6
Bgal
No_RNA

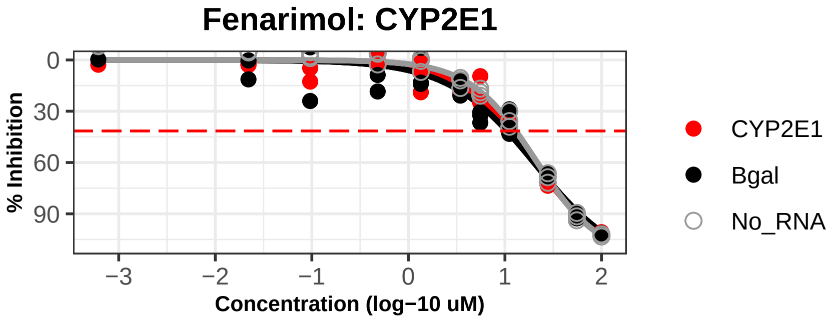
Fenarimol: CYP2E1
% Inhibition
0
30
60
90
−3
−2
−1
0
1
2
Concentration (log−10 uM)
CYP2E1
Bgal
No_RNA

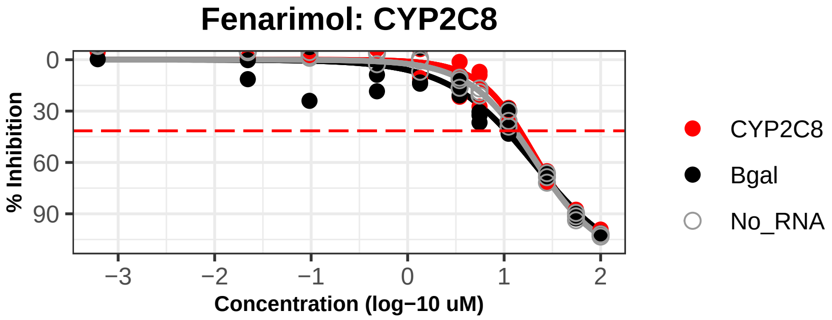
Fenarimol: CYP2C8
% Inhibition
0
30
60
90
−3
−2
−1
0
1
2
Concentration (log−10 uM)
CYP2C8
Bgal
No_RNA

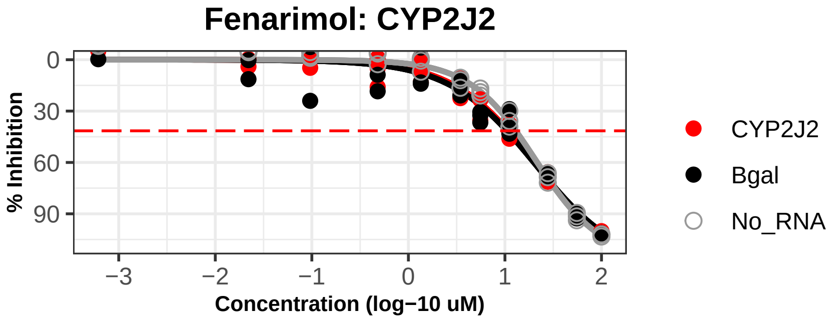
Fenarimol: CYP2J2
% Inhibition
0
30
60
90
−3
−2
−1
0
1
2
Concentration (log−10 uM)
CYP2J2
Bgal
No_RNA

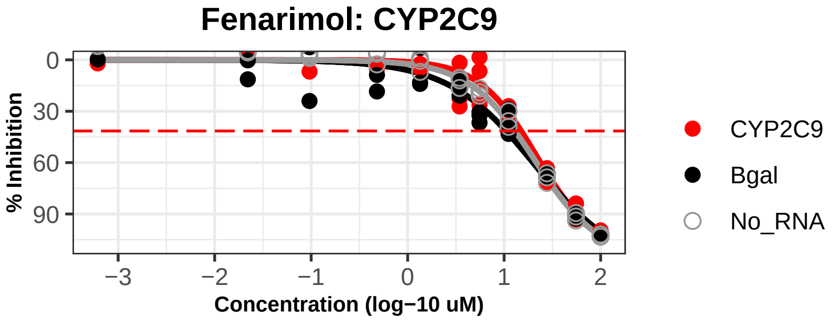
Fenarimol: CYP2C9
% Inhibition
0
30
60
90
−3
−2
−1
0
1
2
Concentration (log−10 uM)
CYP2C9
Bgal
No_RNA

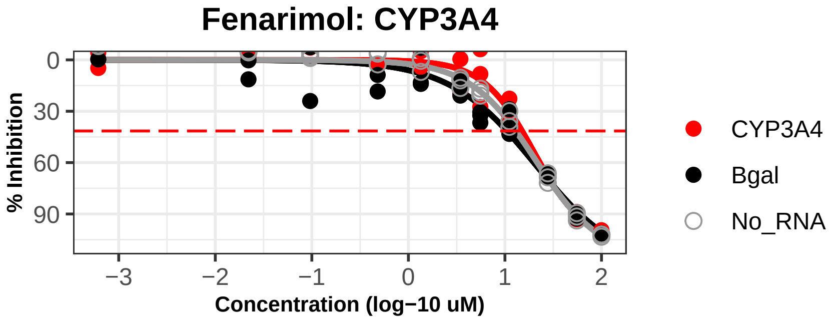
Fenarimol: CYP3A4
% Inhibition
0
30
60
90
−3
−2
−1
0
1
2
Concentration (log−10 uM)
CYP3A4
Bgal
No_RNA

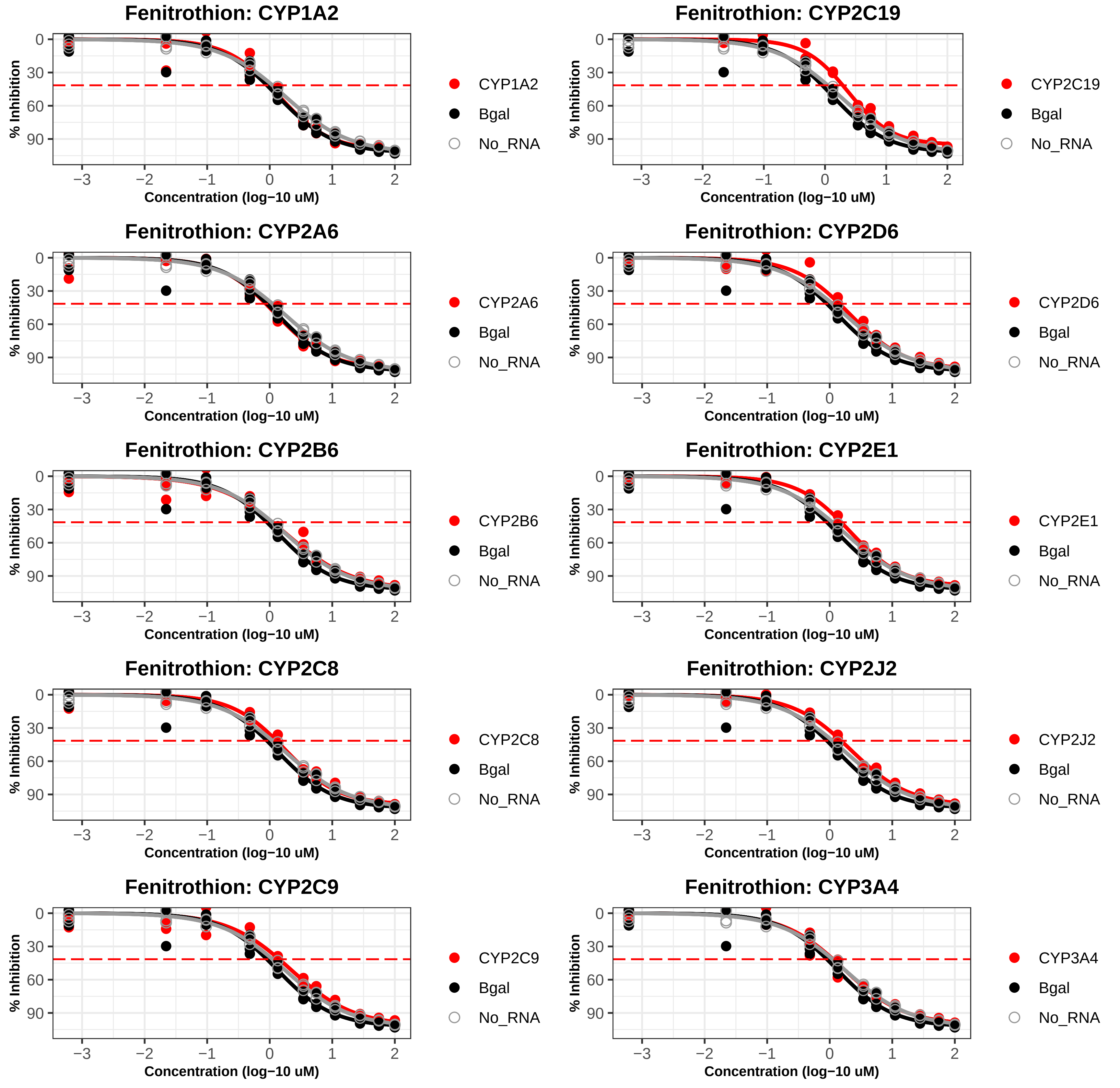
Fenitrothion: CYP1A2
% Inhibition
Concentration (log−10 uM)
CYP1A2
Bgal
No_RNA
Fenitrothion: CYP2C19
% Inhibition
Concentration (log−10 uM)
CYP2C19
Bgal
No_RNA
Fenitrothion: CYP2A6
% Inhibition
Concentration (log−10 uM)
CYP2A6
Bgal
No_RNA
Fenitrothion: CYP2D6
% Inhibition
Concentration (log−10 uM)
CYP2D6
Bgal
No_RNA
Fenitrothion: CYP2B6
% Inhibition
Concentration (log−10 uM)
CYP2B6
Bgal
No_RNA
Fenitrothion: CYP2E1
% Inhibition
Concentration (log−10 uM)
CYP2E1
Bgal
No_RNA
Fenitrothion: CYP2C8
% Inhibition
Concentration (log−10 uM)
CYP2C8
Bgal
No_RNA
Fenitrothion: CYP2J2
% Inhibition
Concentration (log−10 uM)
CYP2J2
Bgal
No_RNA
Fenitrothion: CYP2C9
% Inhibition
Concentration (log−10 uM)
CYP2C9
Bgal
No_RNA
Fenitrothion: CYP3A4
% Inhibition
Concentration (log−10 uM)
CYP3A4
Bgal
No_RNA

Fenthion: CYP1A2

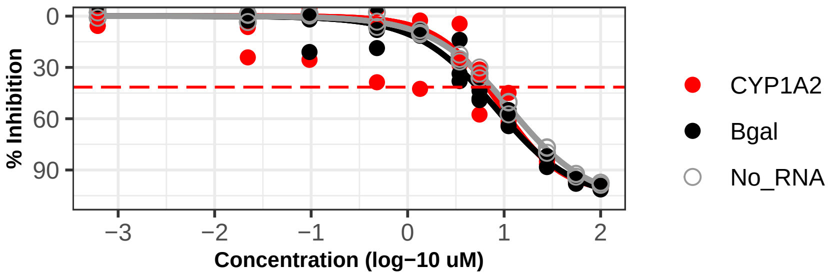

Fenthion: CYP2C19

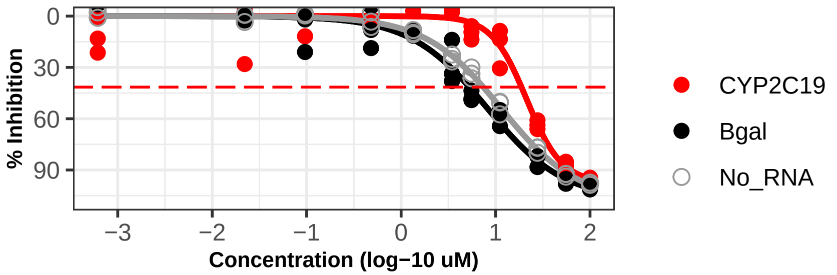

Fenthion: CYP2A6

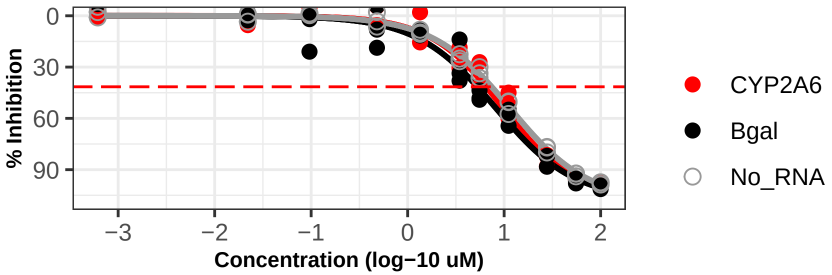

Fenthion: CYP2D6

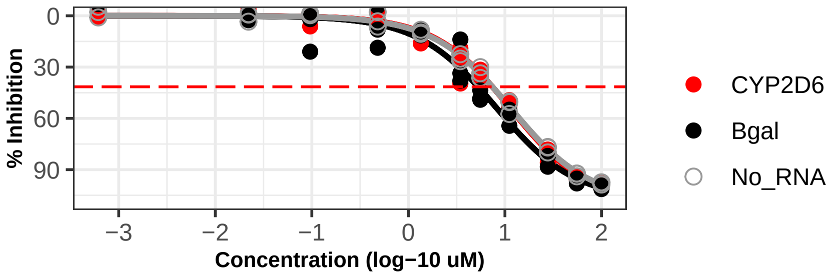

Fenthion: CYP2B6

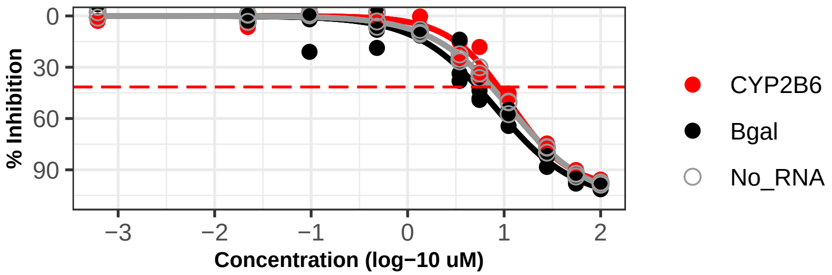

Fenthion: CYP2E1

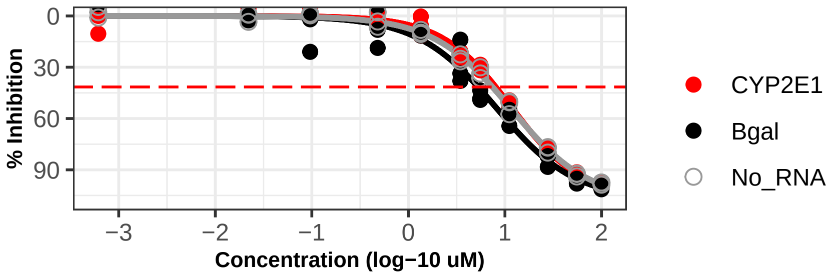

Fenthion: CYP2C8

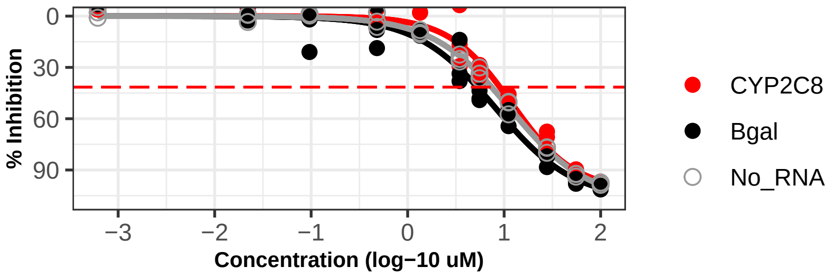

Fenthion: CYP2J2

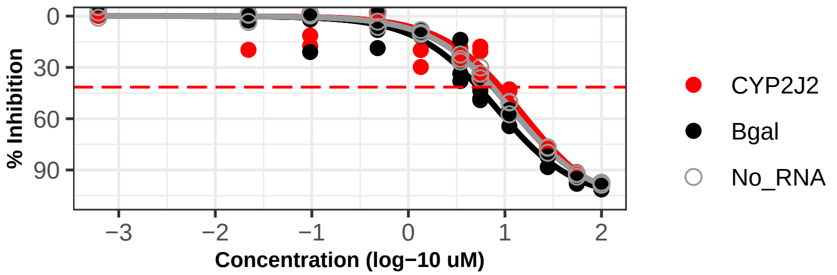

Fenthion: CYP2C9

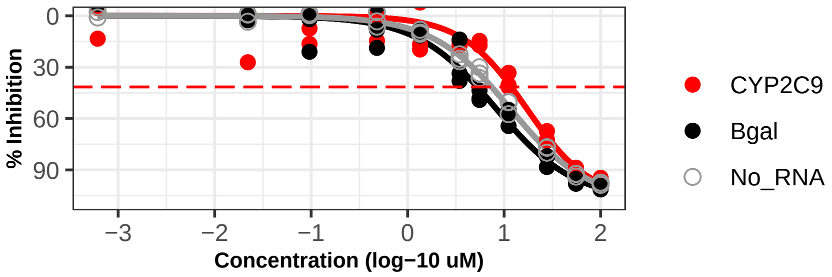

Fenthion: CYP3A4

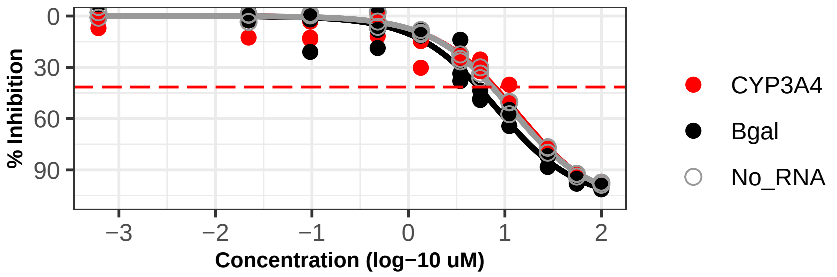

Fenvalerate: CYP1A2

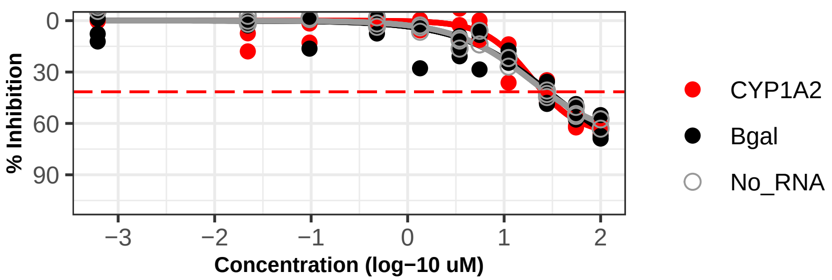

Fenvalerate: CYP2C19

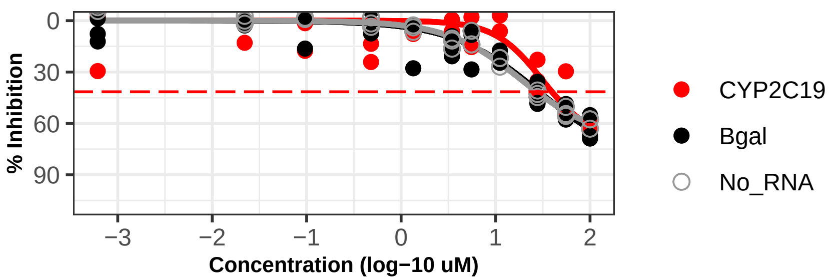

Fenvalerate: CYP2A6

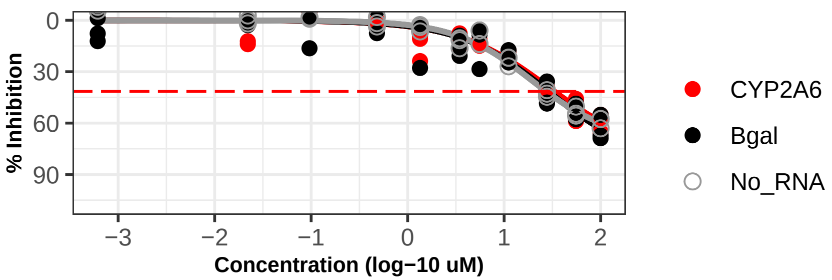

Fenvalerate: CYP2D6

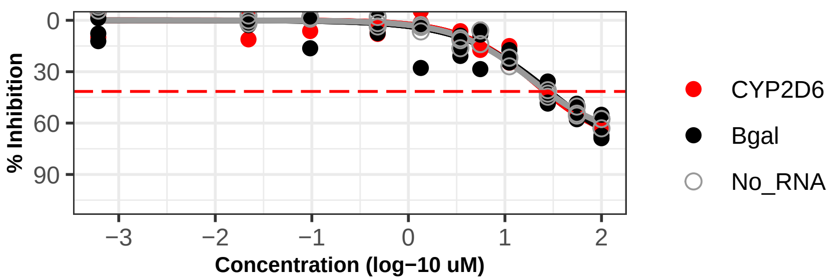

Fenvalerate: CYP2B6

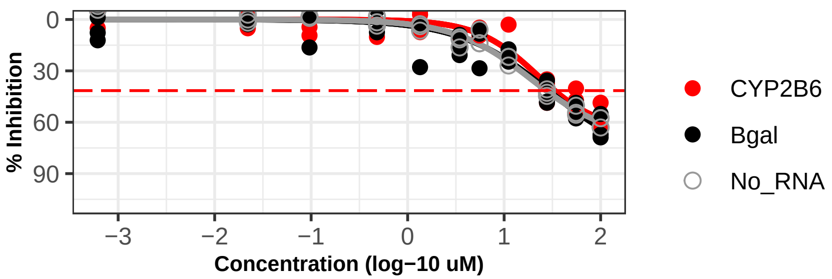

Fenvalerate: CYP2E1

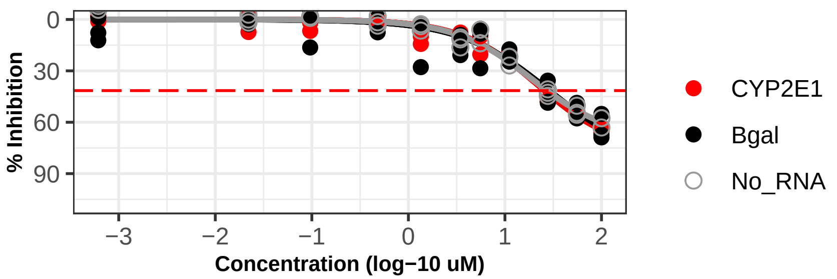

Fenvalerate: CYP2C8

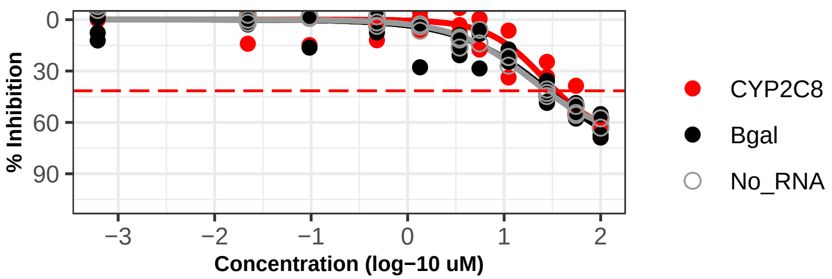

Fenvalerate: CYP2J2

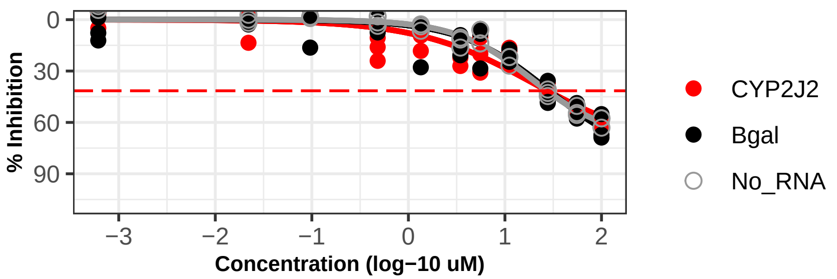

Fenvalerate: CYP2C9

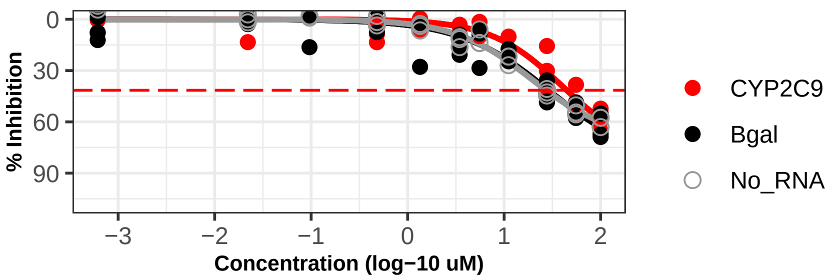

Fenvalerate: CYP3A4

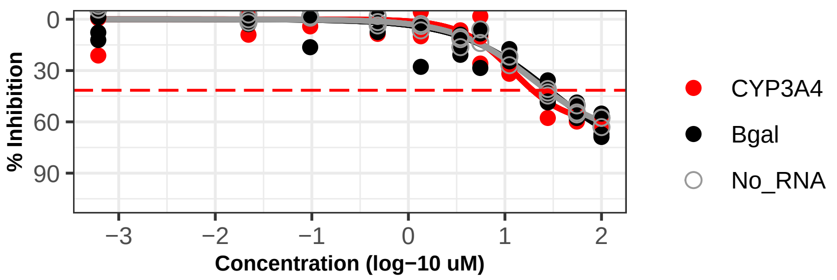

Finasteride: CYP1A2

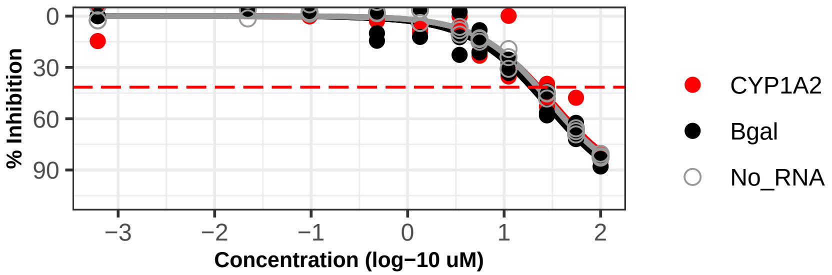

Finasteride: CYP2C19

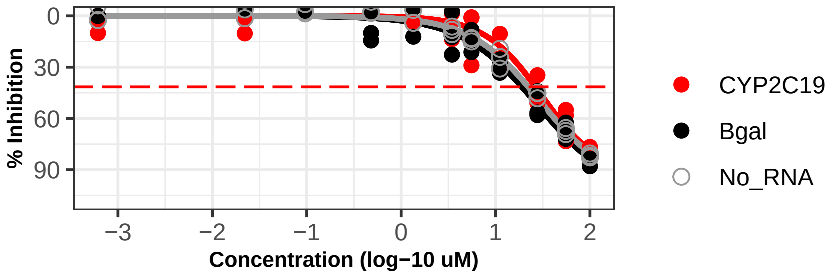

Finasteride: CYP2A6

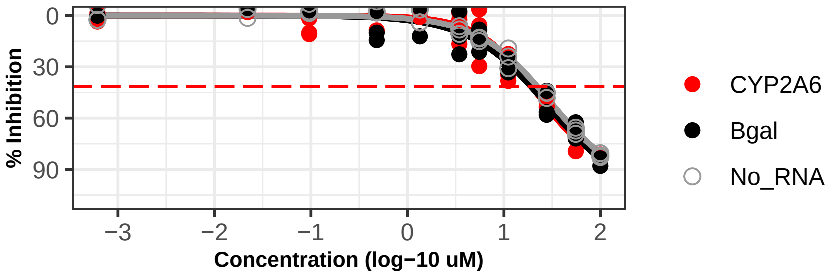

Finasteride: CYP2D6

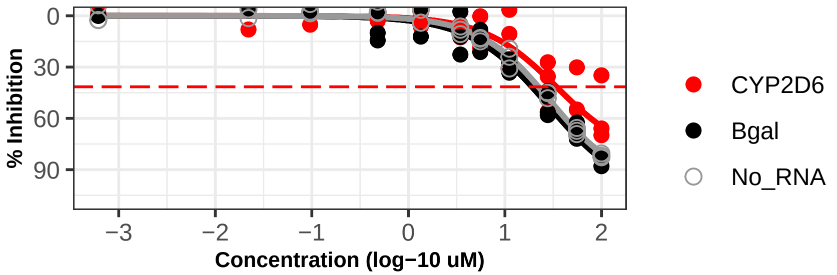

Finasteride: CYP2B6

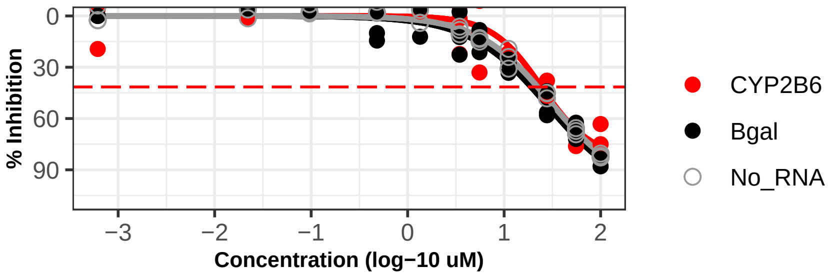

Finasteride: CYP2E1

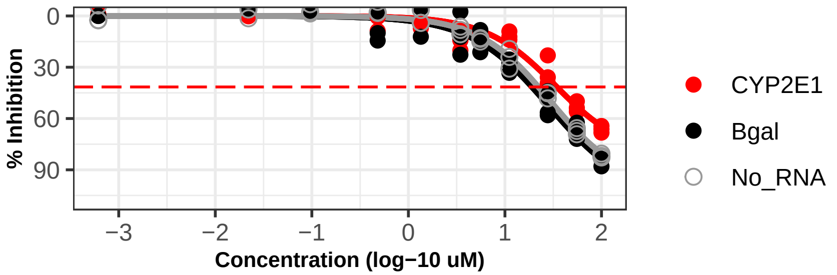

Finasteride: CYP2C8

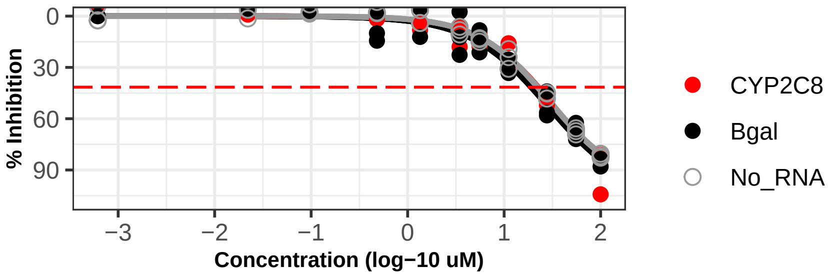

Finasteride: CYP2J2

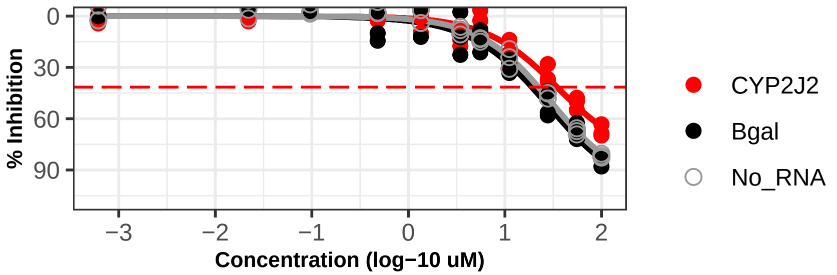

Finasteride: CYP2C9

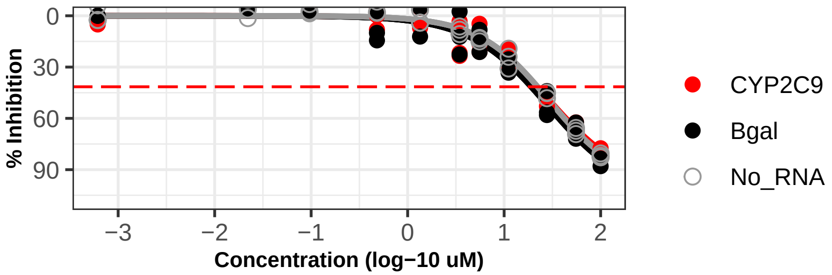

Finasteride: CYP3A4

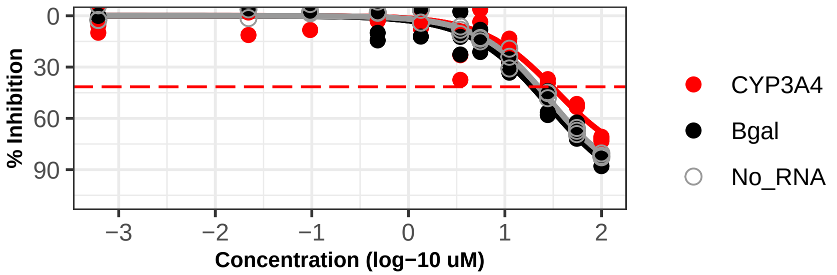

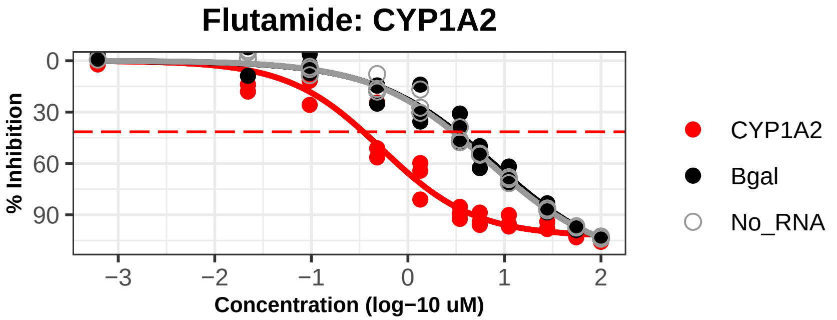
Flutamide: CYP1A2
% Inhibition
Concentration (log−10 uM)
CYP1A2
Bgal
No_RNA

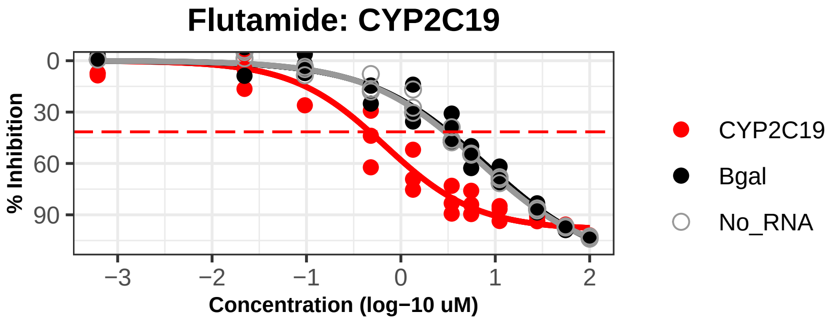
Flutamide: CYP2C19
% Inhibition
Concentration (log−10 uM)
CYP2C19
Bgal
No_RNA

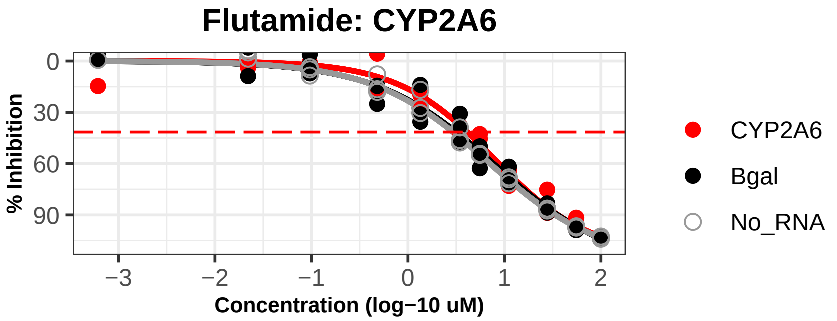
Flutamide: CYP2A6
% Inhibition
Concentration (log−10 uM)
CYP2A6
Bgal
No_RNA

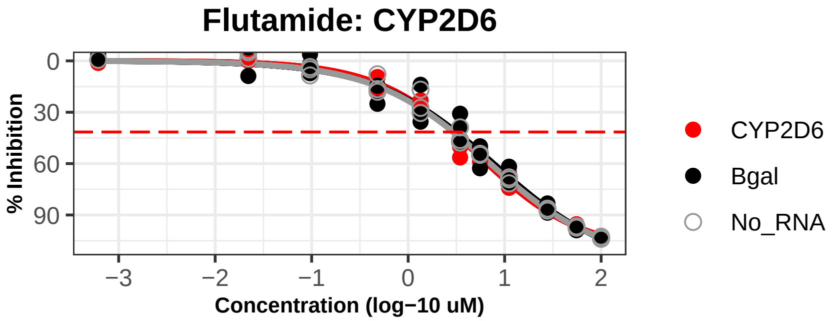
Flutamide: CYP2D6
% Inhibition
Concentration (log−10 uM)
CYP2D6
Bgal
No_RNA

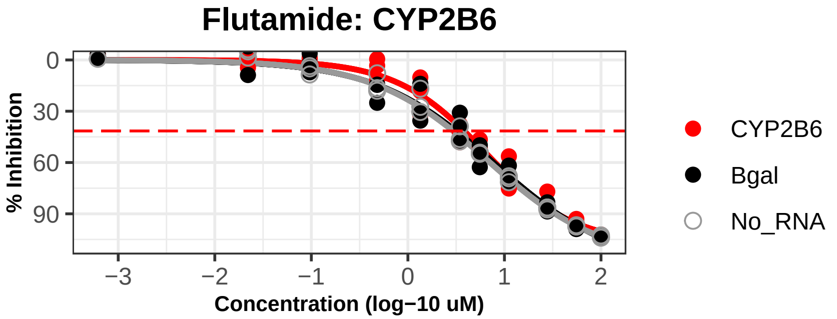
Flutamide: CYP2B6
% Inhibition
Concentration (log−10 uM)
CYP2B6
Bgal
No_RNA

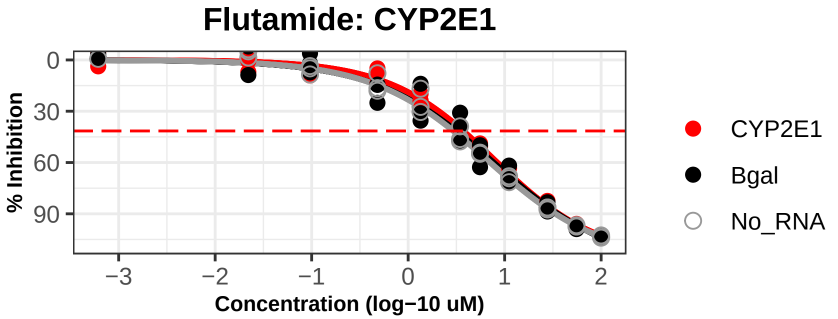
Flutamide: CYP2E1
% Inhibition
Concentration (log−10 uM)
CYP2E1
Bgal
No_RNA

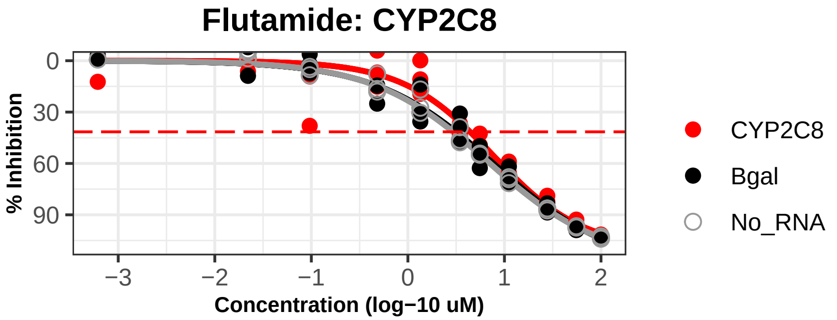
Flutamide: CYP2C8
% Inhibition
Concentration (log−10 uM)
CYP2C8
Bgal
No_RNA

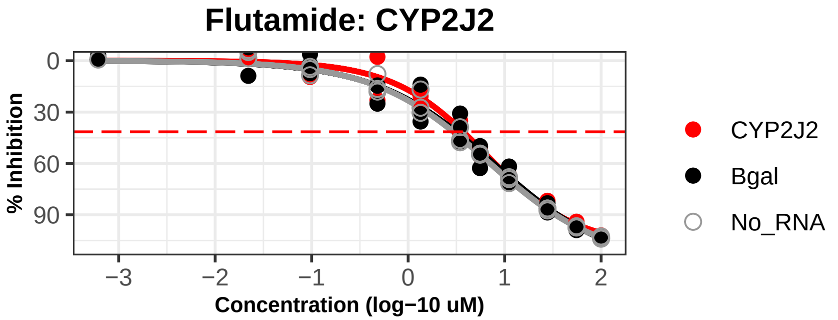
Flutamide: CYP2J2
% Inhibition
Concentration (log−10 uM)
CYP2J2
Bgal
No_RNA

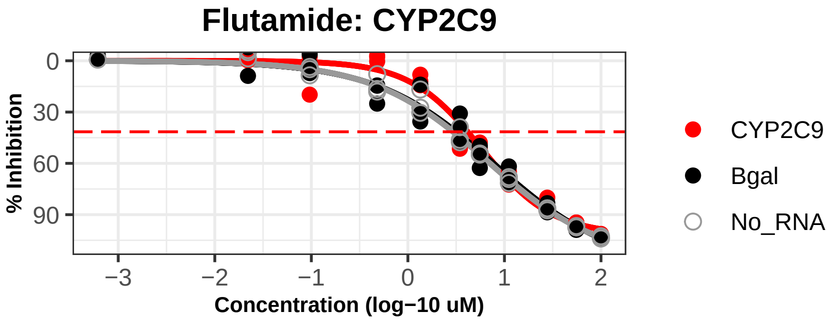
Flutamide: CYP2C9
% Inhibition
Concentration (log−10 uM)
CYP2C9
Bgal
No_RNA

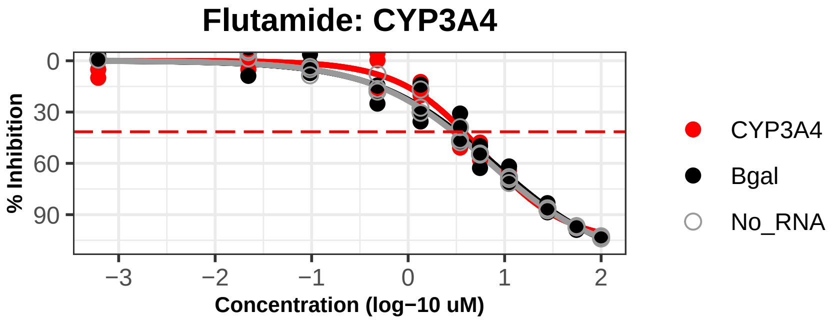
Flutamide: CYP3A4
% Inhibition
Concentration (log−10 uM)
CYP3A4
Bgal
No_RNA

Flutolanil: CYP1A2

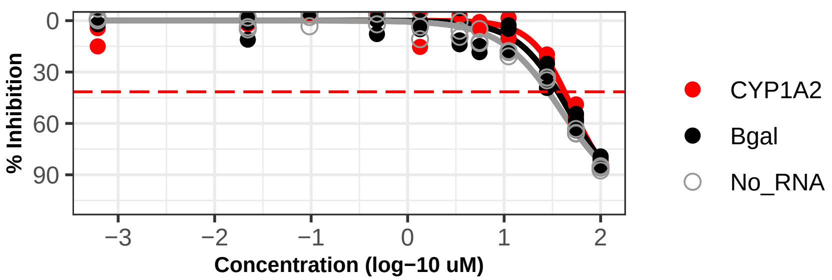

Flutolanil: CYP2C19

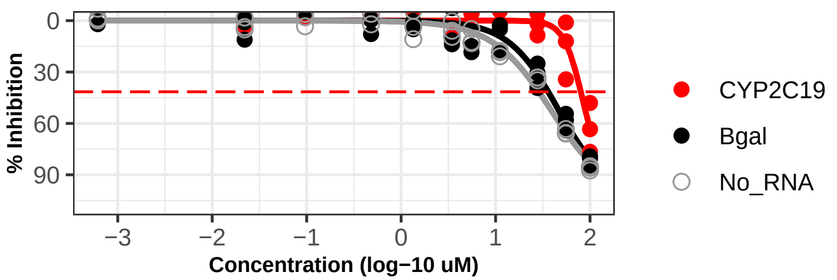

Flutolanil: CYP2A6

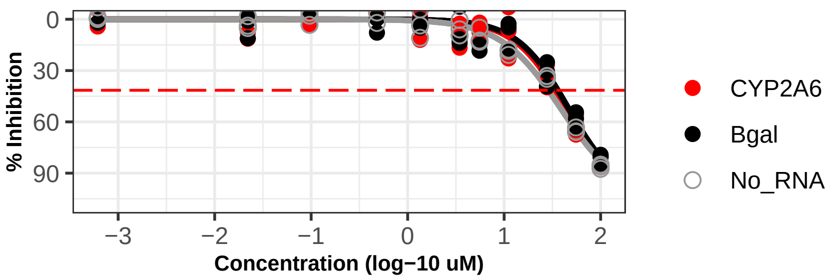

Flutolanil: CYP2D6

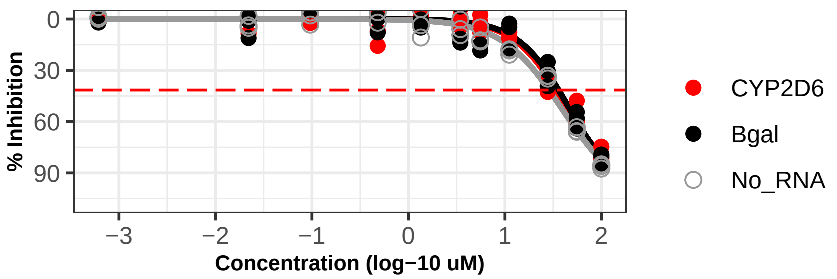

Flutolanil: CYP2B6

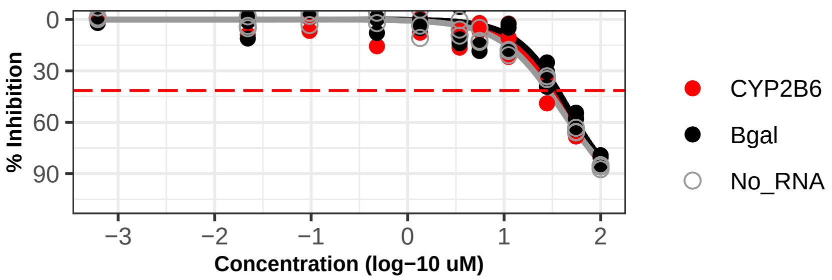

Flutolanil: CYP2E1

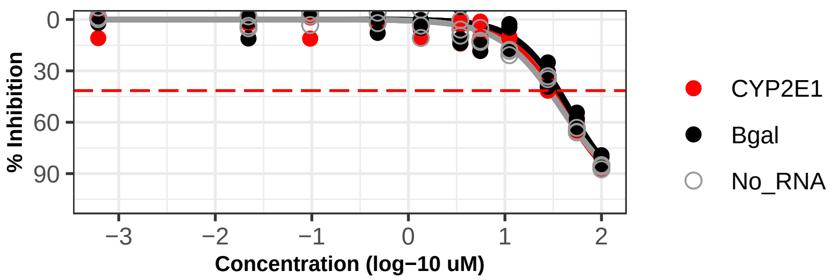

Flutolanil: CYP2C8

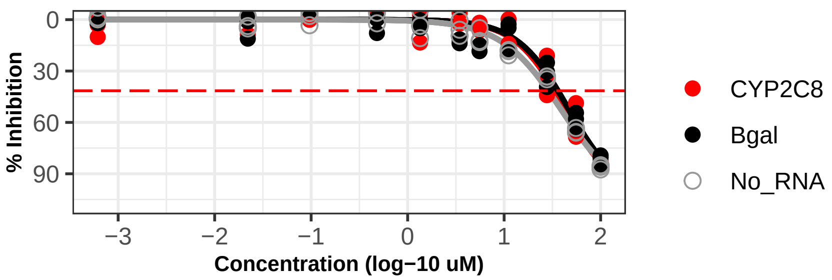

Flutolanil: CYP2J2

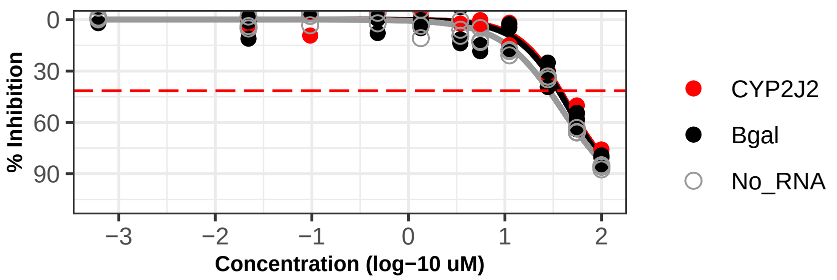

Flutolanil: CYP2C9

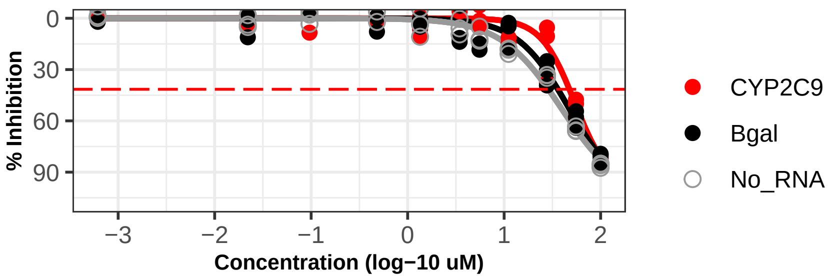

Flutolanil: CYP3A4

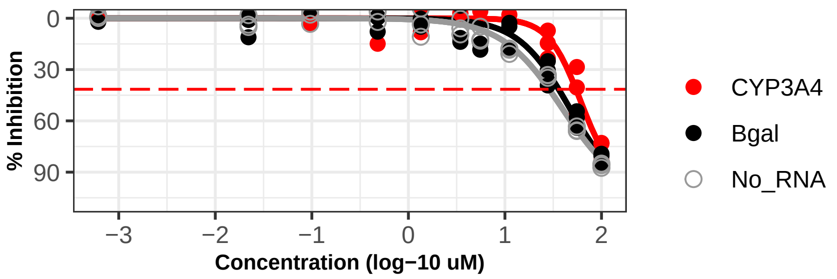

Folpet: CYP1A2

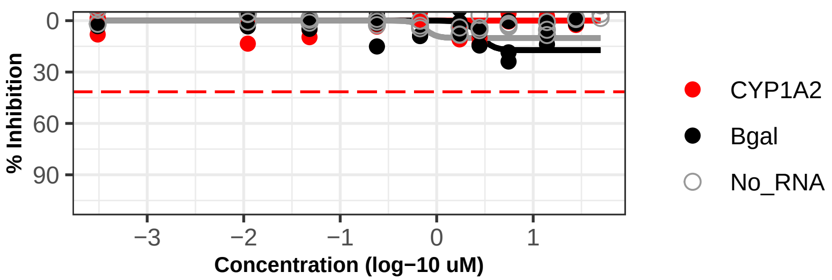

Folpet: CYP2C19

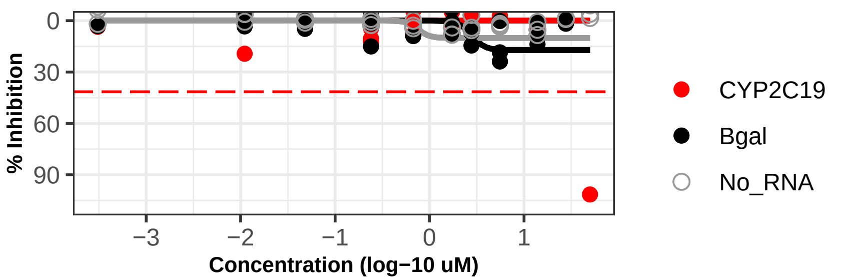

Folpet: CYP2A6

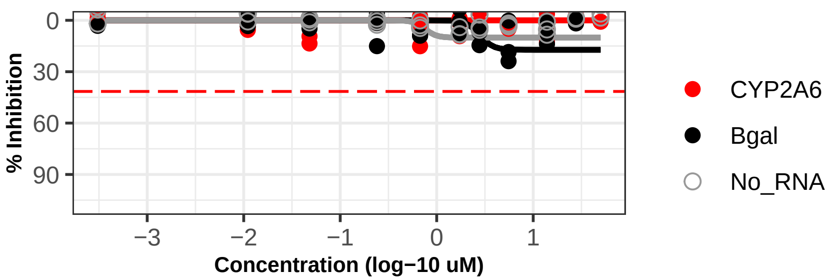

Folpet: CYP2D6

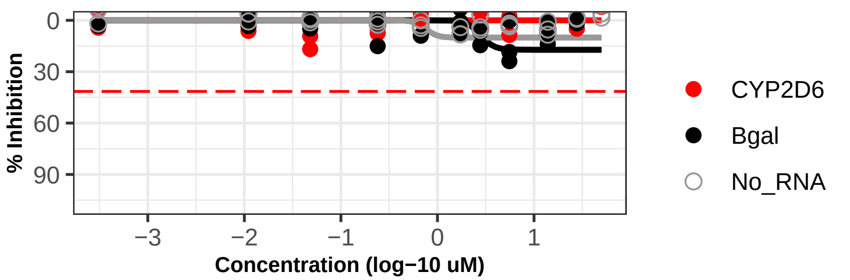

Folpet: CYP2B6

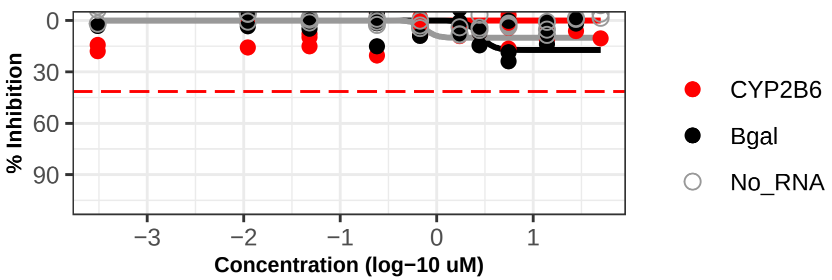

Folpet: CYP2E1

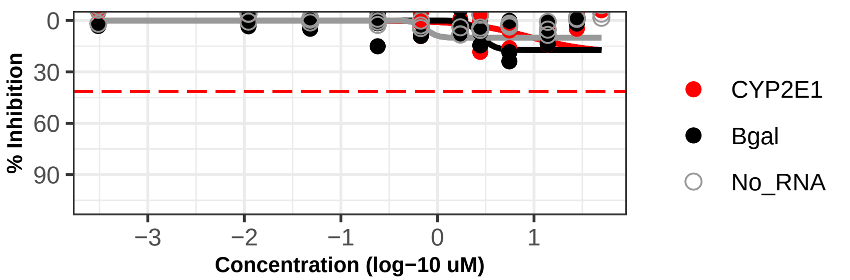

Folpet: CYP2C8

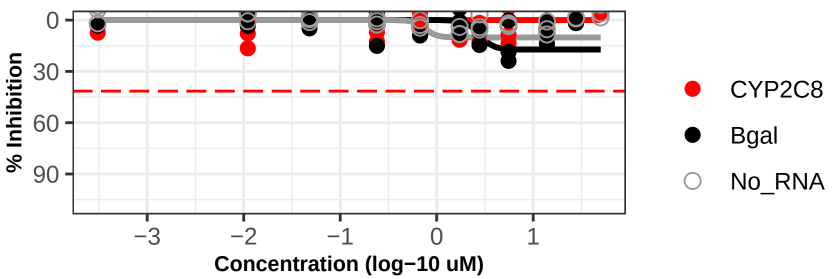

Folpet: CYP2J2

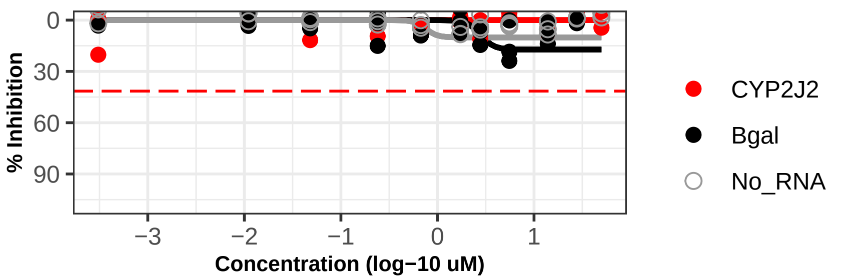

Folpet: CYP2C9

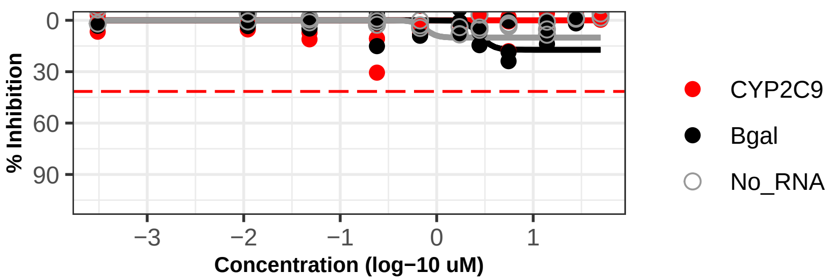

Folpet: CYP3A4

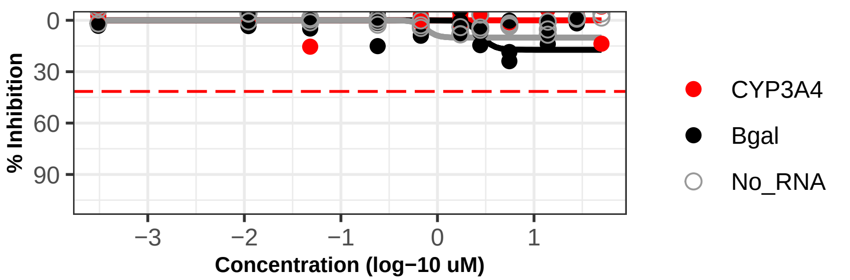

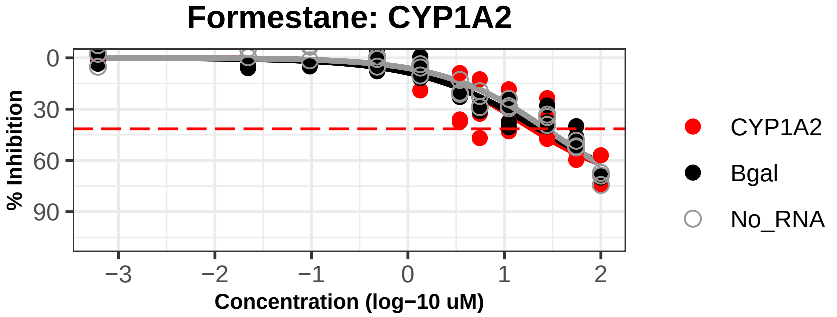
Formestane: CYP1A2
% Inhibition
Concentration (log−10 uM)
CYP1A2
Bgal
No_RNA

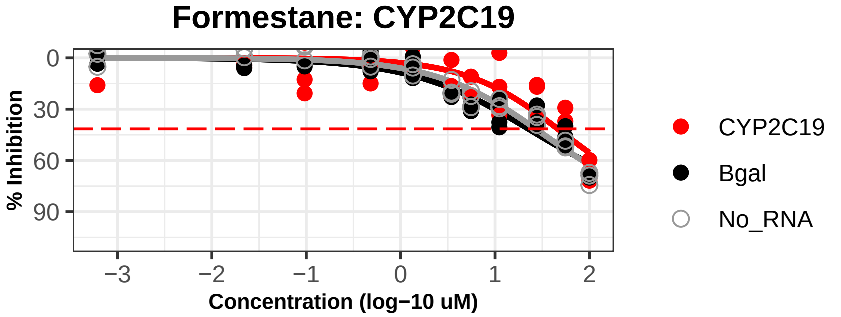
Formestane: CYP2C19
% Inhibition
Concentration (log−10 uM)
CYP2C19
Bgal
No_RNA

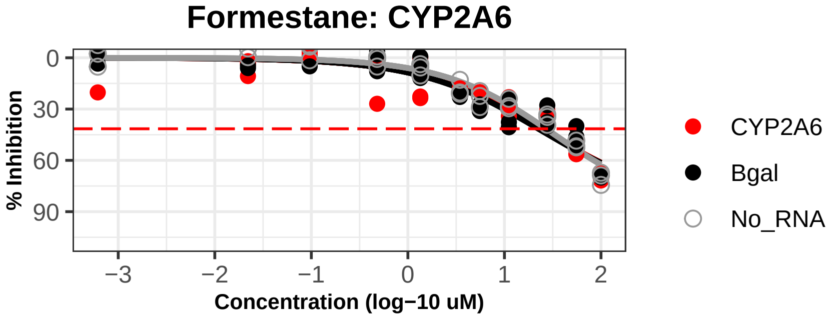
Formestane: CYP2A6
% Inhibition
Concentration (log−10 uM)
CYP2A6
Bgal
No_RNA

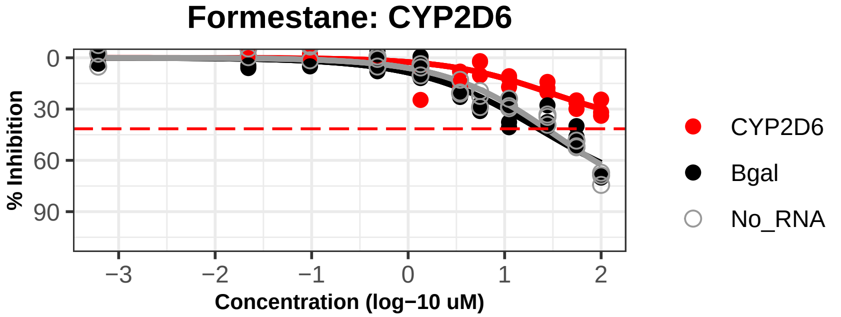
Formestane: CYP2D6
% Inhibition
Concentration (log−10 uM)
CYP2D6
Bgal
No_RNA

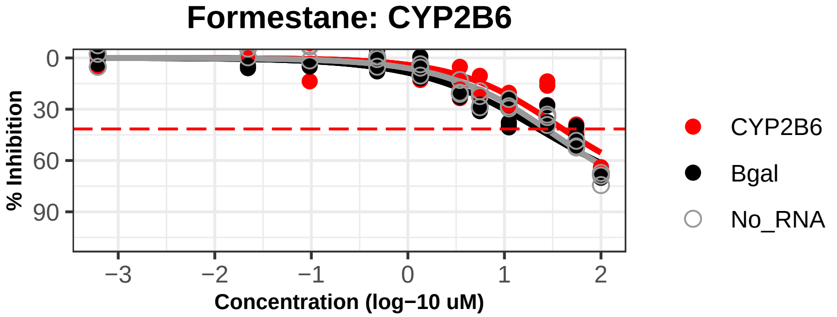
Formestane: CYP2B6
% Inhibition
Concentration (log−10 uM)
CYP2B6
Bgal
No_RNA

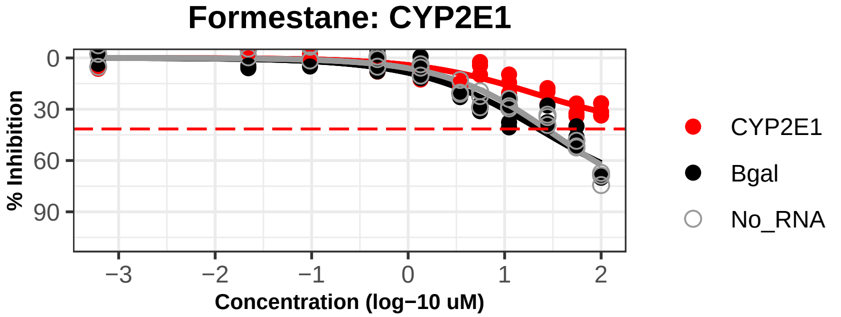
Formestane: CYP2E1
% Inhibition
Concentration (log−10 uM)
CYP2E1
Bgal
No_RNA

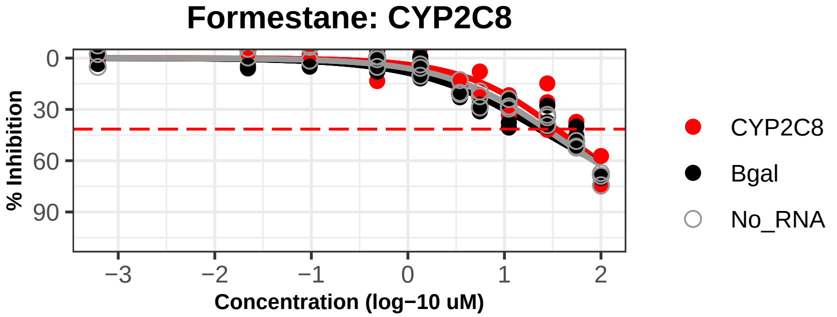
Formestane: CYP2C8
% Inhibition
Concentration (log−10 uM)
CYP2C8
Bgal
No_RNA

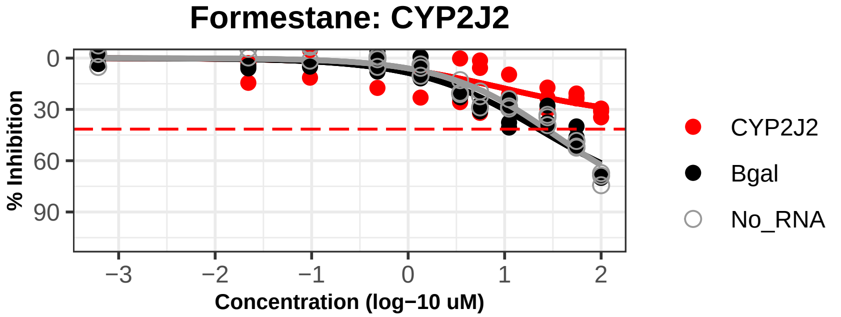
Formestane: CYP2J2
% Inhibition
Concentration (log−10 uM)
CYP2J2
Bgal
No_RNA

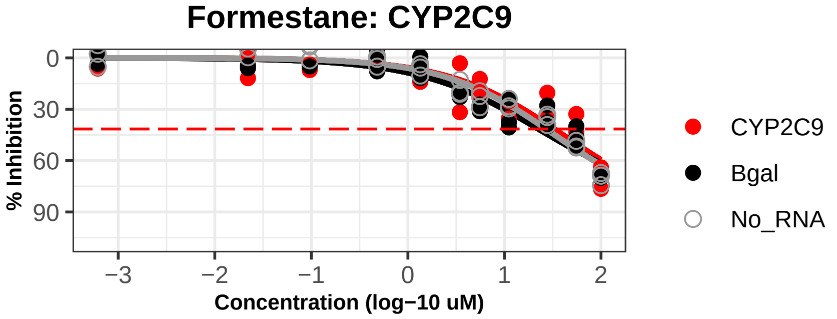
Formestane: CYP2C9
% Inhibition
Concentration (log−10 uM)
CYP2C9
Bgal
No_RNA

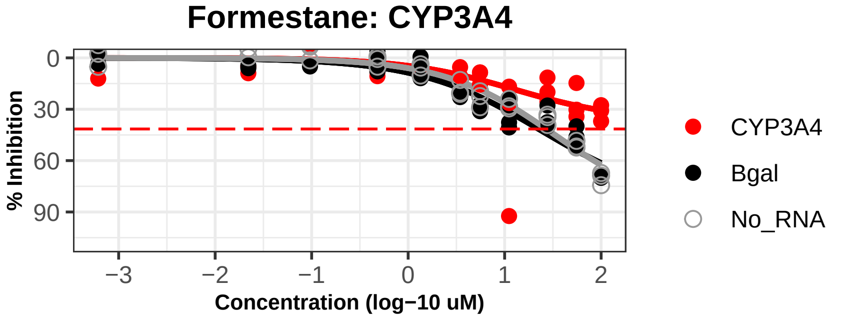
Formestane: CYP3A4
% Inhibition
Concentration (log−10 uM)
CYP3A4
Bgal
No_RNA

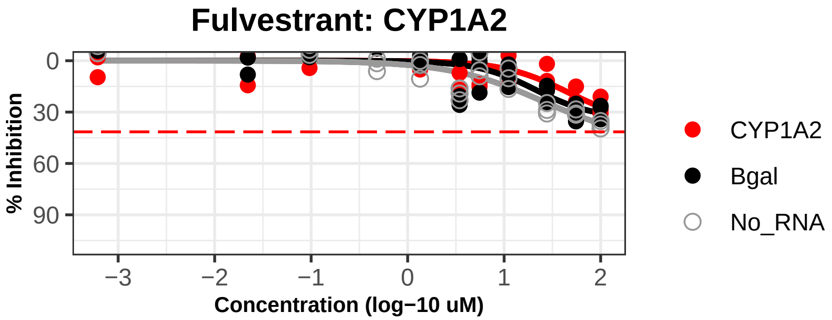
Fulvestrant: CYP1A2
% Inhibition
0
30
60
90
−3
−2
−1
0
1
2
Concentration (log−10 uM)
CYP1A2
Bgal
No_RNA

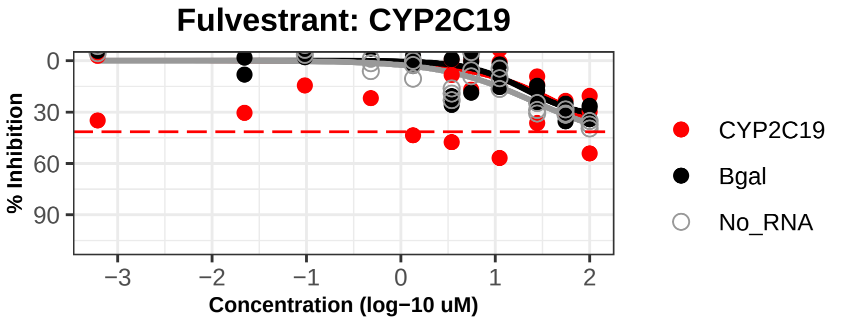
Fulvestrant: CYP2C19
% Inhibition
0
30
60
90
−3
−2
−1
0
1
2
Concentration (log−10 uM)
CYP2C19
Bgal
No_RNA

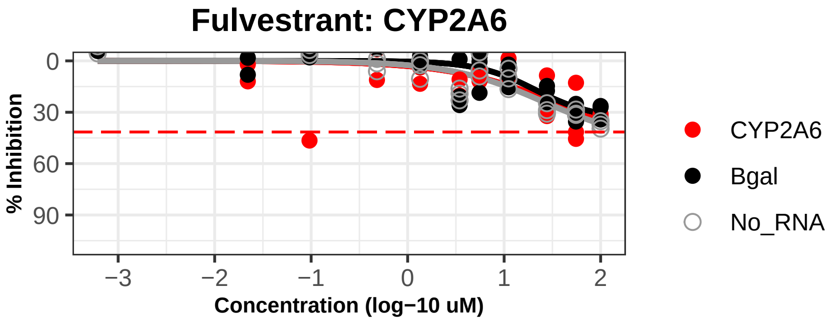
Fulvestrant: CYP2A6
% Inhibition
0
30
60
90
−3
−2
−1
0
1
2
Concentration (log−10 uM)
CYP2A6
Bgal
No_RNA

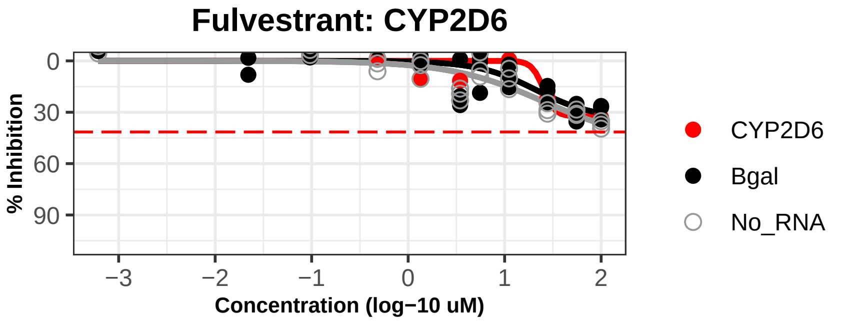
Fulvestrant: CYP2D6
% Inhibition
0
30
60
90
−3
−2
−1
0
1
2
Concentration (log−10 uM)
CYP2D6
Bgal
No_RNA

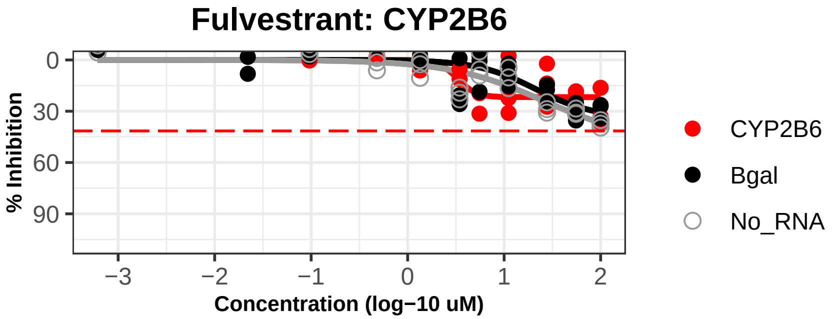
Fulvestrant: CYP2B6
% Inhibition
0
30
60
90
−3
−2
−1
0
1
2
Concentration (log−10 uM)
CYP2B6
Bgal
No_RNA

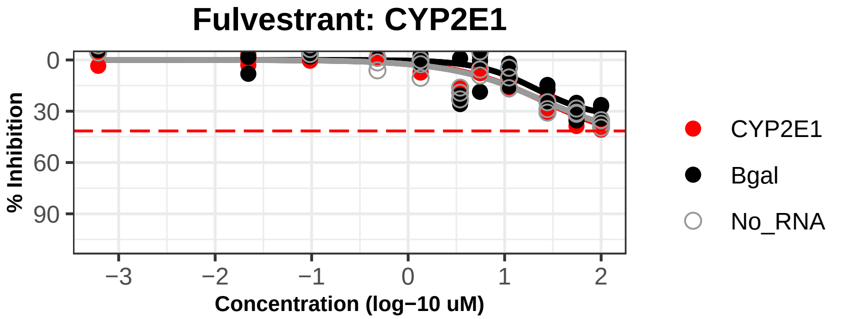
Fulvestrant: CYP2E1
% Inhibition
0
30
60
90
−3
−2
−1
0
1
2
Concentration (log−10 uM)
CYP2E1
Bgal
No_RNA

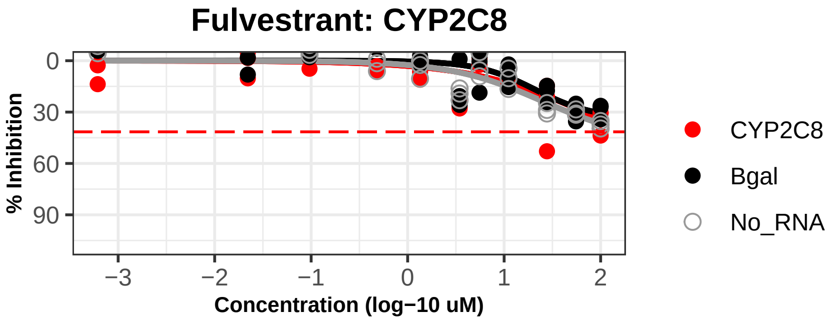
Fulvestrant: CYP2C8
% Inhibition
0
30
60
90
−3
−2
−1
0
1
2
Concentration (log−10 uM)
CYP2C8
Bgal
No_RNA

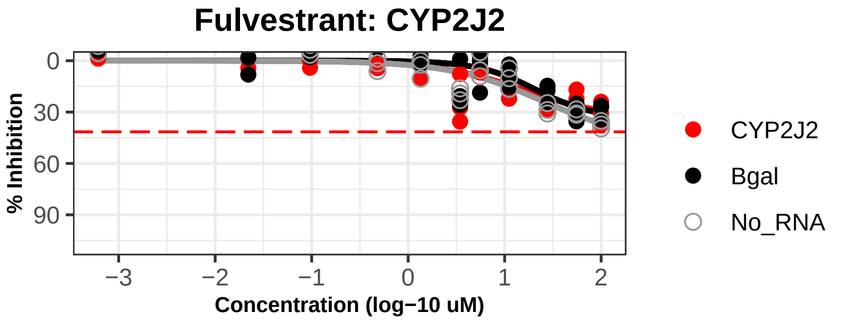
Fulvestrant: CYP2J2
% Inhibition
0
30
60
90
−3
−2
−1
0
1
2
Concentration (log−10 uM)
CYP2J2
Bgal
No_RNA

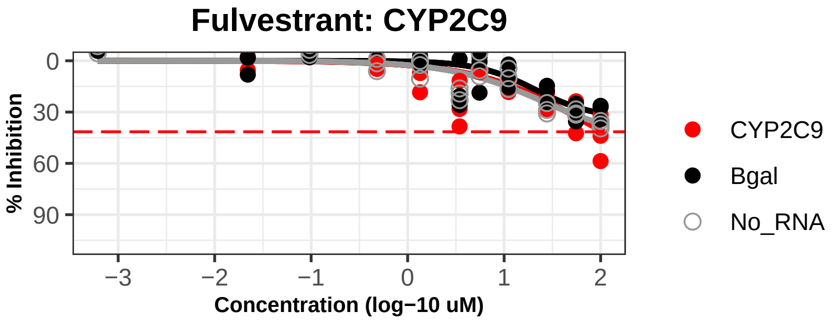
Fulvestrant: CYP2C9
% Inhibition
0
30
60
90
−3
−2
−1
0
1
2
Concentration (log−10 uM)
CYP2C9
Bgal
No_RNA

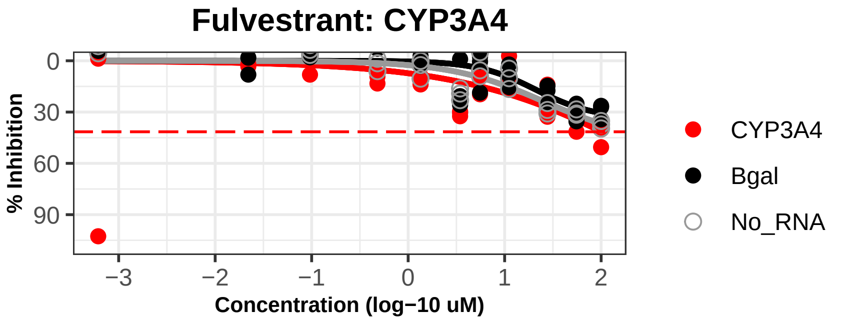
Fulvestrant: CYP3A4
% Inhibition
0
30
60
90
−3
−2
−1
0
1
2
Concentration (log−10 uM)
CYP3A4
Bgal
No_RNA

Genistein: CYP1A2

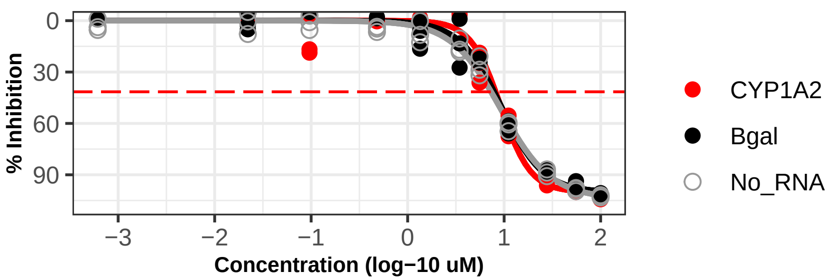

Genistein: CYP2C19

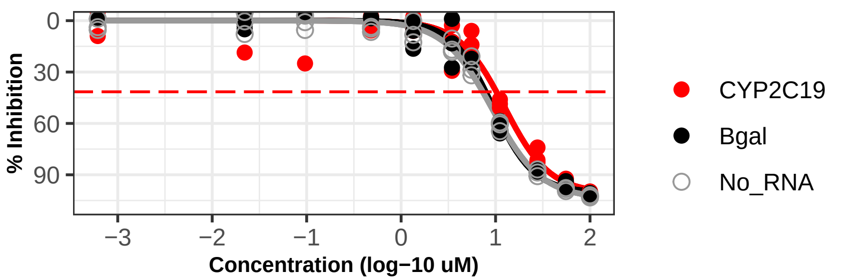

Genistein: CYP2A6

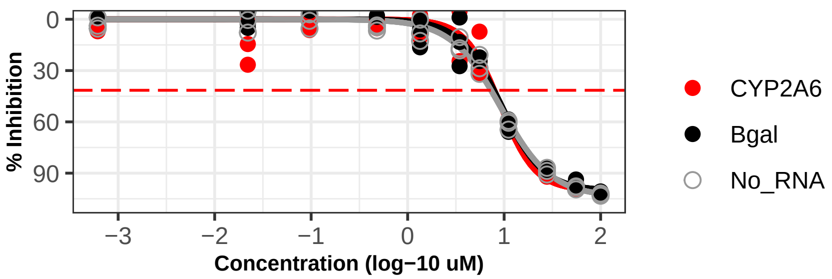

Genistein: CYP2D6

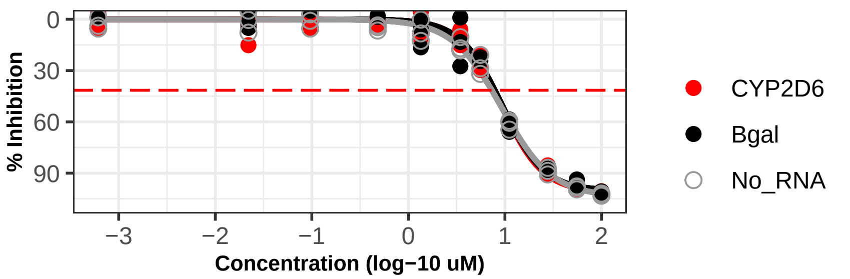

Genistein: CYP2B6

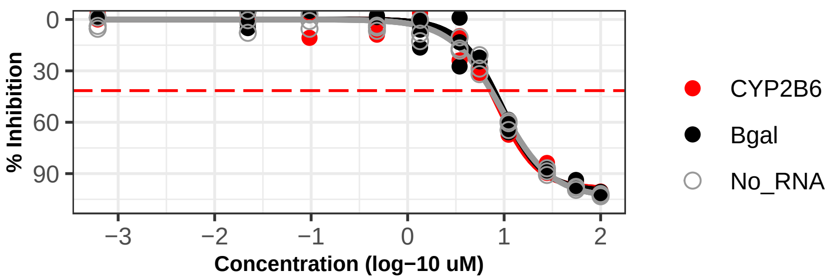

Genistein: CYP2E1

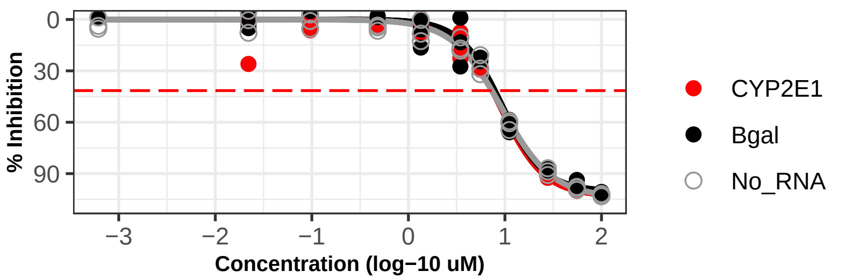

Genistein: CYP2C8

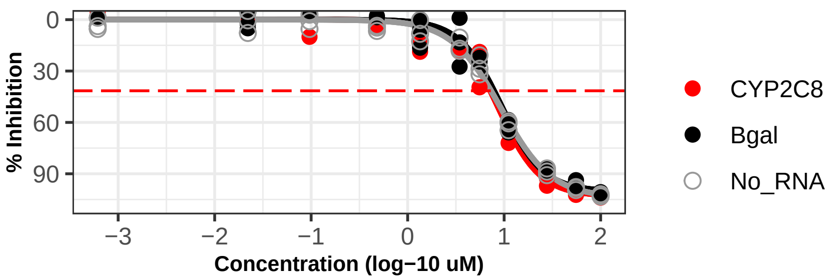

Genistein: CYP2J2

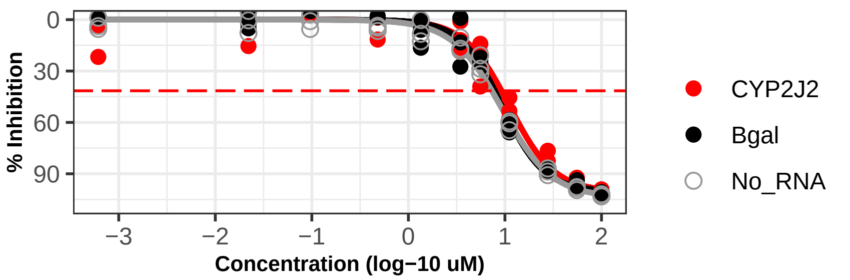

Genistein: CYP2C9

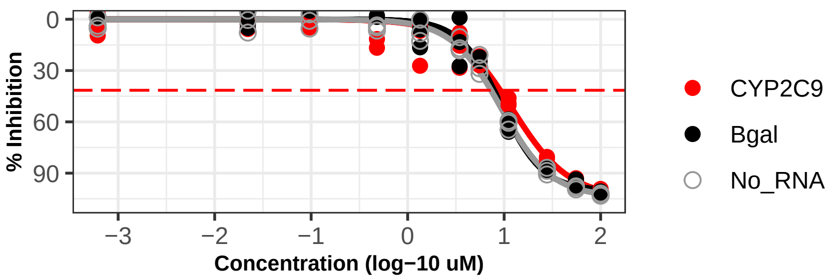

Genistein: CYP3A4

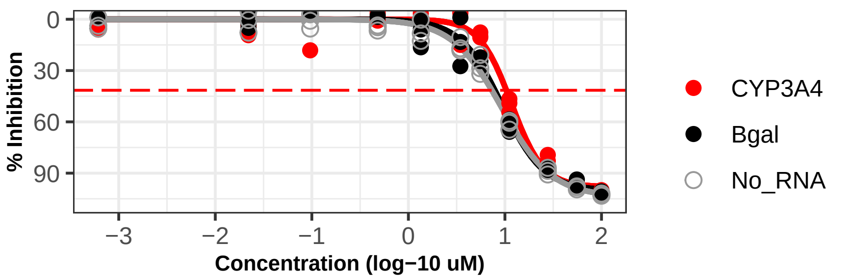

Hydroxyflutamide: CYP1A2

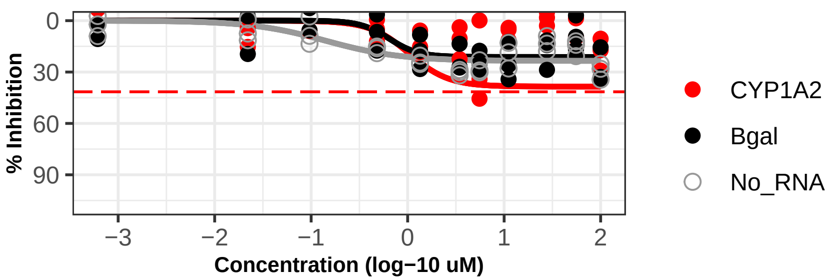

Hydroxyflutamide: CYP2C19

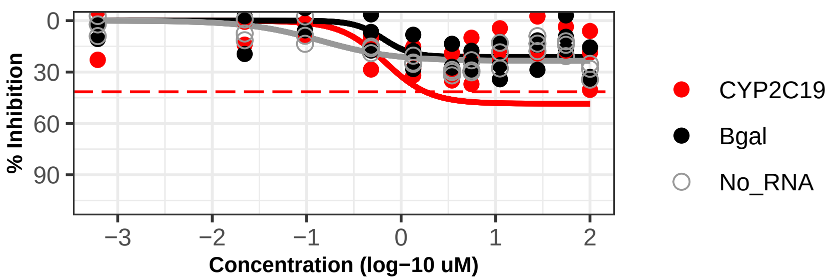

Hydroxyflutamide: CYP2A6

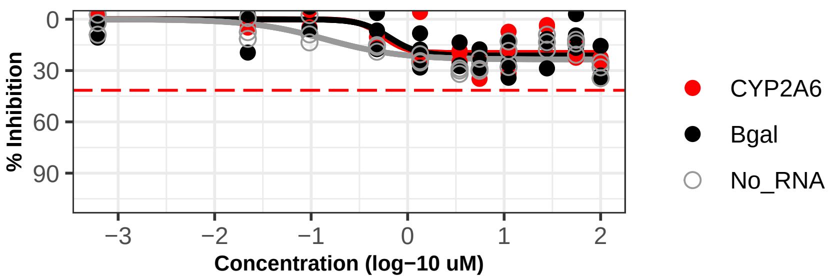

Hydroxyflutamide: CYP2D6

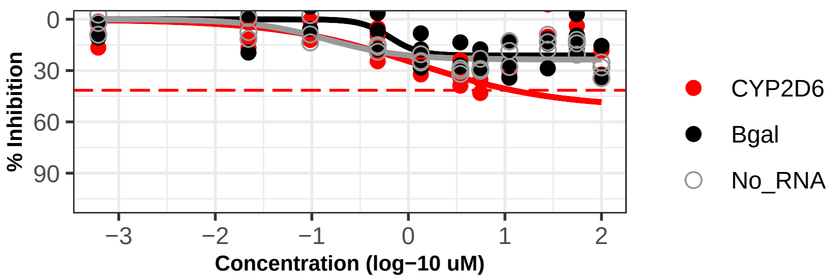

Hydroxyflutamide: CYP2B6

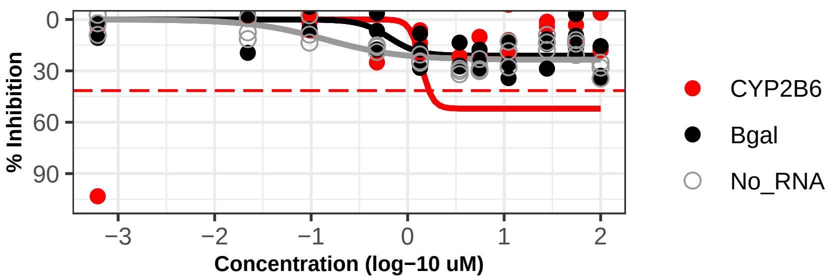

Hydroxyflutamide: CYP2E1

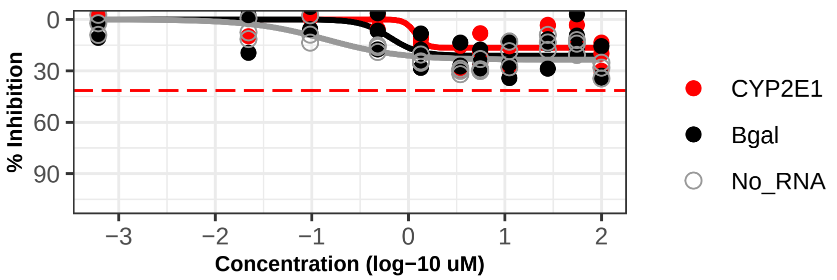

Hydroxyflutamide: CYP2C8

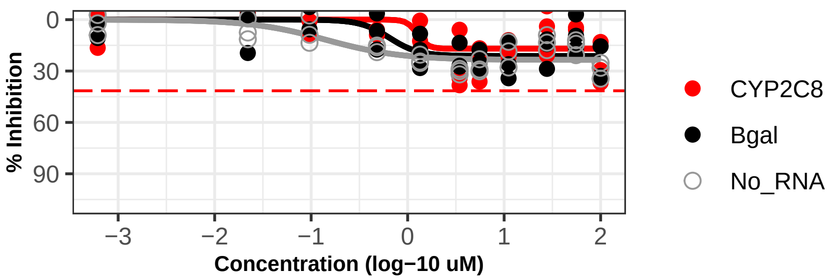

Hydroxyflutamide: CYP2J2

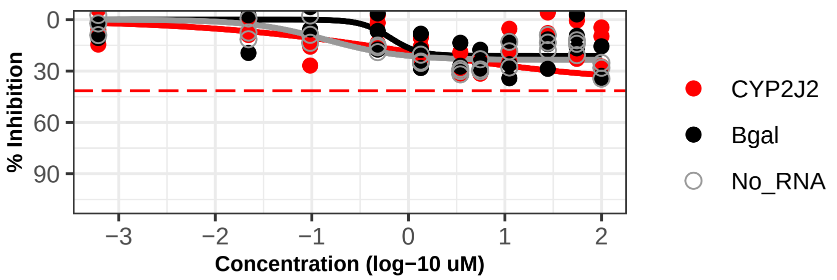

Hydroxyflutamide: CYP2C9

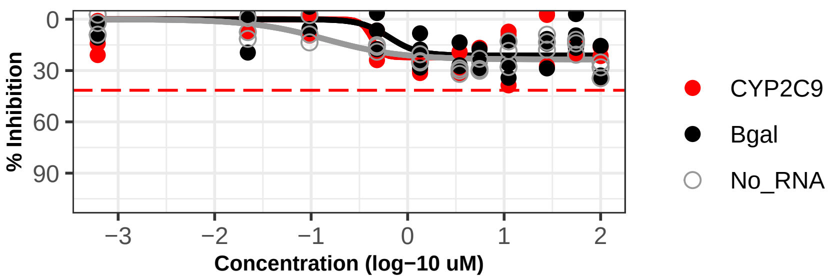

Hydroxyflutamide: CYP3A4

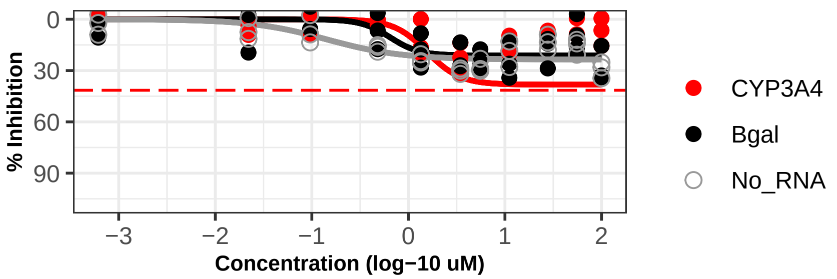

Hydroxyprogesterone caproate: CYP1A2

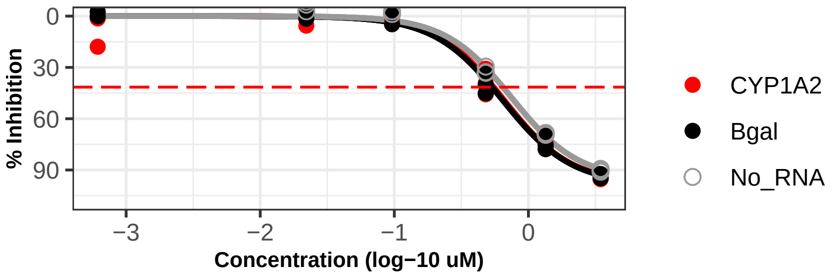

Hydroxyprogesterone caproate: CYP2C19

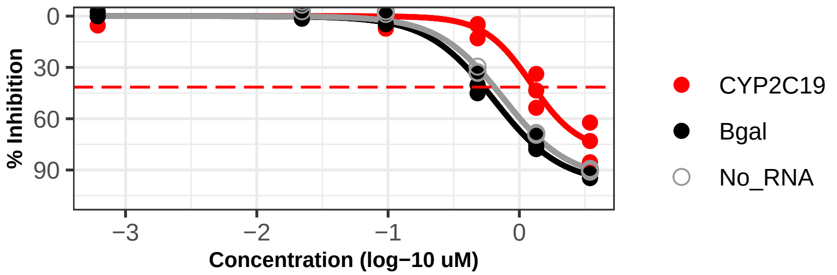

Hydroxyprogesterone caproate: CYP2A6

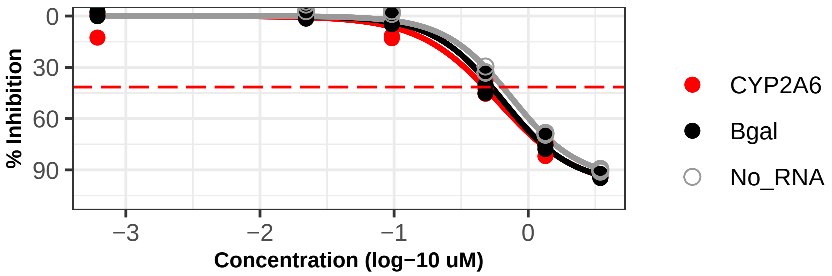

Hydroxyprogesterone caproate: CYP2D6

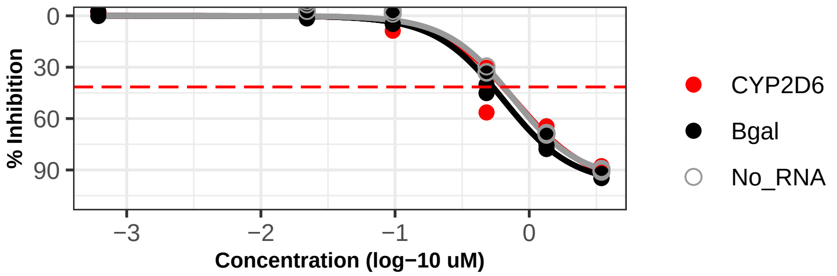

Hydroxyprogesterone caproate: CYP2B6

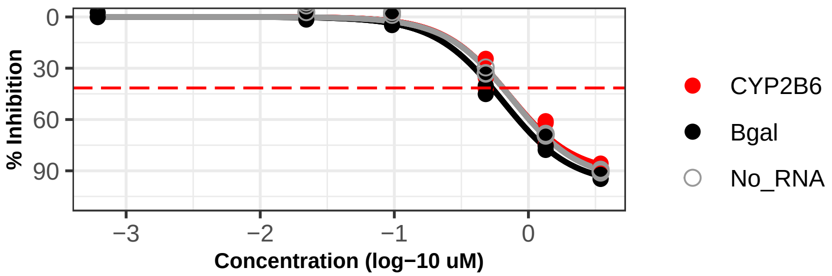

Hydroxyprogesterone caproate: CYP2E1

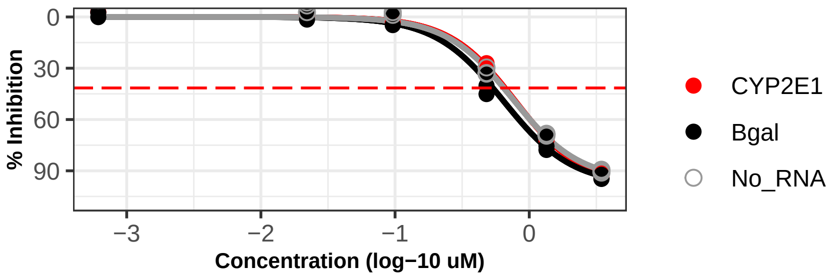

Hydroxyprogesterone caproate: CYP2C8

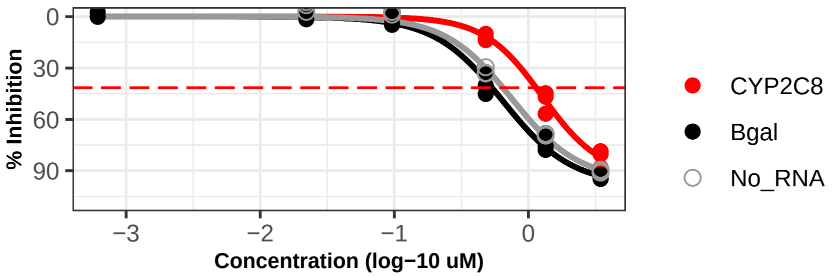

Hydroxyprogesterone caproate: CYP2J2

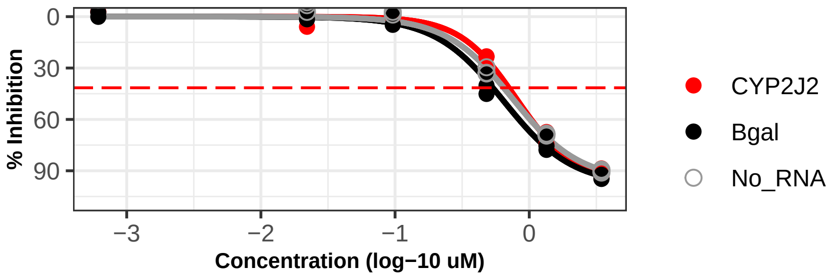

Hydroxyprogesterone caproate: CYP2C9

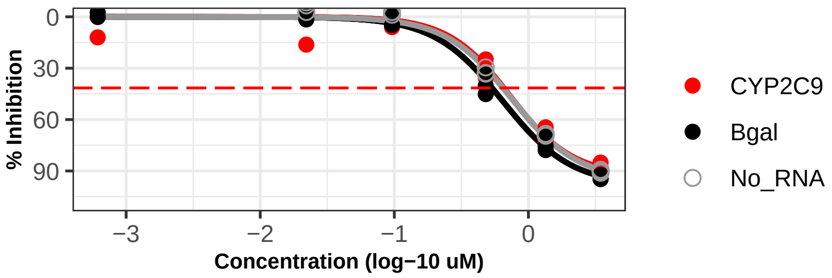

Hydroxyprogesterone caproate: CYP3A4

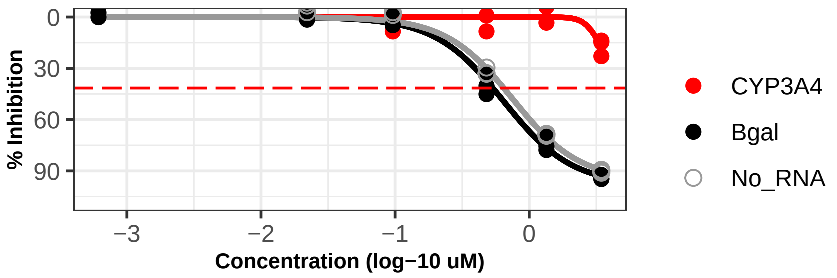

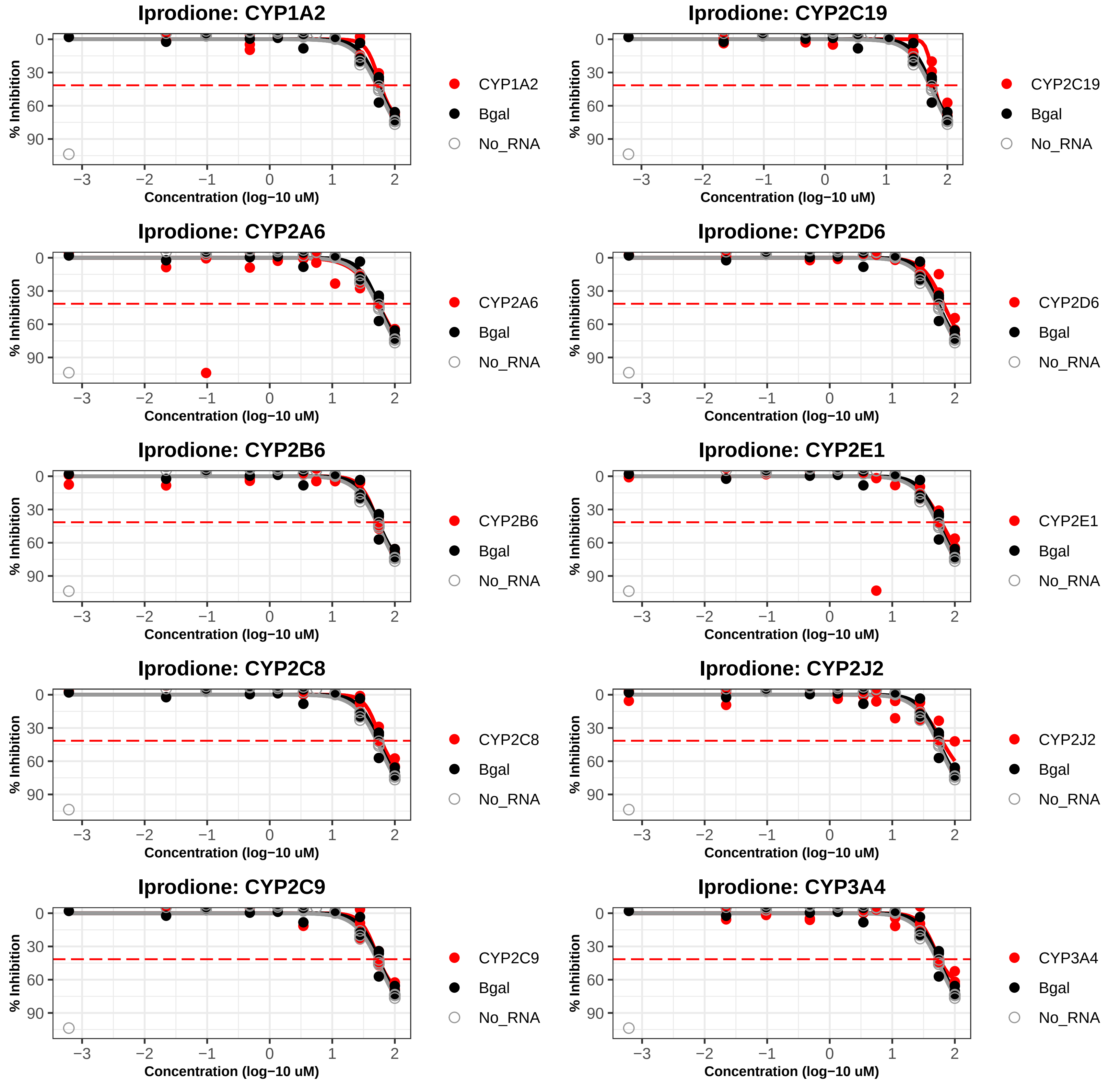

Iprodione: CYP1A2
Iprodione: CYP2C19
Iprodione: CYP2A6
Iprodione: CYP2D6
Iprodione: CYP2B6
Iprodione: CYP2E1
Iprodione: CYP2C8
Iprodione: CYP2J2
Iprodione: CYP2C9
Iprodione: CYP3A4
% Inhibition
0
30
60
90
Concentration (log−10 uM)
−3
−2
−1
0
1
2
CYP1A2
CYP2C19
CYP2A6
CYP2D6
CYP2B6
CYP2E1
CYP2C8
CYP2J2
CYP2C9
CYP3A4
Bgal
No_RNA

Kaempferol: CYP1A2

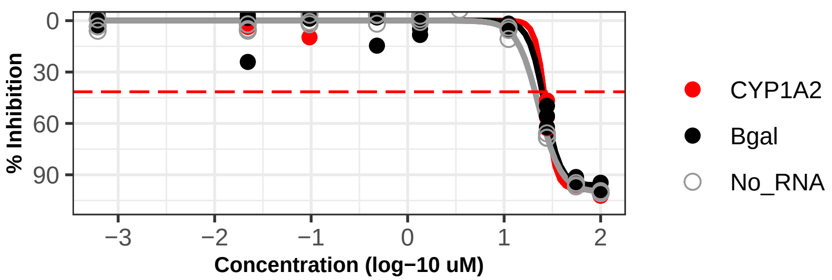

Kaempferol: CYP2C19

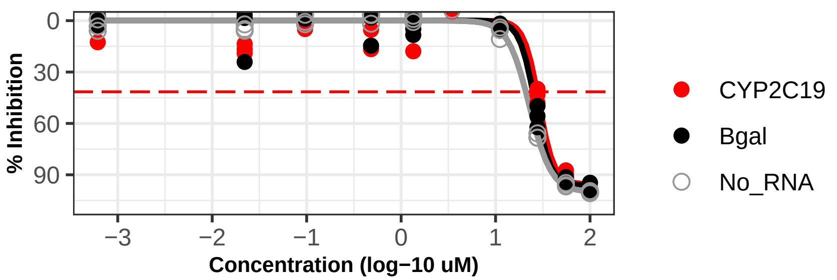

Kaempferol: CYP2A6

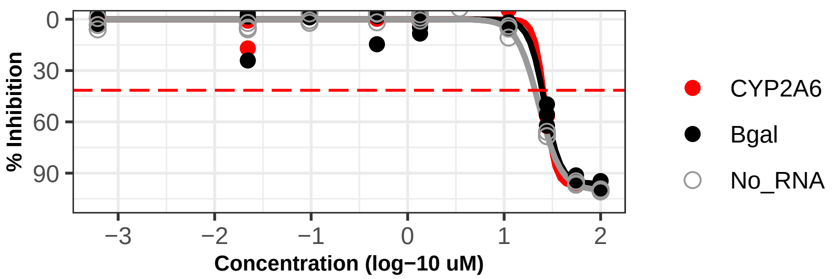

Kaempferol: CYP2D6

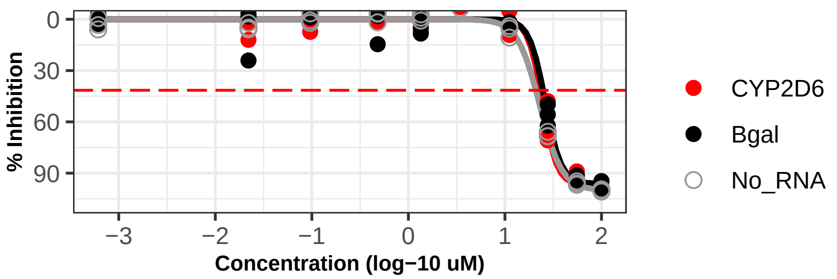

Kaempferol: CYP2B6

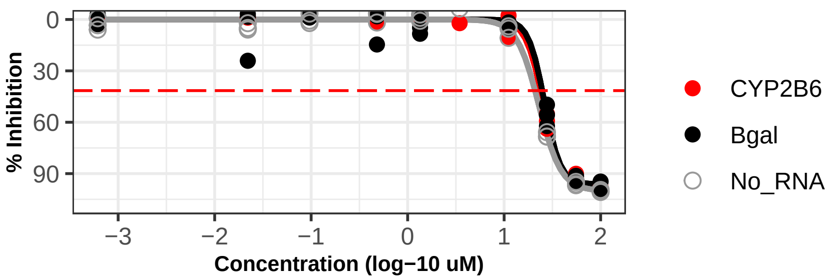

Kaempferol: CYP2E1

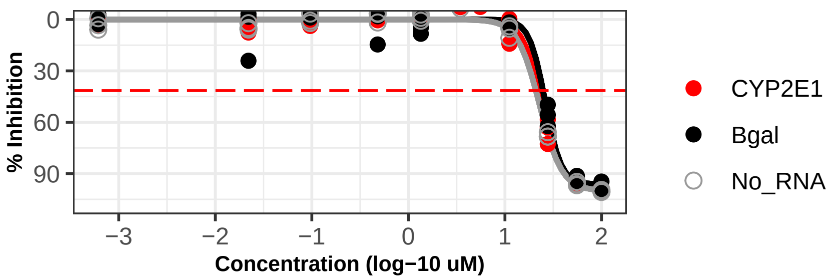

Kaempferol: CYP2C8

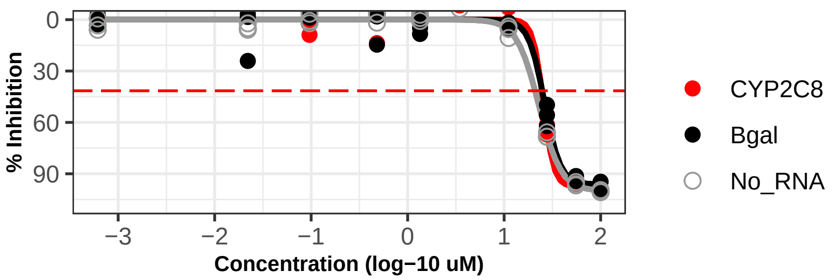

Kaempferol: CYP2J2

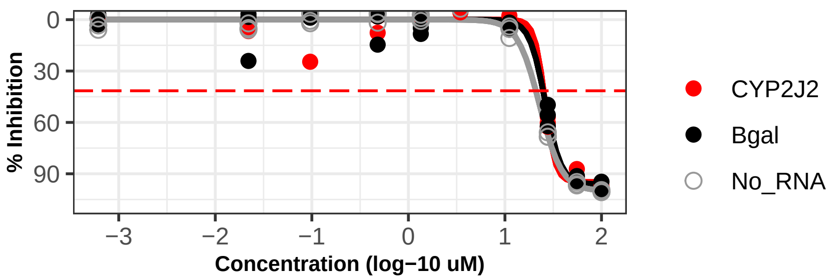

Kaempferol: CYP2C9

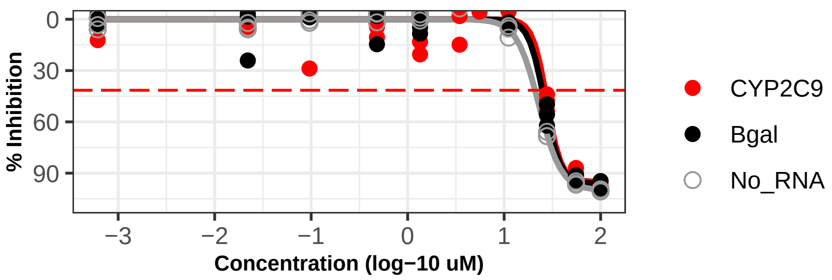

Kaempferol: CYP3A4

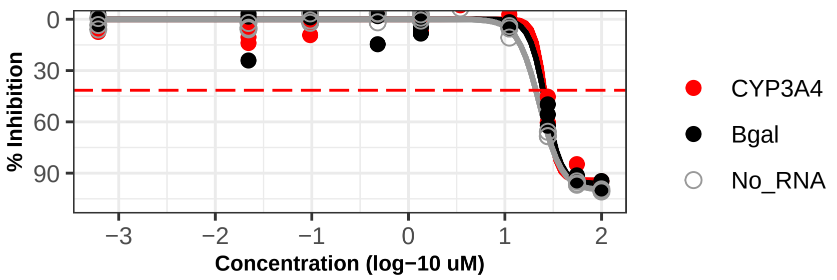

Letrozole: CYP1A2

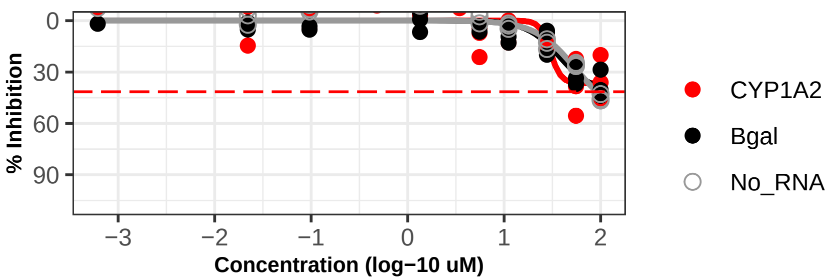

Letrozole: CYP2C19

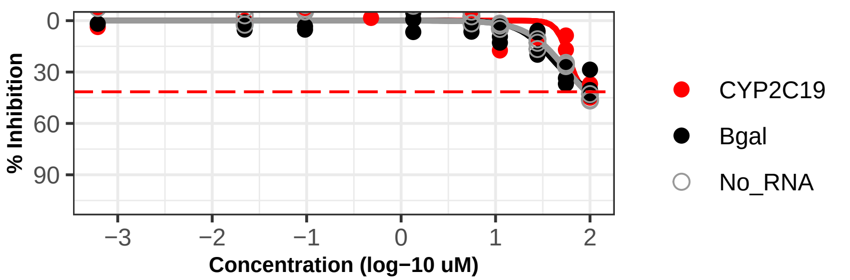

Letrozole: CYP2A6

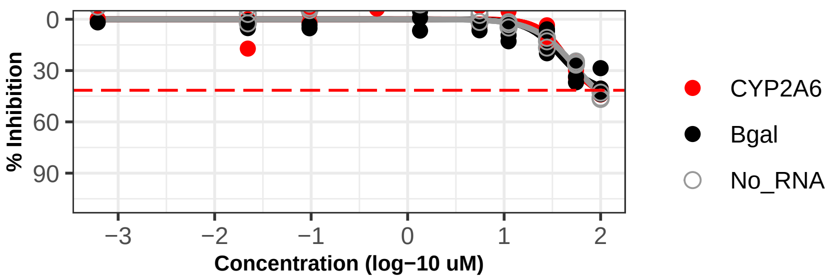

Letrozole: CYP2D6

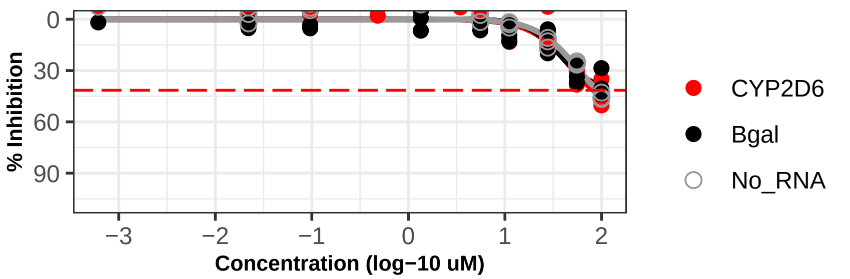

Letrozole: CYP2B6

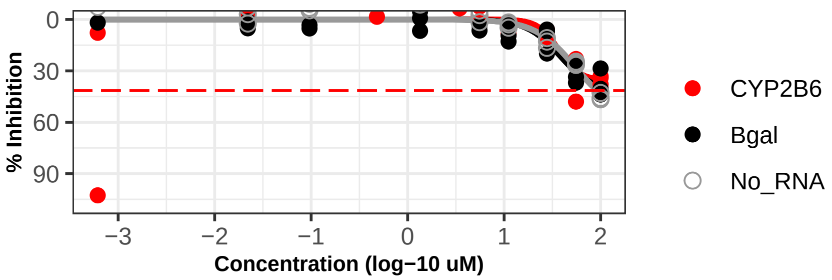

Letrozole: CYP2E1

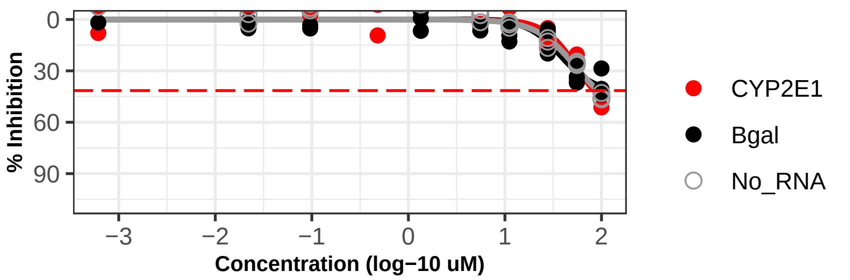

Letrozole: CYP2C8

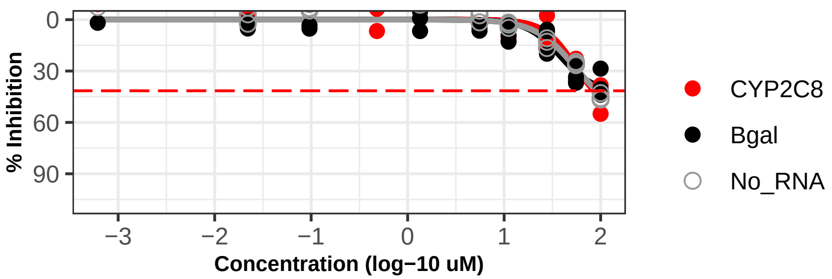

Letrozole: CYP2J2

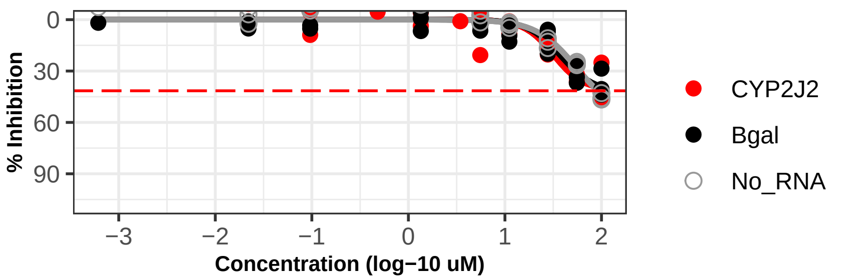

Letrozole: CYP2C9

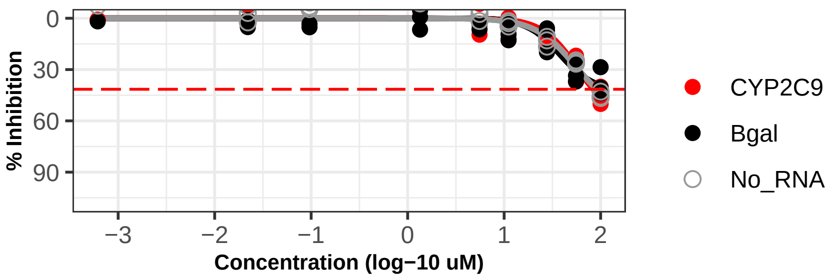

Letrozole: CYP3A4

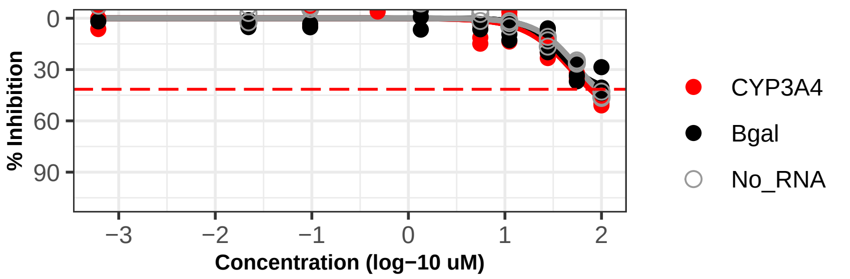

Levonorgestrel: CYP1A2

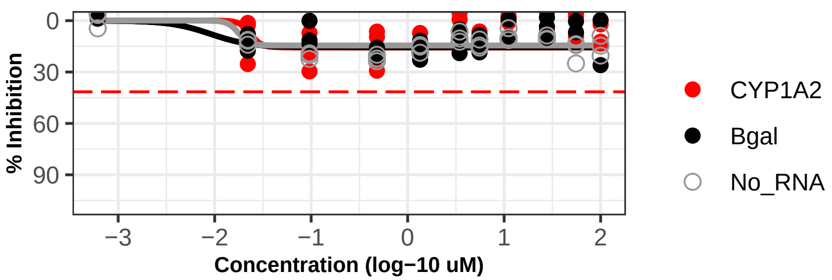

Levonorgestrel: CYP2C19

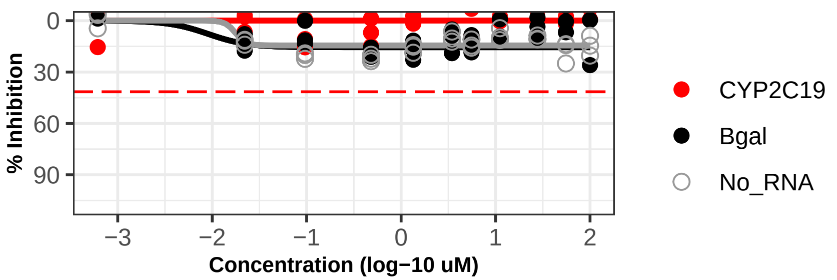

Levonorgestrel: CYP2A6

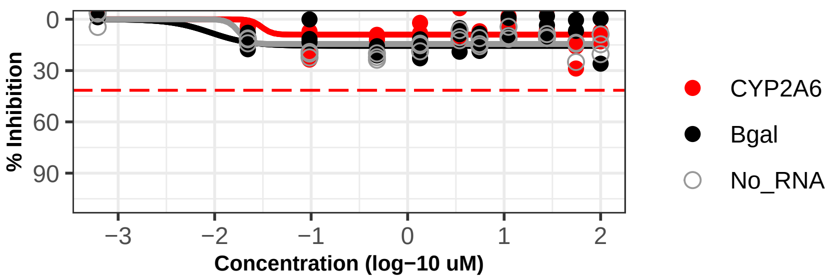

Levonorgestrel: CYP2D6

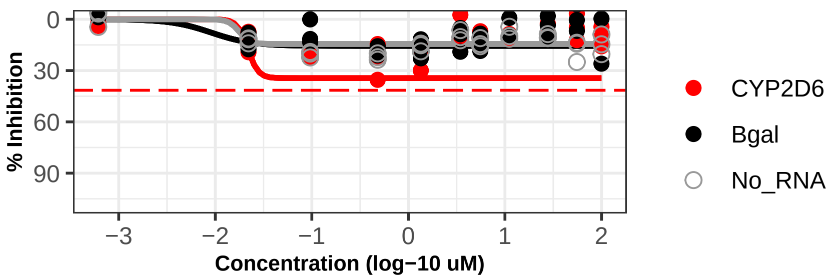

Levonorgestrel: CYP2B6

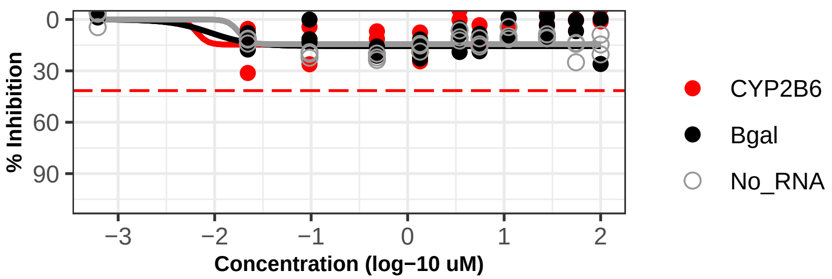

Levonorgestrel: CYP2E1

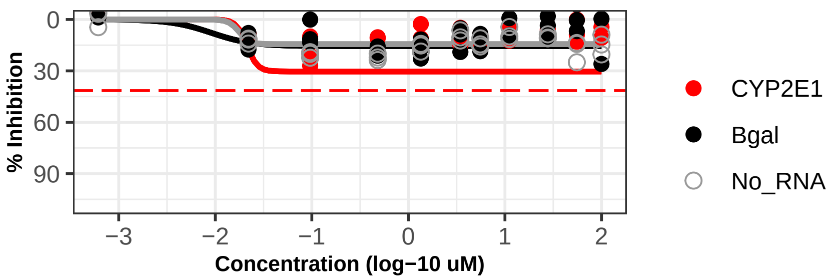

Levonorgestrel: CYP2C8

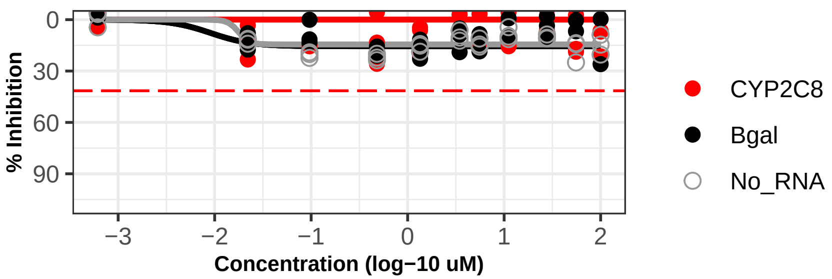

Levonorgestrel: CYP2J2

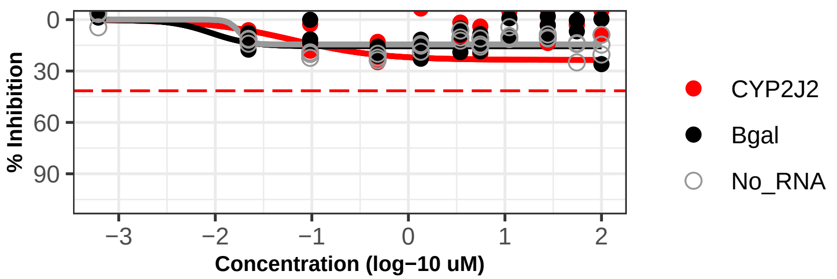

Levonorgestrel: CYP2C9

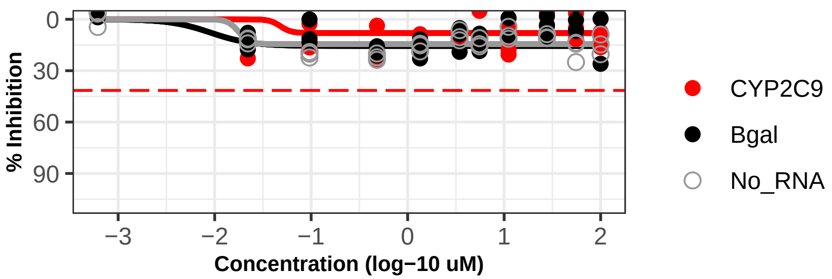

Levonorgestrel: CYP3A4

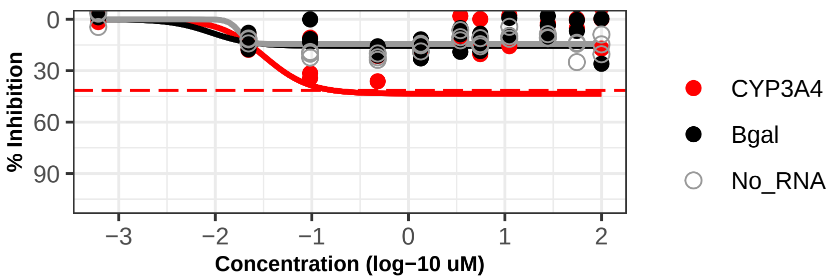

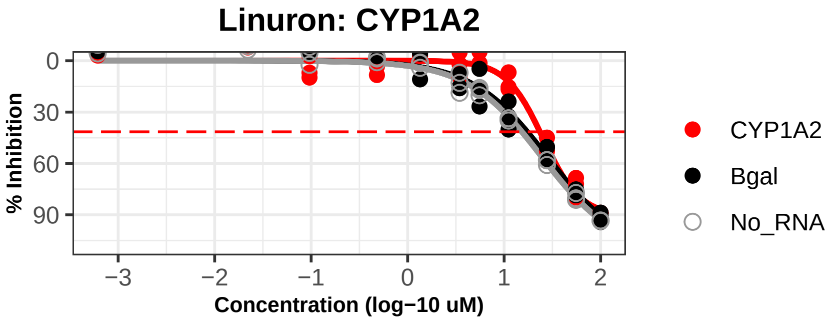
Linuron: CYP1A2
% Inhibition
0
30
60
90
−3
−2
−1
0
1
2
Concentration (log−10 uM)
CYP1A2
Bgal
No_RNA

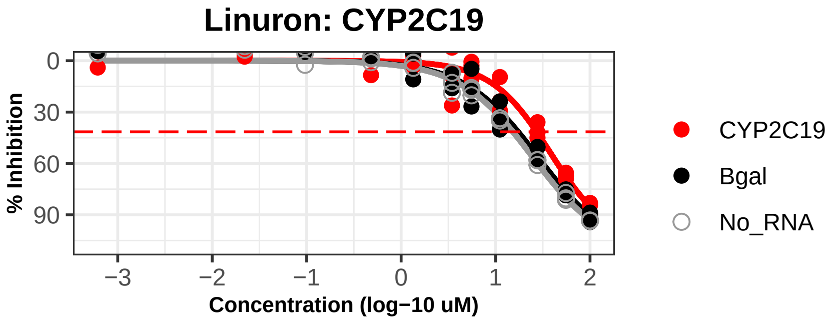
Linuron: CYP2C19
% Inhibition
0
30
60
90
−3
−2
−1
0
1
2
Concentration (log−10 uM)
CYP2C19
Bgal
No_RNA

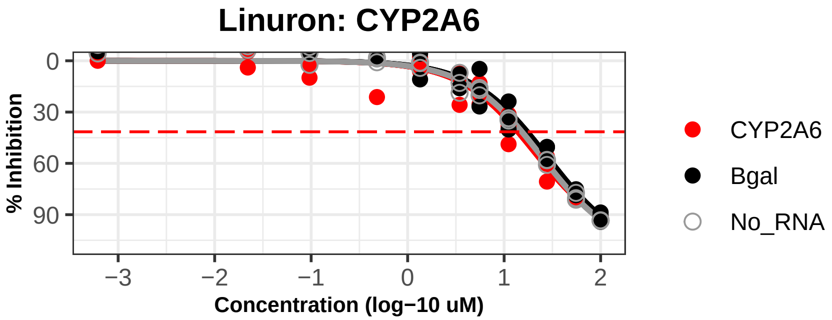
Linuron: CYP2A6
% Inhibition
0
30
60
90
−3
−2
−1
0
1
2
Concentration (log−10 uM)
CYP2A6
Bgal
No_RNA

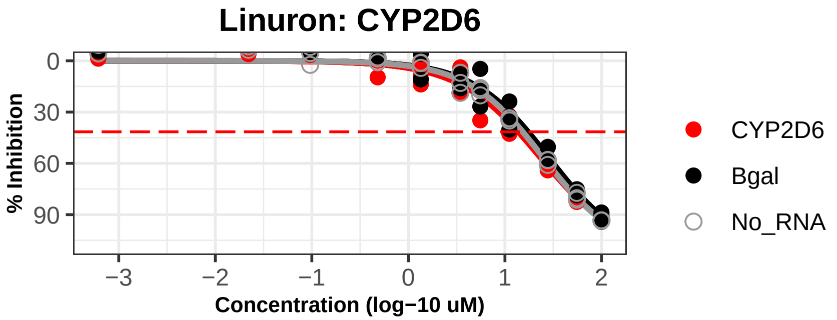
Linuron: CYP2D6
% Inhibition
0
30
60
90
−3
−2
−1
0
1
2
Concentration (log−10 uM)
CYP2D6
Bgal
No_RNA

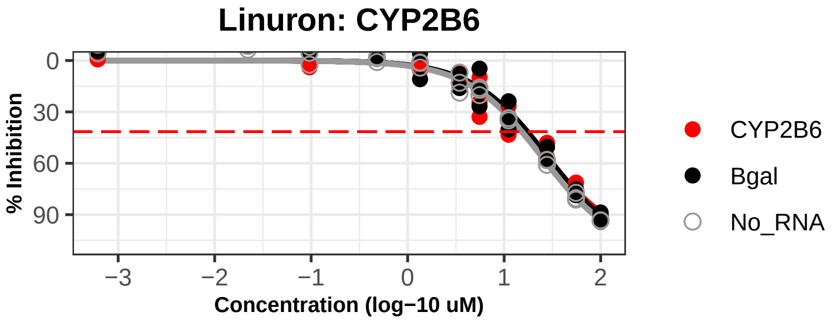
Linuron: CYP2B6
% Inhibition
0
30
60
90
−3
−2
−1
0
1
2
Concentration (log−10 uM)
CYP2B6
Bgal
No_RNA

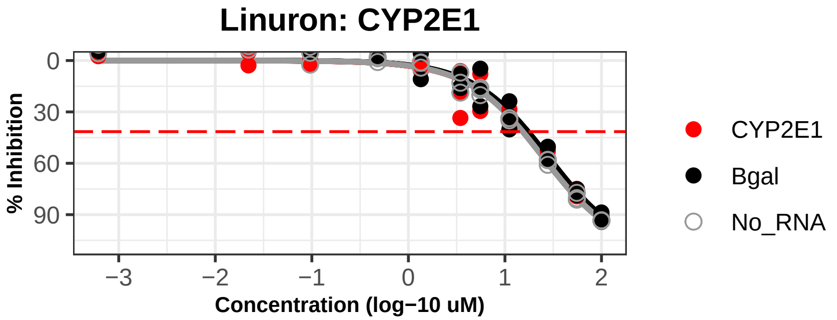
Linuron: CYP2E1
% Inhibition
0
30
60
90
−3
−2
−1
0
1
2
Concentration (log−10 uM)
CYP2E1
Bgal
No_RNA

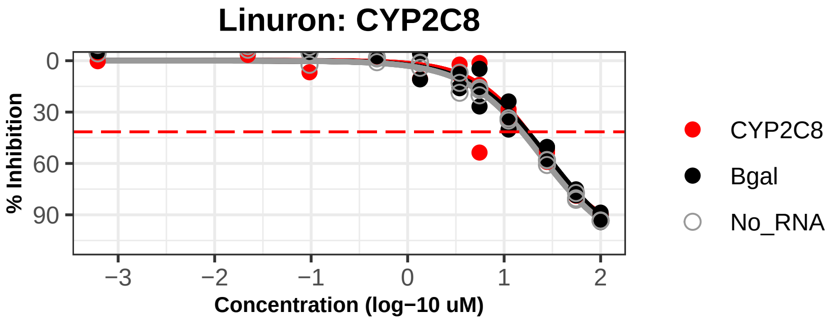
Linuron: CYP2C8
% Inhibition
0
30
60
90
−3
−2
−1
0
1
2
Concentration (log−10 uM)
CYP2C8
Bgal
No_RNA

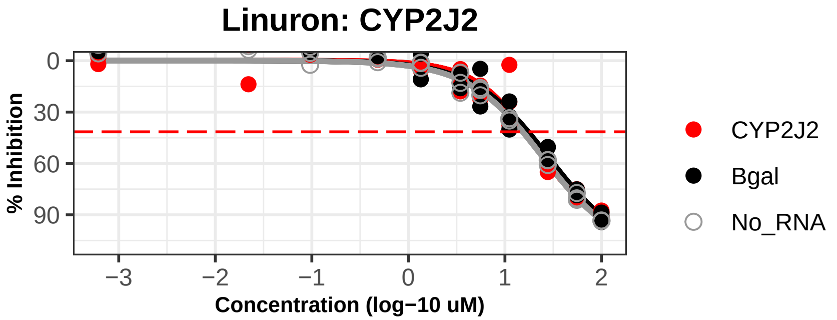
Linuron: CYP2J2
% Inhibition
0
30
60
90
−3
−2
−1
0
1
2
Concentration (log−10 uM)
CYP2J2
Bgal
No_RNA

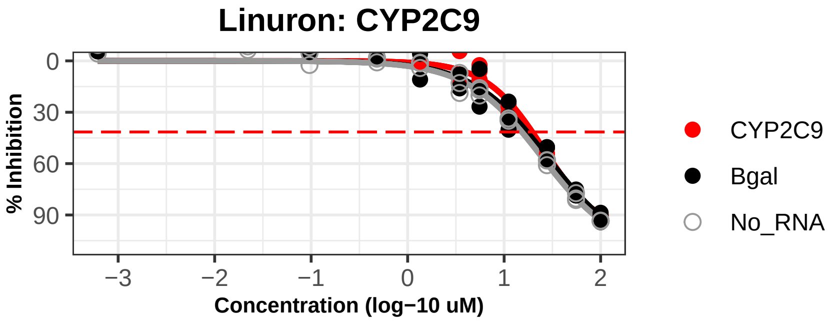
Linuron: CYP2C9
% Inhibition
0
30
60
90
−3
−2
−1
0
1
2
Concentration (log−10 uM)
CYP2C9
Bgal
No_RNA

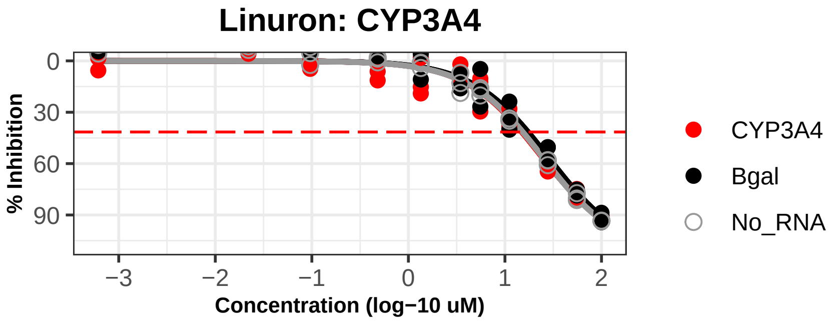
Linuron: CYP3A4
% Inhibition
0
30
60
90
−3
−2
−1
0
1
2
Concentration (log−10 uM)
CYP3A4
Bgal
No_RNA

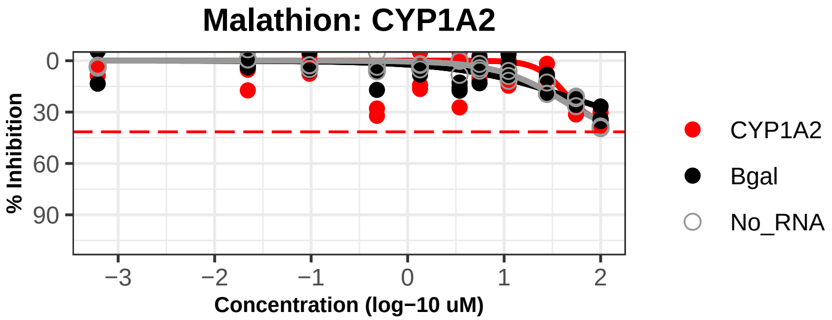
Malathion: CYP1A2
% Inhibition
Concentration (log−10 uM)
CYP1A2
Bgal
No_RNA

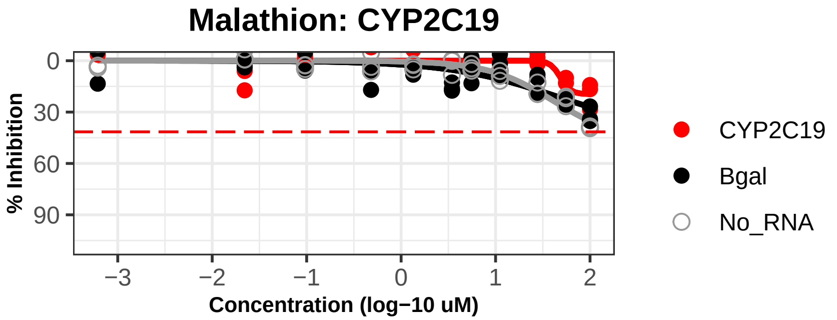
Malathion: CYP2C19
% Inhibition
Concentration (log−10 uM)
CYP2C19
Bgal
No_RNA

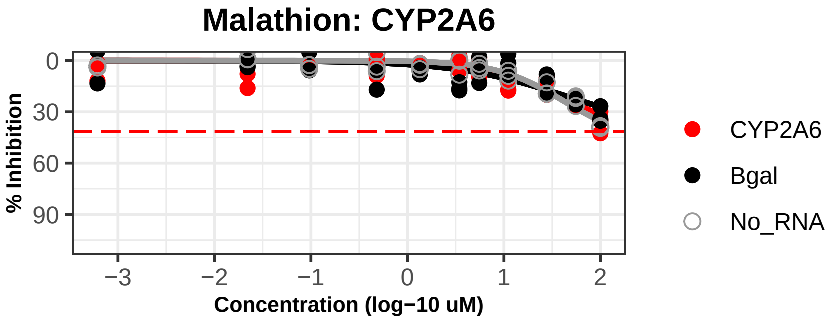
Malathion: CYP2A6
% Inhibition
Concentration (log−10 uM)
CYP2A6
Bgal
No_RNA

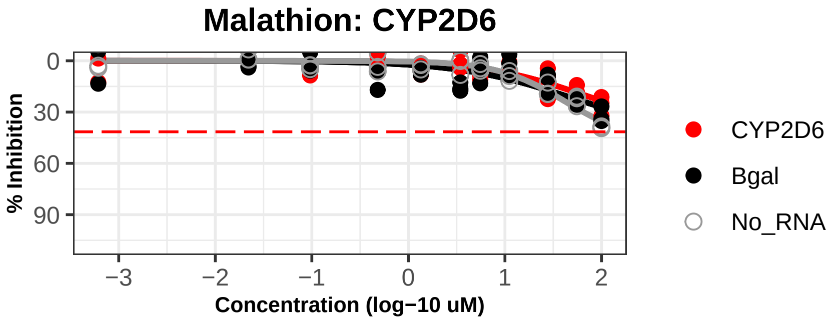
Malathion: CYP2D6
% Inhibition
Concentration (log−10 uM)
CYP2D6
Bgal
No_RNA

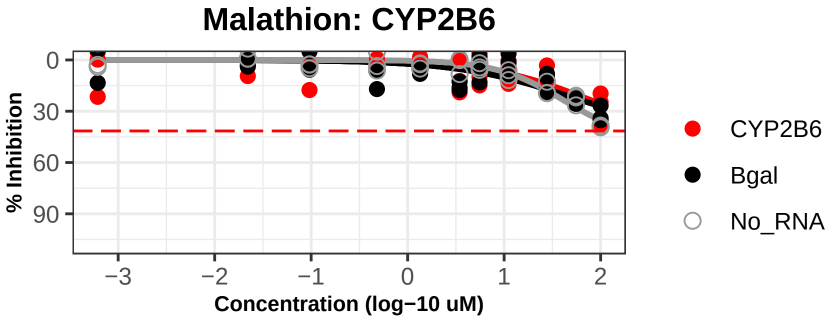
Malathion: CYP2B6
% Inhibition
Concentration (log−10 uM)
CYP2B6
Bgal
No_RNA

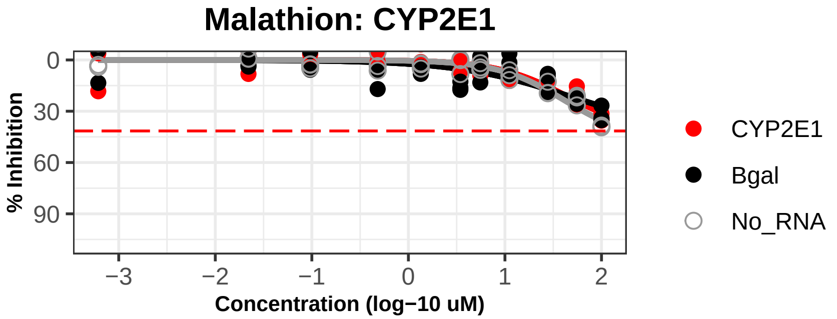
Malathion: CYP2E1
% Inhibition
Concentration (log−10 uM)
CYP2E1
Bgal
No_RNA

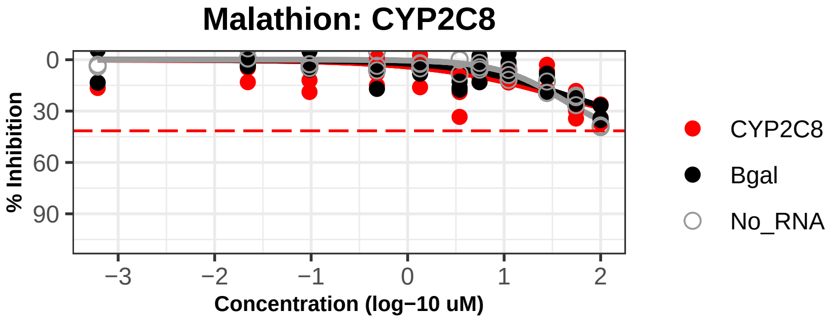
Malathion: CYP2C8
% Inhibition
Concentration (log−10 uM)
CYP2C8
Bgal
No_RNA

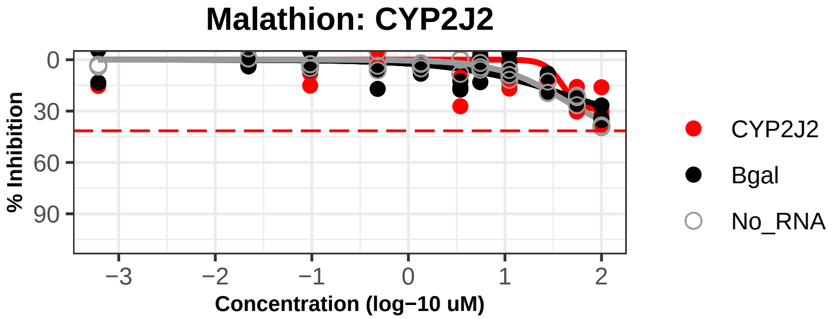
Malathion: CYP2J2
% Inhibition
Concentration (log−10 uM)
CYP2J2
Bgal
No_RNA

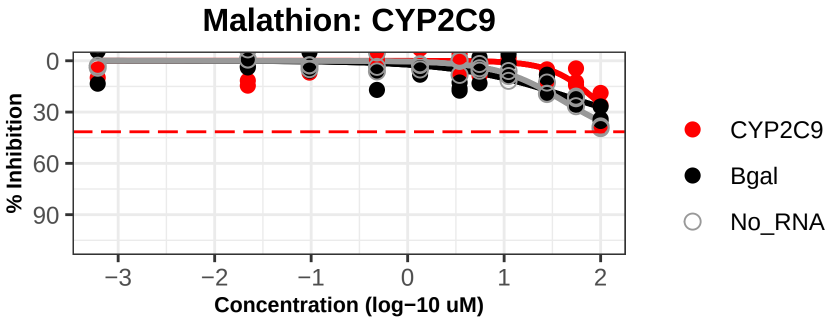
Malathion: CYP2C9
% Inhibition
Concentration (log−10 uM)
CYP2C9
Bgal
No_RNA

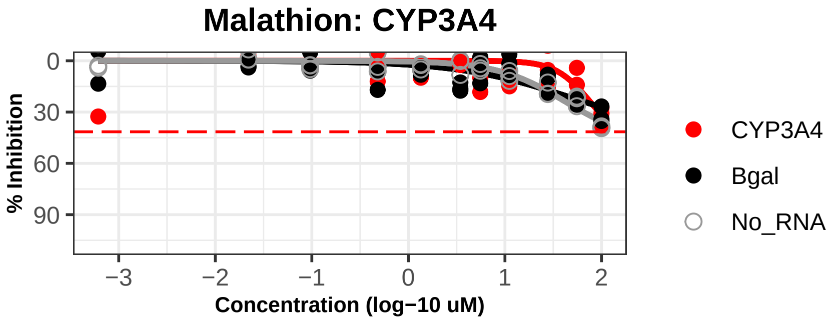
Malathion: CYP3A4
% Inhibition
Concentration (log−10 uM)
CYP3A4
Bgal
No_RNA

Mestranol: CYP1A2

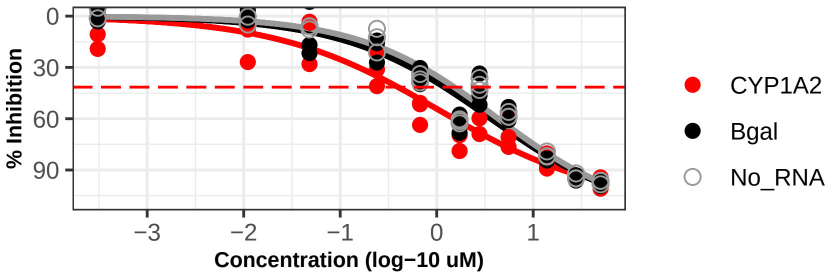

Mestranol: CYP2C19

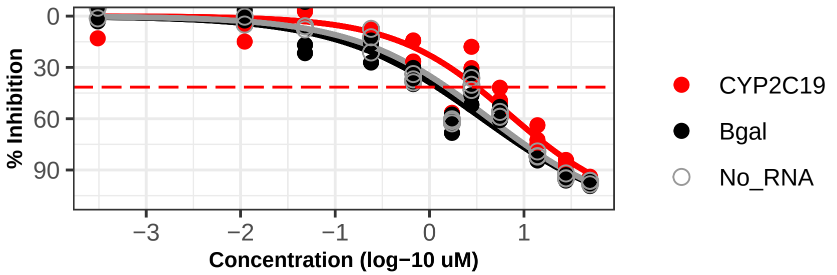

Mestranol: CYP2A6

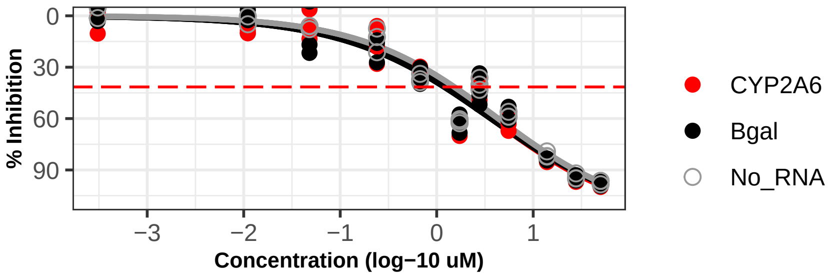

Mestranol: CYP2D6

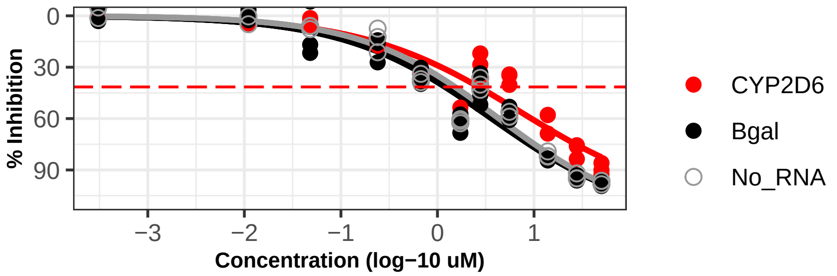

Mestranol: CYP2B6

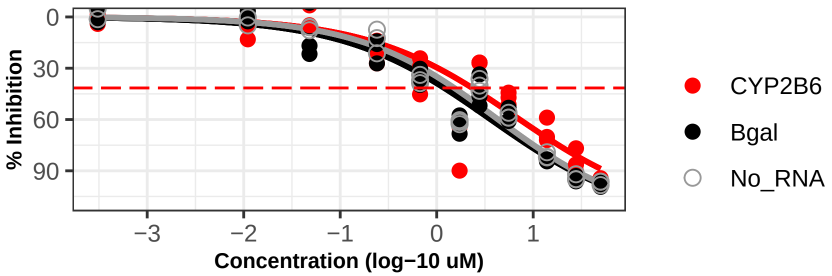

Mestranol: CYP2E1

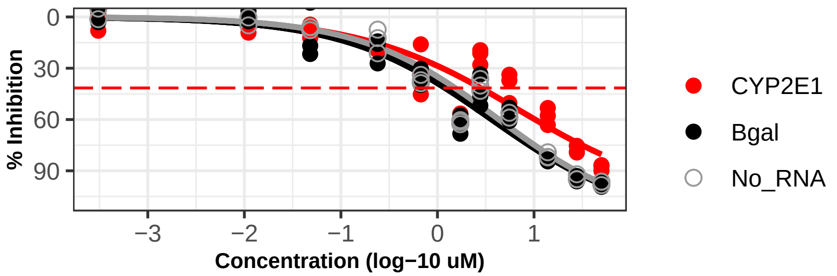

Mestranol: CYP2C8

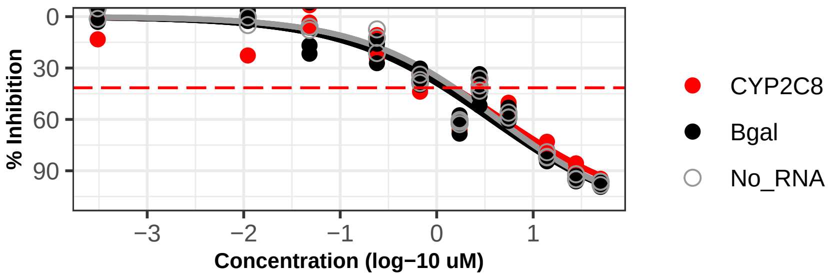

Mestranol: CYP2J2

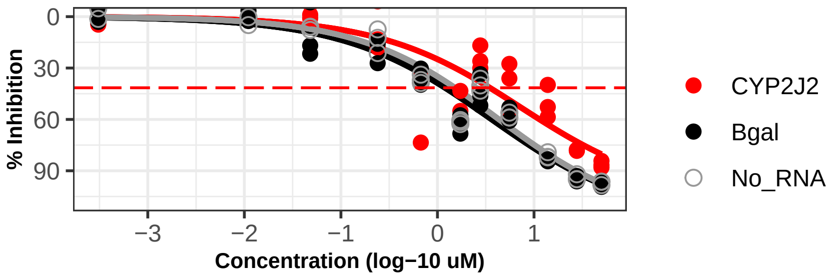

Mestranol: CYP2C9

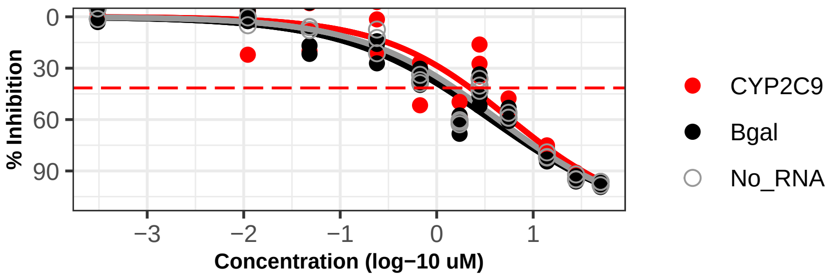

Mestranol: CYP3A4

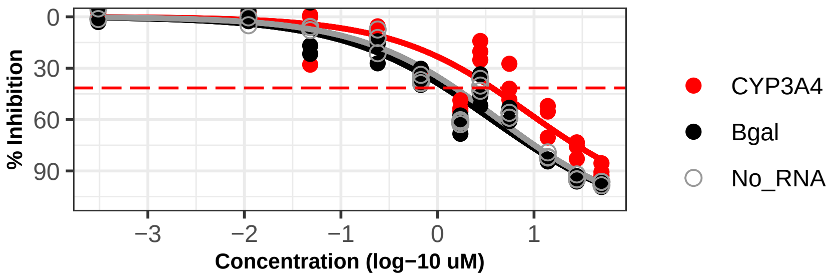

Metalaxyl: CYP1A2

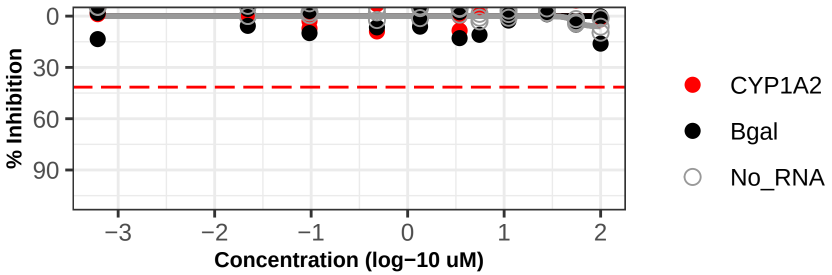

Metalaxyl: CYP2C19

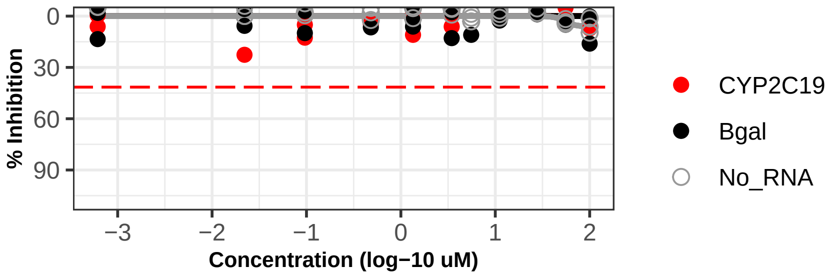

Metalaxyl: CYP2A6

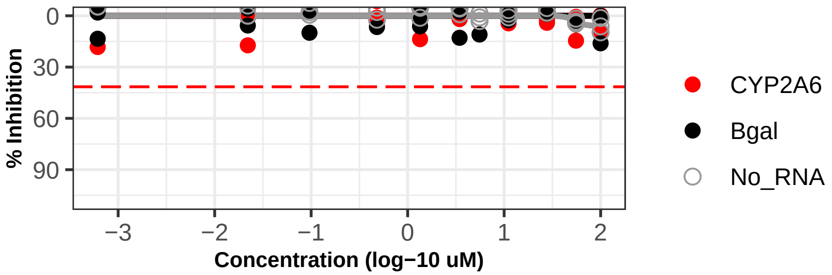

Metalaxyl: CYP2D6

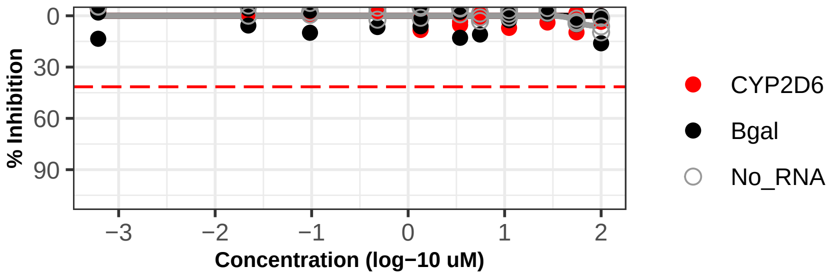

Metalaxyl: CYP2B6

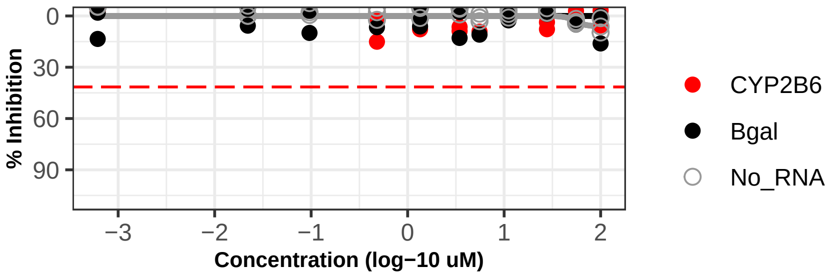

Metalaxyl: CYP2E1

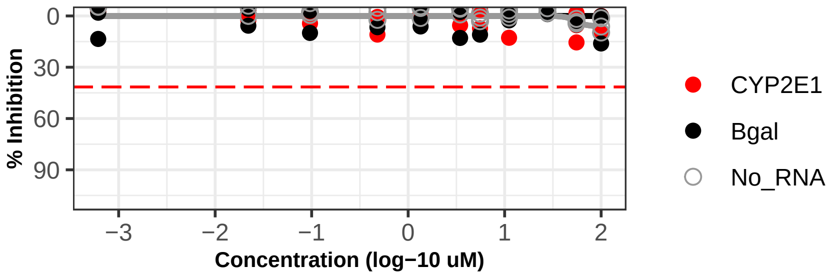

Metalaxyl: CYP2C8

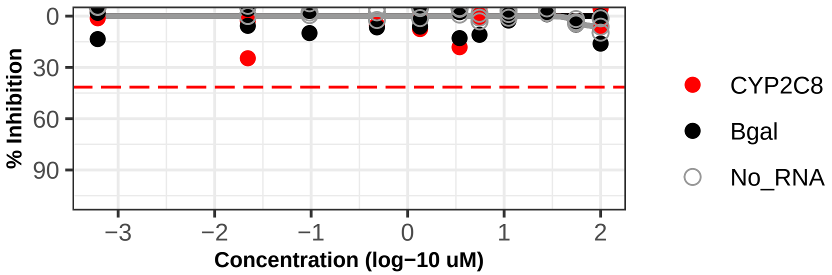

Metalaxyl: CYP2J2

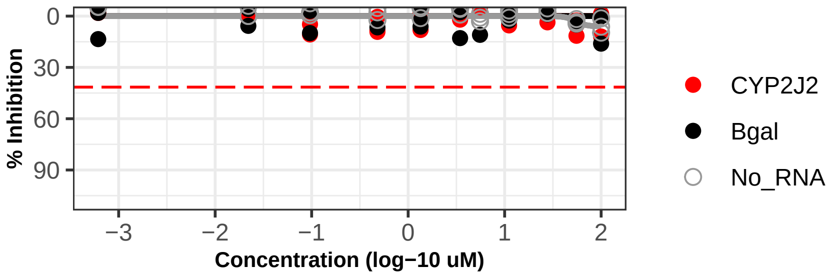

Metalaxyl: CYP2C9

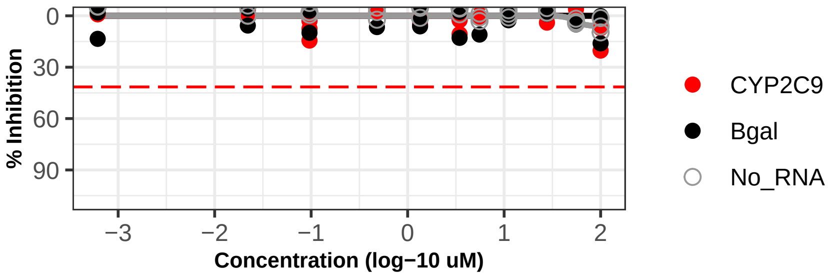

Metalaxyl: CYP3A4

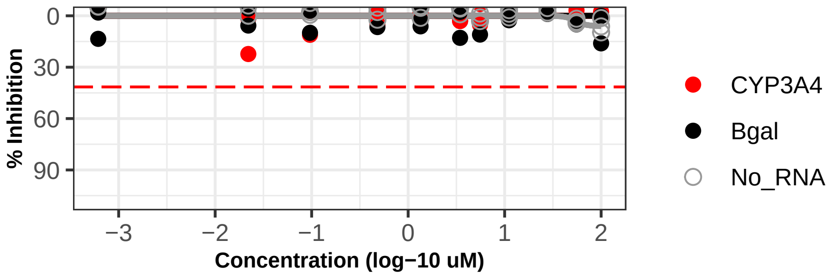

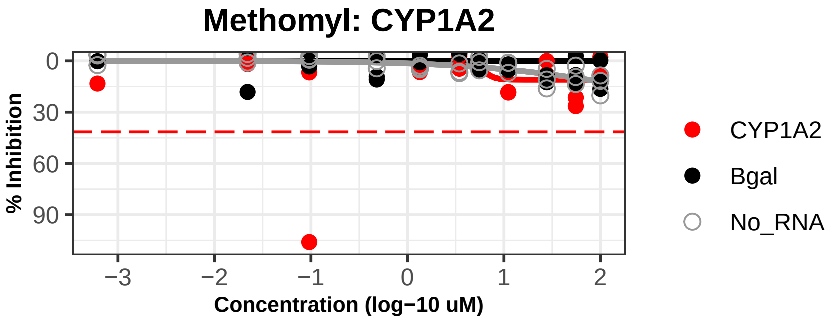
Methomyl: CYP1A2
% Inhibition
0
30
60
90
−3
−2
−1
0
1
2
Concentration (log−10 uM)
CYP1A2
Bgal
No_RNA

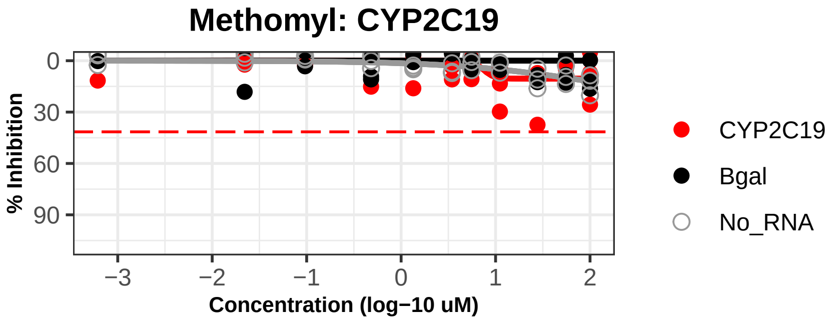
Methomyl: CYP2C19
% Inhibition
0
30
60
90
−3
−2
−1
0
1
2
Concentration (log−10 uM)
CYP2C19
Bgal
No_RNA

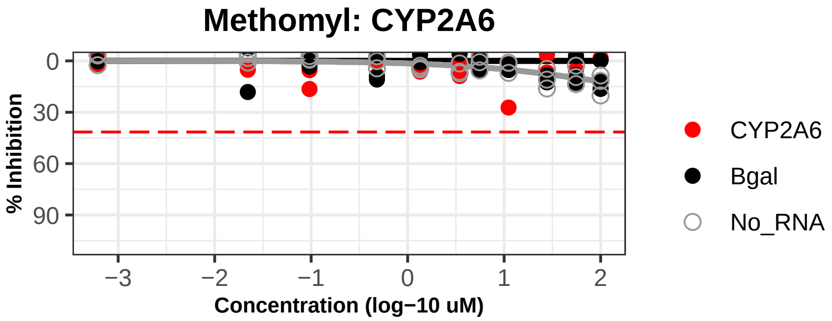
Methomyl: CYP2A6
% Inhibition
0
30
60
90
−3
−2
−1
0
1
2
Concentration (log−10 uM)
CYP2A6
Bgal
No_RNA

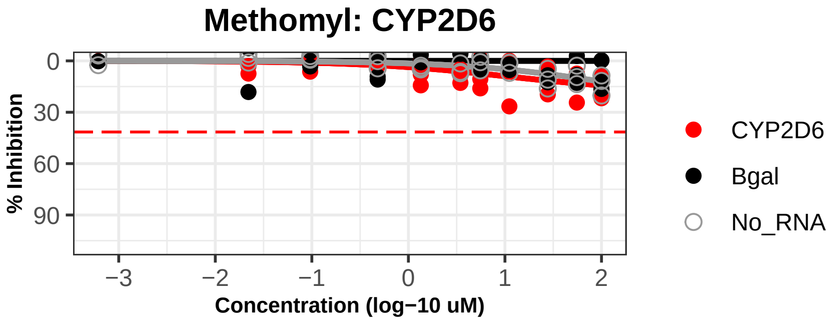
Methomyl: CYP2D6
% Inhibition
0
30
60
90
−3
−2
−1
0
1
2
Concentration (log−10 uM)
CYP2D6
Bgal
No_RNA

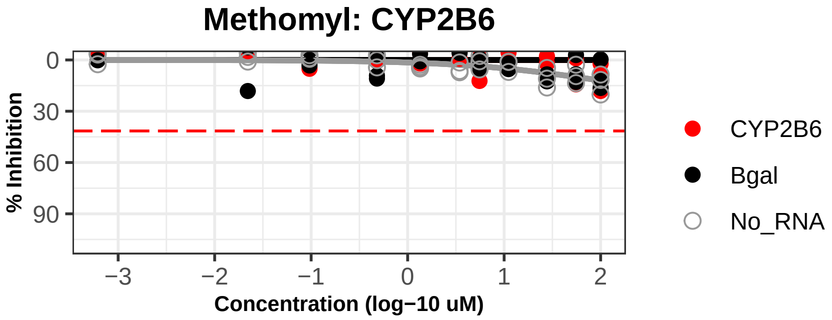
Methomyl: CYP2B6
% Inhibition
0
30
60
90
−3
−2
−1
0
1
2
Concentration (log−10 uM)
CYP2B6
Bgal
No_RNA

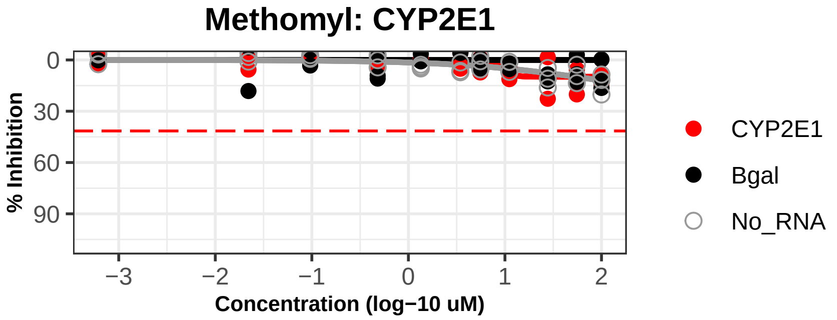
Methomyl: CYP2E1
% Inhibition
0
30
60
90
−3
−2
−1
0
1
2
Concentration (log−10 uM)
CYP2E1
Bgal
No_RNA

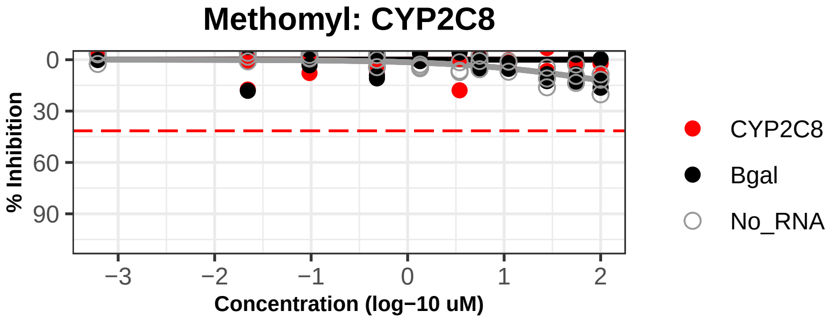
Methomyl: CYP2C8
% Inhibition
0
30
60
90
−3
−2
−1
0
1
2
Concentration (log−10 uM)
CYP2C8
Bgal
No_RNA

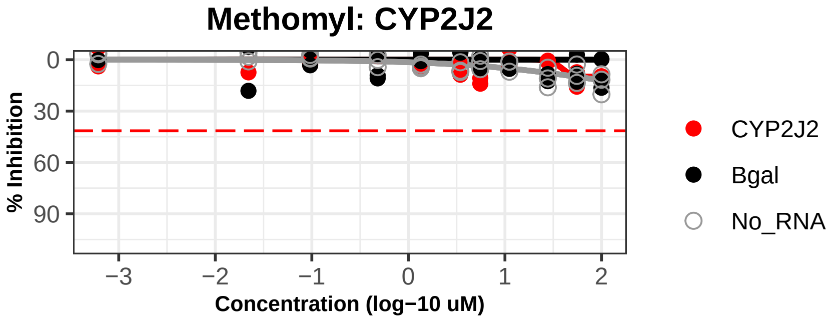
Methomyl: CYP2J2
% Inhibition
0
30
60
90
−3
−2
−1
0
1
2
Concentration (log−10 uM)
CYP2J2
Bgal
No_RNA

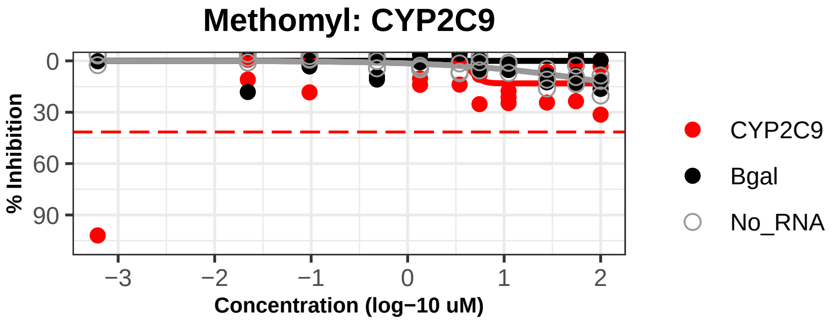
Methomyl: CYP2C9
% Inhibition
0
30
60
90
−3
−2
−1
0
1
2
Concentration (log−10 uM)
CYP2C9
Bgal
No_RNA

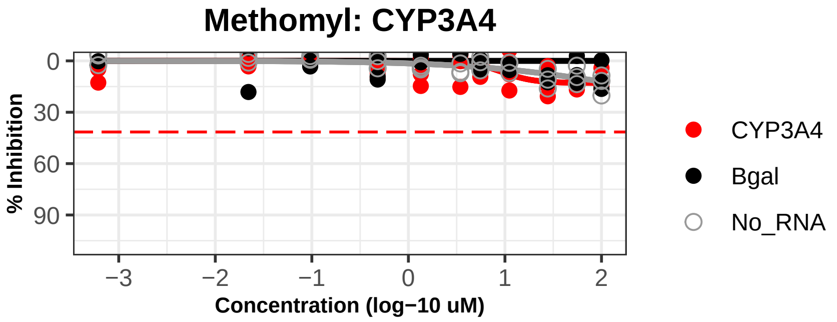
Methomyl: CYP3A4
% Inhibition
0
30
60
90
−3
−2
−1
0
1
2
Concentration (log−10 uM)
CYP3A4
Bgal
No_RNA

Methoxychlor: CYP1A2

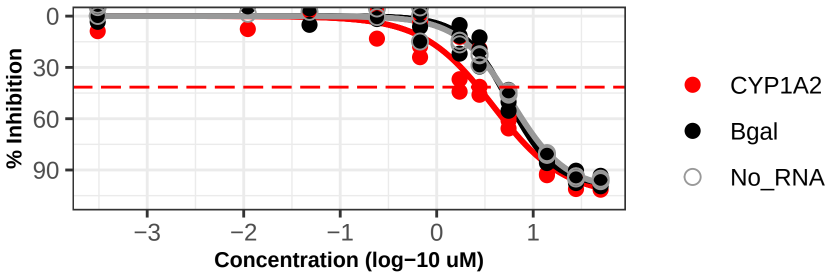

Methoxychlor: CYP2C19

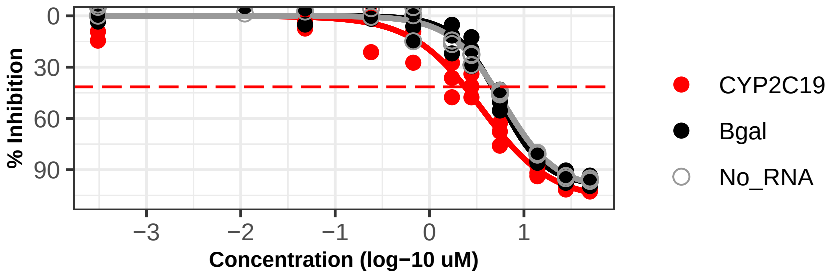

Methoxychlor: CYP2A6

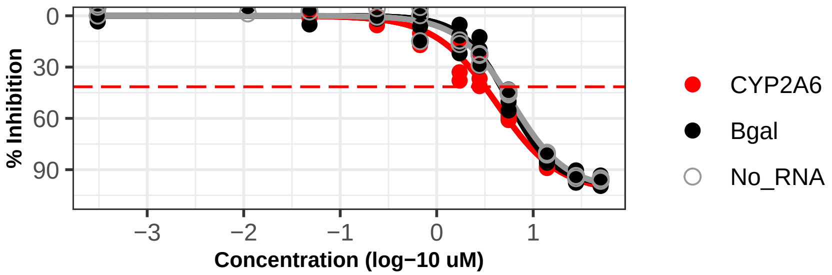

Methoxychlor: CYP2D6

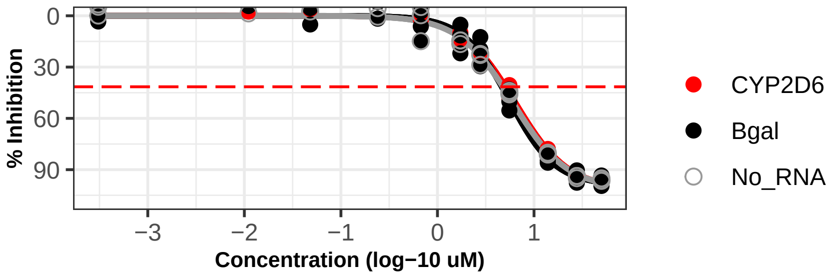

Methoxychlor: CYP2B6

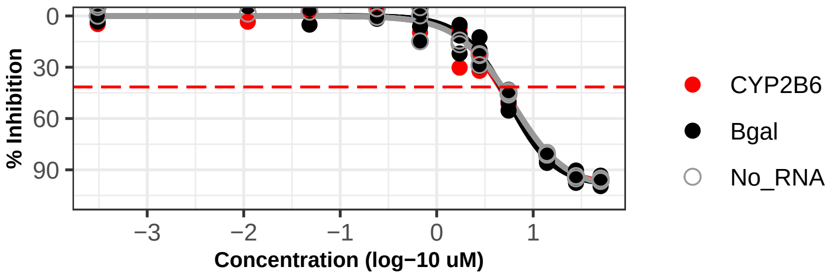

Methoxychlor: CYP2E1

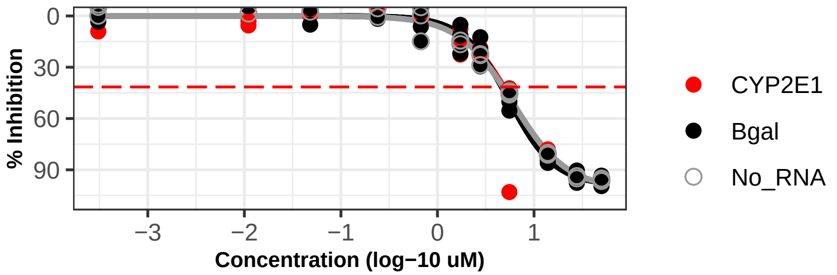

Methoxychlor: CYP2C8

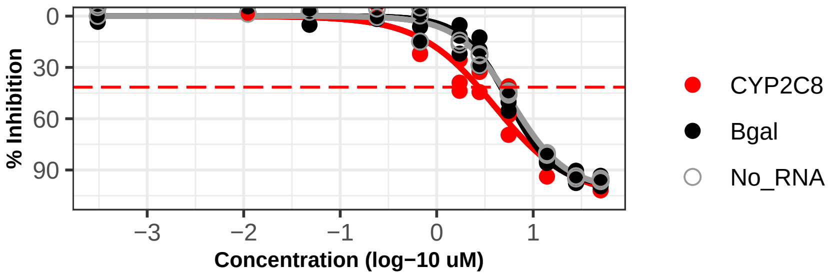

Methoxychlor: CYP2J2

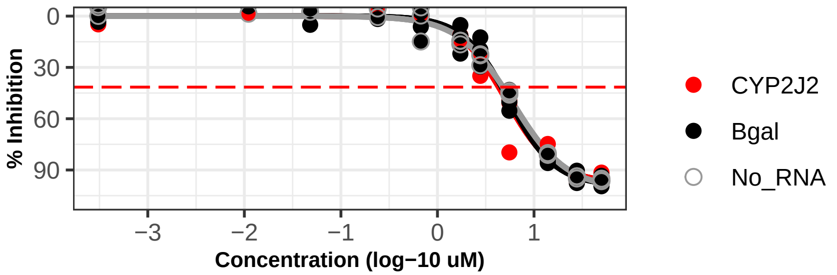

Methoxychlor: CYP2C9

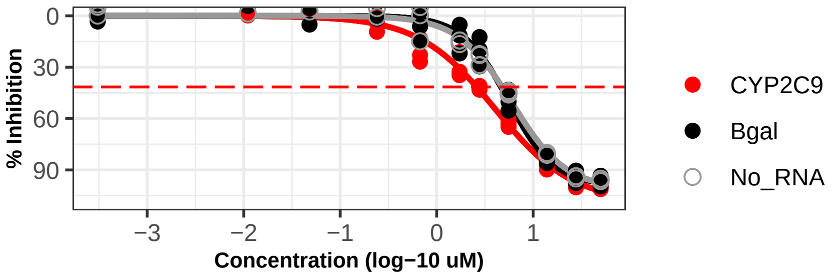

Methoxychlor: CYP3A4

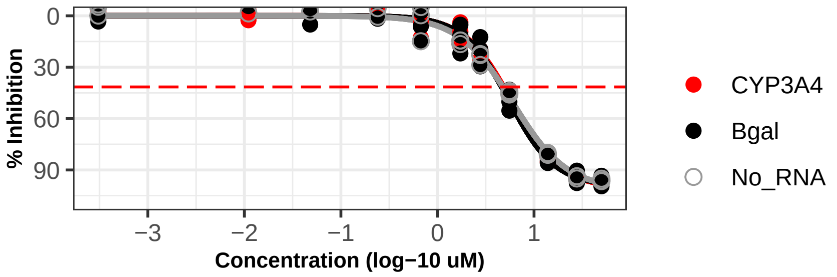

Metolachlor: CYP1A2

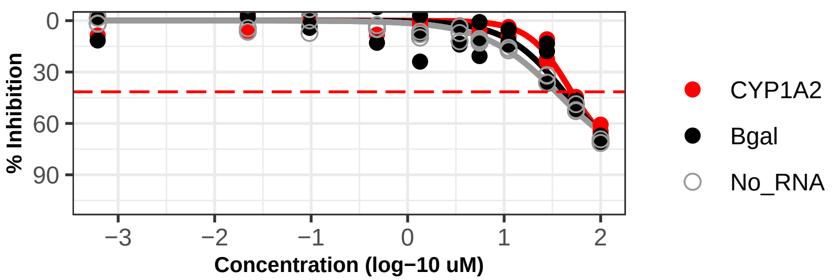

Metolachlor: CYP2C19

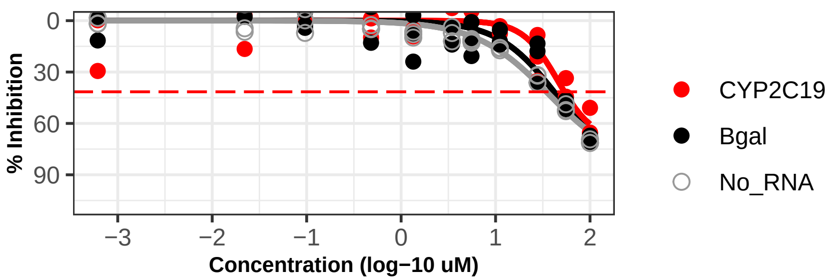

Metolachlor: CYP2A6

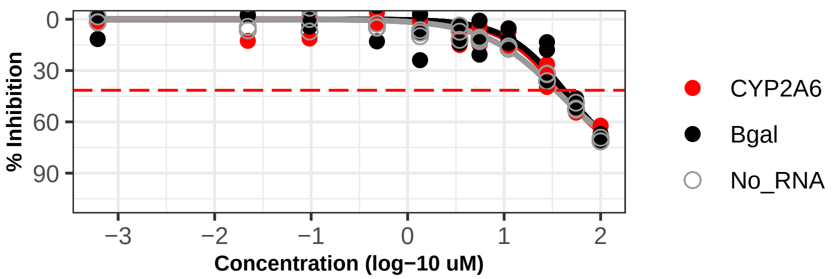

Metolachlor: CYP2D6

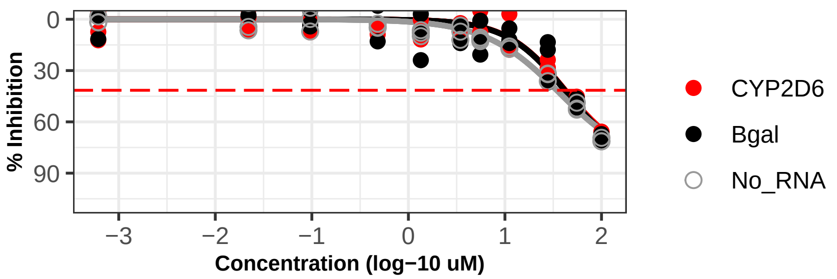

Metolachlor: CYP2B6

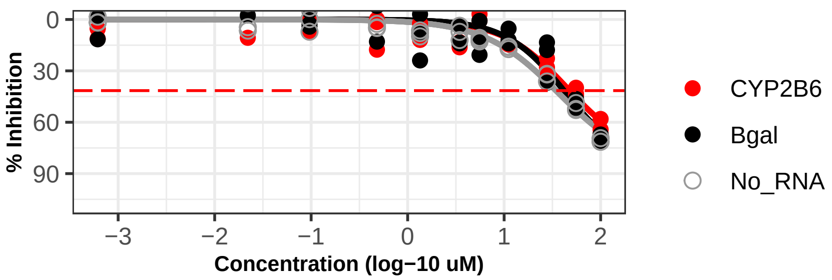

Metolachlor: CYP2E1

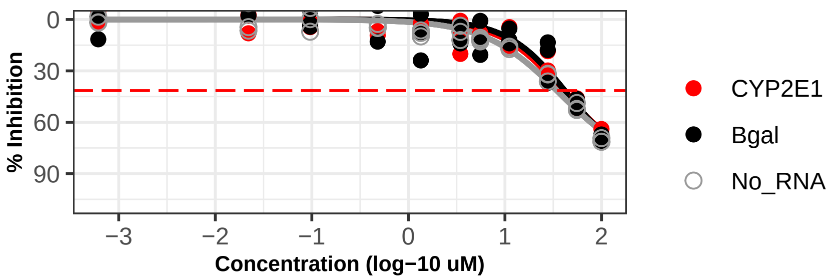

Metolachlor: CYP2C8

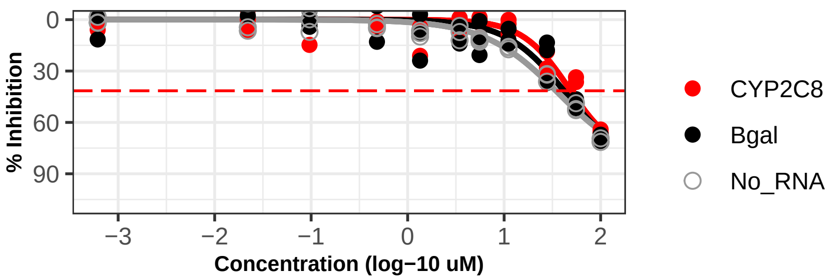

Metolachlor: CYP2J2

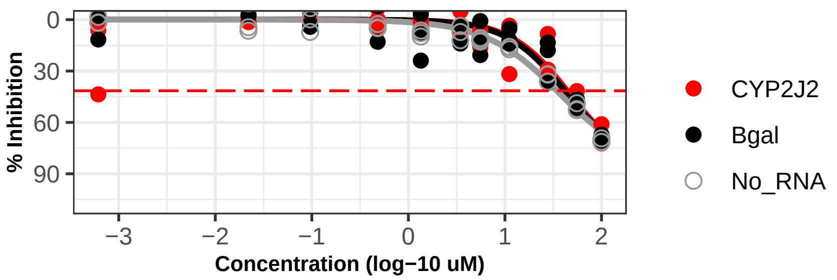

Metolachlor: CYP2C9

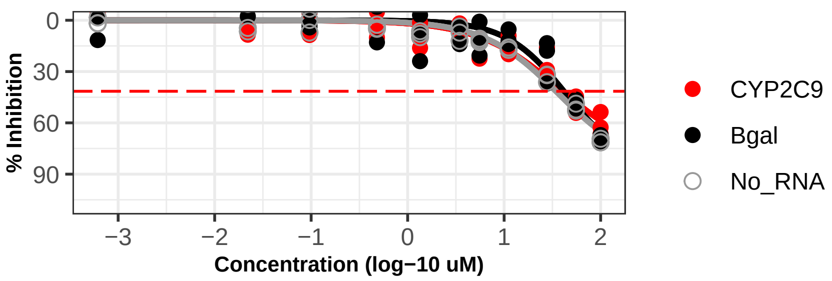

Metolachlor: CYP3A4

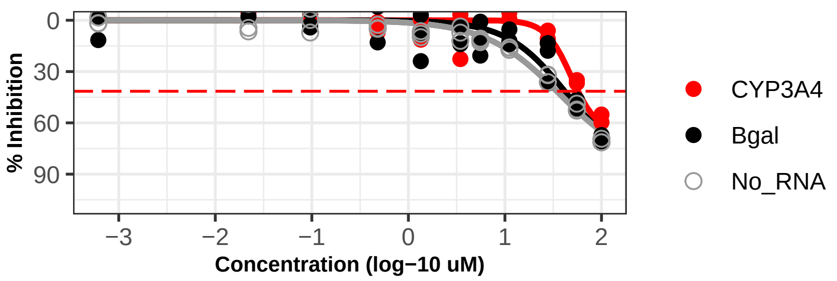

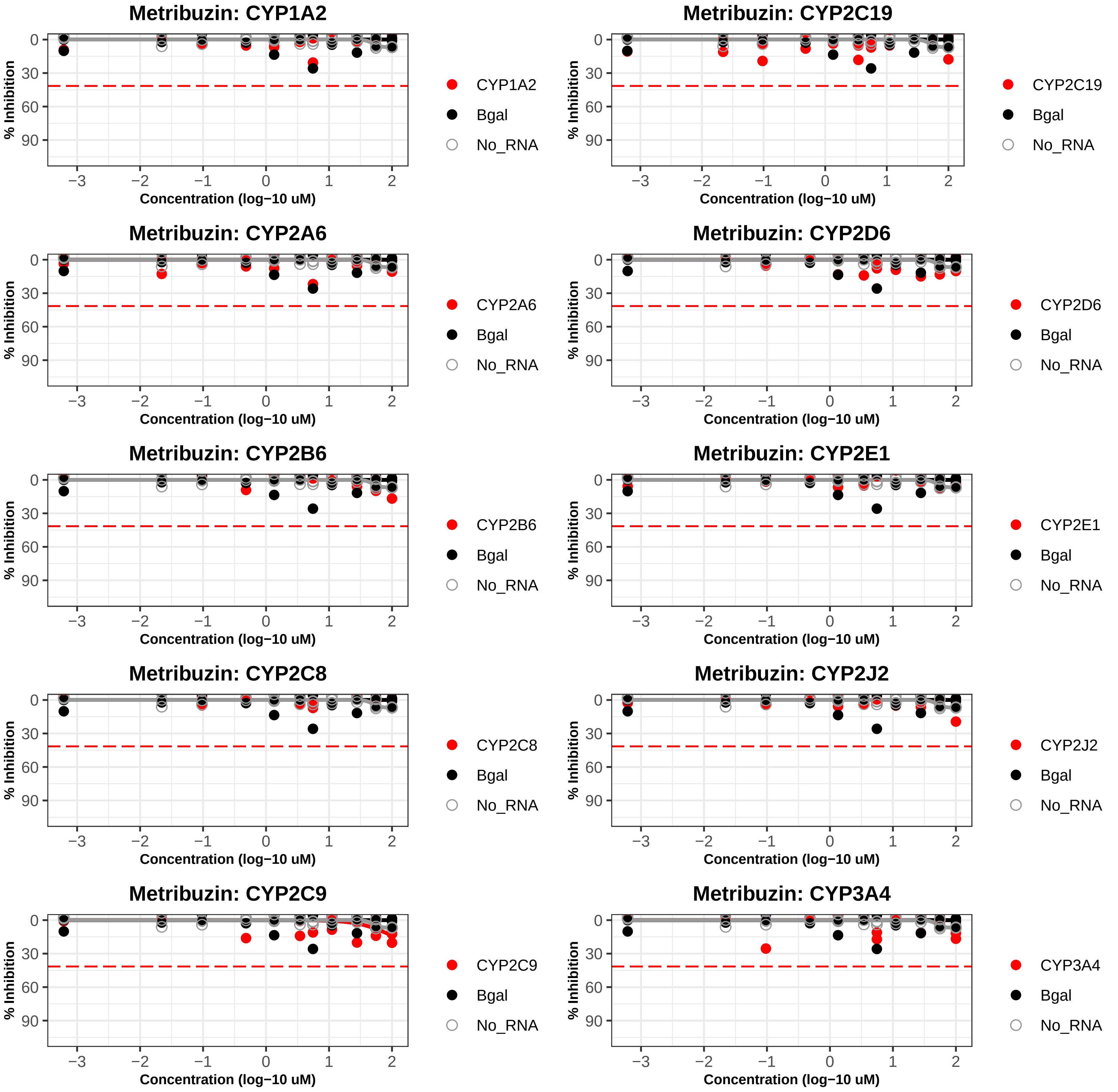
Metribuzin: CYP1A2
Metribuzin: CYP2C19
Metribuzin: CYP2A6
Metribuzin: CYP2D6
Metribuzin: CYP2B6
Metribuzin: CYP2E1
Metribuzin: CYP2C8
Metribuzin: CYP2J2
Metribuzin: CYP2C9
Metribuzin: CYP3A4
% Inhibition
Concentration (log−10 uM)
CYP1A2
CYP2C19
CYP2A6
CYP2D6
CYP2B6
CYP2E1
CYP2C8
CYP2J2
CYP2C9
CYP3A4
Bgal
No_RNA

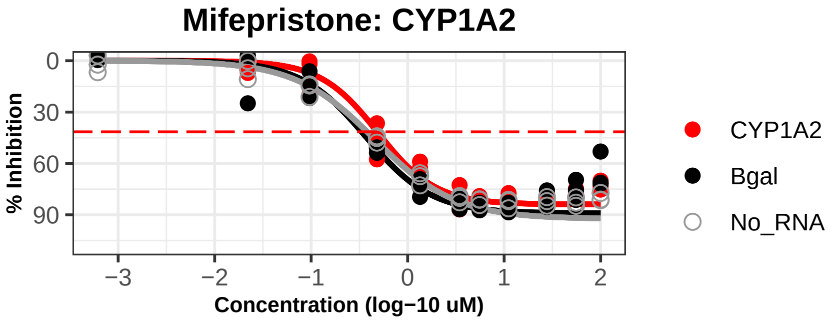
Mifepristone: CYP1A2
% Inhibition
Concentration (log−10 uM)
CYP1A2
Bgal
No_RNA

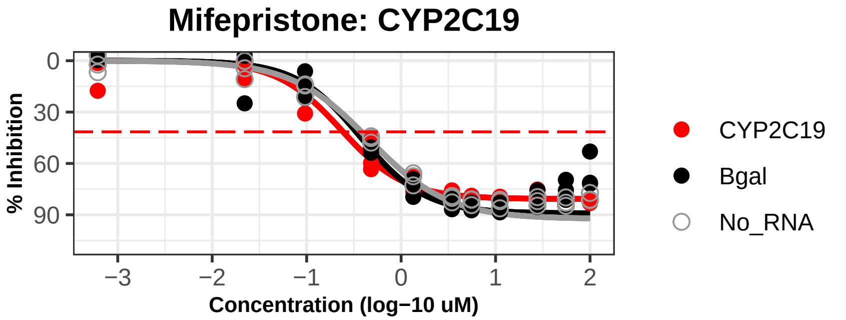
Mifepristone: CYP2C19
% Inhibition
Concentration (log−10 uM)
CYP2C19
Bgal
No_RNA

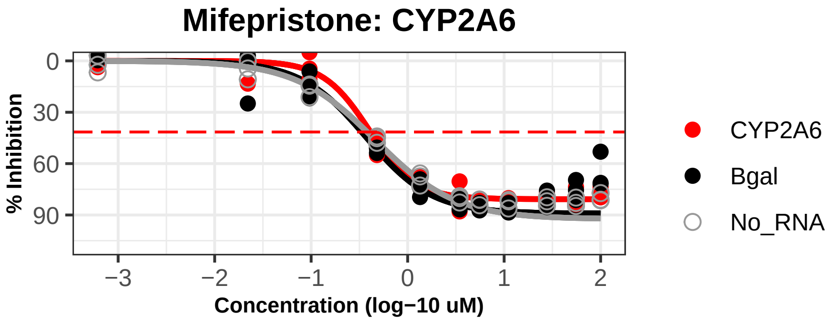
Mifepristone: CYP2A6
% Inhibition
Concentration (log−10 uM)
CYP2A6
Bgal
No_RNA

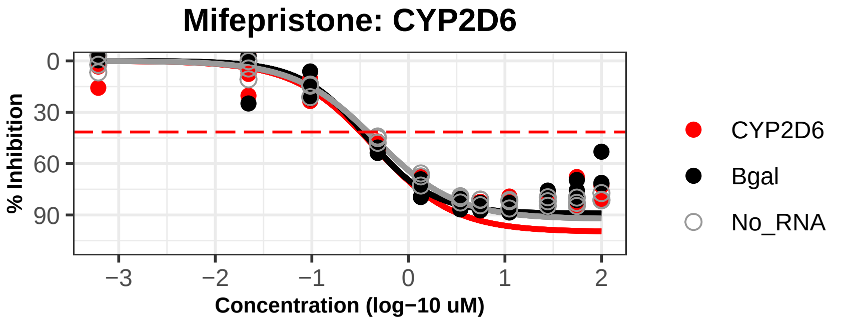
Mifepristone: CYP2D6
% Inhibition
Concentration (log−10 uM)
CYP2D6
Bgal
No_RNA

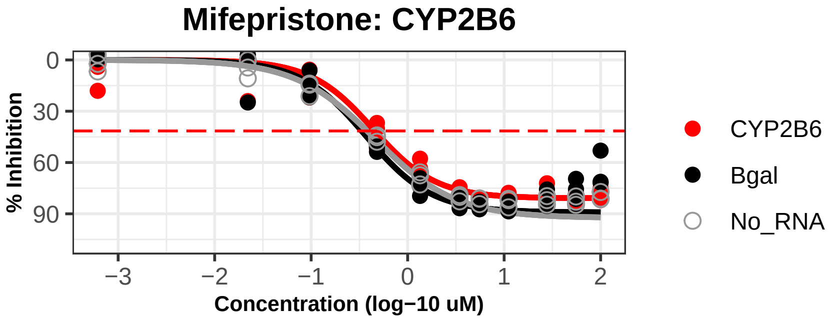
Mifepristone: CYP2B6
% Inhibition
Concentration (log−10 uM)
CYP2B6
Bgal
No_RNA

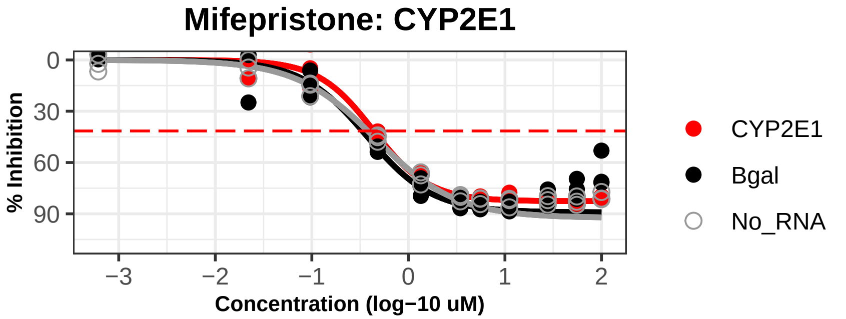
Mifepristone: CYP2E1
% Inhibition
Concentration (log−10 uM)
CYP2E1
Bgal
No_RNA

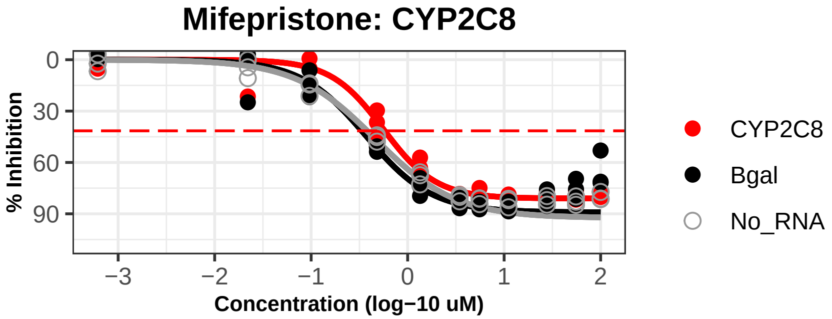
Mifepristone: CYP2C8
% Inhibition
Concentration (log−10 uM)
CYP2C8
Bgal
No_RNA

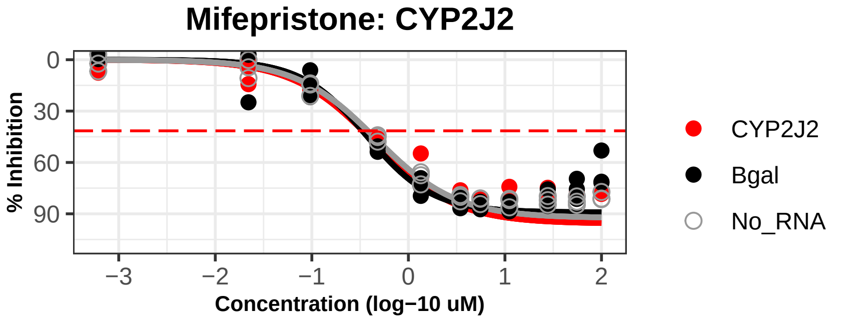
Mifepristone: CYP2J2
% Inhibition
Concentration (log−10 uM)
CYP2J2
Bgal
No_RNA

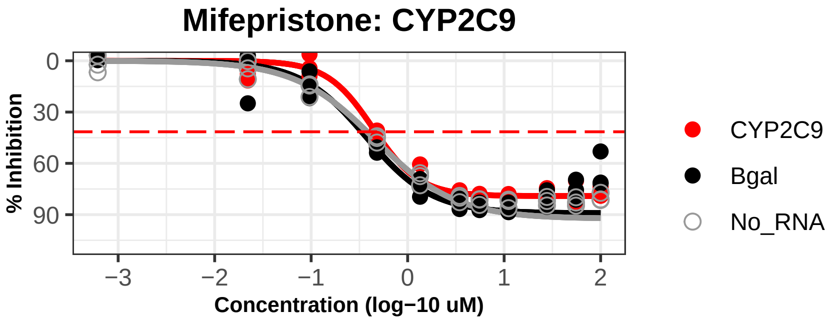
Mifepristone: CYP2C9
% Inhibition
Concentration (log−10 uM)
CYP2C9
Bgal
No_RNA

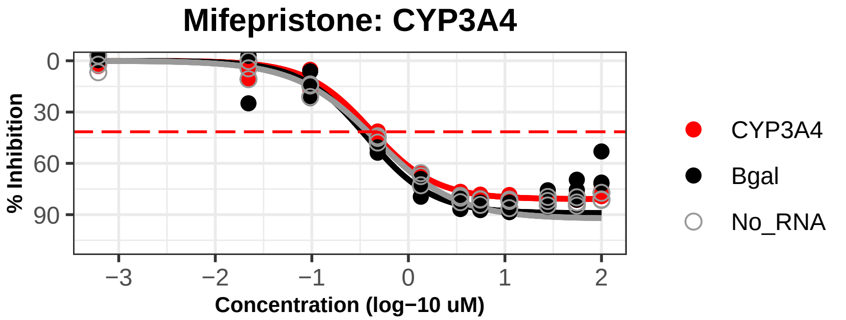
Mifepristone: CYP3A4
% Inhibition
Concentration (log−10 uM)
CYP3A4
Bgal
No_RNA

Mono(2−ethylhexyl) phthalate: CYP1A2

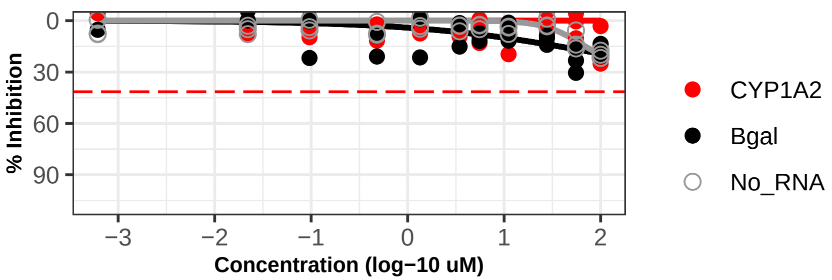

Mono(2−ethylhexyl) phthalate: CYP2C19

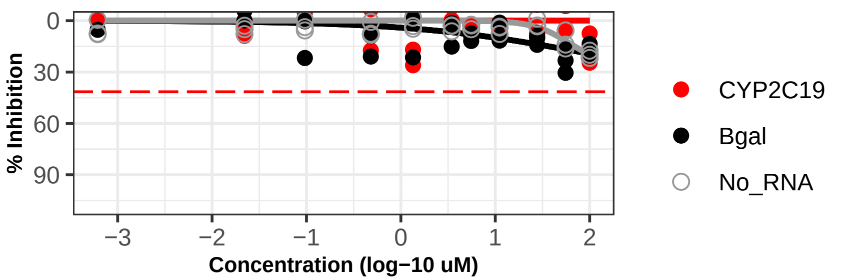

Mono(2−ethylhexyl) phthalate: CYP2A6

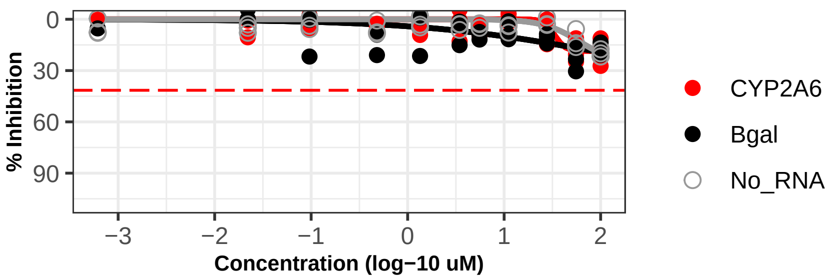

Mono(2−ethylhexyl) phthalate: CYP2D6

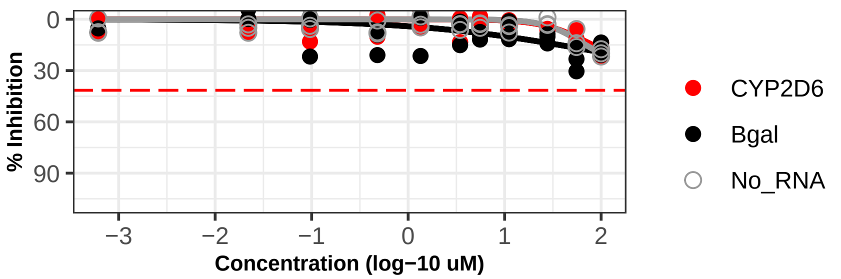

Mono(2−ethylhexyl) phthalate: CYP2B6

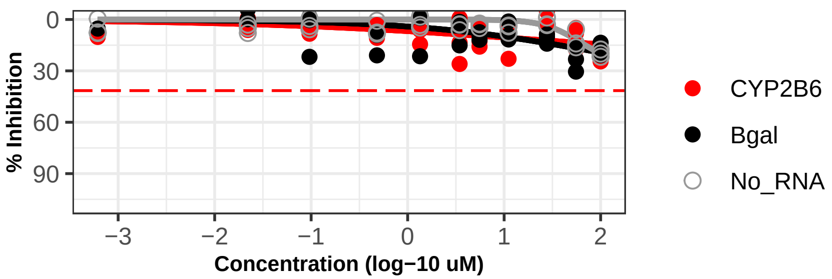

Mono(2−ethylhexyl) phthalate: CYP2E1

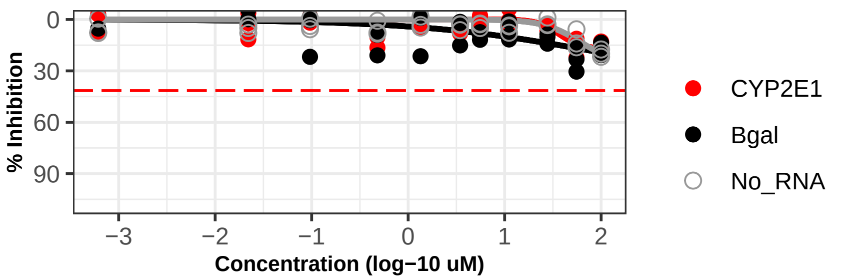

Mono(2−ethylhexyl) phthalate: CYP2C8

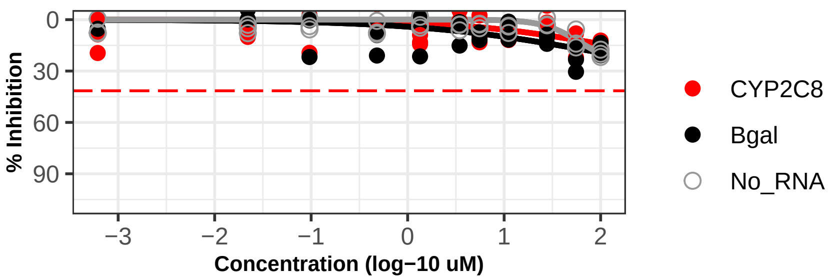

Mono(2−ethylhexyl) phthalate: CYP2J2

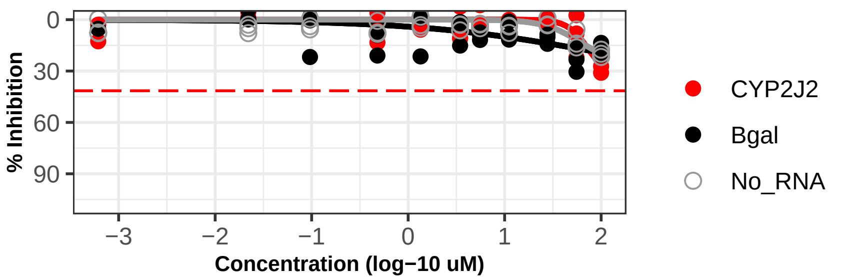

Mono(2−ethylhexyl) phthalate: CYP2C9

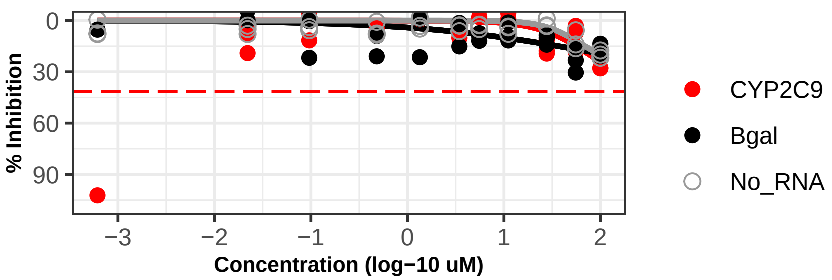

Mono(2−ethylhexyl) phthalate: CYP3A4

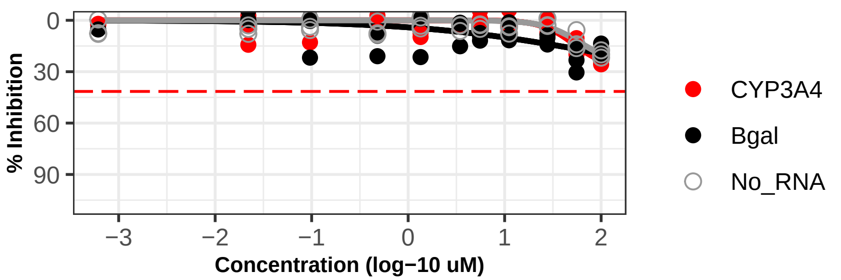

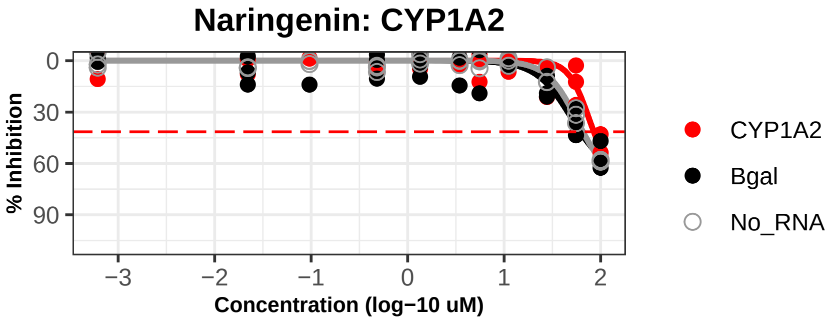
Naringenin: CYP1A2
% Inhibition
0
30
60
90
−3
−2
−1
0
1
2
Concentration (log−10 uM)
CYP1A2
Bgal
No_RNA

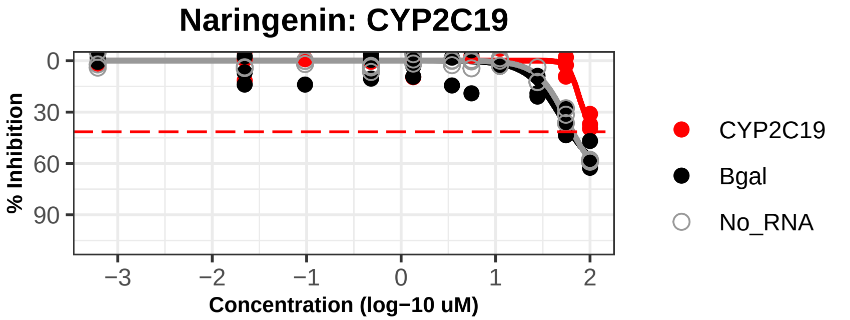
Naringenin: CYP2C19
% Inhibition
0
30
60
90
−3
−2
−1
0
1
2
Concentration (log−10 uM)
CYP2C19
Bgal
No_RNA

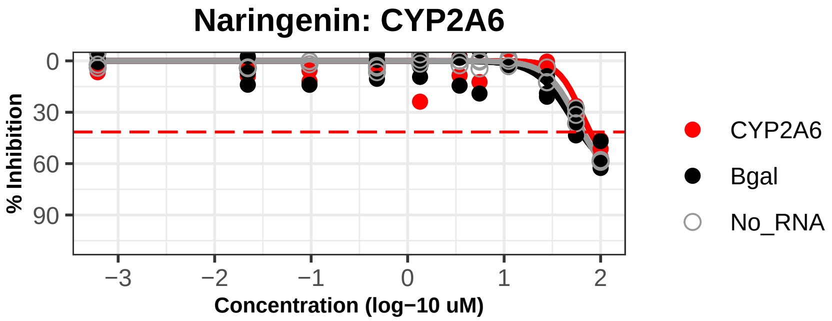
Naringenin: CYP2A6
% Inhibition
0
30
60
90
−3
−2
−1
0
1
2
Concentration (log−10 uM)
CYP2A6
Bgal
No_RNA

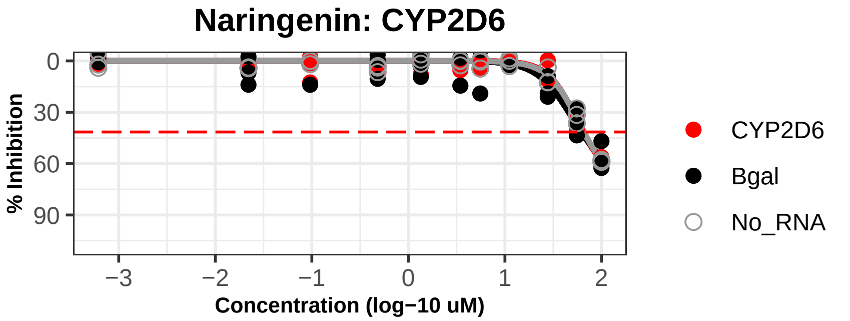
Naringenin: CYP2D6
% Inhibition
0
30
60
90
−3
−2
−1
0
1
2
Concentration (log−10 uM)
CYP2D6
Bgal
No_RNA

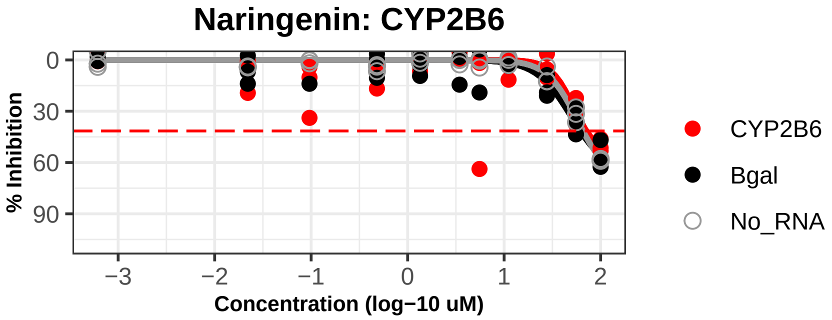
Naringenin: CYP2B6
% Inhibition
0
30
60
90
−3
−2
−1
0
1
2
Concentration (log−10 uM)
CYP2B6
Bgal
No_RNA

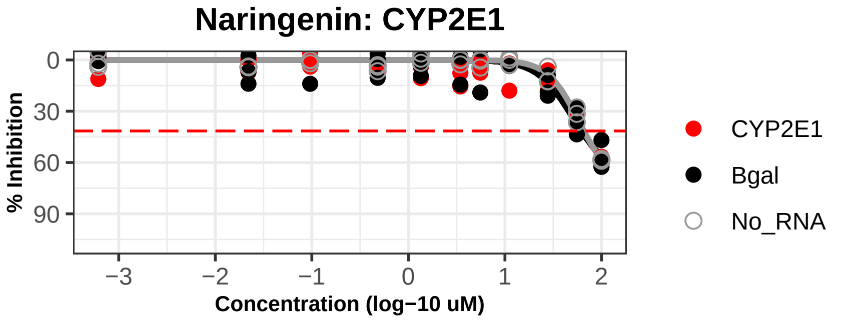
Naringenin: CYP2E1
% Inhibition
0
30
60
90
−3
−2
−1
0
1
2
Concentration (log−10 uM)
CYP2E1
Bgal
No_RNA

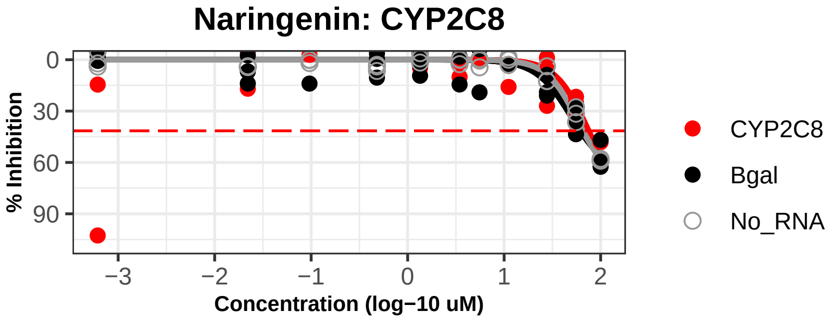
Naringenin: CYP2C8
% Inhibition
0
30
60
90
−3
−2
−1
0
1
2
Concentration (log−10 uM)
CYP2C8
Bgal
No_RNA

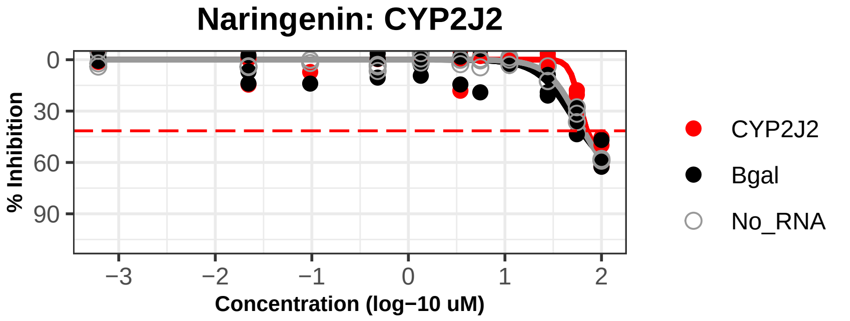
Naringenin: CYP2J2
% Inhibition
0
30
60
90
−3
−2
−1
0
1
2
Concentration (log−10 uM)
CYP2J2
Bgal
No_RNA

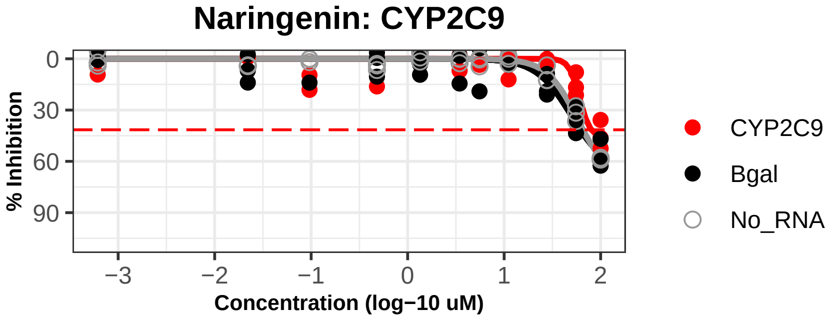
Naringenin: CYP2C9
% Inhibition
0
30
60
90
−3
−2
−1
0
1
2
Concentration (log−10 uM)
CYP2C9
Bgal
No_RNA

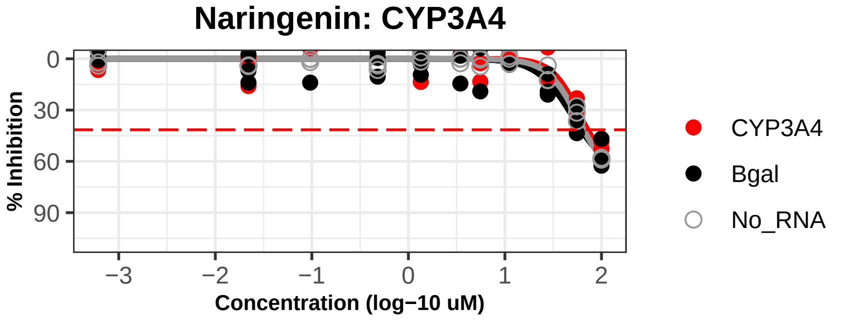
Naringenin: CYP3A4
% Inhibition
0
30
60
90
−3
−2
−1
0
1
2
Concentration (log−10 uM)
CYP3A4
Bgal
No_RNA

Nilutamide: CYP1A2

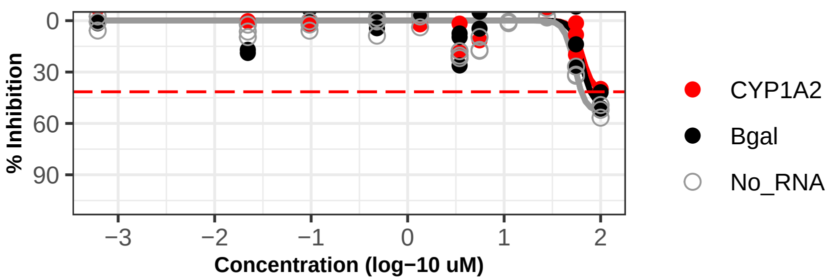

Nilutamide: CYP2C19

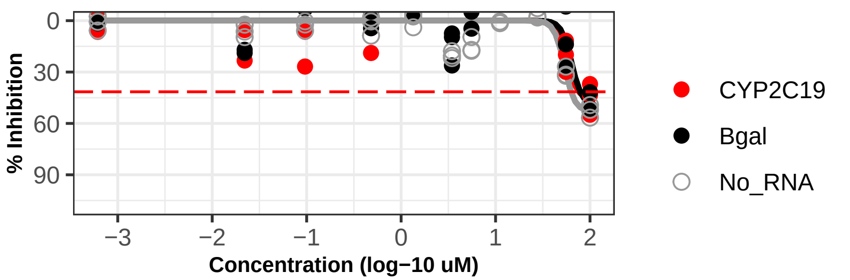

Nilutamide: CYP2A6

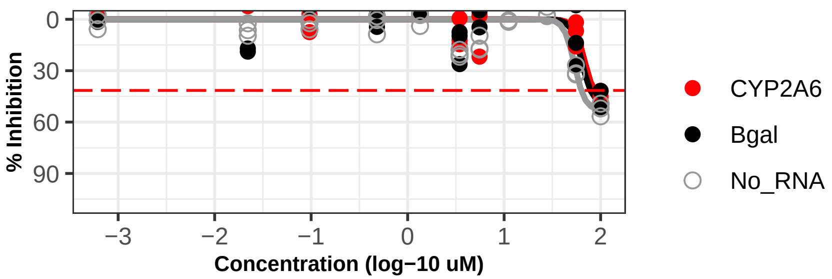

Nilutamide: CYP2D6

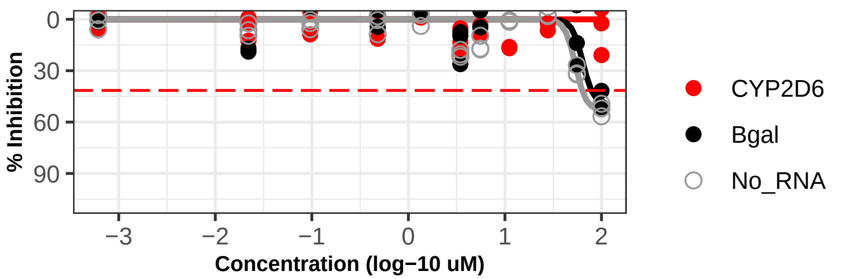

Nilutamide: CYP2B6

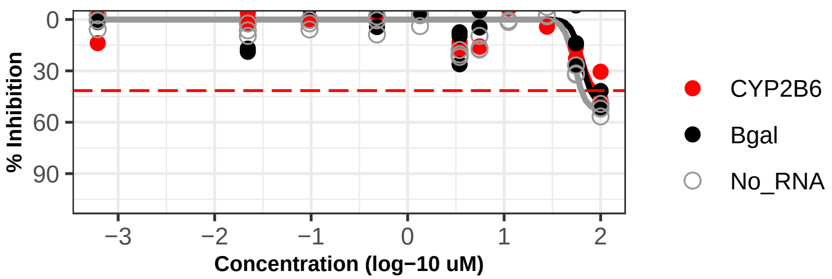

Nilutamide: CYP2E1

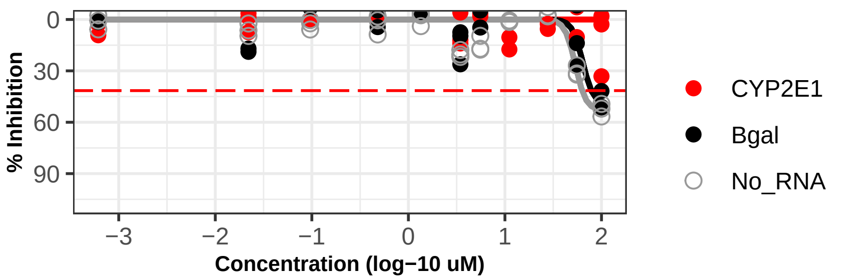

Nilutamide: CYP2C8

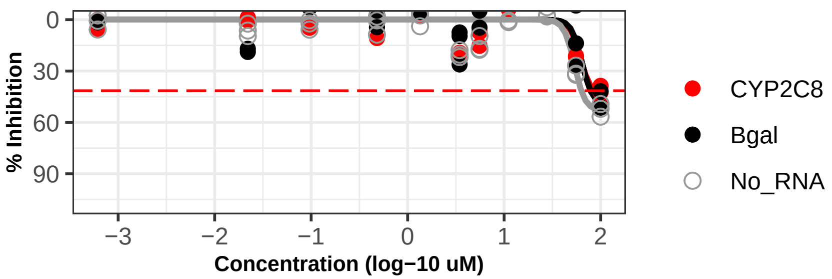

Nilutamide: CYP2J2

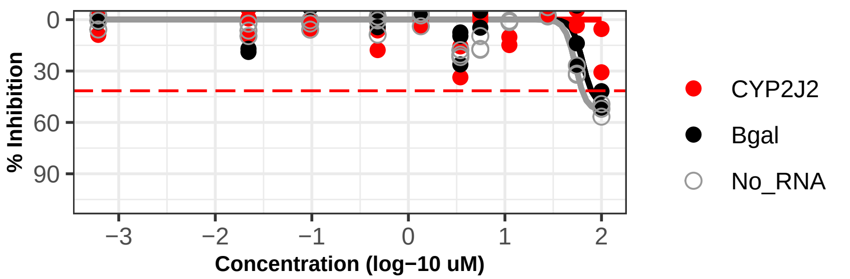

Nilutamide: CYP2C9

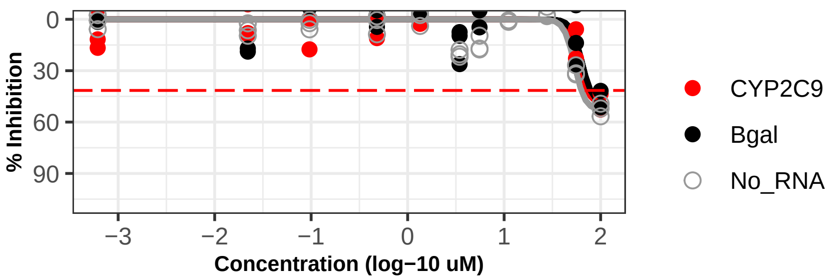

Nilutamide: CYP3A4

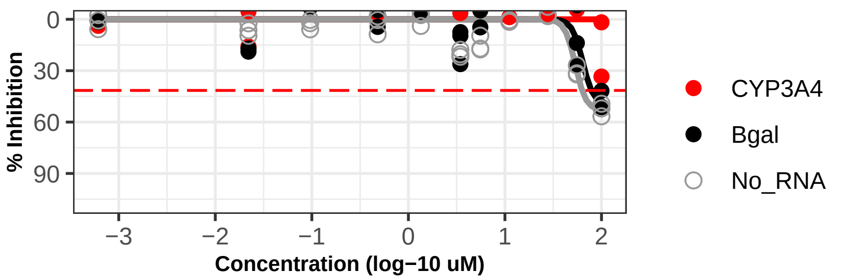

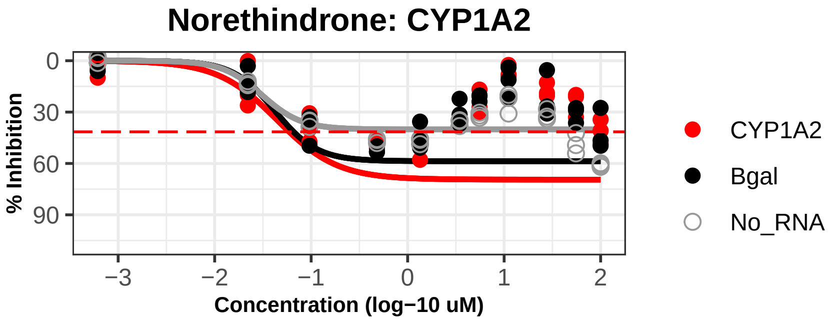
Norethindrone: CYP1A2
% Inhibition
0
30
60
90
−3
−2
−1
0
1
2
Concentration (log−10 uM)
CYP1A2
Bgal
No_RNA

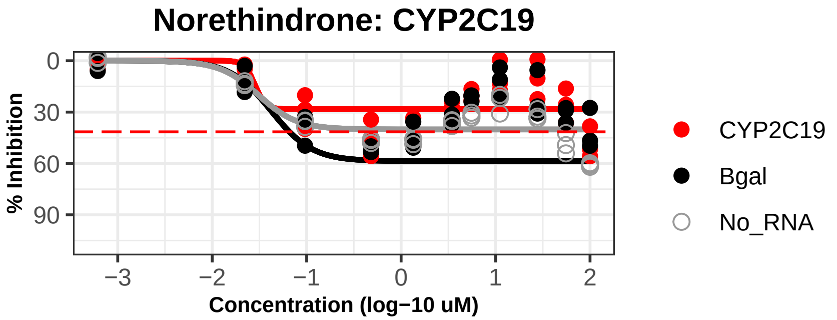
Norethindrone: CYP2C19
% Inhibition
0
30
60
90
−3
−2
−1
0
1
2
Concentration (log−10 uM)
CYP2C19
Bgal
No_RNA

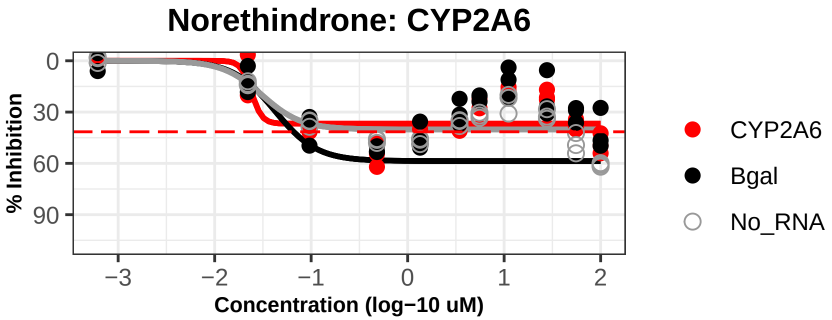
Norethindrone: CYP2A6
% Inhibition
0
30
60
90
−3
−2
−1
0
1
2
Concentration (log−10 uM)
CYP2A6
Bgal
No_RNA

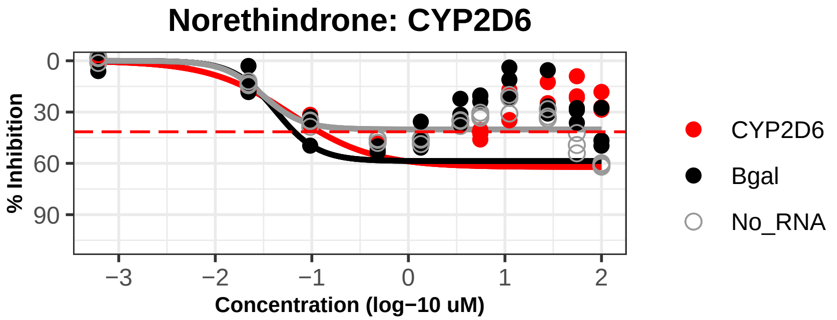
Norethindrone: CYP2D6
% Inhibition
0
30
60
90
−3
−2
−1
0
1
2
Concentration (log−10 uM)
CYP2D6
Bgal
No_RNA

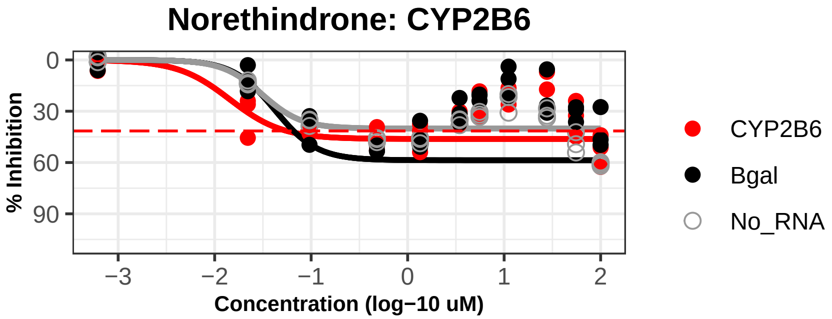
Norethindrone: CYP2B6
% Inhibition
0
30
60
90
−3
−2
−1
0
1
2
Concentration (log−10 uM)
CYP2B6
Bgal
No_RNA

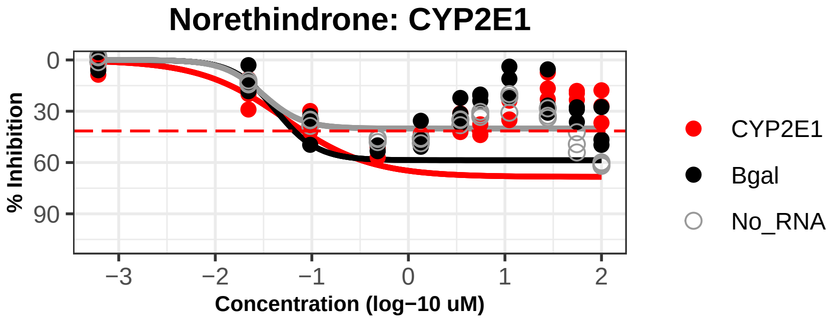
Norethindrone: CYP2E1
% Inhibition
0
30
60
90
−3
−2
−1
0
1
2
Concentration (log−10 uM)
CYP2E1
Bgal
No_RNA

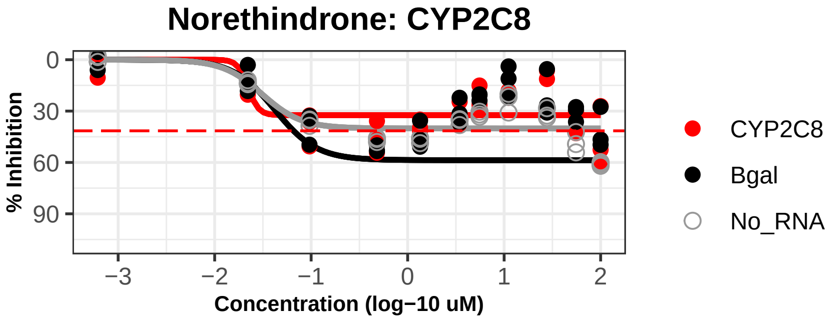
Norethindrone: CYP2C8
% Inhibition
0
30
60
90
−3
−2
−1
0
1
2
Concentration (log−10 uM)
CYP2C8
Bgal
No_RNA

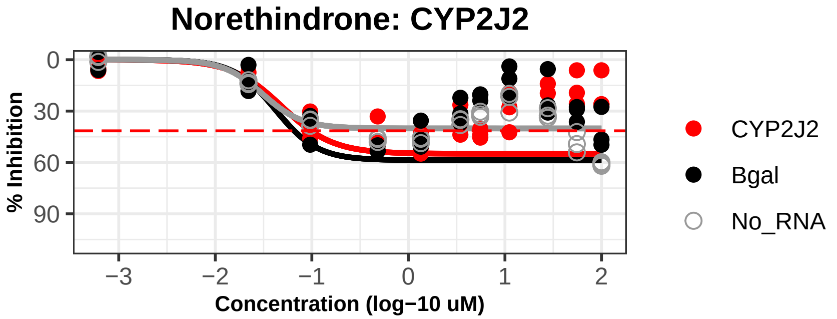
Norethindrone: CYP2J2
% Inhibition
0
30
60
90
−3
−2
−1
0
1
2
Concentration (log−10 uM)
CYP2J2
Bgal
No_RNA

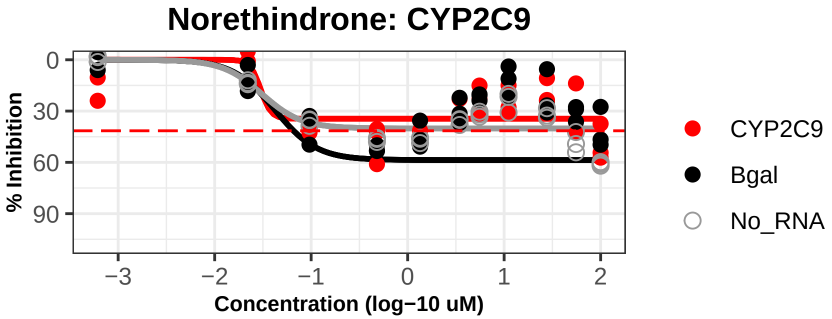
Norethindrone: CYP2C9
% Inhibition
0
30
60
90
−3
−2
−1
0
1
2
Concentration (log−10 uM)
CYP2C9
Bgal
No_RNA

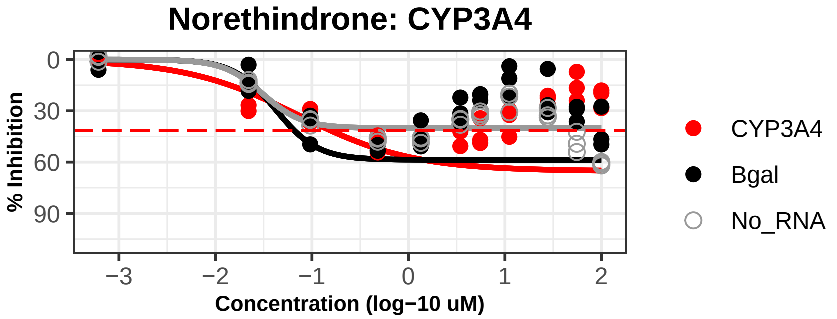
Norethindrone: CYP3A4
% Inhibition
0
30
60
90
−3
−2
−1
0
1
2
Concentration (log−10 uM)
CYP3A4
Bgal
No_RNA

Norflurazon: CYP1A2

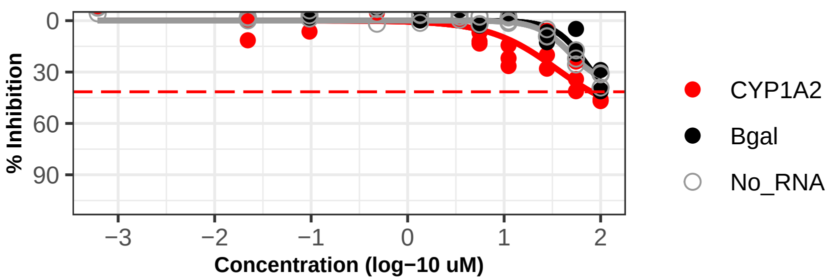

Norflurazon: CYP2C19

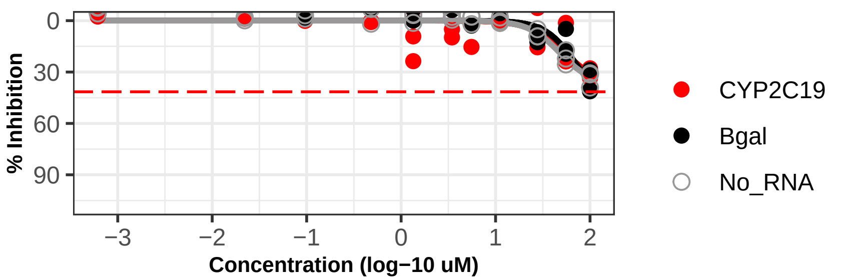

Norflurazon: CYP2A6

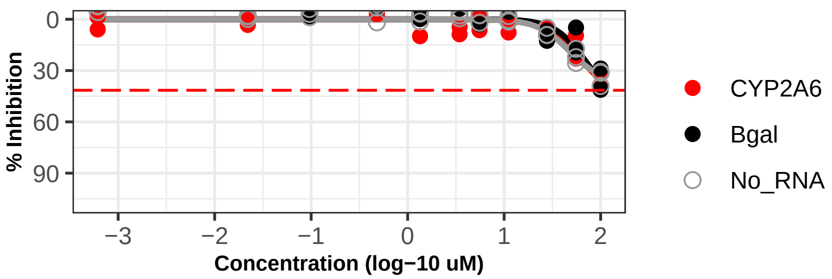

Norflurazon: CYP2D6

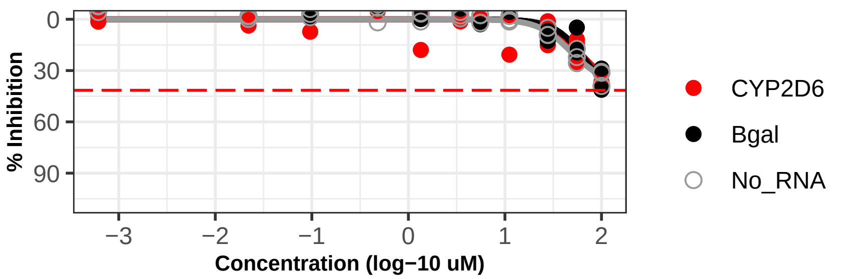

Norflurazon: CYP2B6

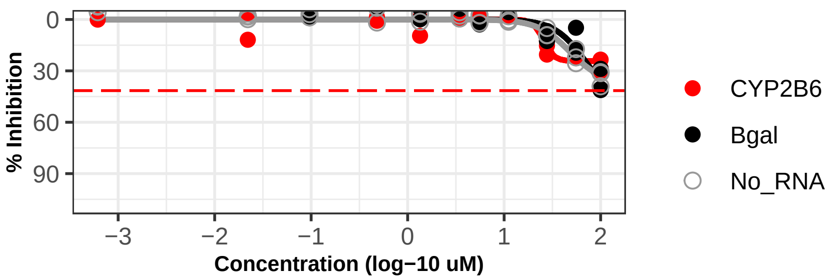

Norflurazon: CYP2E1

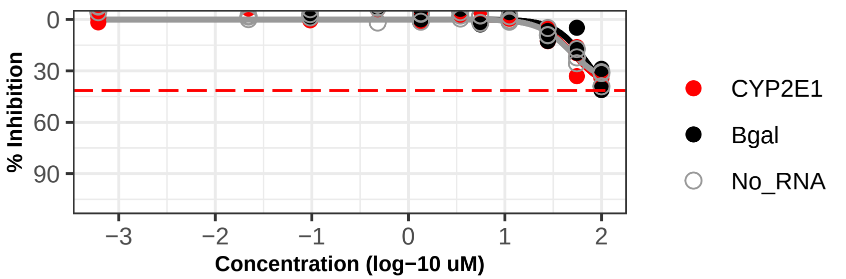

Norflurazon: CYP2C8

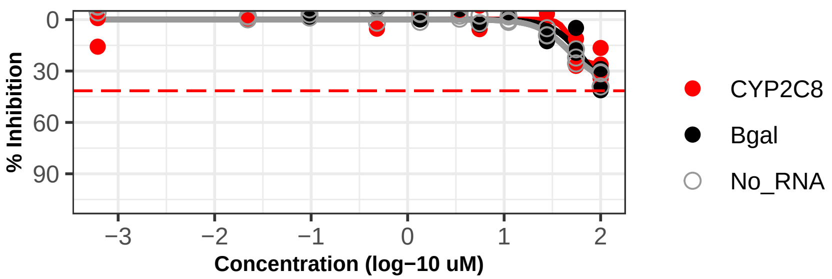

Norflurazon: CYP2J2

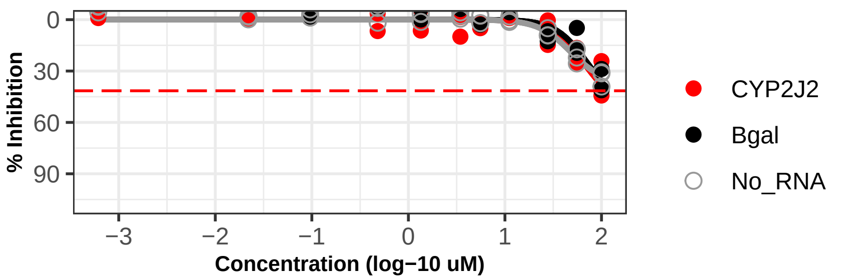

Norflurazon: CYP2C9

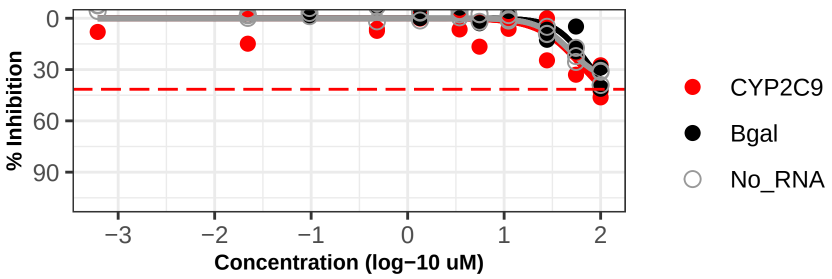

Norflurazon: CYP3A4

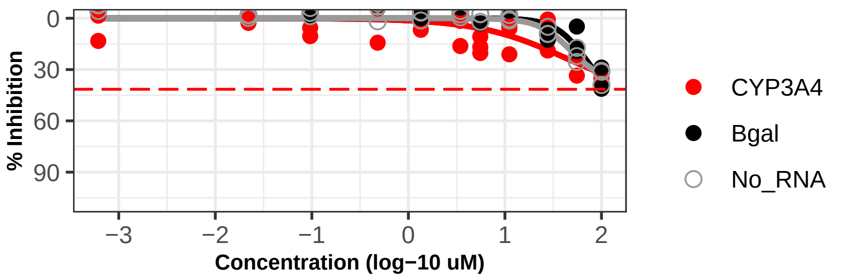

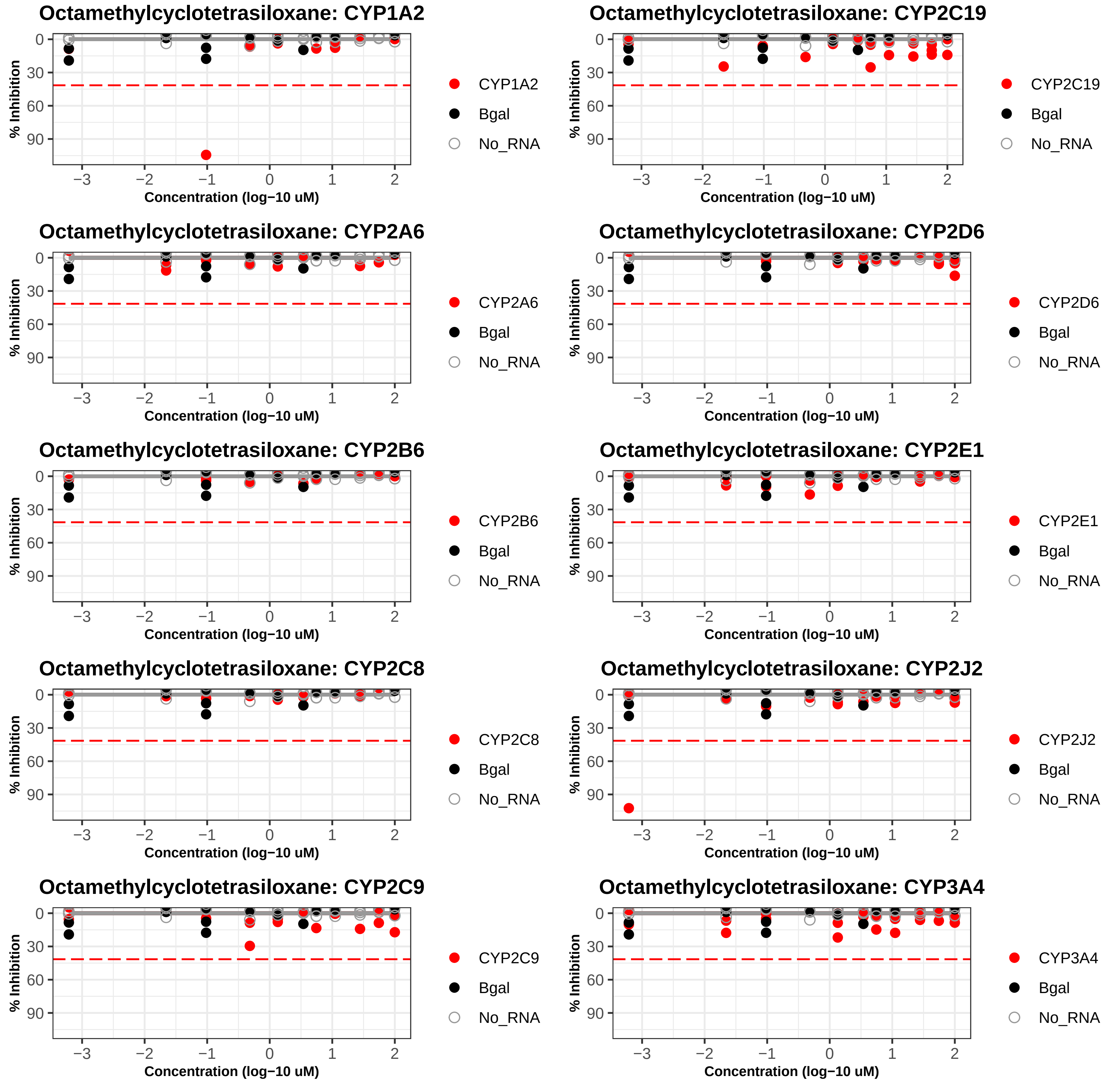
Octamethylcyclotetrasiloxane: CYP1A2
Octamethylcyclotetrasiloxane: CYP2C19
Octamethylcyclotetrasiloxane: CYP2A6
Octamethylcyclotetrasiloxane: CYP2D6
Octamethylcyclotetrasiloxane: CYP2B6
Octamethylcyclotetrasiloxane: CYP2E1
Octamethylcyclotetrasiloxane: CYP2C8
Octamethylcyclotetrasiloxane: CYP2J2
Octamethylcyclotetrasiloxane: CYP2C9
Octamethylcyclotetrasiloxane: CYP3A4
% Inhibition
Concentration (log−10 uM)
CYP1A2
CYP2C19
CYP2A6
CYP2D6
CYP2B6
CYP2E1
CYP2C8
CYP2J2
CYP2C9
CYP3A4
Bgal
No_RNA

Octylbicycloheptenedicarboximide: CYP1A2

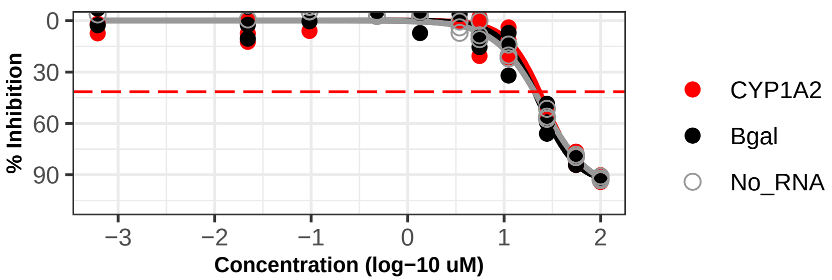

Octylbicycloheptenedicarboximide: CYP2C19

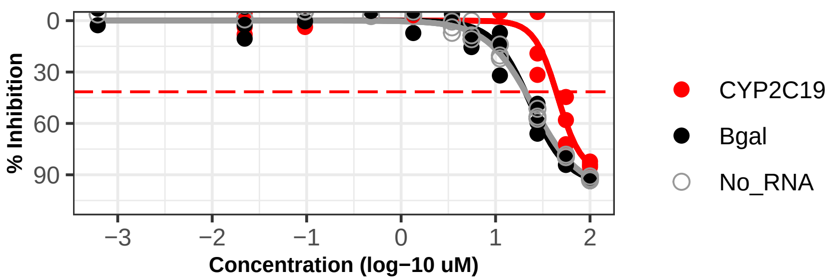

Octylbicycloheptenedicarboximide: CYP2A6

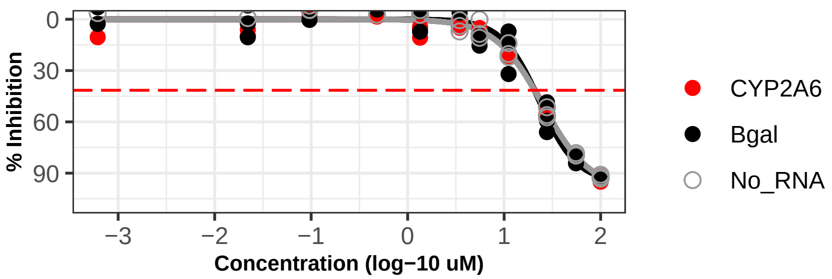

Octylbicycloheptenedicarboximide: CYP2D6

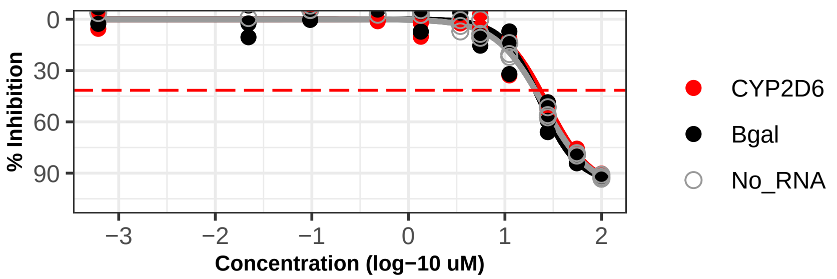

Octylbicycloheptenedicarboximide: CYP2B6

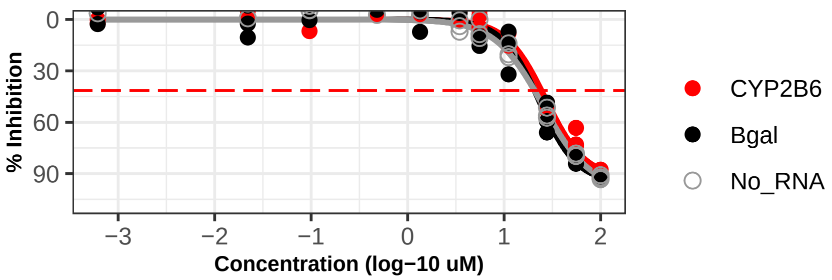

Octylbicycloheptenedicarboximide: CYP2E1

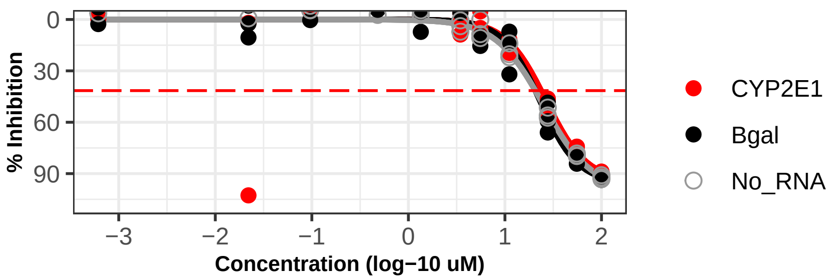

Octylbicycloheptenedicarboximide: CYP2C8

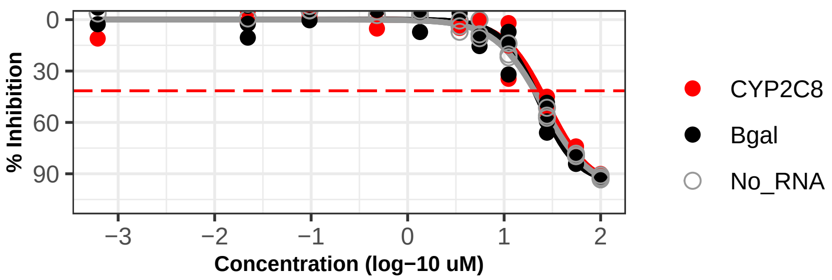

Octylbicycloheptenedicarboximide: CYP2J2

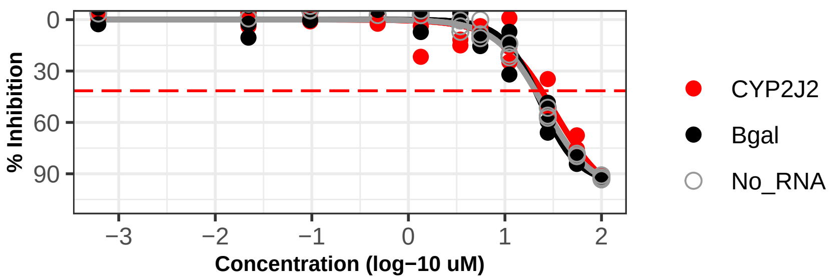

Octylbicycloheptenedicarboximide: CYP2C9

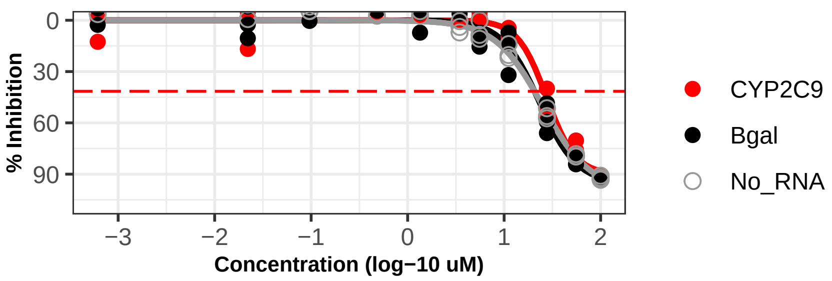

Octylbicycloheptenedicarboximide: CYP3A4

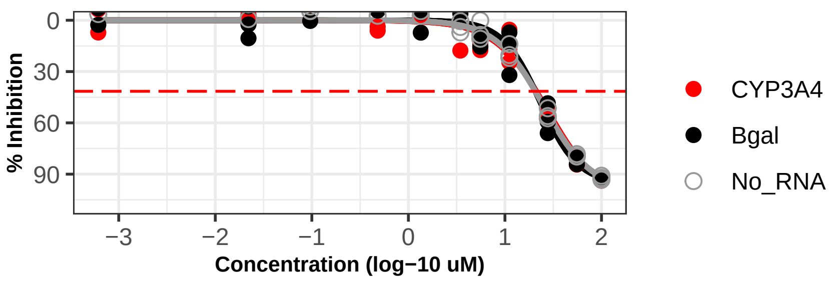

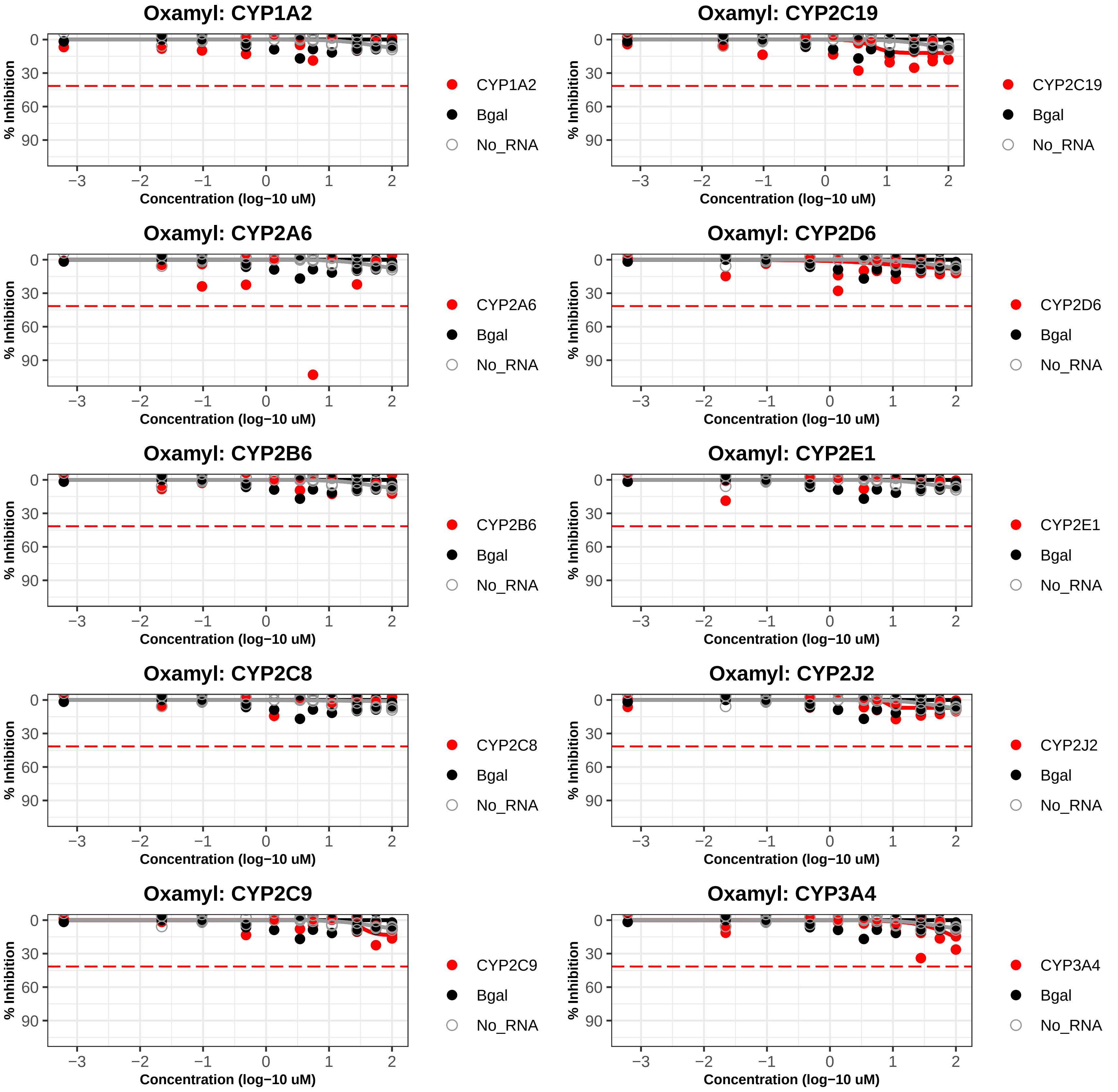
Oxamyl: CYP1A2
Oxamyl: CYP2C19
Oxamyl: CYP2A6
Oxamyl: CYP2D6
Oxamyl: CYP2B6
Oxamyl: CYP2E1
Oxamyl: CYP2C8
Oxamyl: CYP2J2
Oxamyl: CYP2C9
Oxamyl: CYP3A4
% Inhibition
Concentration (log−10 uM)
CYP1A2
CYP2C19
CYP2A6
CYP2D6
CYP2B6
CYP2E1
CYP2C8
CYP2J2
CYP2C9
CYP3A4
Bgal
No_RNA

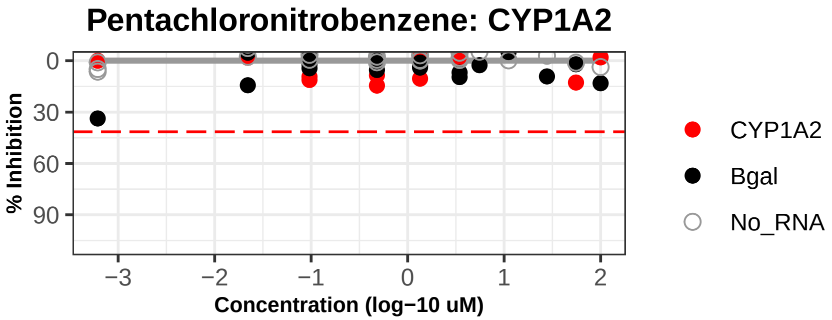
Pentachloronitrobenzene: CYP1A2
% Inhibition
0
30
60
90
−3
−2
−1
0
1
2
Concentration (log−10 uM)
CYP1A2
Bgal
No_RNA

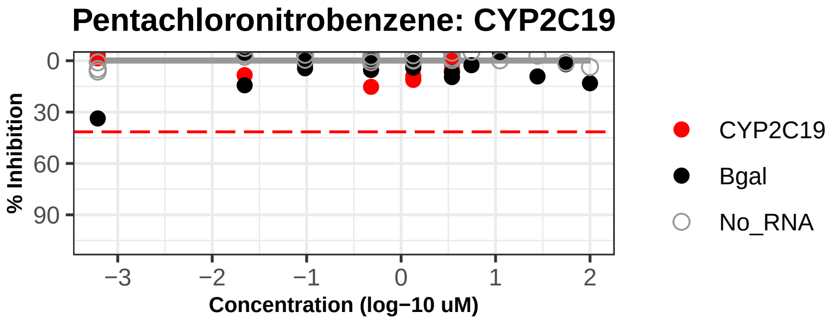
Pentachloronitrobenzene: CYP2C19
% Inhibition
0
30
60
90
−3
−2
−1
0
1
2
Concentration (log−10 uM)
CYP2C19
Bgal
No_RNA

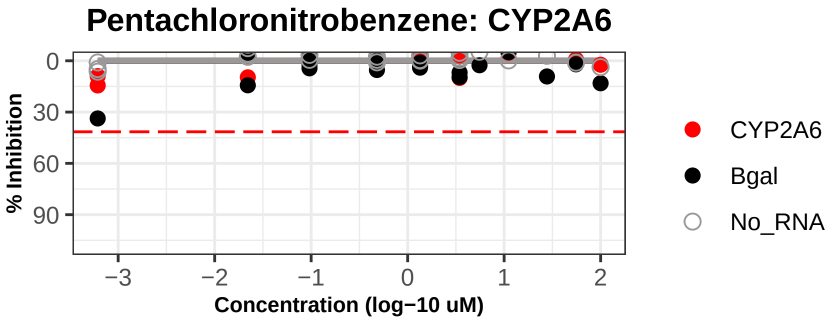
Pentachloronitrobenzene: CYP2A6
% Inhibition
0
30
60
90
−3
−2
−1
0
1
2
Concentration (log−10 uM)
CYP2A6
Bgal
No_RNA

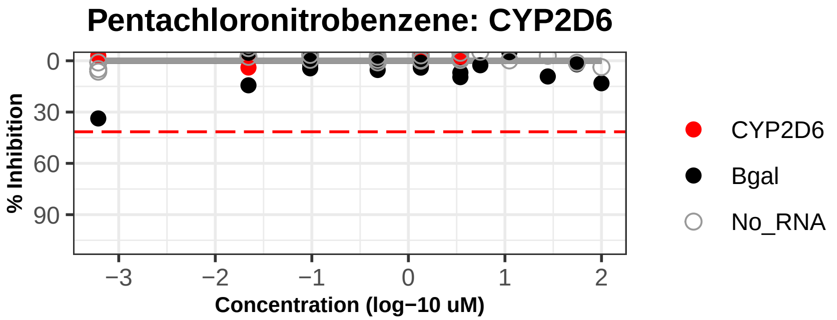
Pentachloronitrobenzene: CYP2D6
% Inhibition
0
30
60
90
−3
−2
−1
0
1
2
Concentration (log−10 uM)
CYP2D6
Bgal
No_RNA

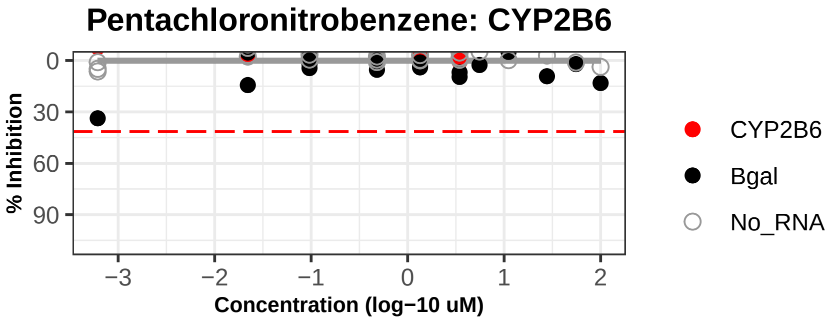
Pentachloronitrobenzene: CYP2B6
% Inhibition
0
30
60
90
−3
−2
−1
0
1
2
Concentration (log−10 uM)
CYP2B6
Bgal
No_RNA

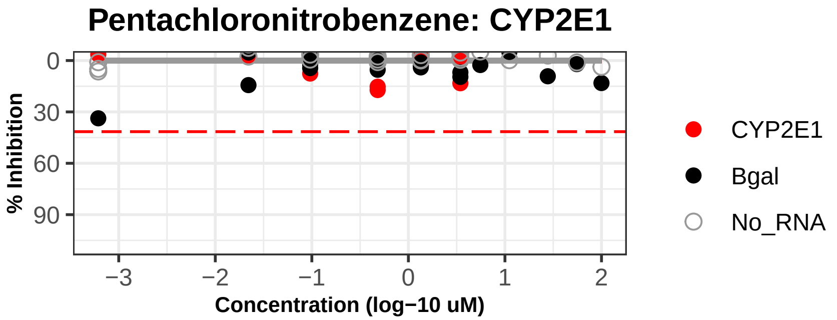
Pentachloronitrobenzene: CYP2E1
% Inhibition
0
30
60
90
−3
−2
−1
0
1
2
Concentration (log−10 uM)
CYP2E1
Bgal
No_RNA

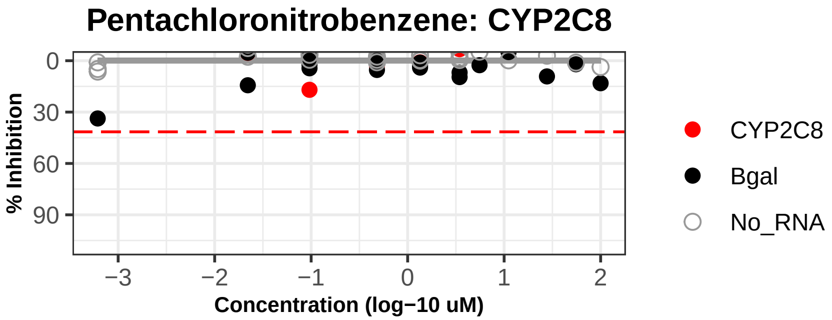
Pentachloronitrobenzene: CYP2C8
% Inhibition
0
30
60
90
−3
−2
−1
0
1
2
Concentration (log−10 uM)
CYP2C8
Bgal
No_RNA

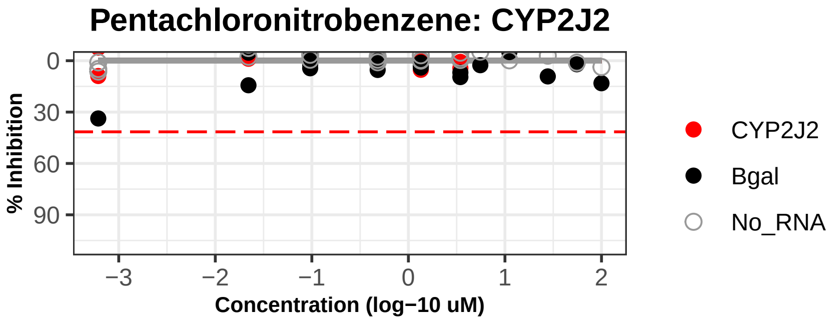
Pentachloronitrobenzene: CYP2J2
% Inhibition
0
30
60
90
−3
−2
−1
0
1
2
Concentration (log−10 uM)
CYP2J2
Bgal
No_RNA

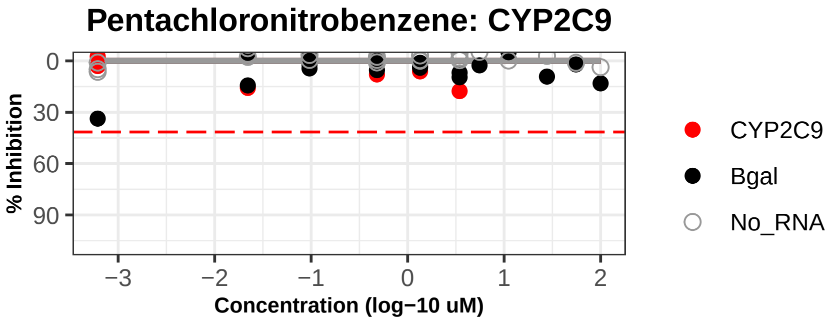
Pentachloronitrobenzene: CYP2C9
% Inhibition
0
30
60
90
−3
−2
−1
0
1
2
Concentration (log−10 uM)
CYP2C9
Bgal
No_RNA

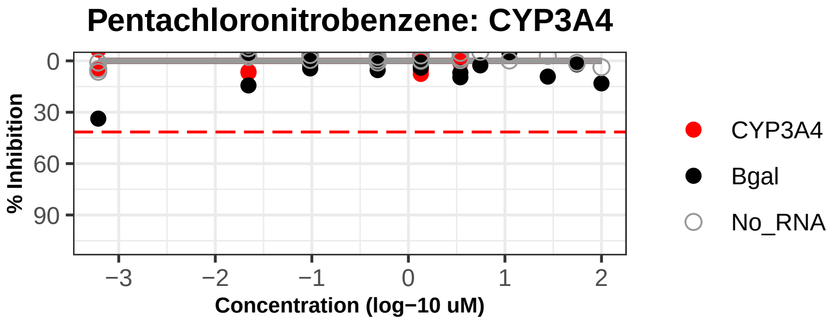
Pentachloronitrobenzene: CYP3A4
% Inhibition
0
30
60
90
−3
−2
−1
0
1
2
Concentration (log−10 uM)
CYP3A4
Bgal
No_RNA

Pentachlorophenol: CYP1A2

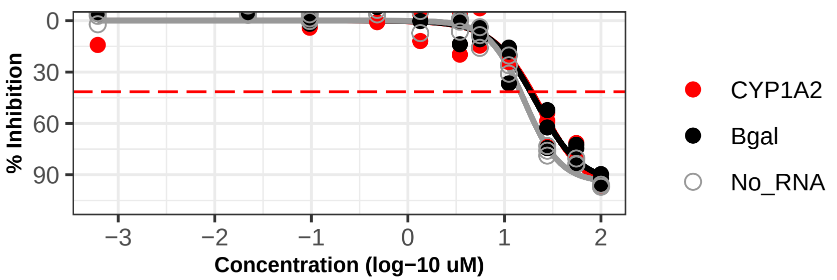

Pentachlorophenol: CYP2C19

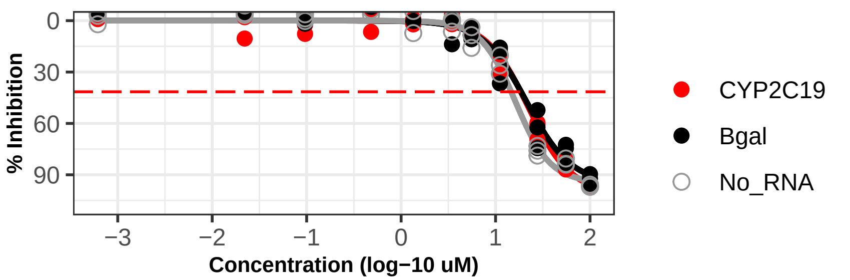

Pentachlorophenol: CYP2A6

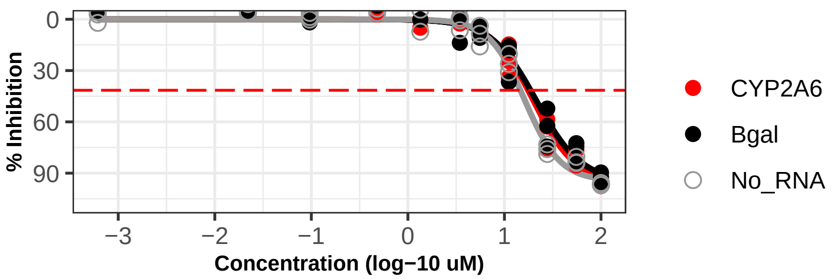

Pentachlorophenol: CYP2D6

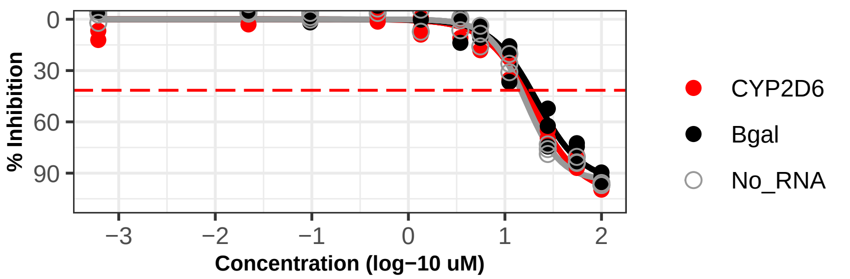

Pentachlorophenol: CYP2B6

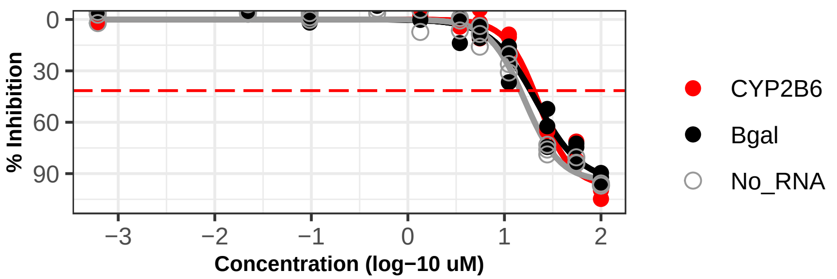

Pentachlorophenol: CYP2E1

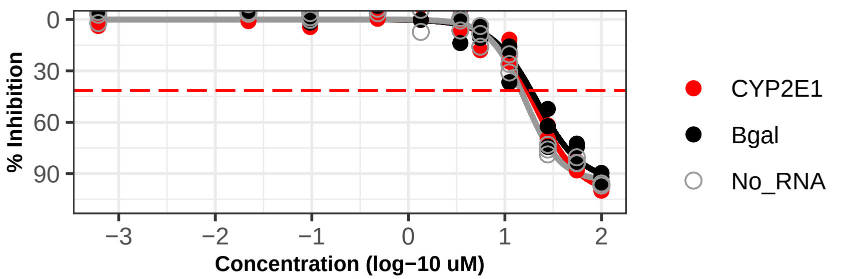

Pentachlorophenol: CYP2C8

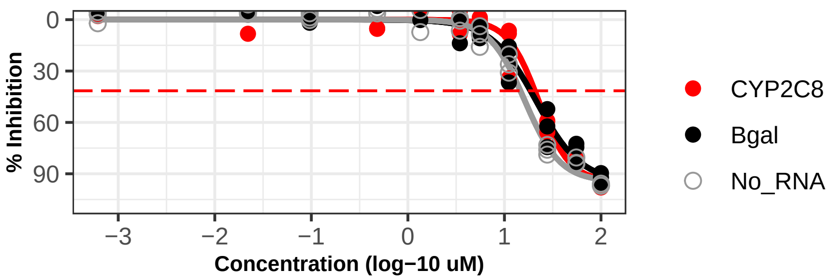

Pentachlorophenol: CYP2J2

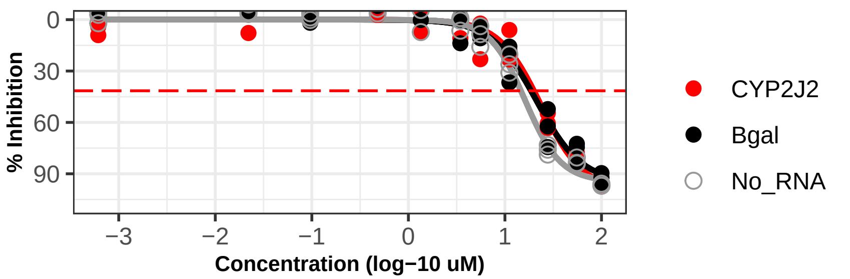

Pentachlorophenol: CYP2C9

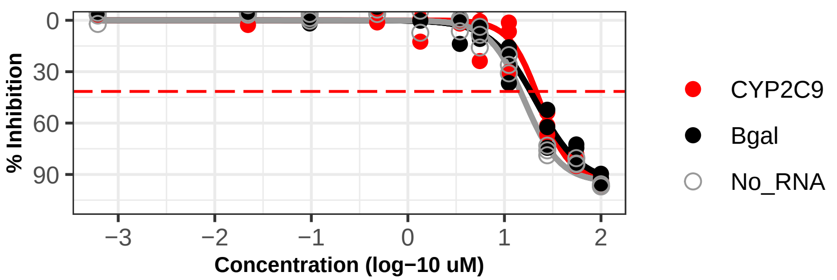

Pentachlorophenol: CYP3A4

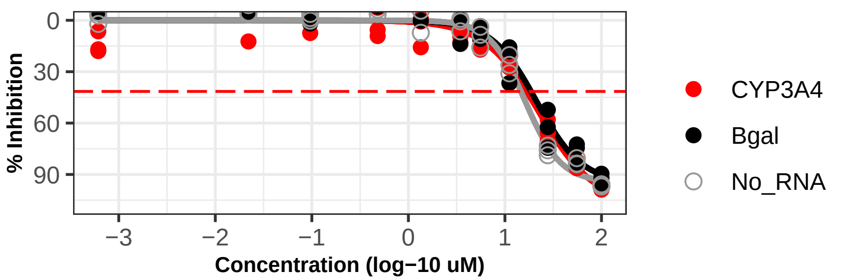

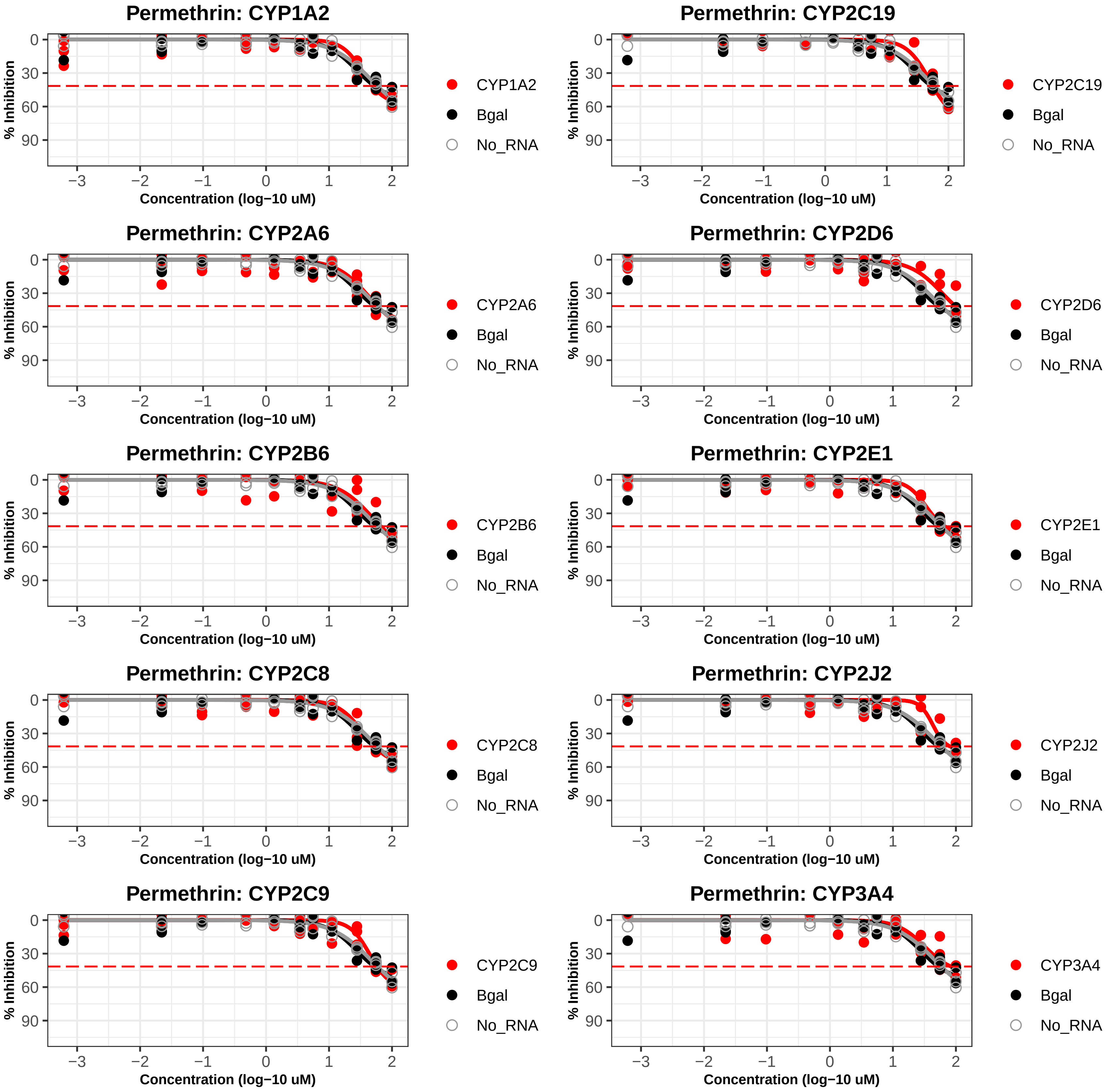
Permethrin: CYP1A2
Permethrin: CYP2C19
Permethrin: CYP2A6
Permethrin: CYP2D6
Permethrin: CYP2B6
Permethrin: CYP2E1
Permethrin: CYP2C8
Permethrin: CYP2J2
Permethrin: CYP2C9
Permethrin: CYP3A4
% Inhibition
Concentration (log−10 uM)
CYP1A2
CYP2C19
CYP2A6
CYP2D6
CYP2B6
CYP2E1
CYP2C8
CYP2J2
CYP2C9
CYP3A4
Bgal
No_RNA

Phenothrin: CYP1A2

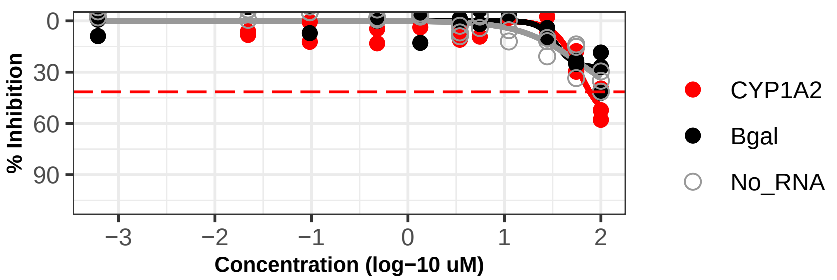

Phenothrin: CYP2C19

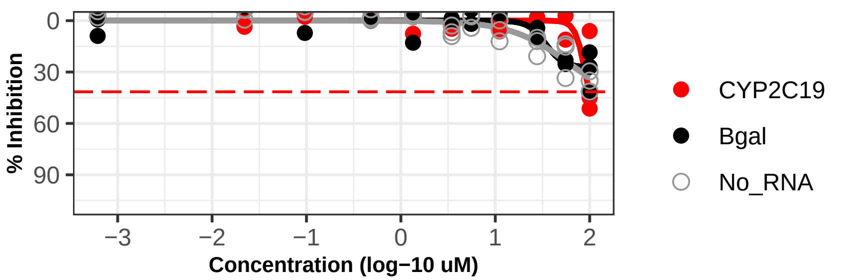

Phenothrin: CYP2A6

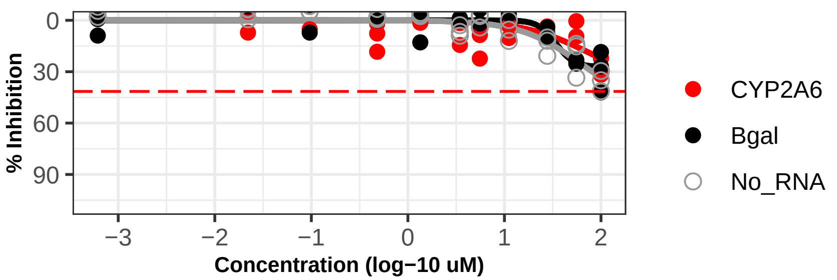

Phenothrin: CYP2D6

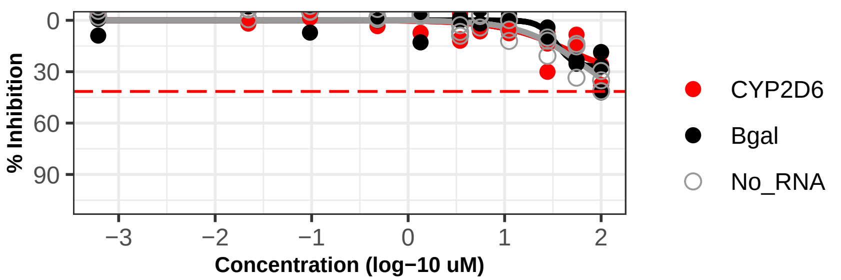

Phenothrin: CYP2B6

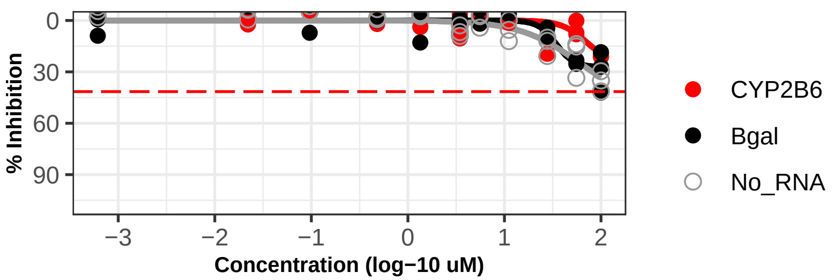

Phenothrin: CYP2E1

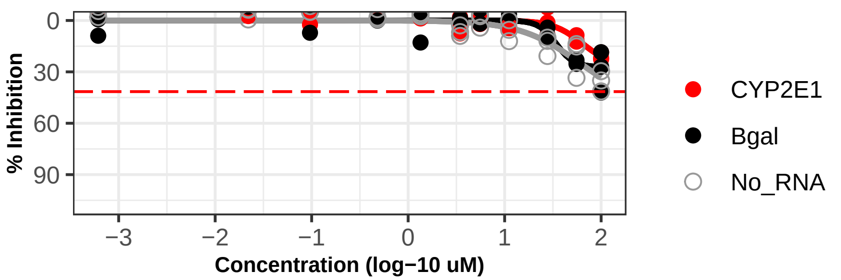

Phenothrin: CYP2C8

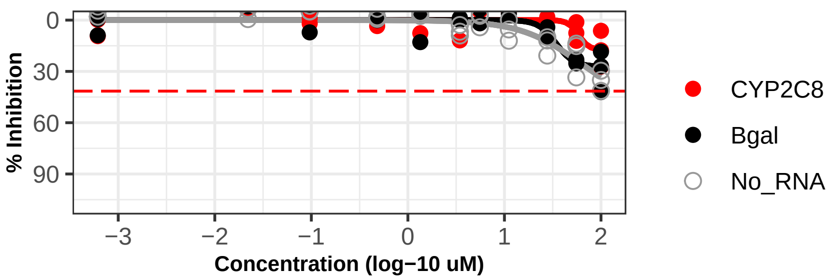

Phenothrin: CYP2J2

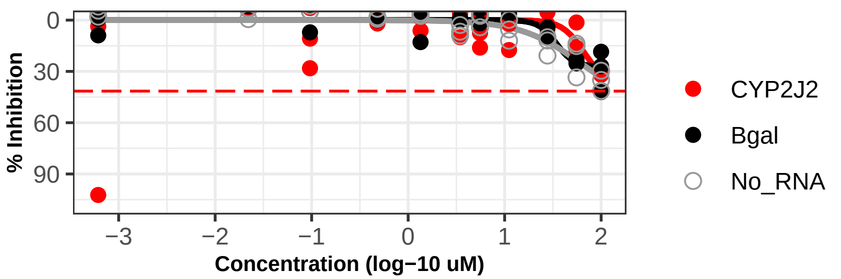

Phenothrin: CYP2C9

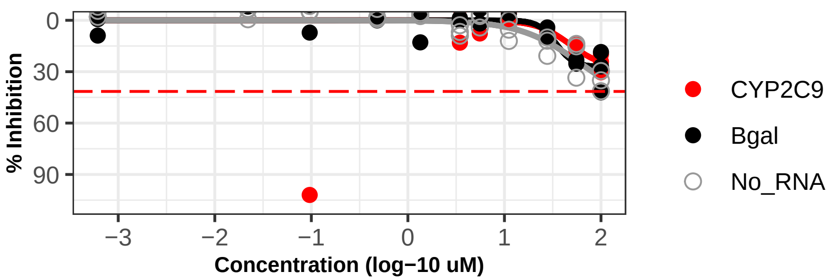

Phenothrin: CYP3A4

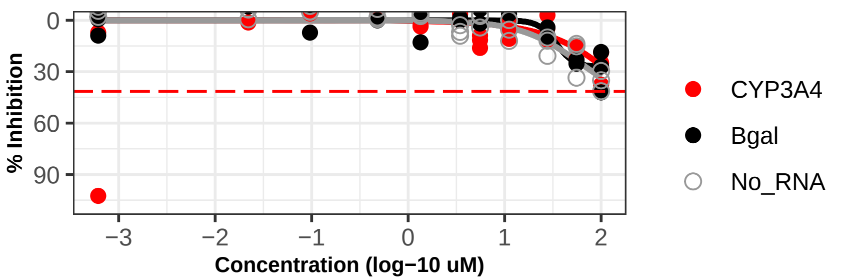

Phosmet: CYP1A2

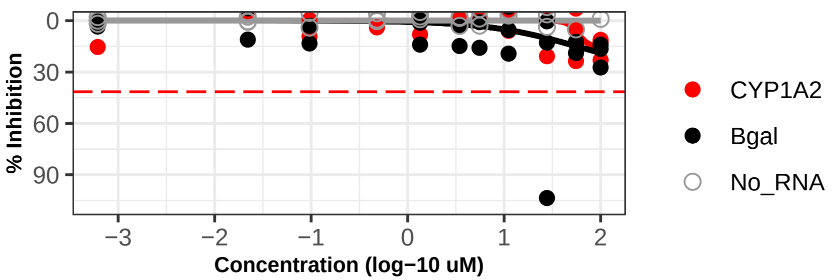

Phosmet: CYP2C19

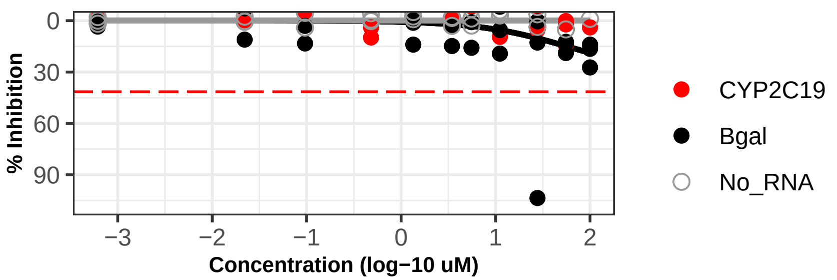

Phosmet: CYP2A6

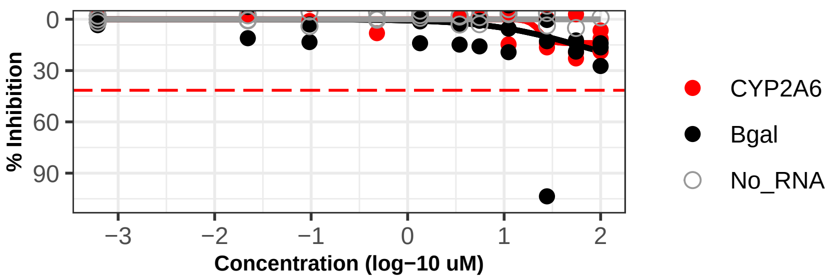

Phosmet: CYP2D6

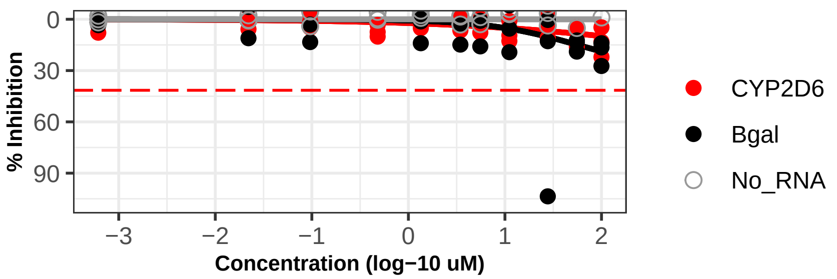

Phosmet: CYP2B6

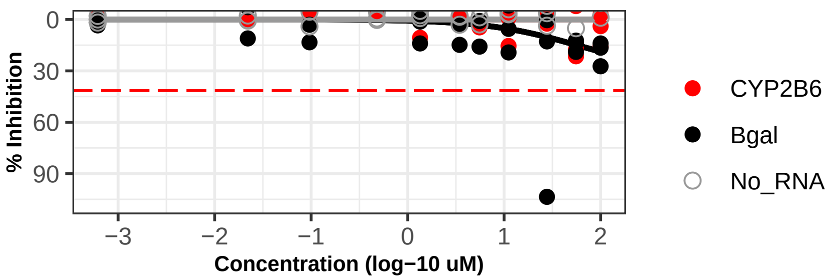

Phosmet: CYP2E1

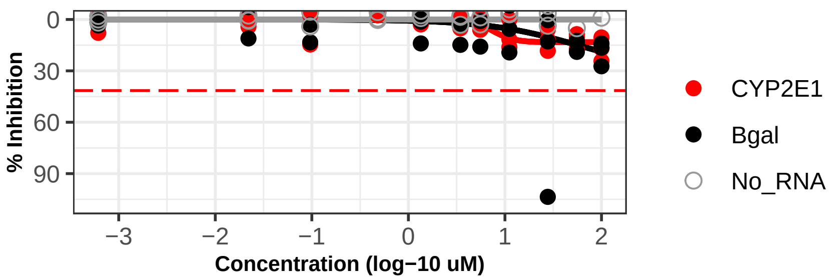

Phosmet: CYP2C8

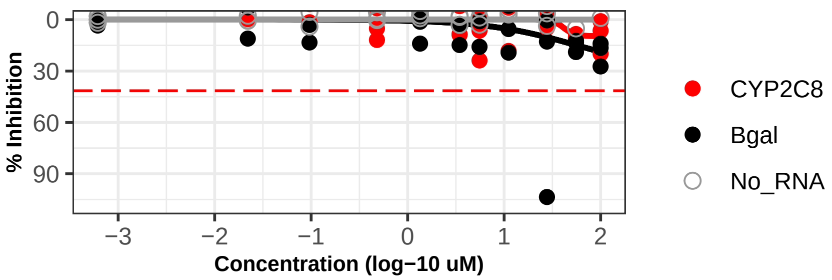

Phosmet: CYP2J2

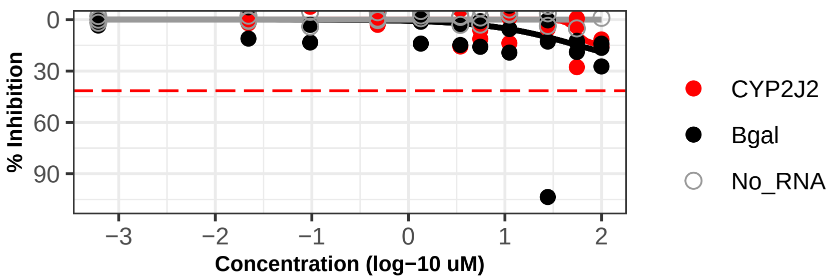

Phosmet: CYP2C9

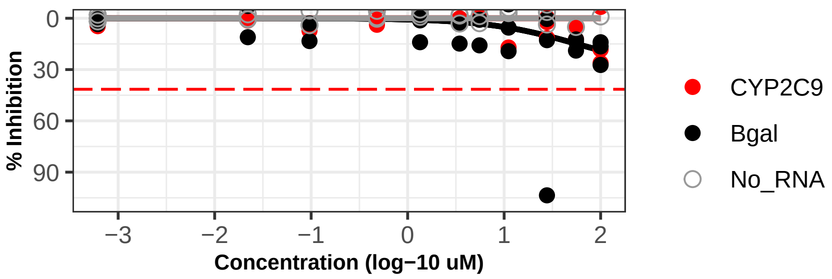

Phosmet: CYP3A4

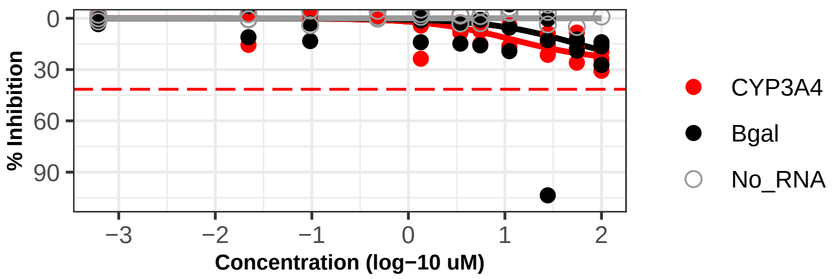

Prochloraz: CYP1A2

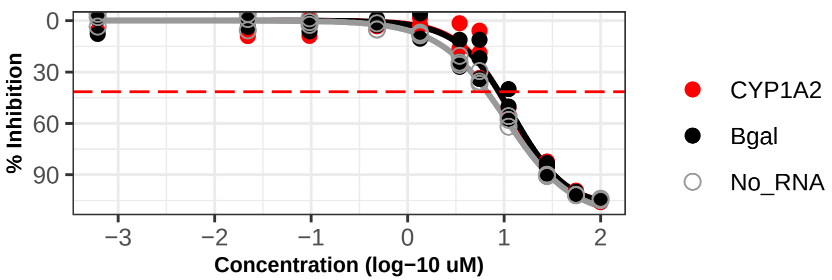

Prochloraz: CYP2C19

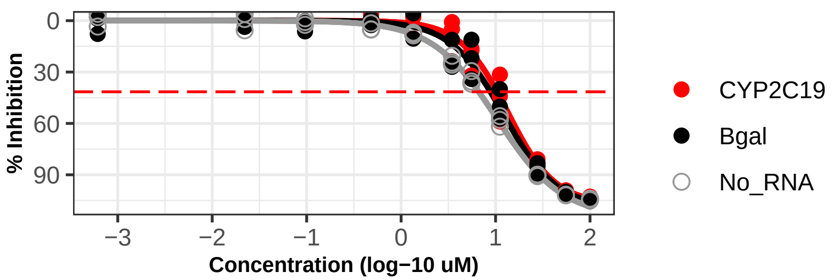

Prochloraz: CYP2A6

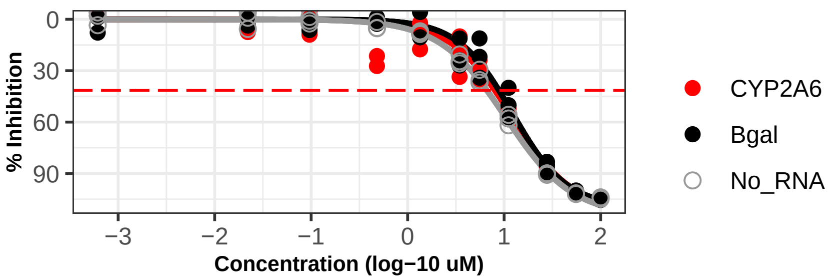

Prochloraz: CYP2D6

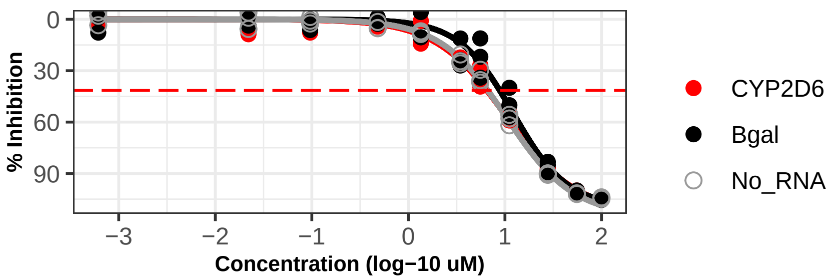

Prochloraz: CYP2B6

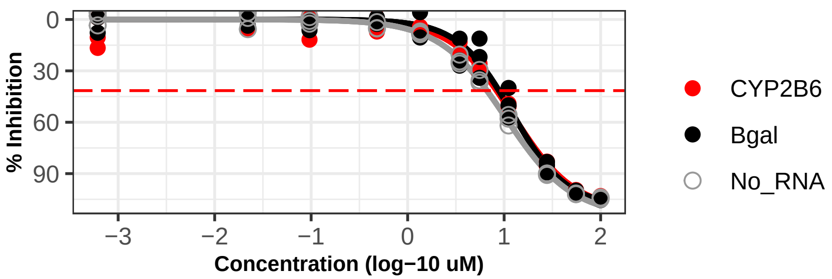

Prochloraz: CYP2E1

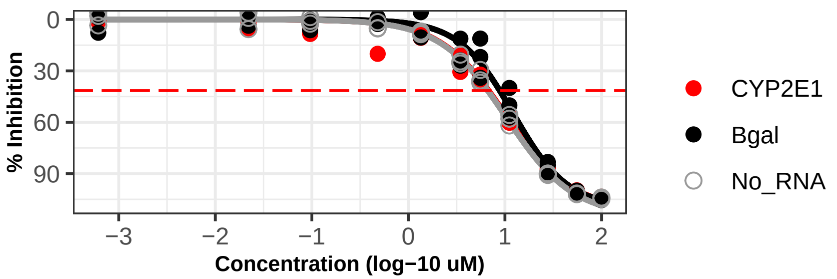

Prochloraz: CYP2C8

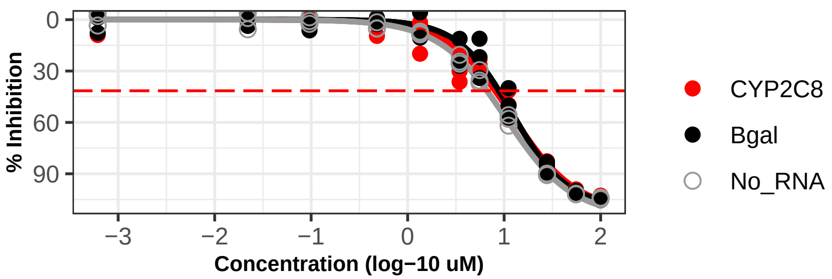

Prochloraz: CYP2J2

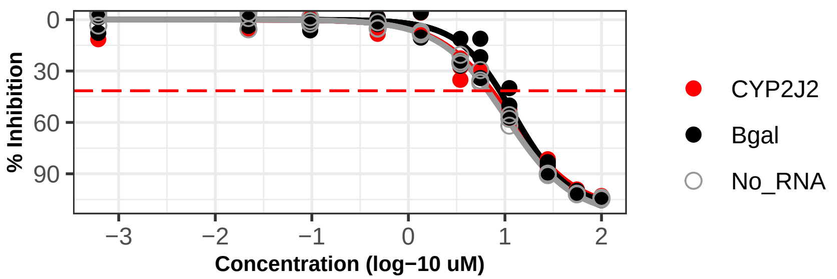

Prochloraz: CYP2C9

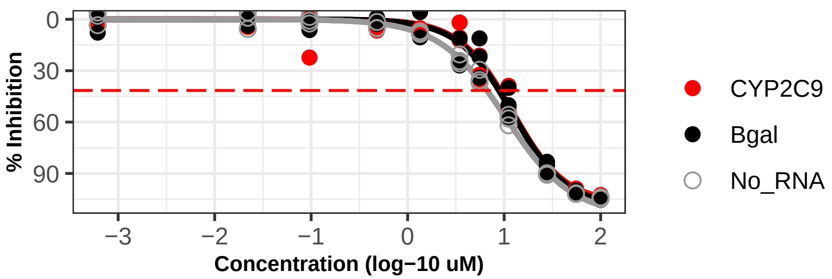

Prochloraz: CYP3A4

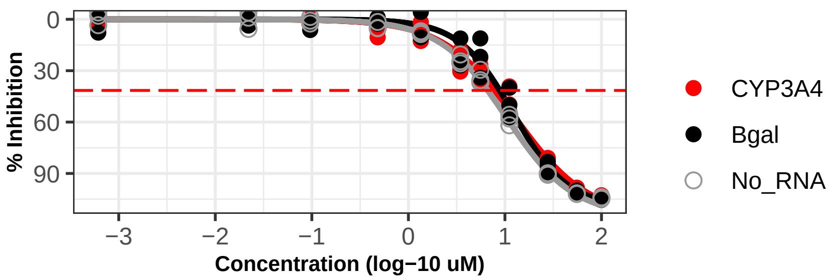

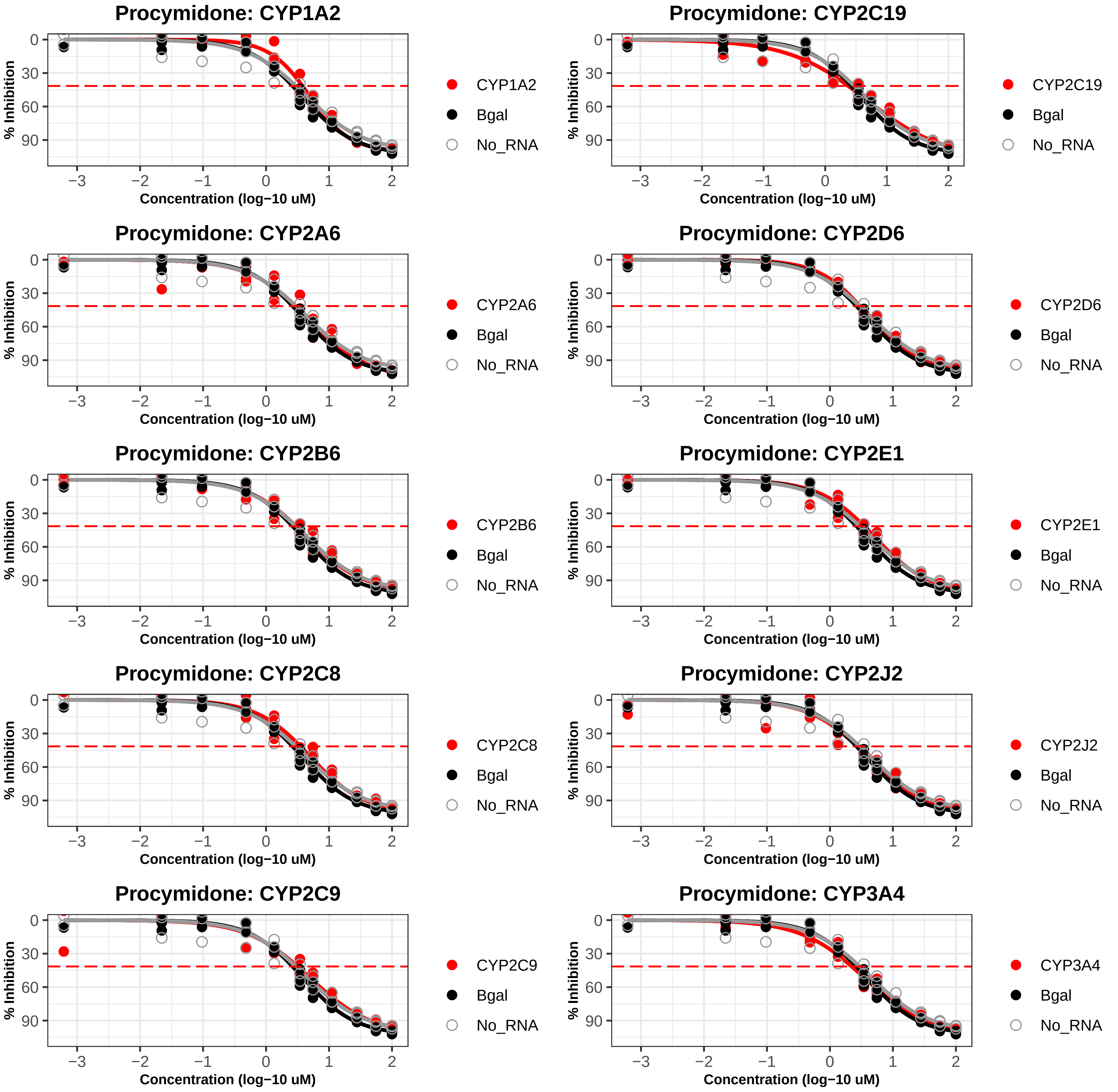
Procymidone: CYP1A2
Procymidone: CYP2C19
Procymidone: CYP2A6
Procymidone: CYP2D6
Procymidone: CYP2B6
Procymidone: CYP2E1
Procymidone: CYP2C8
Procymidone: CYP2J2
Procymidone: CYP2C9
Procymidone: CYP3A4
% Inhibition
0
30
60
90
−3
−2
−1
0
1
2
Concentration (log−10 uM)
CYP1A2
CYP2C19
CYP2A6
CYP2D6
CYP2B6
CYP2E1
CYP2C8
CYP2J2
CYP2C9
CYP3A4
Bgal
No_RNA

Propargite: CYP1A2

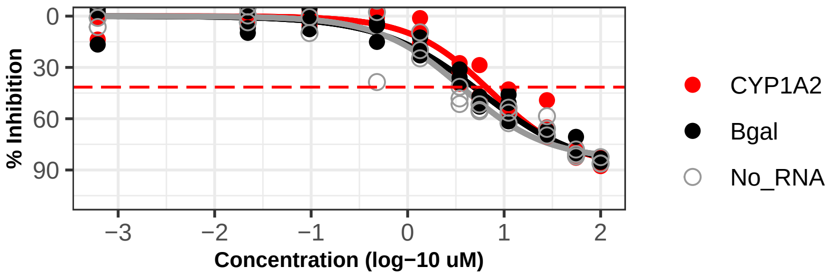

Propargite: CYP2C19

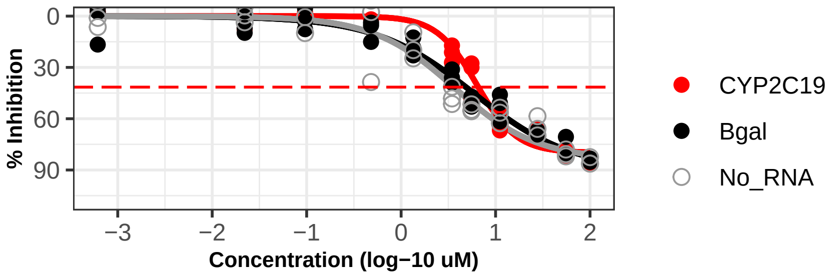

Propargite: CYP2A6

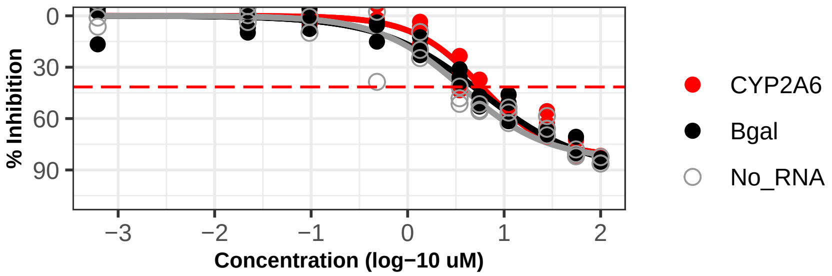

Propargite: CYP2D6

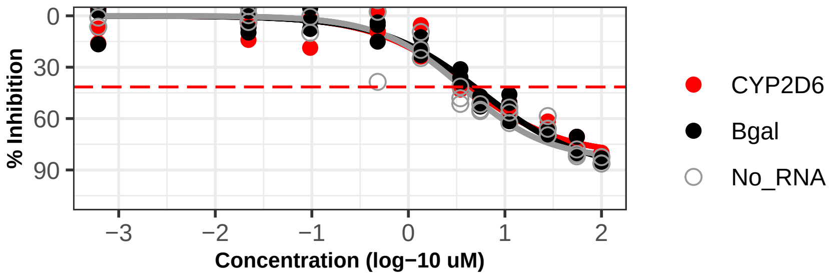

Propargite: CYP2B6

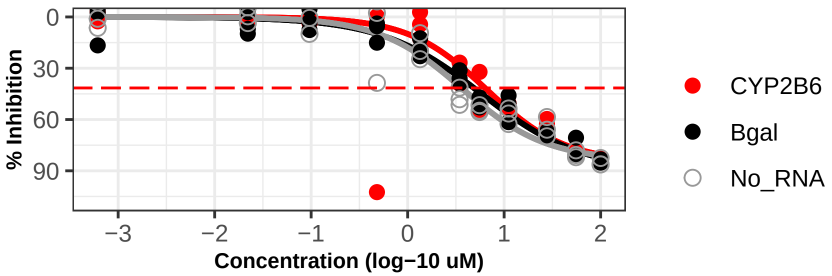

Propargite: CYP2E1

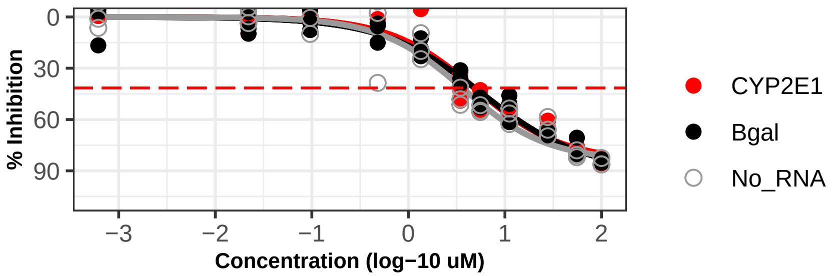

Propargite: CYP2C8

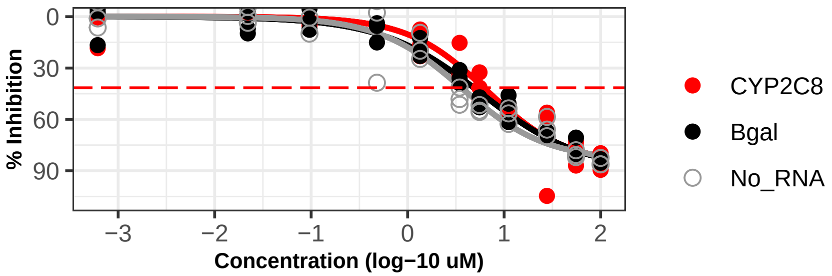

Propargite: CYP2J2

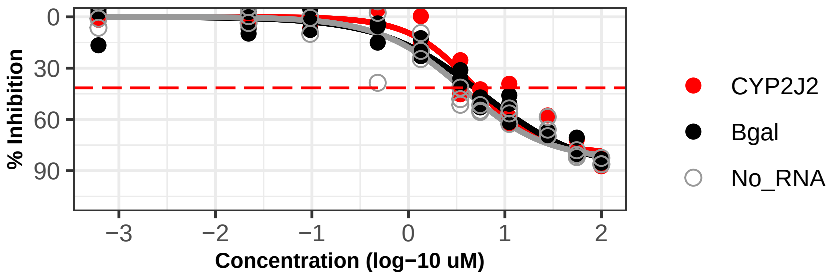

Propargite: CYP2C9

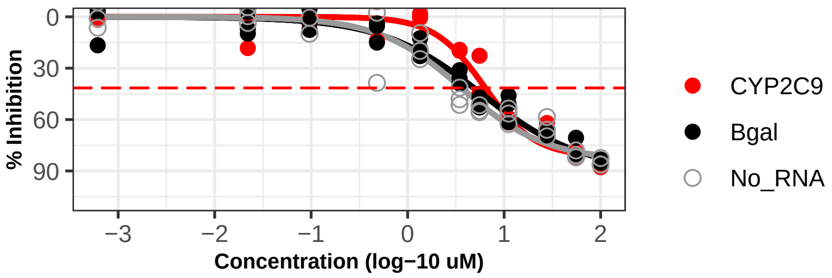

Propargite: CYP3A4

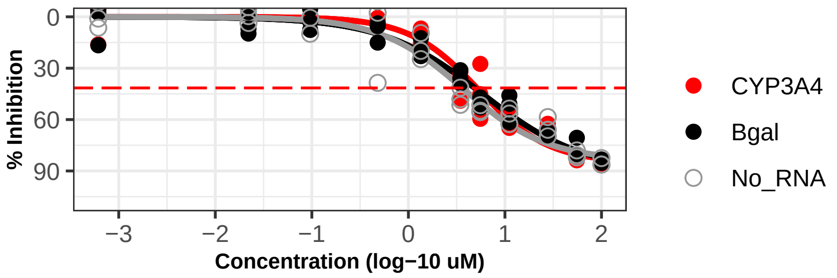

Propiconazole: CYP1A2

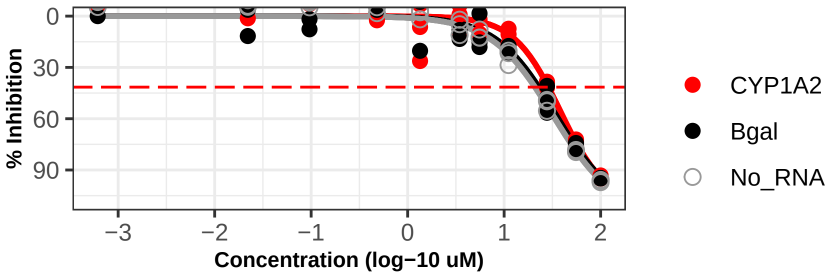

Propiconazole: CYP2C19

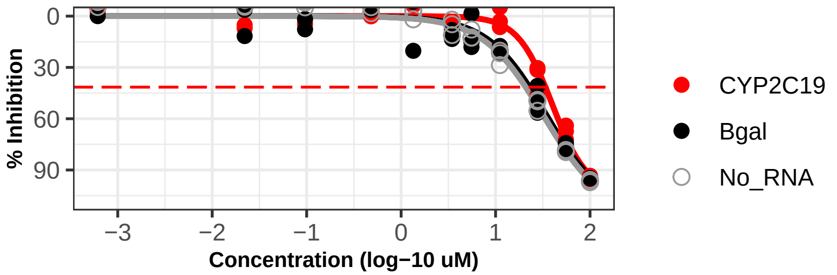

Propiconazole: CYP2A6

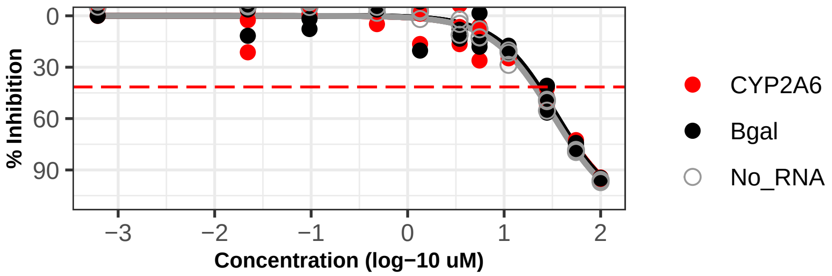

Propiconazole: CYP2D6

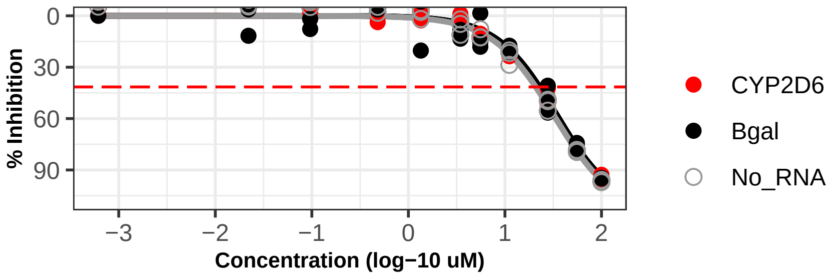

Propiconazole: CYP2B6

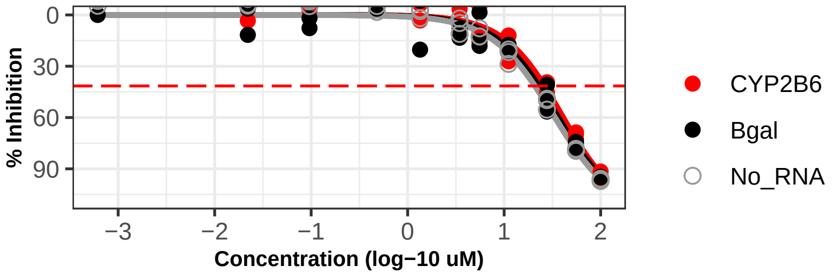

Propiconazole: CYP2E1

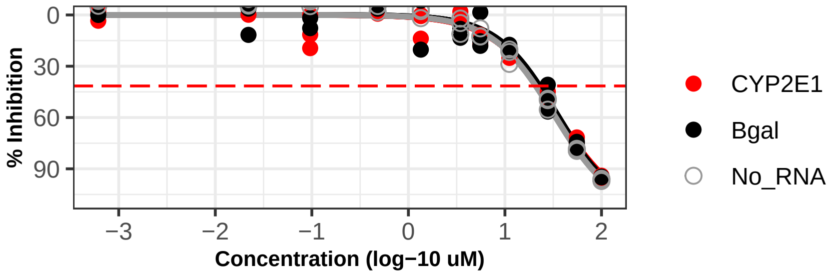

Propiconazole: CYP2C8

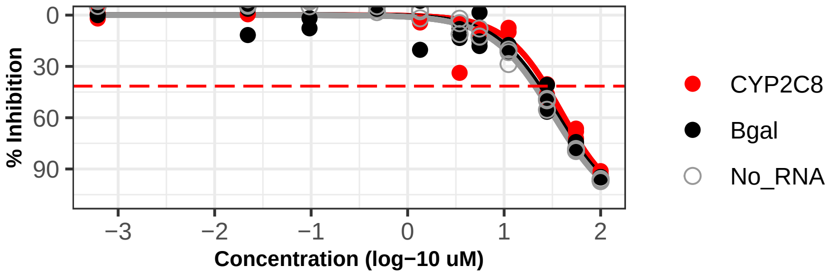

Propiconazole: CYP2J2

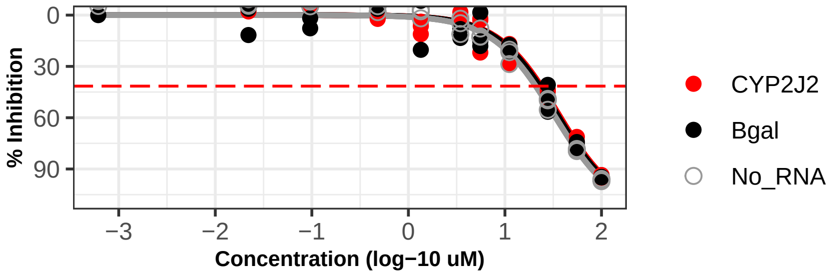

Propiconazole: CYP2C9

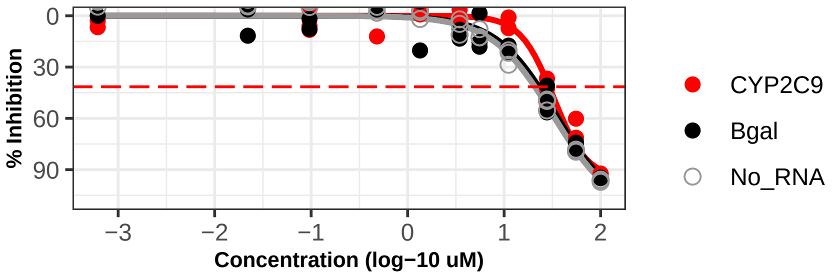

Propiconazole: CYP3A4

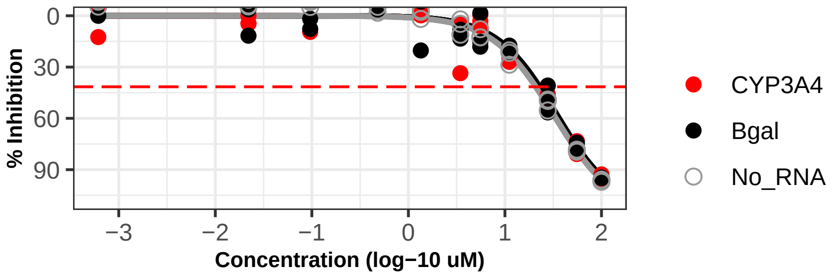

Propyzamide: CYP1A2

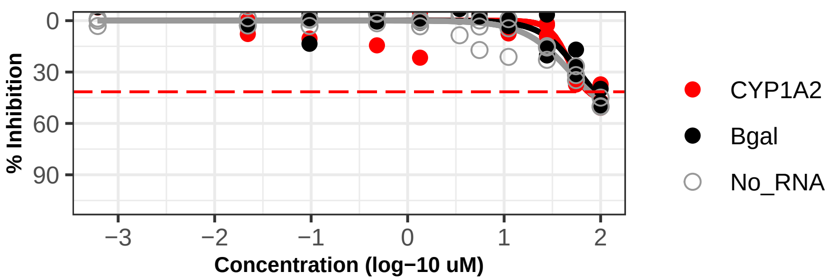

Propyzamide: CYP2C19

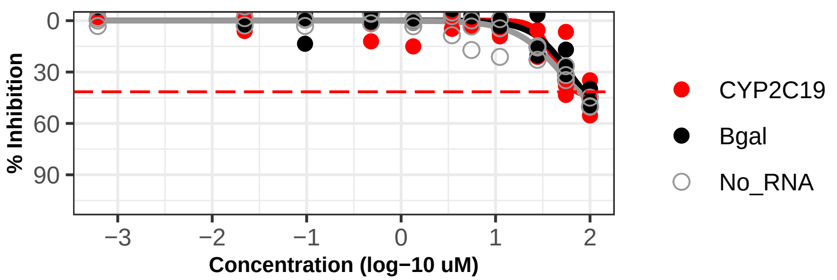

Propyzamide: CYP2A6

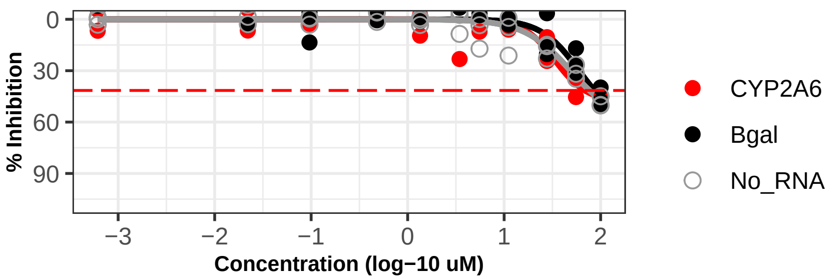

Propyzamide: CYP2D6

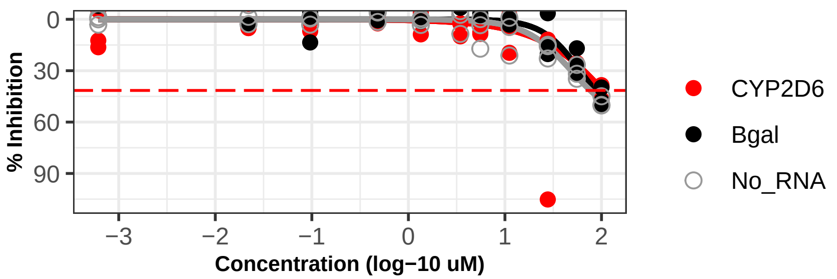

Propyzamide: CYP2B6

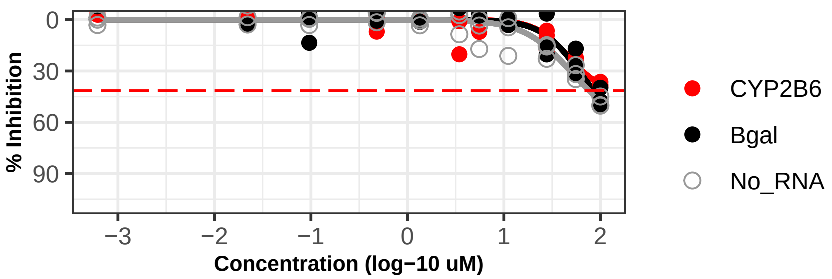

Propyzamide: CYP2E1

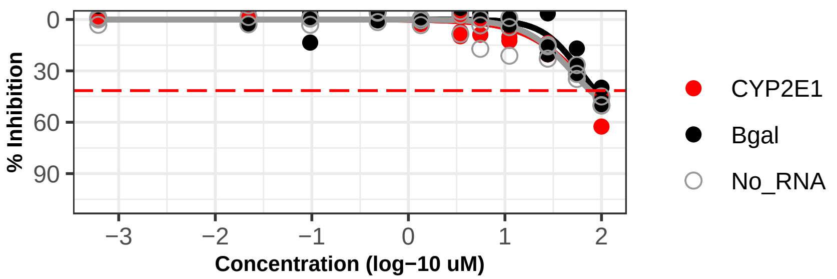

Propyzamide: CYP2C8

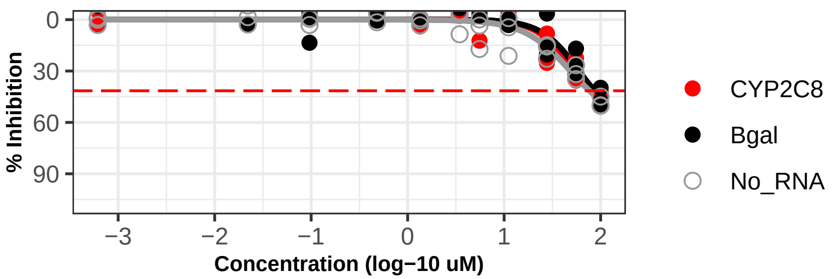

Propyzamide: CYP2J2

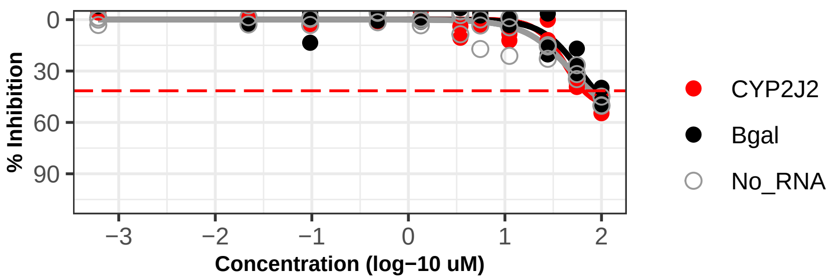

Propyzamide: CYP2C9

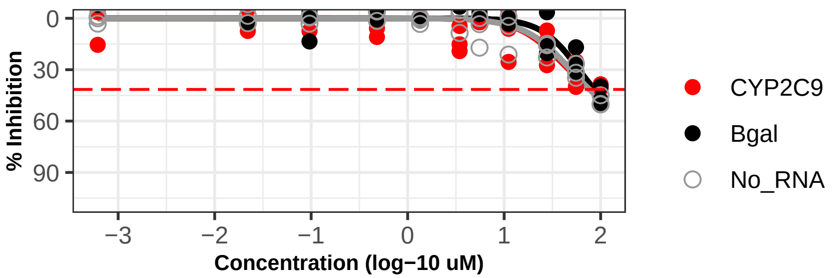

Propyzamide: CYP3A4

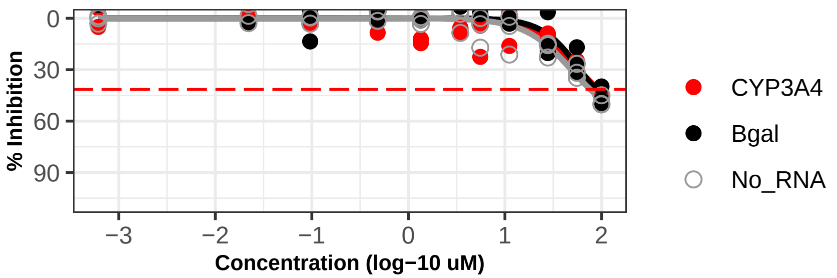

Raloxifene hydrochloride: CYP1A2

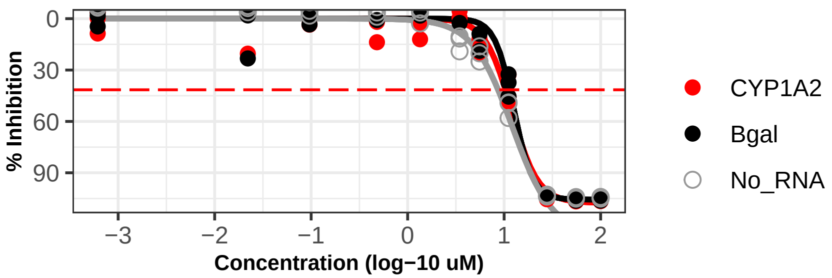

Raloxifene hydrochloride: CYP2C19

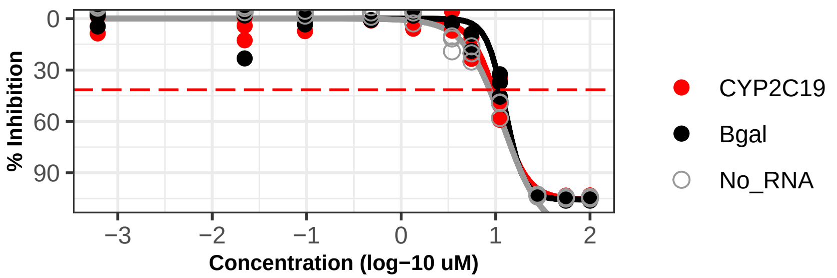

Raloxifene hydrochloride: CYP2A6

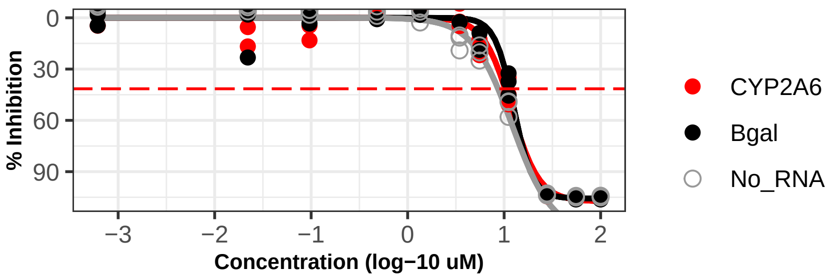

Raloxifene hydrochloride: CYP2D6

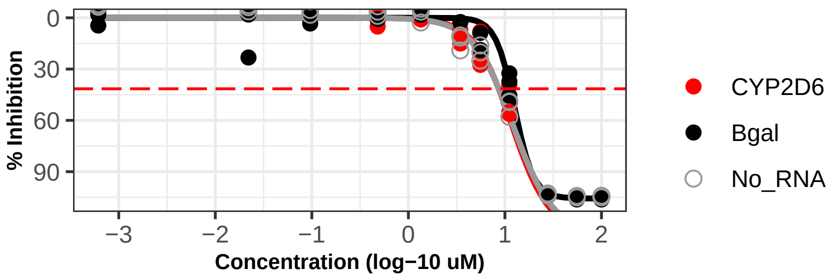

Raloxifene hydrochloride: CYP2B6

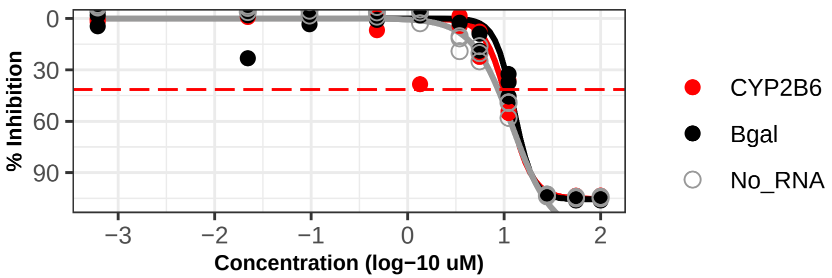

Raloxifene hydrochloride: CYP2E1

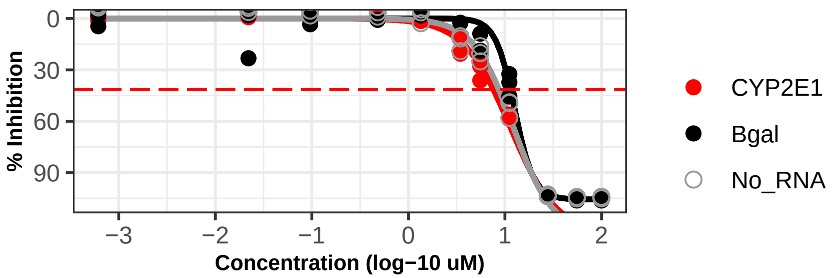

Raloxifene hydrochloride: CYP2C8

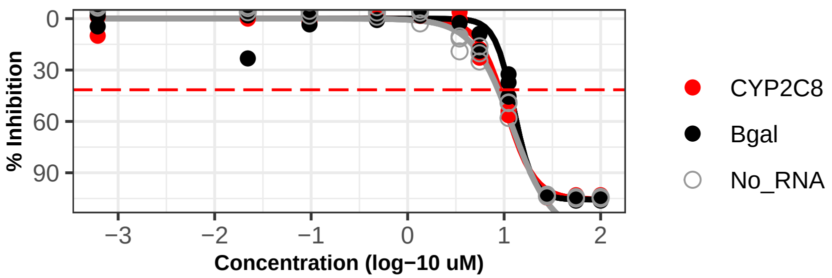

Raloxifene hydrochloride: CYP2J2

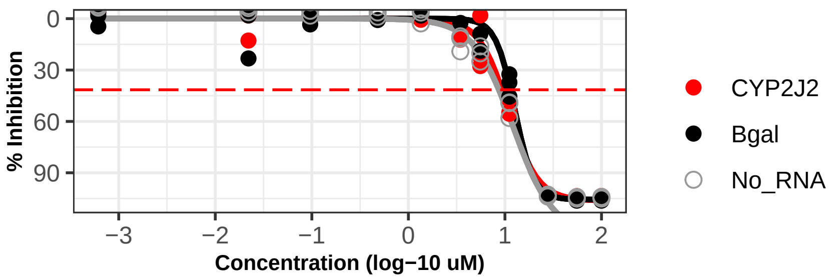

Raloxifene hydrochloride: CYP2C9

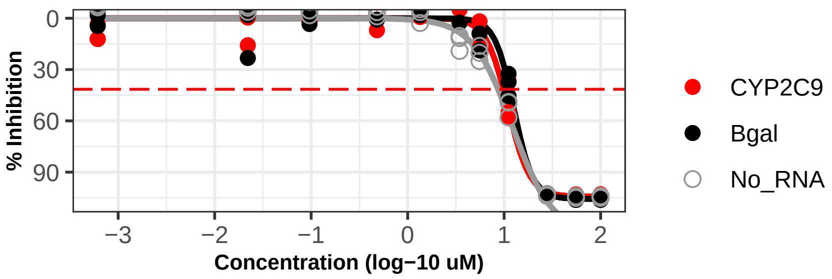

Raloxifene hydrochloride: CYP3A4

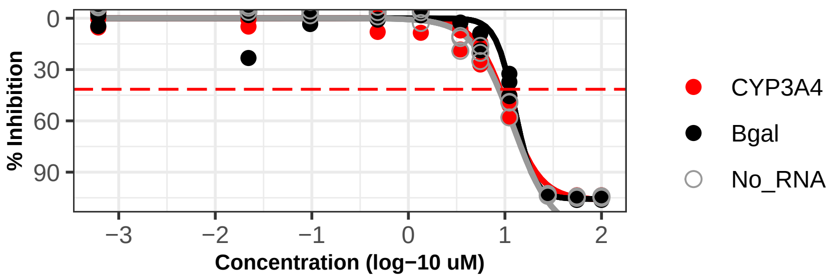

Simazine: CYP1A2

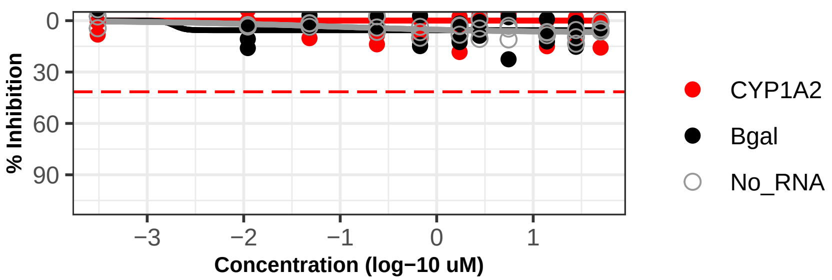

Simazine: CYP2C19

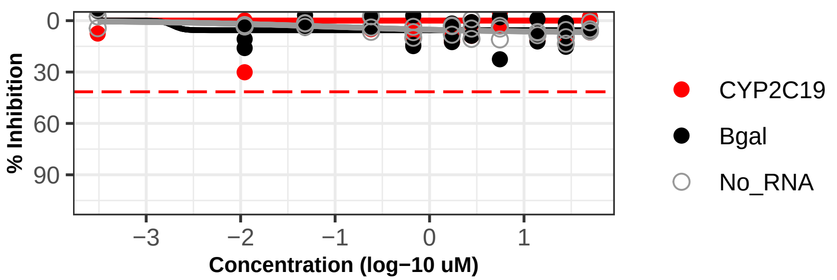

Simazine: CYP2A6

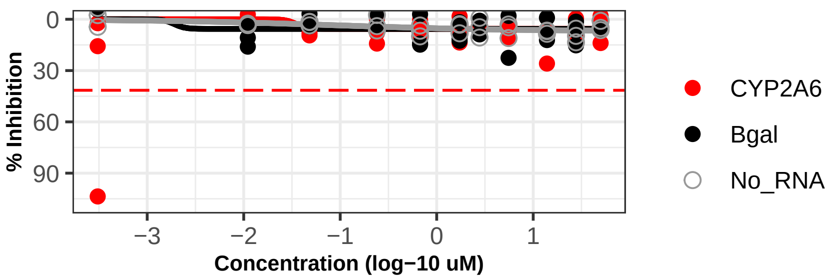

Simazine: CYP2D6

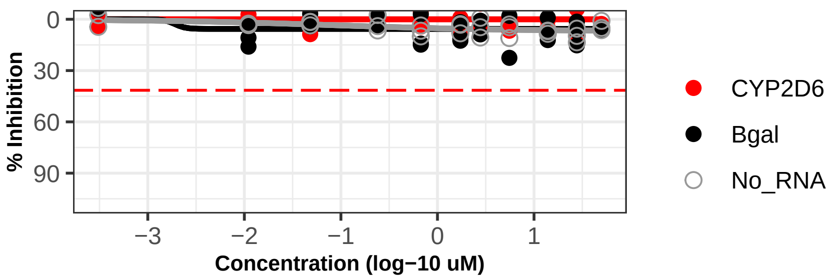

Simazine: CYP2B6

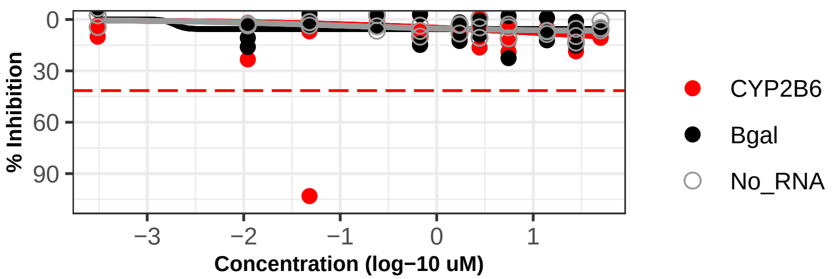

Simazine: CYP2E1

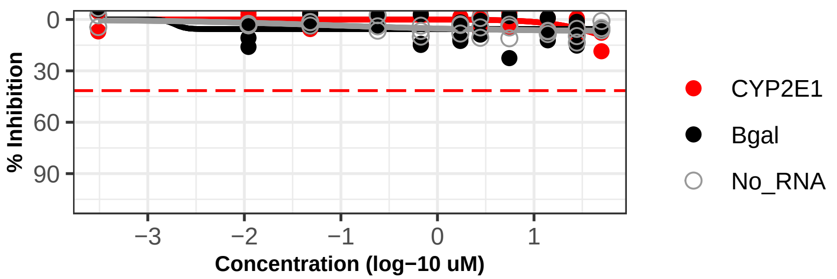

Simazine: CYP2C8

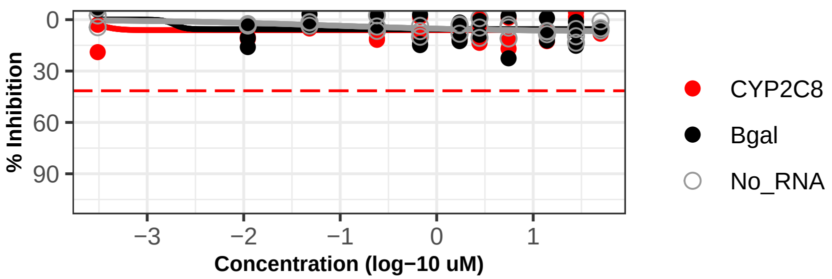

Simazine: CYP2J2

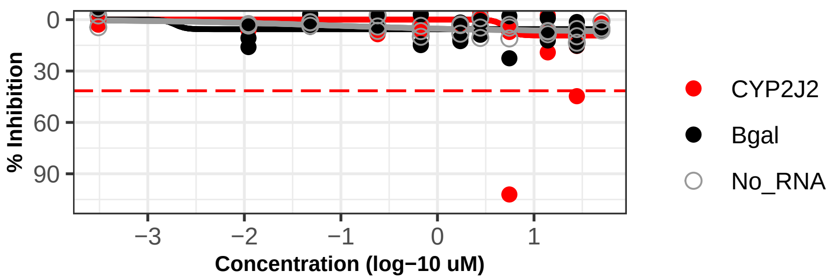

Simazine: CYP2C9

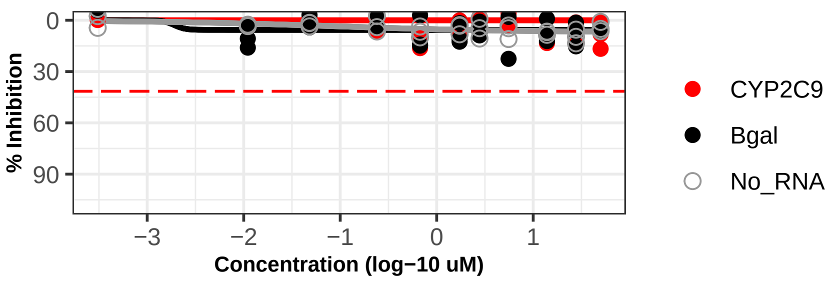

Simazine: CYP3A4

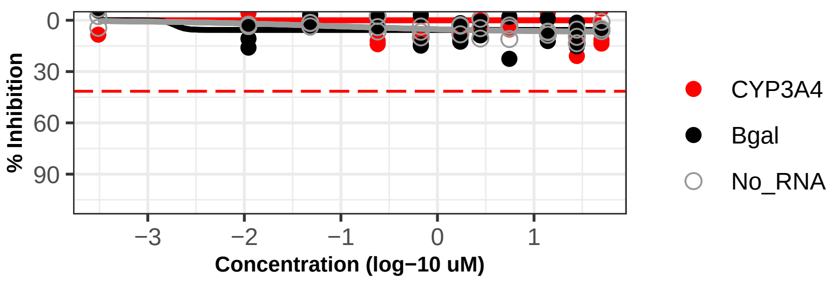

Spironolactone: CYP1A2

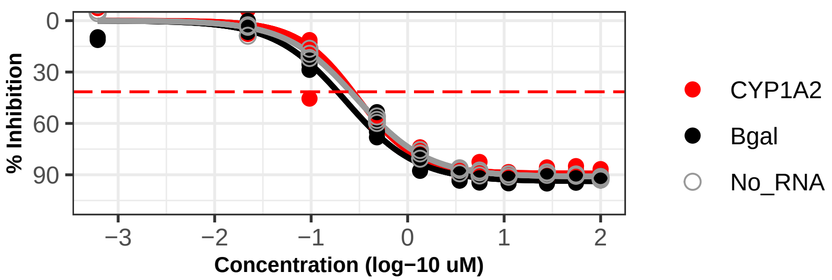

Spironolactone: CYP2C19

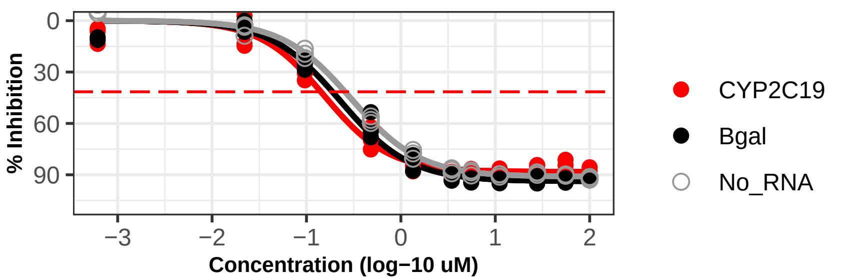

Spironolactone: CYP2A6

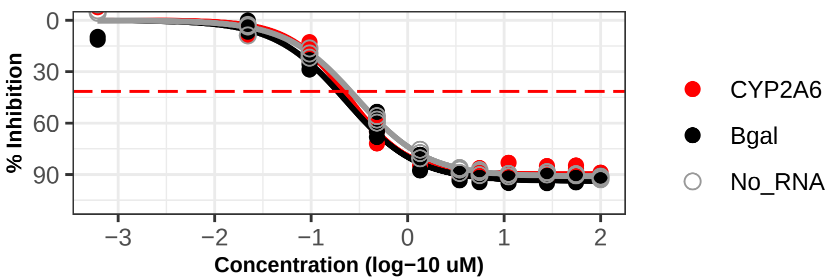

Spironolactone: CYP2D6

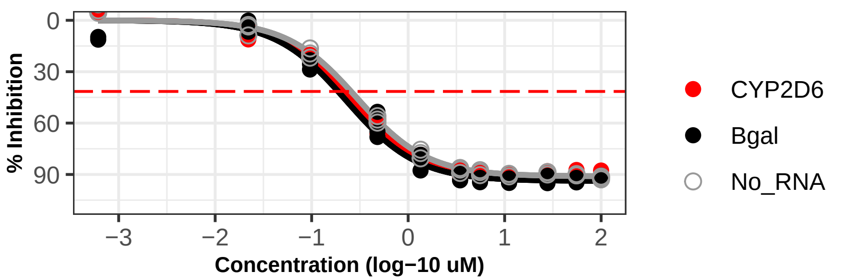

Spironolactone: CYP2B6

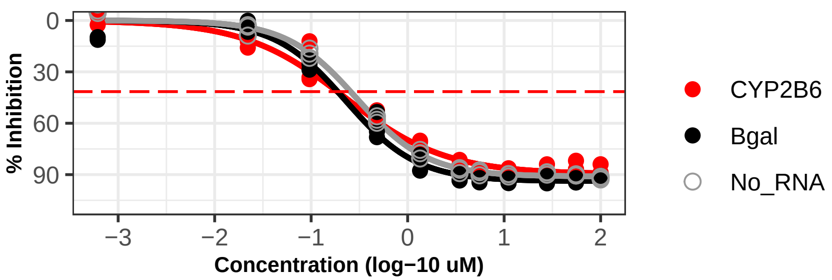

Spironolactone: CYP2E1

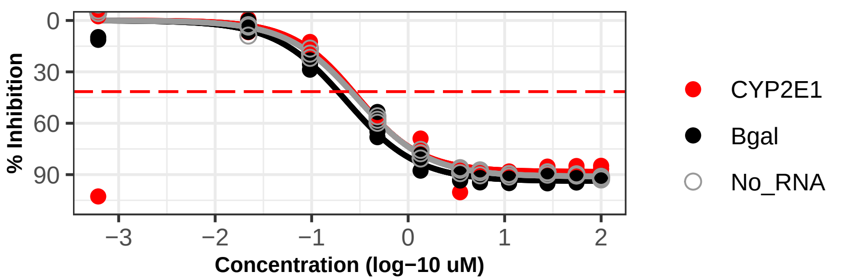

Spironolactone: CYP2C8

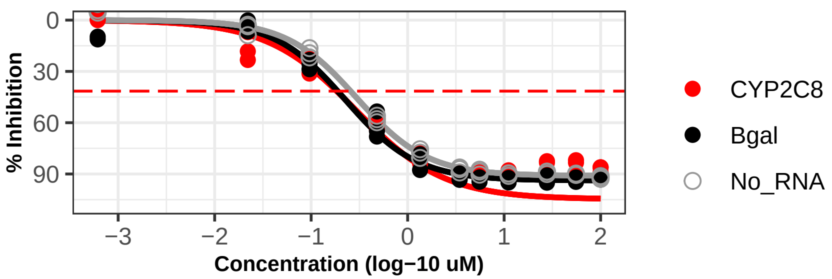

Spironolactone: CYP2J2

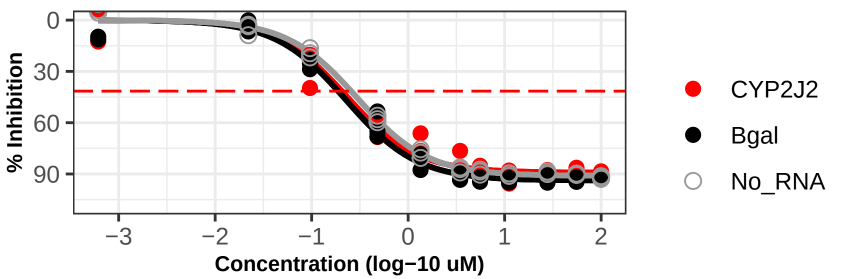

Spironolactone: CYP2C9

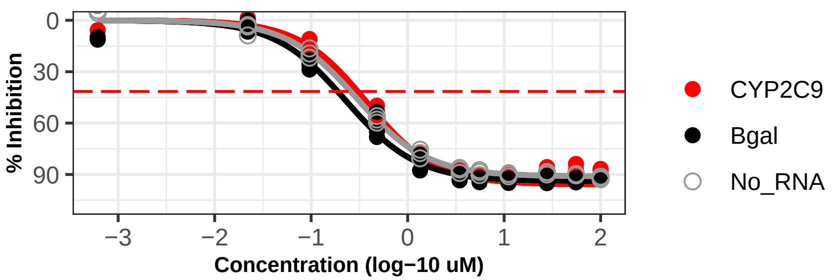

Spironolactone: CYP3A4

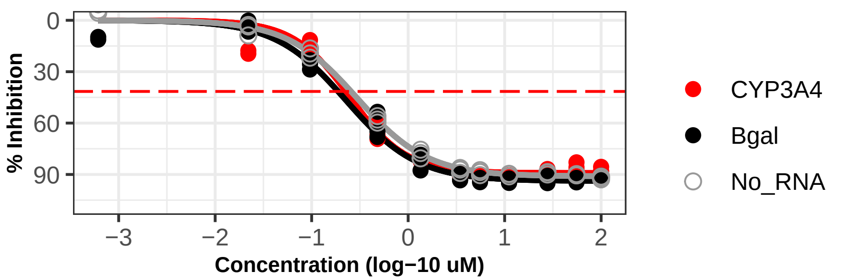

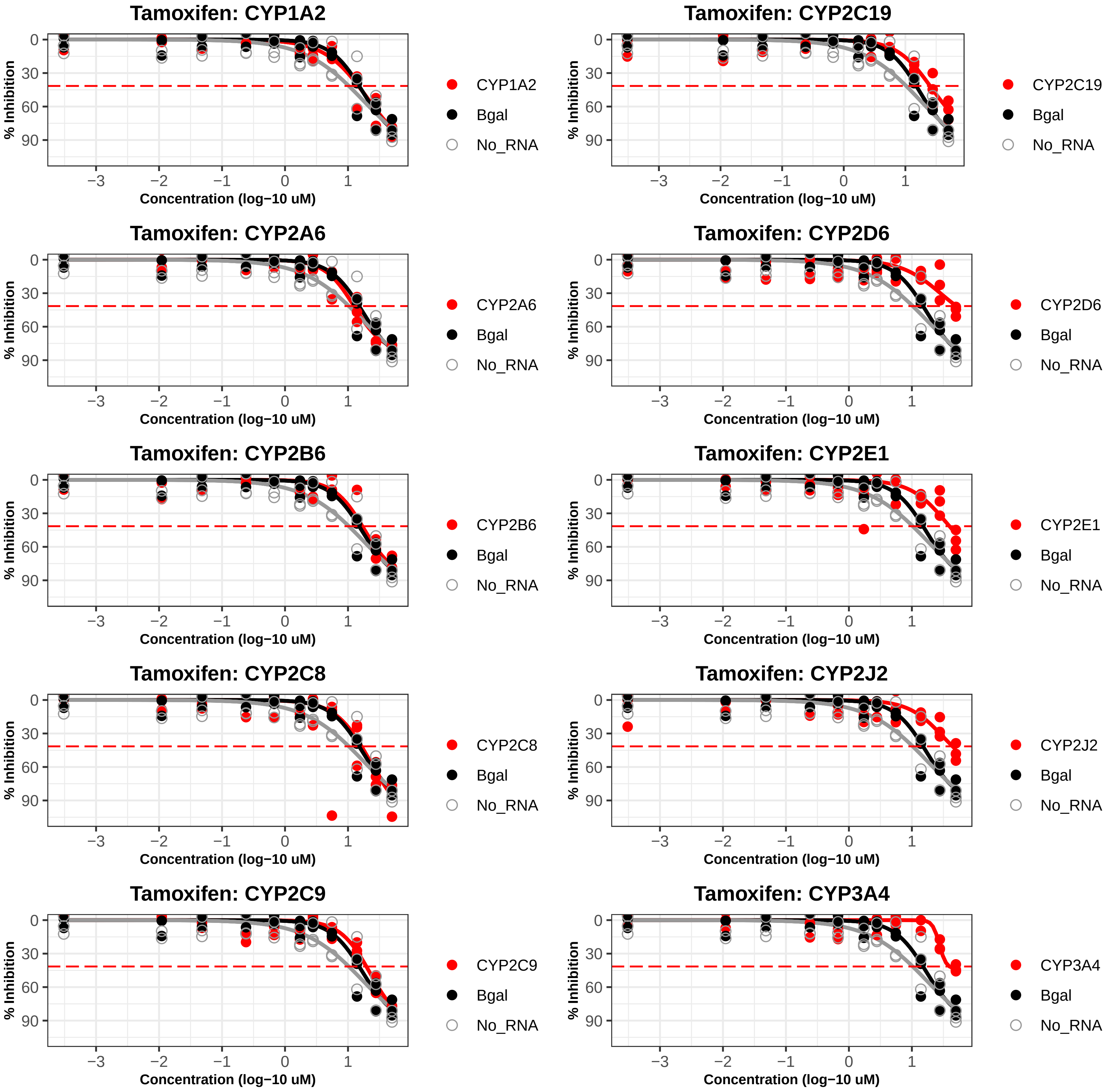

Tamoxifen: CYP1A2
CYP1A2
Bgal
No_RNA
Tamoxifen: CYP2C19
CYP2C19
Bgal
No_RNA
Tamoxifen: CYP2A6
CYP2A6
Bgal
No_RNA
Tamoxifen: CYP2D6
CYP2D6
Bgal
No_RNA
Tamoxifen: CYP2B6
CYP2B6
Bgal
No_RNA
Tamoxifen: CYP2E1
CYP2E1
Bgal
No_RNA
Tamoxifen: CYP2C8
CYP2C8
Bgal
No_RNA
Tamoxifen: CYP2J2
CYP2J2
Bgal
No_RNA
Tamoxifen: CYP2C9
CYP2C9
Bgal
No_RNA
Tamoxifen: CYP3A4
CYP3A4
Bgal
No_RNA
% Inhibition
Concentration (log−10 uM)

Tebuconazole: CYP1A2

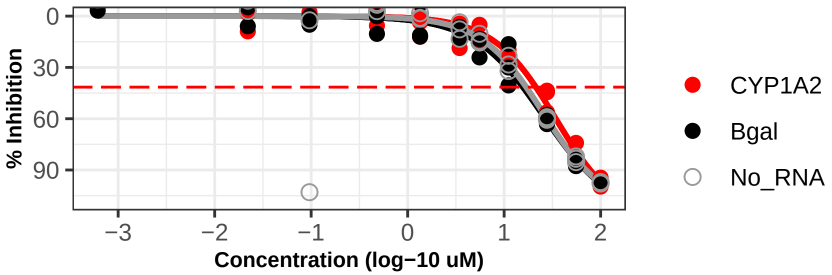

Tebuconazole: CYP2C19

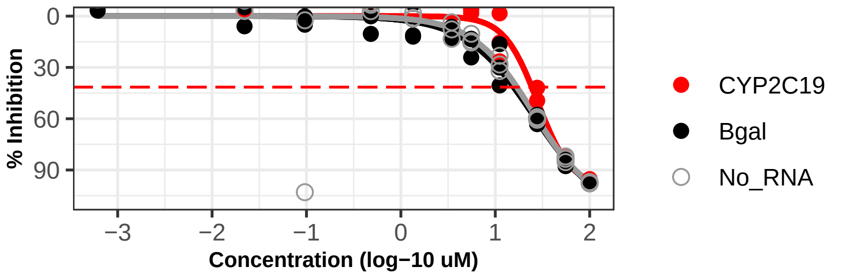

Tebuconazole: CYP2A6

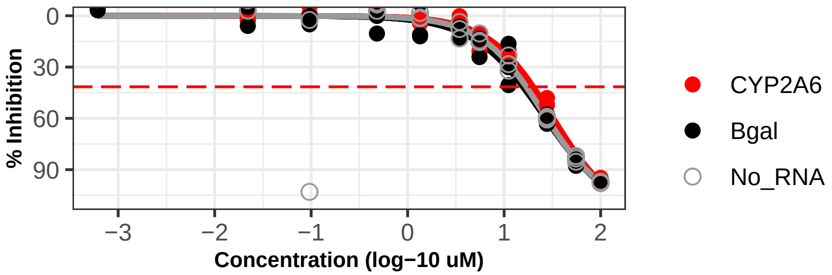

Tebuconazole: CYP2D6

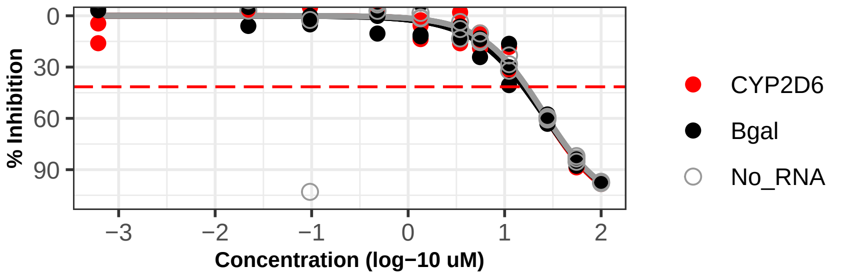

Tebuconazole: CYP2B6

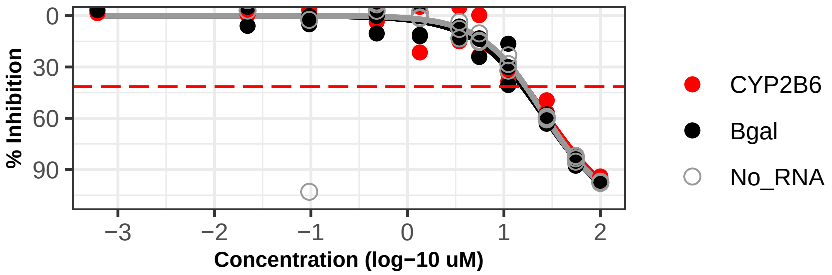

Tebuconazole: CYP2E1

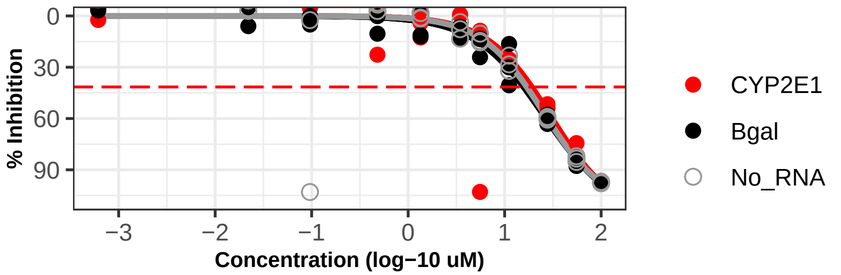

Tebuconazole: CYP2C8

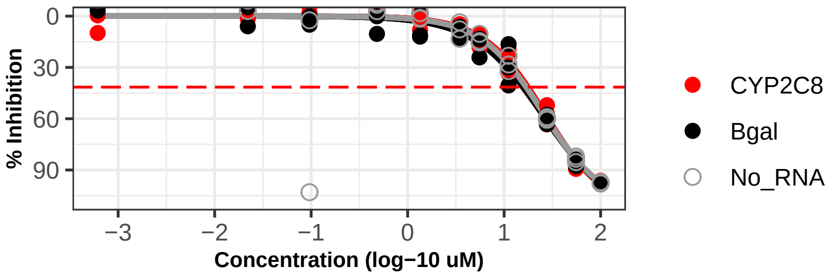

Tebuconazole: CYP2J2

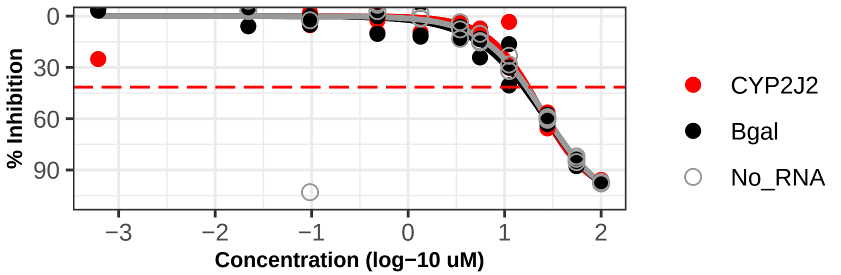

Tebuconazole: CYP2C9

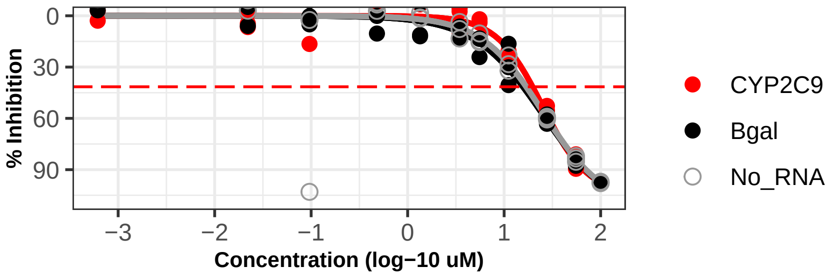

Tebuconazole: CYP3A4

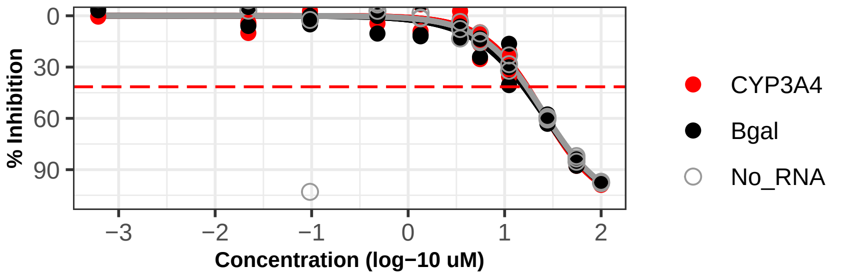

Testosterone propionate: CYP1A2

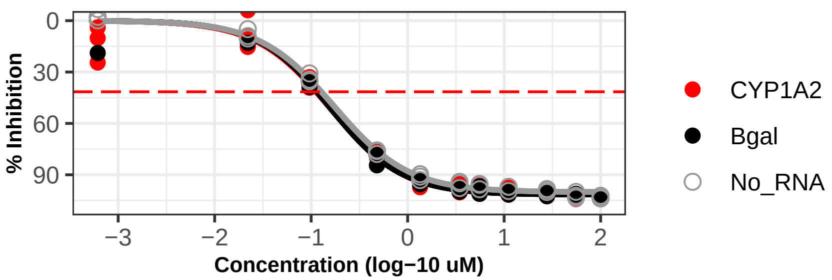

Testosterone propionate: CYP2C19

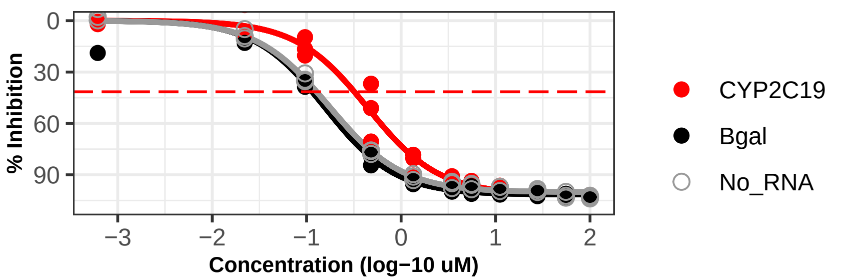

Testosterone propionate: CYP2A6

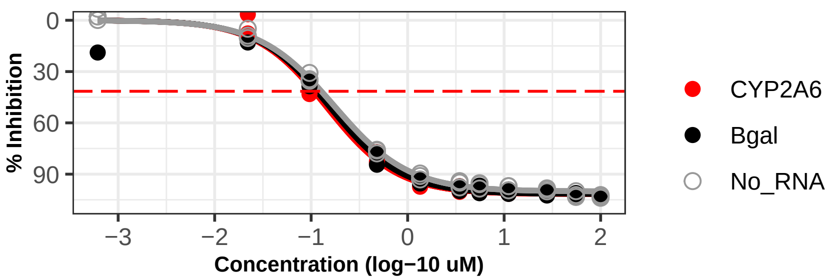

Testosterone propionate: CYP2D6

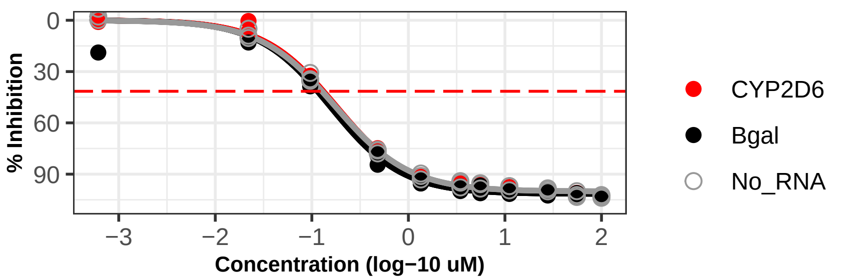

Testosterone propionate: CYP2B6

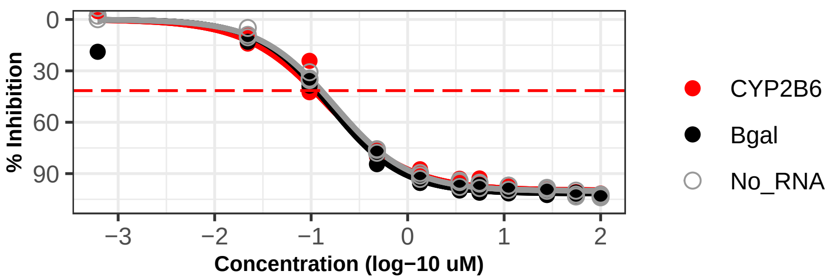

Testosterone propionate: CYP2E1

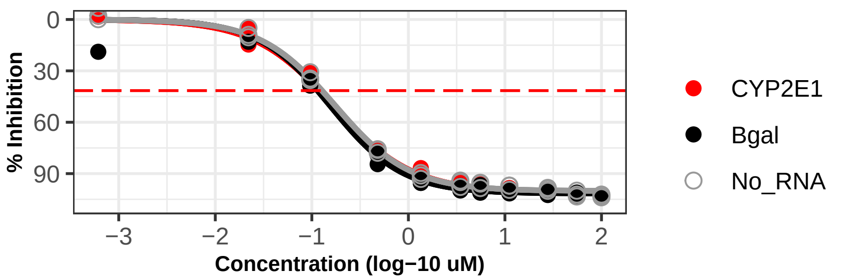

Testosterone propionate: CYP2C8

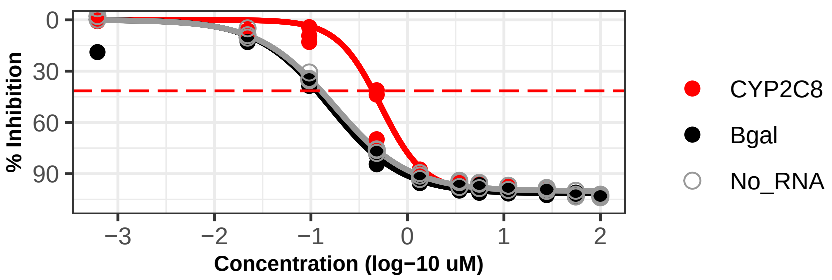

Testosterone propionate: CYP2J2

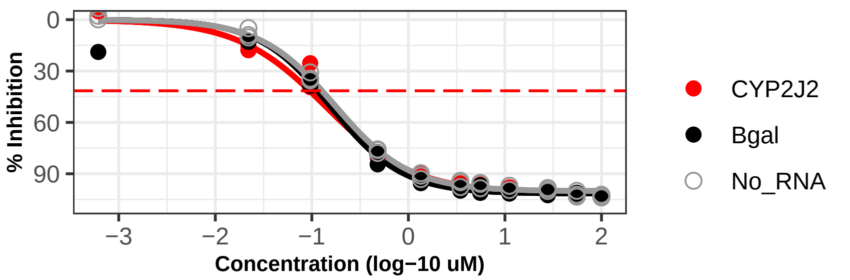

Testosterone propionate: CYP2C9

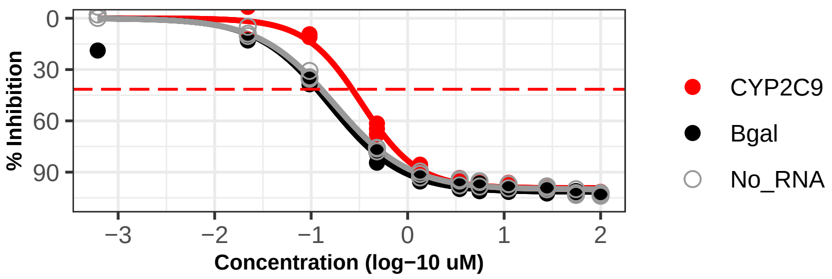

Testosterone propionate: CYP3A4

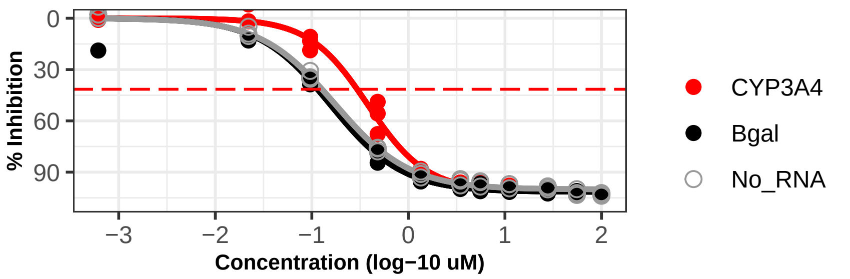

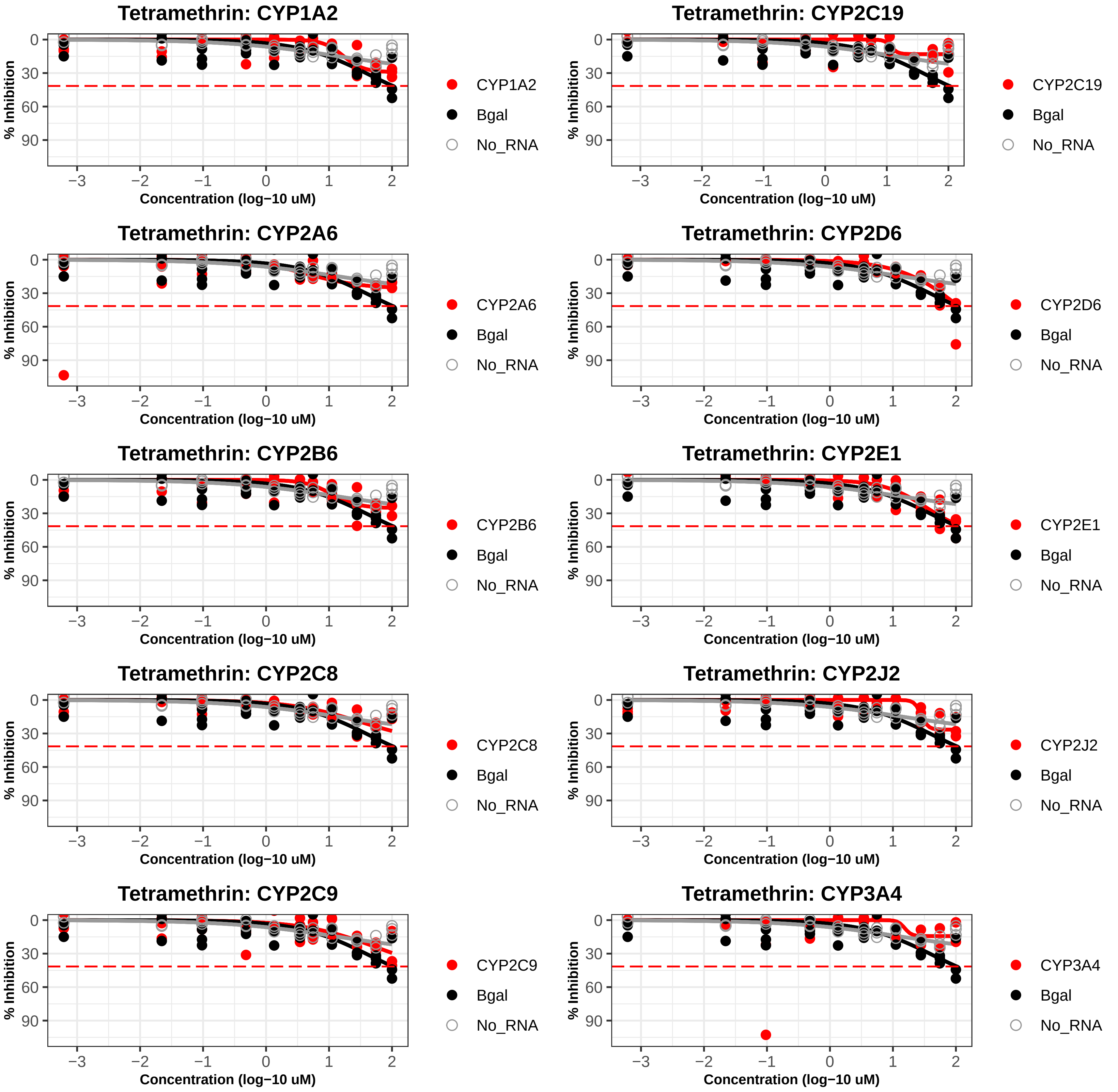
Tetramethrin: CYP1A2
Tetramethrin: CYP2C19
Tetramethrin: CYP2A6
Tetramethrin: CYP2D6
Tetramethrin: CYP2B6
Tetramethrin: CYP2E1
Tetramethrin: CYP2C8
Tetramethrin: CYP2J2
Tetramethrin: CYP2C9
Tetramethrin: CYP3A4
% Inhibition
0
30
60
90
Concentration (log−10 uM)
−3
−2
−1
0
1
2
CYP1A2
CYP2C19
CYP2A6
CYP2D6
CYP2B6
CYP2E1
CYP2C8
CYP2J2
CYP2C9
CYP3A4
Bgal
No_RNA

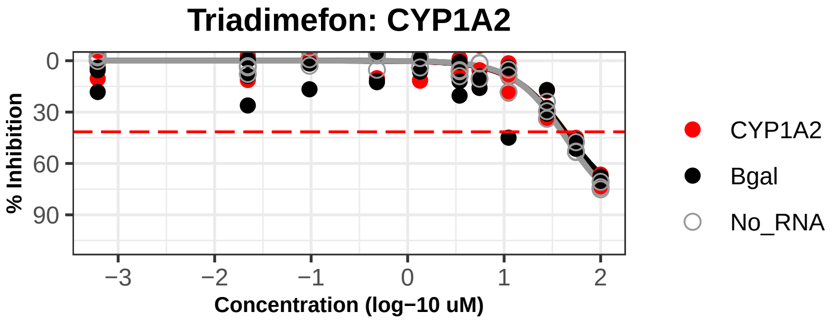
Triadimefon: CYP1A2
% Inhibition
0
30
60
90
−3
−2
−1
0
1
2
Concentration (log−10 uM)
CYP1A2
Bgal
No_RNA

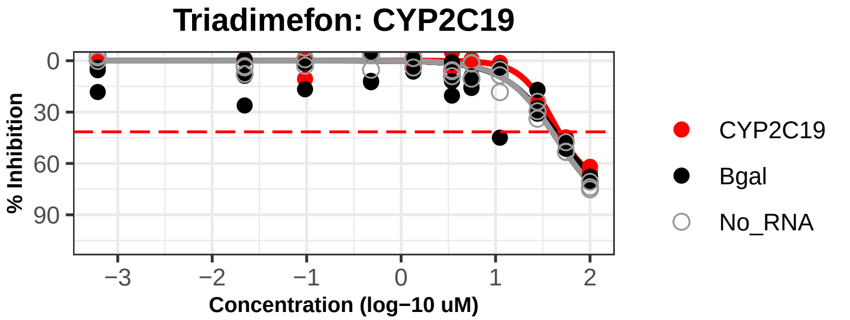
Triadimefon: CYP2C19
% Inhibition
0
30
60
90
−3
−2
−1
0
1
2
Concentration (log−10 uM)
CYP2C19
Bgal
No_RNA

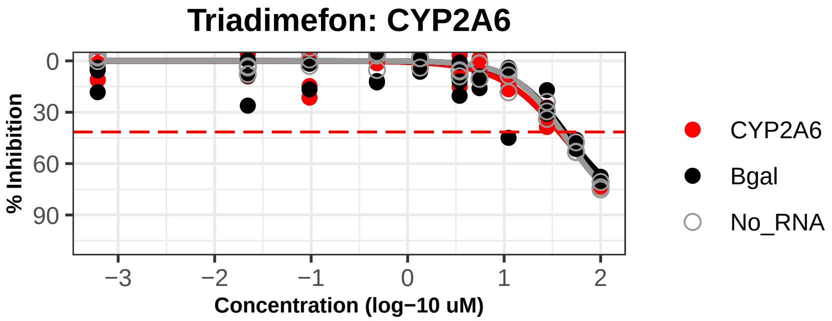
Triadimefon: CYP2A6
% Inhibition
0
30
60
90
−3
−2
−1
0
1
2
Concentration (log−10 uM)
CYP2A6
Bgal
No_RNA

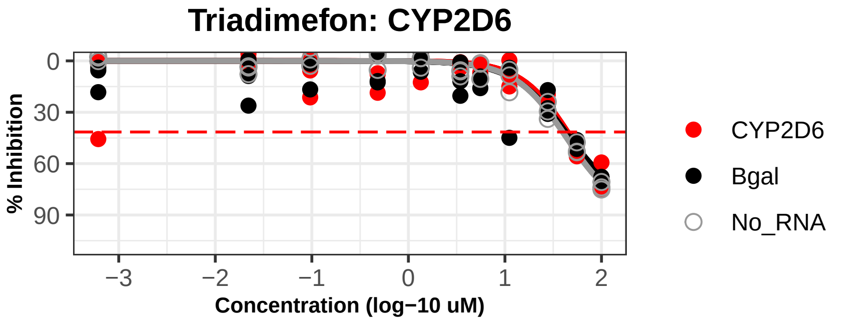
Triadimefon: CYP2D6
% Inhibition
0
30
60
90
−3
−2
−1
0
1
2
Concentration (log−10 uM)
CYP2D6
Bgal
No_RNA

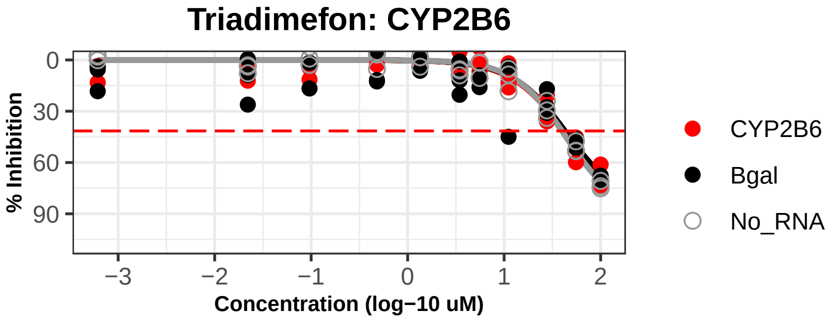
Triadimefon: CYP2B6
% Inhibition
0
30
60
90
−3
−2
−1
0
1
2
Concentration (log−10 uM)
CYP2B6
Bgal
No_RNA

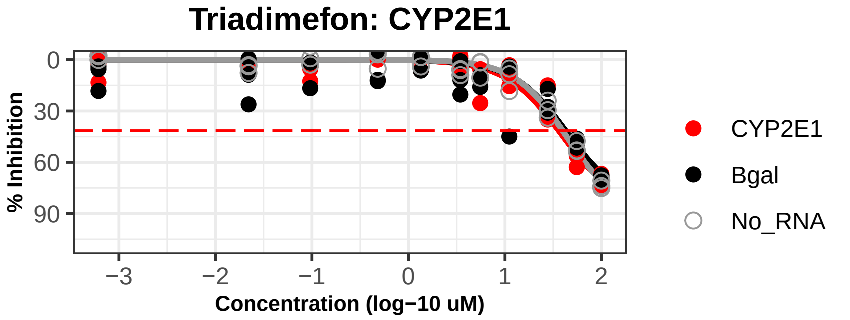
Triadimefon: CYP2E1
% Inhibition
0
30
60
90
−3
−2
−1
0
1
2
Concentration (log−10 uM)
CYP2E1
Bgal
No_RNA

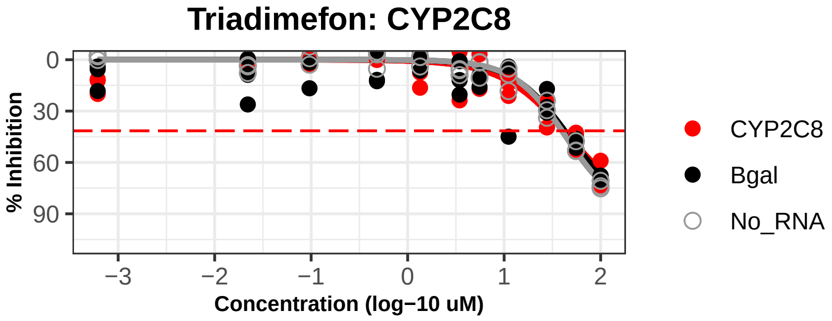
Triadimefon: CYP2C8
% Inhibition
0
30
60
90
−3
−2
−1
0
1
2
Concentration (log−10 uM)
CYP2C8
Bgal
No_RNA

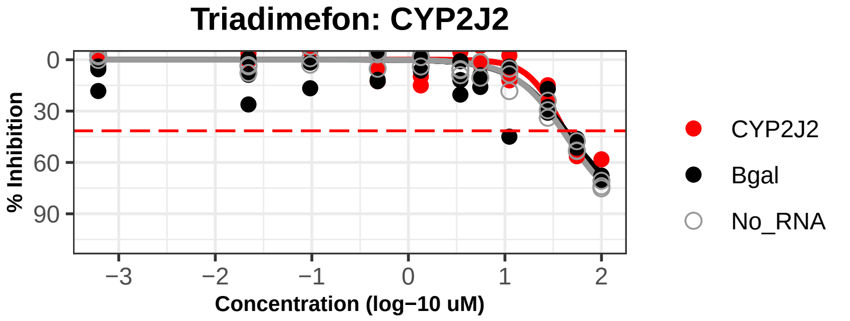
Triadimefon: CYP2J2
% Inhibition
0
30
60
90
−3
−2
−1
0
1
2
Concentration (log−10 uM)
CYP2J2
Bgal
No_RNA

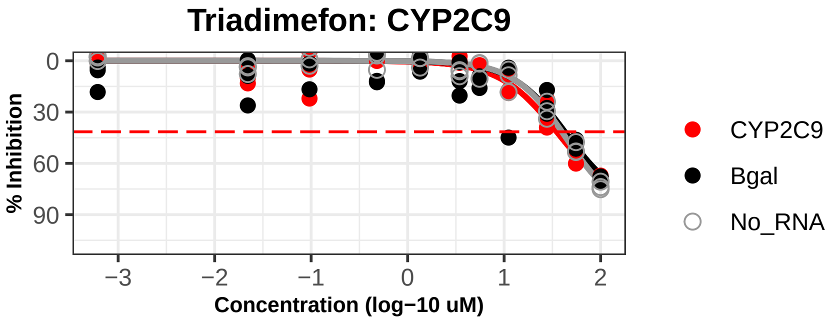
Triadimefon: CYP2C9
% Inhibition
0
30
60
90
−3
−2
−1
0
1
2
Concentration (log−10 uM)
CYP2C9
Bgal
No_RNA

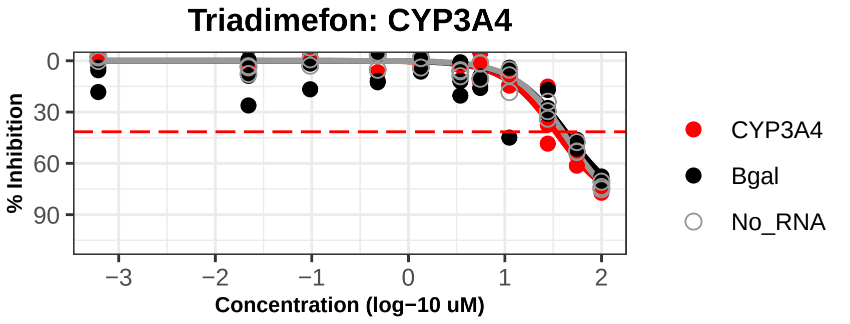
Triadimefon: CYP3A4
% Inhibition
0
30
60
90
−3
−2
−1
0
1
2
Concentration (log−10 uM)
CYP3A4
Bgal
No_RNA

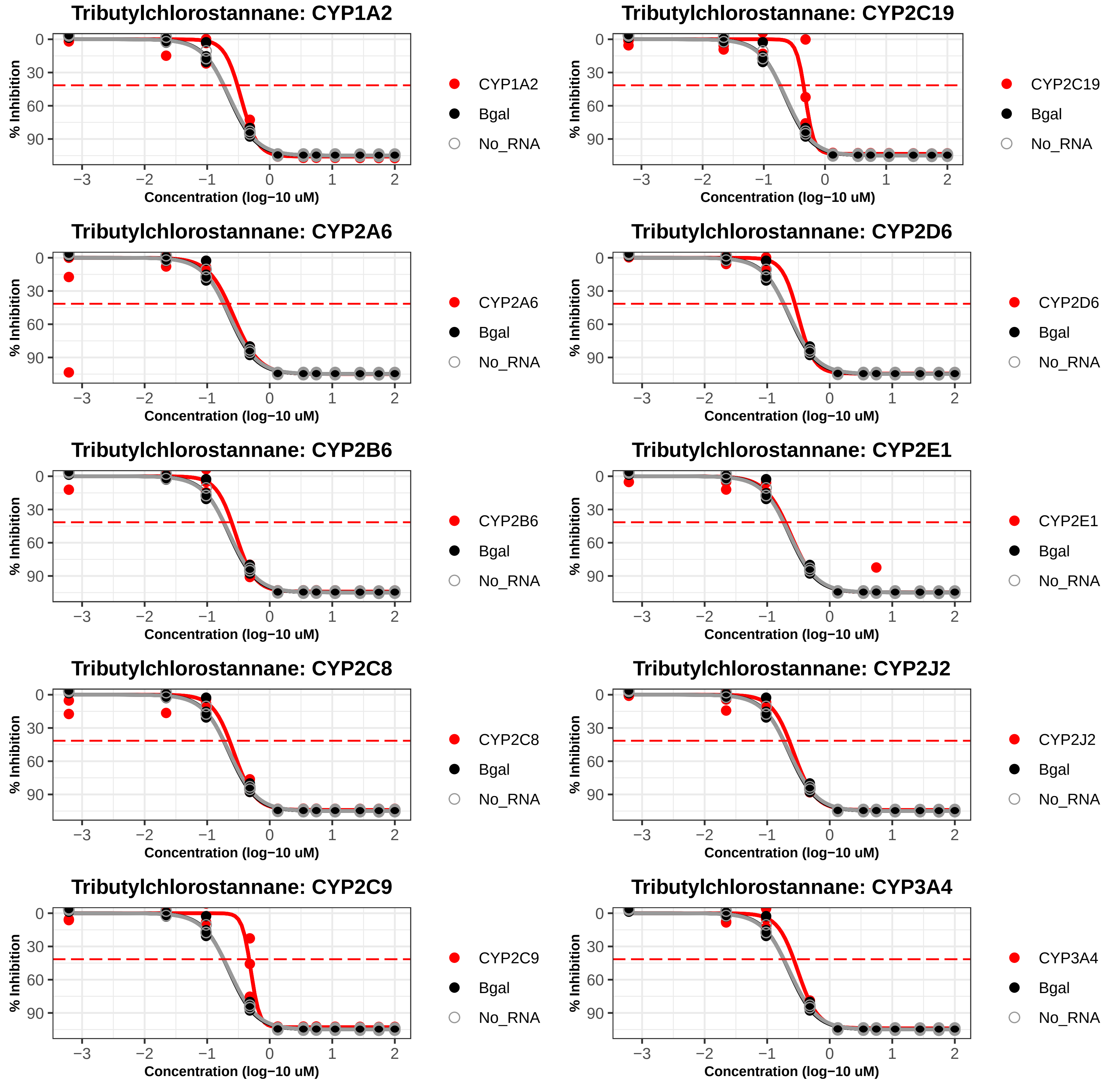
Tributylchlorostannane: CYP1A2
% Inhibition
Concentration (log−10 uM)
CYP1A2
Bgal
No_RNA
Tributylchlorostannane: CYP2C19
% Inhibition
Concentration (log−10 uM)
CYP2C19
Bgal
No_RNA
Tributylchlorostannane: CYP2A6
% Inhibition
Concentration (log−10 uM)
CYP2A6
Bgal
No_RNA
Tributylchlorostannane: CYP2D6
% Inhibition
Concentration (log−10 uM)
CYP2D6
Bgal
No_RNA
Tributylchlorostannane: CYP2B6
% Inhibition
Concentration (log−10 uM)
CYP2B6
Bgal
No_RNA
Tributylchlorostannane: CYP2E1
% Inhibition
Concentration (log−10 uM)
CYP2E1
Bgal
No_RNA
Tributylchlorostannane: CYP2C8
% Inhibition
Concentration (log−10 uM)
CYP2C8
Bgal
No_RNA
Tributylchlorostannane: CYP2J2
% Inhibition
Concentration (log−10 uM)
CYP2J2
Bgal
No_RNA
Tributylchlorostannane: CYP2C9
% Inhibition
Concentration (log−10 uM)
CYP2C9
Bgal
No_RNA
Tributylchlorostannane: CYP3A4
% Inhibition
Concentration (log−10 uM)
CYP3A4
Bgal
No_RNA

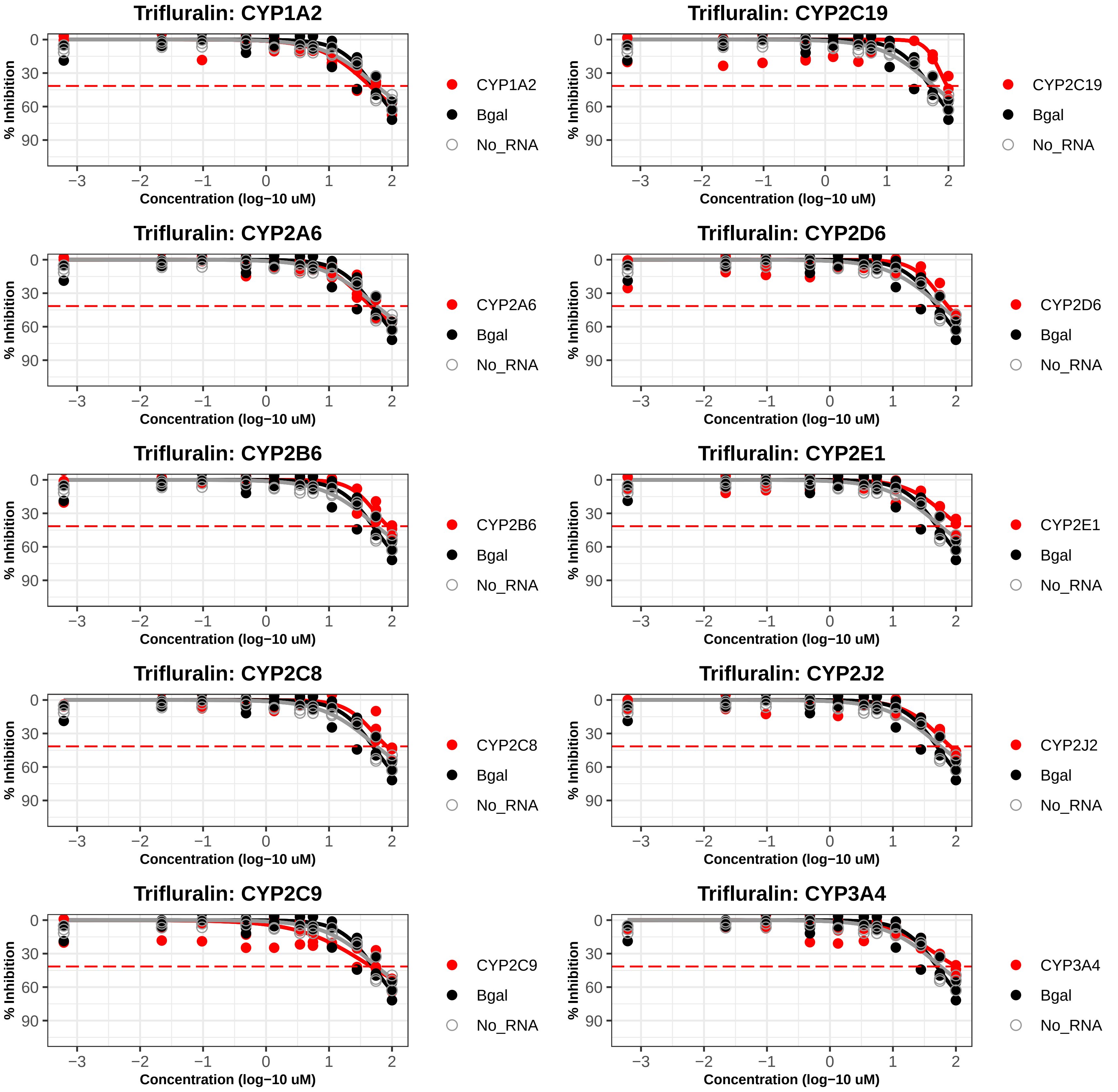
Trifluralin: CYP1A2
Trifluralin: CYP2C19
Trifluralin: CYP2A6
Trifluralin: CYP2D6
Trifluralin: CYP2B6
Trifluralin: CYP2E1
Trifluralin: CYP2C8
Trifluralin: CYP2J2
Trifluralin: CYP2C9
Trifluralin: CYP3A4
% Inhibition
Concentration (log−10 uM)
CYP1A2
CYP2C19
CYP2A6
CYP2D6
CYP2B6
CYP2E1
CYP2C8
CYP2J2
CYP2C9
CYP3A4
Bgal
No_RNA

Vinclozolin: CYP1A2

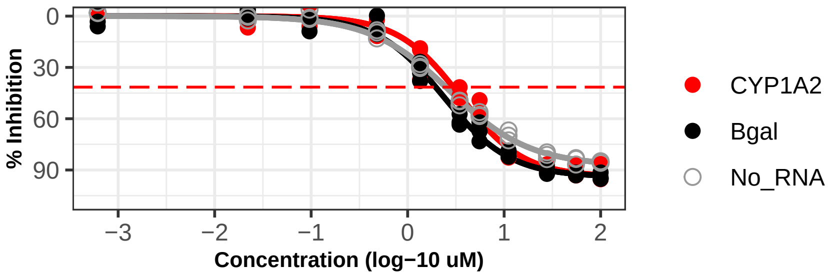

Vinclozolin: CYP2A6

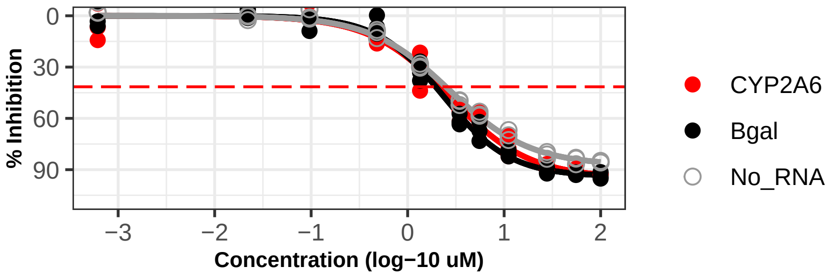

Vinclozolin: CYP2B6

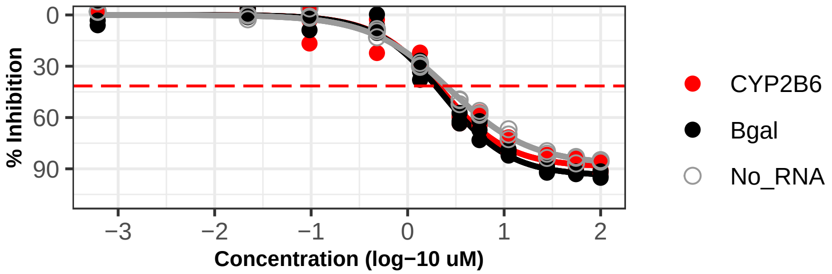

Vinclozolin: CYP2C8

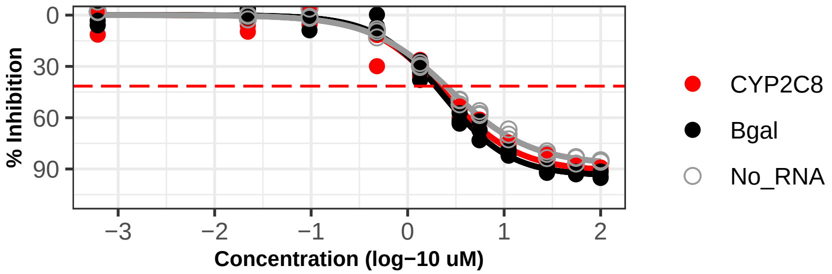

Vinclozolin: CYP2C9

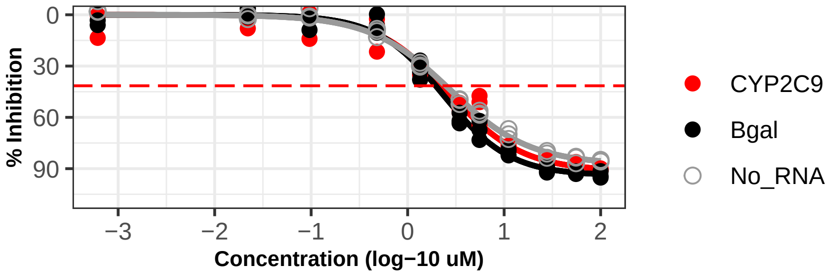

Vinclozolin: CYP2C19

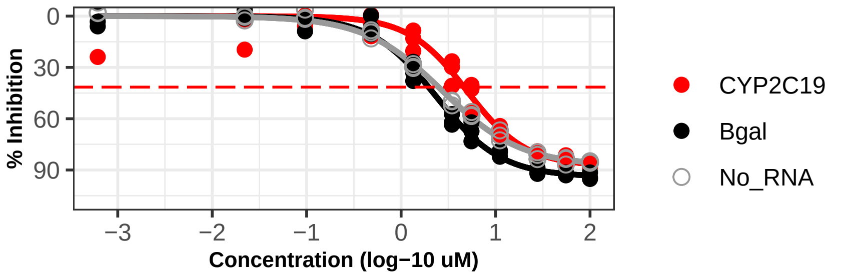

Vinclozolin: CYP2D6

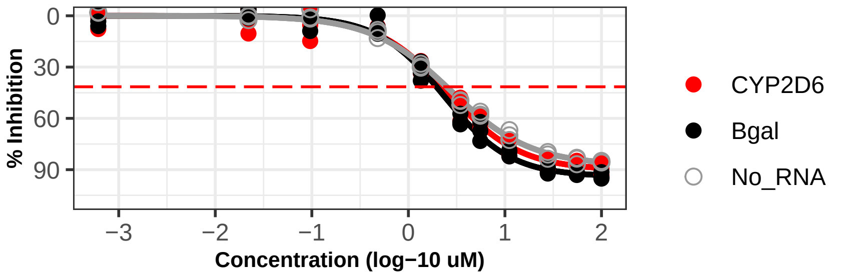

Vinclozolin: CYP2E1

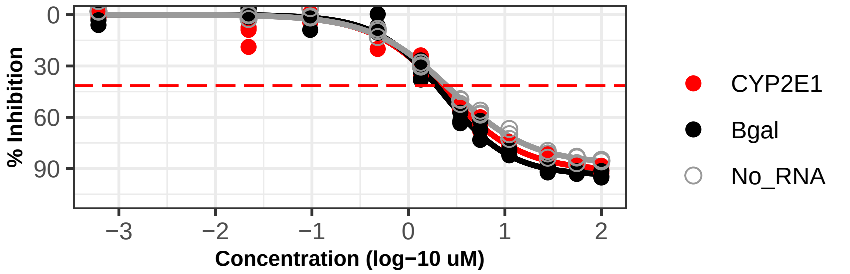

Vinclozolin: CYP2J2

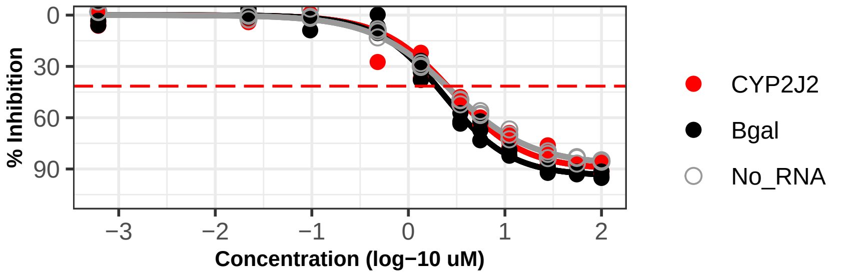

Vinclozolin: CYP3A4

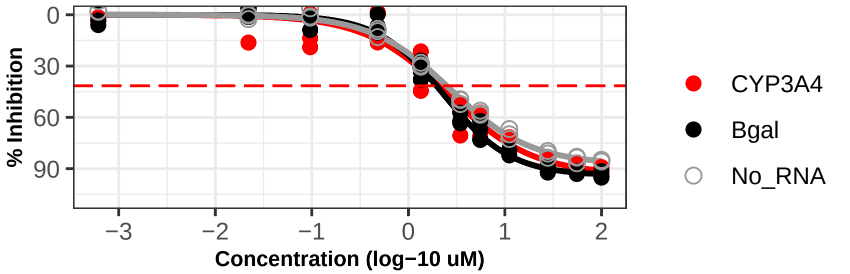

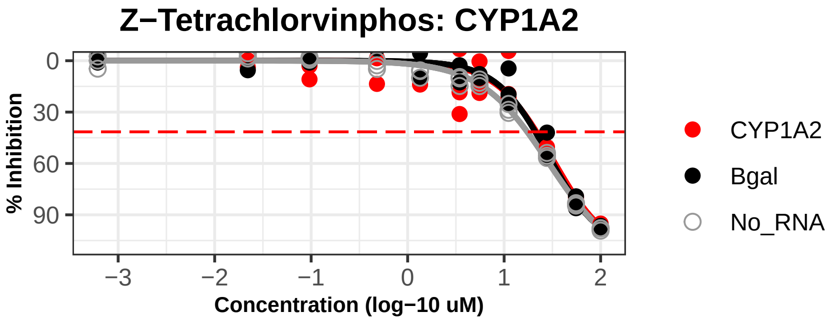
Z−Tetrachlorvinphos: CYP1A2
% Inhibition
0
30
60
90
−3
−2
−1
0
1
2
Concentration (log−10 uM)
CYP1A2
Bgal
No_RNA

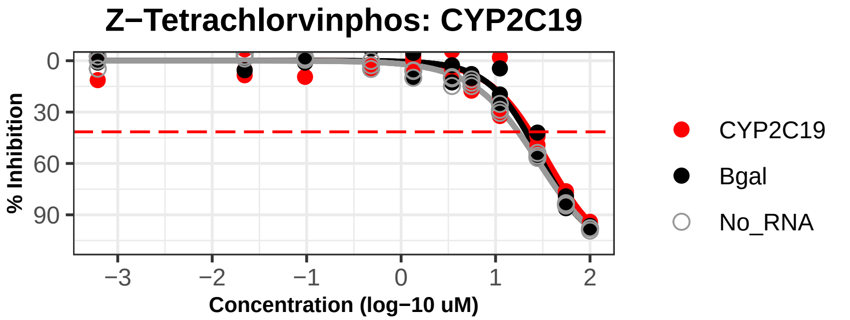
Z−Tetrachlorvinphos: CYP2C19
% Inhibition
0
30
60
90
−3
−2
−1
0
1
2
Concentration (log−10 uM)
CYP2C19
Bgal
No_RNA

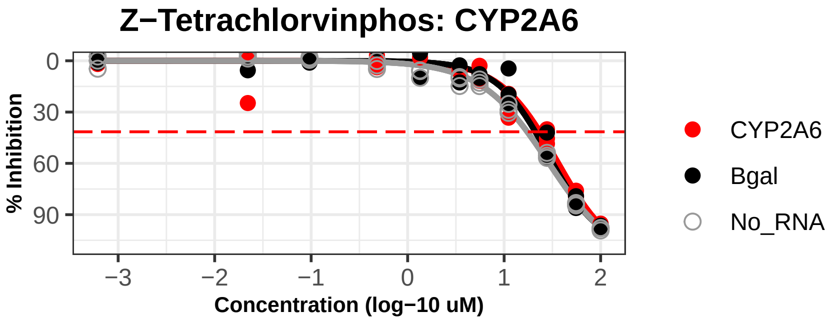
Z−Tetrachlorvinphos: CYP2A6
% Inhibition
0
30
60
90
−3
−2
−1
0
1
2
Concentration (log−10 uM)
CYP2A6
Bgal
No_RNA

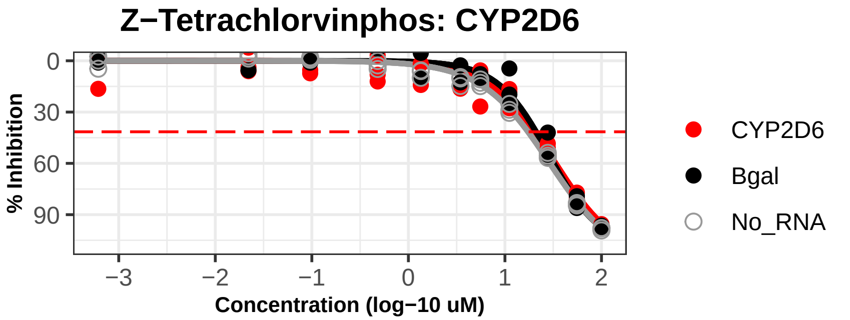
Z−Tetrachlorvinphos: CYP2D6
% Inhibition
0
30
60
90
−3
−2
−1
0
1
2
Concentration (log−10 uM)
CYP2D6
Bgal
No_RNA

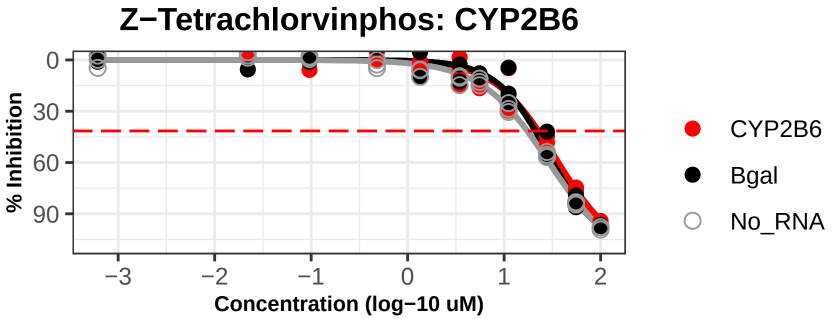
Z−Tetrachlorvinphos: CYP2B6
% Inhibition
0
30
60
90
−3
−2
−1
0
1
2
Concentration (log−10 uM)
CYP2B6
Bgal
No_RNA

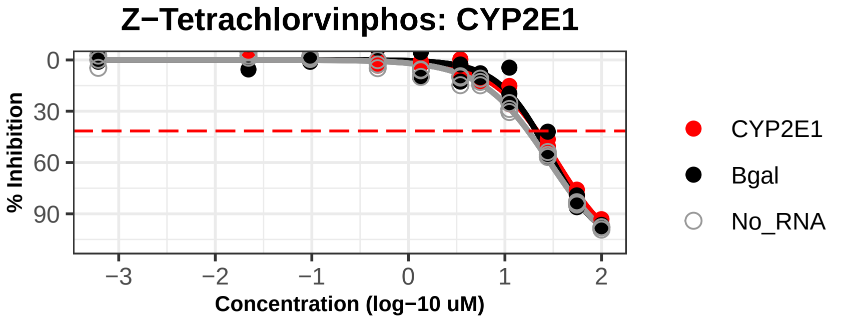
Z−Tetrachlorvinphos: CYP2E1
% Inhibition
0
30
60
90
−3
−2
−1
0
1
2
Concentration (log−10 uM)
CYP2E1
Bgal
No_RNA

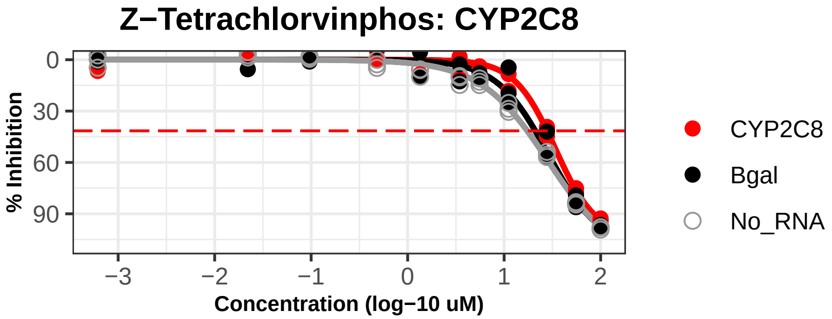
Z−Tetrachlorvinphos: CYP2C8
% Inhibition
0
30
60
90
−3
−2
−1
0
1
2
Concentration (log−10 uM)
CYP2C8
Bgal
No_RNA

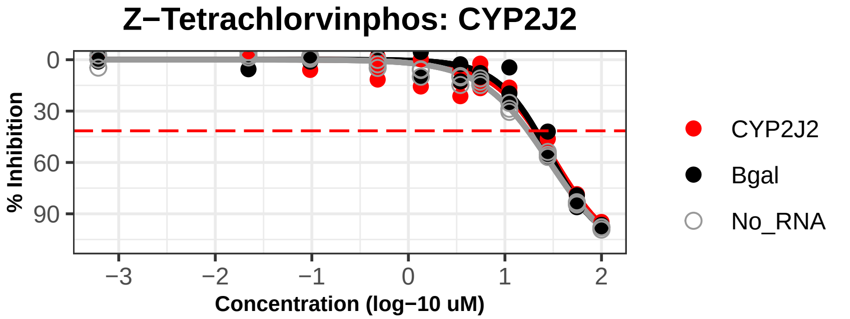
Z−Tetrachlorvinphos: CYP2J2
% Inhibition
0
30
60
90
−3
−2
−1
0
1
2
Concentration (log−10 uM)
CYP2J2
Bgal
No_RNA

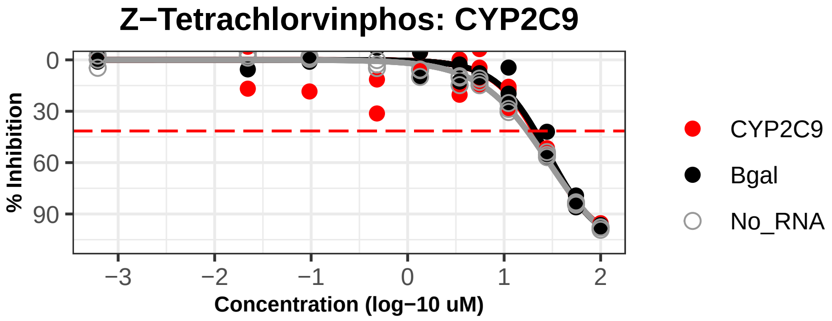
Z−Tetrachlorvinphos: CYP2C9
% Inhibition
0
30
60
90
−3
−2
−1
0
1
2
Concentration (log−10 uM)
CYP2C9
Bgal
No_RNA

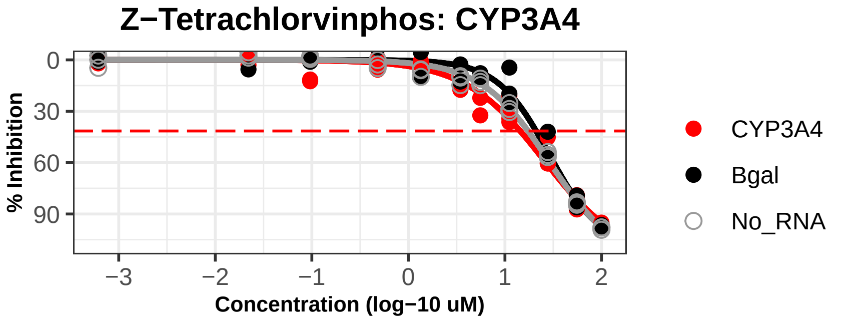
Z−Tetrachlorvinphos: CYP3A4
% Inhibition
0
30
60
90
−3
−2
−1
0
1
2
Concentration (log−10 uM)
CYP3A4
Bgal
No_RNA

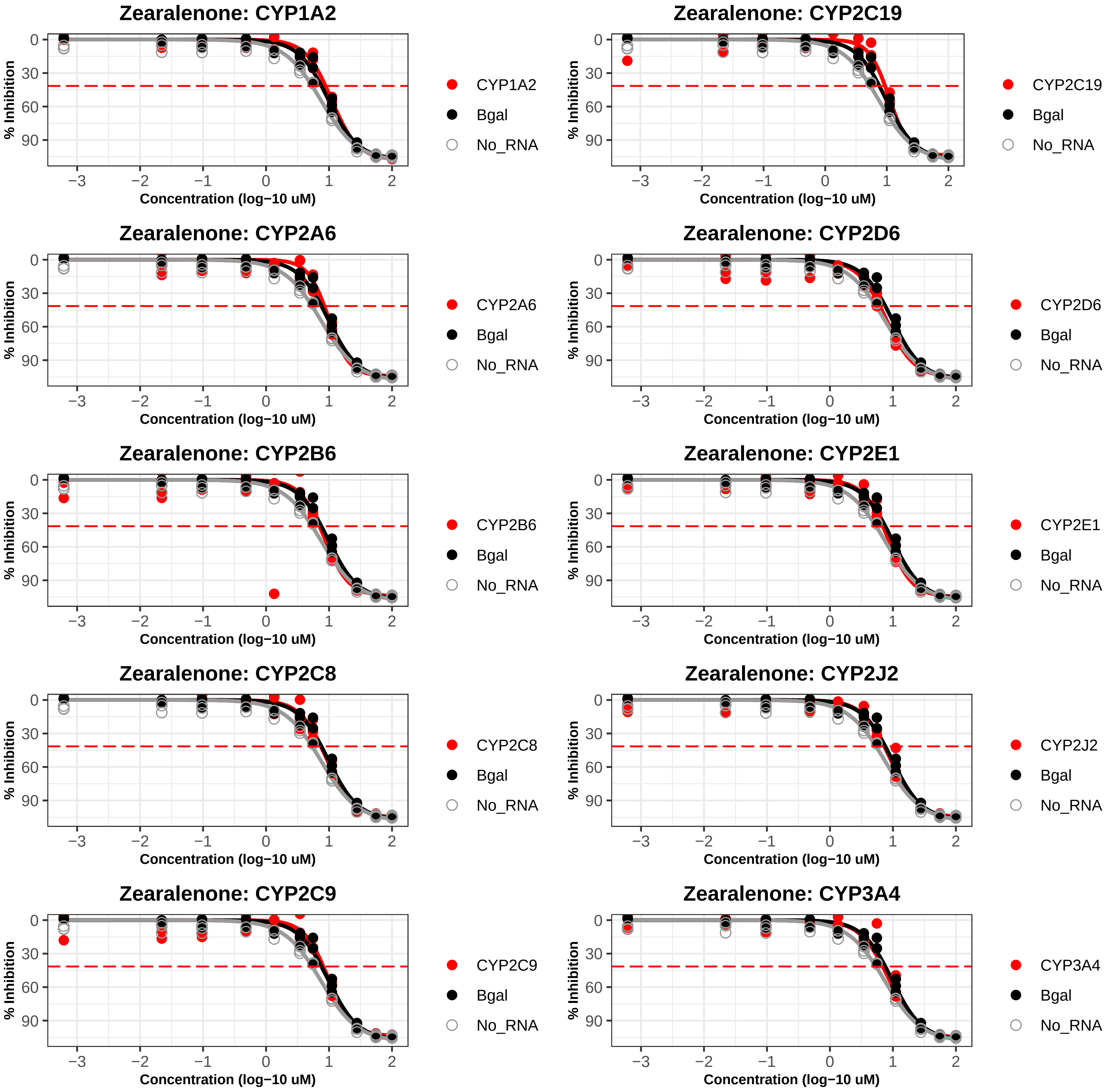
Zearalenone: CYP1A2
% Inhibition
Concentration (log−10 uM)
CYP1A2
Bgal
No_RNA
Zearalenone: CYP2C19
% Inhibition
Concentration (log−10 uM)
CYP2C19
Bgal
No_RNA
Zearalenone: CYP2A6
% Inhibition
Concentration (log−10 uM)
CYP2A6
Bgal
No_RNA
Zearalenone: CYP2D6
% Inhibition
Concentration (log−10 uM)
CYP2D6
Bgal
No_RNA
Zearalenone: CYP2B6
% Inhibition
Concentration (log−10 uM)
CYP2B6
Bgal
No_RNA
Zearalenone: CYP2E1
% Inhibition
Concentration (log−10 uM)
CYP2E1
Bgal
No_RNA
Zearalenone: CYP2C8
% Inhibition
Concentration (log−10 uM)
CYP2C8
Bgal
No_RNA
Zearalenone: CYP2J2
% Inhibition
Concentration (log−10 uM)
CYP2J2
Bgal
No_RNA
Zearalenone: CYP2C9
% Inhibition
Concentration (log−10 uM)
CYP2C9
Bgal
No_RNA
Zearalenone: CYP3A4
% Inhibition
Concentration (log−10 uM)
CYP3A4
Bgal
No_RNA

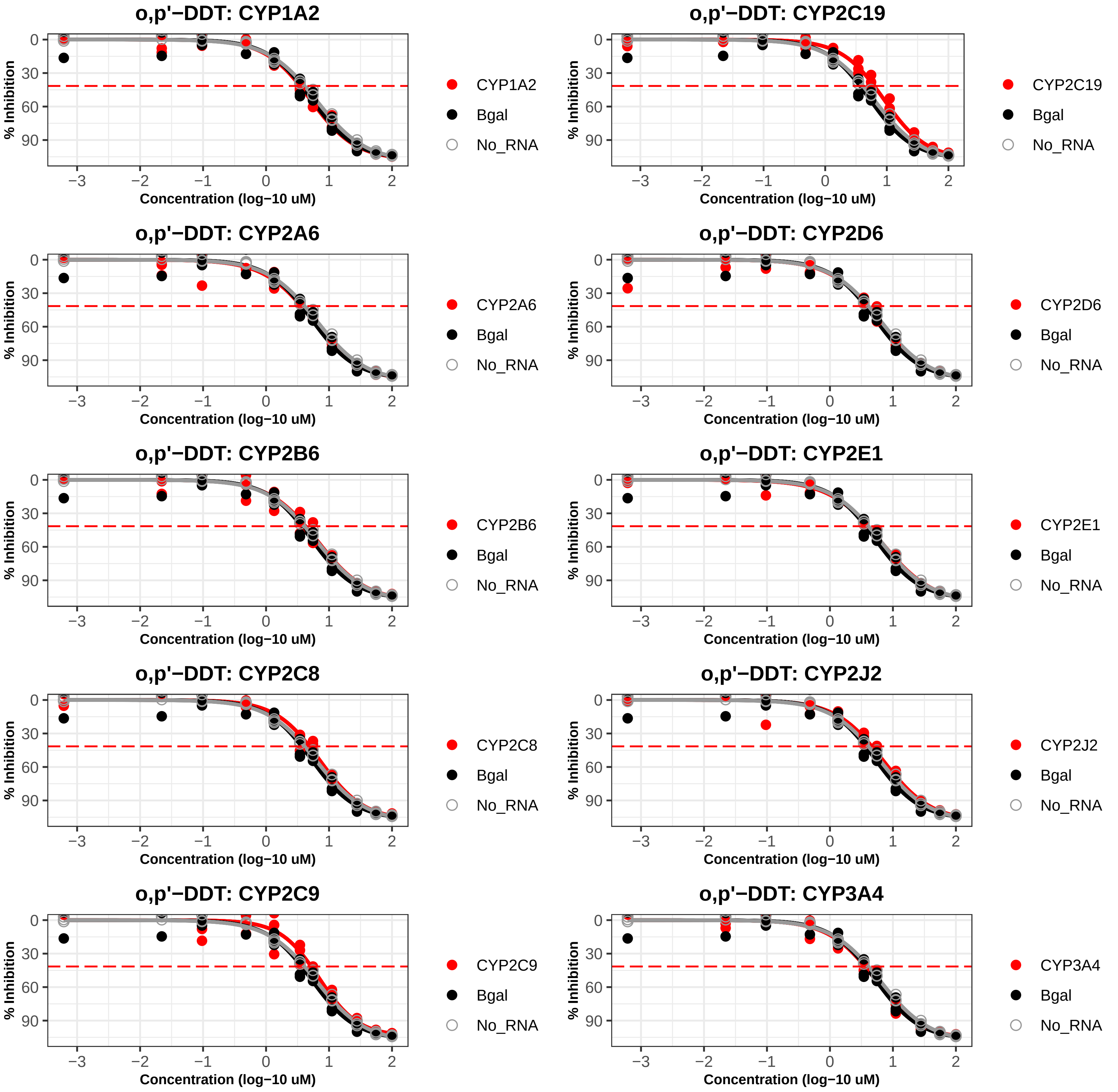
o,p'−DDT: CYP1A2
o,p'−DDT: CYP2C19
o,p'−DDT: CYP2A6
o,p'−DDT: CYP2D6
o,p'−DDT: CYP2B6
o,p'−DDT: CYP2E1
o,p'−DDT: CYP2C8
o,p'−DDT: CYP2J2
o,p'−DDT: CYP2C9
o,p'−DDT: CYP3A4
% Inhibition
Concentration (log−10 uM)
0
30
60
90
−3
−2
−1
0
1
2
CYP1A2
CYP2C19
CYP2A6
CYP2D6
CYP2B6
CYP2E1
CYP2C8
CYP2J2
CYP2C9
CYP3A4
Bgal
No_RNA

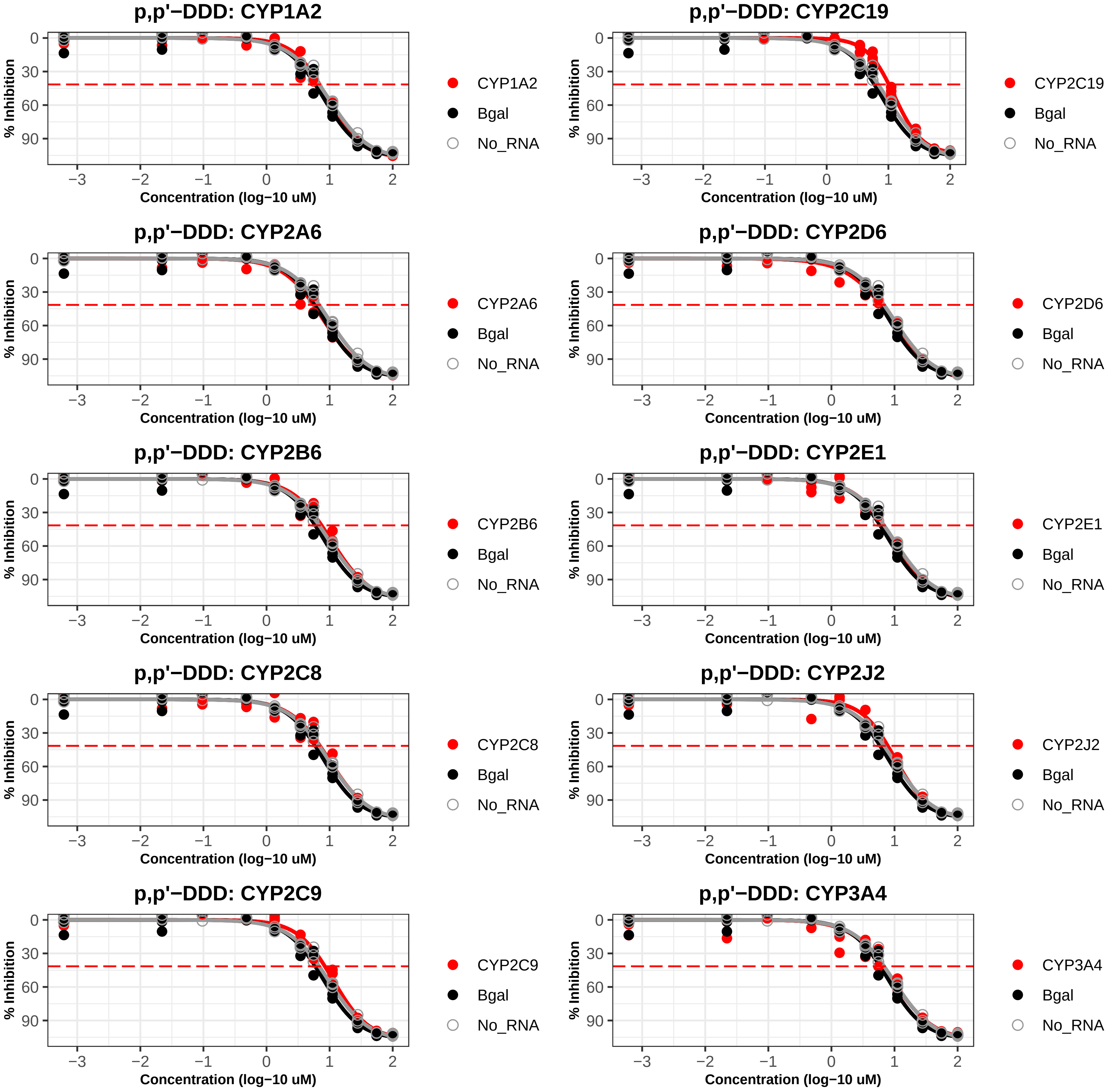
p,p'−DDD: CYP1A2
CYP1A2
Bgal
No_RNA
p,p'−DDD: CYP2C19
CYP2C19
Bgal
No_RNA
p,p'−DDD: CYP2A6
CYP2A6
Bgal
No_RNA
p,p'−DDD: CYP2D6
CYP2D6
Bgal
No_RNA
p,p'−DDD: CYP2B6
CYP2B6
Bgal
No_RNA
p,p'−DDD: CYP2E1
CYP2E1
Bgal
No_RNA
p,p'−DDD: CYP2C8
CYP2C8
Bgal
No_RNA
p,p'−DDD: CYP2J2
CYP2J2
Bgal
No_RNA
p,p'−DDD: CYP2C9
CYP2C9
Bgal
No_RNA
p,p'−DDD: CYP3A4
CYP3A4
Bgal
No_RNA
% Inhibition
Concentration (log−10 uM)

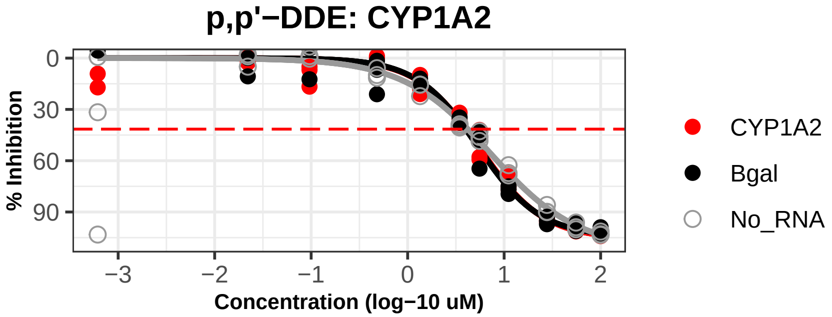
p,p'−DDE: CYP1A2
% Inhibition
Concentration (log−10 uM)
CYP1A2
Bgal
No_RNA

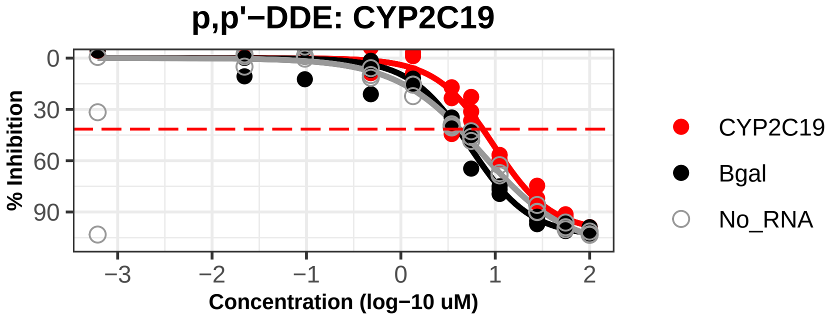
p,p'−DDE: CYP2C19
% Inhibition
Concentration (log−10 uM)
CYP2C19
Bgal
No_RNA

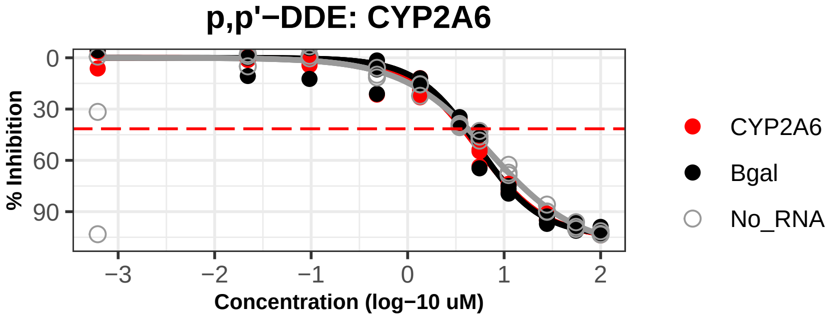
p,p'−DDE: CYP2A6
% Inhibition
Concentration (log−10 uM)
CYP2A6
Bgal
No_RNA

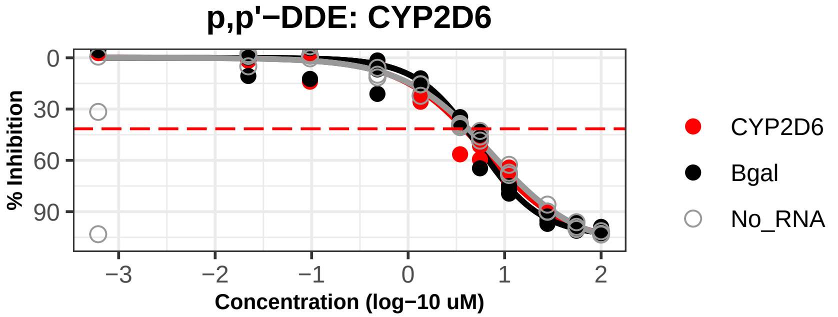
p,p'−DDE: CYP2D6
% Inhibition
Concentration (log−10 uM)
CYP2D6
Bgal
No_RNA

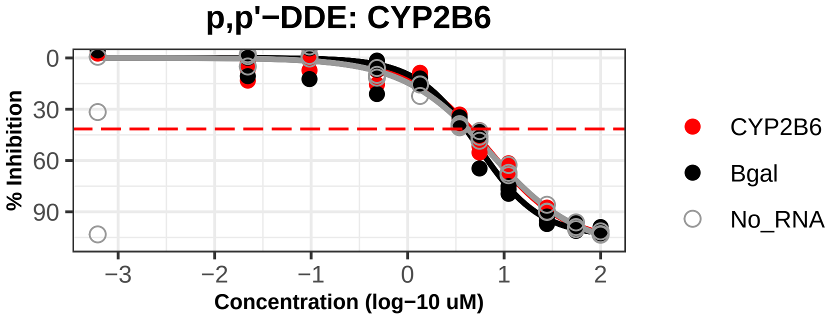
p,p'−DDE: CYP2B6
% Inhibition
Concentration (log−10 uM)
CYP2B6
Bgal
No_RNA

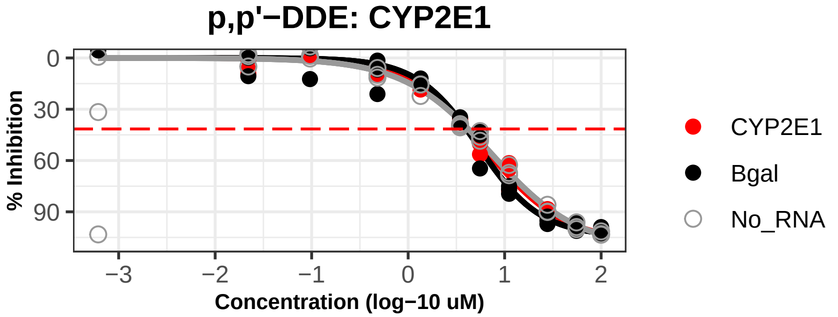
p,p'−DDE: CYP2E1
% Inhibition
Concentration (log−10 uM)
CYP2E1
Bgal
No_RNA

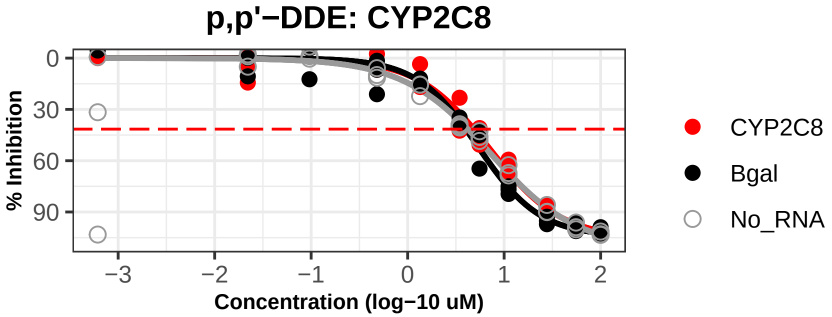
p,p'−DDE: CYP2C8
% Inhibition
Concentration (log−10 uM)
CYP2C8
Bgal
No_RNA

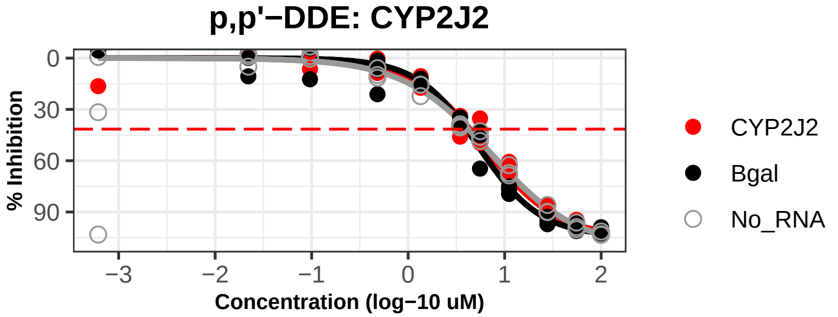
p,p'−DDE: CYP2J2
% Inhibition
Concentration (log−10 uM)
CYP2J2
Bgal
No_RNA

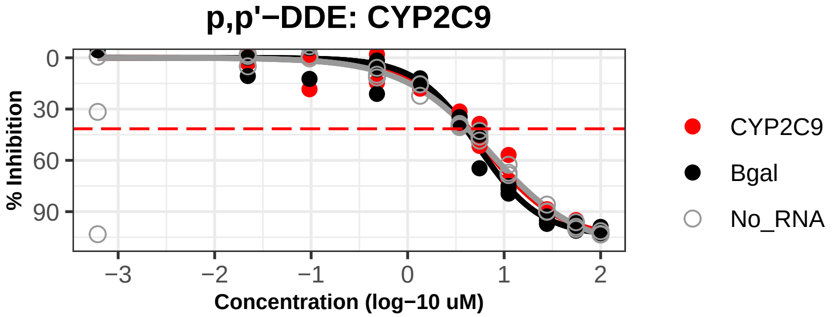
p,p'−DDE: CYP2C9
% Inhibition
Concentration (log−10 uM)
CYP2C9
Bgal
No_RNA

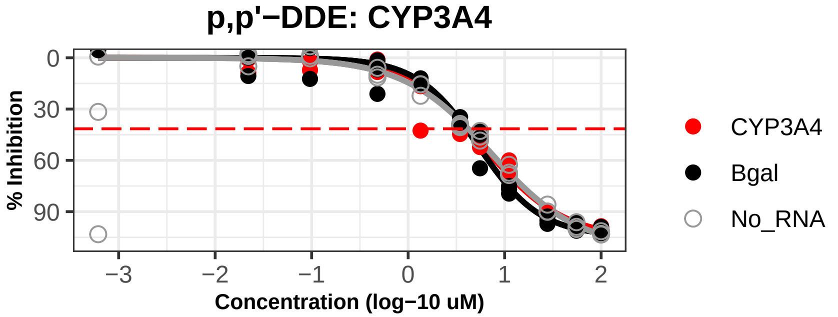
p,p'−DDE: CYP3A4
% Inhibition
Concentration (log−10 uM)
CYP3A4
Bgal
No_RNA

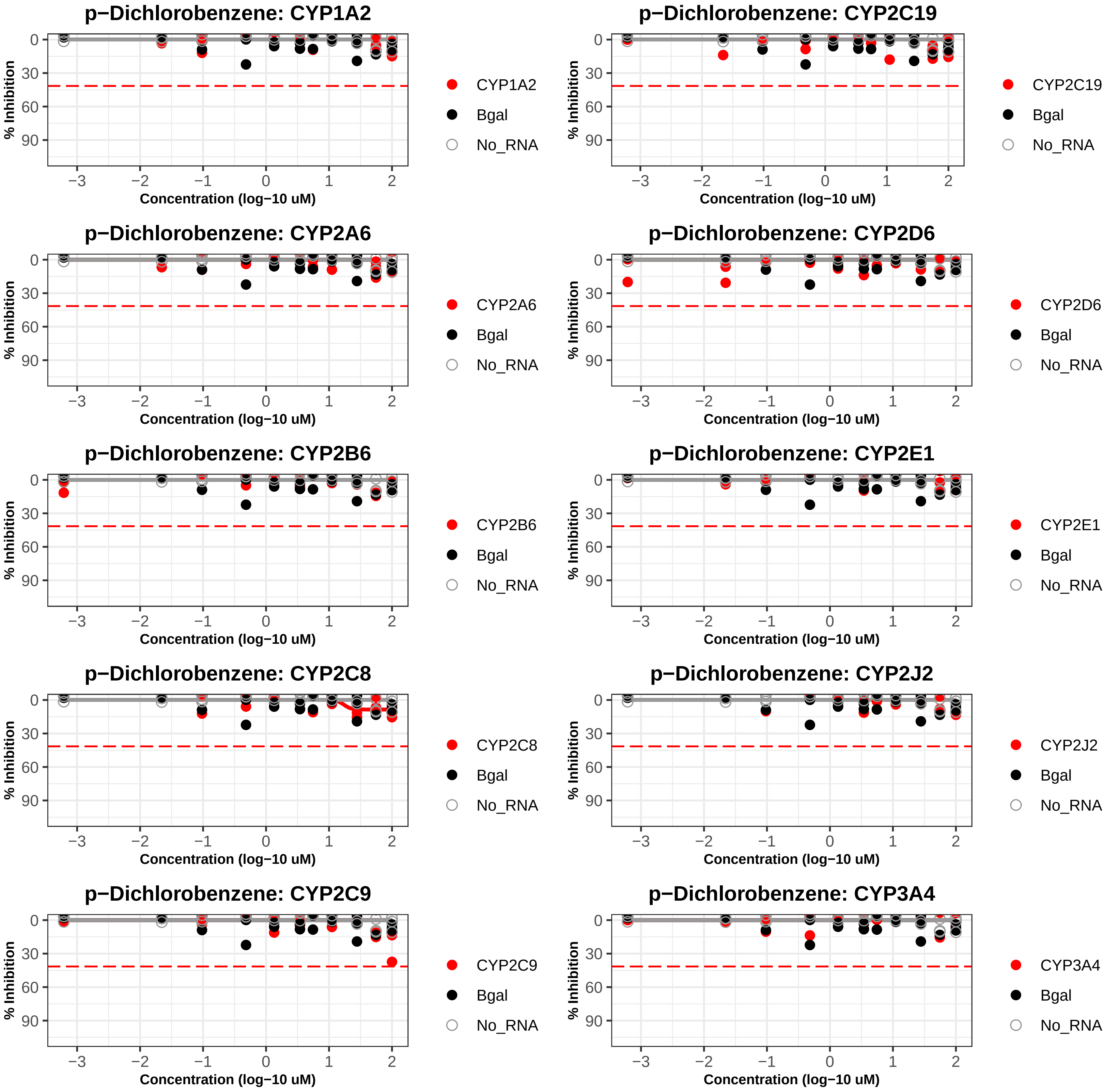
p−Dichlorobenzene: CYP1A2
p−Dichlorobenzene: CYP2C19
p−Dichlorobenzene: CYP2A6
p−Dichlorobenzene: CYP2D6
p−Dichlorobenzene: CYP2B6
p−Dichlorobenzene: CYP2E1
p−Dichlorobenzene: CYP2C8
p−Dichlorobenzene: CYP2J2
p−Dichlorobenzene: CYP2C9
p−Dichlorobenzene: CYP3A4
% Inhibition
0
30
60
90
Concentration (log−10 uM)
−3
−2
−1
0
1
2
CYP1A2
CYP2C19
CYP2A6
CYP2D6
CYP2B6
CYP2E1
CYP2C8
CYP2J2
CYP2C9
CYP3A4
Bgal
No_RNA
