# Supplementary material for: High-throughput AR dimerization assay identifies androgen disrupting chemicals and metabolites
Source: Front Toxicol. 2023 Apr 4;5:1134783. doi: 10.3389/ftox.2023.1134783 (PMC10112521; doi:10.3389/ftox.2023.1134783)

### 17−Methyltestosterone: CYP1A2

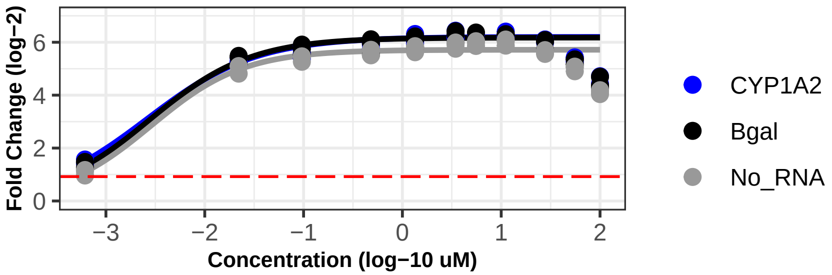


### 17−Methyltestosterone: CYP2C19

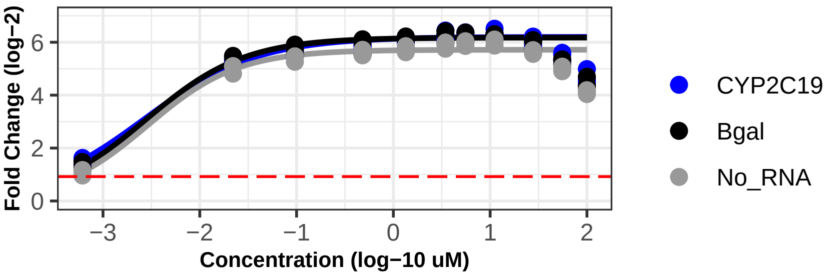


### 17−Methyltestosterone: CYP2A6

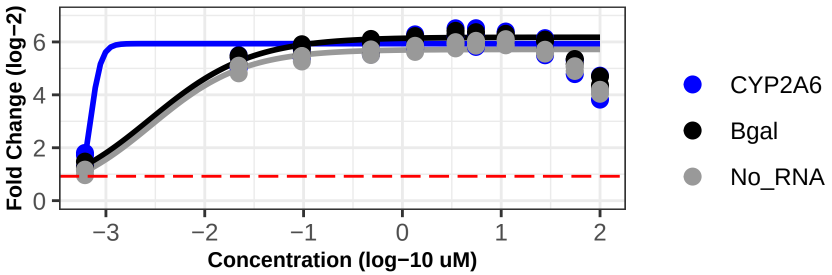


### 17−Methyltestosterone: CYP2D6

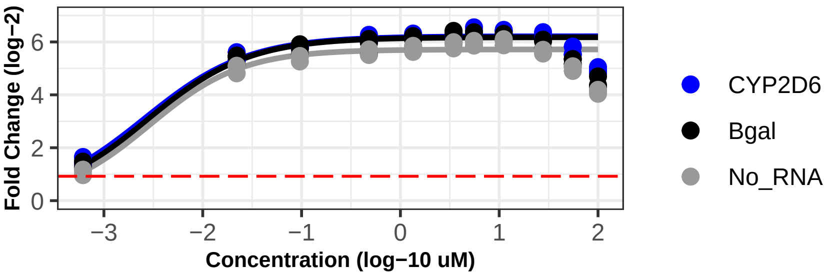


### 17−Methyltestosterone: CYP2B6

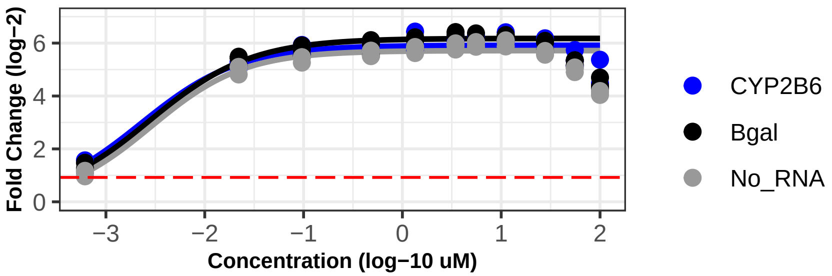


### 17−Methyltestosterone: CYP2E1

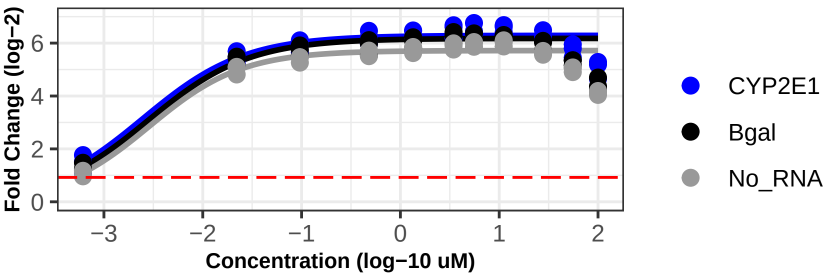


### 17−Methyltestosterone: CYP2C8

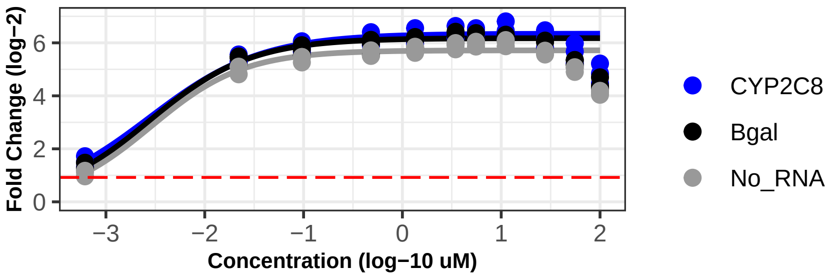


### 17−Methyltestosterone: CYP2J2

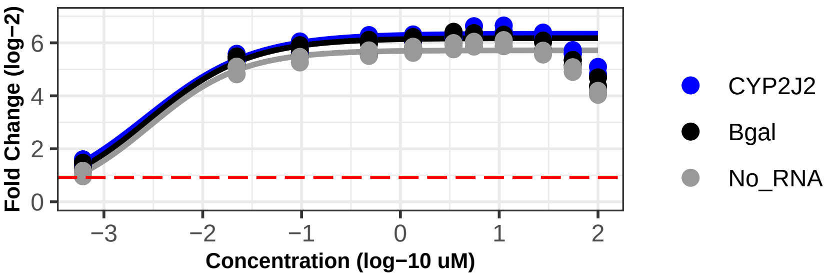


### 17−Methyltestosterone: CYP2C9

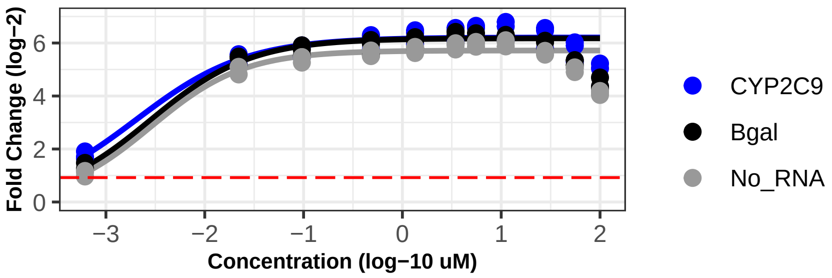


### 17−Methyltestosterone: CYP3A4

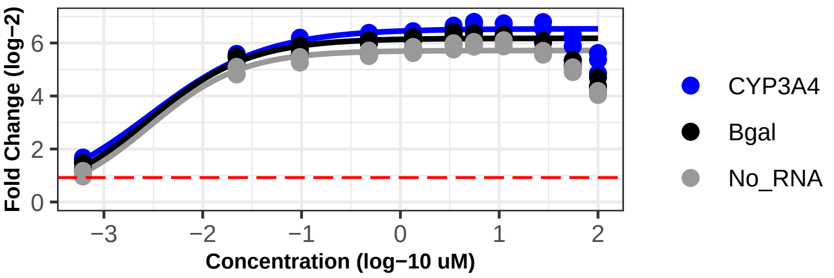

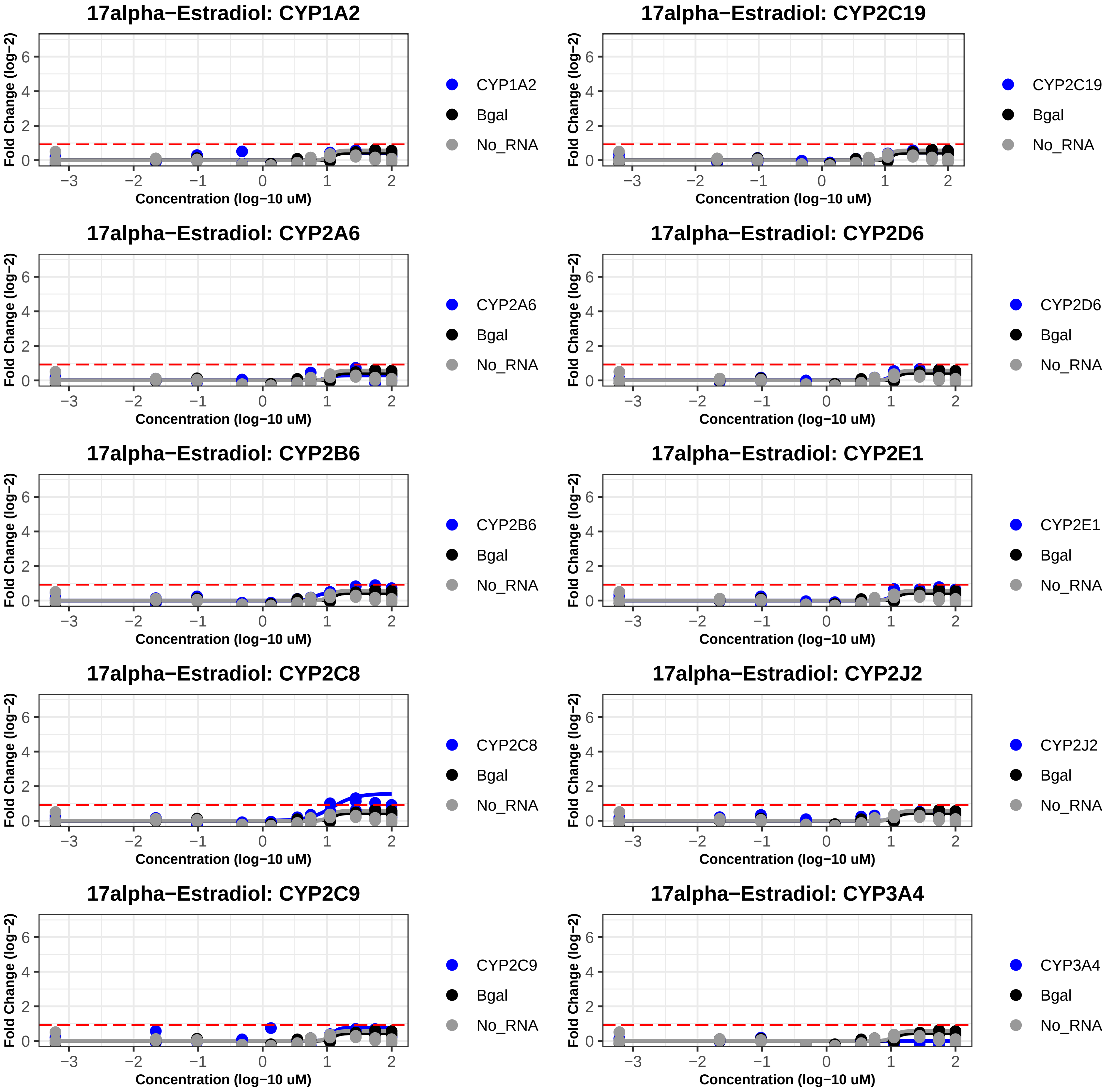
17alpha−Estradiol: CYP1A2
Fold Change (log−2)
Concentration (log−10 uM)
CYP1A2
Bgal
No_RNA
17alpha−Estradiol: CYP2C19
CYP2C19
17alpha−Estradiol: CYP2A6
CYP2A6
17alpha−Estradiol: CYP2D6
CYP2D6
17alpha−Estradiol: CYP2B6
CYP2B6
17alpha−Estradiol: CYP2E1
CYP2E1
17alpha−Estradiol: CYP2C8
CYP2C8
17alpha−Estradiol: CYP2J2
CYP2J2
17alpha−Estradiol: CYP2C9
CYP2C9
17alpha−Estradiol: CYP3A4
CYP3A4

17alpha−Ethinylestradiol: CYP1A2

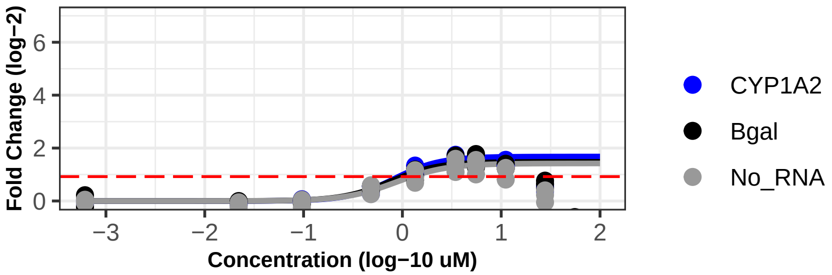


17alpha−Ethinylestradiol: CYP2C19

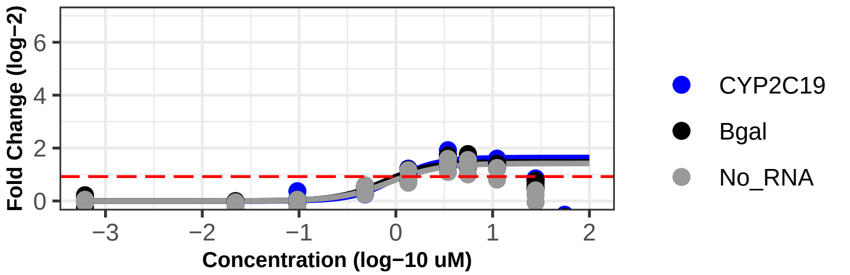


17alpha−Ethinylestradiol: CYP2A6

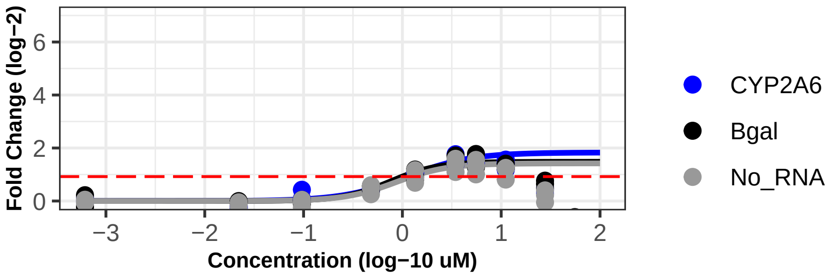


17alpha−Ethinylestradiol: CYP2D6

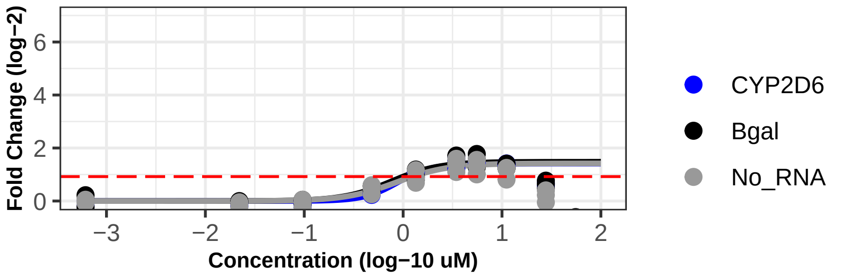


17alpha−Ethinylestradiol: CYP2B6

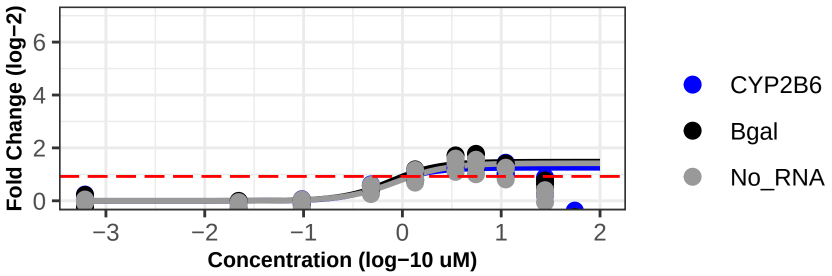


17alpha−Ethinylestradiol: CYP2E1

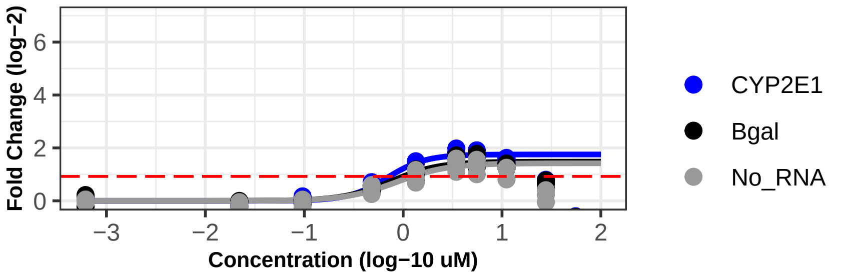


17alpha−Ethinylestradiol: CYP2C8

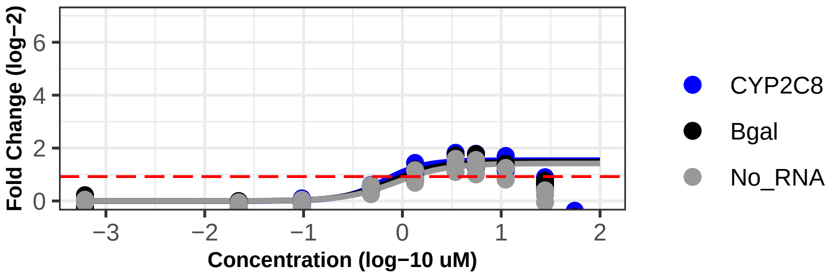


17alpha−Ethinylestradiol: CYP2J2

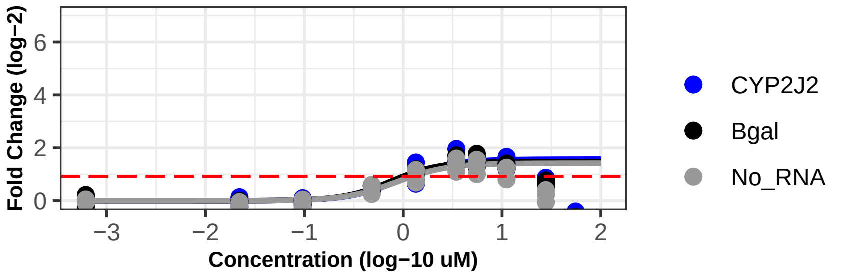


17alpha−Ethinylestradiol: CYP2C9

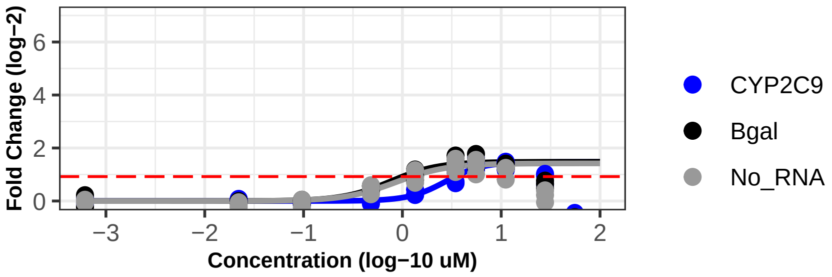


17alpha−Ethinylestradiol: CYP3A4

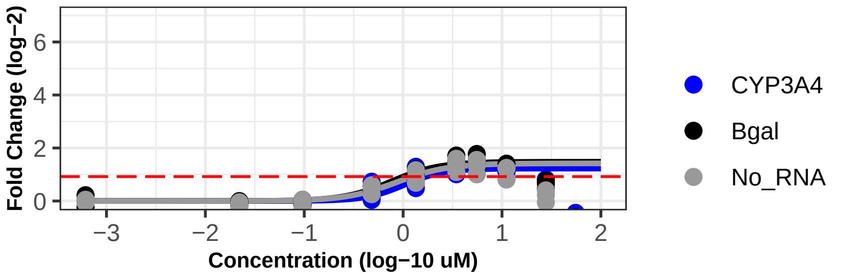

### 17beta−Estradiol: CYP1A2

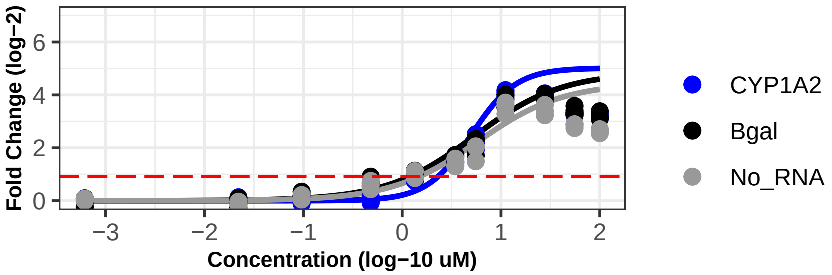


### 17beta−Estradiol: CYP2C19

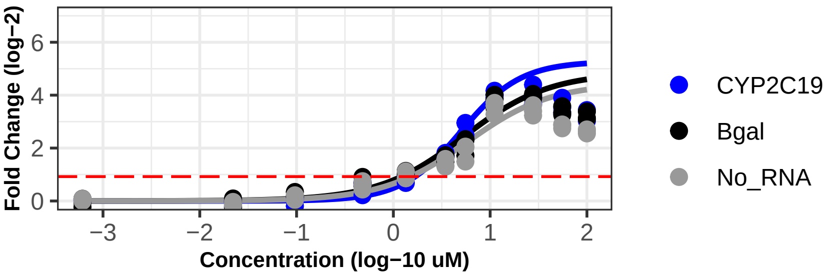


### 17beta−Estradiol: CYP2A6

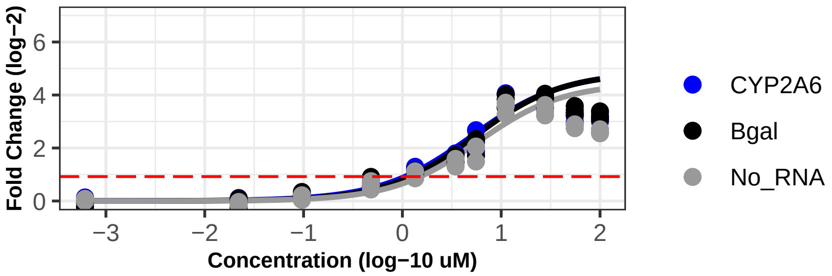


### 17beta−Estradiol: CYP2D6

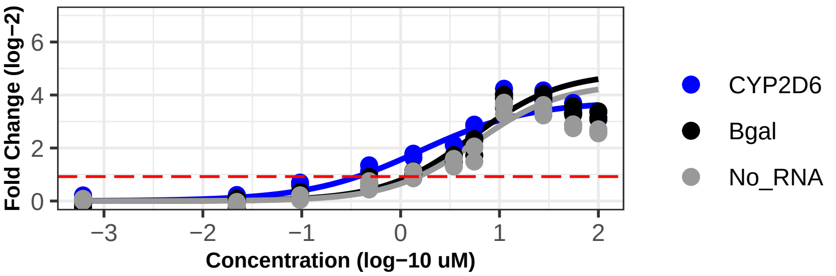


### 17beta−Estradiol: CYP2B6

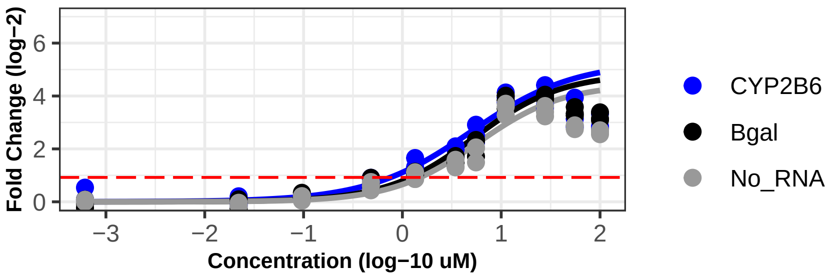


### 17beta−Estradiol: CYP2E1

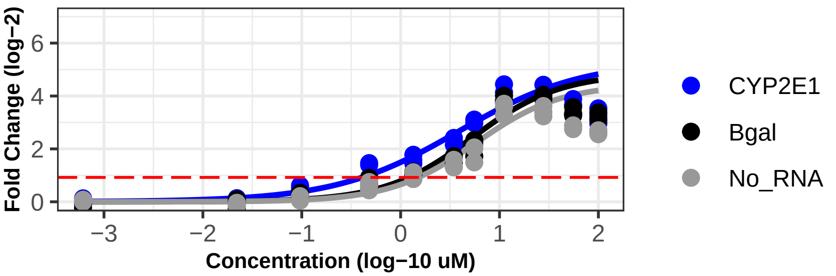


### 17beta−Estradiol: CYP2C8

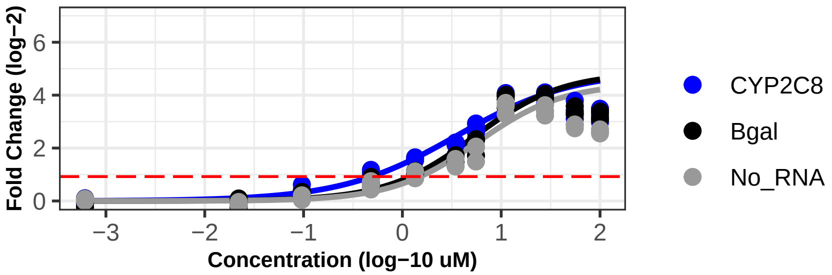


### 17beta−Estradiol: CYP2J2

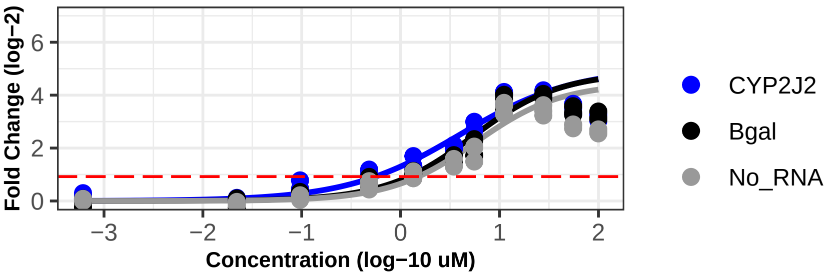


### 17beta−Estradiol: CYP2C9

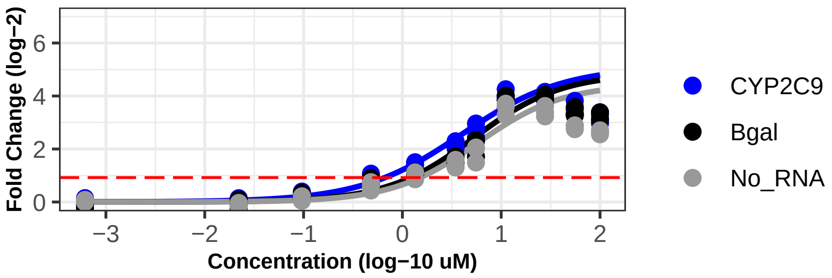


### 17beta−Estradiol: CYP3A4

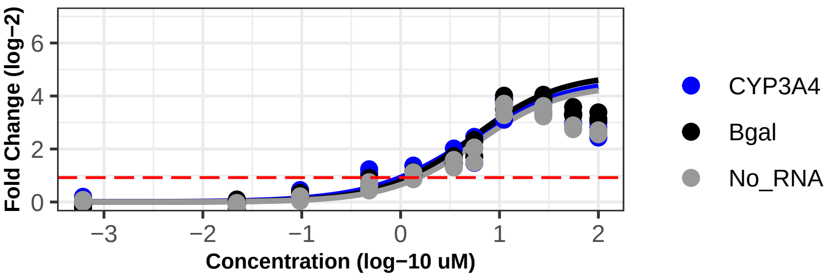

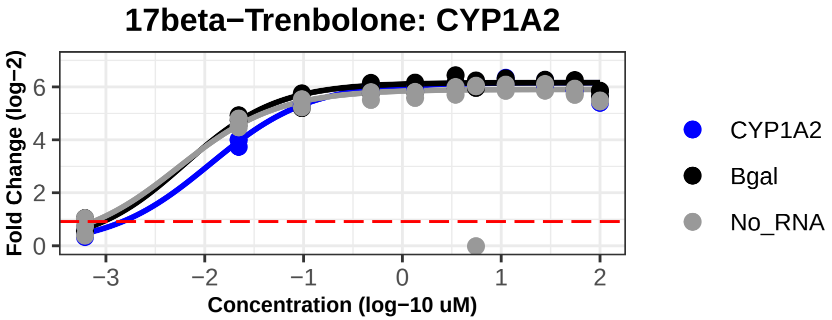
17beta−Trenbolone: CYP1A2
Fold Change (log−2)
Concentration (log−10 uM)
CYP1A2
Bgal
No_RNA

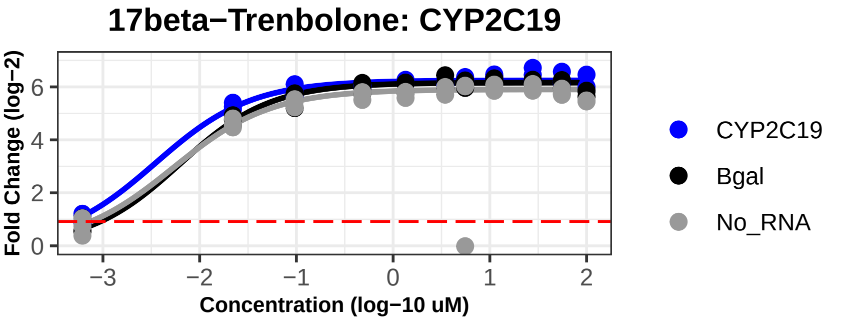
17beta−Trenbolone: CYP2C19
Fold Change (log−2)
Concentration (log−10 uM)
CYP2C19
Bgal
No_RNA

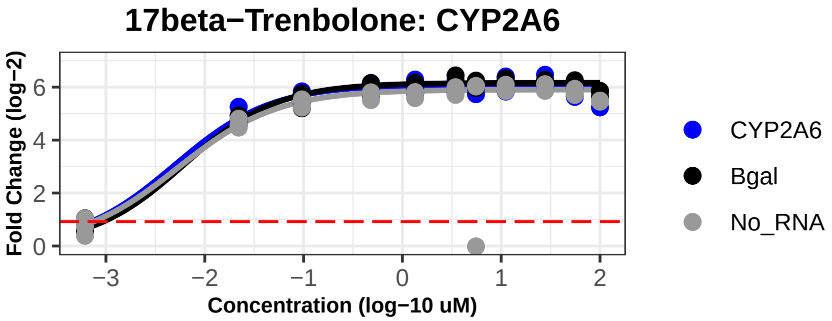
17beta−Trenbolone: CYP2A6
Fold Change (log−2)
Concentration (log−10 uM)
CYP2A6
Bgal
No_RNA

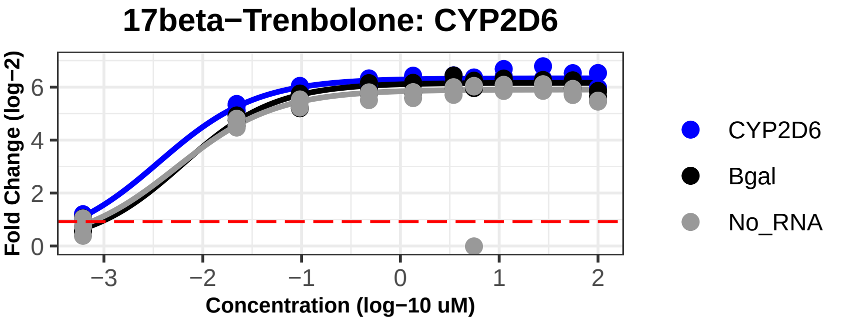
17beta−Trenbolone: CYP2D6
Fold Change (log−2)
Concentration (log−10 uM)
CYP2D6
Bgal
No_RNA

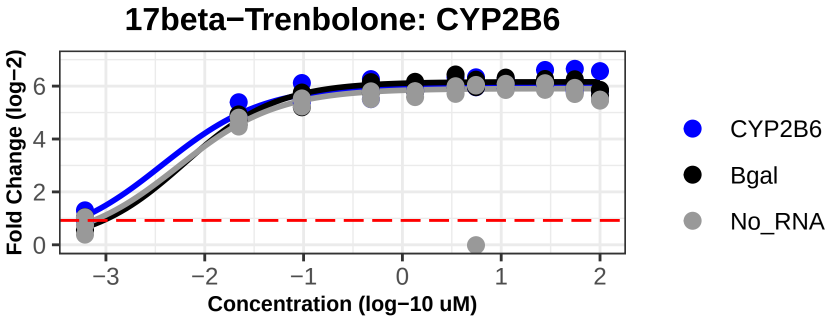
17beta−Trenbolone: CYP2B6
Fold Change (log−2)
Concentration (log−10 uM)
CYP2B6
Bgal
No_RNA

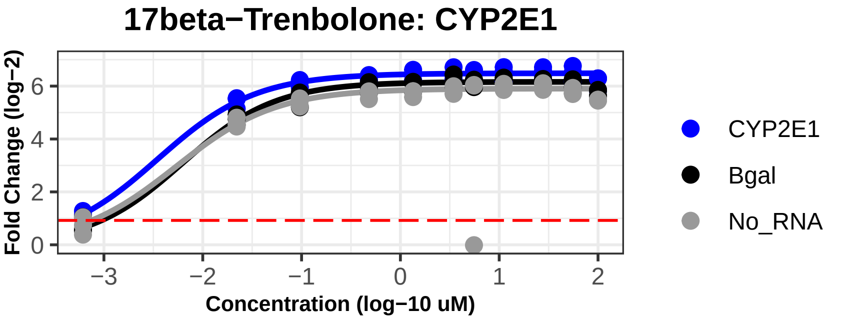
17beta−Trenbolone: CYP2E1
Fold Change (log−2)
Concentration (log−10 uM)
CYP2E1
Bgal
No_RNA

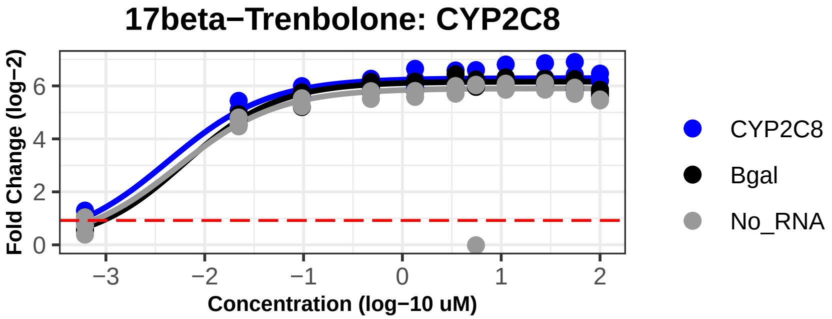
17beta−Trenbolone: CYP2C8
Fold Change (log−2)
Concentration (log−10 uM)
CYP2C8
Bgal
No_RNA

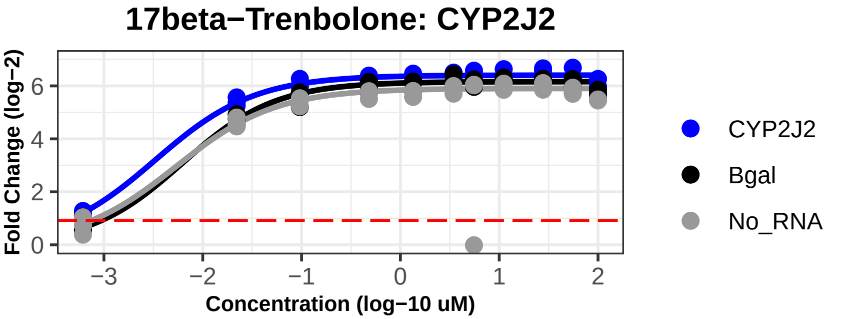
17beta−Trenbolone: CYP2J2
Fold Change (log−2)
Concentration (log−10 uM)
CYP2J2
Bgal
No_RNA

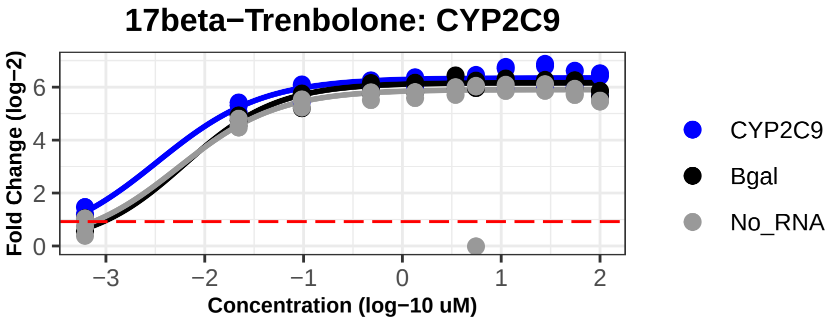
17beta−Trenbolone: CYP2C9
Fold Change (log−2)
Concentration (log−10 uM)
CYP2C9
Bgal
No_RNA

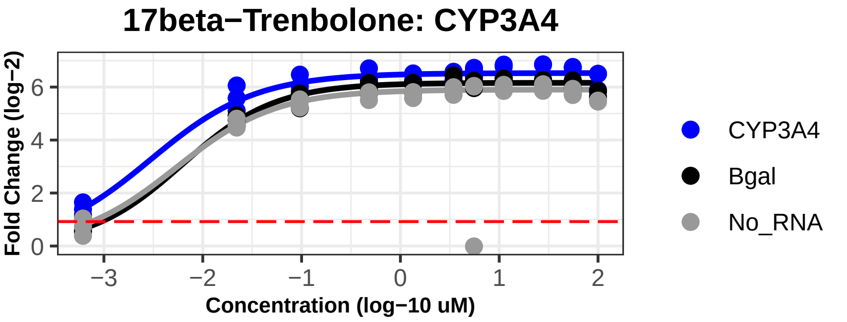
17beta−Trenbolone: CYP3A4
Fold Change (log−2)
Concentration (log−10 uM)
CYP3A4
Bgal
No_RNA

## 2,2',4,4'−Tetrahydroxybenzophenone: CYP1A2

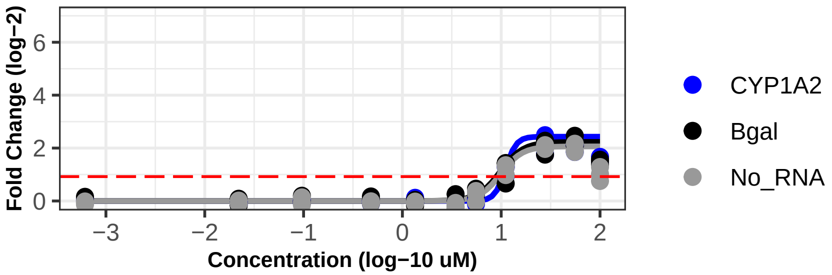


## 2,2',4,4'−Tetrahydroxybenzophenone: CYP2C19

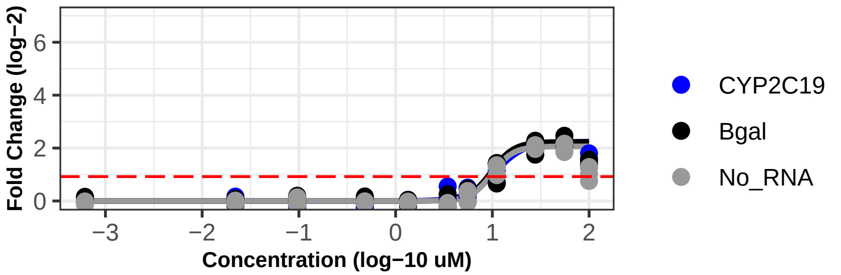


## 2,2',4,4'−Tetrahydroxybenzophenone: CYP2A6

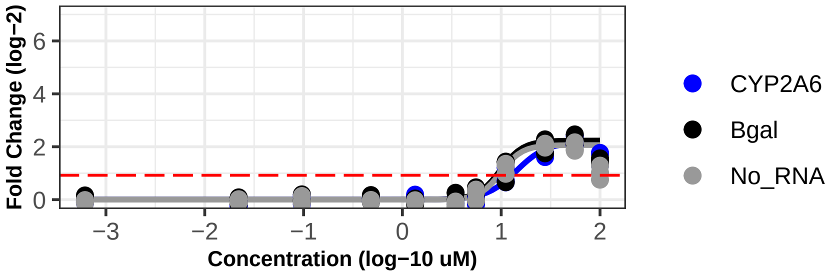


## 2,2',4,4'−Tetrahydroxybenzophenone: CYP2D6

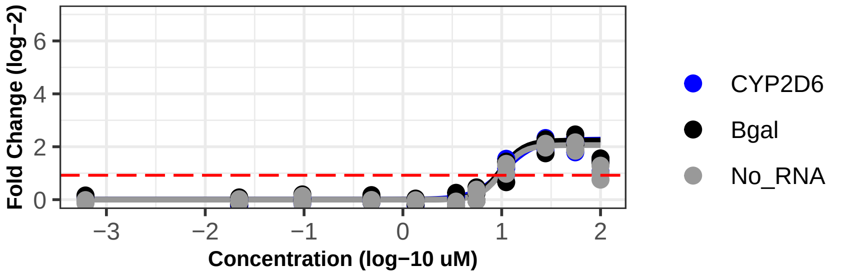


## 2,2',4,4'−Tetrahydroxybenzophenone: CYP2B6

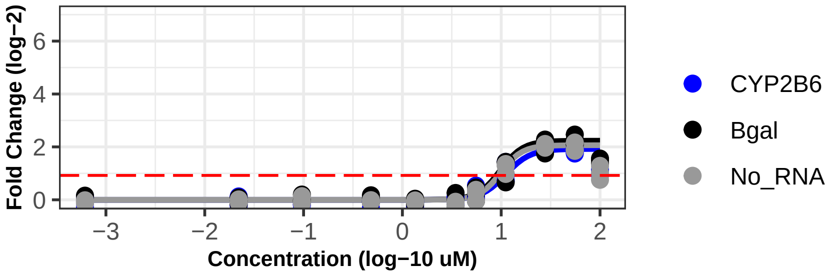


## 2,2',4,4'−Tetrahydroxybenzophenone: CYP2E1

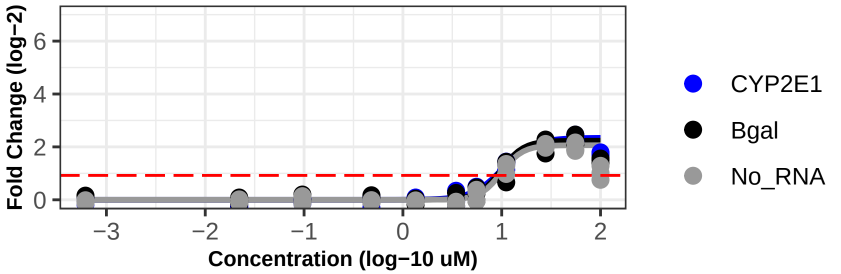


## 2,2',4,4'−Tetrahydroxybenzophenone: CYP2C8

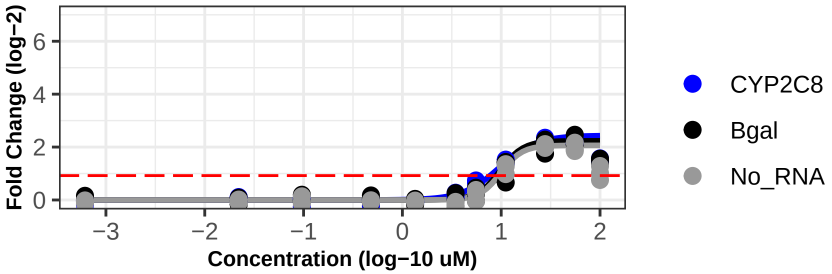


## 2,2',4,4'−Tetrahydroxybenzophenone: CYP2J2

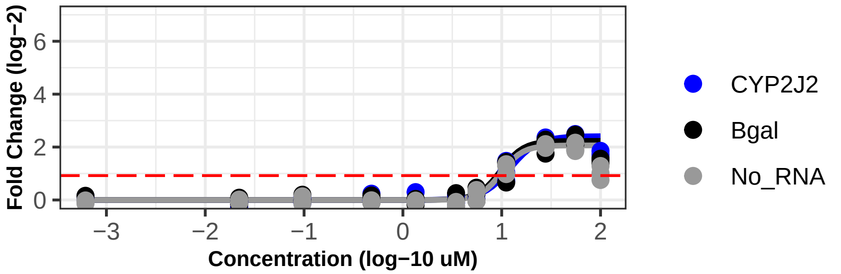


## 2,2',4,4'−Tetrahydroxybenzophenone: CYP2C9

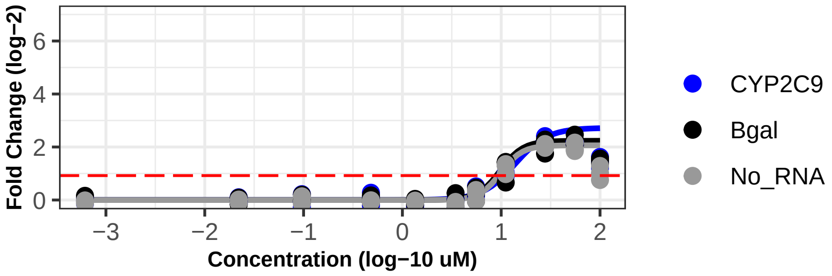


## 2,2',4,4'−Tetrahydroxybenzophenone: CYP3A4

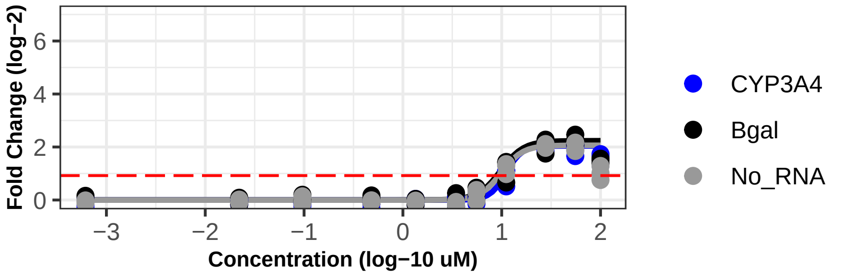

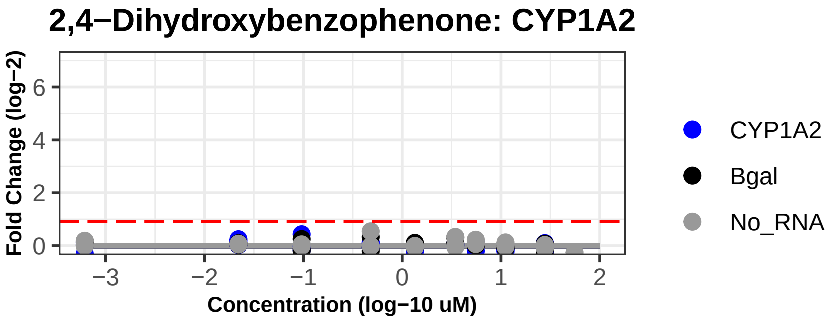
2,4−Dihydroxybenzophenone: CYP1A2
Fold Change (log−2)
6
4
2
0
−3
−2
−1
0
1
2
Concentration (log−10 uM)
CYP1A2
Bgal
No_RNA

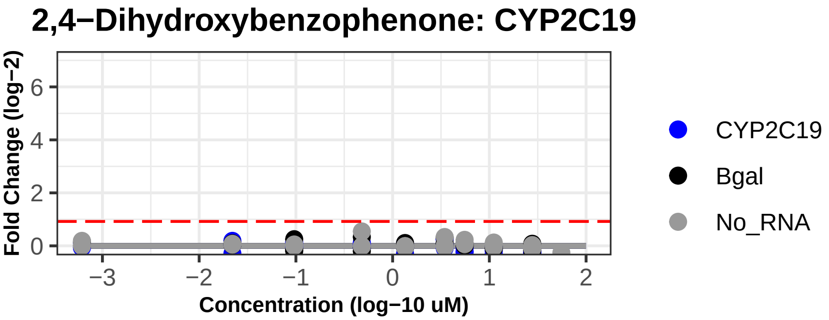
2,4−Dihydroxybenzophenone: CYP2C19
Fold Change (log−2)
6
4
2
0
−3
−2
−1
0
1
2
Concentration (log−10 uM)
CYP2C19
Bgal
No_RNA

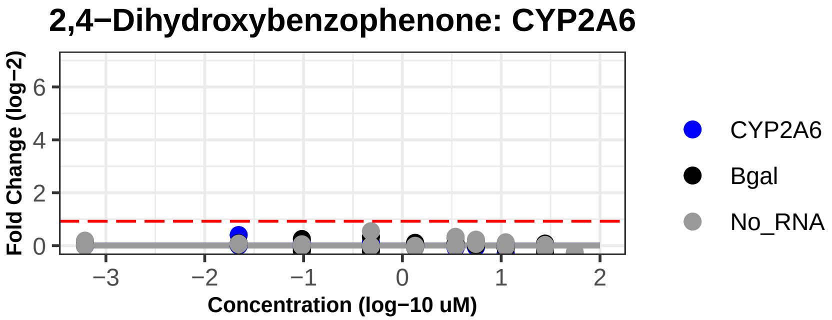
2,4−Dihydroxybenzophenone: CYP2A6
Fold Change (log−2)
6
4
2
0
−3
−2
−1
0
1
2
Concentration (log−10 uM)
CYP2A6
Bgal
No_RNA

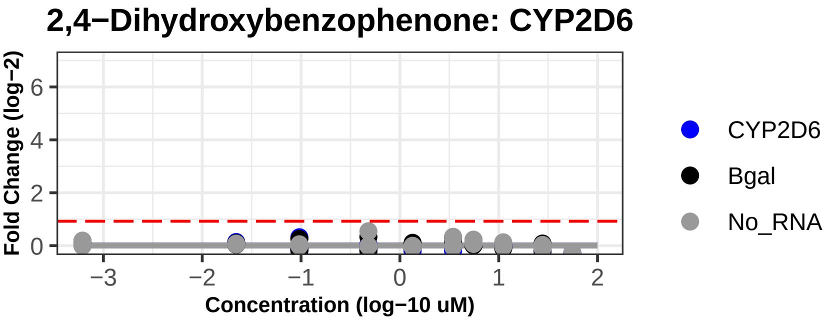
2,4−Dihydroxybenzophenone: CYP2D6
Fold Change (log−2)
6
4
2
0
−3
−2
−1
0
1
2
Concentration (log−10 uM)
CYP2D6
Bgal
No_RNA

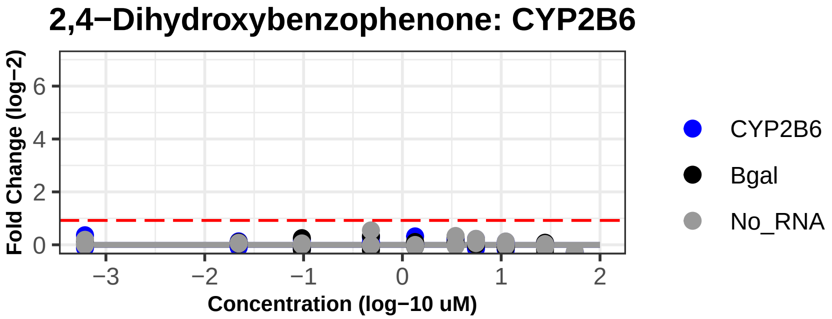
2,4−Dihydroxybenzophenone: CYP2B6
Fold Change (log−2)
6
4
2
0
−3
−2
−1
0
1
2
Concentration (log−10 uM)
CYP2B6
Bgal
No_RNA

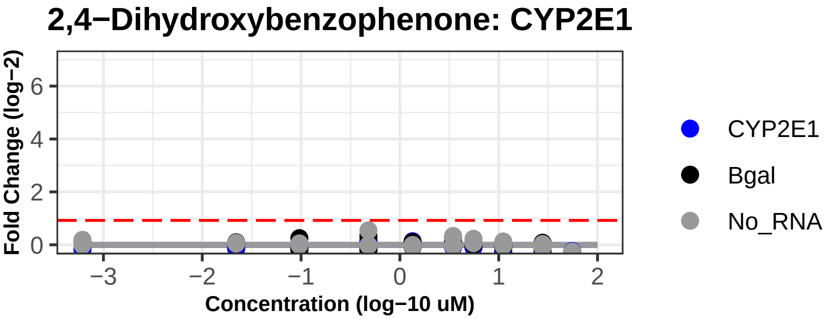
2,4−Dihydroxybenzophenone: CYP2E1
Fold Change (log−2)
6
4
2
0
−3
−2
−1
0
1
2
Concentration (log−10 uM)
CYP2E1
Bgal
No_RNA

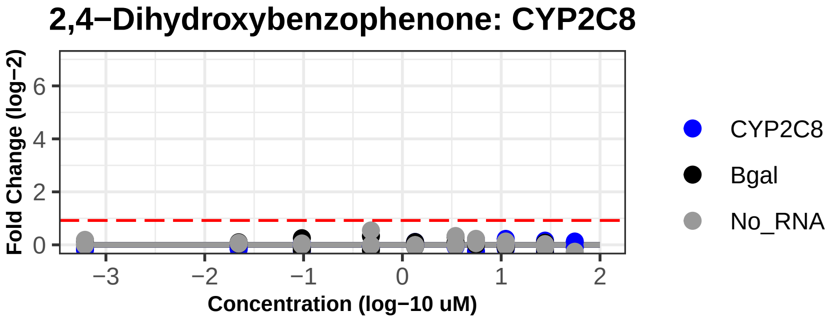
2,4−Dihydroxybenzophenone: CYP2C8
Fold Change (log−2)
6
4
2
0
−3
−2
−1
0
1
2
Concentration (log−10 uM)
CYP2C8
Bgal
No_RNA

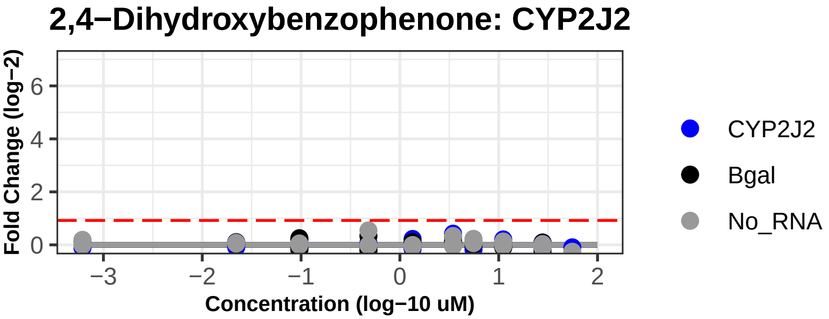
2,4−Dihydroxybenzophenone: CYP2J2
Fold Change (log−2)
6
4
2
0
−3
−2
−1
0
1
2
Concentration (log−10 uM)
CYP2J2
Bgal
No_RNA

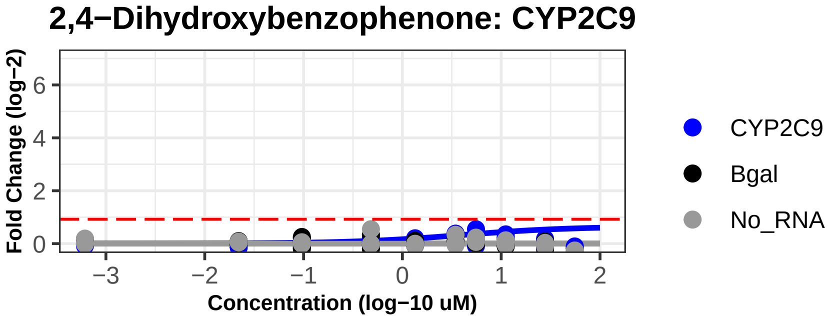
2,4−Dihydroxybenzophenone: CYP2C9
Fold Change (log−2)
6
4
2
0
−3
−2
−1
0
1
2
Concentration (log−10 uM)
CYP2C9
Bgal
No_RNA

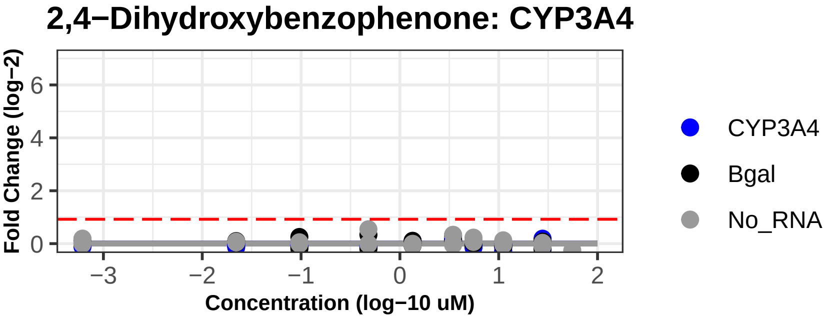
2,4−Dihydroxybenzophenone: CYP3A4
Fold Change (log−2)
6
4
2
0
−3
−2
−1
0
1
2
Concentration (log−10 uM)
CYP3A4
Bgal
No_RNA

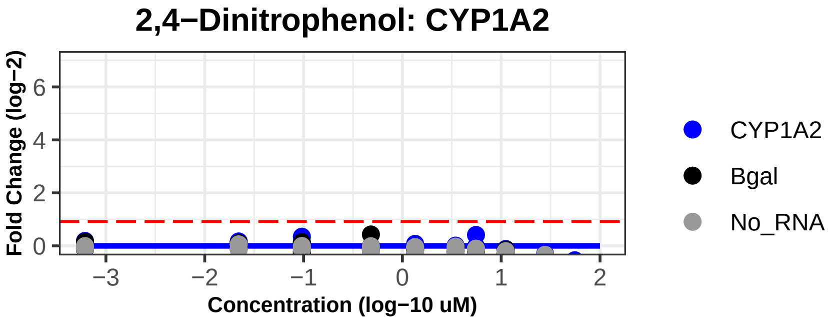
2,4−Dinitrophenol: CYP1A2
Fold Change (log−2)
Concentration (log−10 uM)
CYP1A2
Bgal
No_RNA

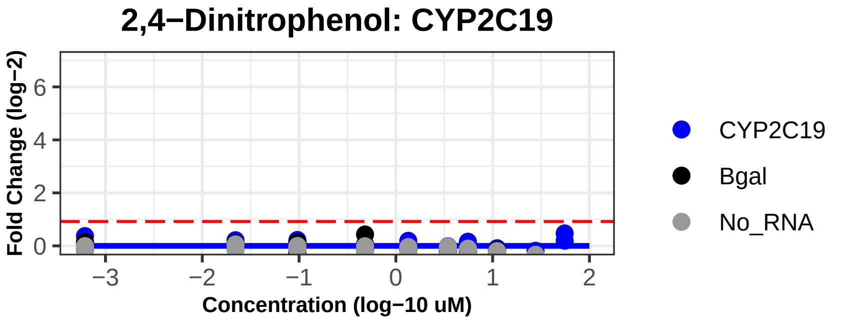
2,4−Dinitrophenol: CYP2C19
Fold Change (log−2)
Concentration (log−10 uM)
CYP2C19
Bgal
No_RNA

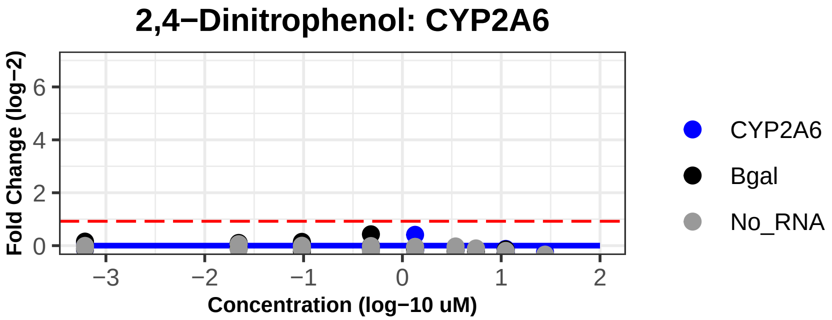
2,4−Dinitrophenol: CYP2A6
Fold Change (log−2)
Concentration (log−10 uM)
CYP2A6
Bgal
No_RNA

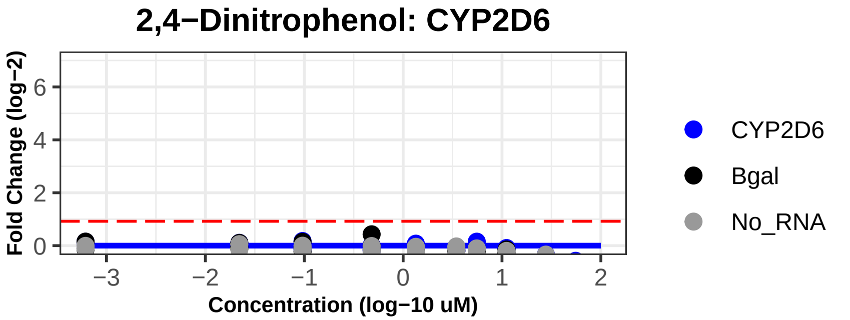
2,4−Dinitrophenol: CYP2D6
Fold Change (log−2)
Concentration (log−10 uM)
CYP2D6
Bgal
No_RNA

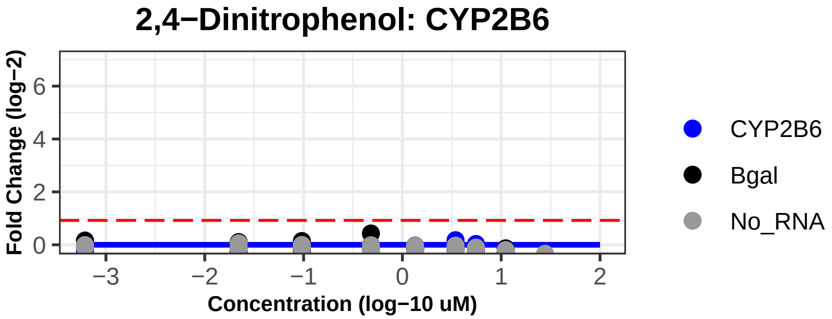
2,4−Dinitrophenol: CYP2B6
Fold Change (log−2)
Concentration (log−10 uM)
CYP2B6
Bgal
No_RNA

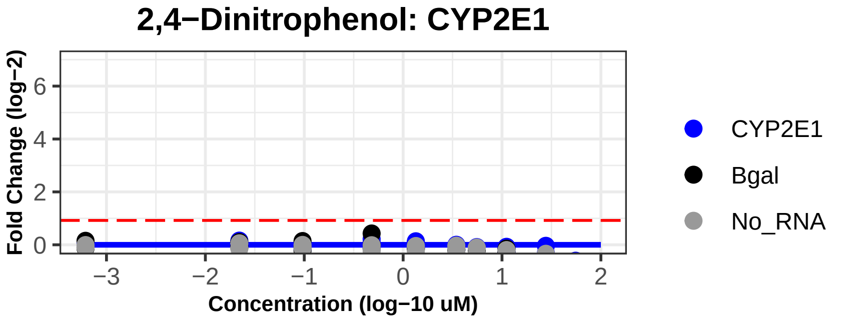
2,4−Dinitrophenol: CYP2E1
Fold Change (log−2)
Concentration (log−10 uM)
CYP2E1
Bgal
No_RNA

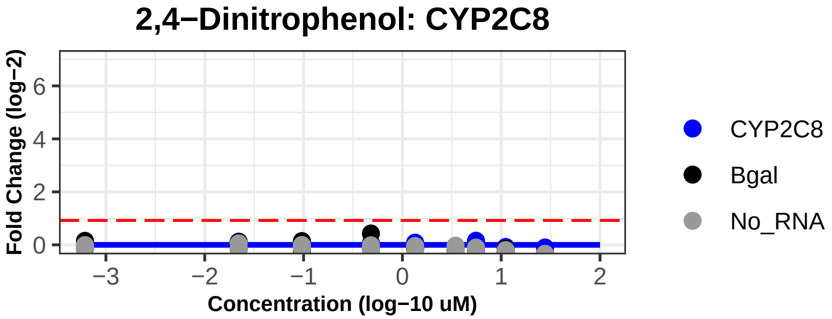
2,4−Dinitrophenol: CYP2C8
Fold Change (log−2)
Concentration (log−10 uM)
CYP2C8
Bgal
No_RNA

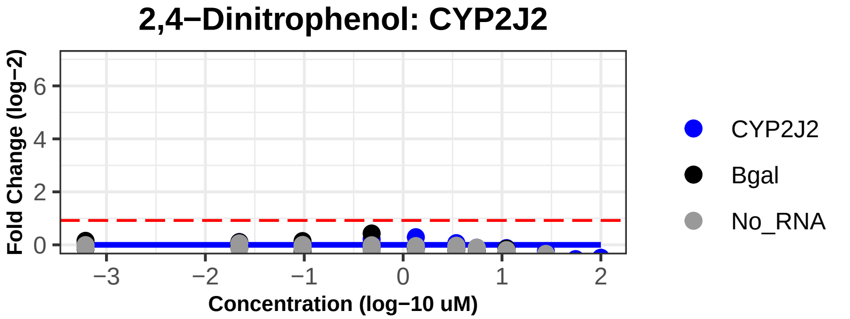
2,4−Dinitrophenol: CYP2J2
Fold Change (log−2)
Concentration (log−10 uM)
CYP2J2
Bgal
No_RNA

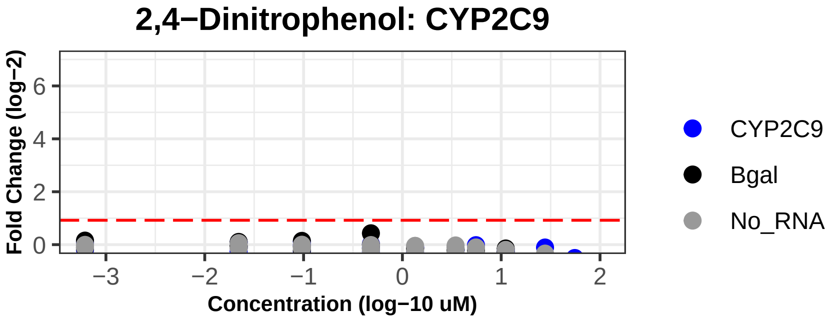
2,4−Dinitrophenol: CYP2C9
Fold Change (log−2)
Concentration (log−10 uM)
CYP2C9
Bgal
No_RNA

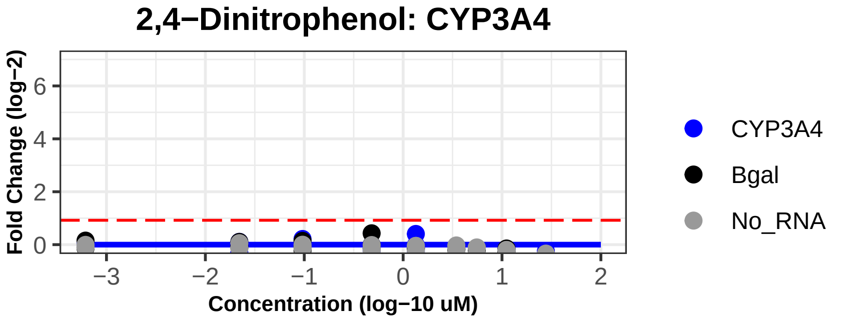
2,4−Dinitrophenol: CYP3A4
Fold Change (log−2)
Concentration (log−10 uM)
CYP3A4
Bgal
No_RNA

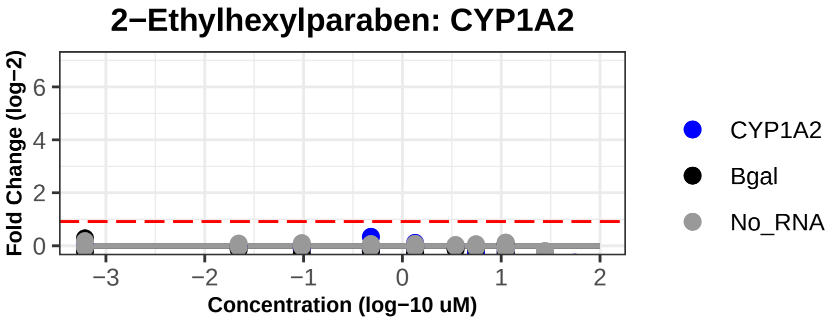
2−Ethylhexylparaben: CYP1A2
Fold Change (log−2)
Concentration (log−10 uM)
CYP1A2
Bgal
No_RNA

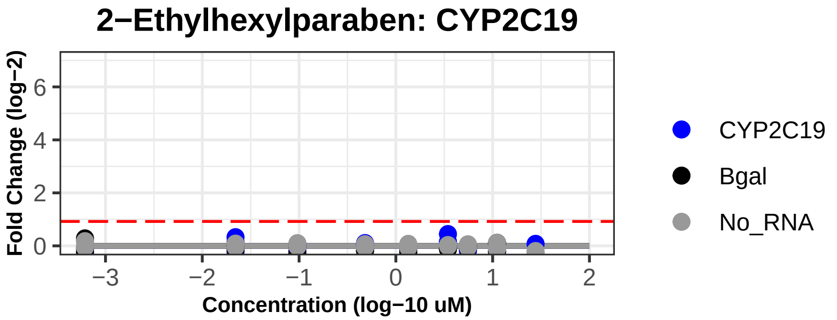
2−Ethylhexylparaben: CYP2C19
Fold Change (log−2)
Concentration (log−10 uM)
CYP2C19
Bgal
No_RNA

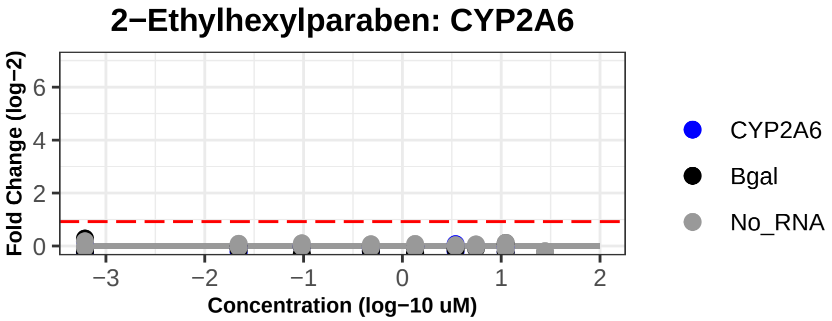
2−Ethylhexylparaben: CYP2A6
Fold Change (log−2)
Concentration (log−10 uM)
CYP2A6
Bgal
No_RNA

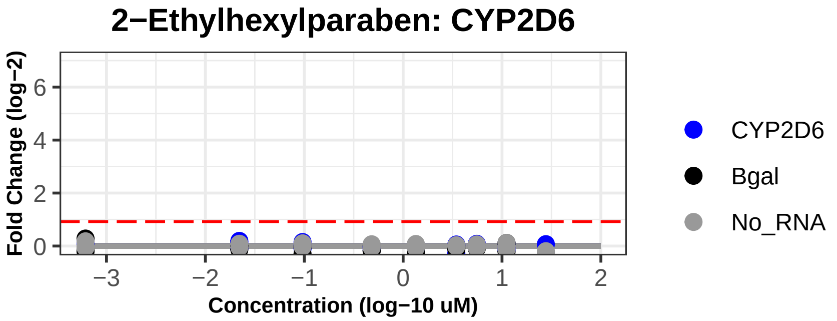
2−Ethylhexylparaben: CYP2D6
Fold Change (log−2)
Concentration (log−10 uM)
CYP2D6
Bgal
No_RNA

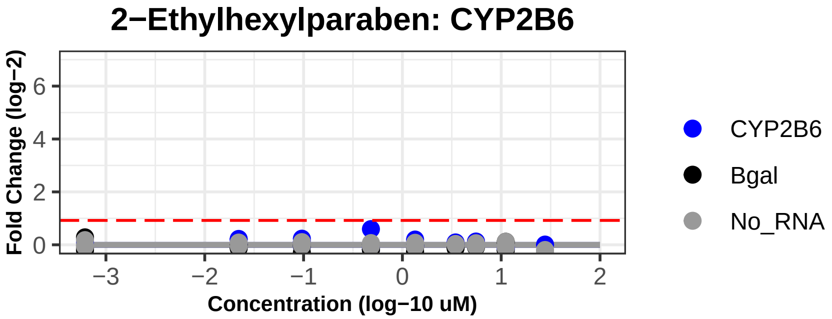
2−Ethylhexylparaben: CYP2B6
Fold Change (log−2)
Concentration (log−10 uM)
CYP2B6
Bgal
No_RNA

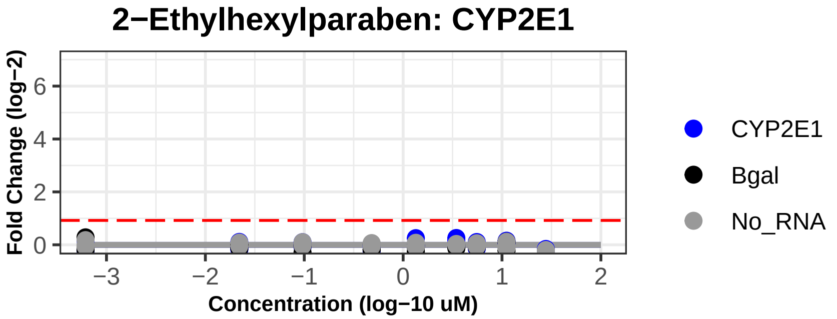
2−Ethylhexylparaben: CYP2E1
Fold Change (log−2)
Concentration (log−10 uM)
CYP2E1
Bgal
No_RNA

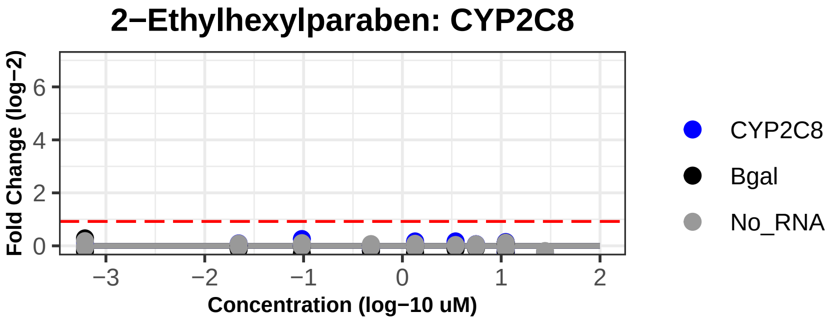
2−Ethylhexylparaben: CYP2C8
Fold Change (log−2)
Concentration (log−10 uM)
CYP2C8
Bgal
No_RNA

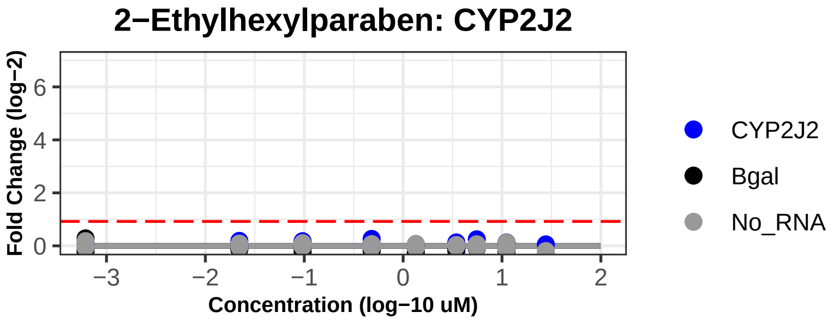
2−Ethylhexylparaben: CYP2J2
Fold Change (log−2)
Concentration (log−10 uM)
CYP2J2
Bgal
No_RNA

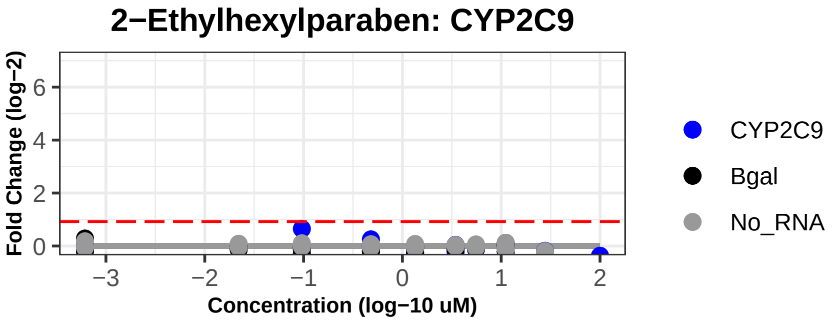
2−Ethylhexylparaben: CYP2C9
Fold Change (log−2)
Concentration (log−10 uM)
CYP2C9
Bgal
No_RNA

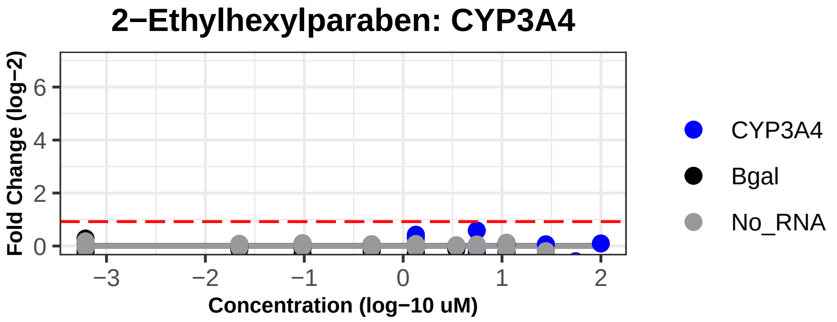
2−Ethylhexylparaben: CYP3A4
Fold Change (log−2)
Concentration (log−10 uM)
CYP3A4
Bgal
No_RNA

4,4'−Sulfonyldiphenol: CYP1A2

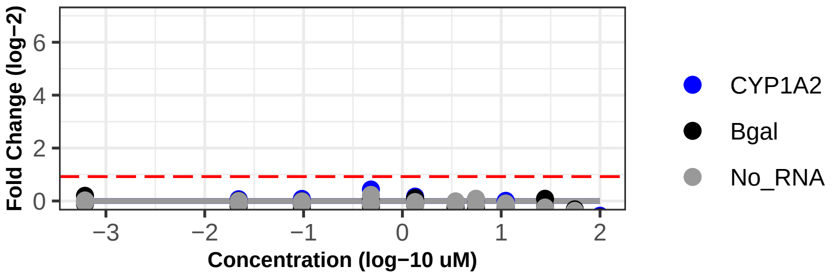


4,4'−Sulfonyldiphenol: CYP2C19

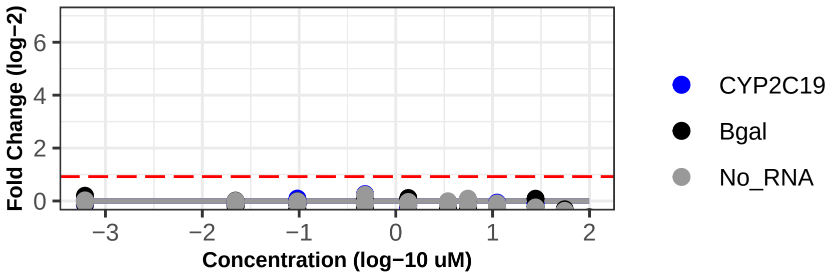


4,4'−Sulfonyldiphenol: CYP2A6

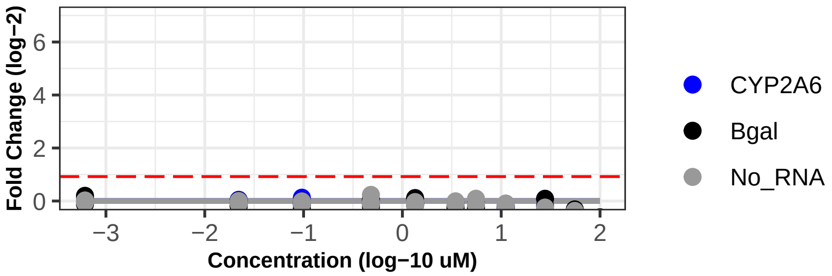


4,4'−Sulfonyldiphenol: CYP2D6

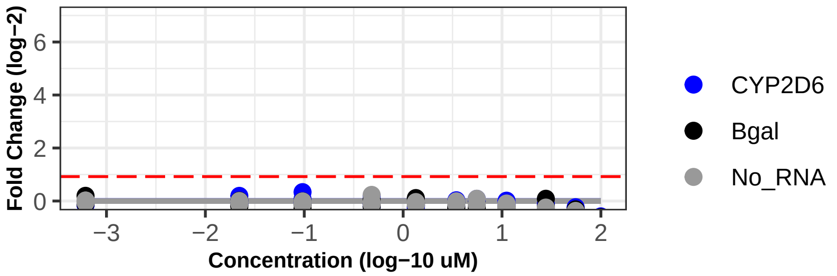


4,4'−Sulfonyldiphenol: CYP2B6

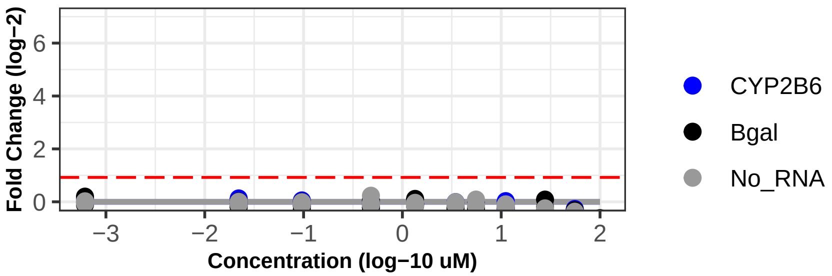


4,4'−Sulfonyldiphenol: CYP2E1

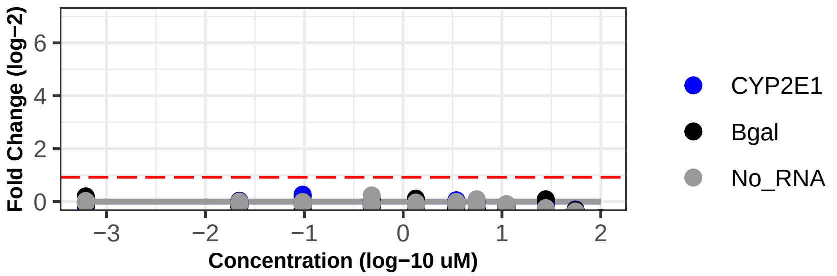


4,4'−Sulfonyldiphenol: CYP2C8

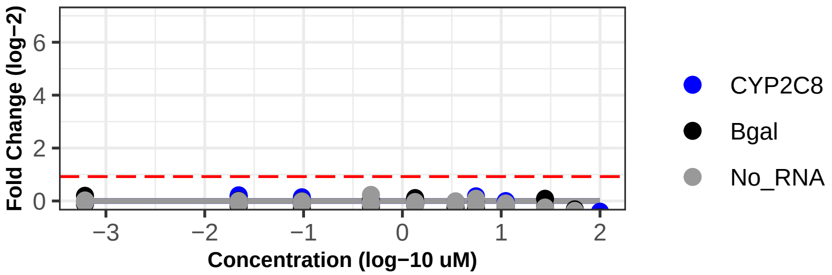


4,4'−Sulfonyldiphenol: CYP2J2

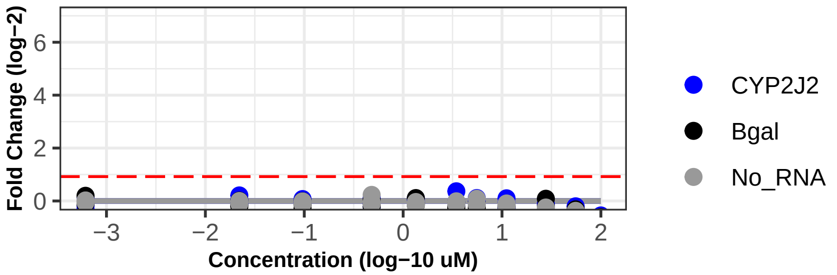


4,4'−Sulfonyldiphenol: CYP2C9

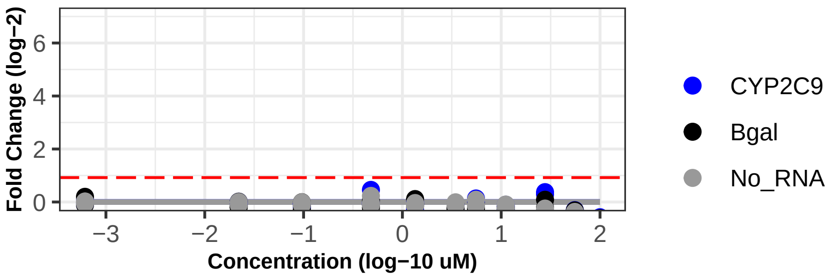


4,4'−Sulfonyldiphenol: CYP3A4

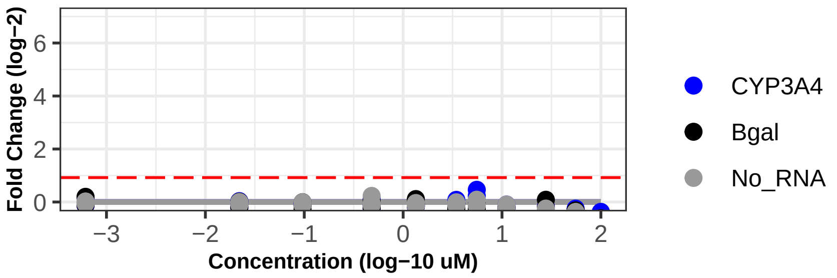

## 4−(1,1,3,3−Tetramethylbutyl)phenol: CYP1A2

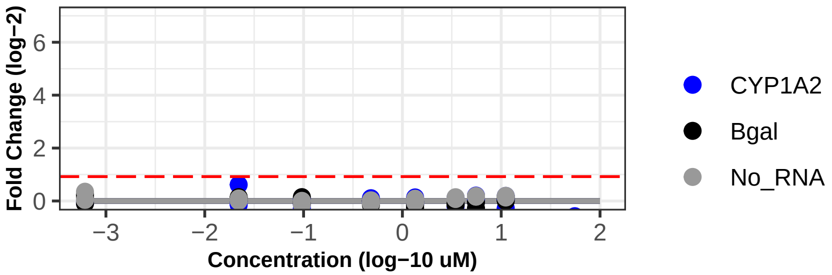


## 4−(1,1,3,3−Tetramethylbutyl)phenol: CYP2A6

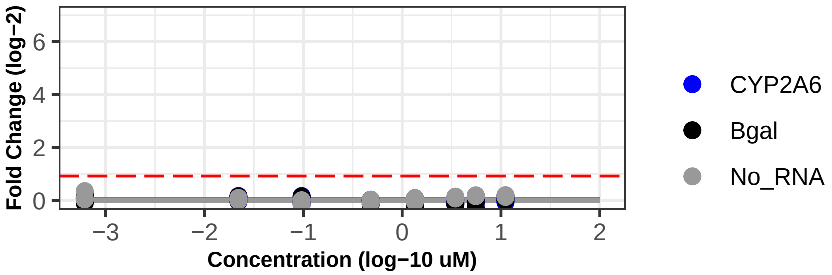


## 4−(1,1,3,3−Tetramethylbutyl)phenol: CYP2B6

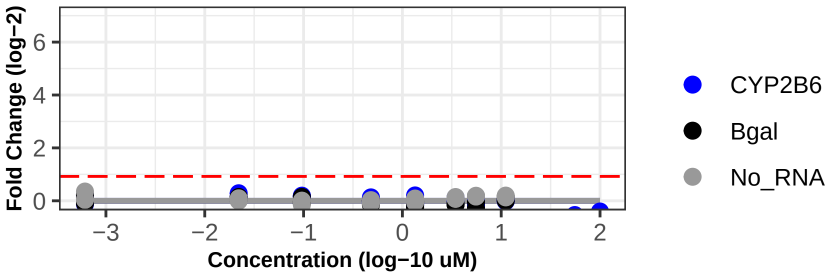


## 4−(1,1,3,3−Tetramethylbutyl)phenol: CYP2C8

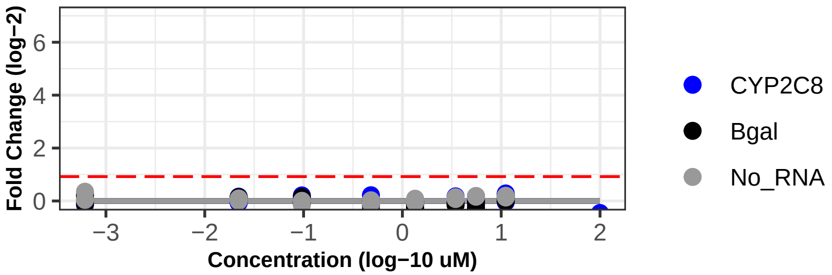


## 4−(1,1,3,3−Tetramethylbutyl)phenol: CYP2C9

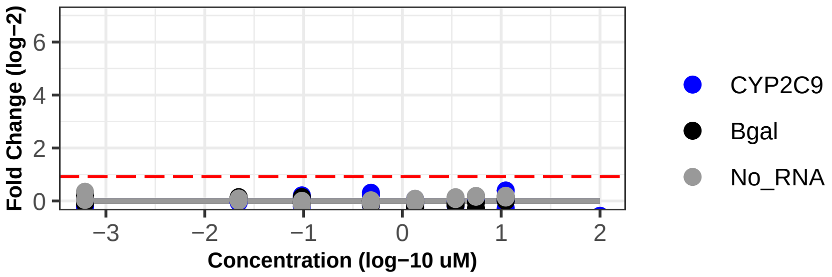


## 4−(1,1,3,3−Tetramethylbutyl)phenol: CYP2C19

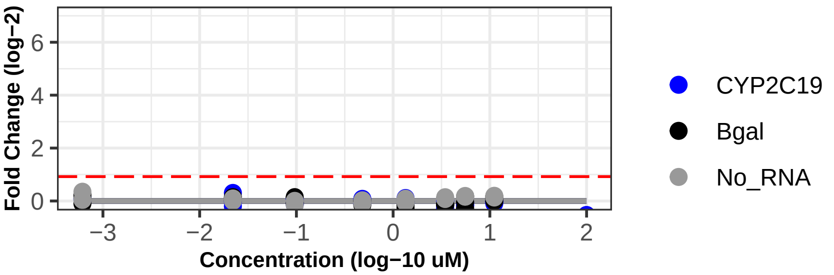


## 4−(1,1,3,3−Tetramethylbutyl)phenol: CYP2D6

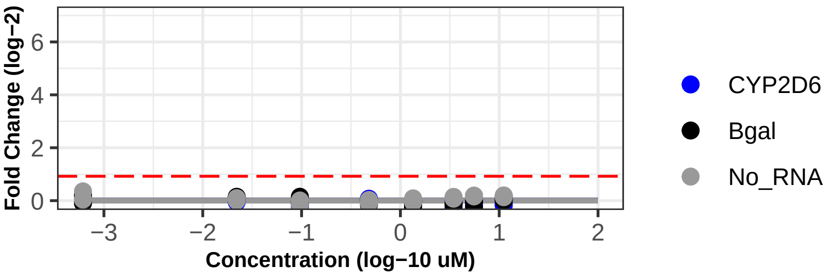


## 4−(1,1,3,3−Tetramethylbutyl)phenol: CYP2E1

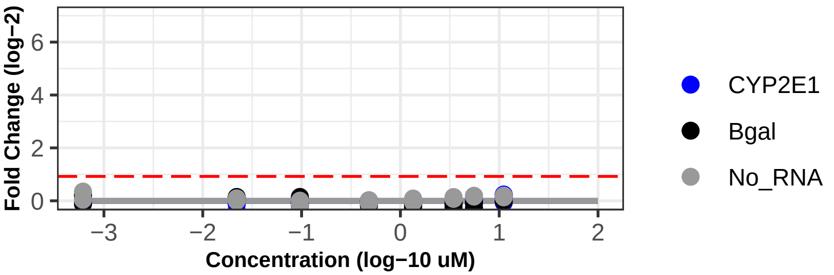


## 4−(1,1,3,3−Tetramethylbutyl)phenol: CYP2J2

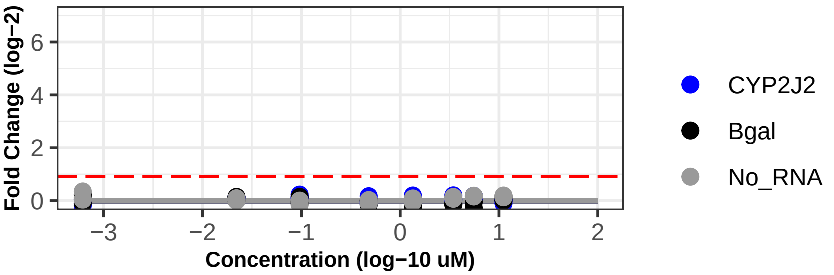


## 4−(1,1,3,3−Tetramethylbutyl)phenol: CYP3A4

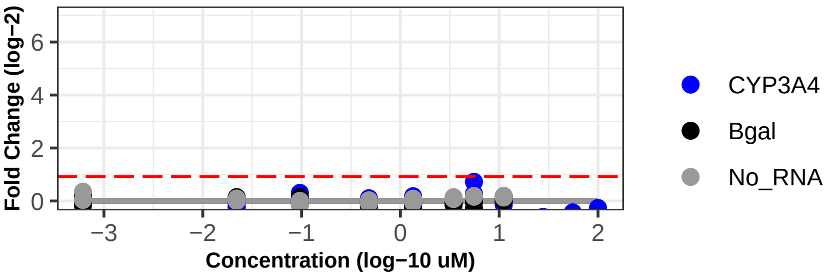

4−(2−Methylbutan−2−yl)phenol: CYP1A2

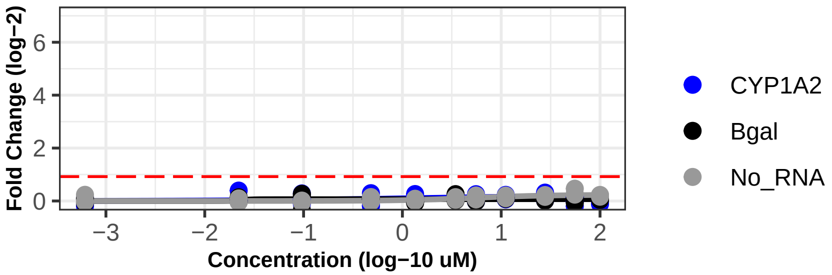


4−(2−Methylbutan−2−yl)phenol: CYP2C19

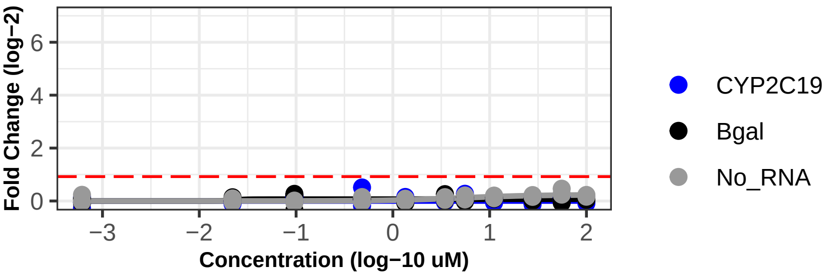


4−(2−Methylbutan−2−yl)phenol: CYP2A6

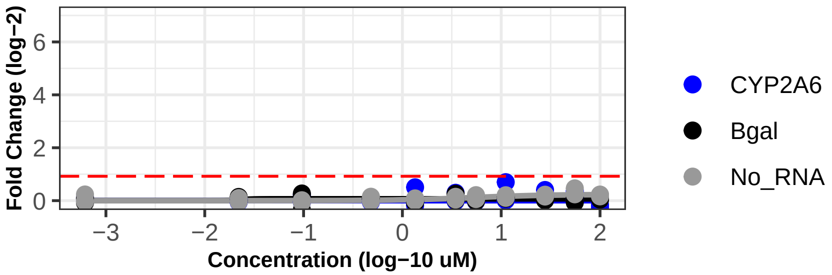


4−(2−Methylbutan−2−yl)phenol: CYP2D6

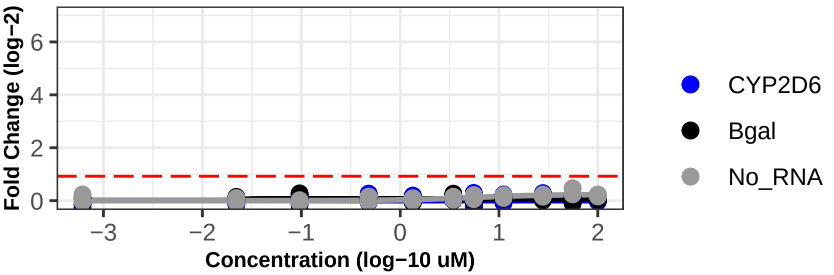


4−(2−Methylbutan−2−yl)phenol: CYP2B6

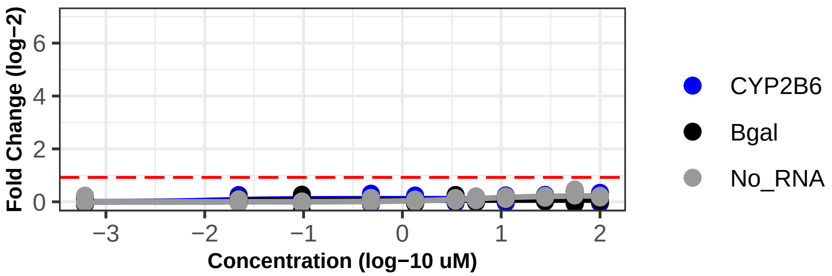


4−(2−Methylbutan−2−yl)phenol: CYP2E1

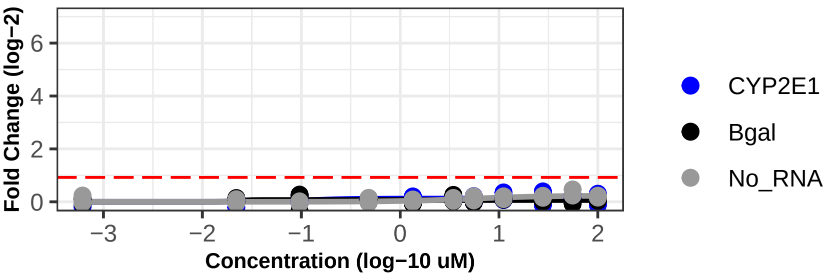


4−(2−Methylbutan−2−yl)phenol: CYP2C8

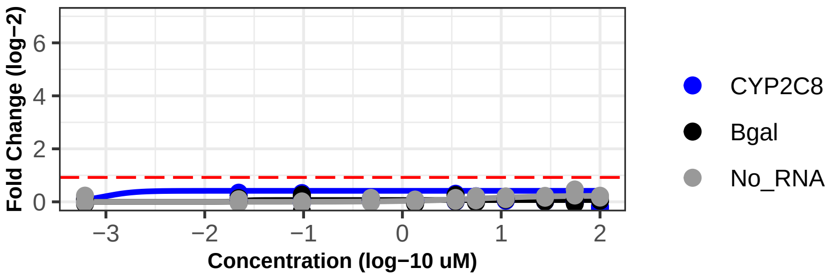


4−(2−Methylbutan−2−yl)phenol: CYP2J2

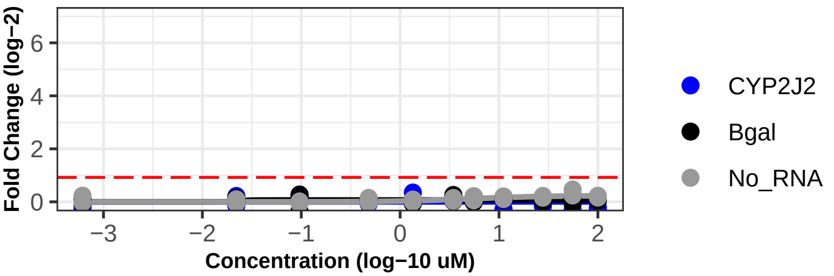


4−(2−Methylbutan−2−yl)phenol: CYP2C9

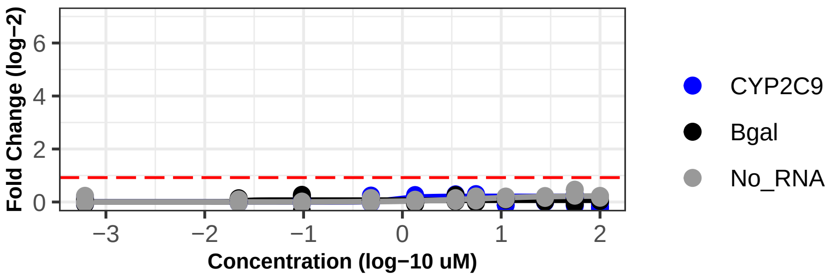


4−(2−Methylbutan−2−yl)phenol: CYP3A4

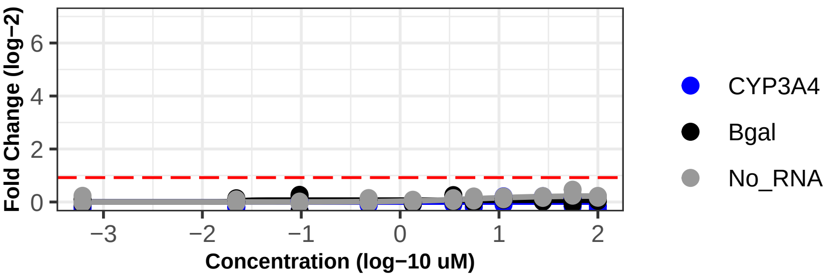

## 4−Androstene−3,17−dione: CYP1A2

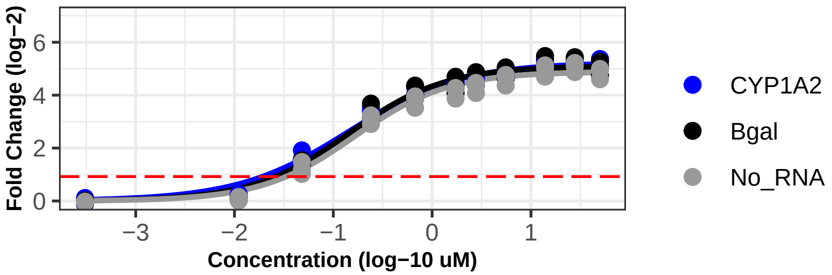


## 4−Androstene−3,17−dione: CYP2C19

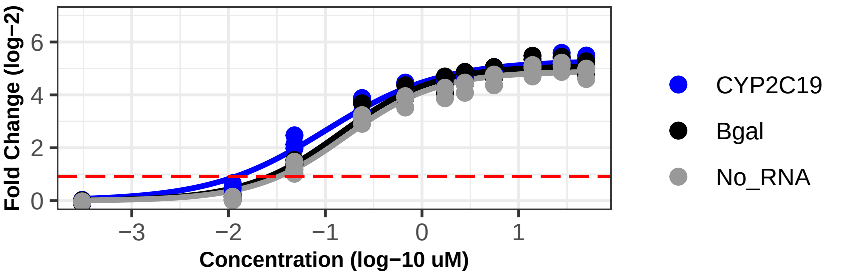


## 4−Androstene−3,17−dione: CYP2A6

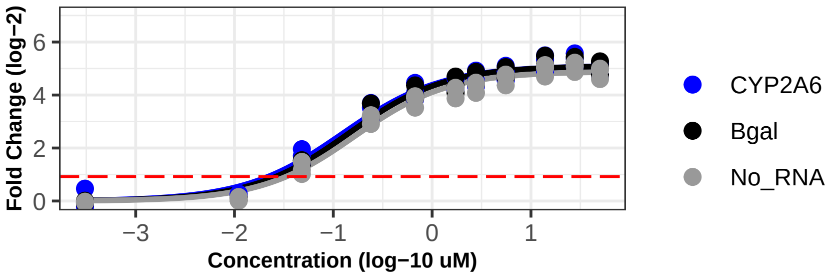


## 4−Androstene−3,17−dione: CYP2D6

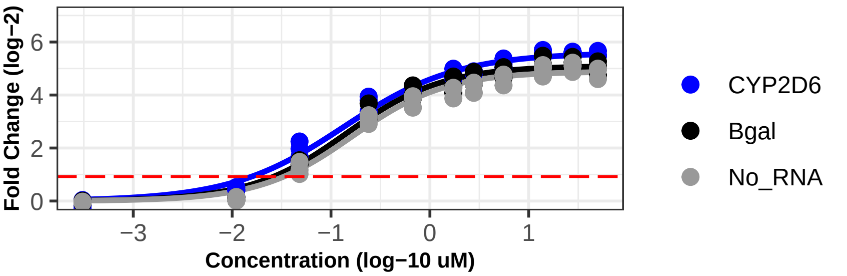


## 4−Androstene−3,17−dione: CYP2B6

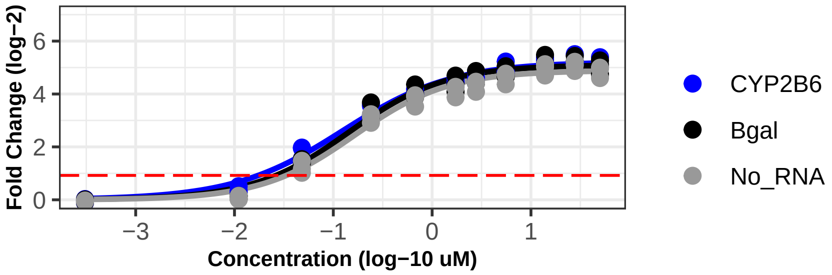


## 4−Androstene−3,17−dione: CYP2E1

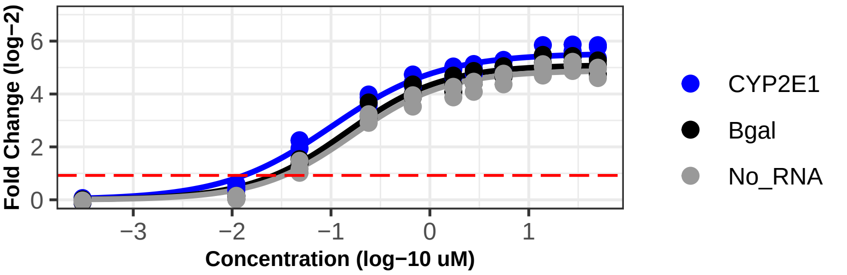


## 4−Androstene−3,17−dione: CYP2C8

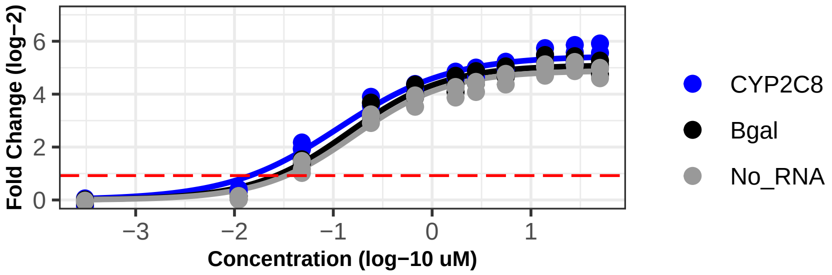


## 4−Androstene−3,17−dione: CYP2J2

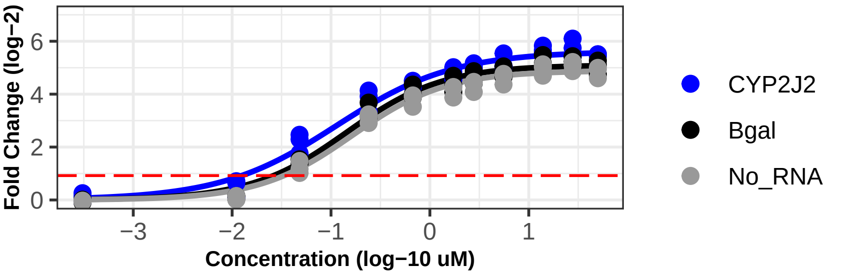


## 4−Androstene−3,17−dione: CYP2C9

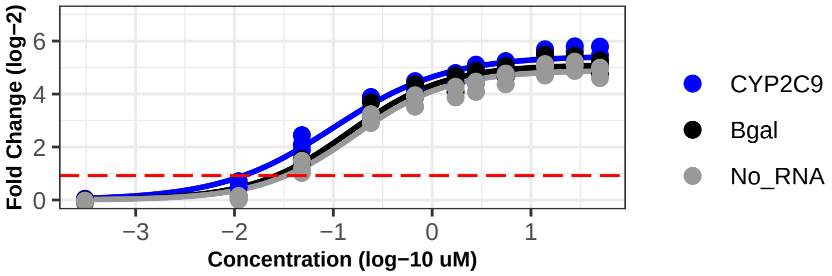


## 4−Androstene−3,17−dione: CYP3A4

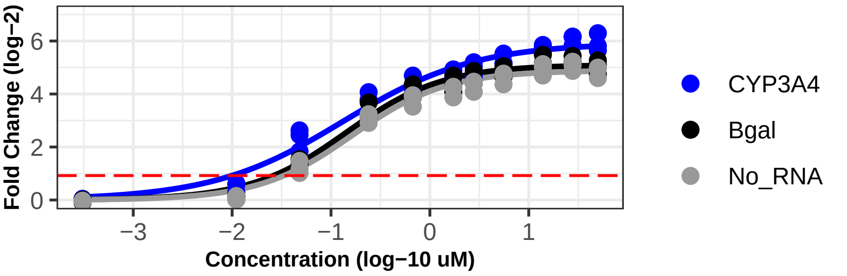

## 4−Cumylphenol: CYP1A2

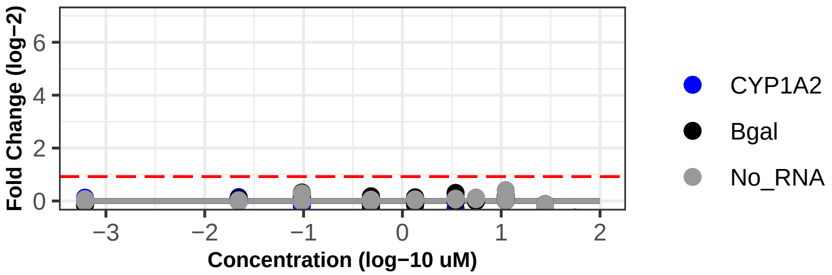


## 4−Cumylphenol: CYP2C19

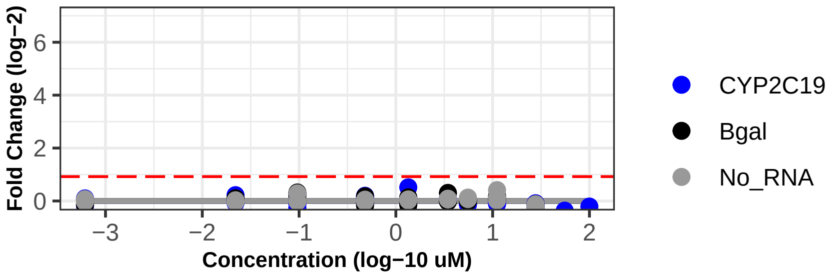


## 4−Cumylphenol: CYP2A6

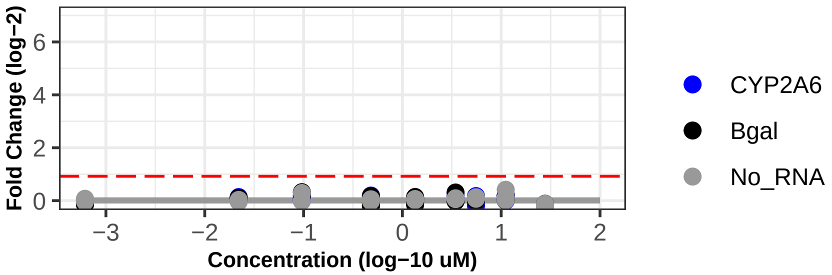


## 4−Cumylphenol: CYP2D6

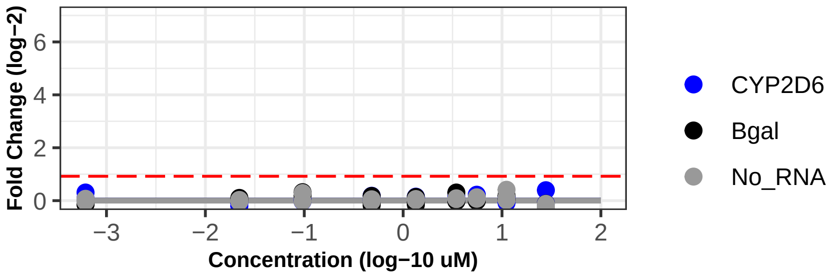


## 4−Cumylphenol: CYP2B6

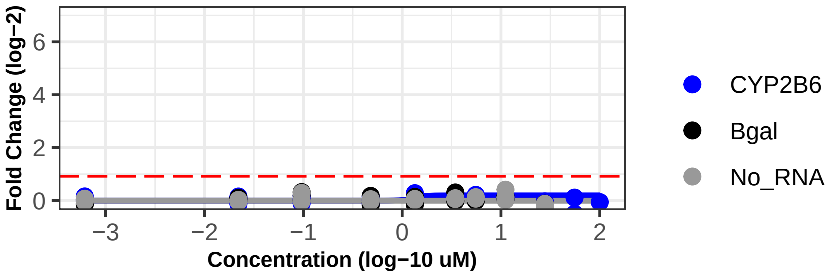


## 4−Cumylphenol: CYP2E1

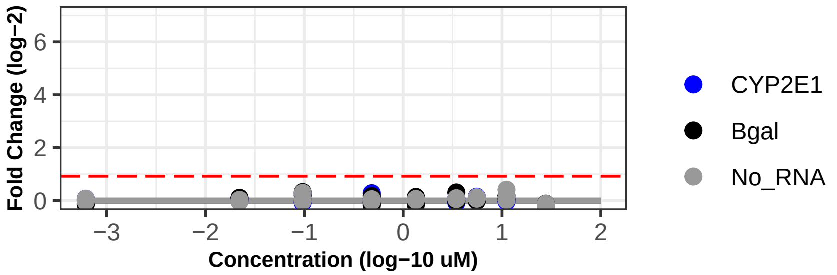


## 4−Cumylphenol: CYP2C8

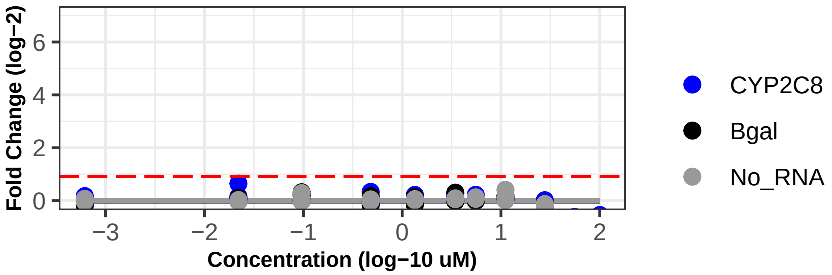


## 4−Cumylphenol: CYP2J2

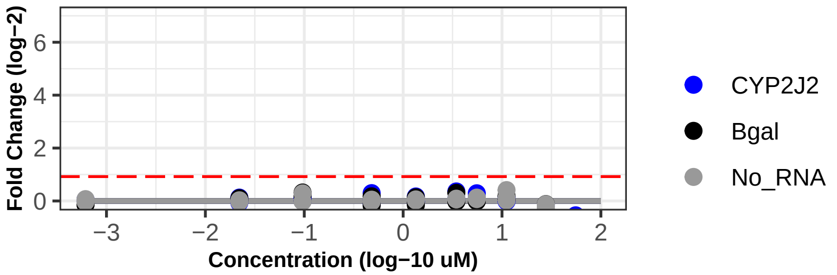


## 4−Cumylphenol: CYP2C9

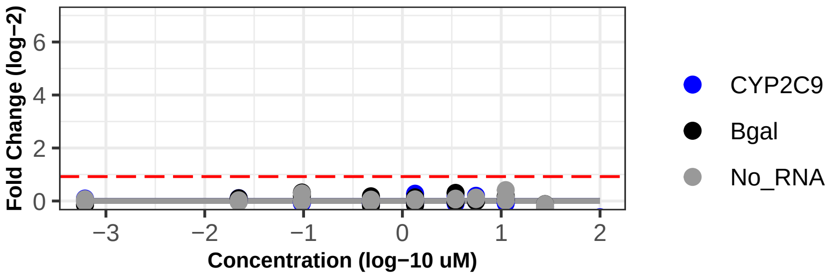


## 4−Cumylphenol: CYP3A4

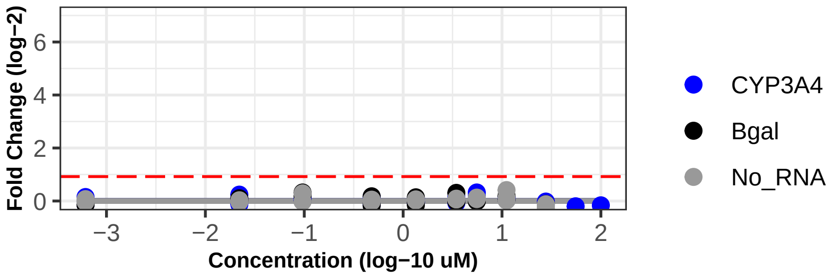

### 4−Dodecylphenol: CYP1A2

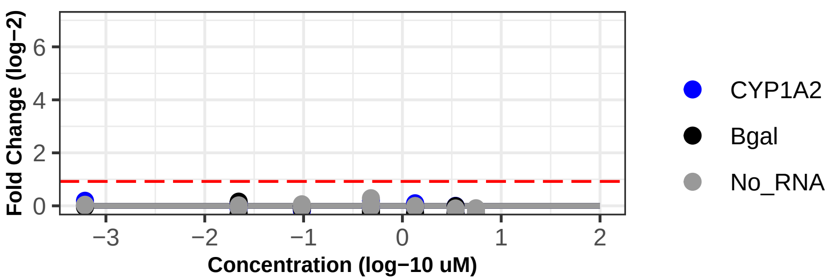


### 4−Dodecylphenol: CYP2C19

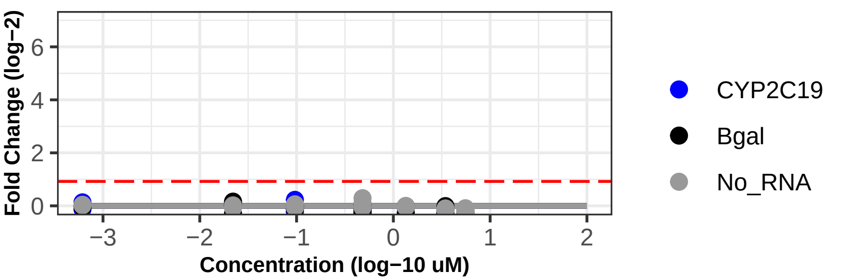


### 4−Dodecylphenol: CYP2A6

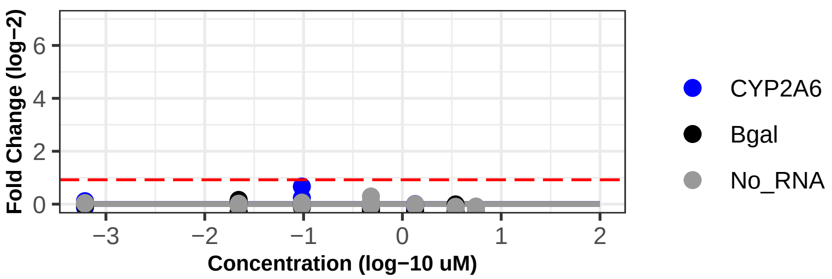


### 4−Dodecylphenol: CYP2D6

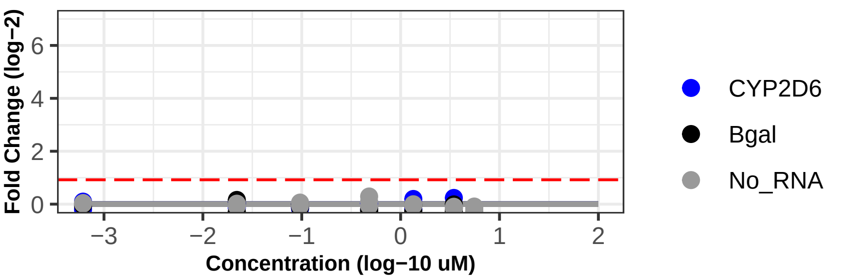


### 4−Dodecylphenol: CYP2B6

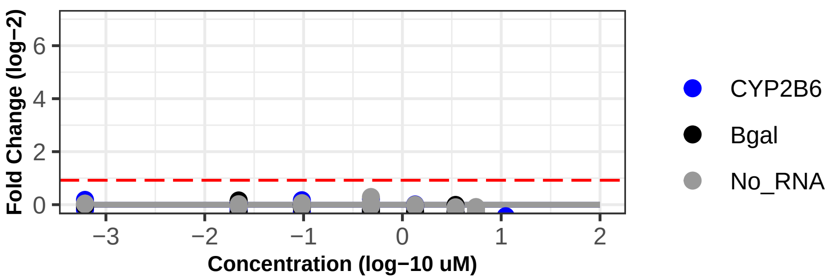


### 4−Dodecylphenol: CYP2E1

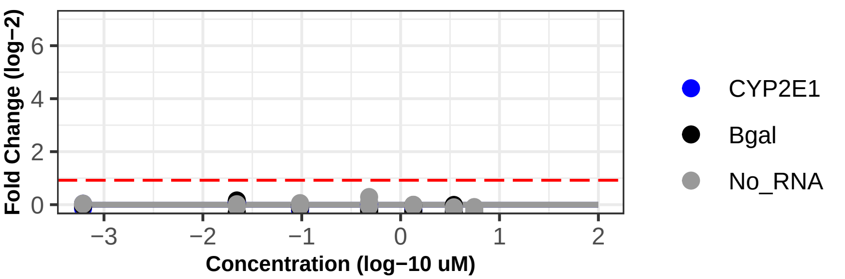


### 4−Dodecylphenol: CYP2C8

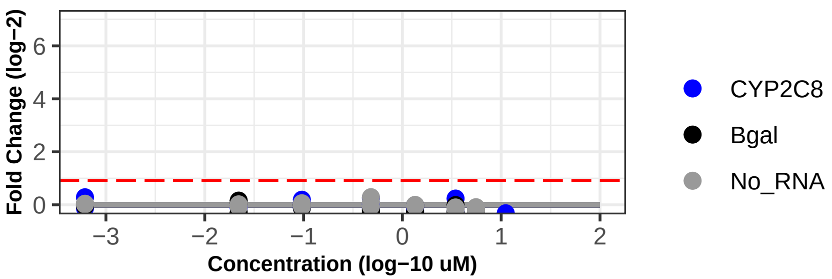


### 4−Dodecylphenol: CYP2J2

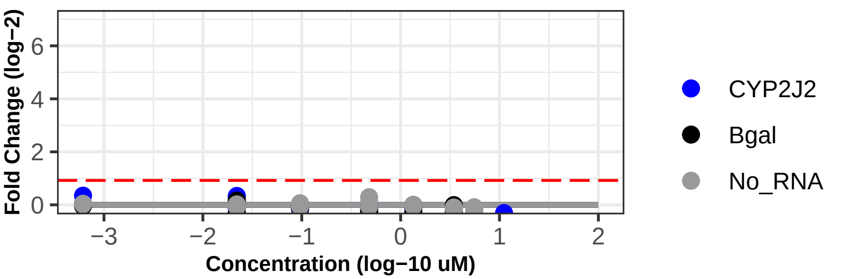


### 4−Dodecylphenol: CYP2C9

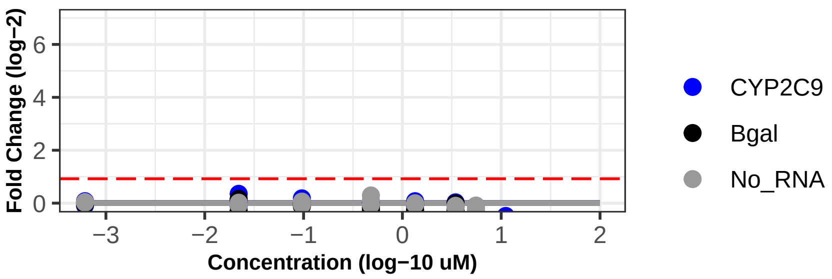


### 4−Dodecylphenol: CYP3A4

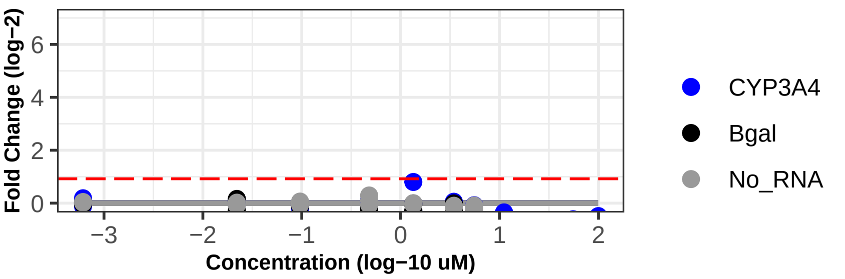

## 4−Hydroxybenzoic acid: CYP1A2

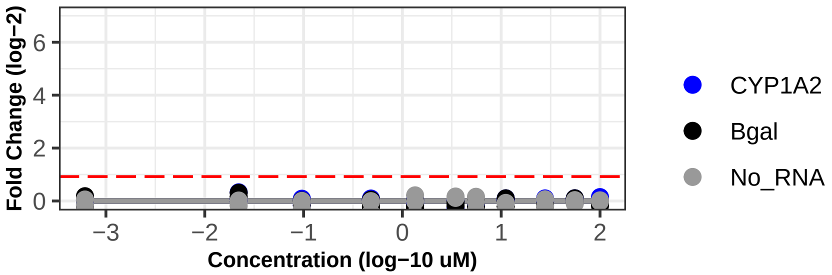


## 4−Hydroxybenzoic acid: CYP2C19

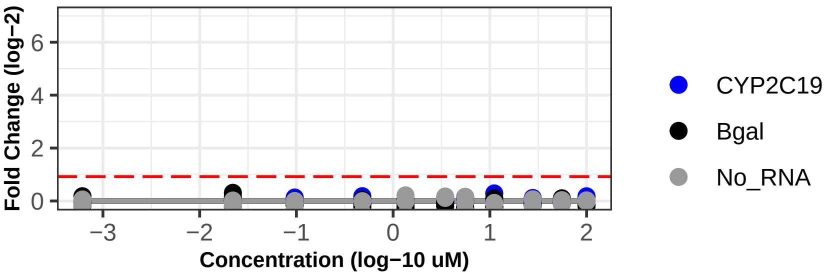


## 4−Hydroxybenzoic acid: CYP2A6

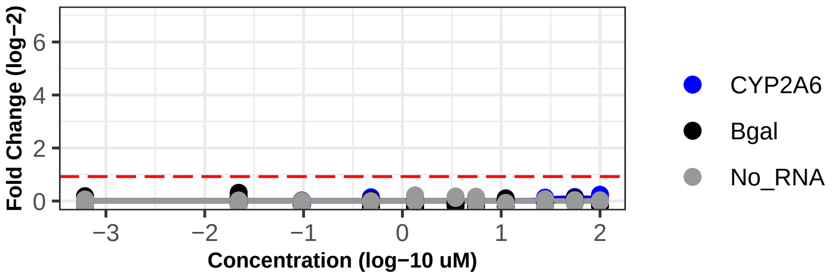


## 4−Hydroxybenzoic acid: CYP2D6

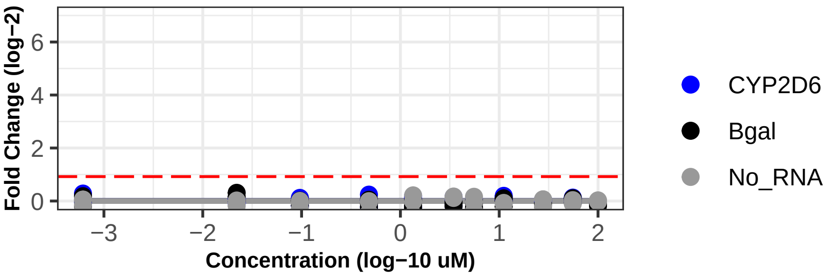


## 4−Hydroxybenzoic acid: CYP2B6

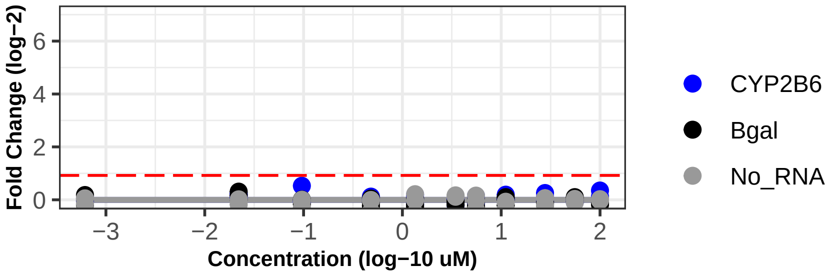


## 4−Hydroxybenzoic acid: CYP2E1

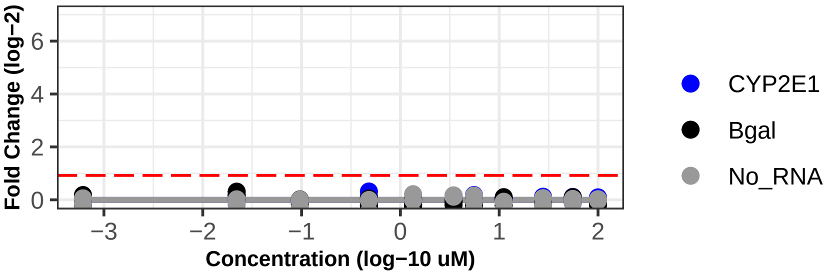


## 4−Hydroxybenzoic acid: CYP2C8

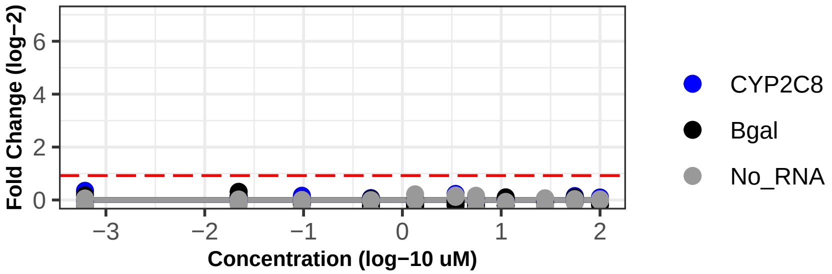


## 4−Hydroxybenzoic acid: CYP2J2

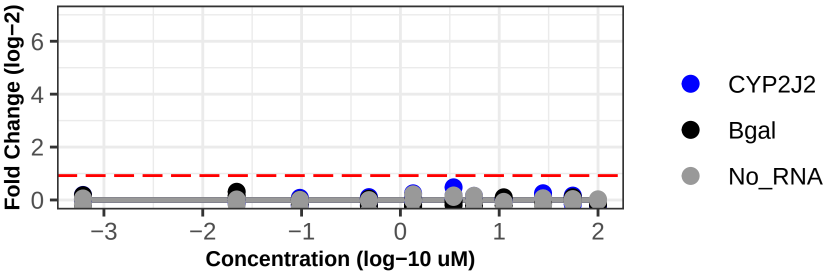


## 4−Hydroxybenzoic acid: CYP2C9

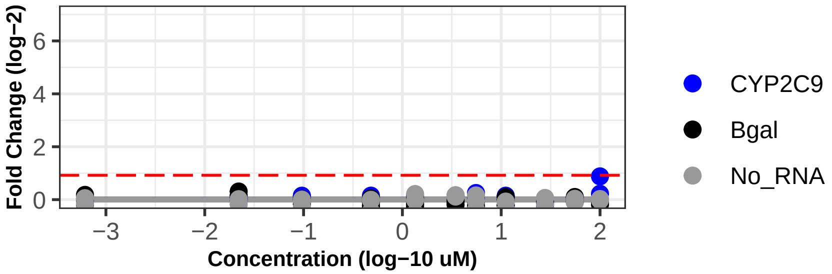


## 4−Hydroxybenzoic acid: CYP3A4

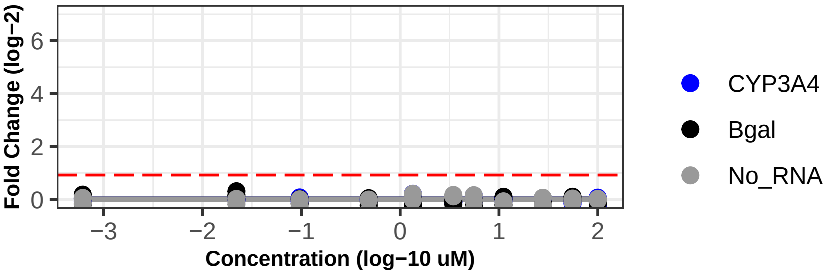

## 4−Nonylphenol: CYP1A2

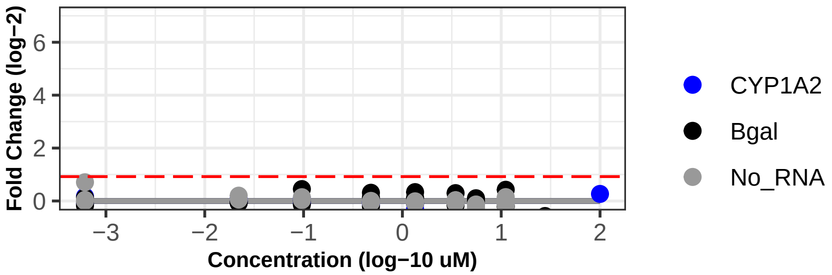


## 4−Nonylphenol: CYP2C19

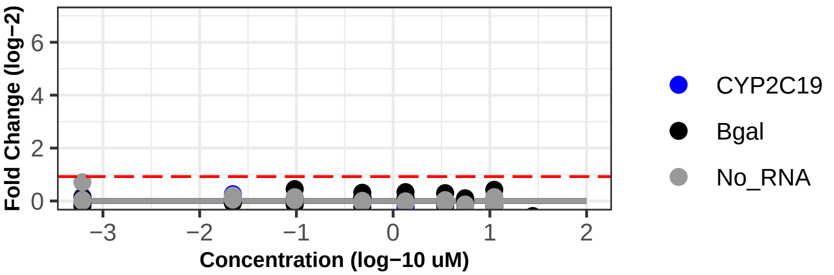


## 4−Nonylphenol: CYP2A6

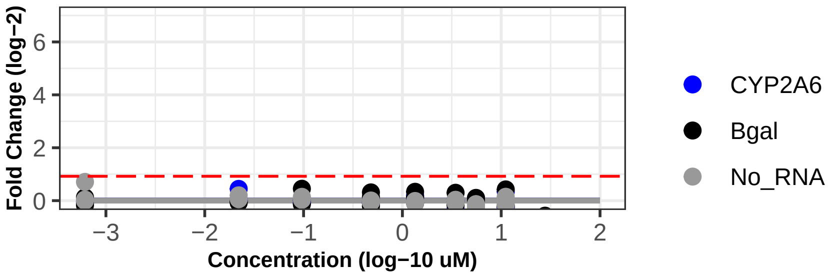


## 4−Nonylphenol: CYP2D6

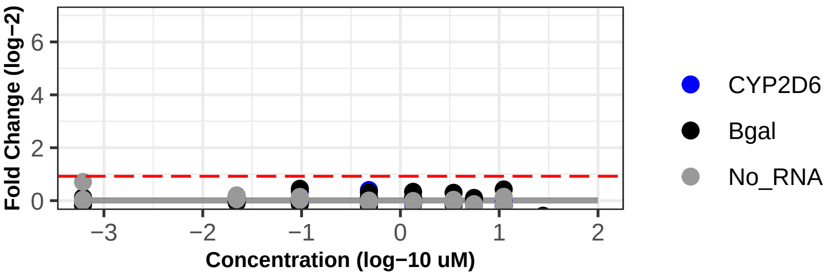


## 4−Nonylphenol: CYP2B6

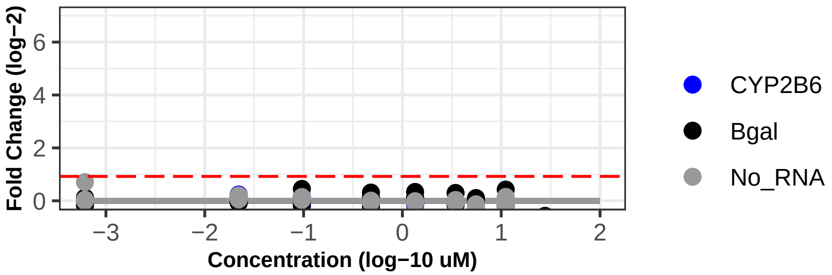


## 4−Nonylphenol: CYP2E1

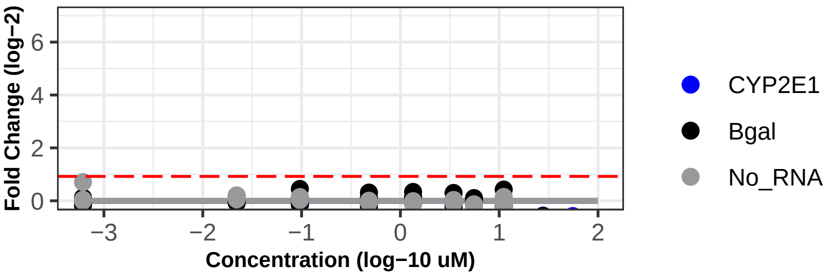


## 4−Nonylphenol: CYP2C8

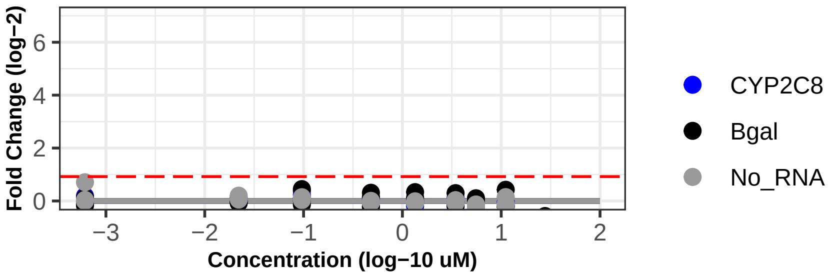


## 4−Nonylphenol: CYP2J2

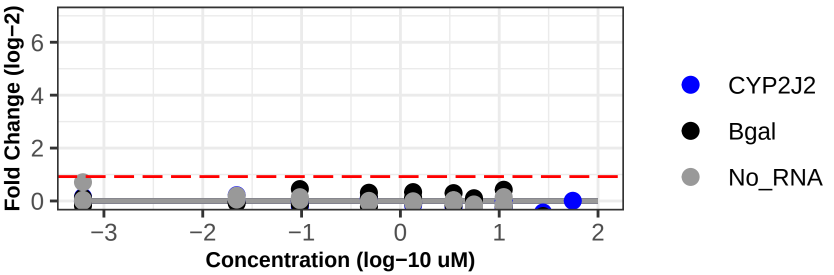


## 4−Nonylphenol: CYP2C9

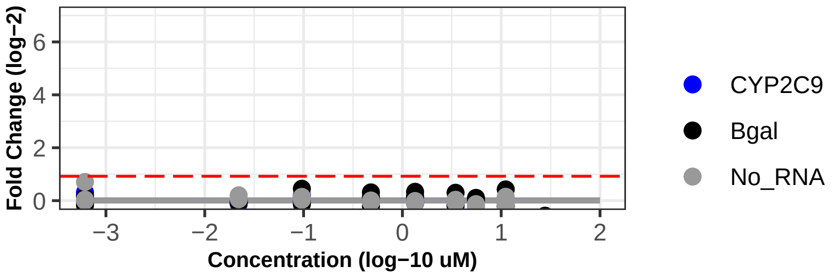


## 4−Nonylphenol: CYP3A4

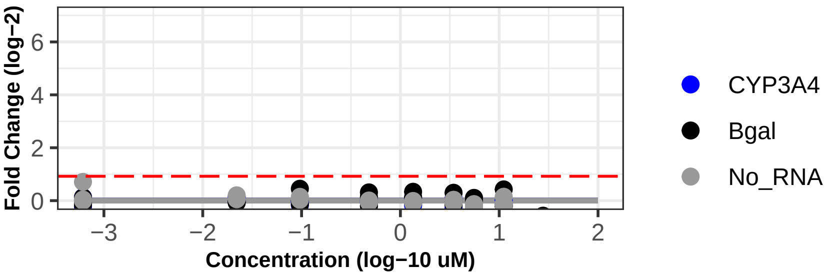

## 4−tert−Butylphenol: CYP1A2

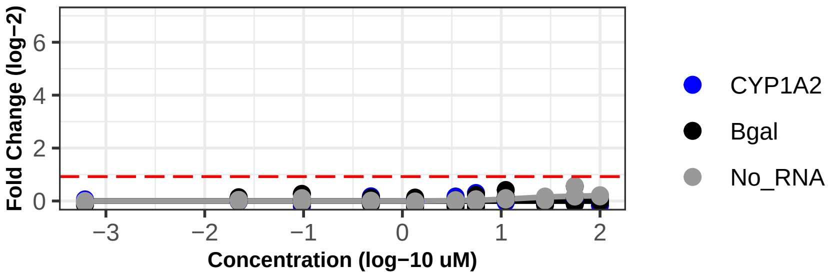


## 4−tert−Butylphenol: CYP2C19

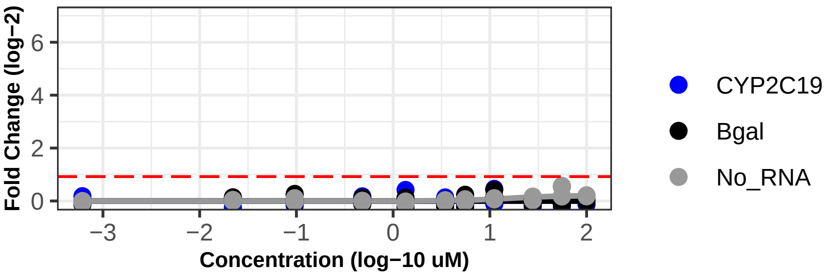


## 4−tert−Butylphenol: CYP2A6

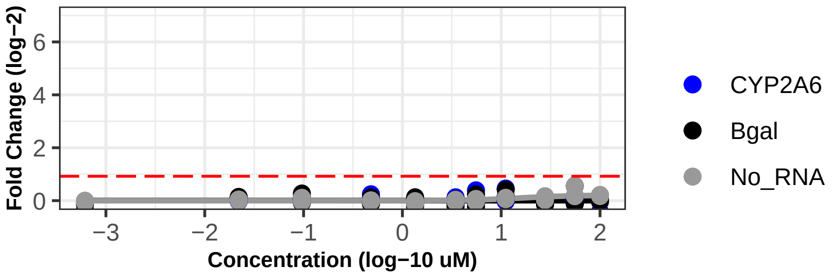


## 4−tert−Butylphenol: CYP2D6

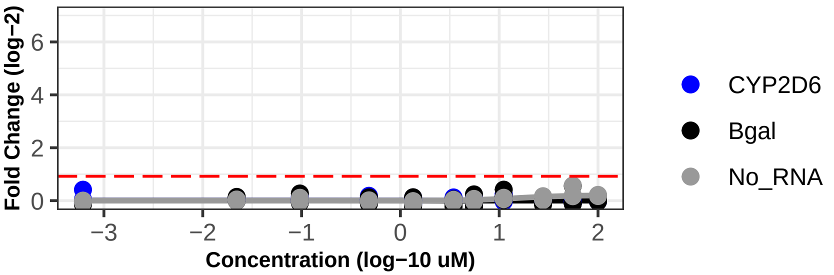


## 4−tert−Butylphenol: CYP2B6

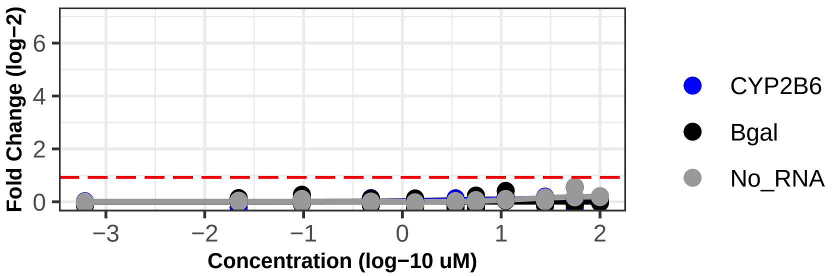


## 4−tert−Butylphenol: CYP2E1

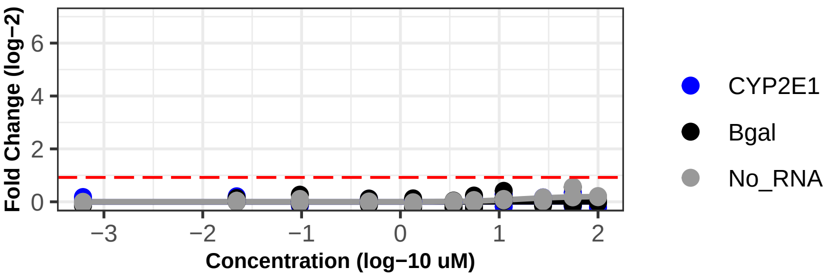


## 4−tert−Butylphenol: CYP2C8

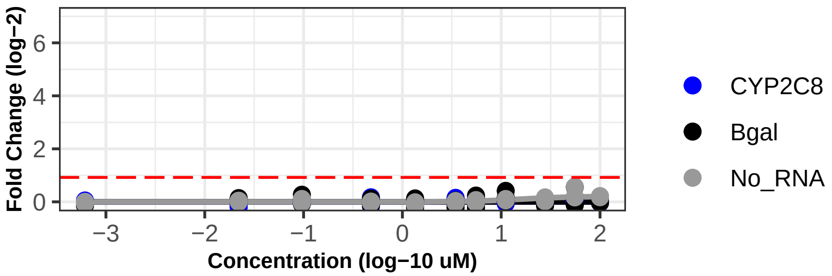


## 4−tert−Butylphenol: CYP2J2

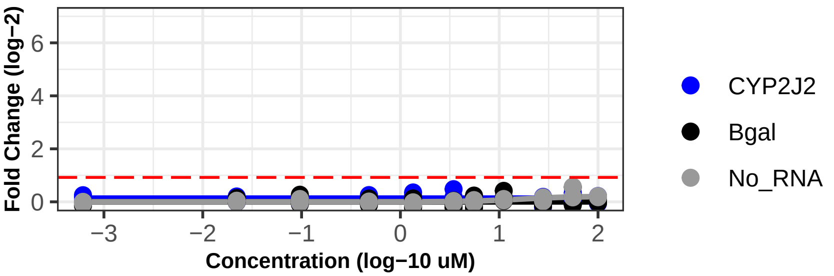


## 4−tert−Butylphenol: CYP2C9

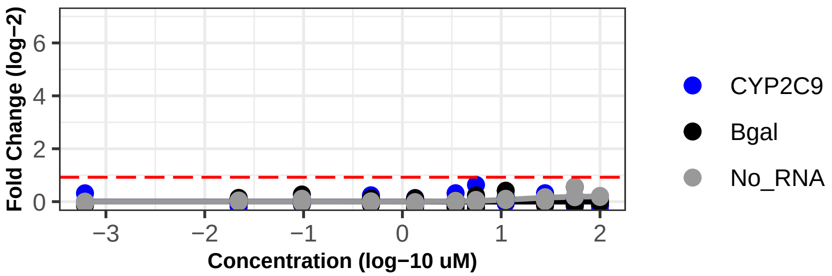


## 4−tert−Butylphenol: CYP3A4

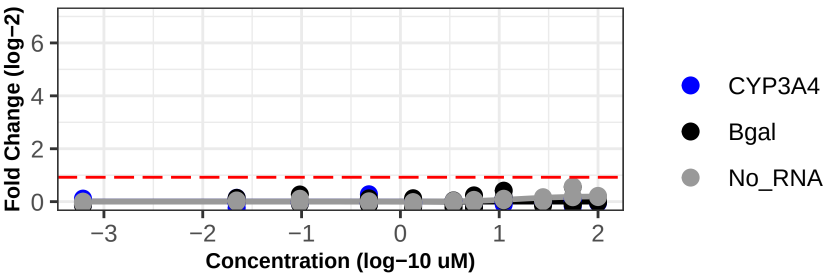

## 5alpha−Dihydrotestosterone: CYP1A2

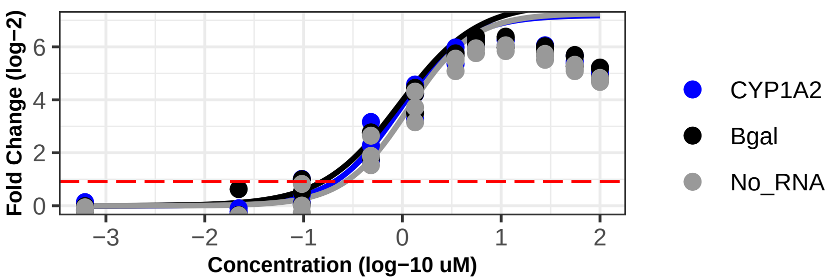


## 5alpha−Dihydrotestosterone: CYP2C19

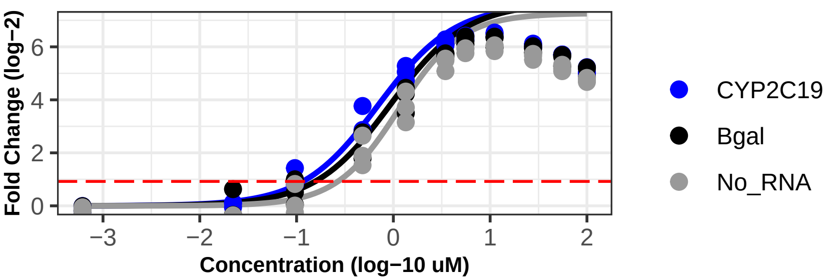


## 5alpha−Dihydrotestosterone: CYP2A6

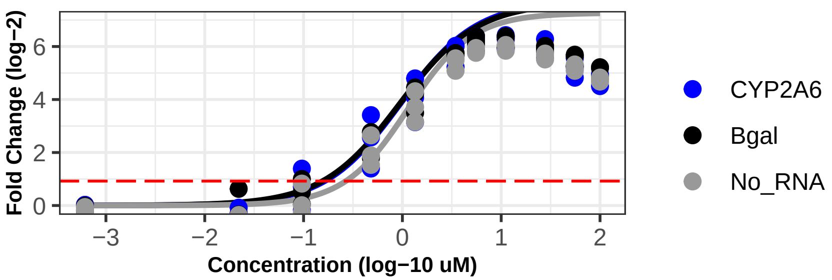


## 5alpha−Dihydrotestosterone: CYP2D6

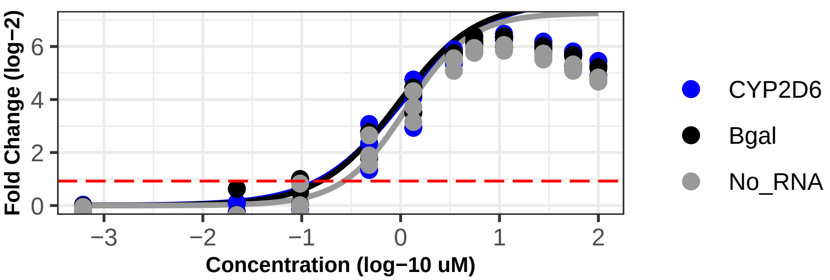


## 5alpha−Dihydrotestosterone: CYP2B6

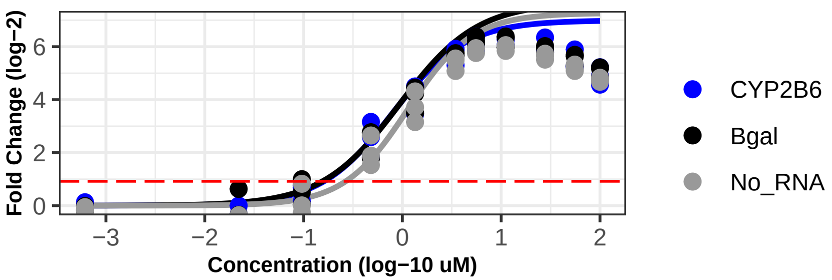


## 5alpha−Dihydrotestosterone: CYP2E1

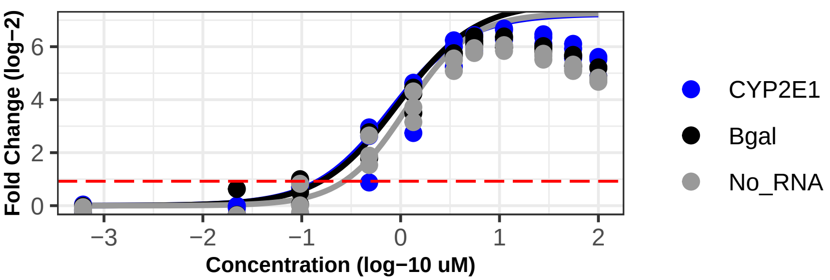


## 5alpha−Dihydrotestosterone: CYP2C8

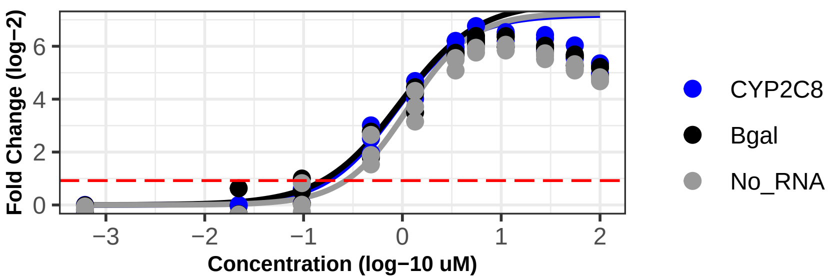


## 5alpha−Dihydrotestosterone: CYP2J2

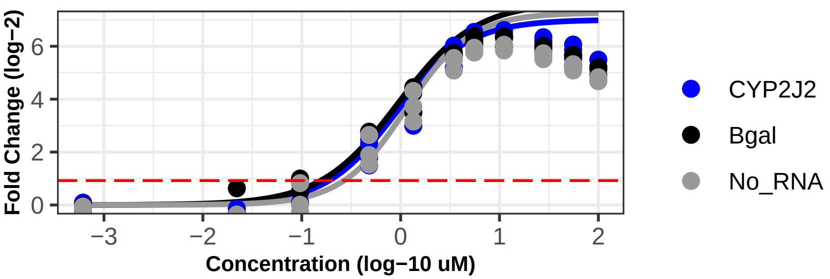


## 5alpha−Dihydrotestosterone: CYP2C9

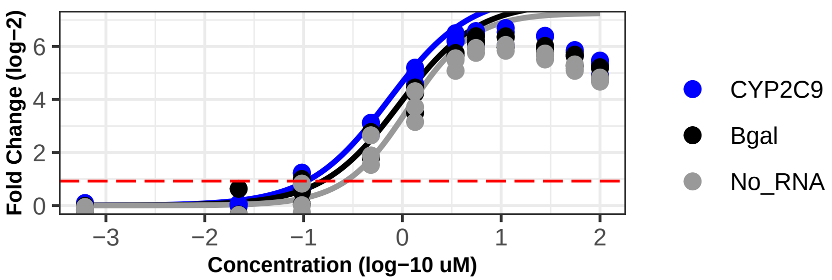


## 5alpha−Dihydrotestosterone: CYP3A4

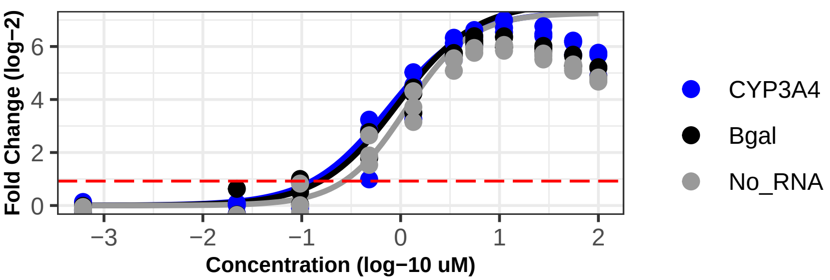

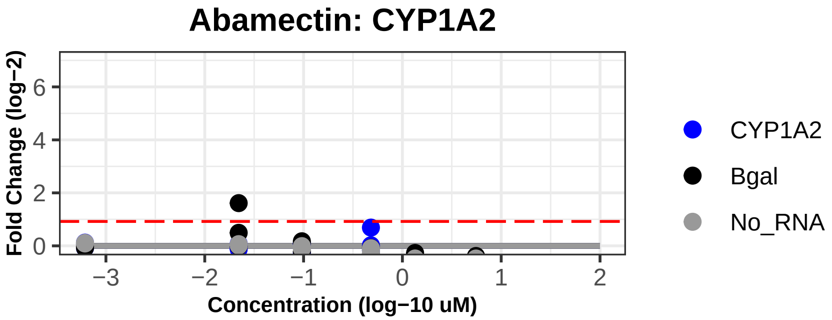
Abamectin: CYP1A2
Fold Change (log−2)
Concentration (log−10 uM)
CYP1A2
Bgal
No_RNA

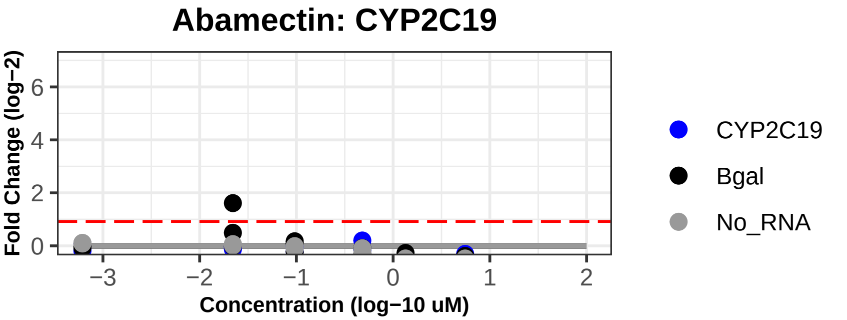
Abamectin: CYP2C19
Fold Change (log−2)
Concentration (log−10 uM)
CYP2C19
Bgal
No_RNA

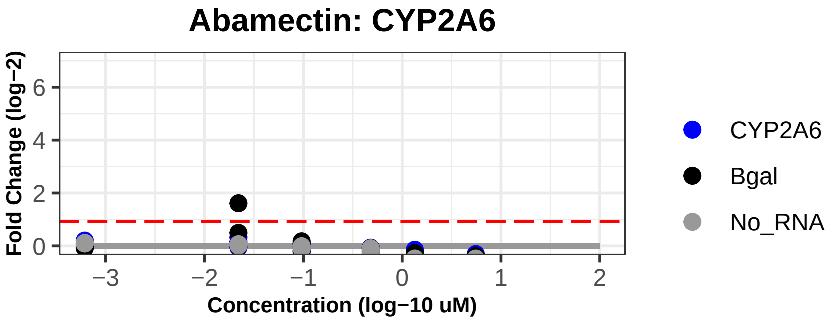
Abamectin: CYP2A6
Fold Change (log−2)
Concentration (log−10 uM)
CYP2A6
Bgal
No_RNA

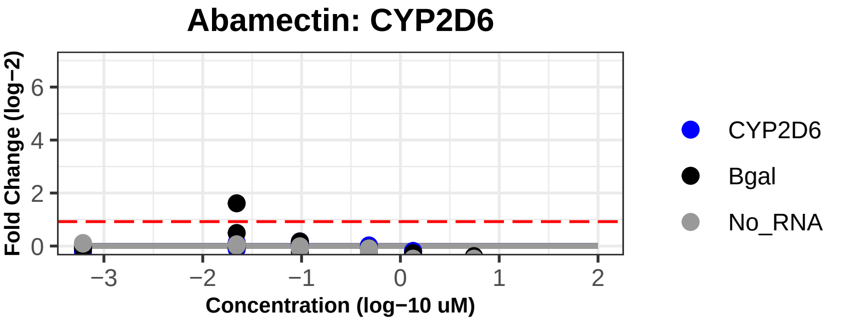
Abamectin: CYP2D6
Fold Change (log−2)
Concentration (log−10 uM)
CYP2D6
Bgal
No_RNA

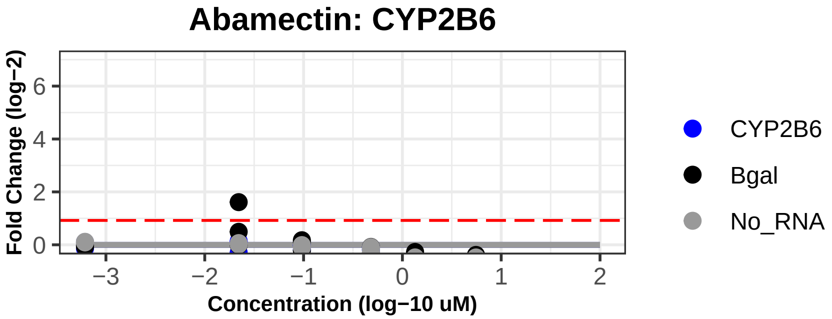
Abamectin: CYP2B6
Fold Change (log−2)
Concentration (log−10 uM)
CYP2B6
Bgal
No_RNA

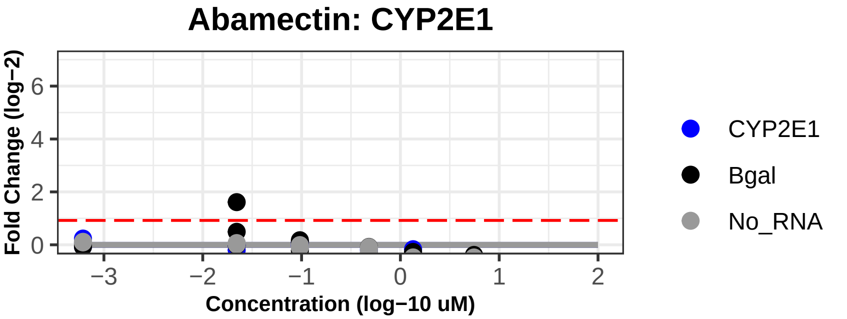
Abamectin: CYP2E1
Fold Change (log−2)
Concentration (log−10 uM)
CYP2E1
Bgal
No_RNA

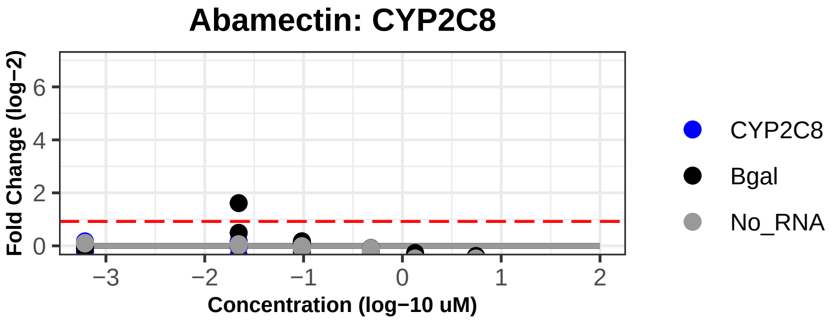
Abamectin: CYP2C8
Fold Change (log−2)
Concentration (log−10 uM)
CYP2C8
Bgal
No_RNA

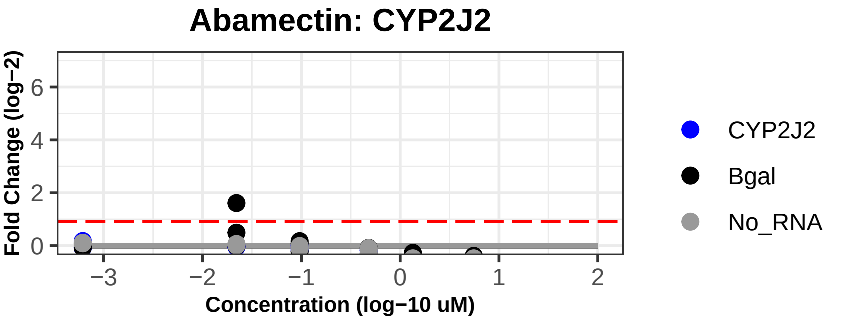
Abamectin: CYP2J2
Fold Change (log−2)
Concentration (log−10 uM)
CYP2J2
Bgal
No_RNA

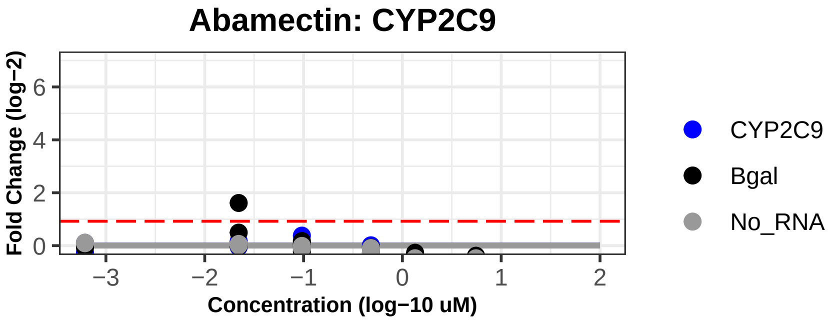
Abamectin: CYP2C9
Fold Change (log−2)
Concentration (log−10 uM)
CYP2C9
Bgal
No_RNA

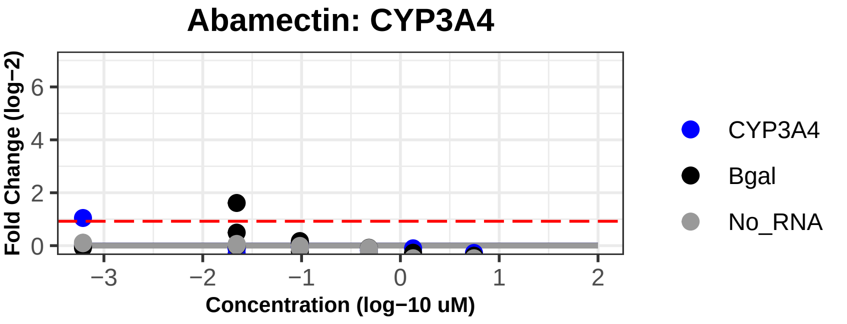
Abamectin: CYP3A4
Fold Change (log−2)
Concentration (log−10 uM)
CYP3A4
Bgal
No_RNA

Acephate: CYP1A2

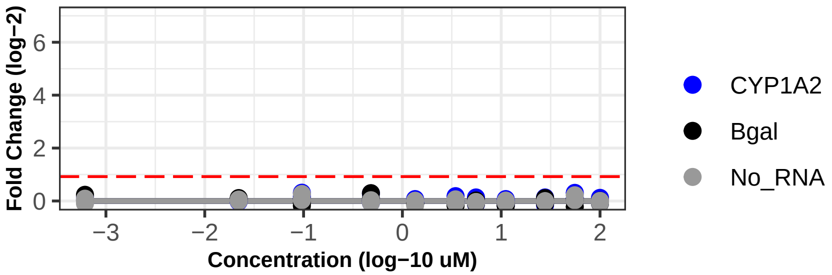


Acephate: CYP2C19

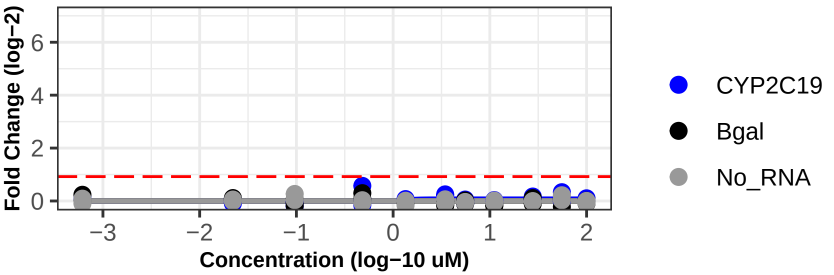


Acephate: CYP2A6

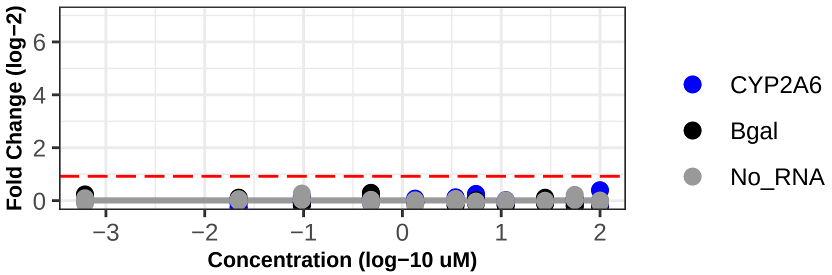


Acephate: CYP2D6

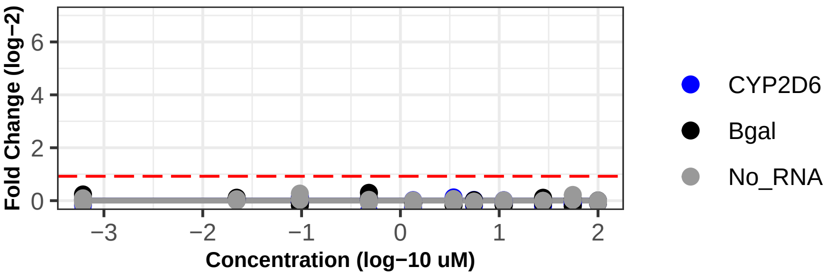


Acephate: CYP2B6

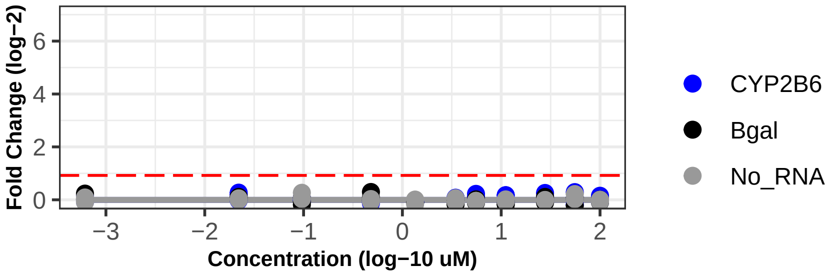


Acephate: CYP2E1

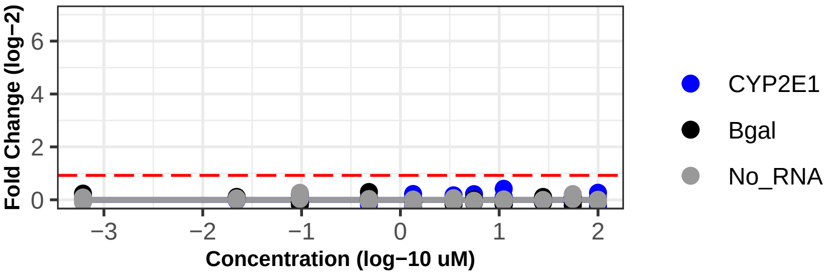


Acephate: CYP2C8

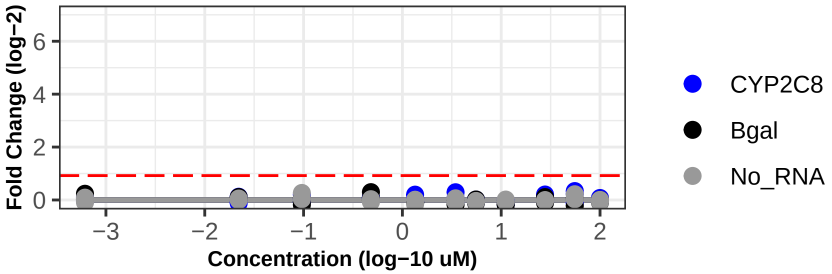


Acephate: CYP2J2

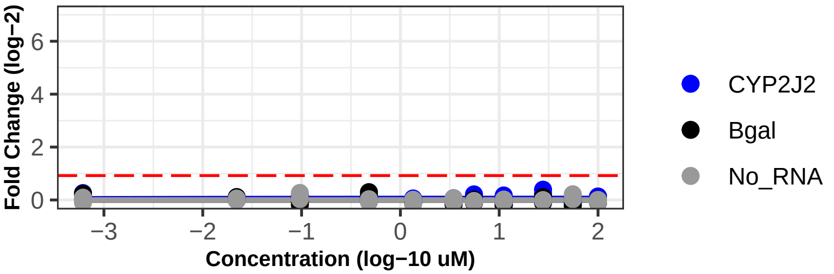


Acephate: CYP2C9

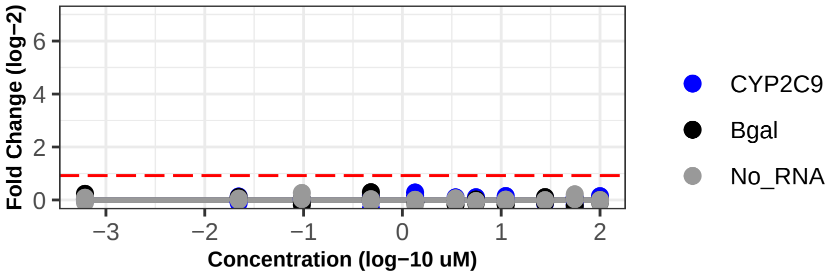


Acephate: CYP3A4

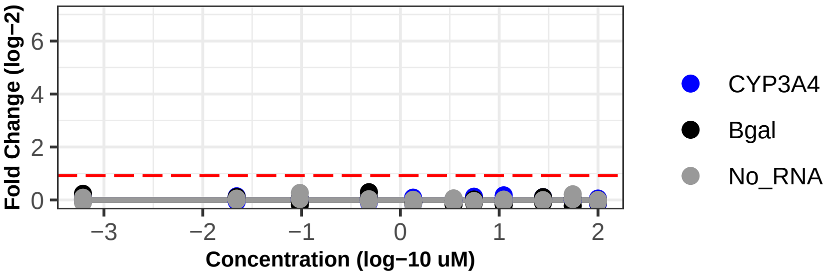

## Afimoxifene (4−Hydroxytamoxifen): CYP1A2

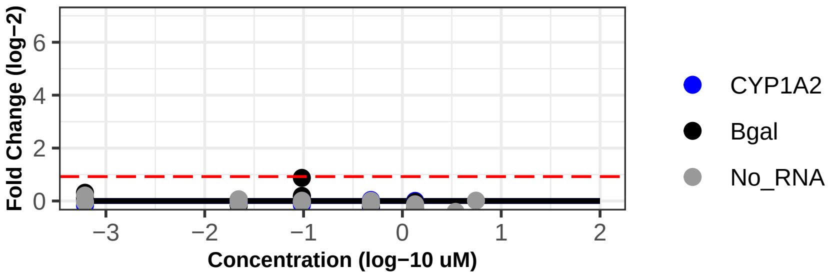


## Afimoxifene (4−Hydroxytamoxifen): CYP2C19

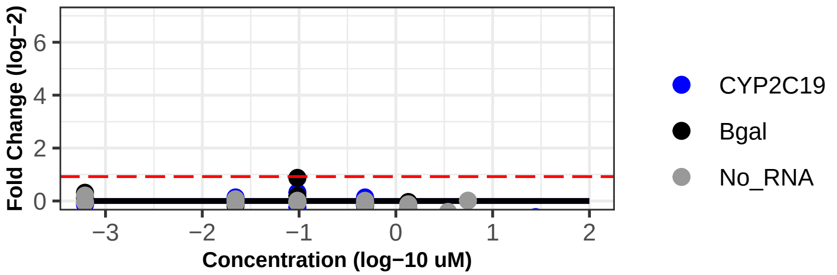


## Afimoxifene (4−Hydroxytamoxifen): CYP2A6

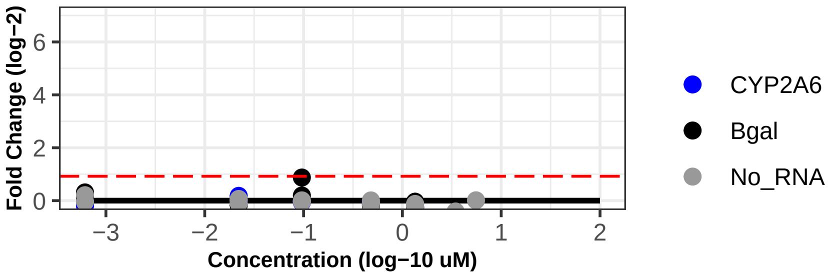


## Afimoxifene (4−Hydroxytamoxifen): CYP2D6

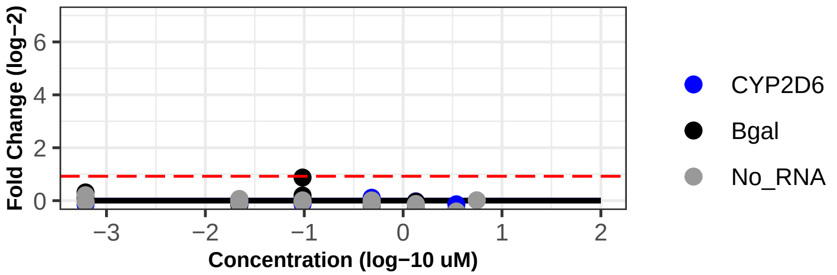


## Afimoxifene (4−Hydroxytamoxifen): CYP2B6

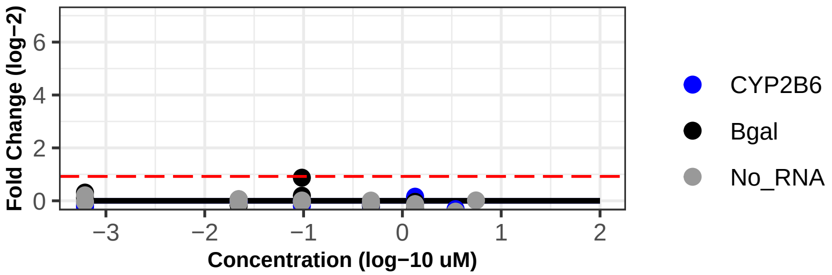


## Afimoxifene (4−Hydroxytamoxifen): CYP2E1

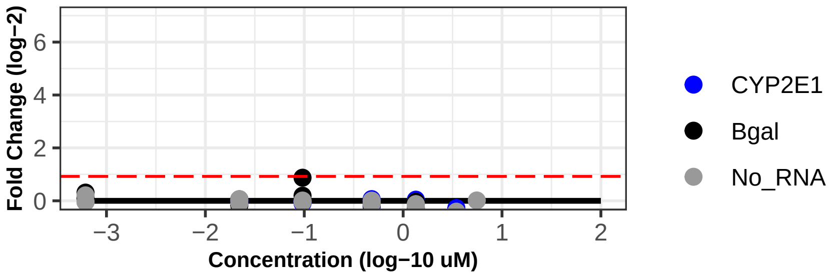


## Afimoxifene (4−Hydroxytamoxifen): CYP2C8

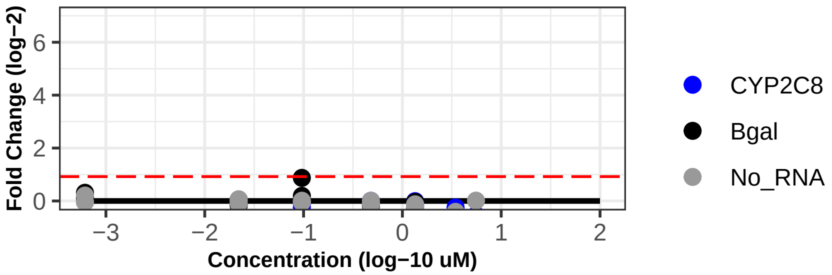


## Afimoxifene (4−Hydroxytamoxifen): CYP2J2

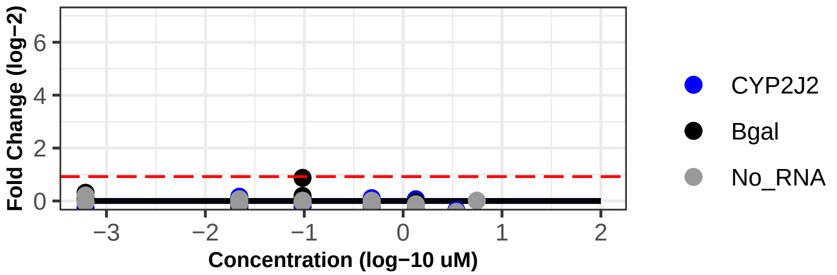


## Afimoxifene (4−Hydroxytamoxifen): CYP2C9

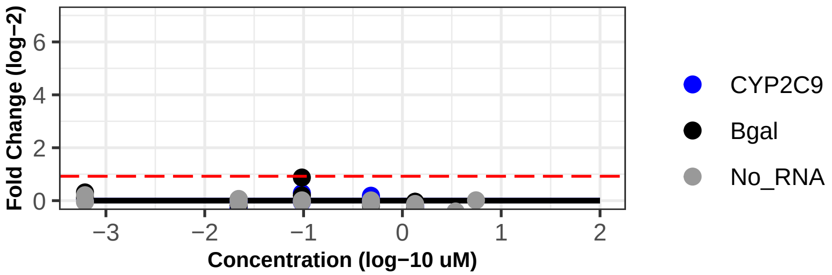


## Afimoxifene (4−Hydroxytamoxifen): CYP3A4

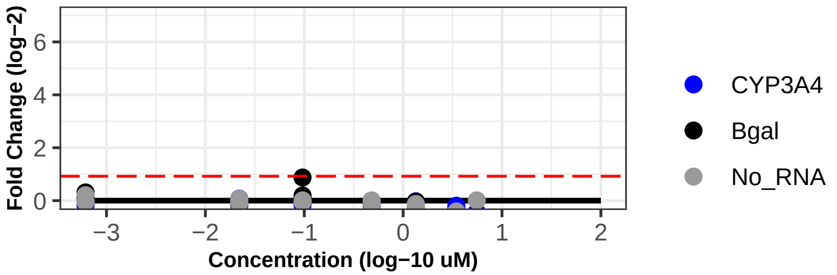

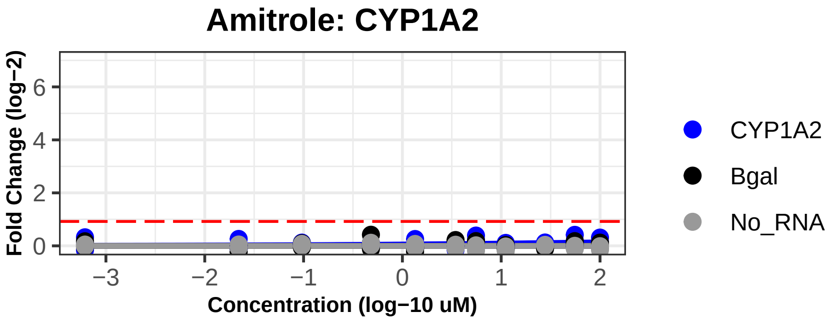
Amitrole: CYP1A2
Fold Change (log−2)
Concentration (log−10 uM)
CYP1A2
Bgal
No_RNA

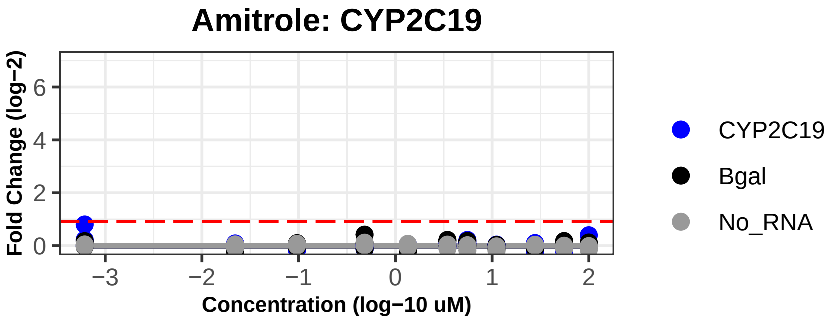
Amitrole: CYP2C19
Fold Change (log−2)
Concentration (log−10 uM)
CYP2C19
Bgal
No_RNA

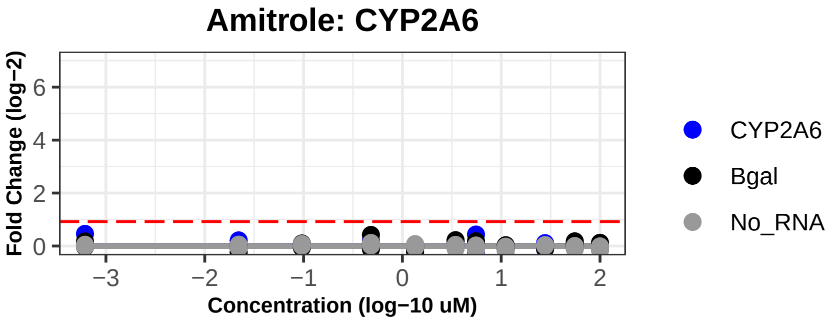
Amitrole: CYP2A6
Fold Change (log−2)
Concentration (log−10 uM)
CYP2A6
Bgal
No_RNA

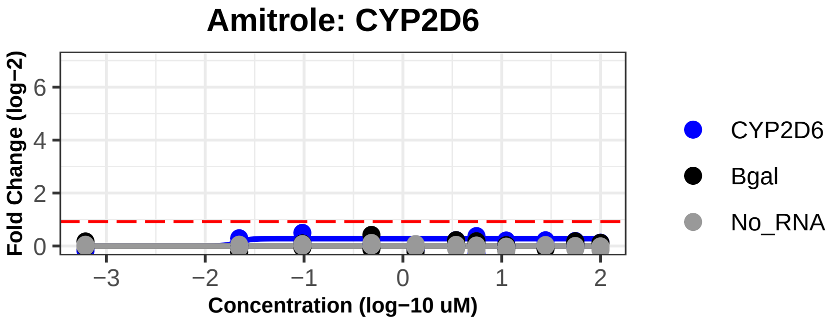
Amitrole: CYP2D6
Fold Change (log−2)
Concentration (log−10 uM)
CYP2D6
Bgal
No_RNA

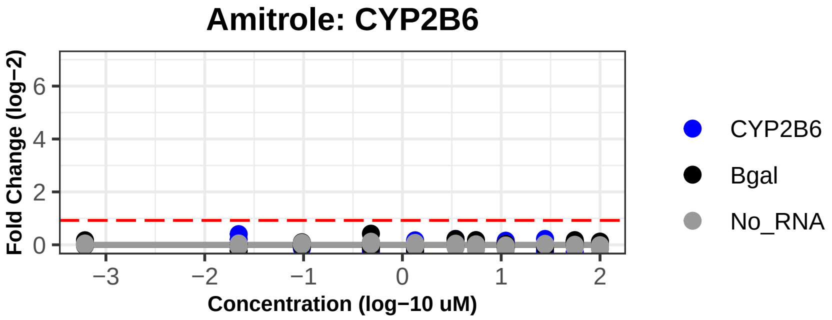
Amitrole: CYP2B6
Fold Change (log−2)
Concentration (log−10 uM)
CYP2B6
Bgal
No_RNA

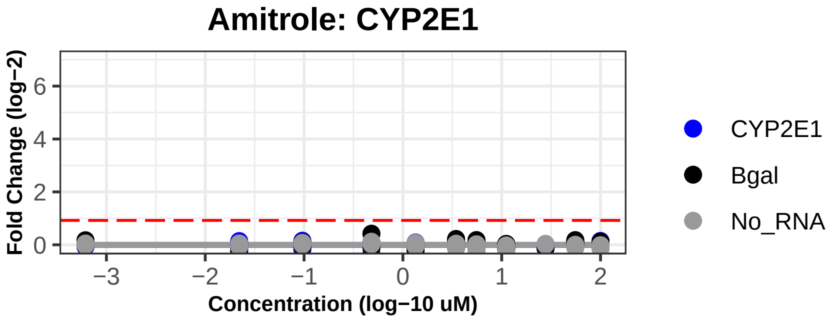
Amitrole: CYP2E1
Fold Change (log−2)
Concentration (log−10 uM)
CYP2E1
Bgal
No_RNA

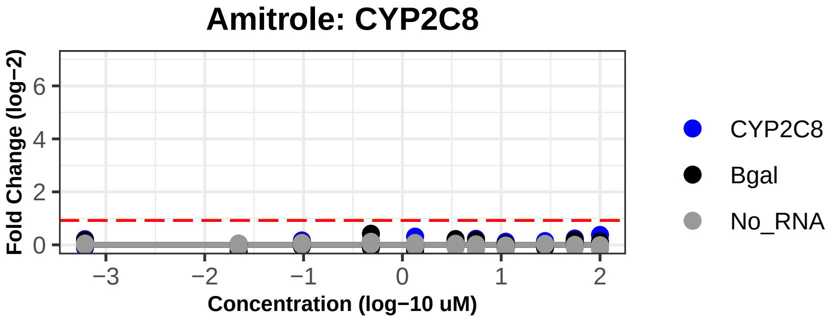
Amitrole: CYP2C8
Fold Change (log−2)
Concentration (log−10 uM)
CYP2C8
Bgal
No_RNA

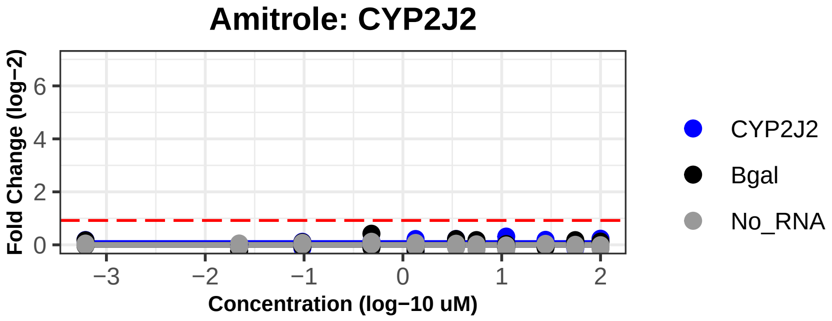
Amitrole: CYP2J2
Fold Change (log−2)
Concentration (log−10 uM)
CYP2J2
Bgal
No_RNA

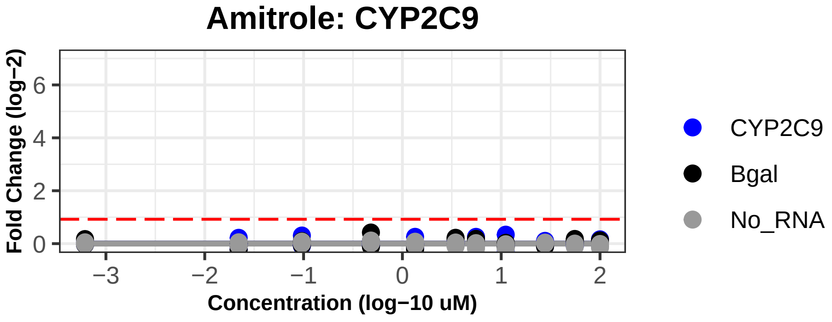
Amitrole: CYP2C9
Fold Change (log−2)
Concentration (log−10 uM)
CYP2C9
Bgal
No_RNA

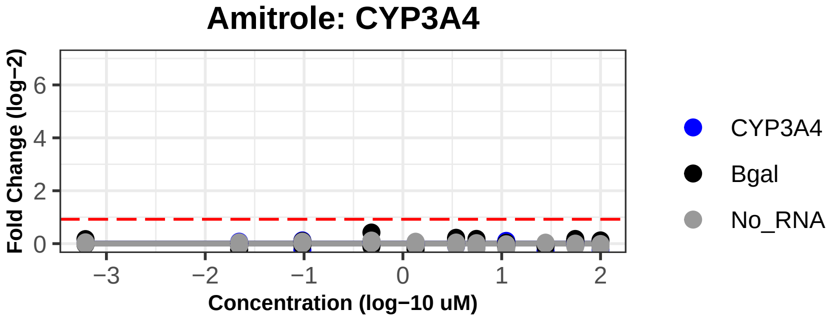
Amitrole: CYP3A4
Fold Change (log−2)
Concentration (log−10 uM)
CYP3A4
Bgal
No_RNA

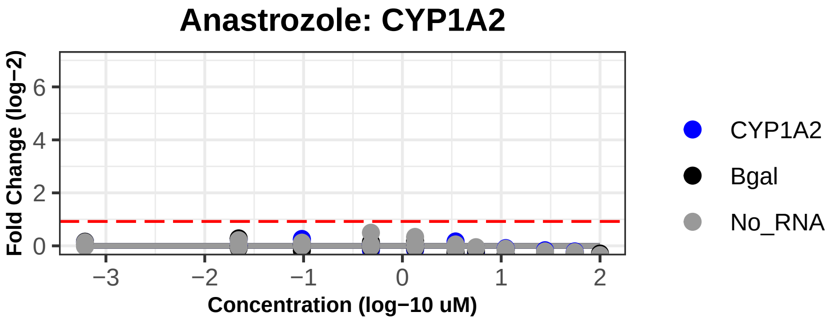
Anastrozole: CYP1A2
Fold Change (log−2)
Concentration (log−10 uM)
CYP1A2
Bgal
No_RNA

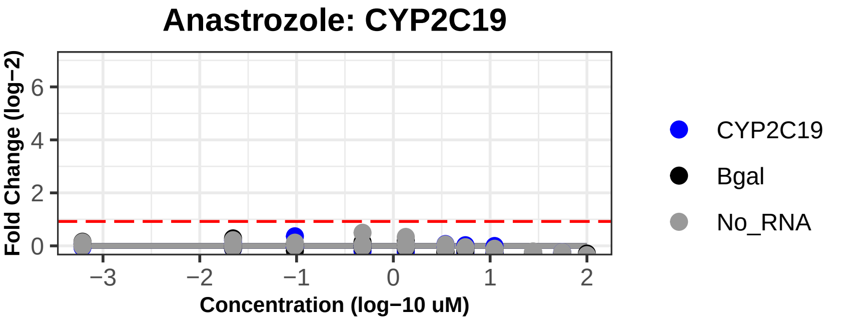
Anastrozole: CYP2C19
Fold Change (log−2)
Concentration (log−10 uM)
CYP2C19
Bgal
No_RNA

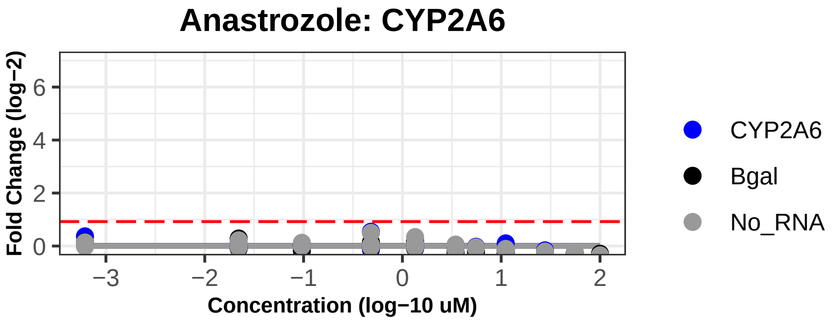
Anastrozole: CYP2A6
Fold Change (log−2)
Concentration (log−10 uM)
CYP2A6
Bgal
No_RNA

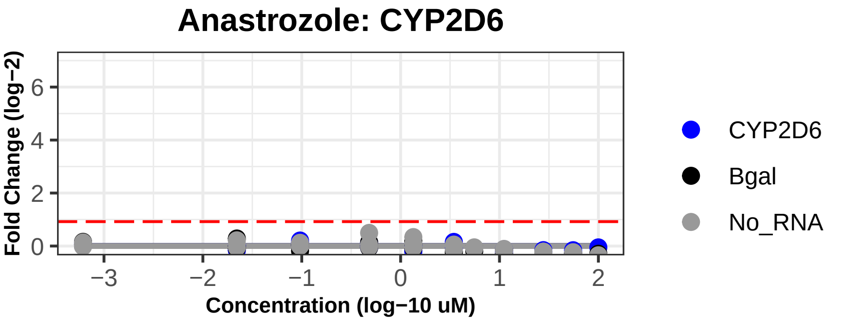
Anastrozole: CYP2D6
Fold Change (log−2)
Concentration (log−10 uM)
CYP2D6
Bgal
No_RNA

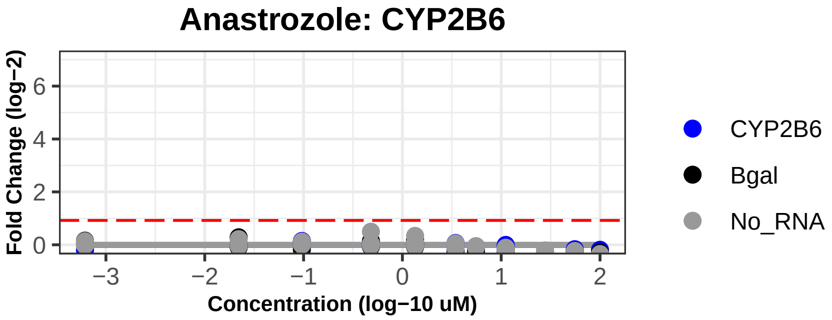
Anastrozole: CYP2B6
Fold Change (log−2)
Concentration (log−10 uM)
CYP2B6
Bgal
No_RNA

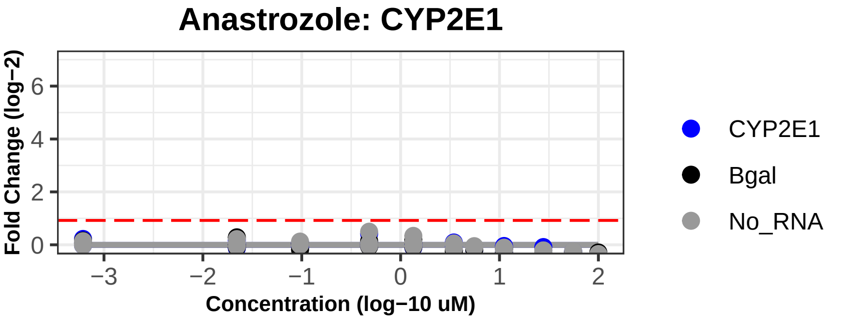
Anastrozole: CYP2E1
Fold Change (log−2)
Concentration (log−10 uM)
CYP2E1
Bgal
No_RNA

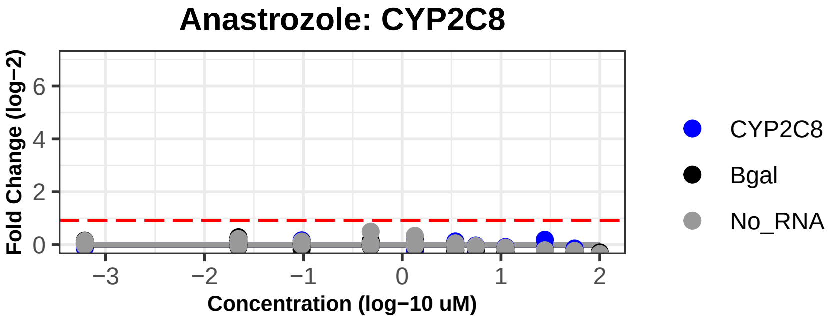
Anastrozole: CYP2C8
Fold Change (log−2)
Concentration (log−10 uM)
CYP2C8
Bgal
No_RNA

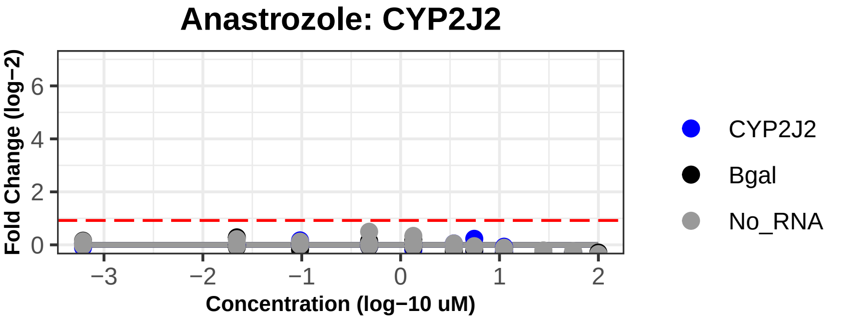
Anastrozole: CYP2J2
Fold Change (log−2)
Concentration (log−10 uM)
CYP2J2
Bgal
No_RNA

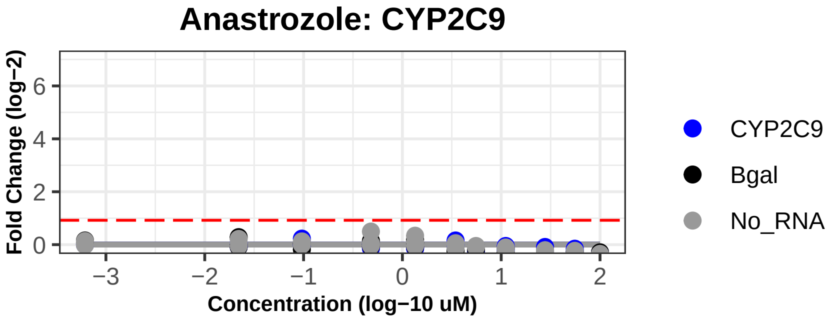
Anastrozole: CYP2C9
Fold Change (log−2)
Concentration (log−10 uM)
CYP2C9
Bgal
No_RNA

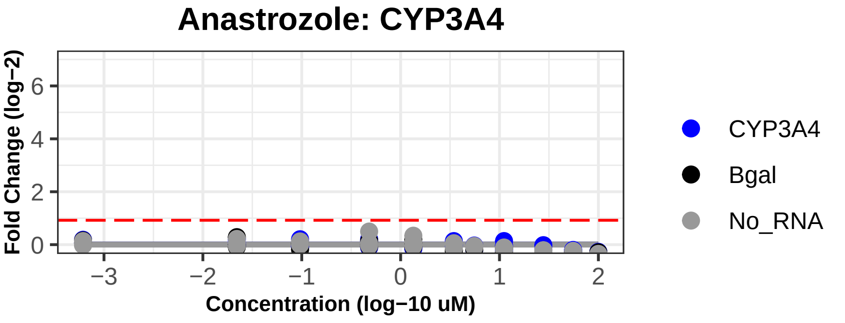
Anastrozole: CYP3A4
Fold Change (log−2)
Concentration (log−10 uM)
CYP3A4
Bgal
No_RNA

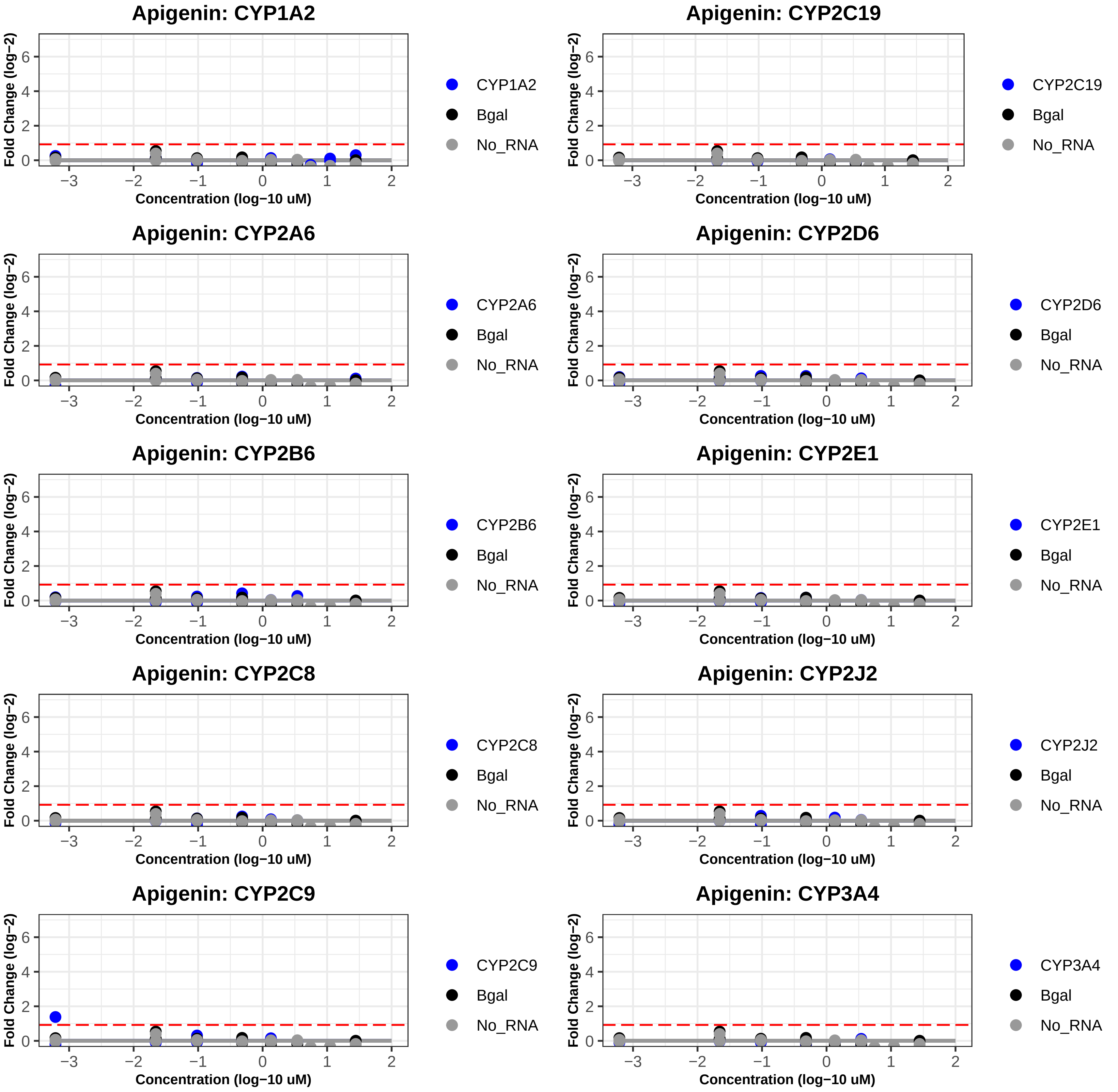
Apigenin: CYP1A2
CYP1A2
Bgal
No_RNA
Apigenin: CYP2C19
CYP2C19
Bgal
No_RNA
Apigenin: CYP2A6
CYP2A6
Bgal
No_RNA
Apigenin: CYP2D6
CYP2D6
Bgal
No_RNA
Apigenin: CYP2B6
CYP2B6
Bgal
No_RNA
Apigenin: CYP2E1
CYP2E1
Bgal
No_RNA
Apigenin: CYP2C8
CYP2C8
Bgal
No_RNA
Apigenin: CYP2J2
CYP2J2
Bgal
No_RNA
Apigenin: CYP2C9
CYP2C9
Bgal
No_RNA
Apigenin: CYP3A4
CYP3A4
Bgal
No_RNA
Fold Change (log−2)
Concentration (log−10 uM)
0
2
4
6
−3
−2
−1
0
1
2

Atrazine: CYP1A2

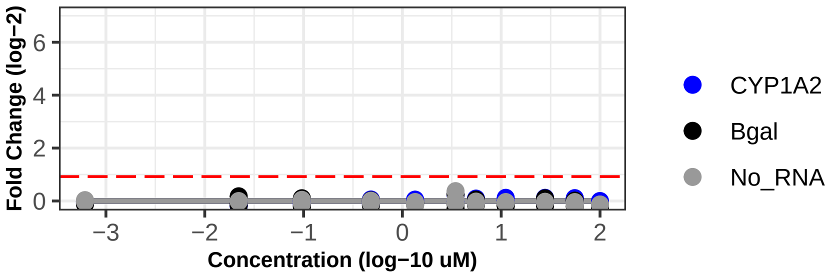


Atrazine: CYP2C19

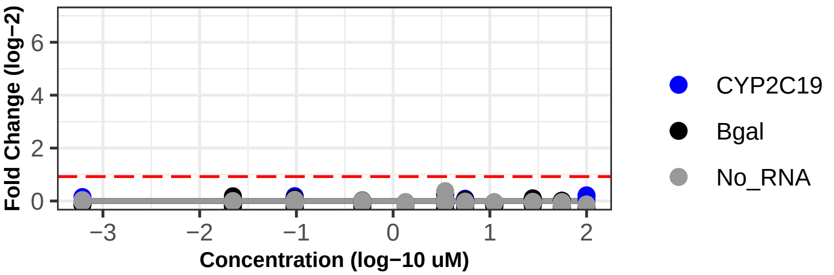


Atrazine: CYP2A6

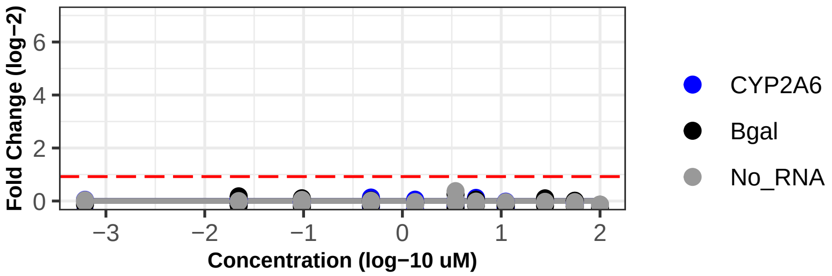


Atrazine: CYP2D6

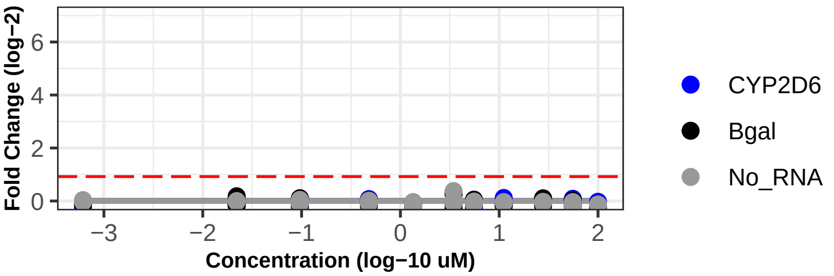


Atrazine: CYP2B6

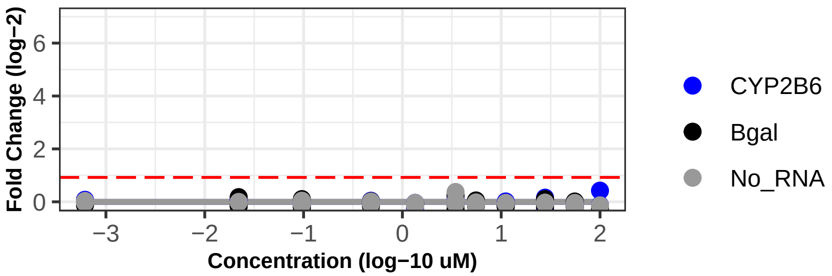


Atrazine: CYP2E1

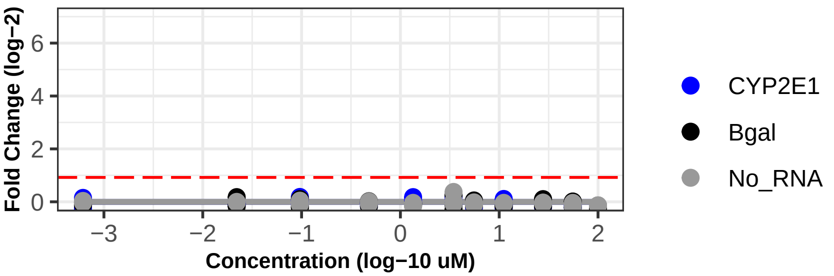


Atrazine: CYP2C8

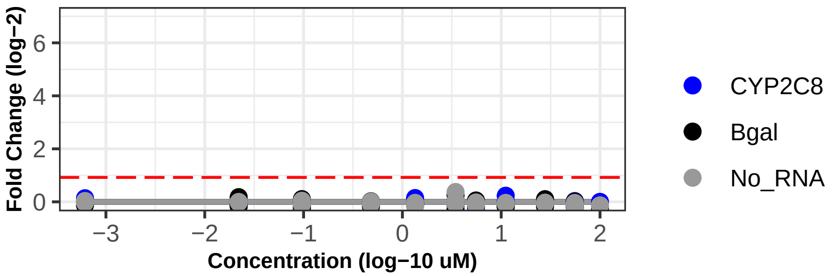


Atrazine: CYP2J2

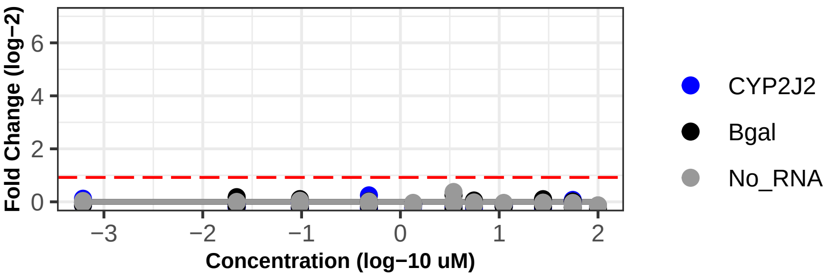


Atrazine: CYP2C9

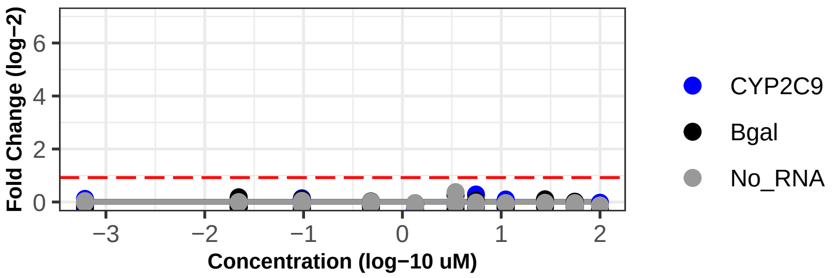


Atrazine: CYP3A4

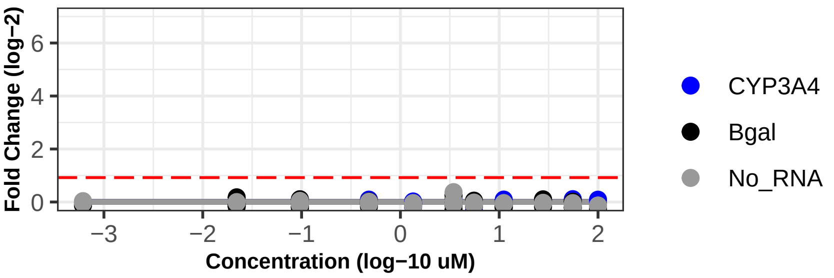

## Benfluralin: CYP1A2

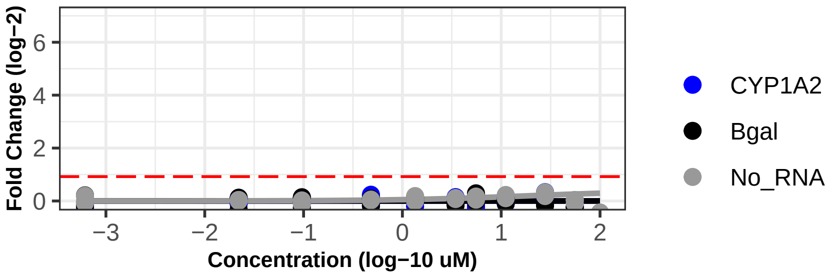


## Benfluralin: CYP2C19

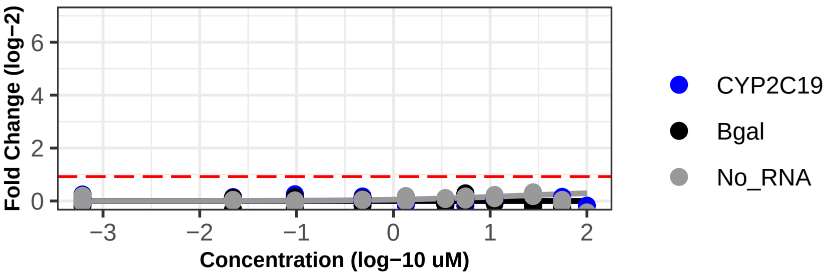


## Benfluralin: CYP2A6

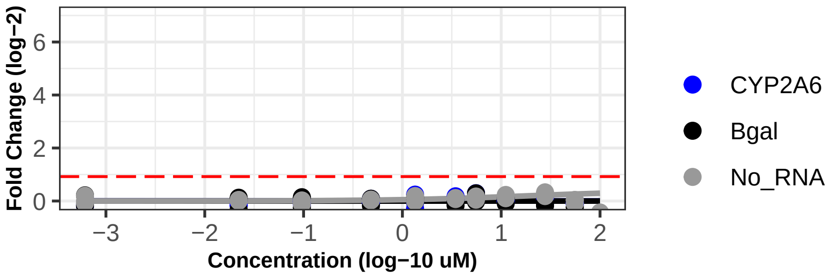


## Benfluralin: CYP2D6

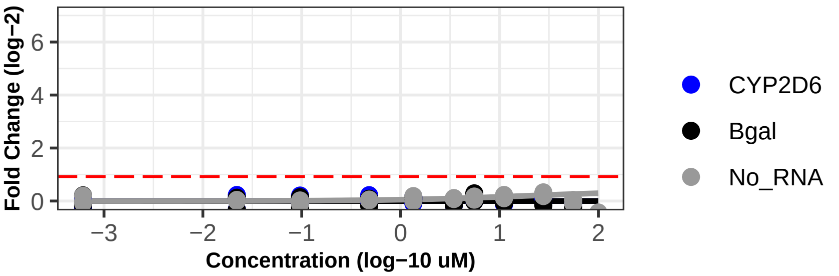


## Benfluralin: CYP2B6

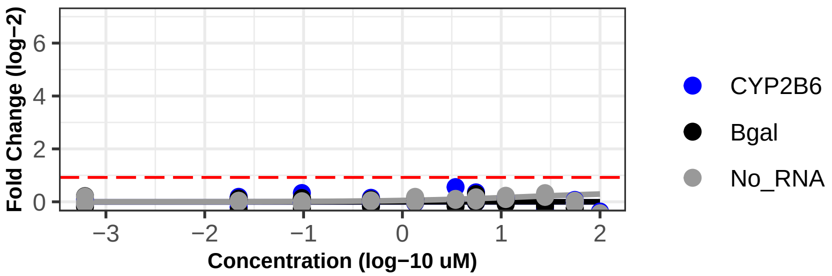


## Benfluralin: CYP2E1

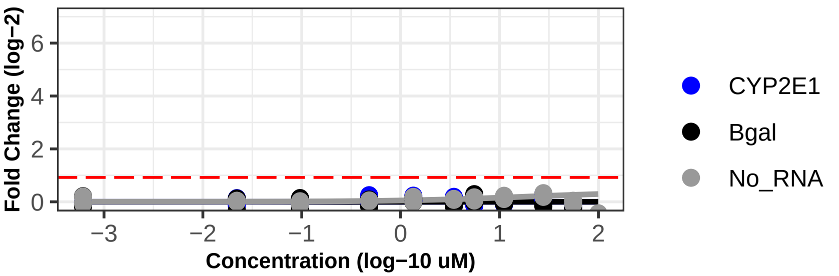


## Benfluralin: CYP2C8

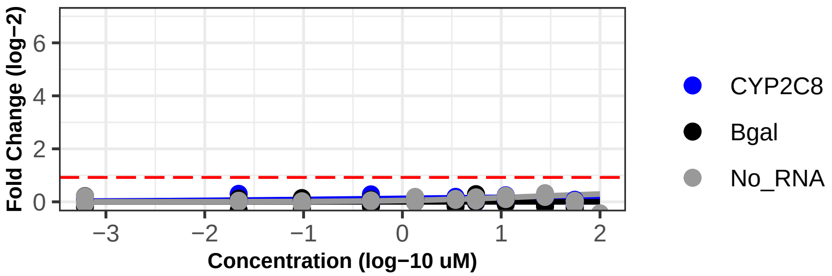


## Benfluralin: CYP2J2

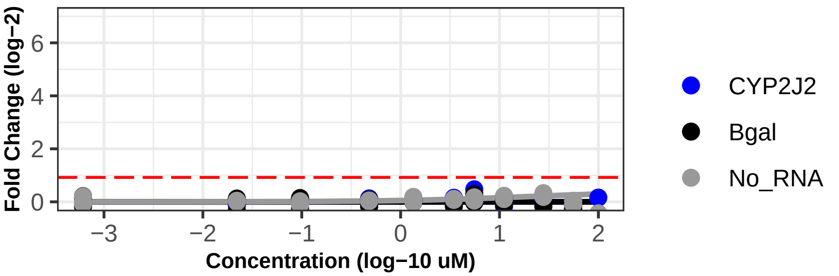


## Benfluralin: CYP2C9

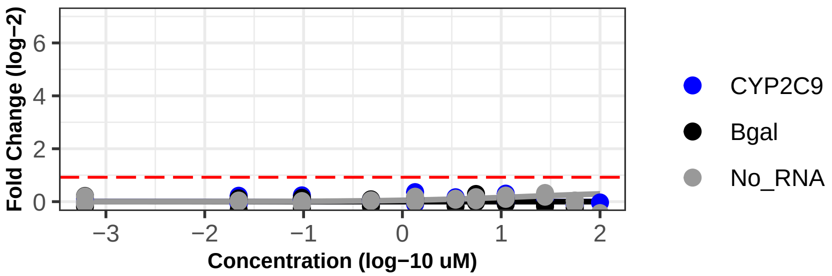


## Benfluralin: CYP3A4

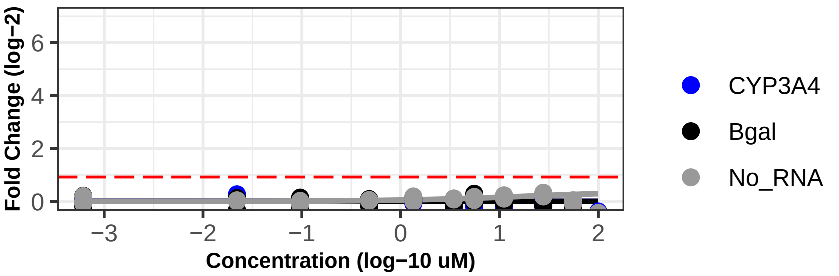

## Benomyl: CYP1A2

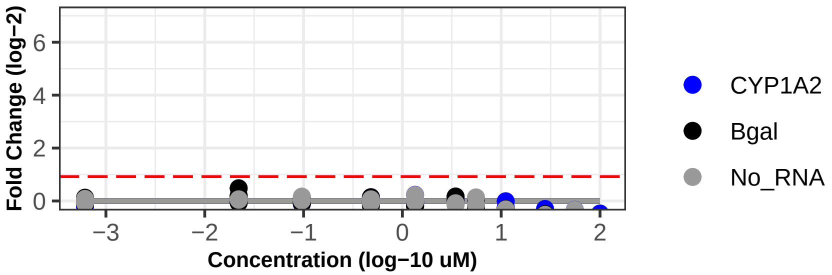


## Benomyl: CYP2C19

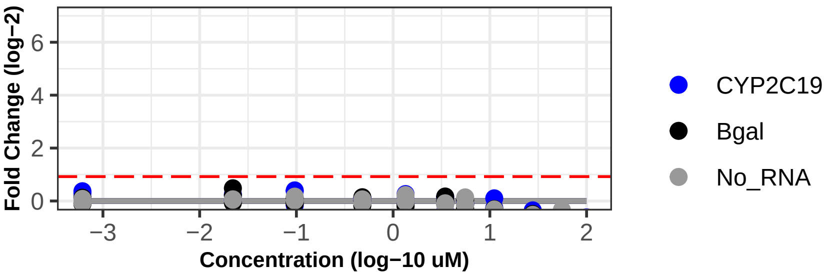


## Benomyl: CYP2A6

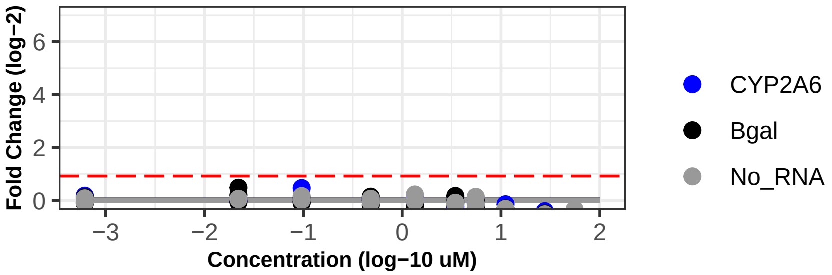


## Benomyl: CYP2D6

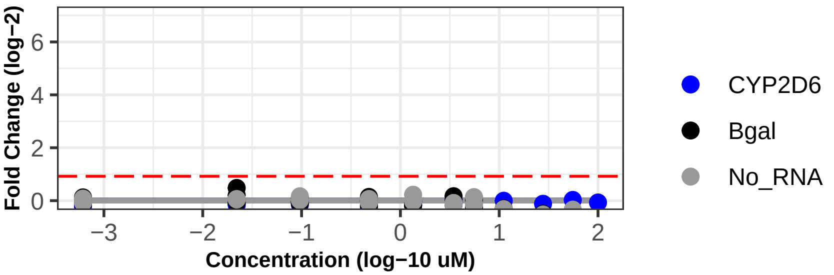


## Benomyl: CYP2B6

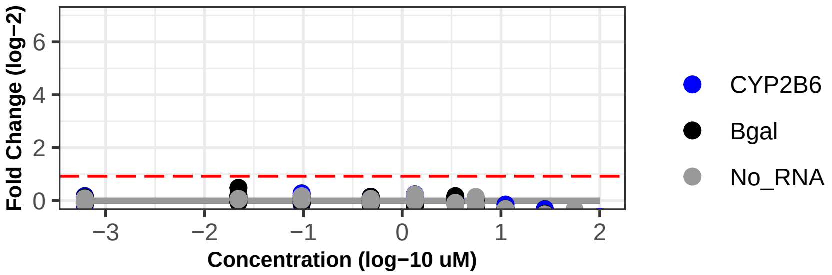


## Benomyl: CYP2E1

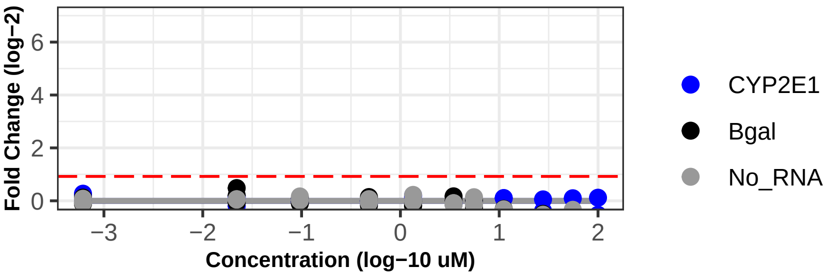


## Benomyl: CYP2C8

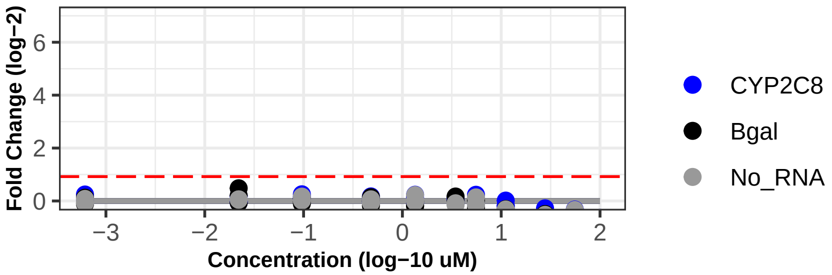


## Benomyl: CYP2J2

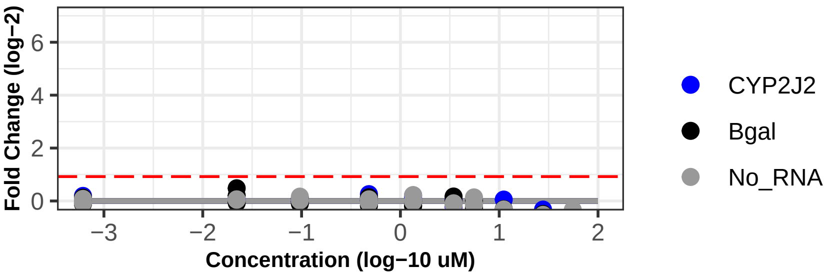


## Benomyl: CYP2C9

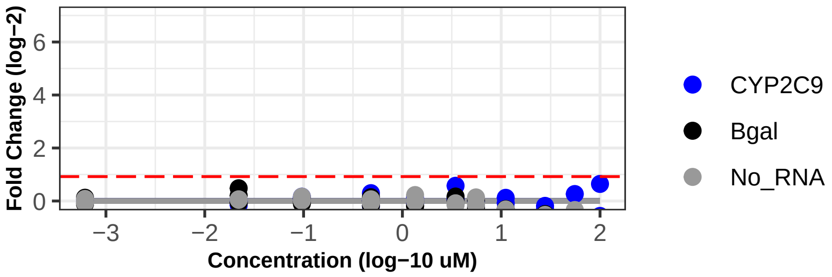


## Benomyl: CYP3A4

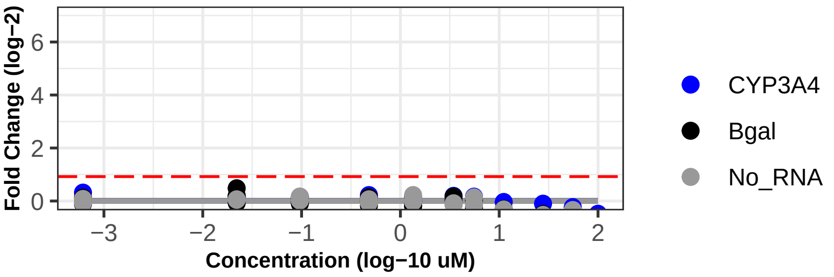

## Bicalutamide: CYP1A2

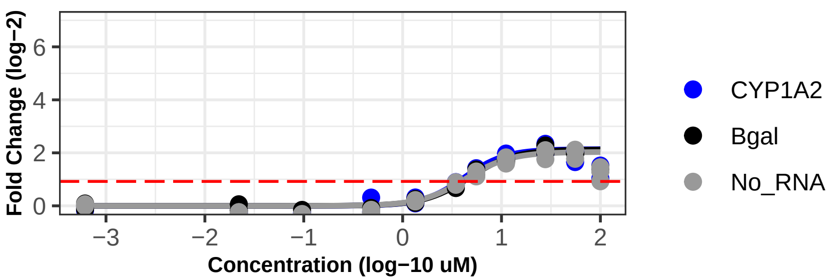


## Bicalutamide: CYP2C19

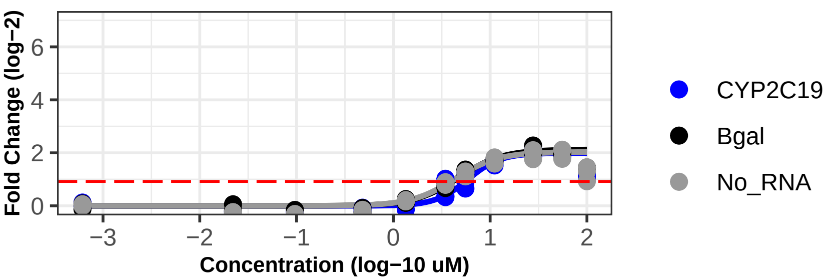


## Bicalutamide: CYP2A6

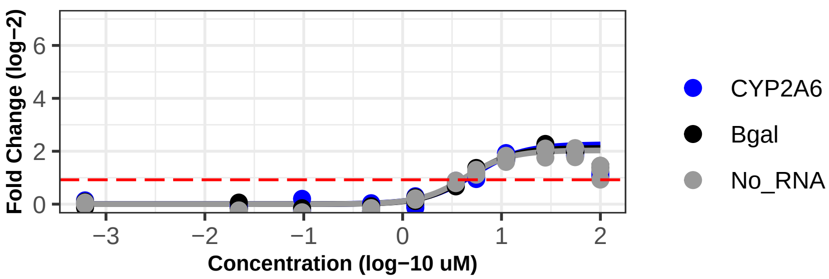


## Bicalutamide: CYP2D6

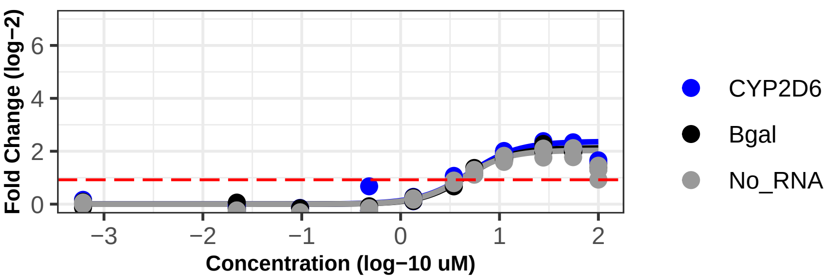


## Bicalutamide: CYP2B6

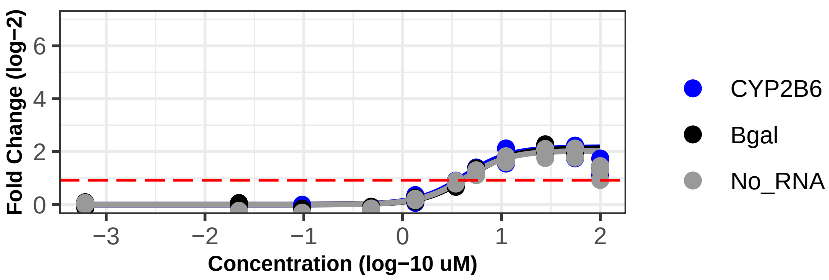


## Bicalutamide: CYP2E1

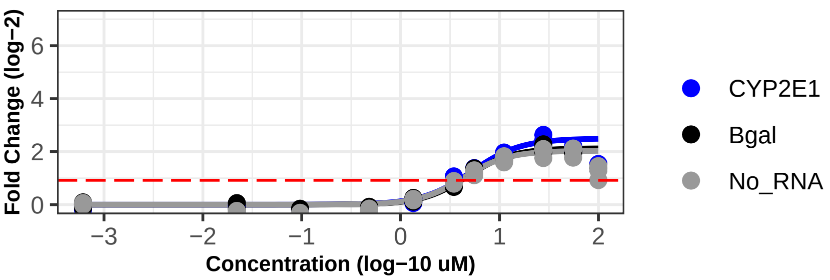


## Bicalutamide: CYP2C8

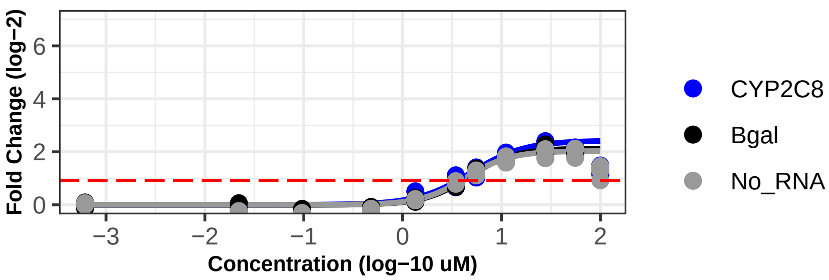


## Bicalutamide: CYP2J2

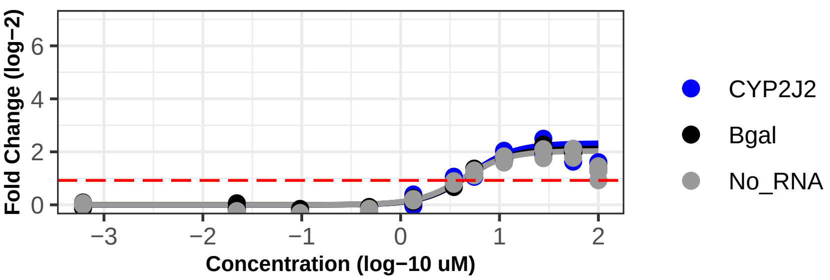


## Bicalutamide: CYP2C9

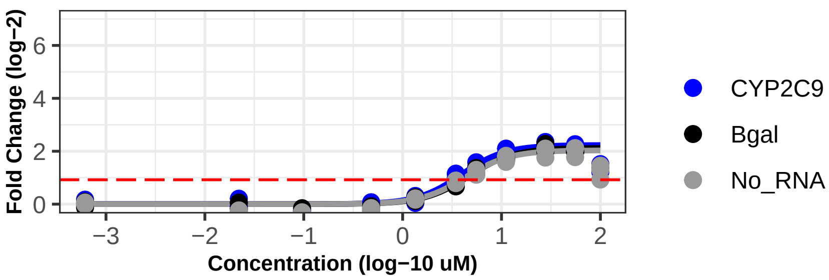


## Bicalutamide: CYP3A4

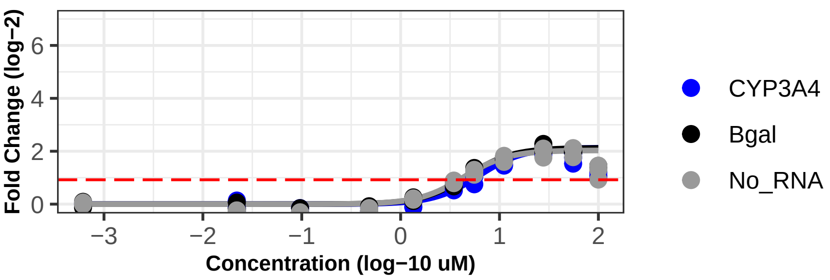

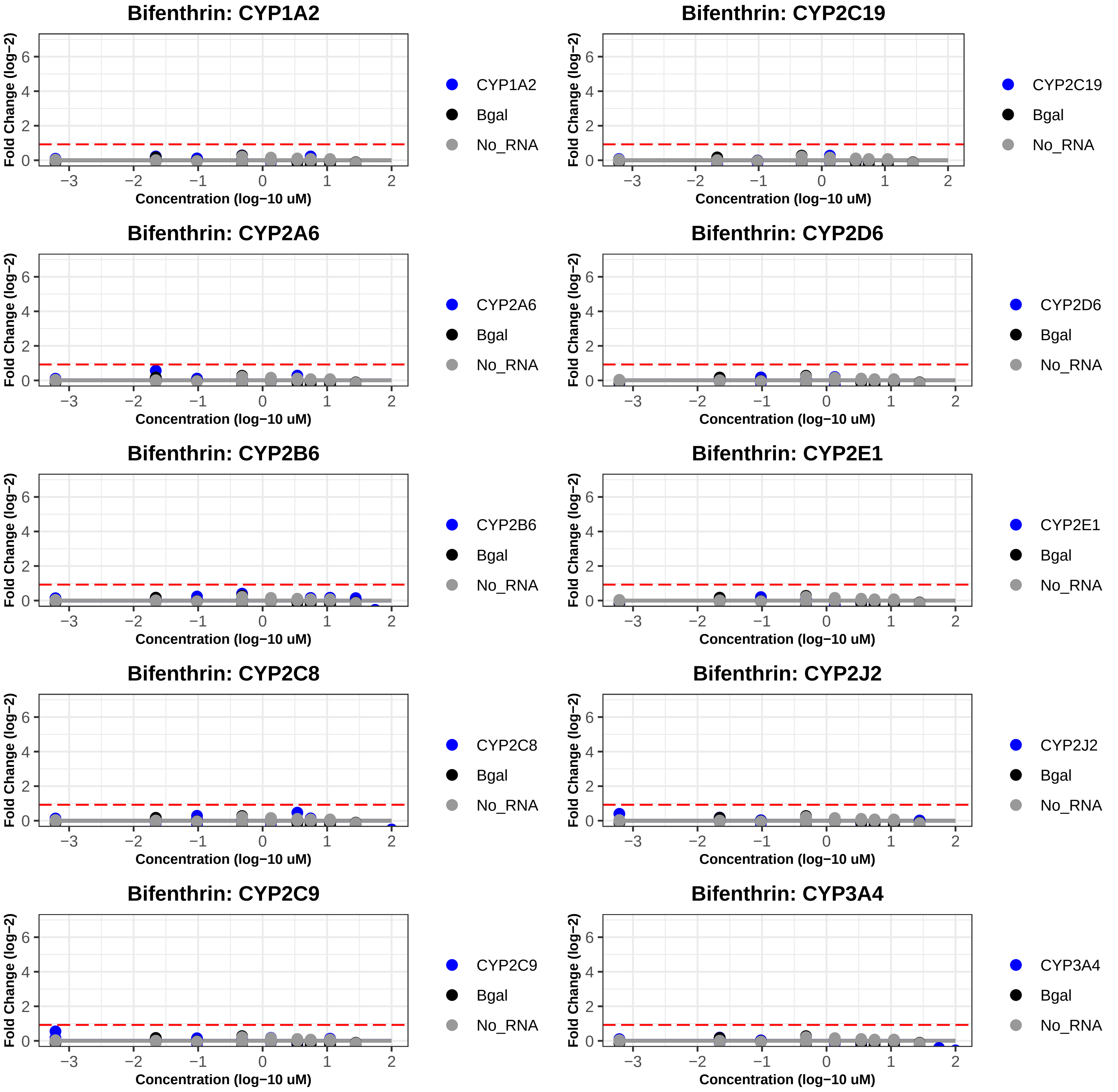

Bifenthrin: CYP1A2
CYP1A2
Bgal
No_RNA
Bifenthrin: CYP2C19
CYP2C19
Bgal
No_RNA
Bifenthrin: CYP2A6
CYP2A6
Bgal
No_RNA
Bifenthrin: CYP2D6
CYP2D6
Bgal
No_RNA
Bifenthrin: CYP2B6
CYP2B6
Bgal
No_RNA
Bifenthrin: CYP2E1
CYP2E1
Bgal
No_RNA
Bifenthrin: CYP2C8
CYP2C8
Bgal
No_RNA
Bifenthrin: CYP2J2
CYP2J2
Bgal
No_RNA
Bifenthrin: CYP2C9
CYP2C9
Bgal
No_RNA
Bifenthrin: CYP3A4
CYP3A4
Bgal
No_RNA
Fold Change (log−2)
Concentration (log−10 uM)

### Bis(2−ethylhexyl)hexanedioate: CYP1A2

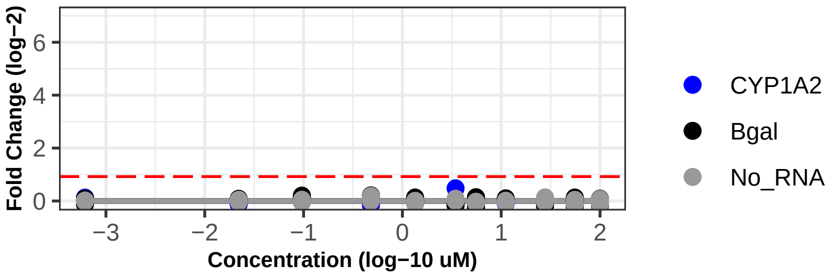


### Bis(2−ethylhexyl)hexanedioate: CYP2C19

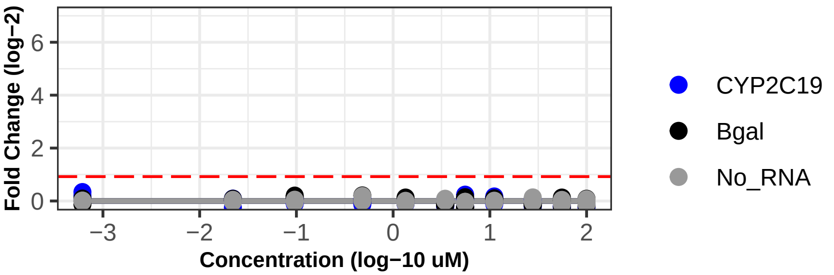


### Bis(2−ethylhexyl)hexanedioate: CYP2A6

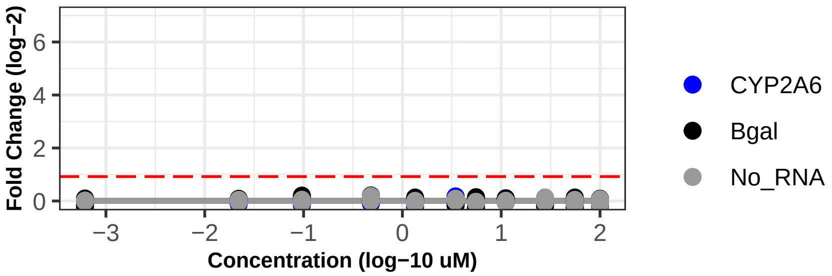


### Bis(2−ethylhexyl)hexanedioate: CYP2D6

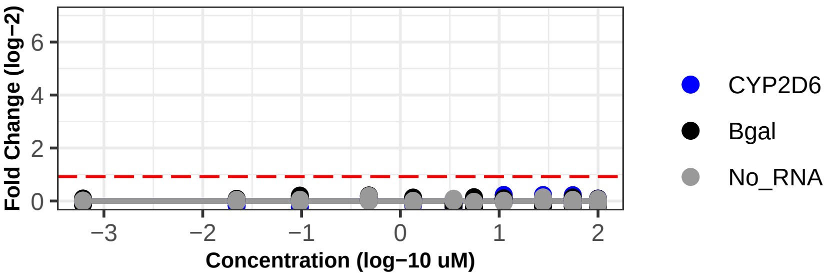


### Bis(2−ethylhexyl)hexanedioate: CYP2B6

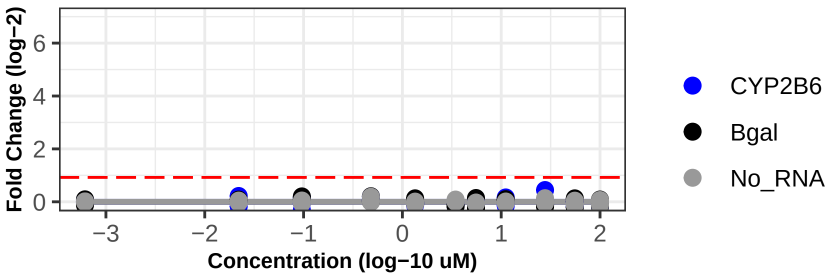


### Bis(2−ethylhexyl)hexanedioate: CYP2E1

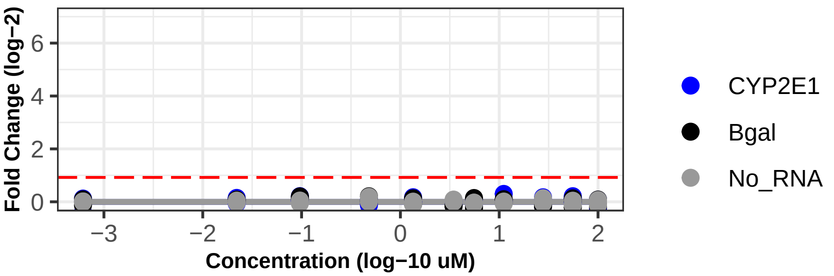


### Bis(2−ethylhexyl)hexanedioate: CYP2C8

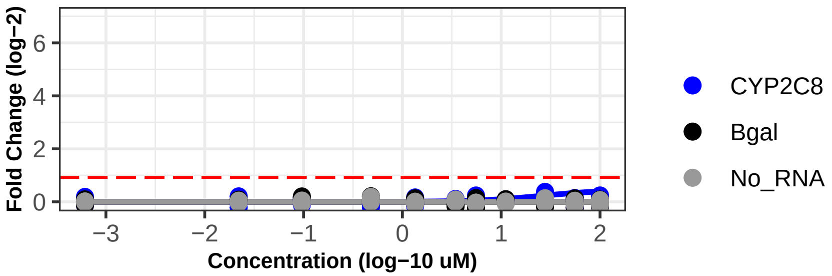


### Bis(2−ethylhexyl)hexanedioate: CYP2J2

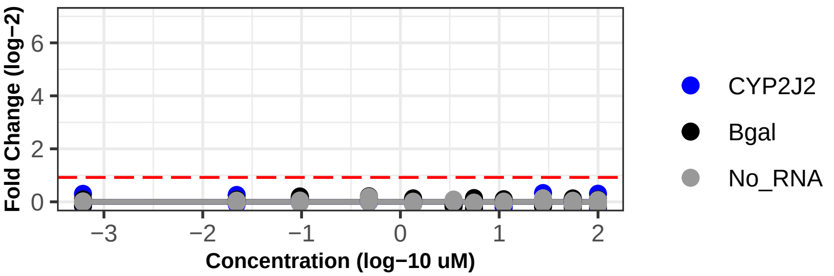


### Bis(2−ethylhexyl)hexanedioate: CYP2C9

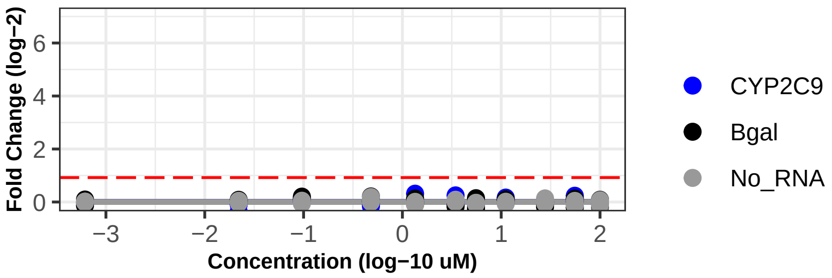


### Bis(2−ethylhexyl)hexanedioate: CYP3A4

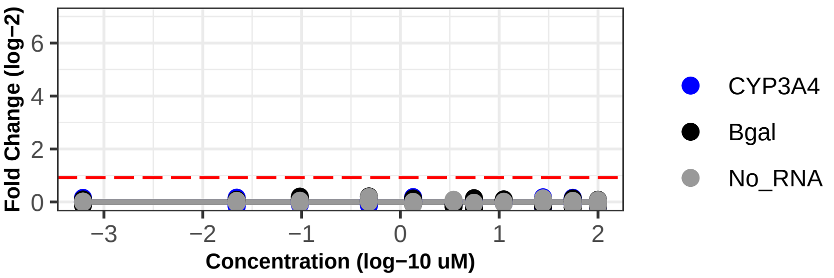

## Bis(2−ethylhexyl)phthalate: CYP1A2

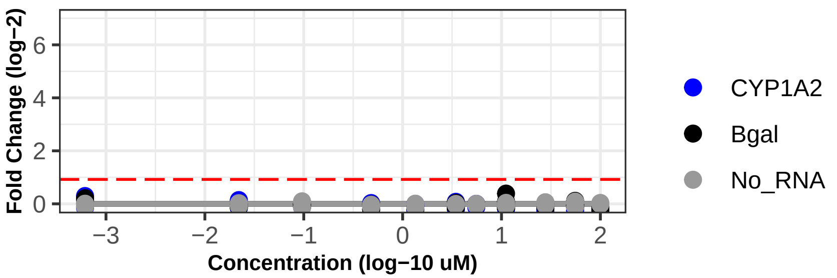


## Bis(2−ethylhexyl)phthalate: CYP2C19

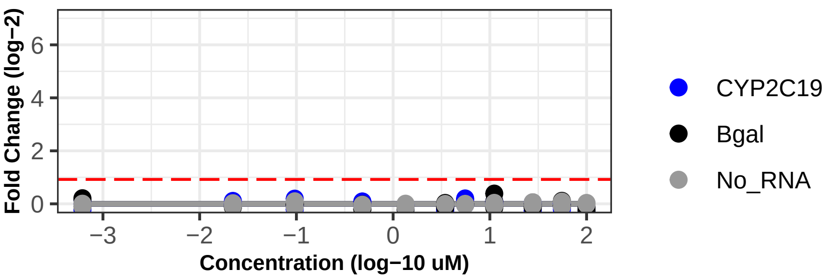


## Bis(2−ethylhexyl)phthalate: CYP2A6

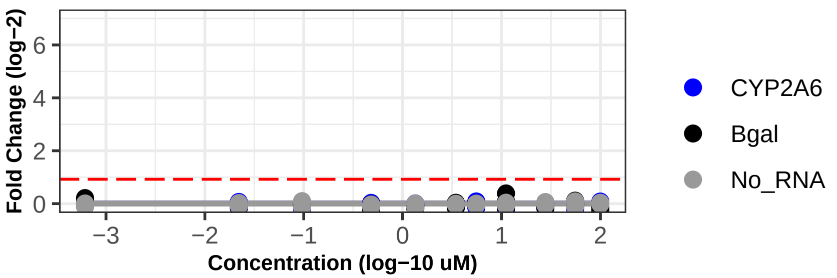


## Bis(2−ethylhexyl)phthalate: CYP2D6

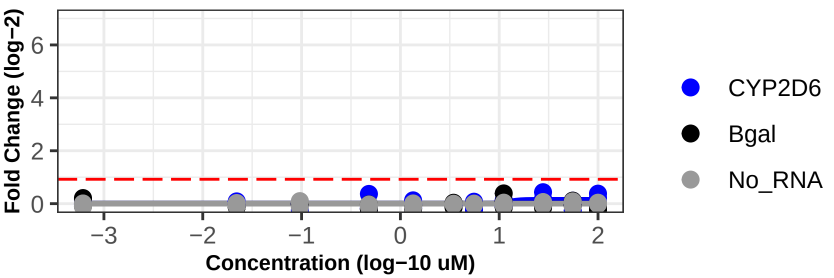


## Bis(2−ethylhexyl)phthalate: CYP2B6

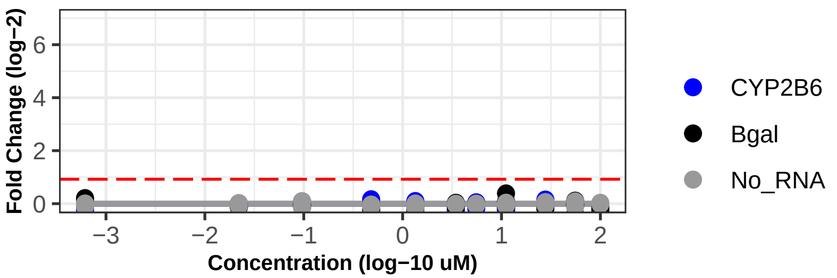


## Bis(2−ethylhexyl)phthalate: CYP2E1

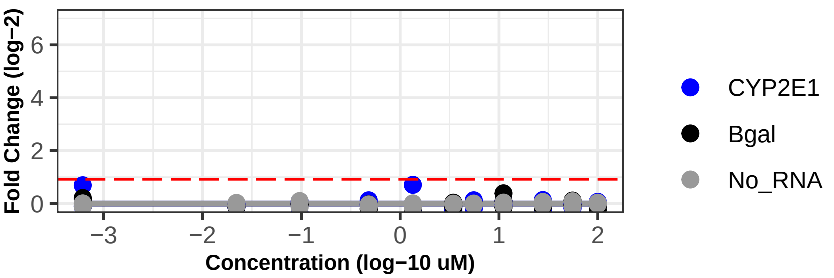


## Bis(2−ethylhexyl)phthalate: CYP2C8

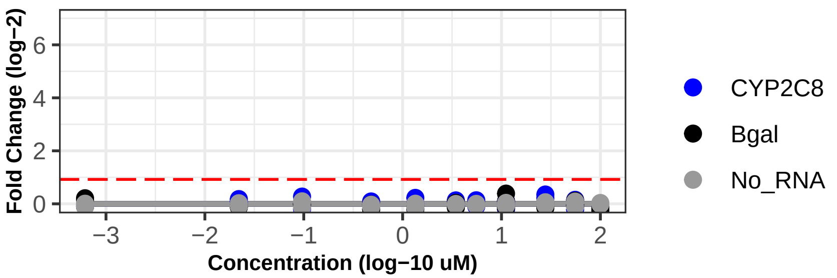


## Bis(2−ethylhexyl)phthalate: CYP2J2

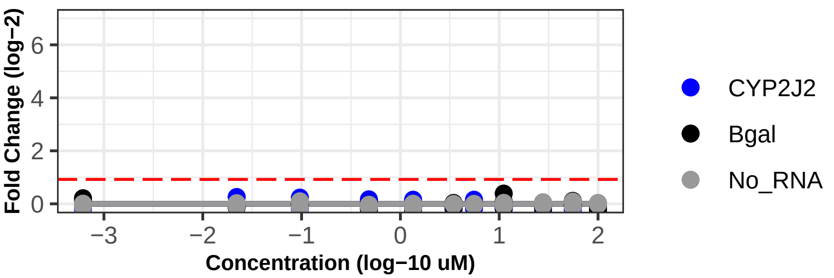


## Bis(2−ethylhexyl)phthalate: CYP2C9

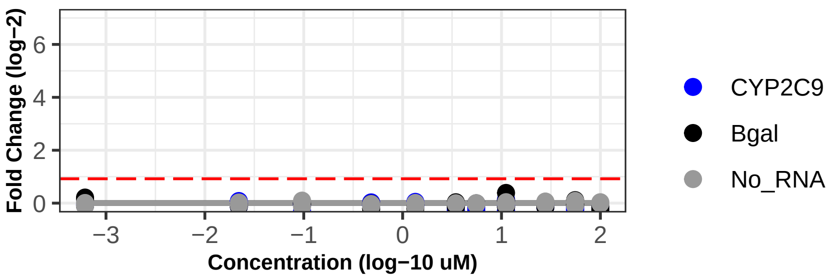


## Bis(2−ethylhexyl)phthalate: CYP3A4

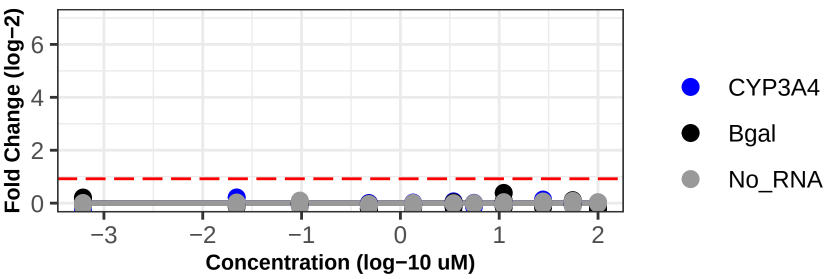

## Bisphenol A: CYP1A2

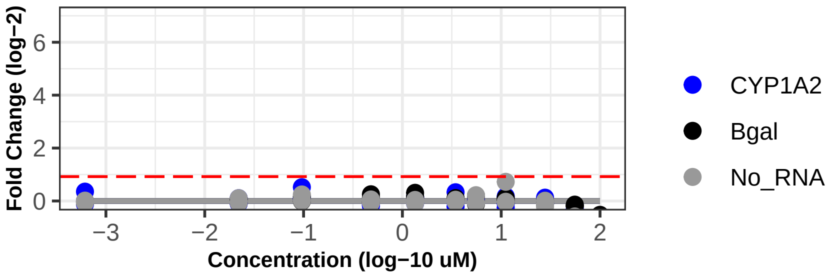


## Bisphenol A: CYP2C19

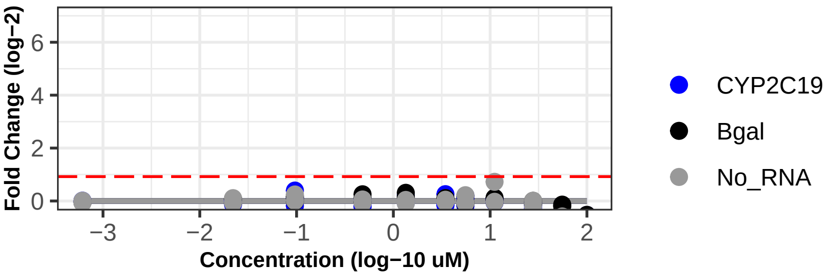


## Bisphenol A: CYP2A6

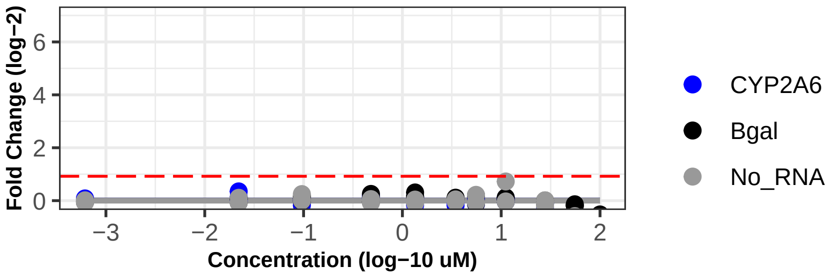


## Bisphenol A: CYP2D6

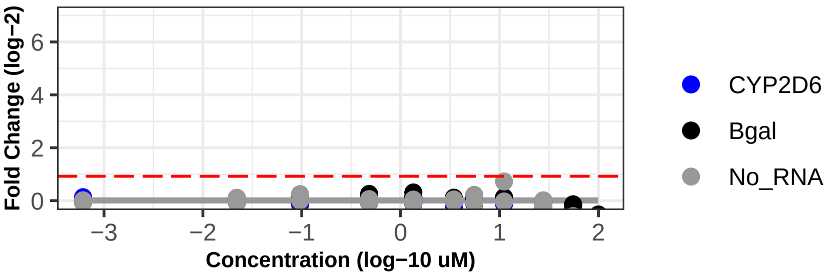


## Bisphenol A: CYP2B6

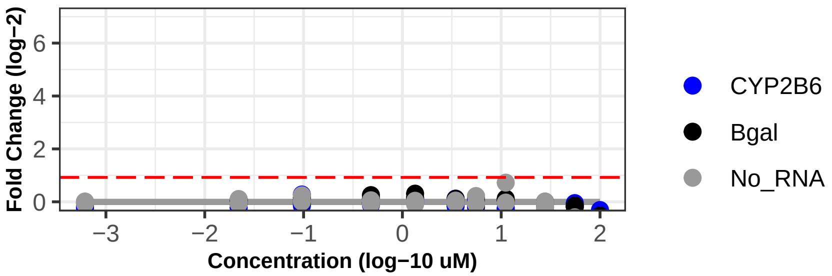


## Bisphenol A: CYP2E1

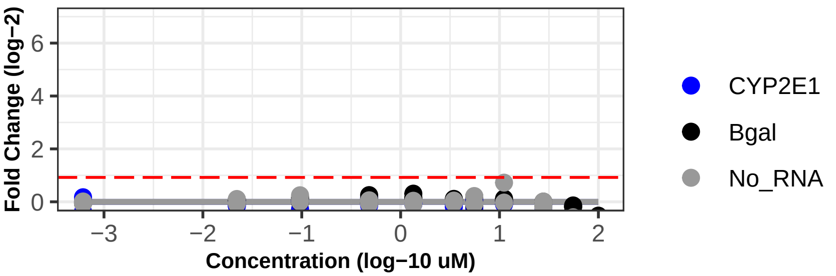


## Bisphenol A: CYP2C8

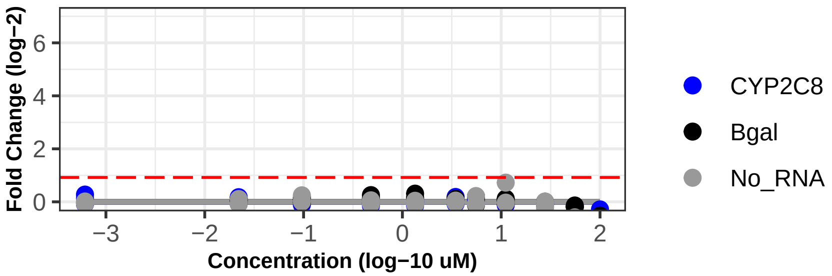


## Bisphenol A: CYP2J2

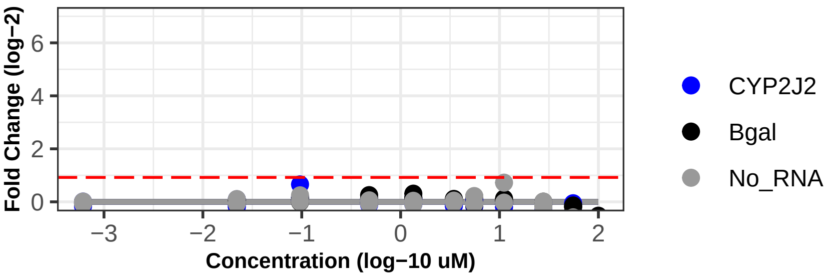


## Bisphenol A: CYP2C9

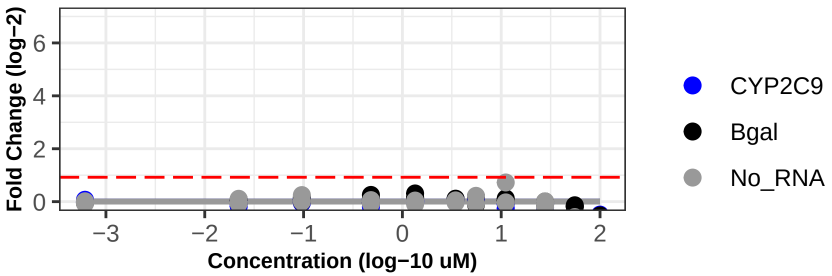


## Bisphenol A: CYP3A4

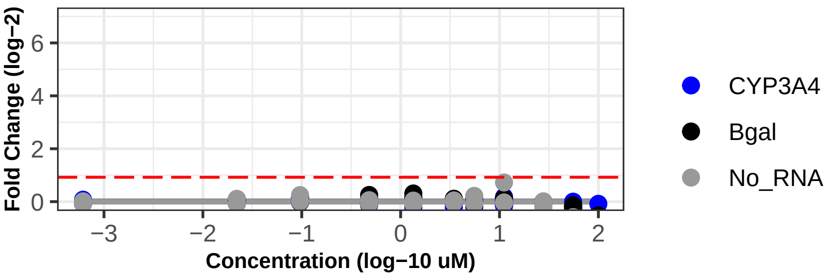

## Bisphenol AF: CYP1A2

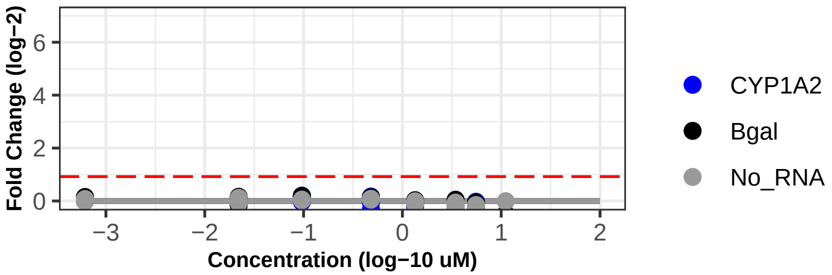


## Bisphenol AF: CYP2C19

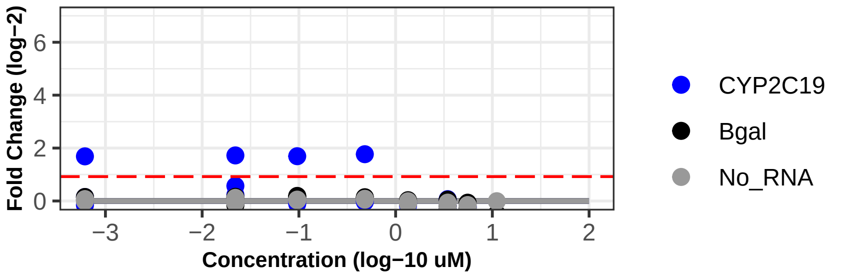


## Bisphenol AF: CYP2A6

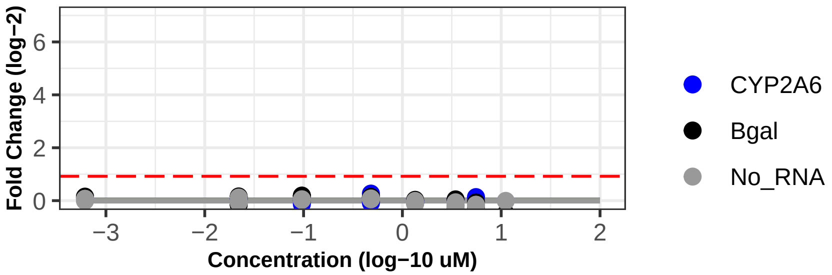


## Bisphenol AF: CYP2D6

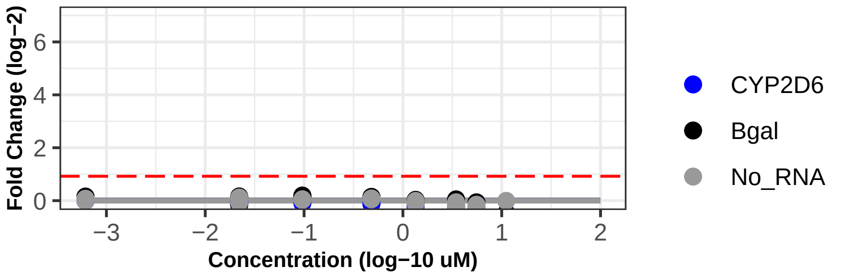


## Bisphenol AF: CYP2B6

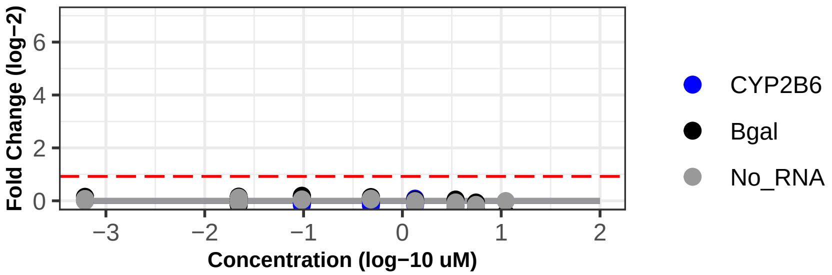


## Bisphenol AF: CYP2E1

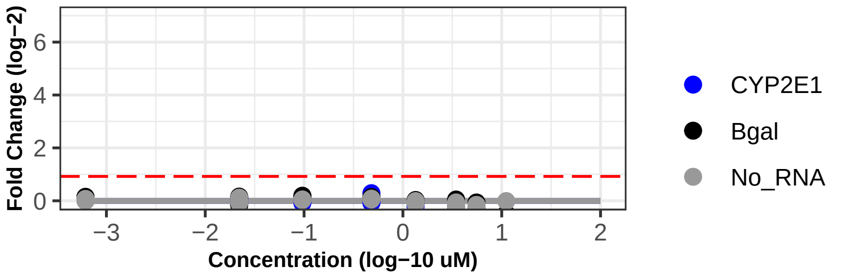


## Bisphenol AF: CYP2C8

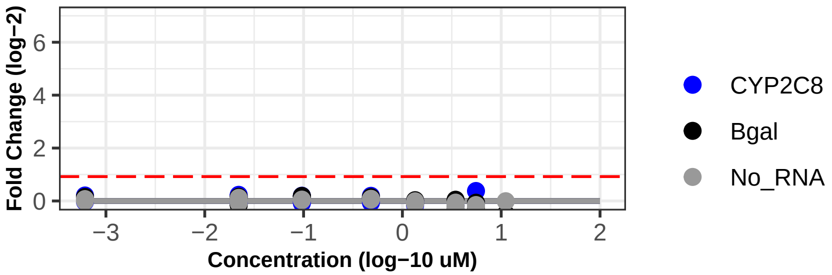


## Bisphenol AF: CYP2J2

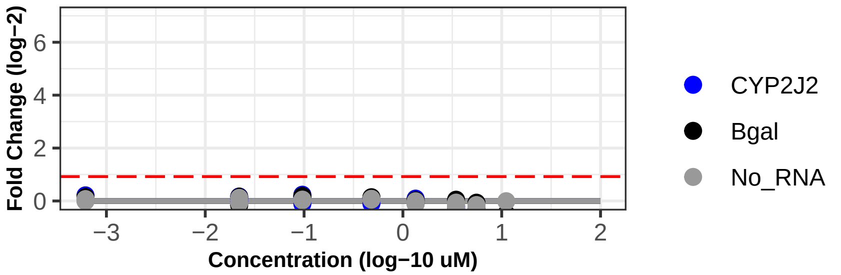


## Bisphenol AF: CYP2C9

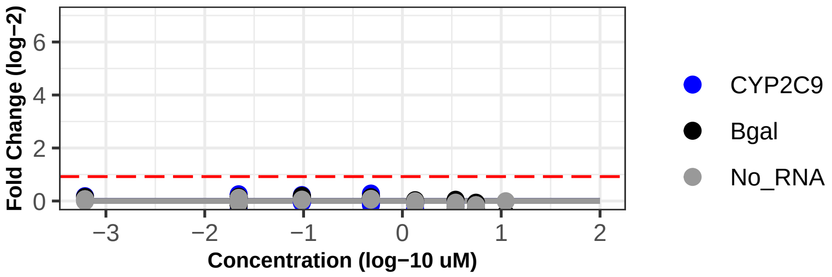


## Bisphenol AF: CYP3A4

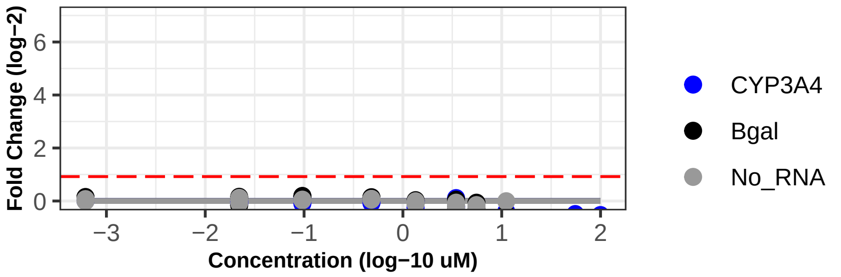

### Bisphenol B: CYP1A2

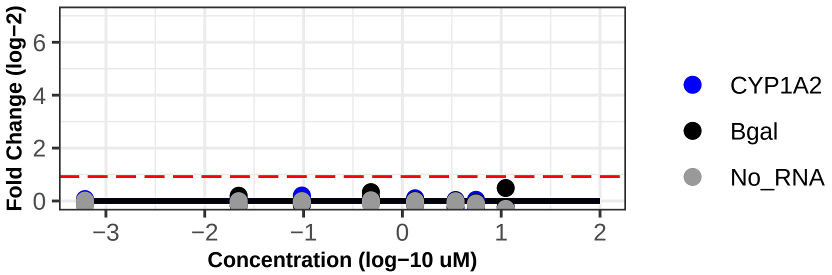


### Bisphenol B: CYP2C19

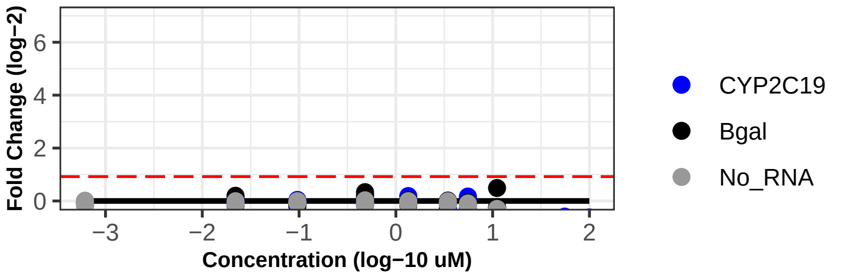


### Bisphenol B: CYP2A6

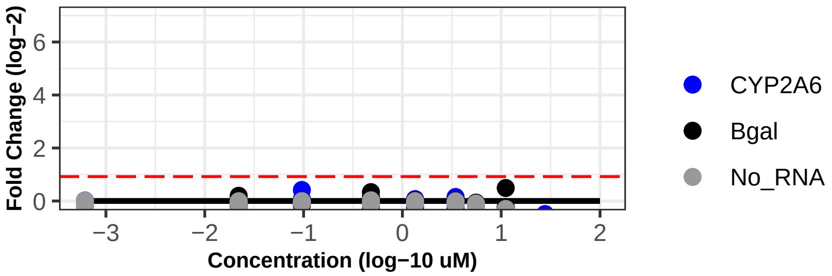


### Bisphenol B: CYP2D6

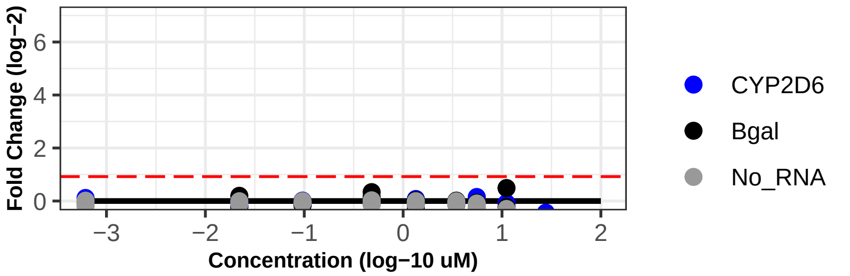


### Bisphenol B: CYP2B6

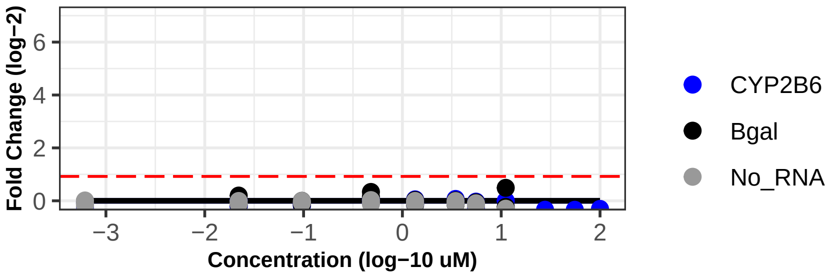


### Bisphenol B: CYP2E1

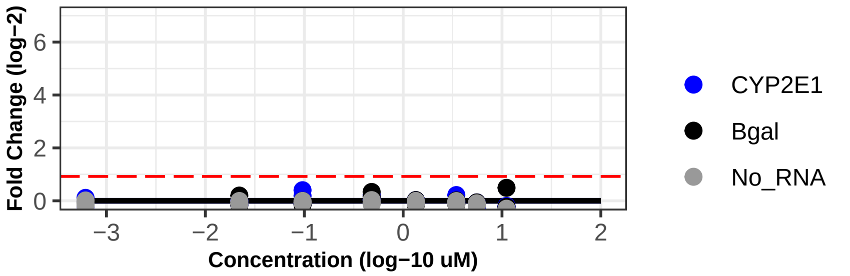


### Bisphenol B: CYP2C8

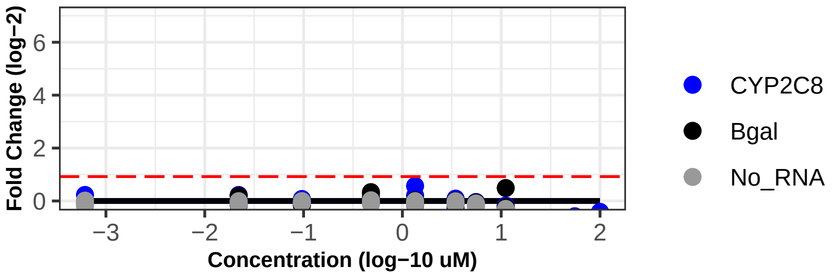


### Bisphenol B: CYP2J2

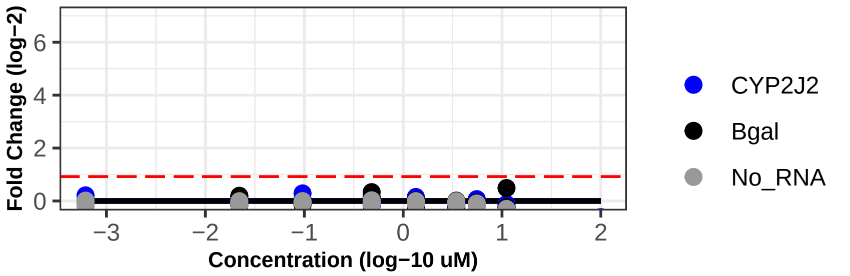


### Bisphenol B: CYP2C9

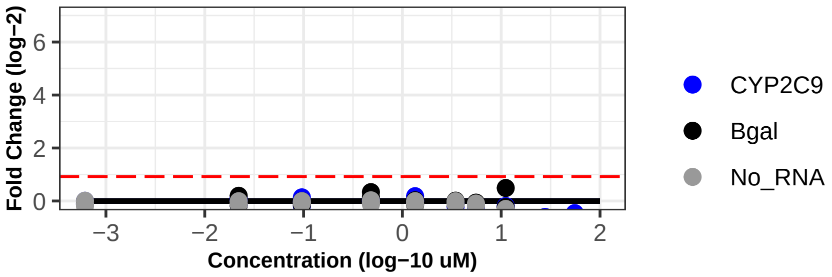


### Bisphenol B: CYP3A4

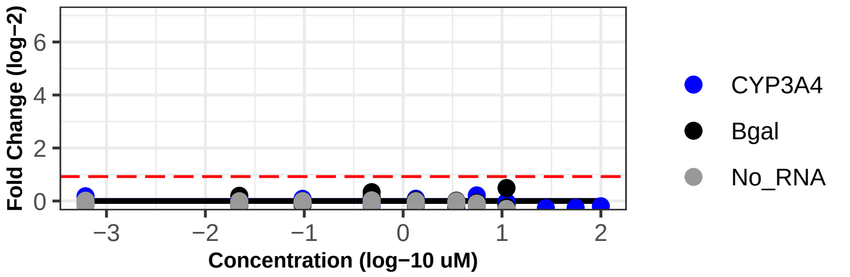

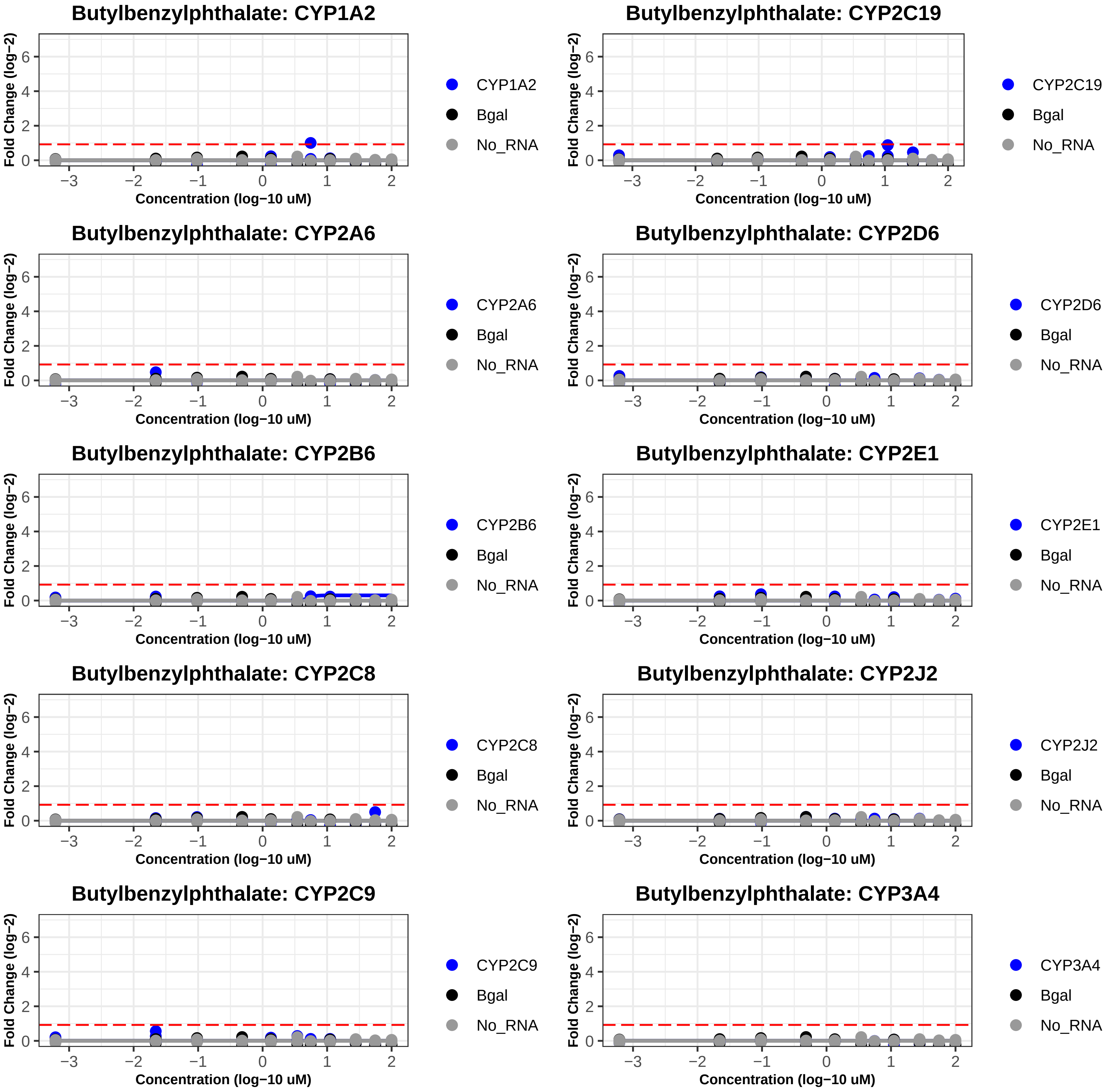
Butylbenzylphthalate: CYP1A2
Fold Change (log−2)
Concentration (log−10 uM)
CYP1A2
Bgal
No_RNA
Butylbenzylphthalate: CYP2C19
Fold Change (log−2)
Concentration (log−10 uM)
CYP2C19
Bgal
No_RNA
Butylbenzylphthalate: CYP2A6
Fold Change (log−2)
Concentration (log−10 uM)
CYP2A6
Bgal
No_RNA
Butylbenzylphthalate: CYP2D6
Fold Change (log−2)
Concentration (log−10 uM)
CYP2D6
Bgal
No_RNA
Butylbenzylphthalate: CYP2B6
Fold Change (log−2)
Concentration (log−10 uM)
CYP2B6
Bgal
No_RNA
Butylbenzylphthalate: CYP2E1
Fold Change (log−2)
Concentration (log−10 uM)
CYP2E1
Bgal
No_RNA
Butylbenzylphthalate: CYP2C8
Fold Change (log−2)
Concentration (log−10 uM)
CYP2C8
Bgal
No_RNA
Butylbenzylphthalate: CYP2J2
Fold Change (log−2)
Concentration (log−10 uM)
CYP2J2
Bgal
No_RNA
Butylbenzylphthalate: CYP2C9
Fold Change (log−2)
Concentration (log−10 uM)
CYP2C9
Bgal
No_RNA
Butylbenzylphthalate: CYP3A4
Fold Change (log−2)
Concentration (log−10 uM)
CYP3A4
Bgal
No_RNA

## Butylparaben: CYP1A2

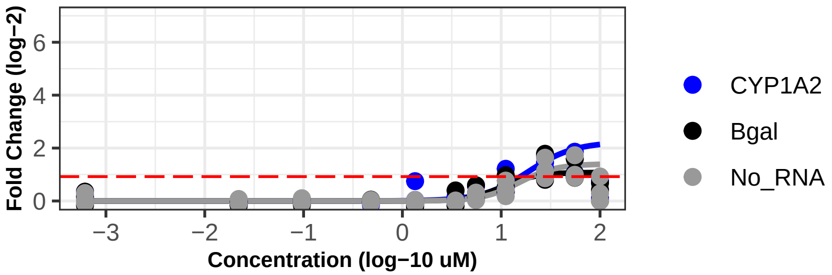


## Butylparaben: CYP2C19

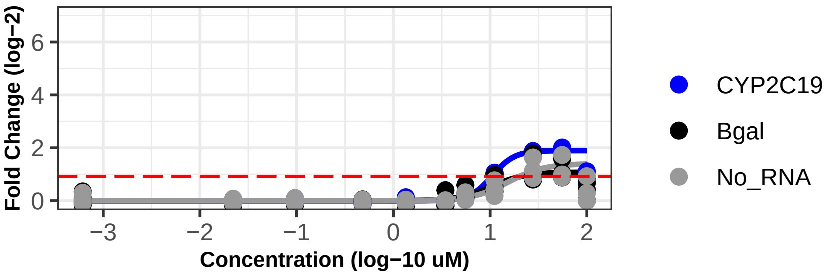


## Butylparaben: CYP2A6

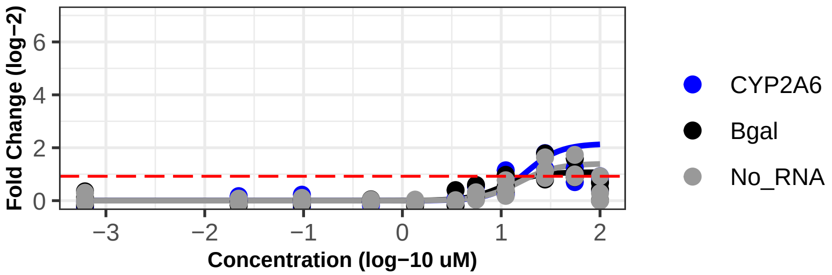


## Butylparaben: CYP2D6

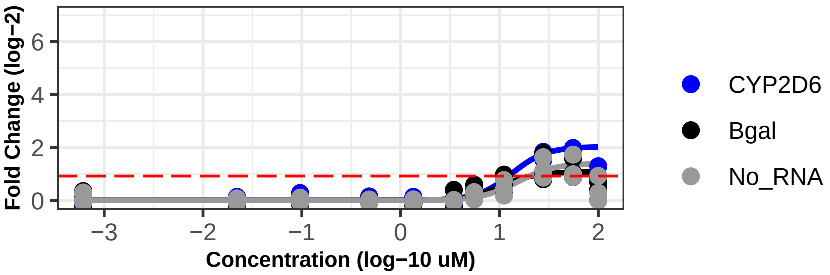


## Butylparaben: CYP2B6

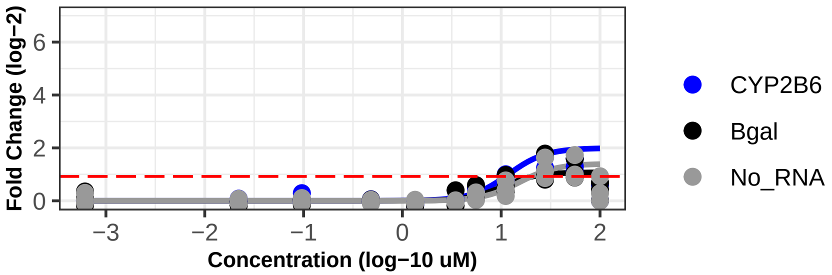


## Butylparaben: CYP2E1

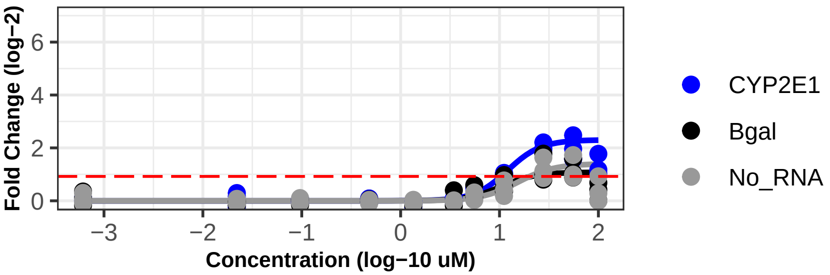


## Butylparaben: CYP2C8

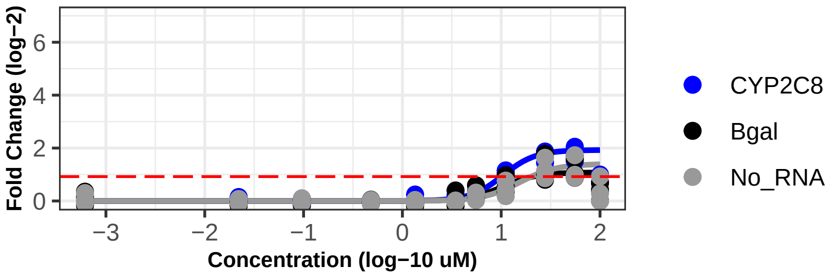


## Butylparaben: CYP2J2

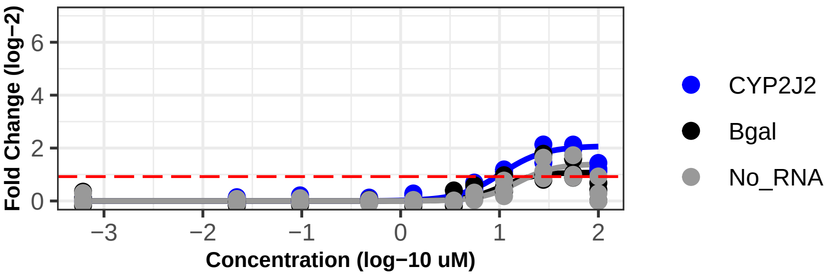


## Butylparaben: CYP2C9

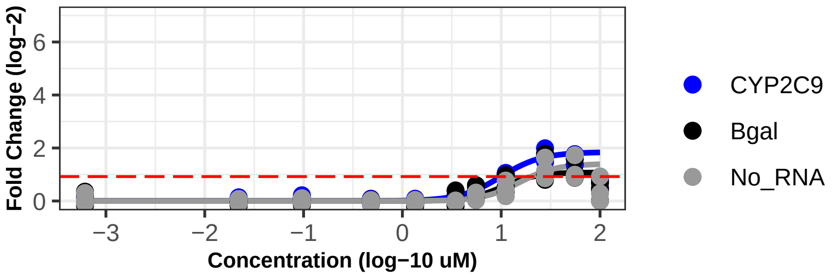


## Butylparaben: CYP3A4

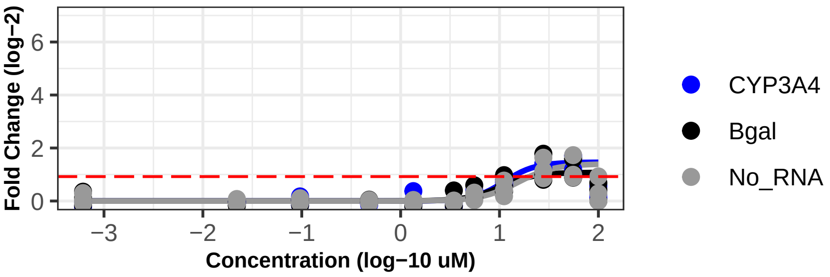

## Carbendazim: CYP1A2

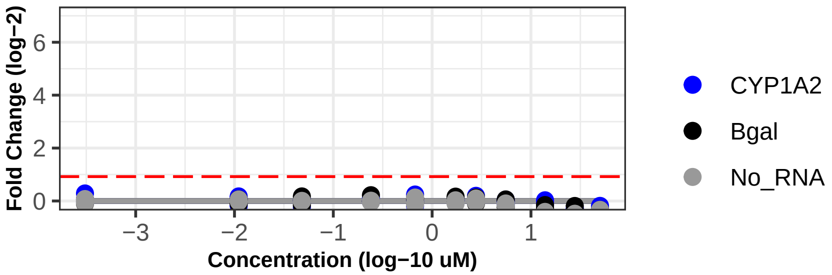


## Carbendazim: CYP2C19

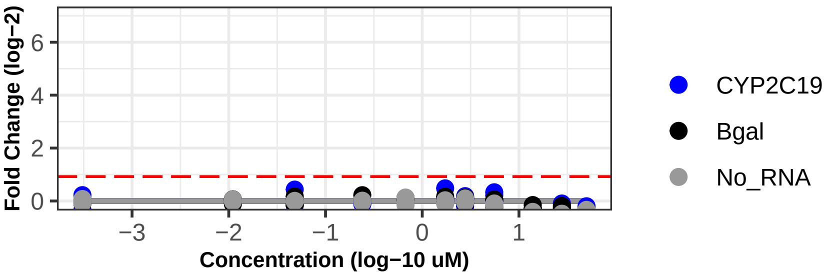


## Carbendazim: CYP2A6

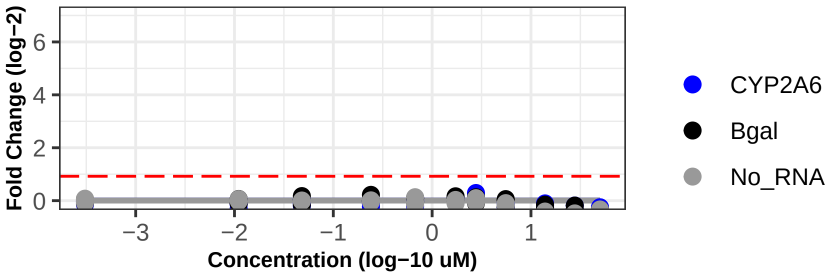


## Carbendazim: CYP2D6

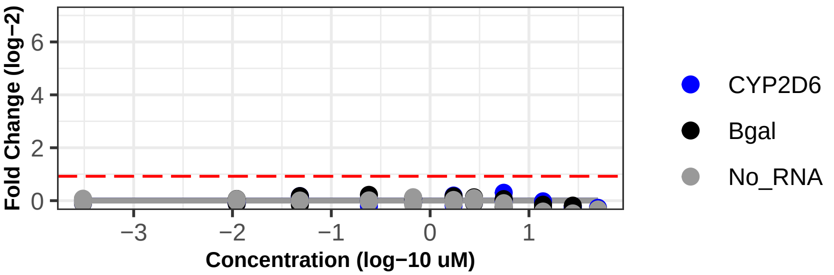


## Carbendazim: CYP2B6

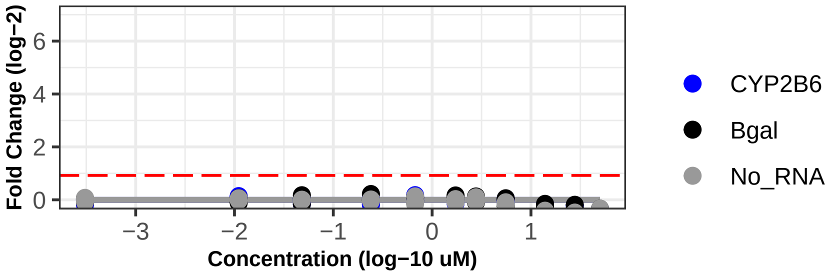


## Carbendazim: CYP2E1

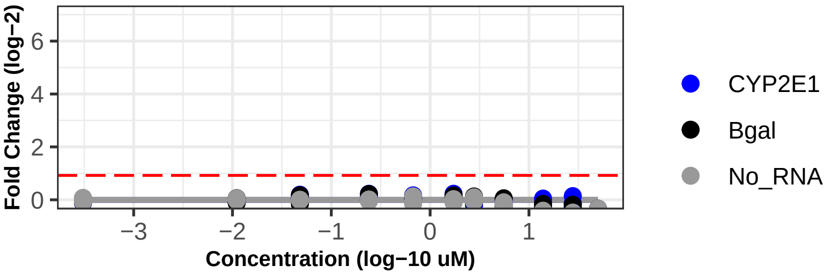


## Carbendazim: CYP2C8

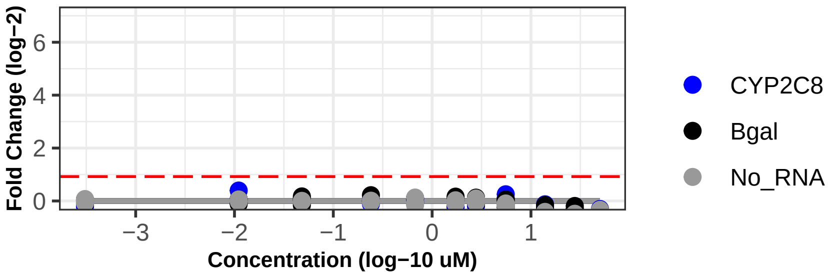


## Carbendazim: CYP2J2

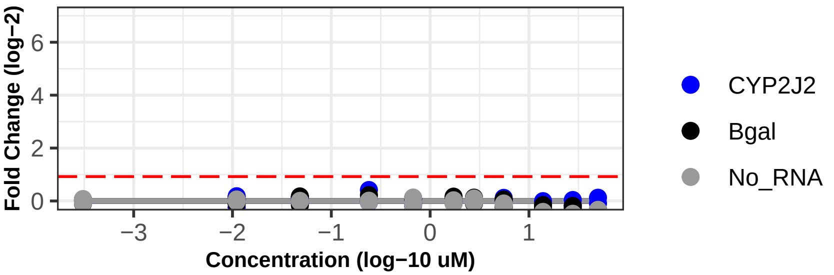


## Carbendazim: CYP2C9

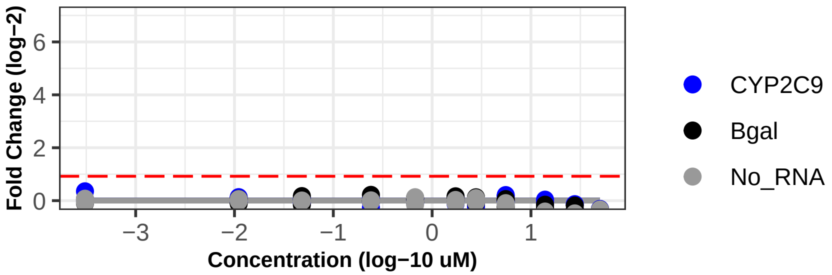


## Carbendazim: CYP3A4

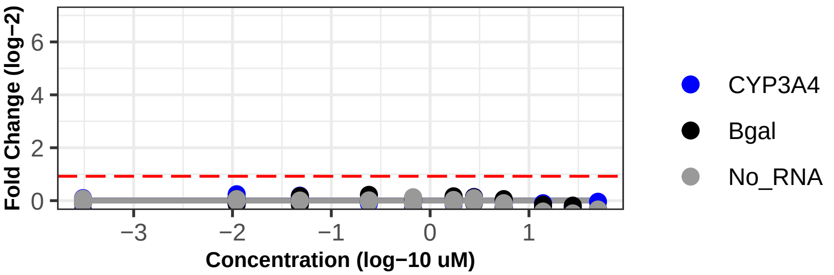

### Carbofuran: CYP1A2

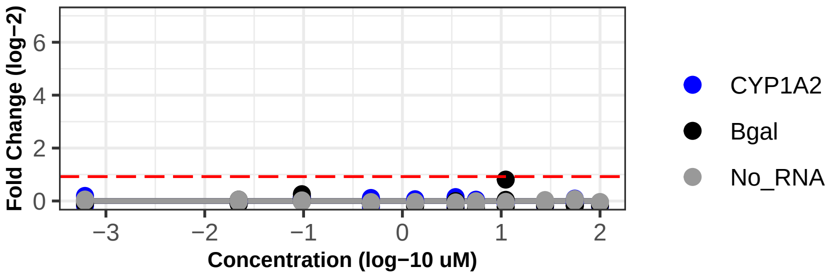


### Carbofuran: CYP2C19

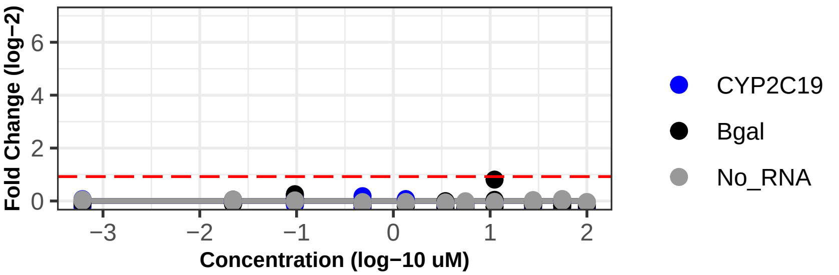


### Carbofuran: CYP2A6

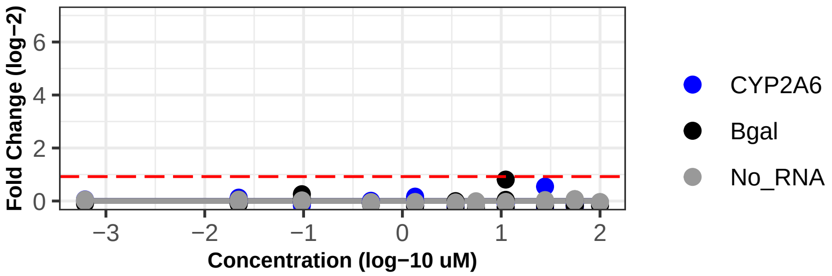


### Carbofuran: CYP2D6

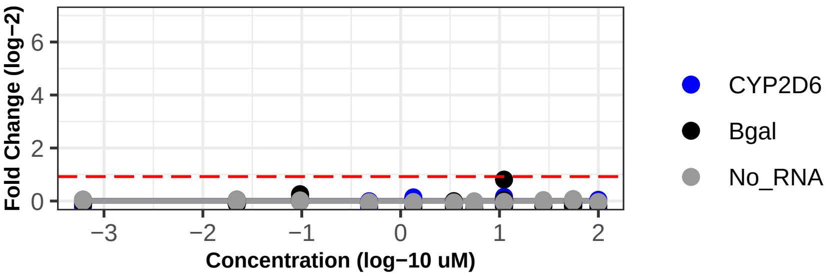


### Carbofuran: CYP2B6

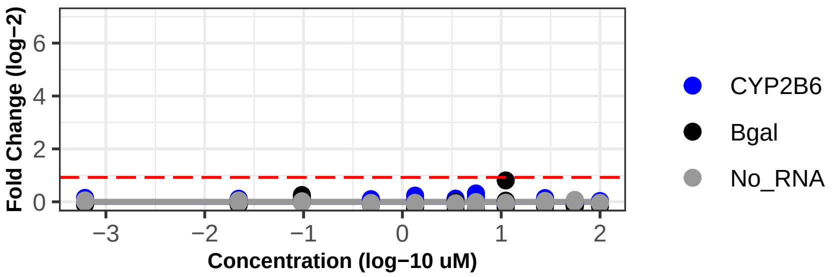


### Carbofuran: CYP2E1

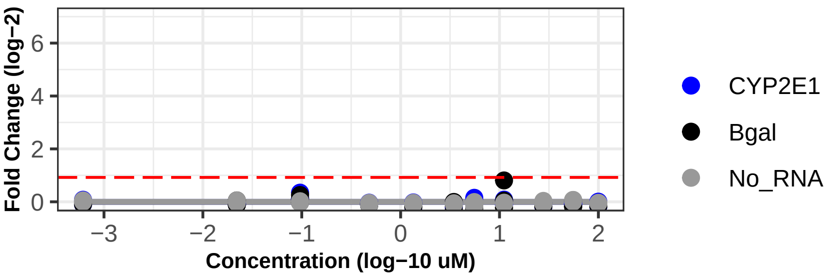


### Carbofuran: CYP2C8

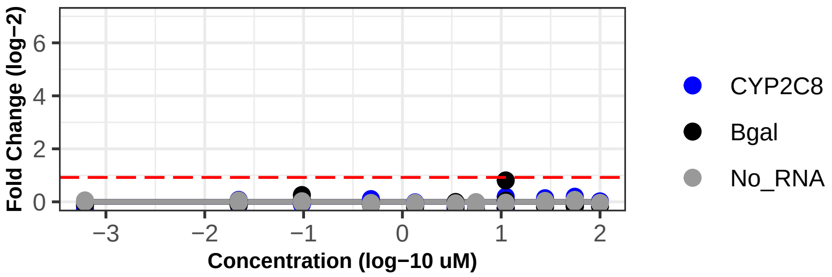


### Carbofuran: CYP2J2

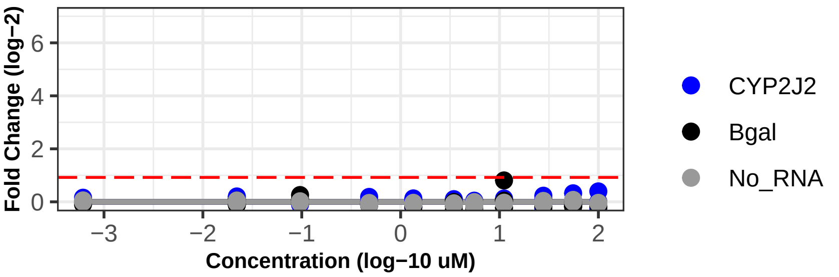


### Carbofuran: CYP2C9

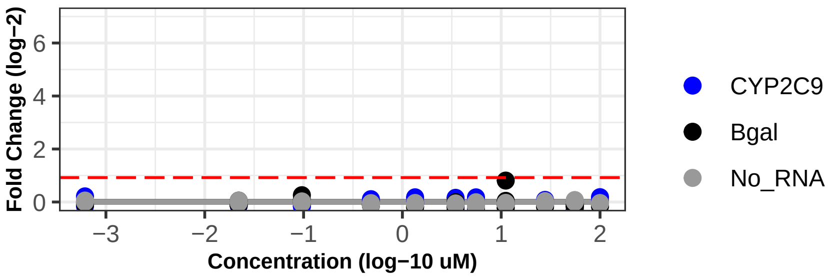


### Carbofuran: CYP3A4

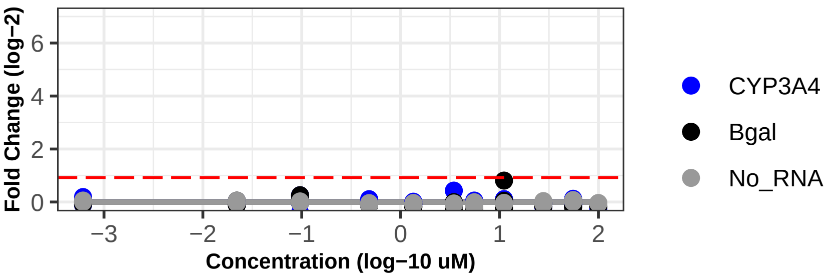

## Chlorothalonil: CYP1A2

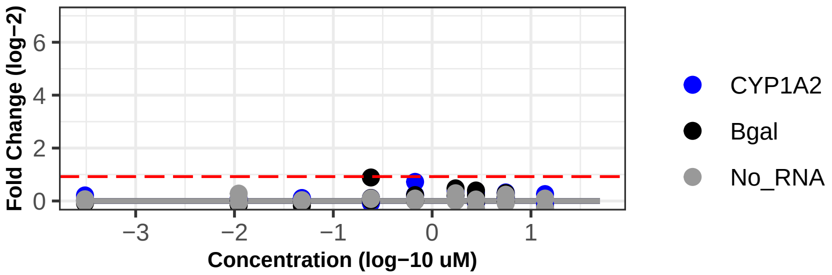


## Chlorothalonil: CYP2C19

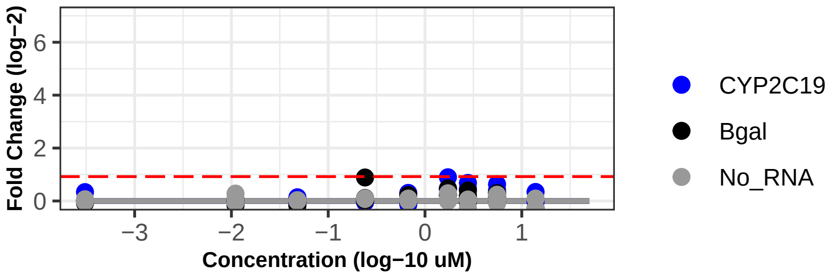


## Chlorothalonil: CYP2A6

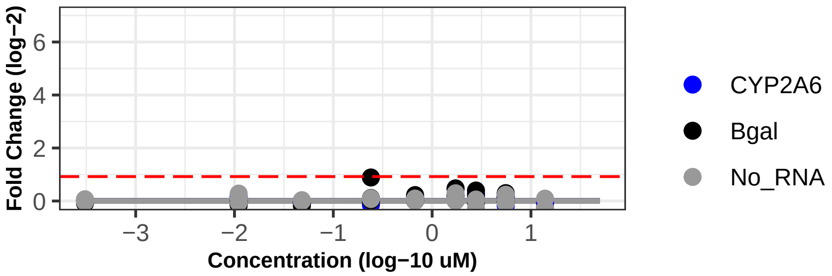


## Chlorothalonil: CYP2D6

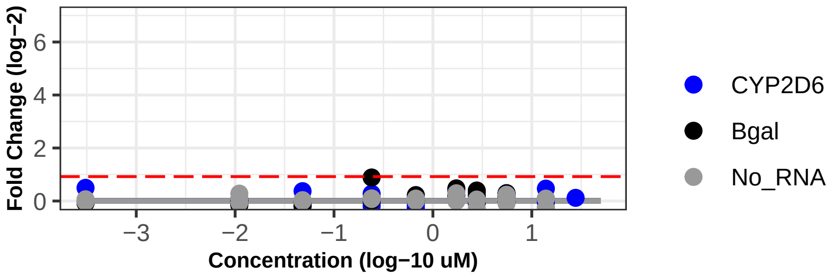


## Chlorothalonil: CYP2B6

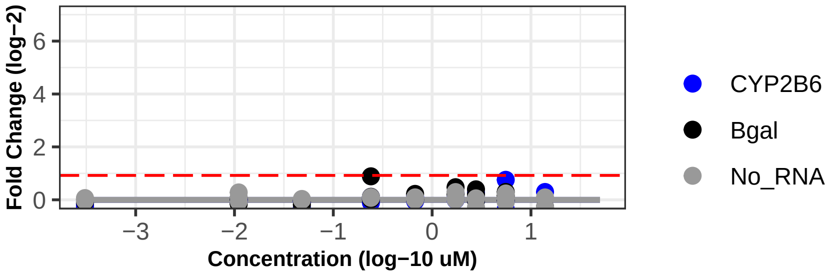


## Chlorothalonil: CYP2E1

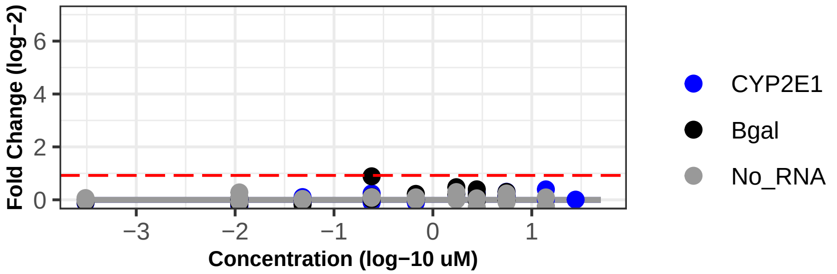


## Chlorothalonil: CYP2C8

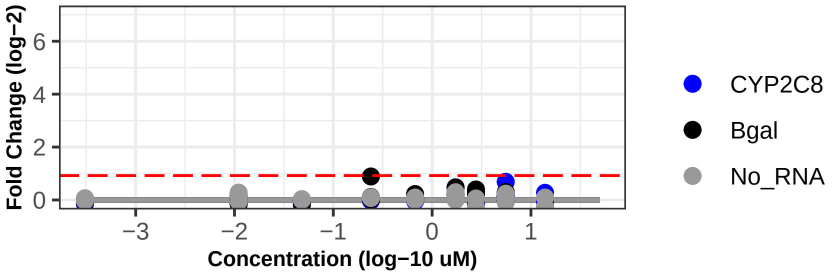


## Chlorothalonil: CYP2J2

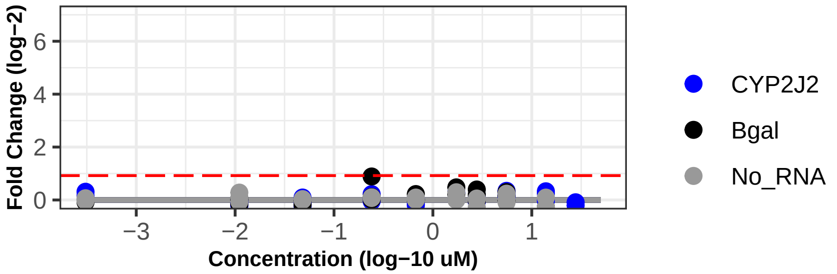


## Chlorothalonil: CYP2C9

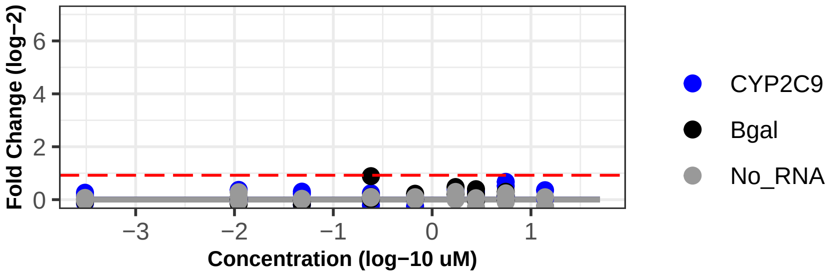


## Chlorothalonil: CYP3A4

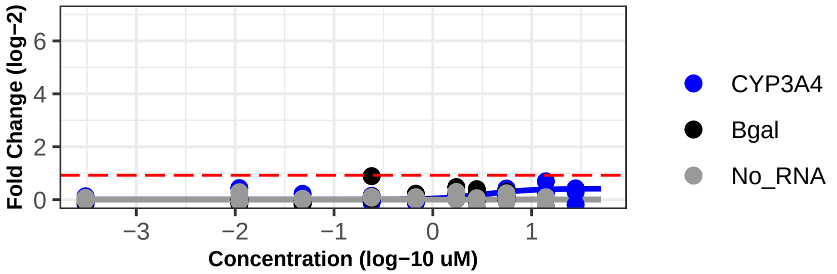

## Chlorpyrifos: CYP1A2

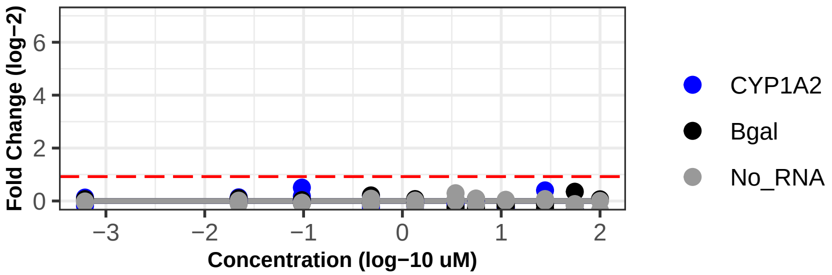


## Chlorpyrifos: CYP2A6

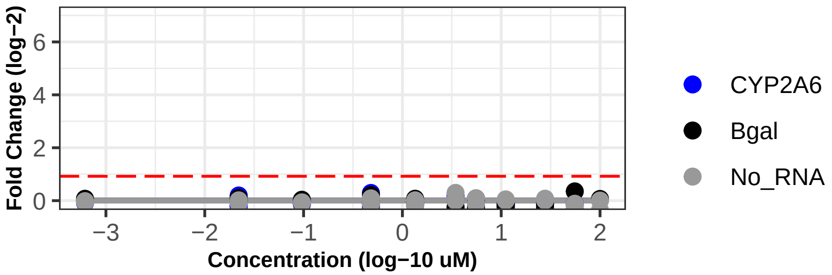


## Chlorpyrifos: CYP2B6

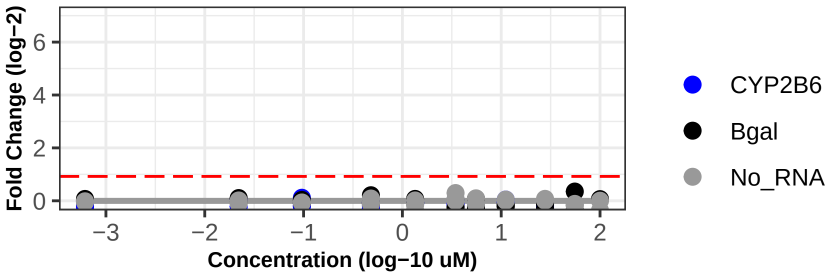


## Chlorpyrifos: CYP2C8

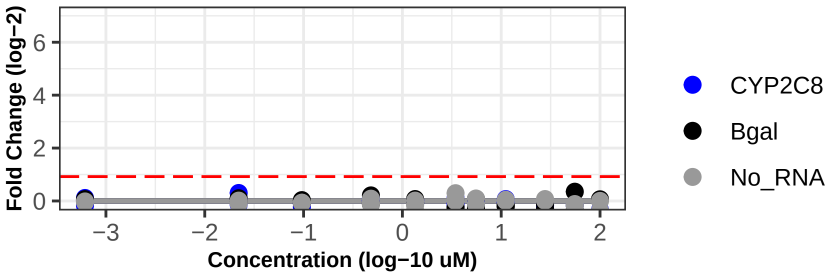


## Chlorpyrifos: CYP2C9

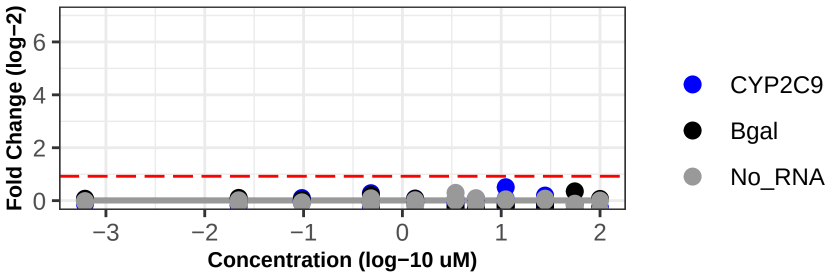


## Chlorpyrifos: CYP2C19

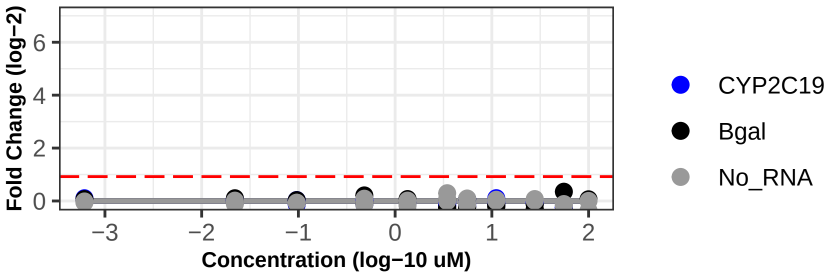


## Chlorpyrifos: CYP2D6

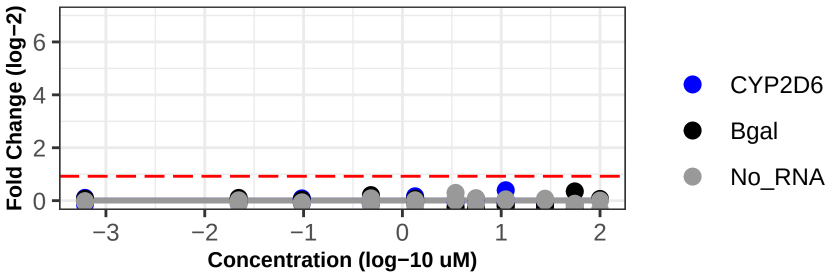


## Chlorpyrifos: CYP2E1

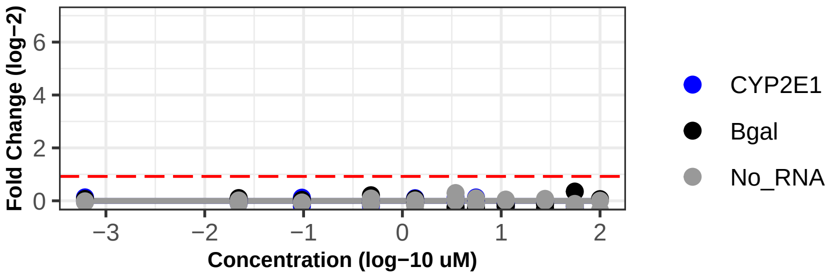


## Chlorpyrifos: CYP2J2

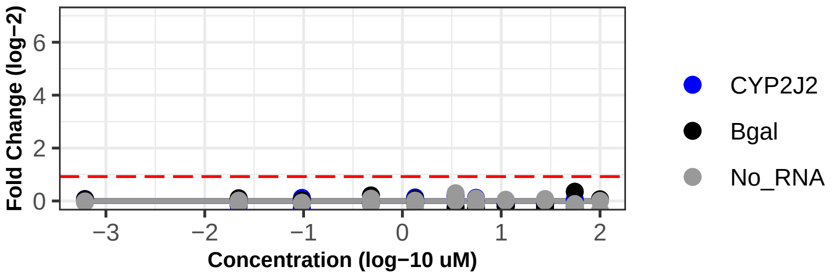


## Chlorpyrifos: CYP3A4

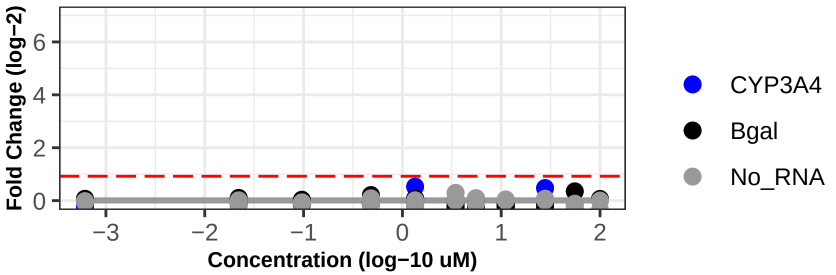

## Chlorpyrifos−methyl: CYP1A2

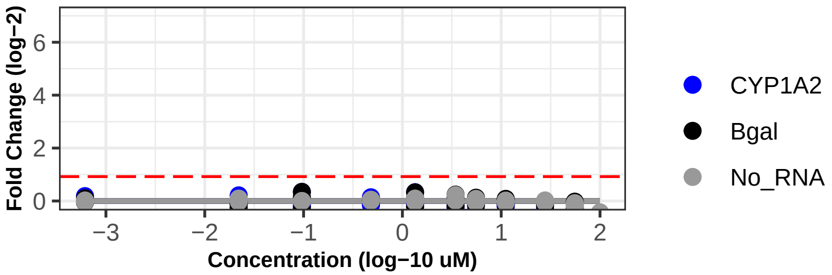


## Chlorpyrifos−methyl: CYP2C19

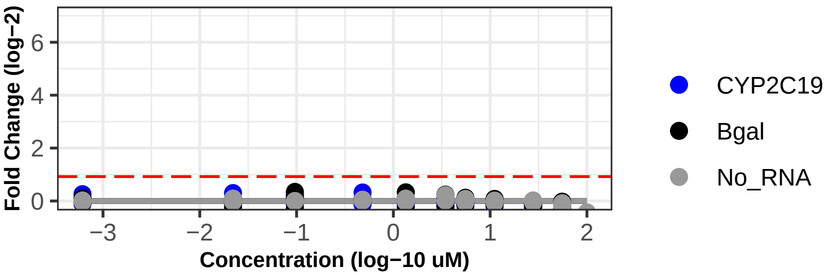


## Chlorpyrifos−methyl: CYP2A6

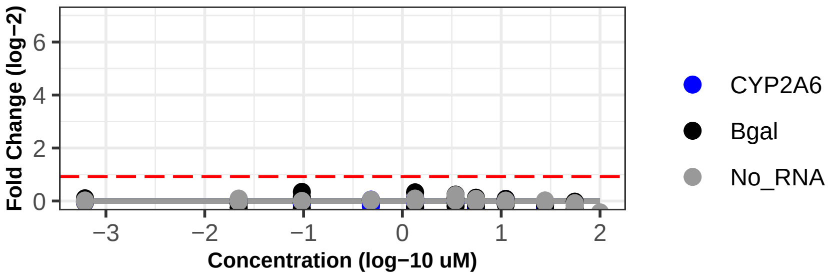


## Chlorpyrifos−methyl: CYP2D6

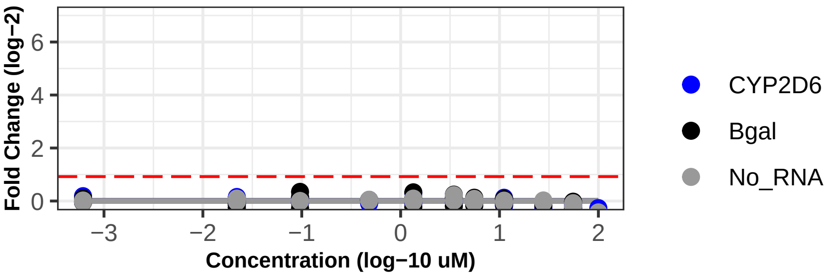


## Chlorpyrifos−methyl: CYP2B6

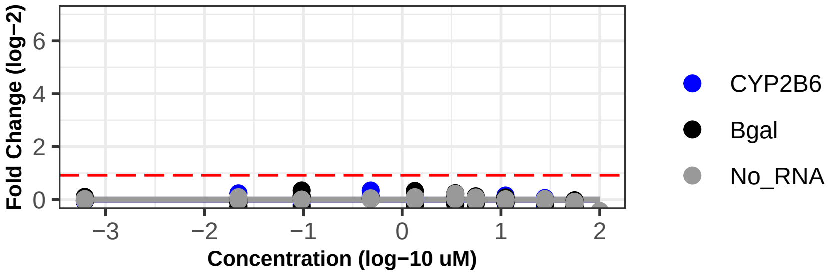


## Chlorpyrifos−methyl: CYP2E1

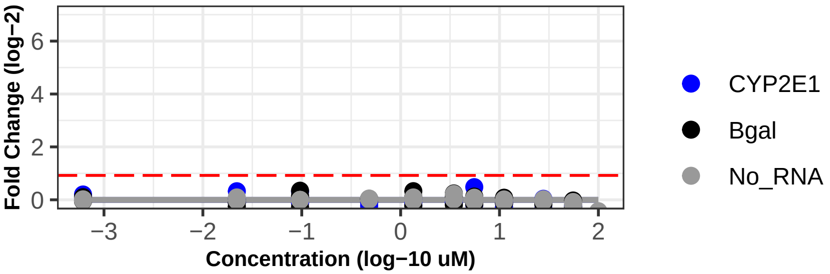


## Chlorpyrifos−methyl: CYP2C8

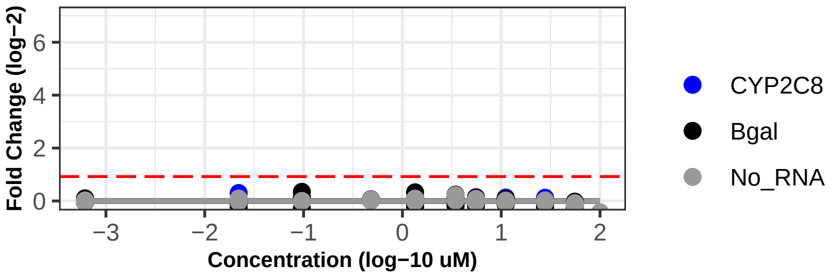


## Chlorpyrifos−methyl: CYP2J2

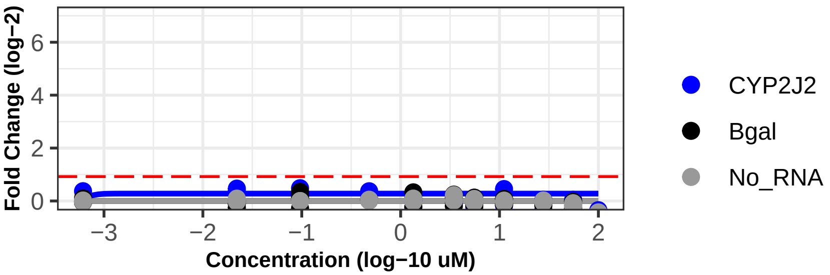


## Chlorpyrifos−methyl: CYP2C9

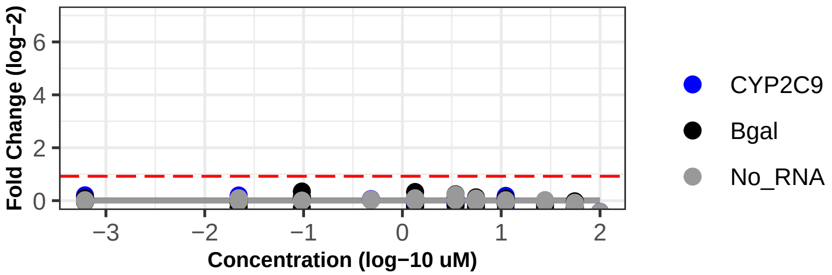


## Chlorpyrifos−methyl: CYP3A4

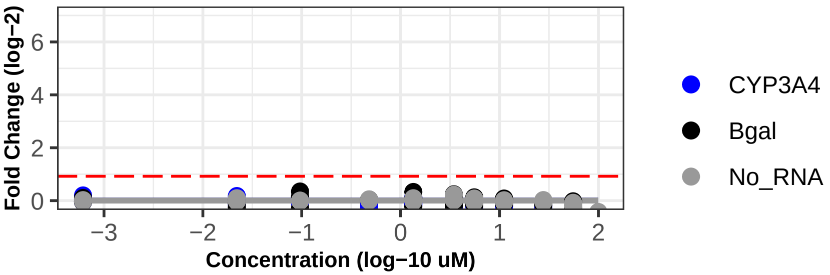

## Clomiphene citrate: CYP1A2

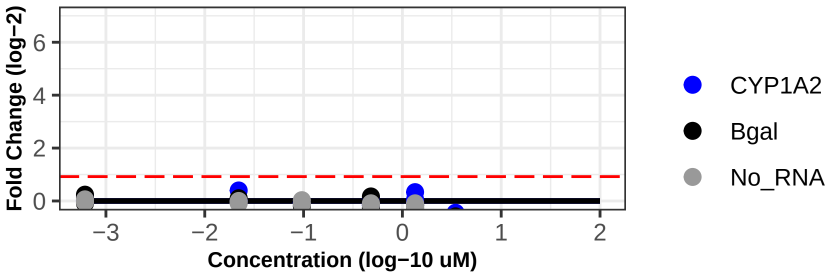


## Clomiphene citrate: CYP2A6

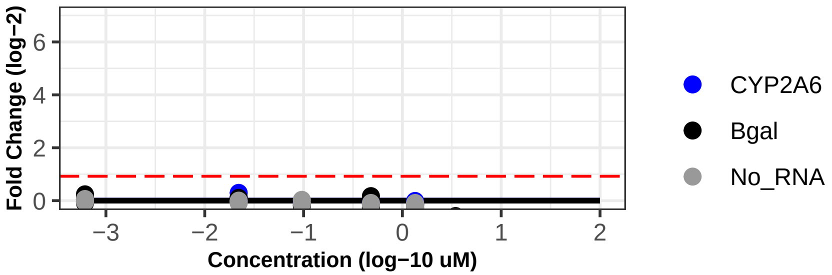


## Clomiphene citrate: CYP2B6

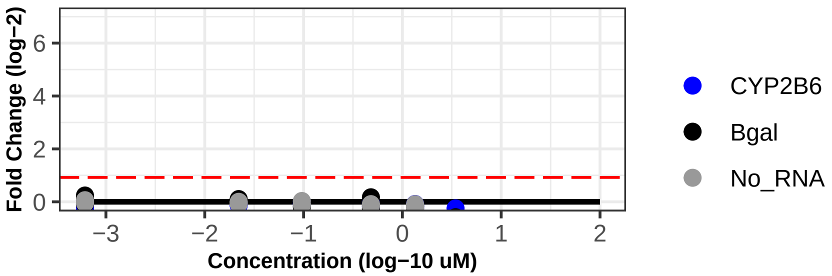


## Clomiphene citrate: CYP2C8

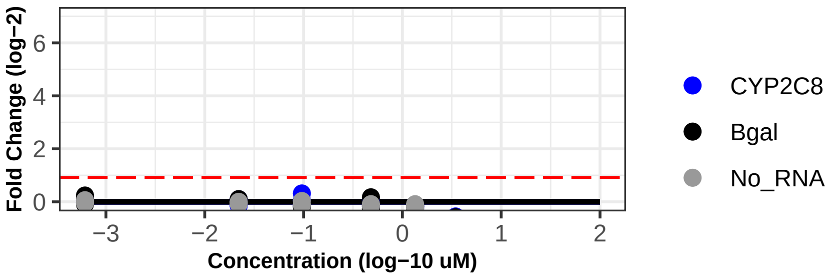


## Clomiphene citrate: CYP2C9

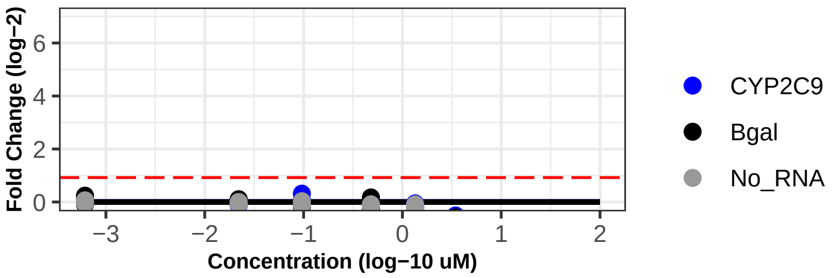


## Clomiphene citrate: CYP2C19

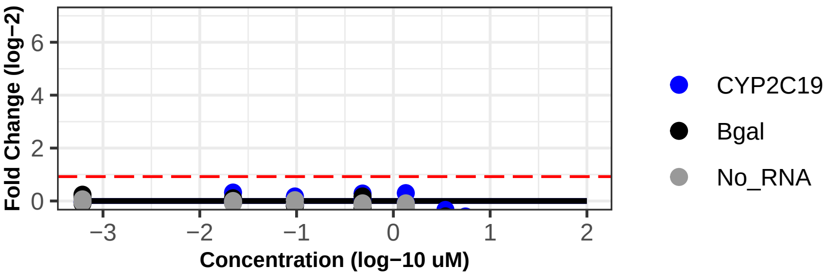


## Clomiphene citrate: CYP2D6

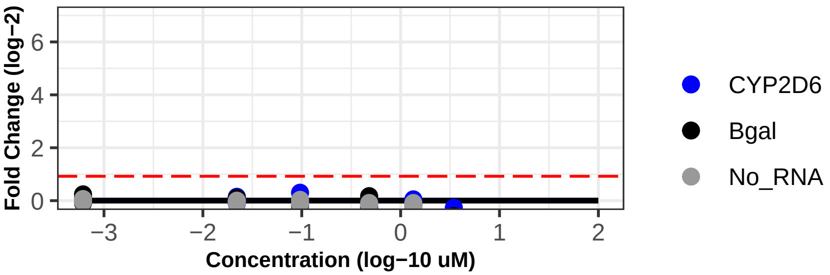


## Clomiphene citrate: CYP2E1

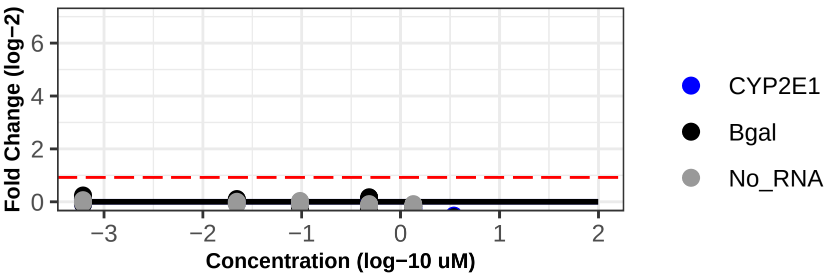


## Clomiphene citrate: CYP2J2

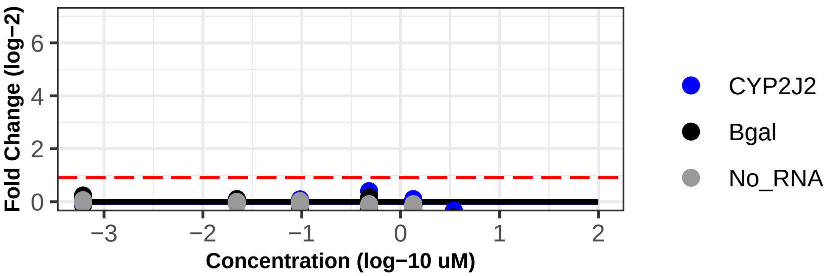


## Clomiphene citrate: CYP3A4

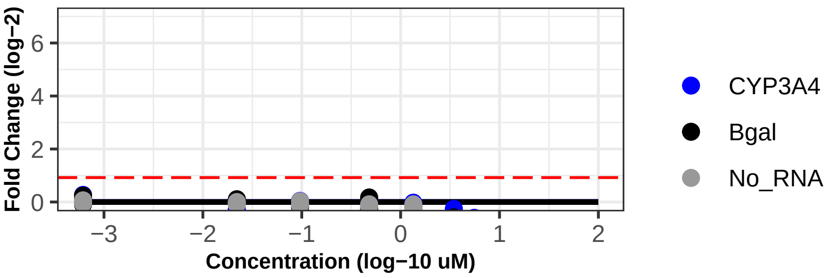

## Corticosterone: CYP1A2

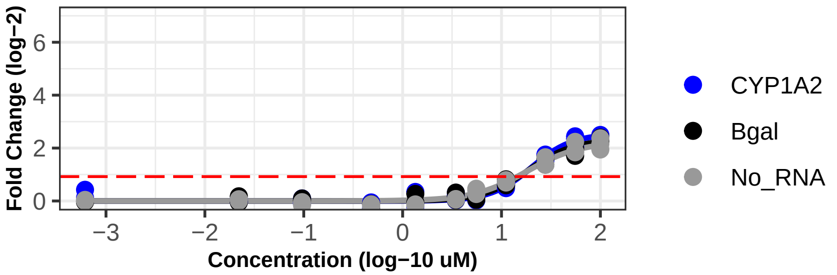


## Corticosterone: CYP2C19

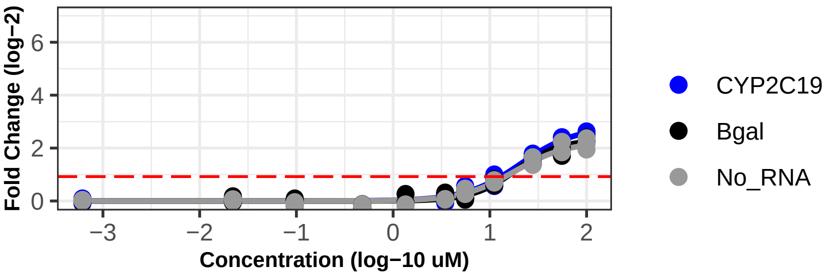


## Corticosterone: CYP2A6

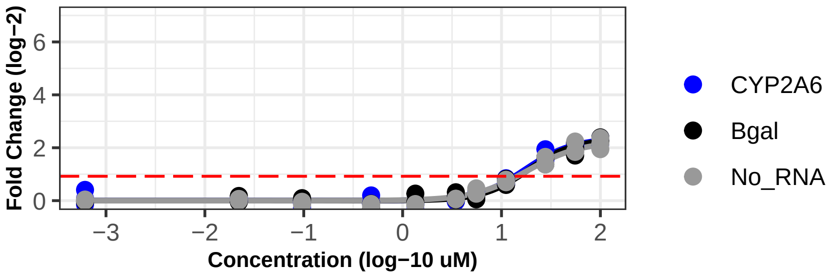


## Corticosterone: CYP2D6

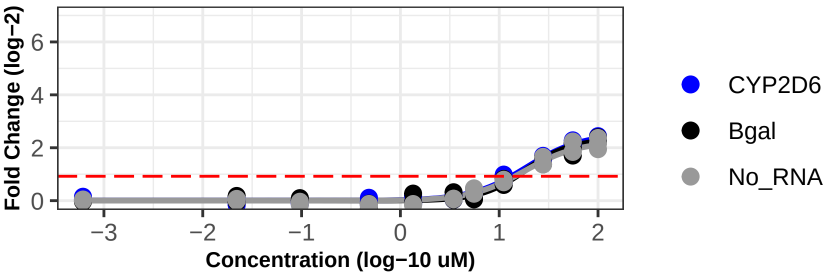


## Corticosterone: CYP2B6

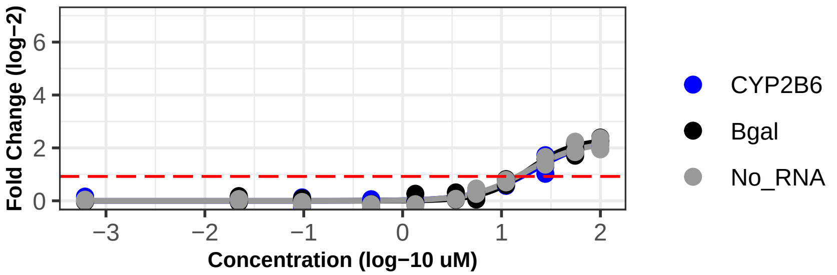


## Corticosterone: CYP2E1

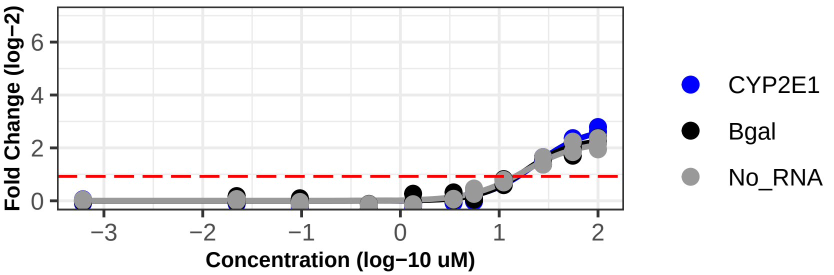


## Corticosterone: CYP2C8

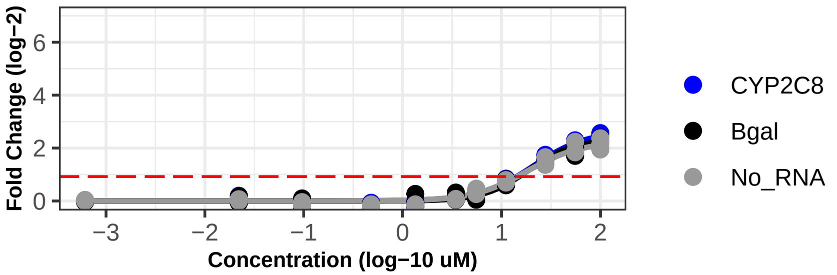


## Corticosterone: CYP2J2

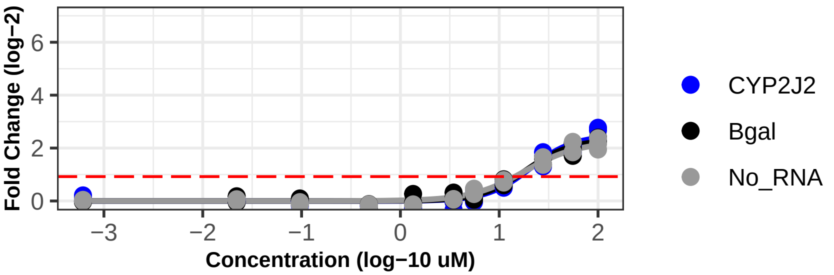


## Corticosterone: CYP2C9

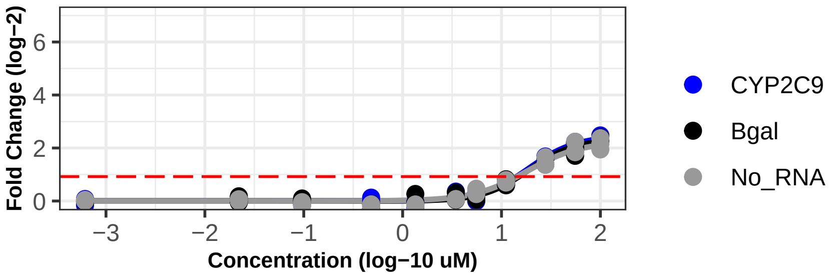


## Corticosterone: CYP3A4

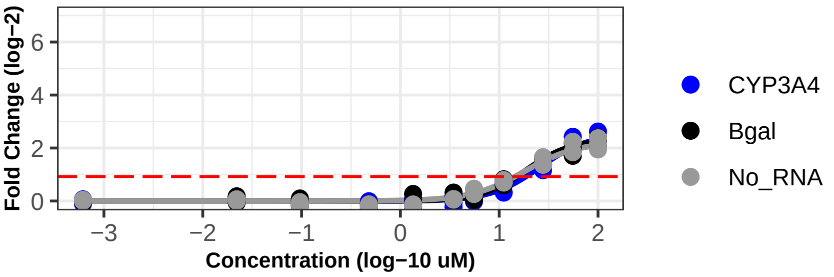

Coumestrol : CYP1A2

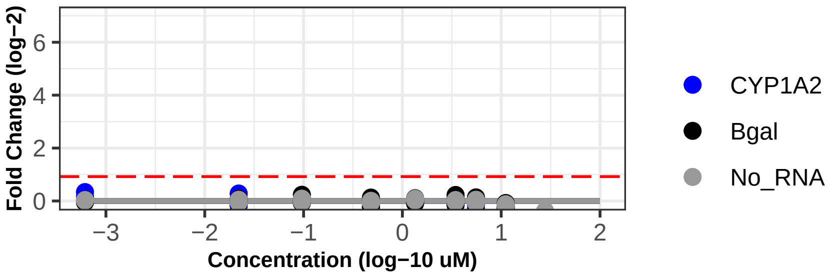


Coumestrol : CYP2C19

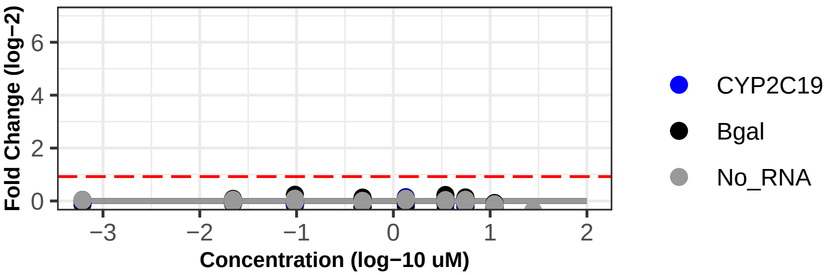


Coumestrol : CYP2A6

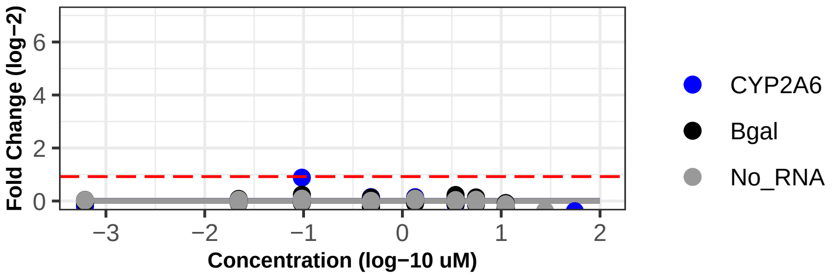


Coumestrol : CYP2D6

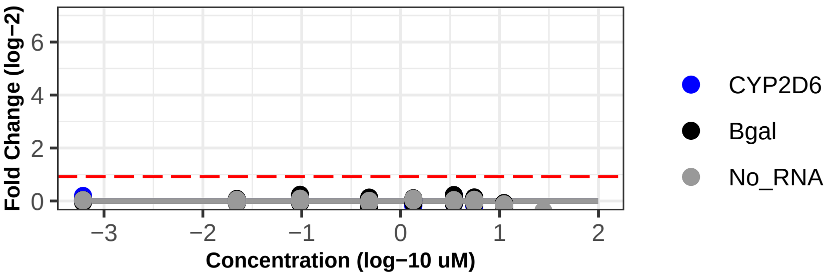


Coumestrol : CYP2B6

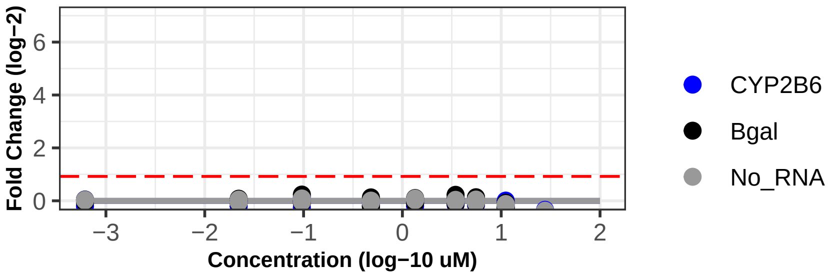


Coumestrol : CYP2E1

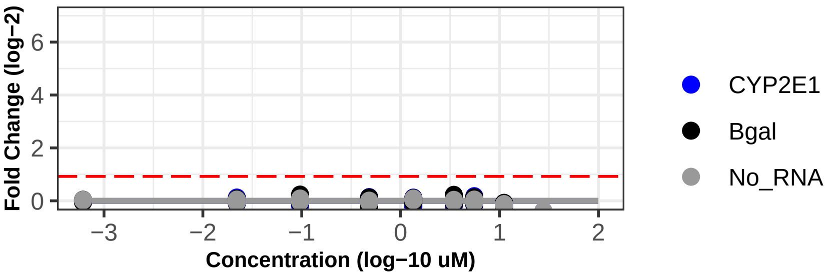


Coumestrol : CYP2C8

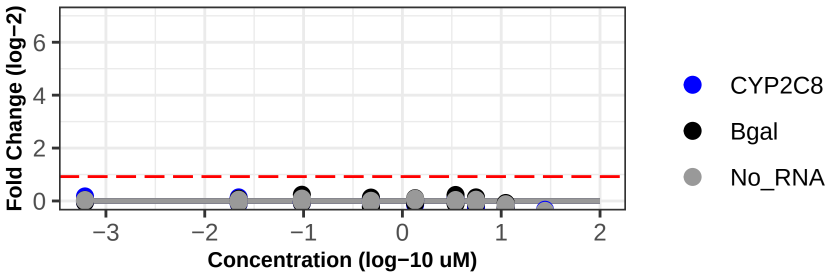


Coumestrol : CYP2J2

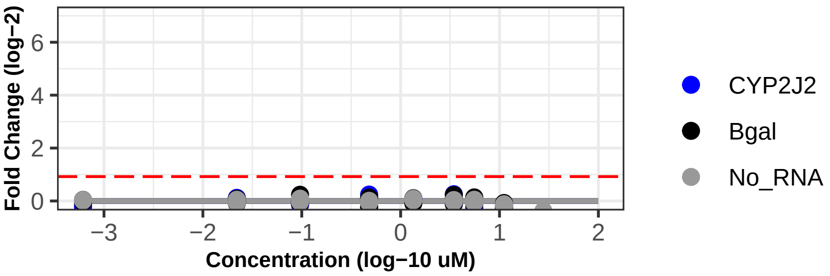


Coumestrol : CYP2C9

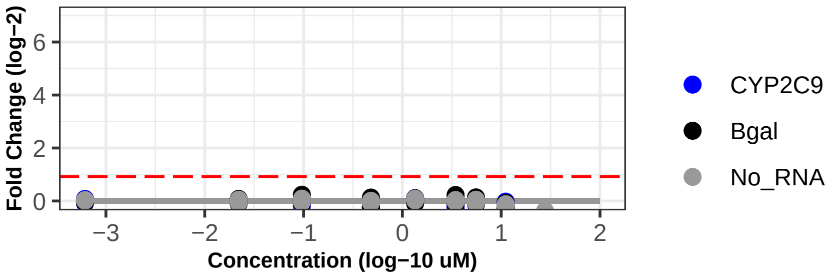


Coumestrol : CYP3A4

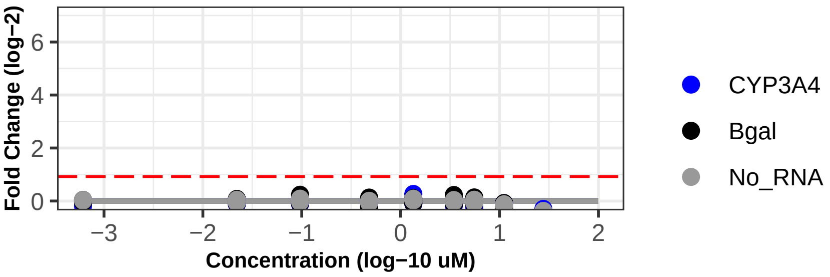

## Cyfluthrin: CYP1A2

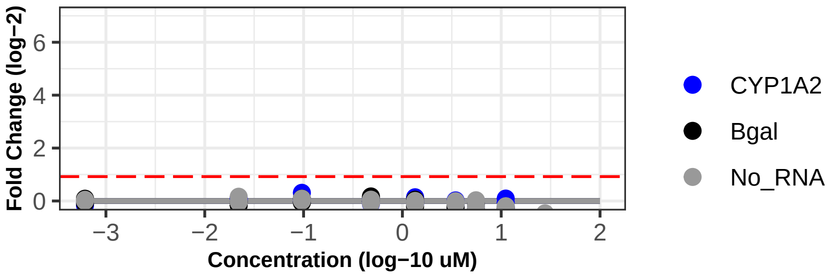


## Cyfluthrin: CYP2C19

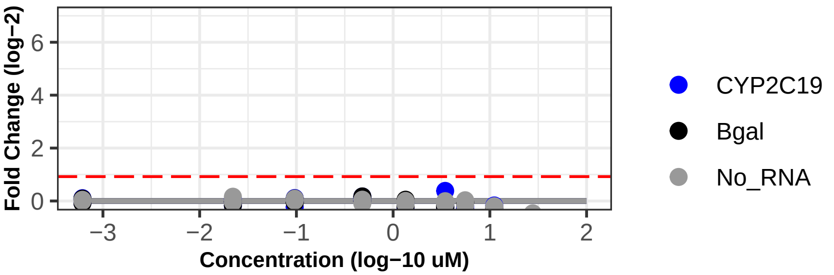


## Cyfluthrin: CYP2A6

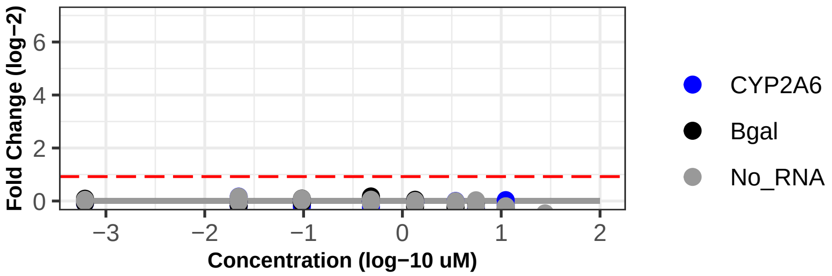


## Cyfluthrin: CYP2D6

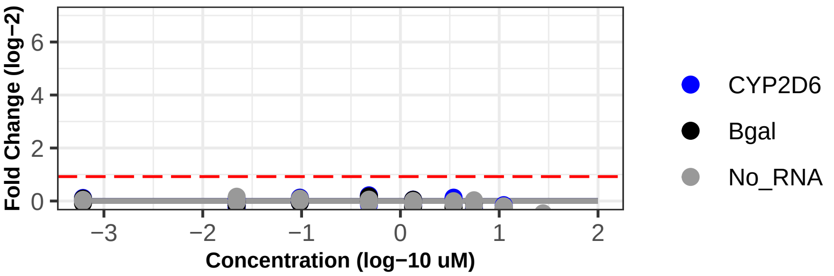


## Cyfluthrin: CYP2B6

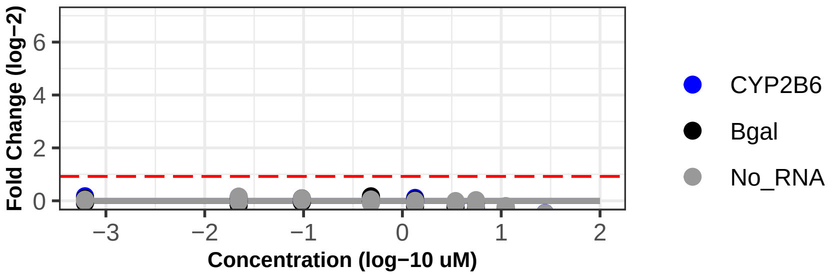


## Cyfluthrin: CYP2E1

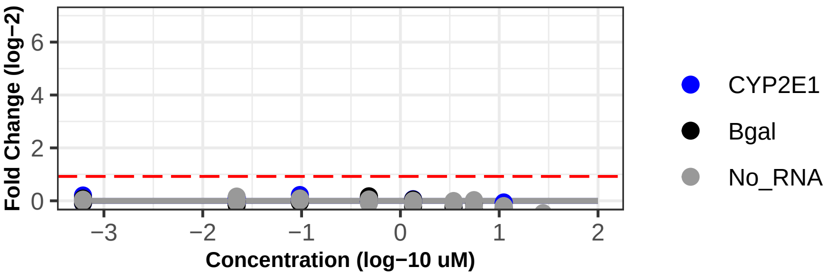


## Cyfluthrin: CYP2C8

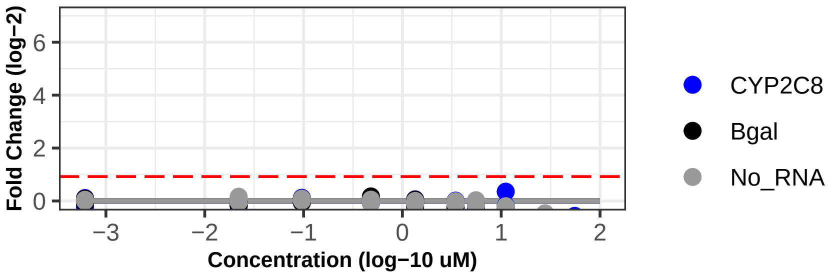


## Cyfluthrin: CYP2J2

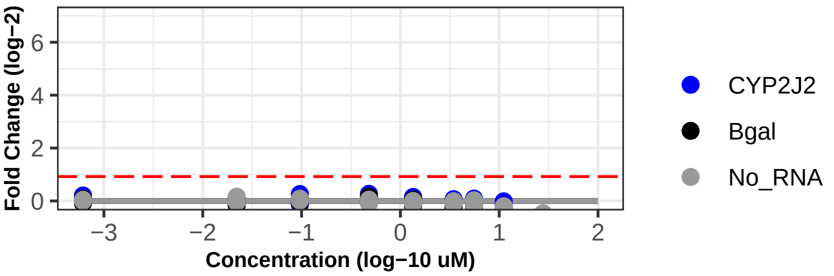


## Cyfluthrin: CYP2C9

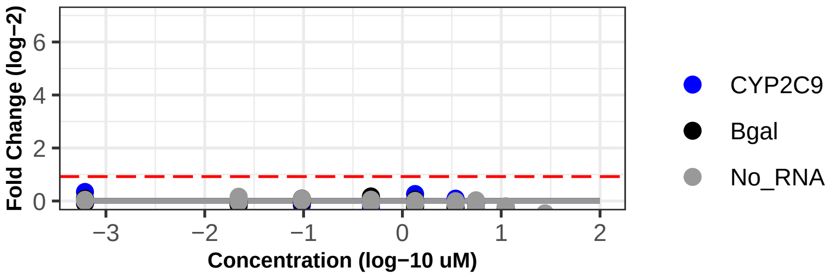


## Cyfluthrin: CYP3A4

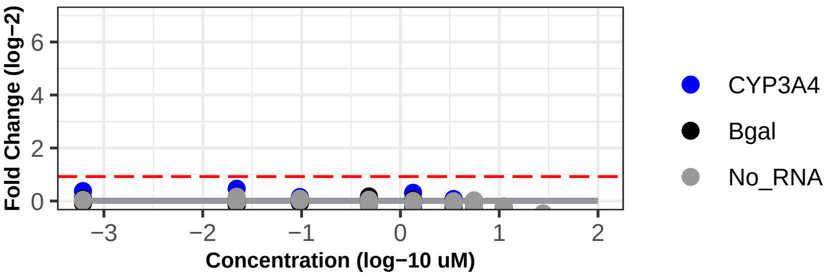

Cypermethrin: CYP1A2

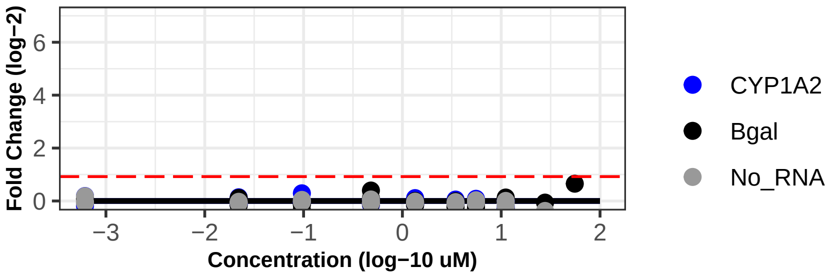


Cypermethrin: CYP2C19

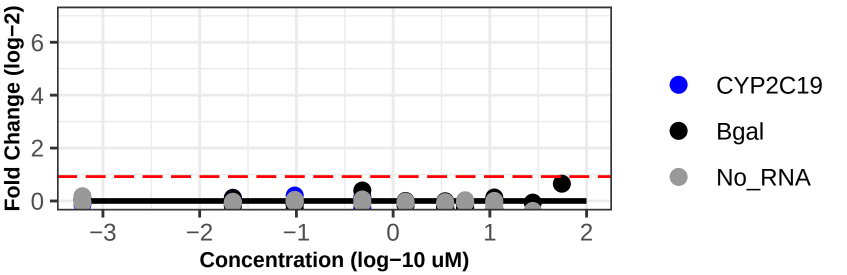


Cypermethrin: CYP2A6

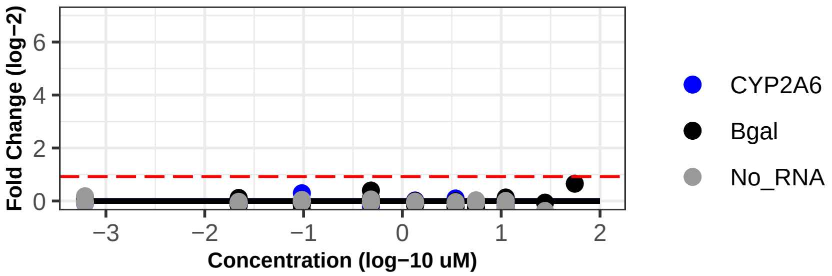


Cypermethrin: CYP2D6

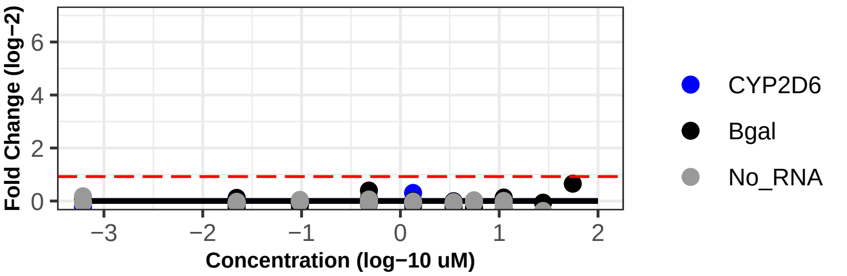


Cypermethrin: CYP2B6

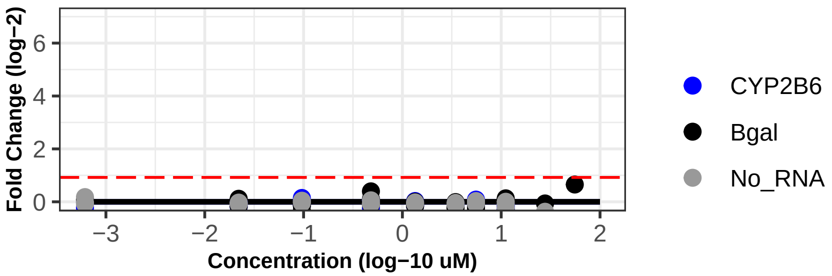


Cypermethrin: CYP2E1

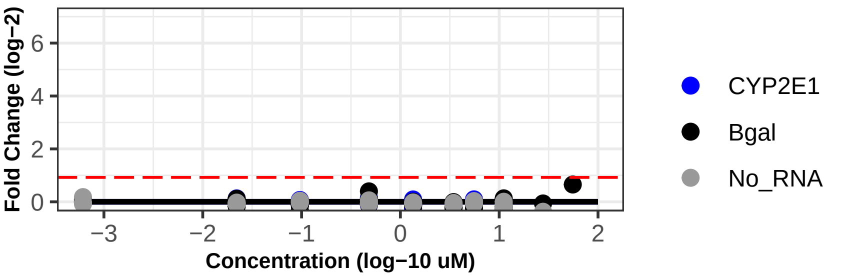


Cypermethrin: CYP2C8

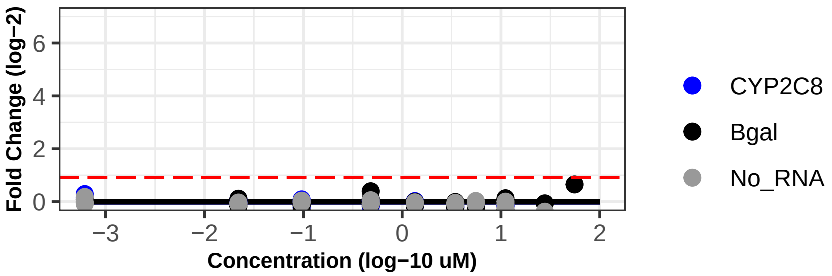


Cypermethrin: CYP2J2

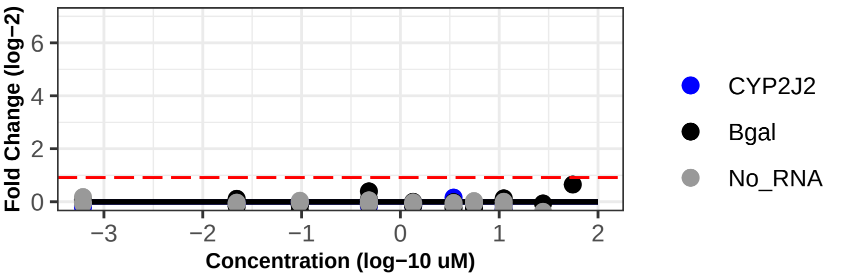


Cypermethrin: CYP2C9

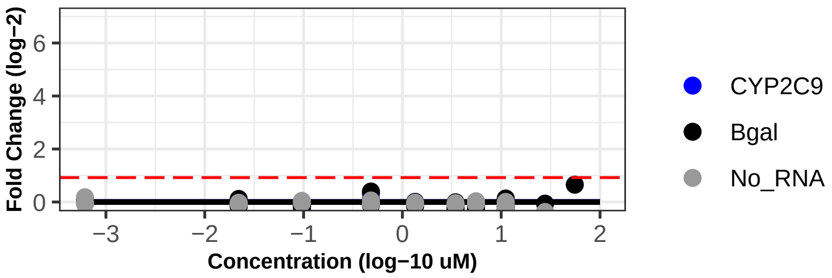


Cypermethrin: CYP3A4

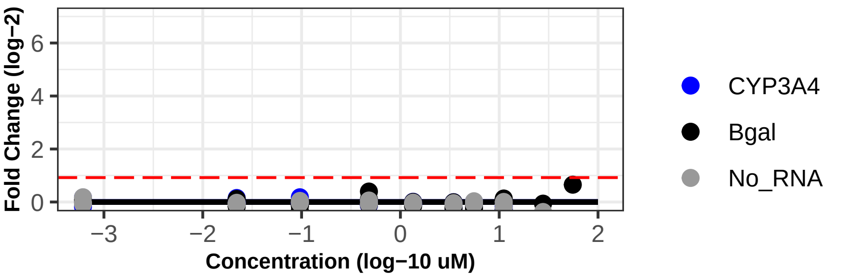

Cyproterone acetate: CYP1A2

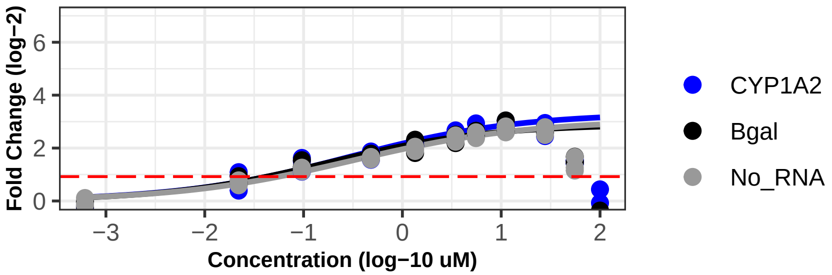


Cyproterone acetate: CYP2C19

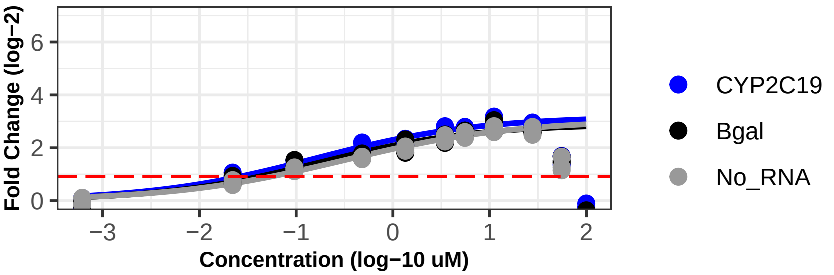


Cyproterone acetate: CYP2A6

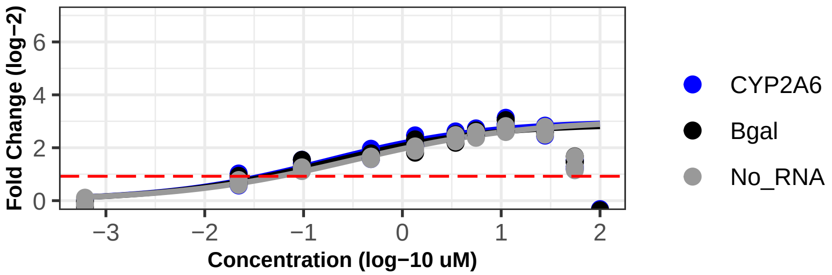


Cyproterone acetate: CYP2D6

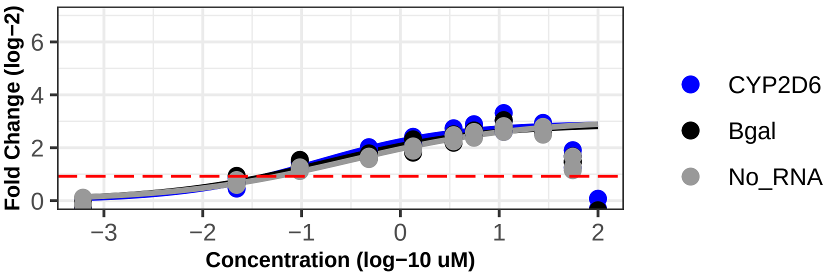


Cyproterone acetate: CYP2B6

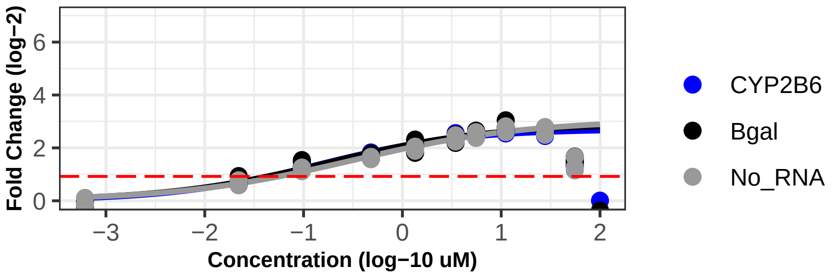


Cyproterone acetate: CYP2E1

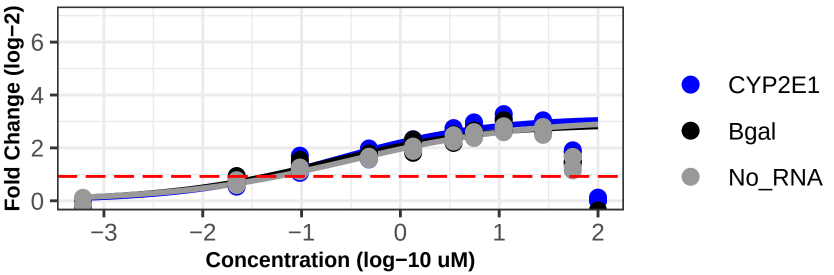


Cyproterone acetate: CYP2C8

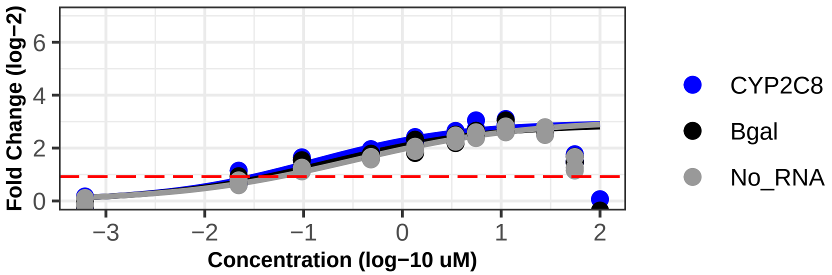


Cyproterone acetate: CYP2J2

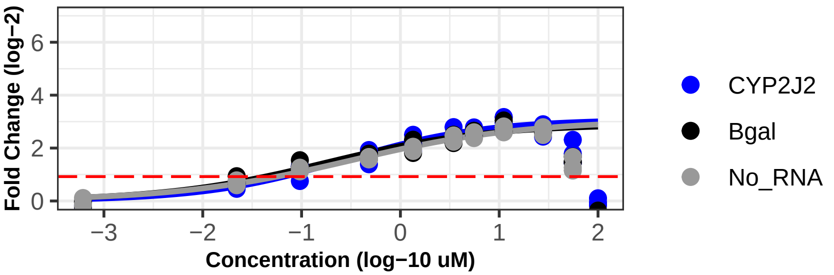


Cyproterone acetate: CYP2C9

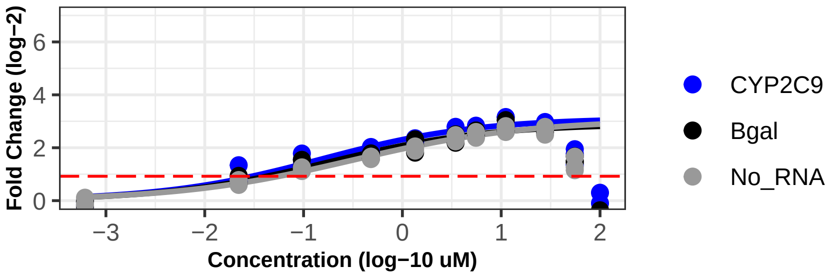


Cyproterone acetate: CYP3A4

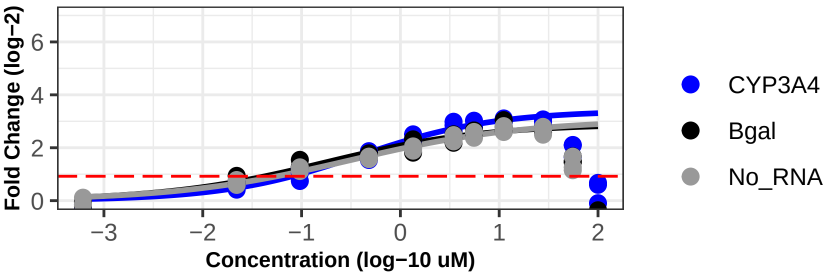

**DCLN: CYP1A2**

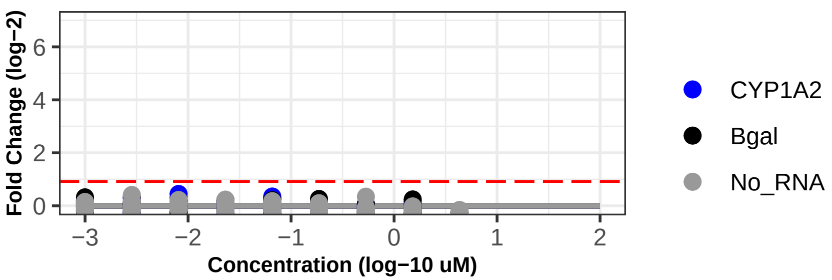


**DCLN: CYP2C19**

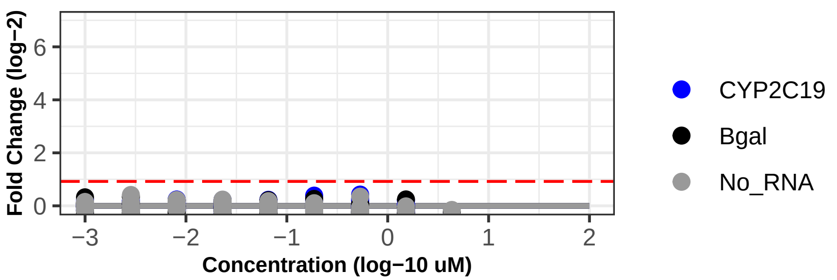


**DCLN: CYP2A6**

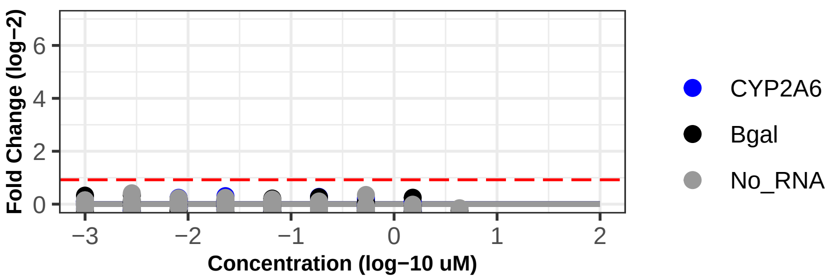


**DCLN: CYP2D6**

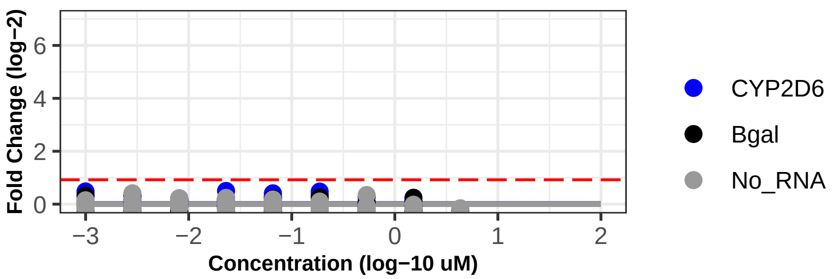


**DCLN: CYP2B6**

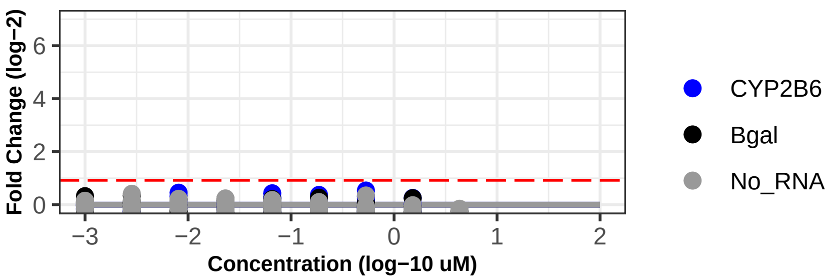


**DCLN: CYP2E1**

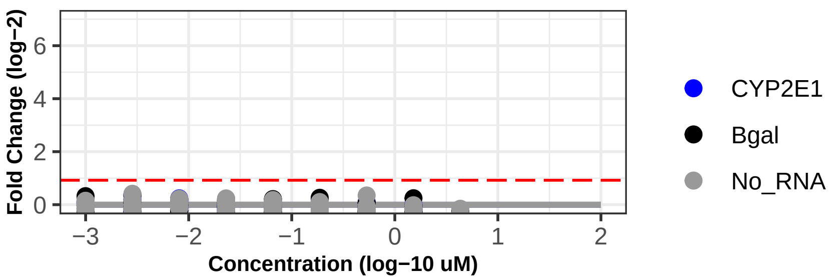


**DCLN: CYP2C8**

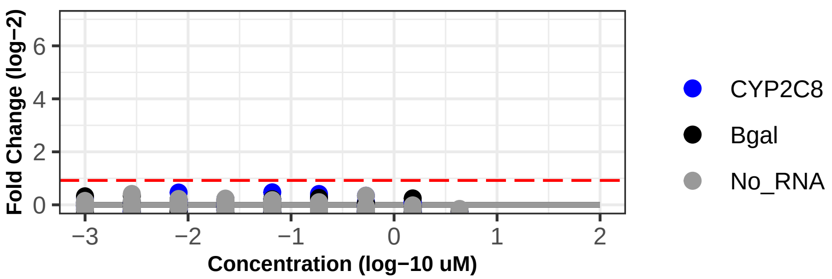


**DCLN: CYP2J2**

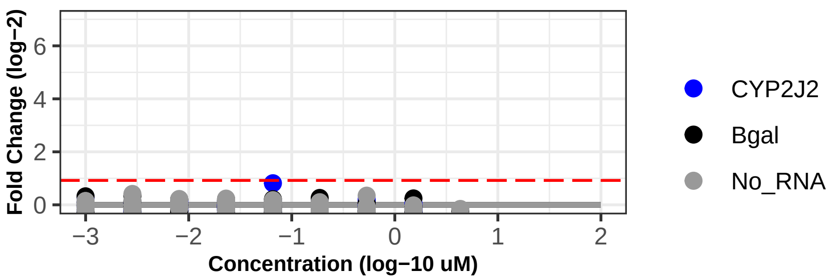


**DCLN: CYP2C9**

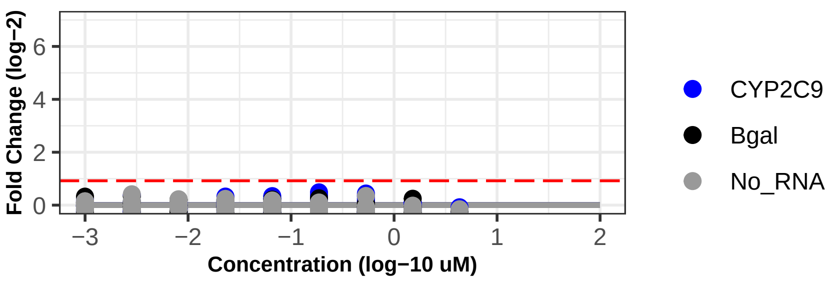


**DCLN: CYP3A4**

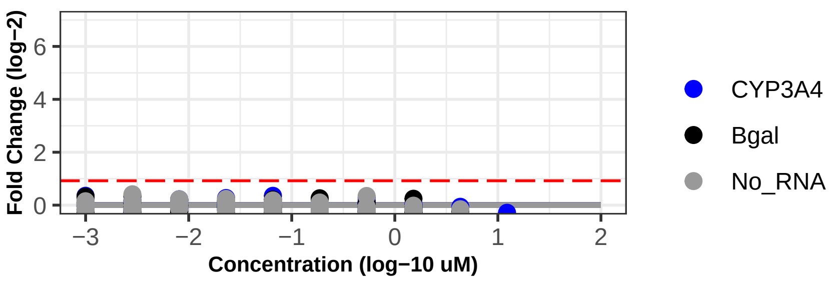

## Daidzein : CYP1A2

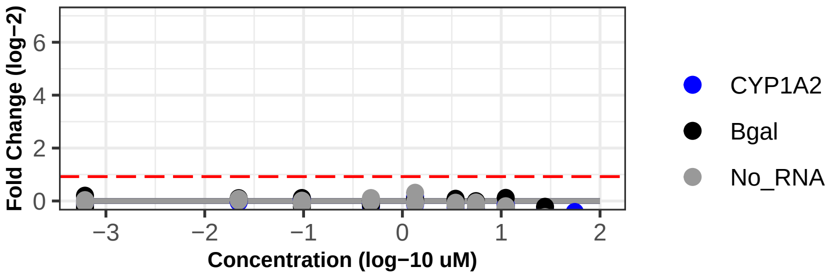


## Daidzein : CYP2A6

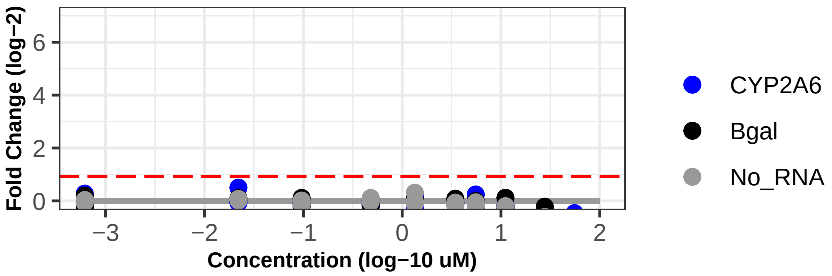


## Daidzein : CYP2B6

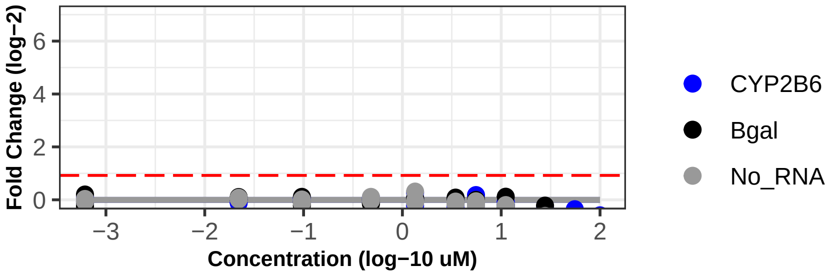


## Daidzein : CYP2C8

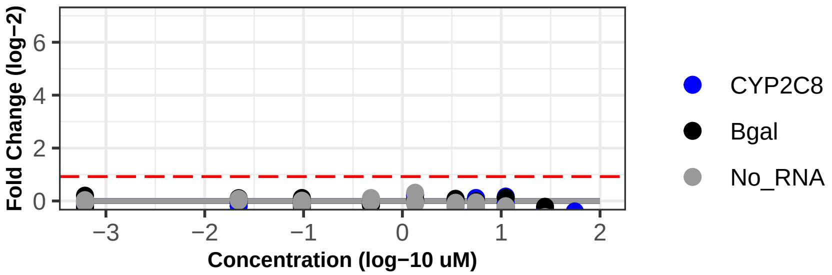


## Daidzein : CYP2C9

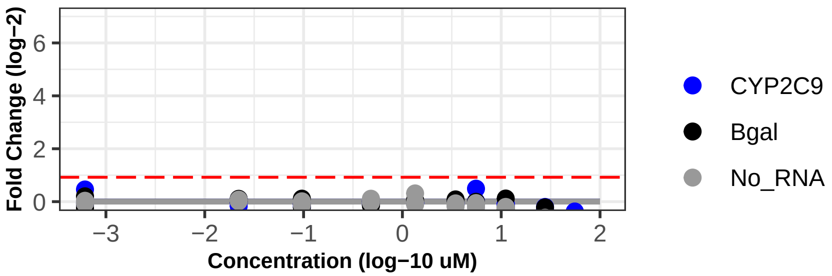


## Daidzein : CYP2C19

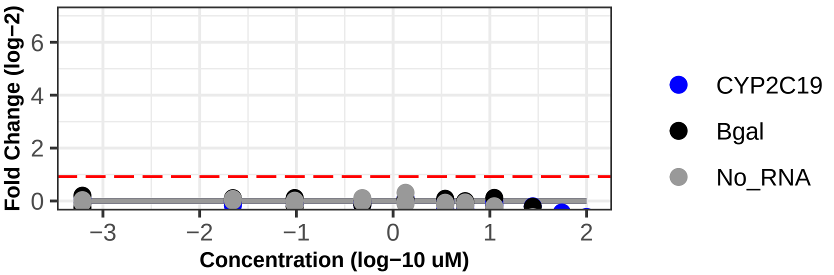


## Daidzein : CYP2D6

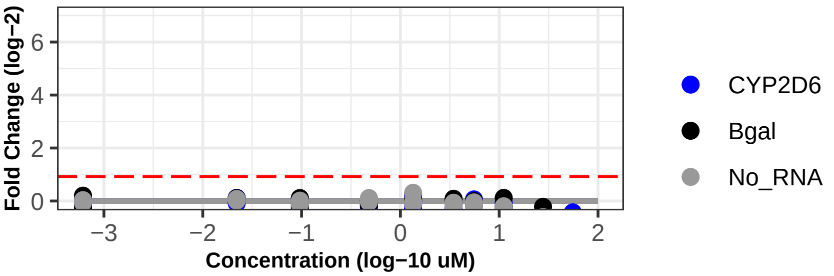


## Daidzein : CYP2E1

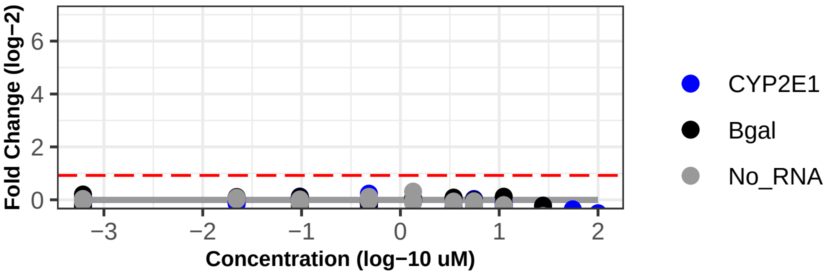


## Daidzein : CYP2J2

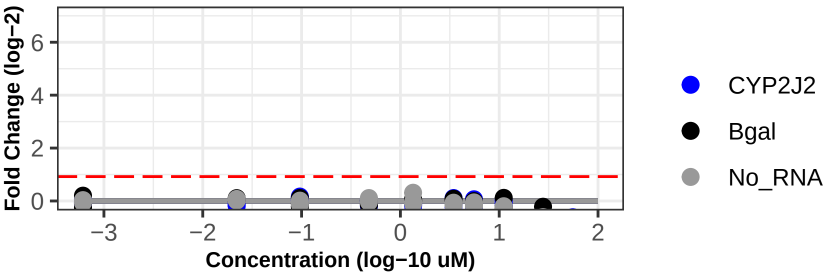


## Daidzein : CYP3A4

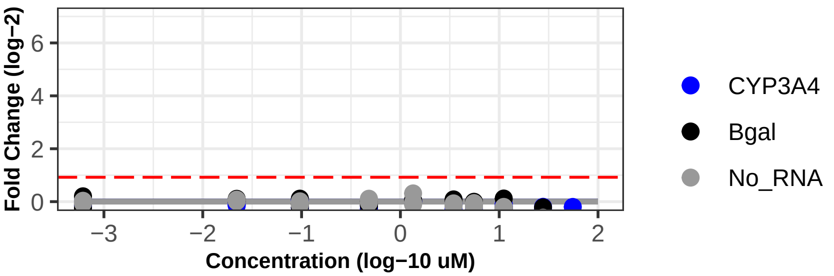

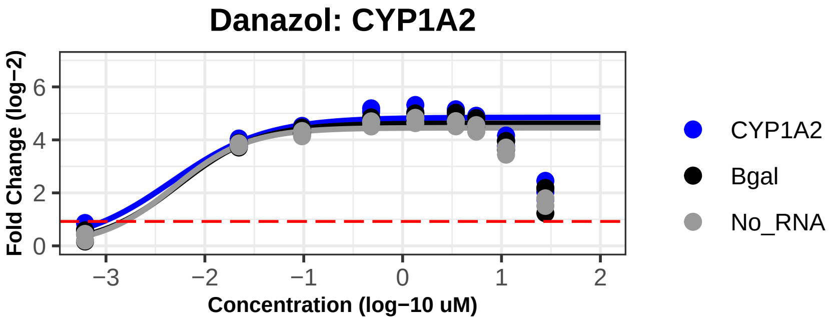
Danazol: CYP1A2
Fold Change (log−2)
Concentration (log−10 uM)
CYP1A2
Bgal
No_RNA

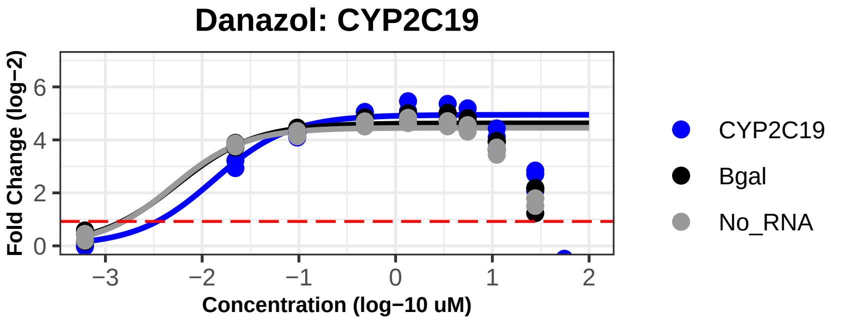
Danazol: CYP2C19
Fold Change (log−2)
Concentration (log−10 uM)
CYP2C19
Bgal
No_RNA

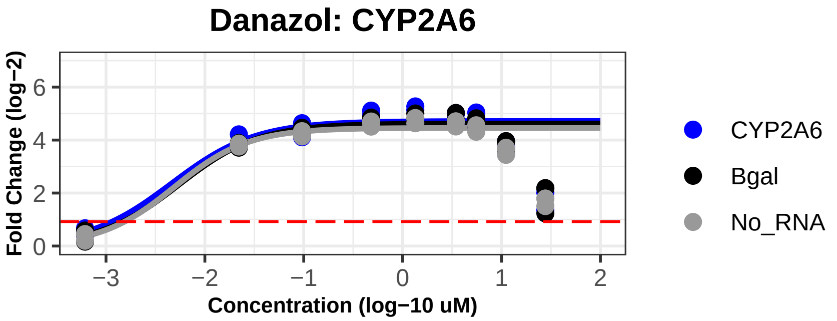
Danazol: CYP2A6
Fold Change (log−2)
Concentration (log−10 uM)
CYP2A6
Bgal
No_RNA

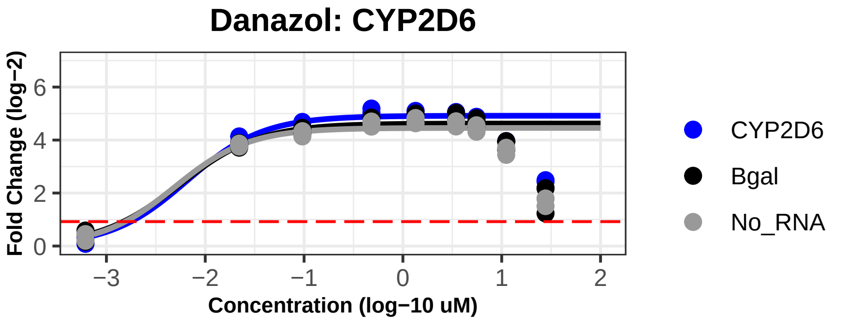
Danazol: CYP2D6
Fold Change (log−2)
Concentration (log−10 uM)
CYP2D6
Bgal
No_RNA

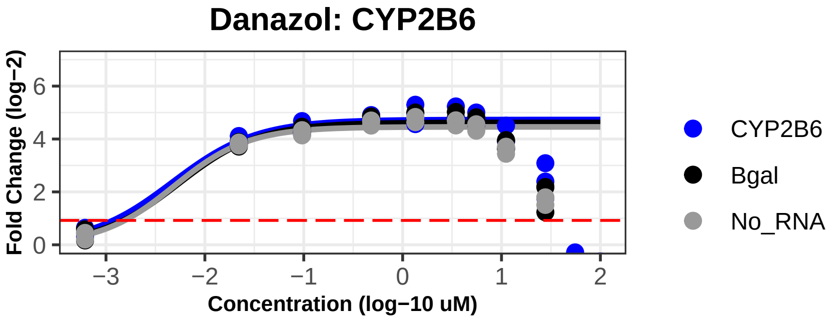
Danazol: CYP2B6
Fold Change (log−2)
Concentration (log−10 uM)
CYP2B6
Bgal
No_RNA

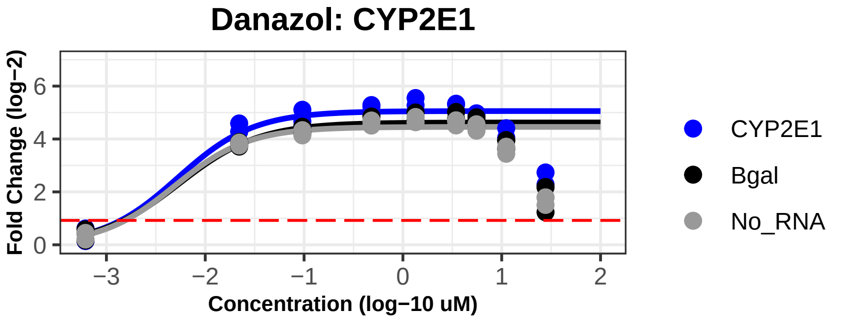
Danazol: CYP2E1
Fold Change (log−2)
Concentration (log−10 uM)
CYP2E1
Bgal
No_RNA

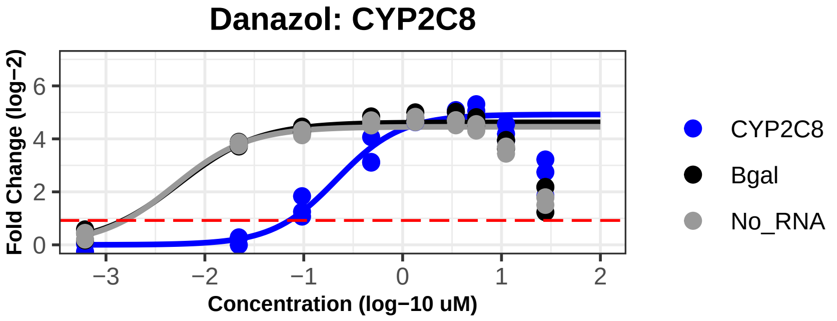
Danazol: CYP2C8
Fold Change (log−2)
Concentration (log−10 uM)
CYP2C8
Bgal
No_RNA

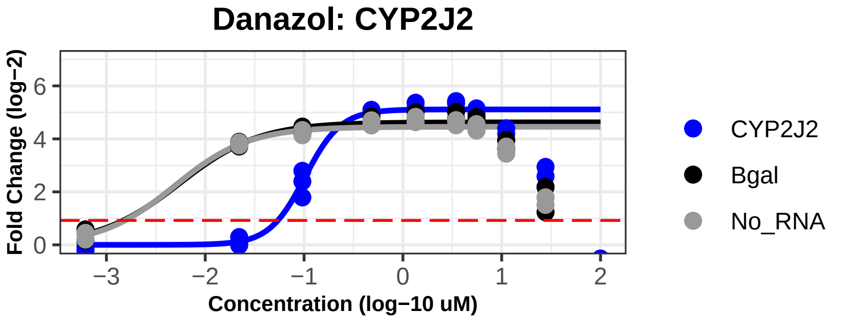
Danazol: CYP2J2
Fold Change (log−2)
Concentration (log−10 uM)
CYP2J2
Bgal
No_RNA

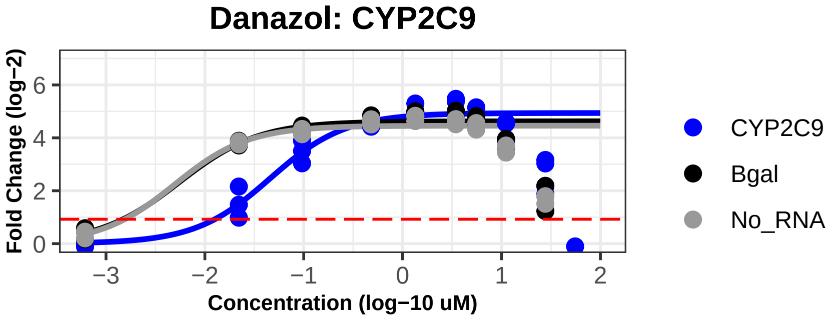
Danazol: CYP2C9
Fold Change (log−2)
Concentration (log−10 uM)
CYP2C9
Bgal
No_RNA

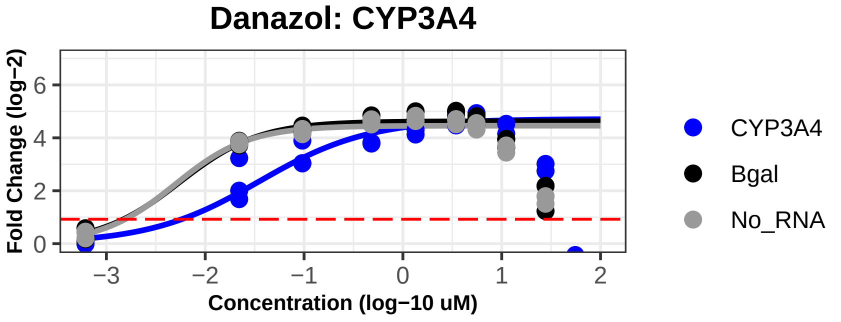
Danazol: CYP3A4
Fold Change (log−2)
Concentration (log−10 uM)
CYP3A4
Bgal
No_RNA

**Deltamethrin: CYP1A2**

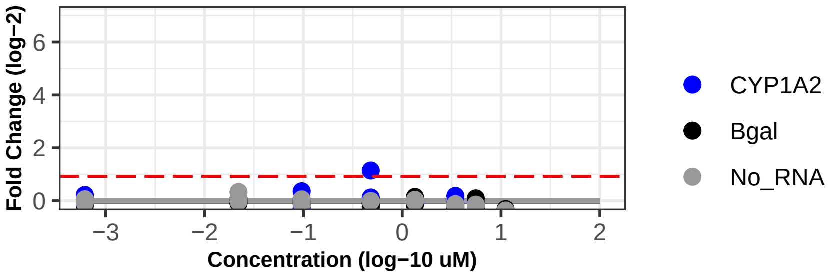


**Deltamethrin: CYP2C19**

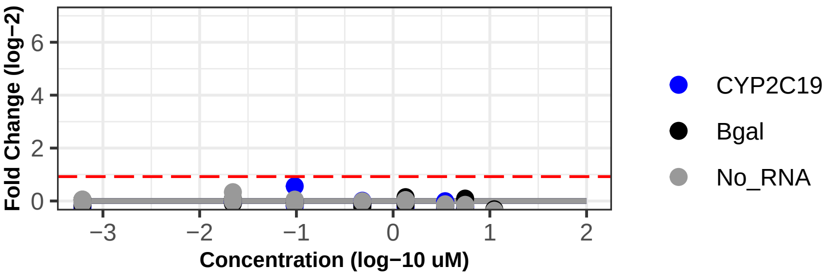


**Deltamethrin: CYP2A6**

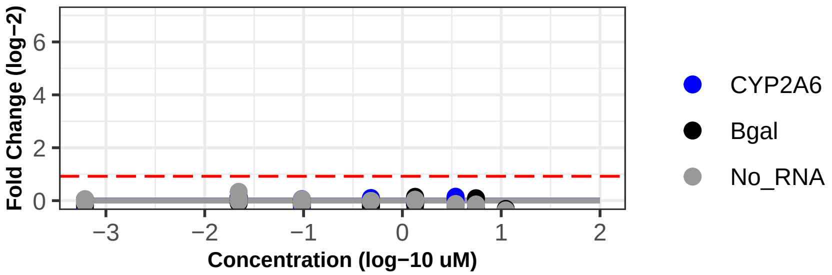


**Deltamethrin: CYP2D6**

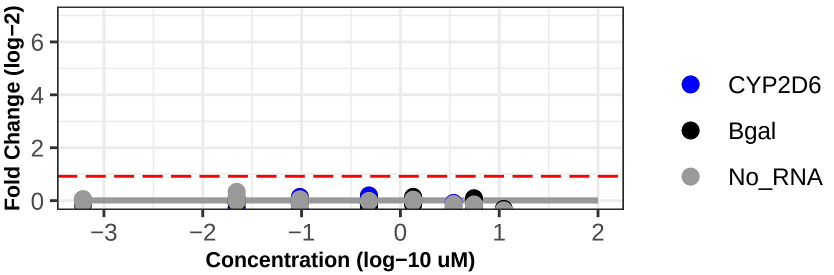


**Deltamethrin: CYP2B6**

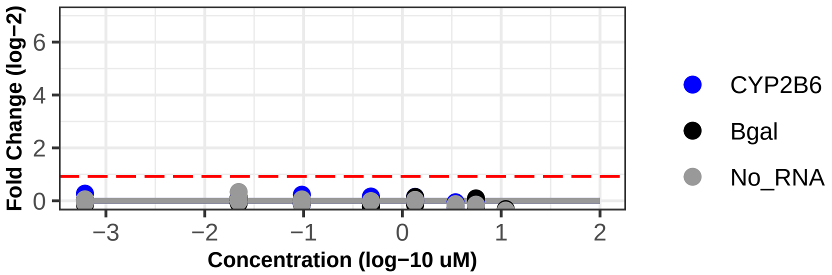


**Deltamethrin: CYP2E1**

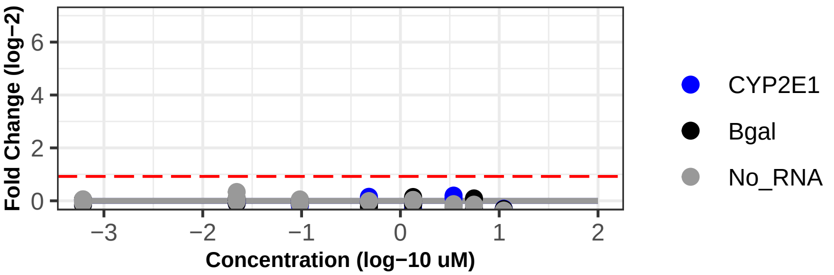


**Deltamethrin: CYP2C8**

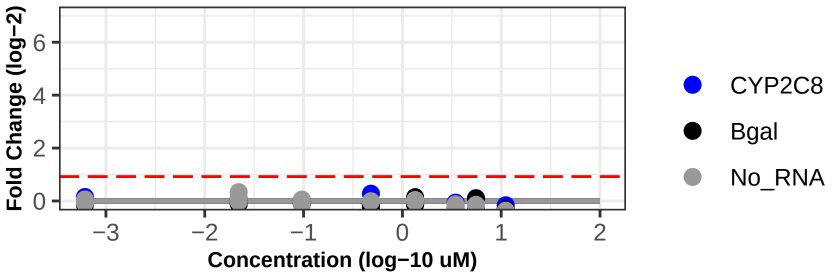


**Deltamethrin: CYP2J2**

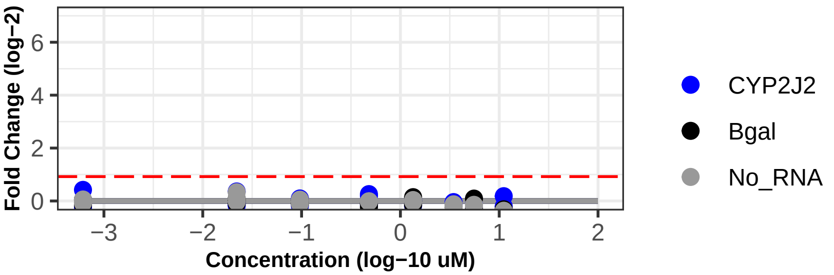


**Deltamethrin: CYP2C9**

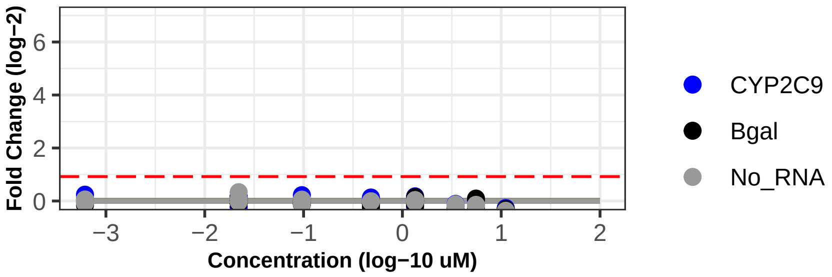


**Deltamethrin: CYP3A4**

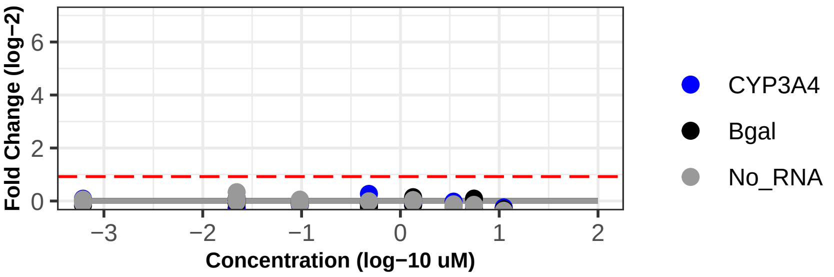

Diazinon: CYP1A2

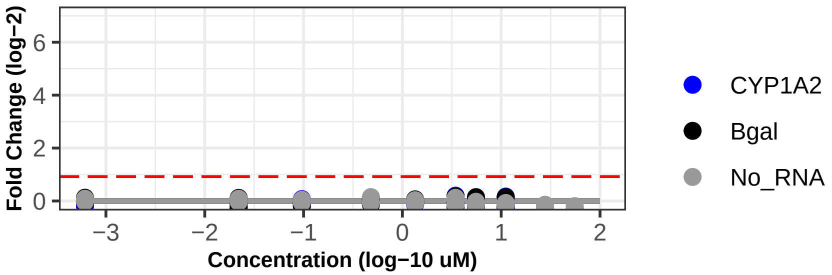


Diazinon: CYP2C19

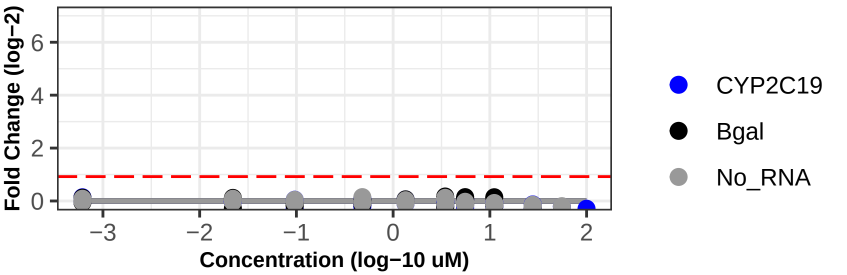


Diazinon: CYP2A6

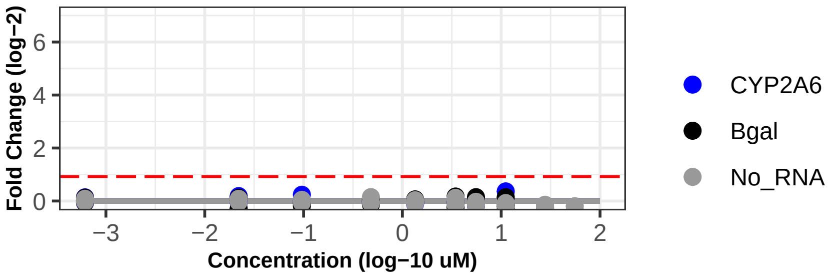


Diazinon: CYP2D6

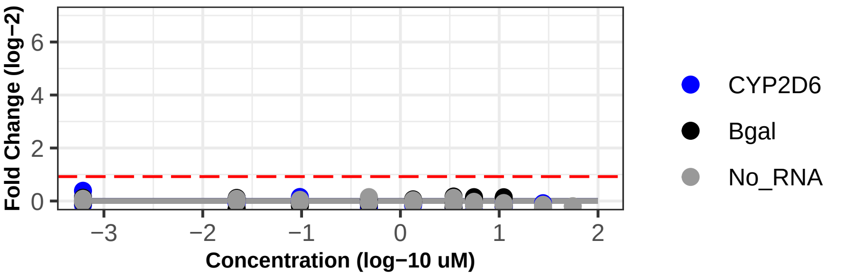


Diazinon: CYP2B6

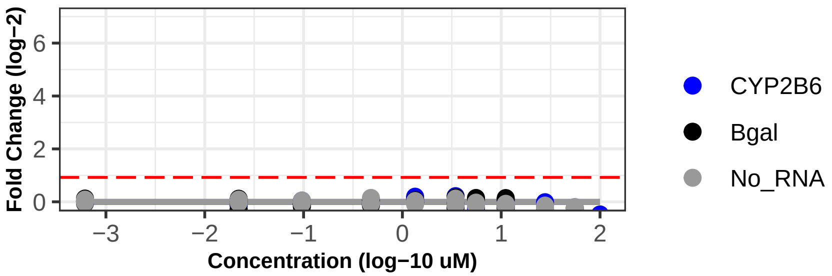


Diazinon: CYP2E1

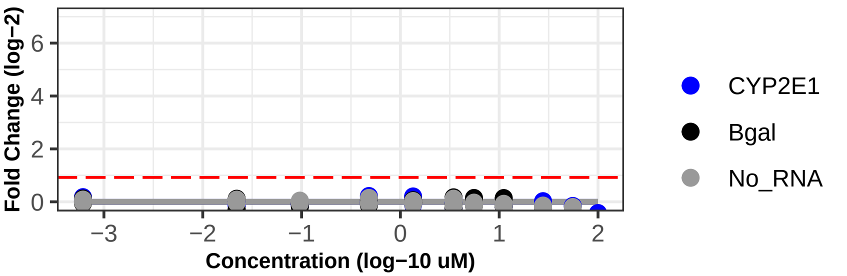


Diazinon: CYP2C8

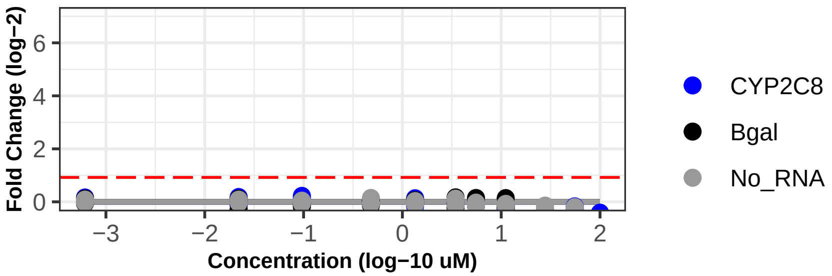


Diazinon: CYP2J2

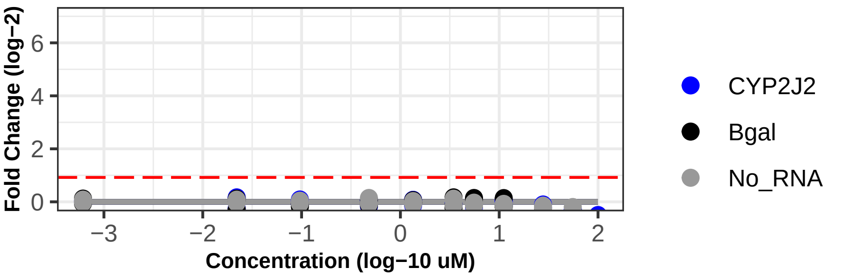


Diazinon: CYP2C9

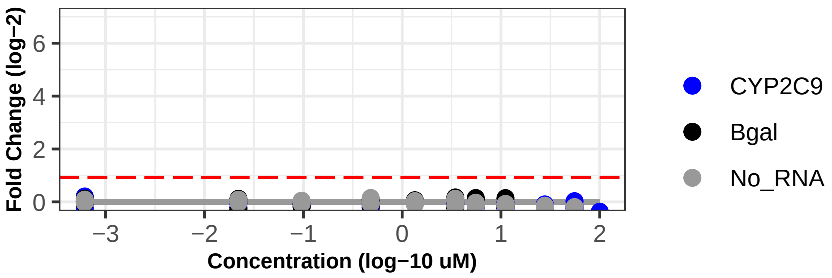


Diazinon: CYP3A4

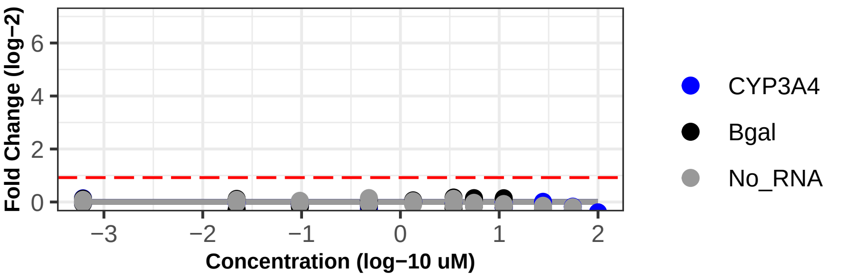

Dibutyl phthalate: CYP1A2

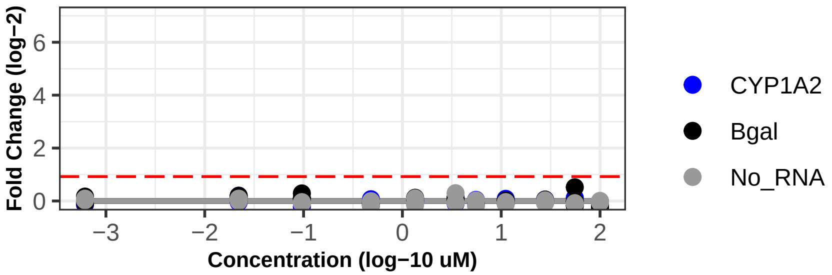


Dibutyl phthalate: CYP2C19

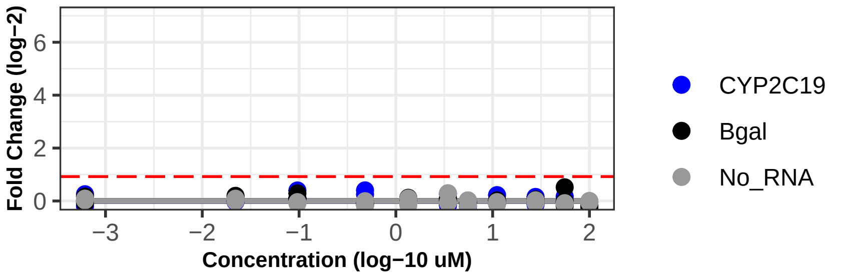


Dibutyl phthalate: CYP2A6

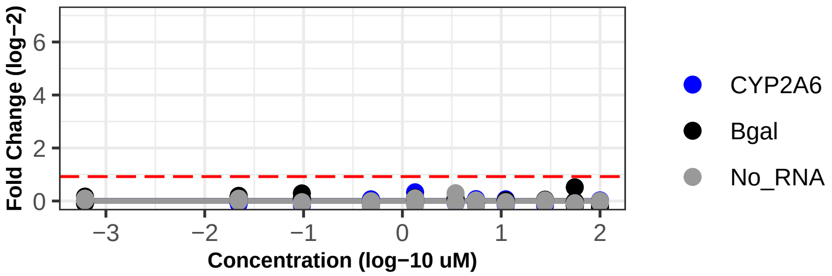


Dibutyl phthalate: CYP2D6

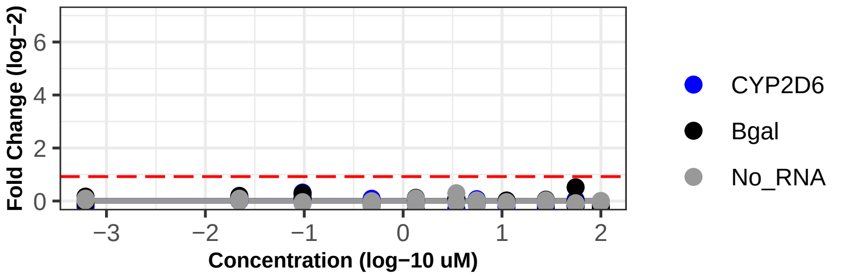


Dibutyl phthalate: CYP2B6

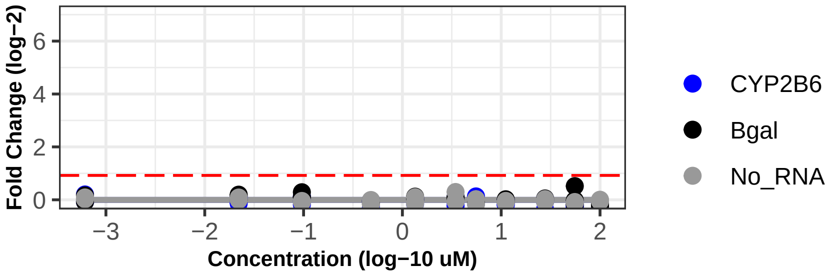


Dibutyl phthalate: CYP2E1

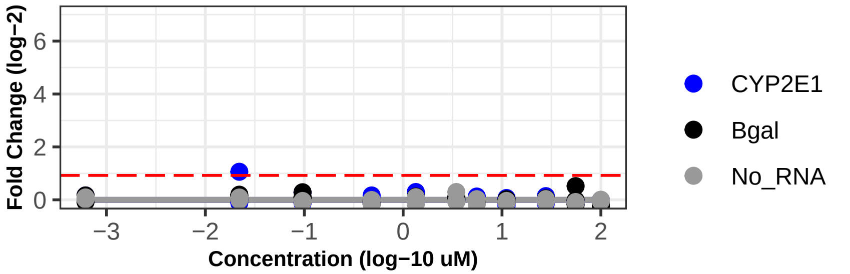


Dibutyl phthalate: CYP2C8

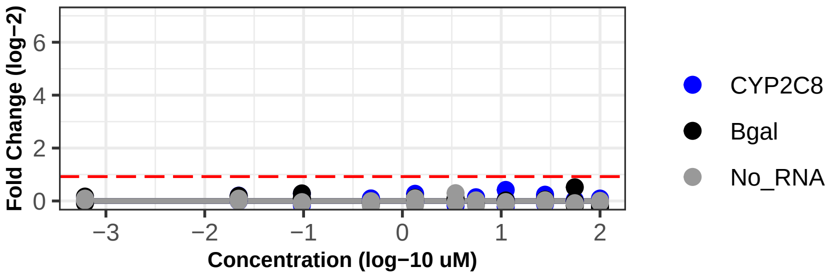


Dibutyl phthalate: CYP2J2

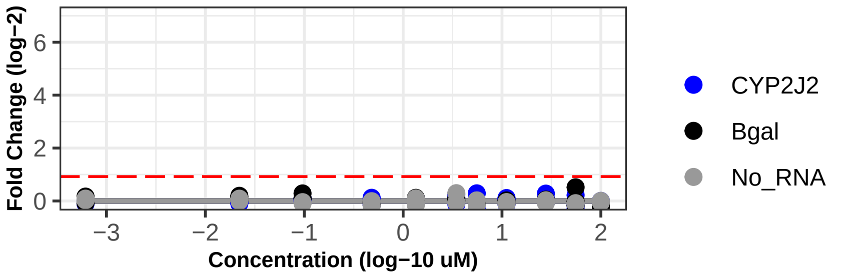


Dibutyl phthalate: CYP2C9

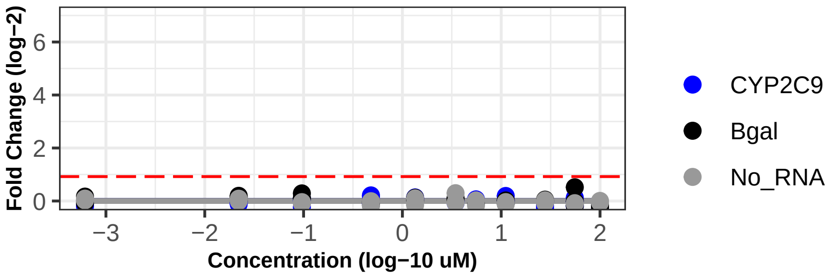


Dibutyl phthalate: CYP3A4

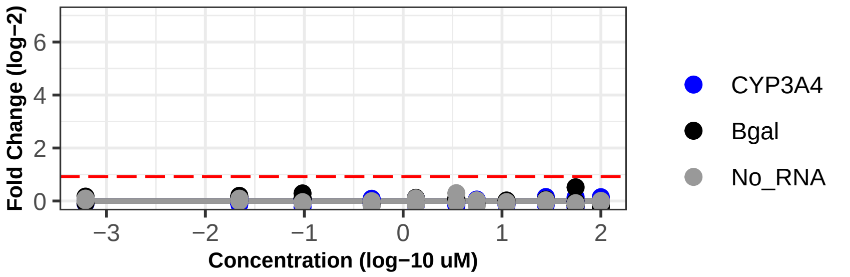

## Dichlobenil: CYP1A2

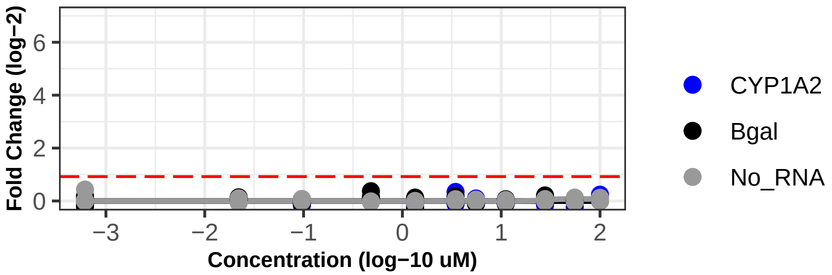


## Dichlobenil: CYP2C19

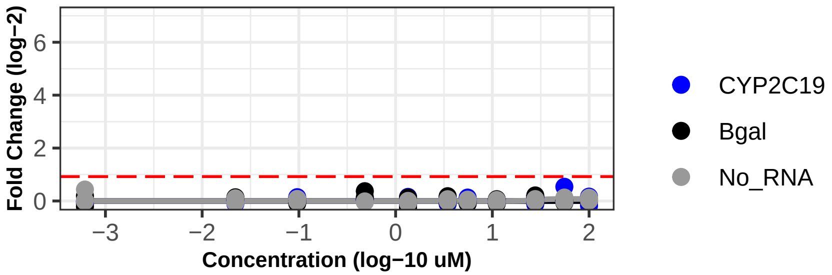


## Dichlobenil: CYP2A6

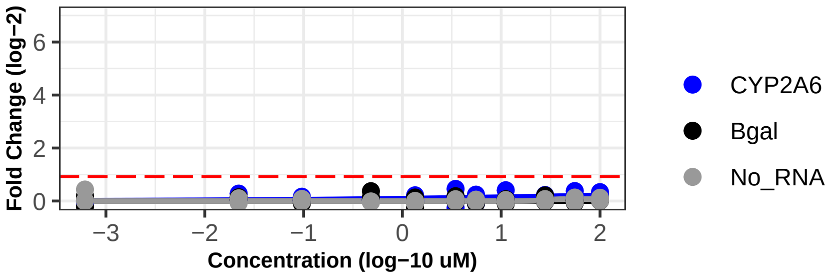


## Dichlobenil: CYP2D6

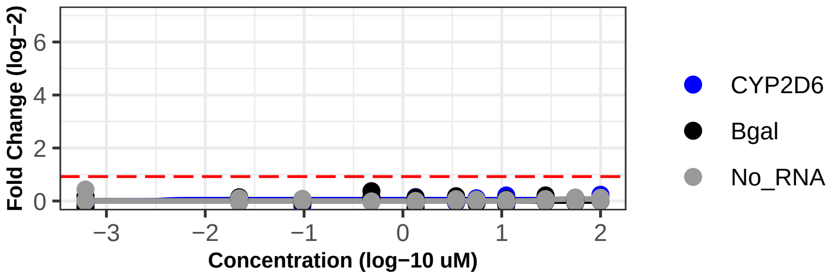


## Dichlobenil: CYP2B6

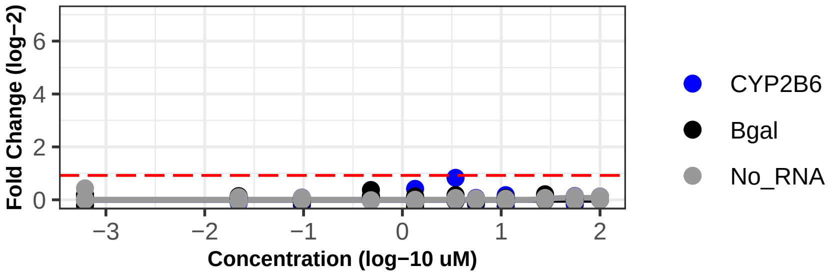


## Dichlobenil: CYP2E1

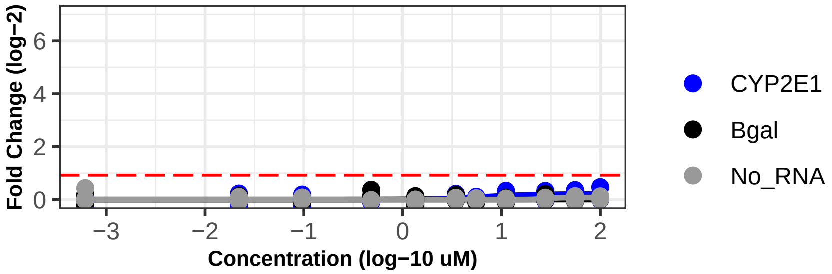


## Dichlobenil: CYP2C8

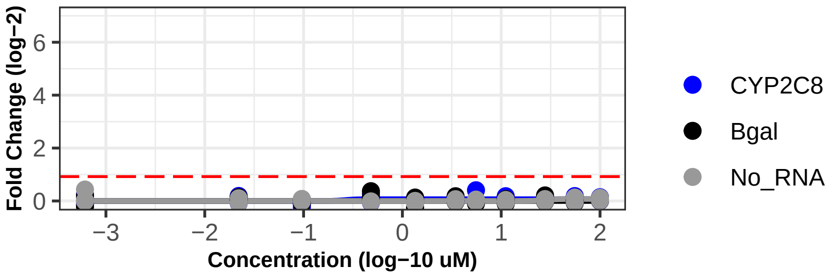


## Dichlobenil: CYP2J2

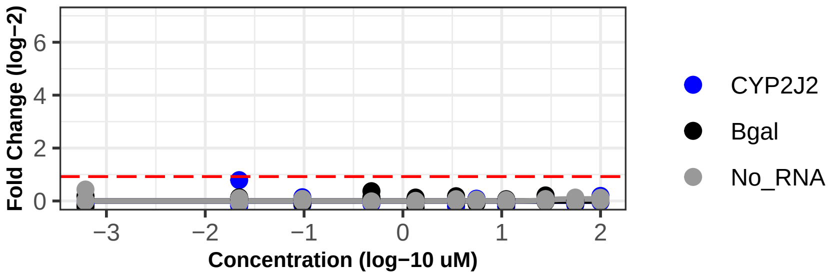


## Dichlobenil: CYP2C9

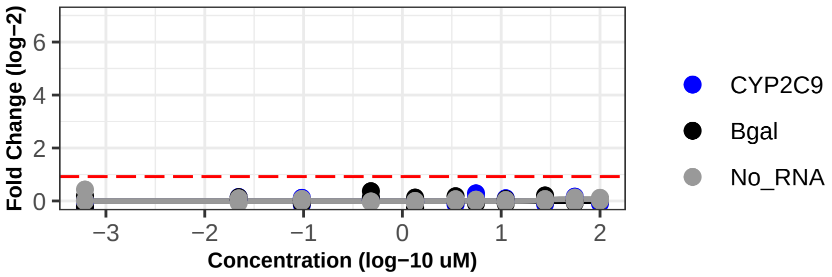


## Dichlobenil: CYP3A4

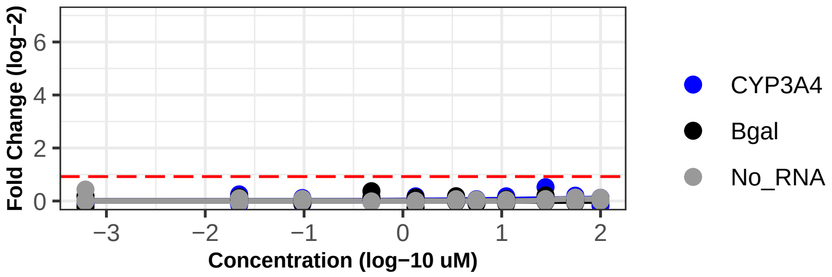

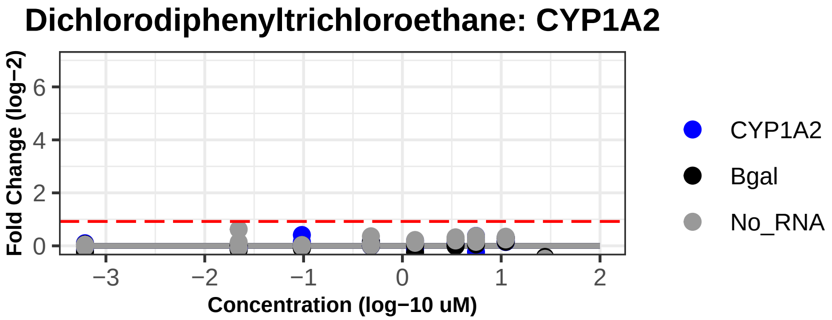
Dichlorodiphenyltrichloroethane: CYP1A2
Fold Change (log−2)
Concentration (log−10 uM)
CYP1A2
Bgal
No_RNA

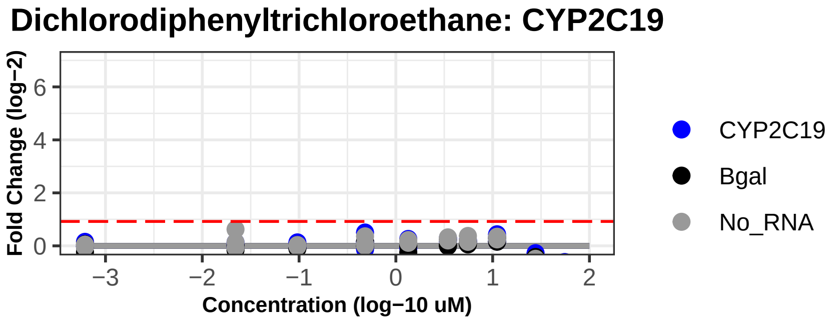
Dichlorodiphenyltrichloroethane: CYP2C19
Fold Change (log−2)
Concentration (log−10 uM)
CYP2C19
Bgal
No_RNA

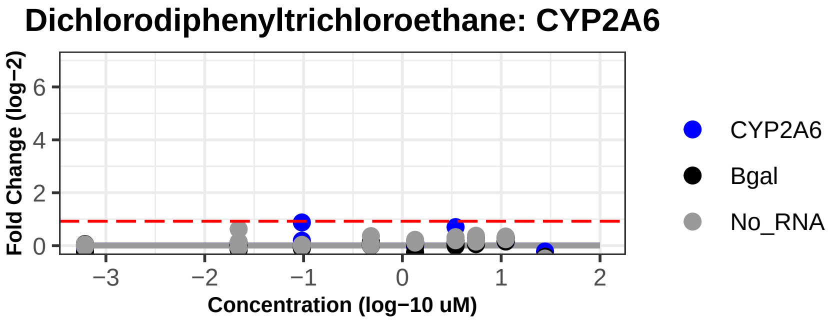
Dichlorodiphenyltrichloroethane: CYP2A6
Fold Change (log−2)
Concentration (log−10 uM)
CYP2A6
Bgal
No_RNA

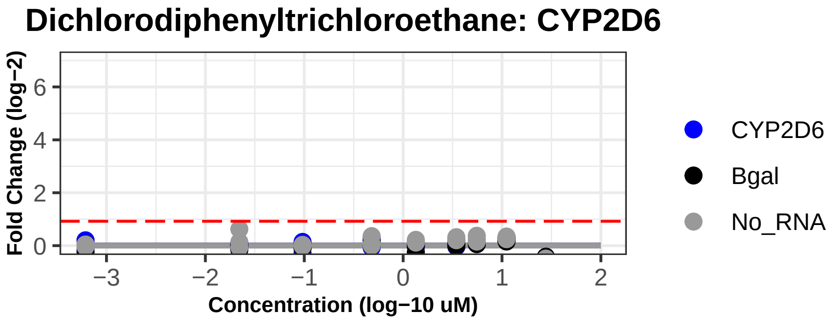
Dichlorodiphenyltrichloroethane: CYP2D6
Fold Change (log−2)
Concentration (log−10 uM)
CYP2D6
Bgal
No_RNA

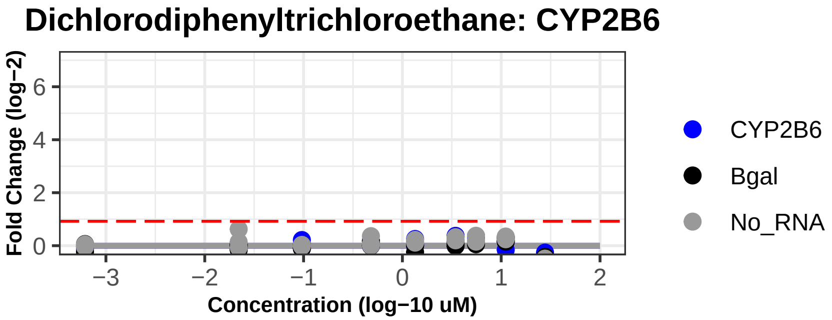
Dichlorodiphenyltrichloroethane: CYP2B6
Fold Change (log−2)
Concentration (log−10 uM)
CYP2B6
Bgal
No_RNA

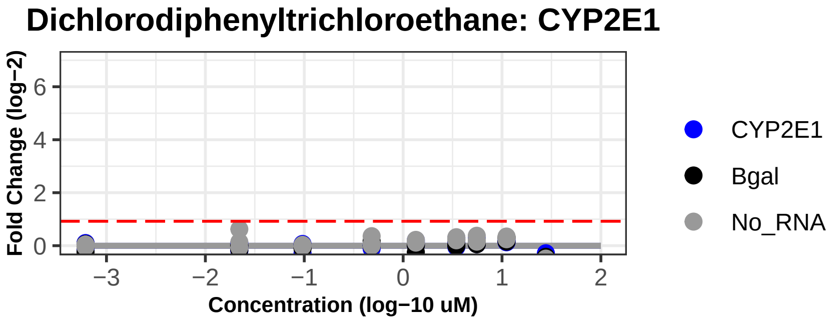
Dichlorodiphenyltrichloroethane: CYP2E1
Fold Change (log−2)
Concentration (log−10 uM)
CYP2E1
Bgal
No_RNA

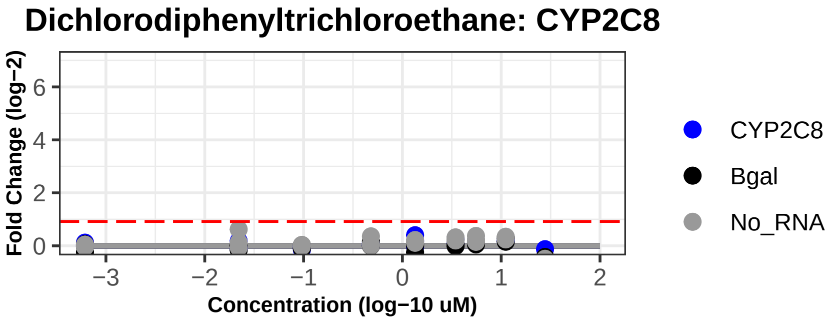
Dichlorodiphenyltrichloroethane: CYP2C8
Fold Change (log−2)
Concentration (log−10 uM)
CYP2C8
Bgal
No_RNA

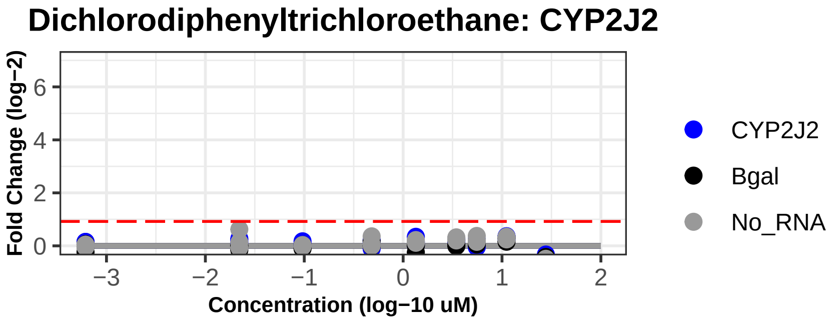
Dichlorodiphenyltrichloroethane: CYP2J2
Fold Change (log−2)
Concentration (log−10 uM)
CYP2J2
Bgal
No_RNA

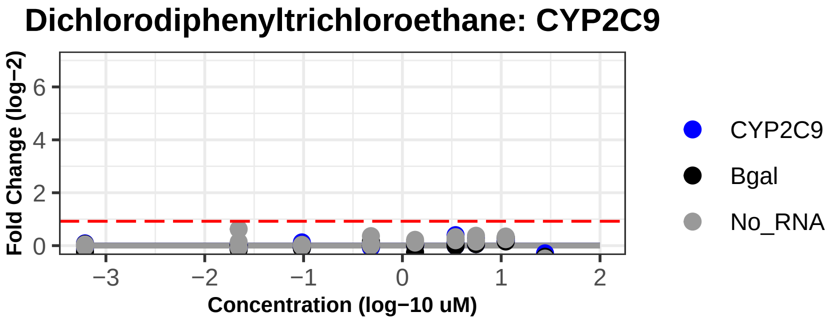
Dichlorodiphenyltrichloroethane: CYP2C9
Fold Change (log−2)
Concentration (log−10 uM)
CYP2C9
Bgal
No_RNA

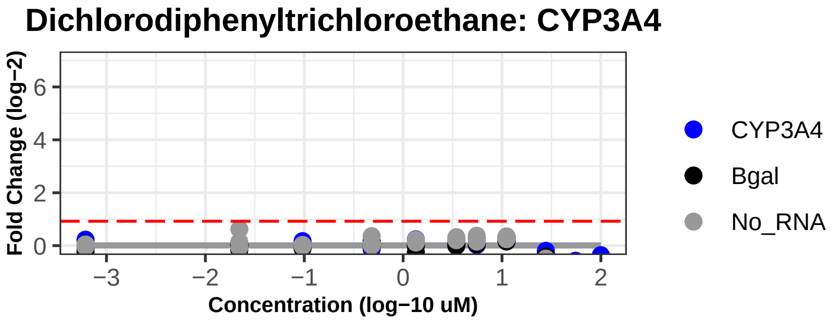
Dichlorodiphenyltrichloroethane: CYP3A4
Fold Change (log−2)
Concentration (log−10 uM)
CYP3A4
Bgal
No_RNA

## Dicyclohexyl phthalate: CYP1A2

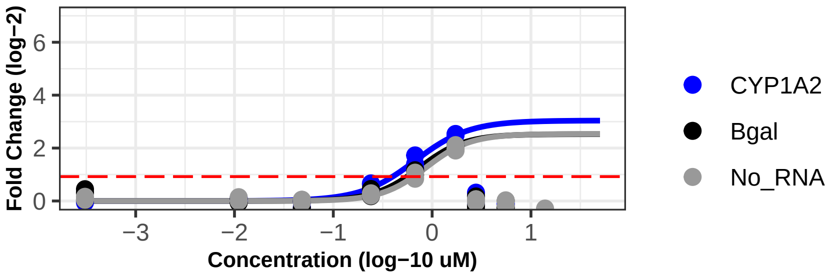


## Dicyclohexyl phthalate: CYP2C19

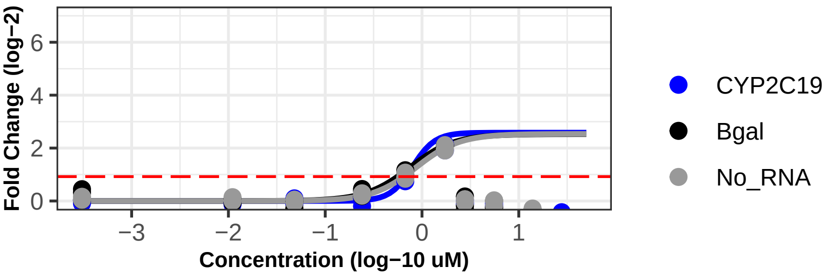


## Dicyclohexyl phthalate: CYP2A6

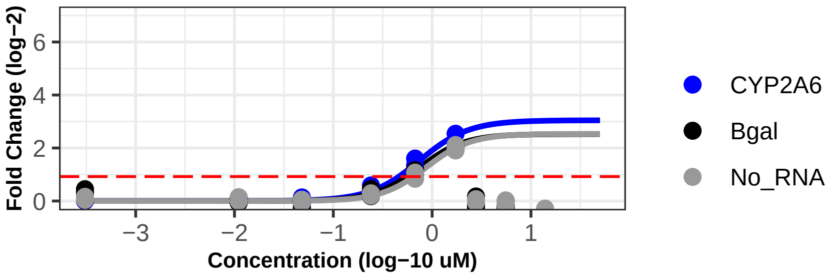


## Dicyclohexyl phthalate: CYP2D6

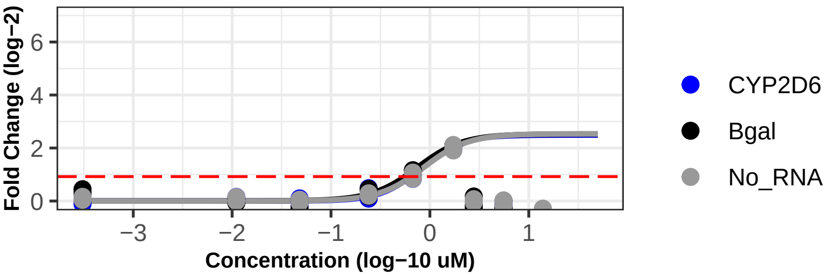


## Dicyclohexyl phthalate: CYP2B6

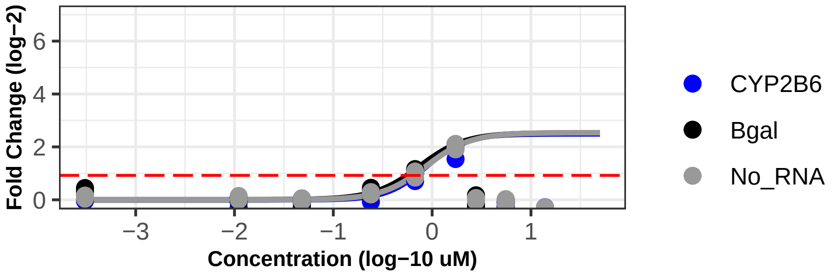


## Dicyclohexyl phthalate: CYP2E1

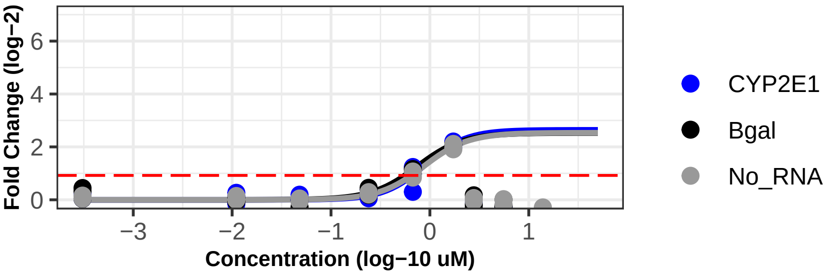


## Dicyclohexyl phthalate: CYP2C8

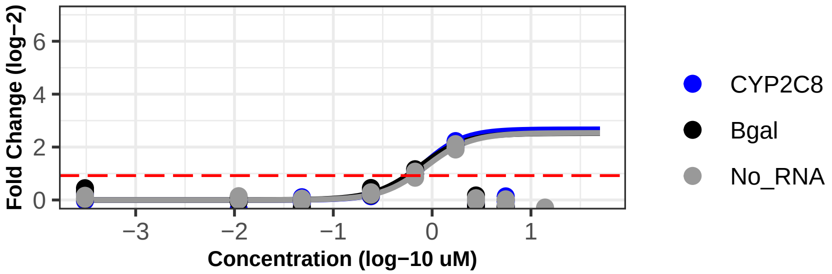


## Dicyclohexyl phthalate: CYP2J2

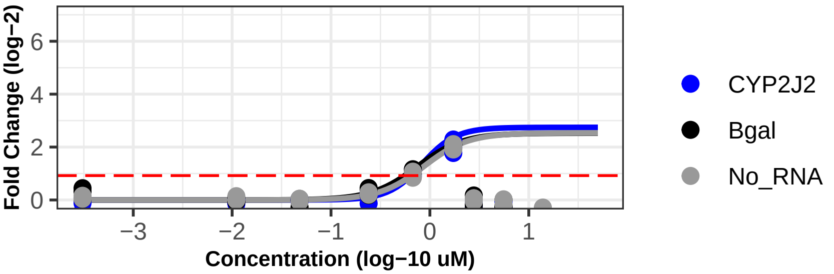


## Dicyclohexyl phthalate: CYP2C9

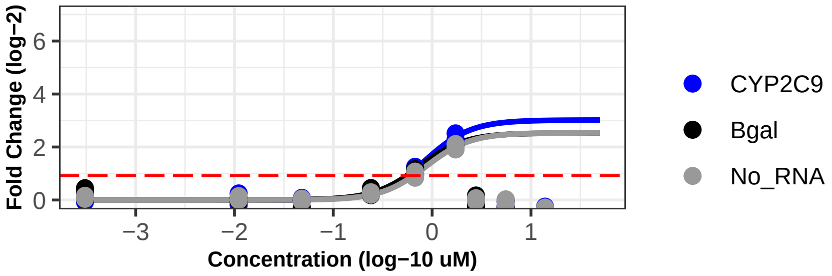


## Dicyclohexyl phthalate: CYP3A4

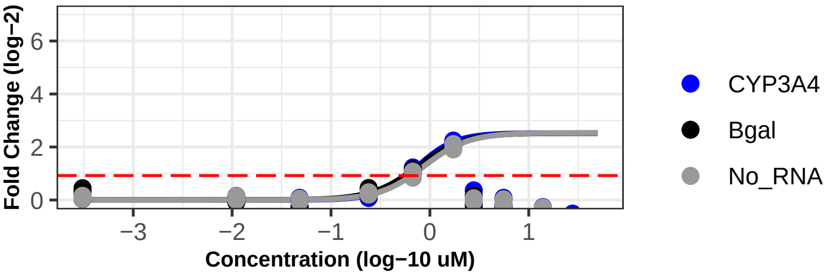

## Diethyl phthalate: CYP1A2

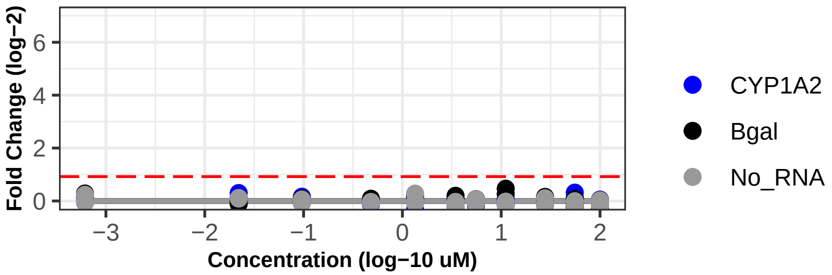


## Diethyl phthalate: CYP2C19

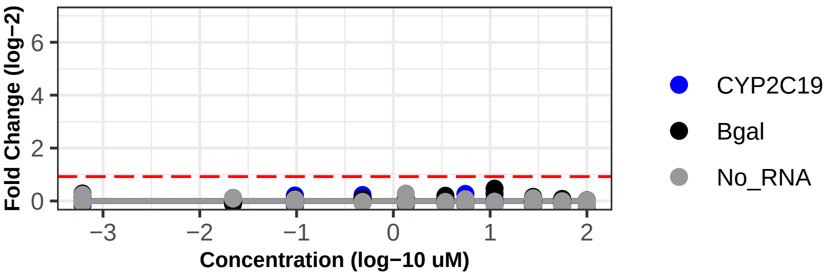


## Diethyl phthalate: CYP2A6

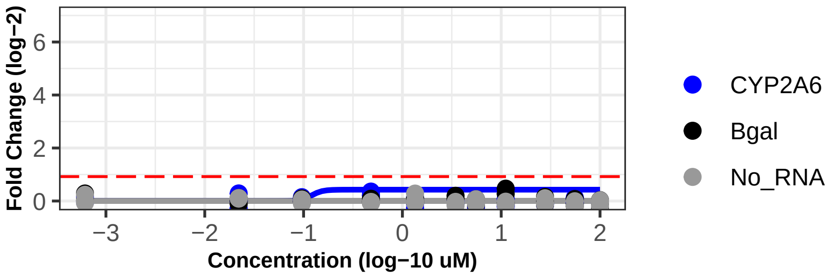


## Diethyl phthalate: CYP2D6

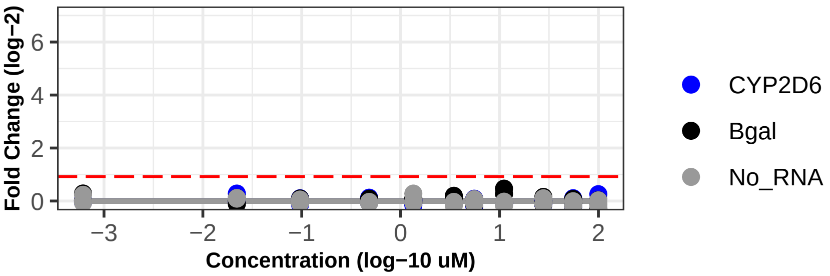


## Diethyl phthalate: CYP2B6

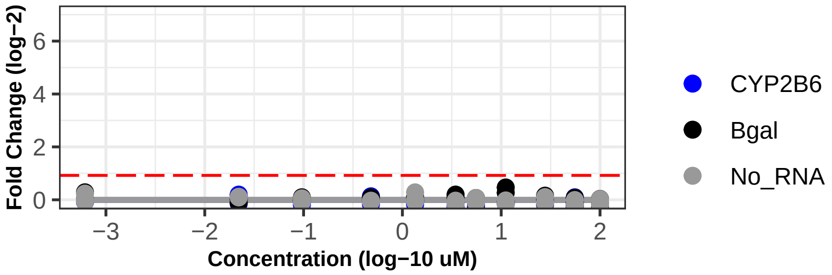


## Diethyl phthalate: CYP2E1

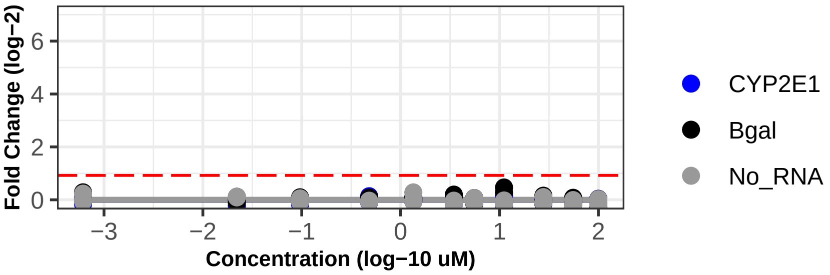


## Diethyl phthalate: CYP2C8

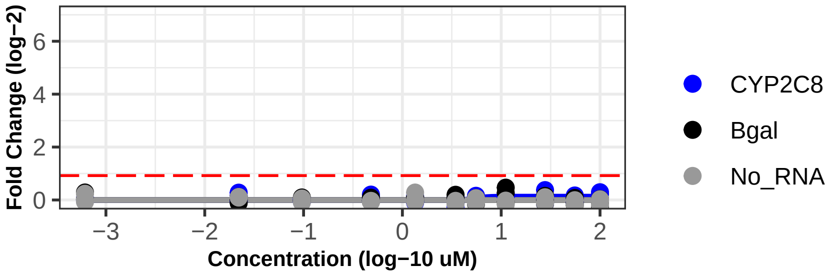


## Diethyl phthalate: CYP2J2

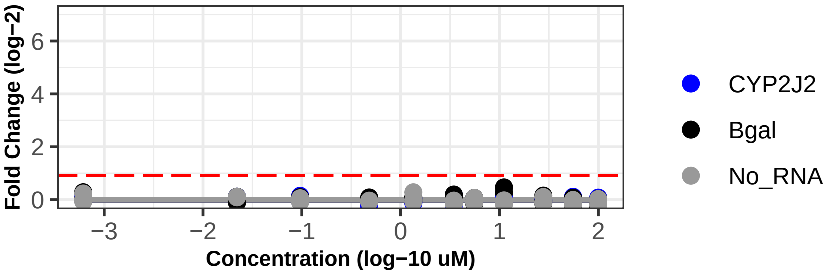


## Diethyl phthalate: CYP2C9

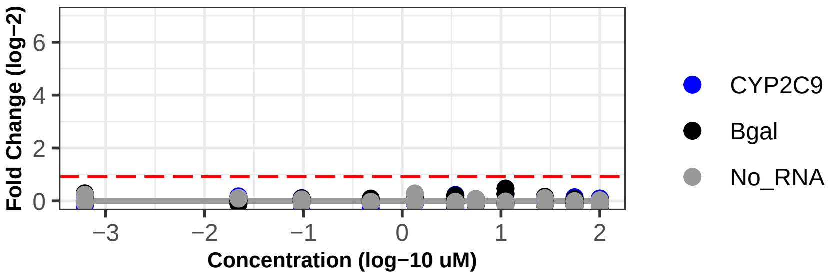


## Diethyl phthalate: CYP3A4

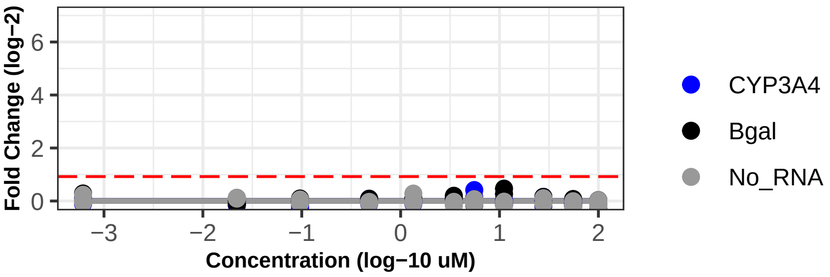

## Diethylstilbestrol: CYP1A2

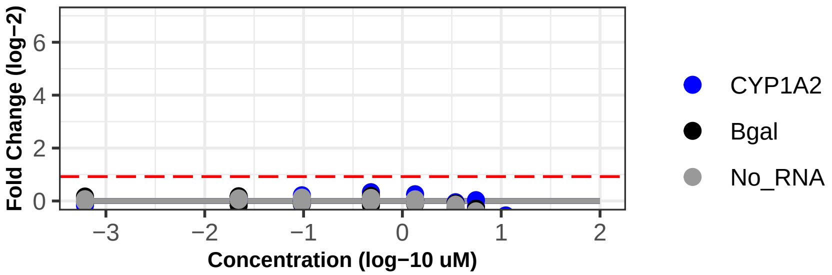


## Diethylstilbestrol: CYP2C19

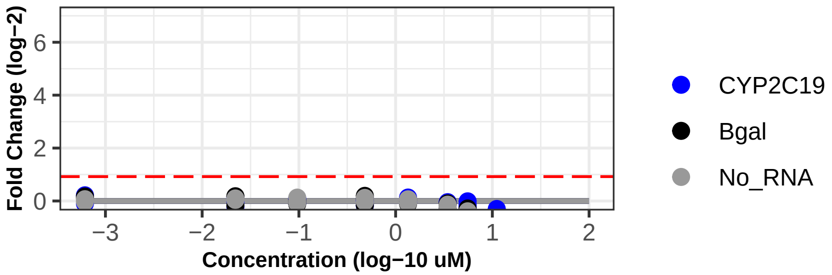


## Diethylstilbestrol: CYP2A6

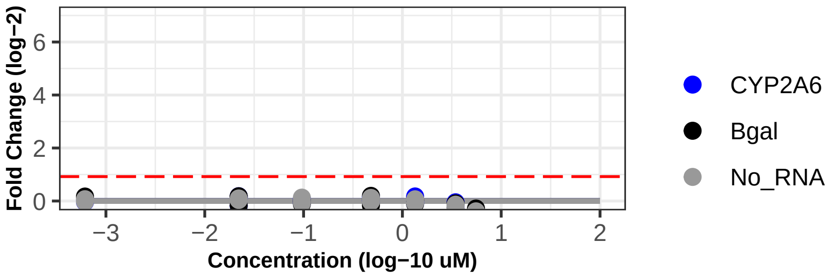


## Diethylstilbestrol: CYP2D6

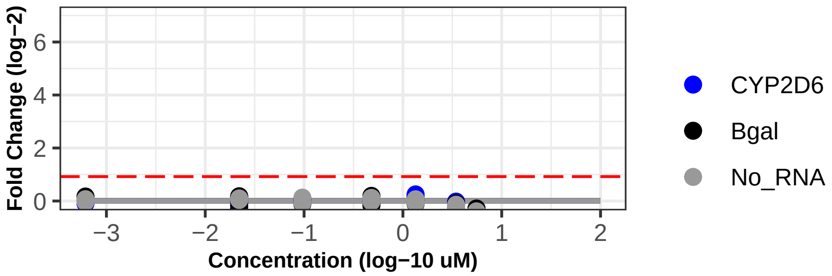


## Diethylstilbestrol: CYP2B6

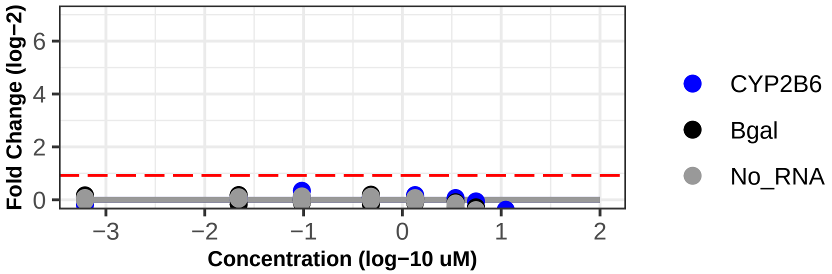


## Diethylstilbestrol: CYP2E1

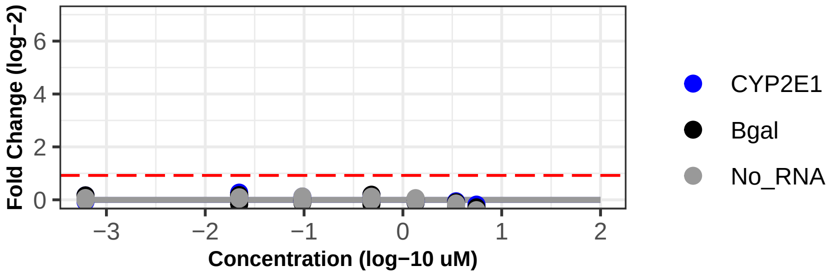


## Diethylstilbestrol: CYP2C8

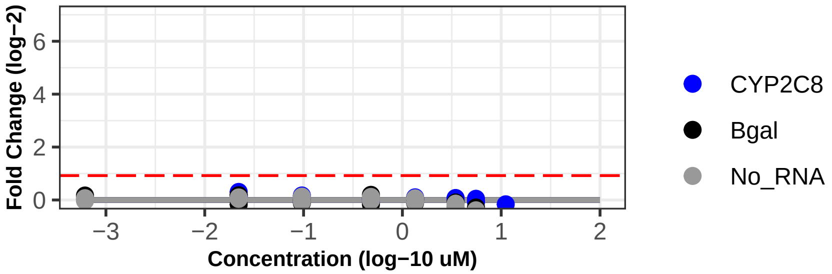


## Diethylstilbestrol: CYP2J2

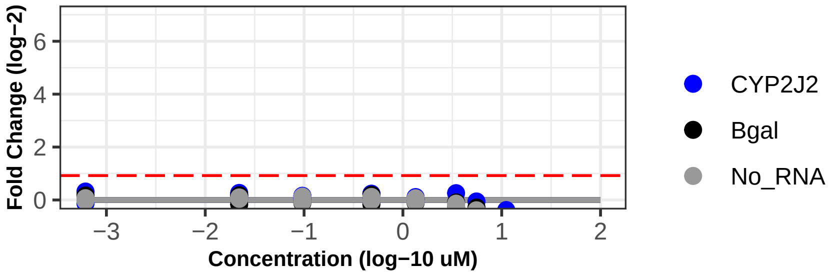


## Diethylstilbestrol: CYP2C9

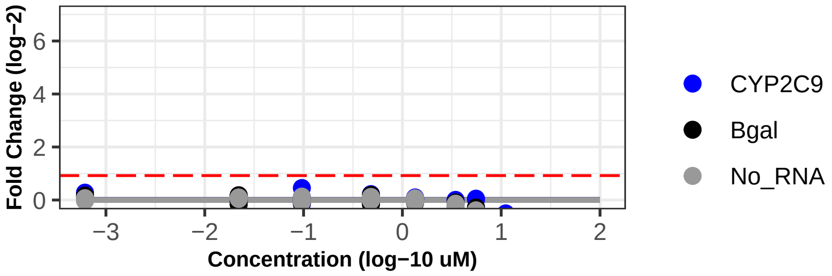


## Diethylstilbestrol: CYP3A4

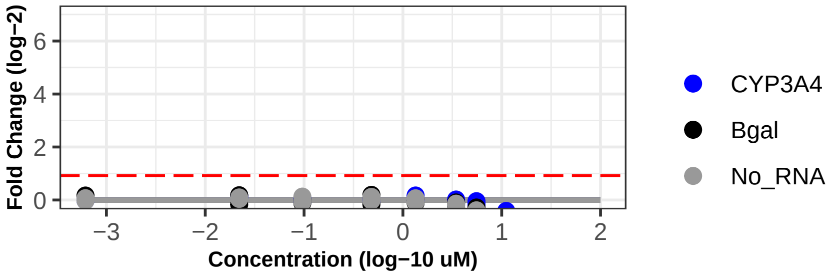

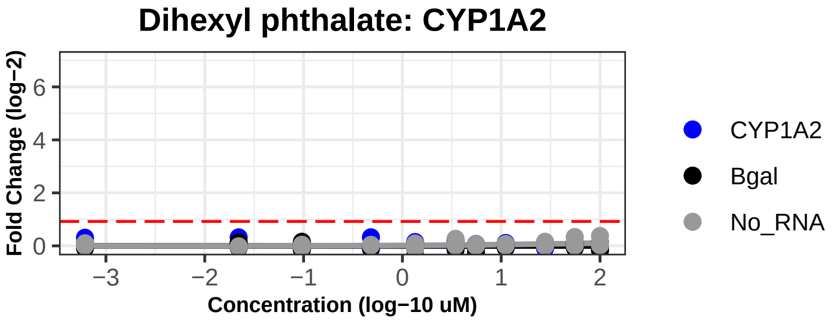
Dihexyl phthalate: CYP1A2
Fold Change (log−2)
Concentration (log−10 uM)
CYP1A2
Bgal
No_RNA

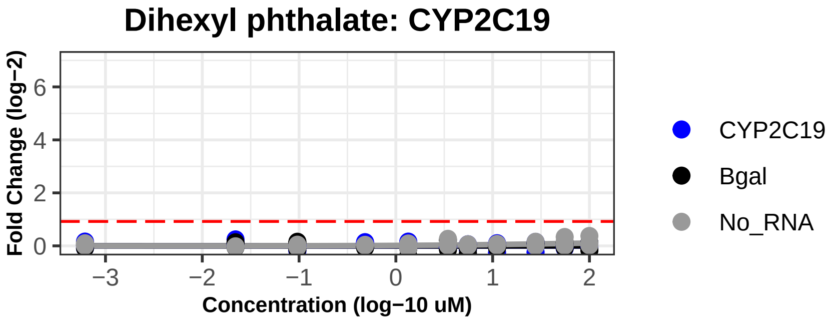
Dihexyl phthalate: CYP2C19
Fold Change (log−2)
Concentration (log−10 uM)
CYP2C19
Bgal
No_RNA

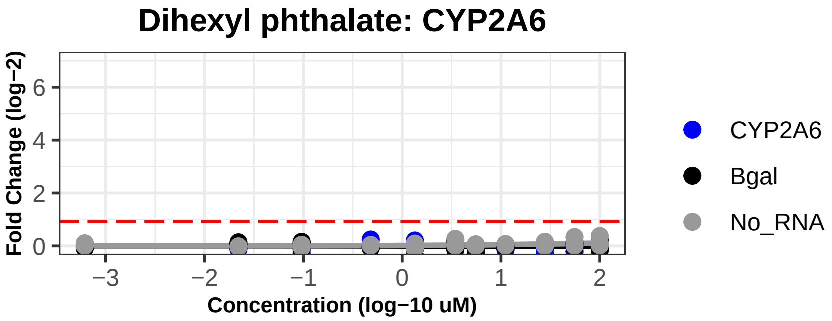
Dihexyl phthalate: CYP2A6
Fold Change (log−2)
Concentration (log−10 uM)
CYP2A6
Bgal
No_RNA

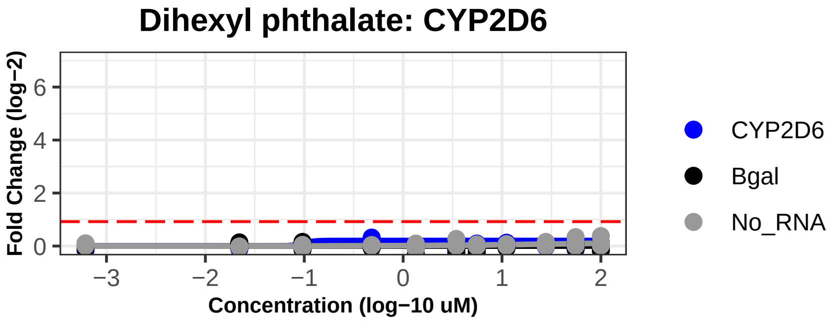
Dihexyl phthalate: CYP2D6
Fold Change (log−2)
Concentration (log−10 uM)
CYP2D6
Bgal
No_RNA

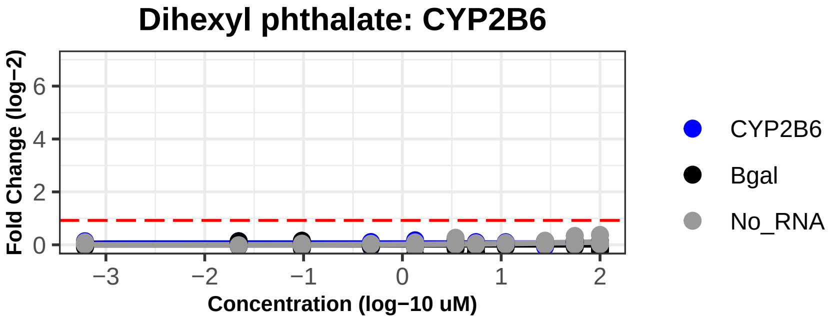
Dihexyl phthalate: CYP2B6
Fold Change (log−2)
Concentration (log−10 uM)
CYP2B6
Bgal
No_RNA

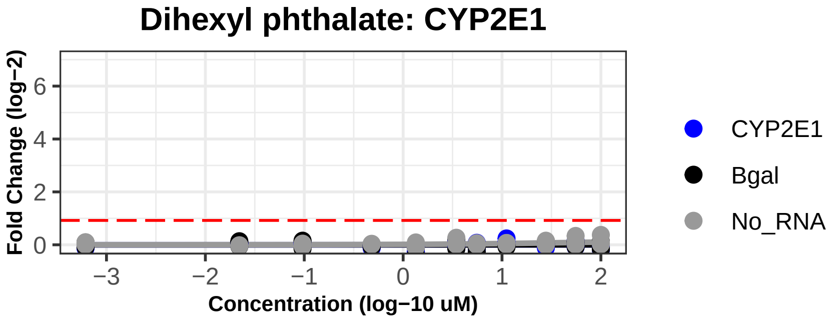
Dihexyl phthalate: CYP2E1
Fold Change (log−2)
Concentration (log−10 uM)
CYP2E1
Bgal
No_RNA

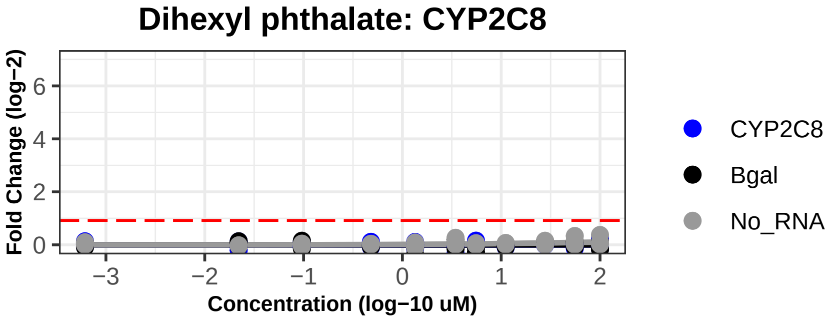
Dihexyl phthalate: CYP2C8
Fold Change (log−2)
Concentration (log−10 uM)
CYP2C8
Bgal
No_RNA

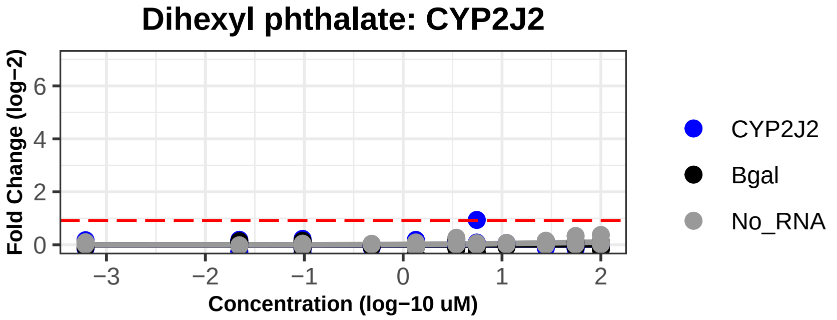
Dihexyl phthalate: CYP2J2
Fold Change (log−2)
Concentration (log−10 uM)
CYP2J2
Bgal
No_RNA

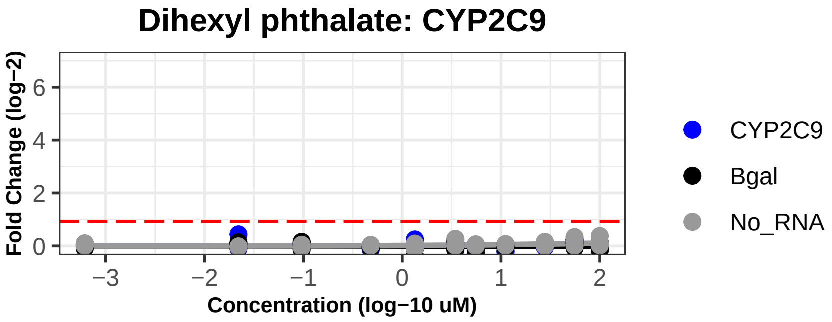
Dihexyl phthalate: CYP2C9
Fold Change (log−2)
Concentration (log−10 uM)
CYP2C9
Bgal
No_RNA

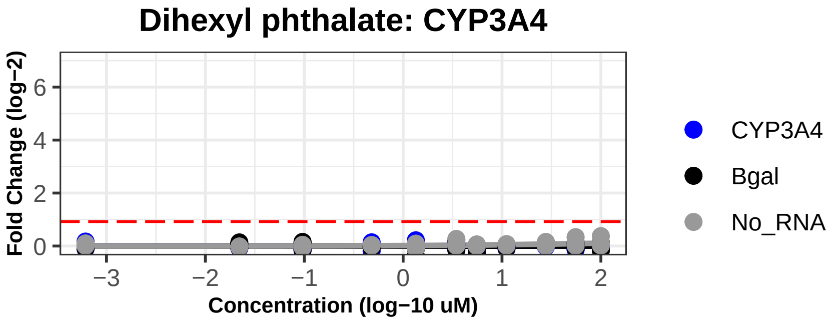
Dihexyl phthalate: CYP3A4
Fold Change (log−2)
Concentration (log−10 uM)
CYP3A4
Bgal
No_RNA

## Dipentyl phthalate: CYP1A2

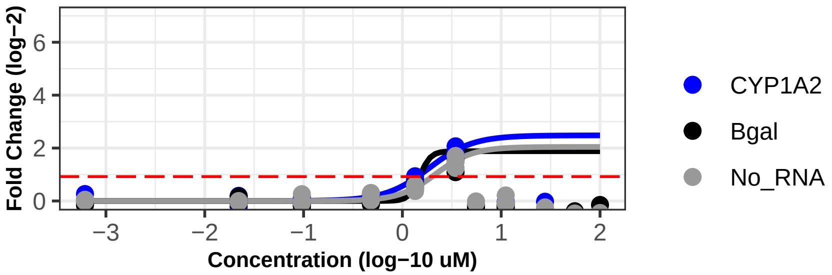


## Dipentyl phthalate: CYP2C19

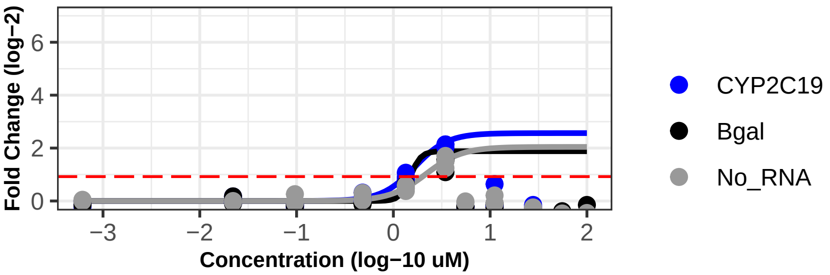


## Dipentyl phthalate: CYP2A6

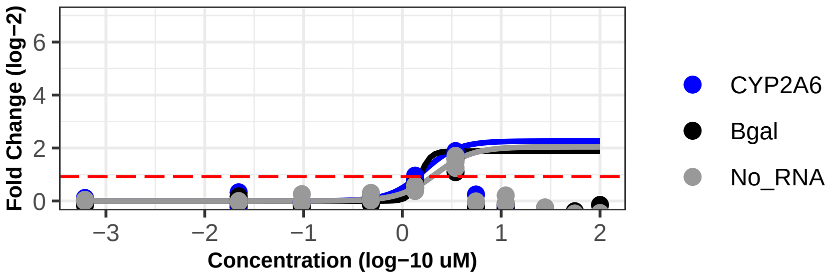


## Dipentyl phthalate: CYP2D6

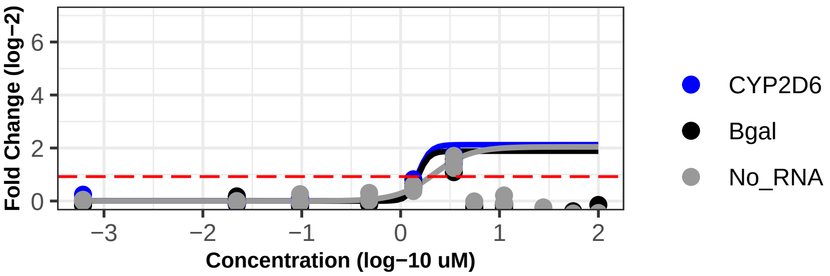


## Dipentyl phthalate: CYP2B6

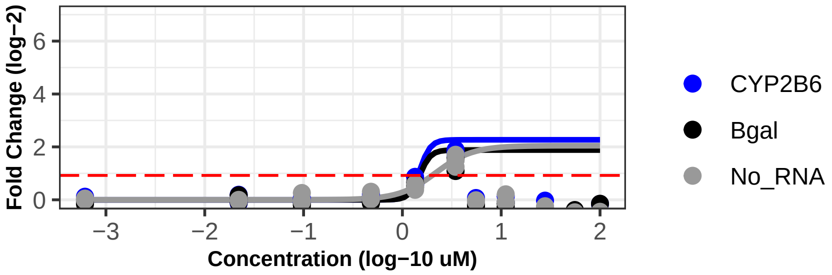


## Dipentyl phthalate: CYP2E1

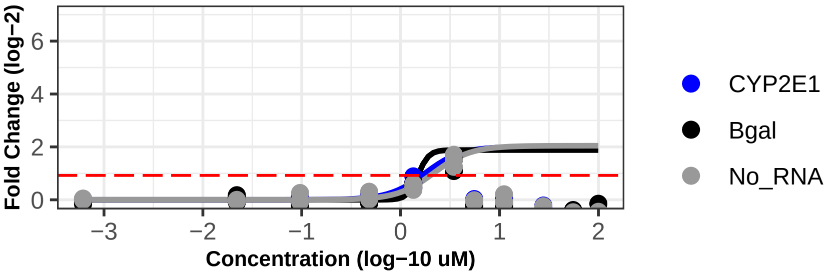


## Dipentyl phthalate: CYP2C8

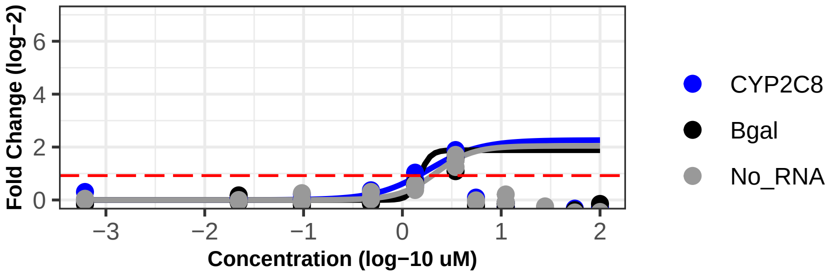


## Dipentyl phthalate: CYP2J2

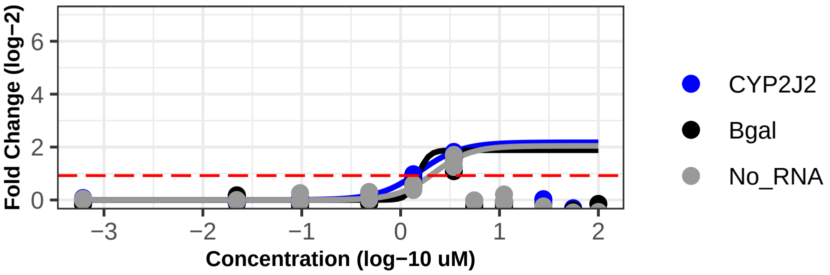


## Dipentyl phthalate: CYP2C9

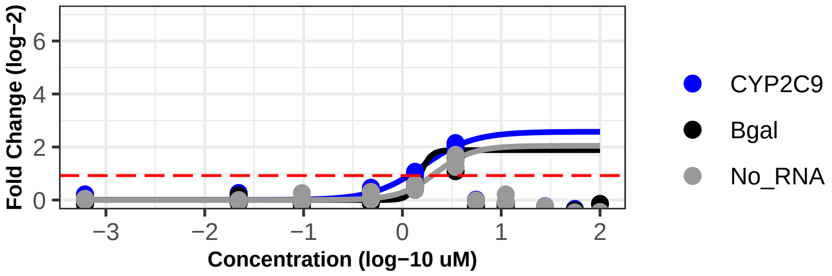


## Dipentyl phthalate: CYP3A4

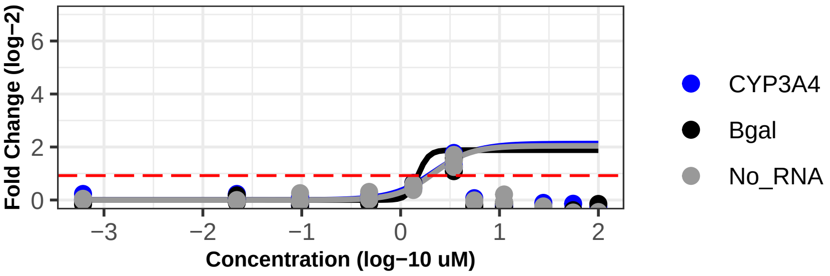

### Enclomiphene hydrochloride: CYP1A2

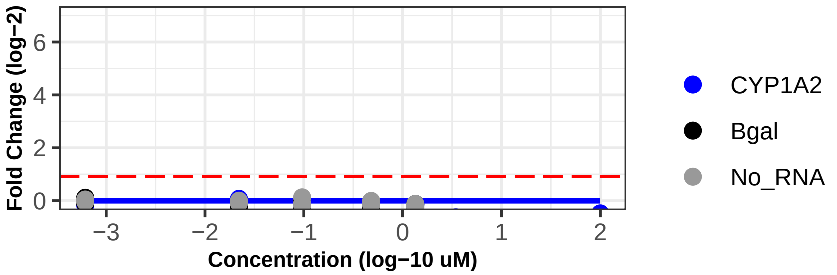


### Enclomiphene hydrochloride: CYP2C19

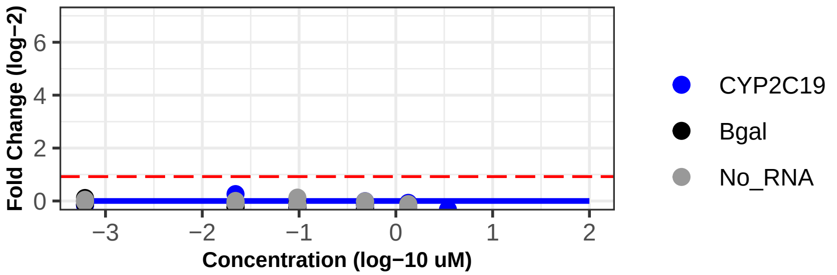


### Enclomiphene hydrochloride: CYP2A6

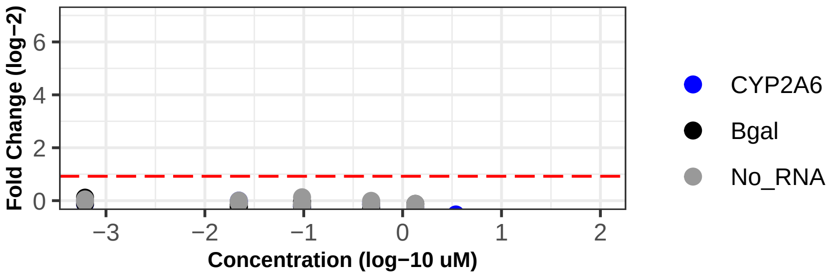


### Enclomiphene hydrochloride: CYP2D6

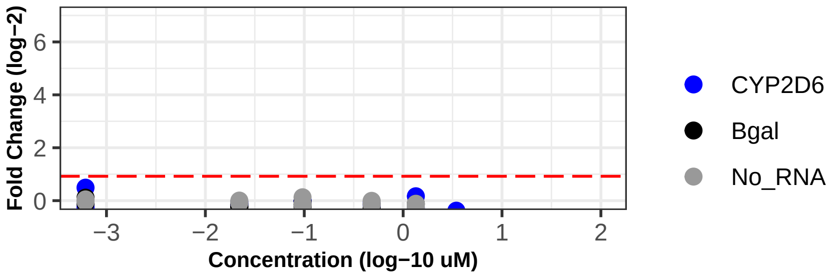


### Enclomiphene hydrochloride: CYP2B6

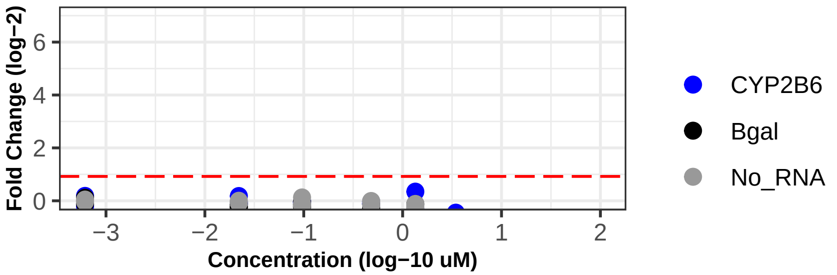


### Enclomiphene hydrochloride: CYP2E1

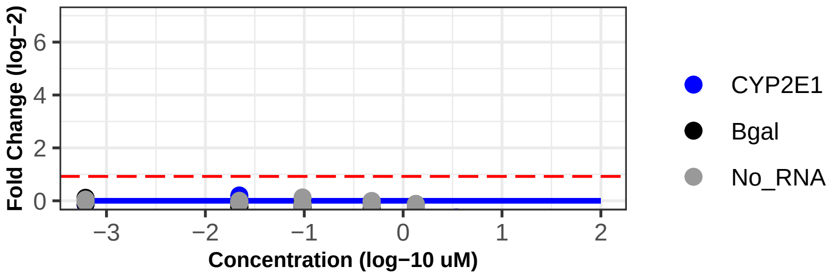


### Enclomiphene hydrochloride: CYP2C8

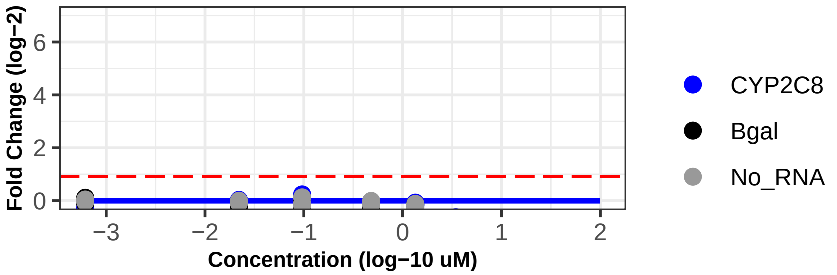


### Enclomiphene hydrochloride: CYP2J2

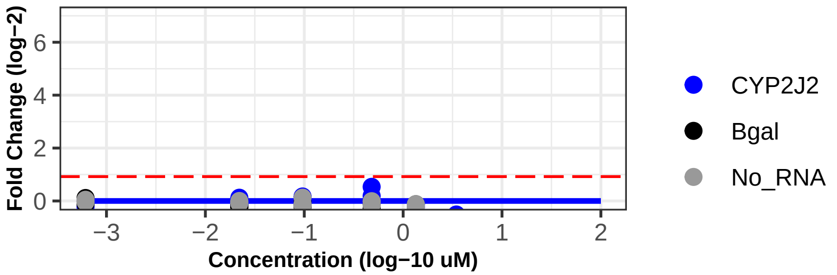


### Enclomiphene hydrochloride: CYP2C9

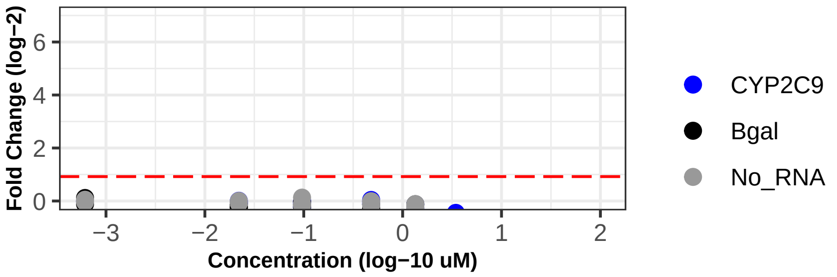


### Enclomiphene hydrochloride: CYP3A4

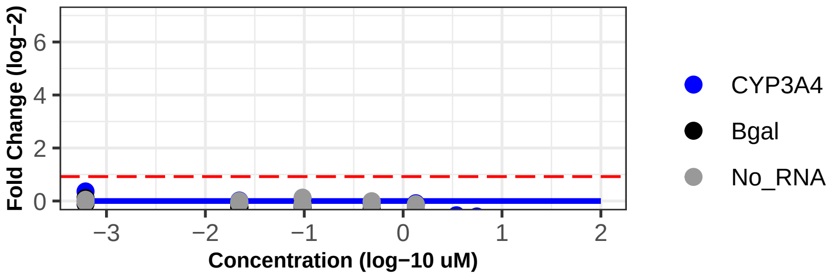

### Equilin: CYP1A2

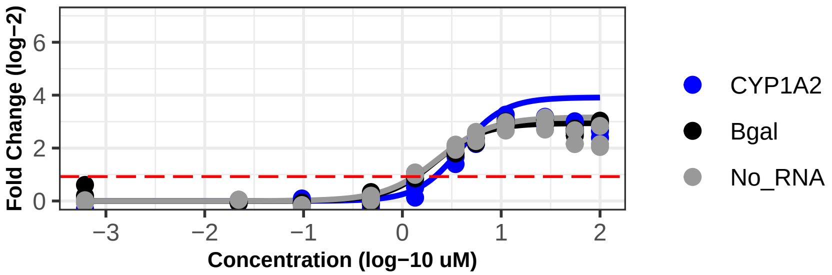


### Equilin: CYP2C19

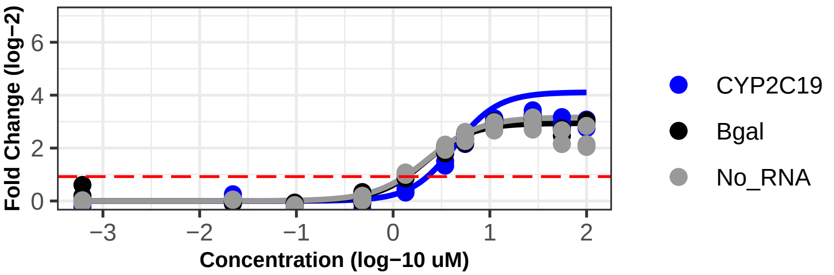


### Equilin: CYP2A6

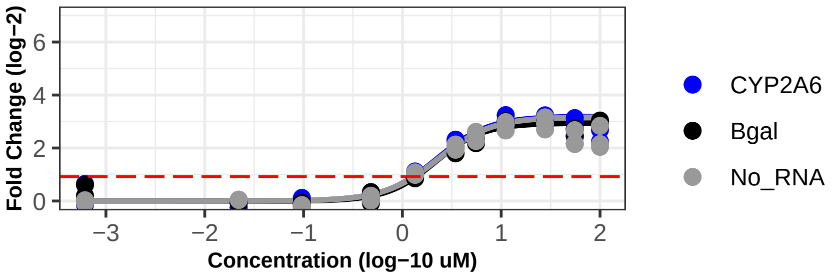


### Equilin: CYP2D6

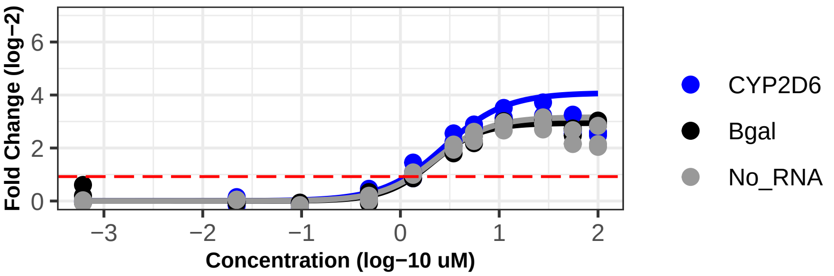


### Equilin: CYP2B6

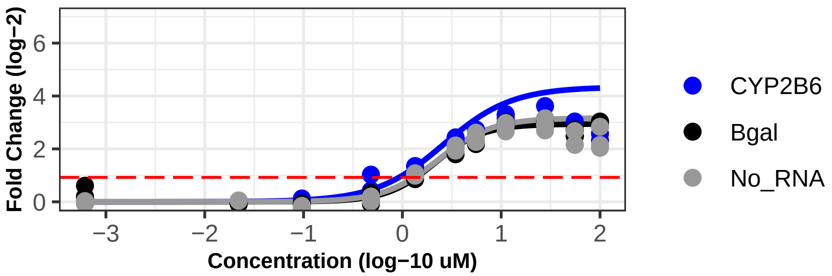


### Equilin: CYP2E1

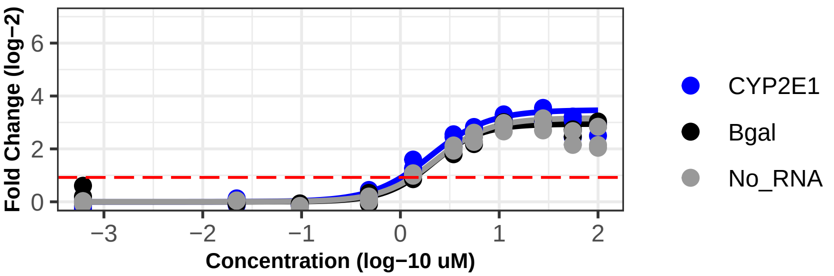


### Equilin: CYP2C8

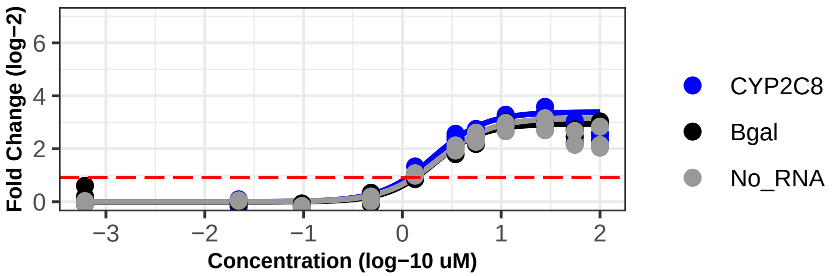


### Equilin: CYP2J2

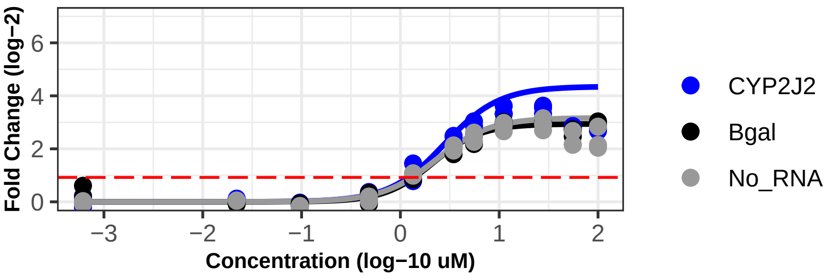


### Equilin: CYP2C9

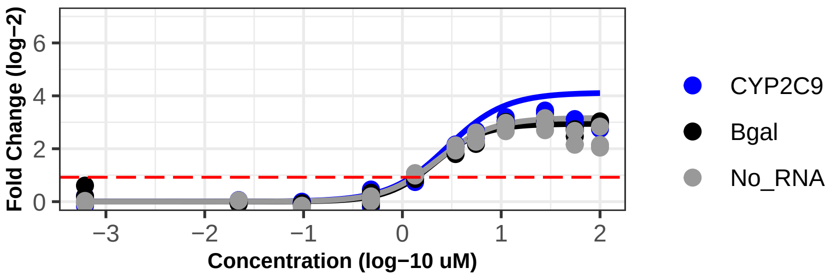


### Equilin: CYP3A4

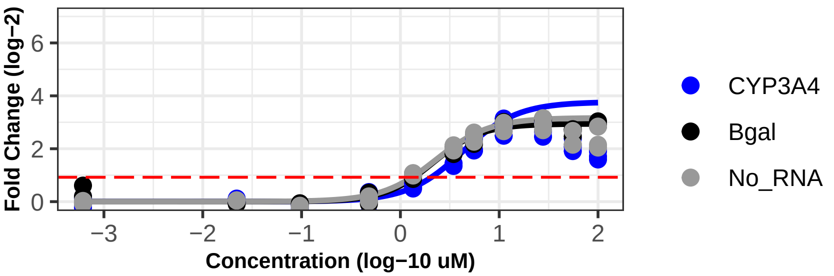

Esfenvalerate: CYP1A2

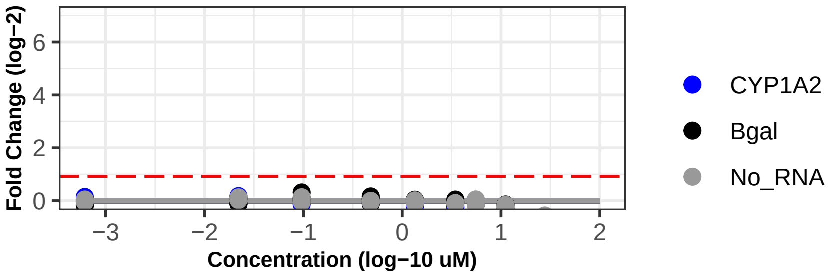


Esfenvalerate: CYP2C19

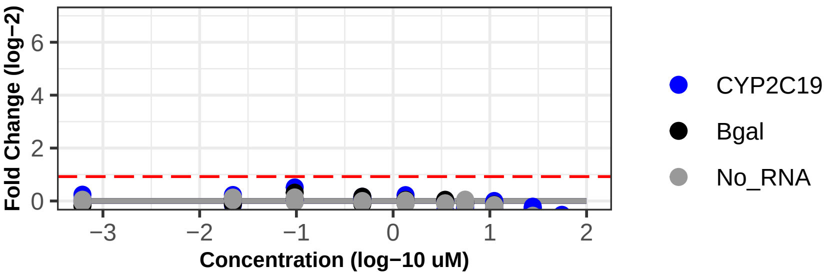


Esfenvalerate: CYP2A6

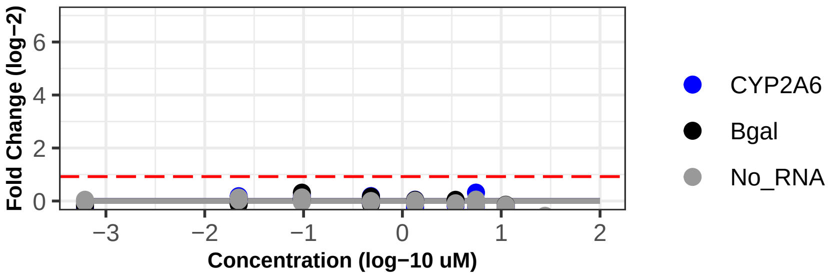


Esfenvalerate: CYP2D6

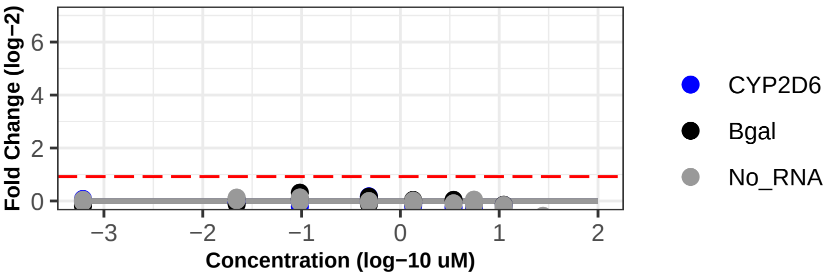


Esfenvalerate: CYP2B6

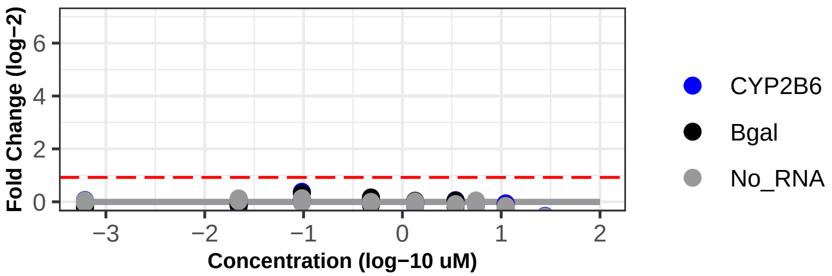


Esfenvalerate: CYP2E1

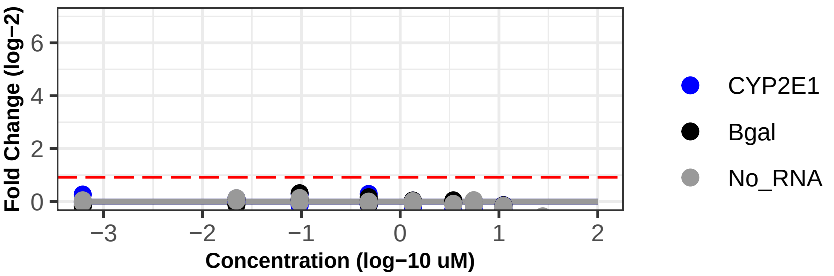


Esfenvalerate: CYP2C8

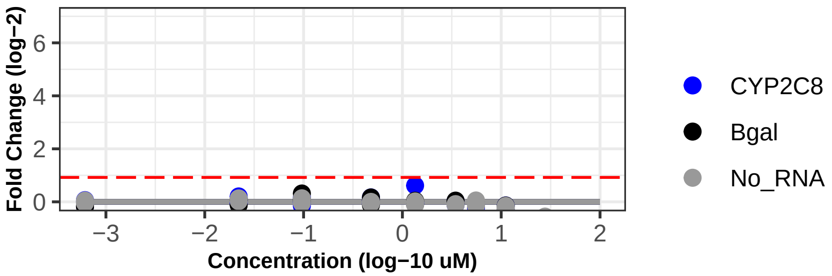


Esfenvalerate: CYP2J2

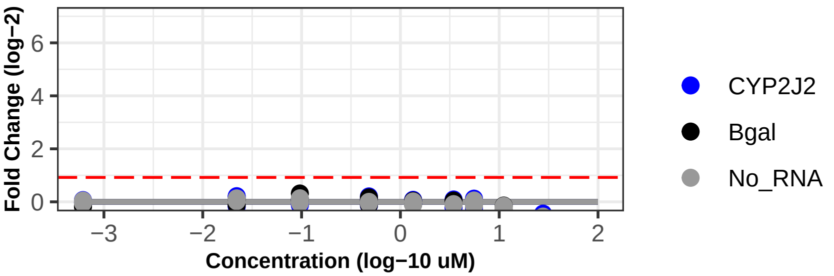


Esfenvalerate: CYP2C9

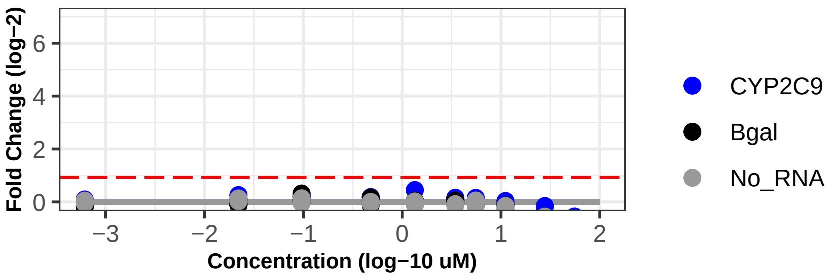


Esfenvalerate: CYP3A4

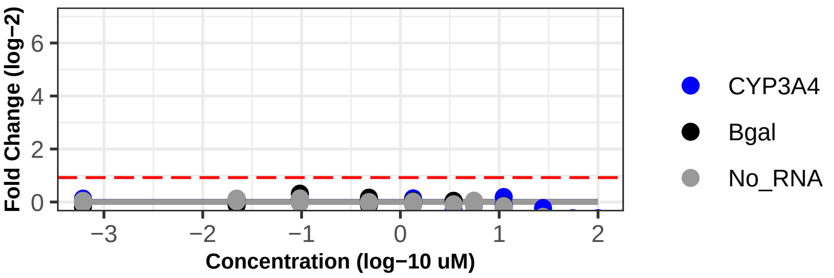

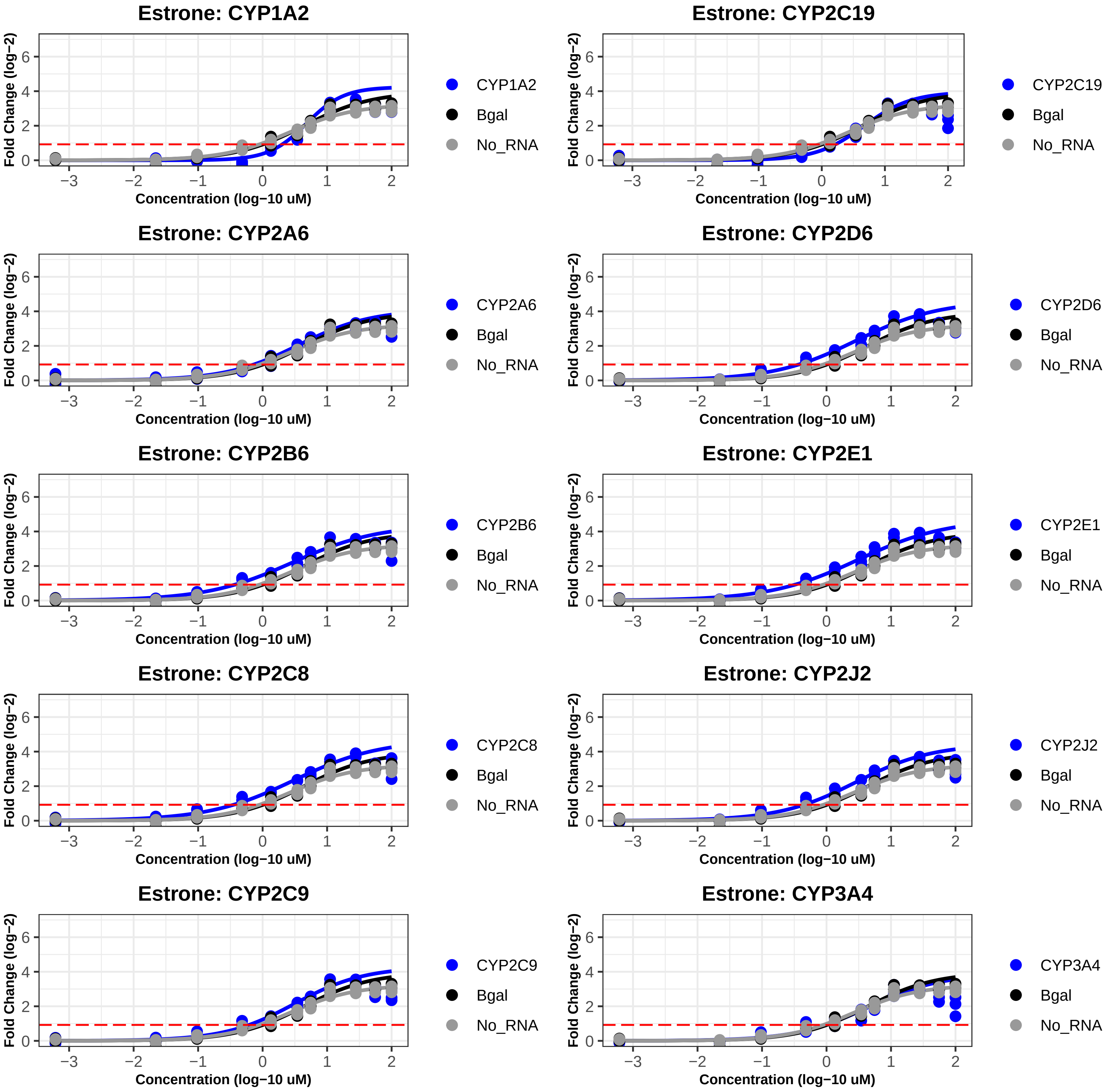
Estrone: CYP1A2
CYP1A2
Bgal
No_RNA
Estrone: CYP2C19
CYP2C19
Bgal
No_RNA
Estrone: CYP2A6
CYP2A6
Bgal
No_RNA
Estrone: CYP2D6
CYP2D6
Bgal
No_RNA
Estrone: CYP2B6
CYP2B6
Bgal
No_RNA
Estrone: CYP2E1
CYP2E1
Bgal
No_RNA
Estrone: CYP2C8
CYP2C8
Bgal
No_RNA
Estrone: CYP2J2
CYP2J2
Bgal
No_RNA
Estrone: CYP2C9
CYP2C9
Bgal
No_RNA
Estrone: CYP3A4
CYP3A4
Bgal
No_RNA
Fold Change (log−2)
Concentration (log−10 uM)

Ethoprop: CYP1A2

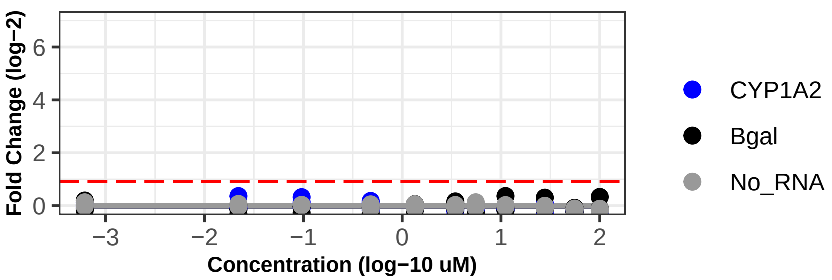


Ethoprop: CYP2C19

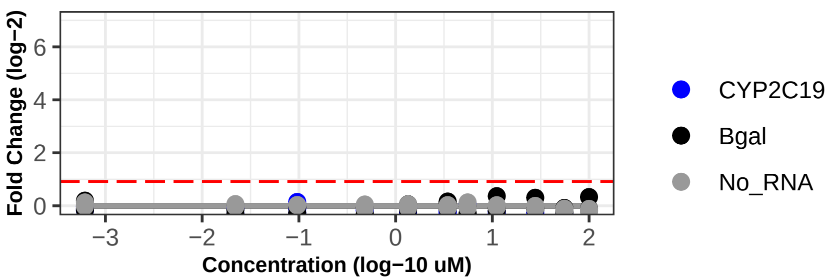


Ethoprop: CYP2A6

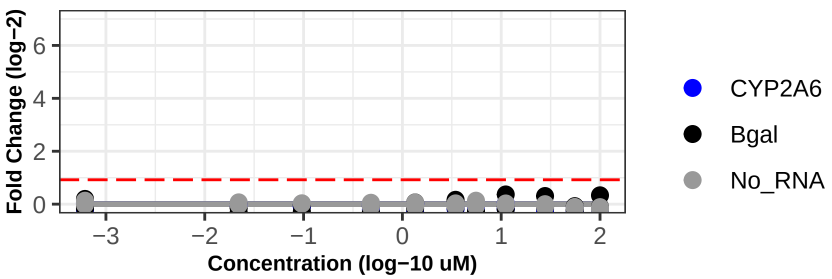


Ethoprop: CYP2D6

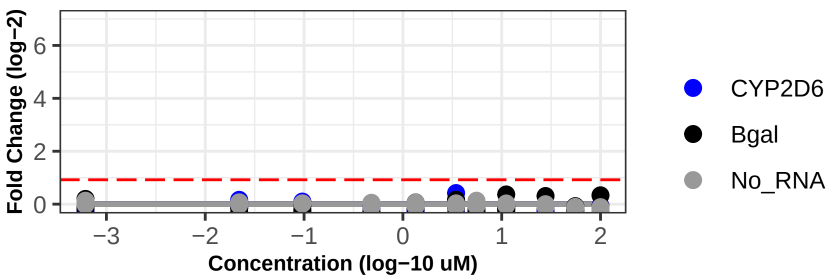


Ethoprop: CYP2B6

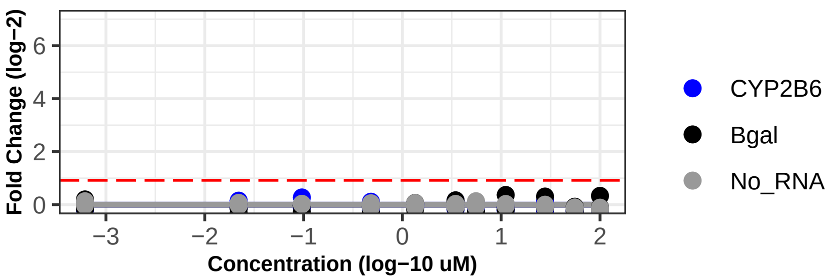


Ethoprop: CYP2E1

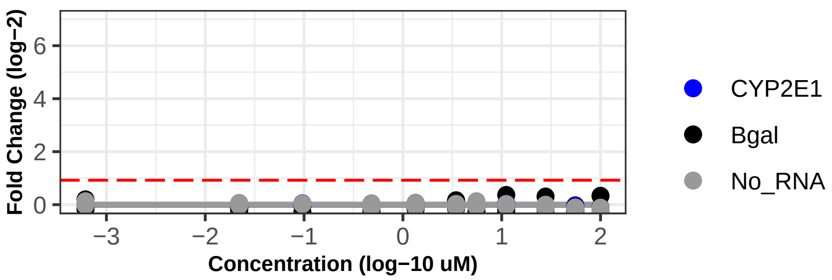


Ethoprop: CYP2C8

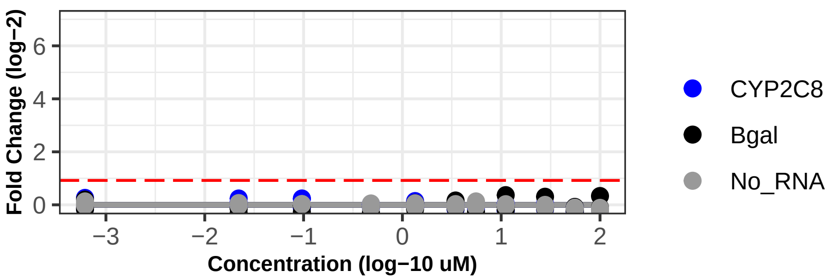


Ethoprop: CYP2J2

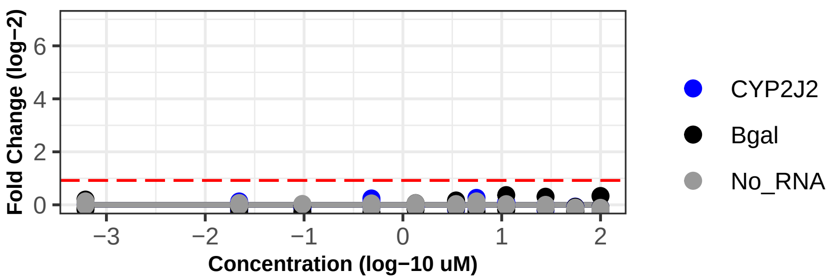


Ethoprop: CYP2C9

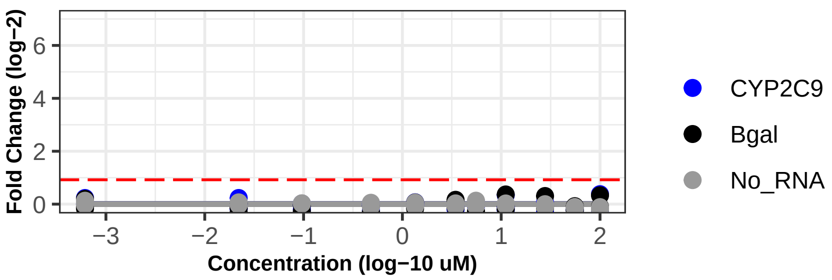


Ethoprop: CYP3A4

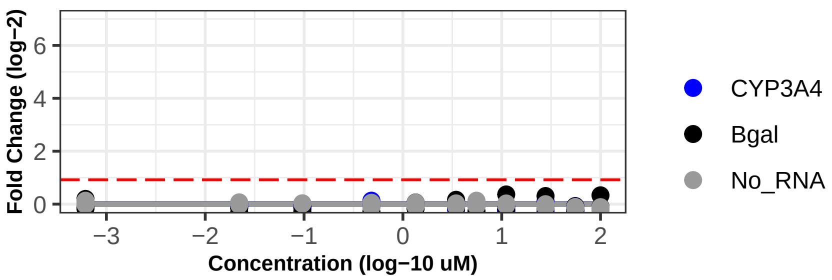

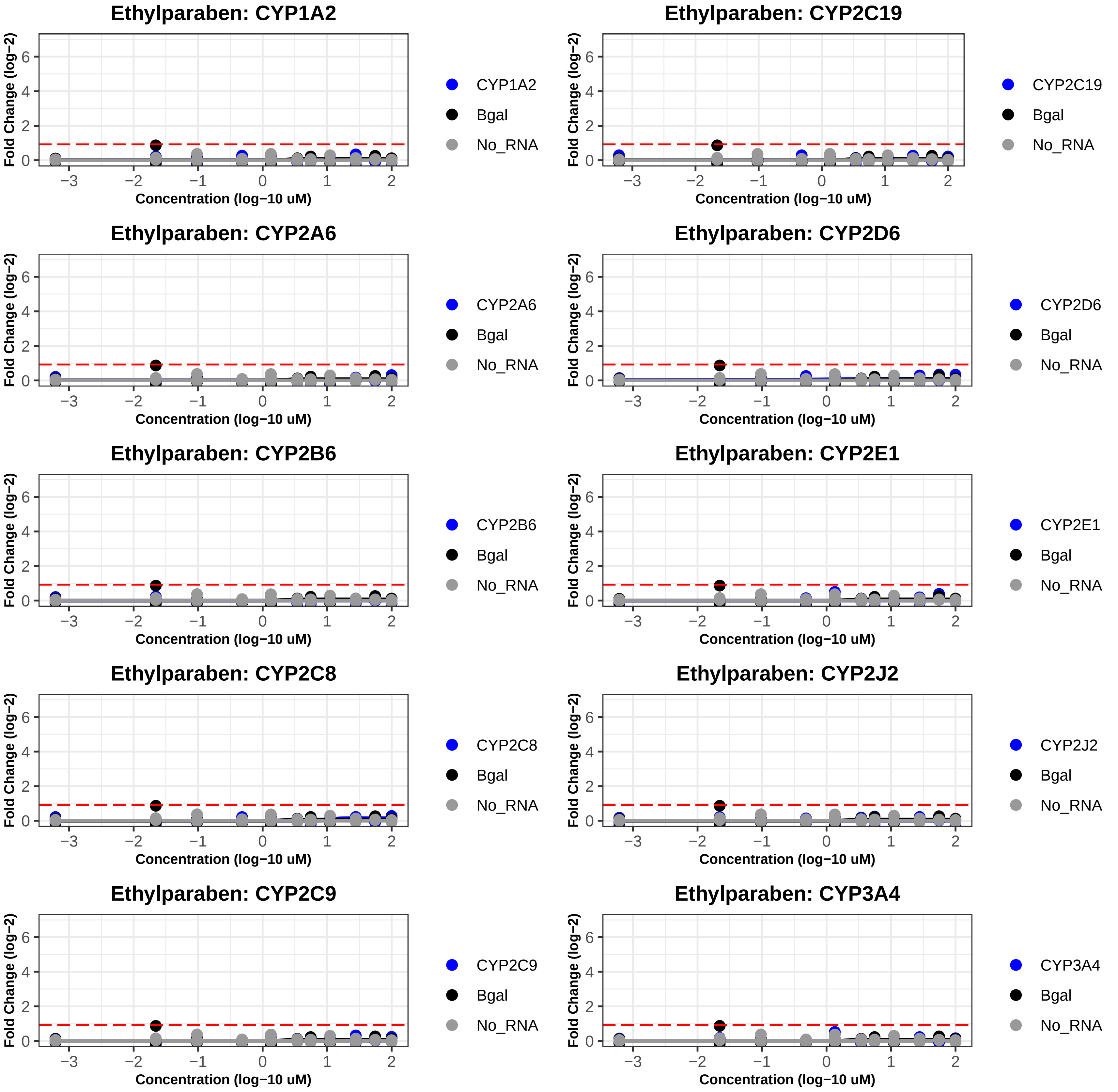
Ethylparaben: CYP1A2
Fold Change (log−2)
Concentration (log−10 uM)
CYP1A2
Bgal
No_RNA
Ethylparaben: CYP2C19
CYP2C19
Ethylparaben: CYP2A6
CYP2A6
Ethylparaben: CYP2D6
CYP2D6
Ethylparaben: CYP2B6
CYP2B6
Ethylparaben: CYP2E1
CYP2E1
Ethylparaben: CYP2C8
CYP2C8
Ethylparaben: CYP2J2
CYP2J2
Ethylparaben: CYP2C9
CYP2C9
Ethylparaben: CYP3A4
CYP3A4

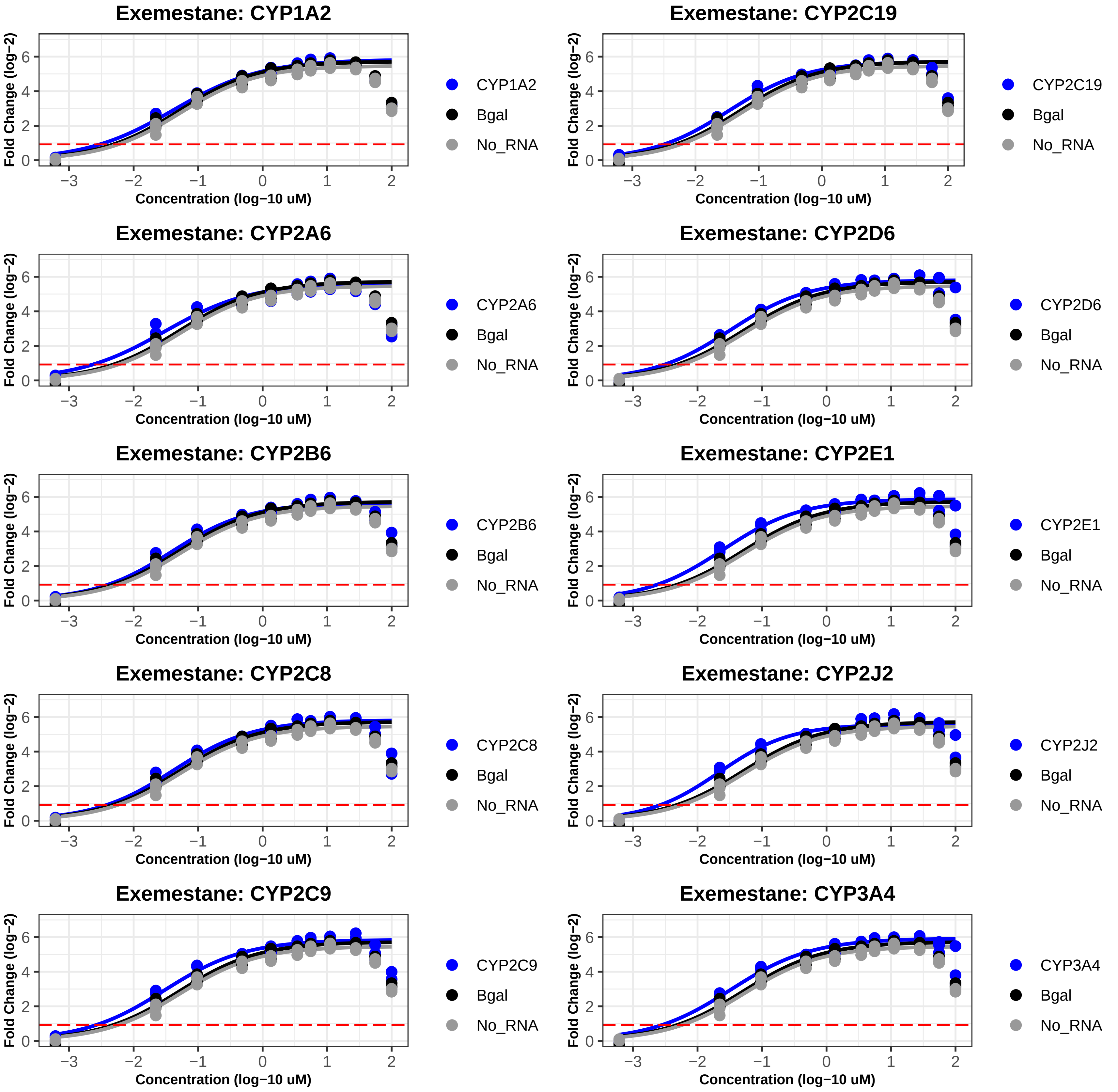
Exemestane: CYP1A2
Exemestane: CYP2C19
Exemestane: CYP2A6
Exemestane: CYP2D6
Exemestane: CYP2B6
Exemestane: CYP2E1
Exemestane: CYP2C8
Exemestane: CYP2J2
Exemestane: CYP2C9
Exemestane: CYP3A4
Fold Change (log−2)
Concentration (log−10 uM)
CYP1A2
CYP2C19
CYP2A6
CYP2D6
CYP2B6
CYP2E1
CYP2C8
CYP2J2
CYP2C9
CYP3A4
Bgal
No_RNA

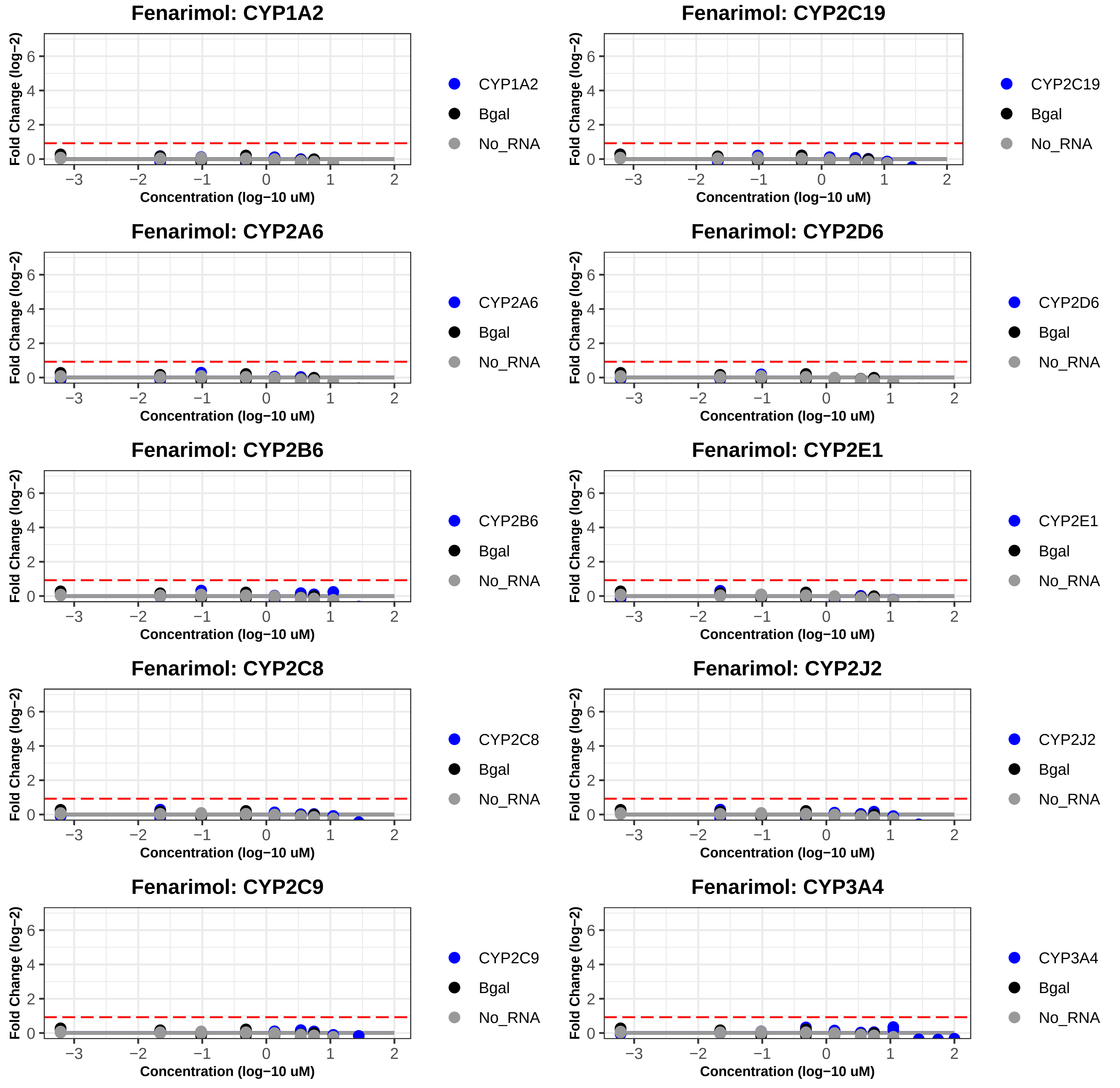
Fenarimol: CYP1A2
Fold Change (log−2)
Concentration (log−10 uM)
CYP1A2
Bgal
No_RNA
Fenarimol: CYP2C19
CYP2C19
Bgal
No_RNA
Fenarimol: CYP2A6
CYP2A6
Bgal
No_RNA
Fenarimol: CYP2D6
CYP2D6
Bgal
No_RNA
Fenarimol: CYP2B6
CYP2B6
Bgal
No_RNA
Fenarimol: CYP2E1
CYP2E1
Bgal
No_RNA
Fenarimol: CYP2C8
CYP2C8
Bgal
No_RNA
Fenarimol: CYP2J2
CYP2J2
Bgal
No_RNA
Fenarimol: CYP2C9
CYP2C9
Bgal
No_RNA
Fenarimol: CYP3A4
CYP3A4
Bgal
No_RNA

## Fenitrothion: CYP1A2

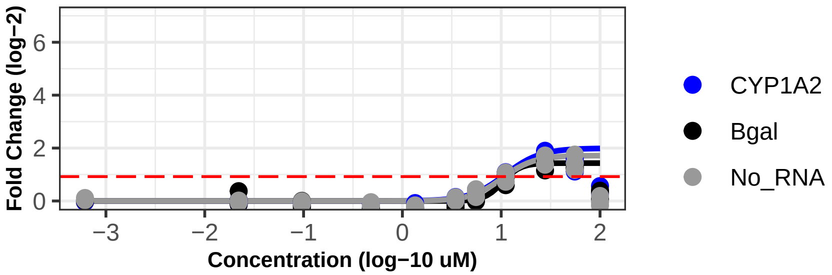


## Fenitrothion: CYP2C19

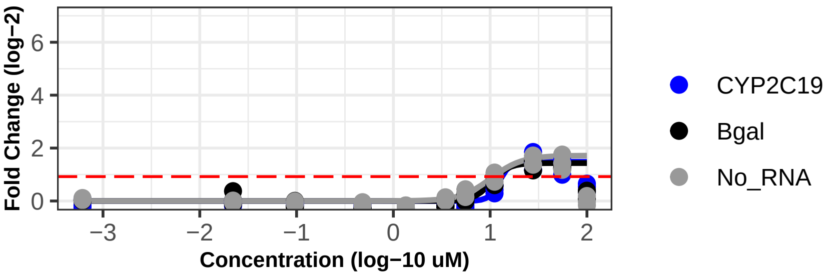


## Fenitrothion: CYP2A6

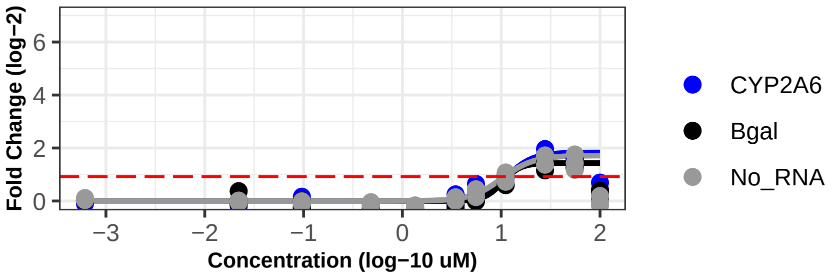


## Fenitrothion: CYP2D6

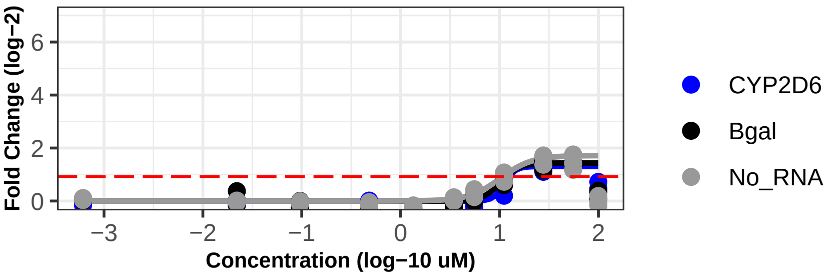


## Fenitrothion: CYP2B6

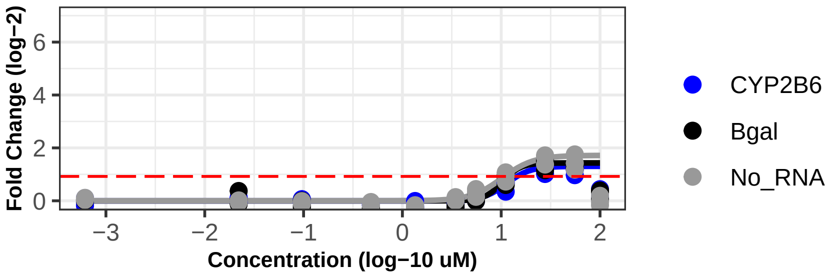


## Fenitrothion: CYP2E1

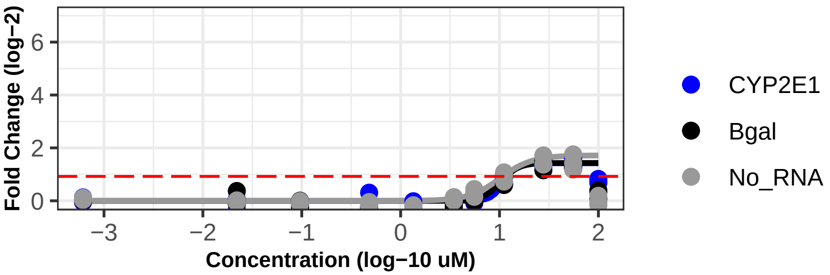


## Fenitrothion: CYP2C8

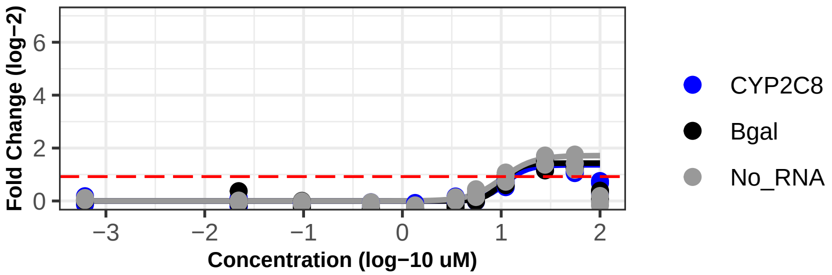


## Fenitrothion: CYP2J2

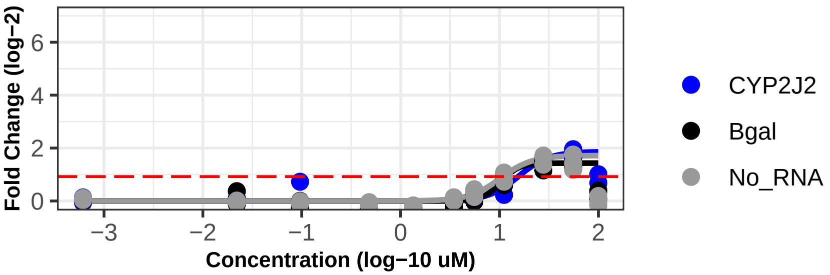


## Fenitrothion: CYP2C9

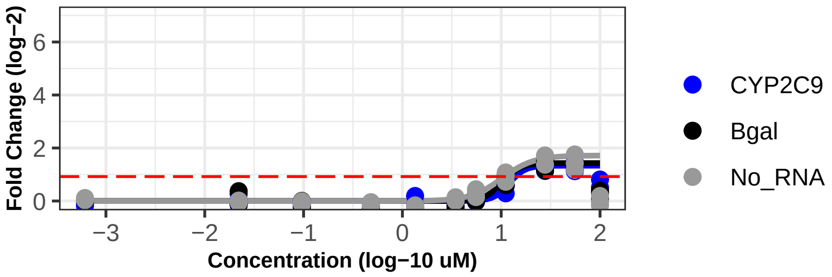


## Fenitrothion: CYP3A4

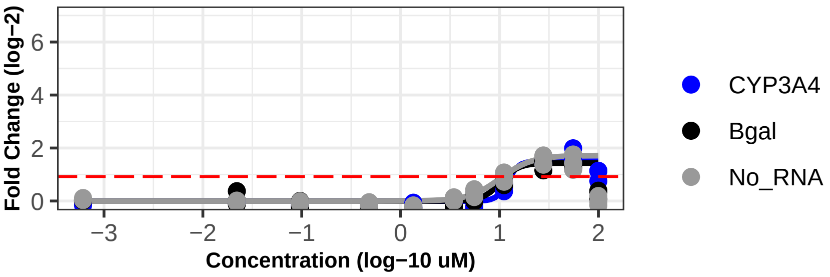

Fenthion: CYP1A2

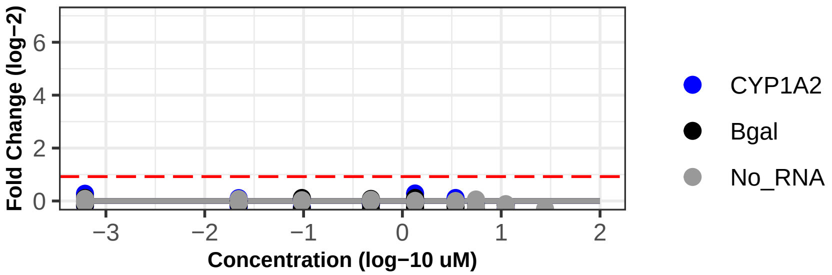


Fenthion: CYP2C19

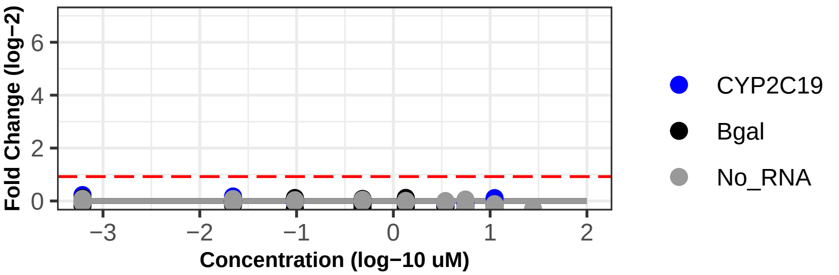


Fenthion: CYP2A6

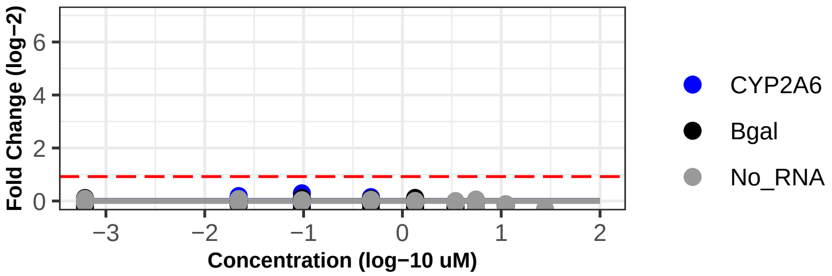


Fenthion: CYP2D6

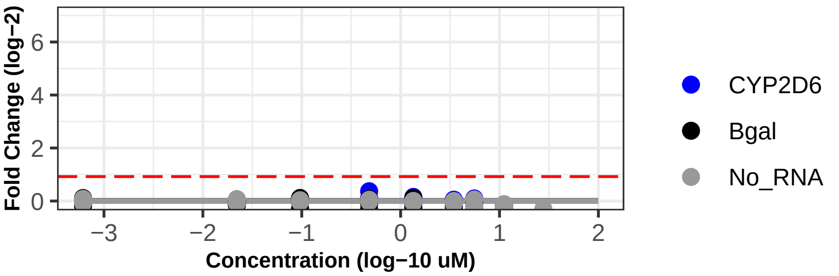


Fenthion: CYP2B6

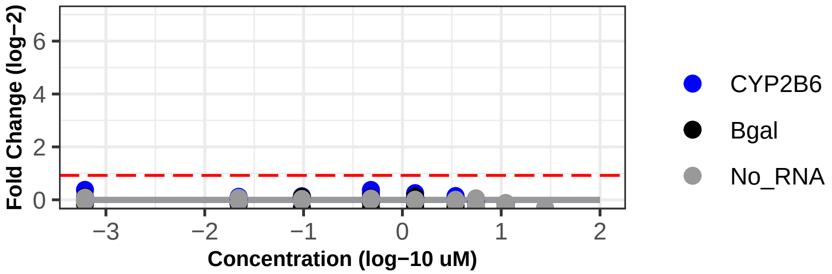


Fenthion: CYP2E1

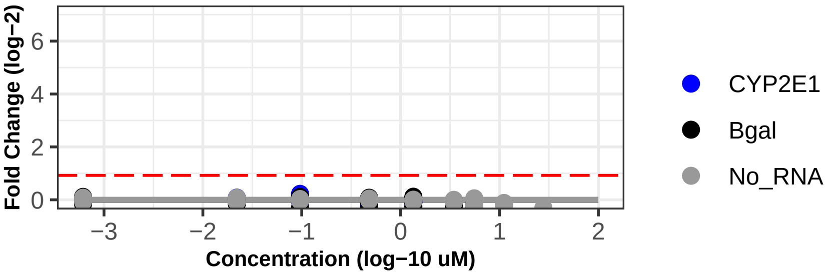


Fenthion: CYP2C8

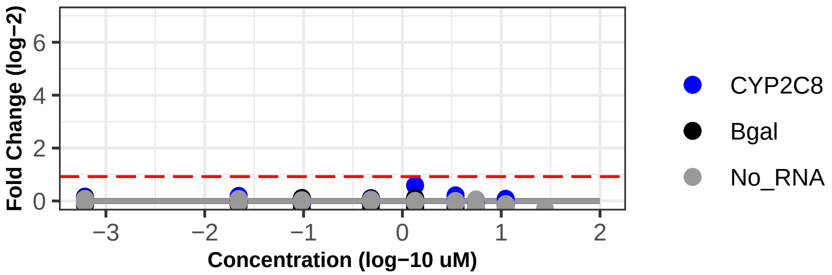


Fenthion: CYP2J2

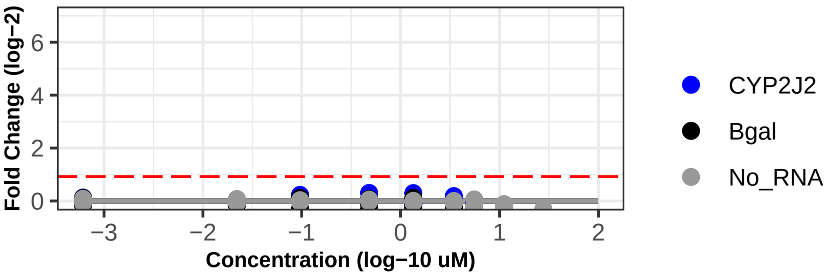


Fenthion: CYP2C9

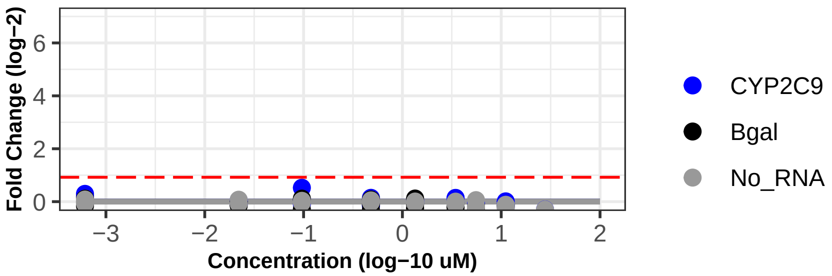


Fenthion: CYP3A4

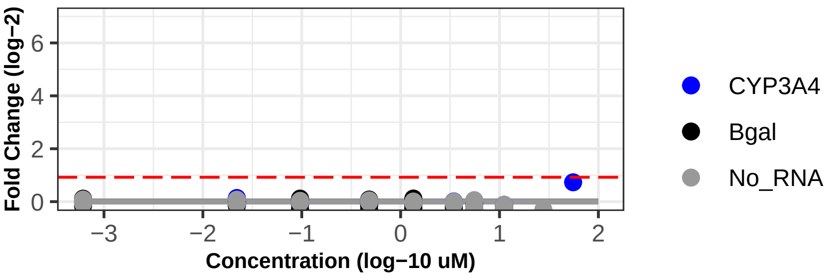

Fenvalerate: CYP1A2

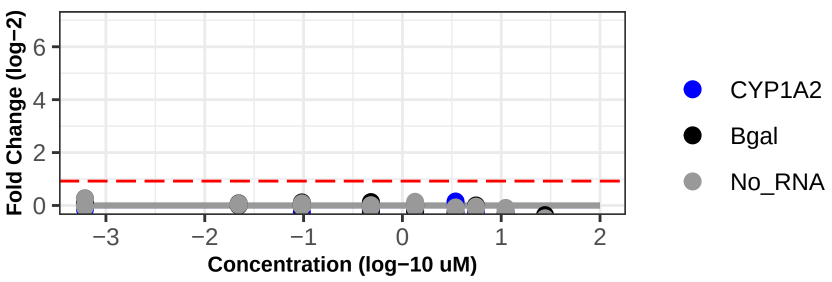


Fenvalerate: CYP2C19

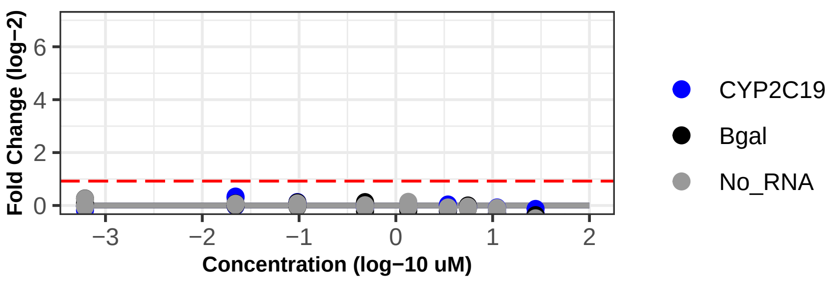


Fenvalerate: CYP2A6

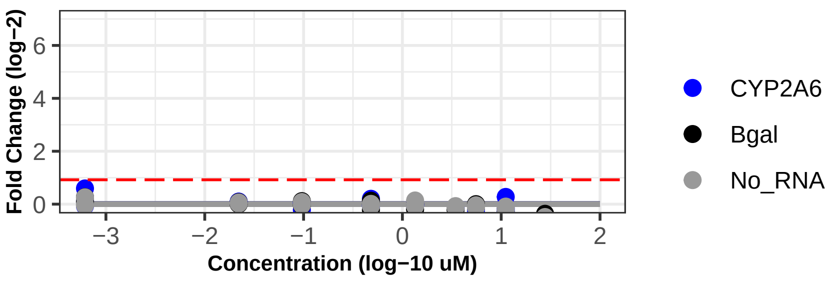


Fenvalerate: CYP2D6

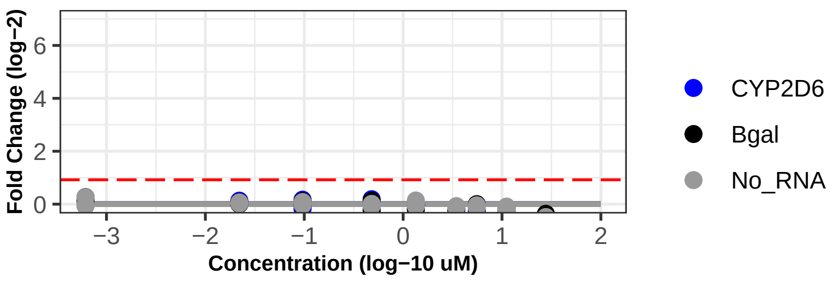


Fenvalerate: CYP2B6

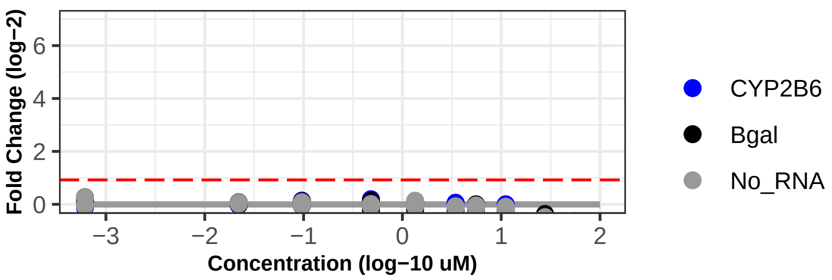


Fenvalerate: CYP2E1

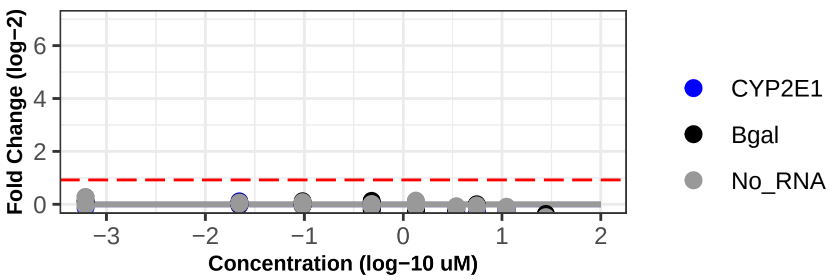


Fenvalerate: CYP2C8

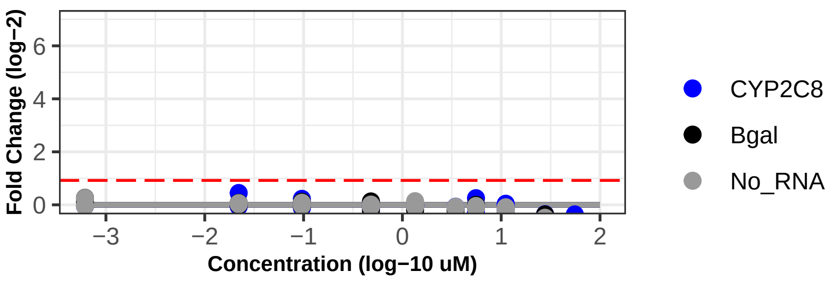


Fenvalerate: CYP2J2

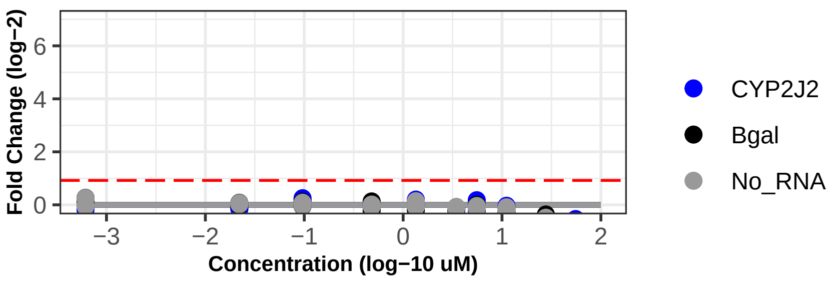


Fenvalerate: CYP2C9

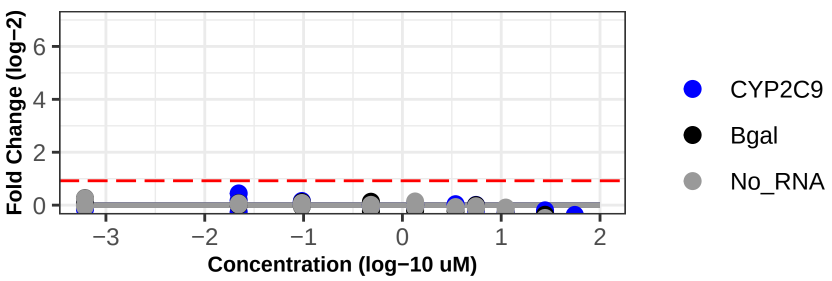


Fenvalerate: CYP3A4

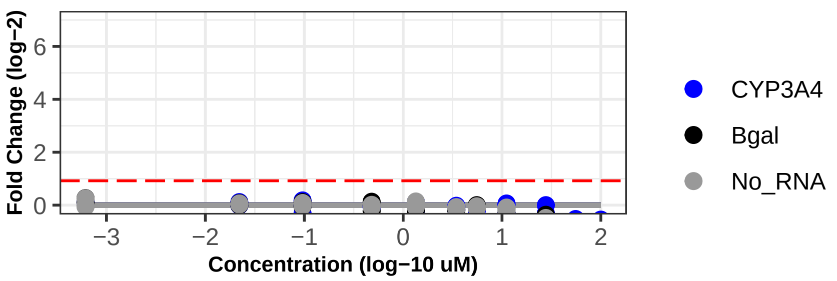

## Finasteride: CYP1A2

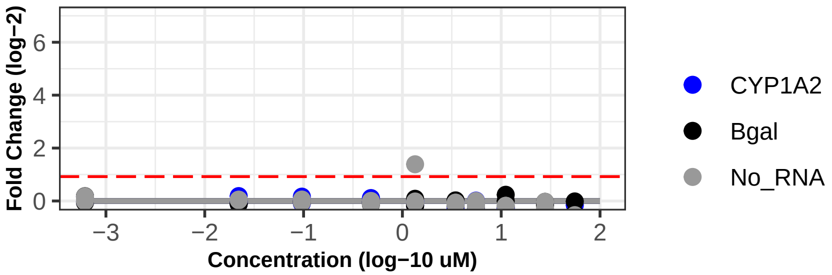


## Finasteride: CYP2C19

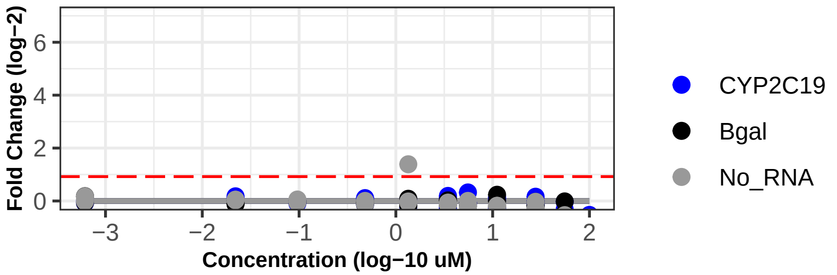


## Finasteride: CYP2A6

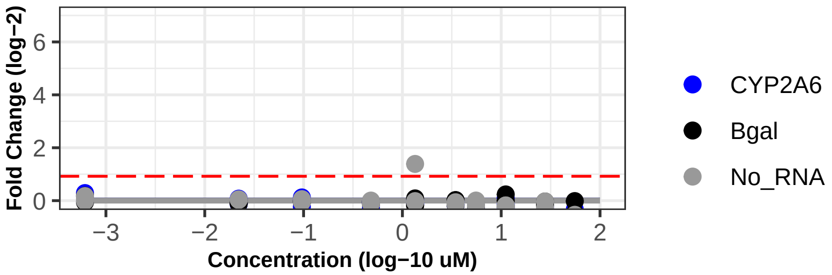


## Finasteride: CYP2D6

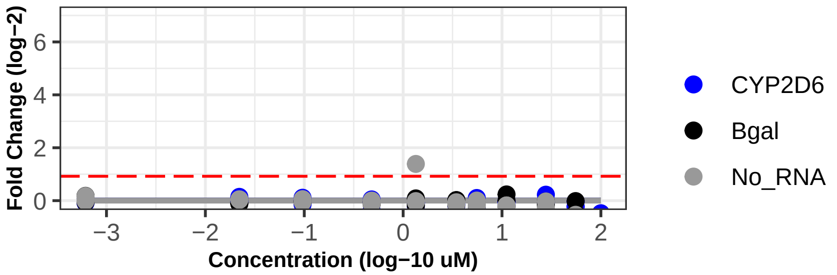


## Finasteride: CYP2B6

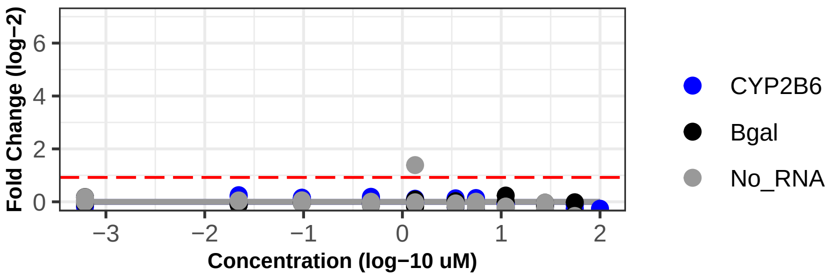


## Finasteride: CYP2E1

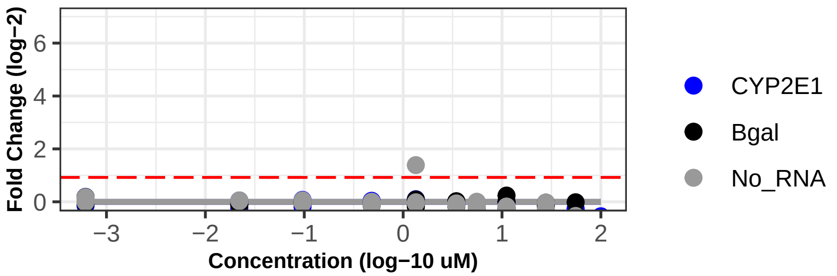


## Finasteride: CYP2C8

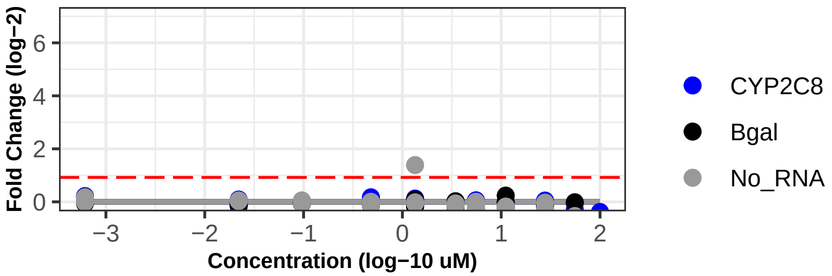


## Finasteride: CYP2J2

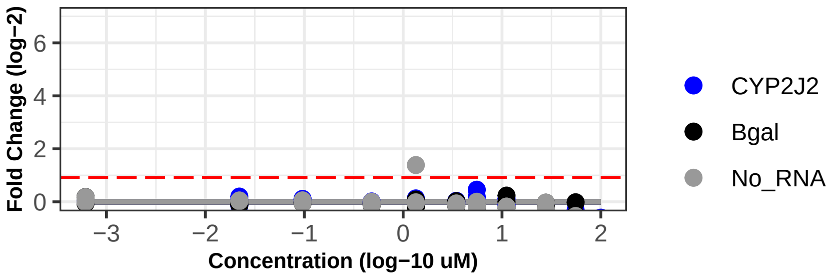


## Finasteride: CYP2C9

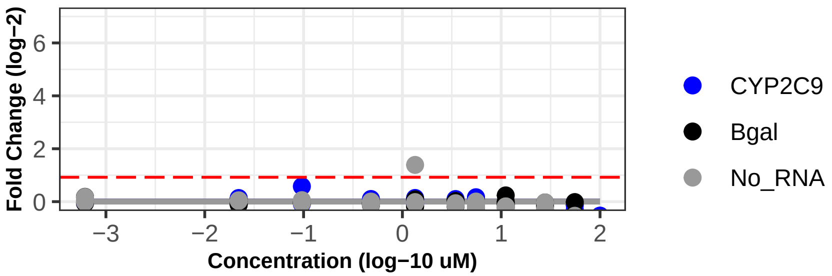


## Finasteride: CYP3A4

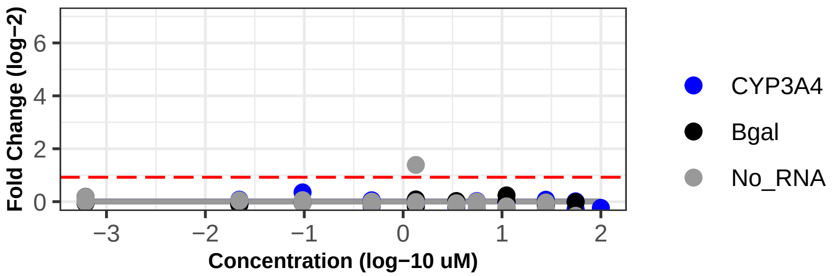

## Flutamide: CYP1A2

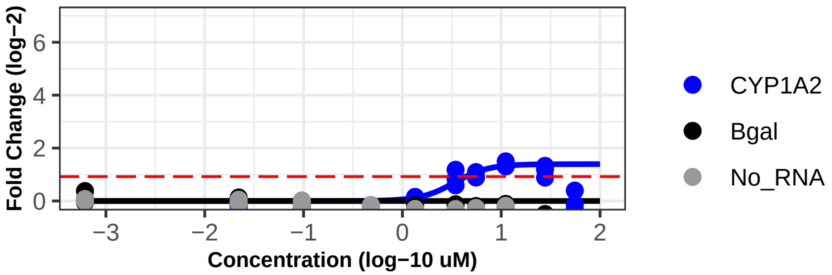


## Flutamide: CYP2A6

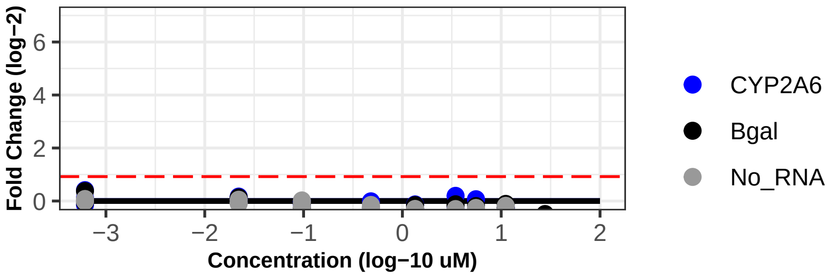


## Flutamide: CYP2B6

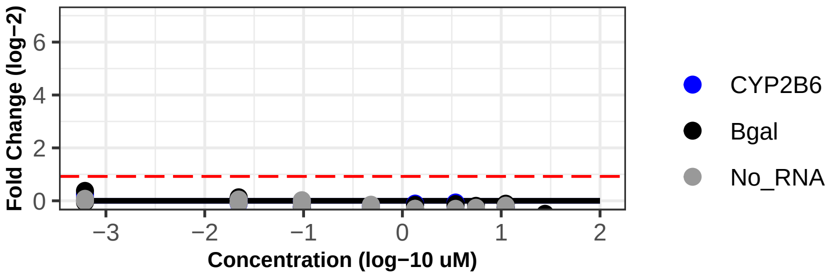


## Flutamide: CYP2C8

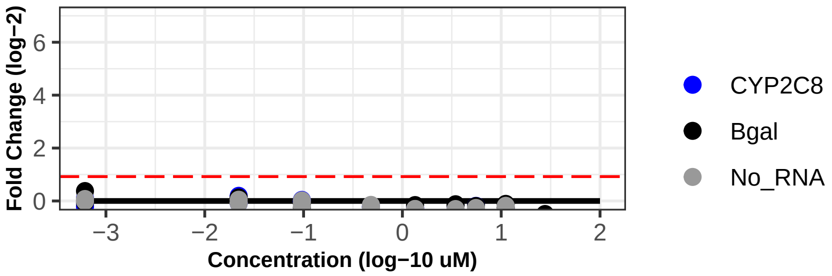


## Flutamide: CYP2C9

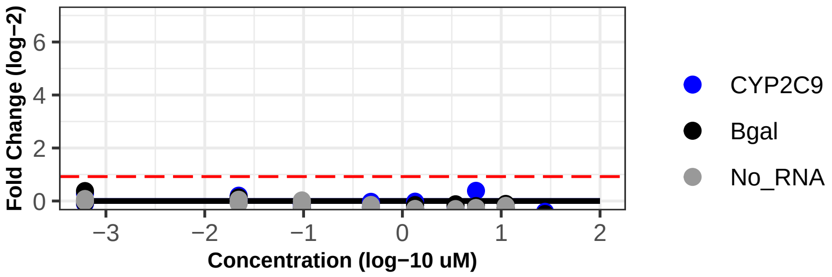


## Flutamide: CYP2C19

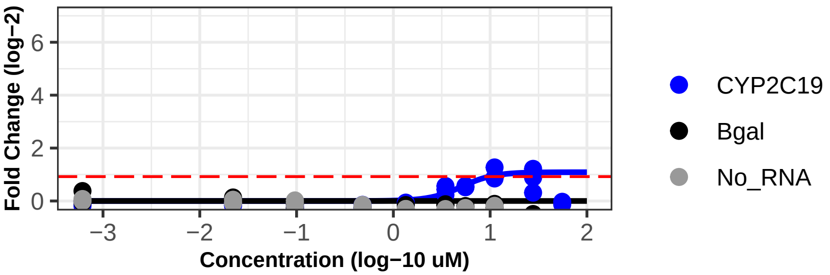


## Flutamide: CYP2D6

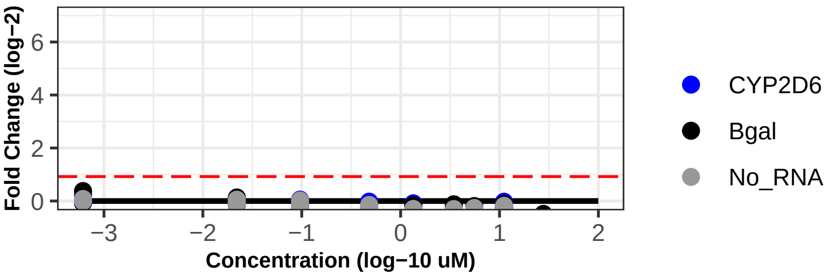


## Flutamide: CYP2E1

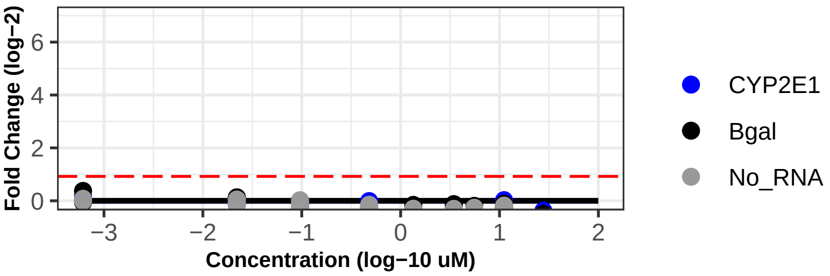


## Flutamide: CYP2J2

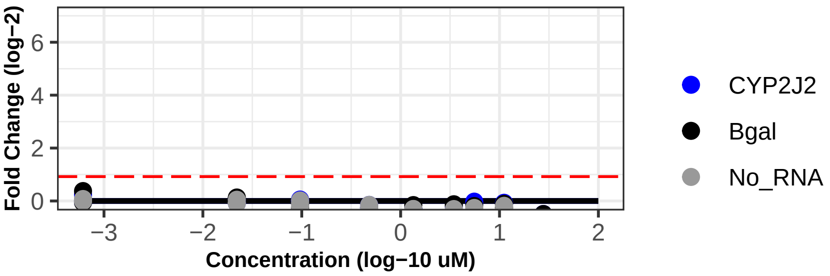


## Flutamide: CYP3A4

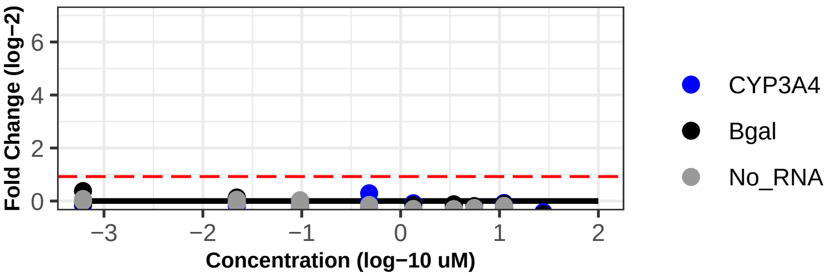

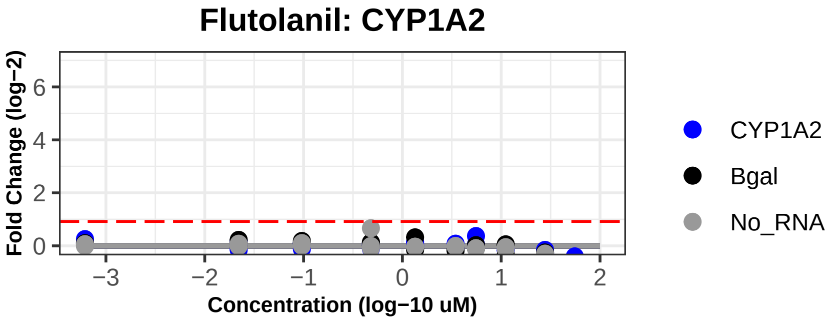
Flutolanil: CYP1A2
Fold Change (log−2)
Concentration (log−10 uM)
CYP1A2
Bgal
No_RNA

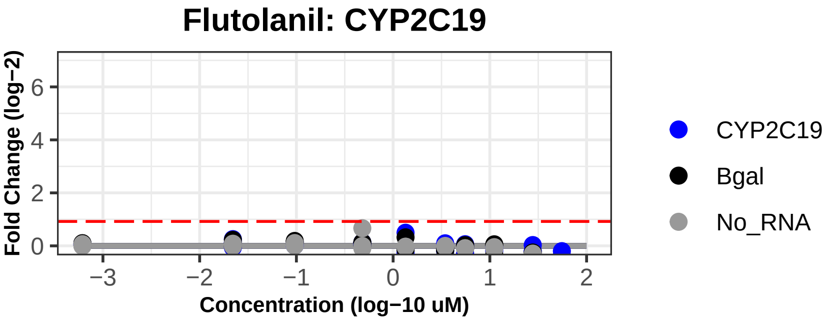
Flutolanil: CYP2C19
Fold Change (log−2)
Concentration (log−10 uM)
CYP2C19
Bgal
No_RNA

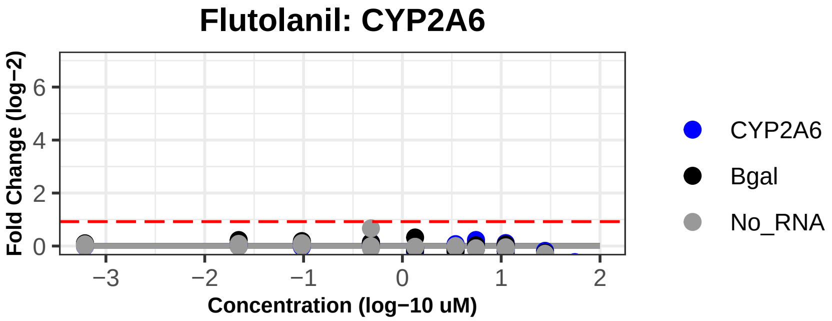
Flutolanil: CYP2A6
Fold Change (log−2)
Concentration (log−10 uM)
CYP2A6
Bgal
No_RNA

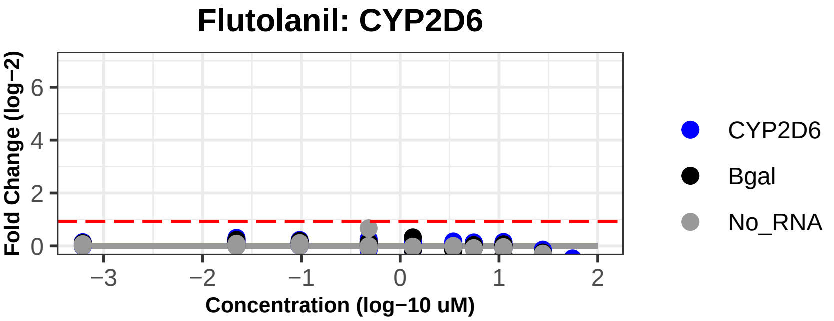
Flutolanil: CYP2D6
Fold Change (log−2)
Concentration (log−10 uM)
CYP2D6
Bgal
No_RNA

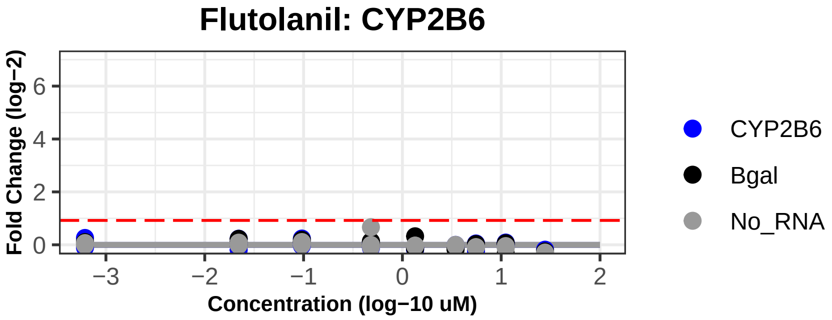
Flutolanil: CYP2B6
Fold Change (log−2)
Concentration (log−10 uM)
CYP2B6
Bgal
No_RNA

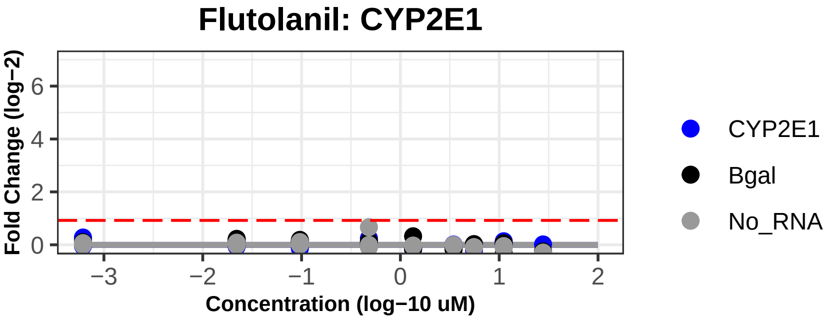
Flutolanil: CYP2E1
Fold Change (log−2)
Concentration (log−10 uM)
CYP2E1
Bgal
No_RNA

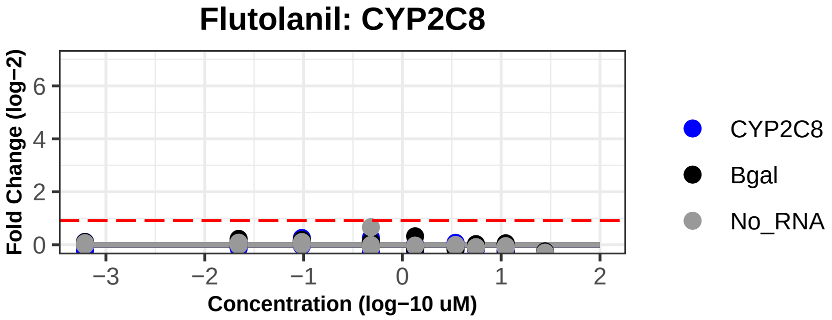
Flutolanil: CYP2C8
Fold Change (log−2)
Concentration (log−10 uM)
CYP2C8
Bgal
No_RNA

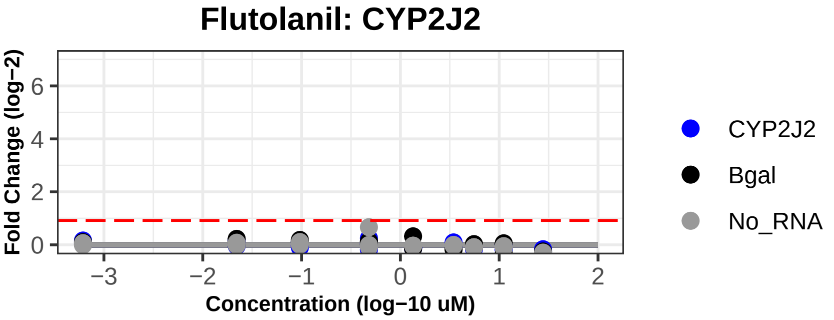
Flutolanil: CYP2J2
Fold Change (log−2)
Concentration (log−10 uM)
CYP2J2
Bgal
No_RNA

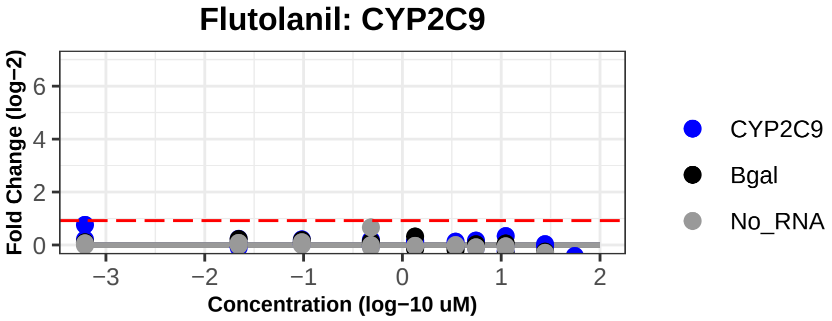
Flutolanil: CYP2C9
Fold Change (log−2)
Concentration (log−10 uM)
CYP2C9
Bgal
No_RNA

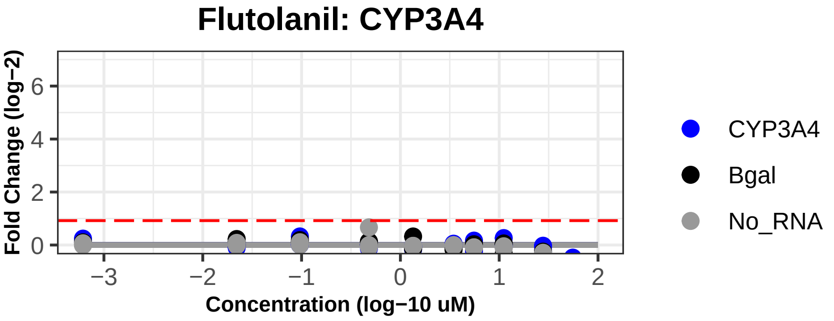
Flutolanil: CYP3A4
Fold Change (log−2)
Concentration (log−10 uM)
CYP3A4
Bgal
No_RNA

## Folpet: CYP1A2

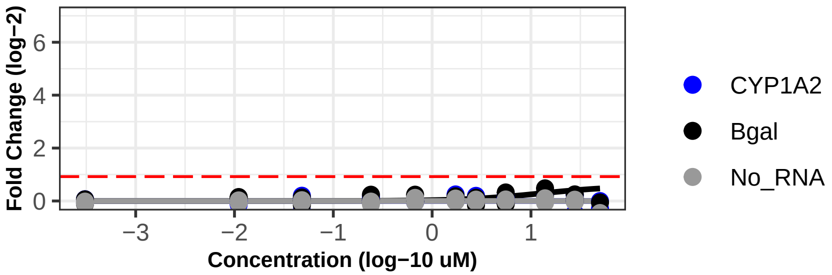


## Folpet: CYP2C19

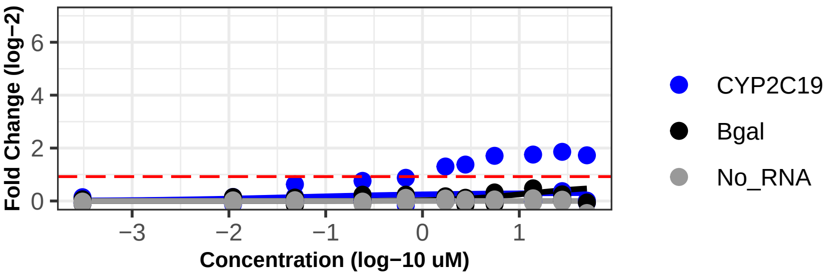


## Folpet: CYP2A6

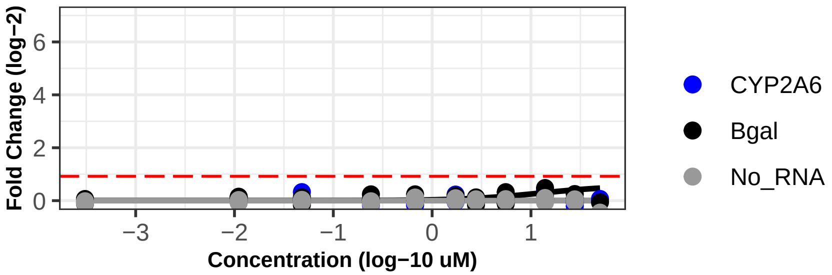


## Folpet: CYP2D6

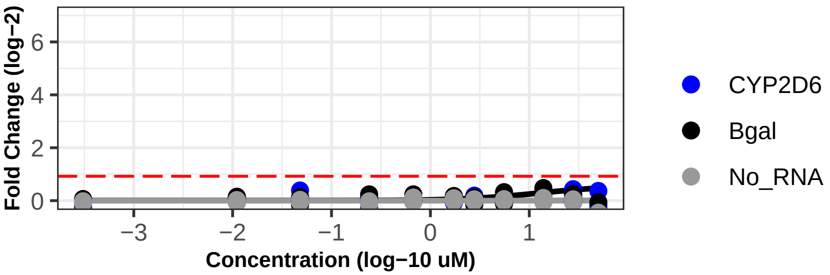


## Folpet: CYP2B6

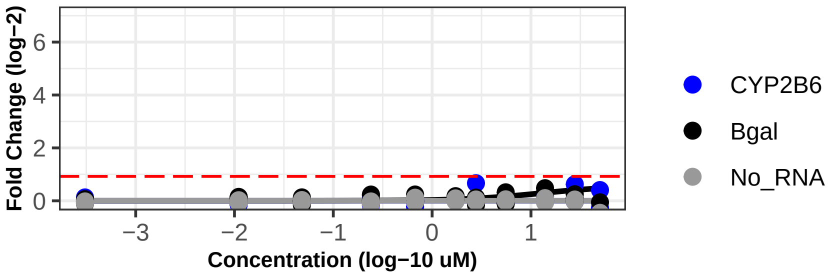


## Folpet: CYP2E1

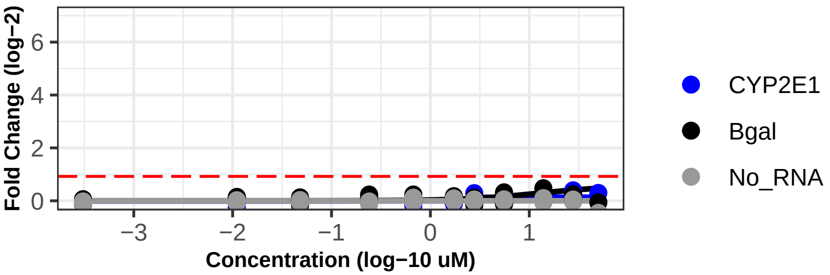


## Folpet: CYP2C8

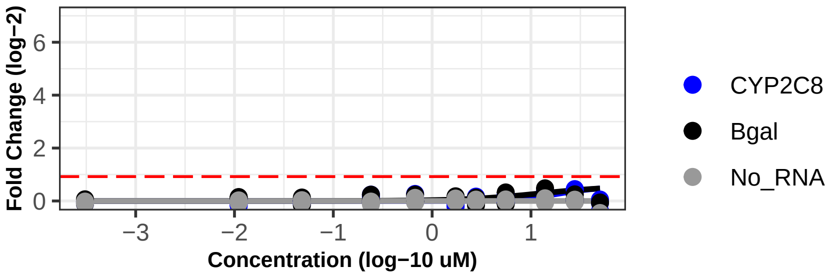


## Folpet: CYP2J2

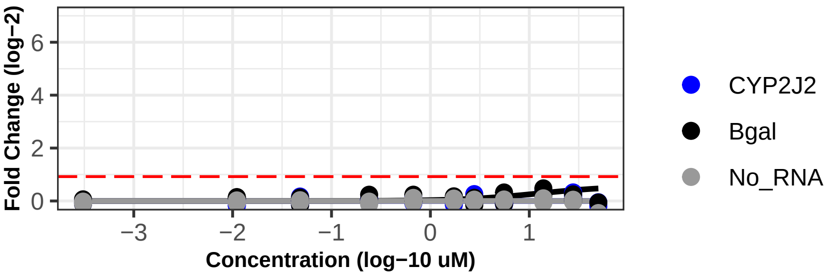


## Folpet: CYP2C9

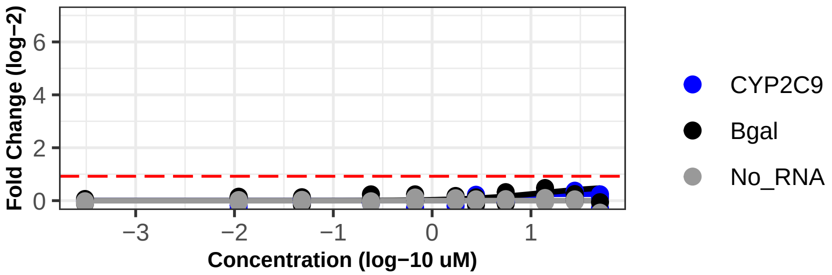


## Folpet: CYP3A4

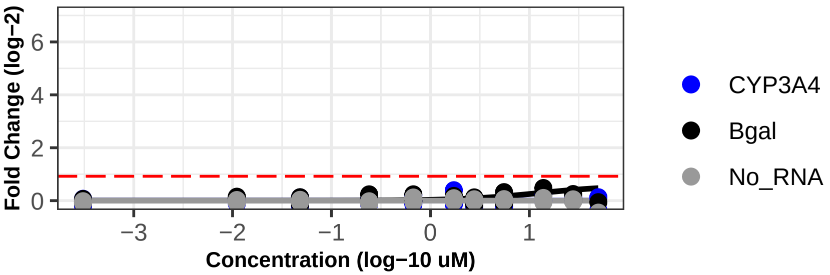

Formestane: CYP1A2

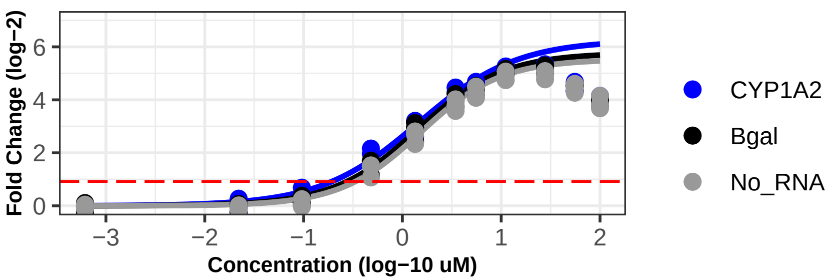


Formestane: CYP2C19

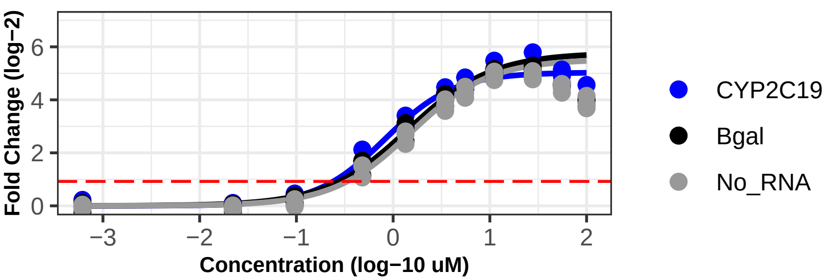


Formestane: CYP2A6

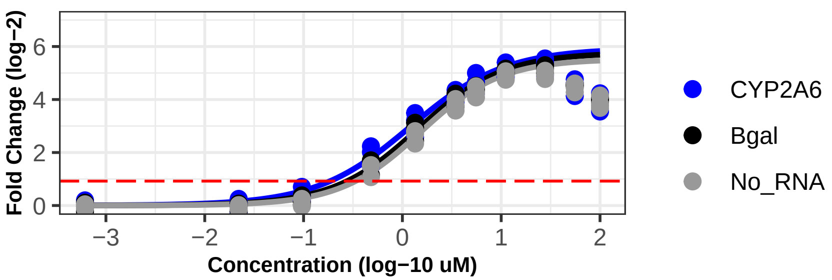


Formestane: CYP2D6

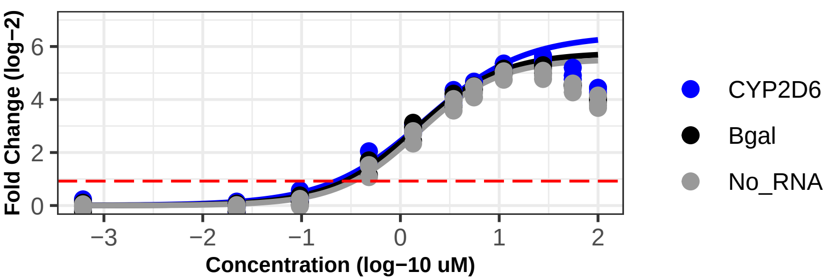


Formestane: CYP2B6

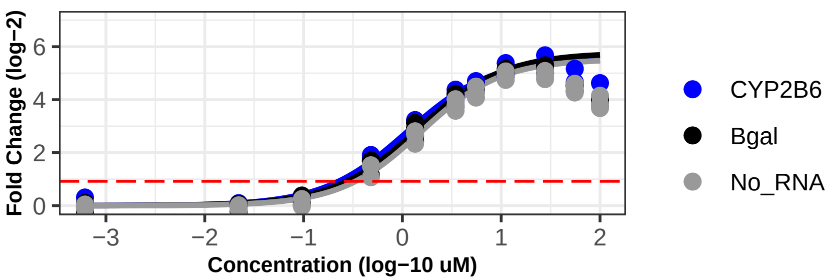


Formestane: CYP2E1

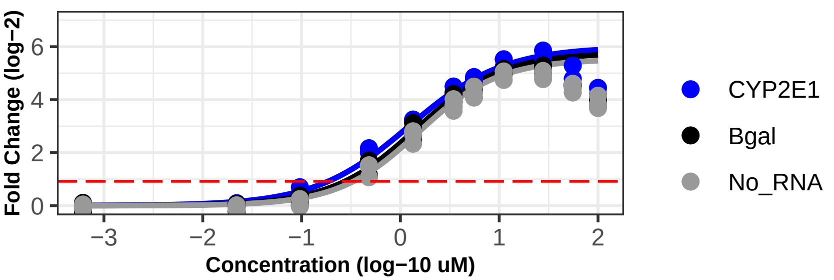


Formestane: CYP2C8

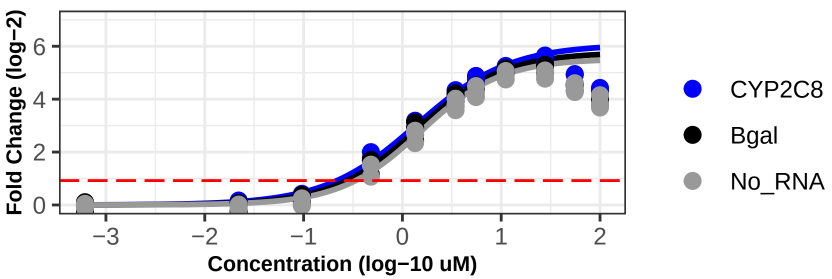


Formestane: CYP2J2

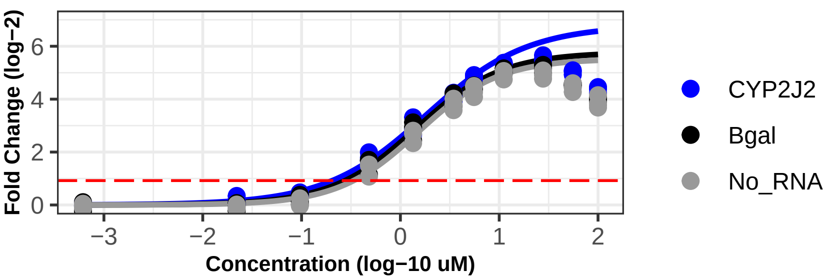


Formestane: CYP2C9

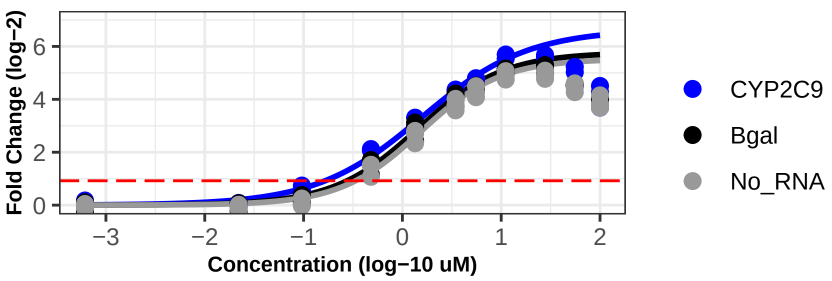


Formestane: CYP3A4

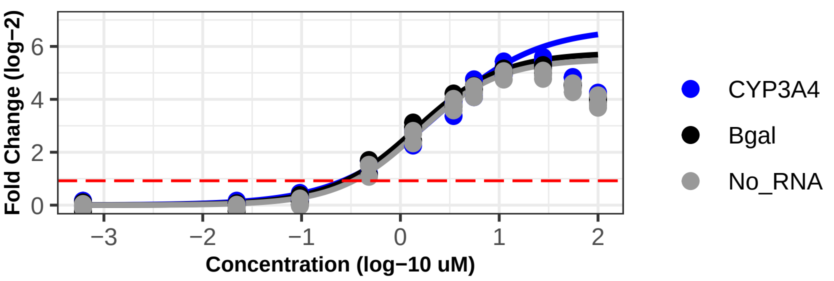

## Fulvestrant: CYP1A2

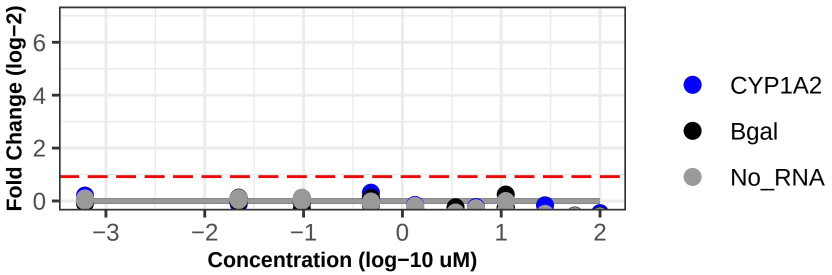


## Fulvestrant: CYP2C19

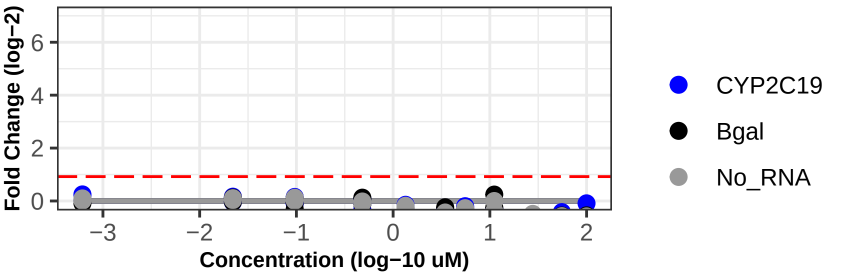


## Fulvestrant: CYP2A6

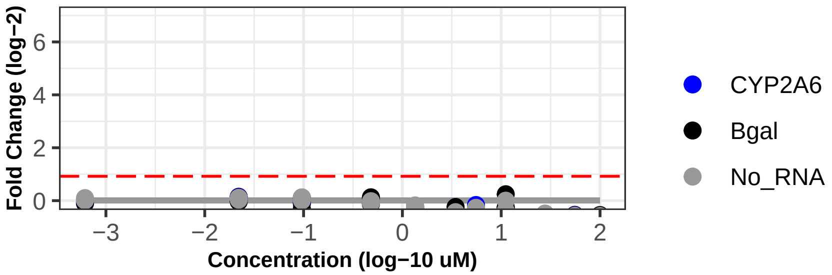


## Fulvestrant: CYP2D6

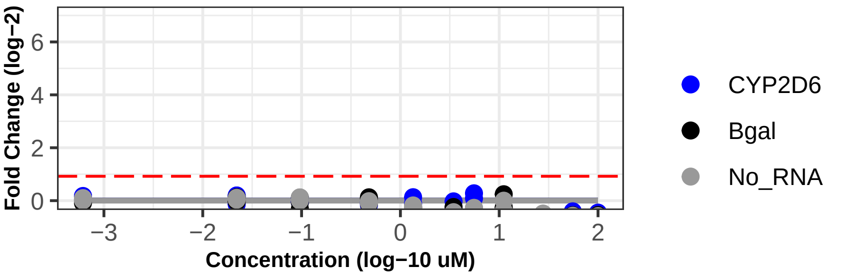


## Fulvestrant: CYP2B6

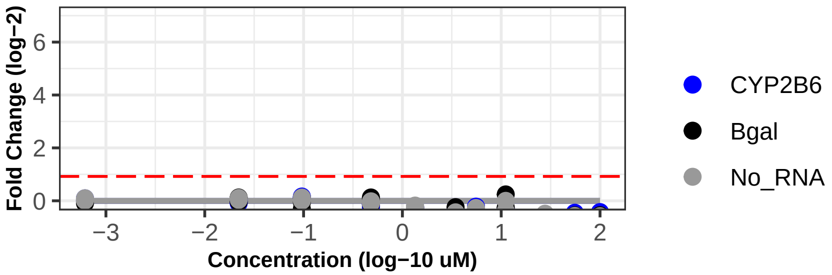


## Fulvestrant: CYP2E1

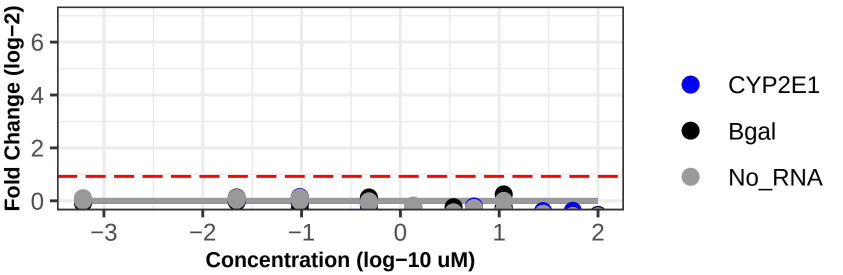


## Fulvestrant: CYP2C8

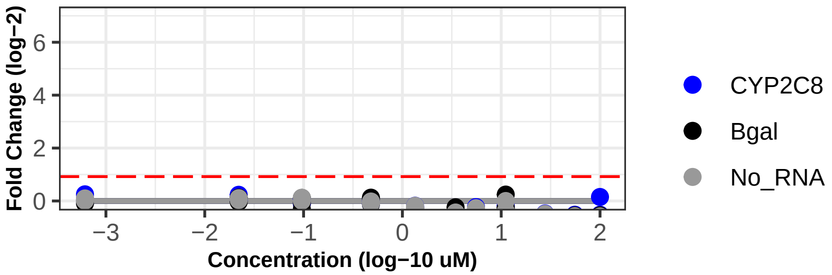


## Fulvestrant: CYP2J2

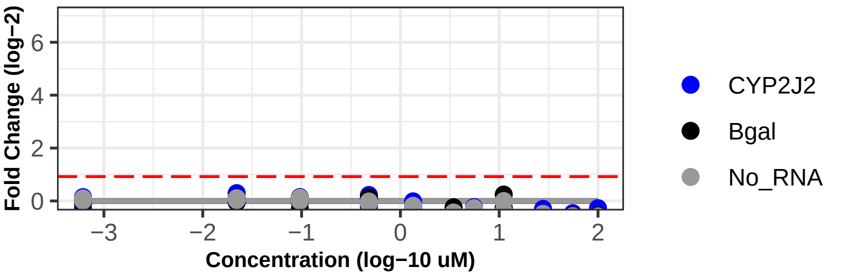


## Fulvestrant: CYP2C9

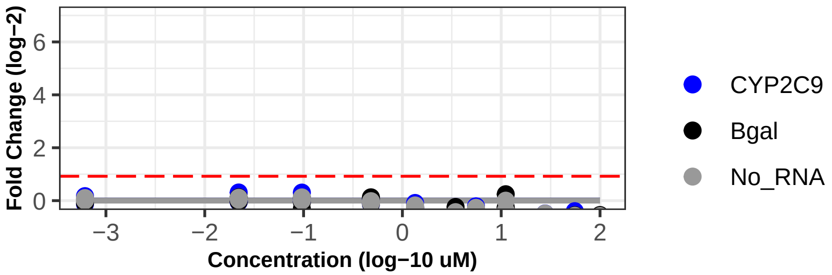


## Fulvestrant: CYP3A4

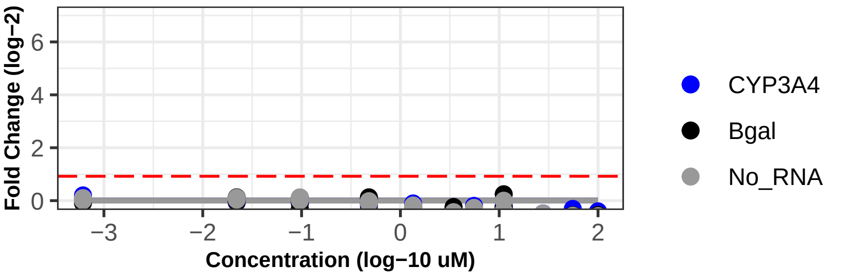

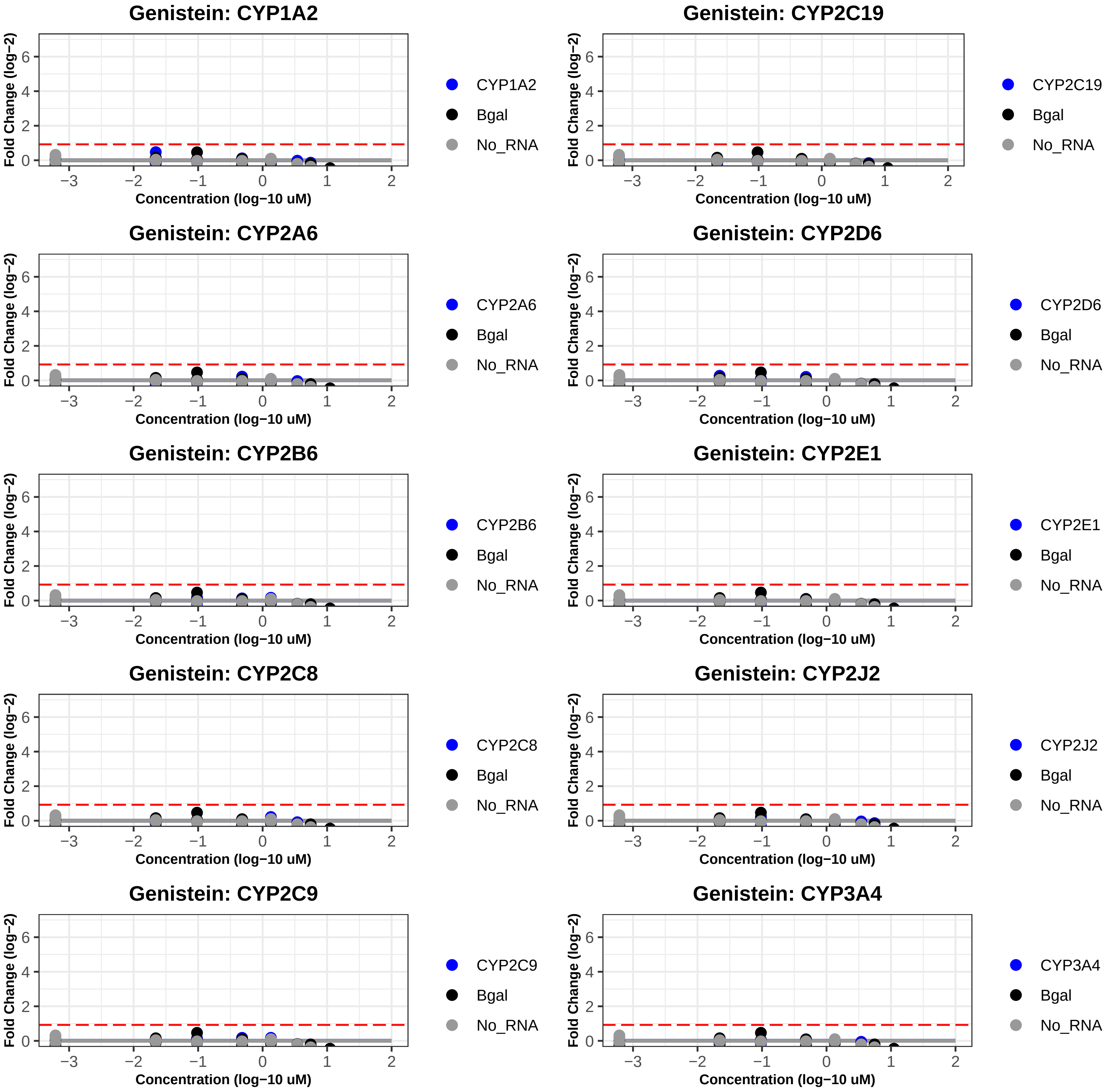
Genistein: CYP1A2
Fold Change (log−2)
Concentration (log−10 uM)
CYP1A2
Bgal
No_RNA
Genistein: CYP2C19
Fold Change (log−2)
Concentration (log−10 uM)
CYP2C19
Bgal
No_RNA
Genistein: CYP2A6
Fold Change (log−2)
Concentration (log−10 uM)
CYP2A6
Bgal
No_RNA
Genistein: CYP2D6
Fold Change (log−2)
Concentration (log−10 uM)
CYP2D6
Bgal
No_RNA
Genistein: CYP2B6
Fold Change (log−2)
Concentration (log−10 uM)
CYP2B6
Bgal
No_RNA
Genistein: CYP2E1
Fold Change (log−2)
Concentration (log−10 uM)
CYP2E1
Bgal
No_RNA
Genistein: CYP2C8
Fold Change (log−2)
Concentration (log−10 uM)
CYP2C8
Bgal
No_RNA
Genistein: CYP2J2
Fold Change (log−2)
Concentration (log−10 uM)
CYP2J2
Bgal
No_RNA
Genistein: CYP2C9
Fold Change (log−2)
Concentration (log−10 uM)
CYP2C9
Bgal
No_RNA
Genistein: CYP3A4
Fold Change (log−2)
Concentration (log−10 uM)
CYP3A4
Bgal
No_RNA

## Hydroxyflutamide: CYP1A2

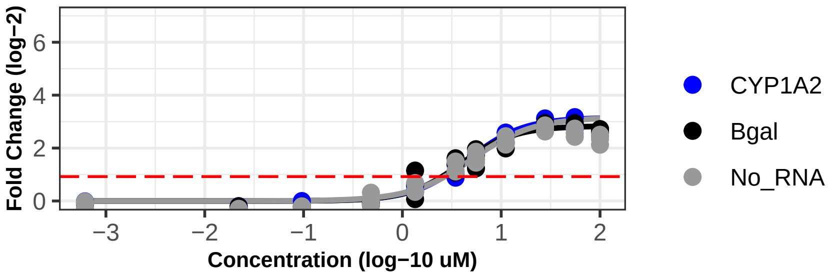


## Hydroxyflutamide: CYP2C19

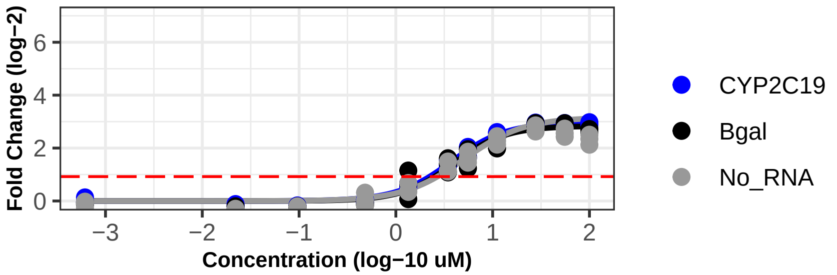


## Hydroxyflutamide: CYP2A6

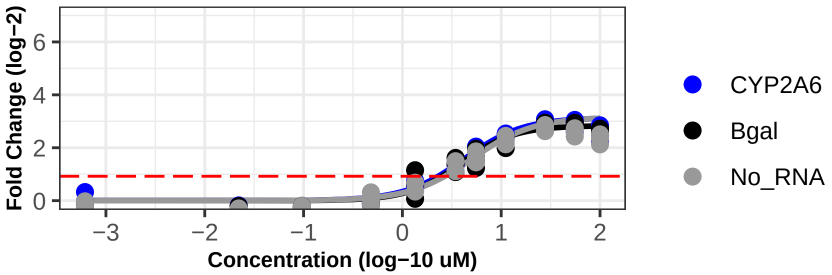


## Hydroxyflutamide: CYP2D6

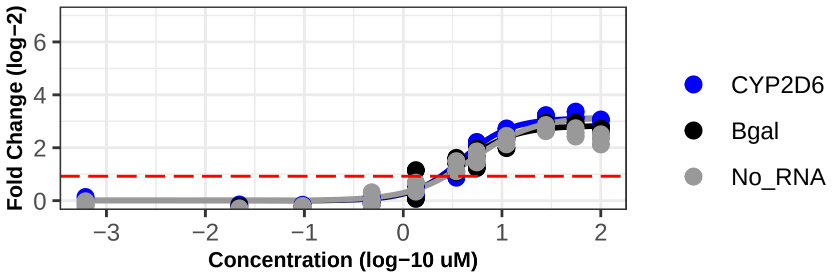


## Hydroxyflutamide: CYP2B6

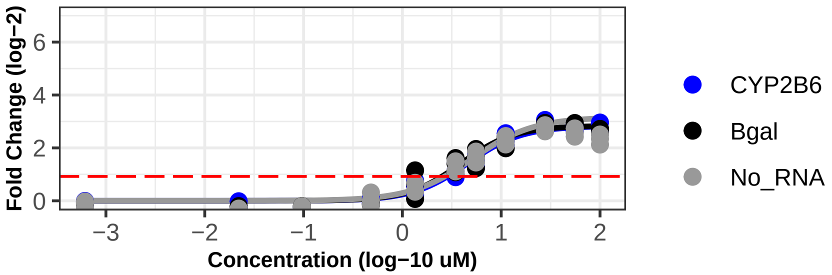


## Hydroxyflutamide: CYP2E1

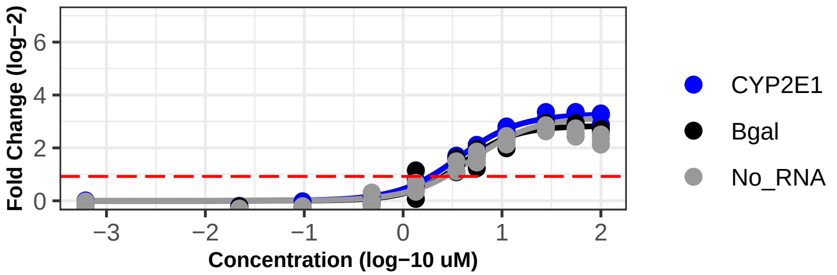


## Hydroxyflutamide: CYP2C8

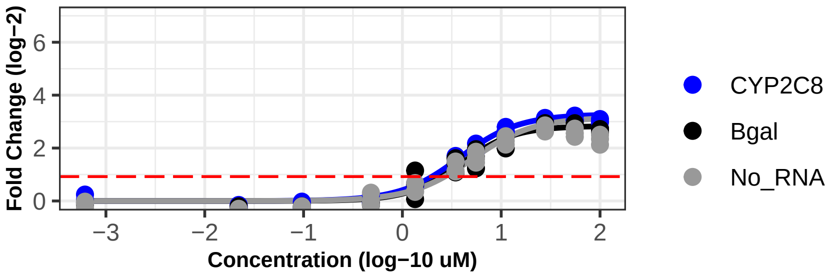


## Hydroxyflutamide: CYP2J2

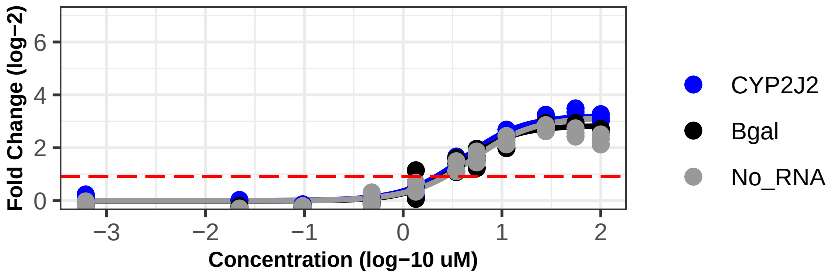


## Hydroxyflutamide: CYP2C9

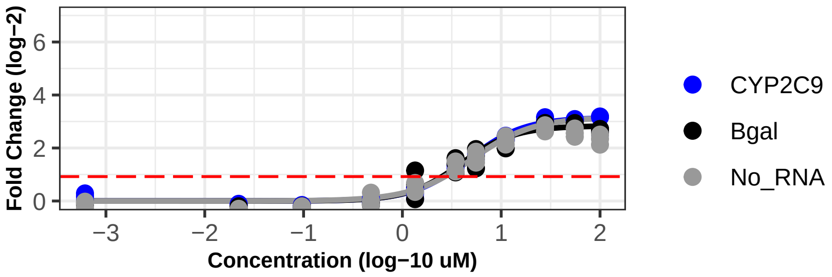


## Hydroxyflutamide: CYP3A4

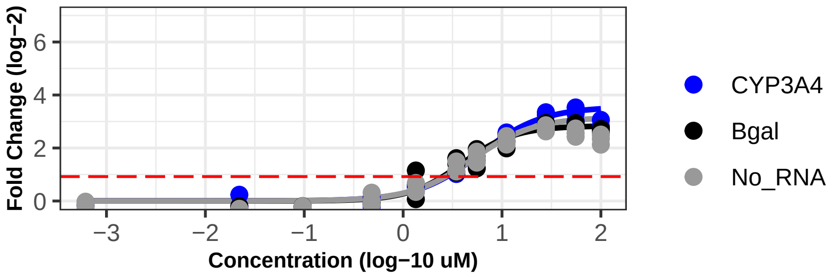

## Hydroxyprogesterone caproate: CYP1A2

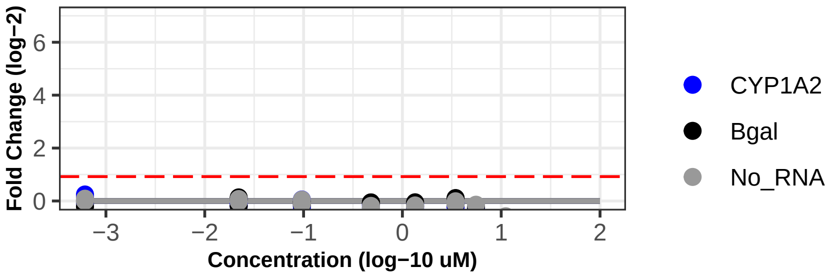


## Hydroxyprogesterone caproate: CYP2C19

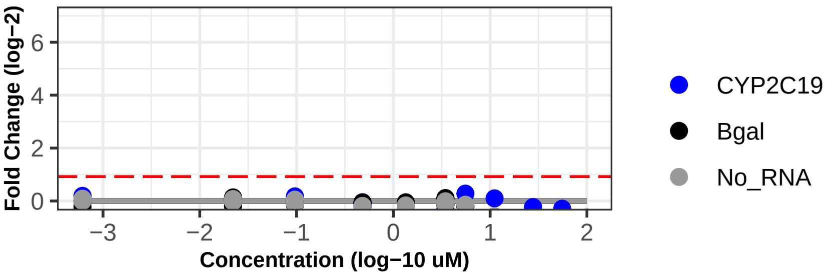


## Hydroxyprogesterone caproate: CYP2A6

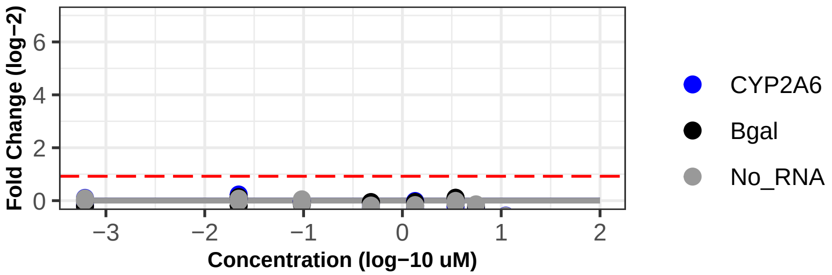


## Hydroxyprogesterone caproate: CYP2D6

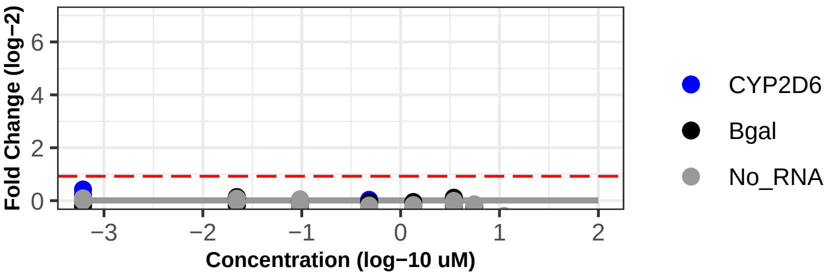


## Hydroxyprogesterone caproate: CYP2B6

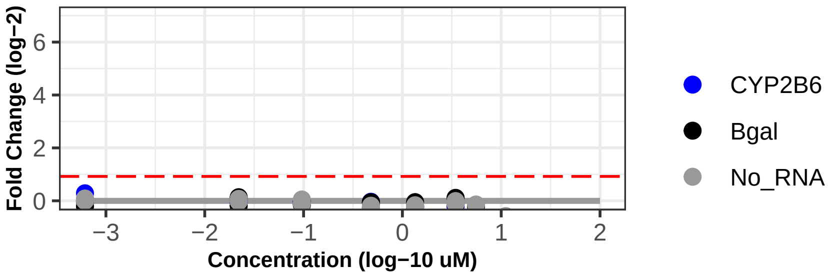


## Hydroxyprogesterone caproate: CYP2E1

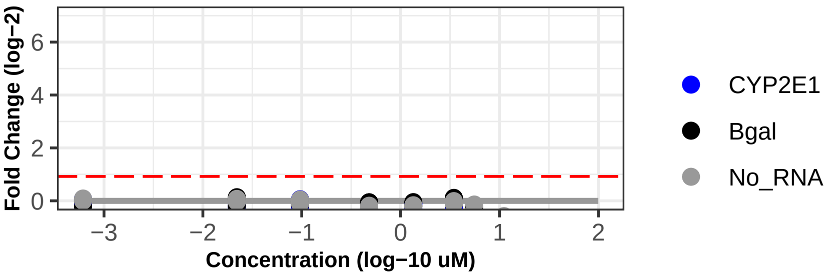


## Hydroxyprogesterone caproate: CYP2C8

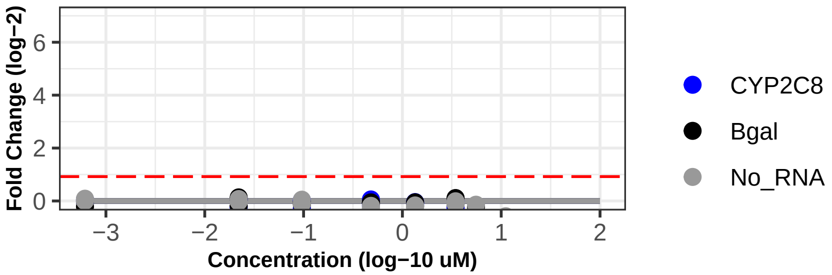


## Hydroxyprogesterone caproate: CYP2J2

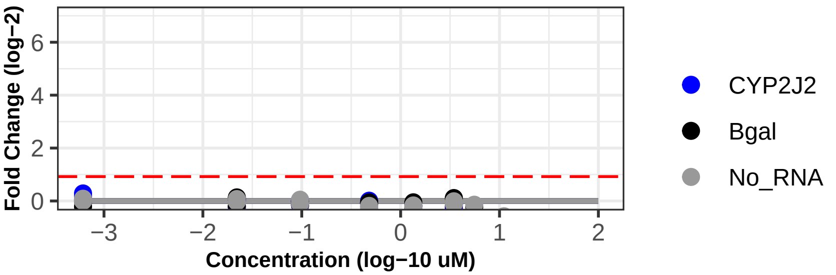


## Hydroxyprogesterone caproate: CYP2C9

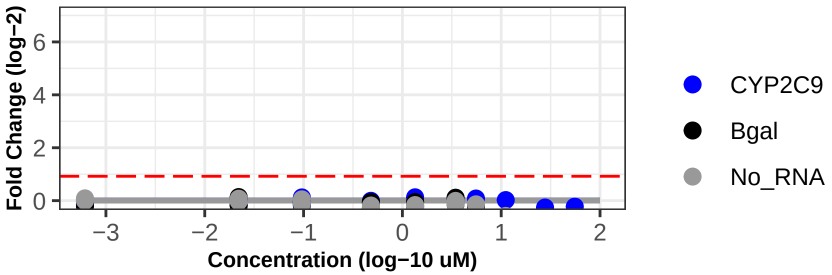


## Hydroxyprogesterone caproate: CYP3A4

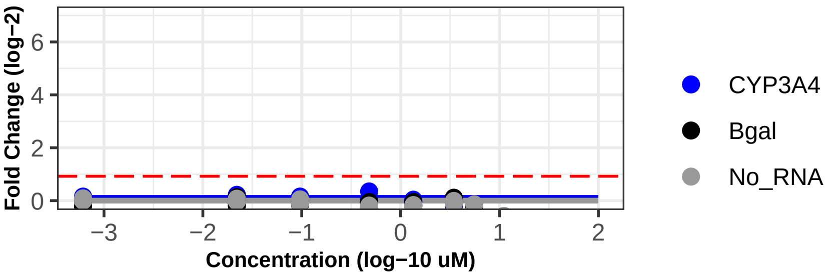

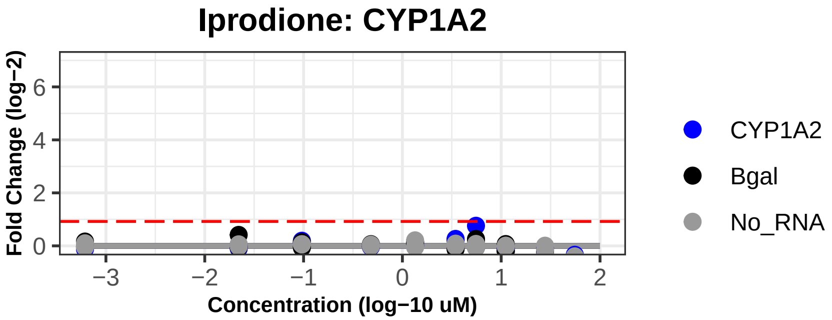
Iprodione: CYP1A2
Fold Change (log−2)
Concentration (log−10 uM)
CYP1A2
Bgal
No_RNA

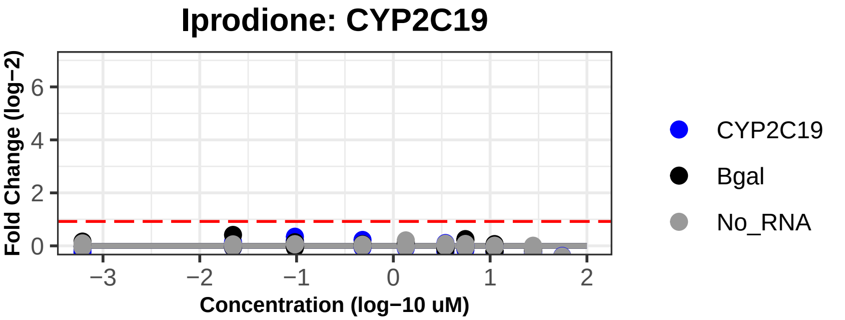
Iprodione: CYP2C19
Fold Change (log−2)
Concentration (log−10 uM)
CYP2C19
Bgal
No_RNA

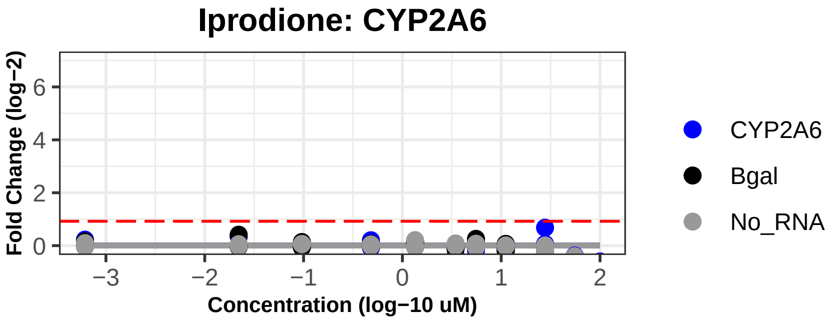
Iprodione: CYP2A6
Fold Change (log−2)
Concentration (log−10 uM)
CYP2A6
Bgal
No_RNA

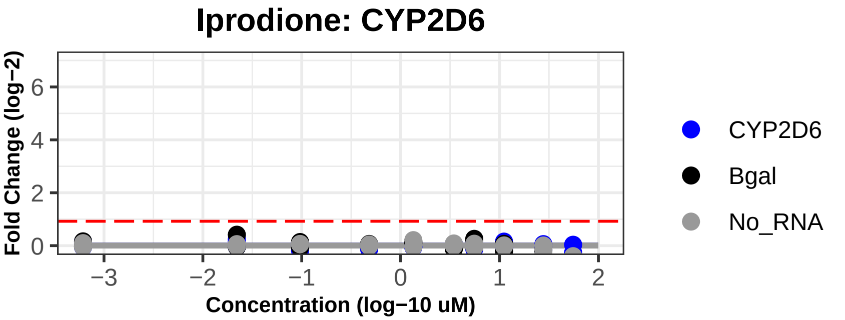
Iprodione: CYP2D6
Fold Change (log−2)
Concentration (log−10 uM)
CYP2D6
Bgal
No_RNA

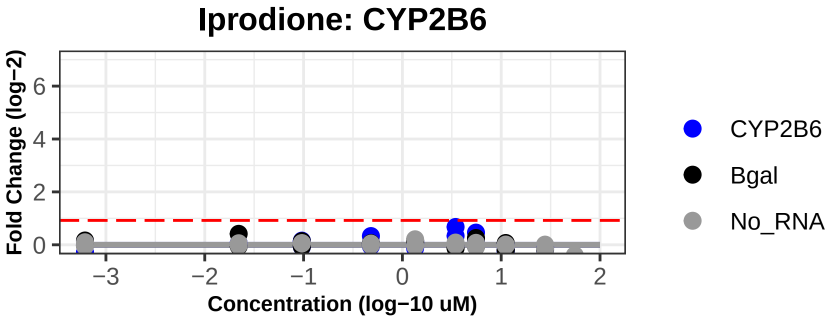
Iprodione: CYP2B6
Fold Change (log−2)
Concentration (log−10 uM)
CYP2B6
Bgal
No_RNA

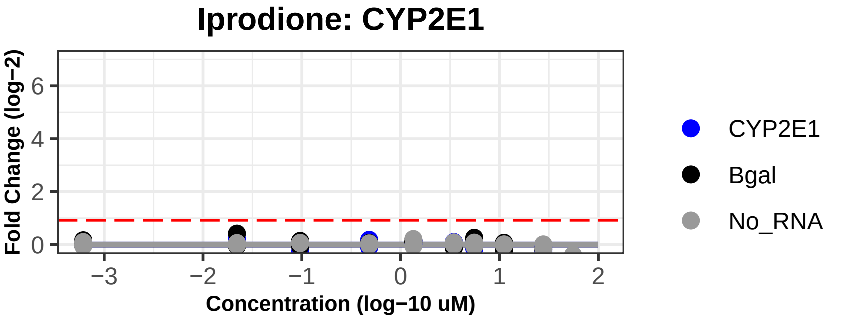
Iprodione: CYP2E1
Fold Change (log−2)
Concentration (log−10 uM)
CYP2E1
Bgal
No_RNA

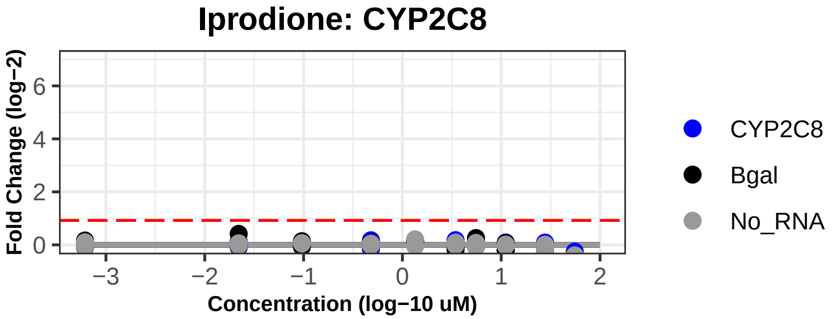
Iprodione: CYP2C8
Fold Change (log−2)
Concentration (log−10 uM)
CYP2C8
Bgal
No_RNA

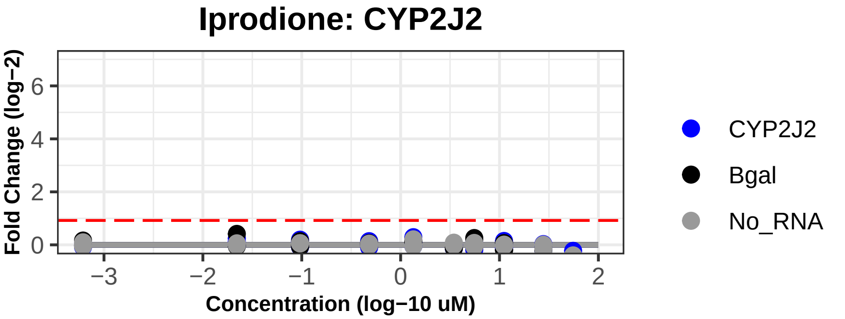
Iprodione: CYP2J2
Fold Change (log−2)
Concentration (log−10 uM)
CYP2J2
Bgal
No_RNA

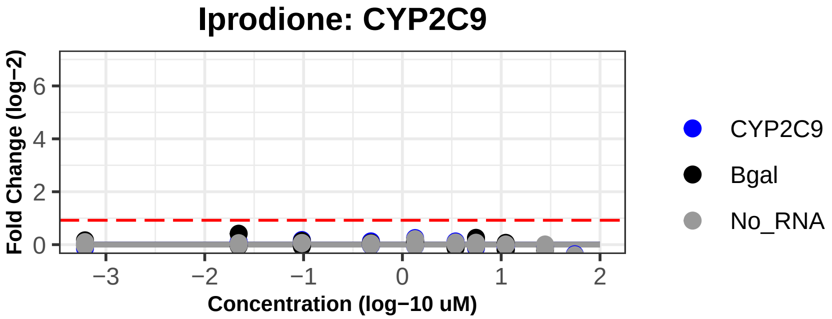
Iprodione: CYP2C9
Fold Change (log−2)
Concentration (log−10 uM)
CYP2C9
Bgal
No_RNA

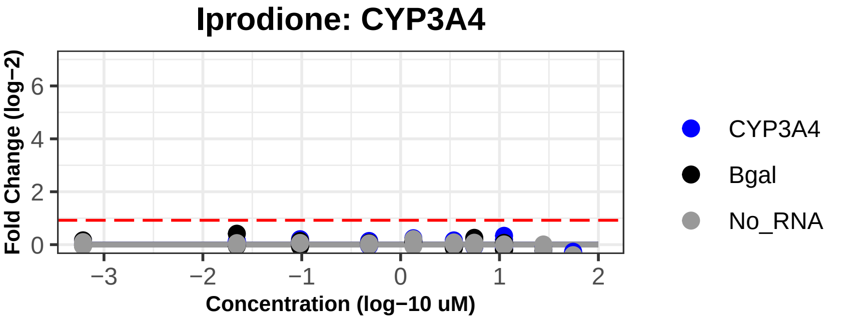
Iprodione: CYP3A4
Fold Change (log−2)
Concentration (log−10 uM)
CYP3A4
Bgal
No_RNA

## Kaempferol: CYP1A2

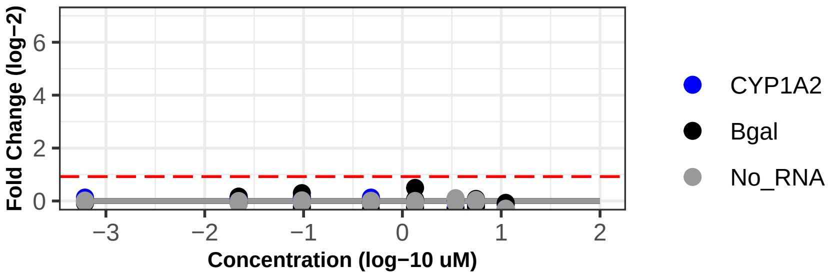


## Kaempferol: CYP2C19

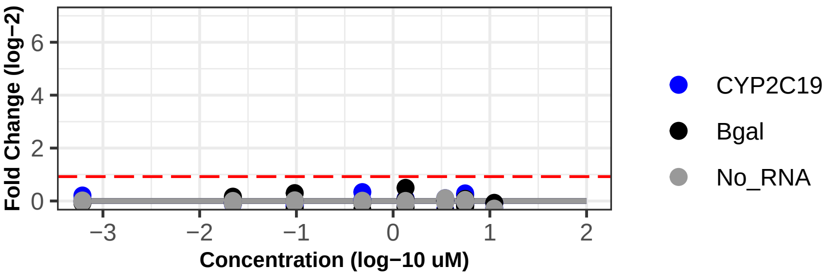


## Kaempferol: CYP2A6

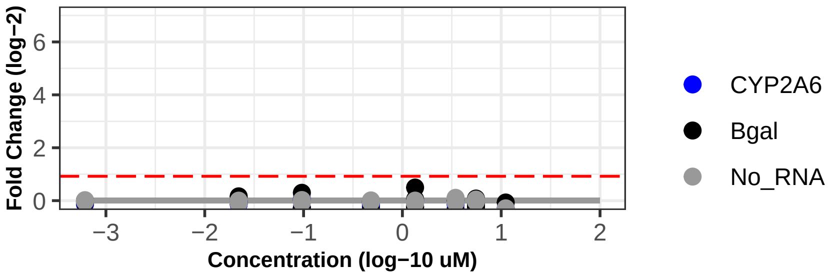


## Kaempferol: CYP2D6

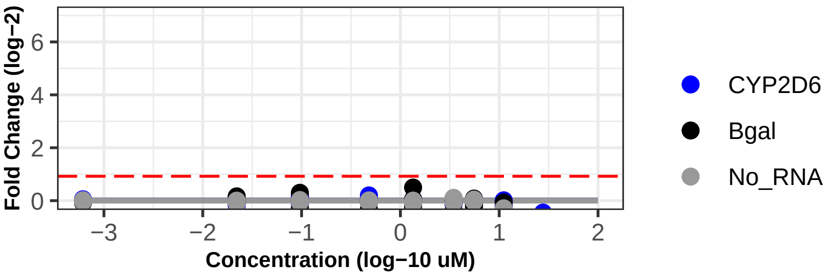


## Kaempferol: CYP2B6

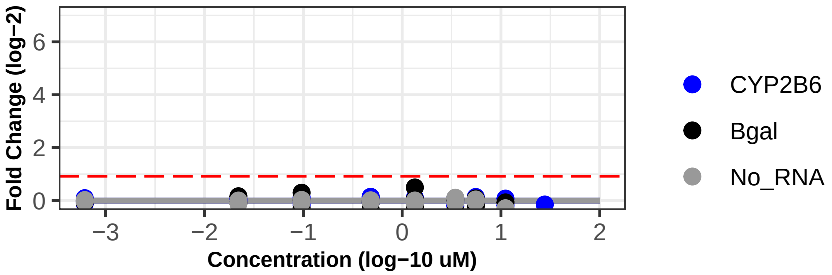


## Kaempferol: CYP2E1

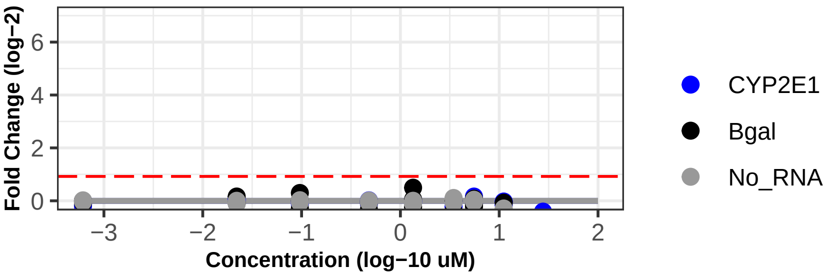


## Kaempferol: CYP2C8

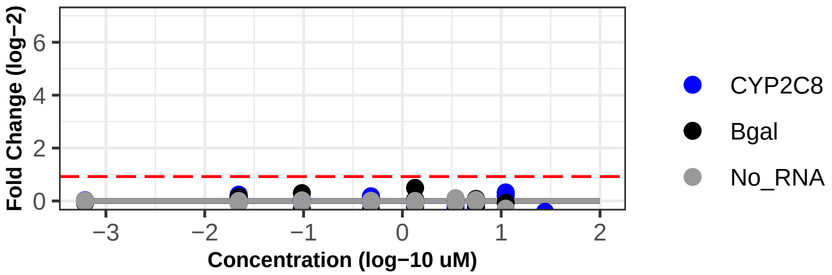


## Kaempferol: CYP2J2

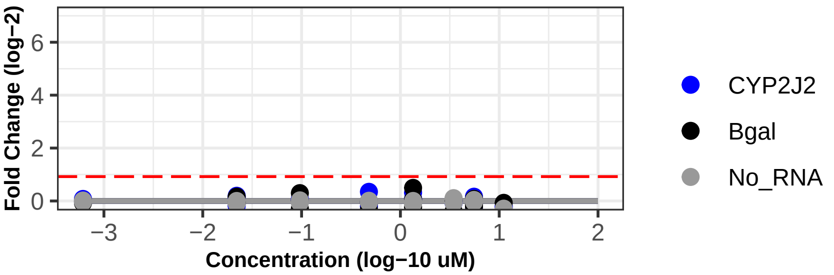


## Kaempferol: CYP2C9

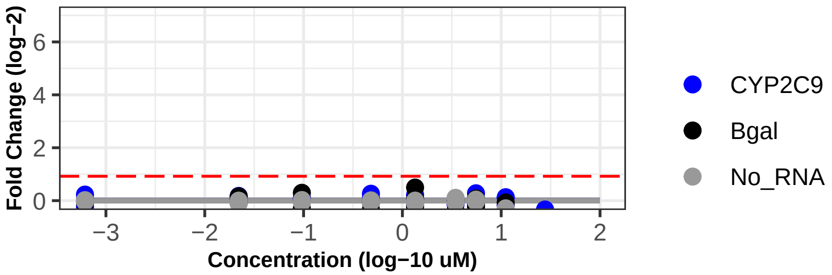


## Kaempferol: CYP3A4

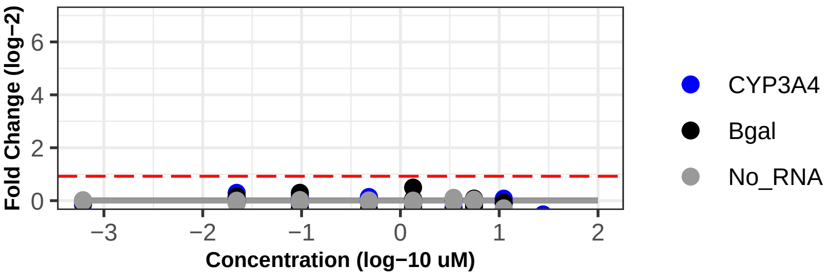

## Letrozole: CYP1A2

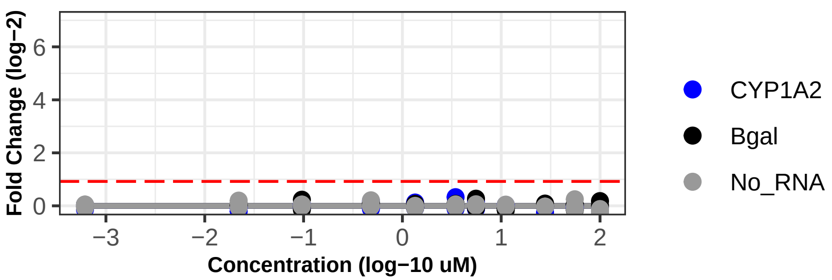


## Letrozole: CYP2C19

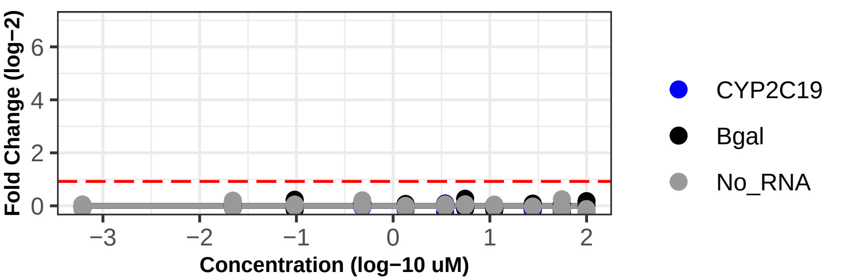


## Letrozole: CYP2A6

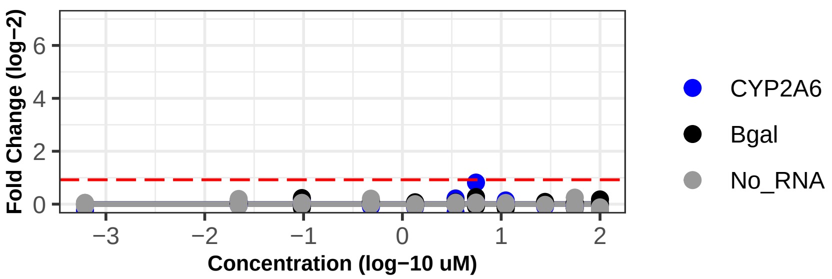


## Letrozole: CYP2D6

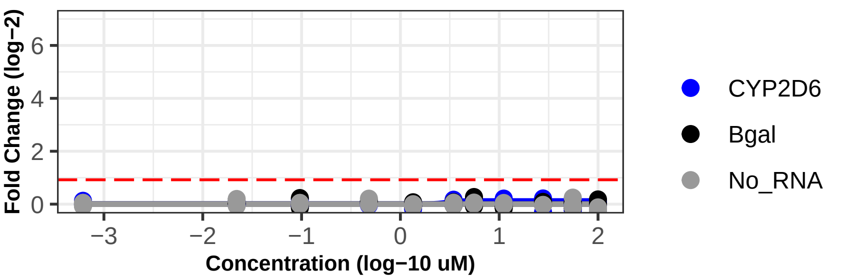


## Letrozole: CYP2B6

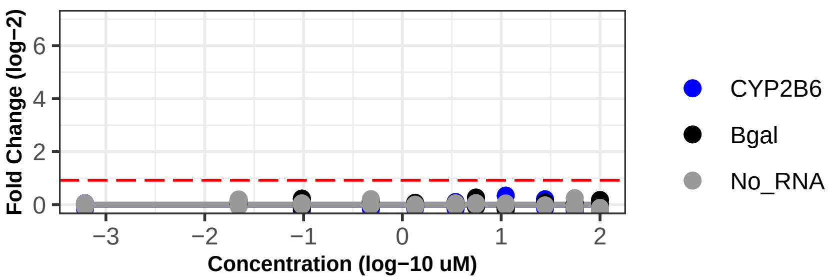


## Letrozole: CYP2E1

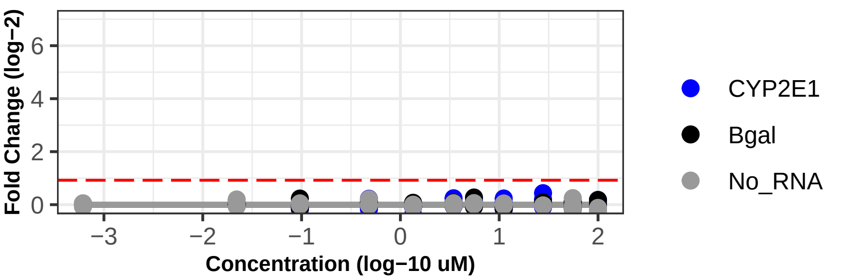


## Letrozole: CYP2C8

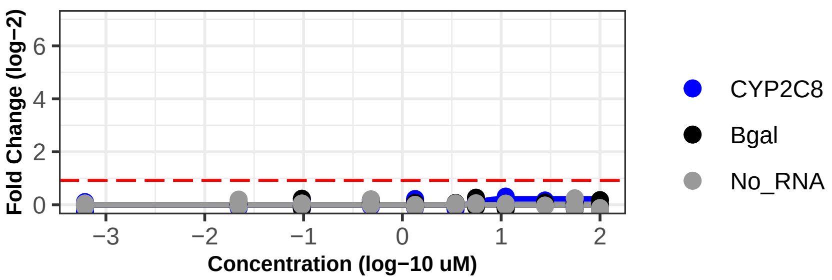


## Letrozole: CYP2J2

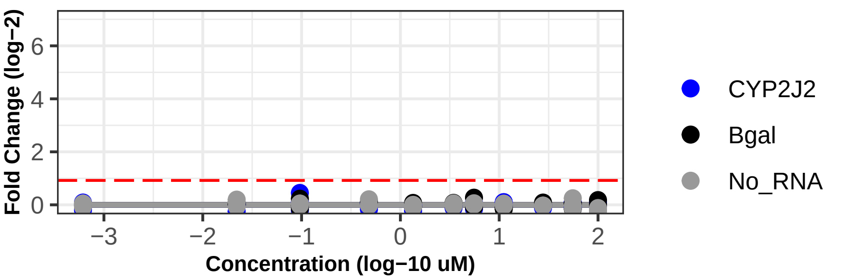


## Letrozole: CYP2C9

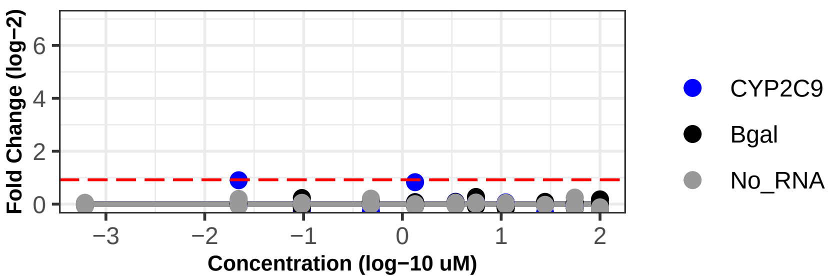


## Letrozole: CYP3A4

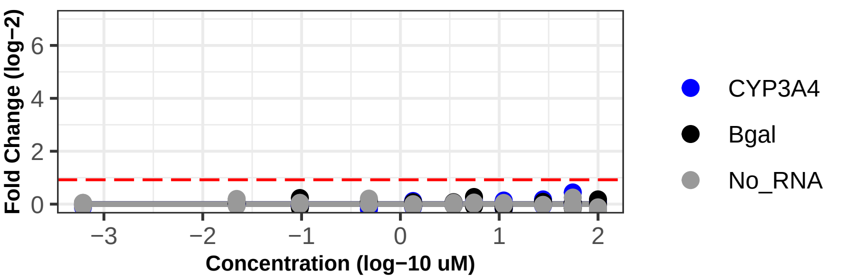

## Levonorgestrel: CYP1A2

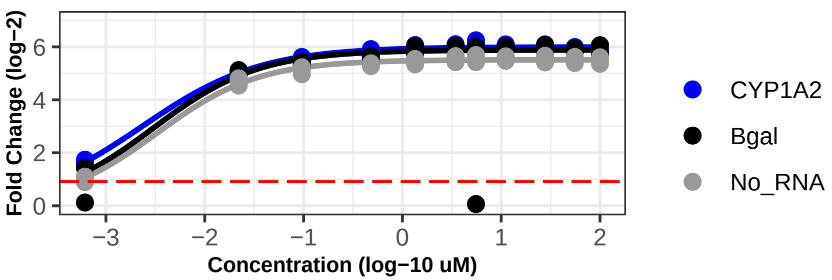


## Levonorgestrel: CYP2C19

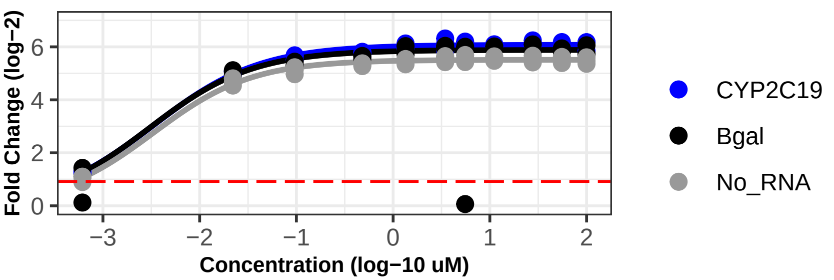


## Levonorgestrel: CYP2A6

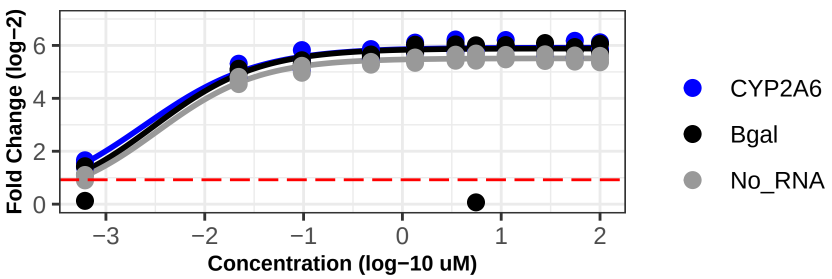


## Levonorgestrel: CYP2D6

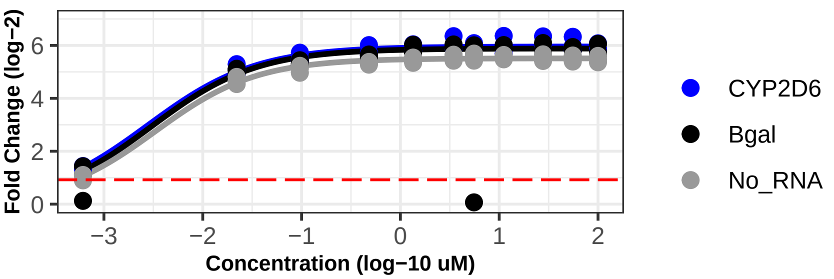


## Levonorgestrel: CYP2B6

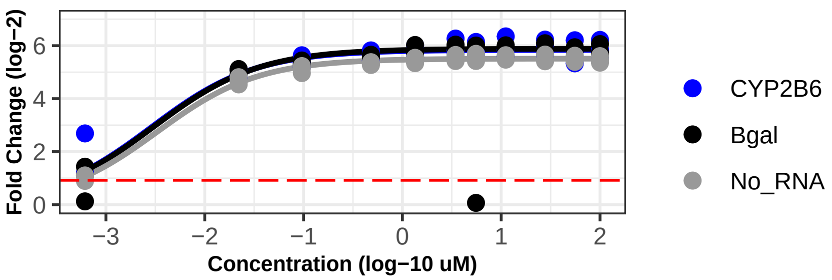


## Levonorgestrel: CYP2E1

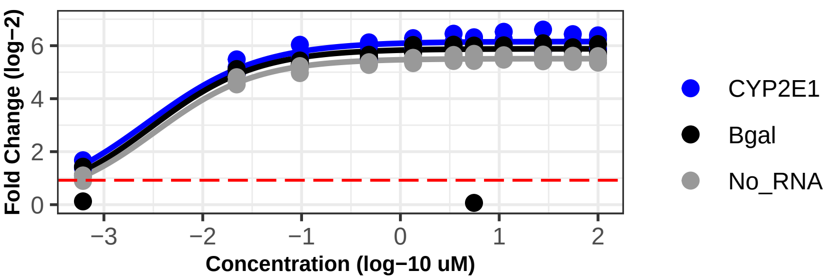


## Levonorgestrel: CYP2C8

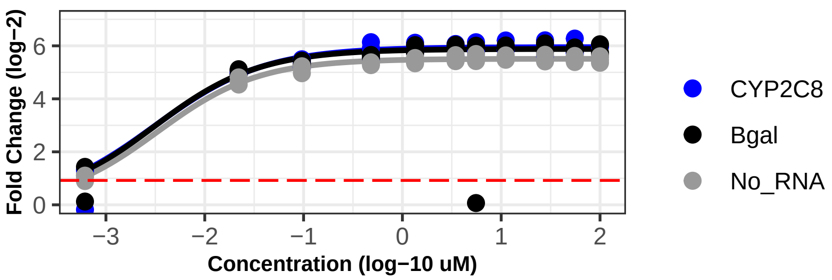


## Levonorgestrel: CYP2J2

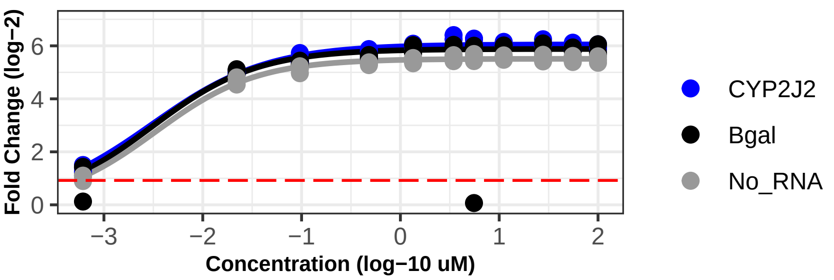


## Levonorgestrel: CYP2C9

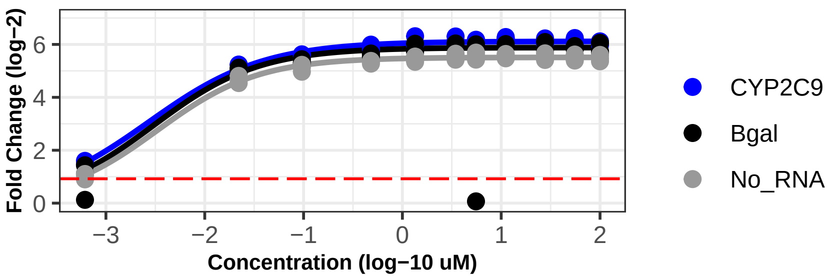


## Levonorgestrel: CYP3A4

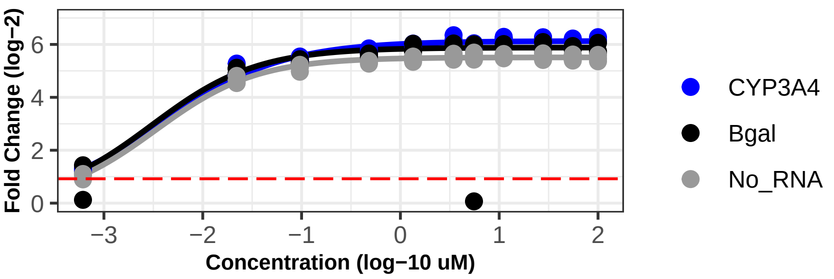

Linuron: CYP1A2

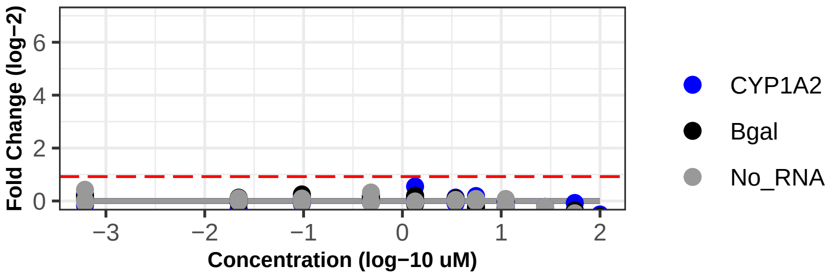


Linuron: CYP2C19

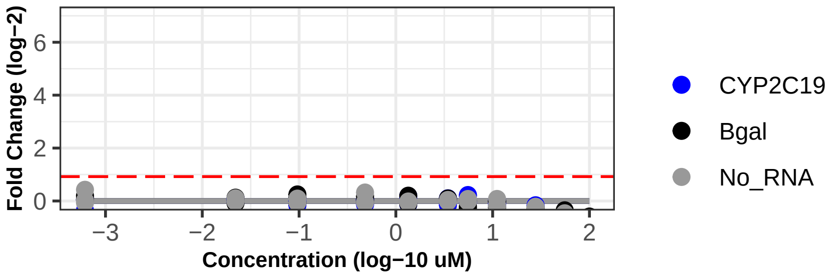


Linuron: CYP2A6

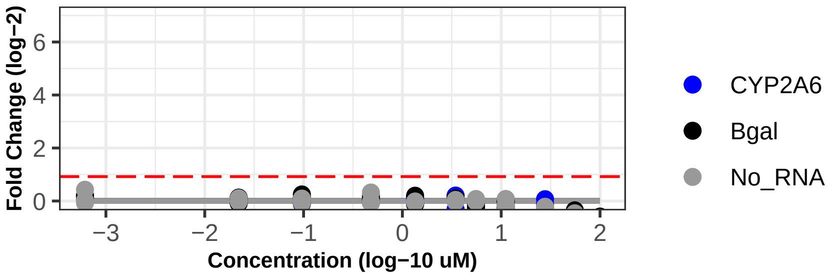


Linuron: CYP2D6

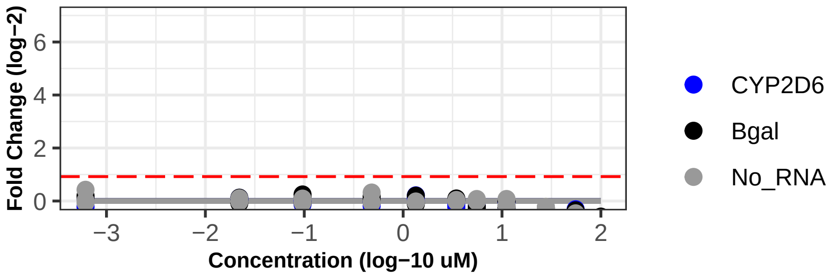


Linuron: CYP2B6

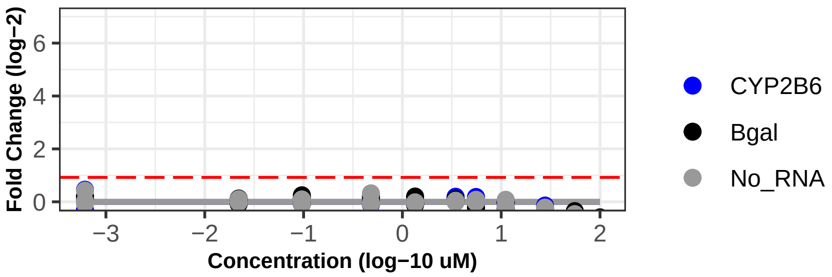


Linuron: CYP2E1

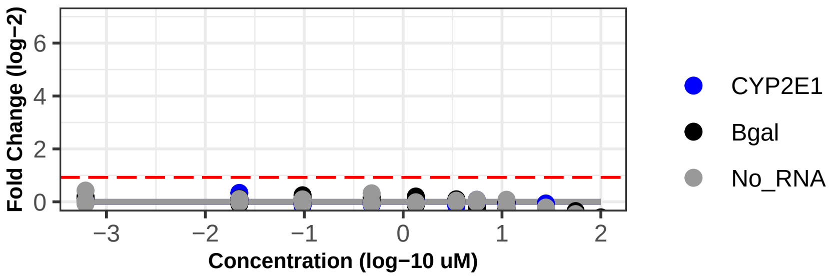


Linuron: CYP2C8

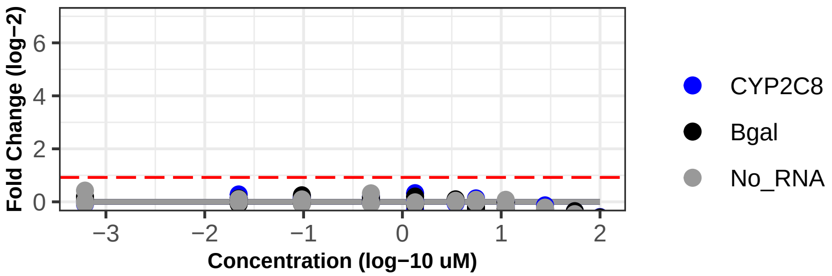


Linuron: CYP2J2

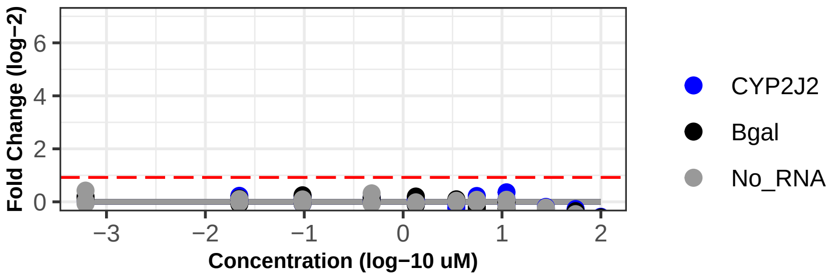


Linuron: CYP2C9

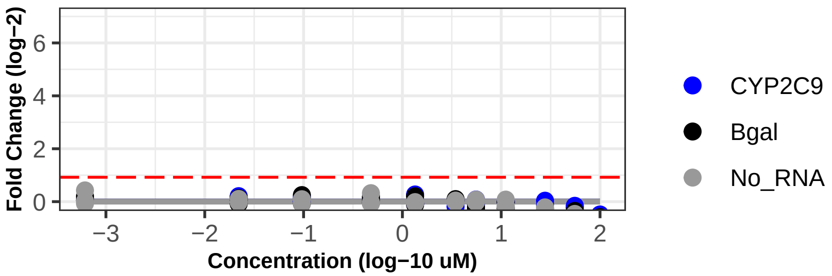


Linuron: CYP3A4

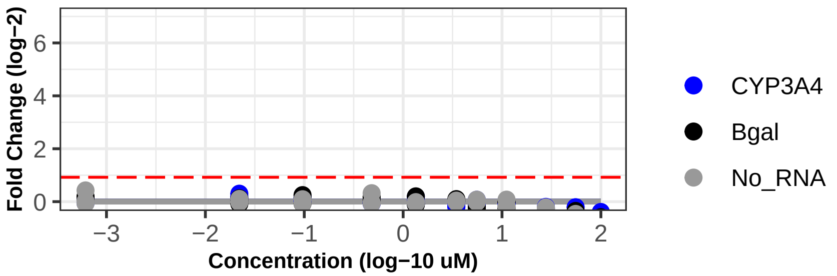

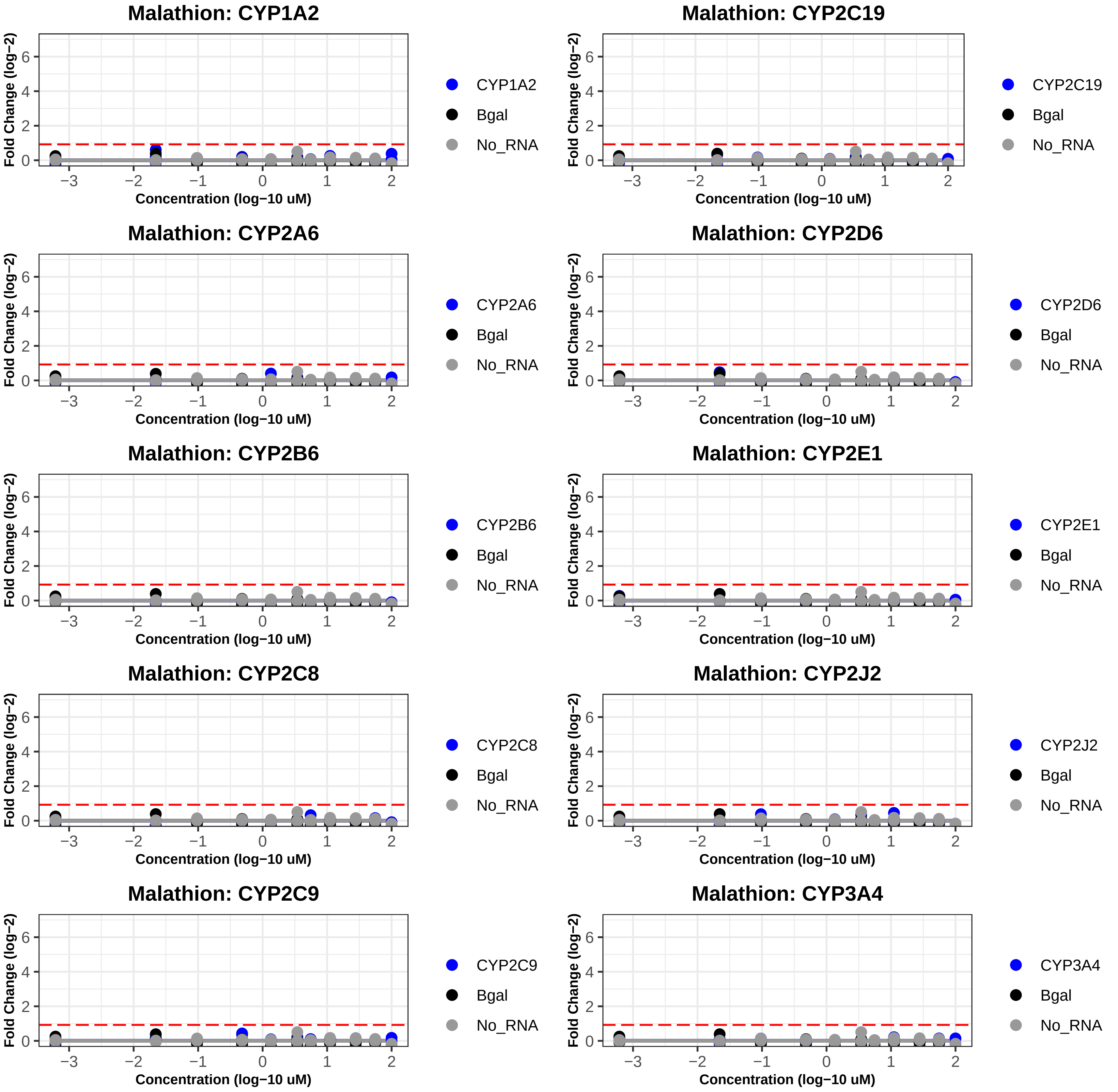
Malathion: CYP1A2
Fold Change (log−2)
Concentration (log−10 uM)
CYP1A2
Bgal
No_RNA
Malathion: CYP2C19
CYP2C19
Malathion: CYP2A6
CYP2A6
Malathion: CYP2D6
CYP2D6
Malathion: CYP2B6
CYP2B6
Malathion: CYP2E1
CYP2E1
Malathion: CYP2C8
CYP2C8
Malathion: CYP2J2
CYP2J2
Malathion: CYP2C9
CYP2C9
Malathion: CYP3A4
CYP3A4

## Mestranol: CYP1A2

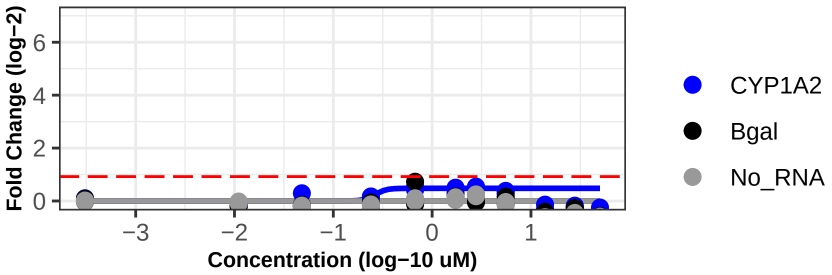


## Mestranol: CYP2C19

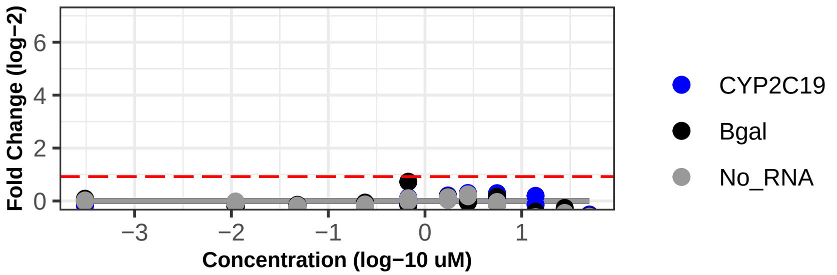


## Mestranol: CYP2A6

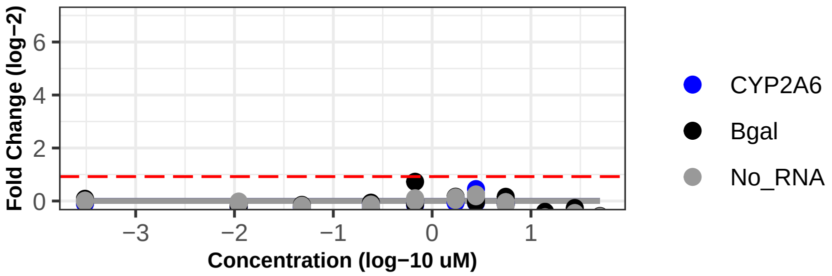


## Mestranol: CYP2D6

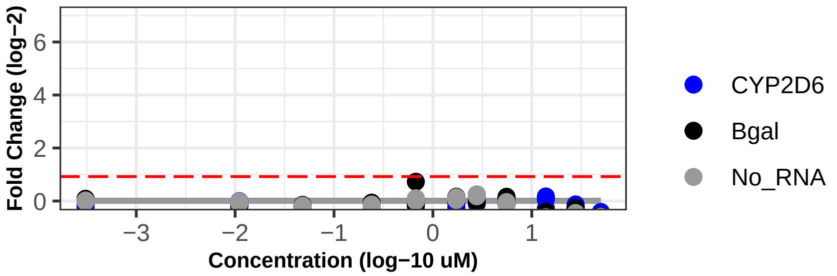


## Mestranol: CYP2B6

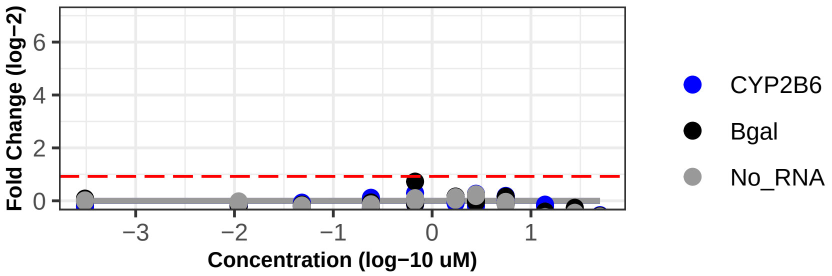


## Mestranol: CYP2E1

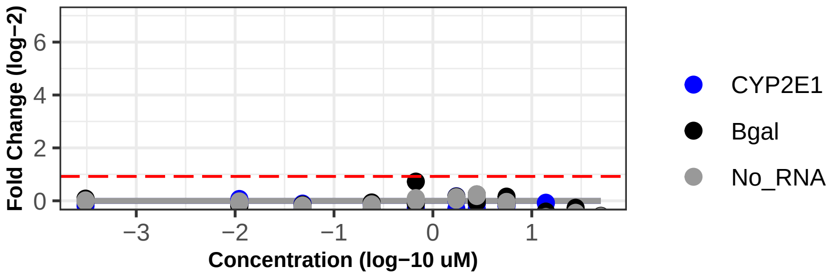


## Mestranol: CYP2C8

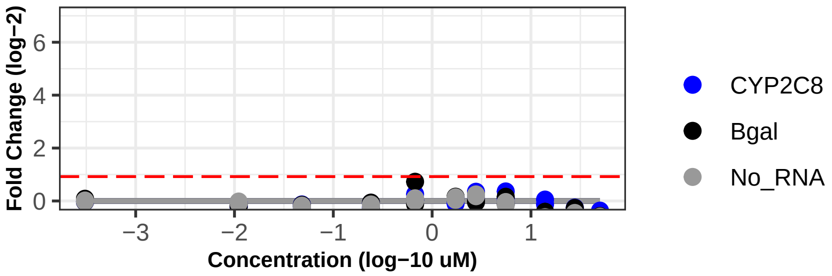


## Mestranol: CYP2J2

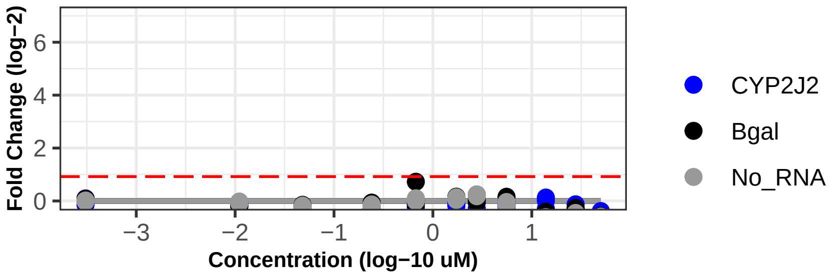


## Mestranol: CYP2C9

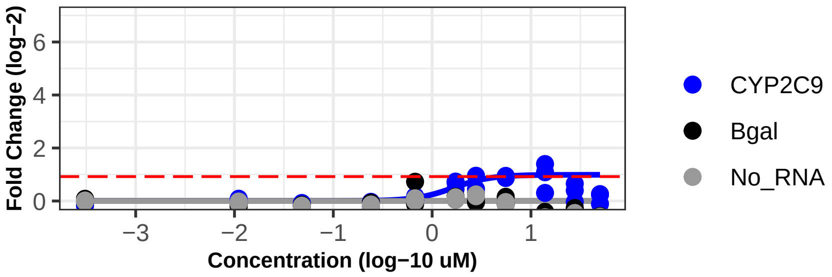


## Mestranol: CYP3A4

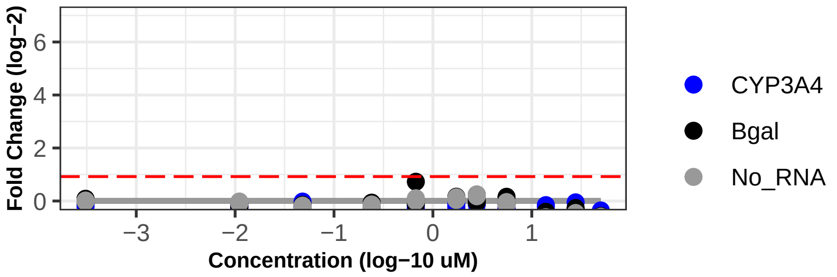

## Metalaxyl: CYP1A2

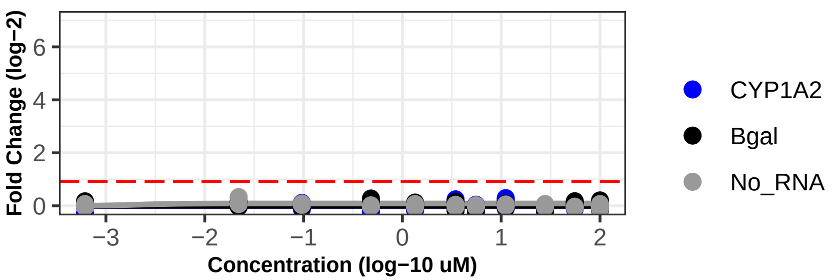


## Metalaxyl: CYP2C19

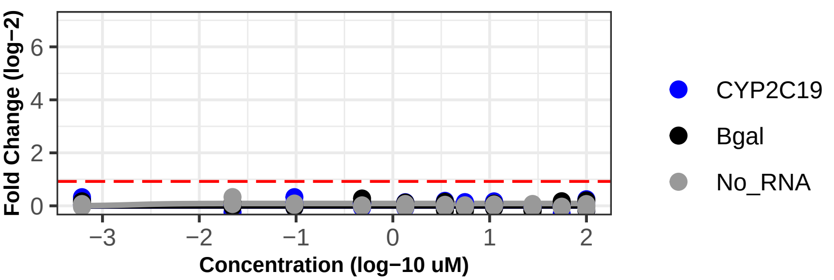


## Metalaxyl: CYP2A6

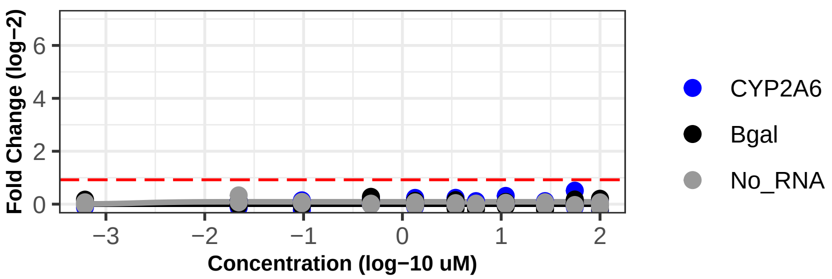


## Metalaxyl: CYP2D6

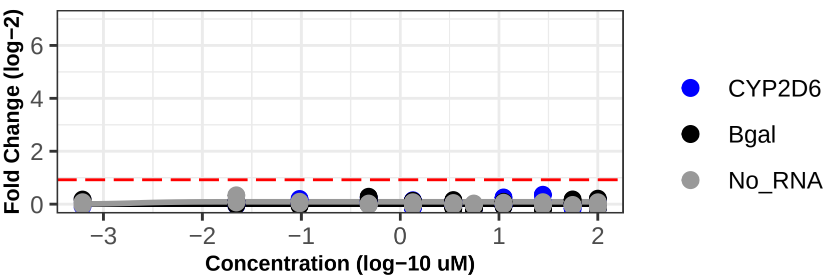


## Metalaxyl: CYP2B6

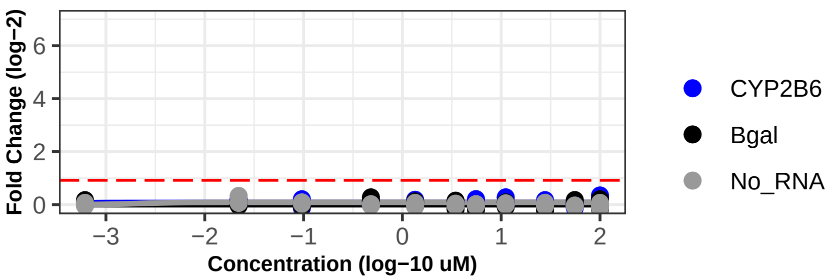


## Metalaxyl: CYP2E1

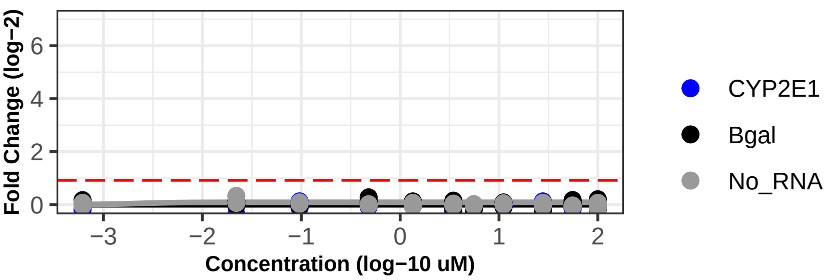


## Metalaxyl: CYP2C8

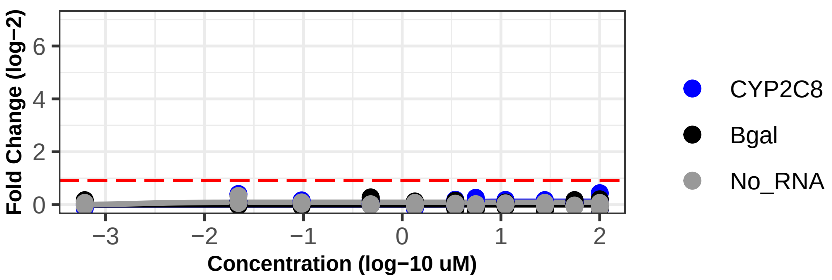


## Metalaxyl: CYP2J2

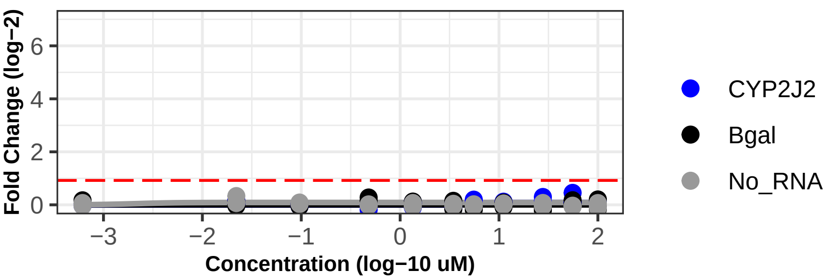


## Metalaxyl: CYP2C9

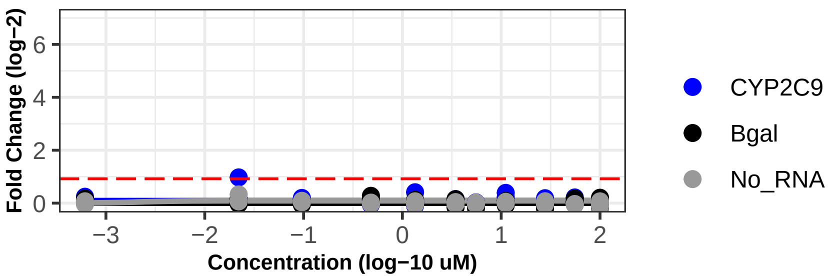


## Metalaxyl: CYP3A4

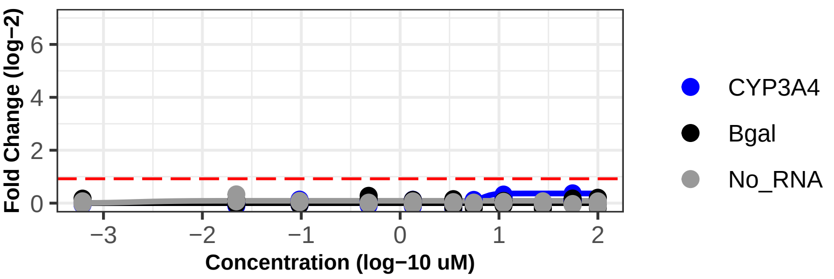

## Methomyl: CYP1A2

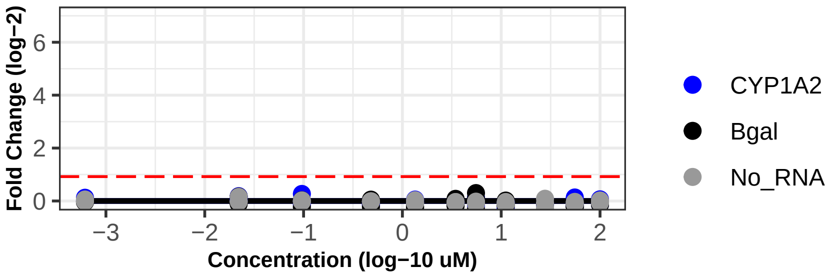


## Methomyl: CYP2C19

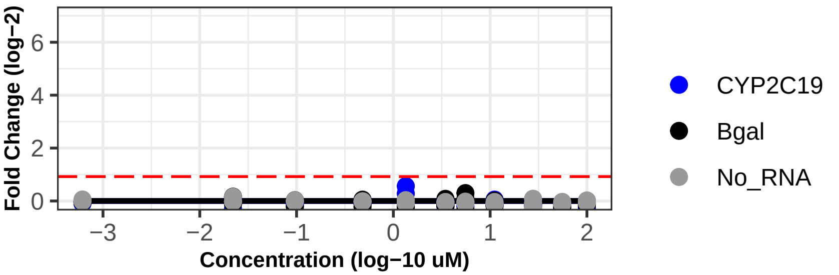


## Methomyl: CYP2A6

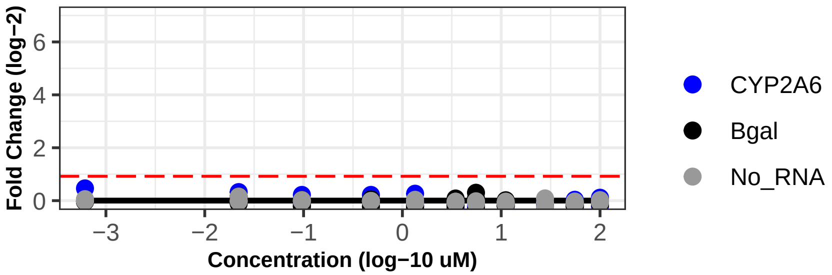


## Methomyl: CYP2D6

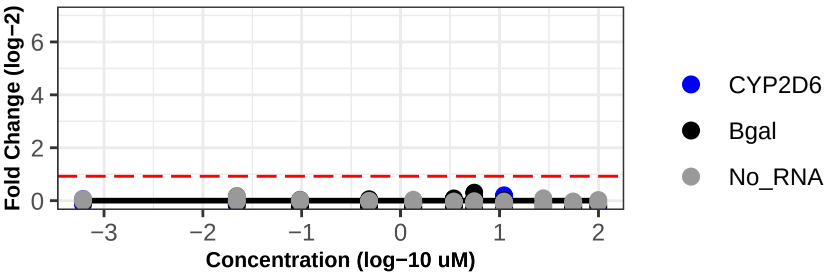


## Methomyl: CYP2B6

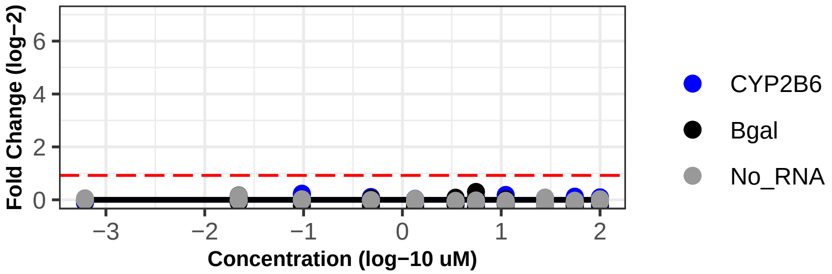


## Methomyl: CYP2E1

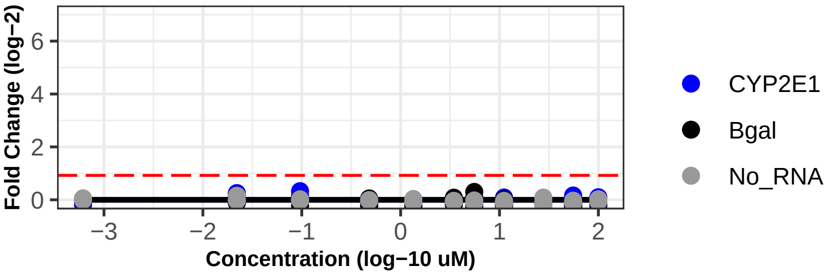


## Methomyl: CYP2C8

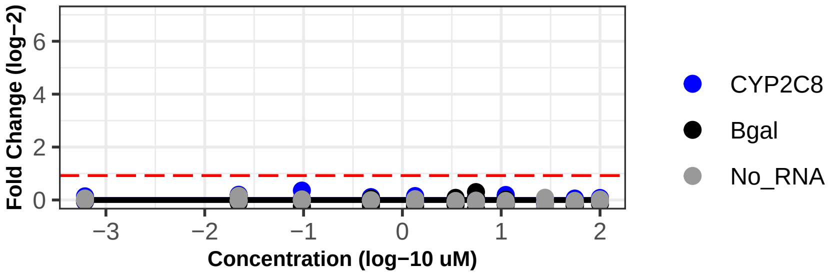


## Methomyl: CYP2J2

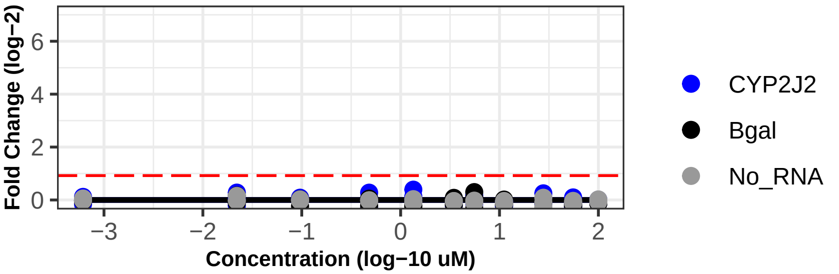


## Methomyl: CYP2C9

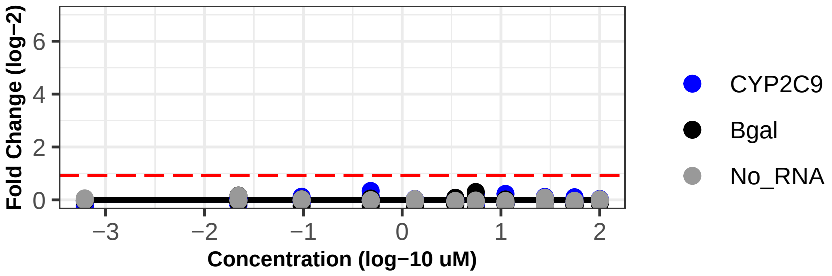


## Methomyl: CYP3A4

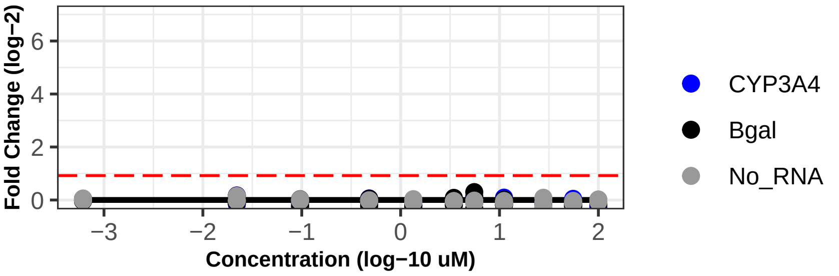

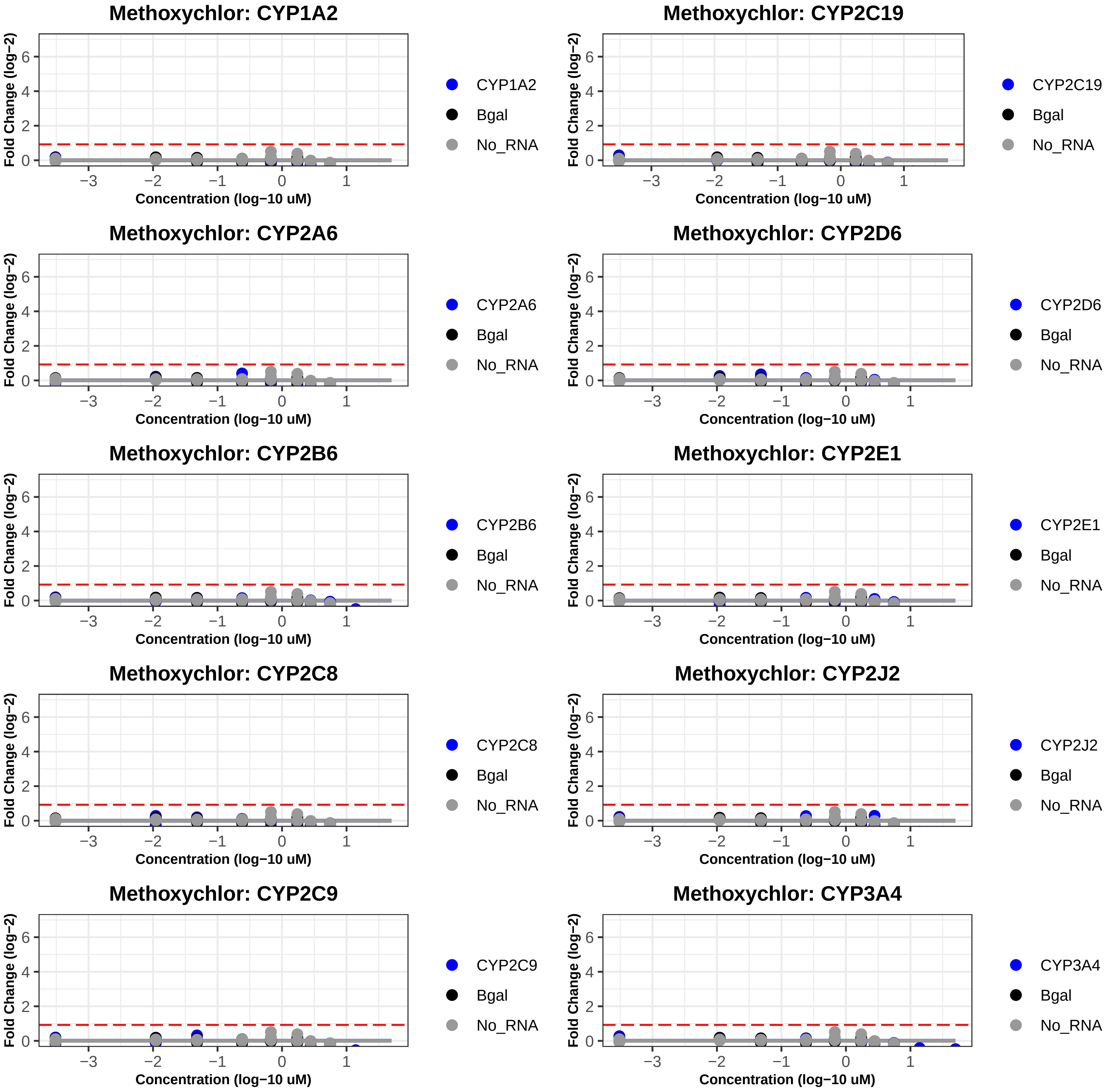
Methoxychlor: CYP1A2
Fold Change (log−2)
Concentration (log−10 uM)
CYP1A2
Bgal
No_RNA
Methoxychlor: CYP2C19
CYP2C19
Methoxychlor: CYP2A6
CYP2A6
Methoxychlor: CYP2D6
CYP2D6
Methoxychlor: CYP2B6
CYP2B6
Methoxychlor: CYP2E1
CYP2E1
Methoxychlor: CYP2C8
CYP2C8
Methoxychlor: CYP2J2
CYP2J2
Methoxychlor: CYP2C9
CYP2C9
Methoxychlor: CYP3A4
CYP3A4

## Metolachlor: CYP1A2

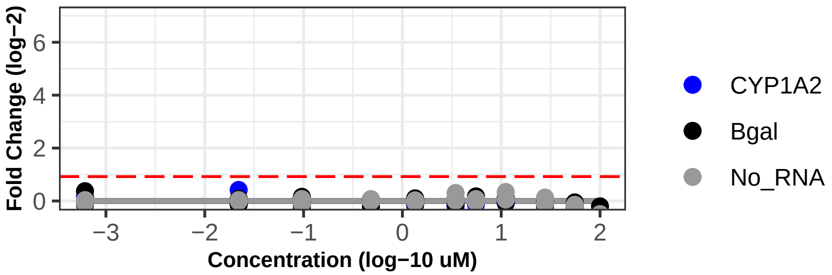


## Metolachlor: CYP2A6

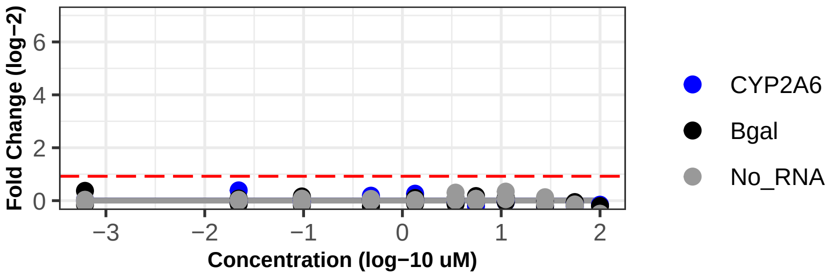


## Metolachlor: CYP2B6

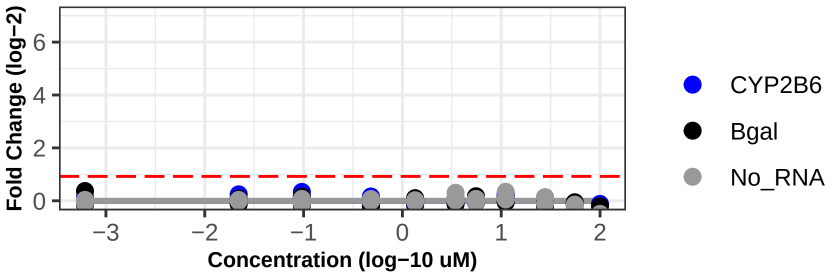


## Metolachlor: CYP2C8

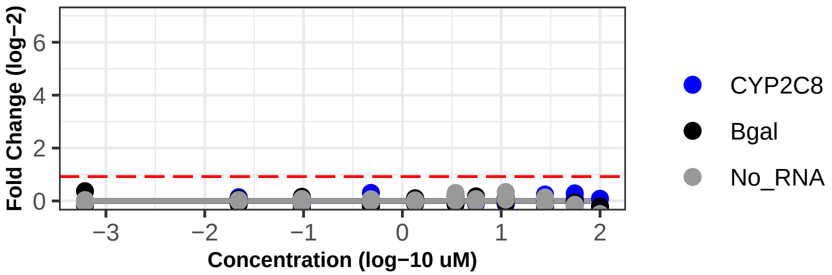


## Metolachlor: CYP2C9

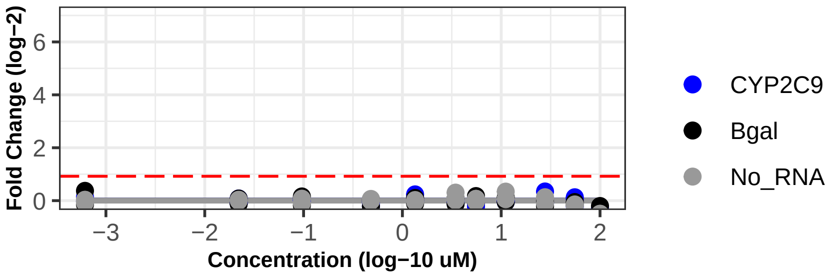


## Metolachlor: CYP2C19

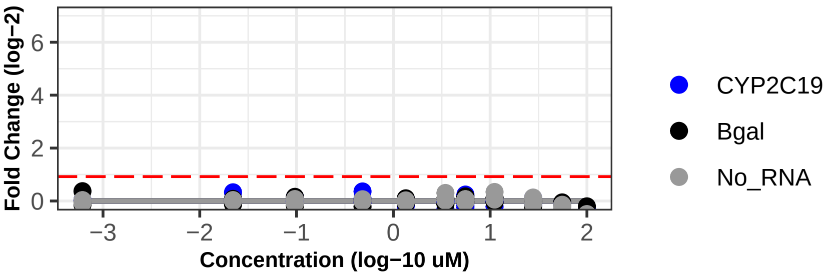


## Metolachlor: CYP2D6

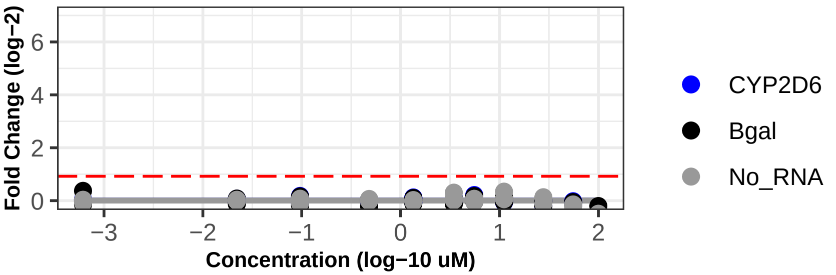


## Metolachlor: CYP2E1

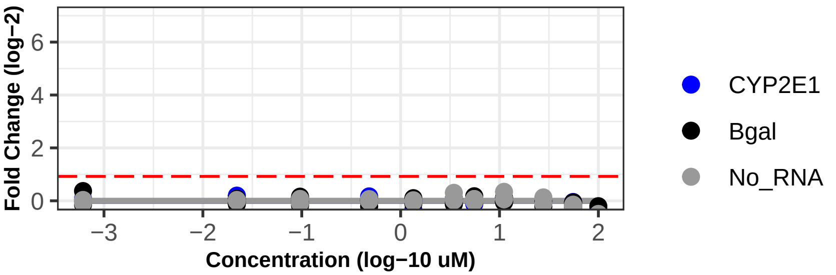


## Metolachlor: CYP2J2

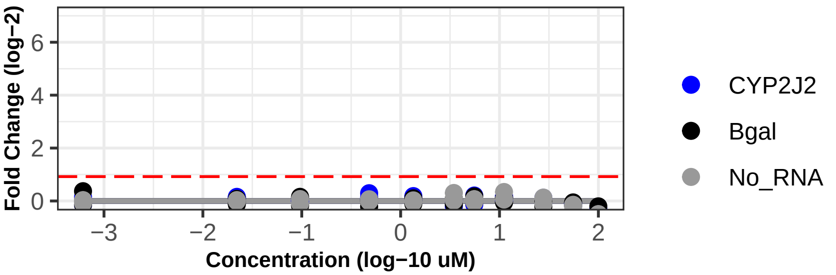


## Metolachlor: CYP3A4

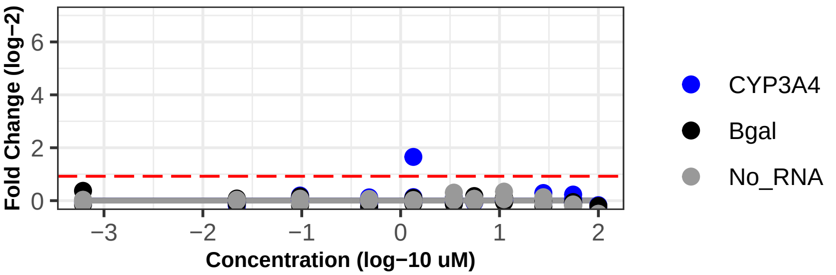

## Metribuzin: CYP1A2

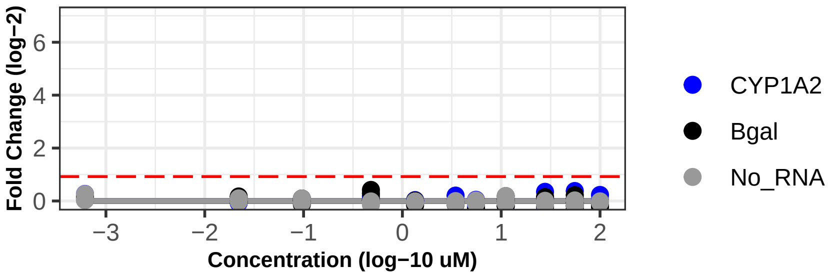


## Metribuzin: CYP2C19

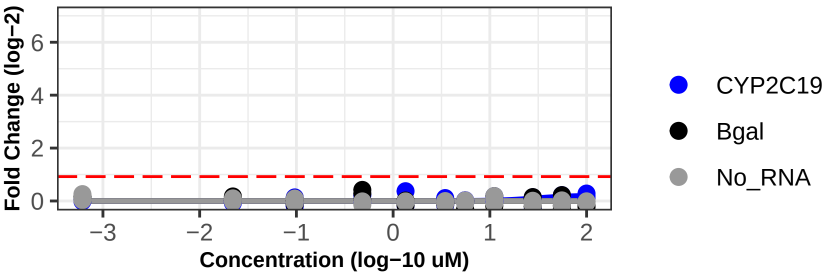


## Metribuzin: CYP2A6

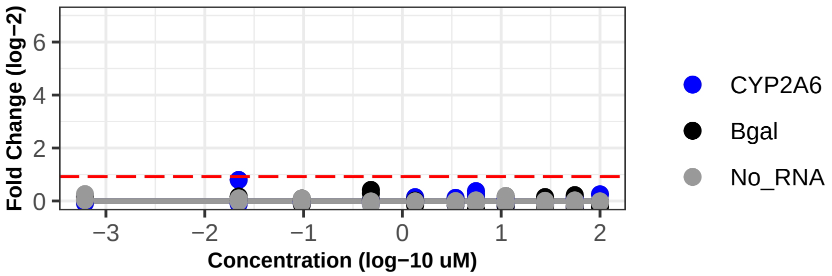


## Metribuzin: CYP2D6

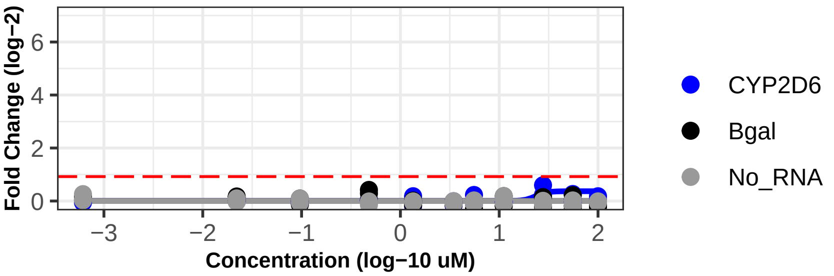


## Metribuzin: CYP2B6

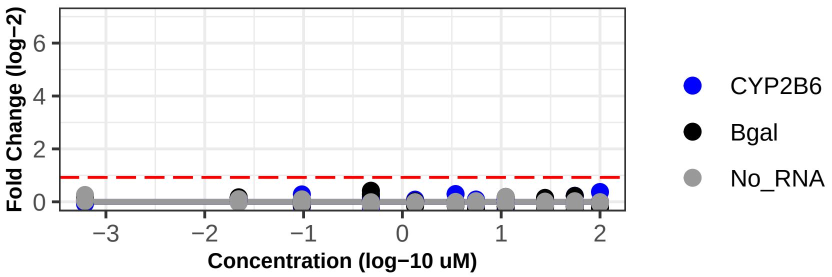


## Metribuzin: CYP2E1

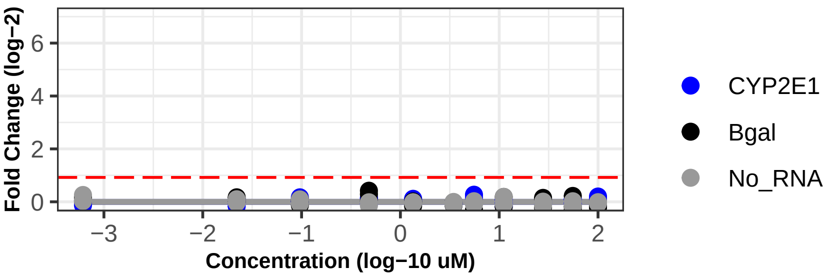


## Metribuzin: CYP2C8

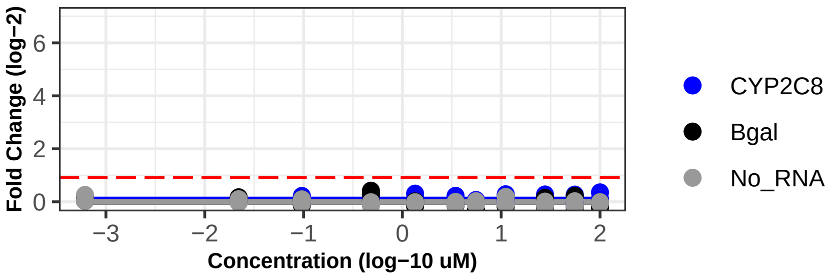


## Metribuzin: CYP2J2

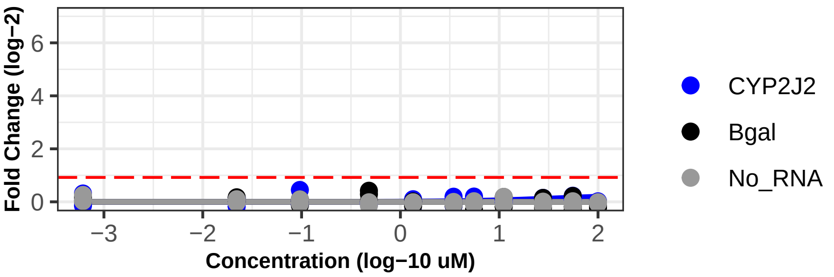


## Metribuzin: CYP2C9

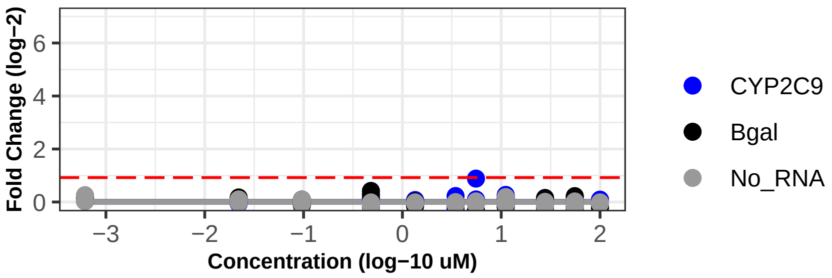


## Metribuzin: CYP3A4

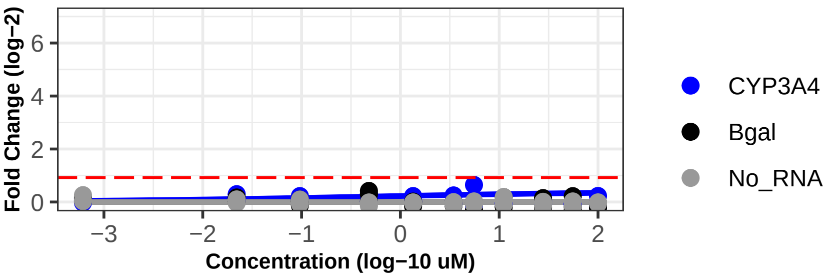

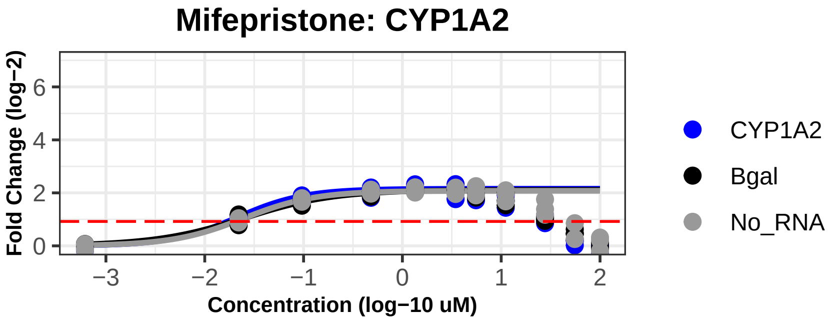
Mifepristone: CYP1A2
Fold Change (log−2)
Concentration (log−10 uM)
CYP1A2
Bgal
No_RNA

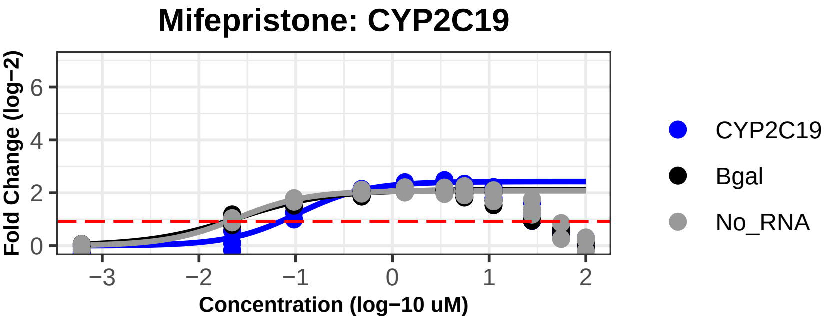
Mifepristone: CYP2C19
Fold Change (log−2)
Concentration (log−10 uM)
CYP2C19
Bgal
No_RNA

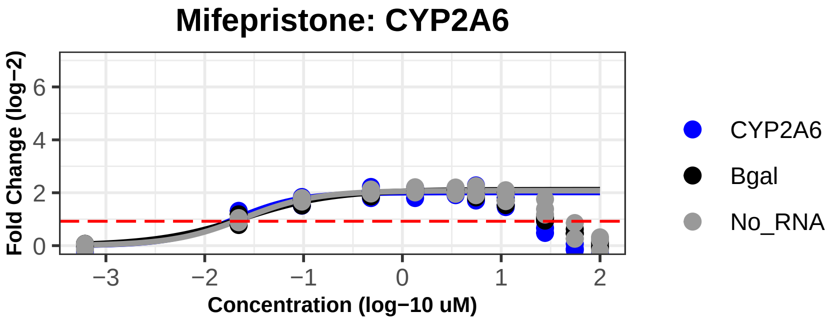
Mifepristone: CYP2A6
Fold Change (log−2)
Concentration (log−10 uM)
CYP2A6
Bgal
No_RNA

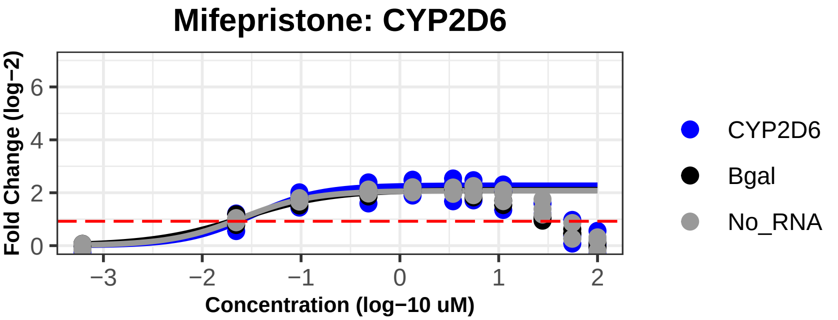
Mifepristone: CYP2D6
Fold Change (log−2)
Concentration (log−10 uM)
CYP2D6
Bgal
No_RNA

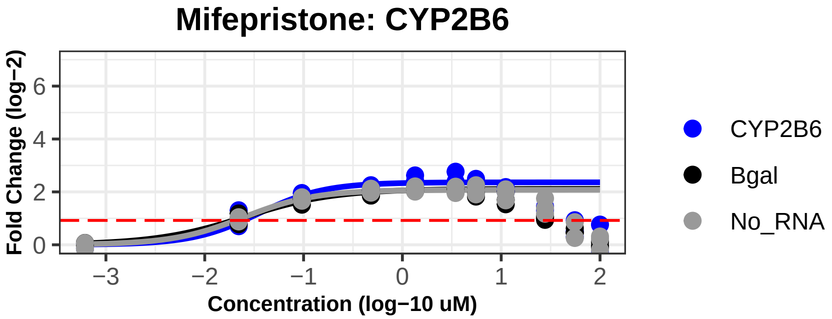
Mifepristone: CYP2B6
Fold Change (log−2)
Concentration (log−10 uM)
CYP2B6
Bgal
No_RNA

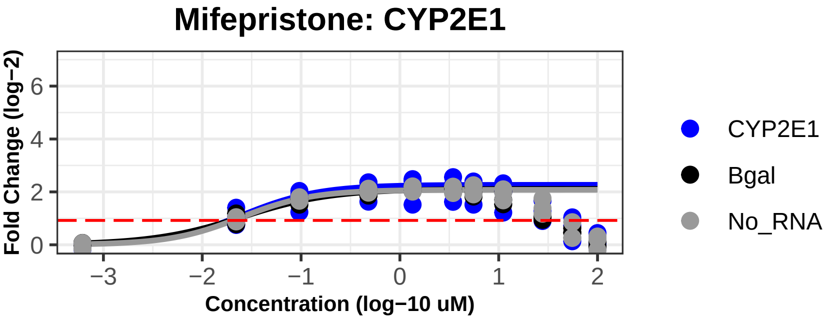
Mifepristone: CYP2E1
Fold Change (log−2)
Concentration (log−10 uM)
CYP2E1
Bgal
No_RNA

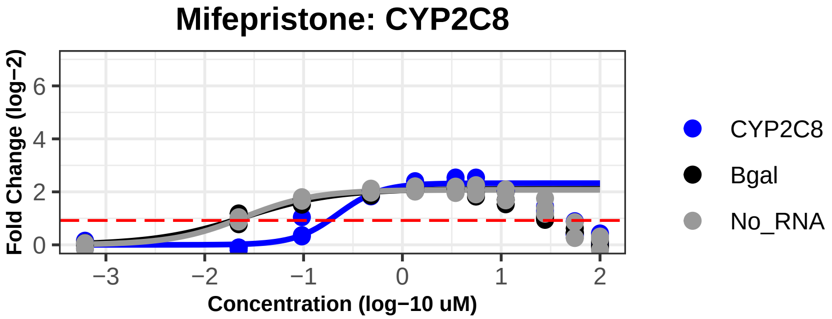
Mifepristone: CYP2C8
Fold Change (log−2)
Concentration (log−10 uM)
CYP2C8
Bgal
No_RNA

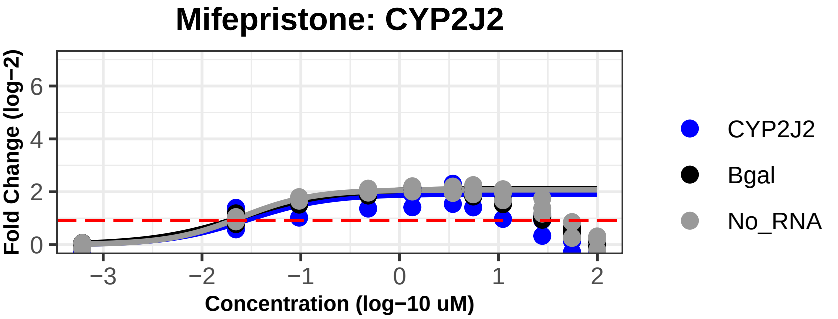
Mifepristone: CYP2J2
Fold Change (log−2)
Concentration (log−10 uM)
CYP2J2
Bgal
No_RNA

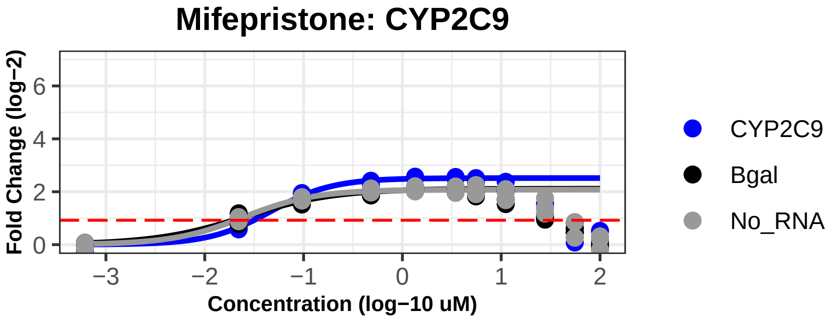
Mifepristone: CYP2C9
Fold Change (log−2)
Concentration (log−10 uM)
CYP2C9
Bgal
No_RNA

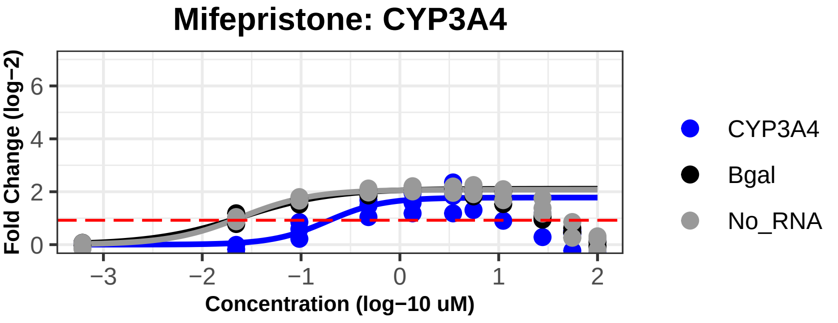
Mifepristone: CYP3A4
Fold Change (log−2)
Concentration (log−10 uM)
CYP3A4
Bgal
No_RNA

## Mono(2−ethylhexyl) phthalate: CYP1A2

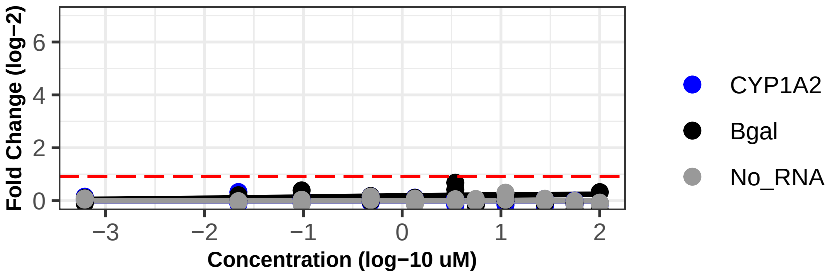


## Mono(2−ethylhexyl) phthalate: CYP2C19

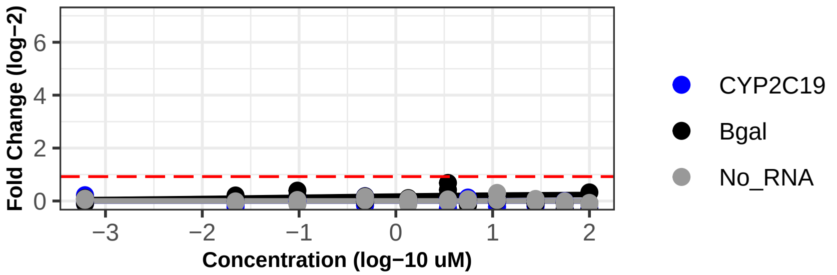


## Mono(2−ethylhexyl) phthalate: CYP2A6

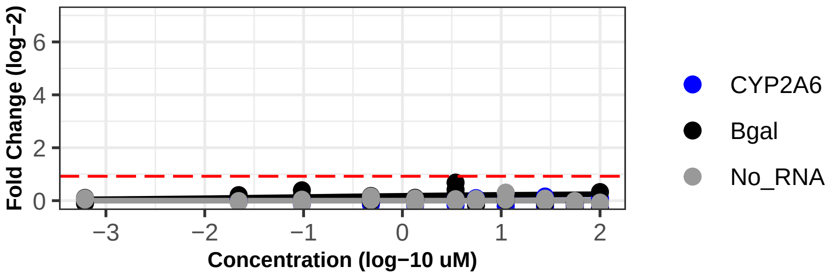


## Mono(2−ethylhexyl) phthalate: CYP2D6

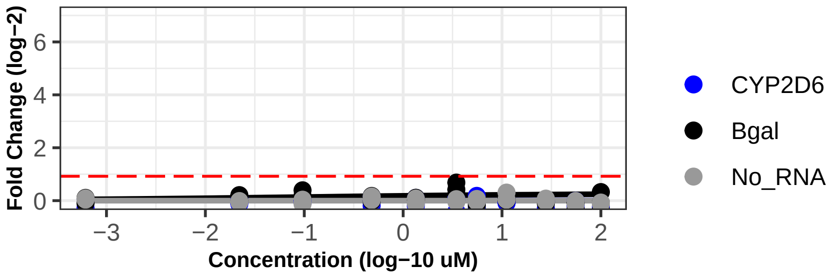


## Mono(2−ethylhexyl) phthalate: CYP2B6

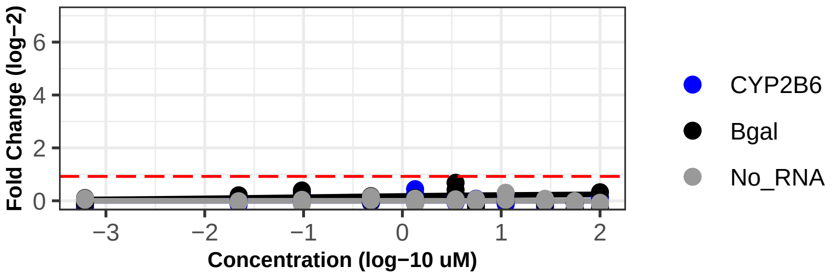


## Mono(2−ethylhexyl) phthalate: CYP2E1

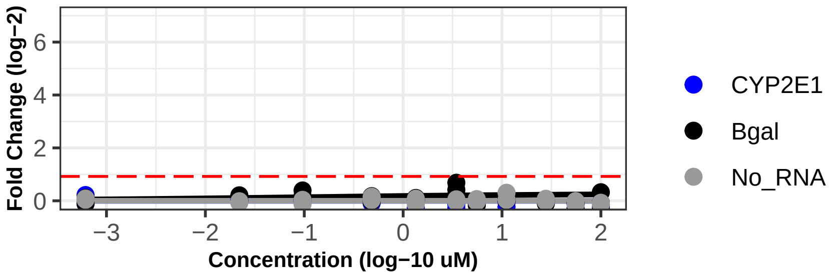


## Mono(2−ethylhexyl) phthalate: CYP2C8

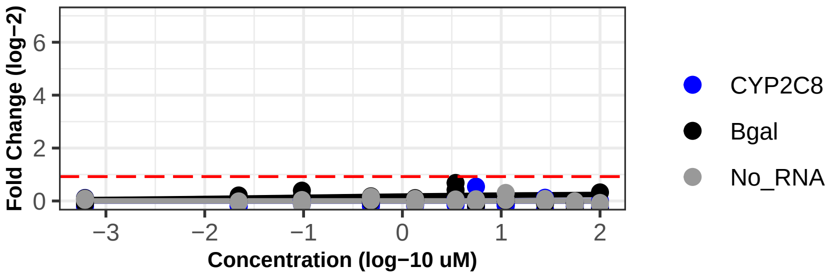


## Mono(2−ethylhexyl) phthalate: CYP2J2

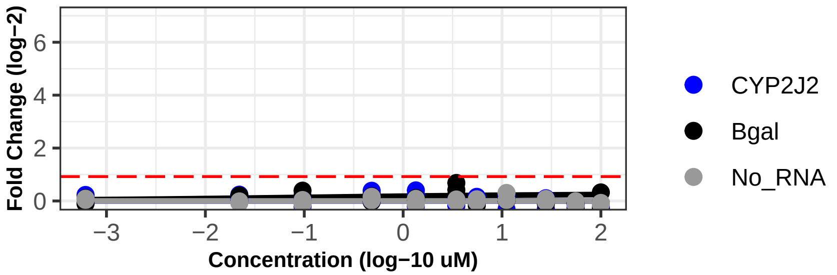


## Mono(2−ethylhexyl) phthalate: CYP2C9

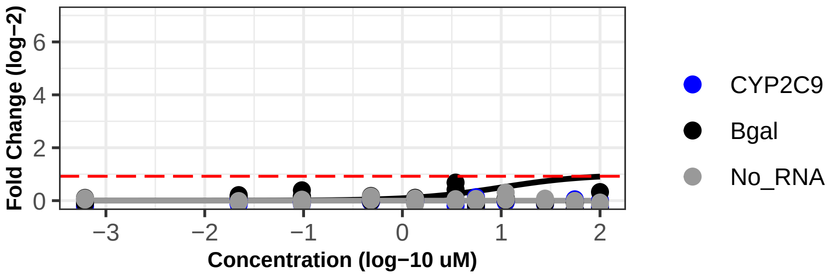


## Mono(2−ethylhexyl) phthalate: CYP3A4

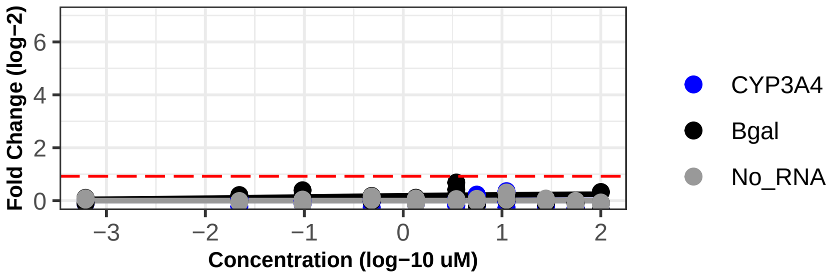

## Naringenin: CYP1A2

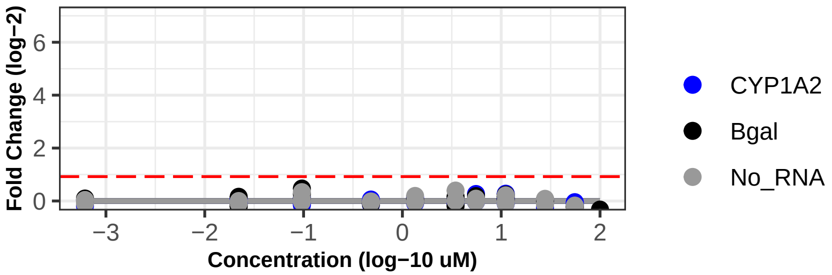


## Naringenin: CYP2C19

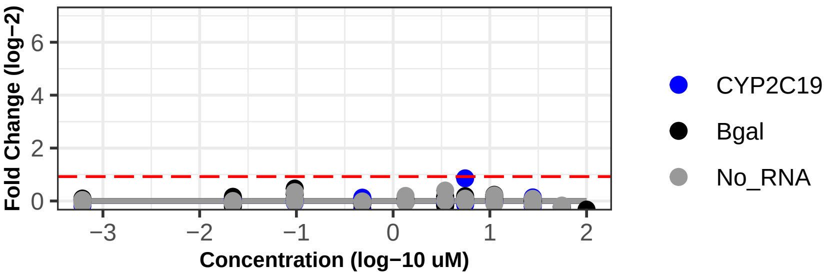


## Naringenin: CYP2A6

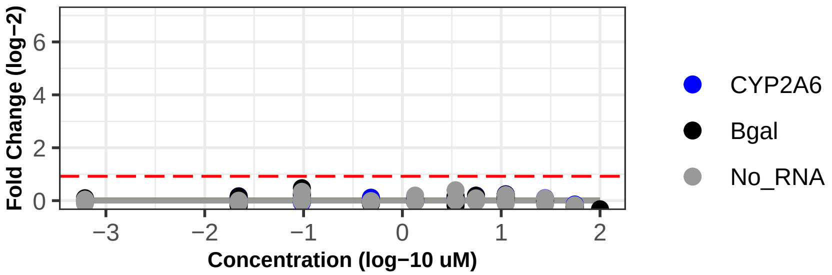


## Naringenin: CYP2D6

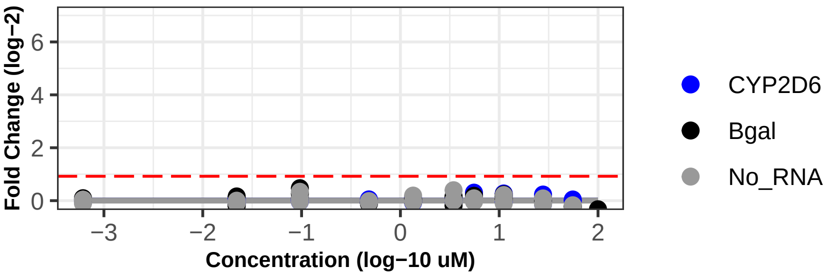


## Naringenin: CYP2B6

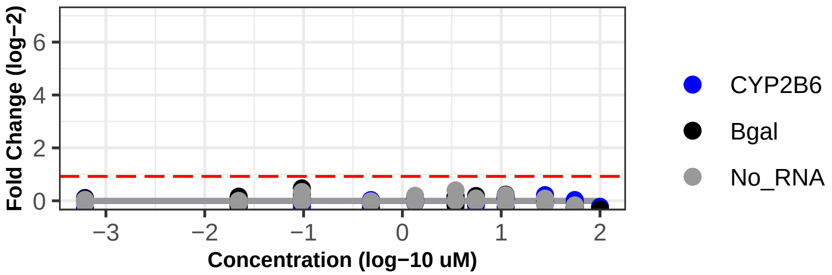


## Naringenin: CYP2E1

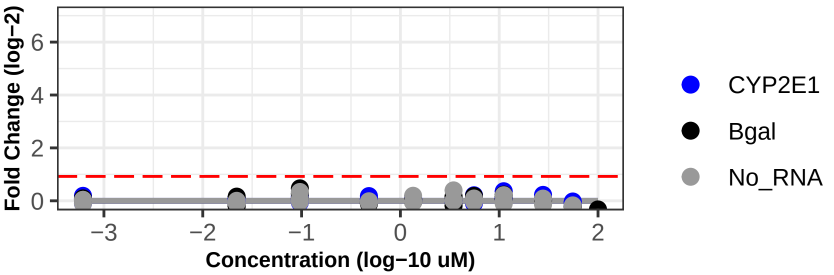


## Naringenin: CYP2C8

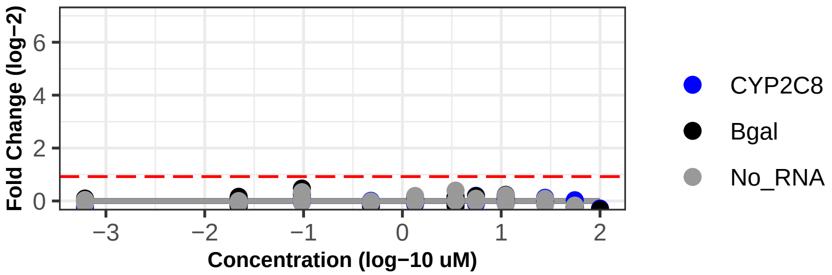


## Naringenin: CYP2J2

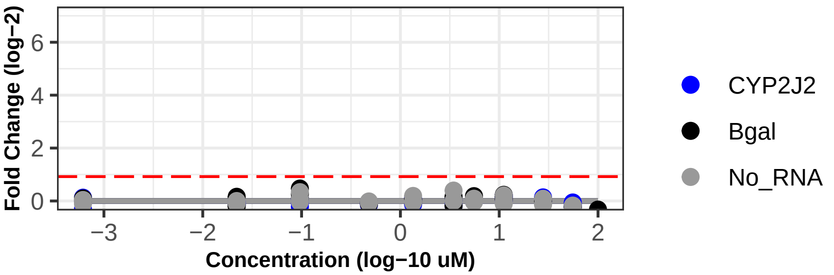


## Naringenin: CYP2C9

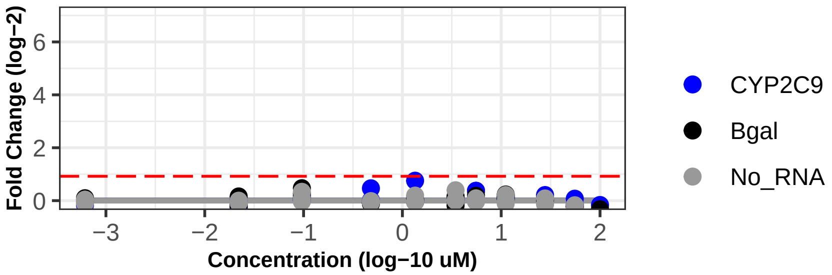


## Naringenin: CYP3A4

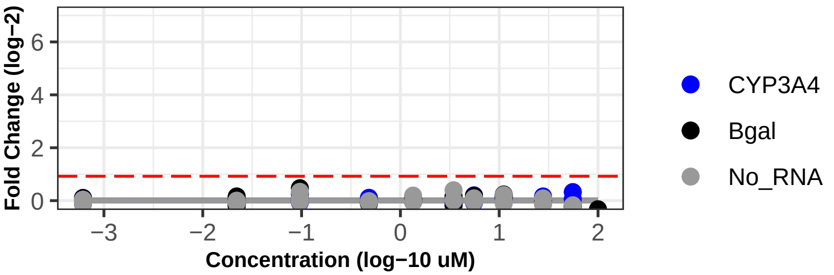

## Nilutamide: CYP1A2

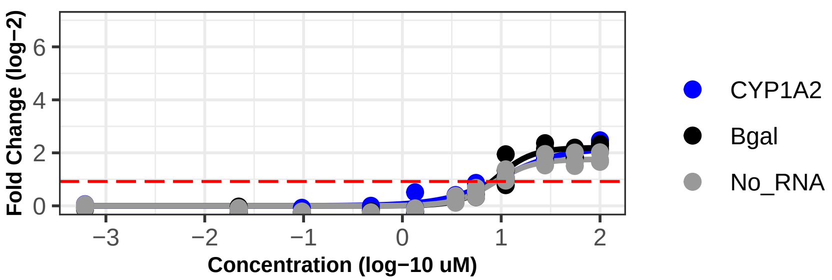


## Nilutamide: CYP2C19

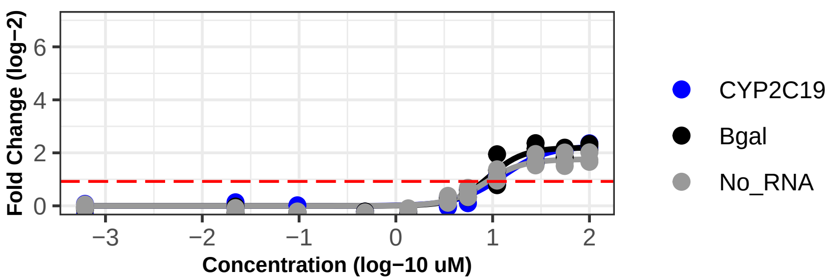


## Nilutamide: CYP2A6

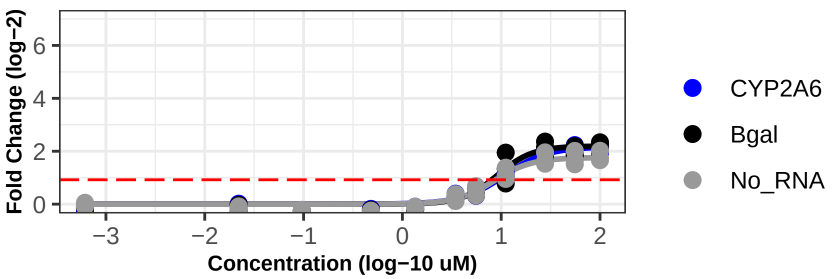


## Nilutamide: CYP2D6

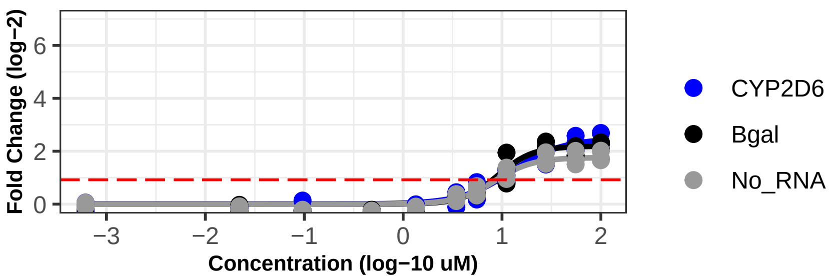


## Nilutamide: CYP2B6

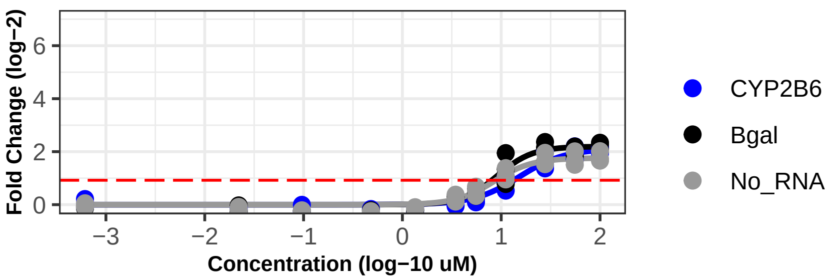


## Nilutamide: CYP2E1

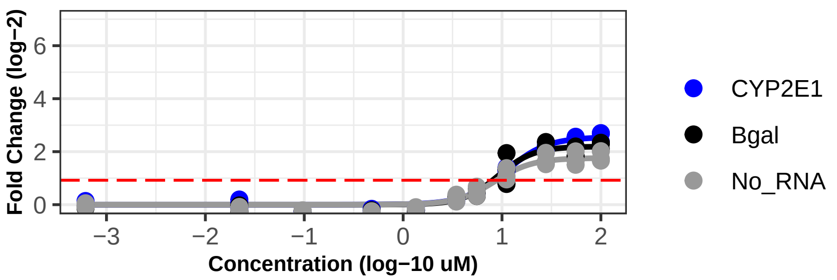


## Nilutamide: CYP2C8

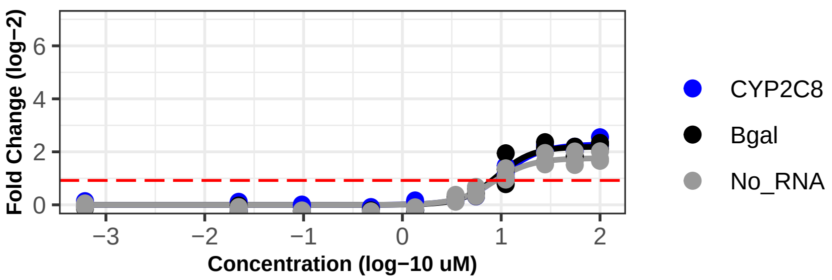


## Nilutamide: CYP2J2

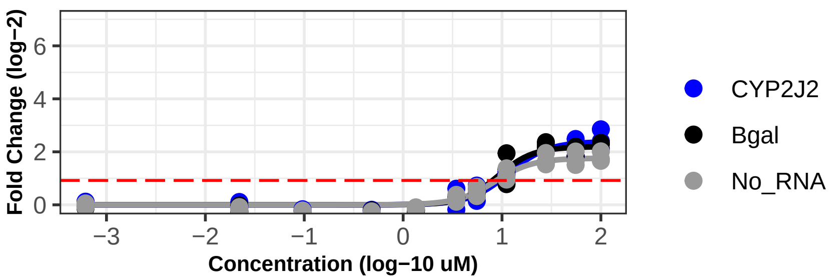


## Nilutamide: CYP2C9

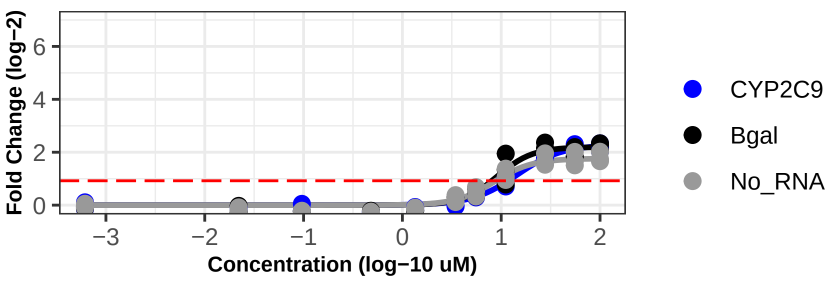


## Nilutamide: CYP3A4

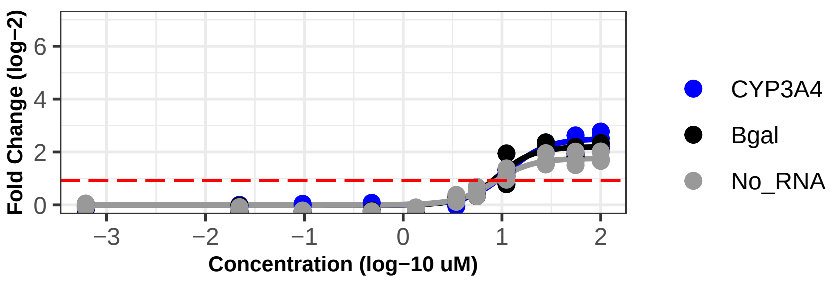

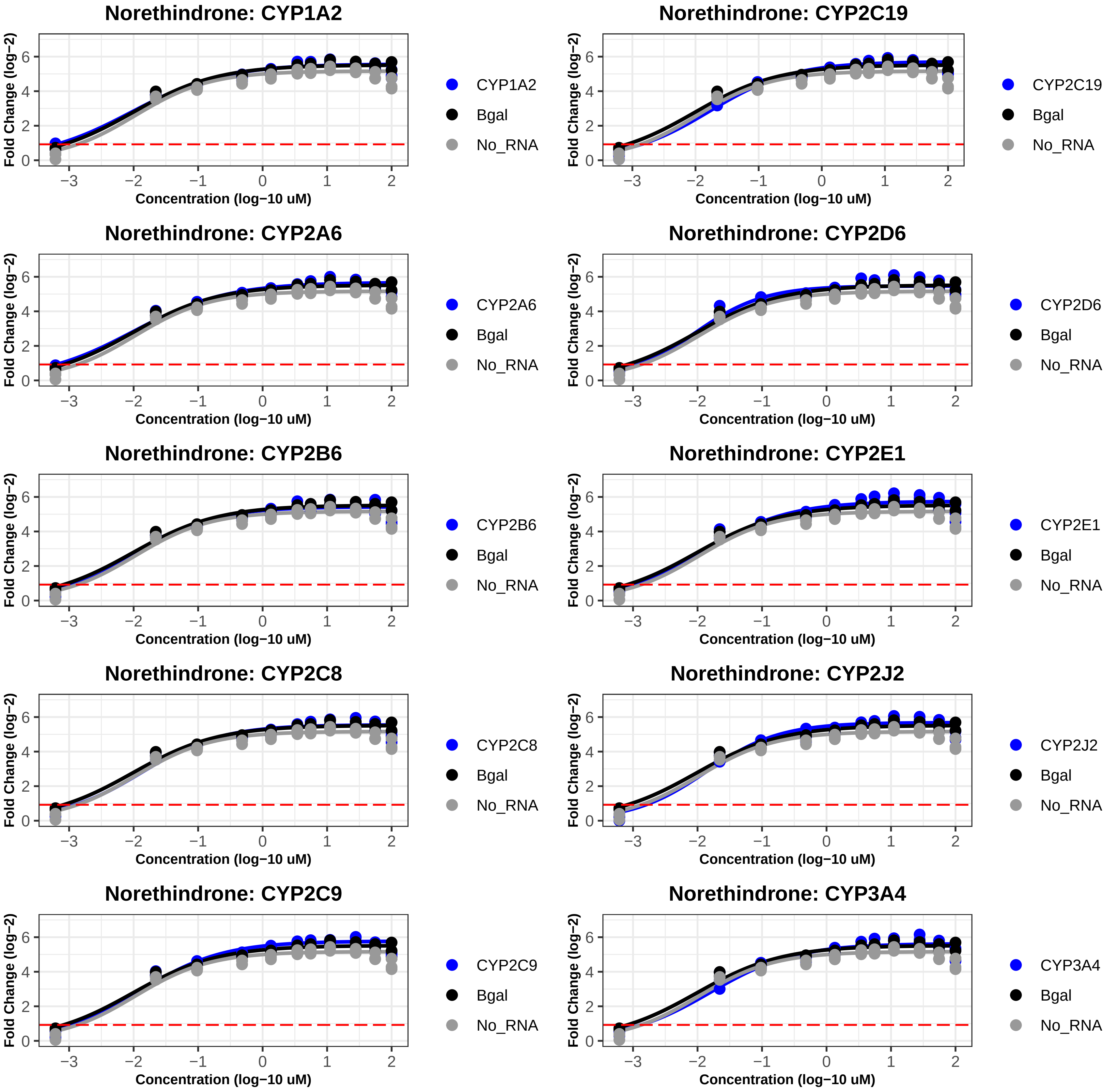
Norethindrone: CYP1A2
CYP1A2
Bgal
No_RNA
Norethindrone: CYP2C19
CYP2C19
Bgal
No_RNA
Norethindrone: CYP2A6
CYP2A6
Bgal
No_RNA
Norethindrone: CYP2D6
CYP2D6
Bgal
No_RNA
Norethindrone: CYP2B6
CYP2B6
Bgal
No_RNA
Norethindrone: CYP2E1
CYP2E1
Bgal
No_RNA
Norethindrone: CYP2C8
CYP2C8
Bgal
No_RNA
Norethindrone: CYP2J2
CYP2J2
Bgal
No_RNA
Norethindrone: CYP2C9
CYP2C9
Bgal
No_RNA
Norethindrone: CYP3A4
CYP3A4
Bgal
No_RNA
Fold Change (log−2)
Concentration (log−10 uM)

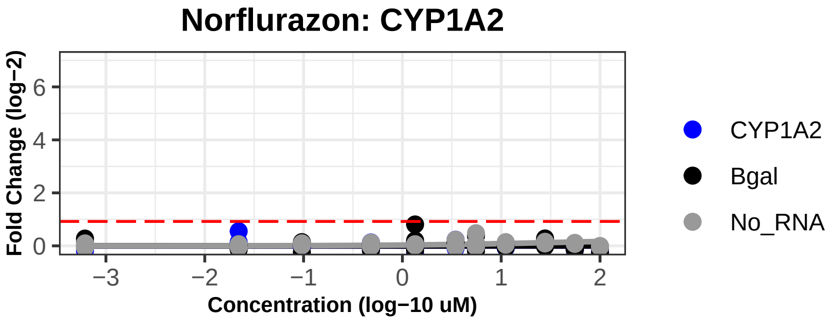
Norflurazon: CYP1A2
Fold Change (log−2)
0 2 4 6
−3 −2 −1 0 1 2
Concentration (log−10 uM)
CYP1A2
Bgal
No_RNA

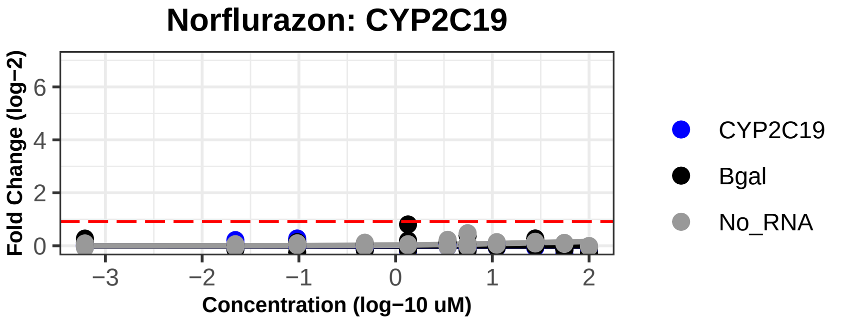
Norflurazon: CYP2C19
Fold Change (log−2)
0 2 4 6
−3 −2 −1 0 1 2
Concentration (log−10 uM)
CYP2C19
Bgal
No_RNA

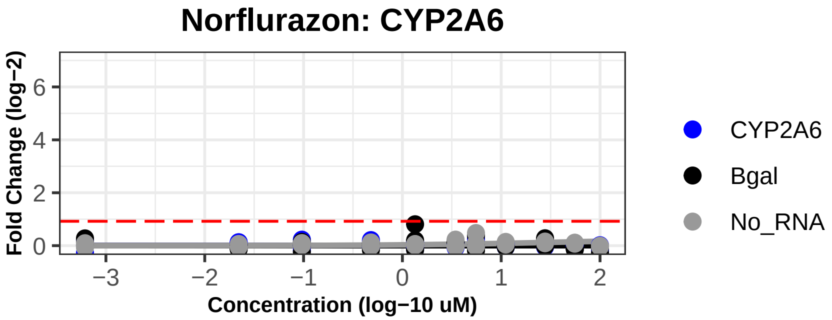
Norflurazon: CYP2A6
Fold Change (log−2)
0 2 4 6
−3 −2 −1 0 1 2
Concentration (log−10 uM)
CYP2A6
Bgal
No_RNA

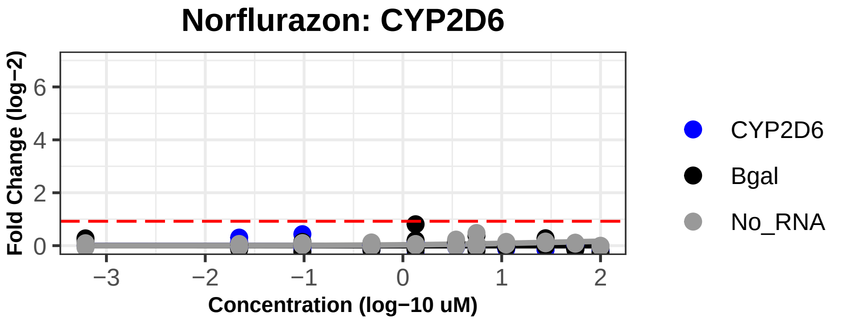
Norflurazon: CYP2D6
Fold Change (log−2)
0 2 4 6
−3 −2 −1 0 1 2
Concentration (log−10 uM)
CYP2D6
Bgal
No_RNA

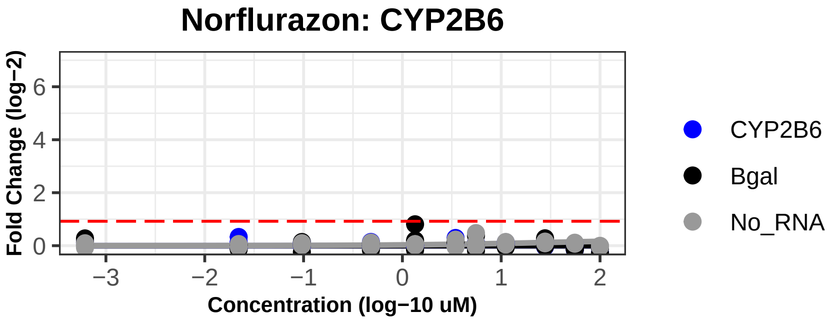
Norflurazon: CYP2B6
Fold Change (log−2)
0 2 4 6
−3 −2 −1 0 1 2
Concentration (log−10 uM)
CYP2B6
Bgal
No_RNA

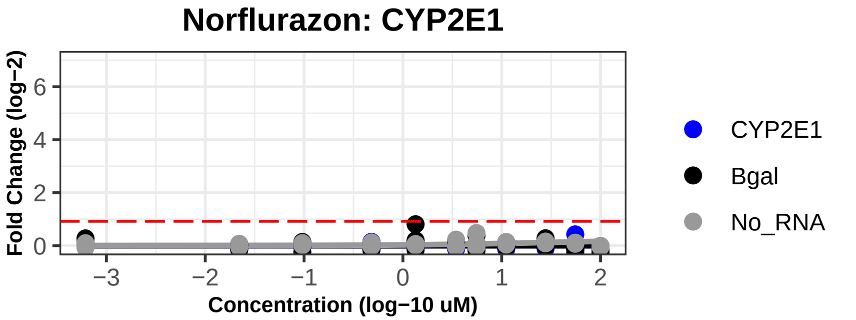
Norflurazon: CYP2E1
Fold Change (log−2)
0 2 4 6
−3 −2 −1 0 1 2
Concentration (log−10 uM)
CYP2E1
Bgal
No_RNA

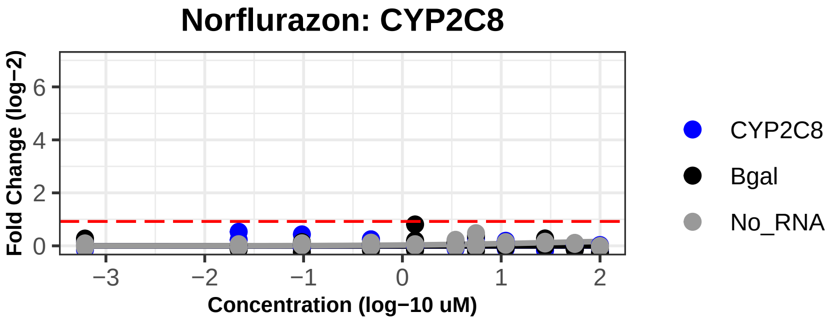
Norflurazon: CYP2C8
Fold Change (log−2)
0 2 4 6
−3 −2 −1 0 1 2
Concentration (log−10 uM)
CYP2C8
Bgal
No_RNA

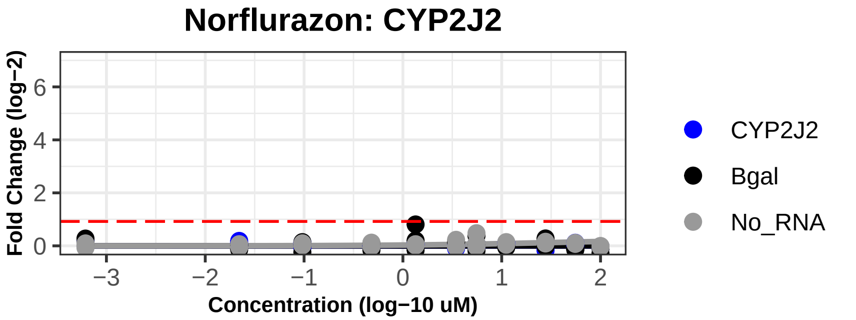
Norflurazon: CYP2J2
Fold Change (log−2)
0 2 4 6
−3 −2 −1 0 1 2
Concentration (log−10 uM)
CYP2J2
Bgal
No_RNA

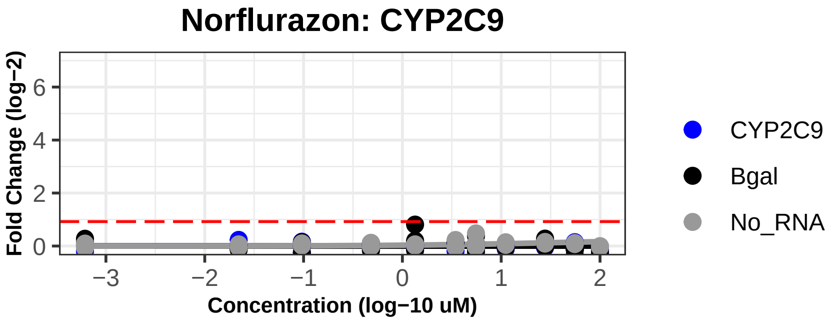
Norflurazon: CYP2C9
Fold Change (log−2)
0 2 4 6
−3 −2 −1 0 1 2
Concentration (log−10 uM)
CYP2C9
Bgal
No_RNA

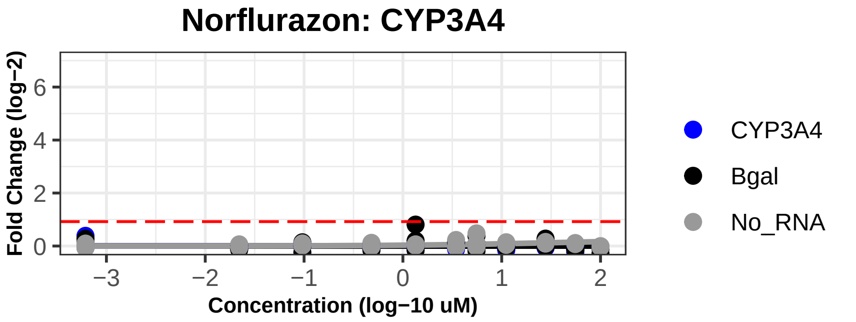
Norflurazon: CYP3A4
Fold Change (log−2)
0 2 4 6
−3 −2 −1 0 1 2
Concentration (log−10 uM)
CYP3A4
Bgal
No_RNA

### Octamethylcyclotetrasiloxane: CYP1A2

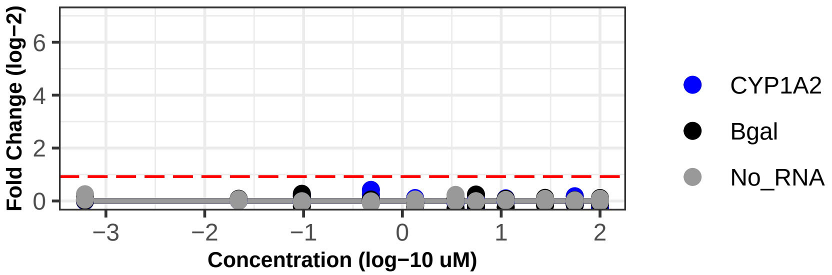


### Octamethylcyclotetrasiloxane: CYP2C19

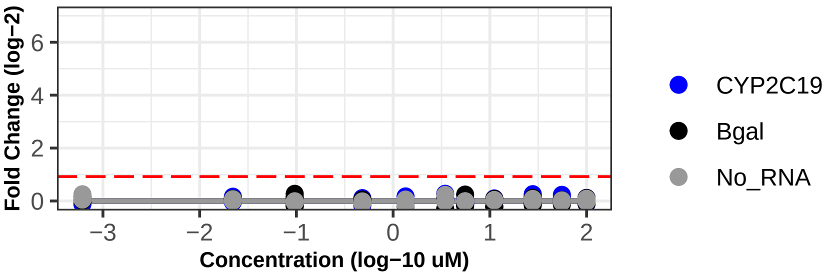


### Octamethylcyclotetrasiloxane: CYP2A6

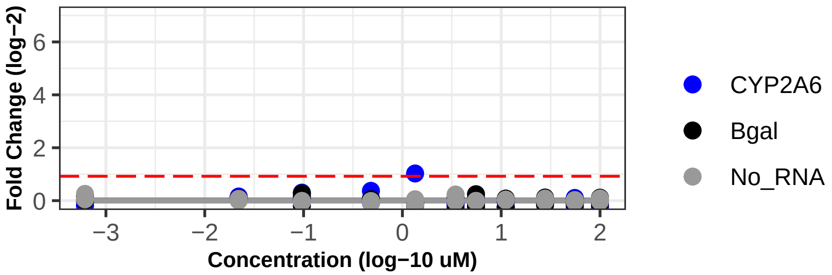


### Octamethylcyclotetrasiloxane: CYP2D6

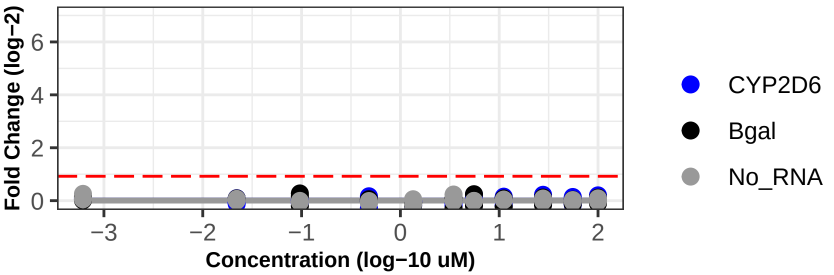


### Octamethylcyclotetrasiloxane: CYP2B6

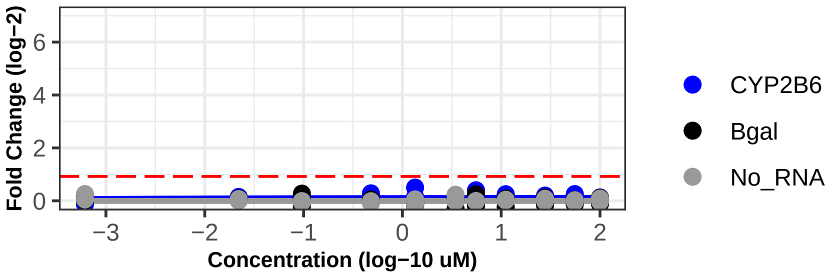


### Octamethylcyclotetrasiloxane: CYP2E1

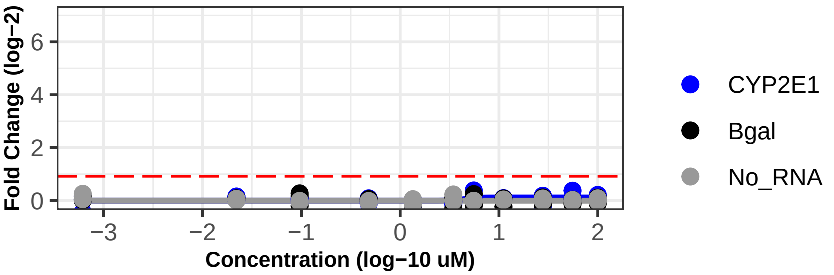


### Octamethylcyclotetrasiloxane: CYP2C8

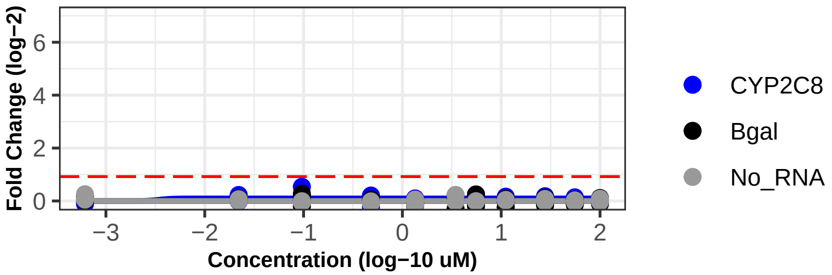


### Octamethylcyclotetrasiloxane: CYP2J2

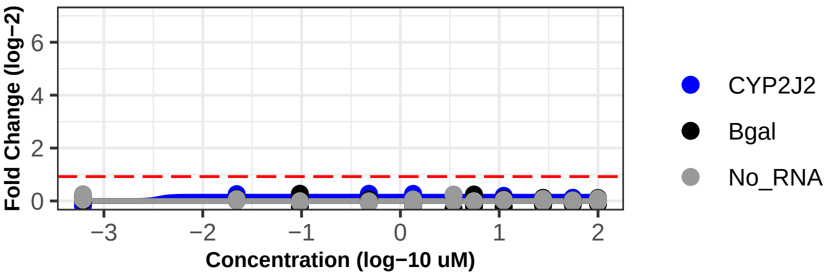


### Octamethylcyclotetrasiloxane: CYP2C9

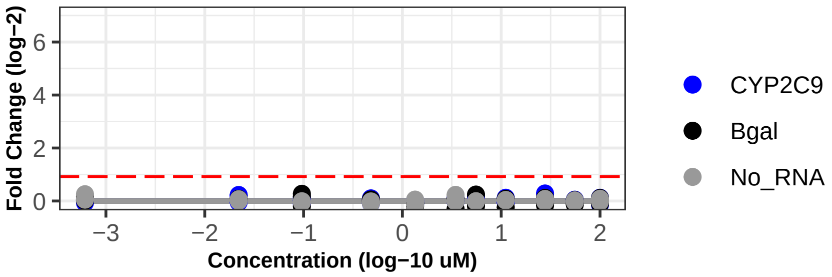


### Octamethylcyclotetrasiloxane: CYP3A4

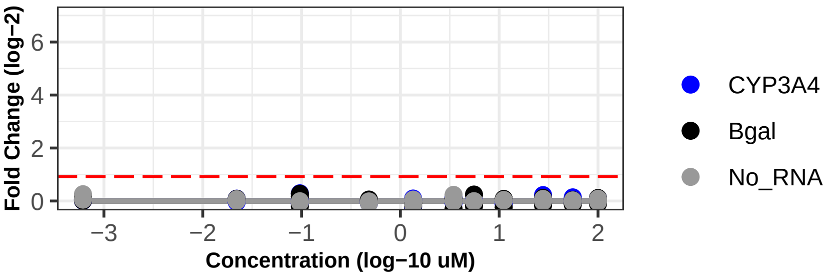

## Octylbicycloheptenedicarboximide: CYP1A2

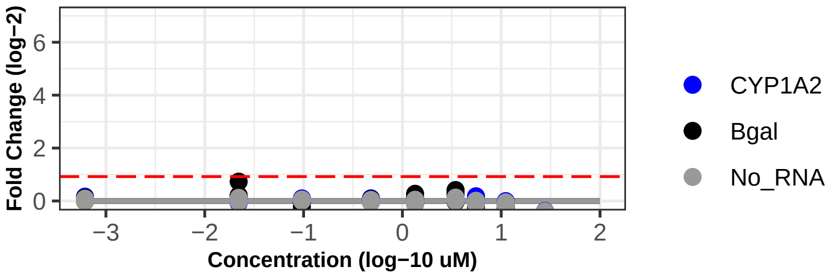


## Octylbicycloheptenedicarboximide: CYP2C19

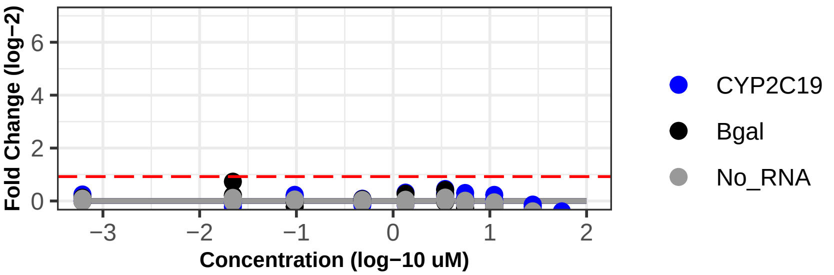


## Octylbicycloheptenedicarboximide: CYP2A6

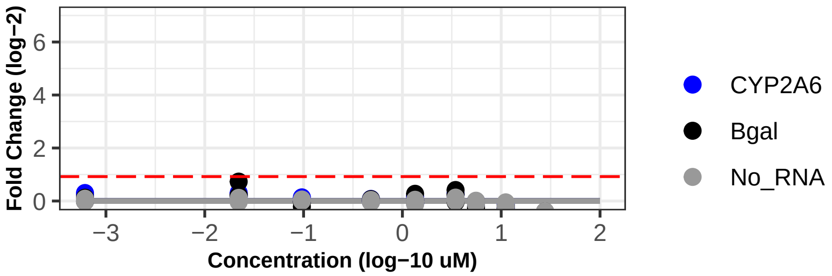


## Octylbicycloheptenedicarboximide: CYP2D6

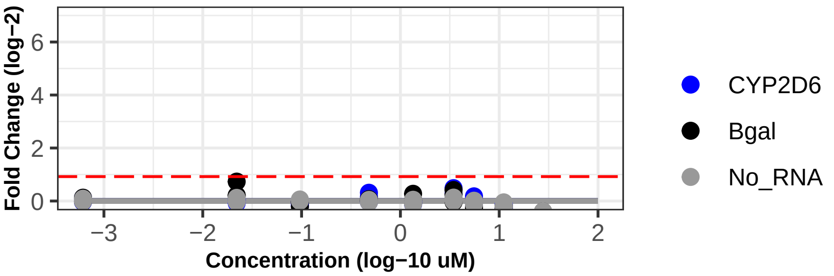


## Octylbicycloheptenedicarboximide: CYP2B6

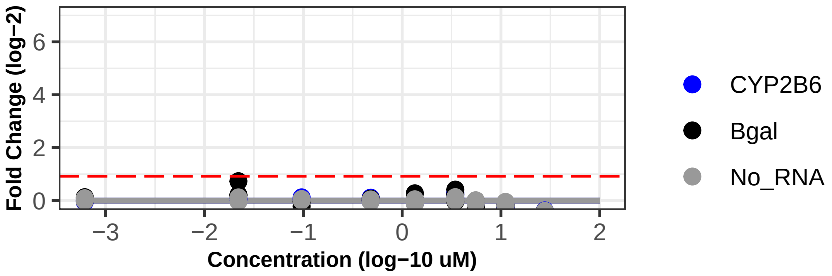


## Octylbicycloheptenedicarboximide: CYP2E1

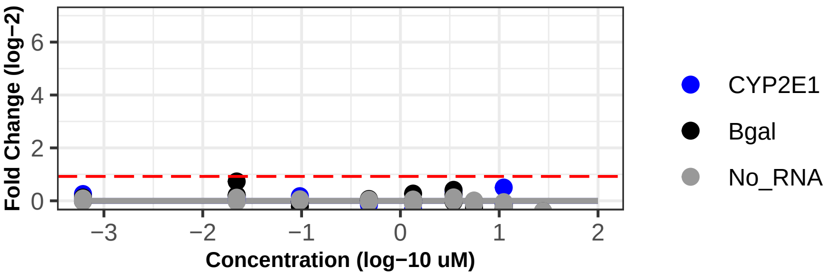


## Octylbicycloheptenedicarboximide: CYP2C8

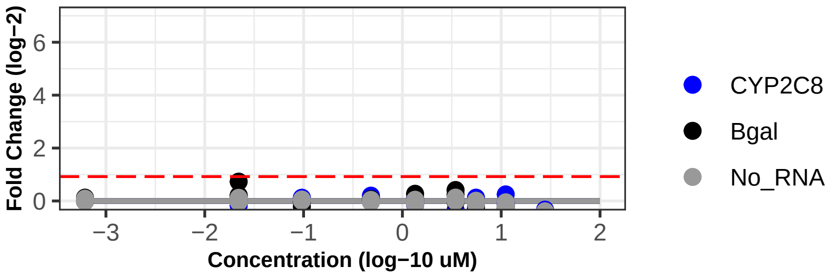


## Octylbicycloheptenedicarboximide: CYP2J2

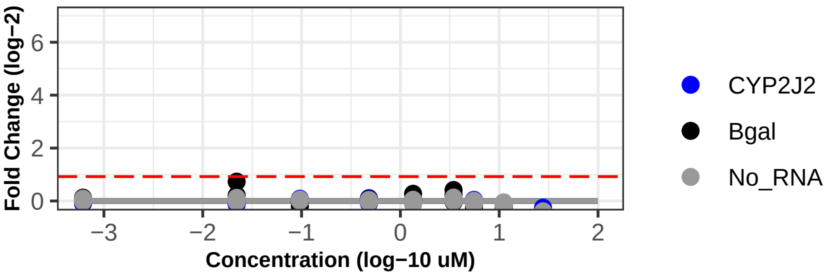


## Octylbicycloheptenedicarboximide: CYP2C9

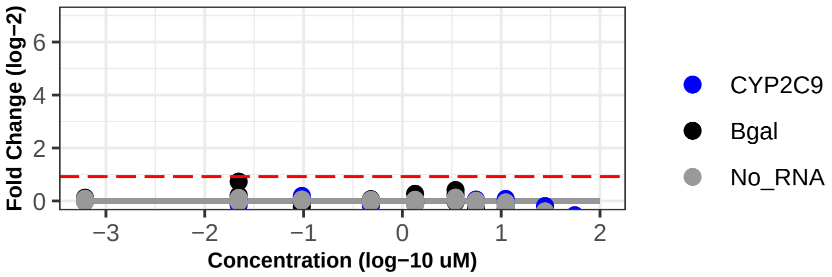


## Octylbicycloheptenedicarboximide: CYP3A4

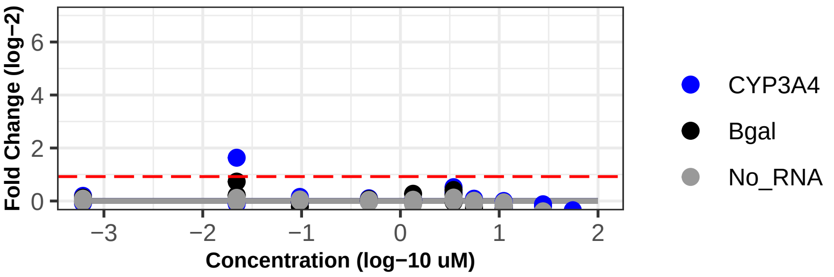

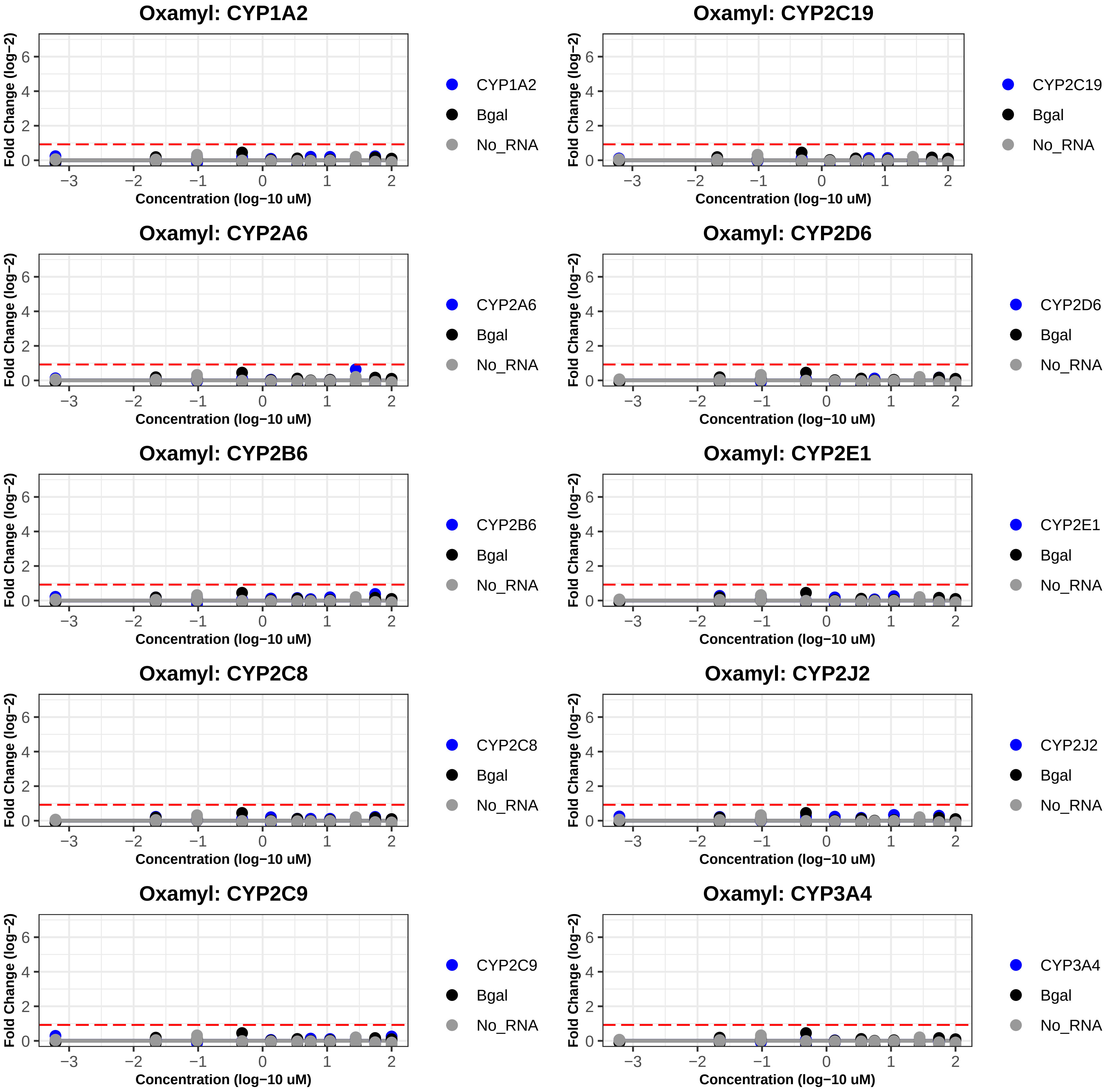
Oxamyl: CYP1A2
Fold Change (log−2)
Concentration (log−10 uM)
CYP1A2
Bgal
No_RNA
Oxamyl: CYP2C19
Fold Change (log−2)
Concentration (log−10 uM)
CYP2C19
Bgal
No_RNA
Oxamyl: CYP2A6
Fold Change (log−2)
Concentration (log−10 uM)
CYP2A6
Bgal
No_RNA
Oxamyl: CYP2D6
Fold Change (log−2)
Concentration (log−10 uM)
CYP2D6
Bgal
No_RNA
Oxamyl: CYP2B6
Fold Change (log−2)
Concentration (log−10 uM)
CYP2B6
Bgal
No_RNA
Oxamyl: CYP2E1
Fold Change (log−2)
Concentration (log−10 uM)
CYP2E1
Bgal
No_RNA
Oxamyl: CYP2C8
Fold Change (log−2)
Concentration (log−10 uM)
CYP2C8
Bgal
No_RNA
Oxamyl: CYP2J2
Fold Change (log−2)
Concentration (log−10 uM)
CYP2J2
Bgal
No_RNA
Oxamyl: CYP2C9
Fold Change (log−2)
Concentration (log−10 uM)
CYP2C9
Bgal
No_RNA
Oxamyl: CYP3A4
Fold Change (log−2)
Concentration (log−10 uM)
CYP3A4
Bgal
No_RNA

## Pentachloronitrobenzene: CYP1A2

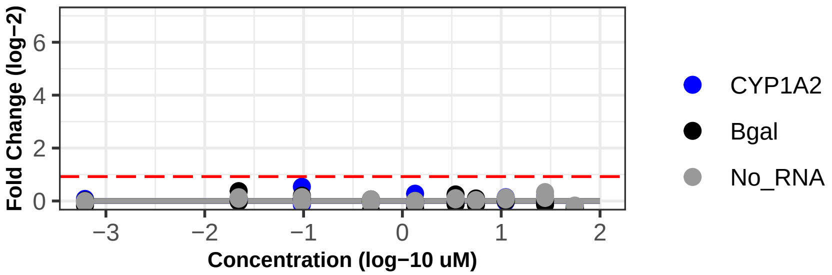


## Pentachloronitrobenzene: CYP2C19

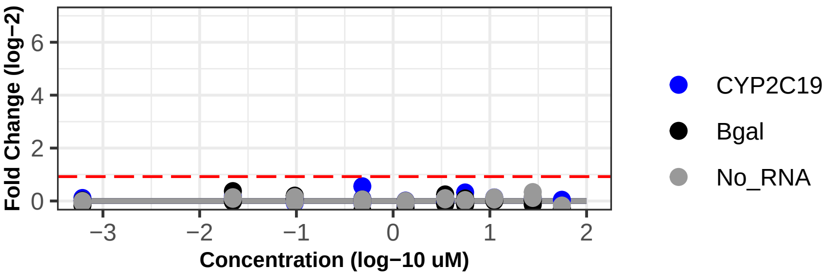


## Pentachloronitrobenzene: CYP2A6

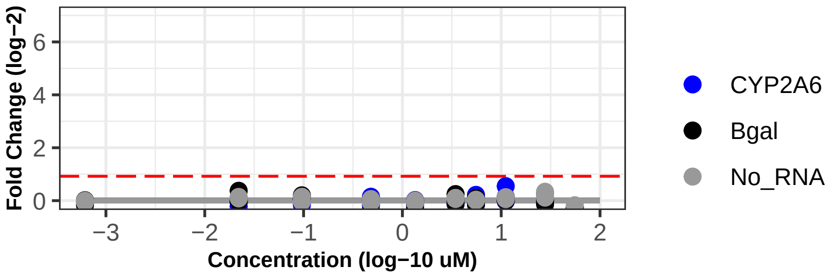


## Pentachloronitrobenzene: CYP2D6

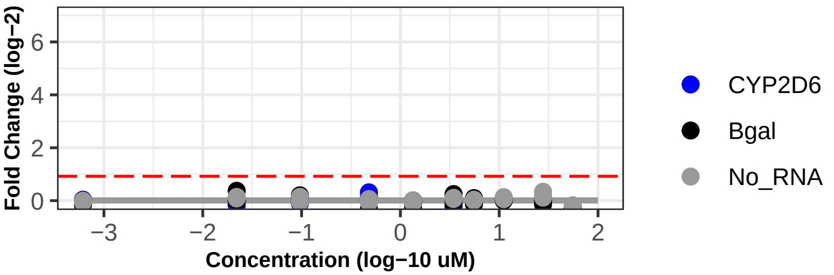


## Pentachloronitrobenzene: CYP2B6

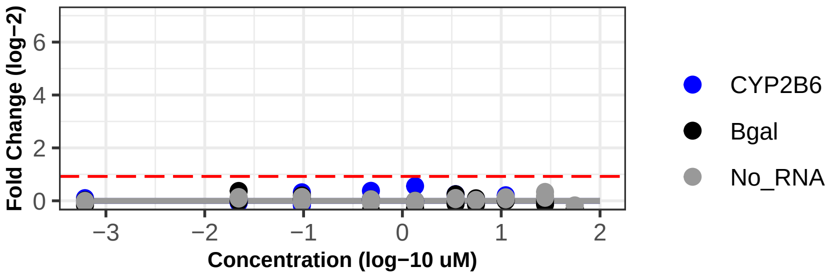


## Pentachloronitrobenzene: CYP2E1

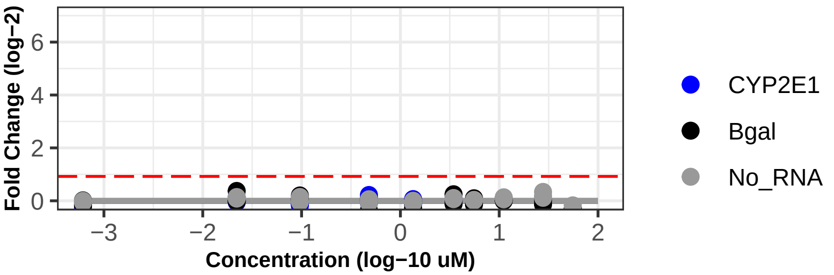


## Pentachloronitrobenzene: CYP2C8

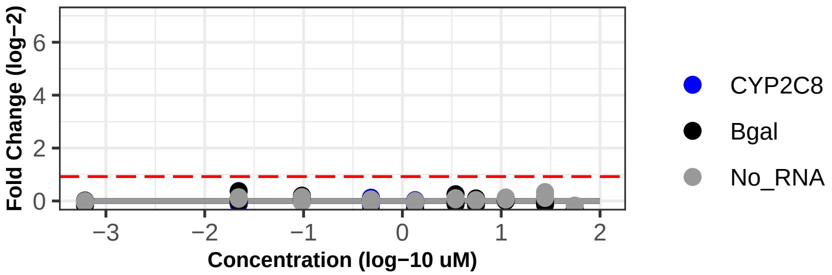


## Pentachloronitrobenzene: CYP2J2

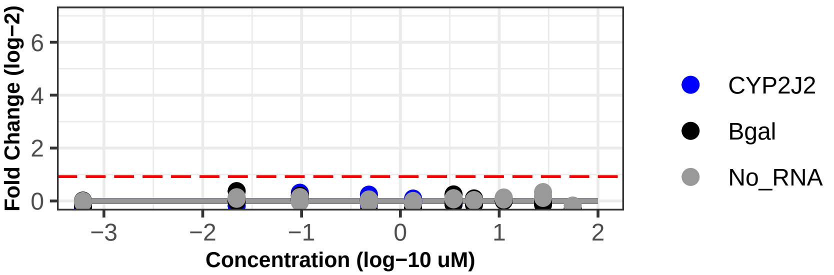


## Pentachloronitrobenzene: CYP2C9

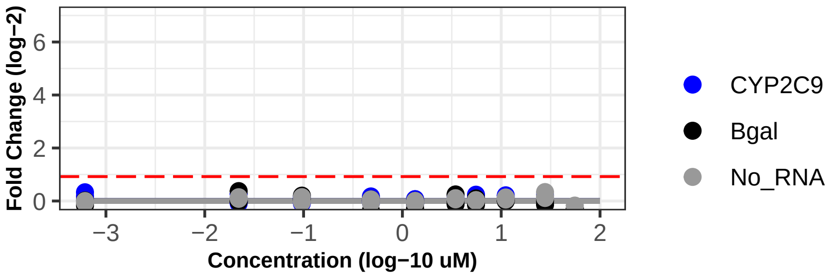


## Pentachloronitrobenzene: CYP3A4

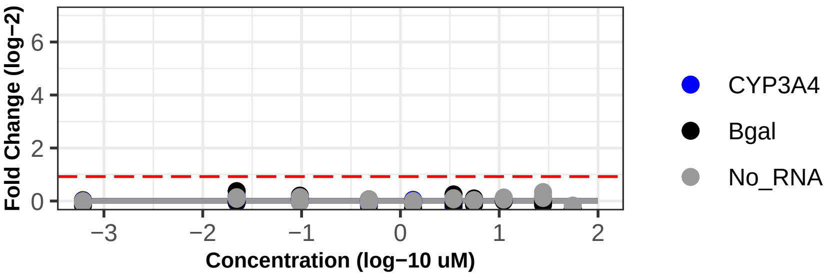

## Pentachlorophenol: CYP1A2

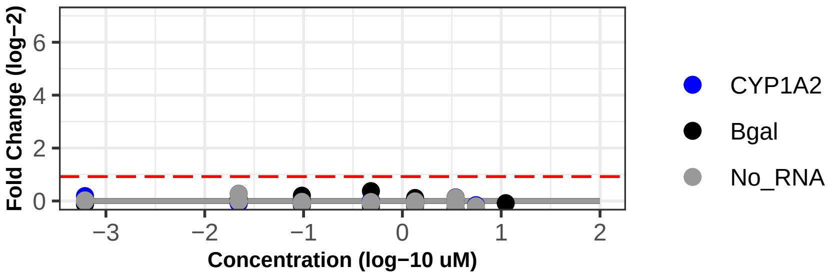


## Pentachlorophenol: CYP2C19

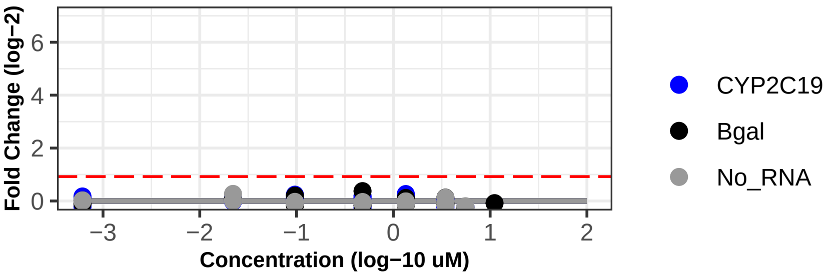


## Pentachlorophenol: CYP2A6

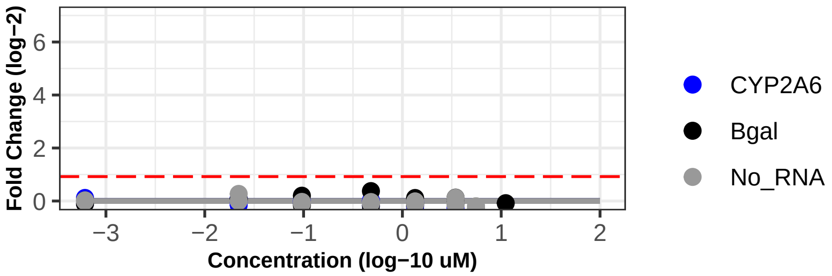


## Pentachlorophenol: CYP2D6

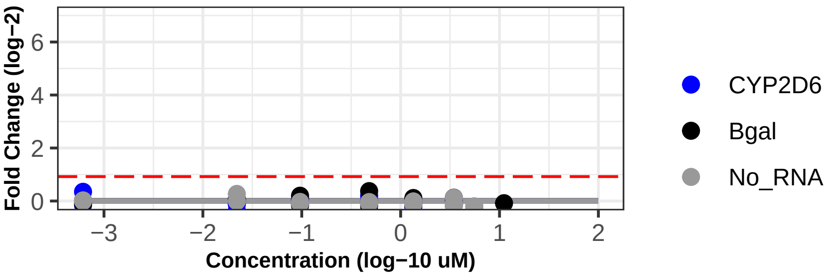


## Pentachlorophenol: CYP2B6

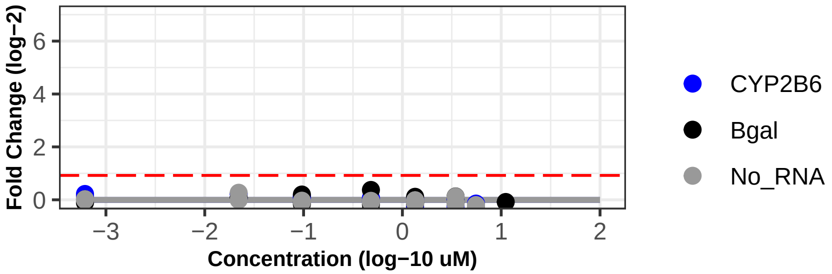


## Pentachlorophenol: CYP2E1

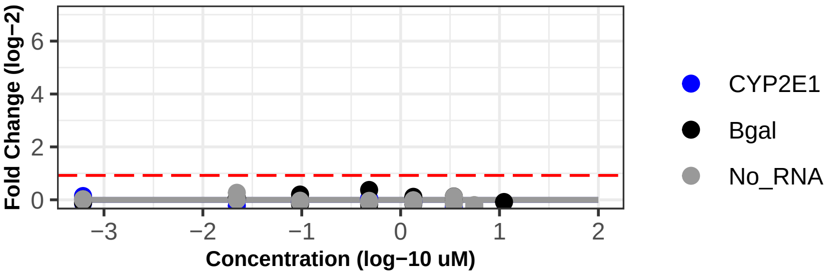


## Pentachlorophenol: CYP2C8

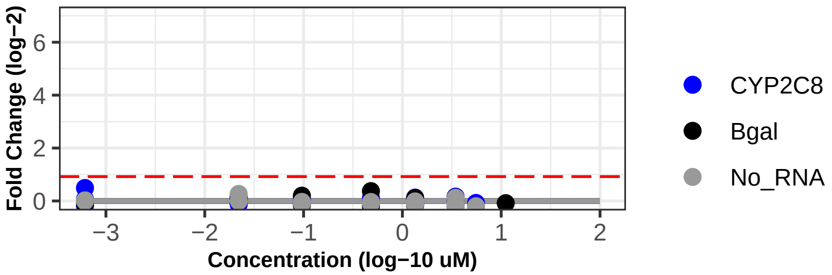


## Pentachlorophenol: CYP2J2

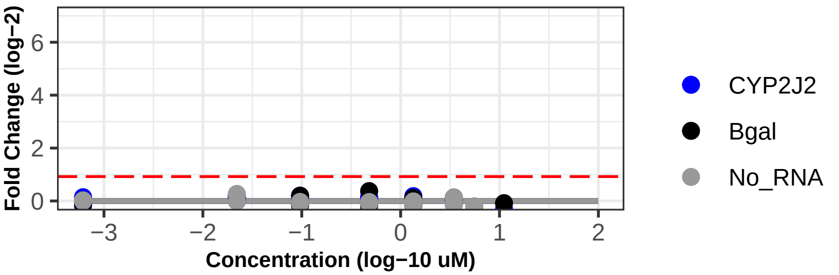


## Pentachlorophenol: CYP2C9

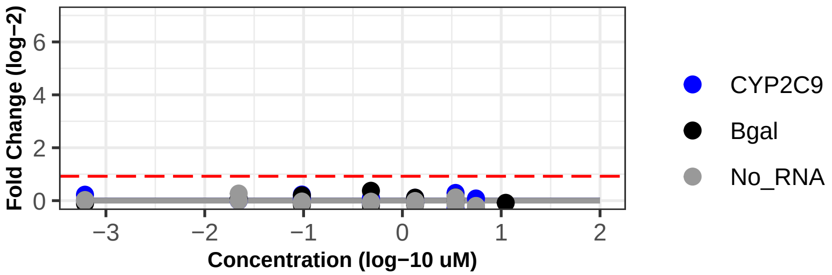


## Pentachlorophenol: CYP3A4

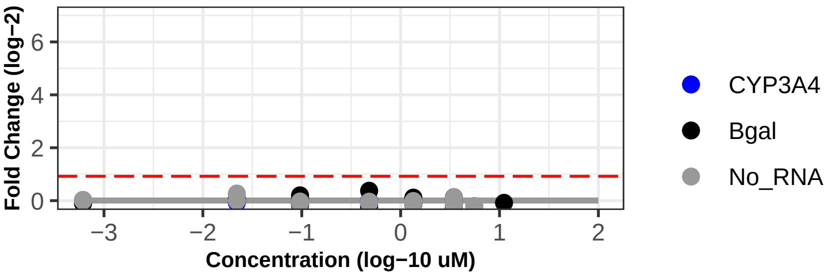

## Permethrin: CYP1A2

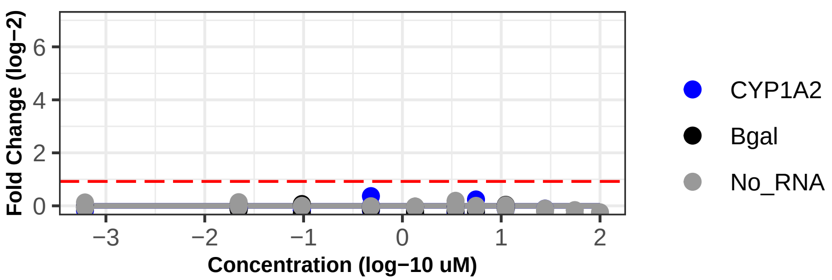


## Permethrin: CYP2C19

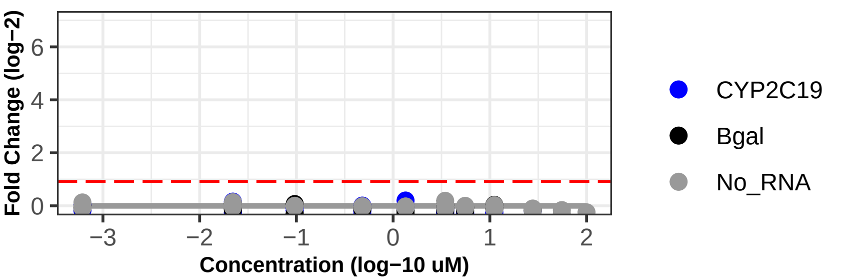


## Permethrin: CYP2A6

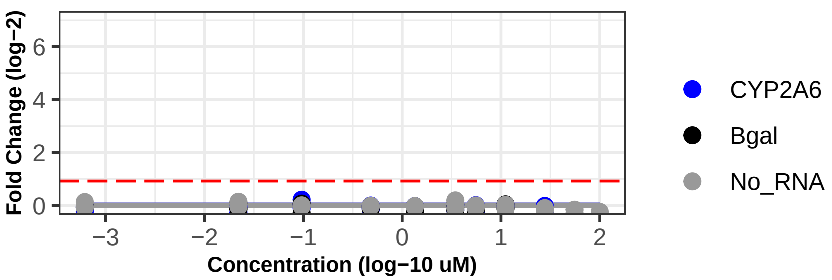


## Permethrin: CYP2D6

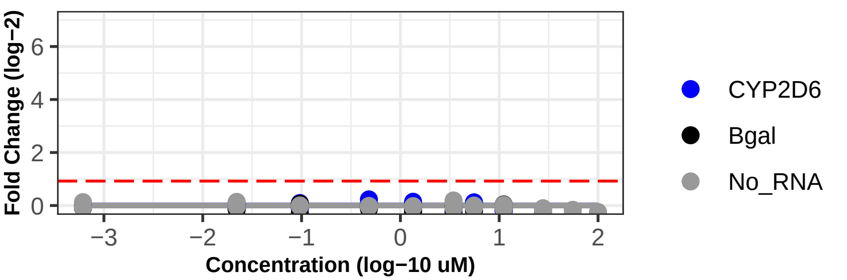


## Permethrin: CYP2B6

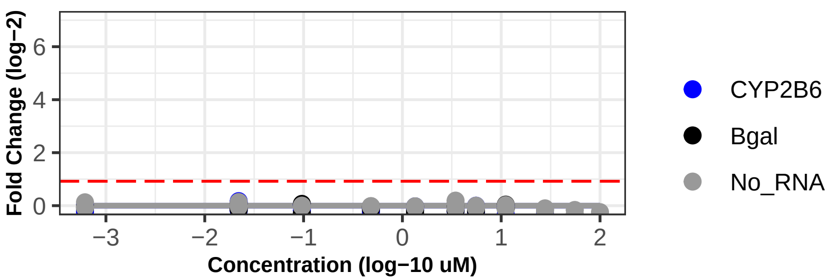


## Permethrin: CYP2E1

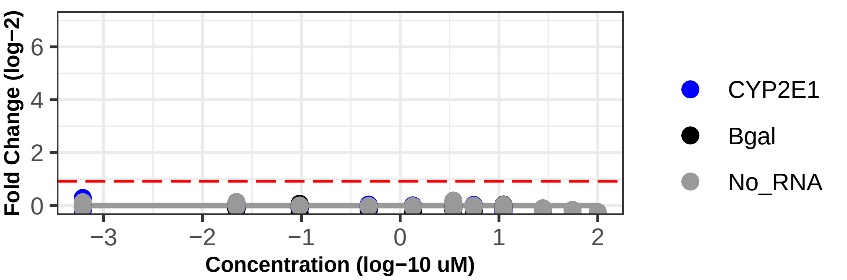


## Permethrin: CYP2C8

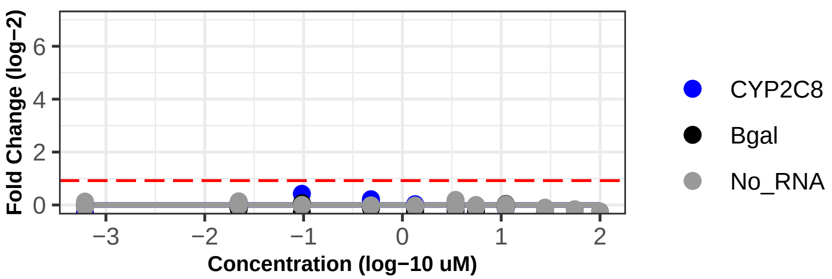


## Permethrin: CYP2J2

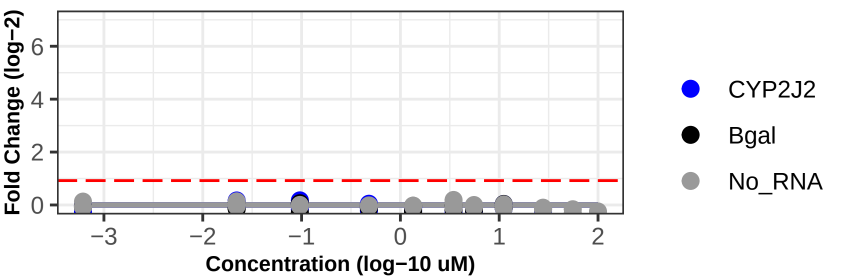


## Permethrin: CYP2C9

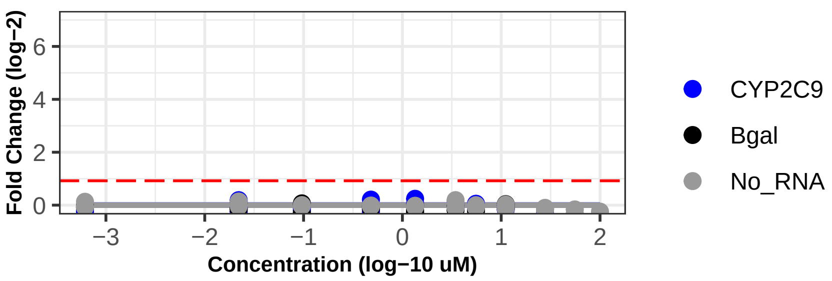


## Permethrin: CYP3A4

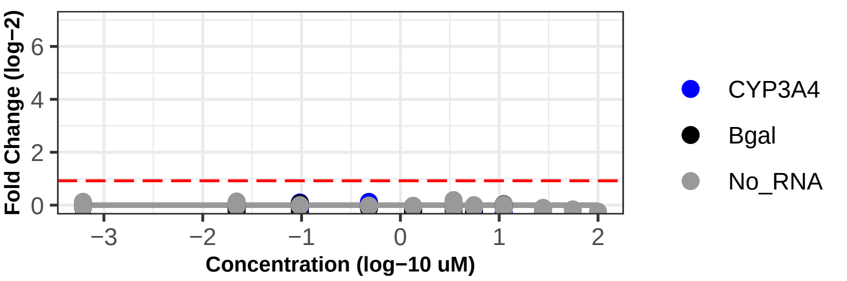

## Phenothrin: CYP1A2

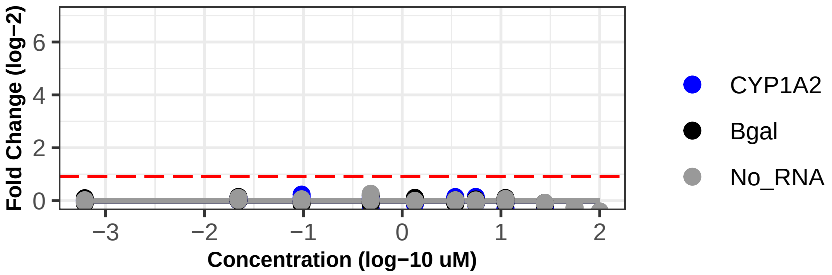


## Phenothrin: CYP2C19

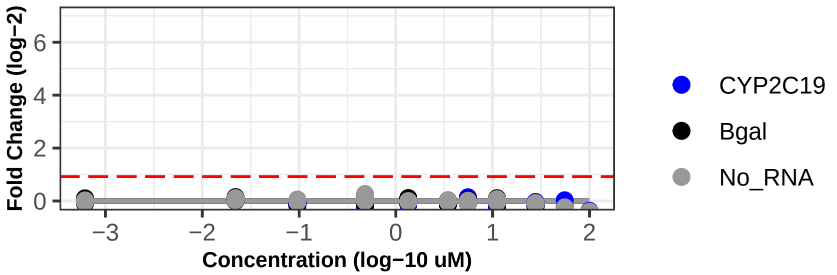


## Phenothrin: CYP2A6

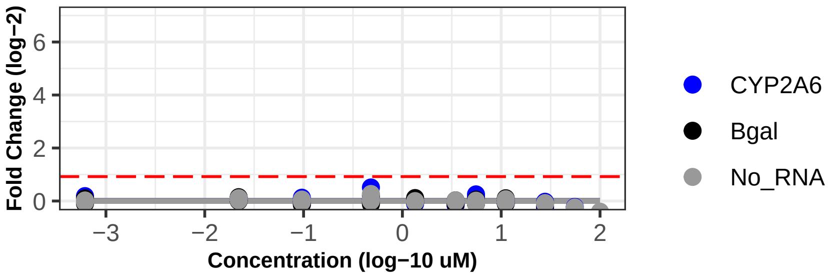


## Phenothrin: CYP2D6

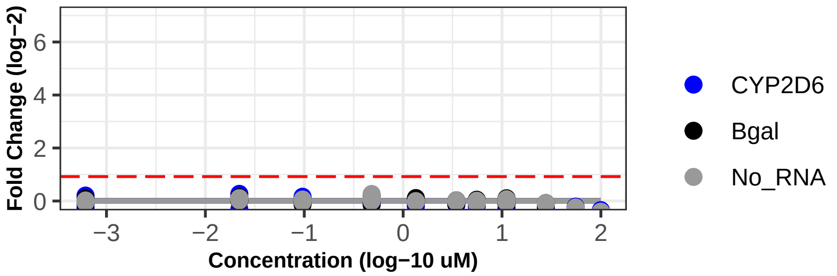


## Phenothrin: CYP2B6

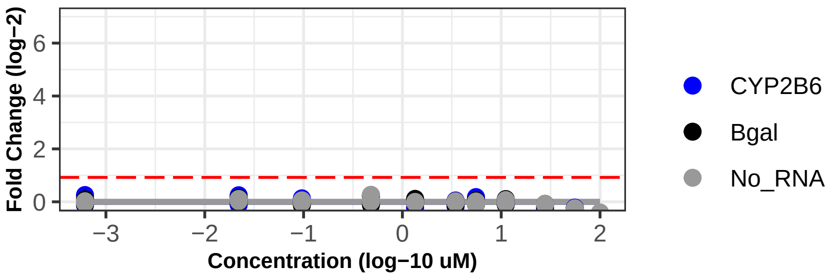


## Phenothrin: CYP2E1

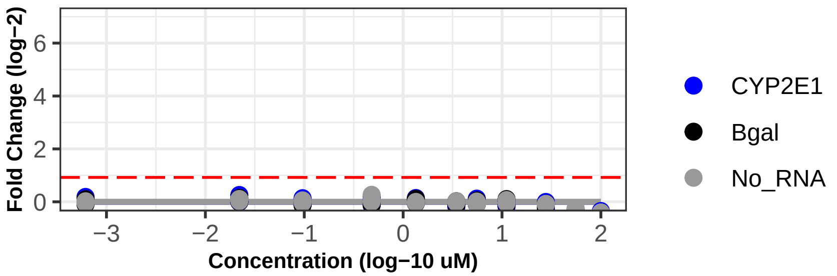


## Phenothrin: CYP2C8

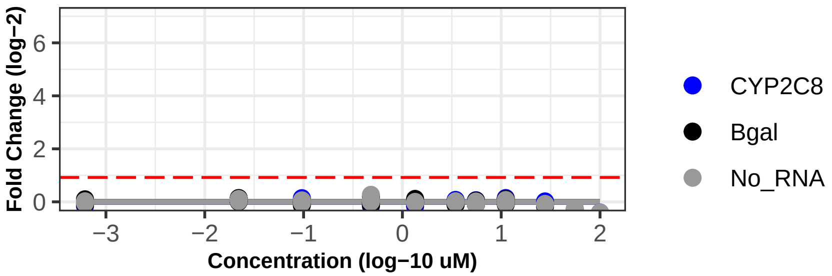


## Phenothrin: CYP2J2

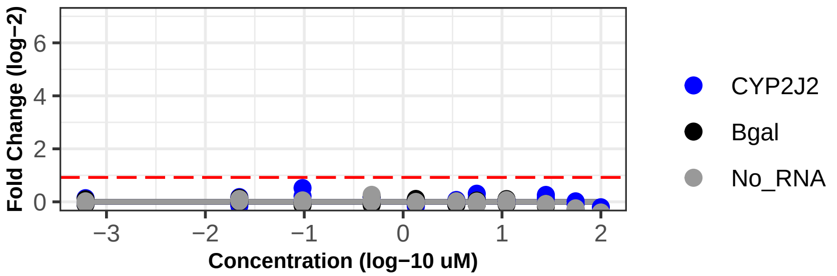


## Phenothrin: CYP2C9

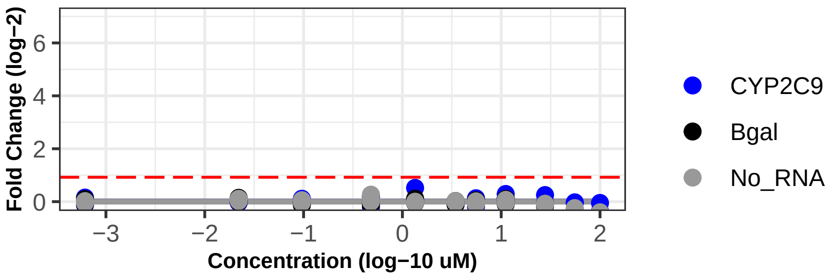


## Phenothrin: CYP3A4

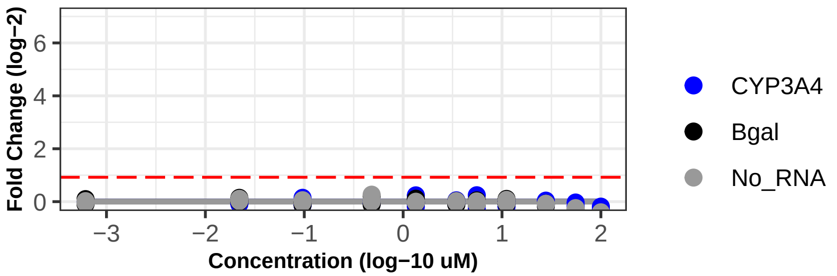

## Phosmet: CYP1A2

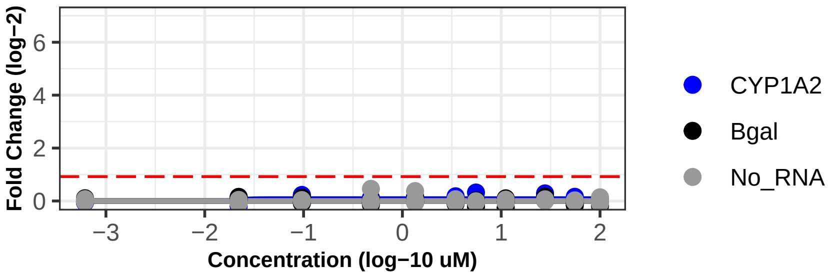


## Phosmet: CYP2C19

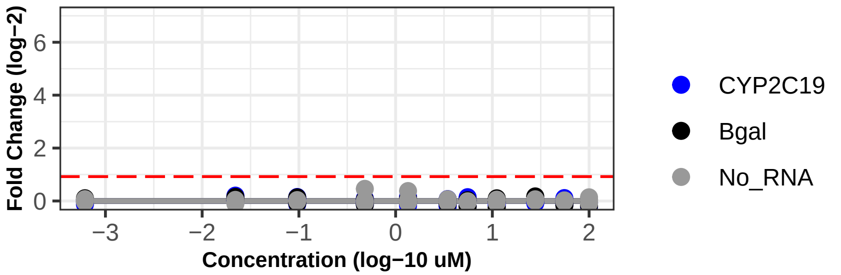


## Phosmet: CYP2A6

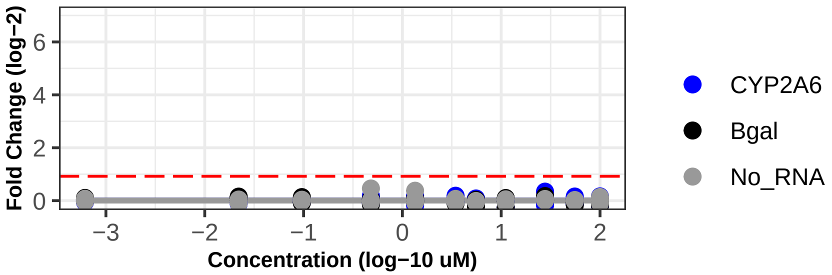


## Phosmet: CYP2D6

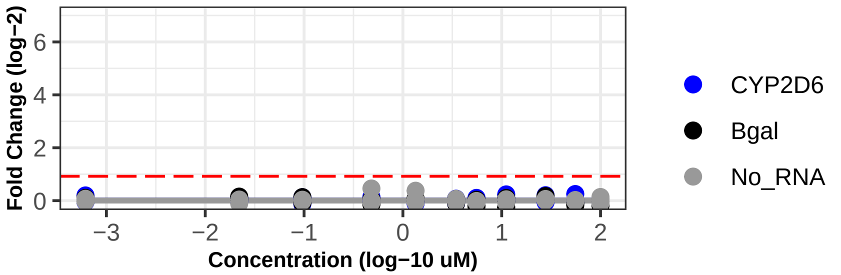


## Phosmet: CYP2B6

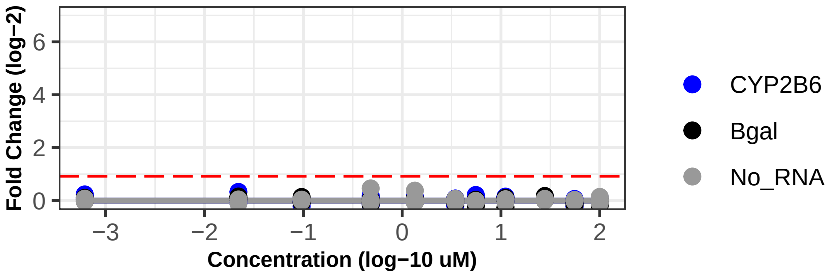


## Phosmet: CYP2E1

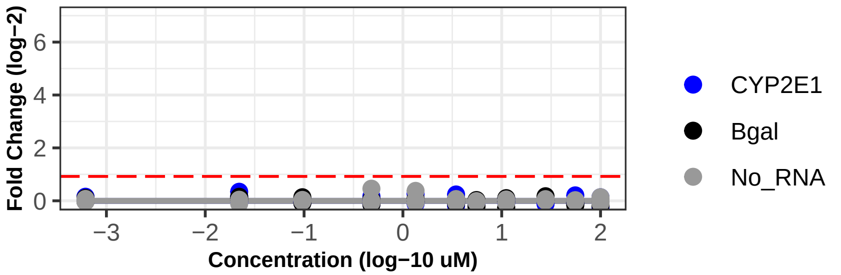


## Phosmet: CYP2C8

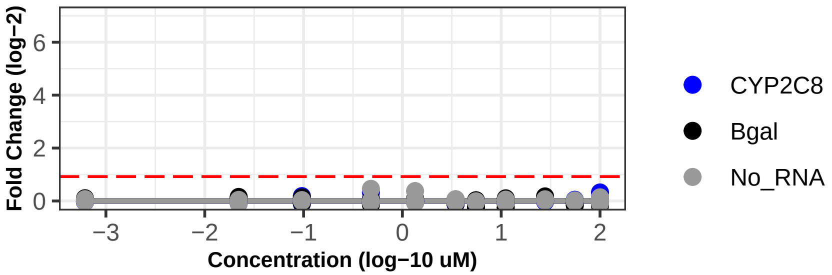


## Phosmet: CYP2J2

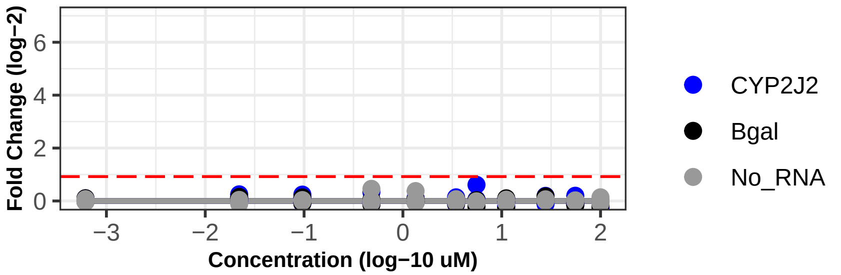


## Phosmet: CYP2C9

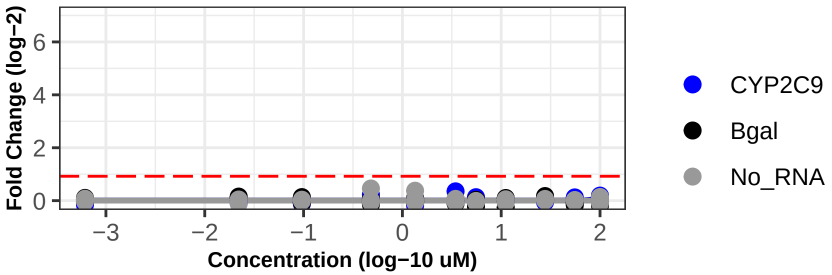


## Phosmet: CYP3A4

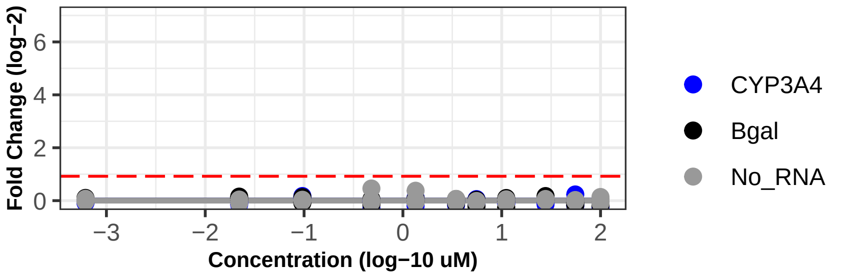

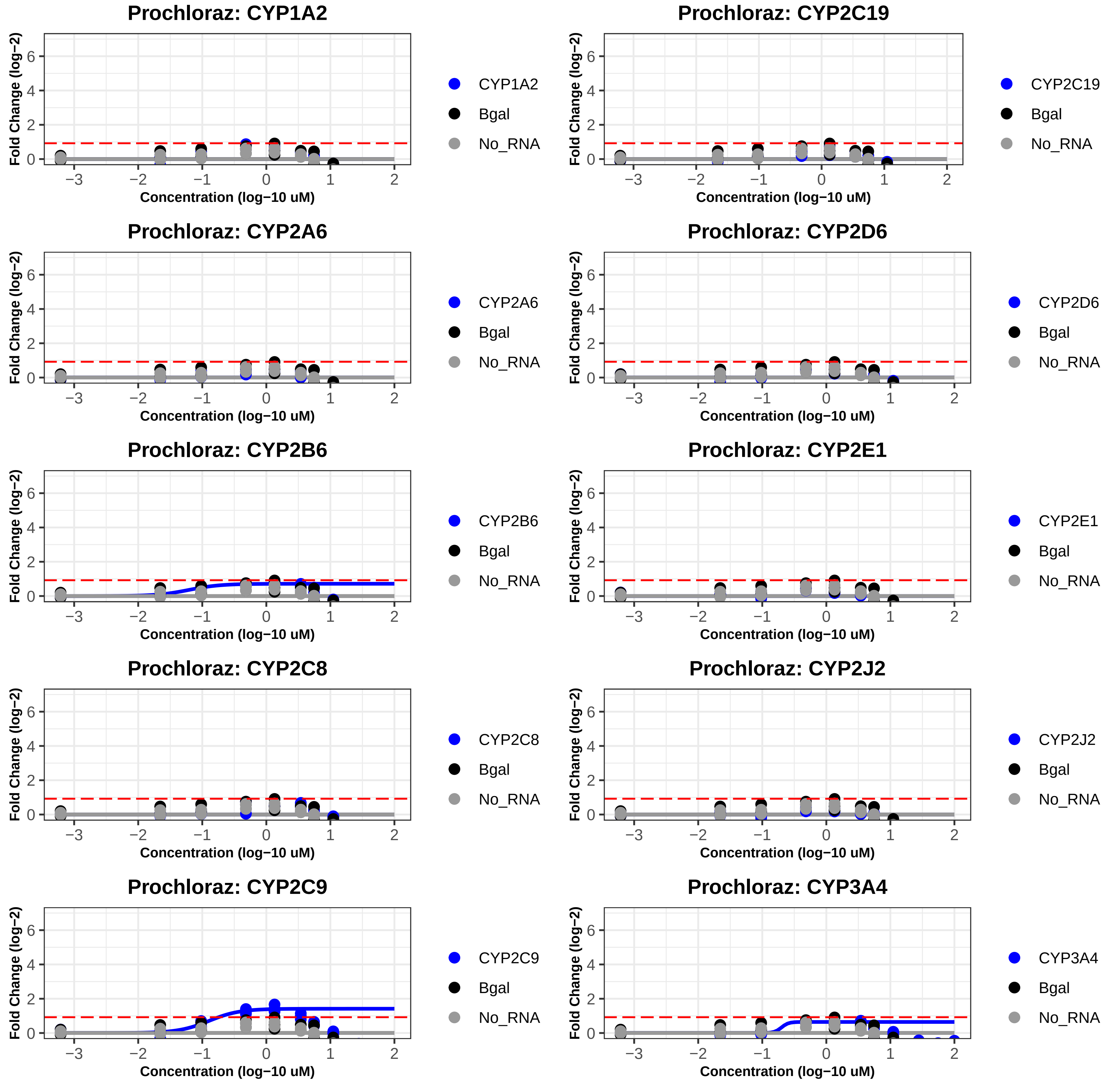
Prochloraz: CYP1A2
Fold Change (log−2)
Concentration (log−10 uM)
CYP1A2
Bgal
No_RNA
Prochloraz: CYP2C19
Fold Change (log−2)
Concentration (log−10 uM)
CYP2C19
Bgal
No_RNA
Prochloraz: CYP2A6
Fold Change (log−2)
Concentration (log−10 uM)
CYP2A6
Bgal
No_RNA
Prochloraz: CYP2D6
Fold Change (log−2)
Concentration (log−10 uM)
CYP2D6
Bgal
No_RNA
Prochloraz: CYP2B6
Fold Change (log−2)
Concentration (log−10 uM)
CYP2B6
Bgal
No_RNA
Prochloraz: CYP2E1
Fold Change (log−2)
Concentration (log−10 uM)
CYP2E1
Bgal
No_RNA
Prochloraz: CYP2C8
Fold Change (log−2)
Concentration (log−10 uM)
CYP2C8
Bgal
No_RNA
Prochloraz: CYP2J2
Fold Change (log−2)
Concentration (log−10 uM)
CYP2J2
Bgal
No_RNA
Prochloraz: CYP2C9
Fold Change (log−2)
Concentration (log−10 uM)
CYP2C9
Bgal
No_RNA
Prochloraz: CYP3A4
Fold Change (log−2)
Concentration (log−10 uM)
CYP3A4
Bgal
No_RNA

## Procymidone: CYP1A2

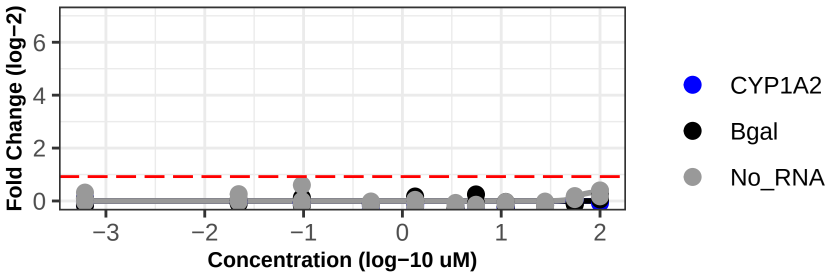


## Procymidone: CYP2C19

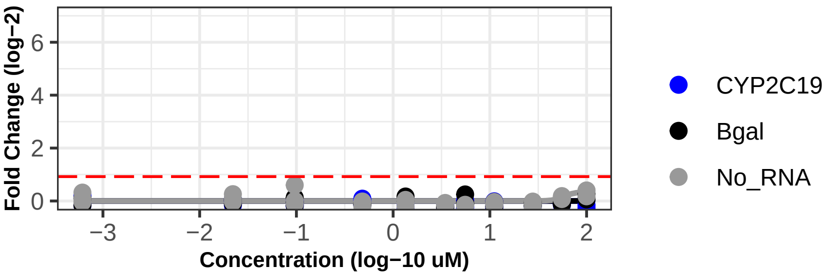


## Procymidone: CYP2A6

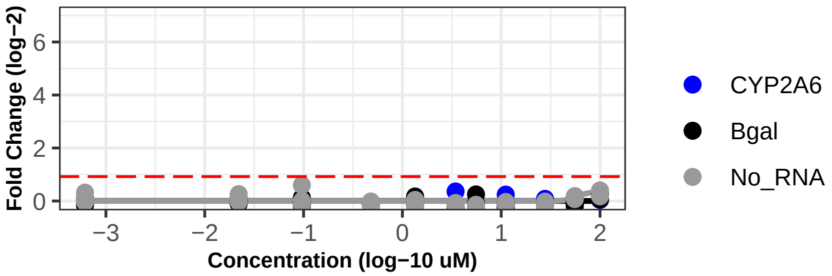


## Procymidone: CYP2D6

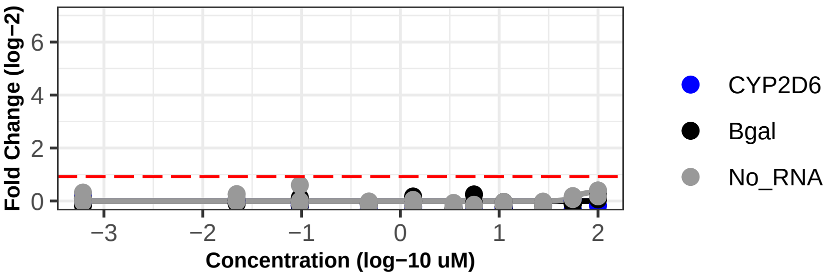


## Procymidone: CYP2B6

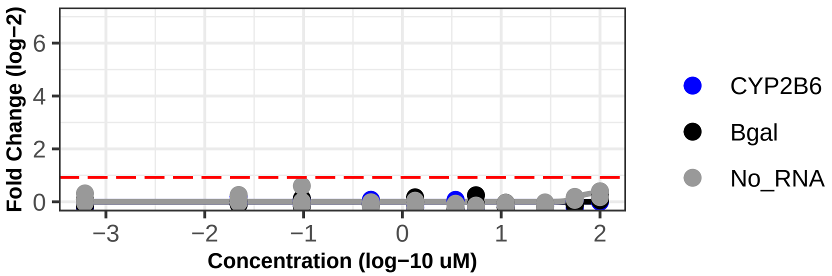


## Procymidone: CYP2E1

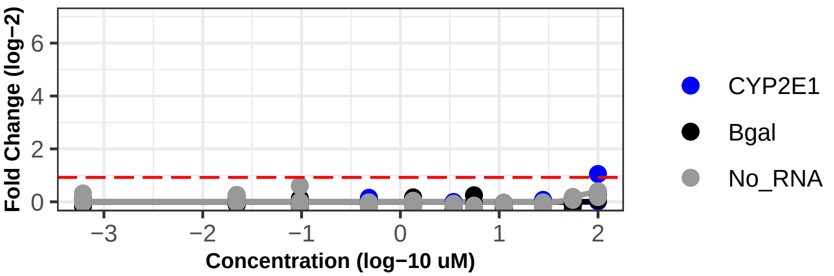


## Procymidone: CYP2C8

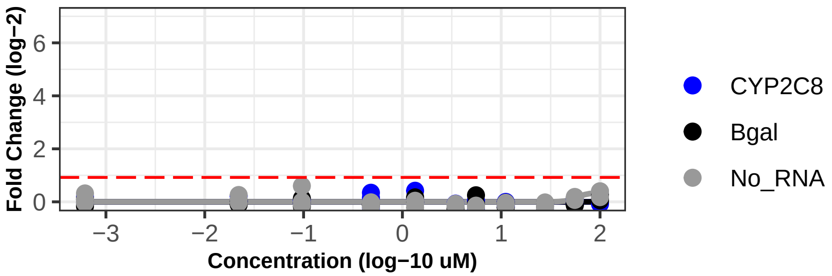


## Procymidone: CYP2J2

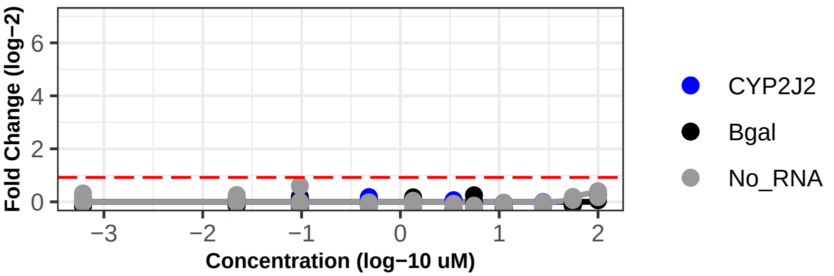


## Procymidone: CYP2C9

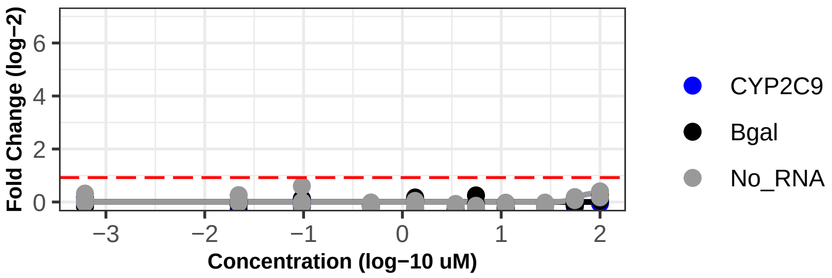


## Procymidone: CYP3A4

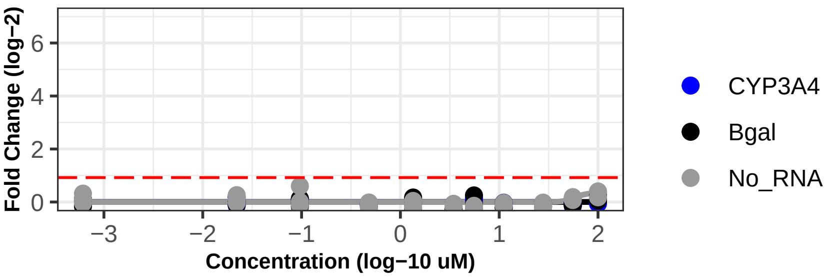

Propargite: CYP1A2

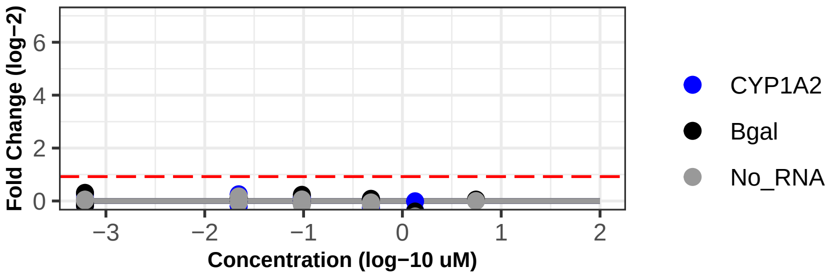


Propargite: CYP2C19

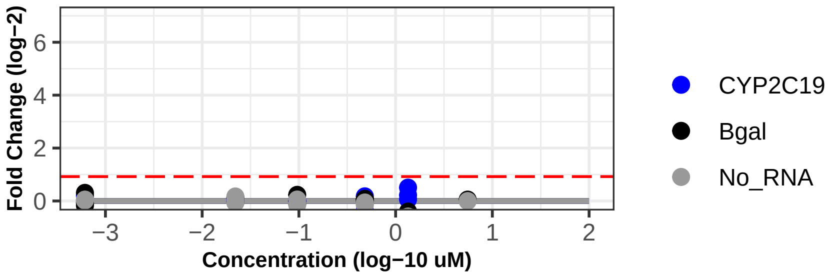


Propargite: CYP2A6

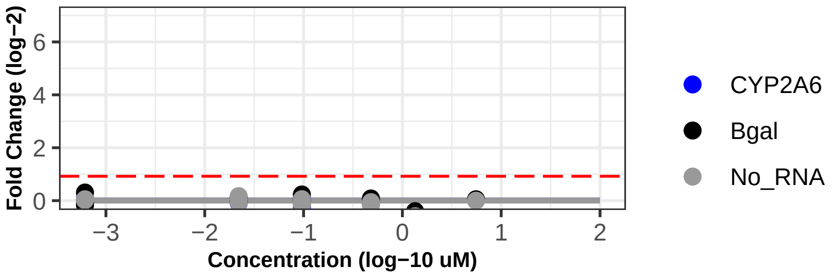


Propargite: CYP2D6

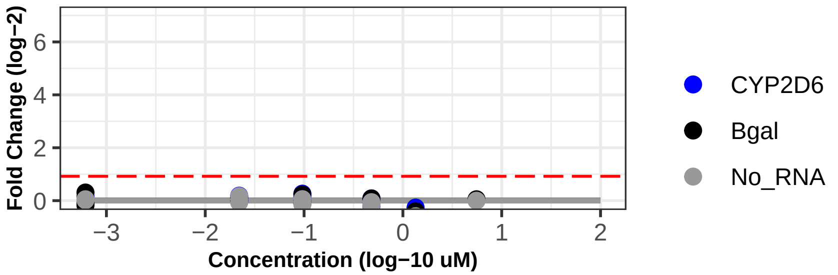


Propargite: CYP2B6

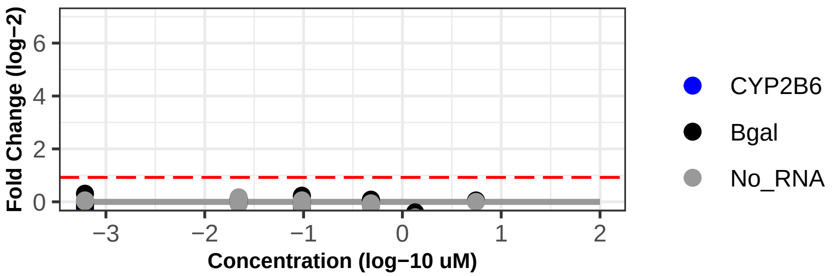


Propargite: CYP2E1

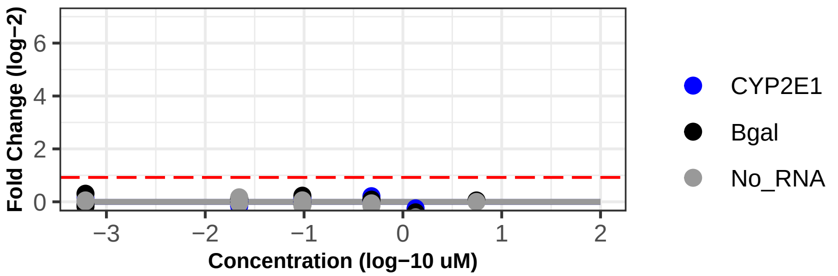


Propargite: CYP2C8

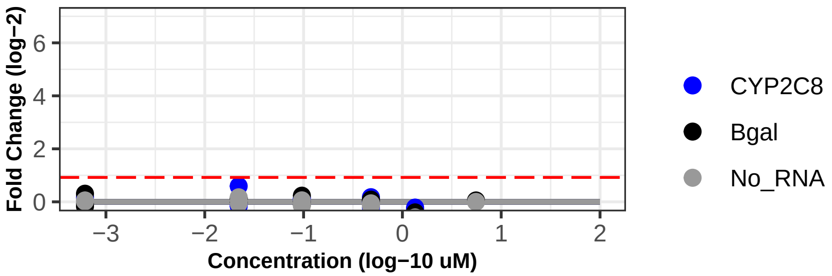


Propargite: CYP2J2

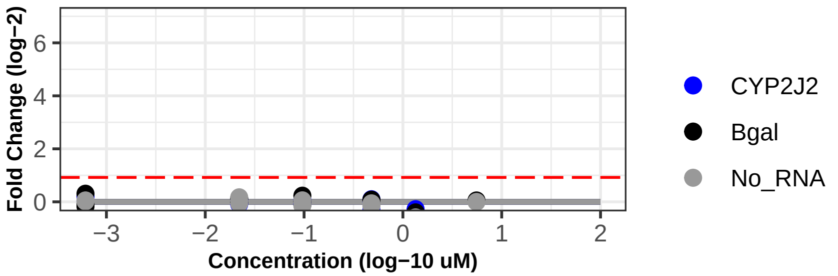


Propargite: CYP2C9

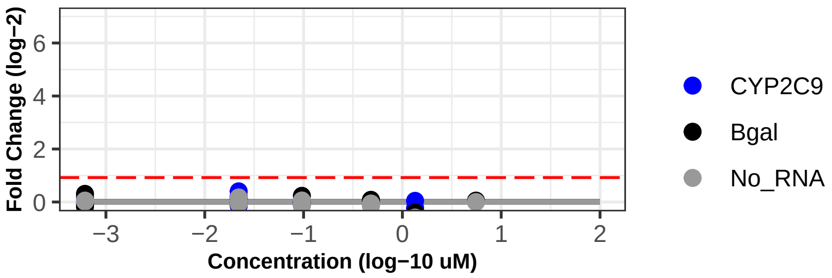


Propargite: CYP3A4

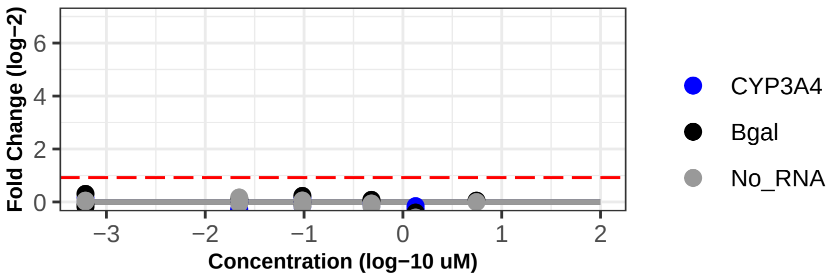

## Propiconazole: CYP1A2

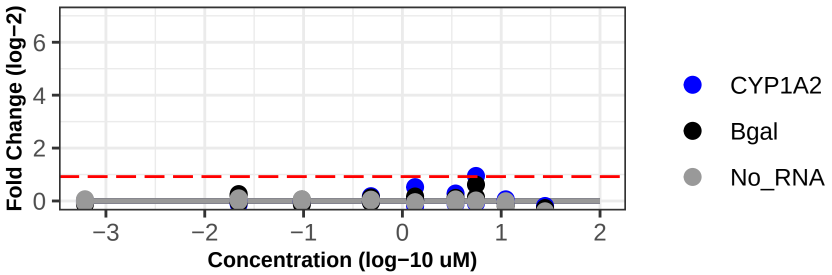


## Propiconazole: CYP2C19

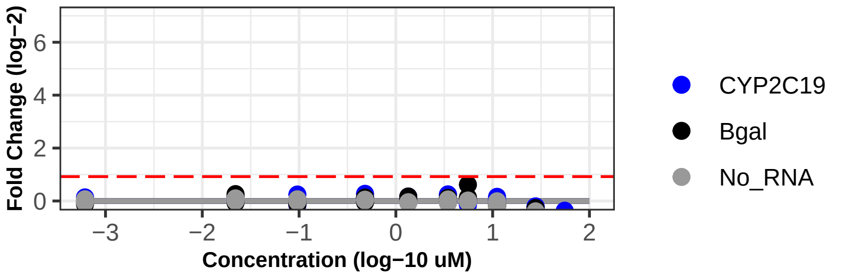


## Propiconazole: CYP2A6

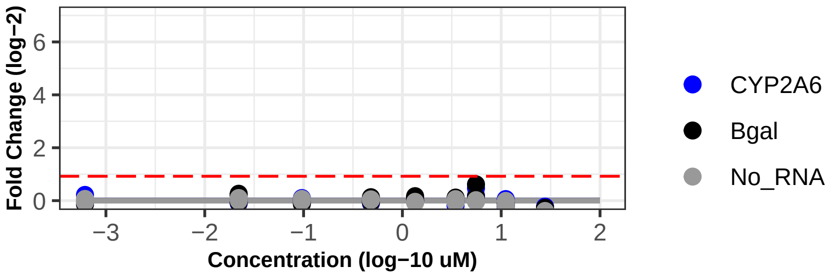


## Propiconazole: CYP2D6

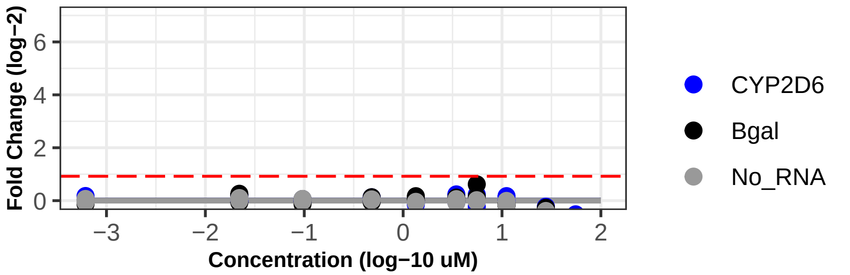


## Propiconazole: CYP2B6

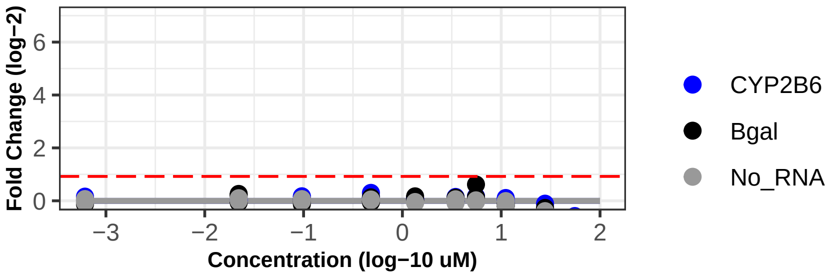


## Propiconazole: CYP2E1

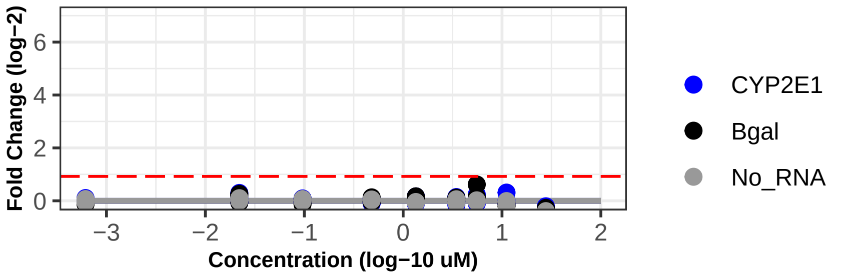


## Propiconazole: CYP2C8

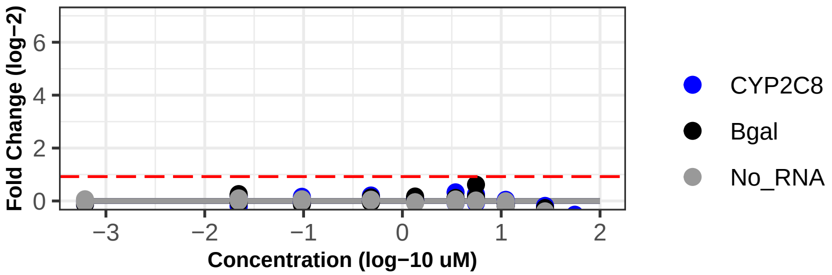


## Propiconazole: CYP2J2

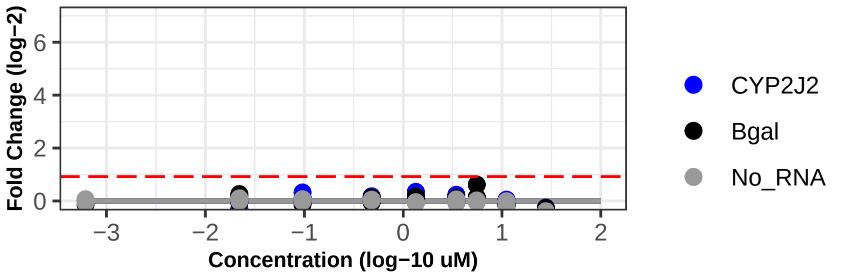


## Propiconazole: CYP2C9

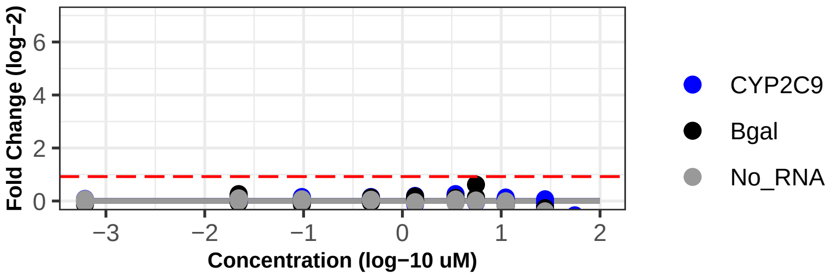


## Propiconazole: CYP3A4

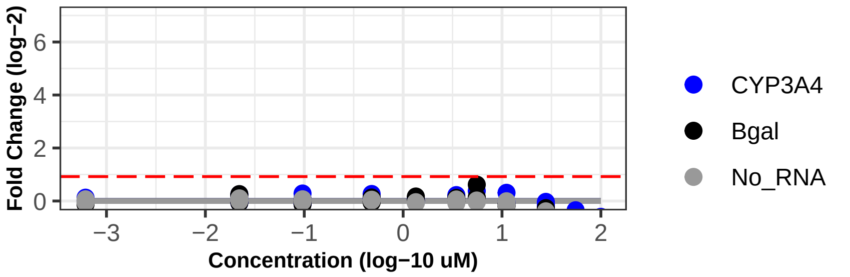

Propyzamide: CYP1A2

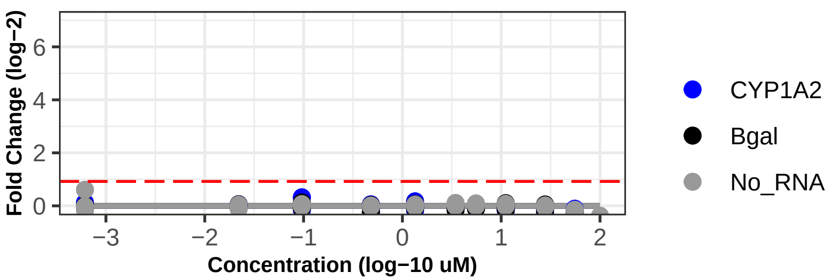


Propyzamide: CYP2C19

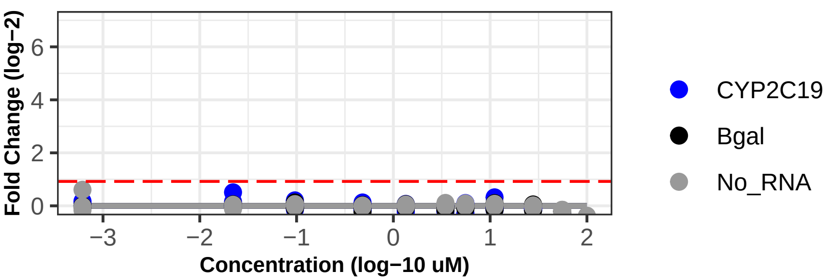


Propyzamide: CYP2A6

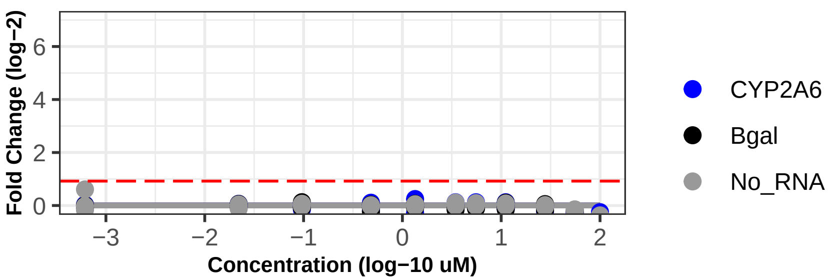


Propyzamide: CYP2D6

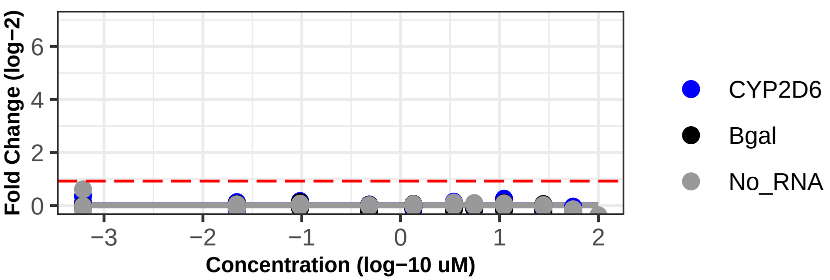


Propyzamide: CYP2B6

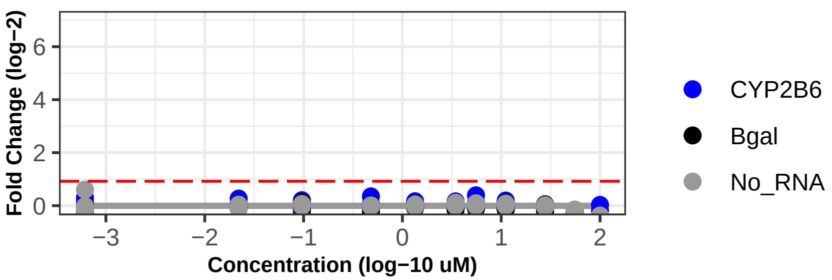


Propyzamide: CYP2E1

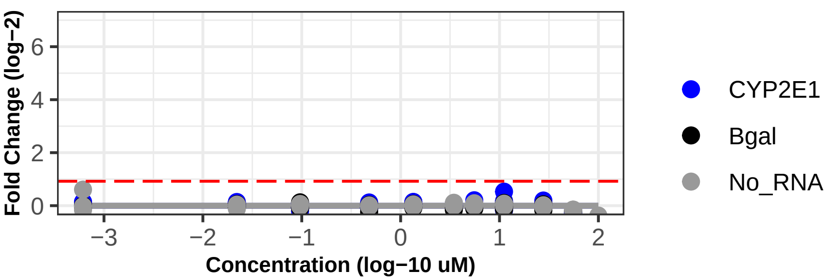


Propyzamide: CYP2C8

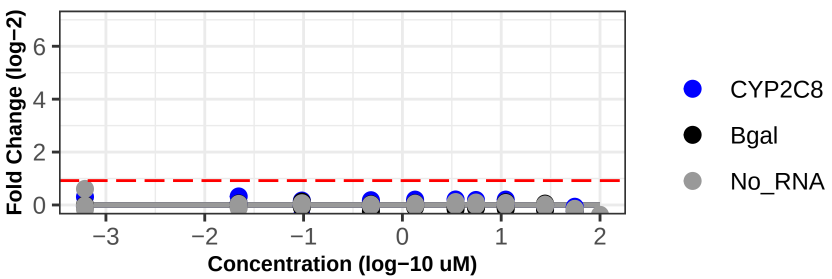


Propyzamide: CYP2J2

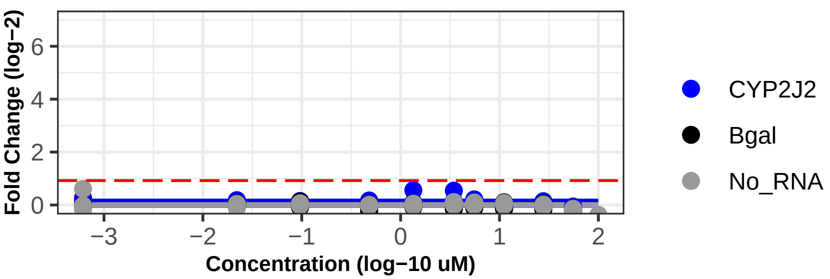


Propyzamide: CYP2C9

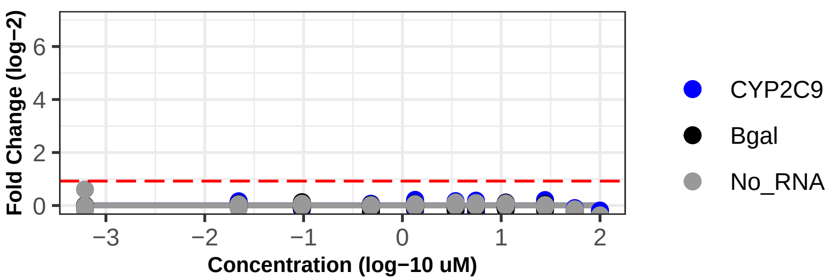


Propyzamide: CYP3A4

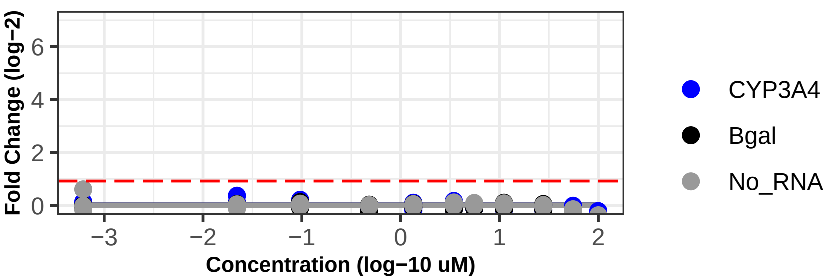

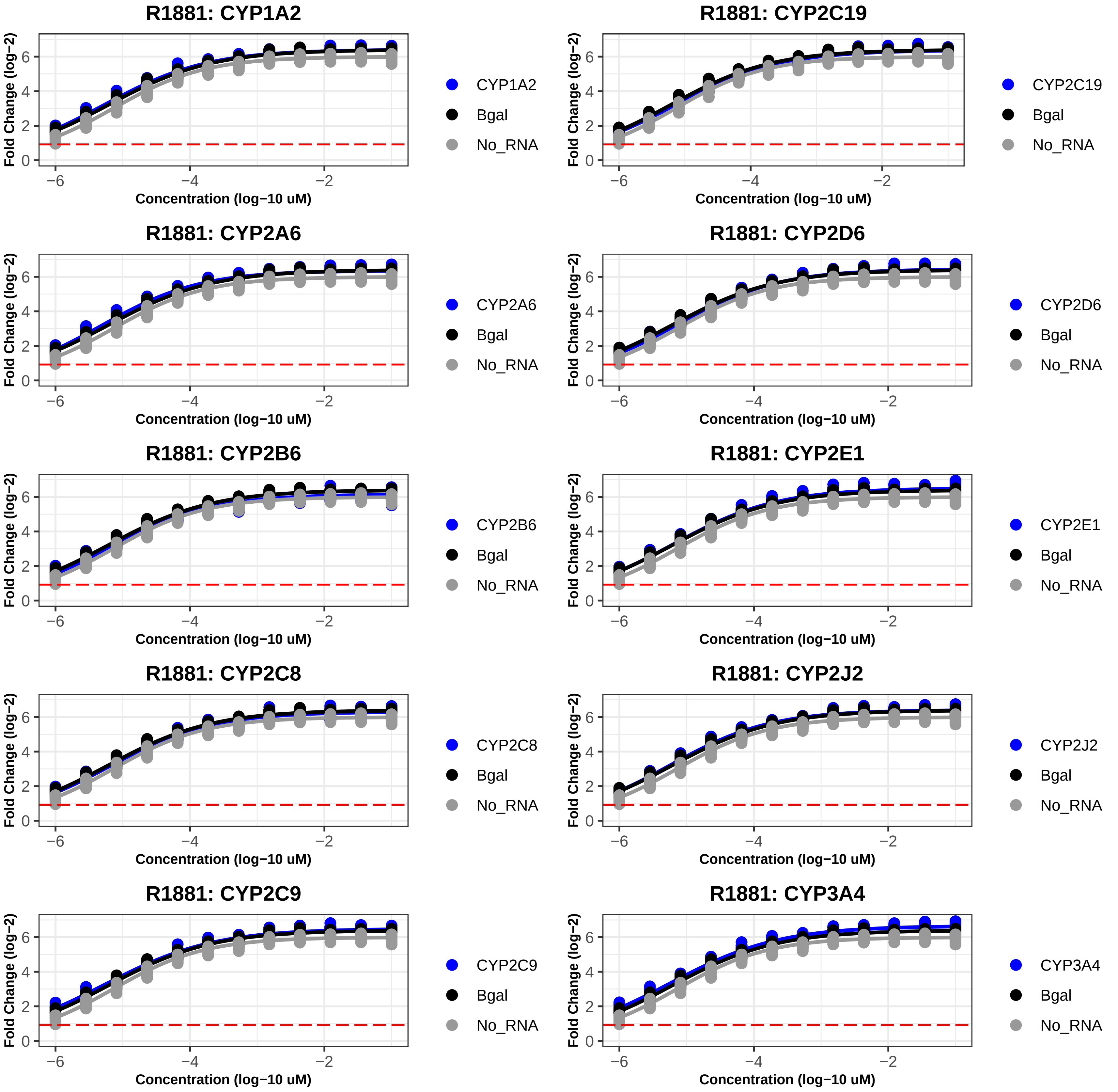
R1881: CYP1A2
R1881: CYP2C19
R1881: CYP2A6
R1881: CYP2D6
R1881: CYP2B6
R1881: CYP2E1
R1881: CYP2C8
R1881: CYP2J2
R1881: CYP2C9
R1881: CYP3A4
Fold Change (log−2)
Concentration (log−10 uM)
CYP1A2
CYP2C19
CYP2A6
CYP2D6
CYP2B6
CYP2E1
CYP2C8
CYP2J2
CYP2C9
CYP3A4
Bgal
No_RNA

**Raloxifene hydrochloride: CYP1A2**

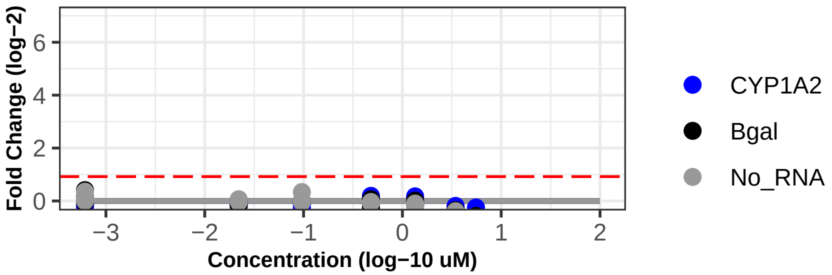


**Raloxifene hydrochloride: CYP2C19**

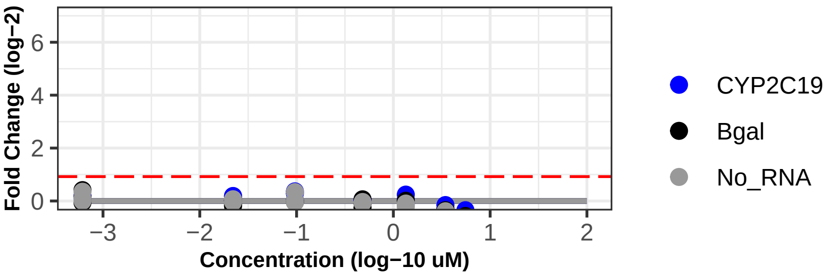


**Raloxifene hydrochloride: CYP2A6**

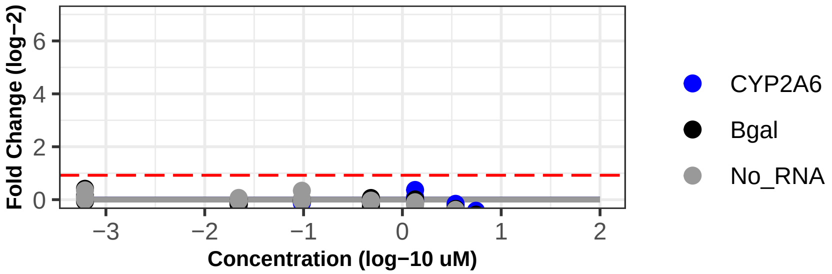


**Raloxifene hydrochloride: CYP2D6**

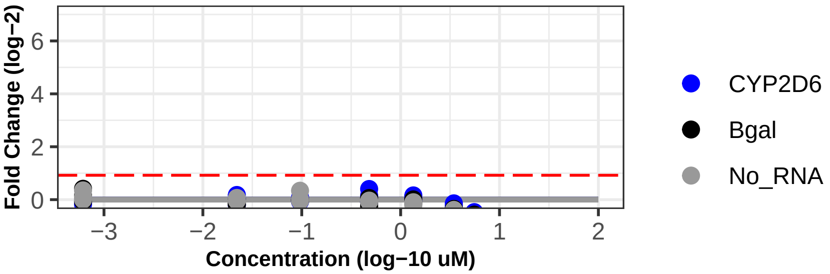


**Raloxifene hydrochloride: CYP2B6**

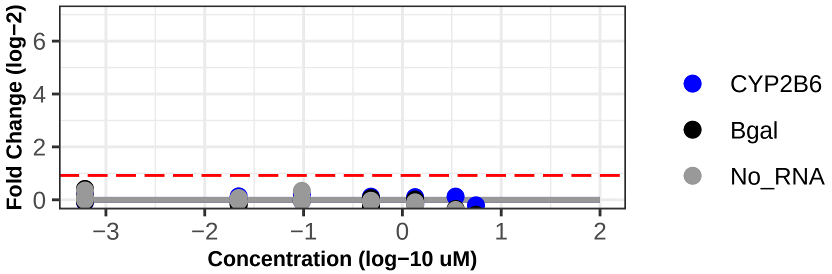


**Raloxifene hydrochloride: CYP2E1**

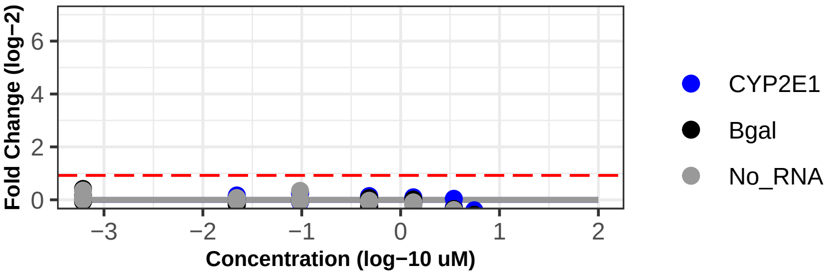


**Raloxifene hydrochloride: CYP2C8**

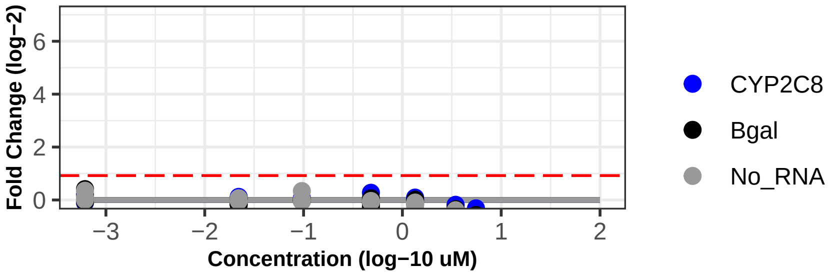


**Raloxifene hydrochloride: CYP2J2**

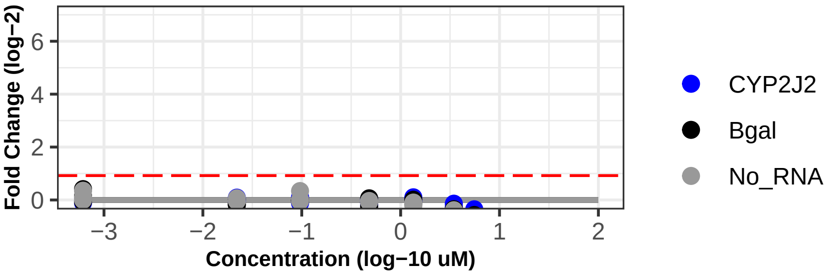


**Raloxifene hydrochloride: CYP2C9**

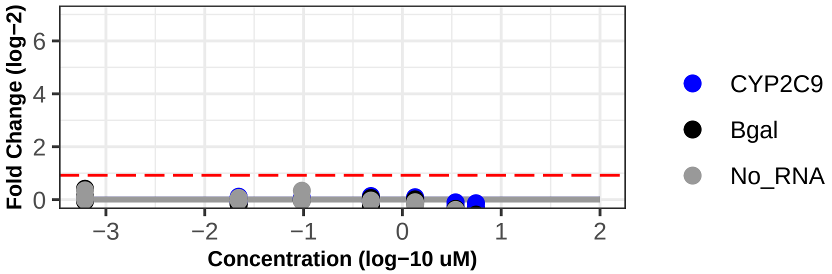


**Raloxifene hydrochloride: CYP3A4**

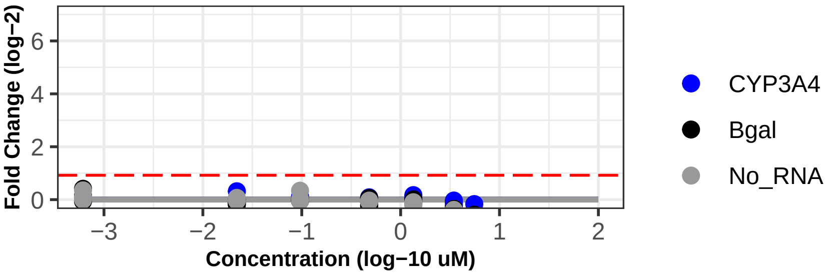

Simazine: CYP1A2

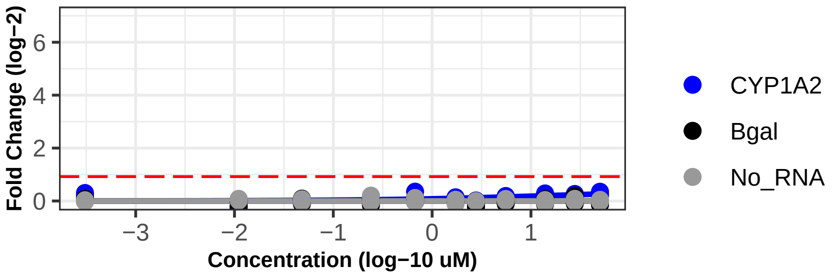


Simazine: CYP2C19

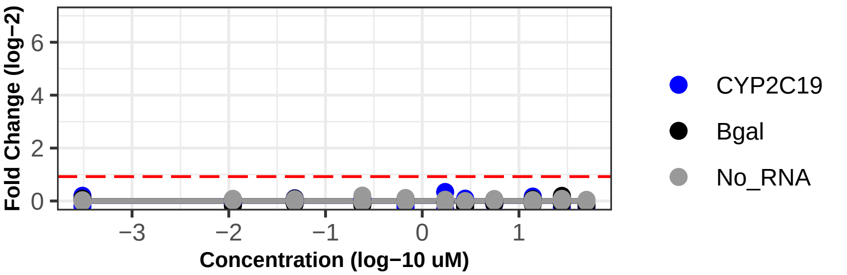


Simazine: CYP2A6

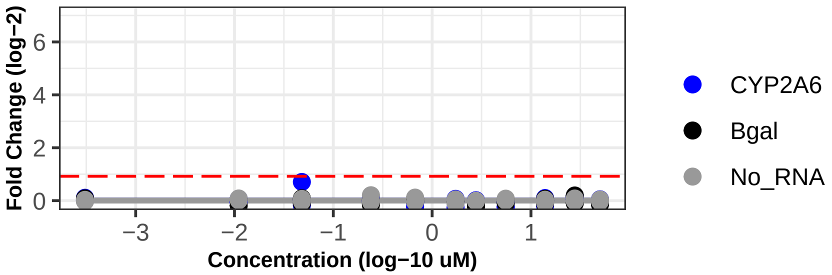


Simazine: CYP2D6

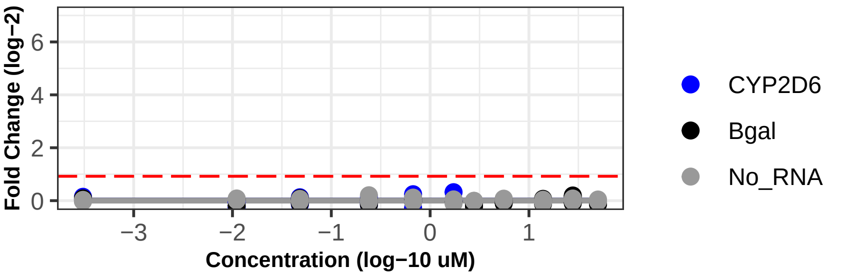


Simazine: CYP2B6

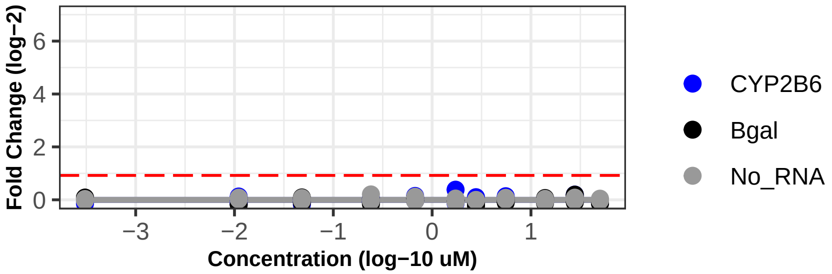


Simazine: CYP2E1

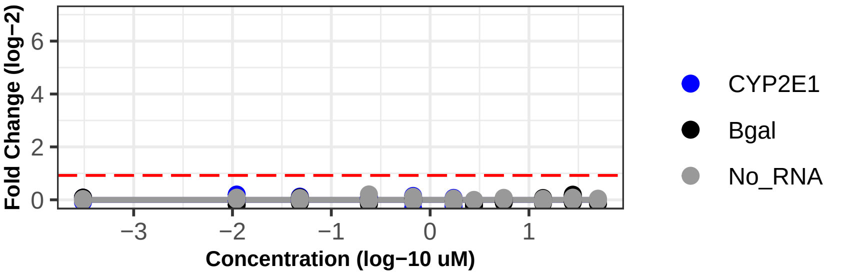


Simazine: CYP2C8

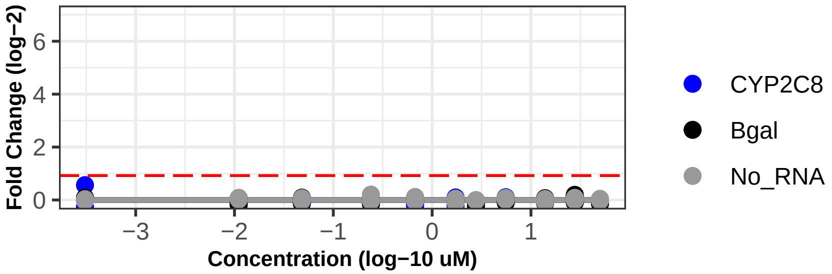


Simazine: CYP2J2

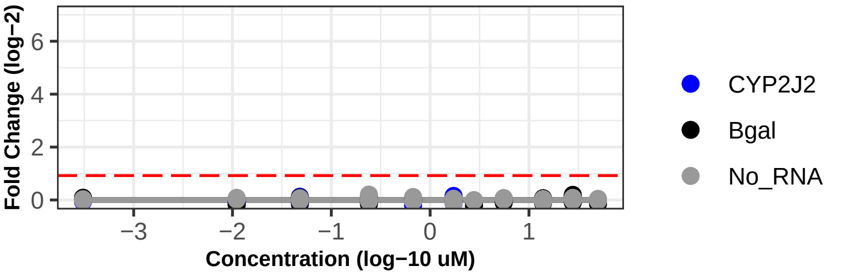


Simazine: CYP2C9

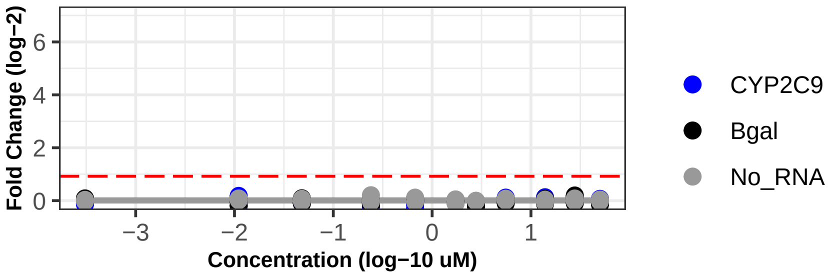


Simazine: CYP3A4

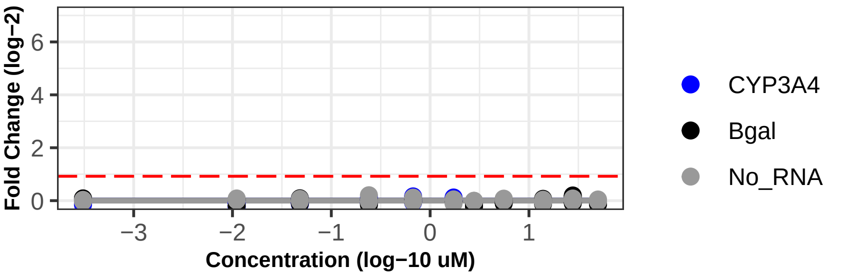

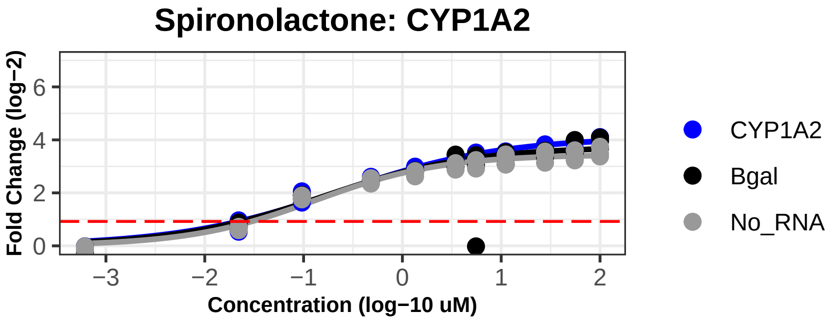
Spironolactone: CYP1A2
Fold Change (log−2)
Concentration (log−10 uM)
CYP1A2
Bgal
No_RNA

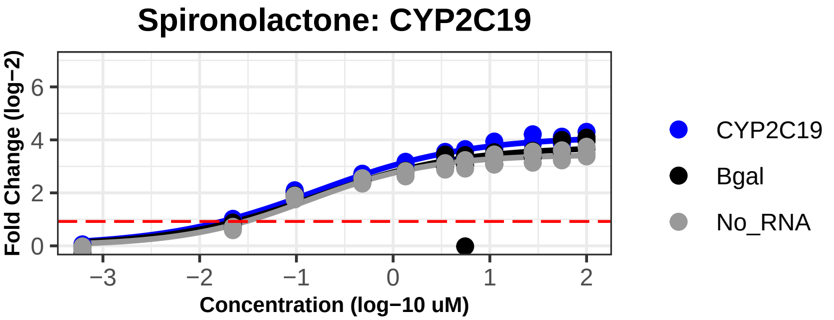
Spironolactone: CYP2C19
Fold Change (log−2)
Concentration (log−10 uM)
CYP2C19
Bgal
No_RNA

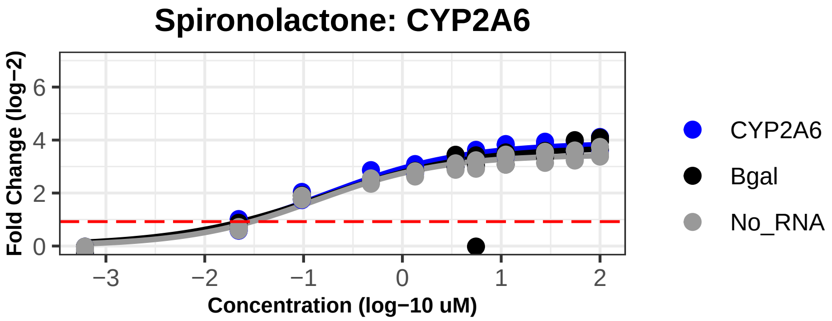
Spironolactone: CYP2A6
Fold Change (log−2)
Concentration (log−10 uM)
CYP2A6
Bgal
No_RNA

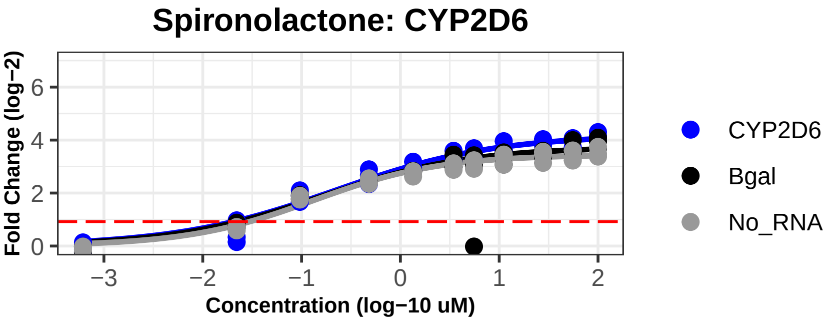
Spironolactone: CYP2D6
Fold Change (log−2)
Concentration (log−10 uM)
CYP2D6
Bgal
No_RNA

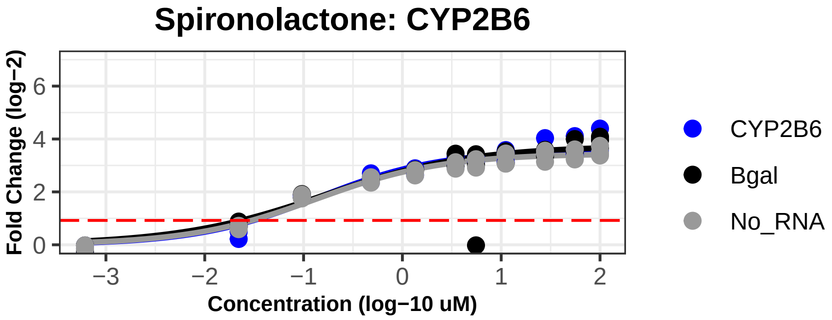
Spironolactone: CYP2B6
Fold Change (log−2)
Concentration (log−10 uM)
CYP2B6
Bgal
No_RNA

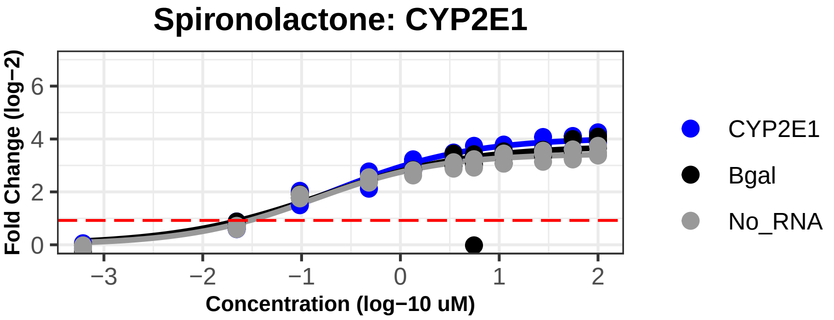
Spironolactone: CYP2E1
Fold Change (log−2)
Concentration (log−10 uM)
CYP2E1
Bgal
No_RNA

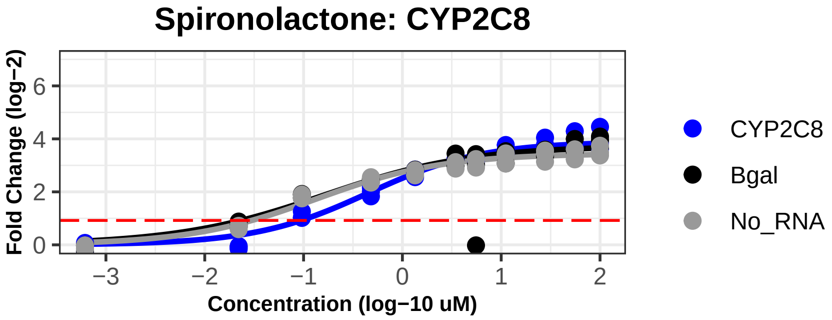
Spironolactone: CYP2C8
Fold Change (log−2)
Concentration (log−10 uM)
CYP2C8
Bgal
No_RNA

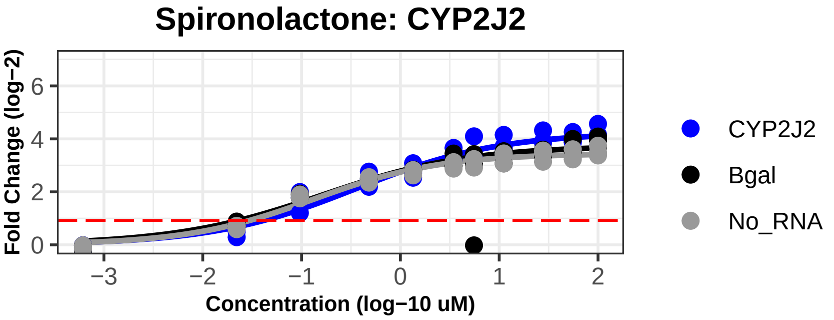
Spironolactone: CYP2J2
Fold Change (log−2)
Concentration (log−10 uM)
CYP2J2
Bgal
No_RNA

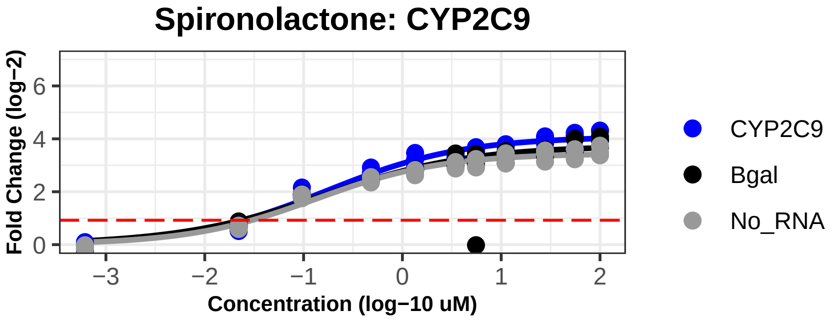
Spironolactone: CYP2C9
Fold Change (log−2)
Concentration (log−10 uM)
CYP2C9
Bgal
No_RNA

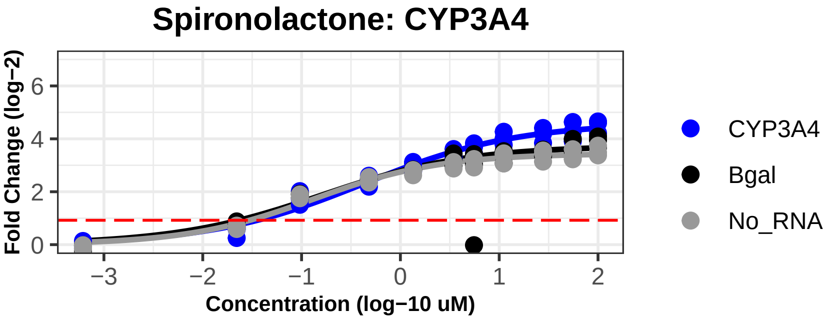
Spironolactone: CYP3A4
Fold Change (log−2)
Concentration (log−10 uM)
CYP3A4
Bgal
No_RNA

## Tamoxifen: CYP1A2

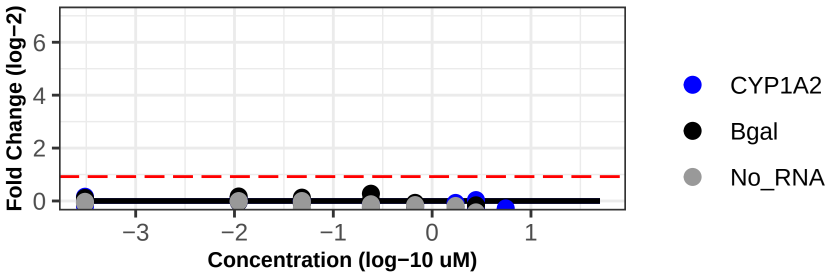


## Tamoxifen: CYP2C19

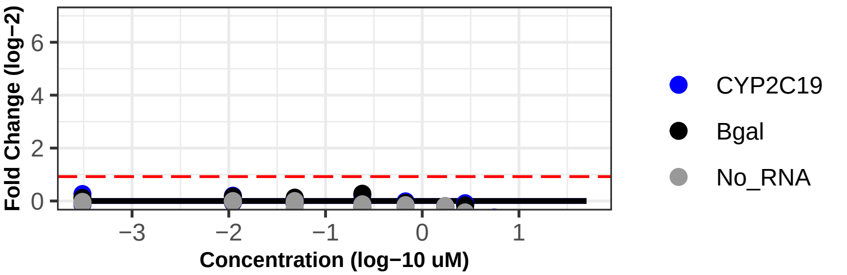


## Tamoxifen: CYP2A6

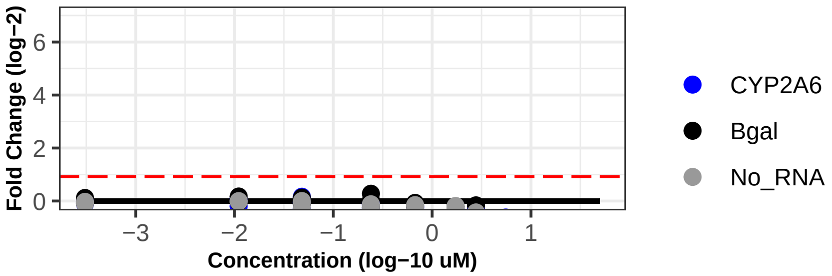


## Tamoxifen: CYP2D6

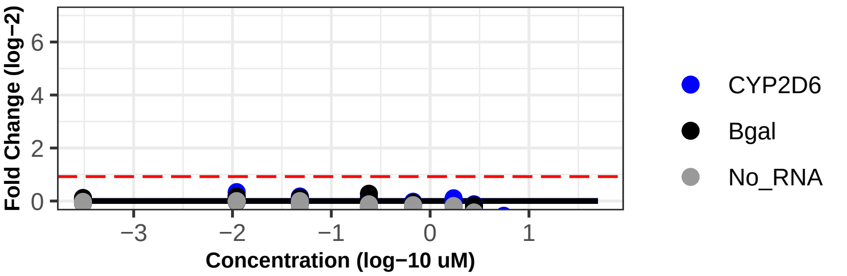


## Tamoxifen: CYP2B6

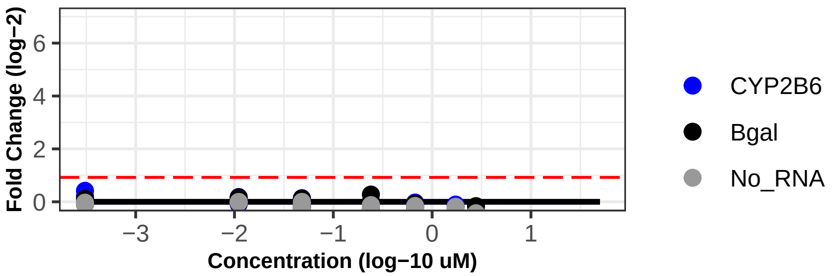


## Tamoxifen: CYP2E1

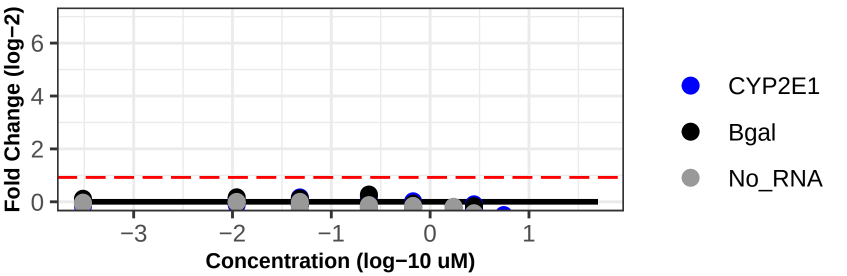


## Tamoxifen: CYP2C8

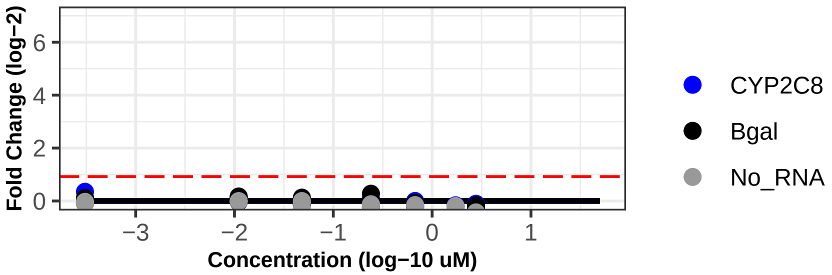


## Tamoxifen: CYP2J2

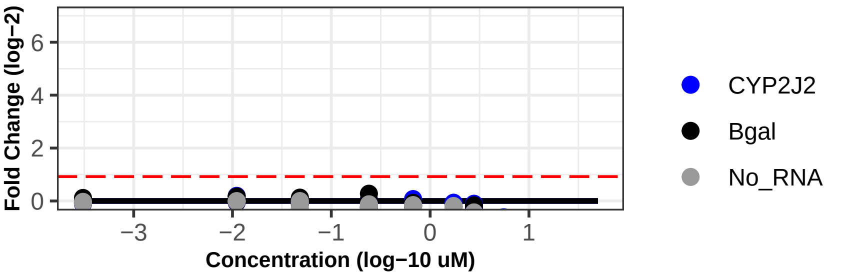


## Tamoxifen: CYP2C9

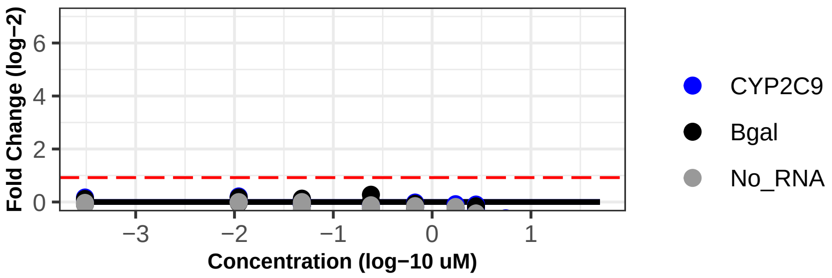


## Tamoxifen: CYP3A4

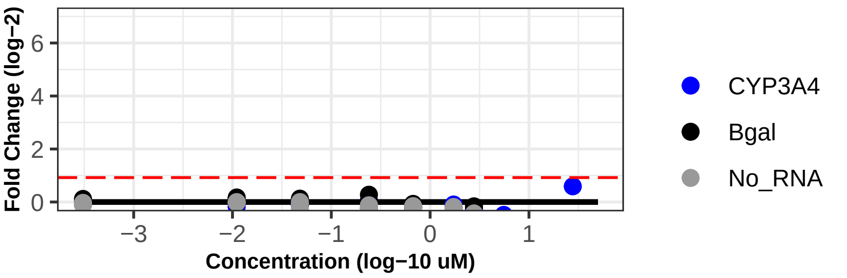

## Tebuconazole: CYP1A2

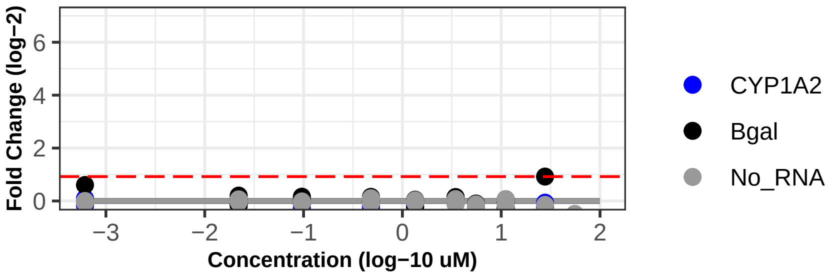


## Tebuconazole: CYP2C19

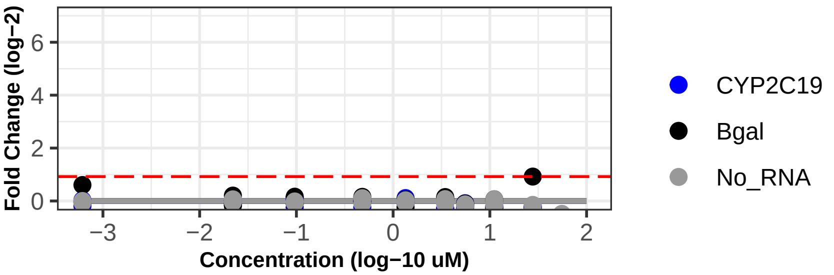


## Tebuconazole: CYP2A6

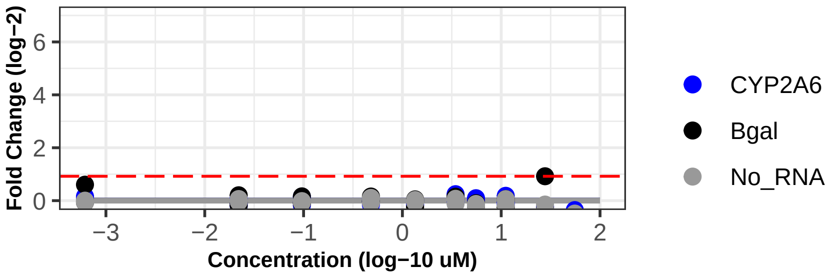


## Tebuconazole: CYP2D6

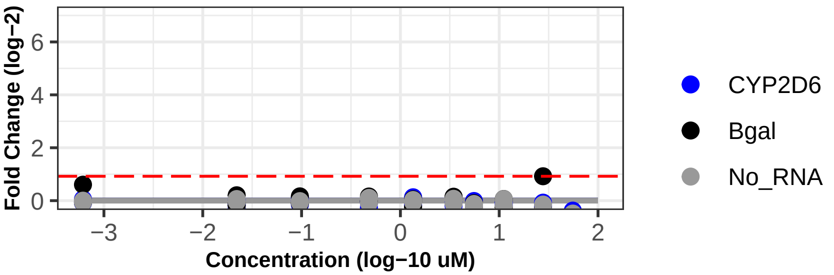


## Tebuconazole: CYP2B6

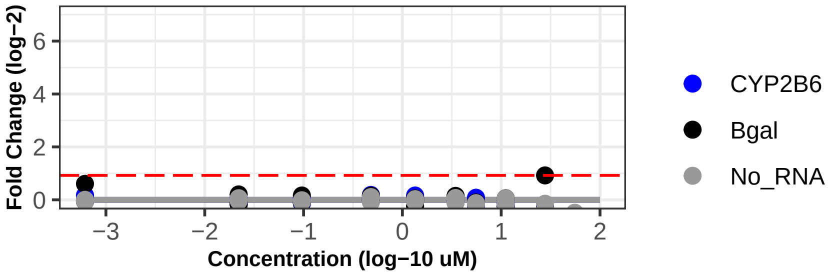


## Tebuconazole: CYP2E1

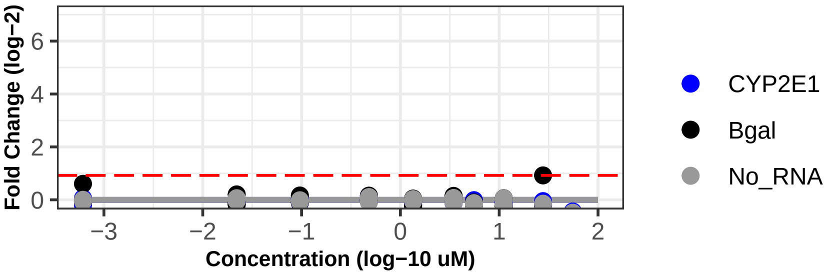


## Tebuconazole: CYP2C8

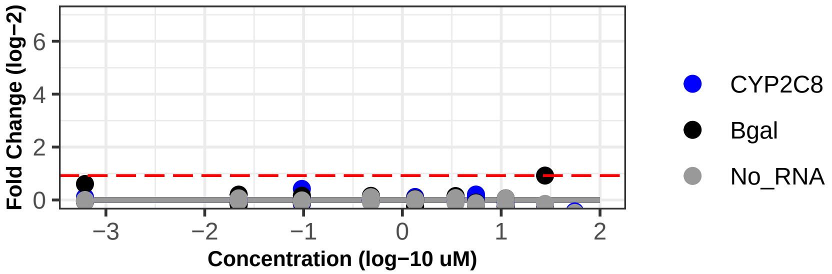


## Tebuconazole: CYP2J2

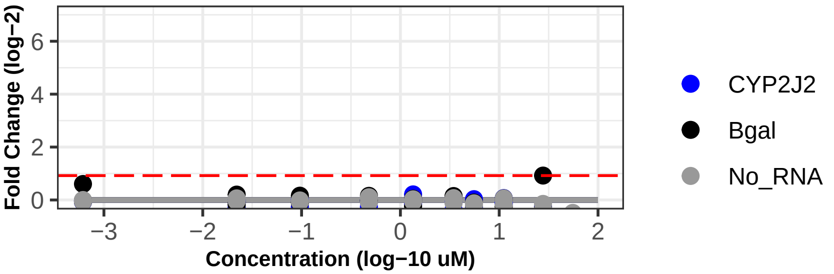


## Tebuconazole: CYP2C9

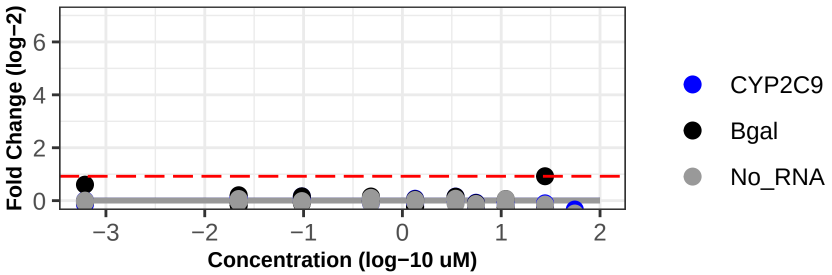


## Tebuconazole: CYP3A4

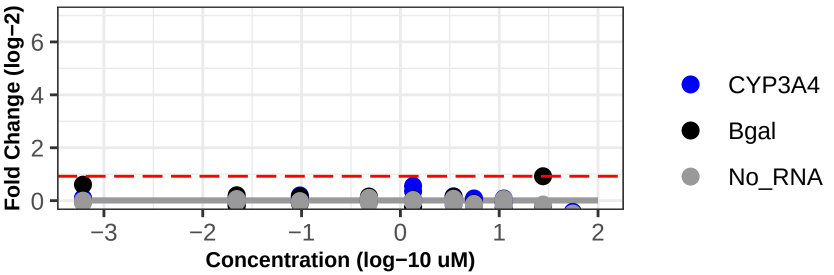

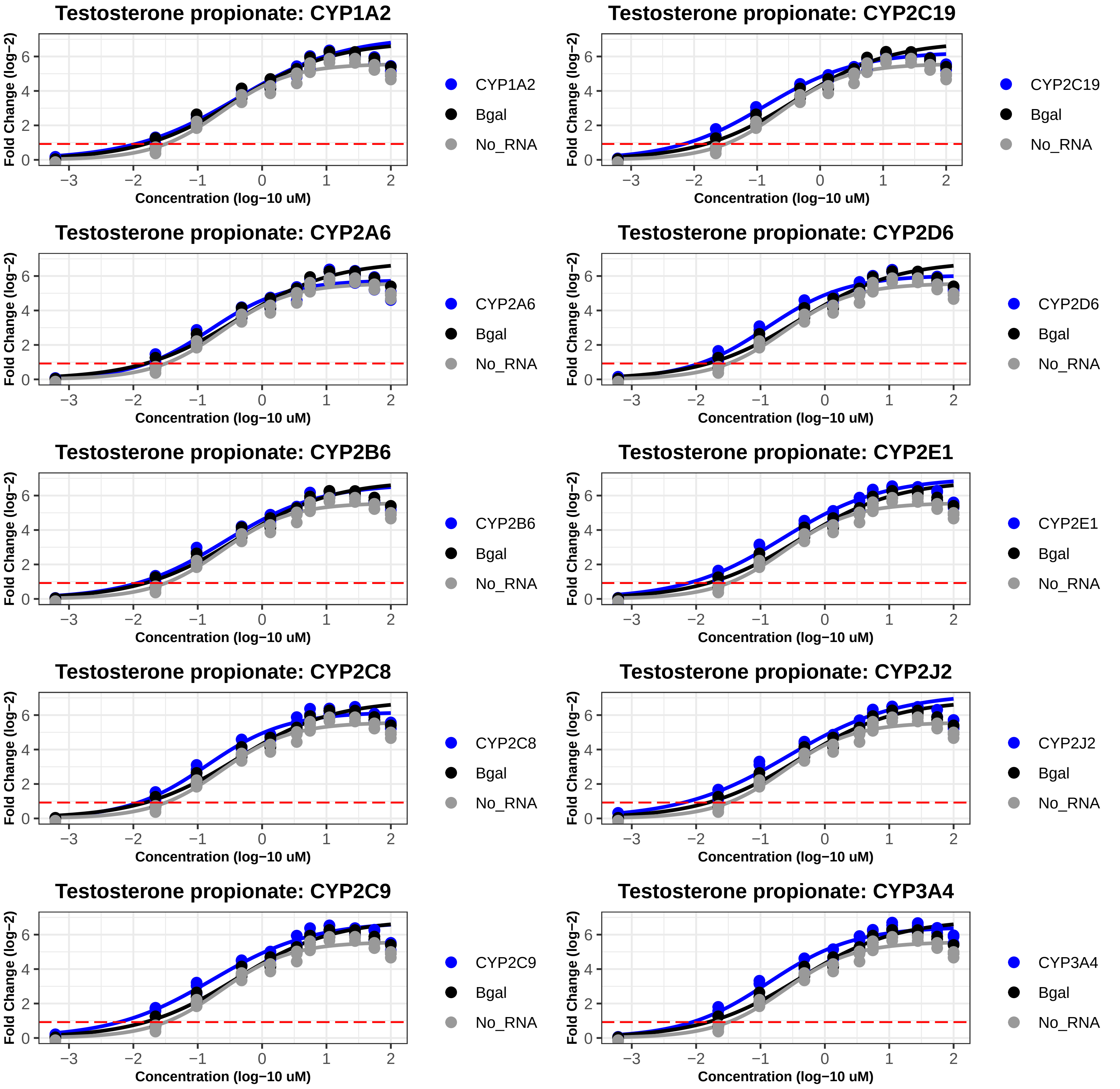
Testosterone propionate: CYP1A2
Testosterone propionate: CYP2C19
Testosterone propionate: CYP2A6
Testosterone propionate: CYP2D6
Testosterone propionate: CYP2B6
Testosterone propionate: CYP2E1
Testosterone propionate: CYP2C8
Testosterone propionate: CYP2J2
Testosterone propionate: CYP2C9
Testosterone propionate: CYP3A4
Fold Change (log−2)
Concentration (log−10 uM)
CYP1A2
CYP2C19
CYP2A6
CYP2D6
CYP2B6
CYP2E1
CYP2C8
CYP2J2
CYP2C9
CYP3A4
Bgal
No_RNA

### Tetramethrin: CYP1A2

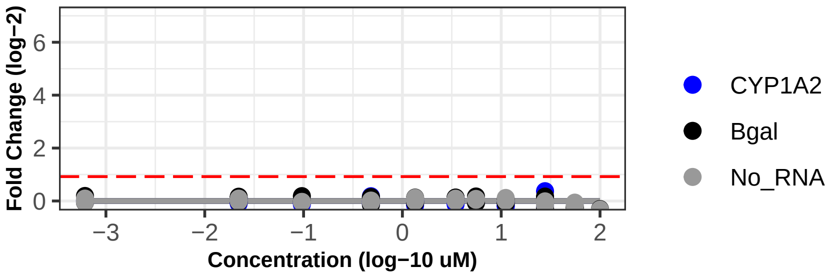


### Tetramethrin: CYP2C19

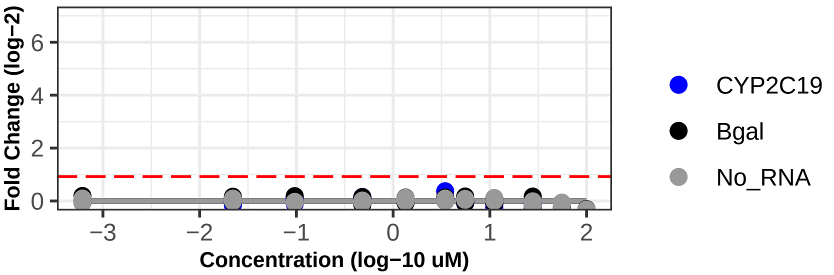


### Tetramethrin: CYP2A6

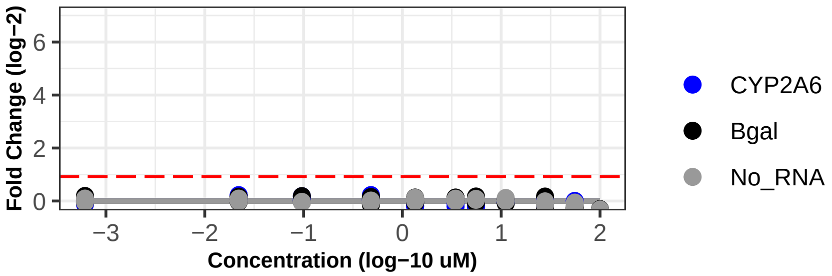


### Tetramethrin: CYP2D6

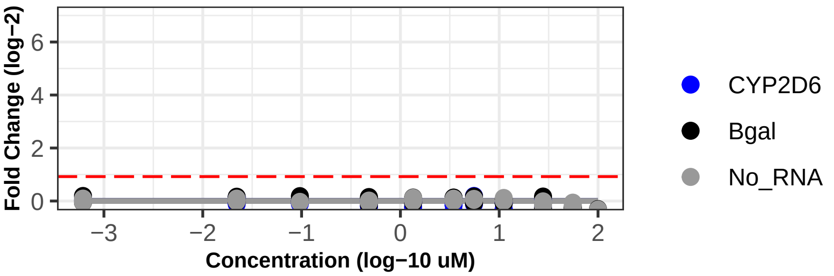


### Tetramethrin: CYP2B6

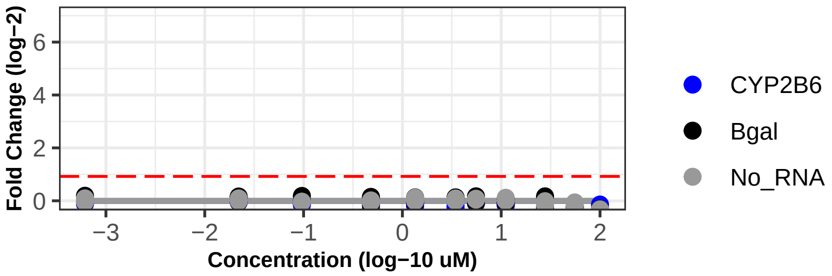


### Tetramethrin: CYP2E1

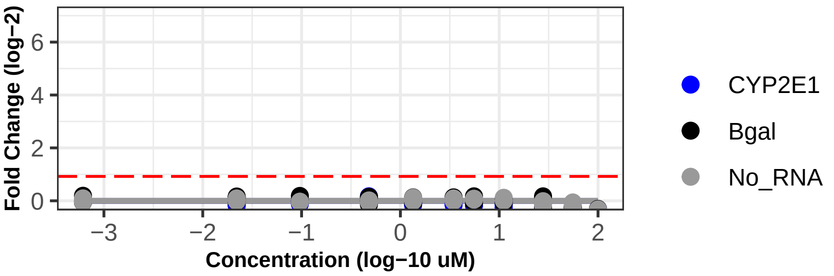


### Tetramethrin: CYP2C8

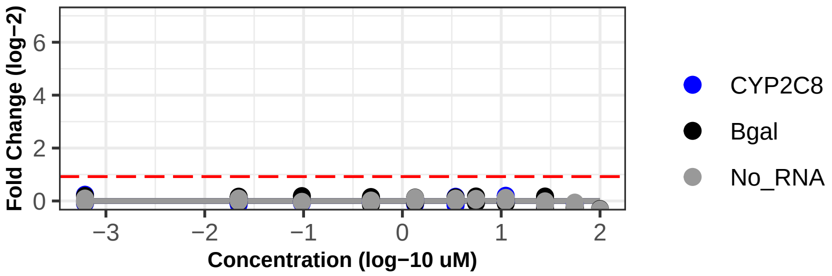


### Tetramethrin: CYP2J2

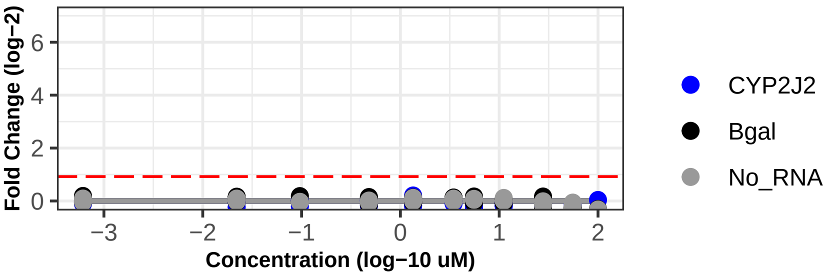


### Tetramethrin: CYP2C9

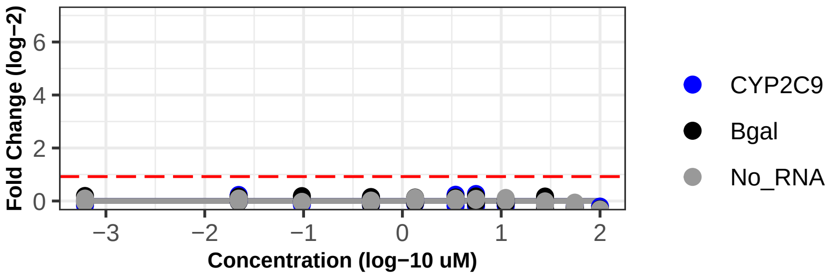


### Tetramethrin: CYP3A4

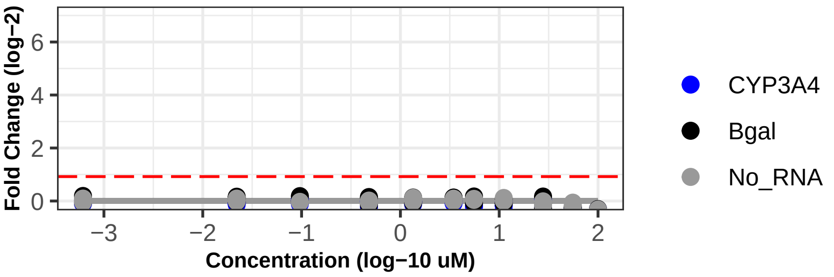

## Triadimefon: CYP1A2

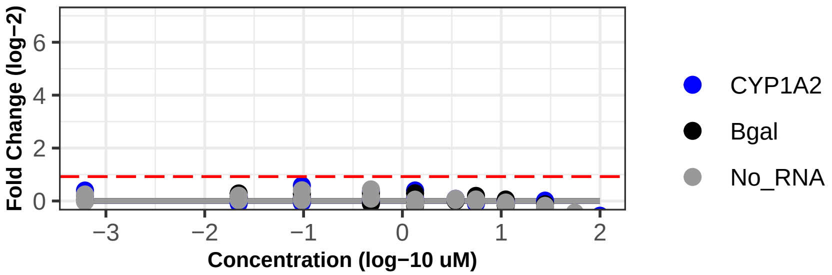


## Triadimefon: CYP2C19

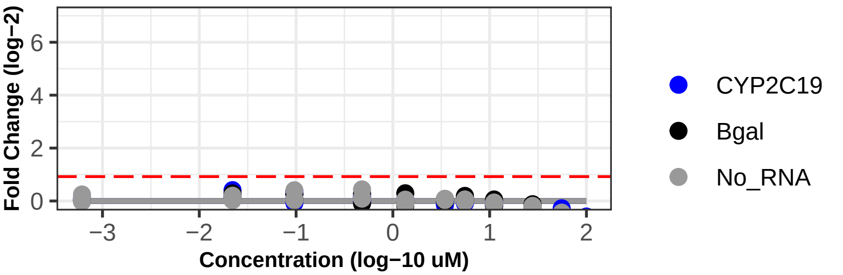


## Triadimefon: CYP2A6

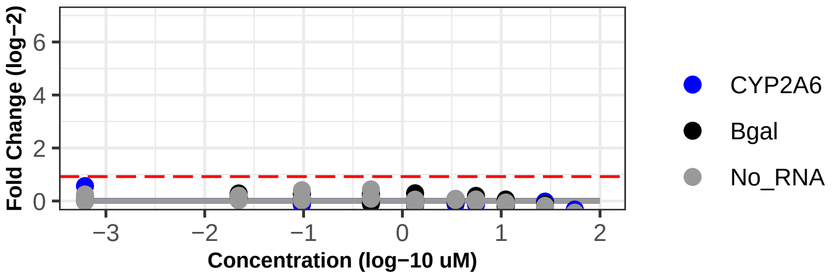


## Triadimefon: CYP2D6

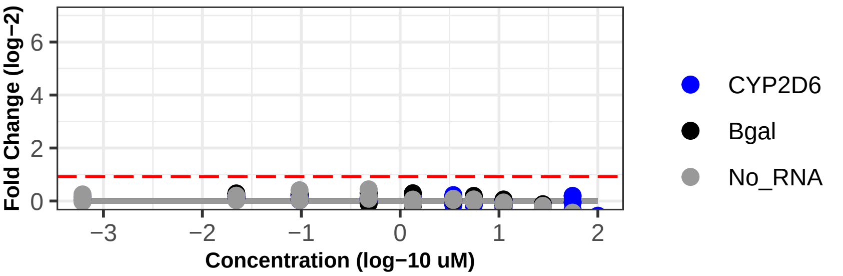


## Triadimefon: CYP2B6

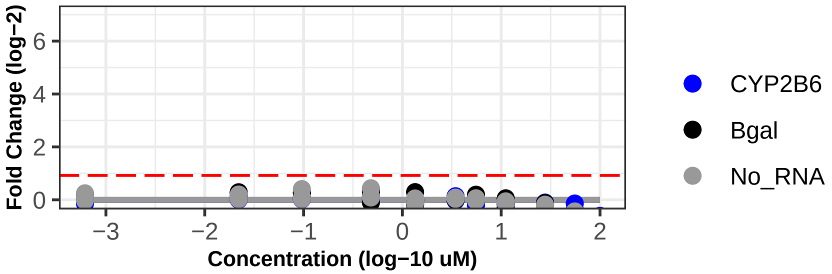


## Triadimefon: CYP2E1

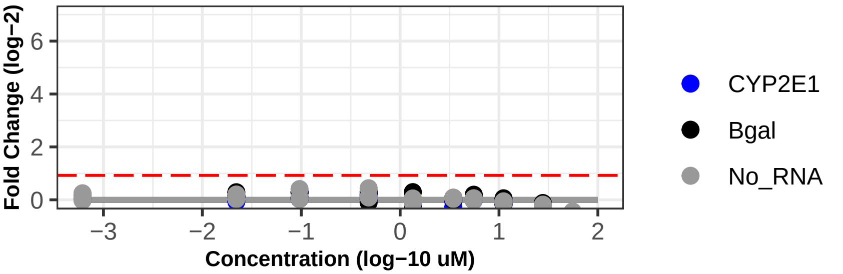


## Triadimefon: CYP2C8

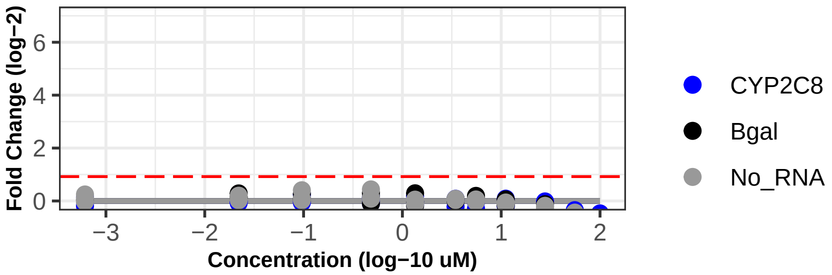


## Triadimefon: CYP2J2

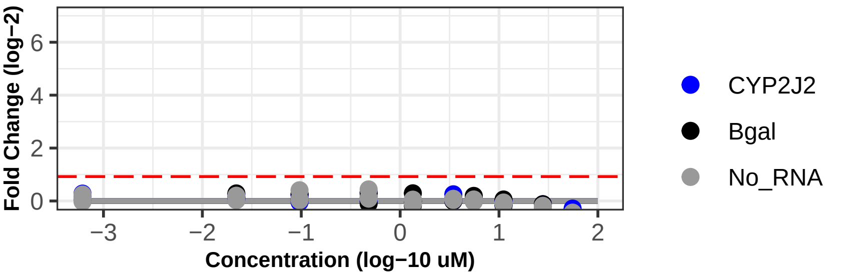


## Triadimefon: CYP2C9

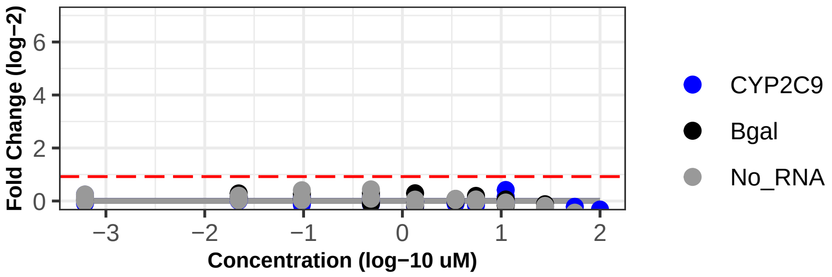


## Triadimefon: CYP3A4

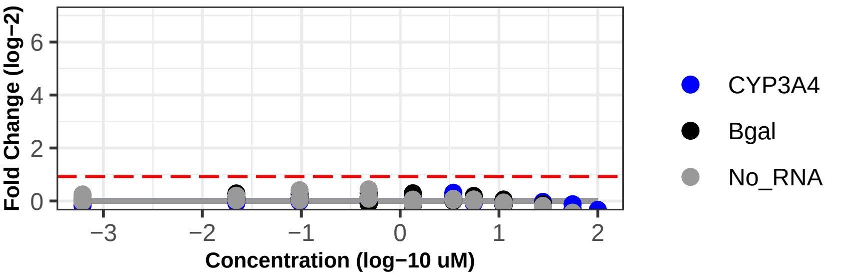

## Tributylchlorostannane: CYP1A2

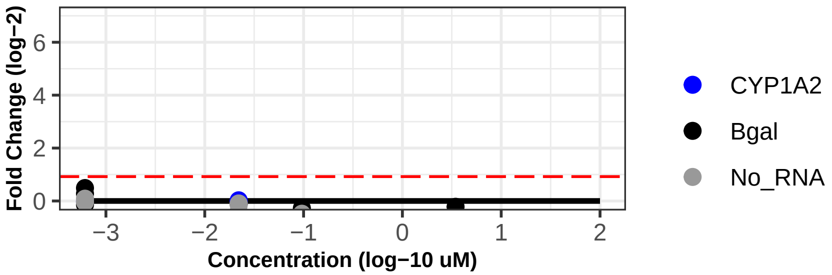


## Tributylchlorostannane: CYP2C19

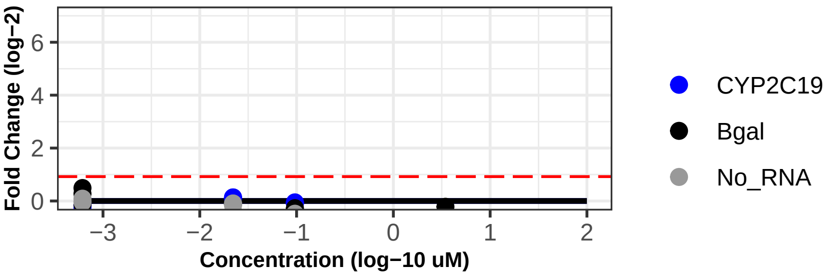


## Tributylchlorostannane: CYP2A6

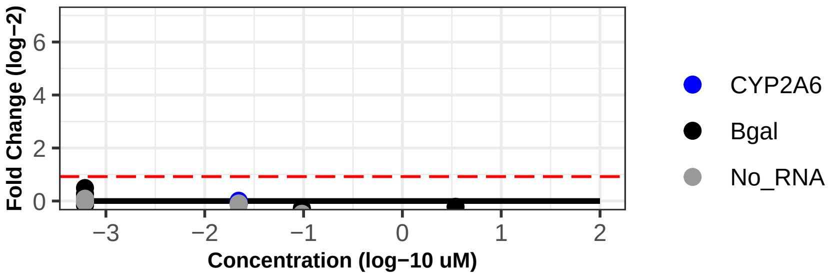


## Tributylchlorostannane: CYP2D6

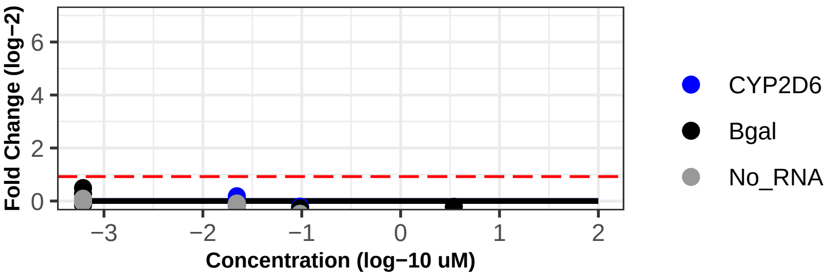


## Tributylchlorostannane: CYP2B6

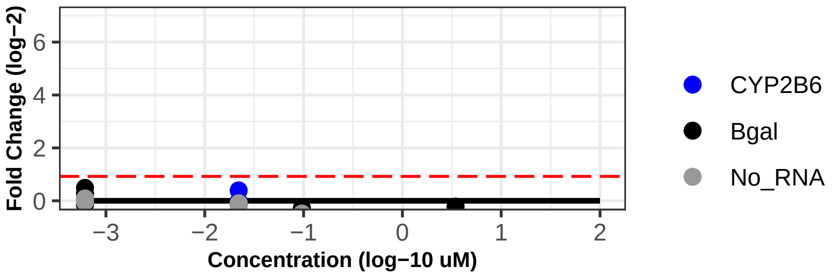


## Tributylchlorostannane: CYP2E1

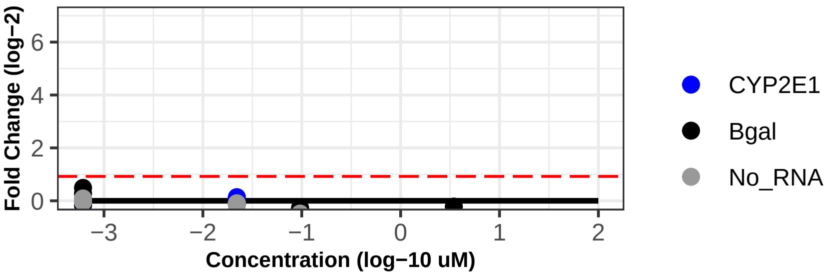


## Tributylchlorostannane: CYP2C8

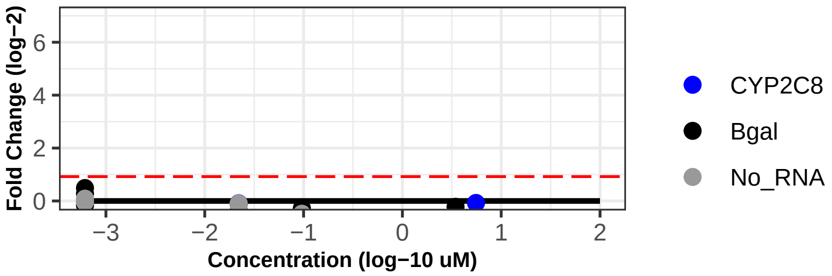


## Tributylchlorostannane: CYP2J2

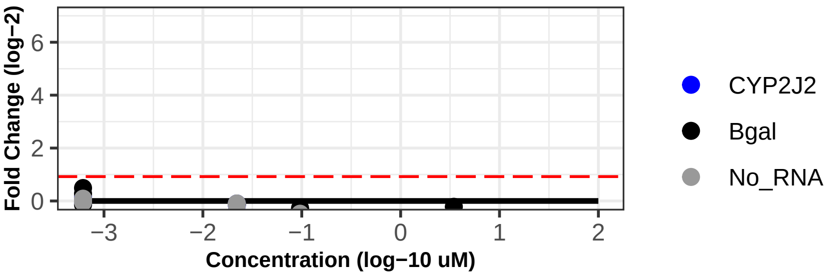


## Tributylchlorostannane: CYP2C9

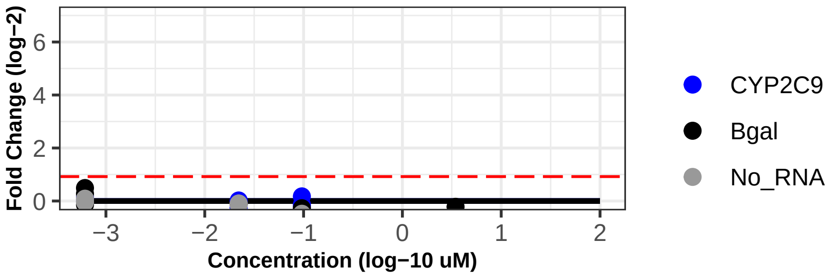


## Tributylchlorostannane: CYP3A4

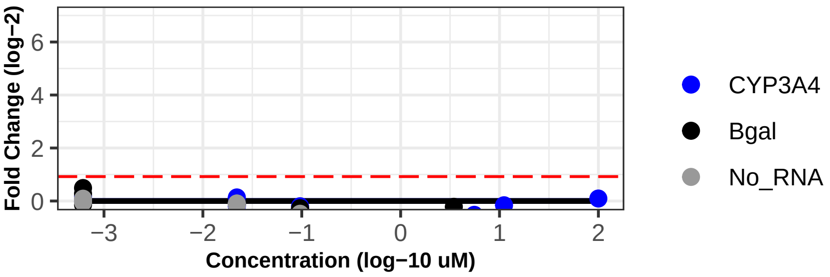

Trifluralin: CYP1A2

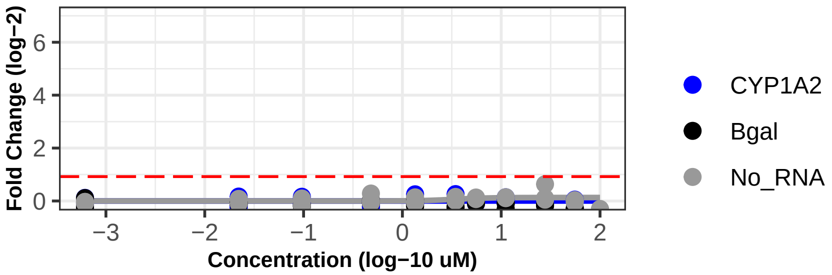


Trifluralin: CYP2C19

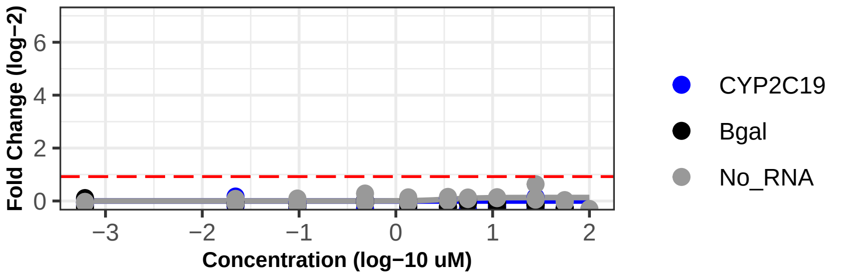


Trifluralin: CYP2A6

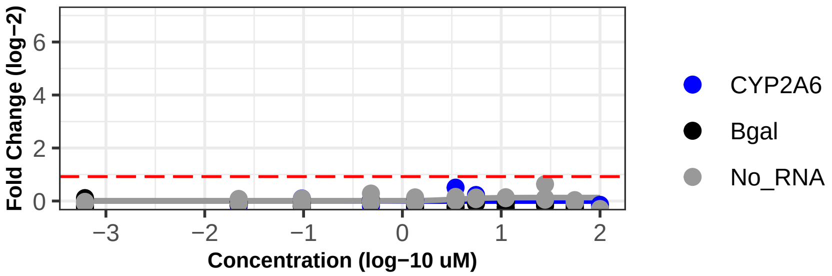


Trifluralin: CYP2D6

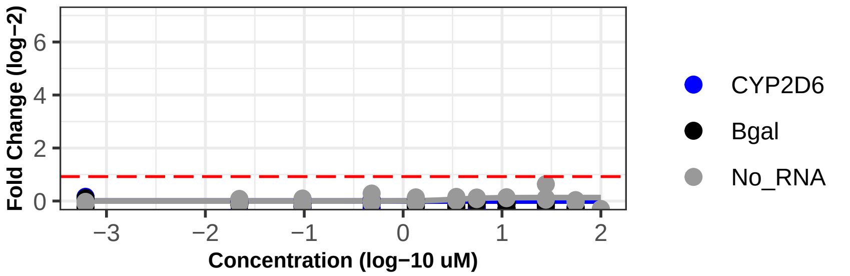


Trifluralin: CYP2B6

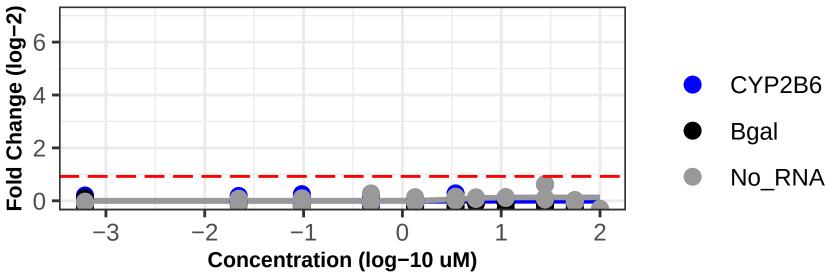


Trifluralin: CYP2E1

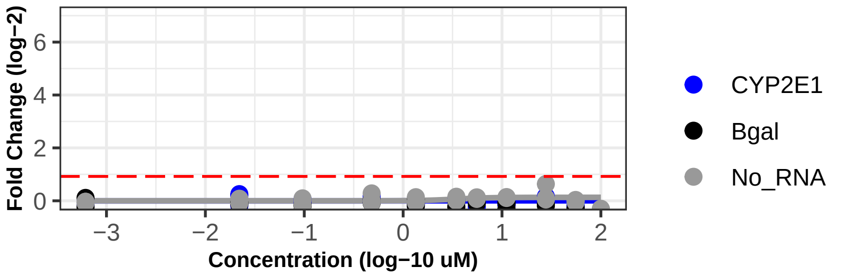


Trifluralin: CYP2C8

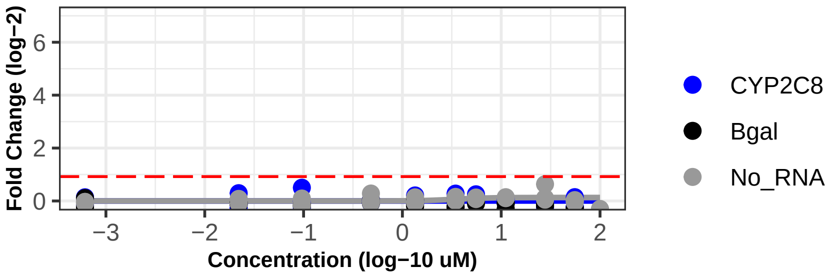


Trifluralin: CYP2J2

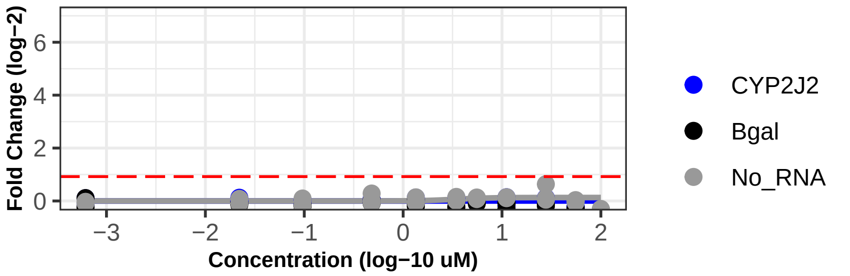


Trifluralin: CYP2C9

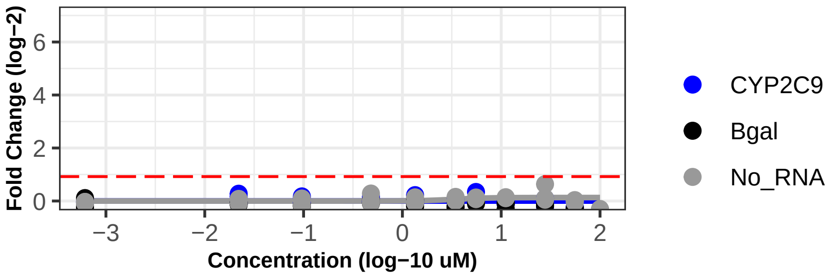


Trifluralin: CYP3A4

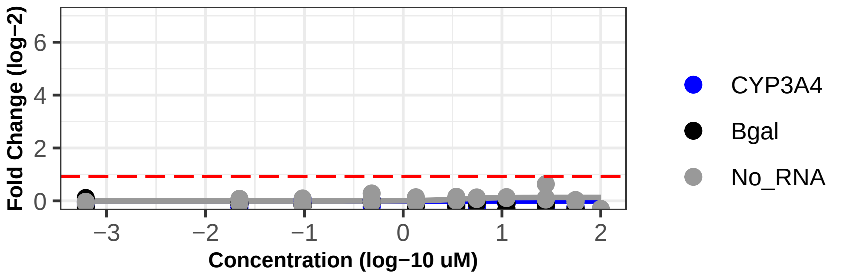

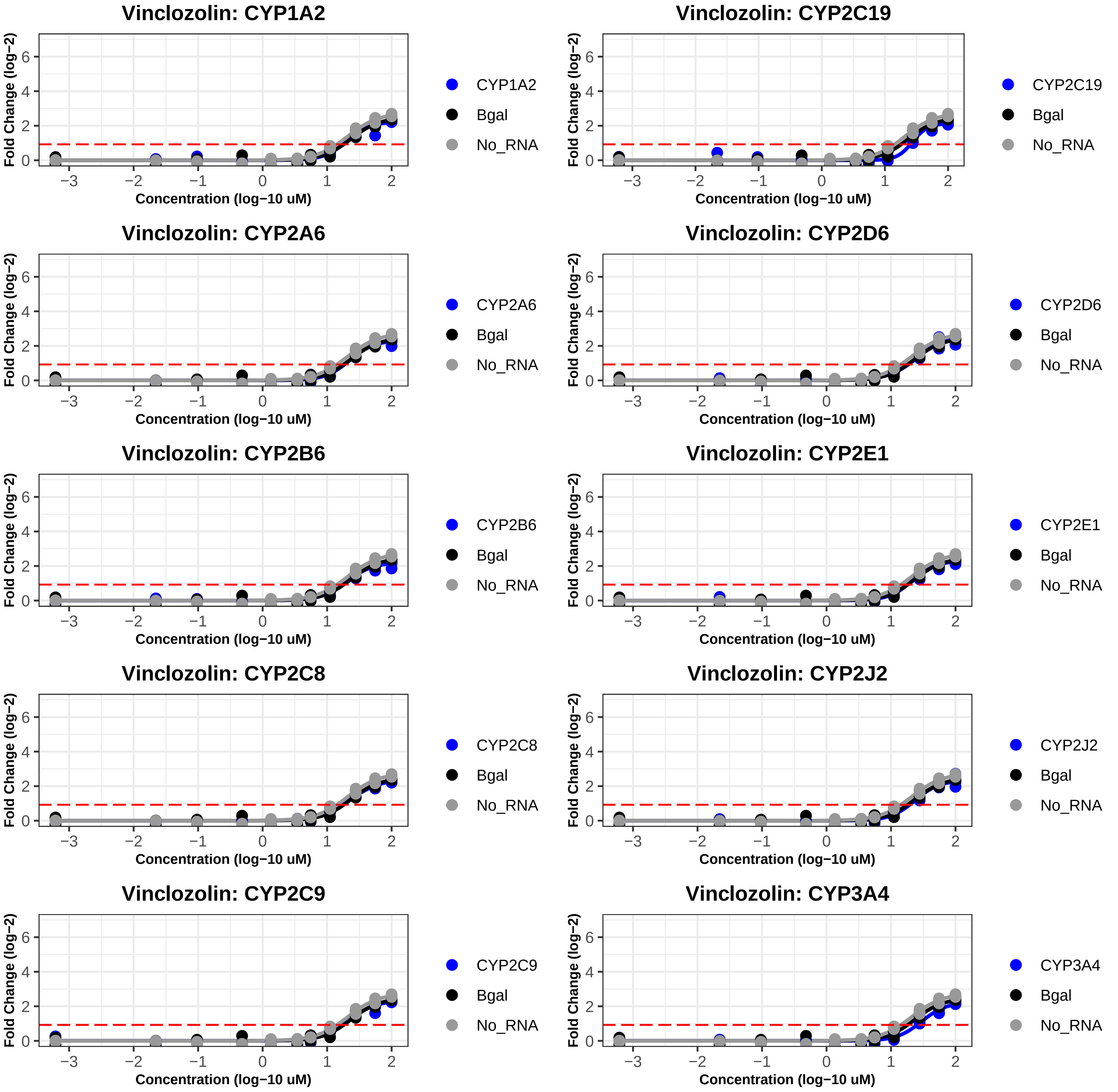
Vinclozolin: CYP1A2
Fold Change (log−2)
Concentration (log−10 uM)
CYP1A2
Bgal
No_RNA
Vinclozolin: CYP2C19
Fold Change (log−2)
Concentration (log−10 uM)
CYP2C19
Bgal
No_RNA
Vinclozolin: CYP2A6
Fold Change (log−2)
Concentration (log−10 uM)
CYP2A6
Bgal
No_RNA
Vinclozolin: CYP2D6
Fold Change (log−2)
Concentration (log−10 uM)
CYP2D6
Bgal
No_RNA
Vinclozolin: CYP2B6
Fold Change (log−2)
Concentration (log−10 uM)
CYP2B6
Bgal
No_RNA
Vinclozolin: CYP2E1
Fold Change (log−2)
Concentration (log−10 uM)
CYP2E1
Bgal
No_RNA
Vinclozolin: CYP2C8
Fold Change (log−2)
Concentration (log−10 uM)
CYP2C8
Bgal
No_RNA
Vinclozolin: CYP2J2
Fold Change (log−2)
Concentration (log−10 uM)
CYP2J2
Bgal
No_RNA
Vinclozolin: CYP2C9
Fold Change (log−2)
Concentration (log−10 uM)
CYP2C9
Bgal
No_RNA
Vinclozolin: CYP3A4
Fold Change (log−2)
Concentration (log−10 uM)
CYP3A4
Bgal
No_RNA

Z−Tetrachlorvinphos: CYP1A2

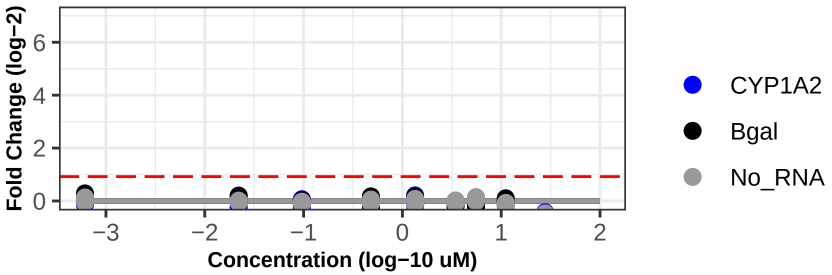


Z−Tetrachlorvinphos: CYP2C19

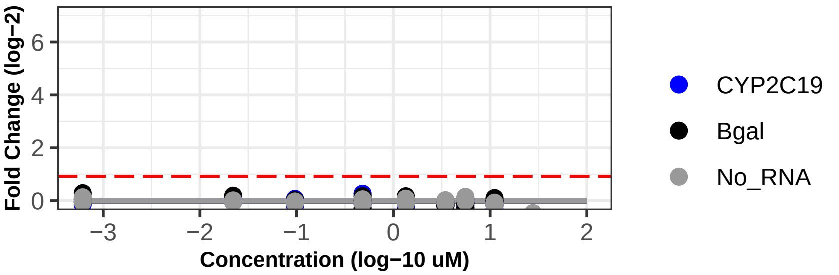


Z−Tetrachlorvinphos: CYP2A6

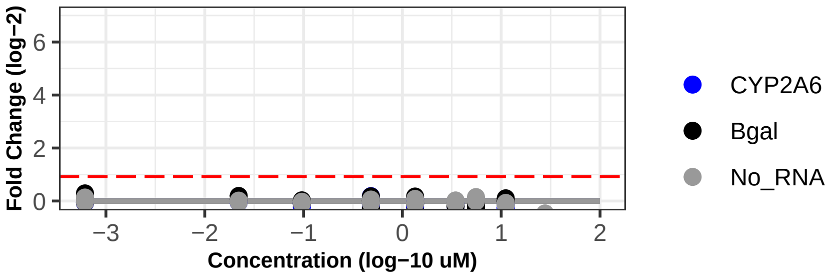


Z−Tetrachlorvinphos: CYP2D6

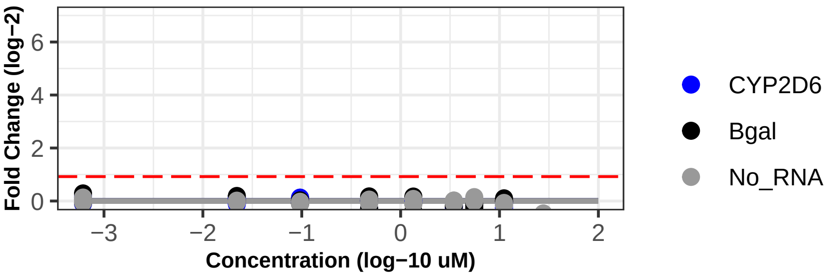


Z−Tetrachlorvinphos: CYP2B6

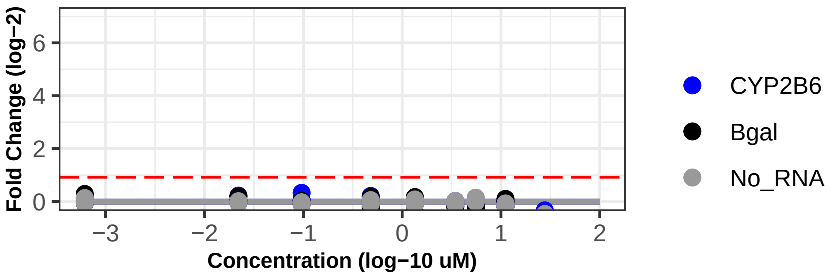


Z−Tetrachlorvinphos: CYP2E1

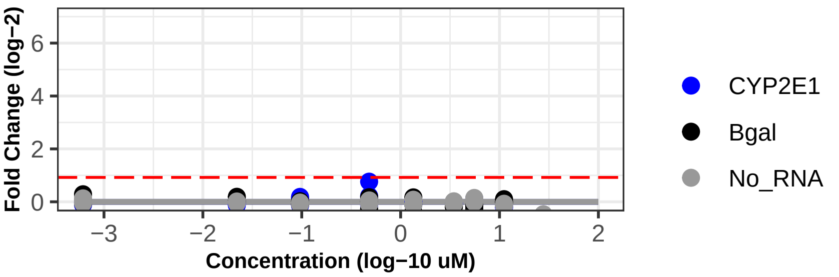


Z−Tetrachlorvinphos: CYP2C8

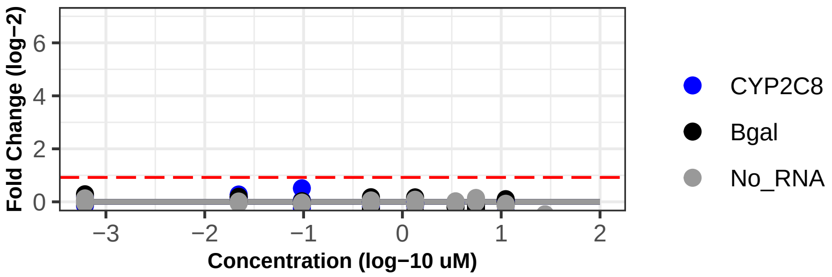


Z−Tetrachlorvinphos: CYP2J2

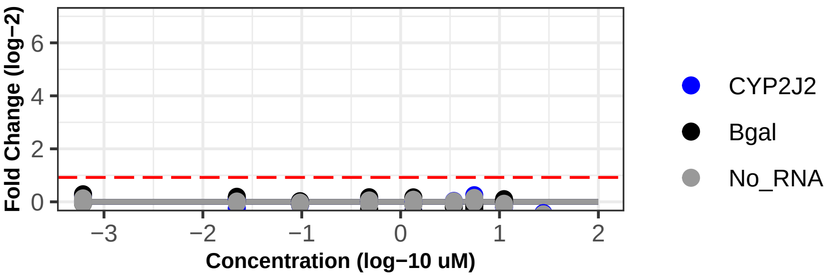


Z−Tetrachlorvinphos: CYP2C9

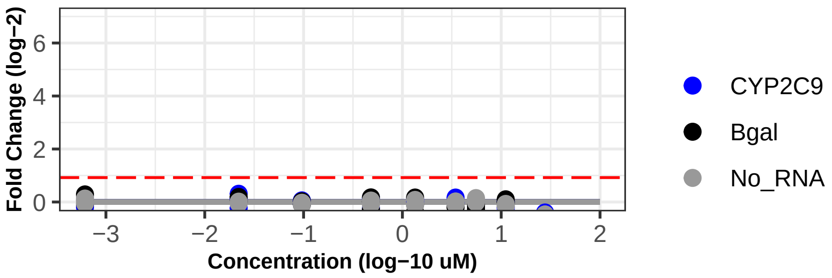


Z−Tetrachlorvinphos: CYP3A4

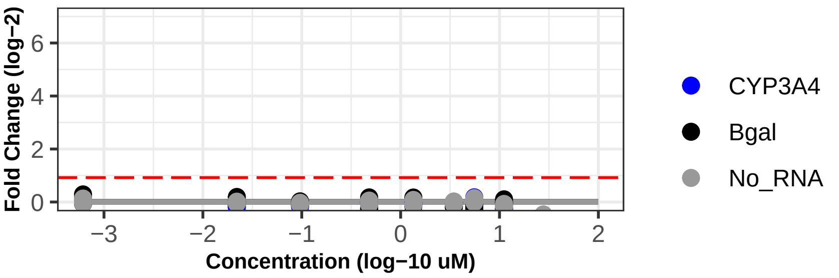

## Zearalenone: CYP1A2

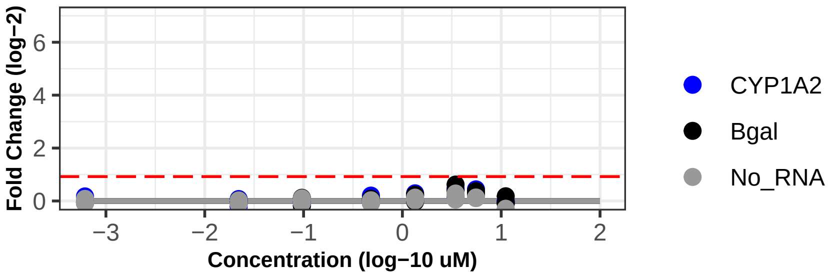


## Zearalenone: CYP2C19

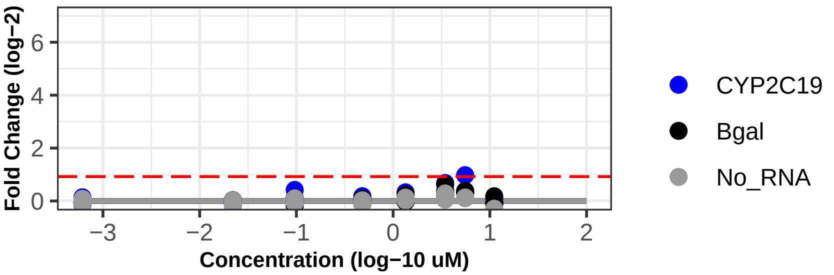


## Zearalenone: CYP2A6

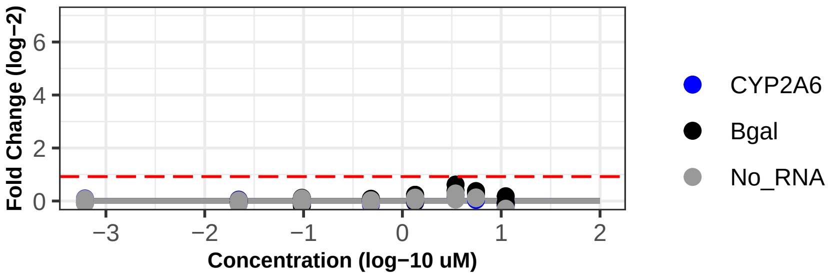


## Zearalenone: CYP2D6

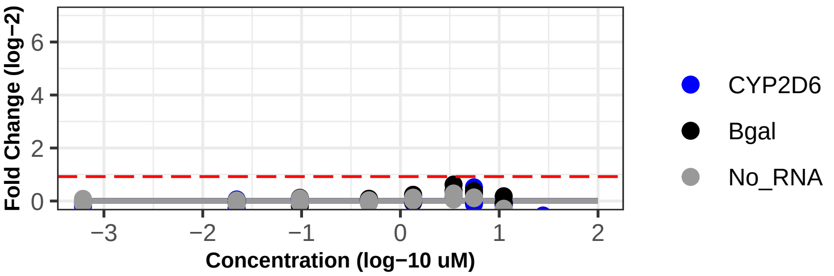


## Zearalenone: CYP2B6

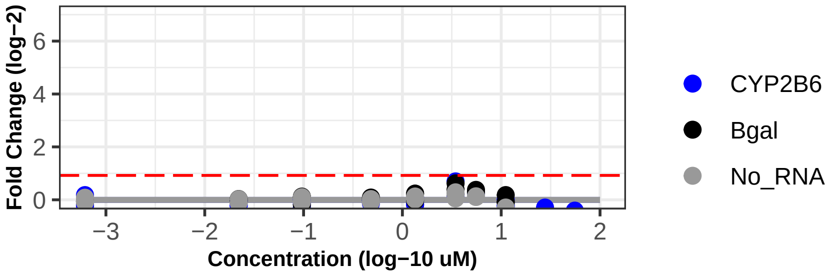


## Zearalenone: CYP2E1

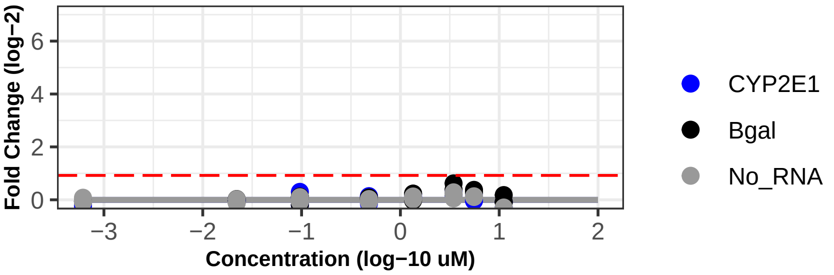


## Zearalenone: CYP2C8

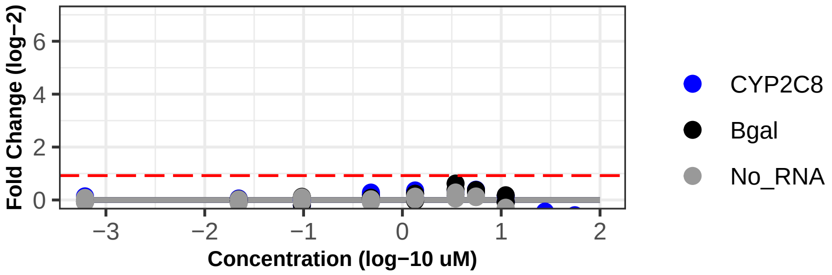


## Zearalenone: CYP2J2

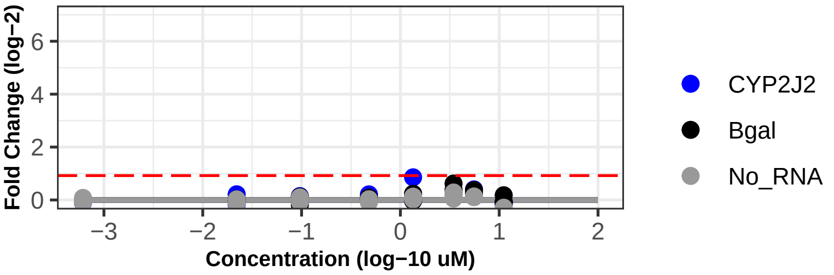


## Zearalenone: CYP2C9

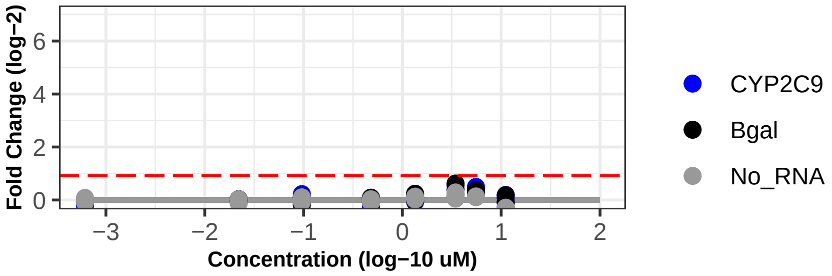


## Zearalenone: CYP3A4

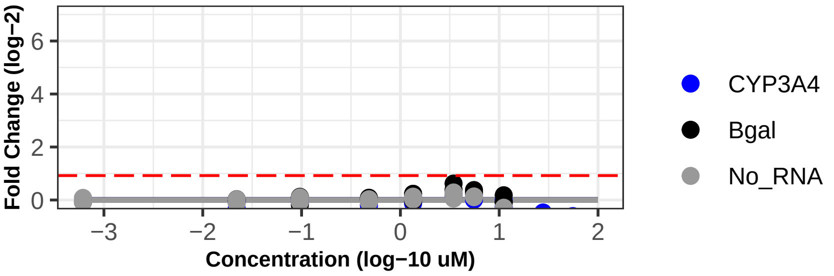

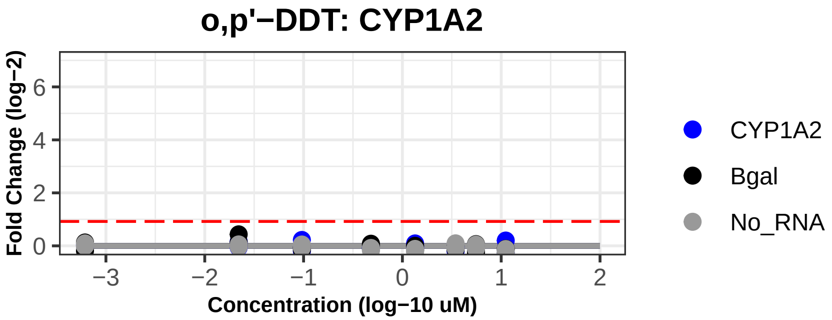
o,p'−DDT: CYP1A2
Fold Change (log−2)
Concentration (log−10 uM)
CYP1A2
Bgal
No_RNA

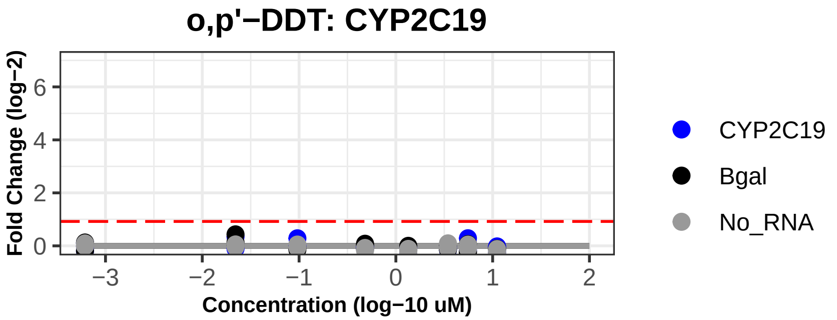
o,p'−DDT: CYP2C19
Fold Change (log−2)
Concentration (log−10 uM)
CYP2C19
Bgal
No_RNA

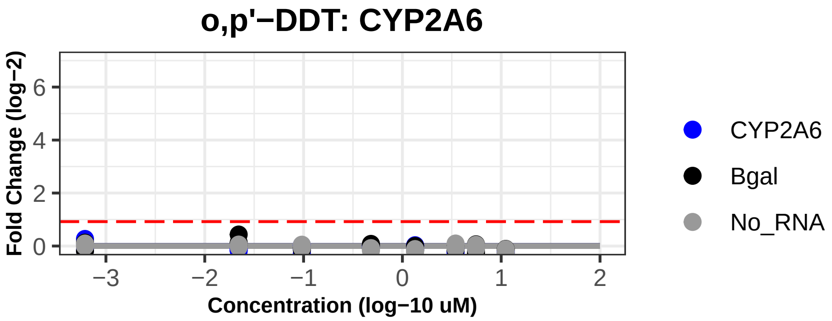
o,p'−DDT: CYP2A6
Fold Change (log−2)
Concentration (log−10 uM)
CYP2A6
Bgal
No_RNA

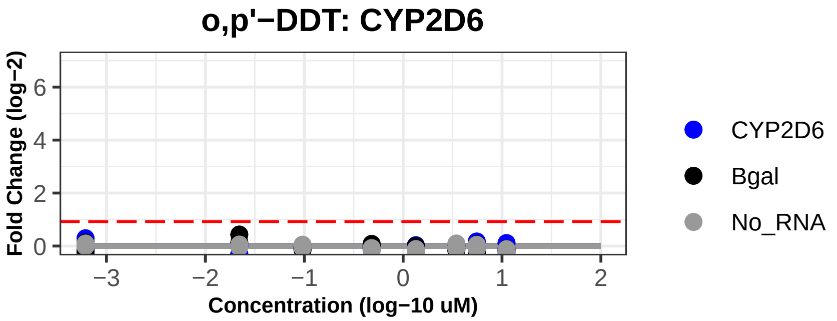
o,p'−DDT: CYP2D6
Fold Change (log−2)
Concentration (log−10 uM)
CYP2D6
Bgal
No_RNA

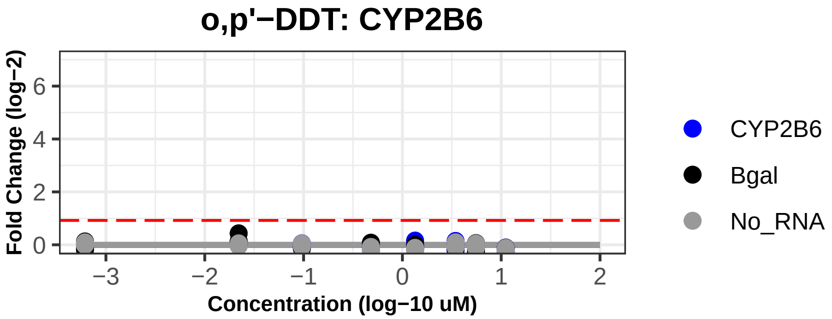
o,p'−DDT: CYP2B6
Fold Change (log−2)
Concentration (log−10 uM)
CYP2B6
Bgal
No_RNA

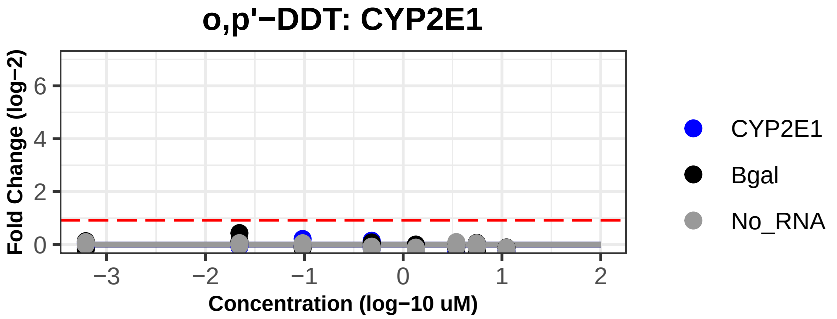
o,p'−DDT: CYP2E1
Fold Change (log−2)
Concentration (log−10 uM)
CYP2E1
Bgal
No_RNA

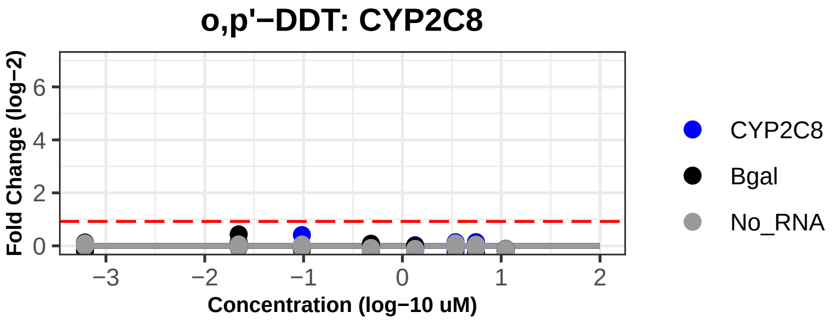
o,p'−DDT: CYP2C8
Fold Change (log−2)
Concentration (log−10 uM)
CYP2C8
Bgal
No_RNA

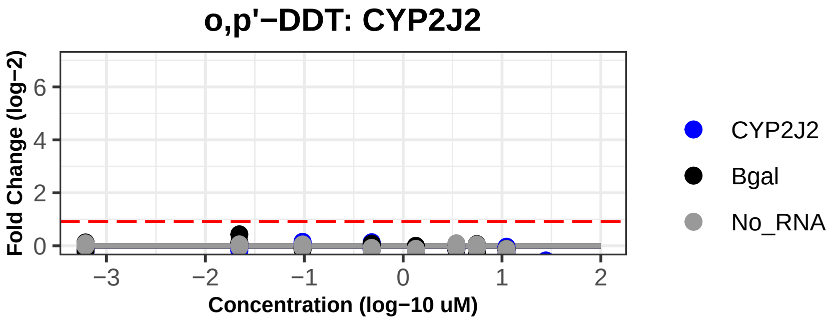
o,p'−DDT: CYP2J2
Fold Change (log−2)
Concentration (log−10 uM)
CYP2J2
Bgal
No_RNA

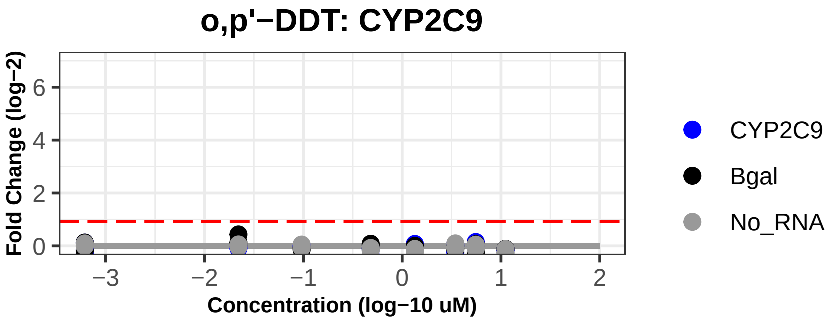
o,p'−DDT: CYP2C9
Fold Change (log−2)
Concentration (log−10 uM)
CYP2C9
Bgal
No_RNA

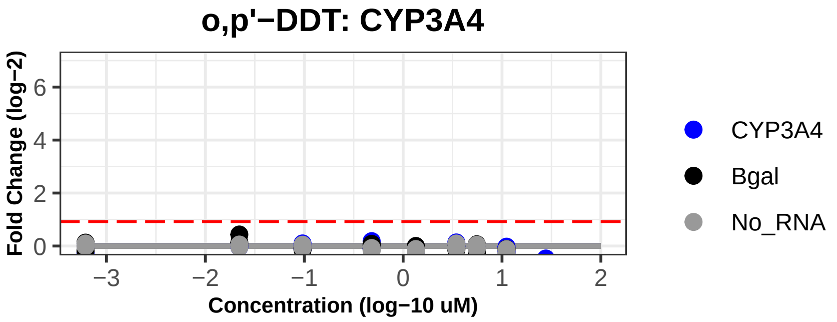
o,p'−DDT: CYP3A4
Fold Change (log−2)
Concentration (log−10 uM)
CYP3A4
Bgal
No_RNA

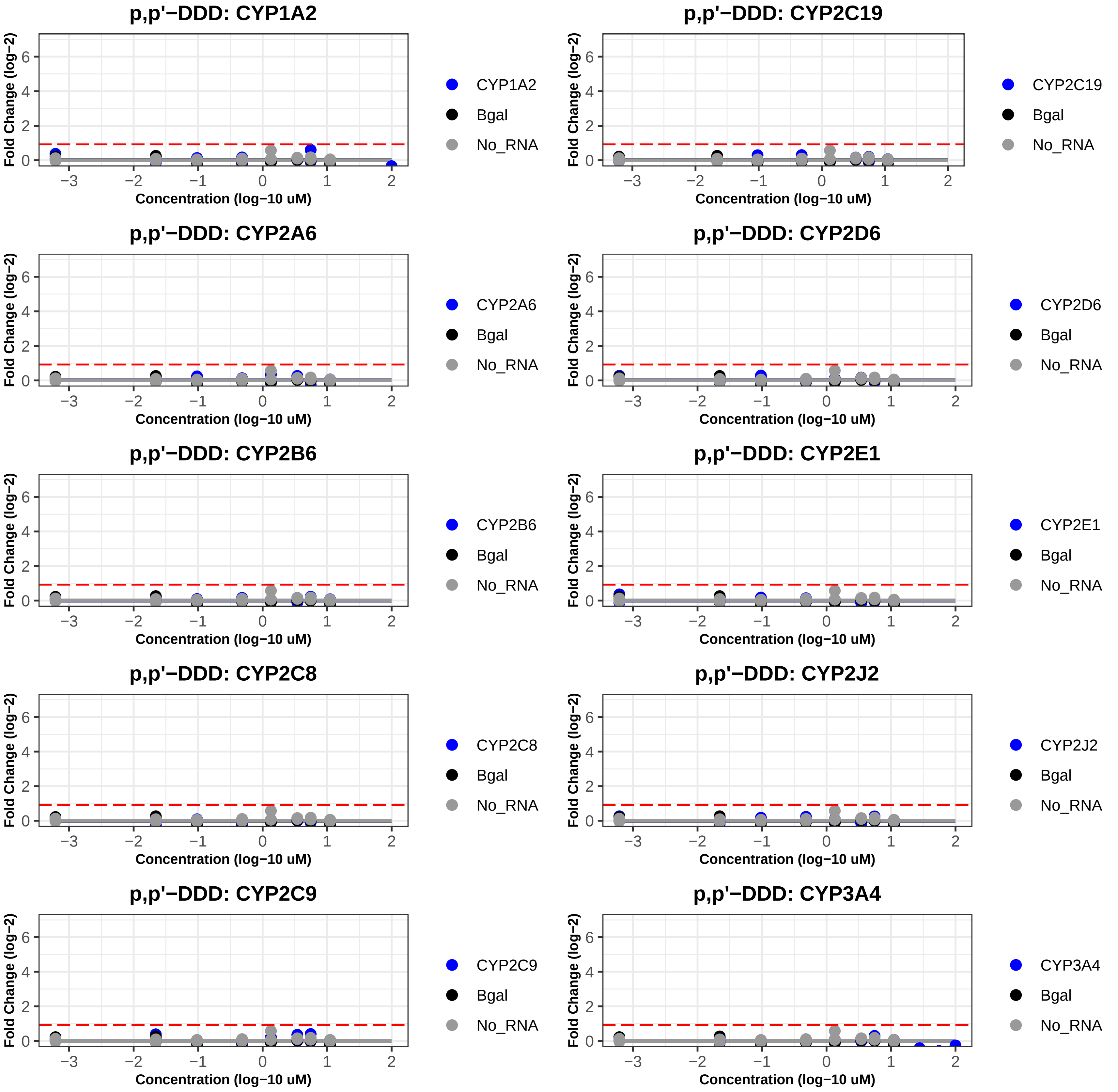
p,p'−DDD: CYP1A2
p,p'−DDD: CYP2C19
p,p'−DDD: CYP2A6
p,p'−DDD: CYP2D6
p,p'−DDD: CYP2B6
p,p'−DDD: CYP2E1
p,p'−DDD: CYP2C8
p,p'−DDD: CYP2J2
p,p'−DDD: CYP2C9
p,p'−DDD: CYP3A4
Fold Change (log−2)
Concentration (log−10 uM)
CYP1A2
CYP2C19
CYP2A6
CYP2D6
CYP2B6
CYP2E1
CYP2C8
CYP2J2
CYP2C9
CYP3A4
Bgal
No_RNA

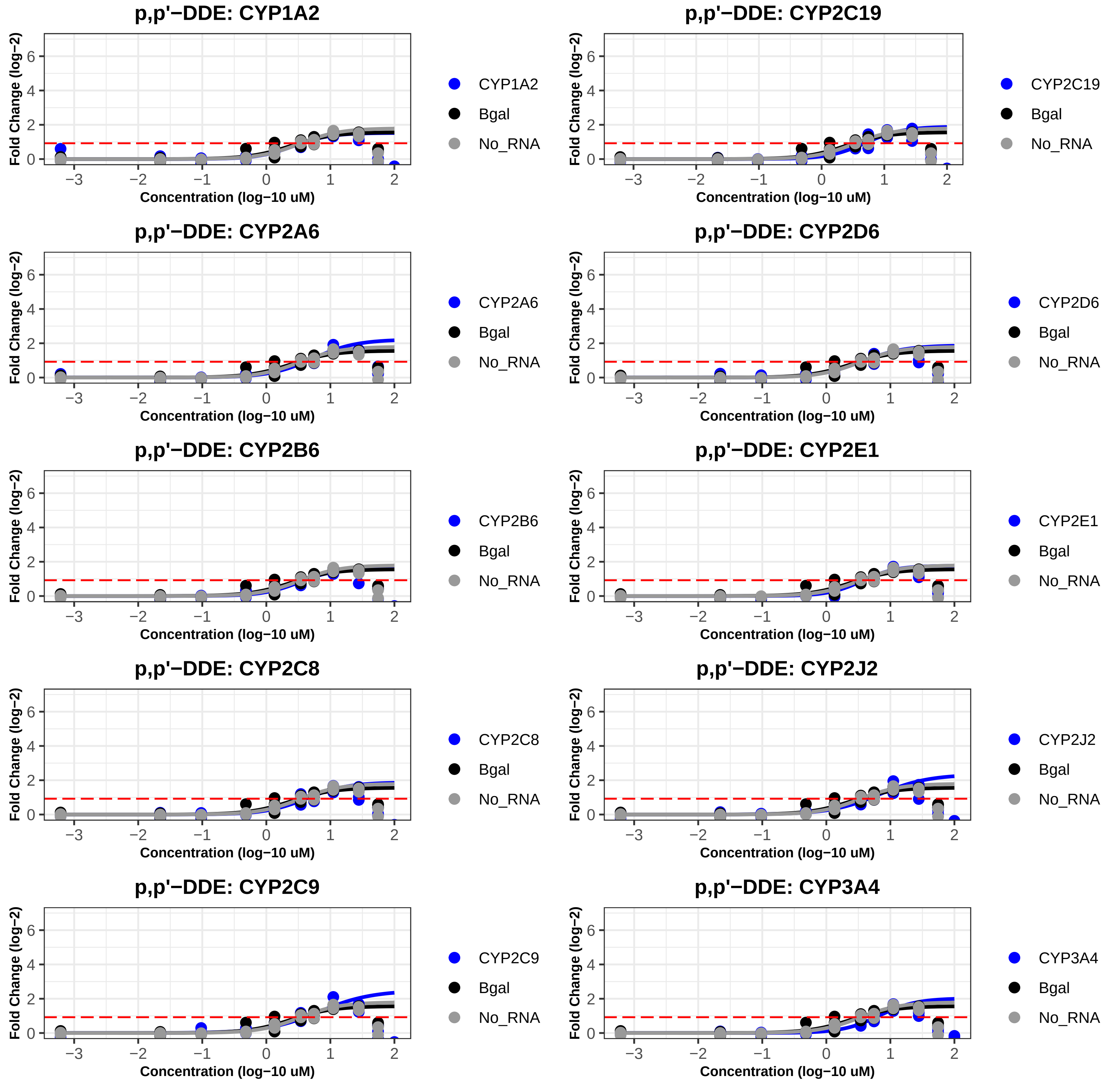
p,p'−DDE: CYP1A2
CYP1A2
Bgal
No_RNA
p,p'−DDE: CYP2C19
CYP2C19
Bgal
No_RNA
p,p'−DDE: CYP2A6
CYP2A6
Bgal
No_RNA
p,p'−DDE: CYP2D6
CYP2D6
Bgal
No_RNA
p,p'−DDE: CYP2B6
CYP2B6
Bgal
No_RNA
p,p'−DDE: CYP2E1
CYP2E1
Bgal
No_RNA
p,p'−DDE: CYP2C8
CYP2C8
Bgal
No_RNA
p,p'−DDE: CYP2J2
CYP2J2
Bgal
No_RNA
p,p'−DDE: CYP2C9
CYP2C9
Bgal
No_RNA
p,p'−DDE: CYP3A4
CYP3A4
Bgal
No_RNA
Fold Change (log−2)
Concentration (log−10 uM)

## p−Dichlorobenzene: CYP1A2

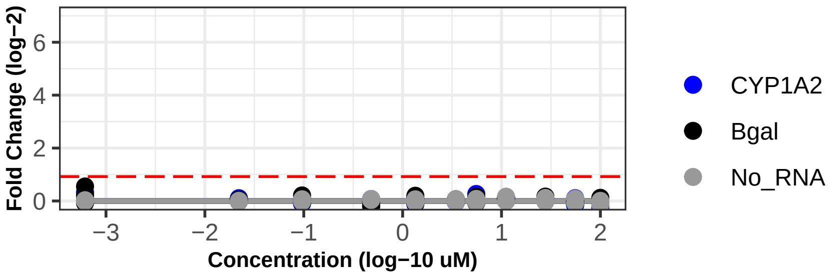


## p−Dichlorobenzene: CYP2C19

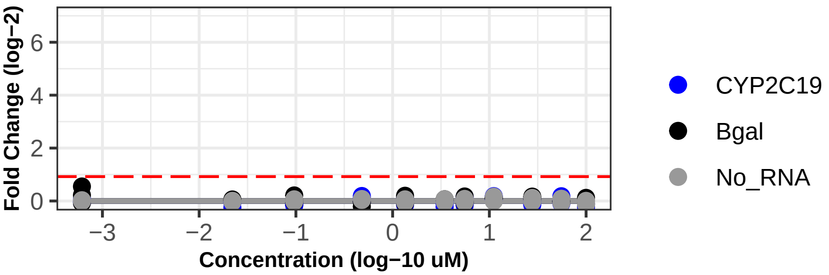


## p−Dichlorobenzene: CYP2A6

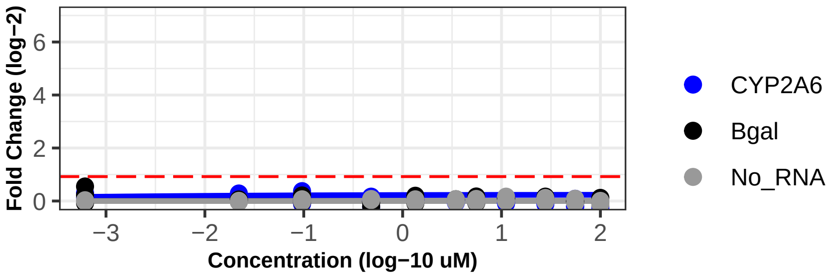


## p−Dichlorobenzene: CYP2D6

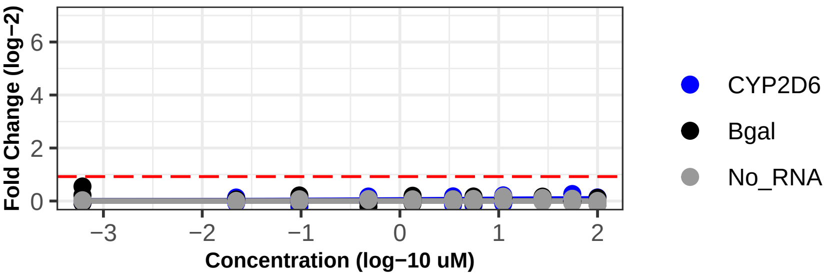


## p−Dichlorobenzene: CYP2B6

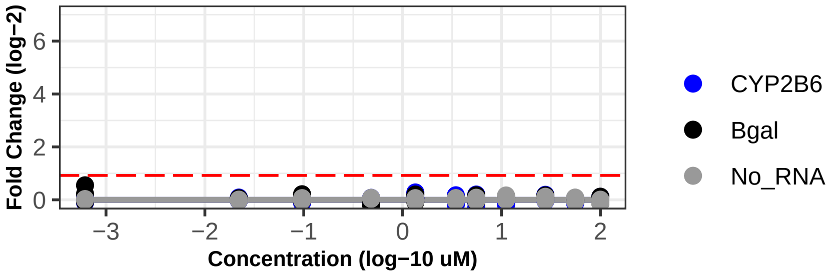


## p−Dichlorobenzene: CYP2E1

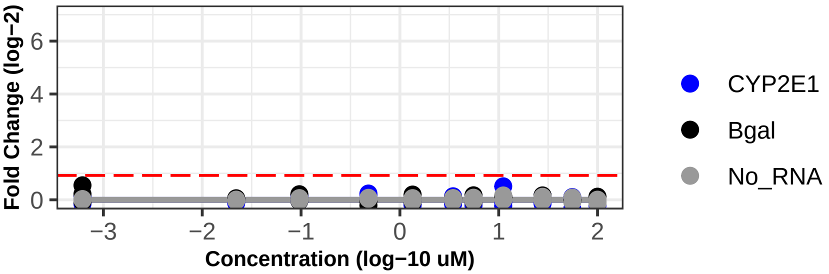


## p−Dichlorobenzene: CYP2C8

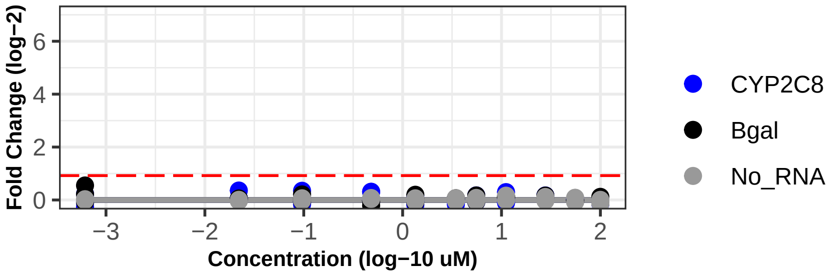


## p−Dichlorobenzene: CYP2J2

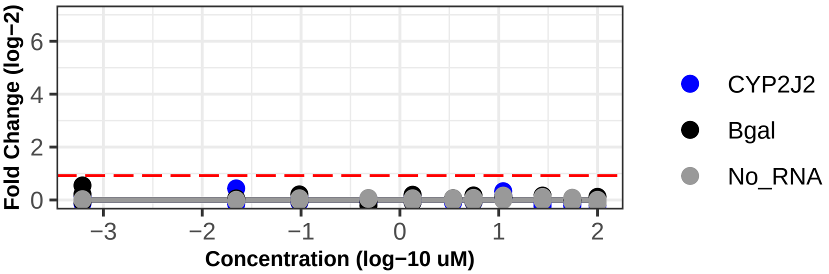


## p−Dichlorobenzene: CYP2C9

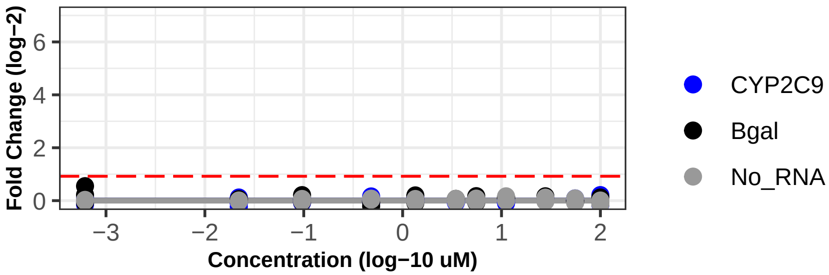


## p−Dichlorobenzene: CYP3A4

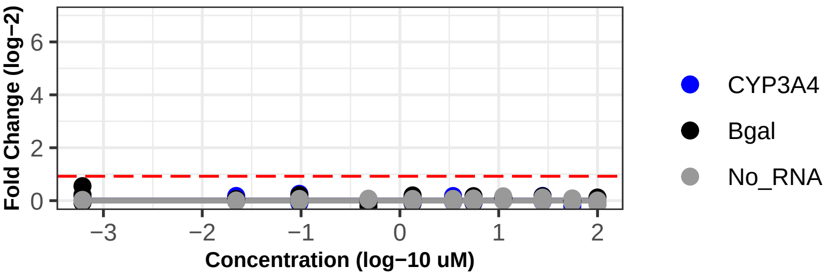

Supplement: Supplementary file 5 [file DataSheet3.PDF]
