# Supplementary material for: High-throughput AR dimerization assay identifies androgen disrupting chemicals and metabolites
Source: Front Toxicol. 2023 Apr 4;5:1134783. doi: 10.3389/ftox.2023.1134783 (PMC10112521; doi:10.3389/ftox.2023.1134783)
Supplement: Supplementary file 6 [file DataSheet1.PDF]

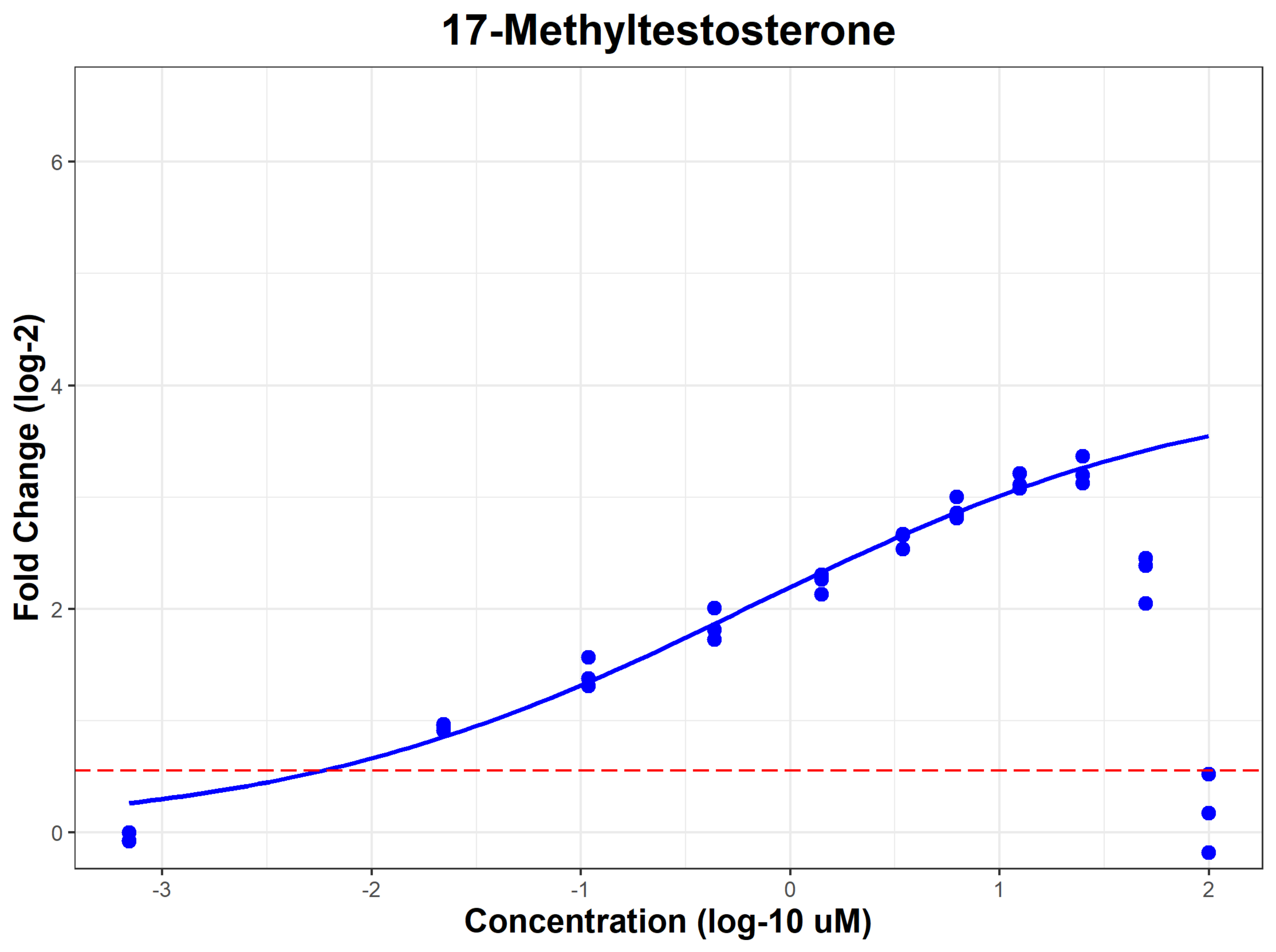

17-Methyltestosterone
Fold Change (log-2)
6
4
2
0
-3
-2
-1
0
1
2
Concentration (log-10 uM)

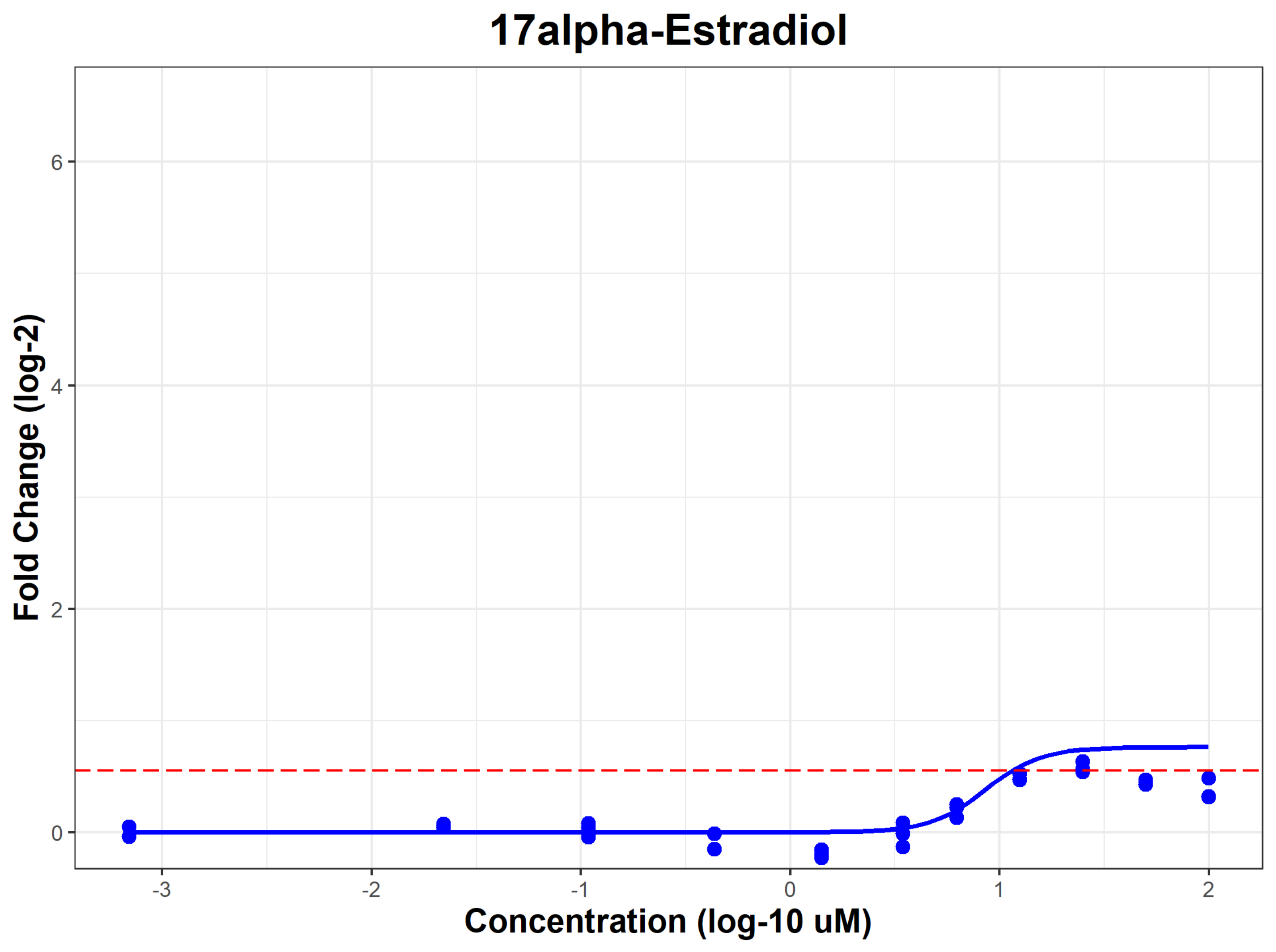
17alpha-Estradiol
Fold Change (log-2)
6
4
2
0
-3
-2
-1
0
1
2
Concentration (log-10 uM)

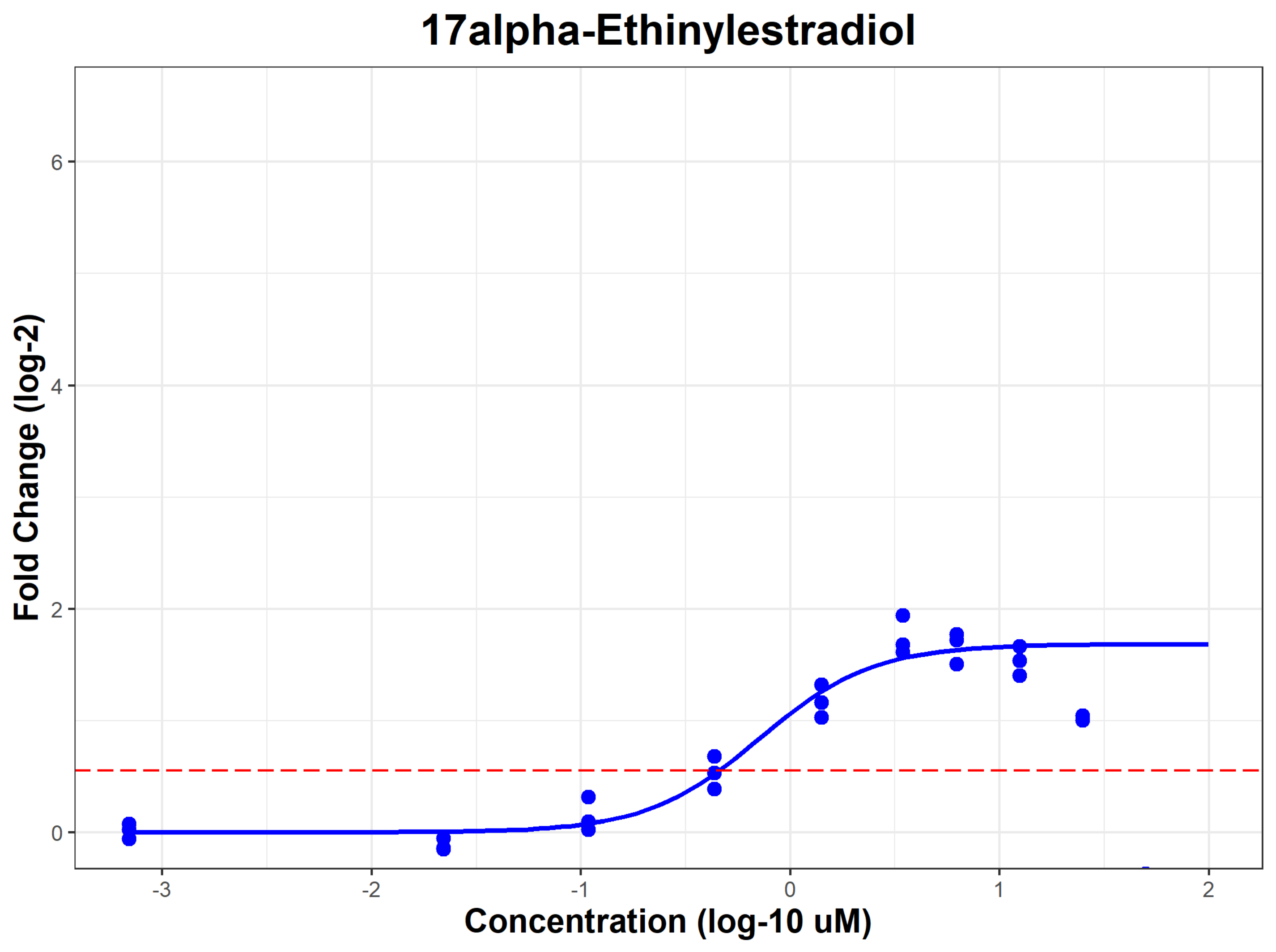

17alpha-Ethinylestradiol
Fold Change (log-2)
0
2
4
6
-3
-2
-1
0
1
2
Concentration (log-10 uM)

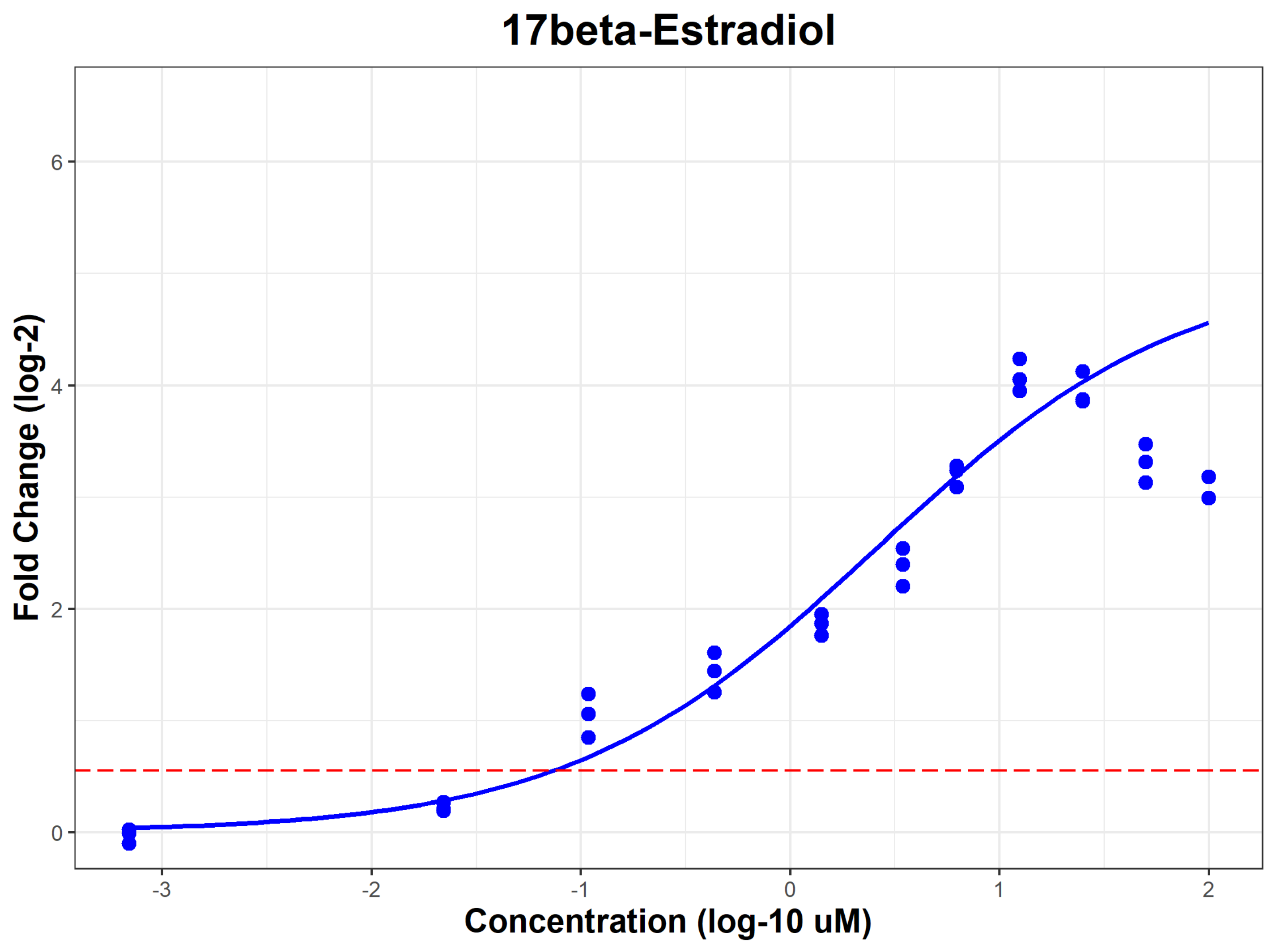
17beta-Estradiol
Fold Change (log-2)
6
4
2
0
-3
-2
-1
0
1
2
Concentration (log-10 uM)

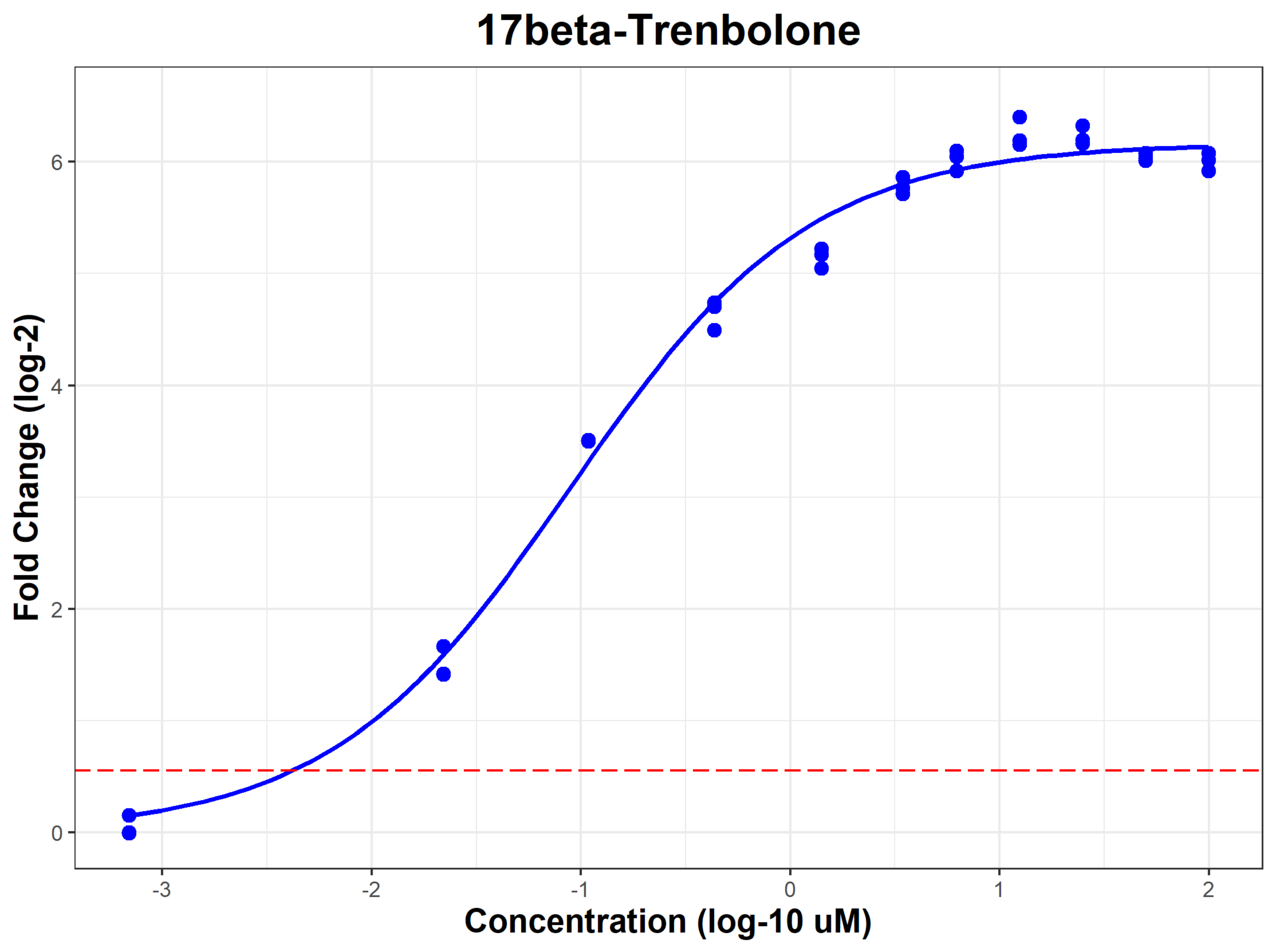
17beta-Trenbolone
Fold Change (log-2)
0
2
4
6
-3
-2
-1
0
1
2
Concentration (log-10 uM)

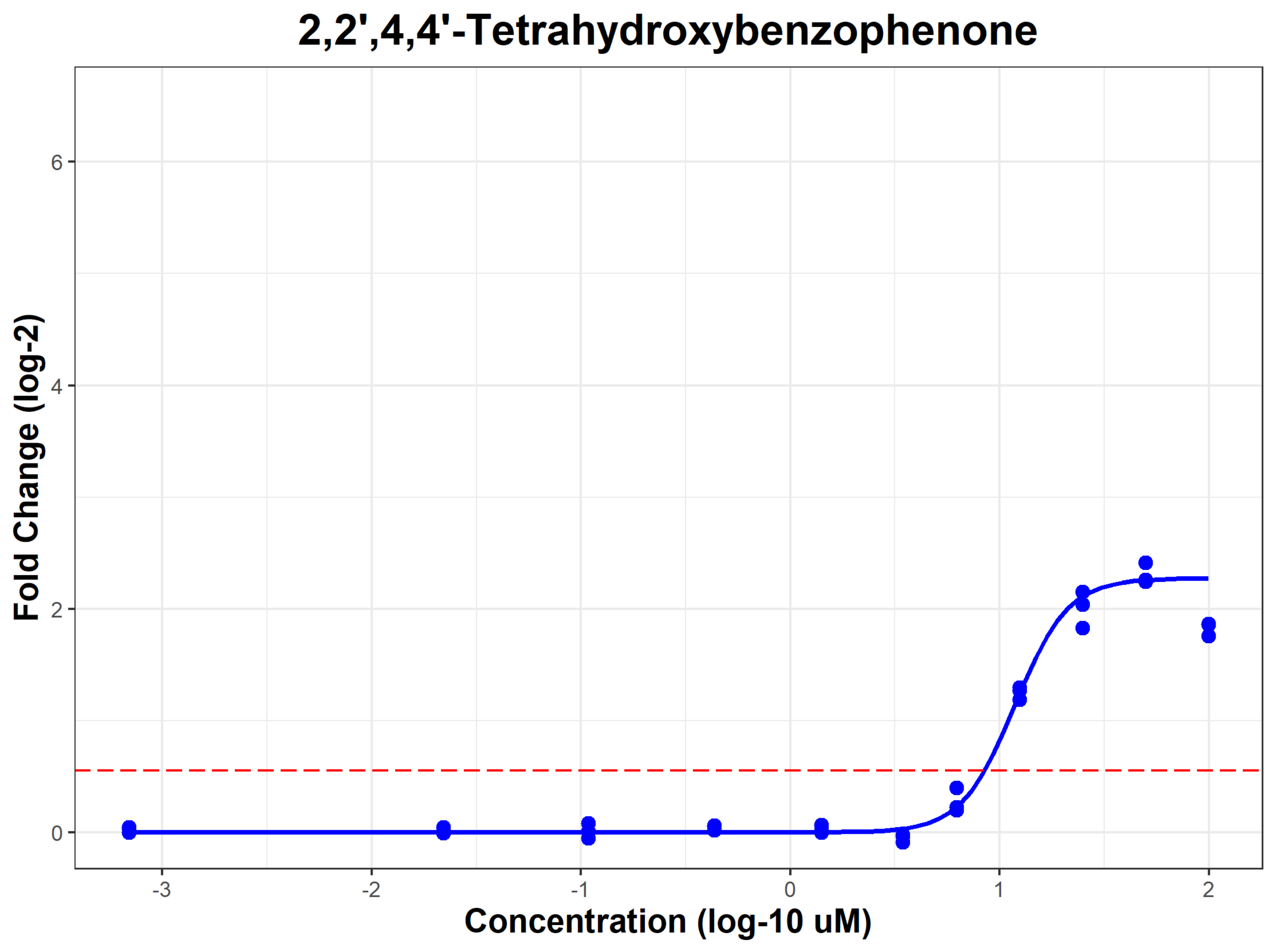
2,2',4,4'-Tetrahydroxybenzophenone
Fold Change (log-2)
Concentration (log-10 uM)
6
4
2
0
-3
-2
-1
0
1
2

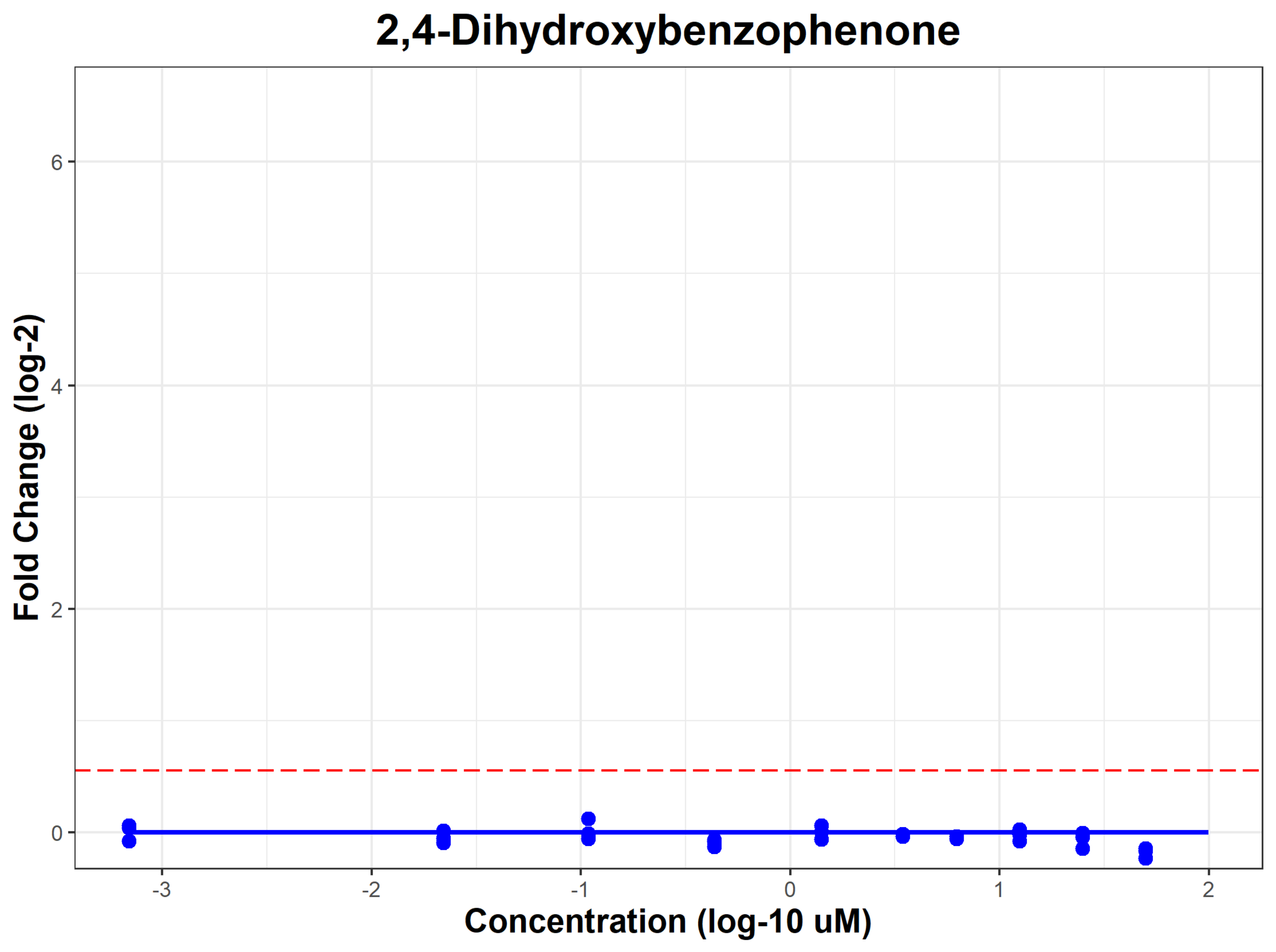
2,4-Dihydroxybenzophenone
Fold Change (log-2)
6
4
2
0
-3
-2
-1
0
1
2
Concentration (log-10 uM)

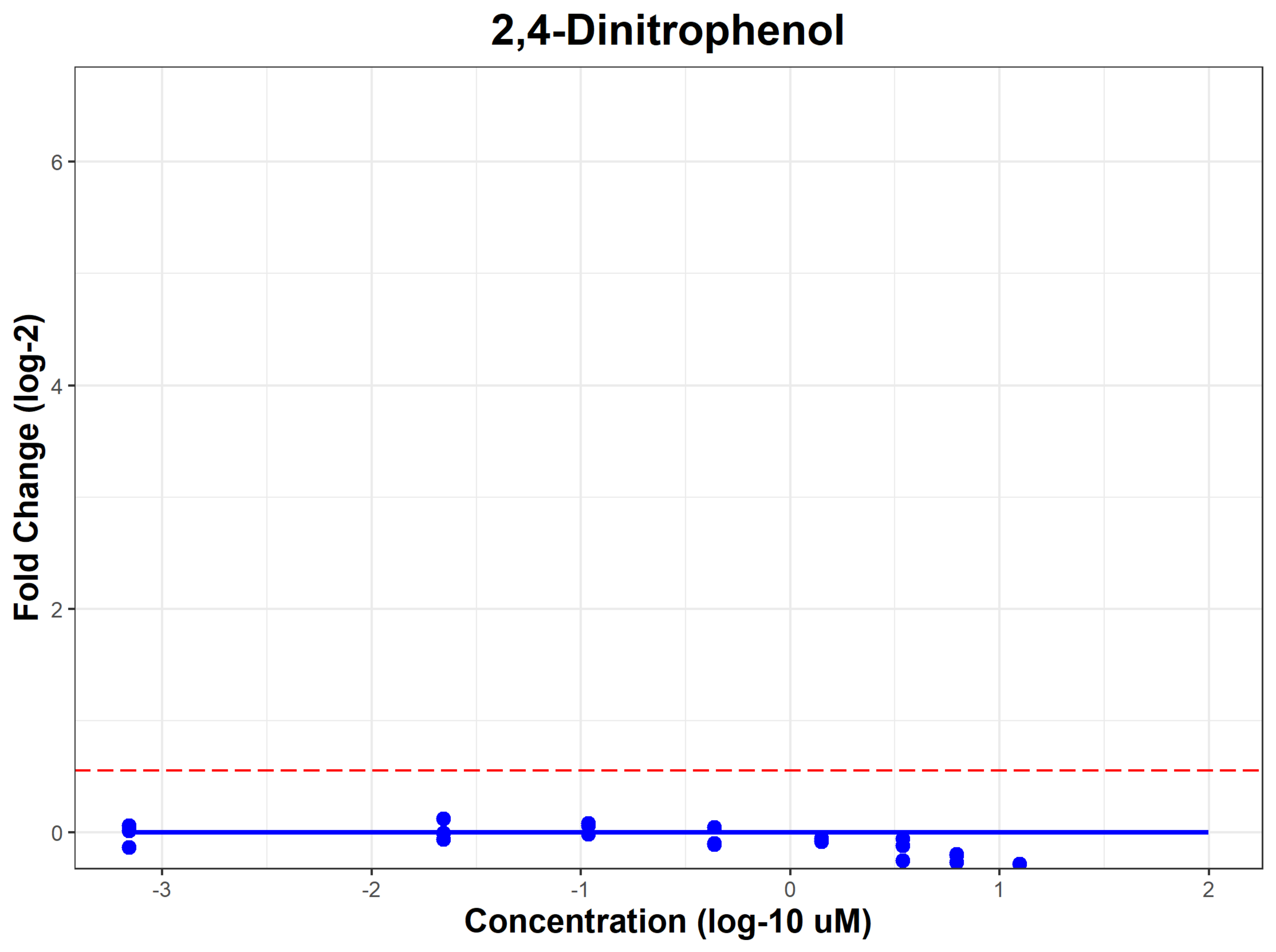
2,4-Dinitrophenol
Fold Change (log-2)
6
4
2
0
-3
-2
-1
0
1
2
Concentration (log-10 uM)

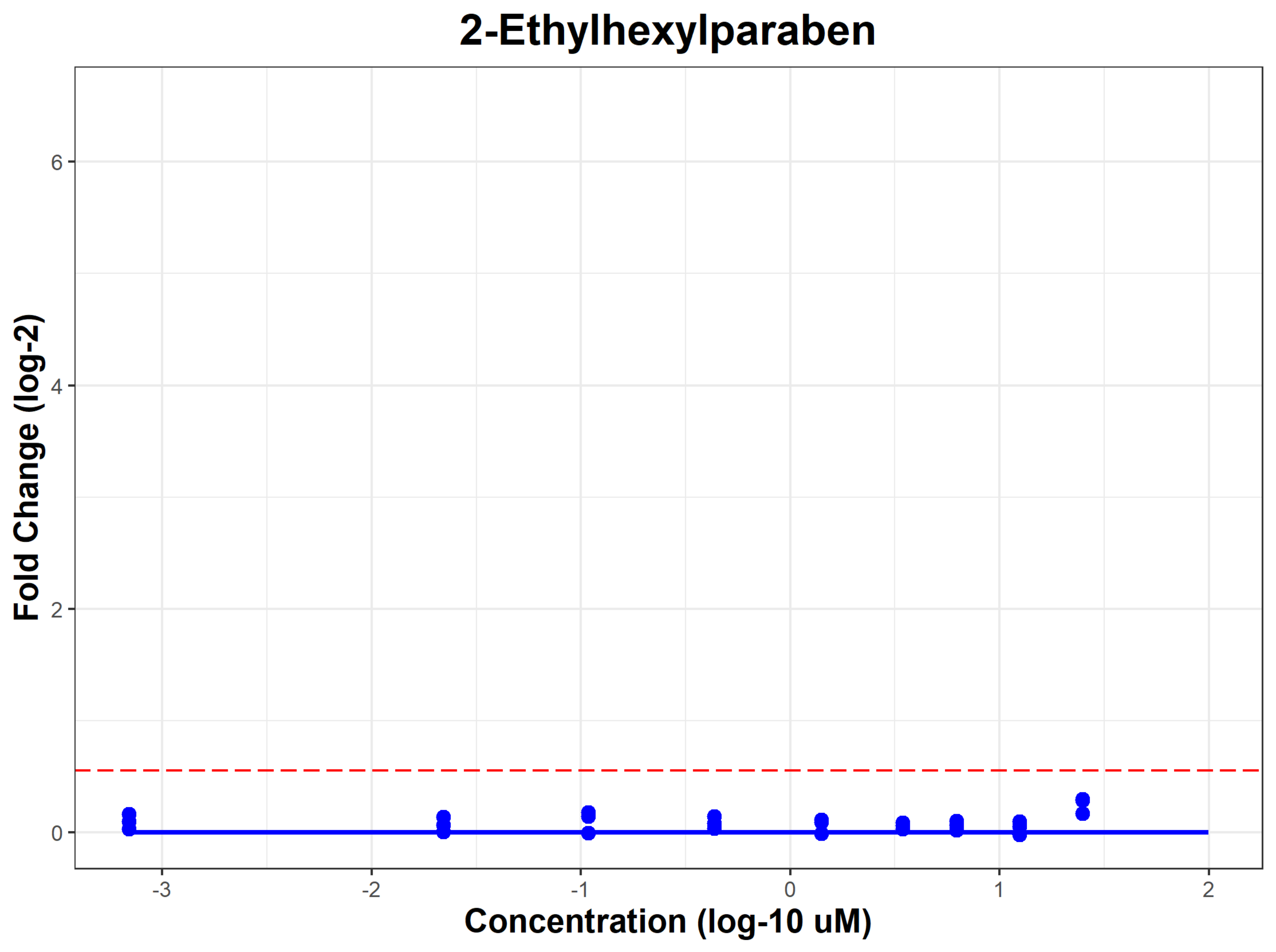
2-Ethylhexylparaben
Fold Change (log-2)
6
4
2
0
-3
-2
-1
0
1
2
Concentration (log-10 uM)

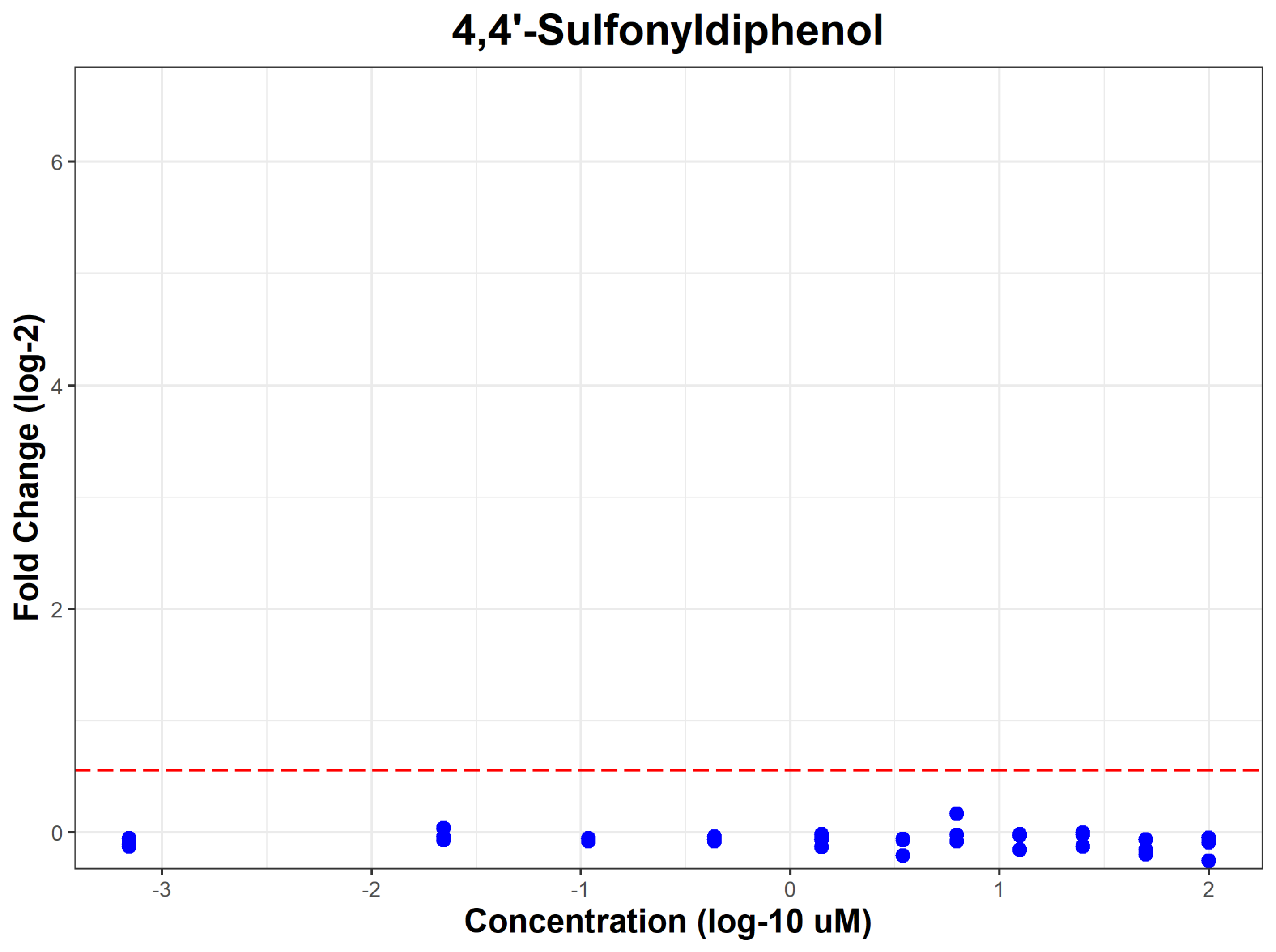
4,4'-Sulfonyldiphenol
Fold Change (log-2)
6
4
2
0
-3
-2
-1
0
1
2
Concentration (log-10 uM)

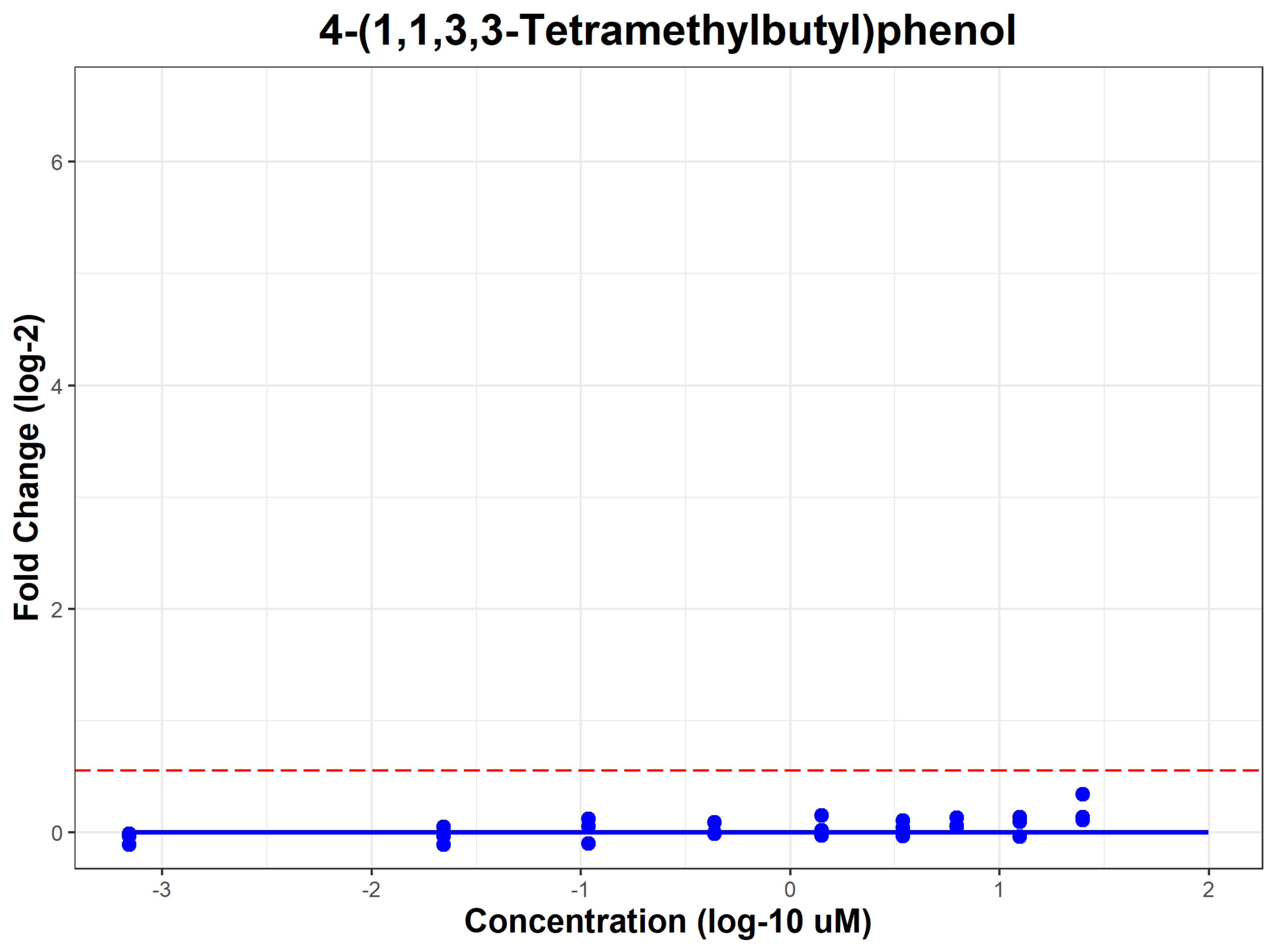
4-(1,1,3,3-Tetramethylbutyl)phenol
Fold Change (log-2)
Concentration (log-10 uM)
6
4
2
0
-3
-2
-1
0
1
2

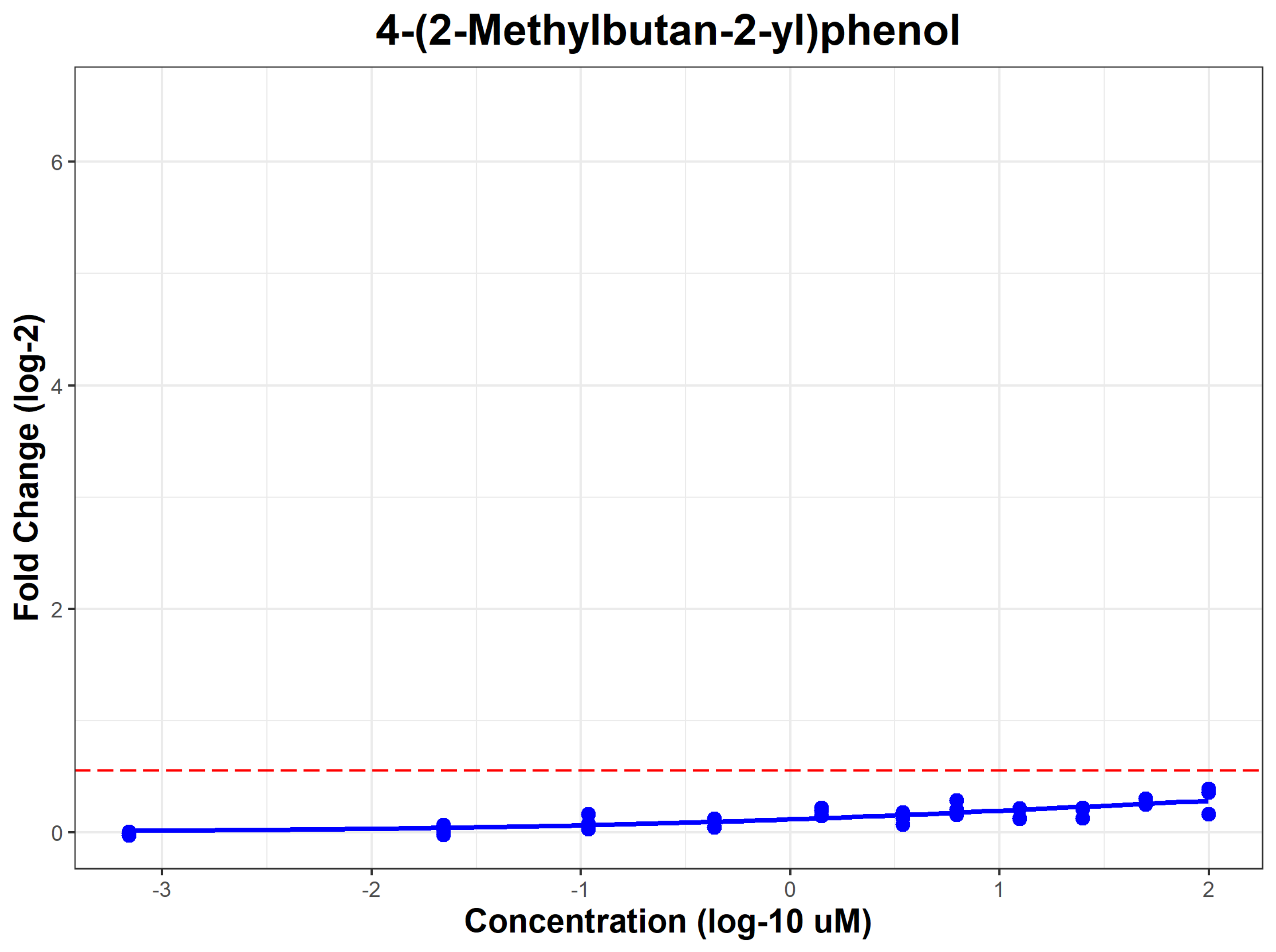
4-(2-Methylbutan-2-yl)phenol
Fold Change (log-2)
6
4
2
0
-3
-2
-1
0
1
2
Concentration (log-10 uM)

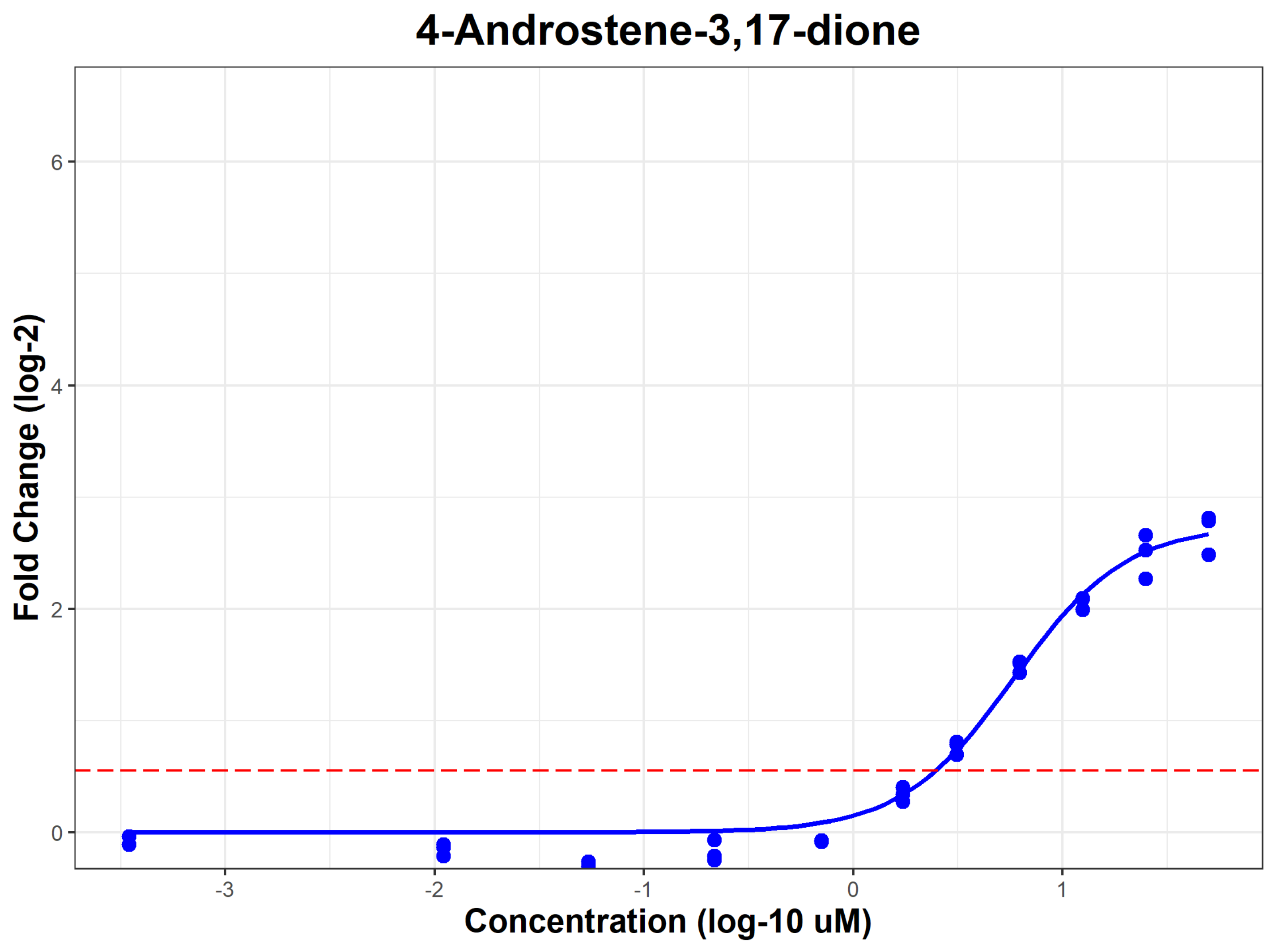
4-Androstene-3,17-dione
Fold Change (log-2)
Concentration (log-10 uM)
6
4
2
0
-3
-2
-1
0
1

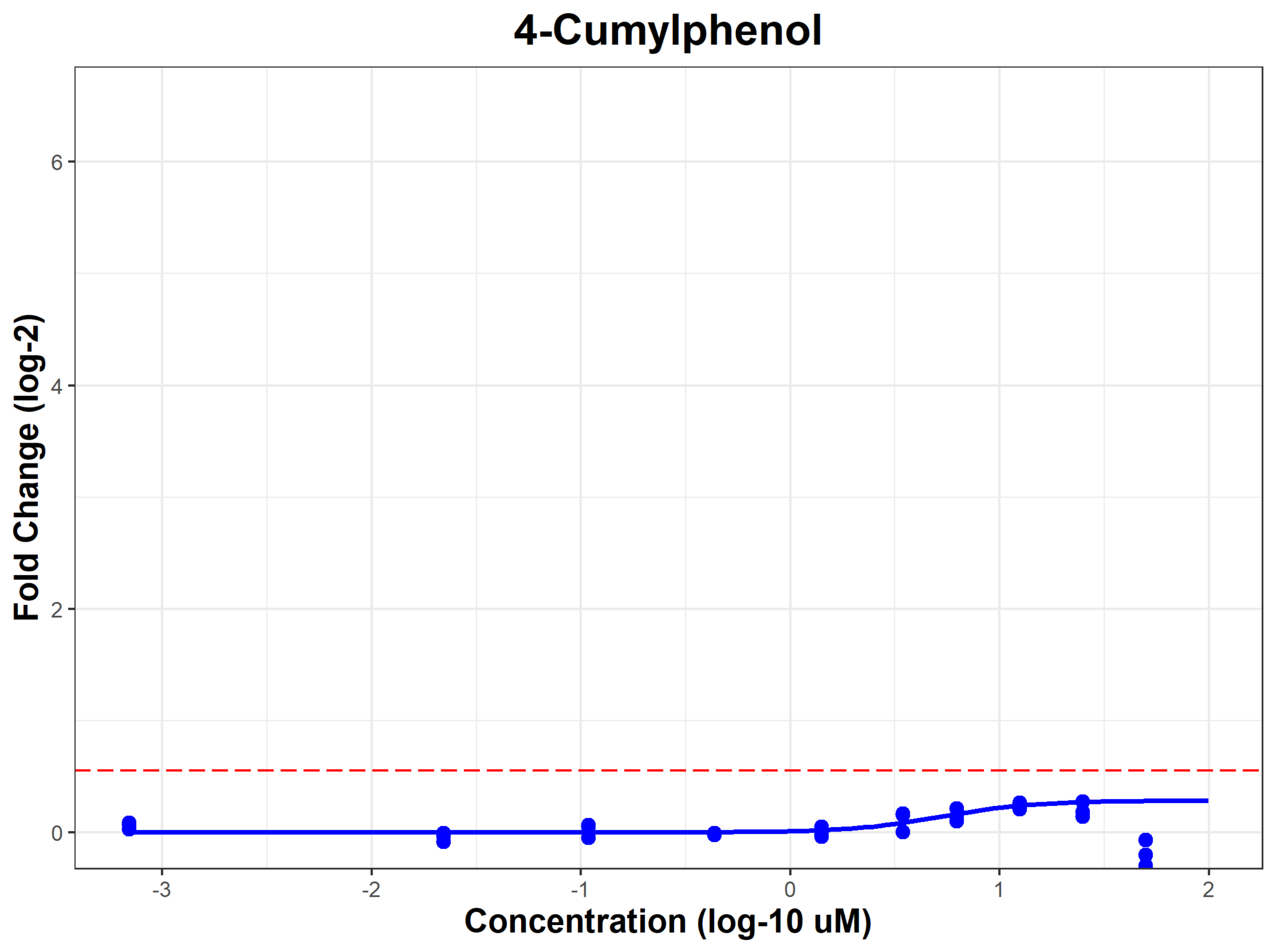
4-Cumylphenol
Fold Change (log-2)
Concentration (log-10 uM)
6
4
2
0
-3
-2
-1
0
1
2

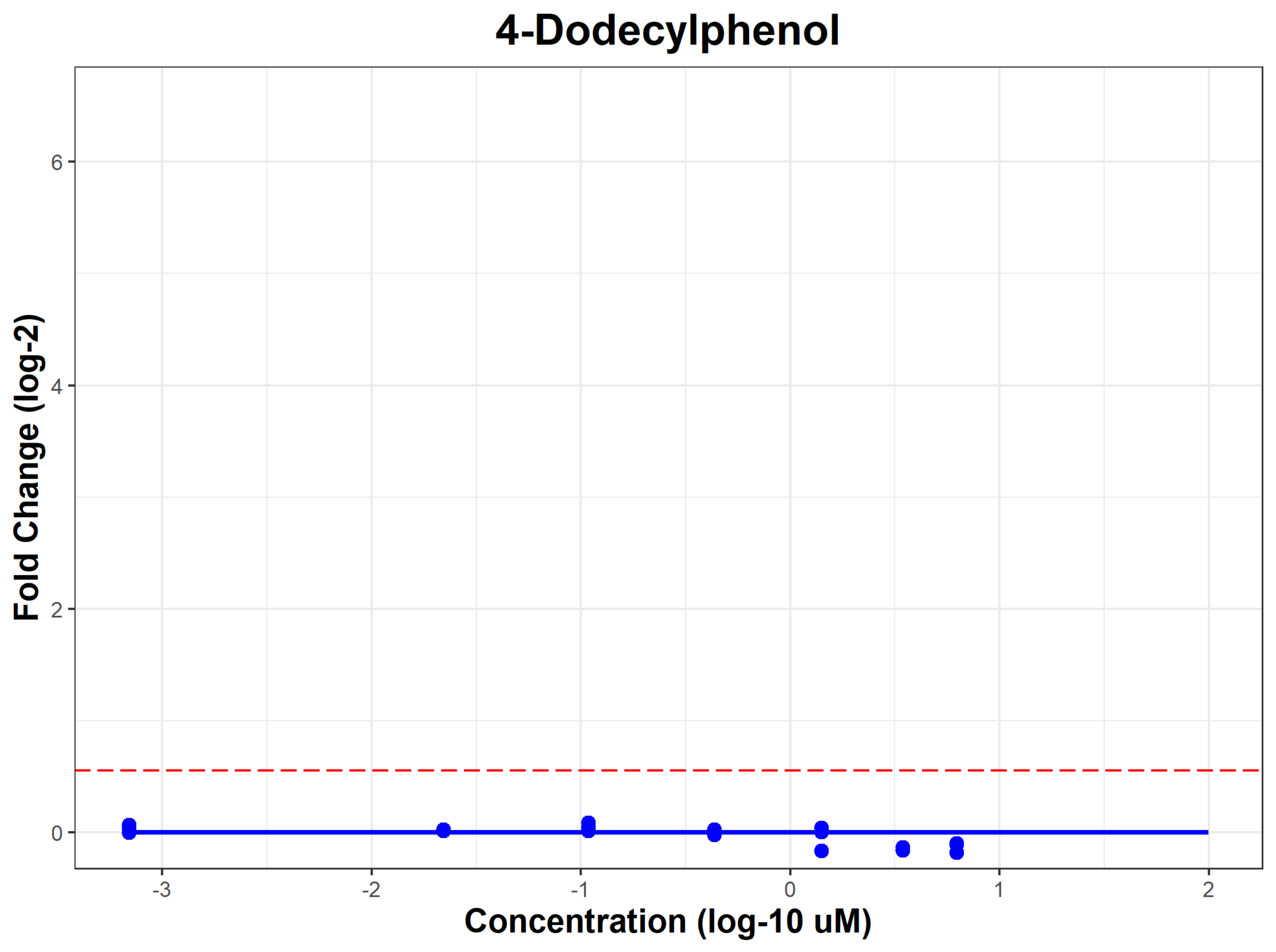

4-Dodecylphenol
Fold Change (log-2)
6
4
2
0
-3
-2
-1
0
1
2
Concentration (log-10 uM)

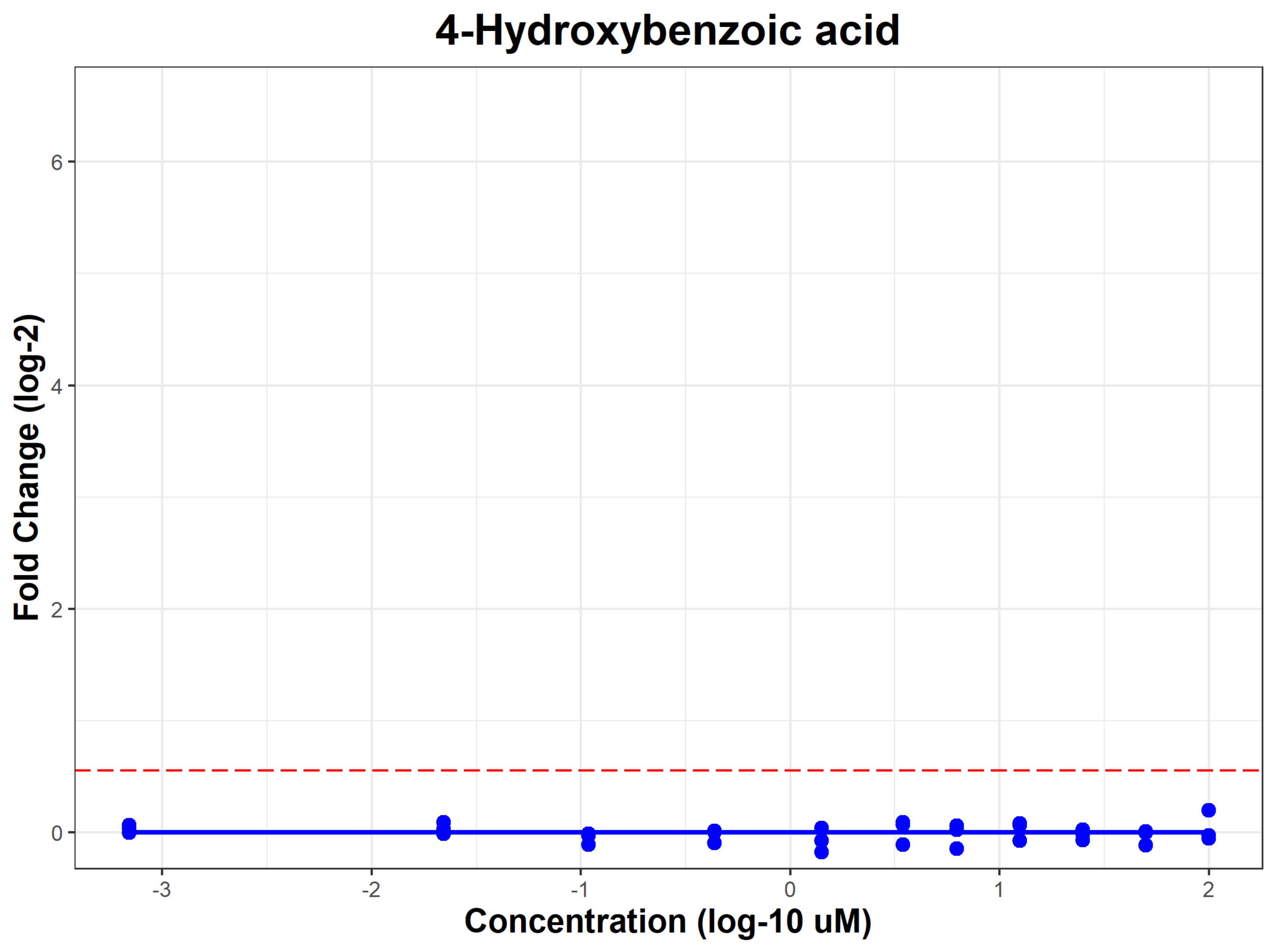

4-Hydroxybenzoic acid
Fold Change (log-2)
6
4
2
0
-3
-2
-1
0
1
2
Concentration (log-10 uM)

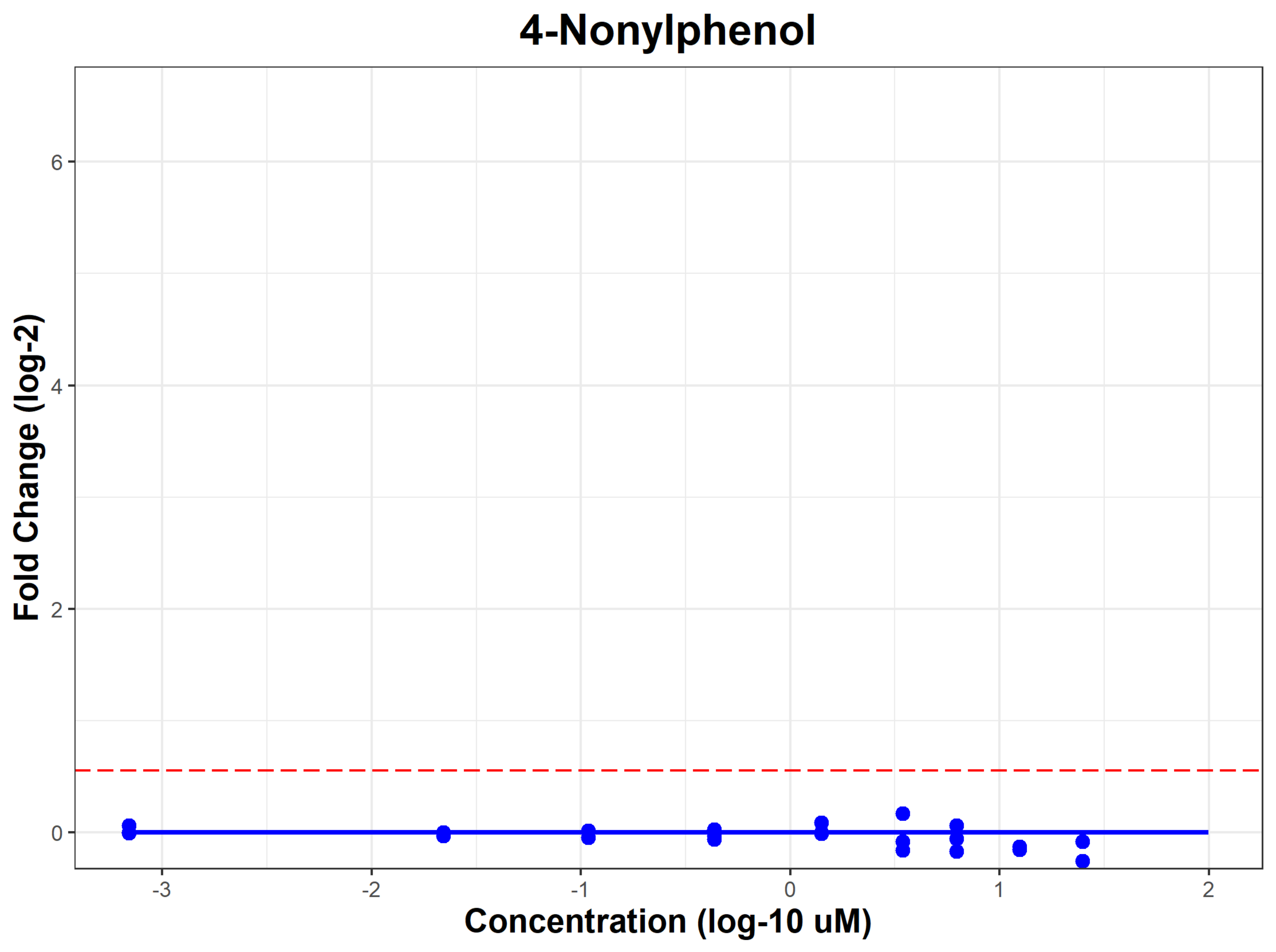
4-Nonylphenol
Fold Change (log-2)
6
4
2
0
-3
-2
-1
0
1
2
Concentration (log-10 uM)

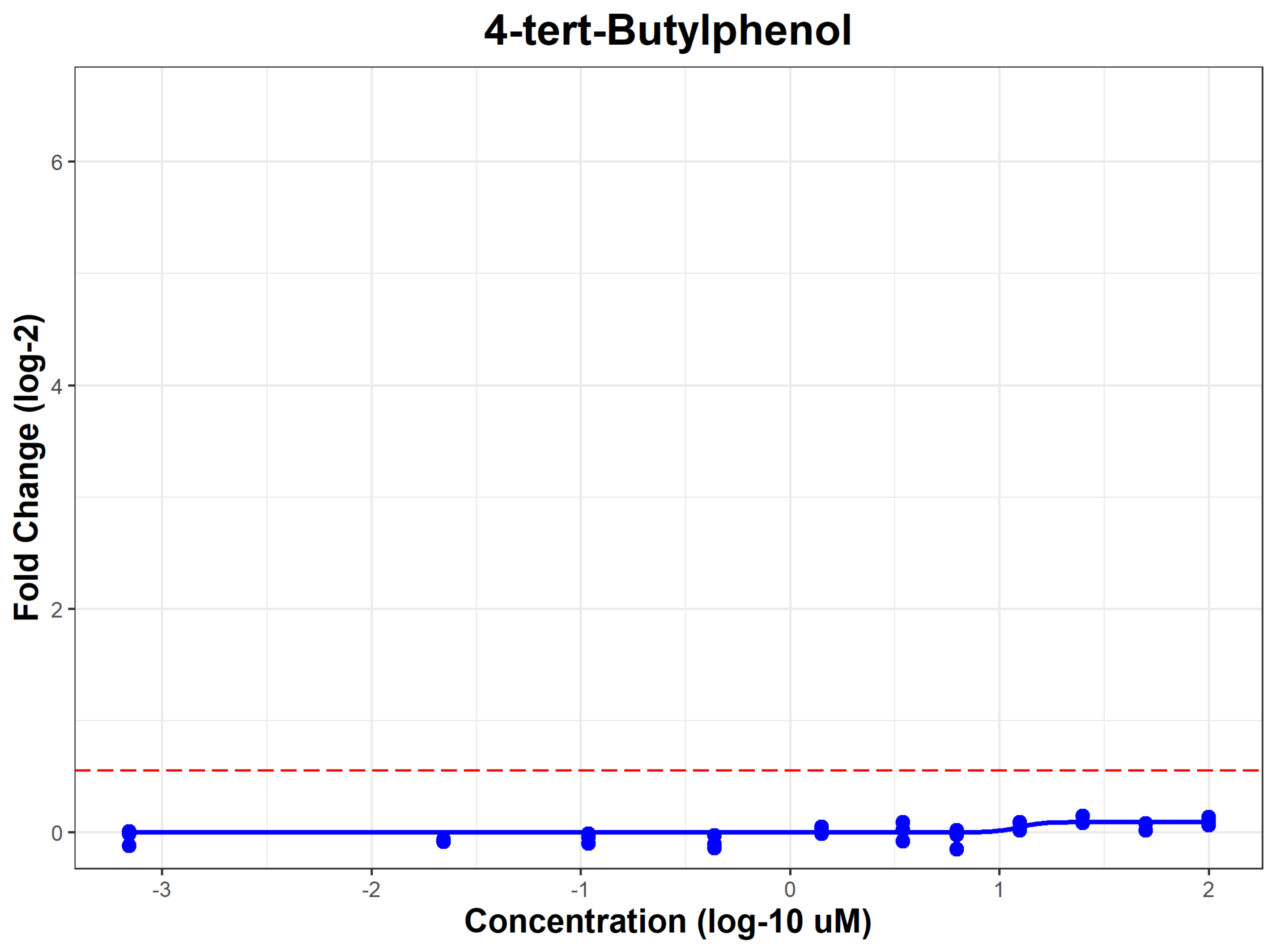

4-tert-Butylphenol
Fold Change (log-2)
6
4
2
0
-3
-2
-1
0
1
2
Concentration (log-10 uM)

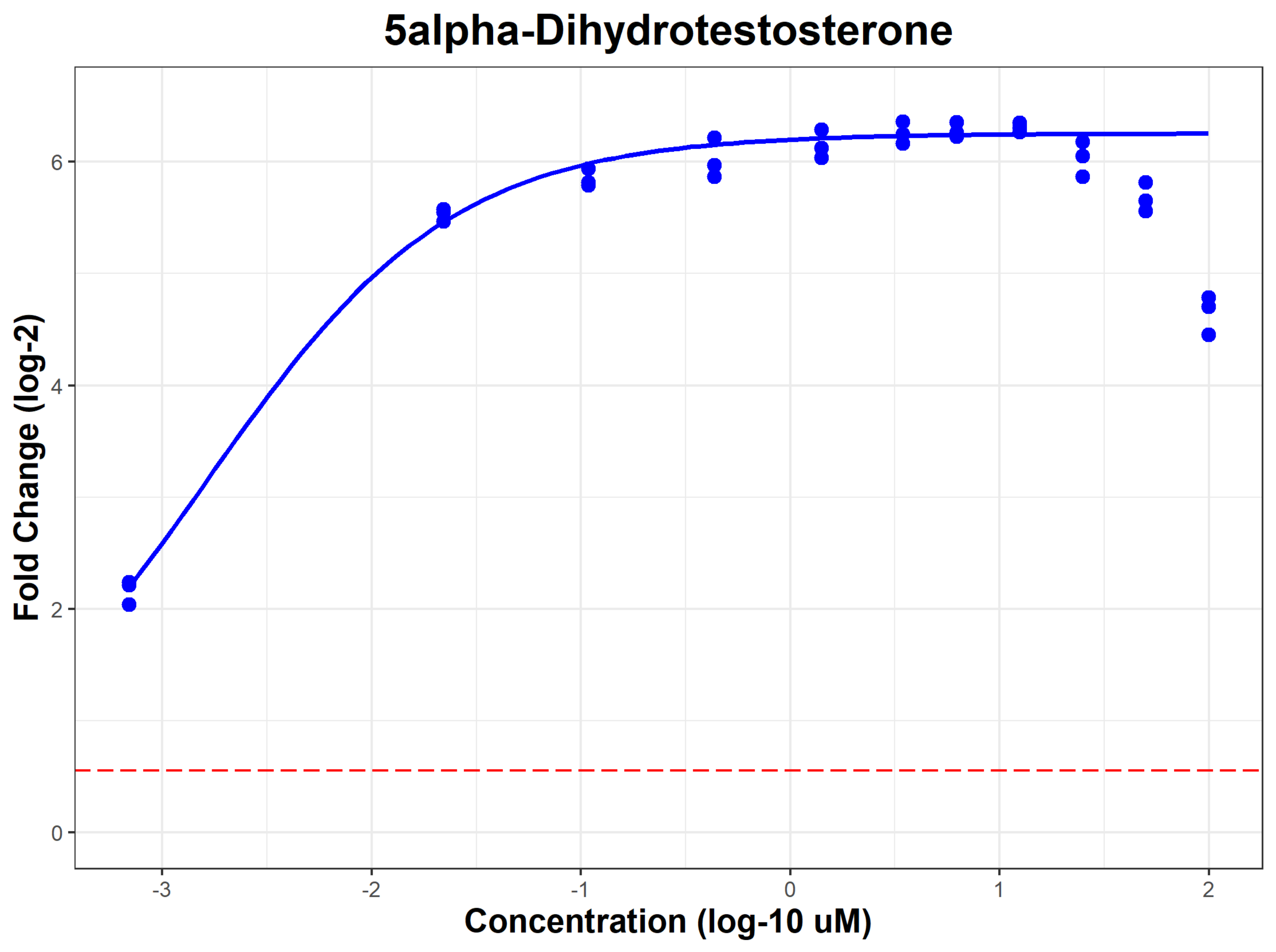

5alpha-Dihydrotestosterone
Fold Change (log-2)
Concentration (log-10 uM)
0
2
4
6
-3
-2
-1
0
1
2

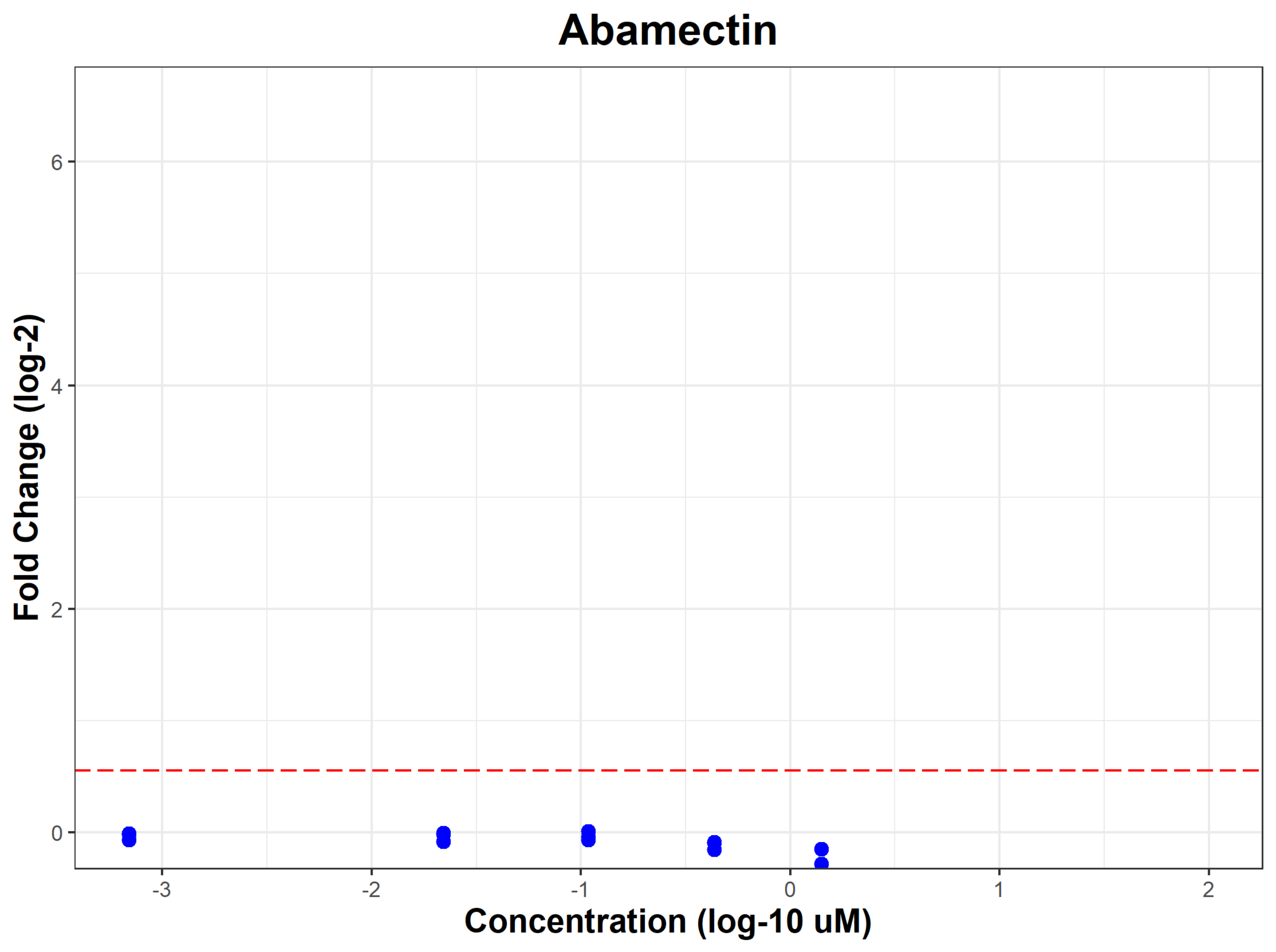

Abamectin
Fold Change (log-2)
6
4
2
0
-3
-2
-1
0
1
2
Concentration (log-10 uM)

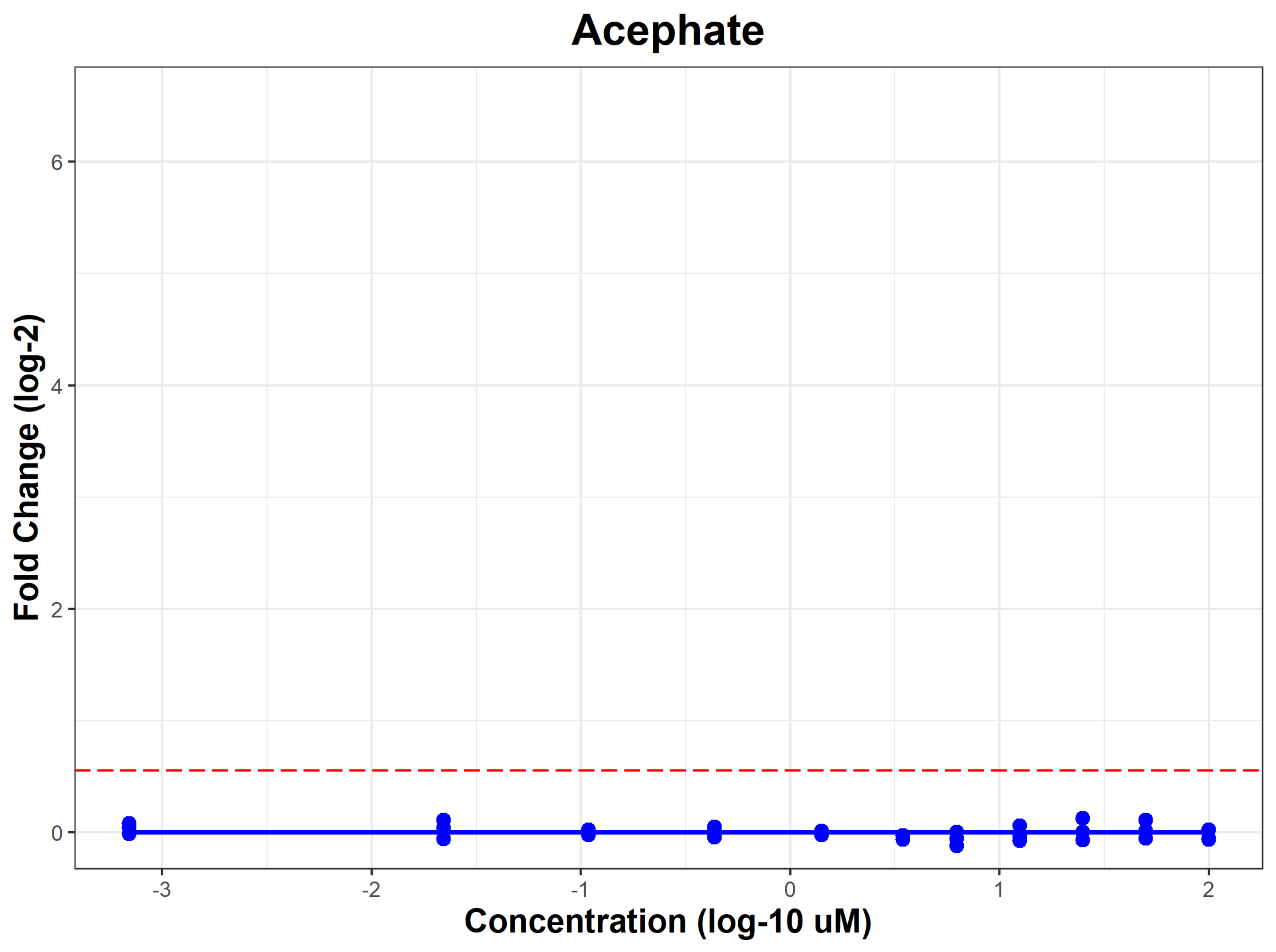

Acephate
Fold Change (log-2)
6
4
2
0
-3
-2
-1
0
1
2
Concentration (log-10 uM)

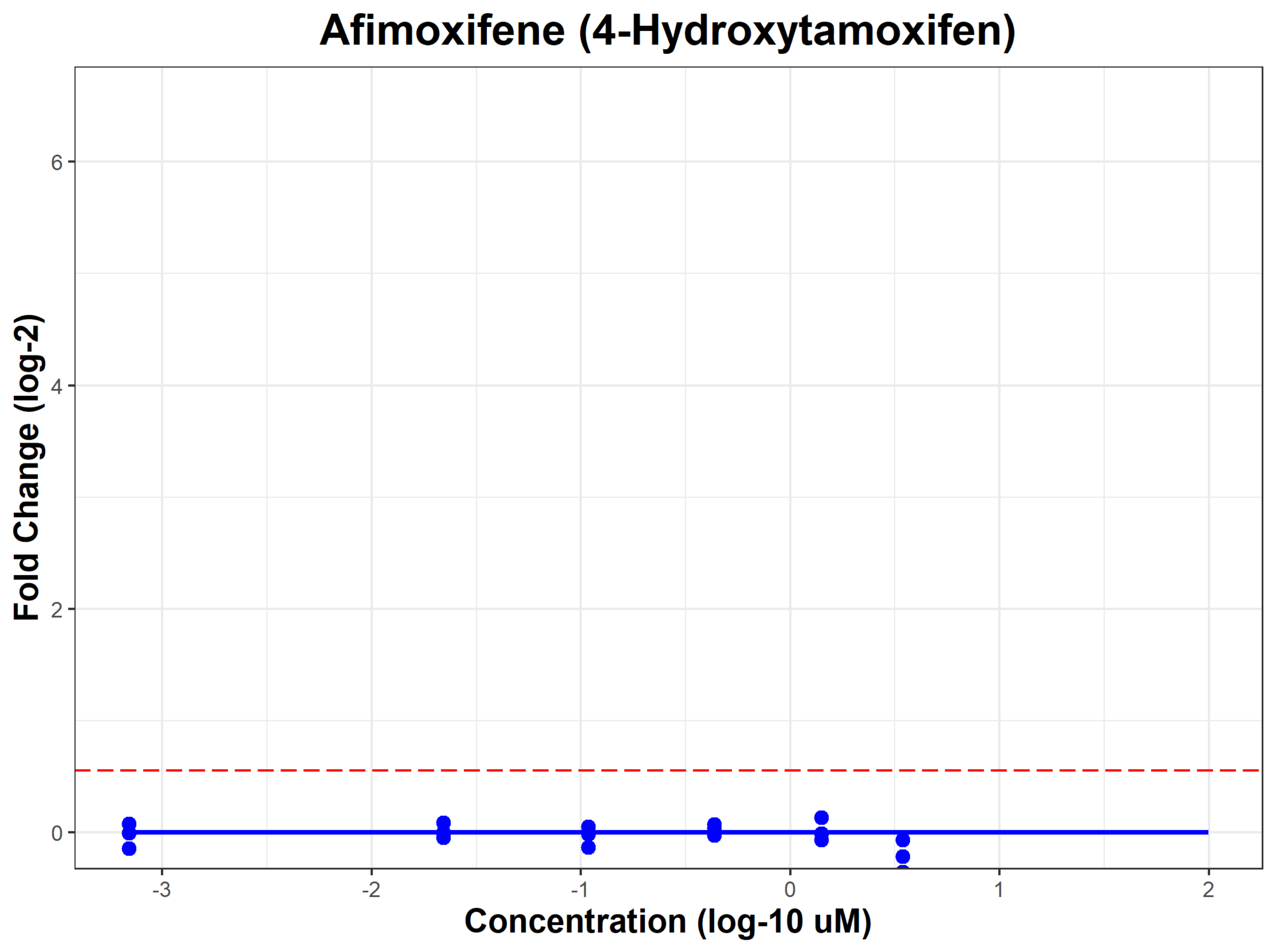

Afimoxifene (4-Hydroxytamoxifen)
Fold Change (log-2)
Concentration (log-10 uM)
6
4
2
0
-3
-2
-1
0
1
2

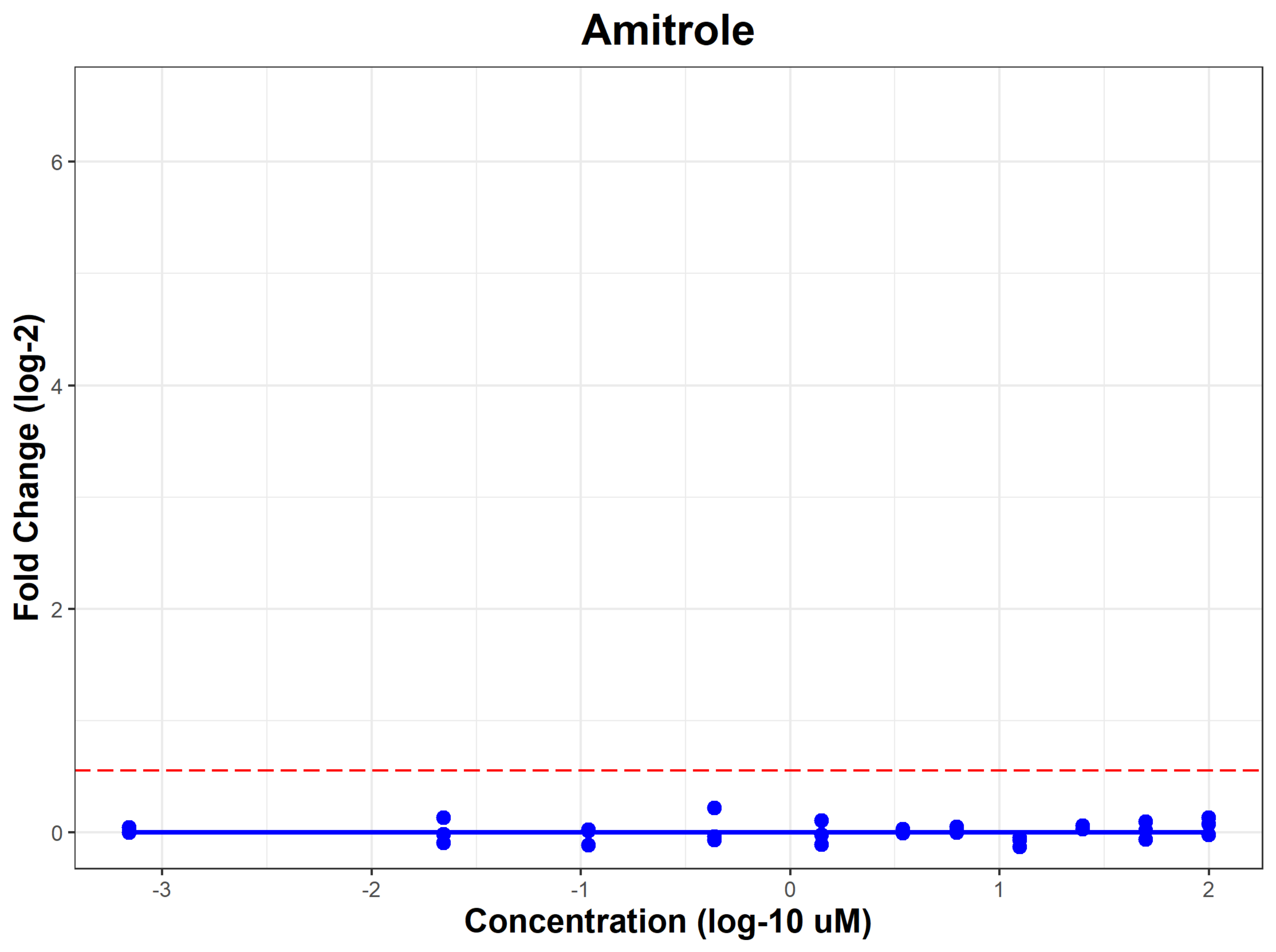

Amitrole
Fold Change (log-2)
6
4
2
0
-3
-2
-1
0
1
2
Concentration (log-10 uM)

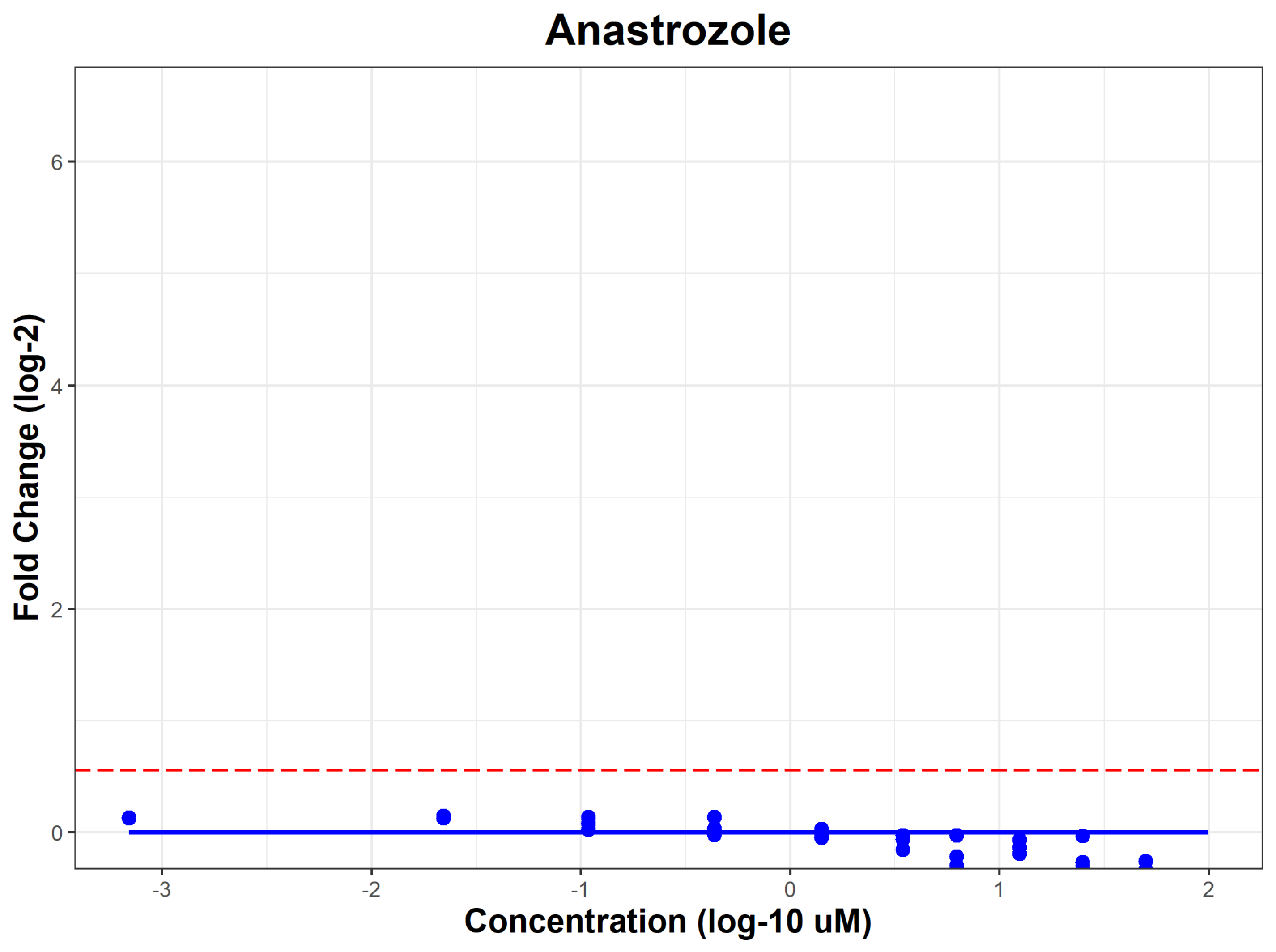

Anastrozole
Fold Change (log-2)
6
4
2
0
-3
-2
-1
0
1
2
Concentration (log-10 uM)

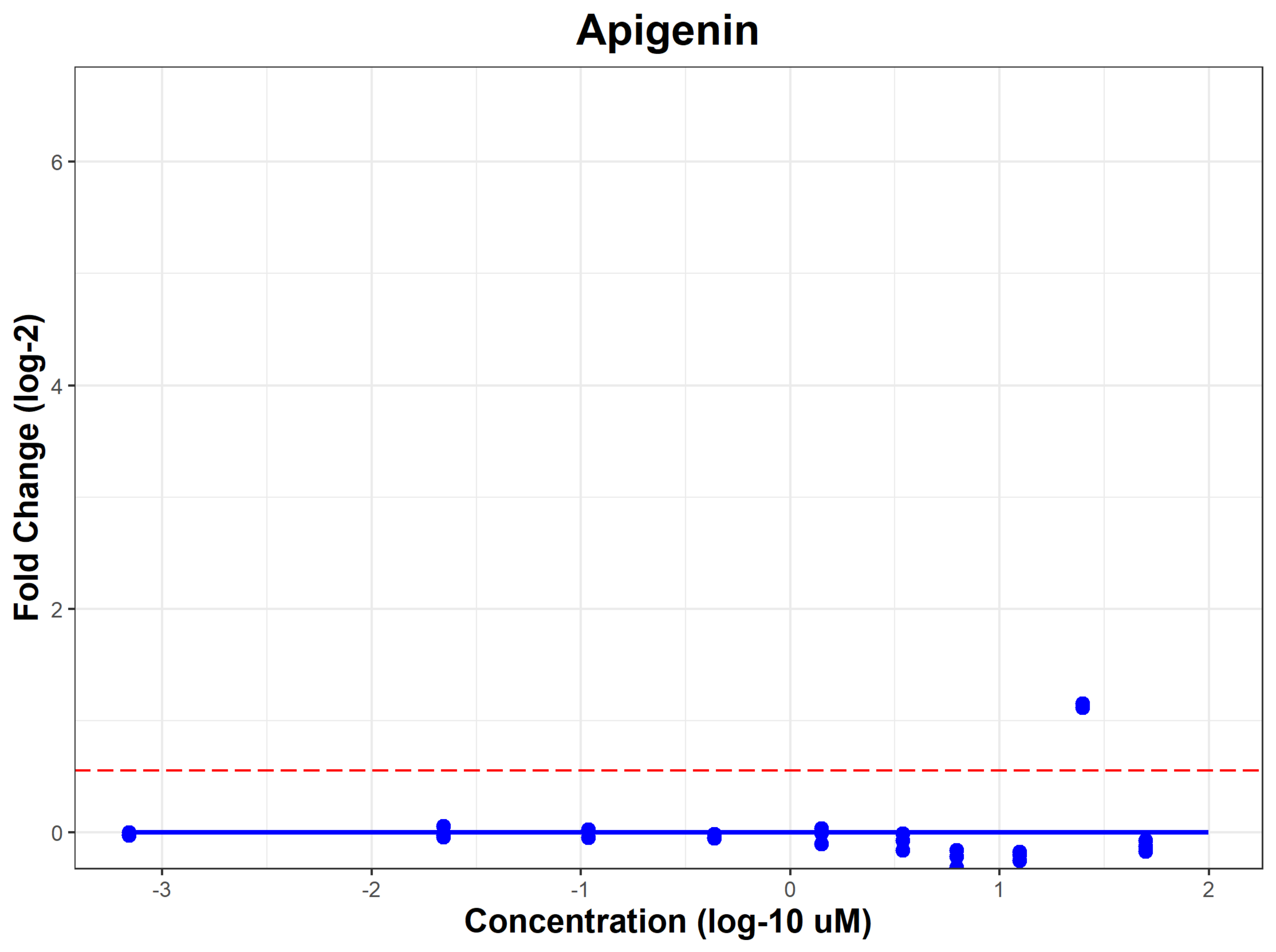
Apigenin
Fold Change (log-2)
6
4
2
0
-3
-2
-1
0
1
2
Concentration (log-10 uM)

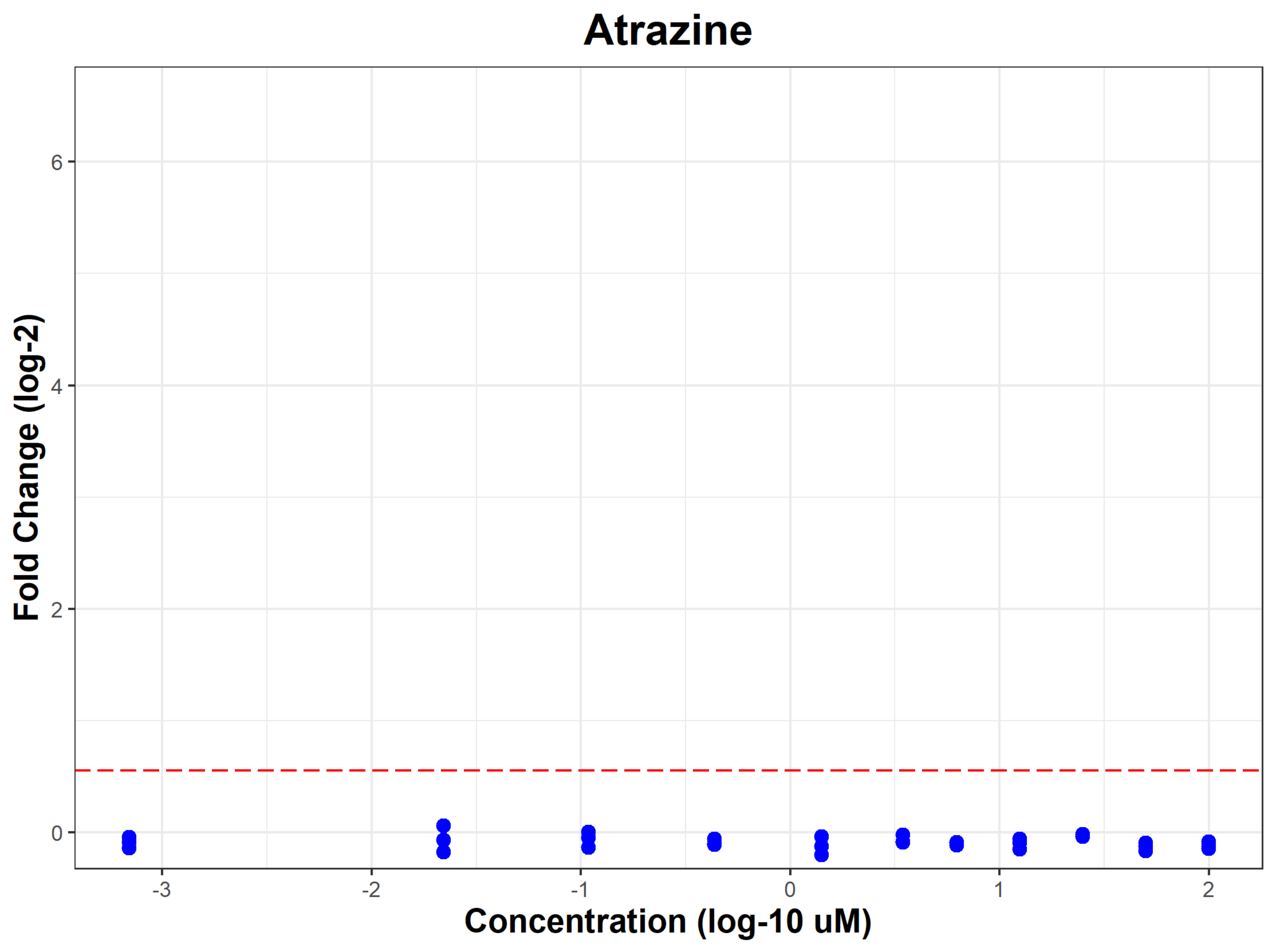

Atrazine
Fold Change (log-2)
6
4
2
0
-3
-2
-1
0
1
2
Concentration (log-10 uM)

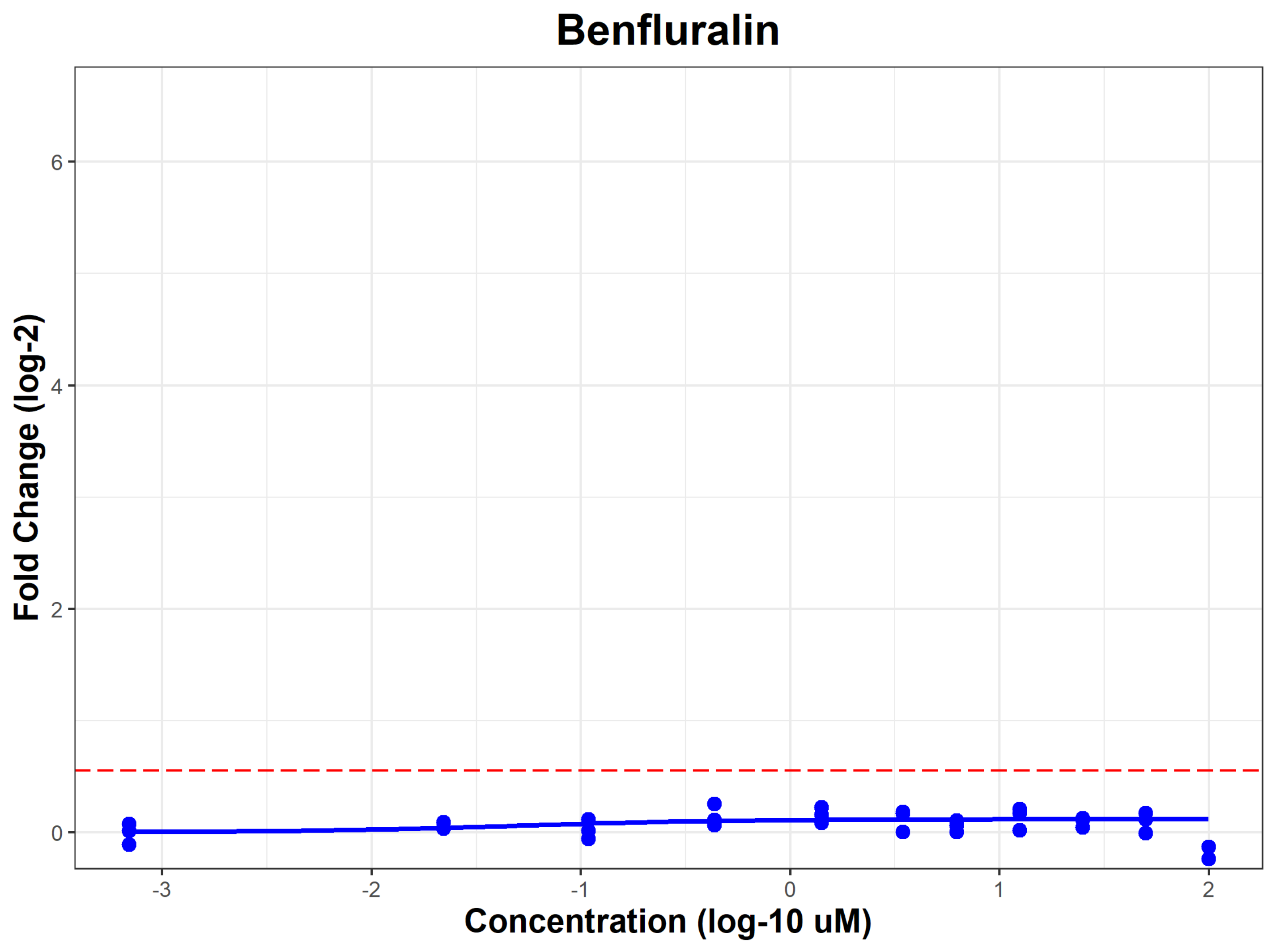

Benfluralin
Fold Change (log-2)
6
4
2
0
-3
-2
-1
0
1
2
Concentration (log-10 uM)

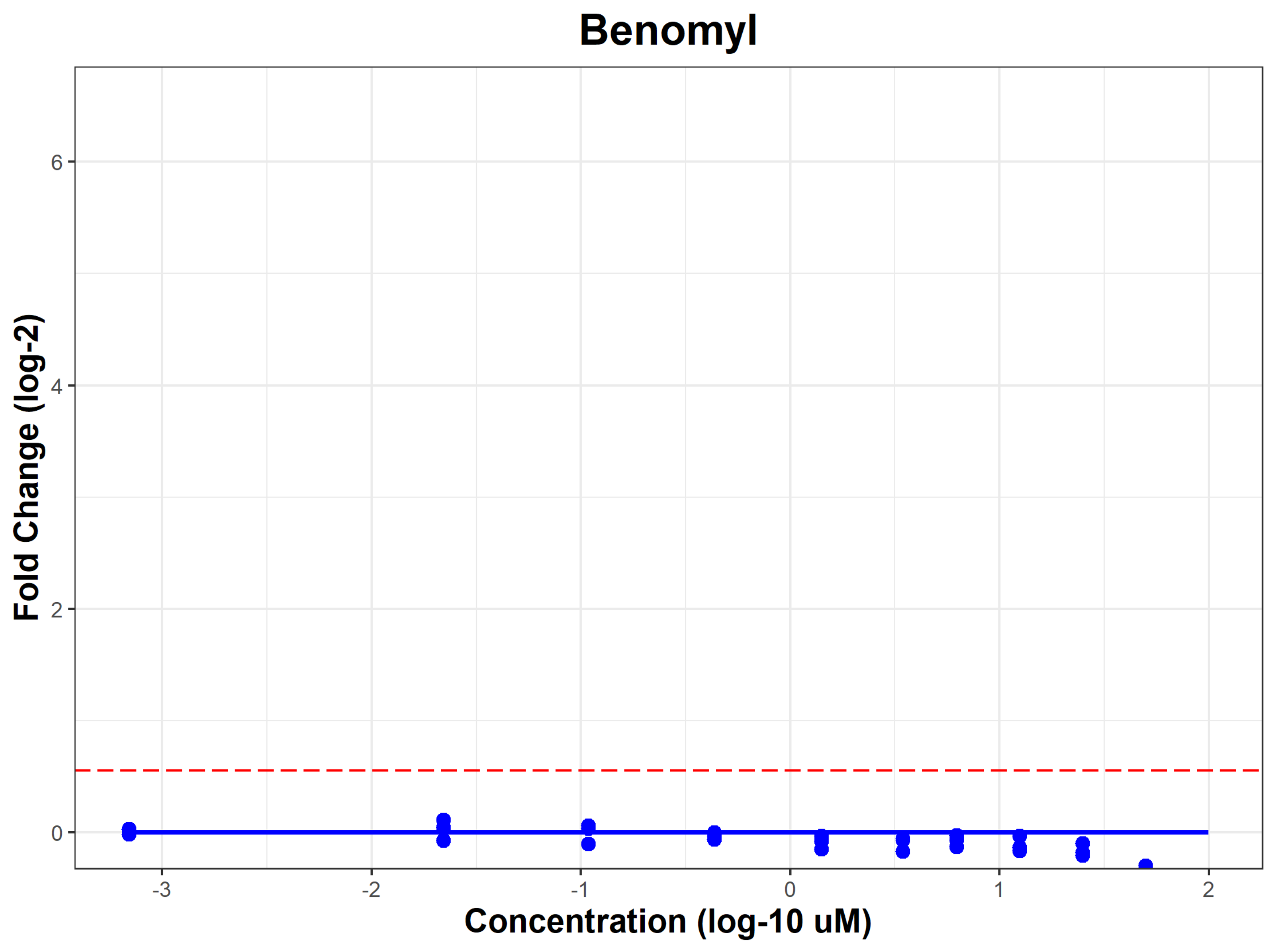
Benomyl
Fold Change (log-2)
6
4
2
0
-3
-2
-1
0
1
2
Concentration (log-10 uM)

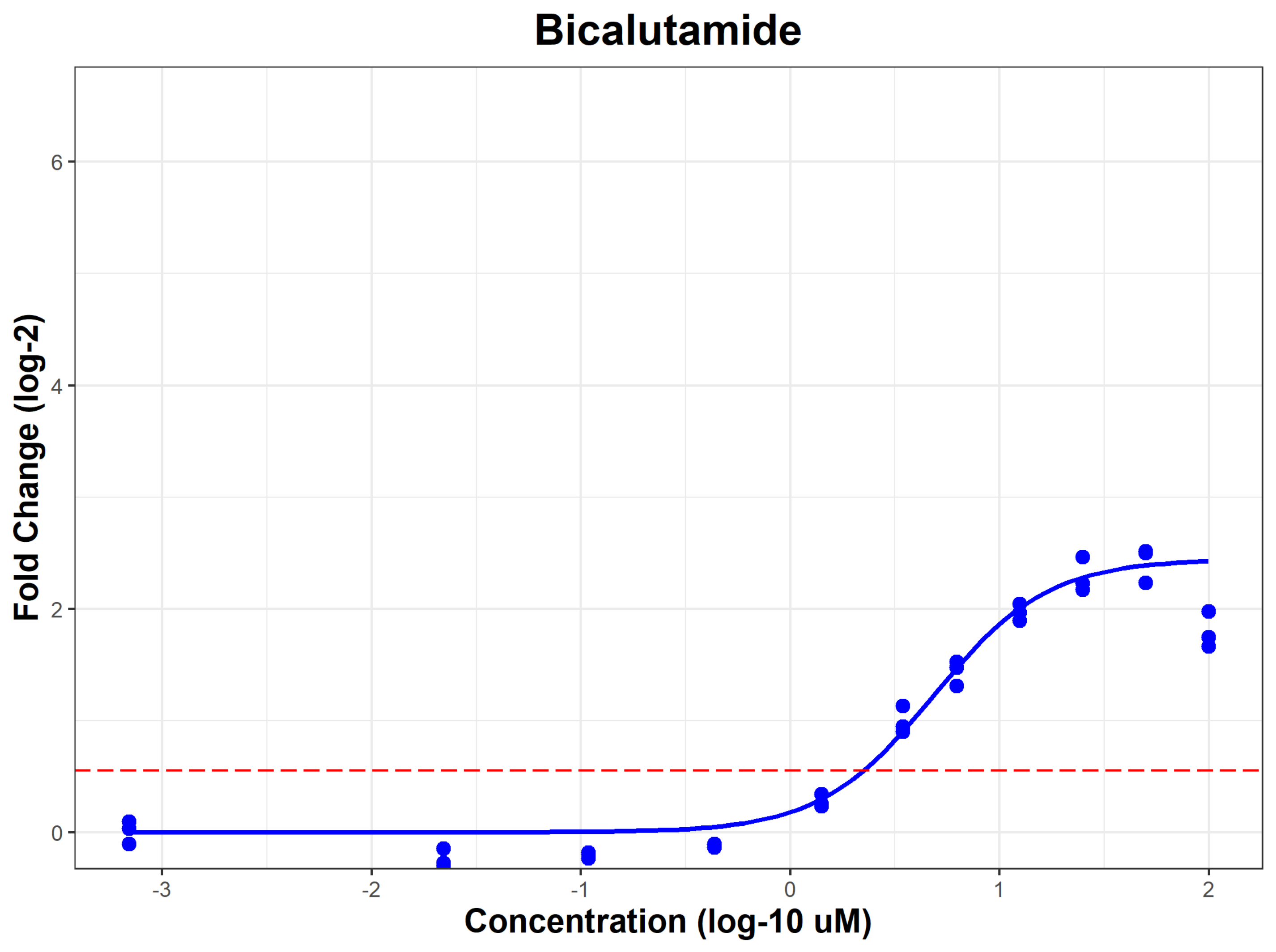

Bicalutamide
Fold Change (log-2)
6
4
2
0
-3
-2
-1
0
1
2
Concentration (log-10 uM)

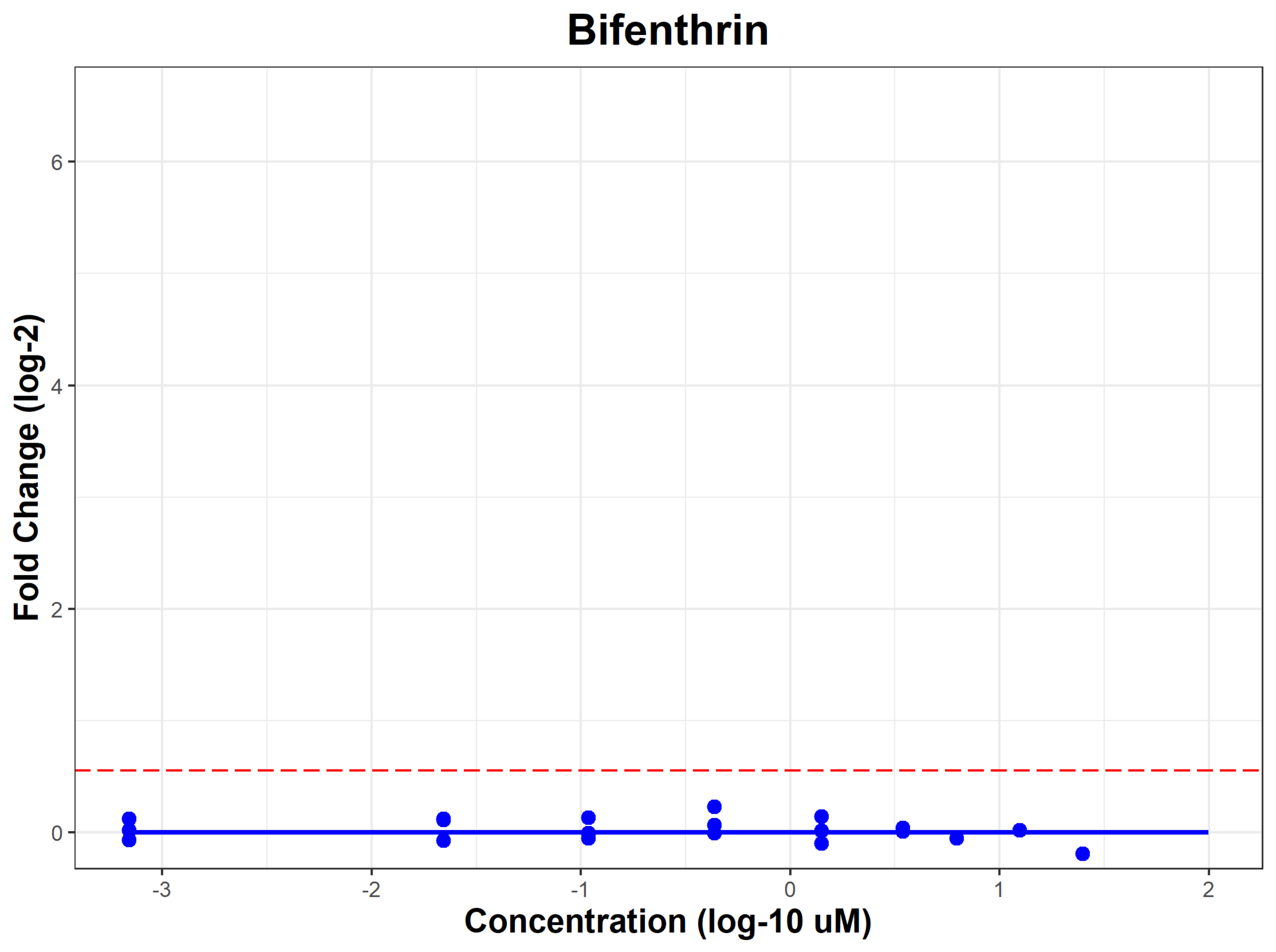
Bifenthrin
Fold Change (log-2)
6
4
2
0
-3
-2
-1
0
1
2
Concentration (log-10 uM)

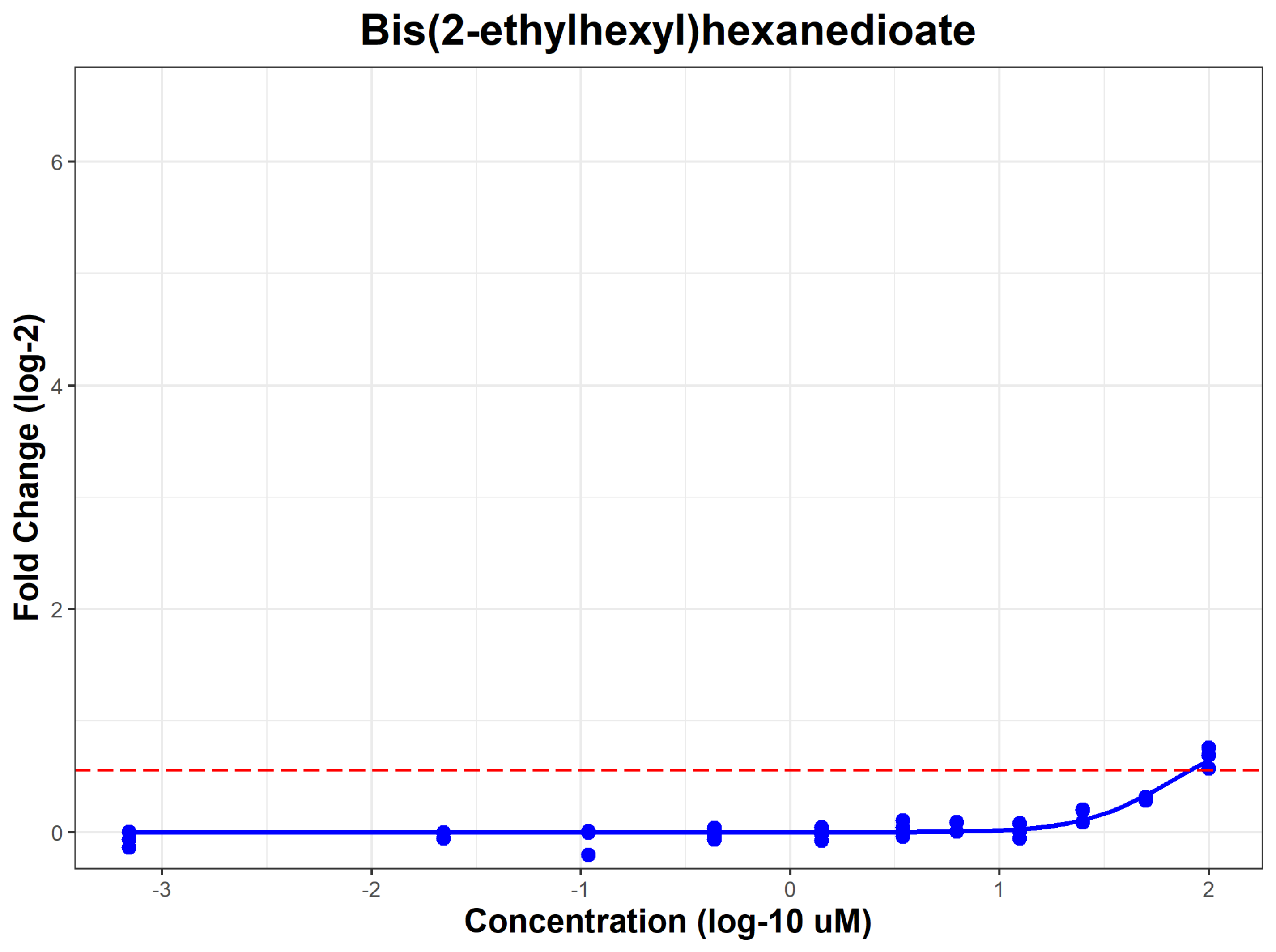
Bis(2-ethylhexyl)hexanedioate
Fold Change (log-2)
6
4
2
0
-3
-2
-1
0
1
2
Concentration (log-10 uM)

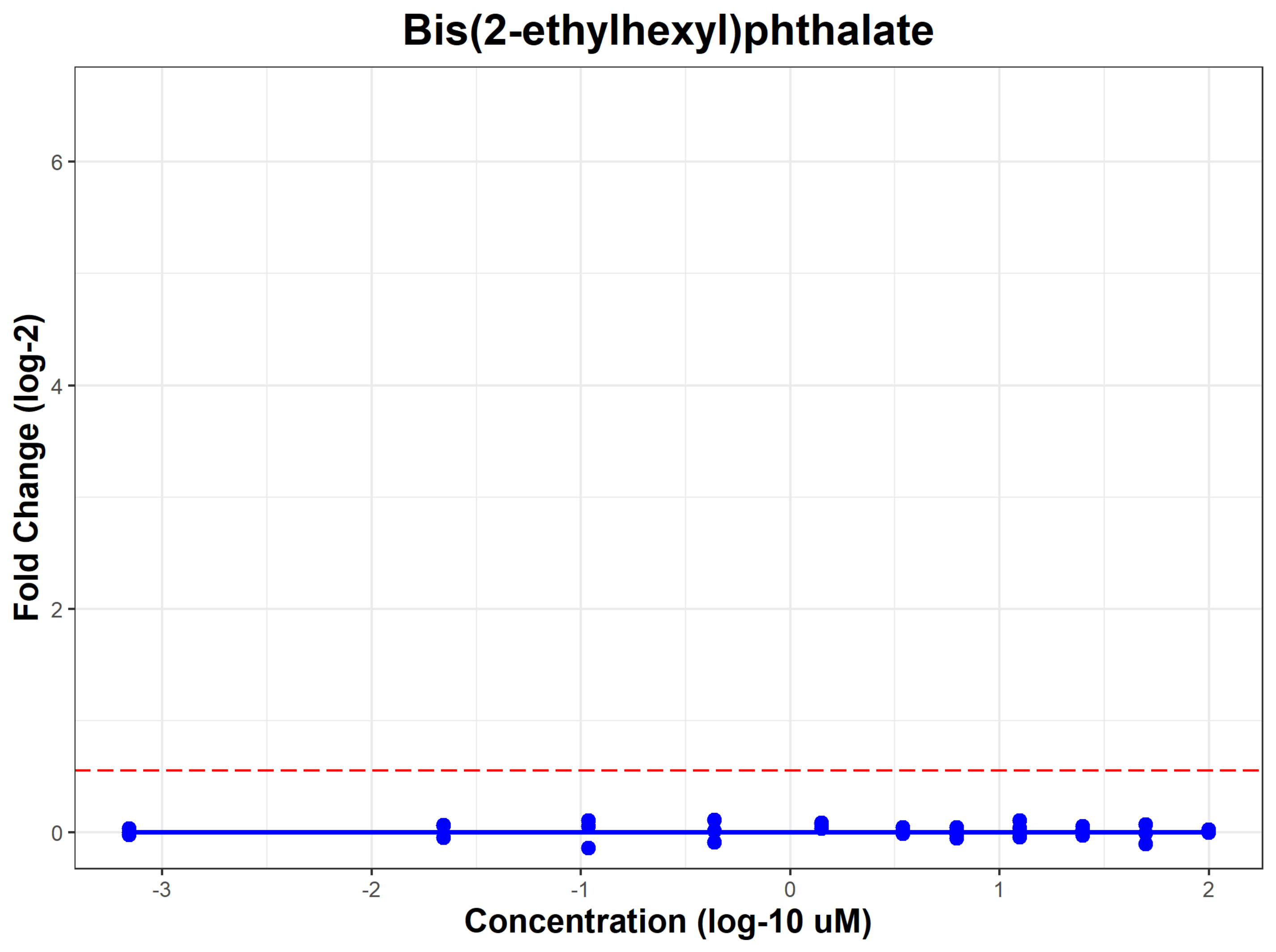

Bis(2-ethylhexyl)phthalate
Fold Change (log-2)
Concentration (log-10 uM)
6
4
2
0
-3
-2
-1
0
1
2

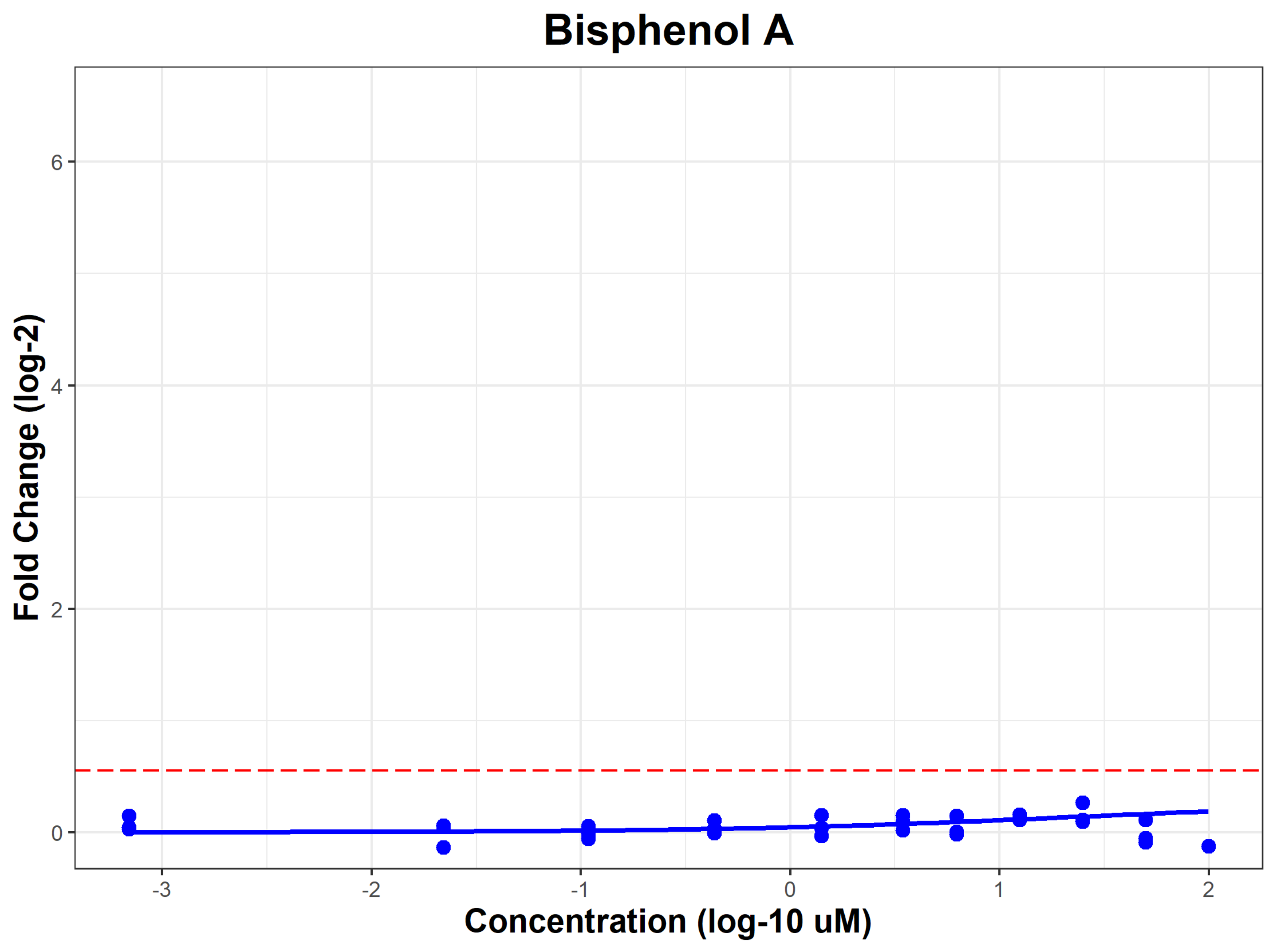

Bisphenol A
Fold Change (log-2)
6
4
2
0
-3
-2
-1
0
1
2
Concentration (log-10 uM)

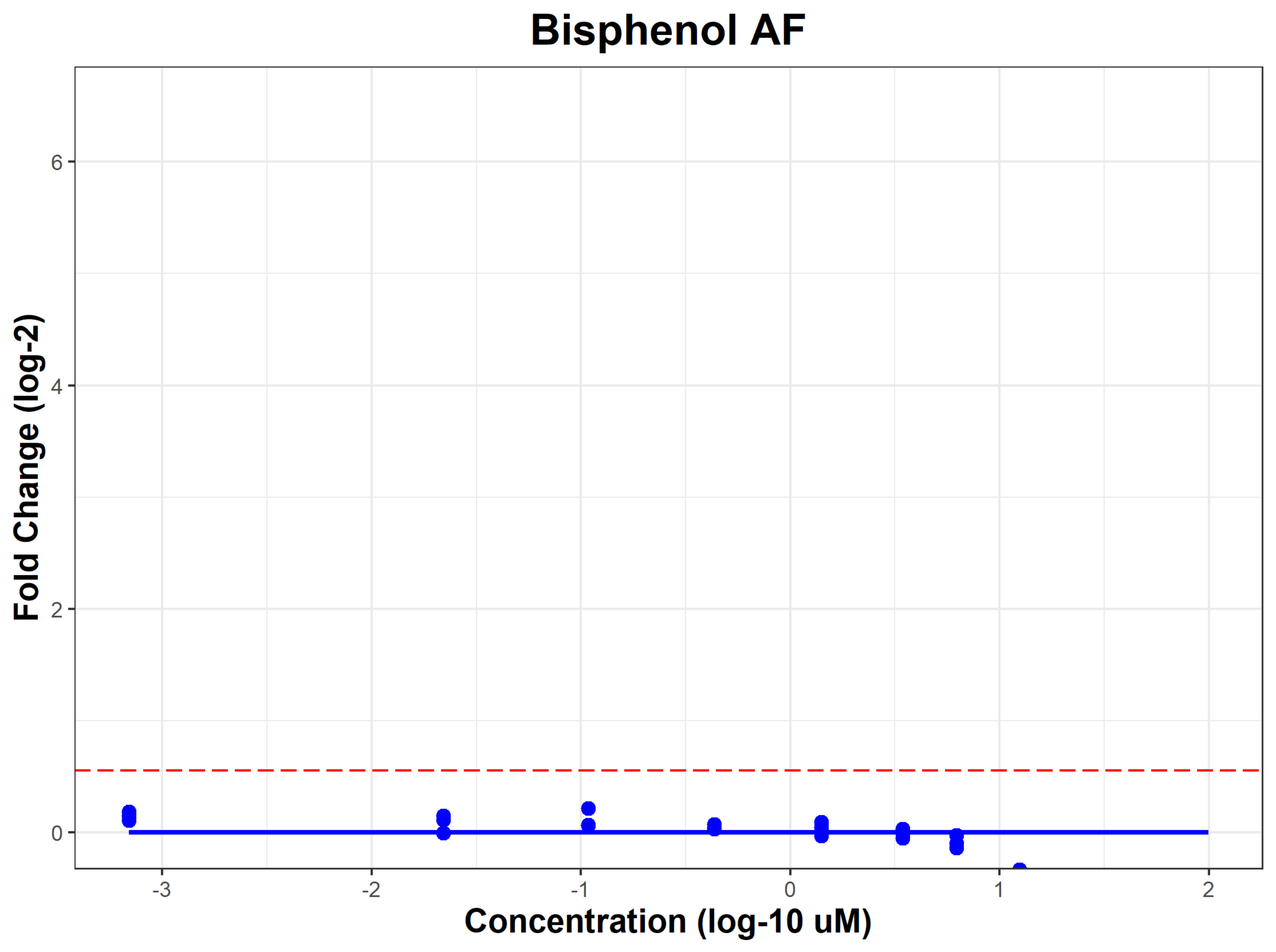

Bisphenol AF
Fold Change (log-2)
Concentration (log-10 uM)
6
4
2
0
-3
-2
-1
0
1
2

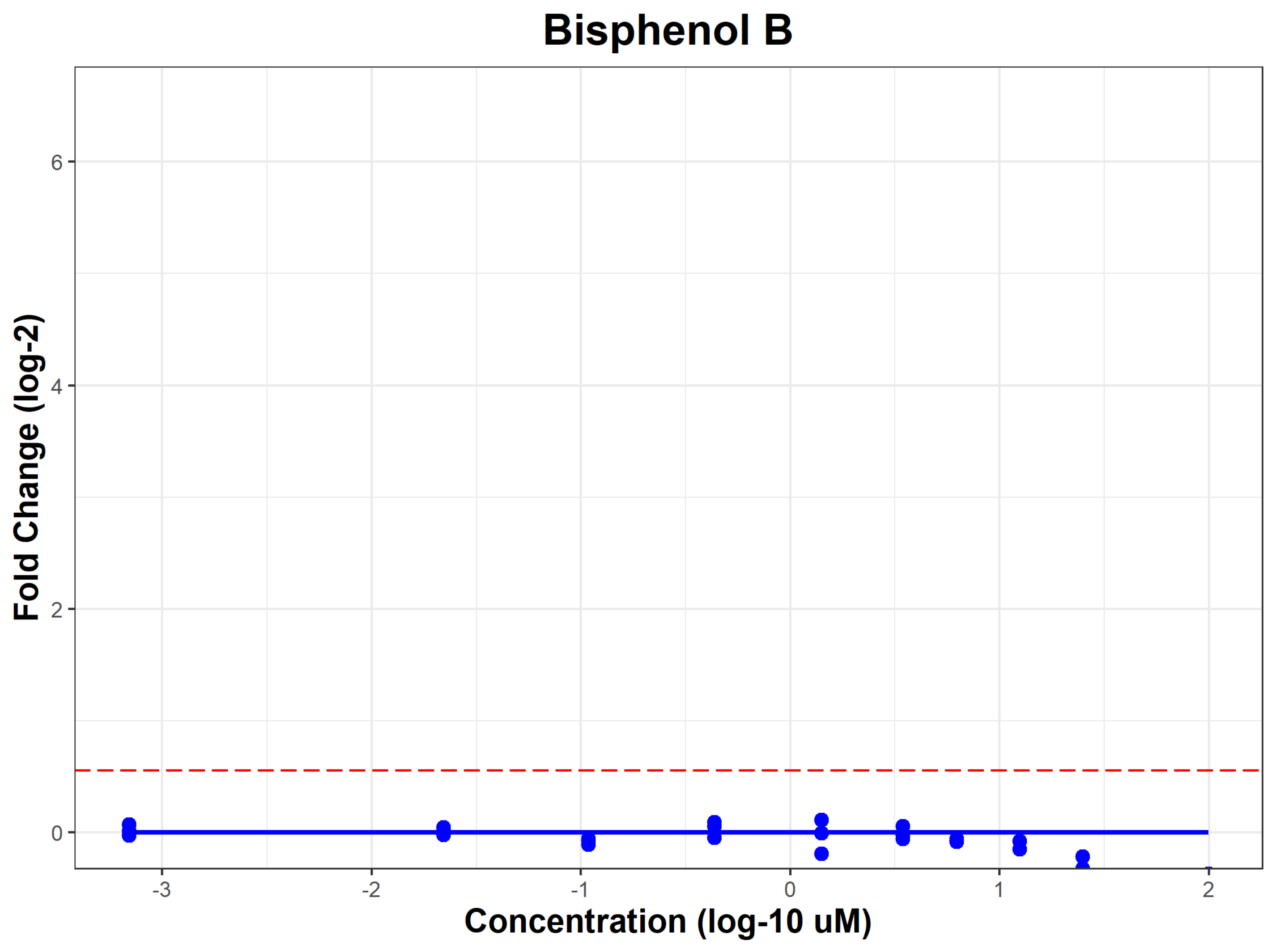

Bisphenol B
Fold Change (log-2)
6
4
2
0
-3
-2
-1
0
1
2
Concentration (log-10 uM)

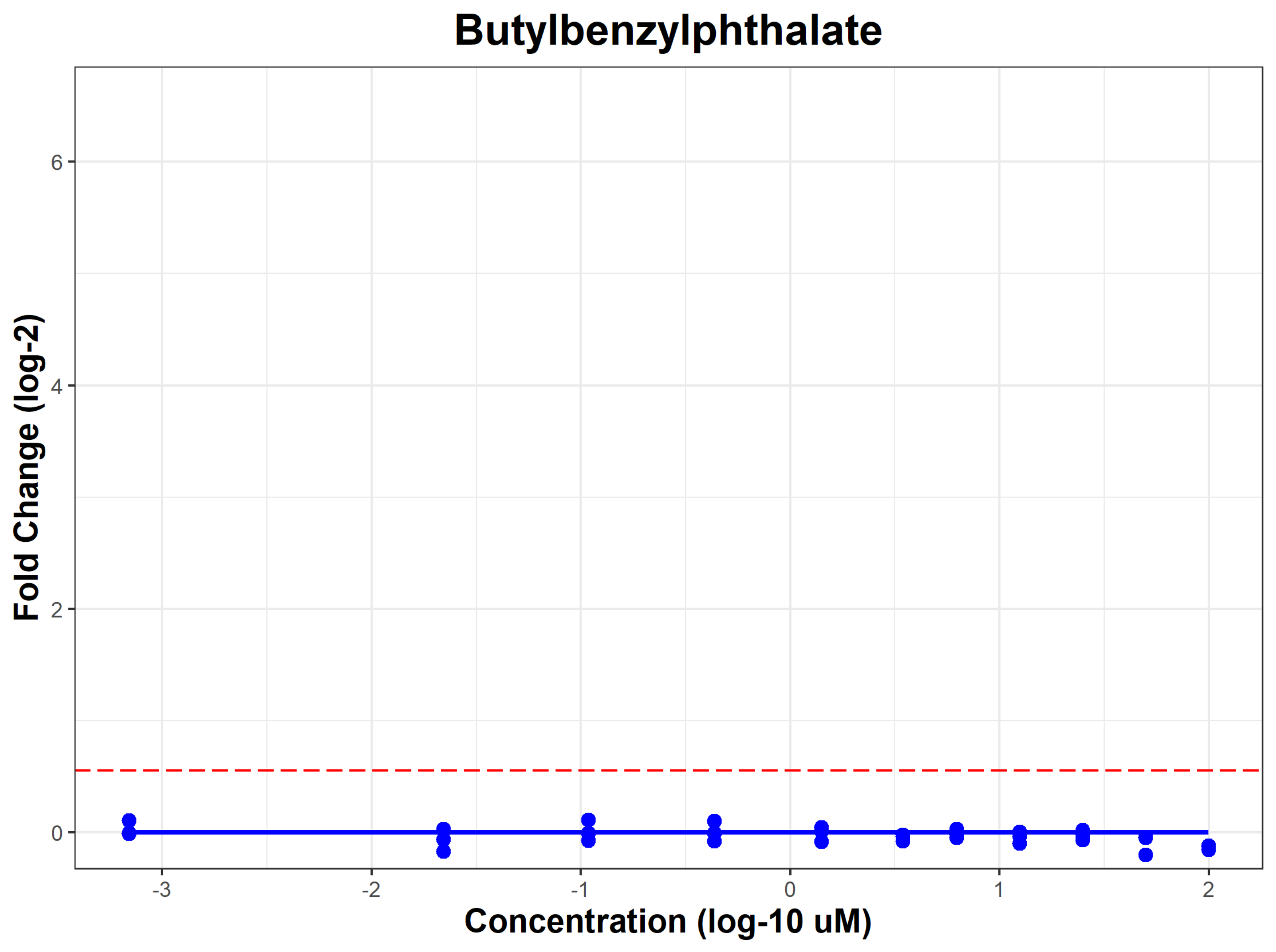

Butylbenzylphthalate
Fold Change (log-2)
6
4
2
0
-3
-2
-1
0
1
2
Concentration (log-10 uM)

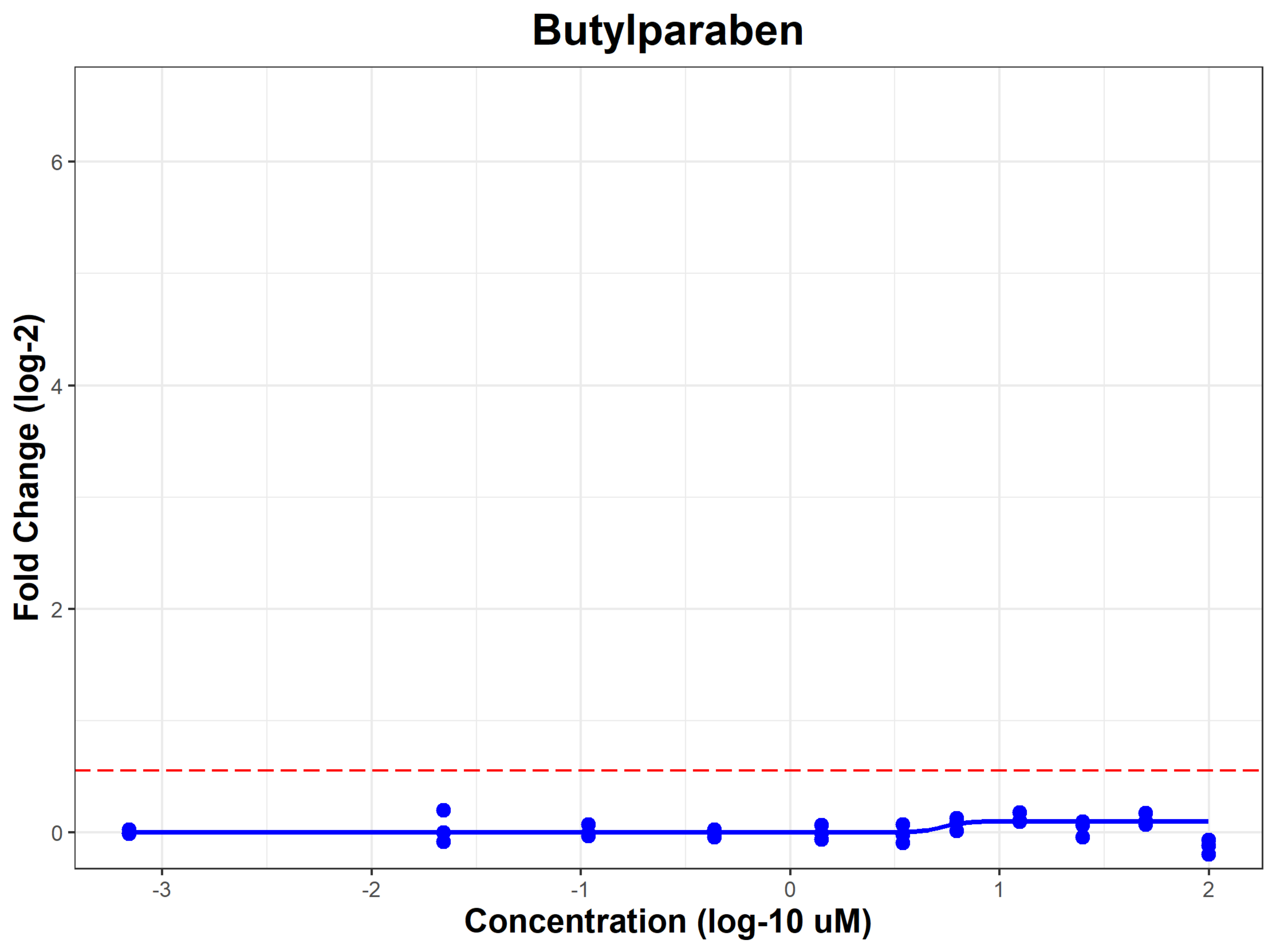

Butylparaben
Fold Change (log-2)
6
4
2
0
-3
-2
-1
0
1
2
Concentration (log-10 uM)

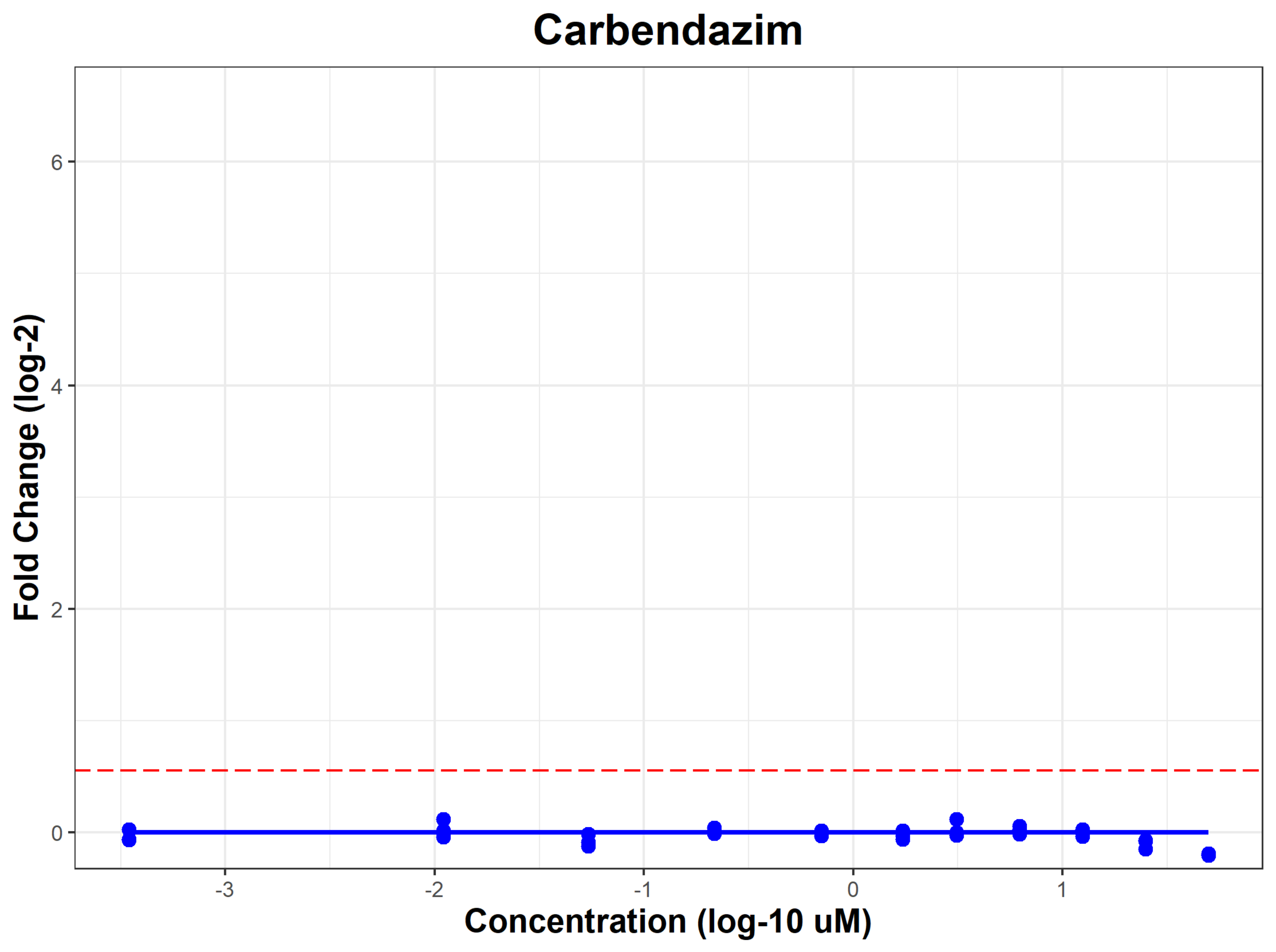

Carbendazim
Fold Change (log-2)
6
4
2
0
-3
-2
-1
0
1
Concentration (log-10 uM)

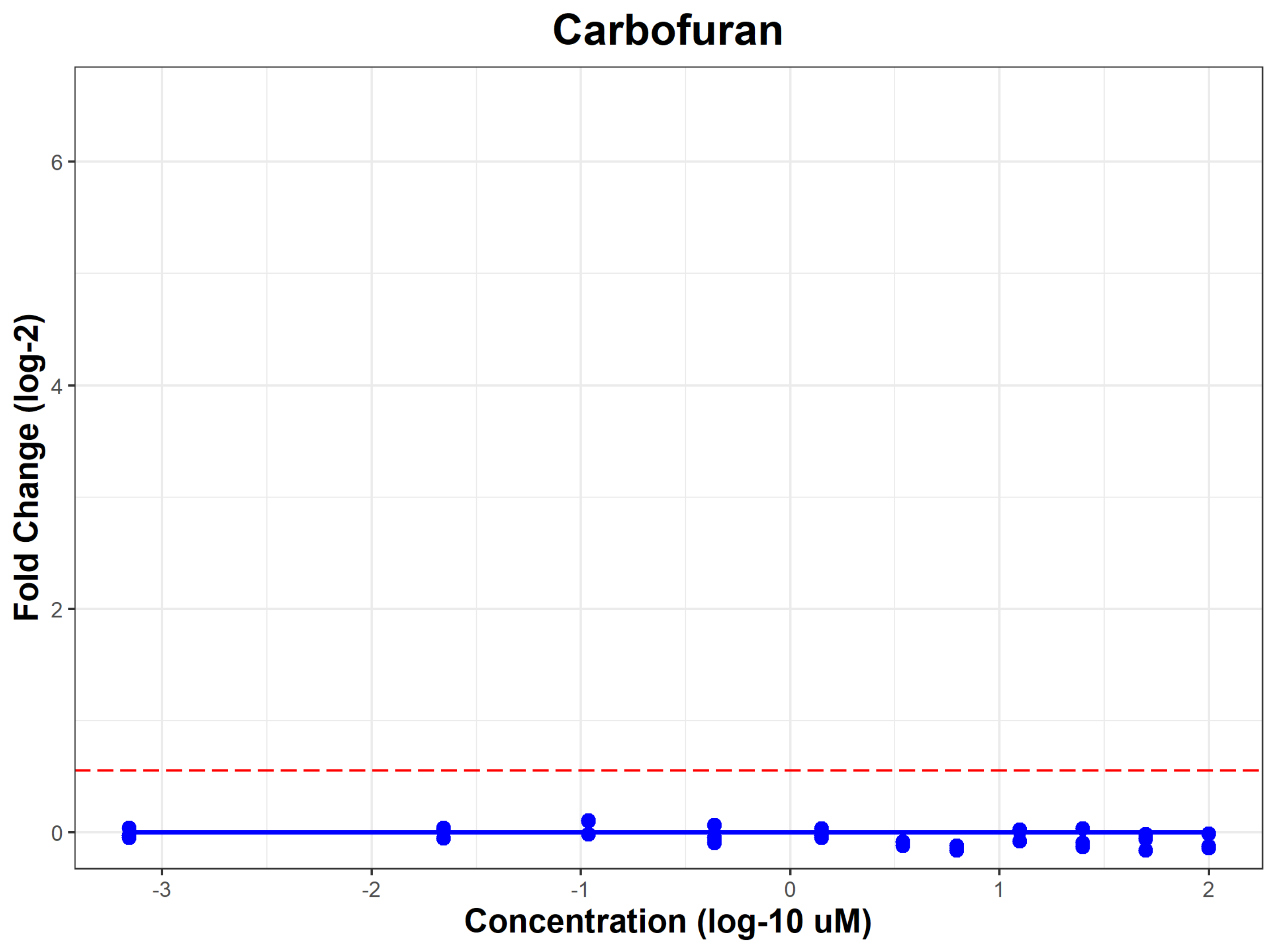

Carbofuran
Fold Change (log-2)
6
4
2
0
-3
-2
-1
0
1
2
Concentration (log-10 uM)

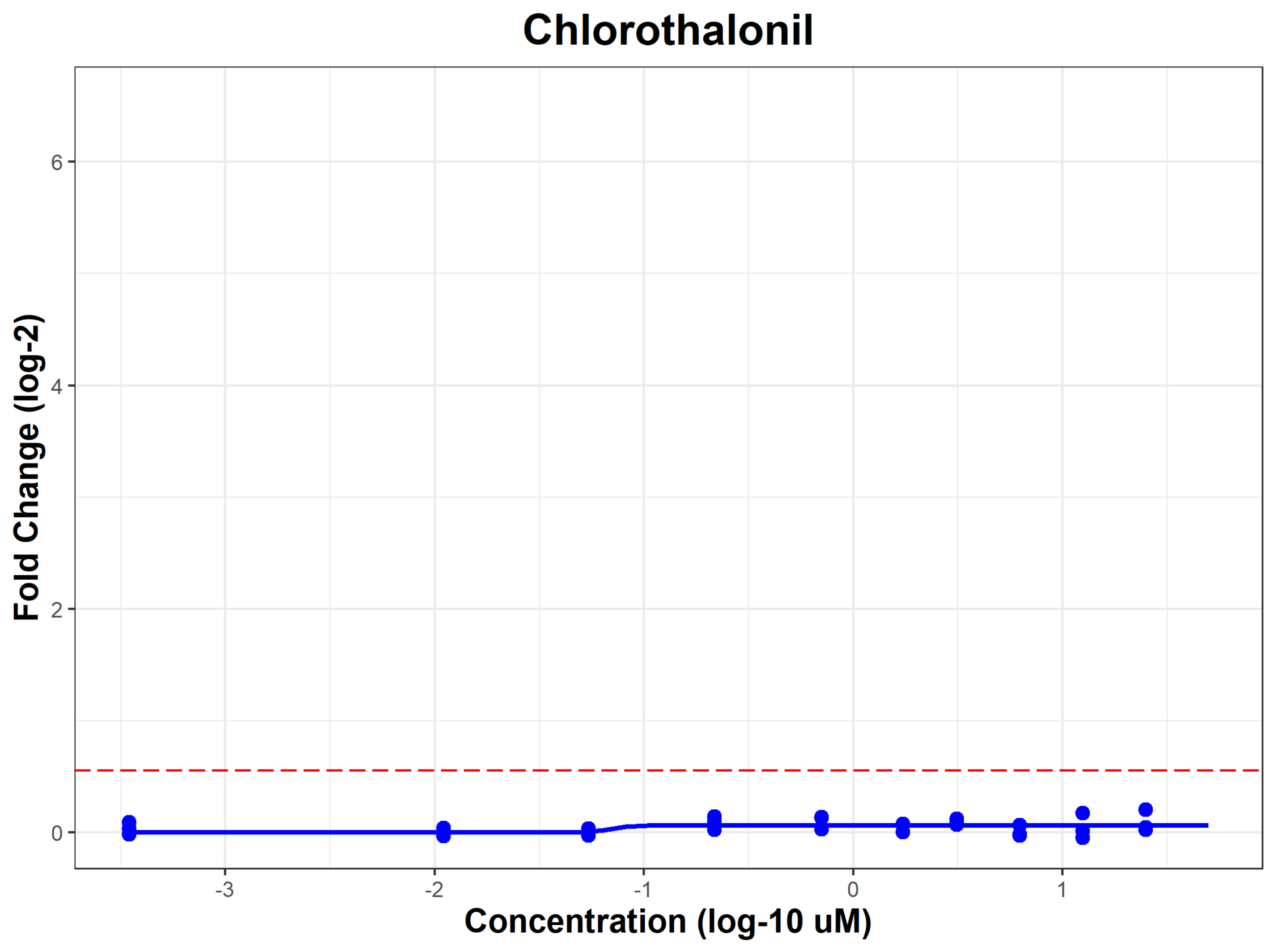
Chlorothalonil
Fold Change (log-2)
0
2
4
6
-3
-2
-1
0
1
Concentration (log-10 uM)

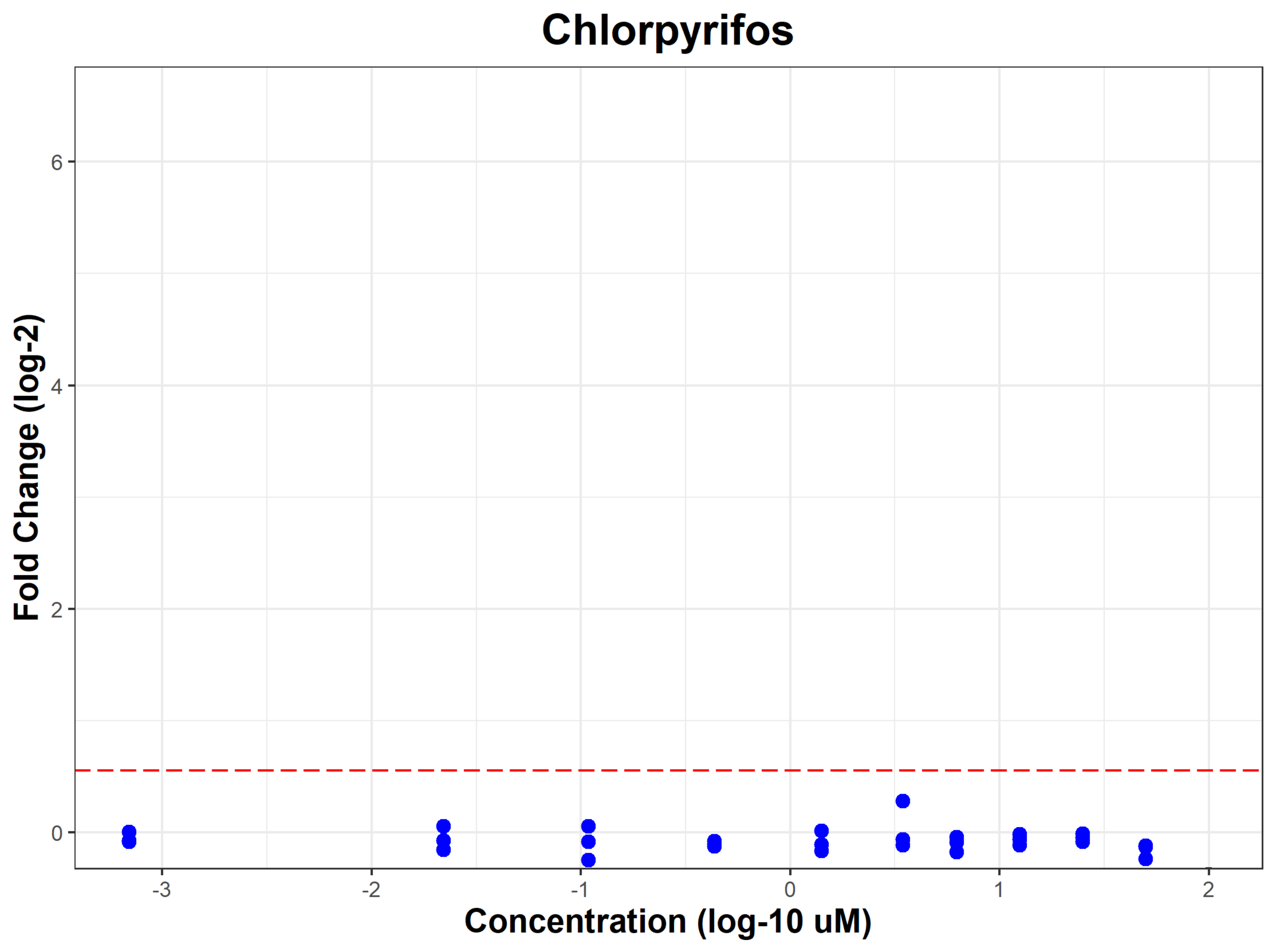

Chlorpyrifos
Fold Change (log-2)
6
4
2
0
-3
-2
-1
0
1
2
Concentration (log-10 uM)

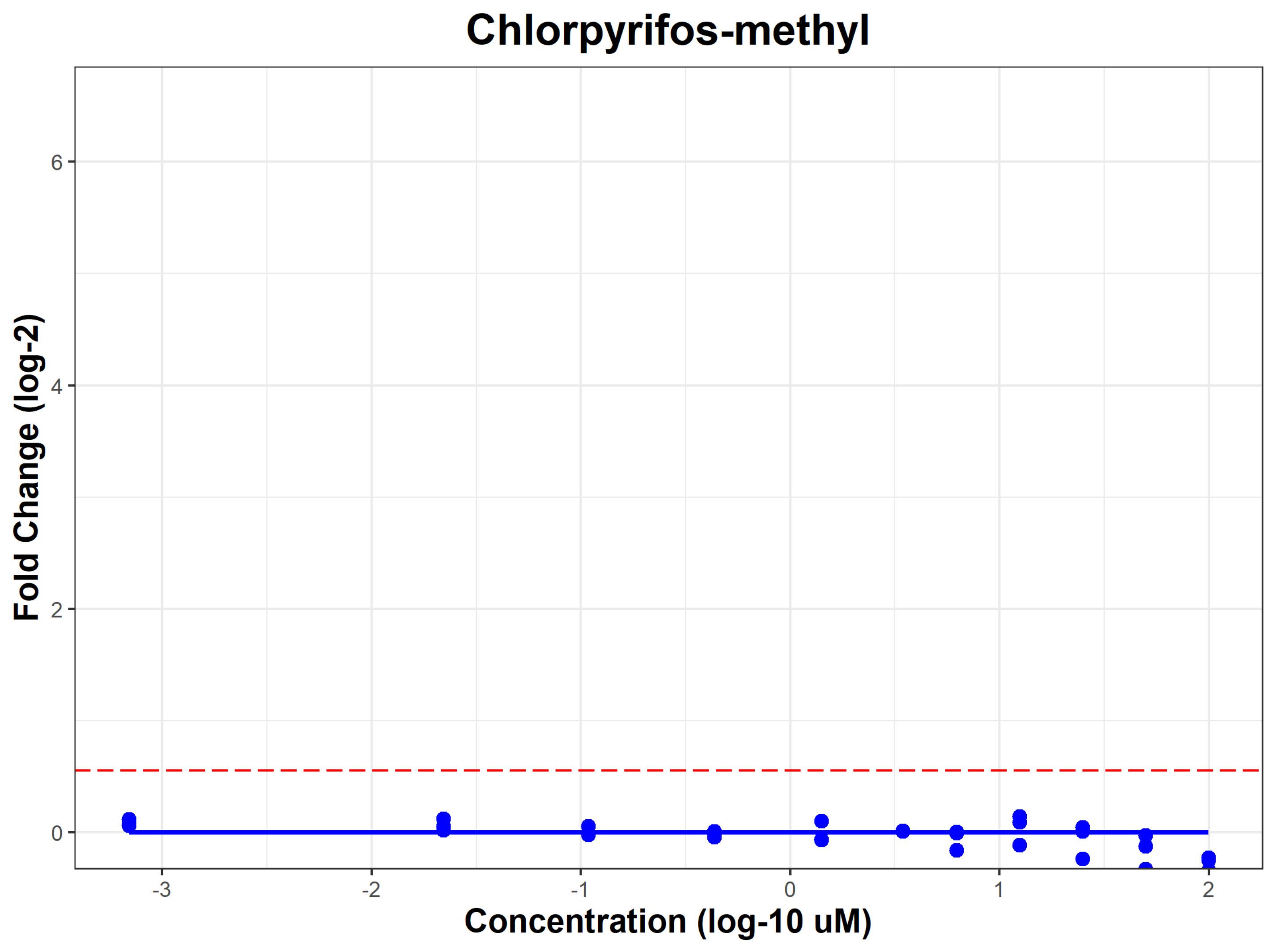

Chlorpyrifos-methyl
Fold Change (log-2)
6
4
2
0
-3
-2
-1
0
1
2
Concentration (log-10 uM)

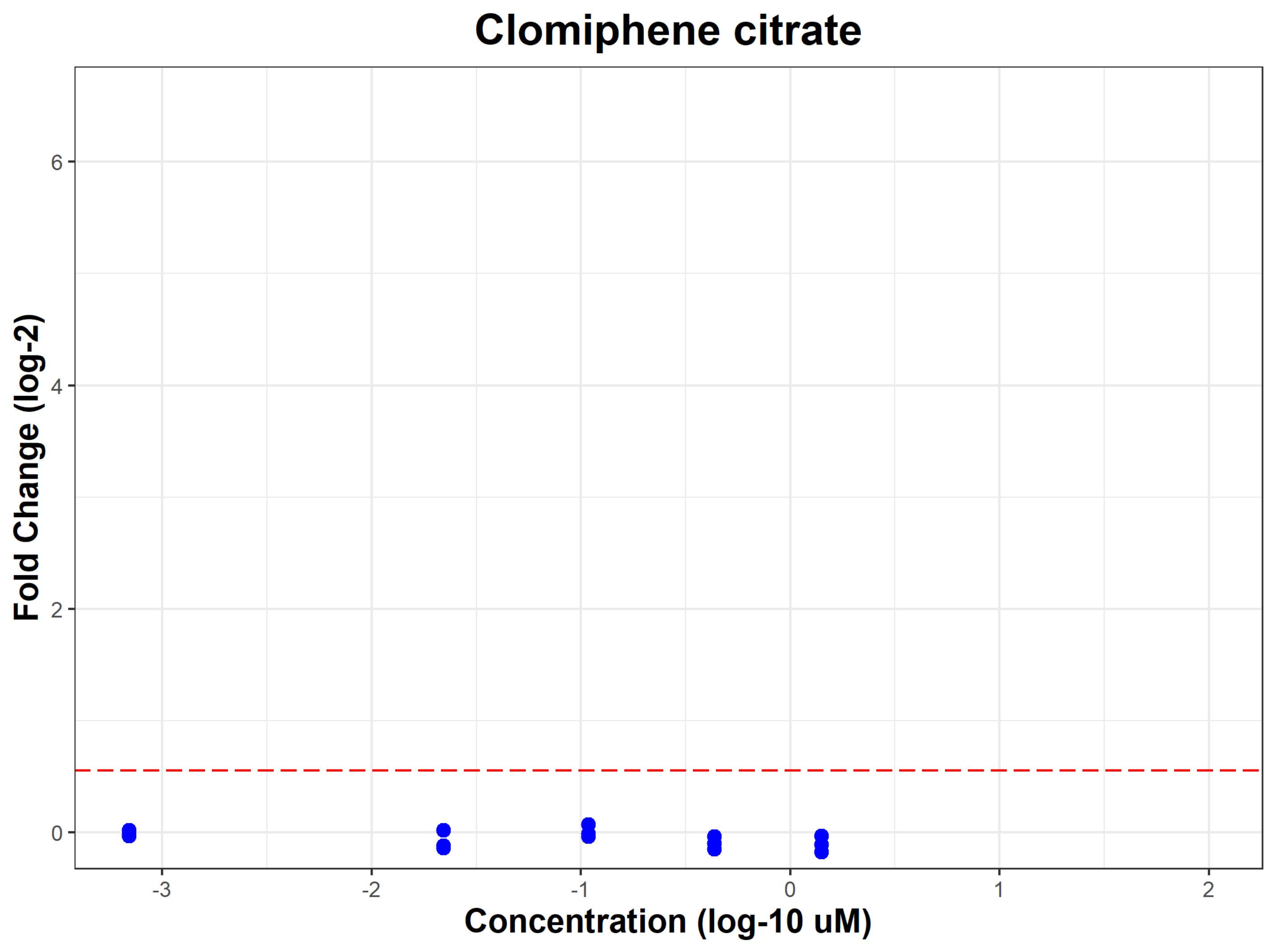
Clomiphene citrate
Fold Change (log-2)
6
4
2
0
-3
-2
-1
0
1
2
Concentration (log-10 uM)

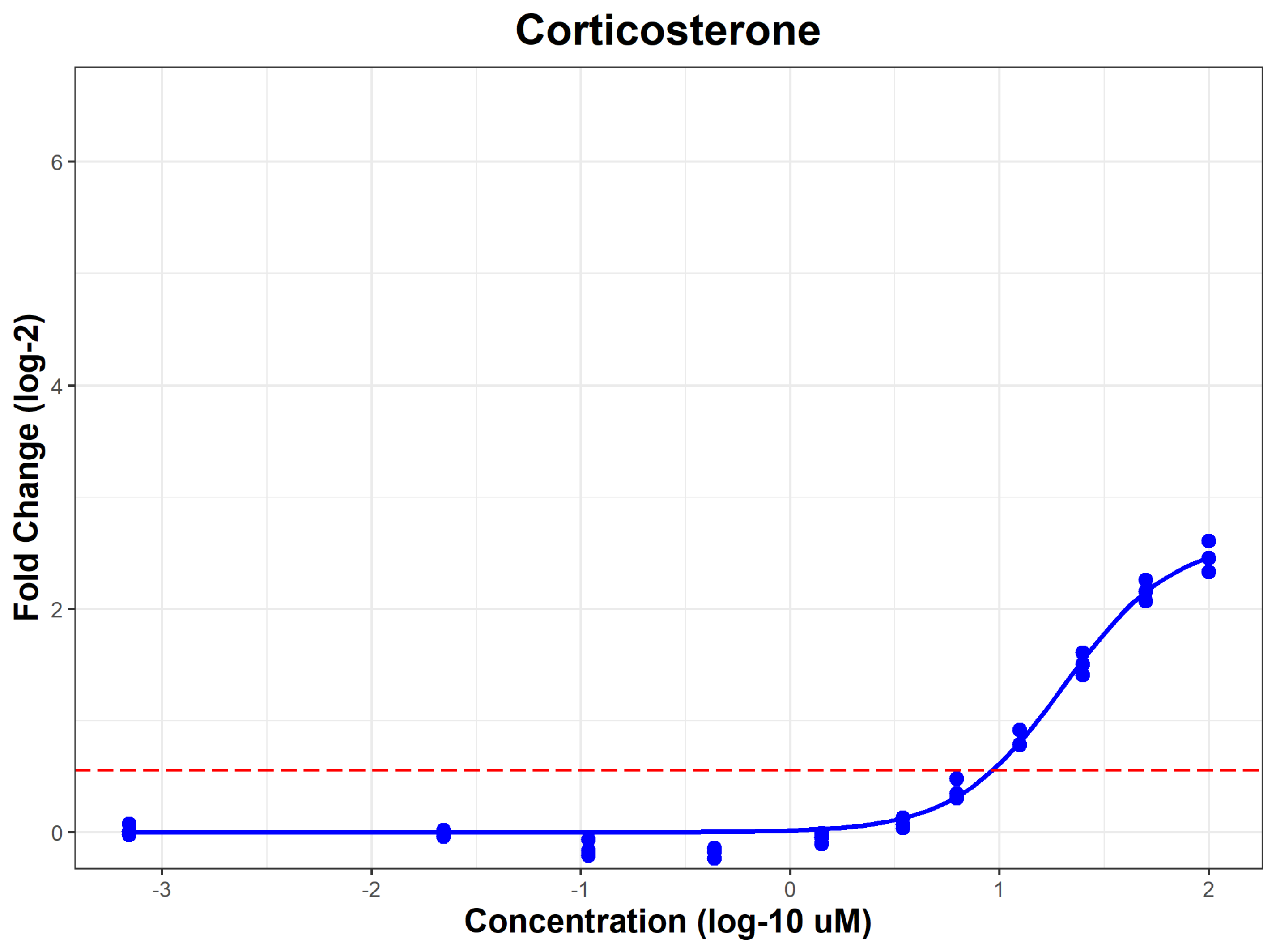

Corticosterone
Fold Change (log-2)
6
4
2
0
-3
-2
-1
0
1
2
Concentration (log-10 uM)

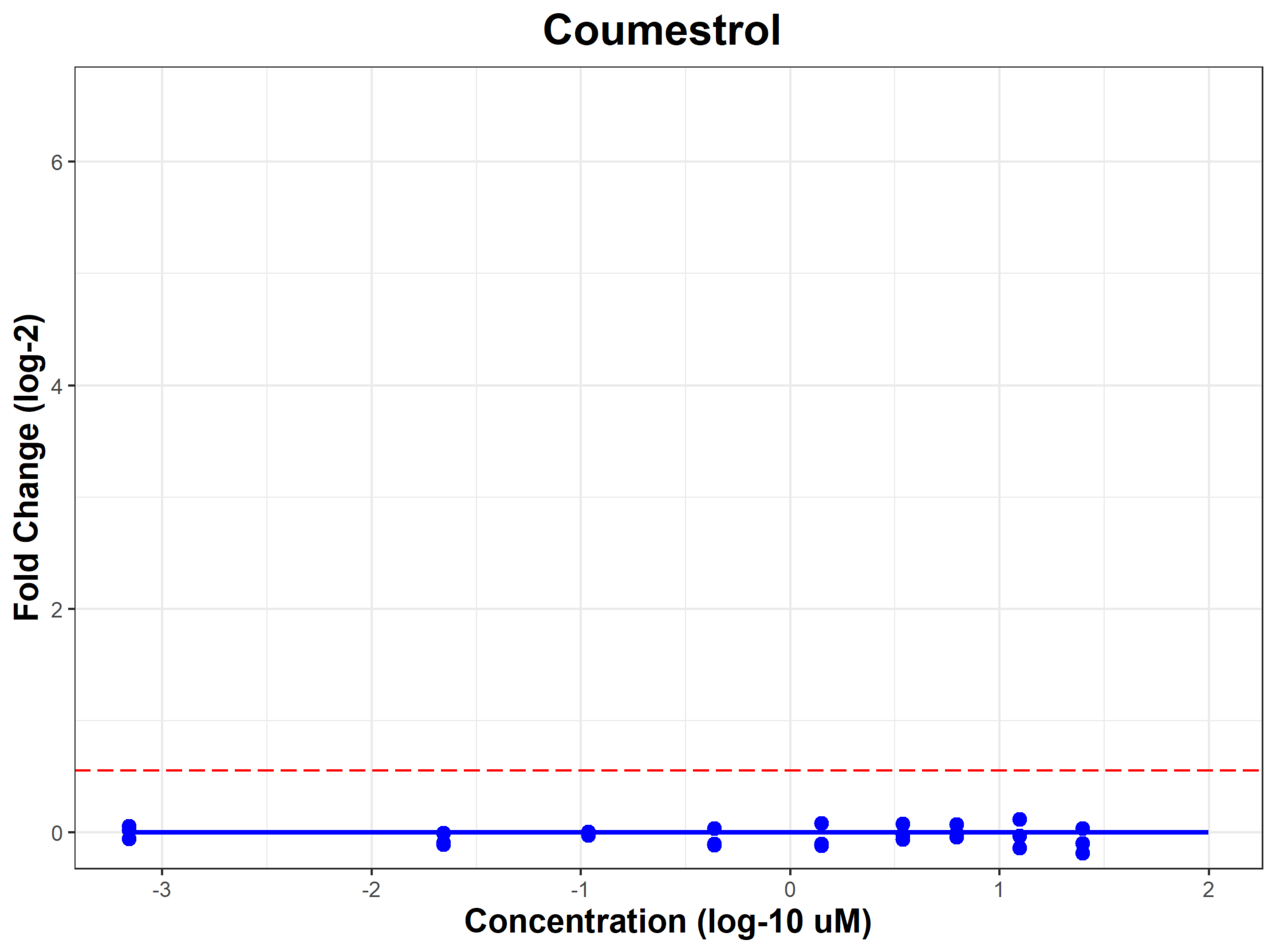
Coumestrol
Fold Change (log-2)
6
4
2
0
-3
-2
-1
0
1
2
Concentration (log-10 uM)

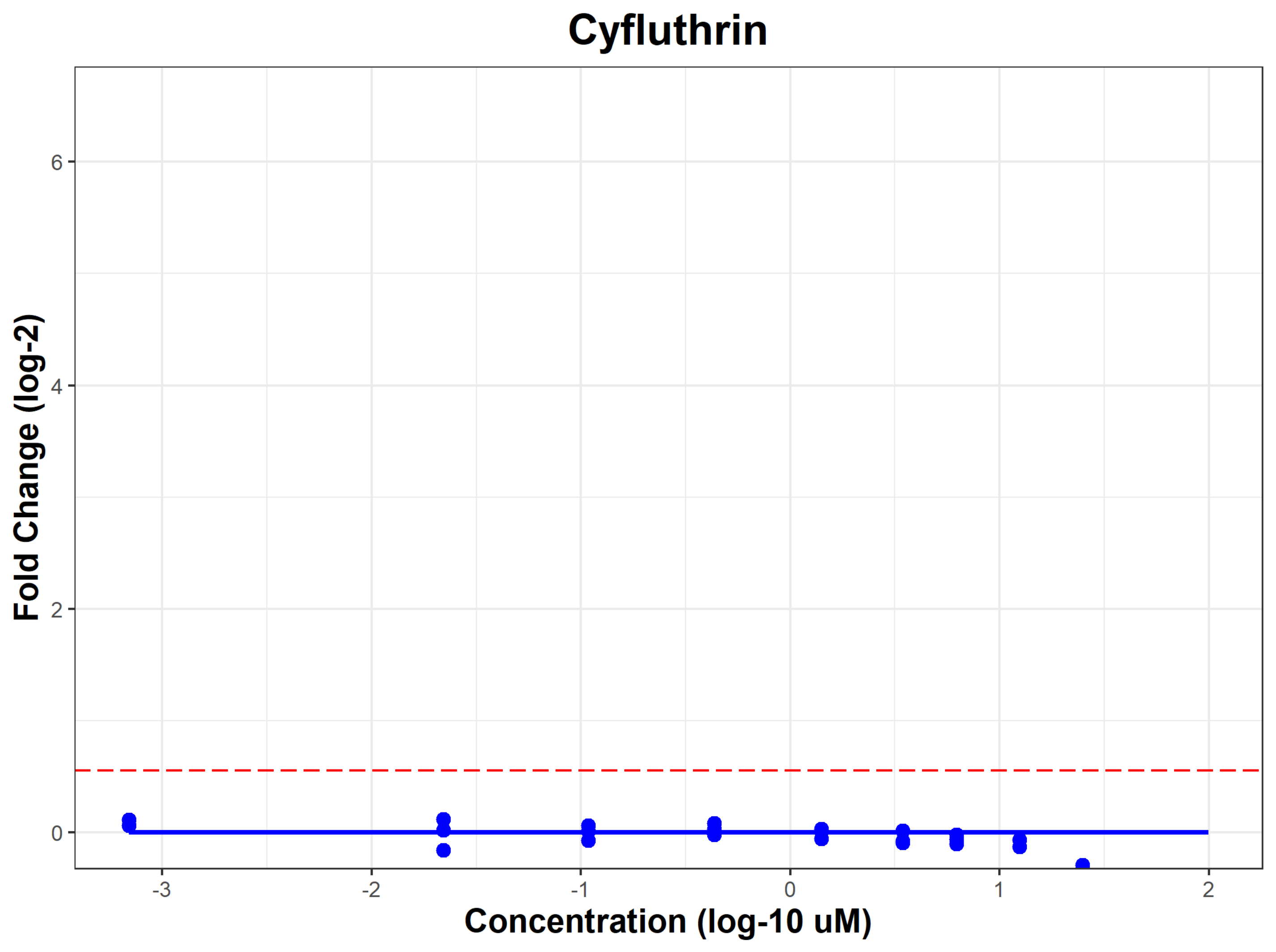

Cyfluthrin
Fold Change (log-2)
6
4
2
0
-3
-2
-1
0
1
2
Concentration (log-10 uM)

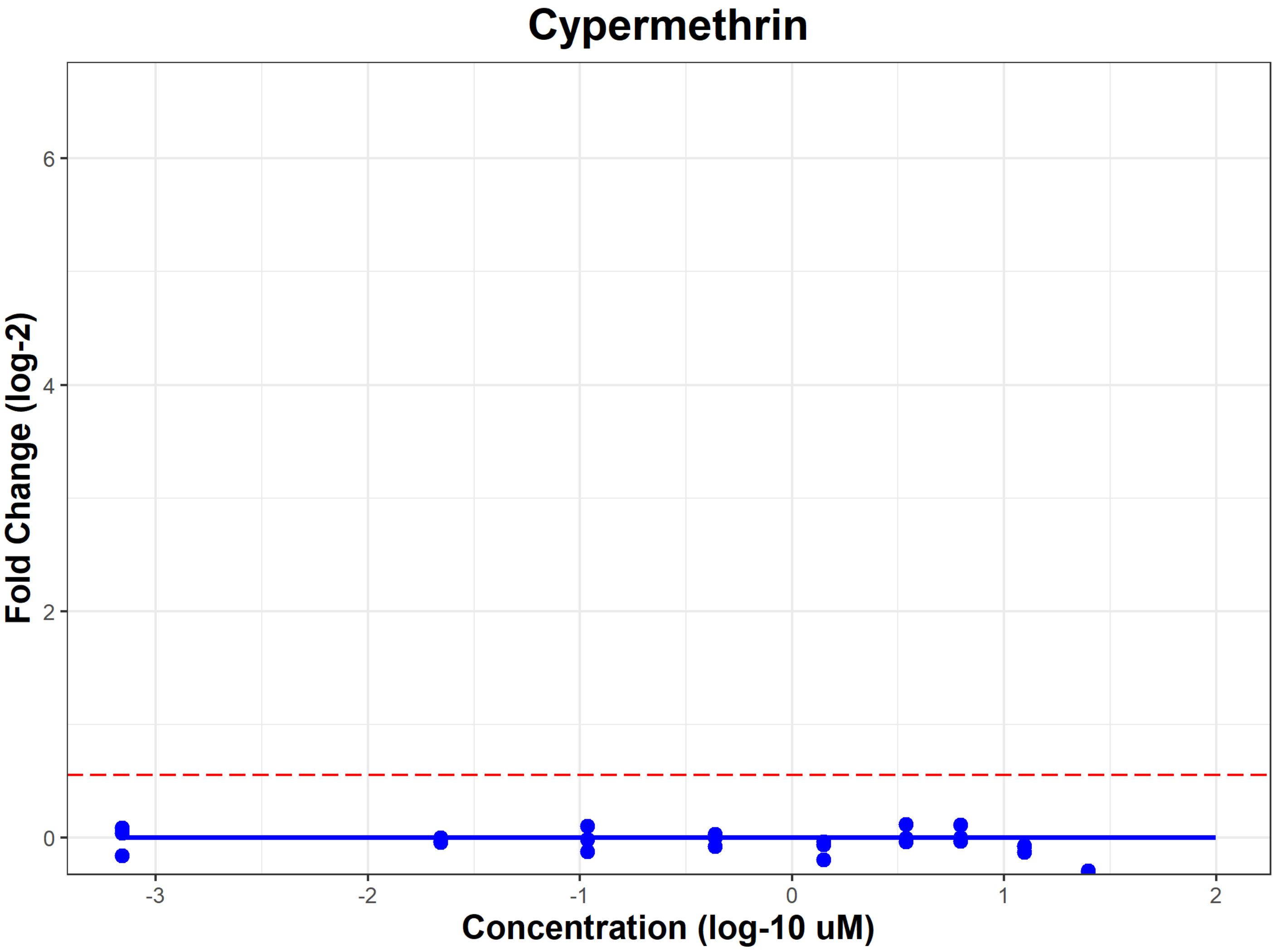

Cypermethrin
Fold Change (log-2)
6
4
2
0
-3
-2
-1
0
1
2
Concentration (log-10 uM)

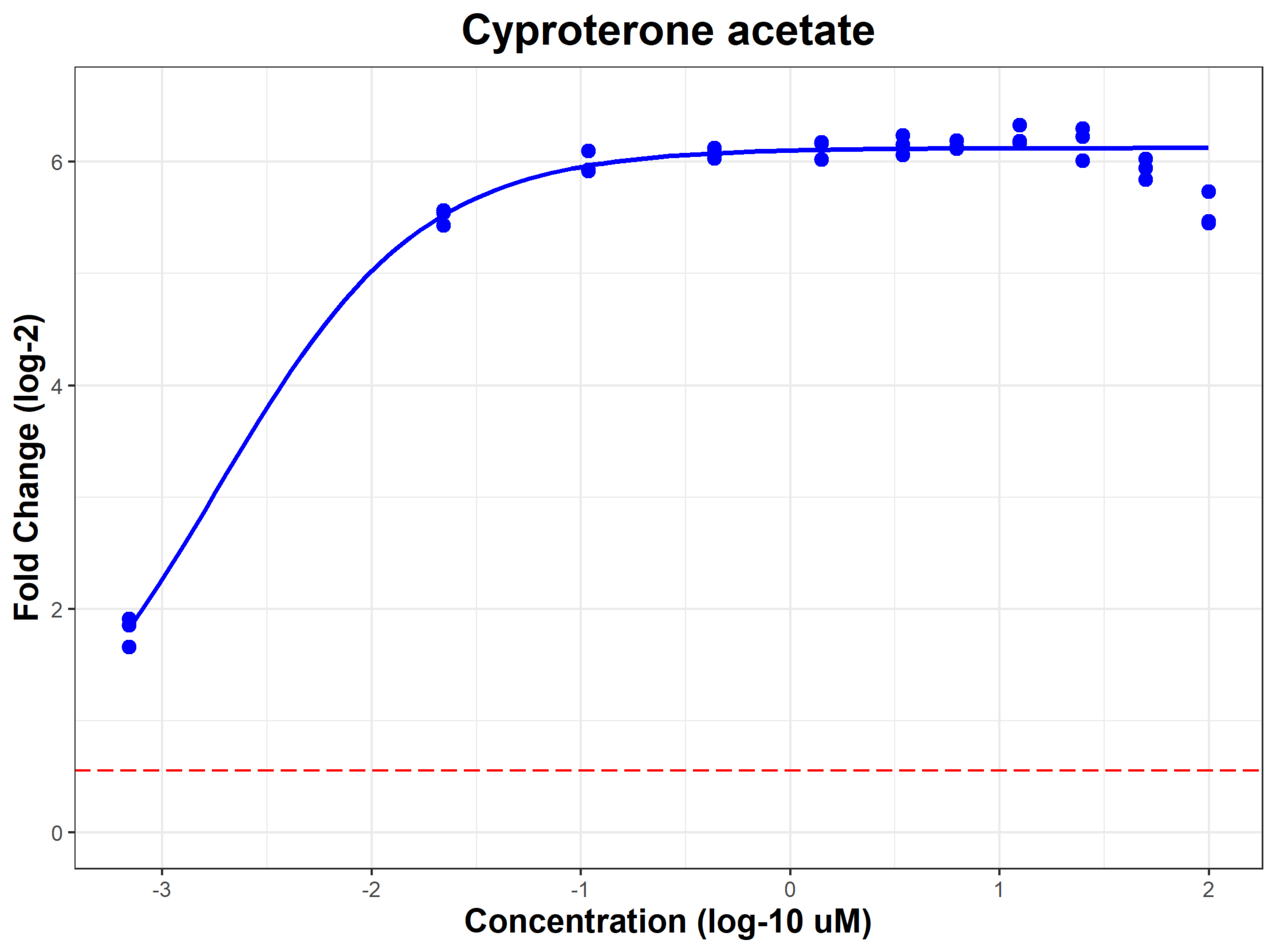

Cyproterone acetate
Fold Change (log-2)
0
2
4
6
-3
-2
-1
0
1
2
Concentration (log-10 uM)

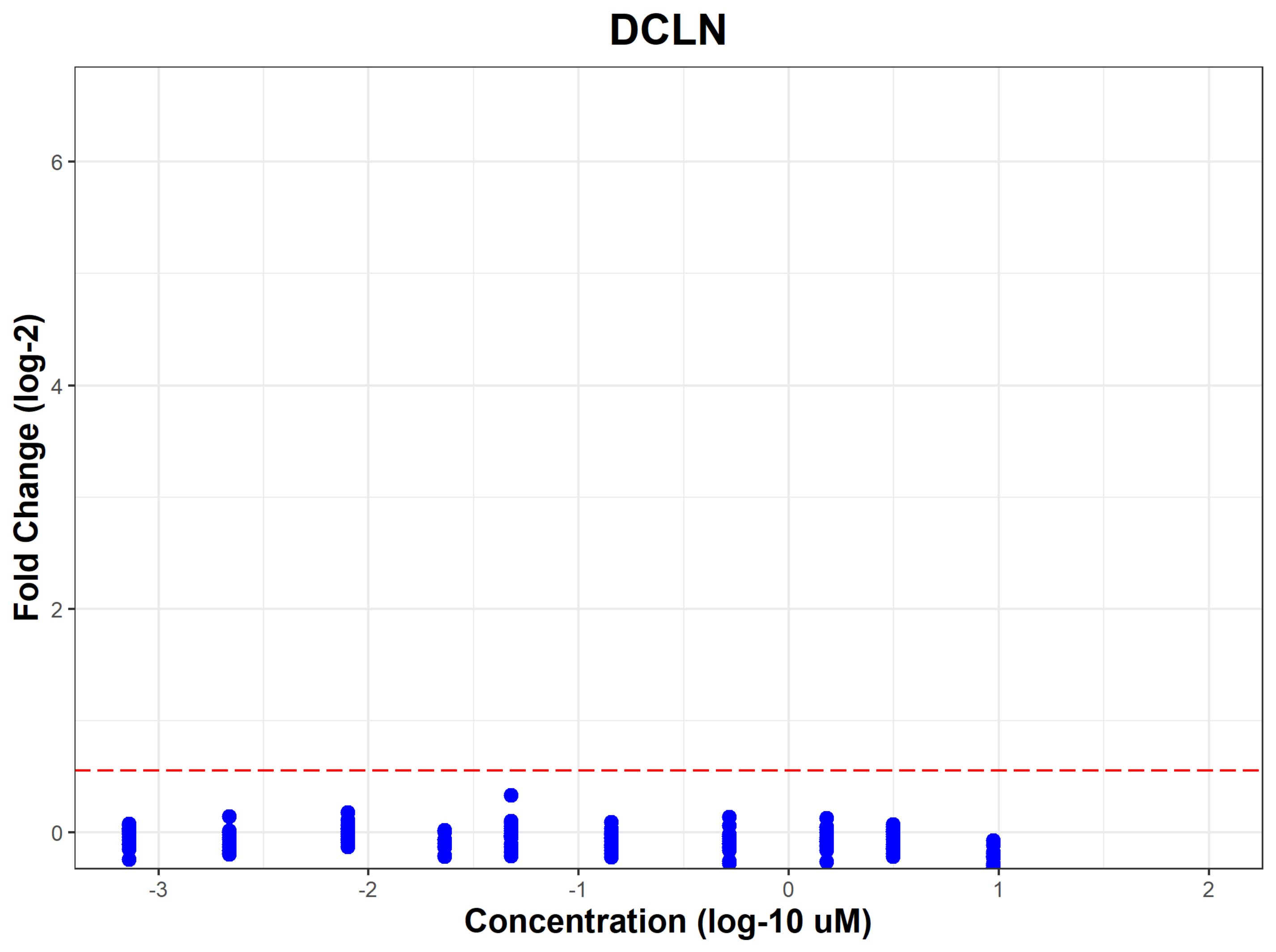
DCLN
Fold Change (log-2)
6
4
2
0
-3
-2
-1
0
1
2
Concentration (log-10 uM)

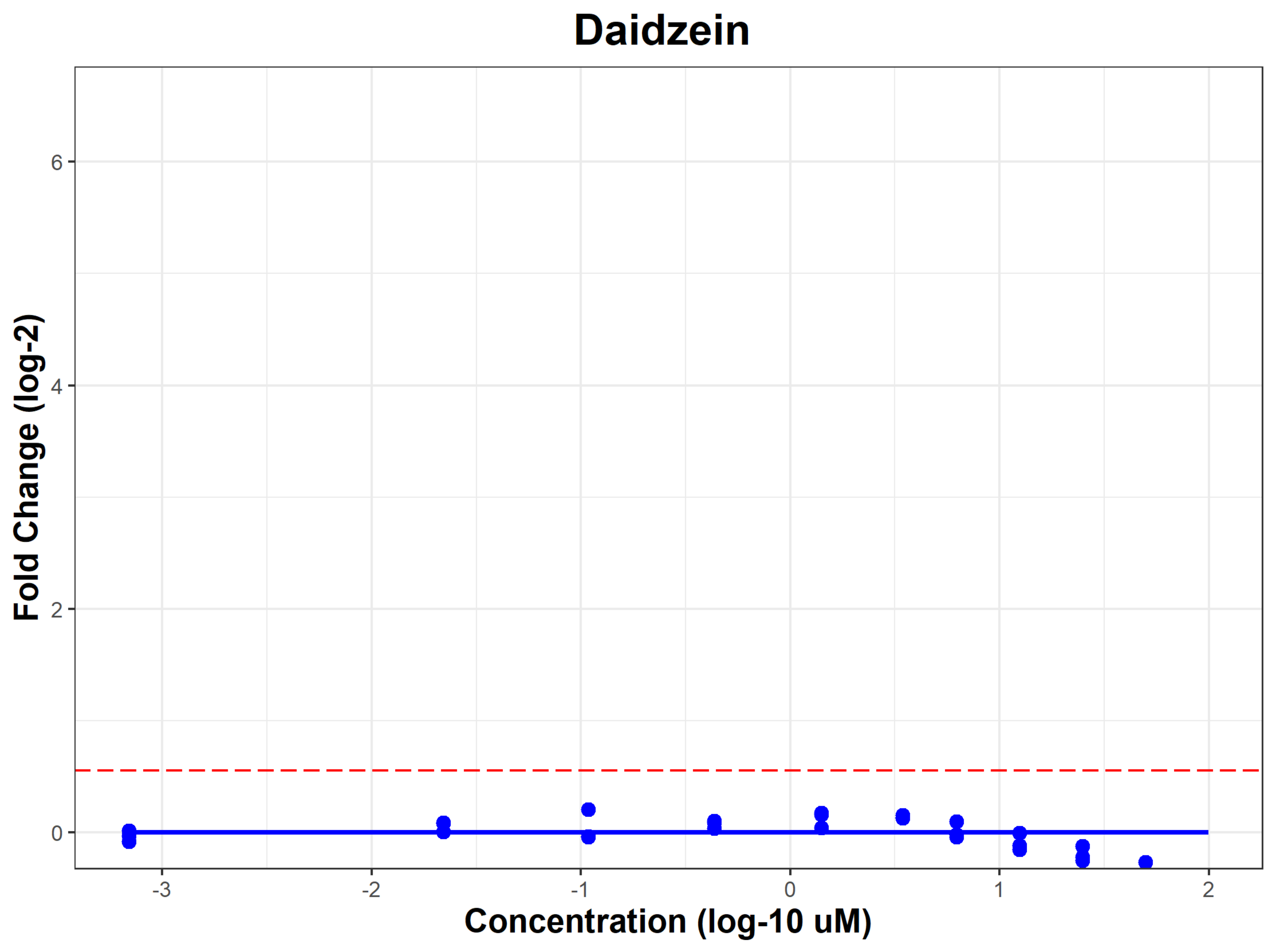

Daidzein
Fold Change (log-2)
6
4
2
0
-3
-2
-1
0
1
2
Concentration (log-10 uM)

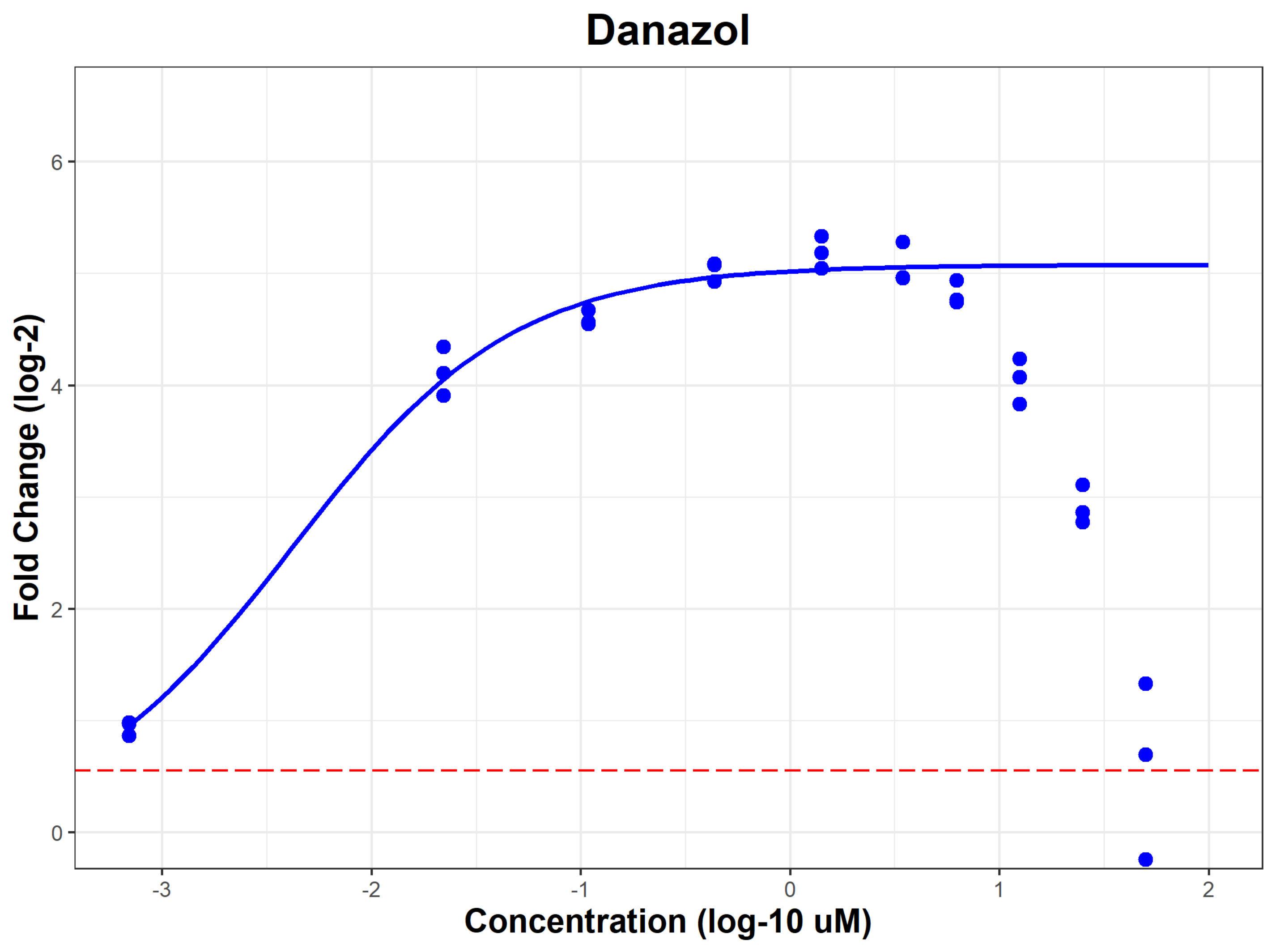

Danazol
Fold Change (log-2)
6
4
2
0
-3
-2
-1
0
1
2
Concentration (log-10 uM)

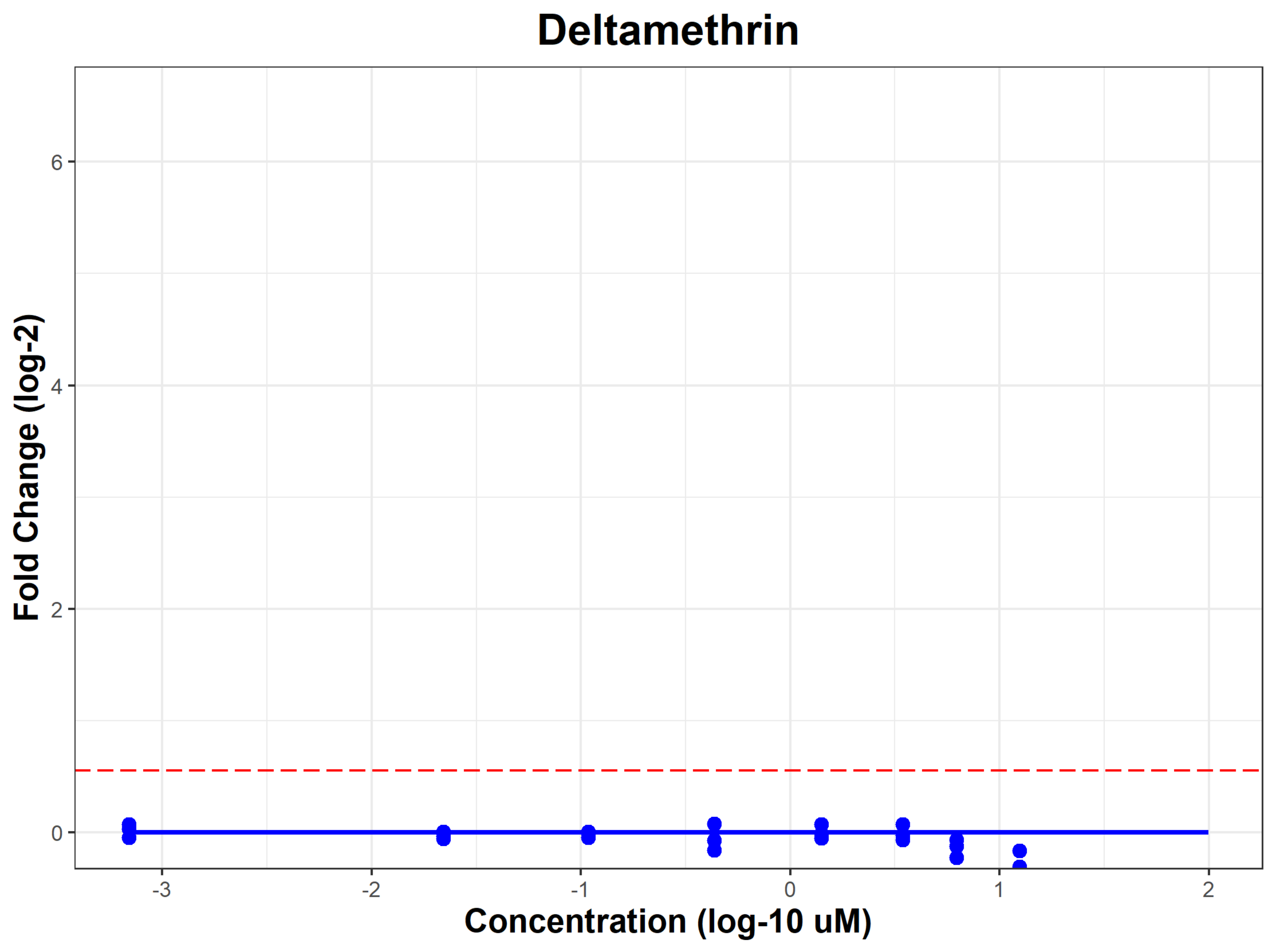

Deltamethrin
Fold Change (log-2)
6
4
2
0
-3
-2
-1
0
1
2
Concentration (log-10 uM)

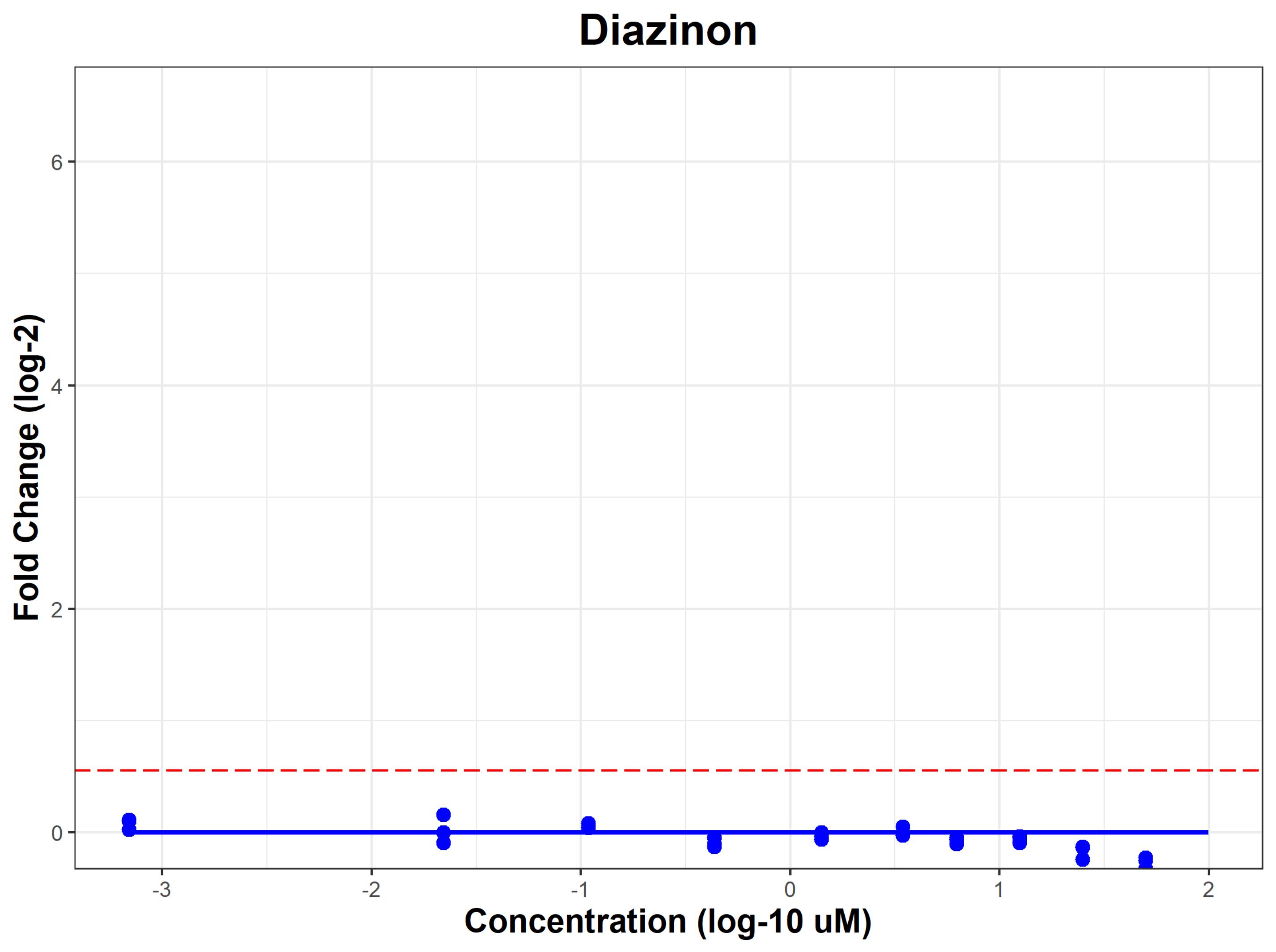

Diazinon
Fold Change (log-2)
6
4
2
0
-3
-2
-1
0
1
2
Concentration (log-10 uM)

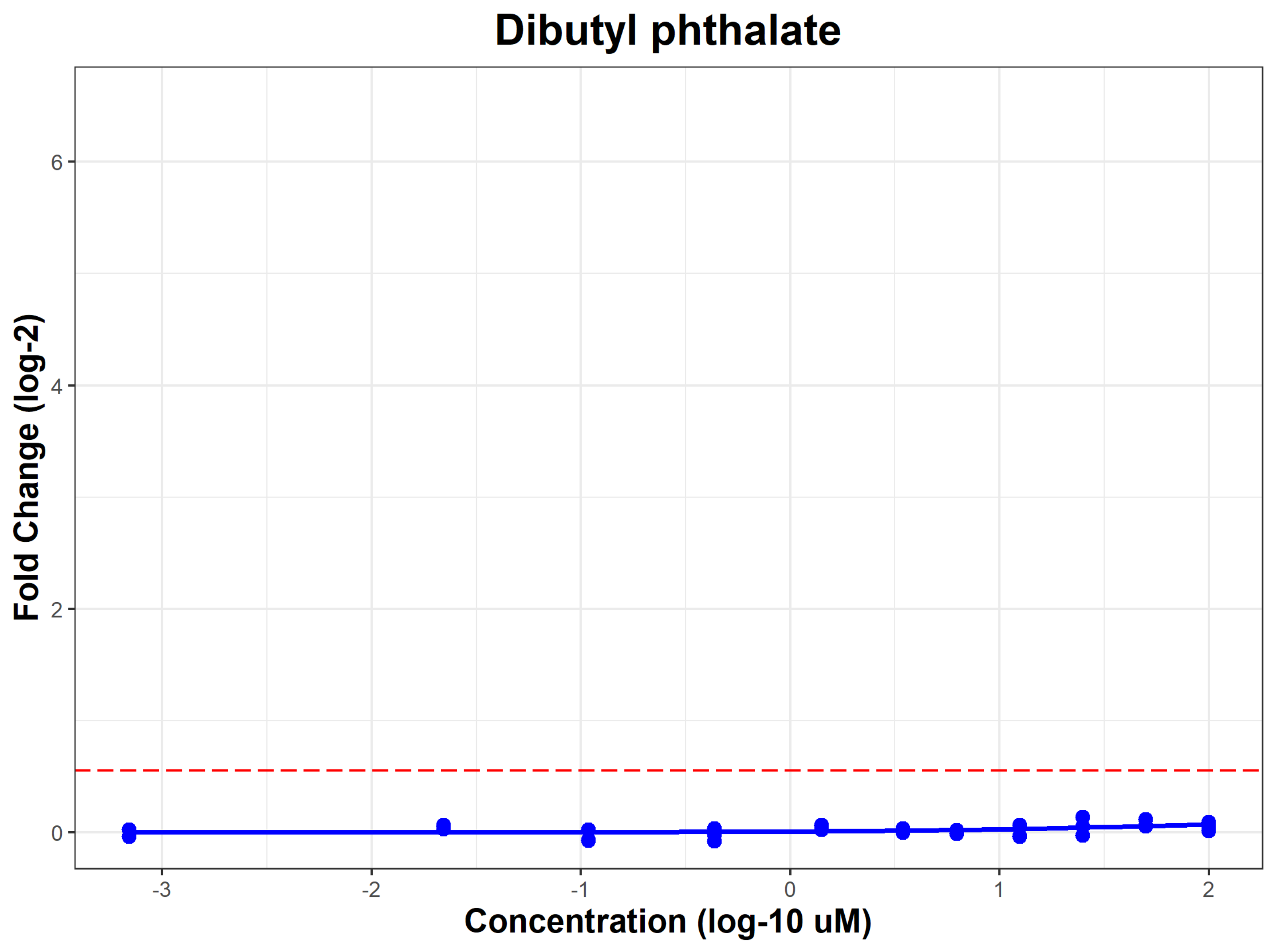
Dibutyl phthalate
Fold Change (log-2)
6
4
2
0
-3
-2
-1
0
1
2
Concentration (log-10 uM)

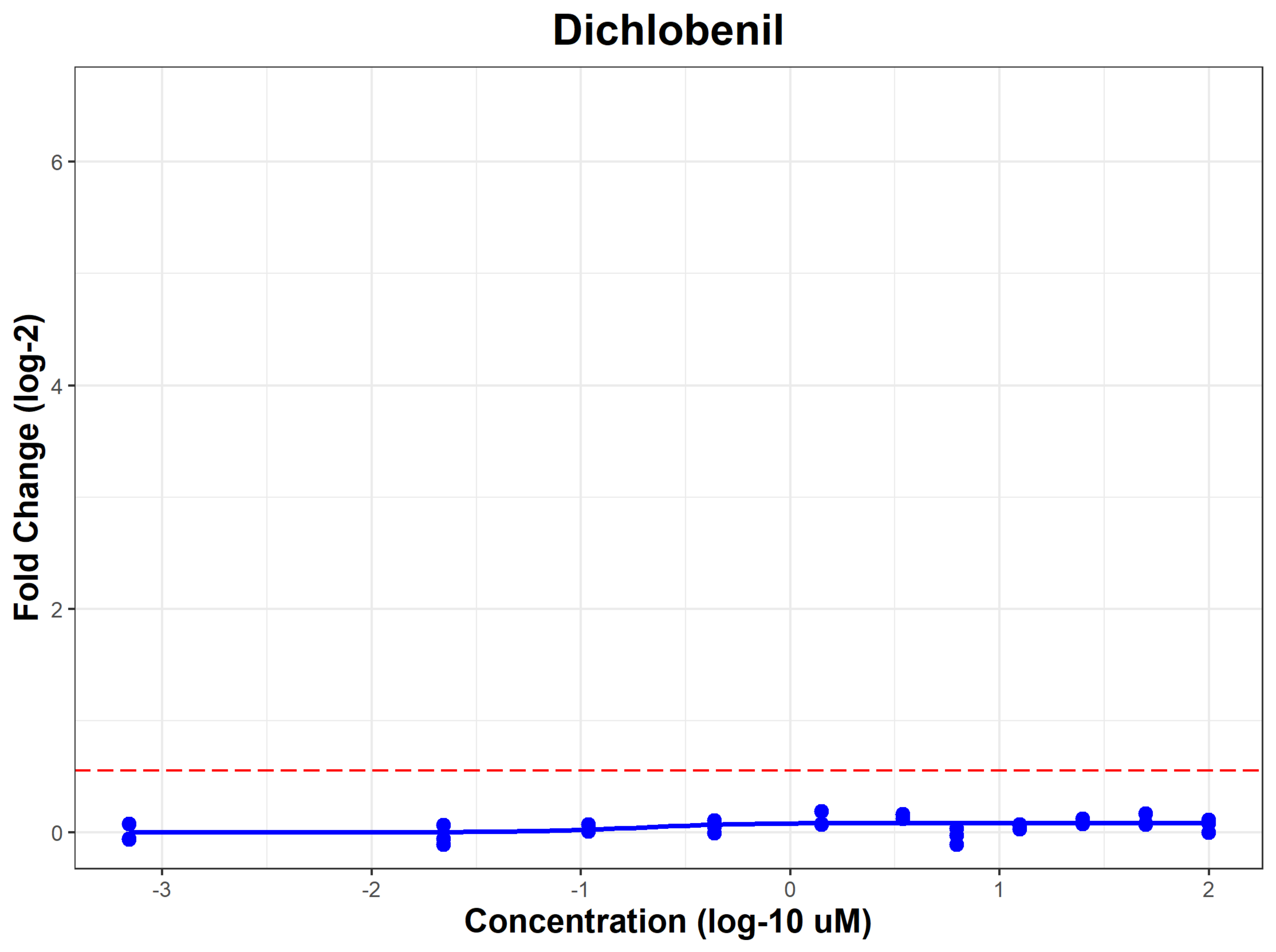
Dichlobenil
Fold Change (log-2)
Concentration (log-10 uM)
6
4
2
0
-3
-2
-1
0
1
2

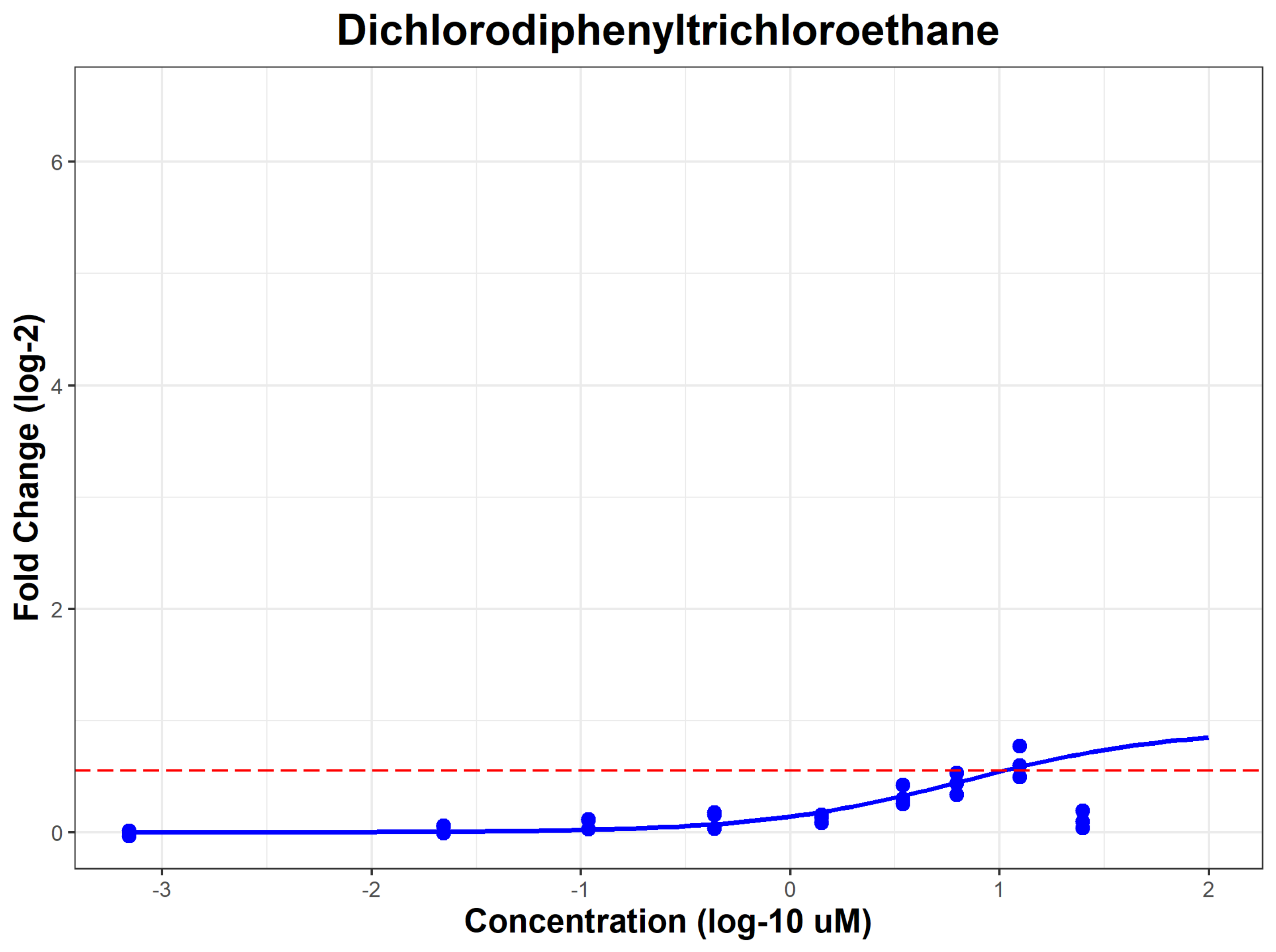

Dichlorodiphenyltrichloroethane
Fold Change (log-2)
6
4
2
0
-3
-2
-1
0
1
2
Concentration (log-10 uM)

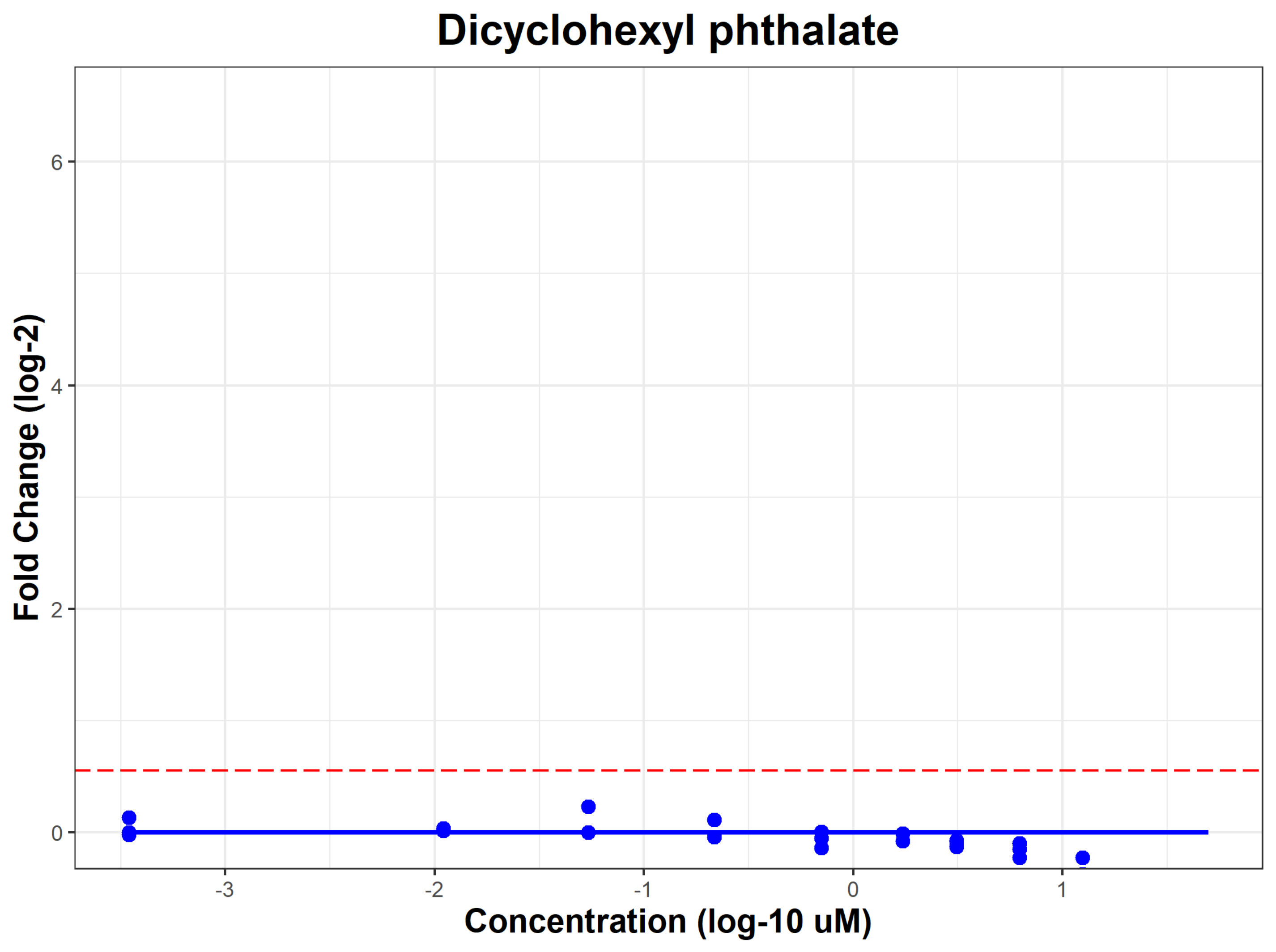
Dicyclohexyl phthalate
Fold Change (log-2)
6
4
2
0
-3
-2
-1
0
1
Concentration (log-10 uM)

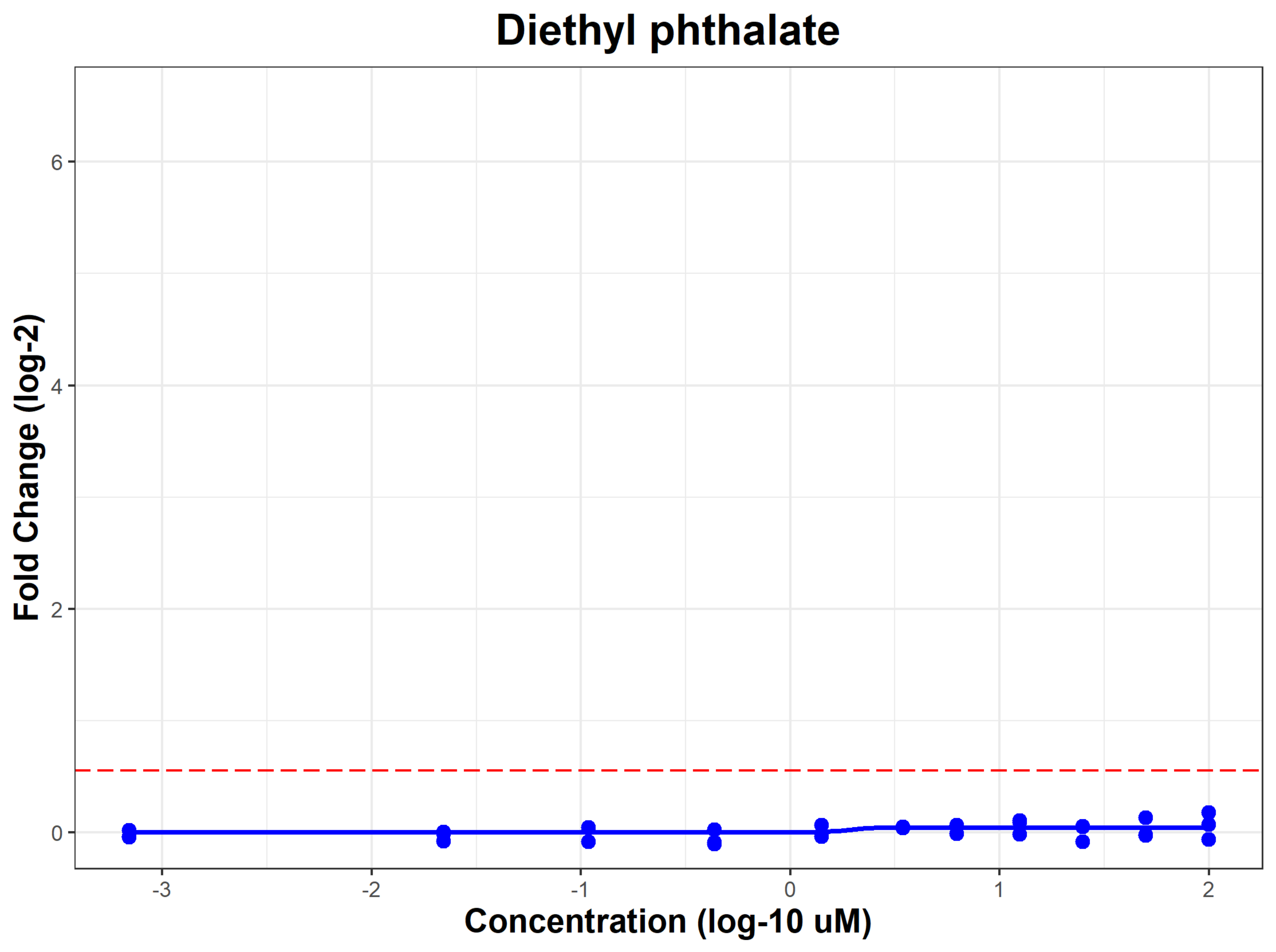

Diethyl phthalate
Fold Change (log-2)
Concentration (log-10 uM)
6
4
2
0
-3
-2
-1
0
1
2

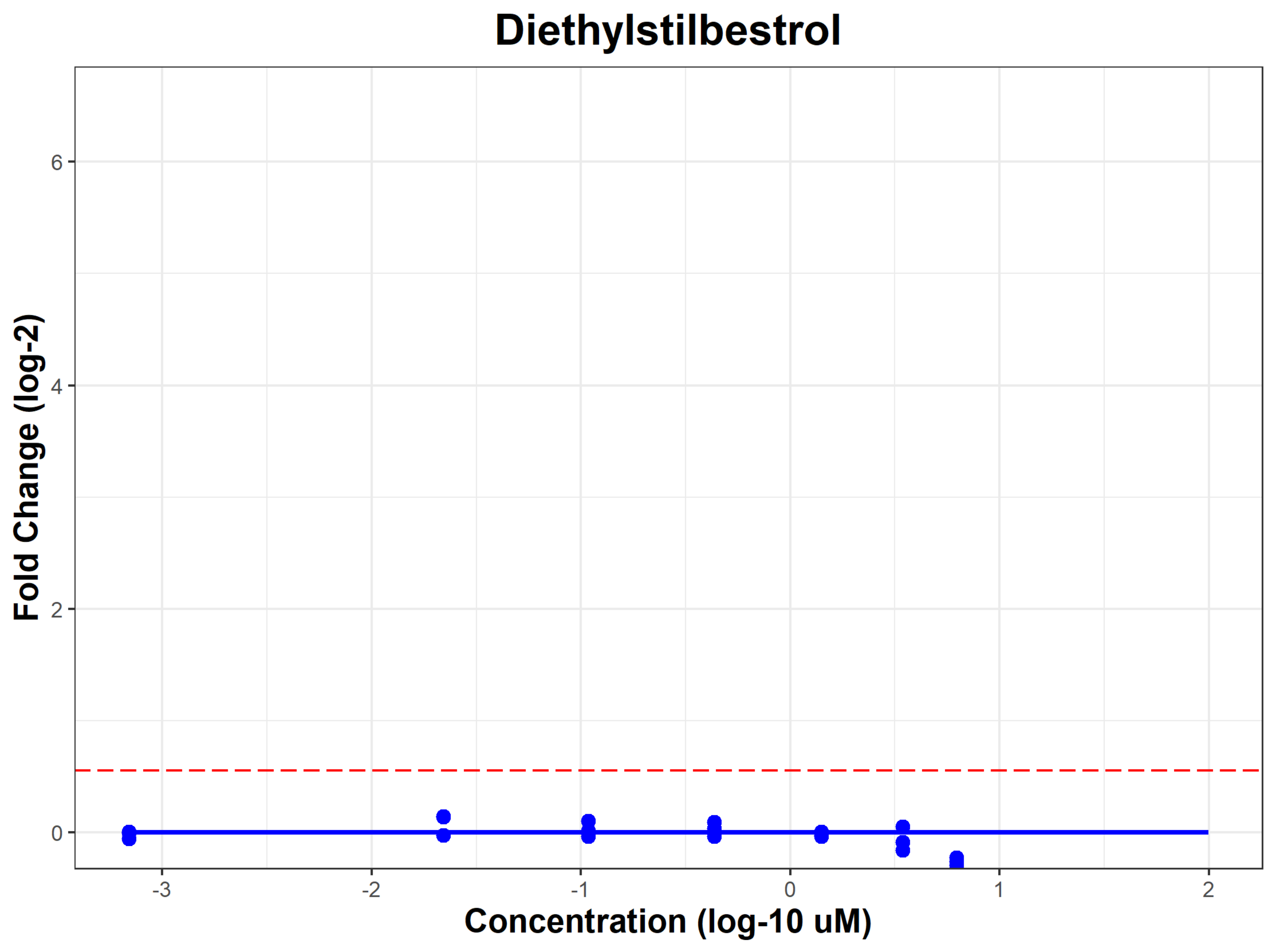

Diethylstilbestrol
Fold Change (log-2)
6
4
2
0
-3
-2
-1
0
1
2
Concentration (log-10 uM)

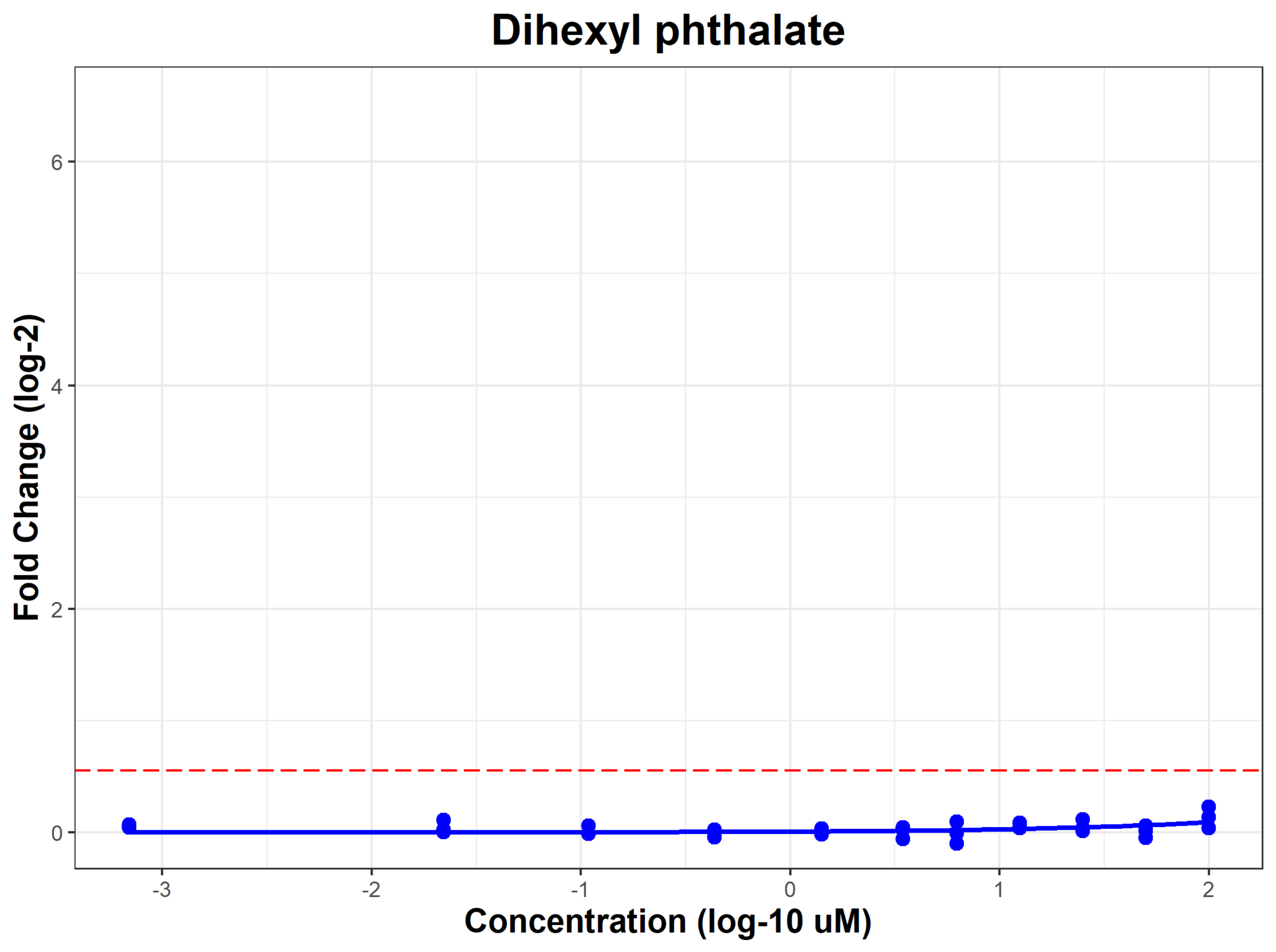

Dihexyl phthalate
Fold Change (log-2)
6
4
2
0
-3
-2
-1
0
1
2
Concentration (log-10 uM)

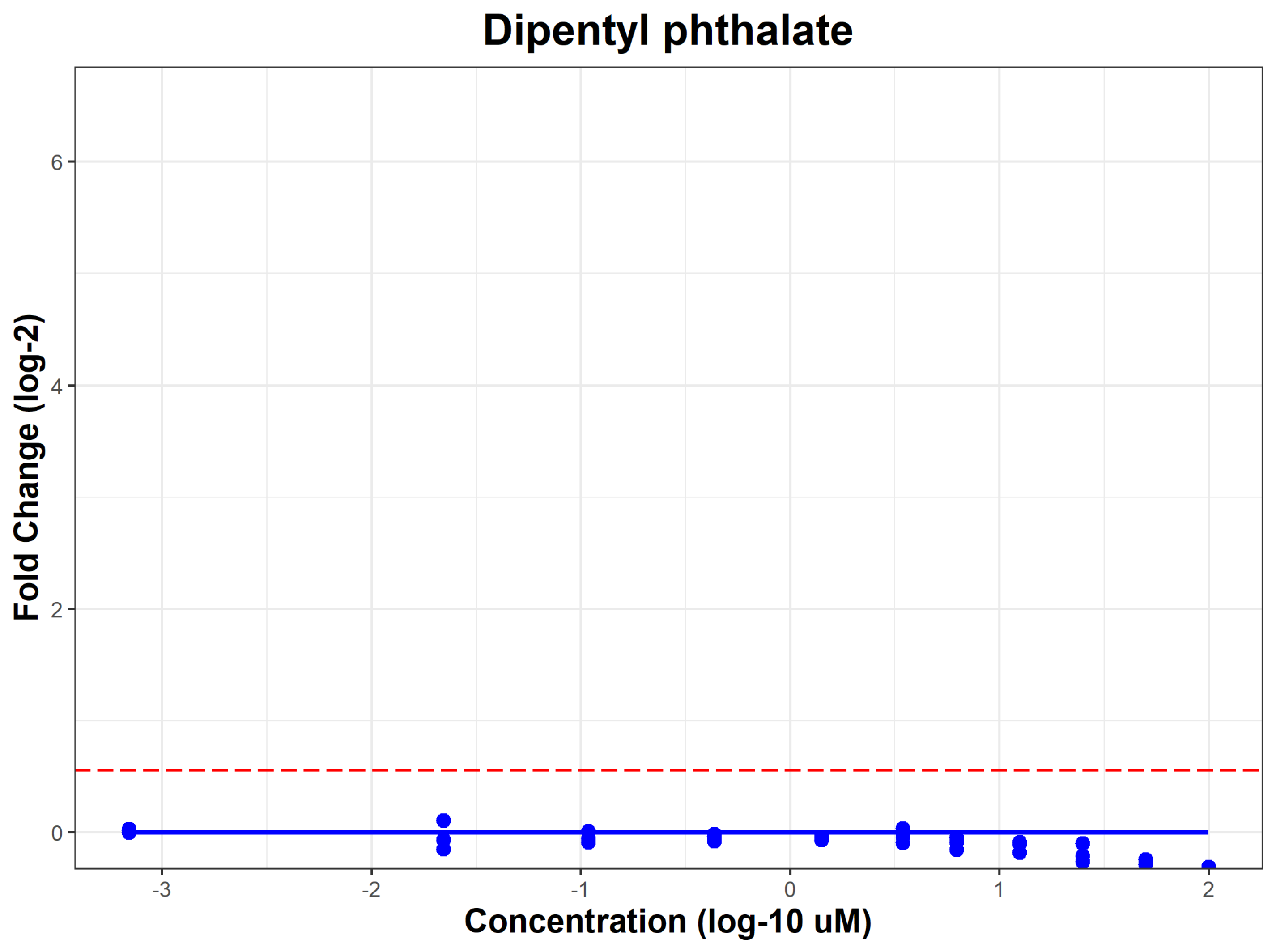
Dipentyl phthalate
Fold Change (log-2)
6
4
2
0
-3
-2
-1
0
1
2
Concentration (log-10 uM)

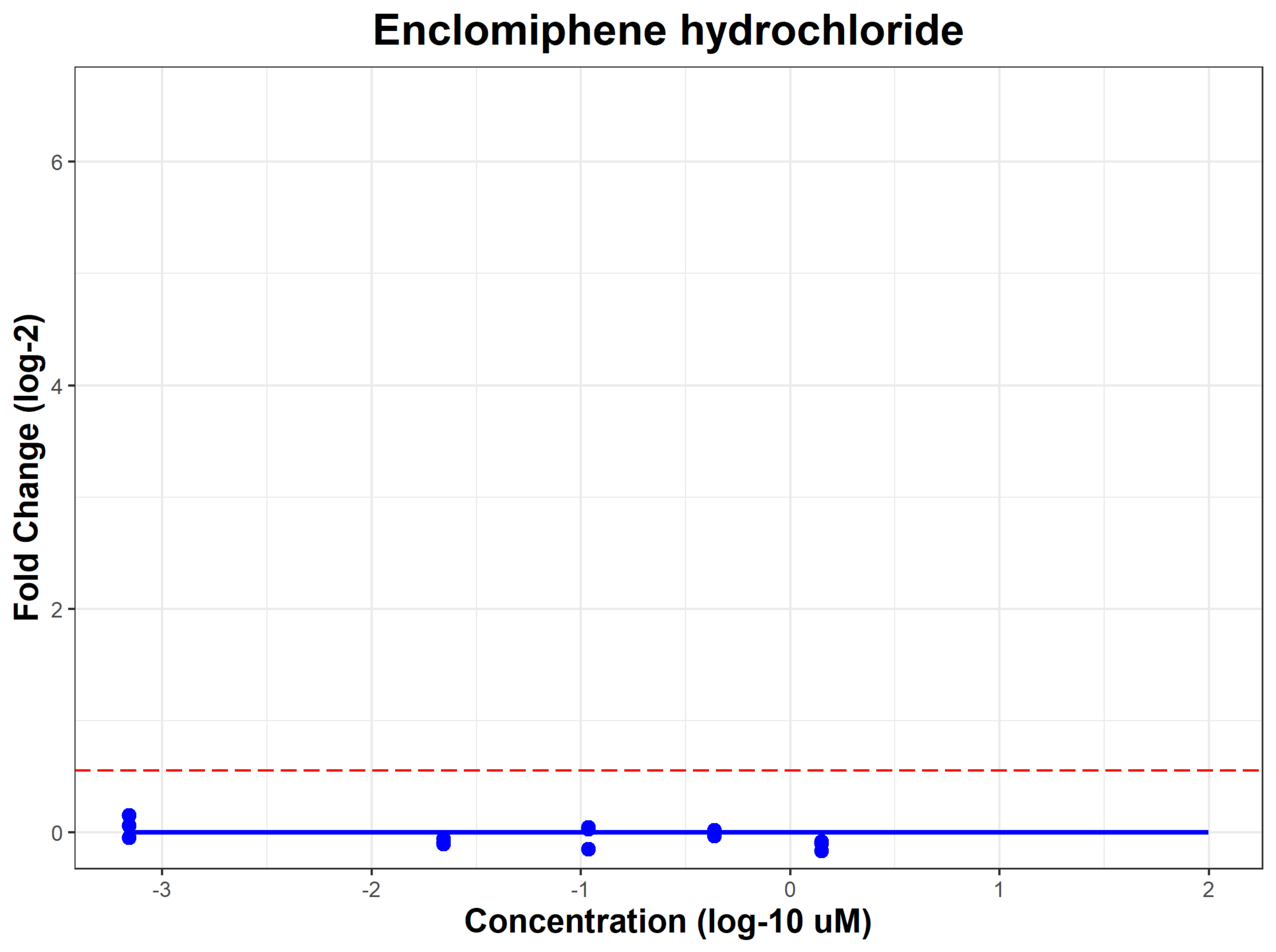
Enclomiphene hydrochloride
Fold Change (log-2)
6
4
2
0
-3
-2
-1
0
1
2
Concentration (log-10 uM)

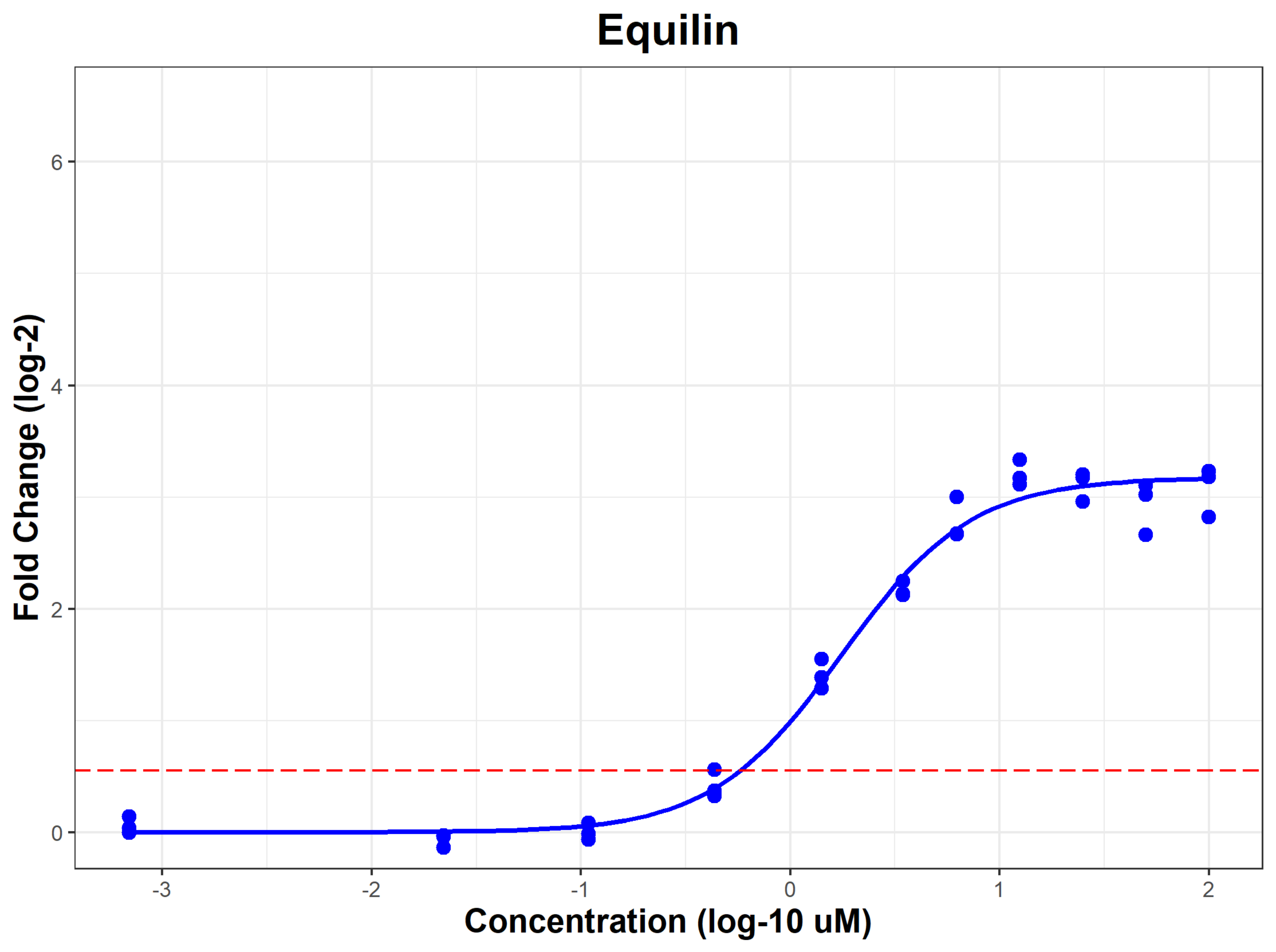
Equilin
Fold Change (log-2)
Concentration (log-10 uM)
0
2
4
6
-3
-2
-1
0
1
2

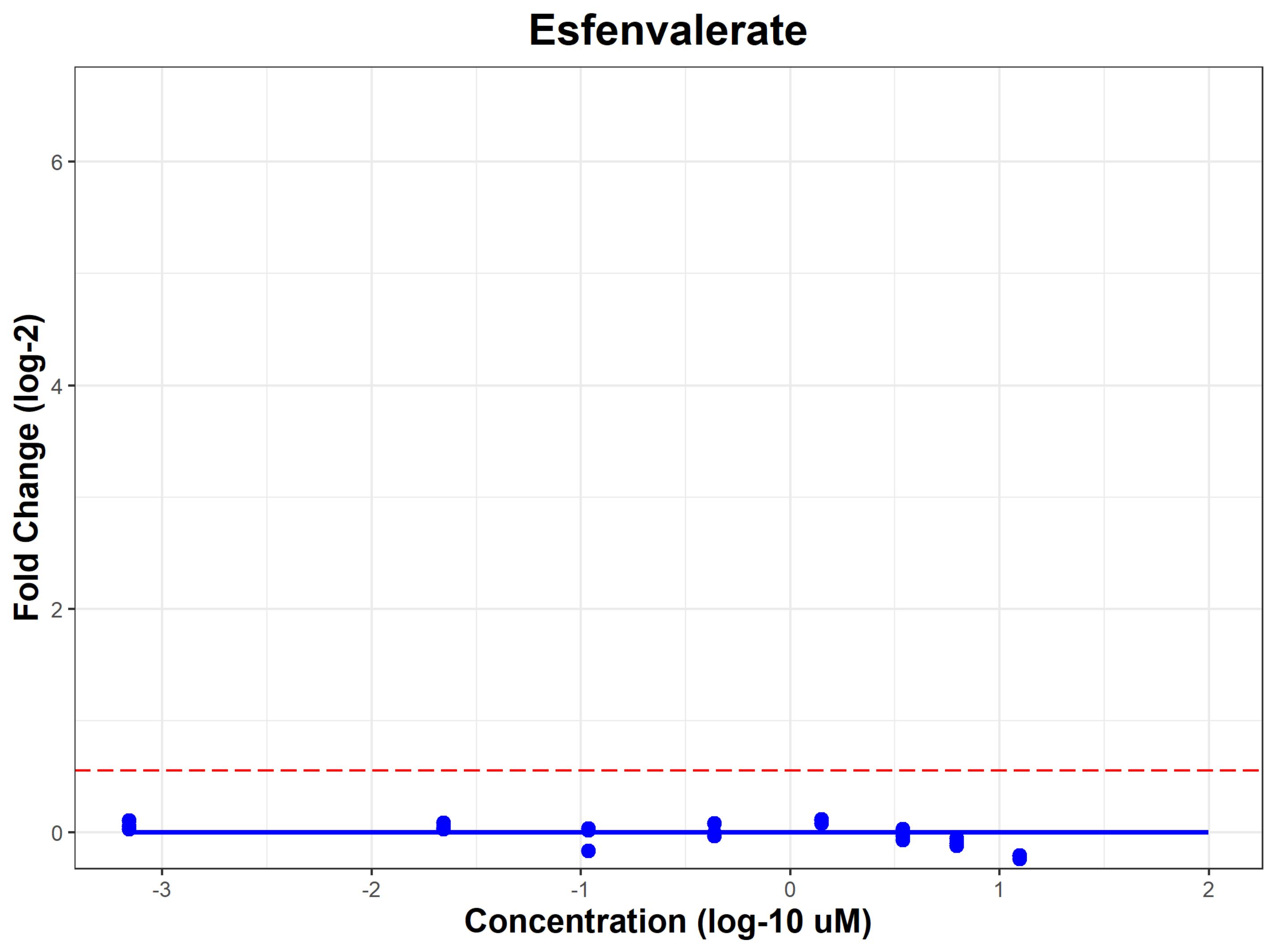
Esfenvalerate
Fold Change (log-2)
6
4
2
0
-3
-2
-1
0
1
2
Concentration (log-10 uM)

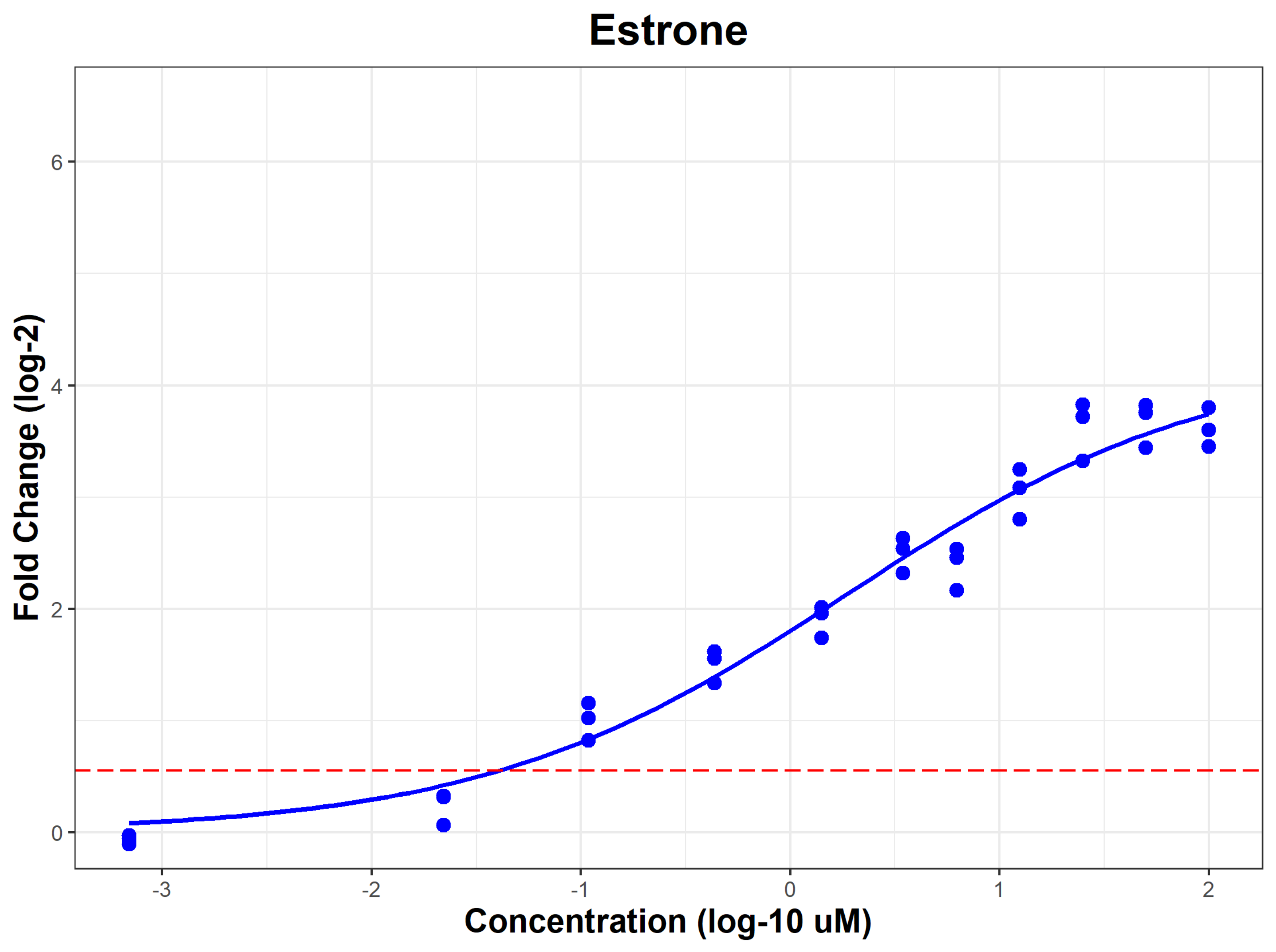
Estrone
Fold Change (log-2)
0
2
4
6
-3
-2
-1
0
1
2
Concentration (log-10 uM)

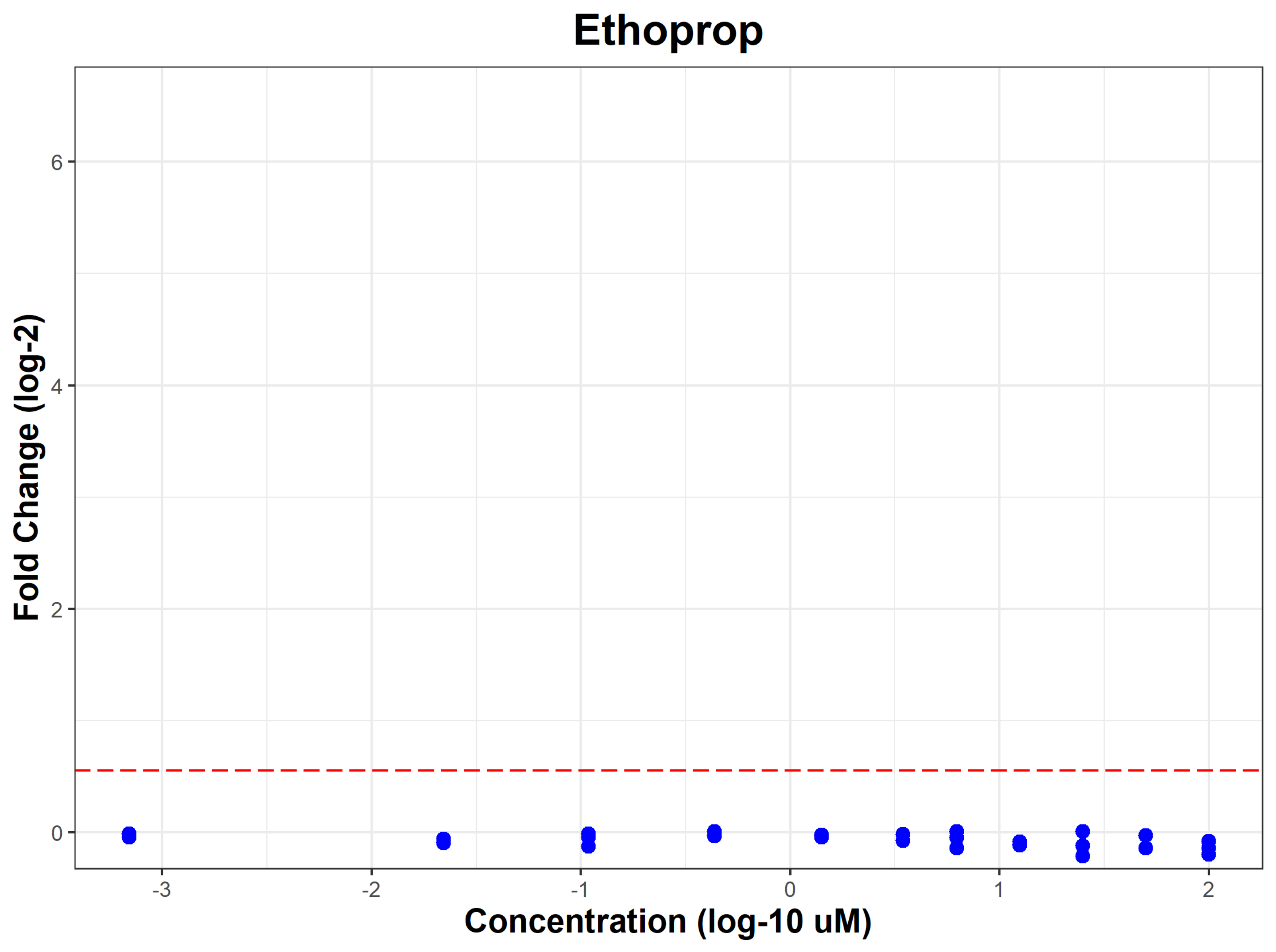

Ethoprop
Fold Change (log-2)
6
4
2
0
-3
-2
-1
0
1
2
Concentration (log-10 uM)

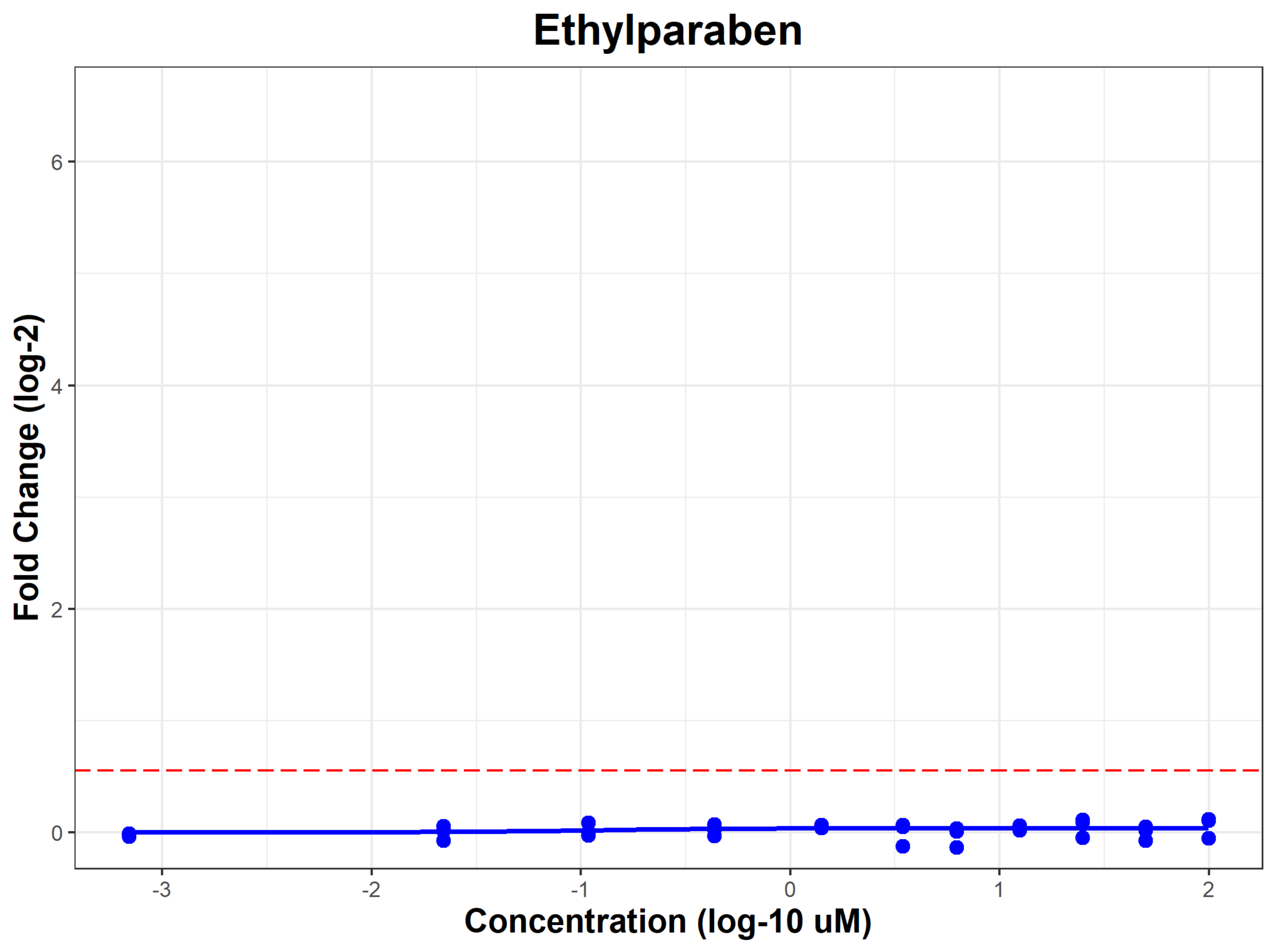
Ethylparaben
Fold Change (log-2)
6
4
2
0
-3
-2
-1
0
1
2
Concentration (log-10 uM)

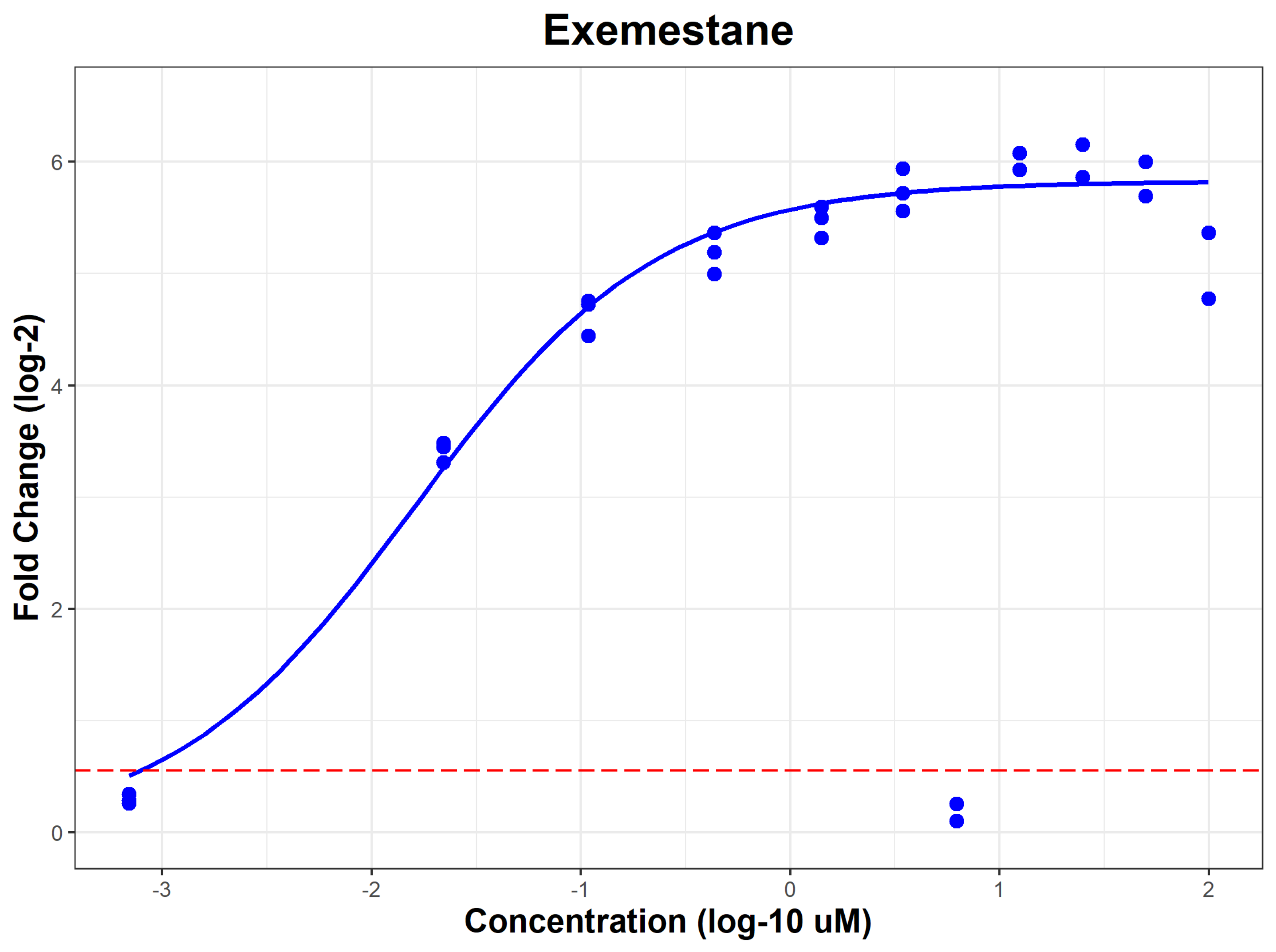
Exemestane
Fold Change (log-2)
0
2
4
6
-3
-2
-1
0
1
2
Concentration (log-10 uM)

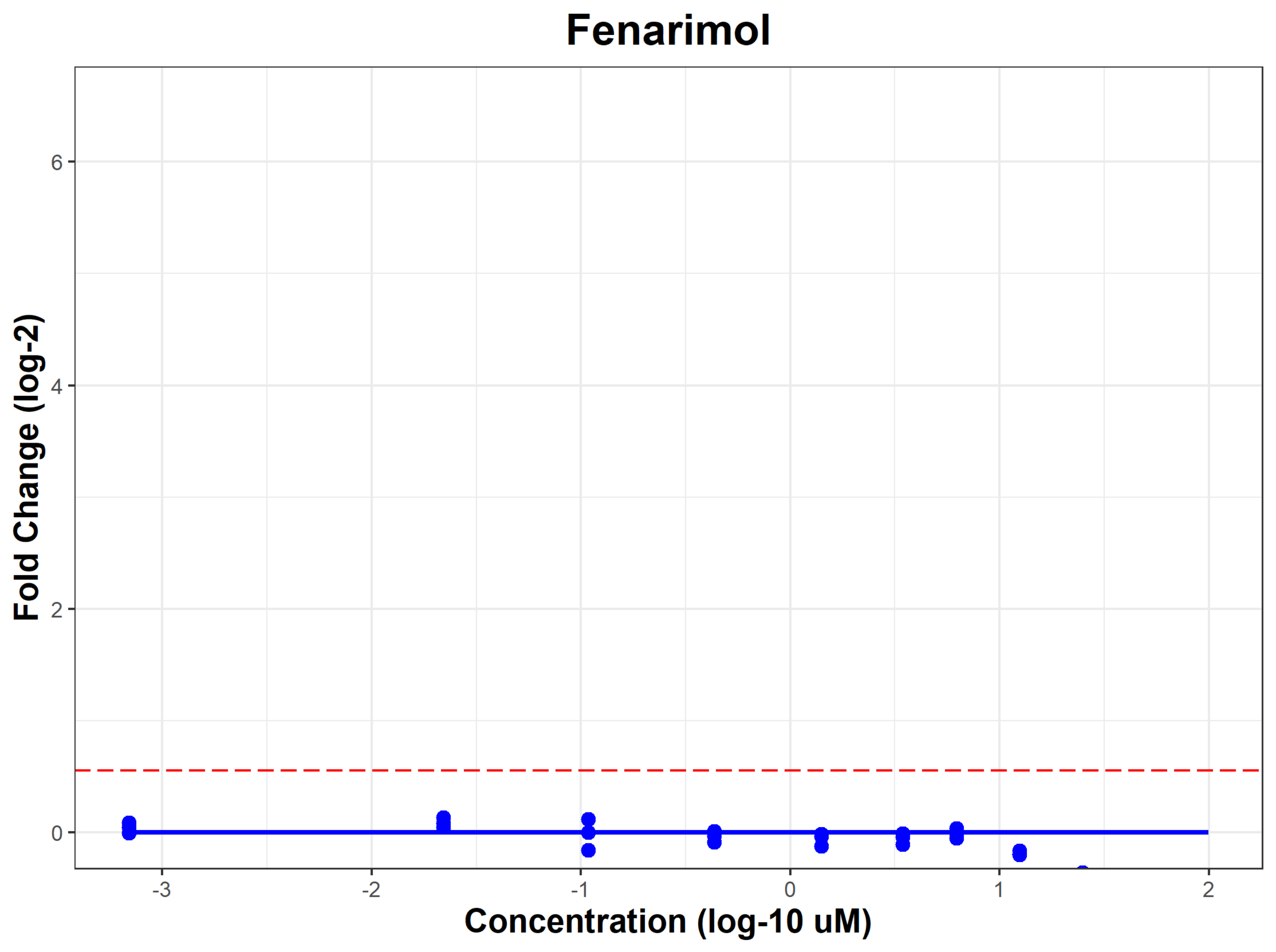

Fenarimol
Fold Change (log-2)
6
4
2
0
-3
-2
-1
0
1
2
Concentration (log-10 uM)

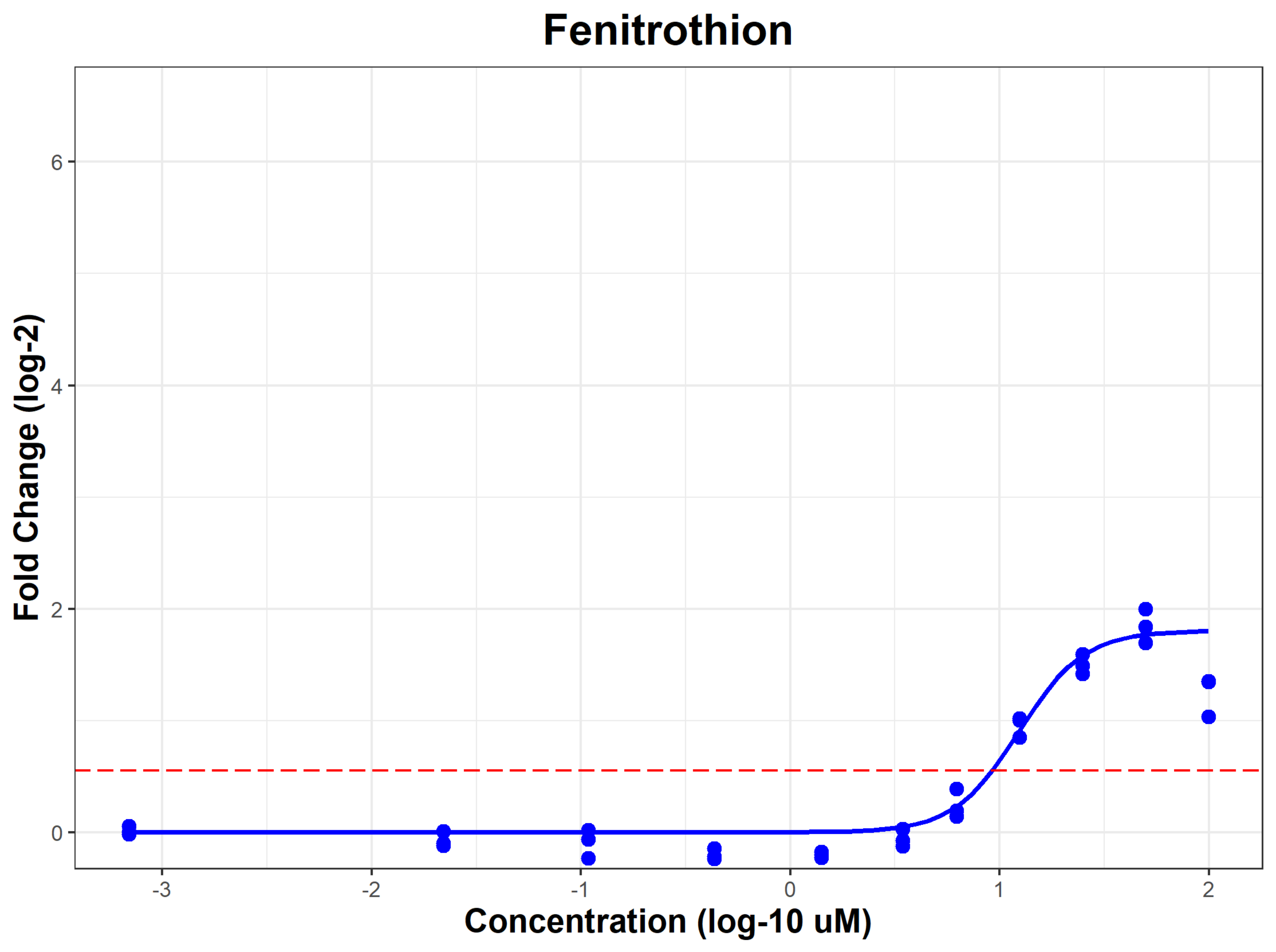

Fenitrothion
Fold Change (log-2)
6
4
2
0
-3
-2
-1
0
1
2
Concentration (log-10 uM)

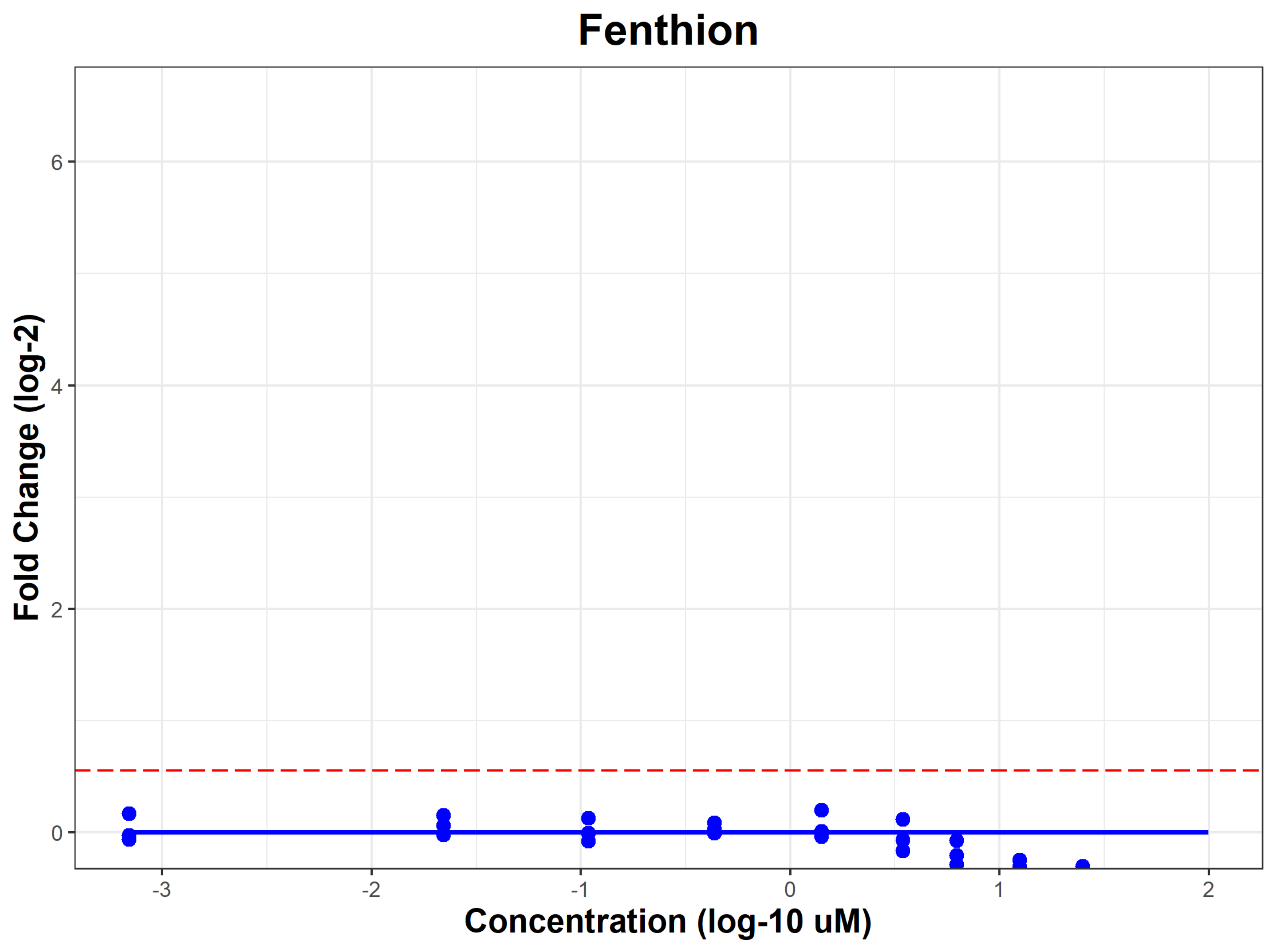

Fenthion
Fold Change (log-2)
6
4
2
0
-3
-2
-1
0
1
2
Concentration (log-10 uM)

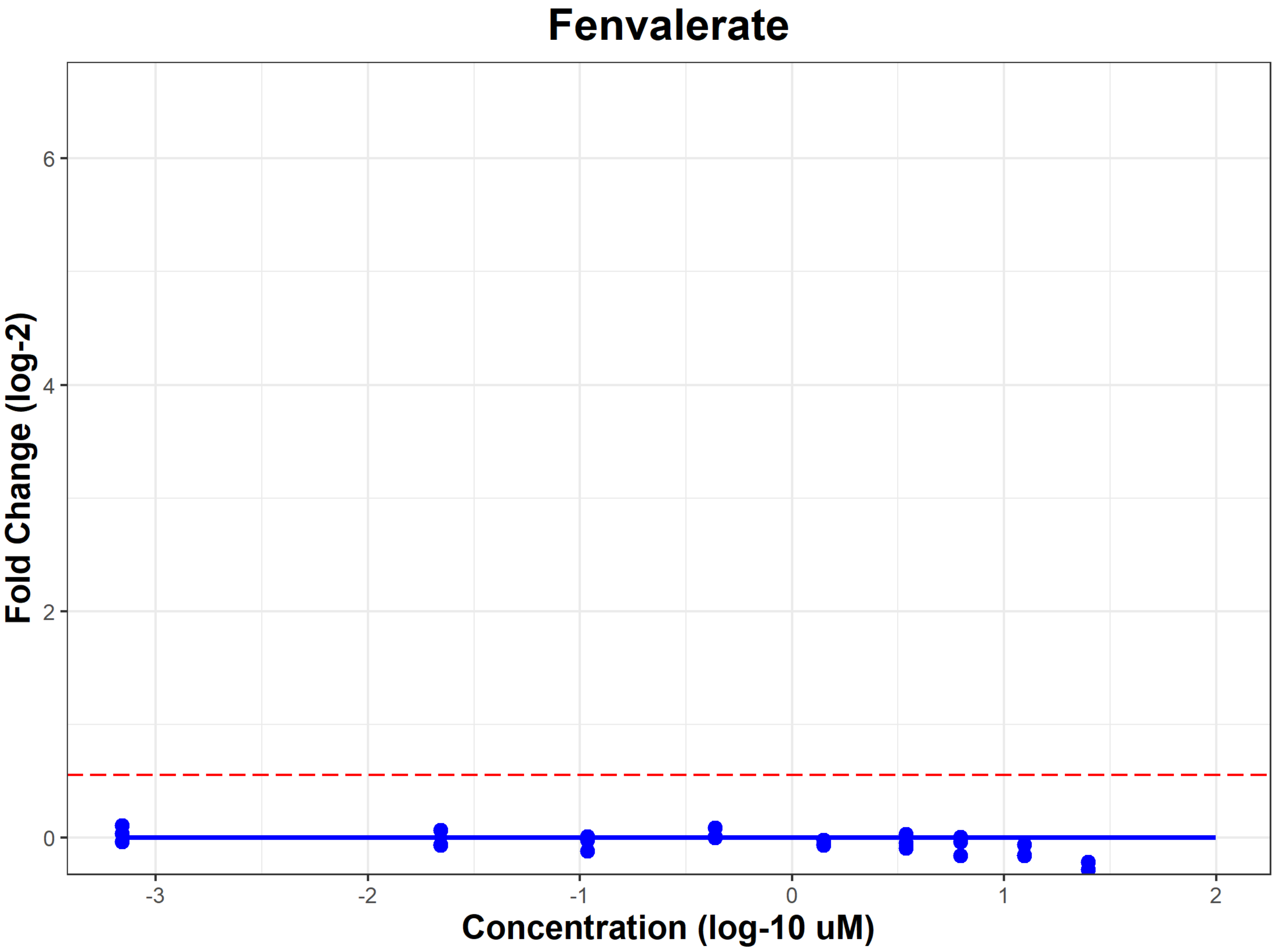

Fenvalerate
Fold Change (log-2)
6
4
2
0
-3
-2
-1
0
1
2
Concentration (log-10 uM)

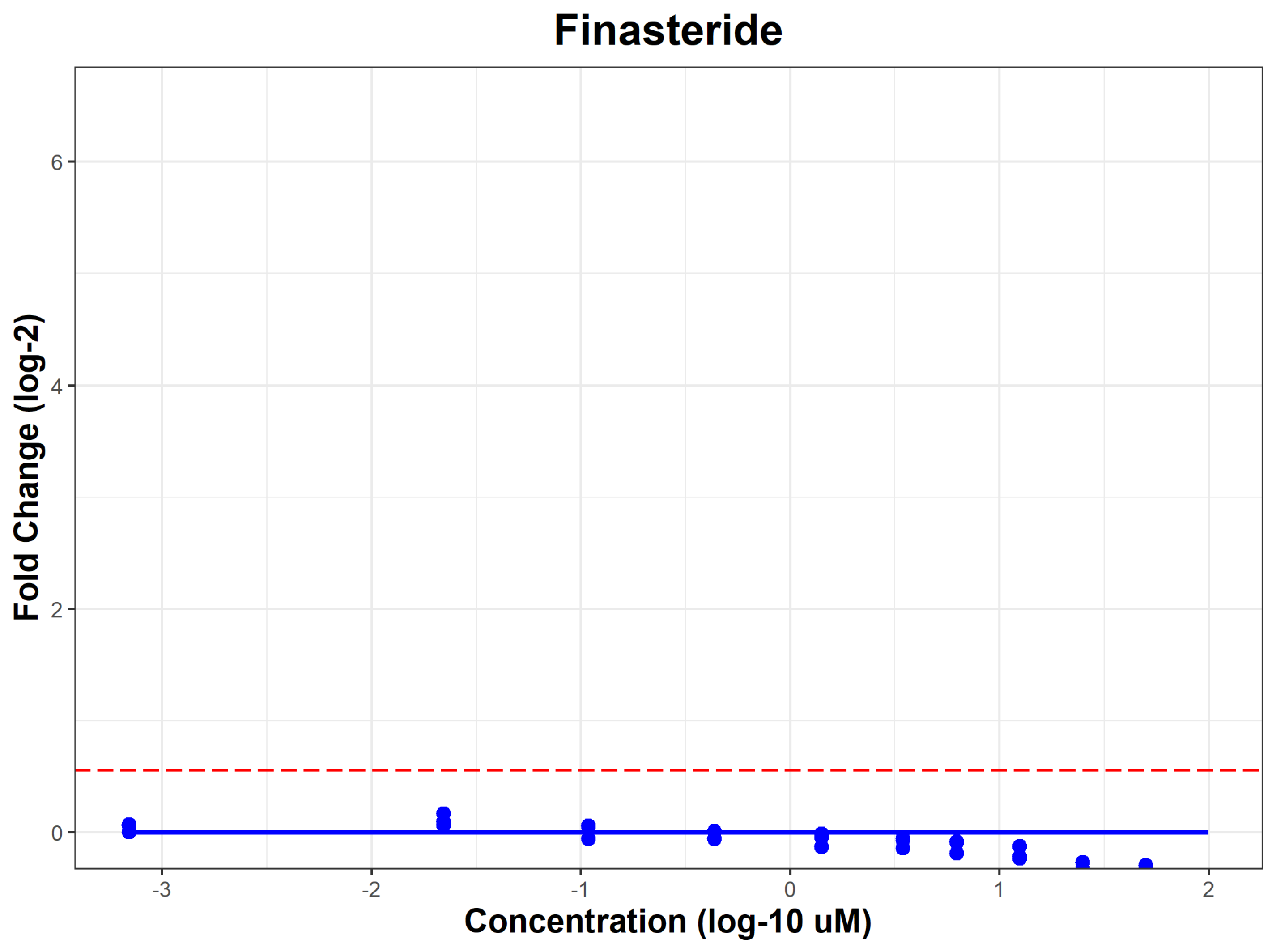
Finasteride
Fold Change (log-2)
6
4
2
0
-3
-2
-1
0
1
2
Concentration (log-10 uM)

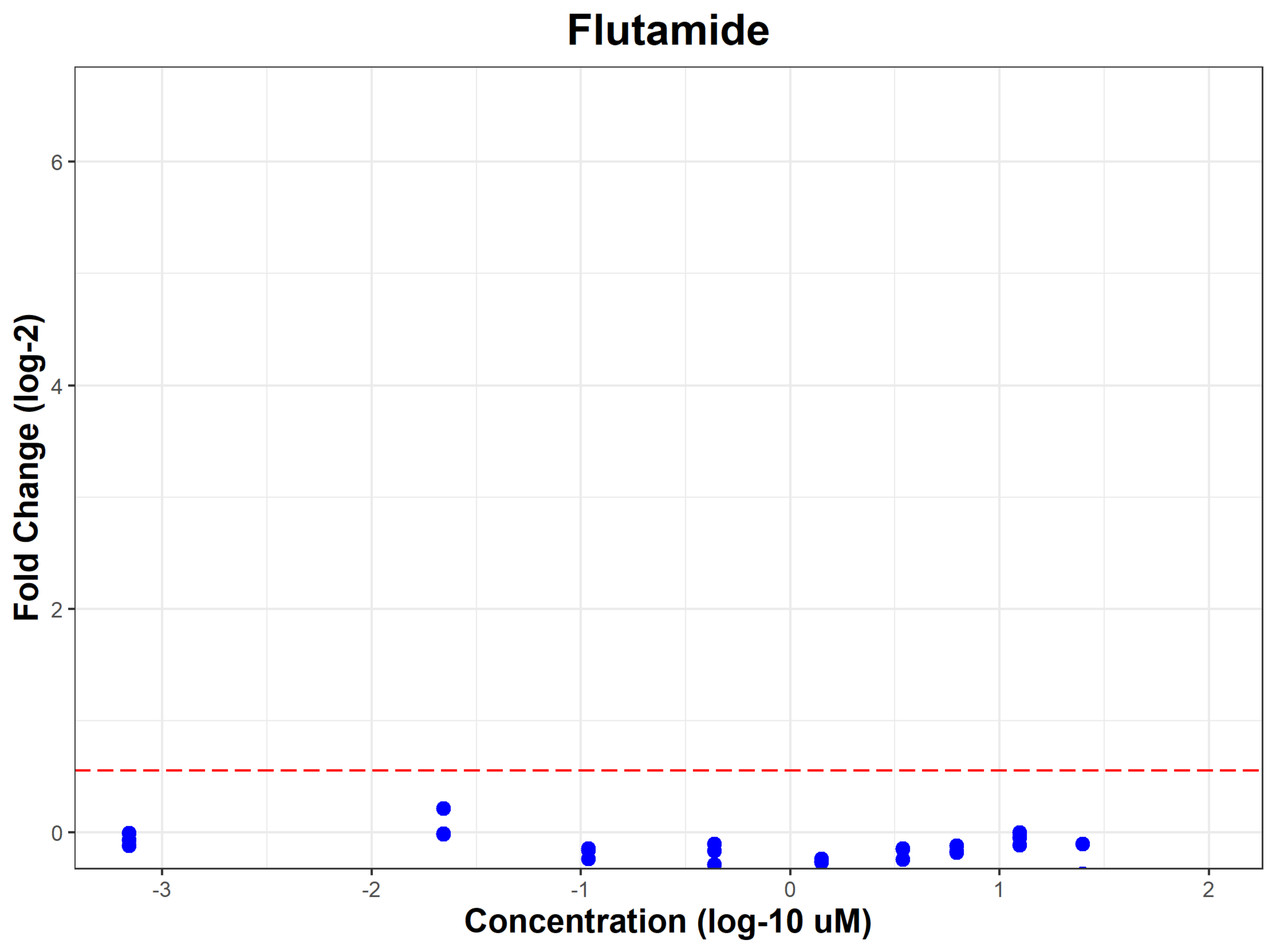
Flutamide
Fold Change (log-2)
6
4
2
0
-3
-2
-1
0
1
2
Concentration (log-10 uM)

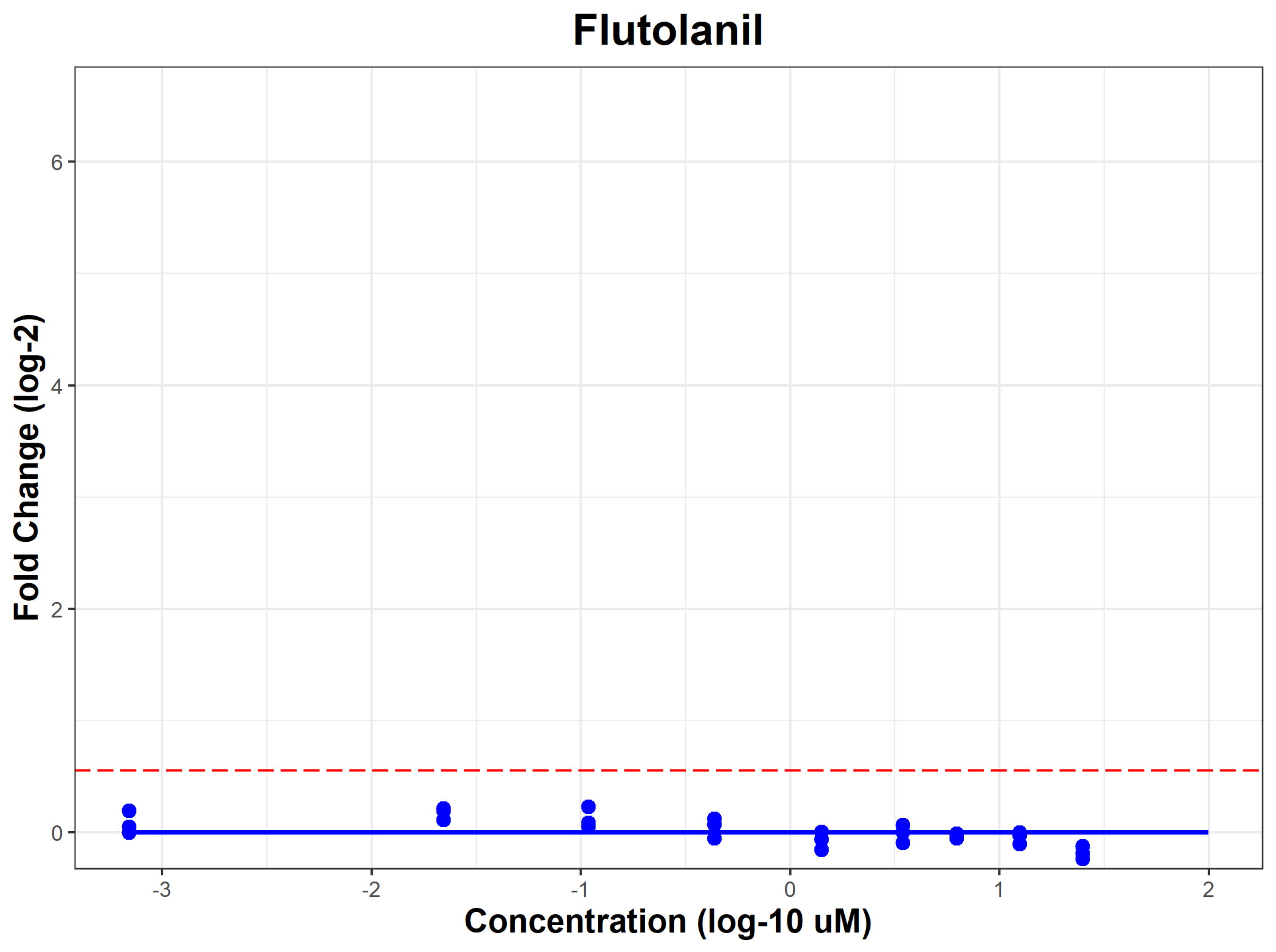
Flutolanil
Fold Change (log-2)
6
4
2
0
-3
-2
-1
0
1
2
Concentration (log-10 uM)

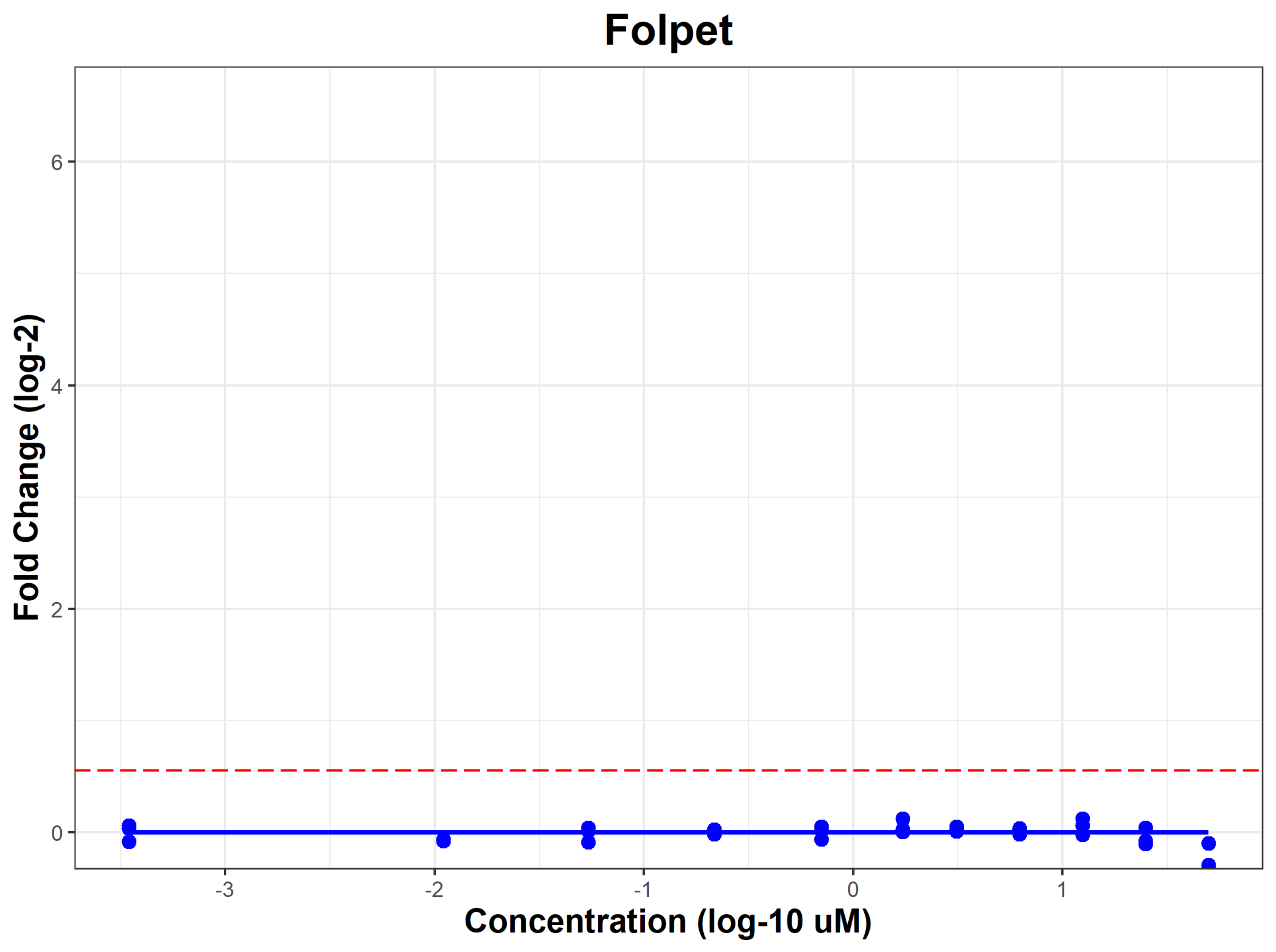
Folpet
Fold Change (log-2)
6
4
2
0
-3
-2
-1
0
1
Concentration (log-10 uM)

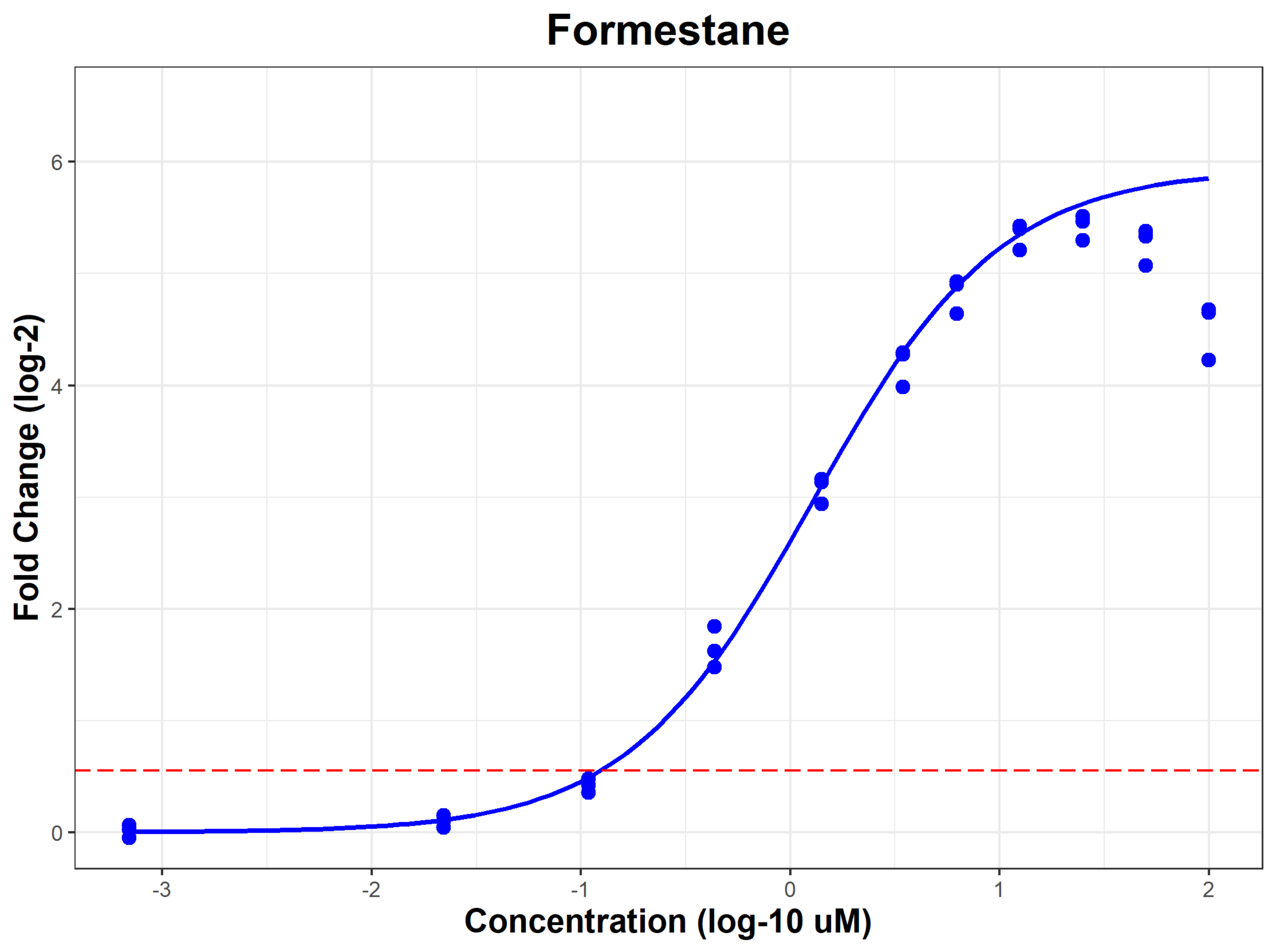

Formestane
Fold Change (log-2)
0
2
4
6
-3
-2
-1
0
1
2
Concentration (log-10 uM)

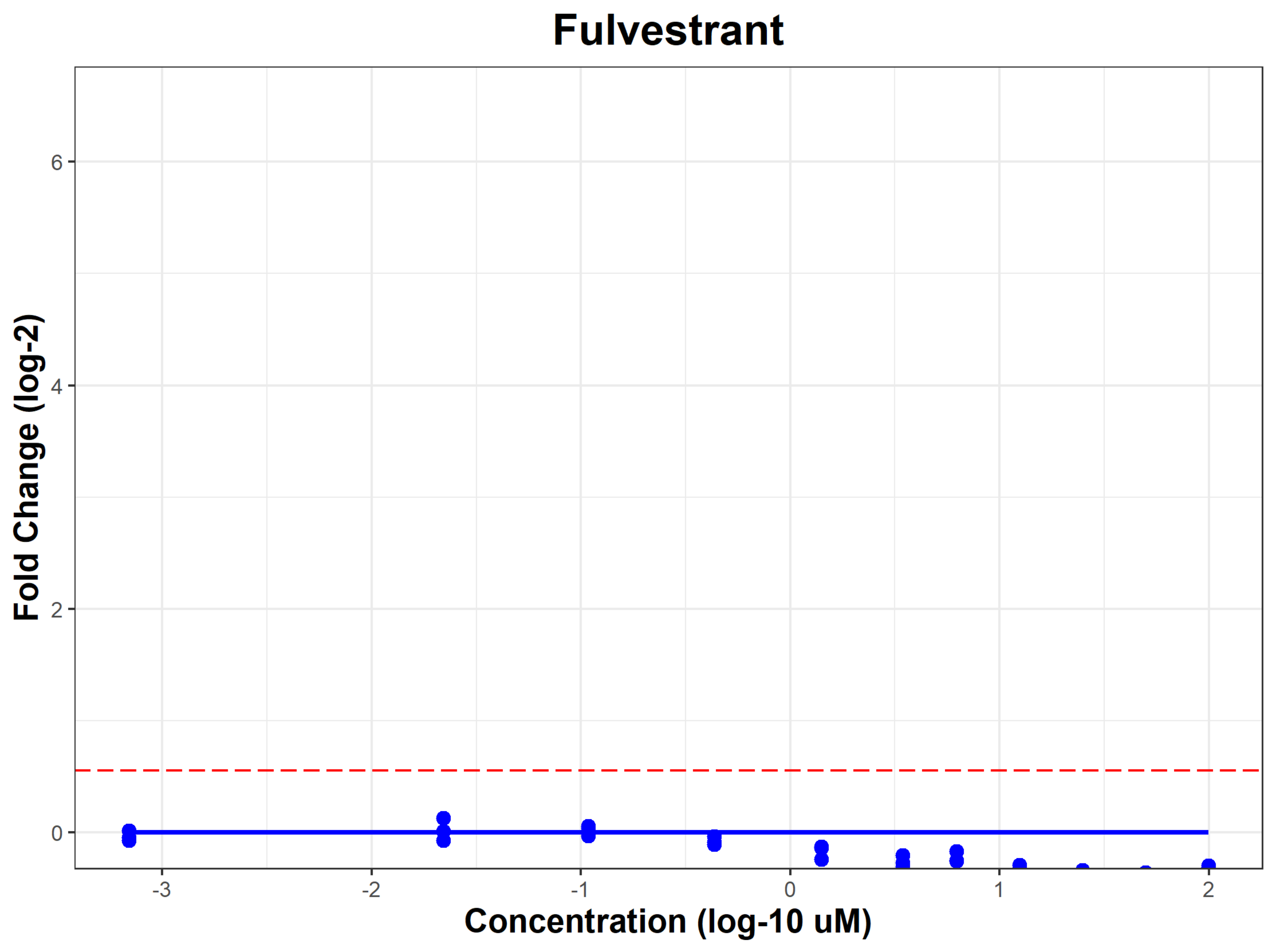

Fulvestrant
Fold Change (log-2)
6
4
2
0
-3
-2
-1
0
1
2
Concentration (log-10 uM)

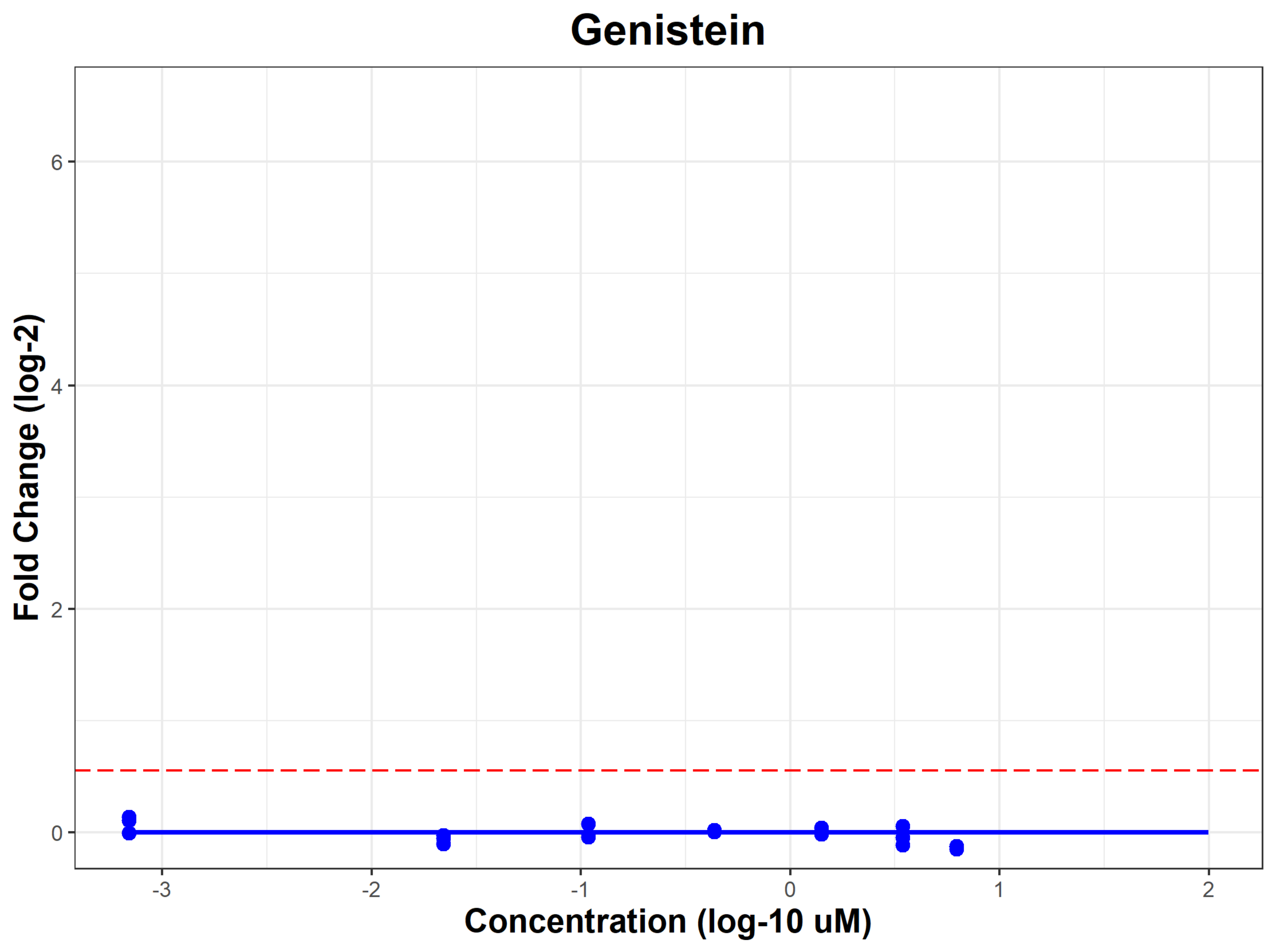

Genistein
Fold Change (log-2)
Concentration (log-10 uM)
6
4
2
0
-3
-2
-1
0
1
2

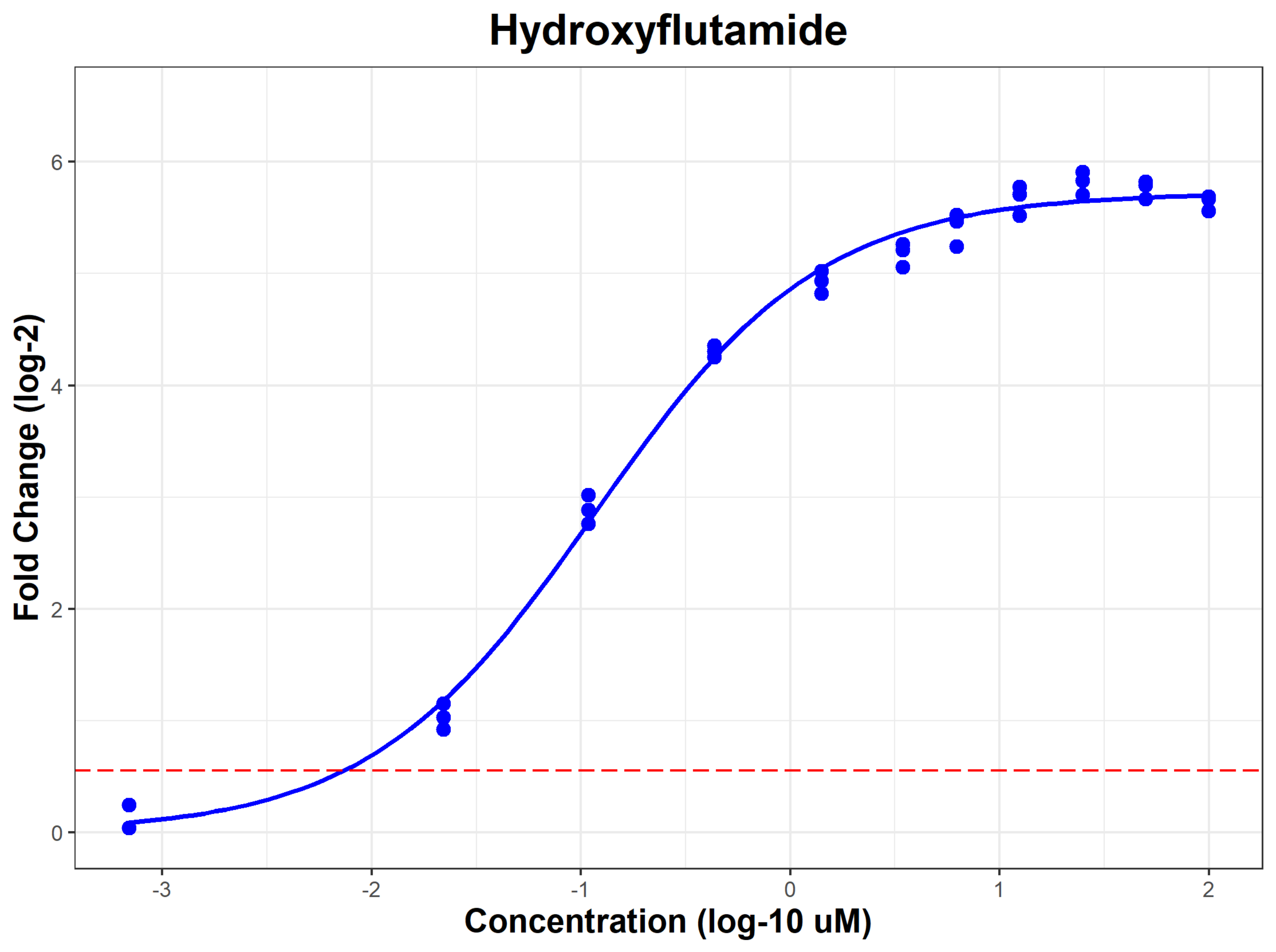
Hydroxyflutamide
Fold Change (log-2)
Concentration (log-10 uM)
0
2
4
6
-3
-2
-1
0
1
2

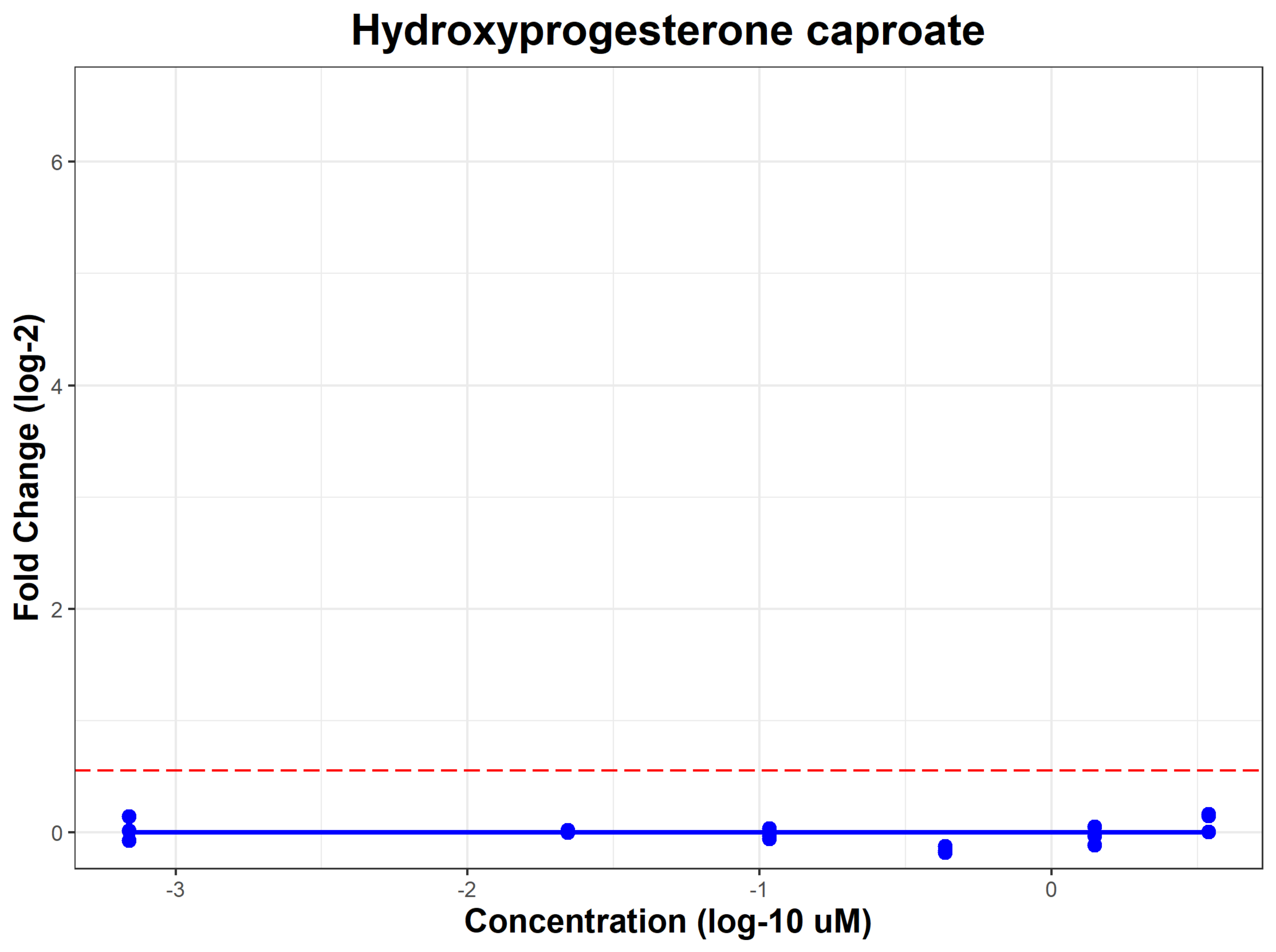

Hydroxyprogesterone caproate
Fold Change (log-2)
6
4
2
0
-3
-2
-1
0
Concentration (log-10 uM)

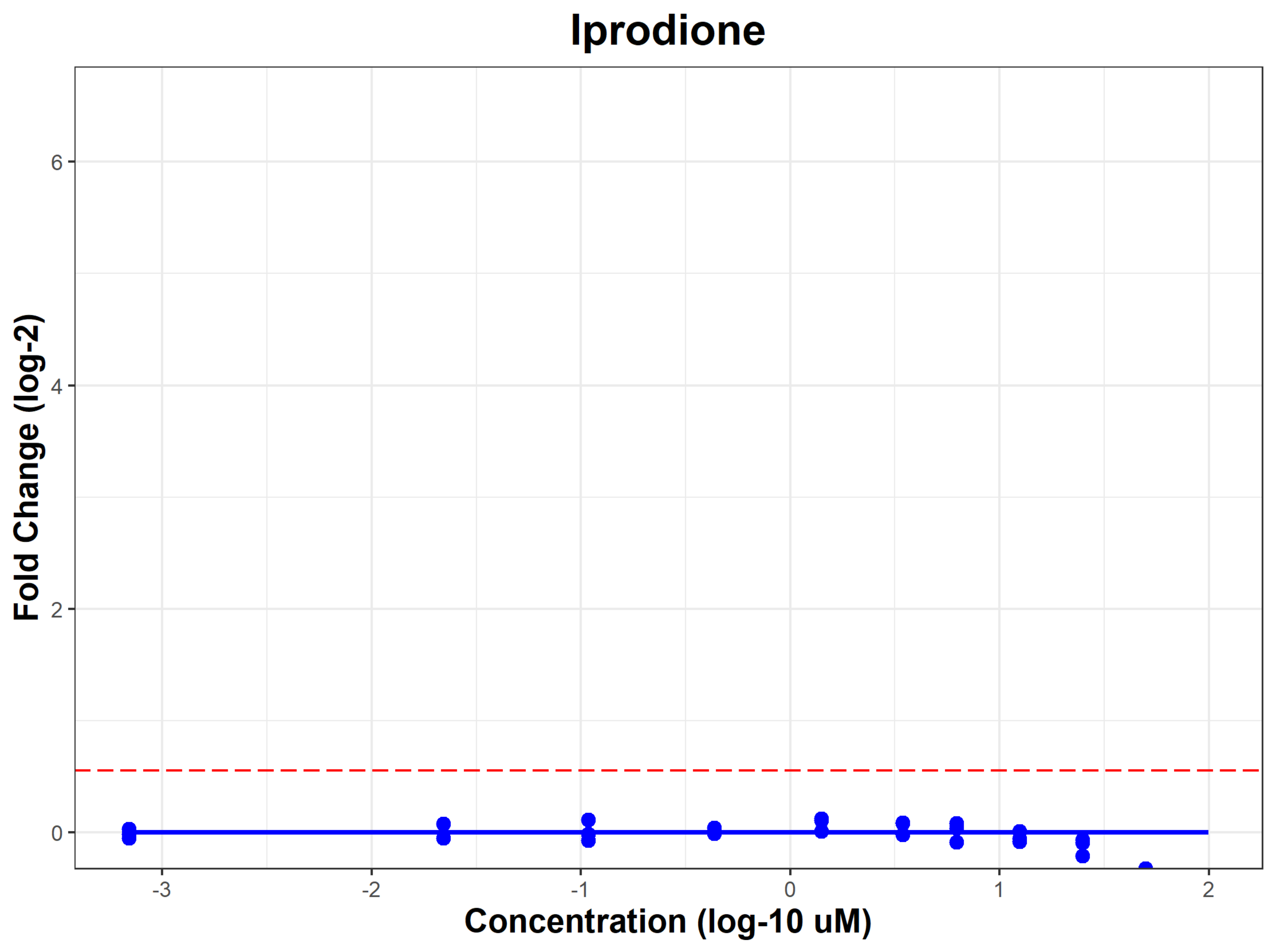

Iprodione
6
4
2
0
-3
-2
-1
0
1
2
Fold Change (log-2)
Concentration (log-10 uM)

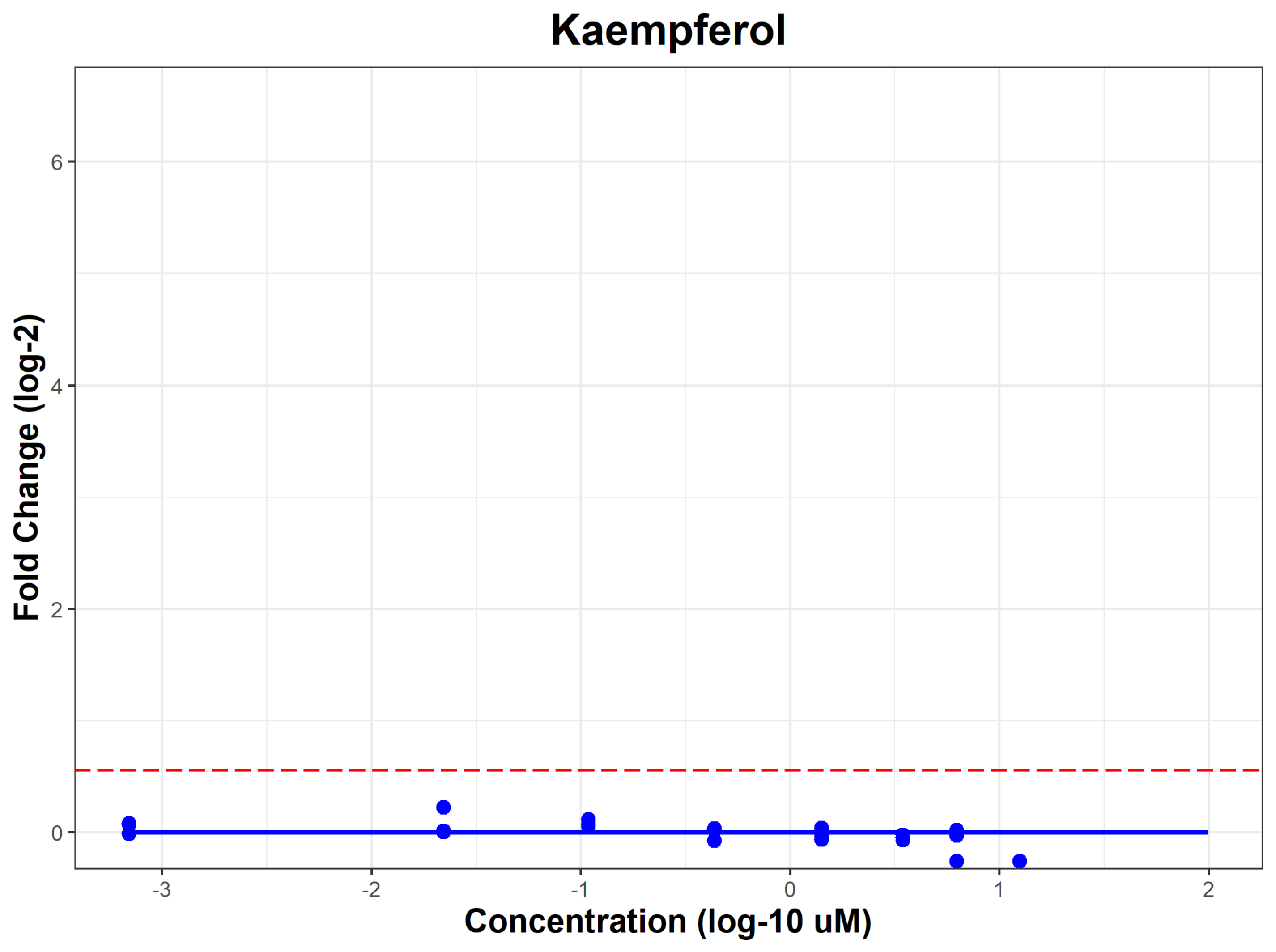
Kaempferol
Fold Change (log-2)
6
4
2
0
-3
-2
-1
0
1
2
Concentration (log-10 uM)

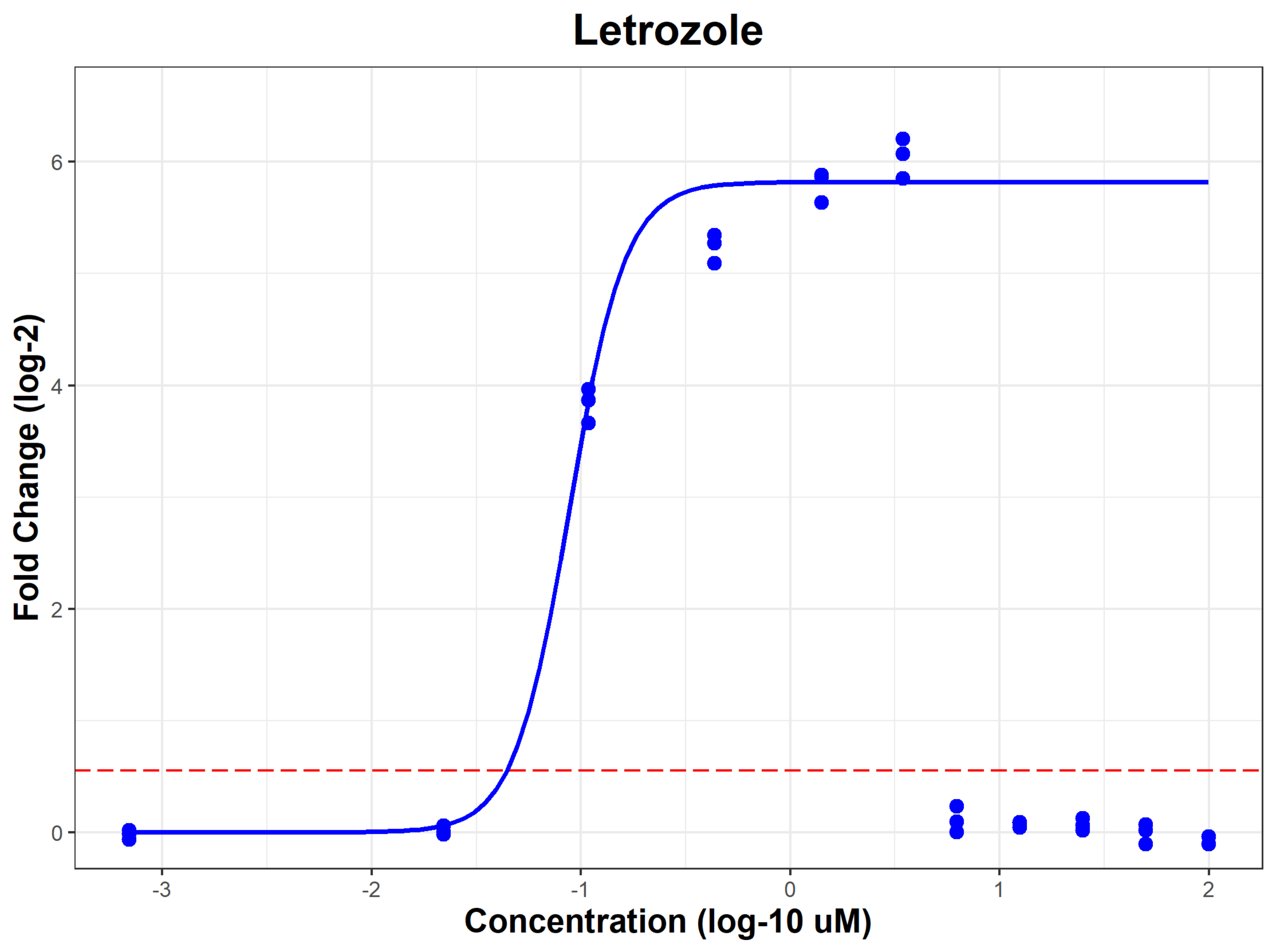
Letrozole
Fold Change (log-2)
6
4
2
0
-3
-2
-1
0
1
2
Concentration (log-10 uM)

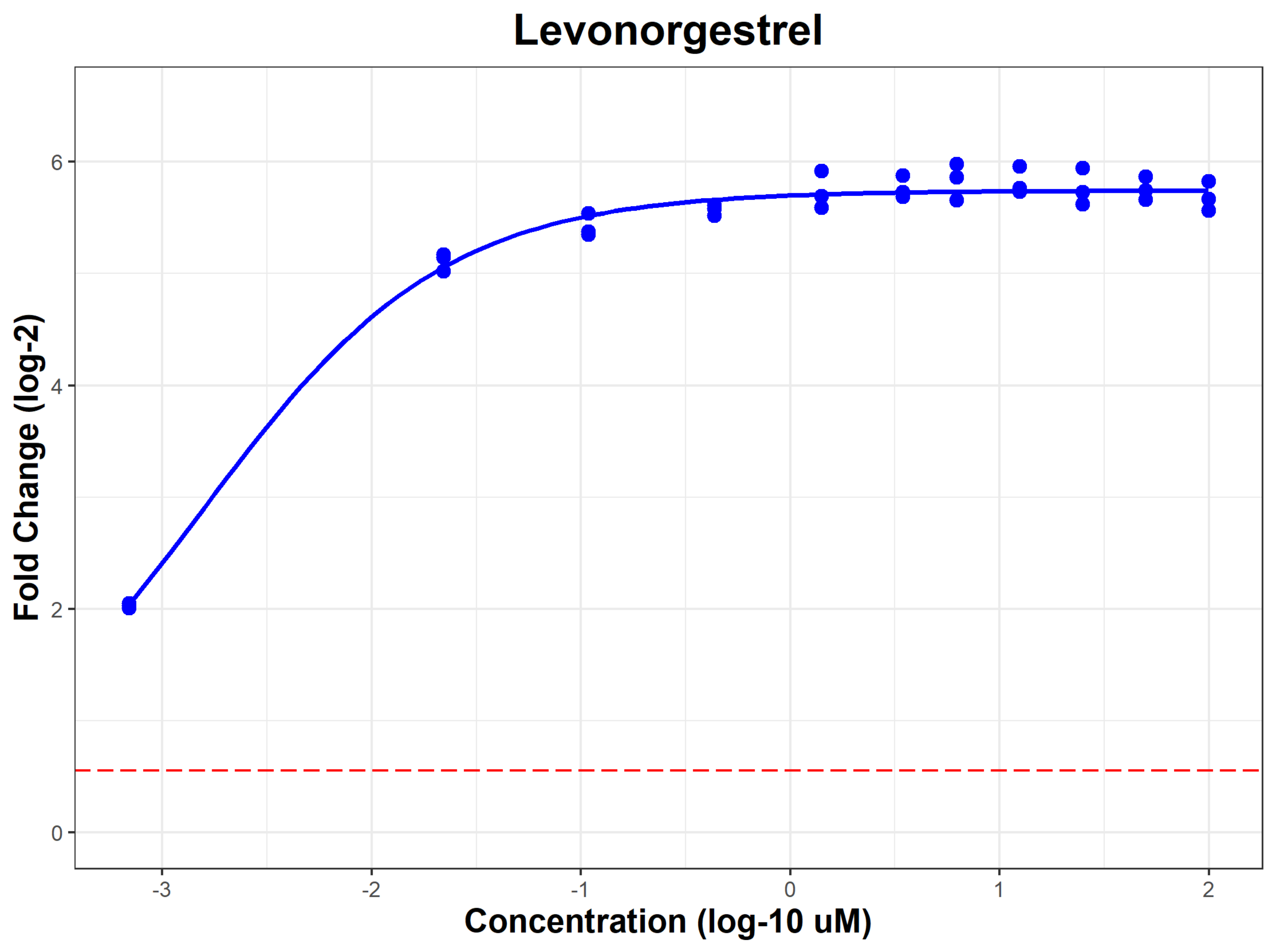

Levonorgestrel
Fold Change (log-2)
Concentration (log-10 uM)
0
2
4
6
-3
-2
-1
0
1
2

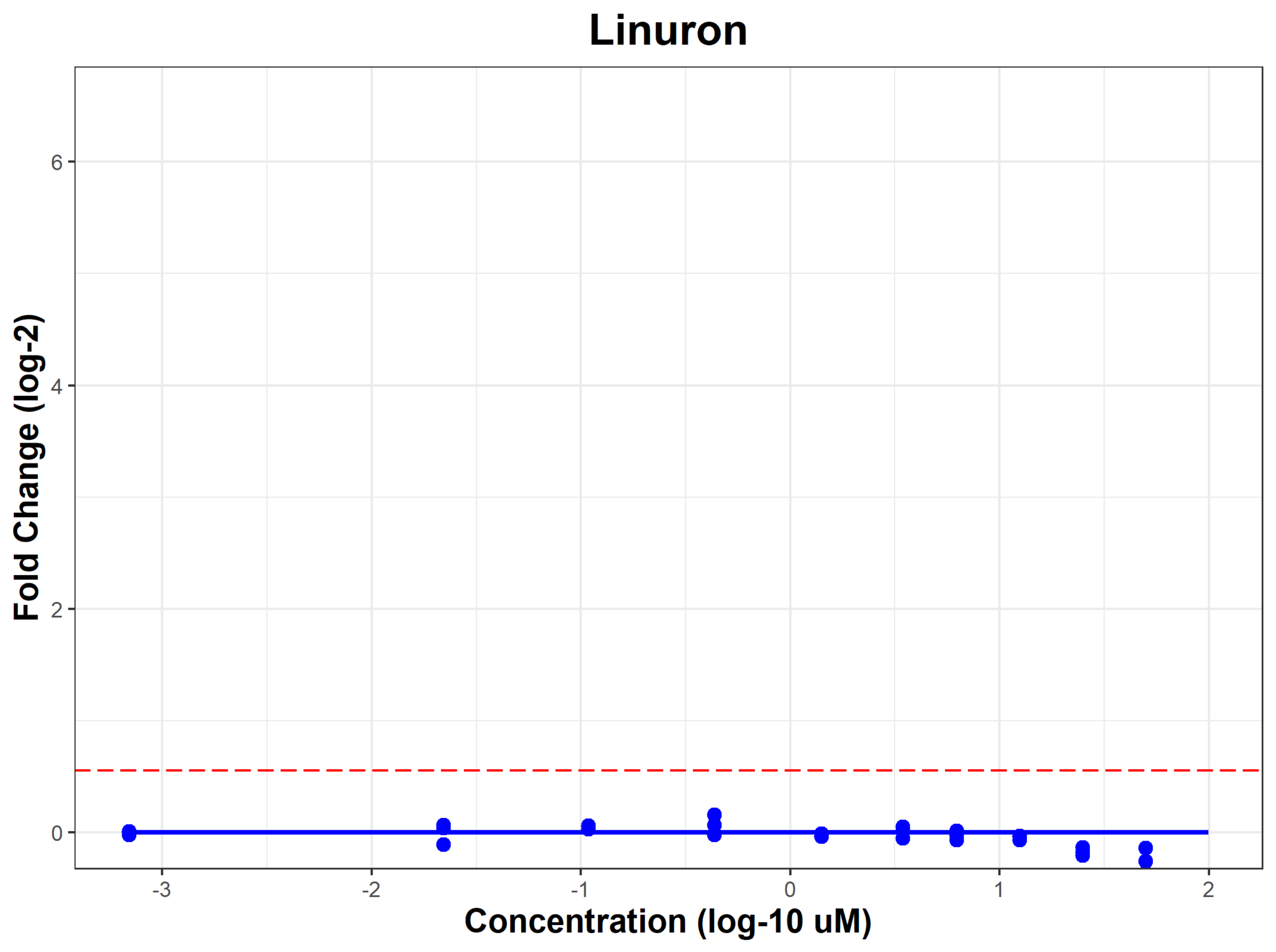

Linuron
Fold Change (log-2)
6
4
2
0
-3
-2
-1
0
1
2
Concentration (log-10 uM)

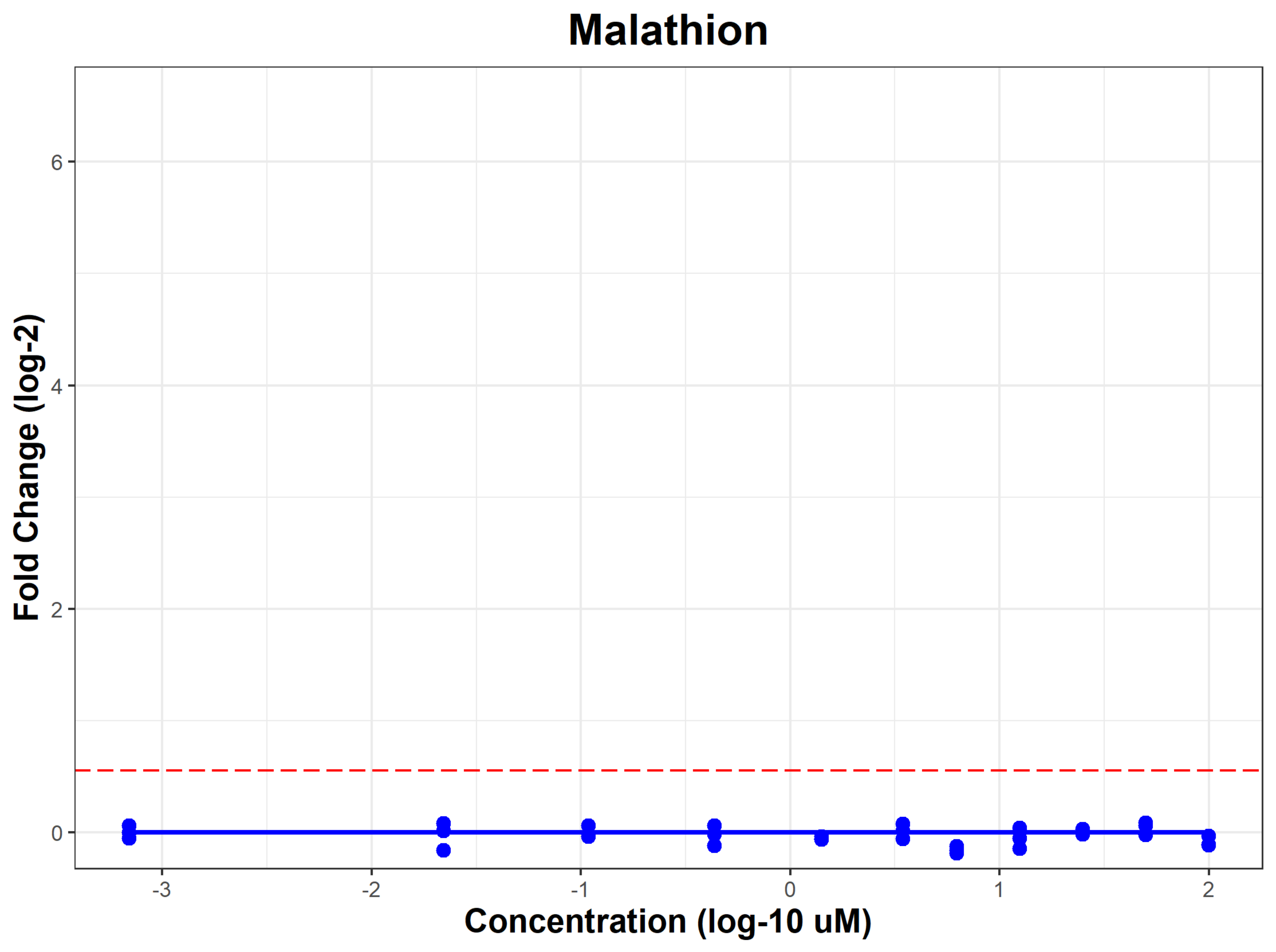
Malathion
Fold Change (log-2)
Concentration (log-10 uM)
6
4
2
0
-3
-2
-1
0
1
2

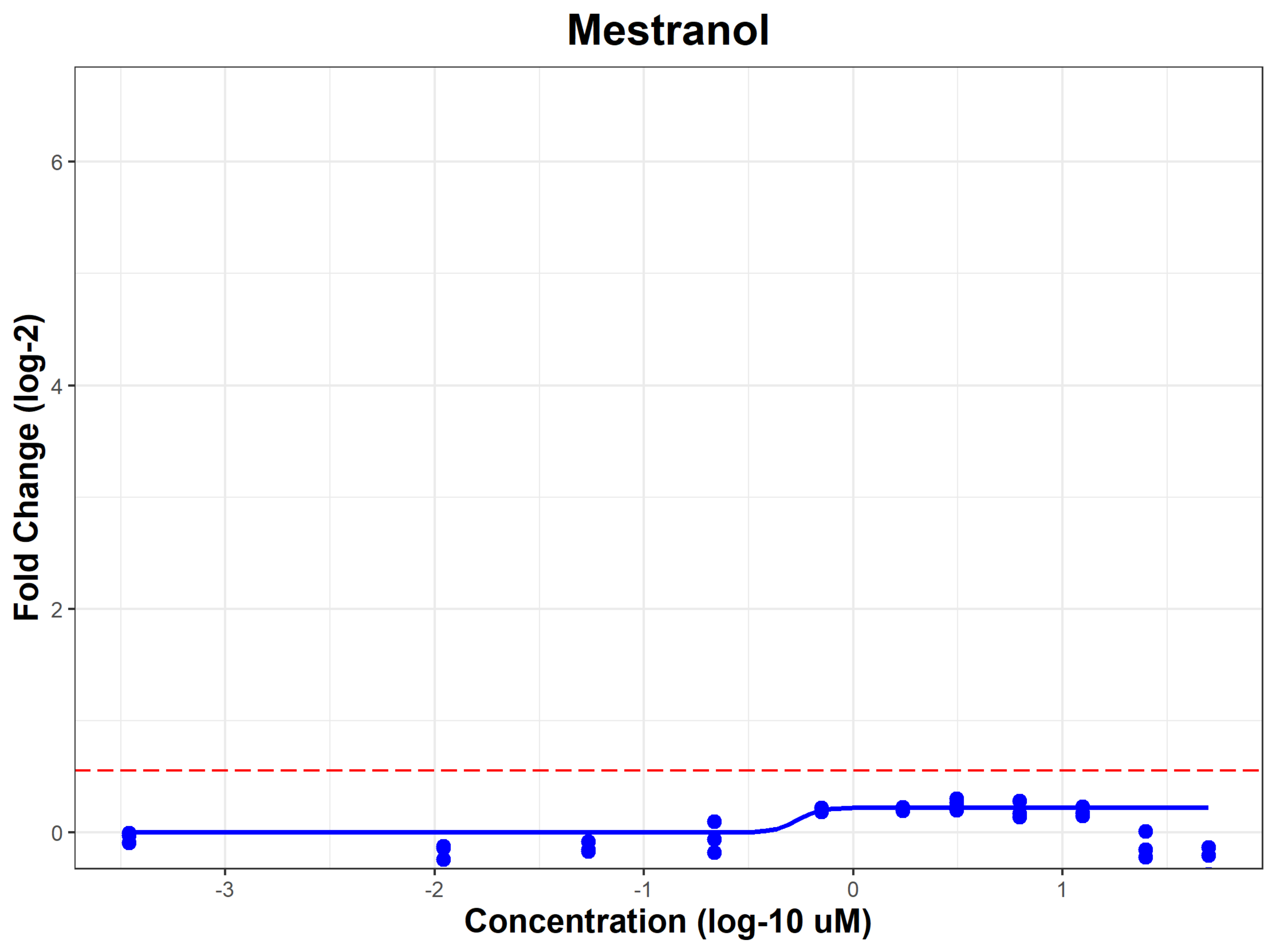

Mestranol
Fold Change (log-2)
6
4
2
0
-3
-2
-1
0
1
Concentration (log-10 uM)

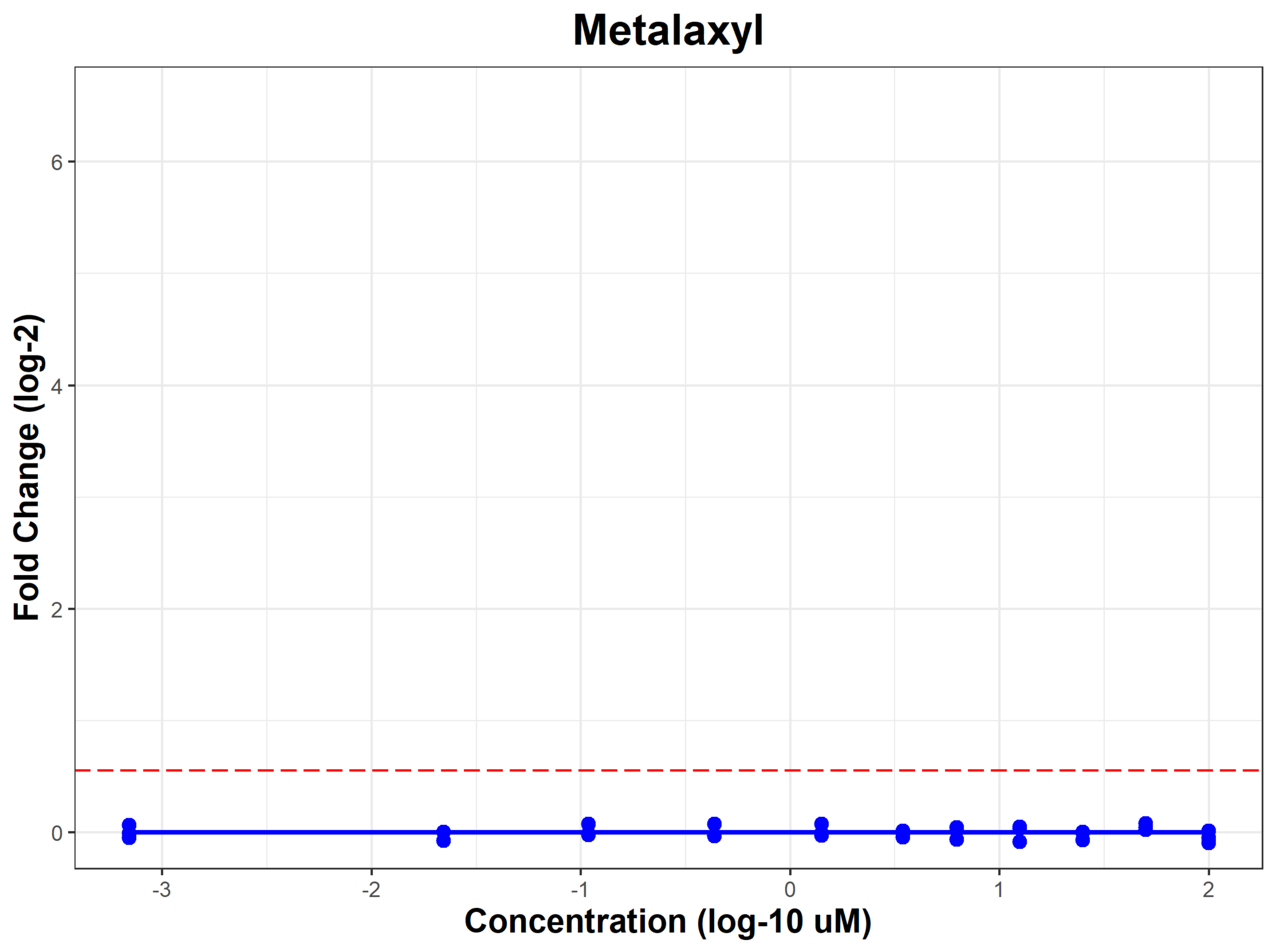

Metalaxyl
Fold Change (log-2)
6
4
2
0
-3
-2
-1
0
1
2
Concentration (log-10 uM)

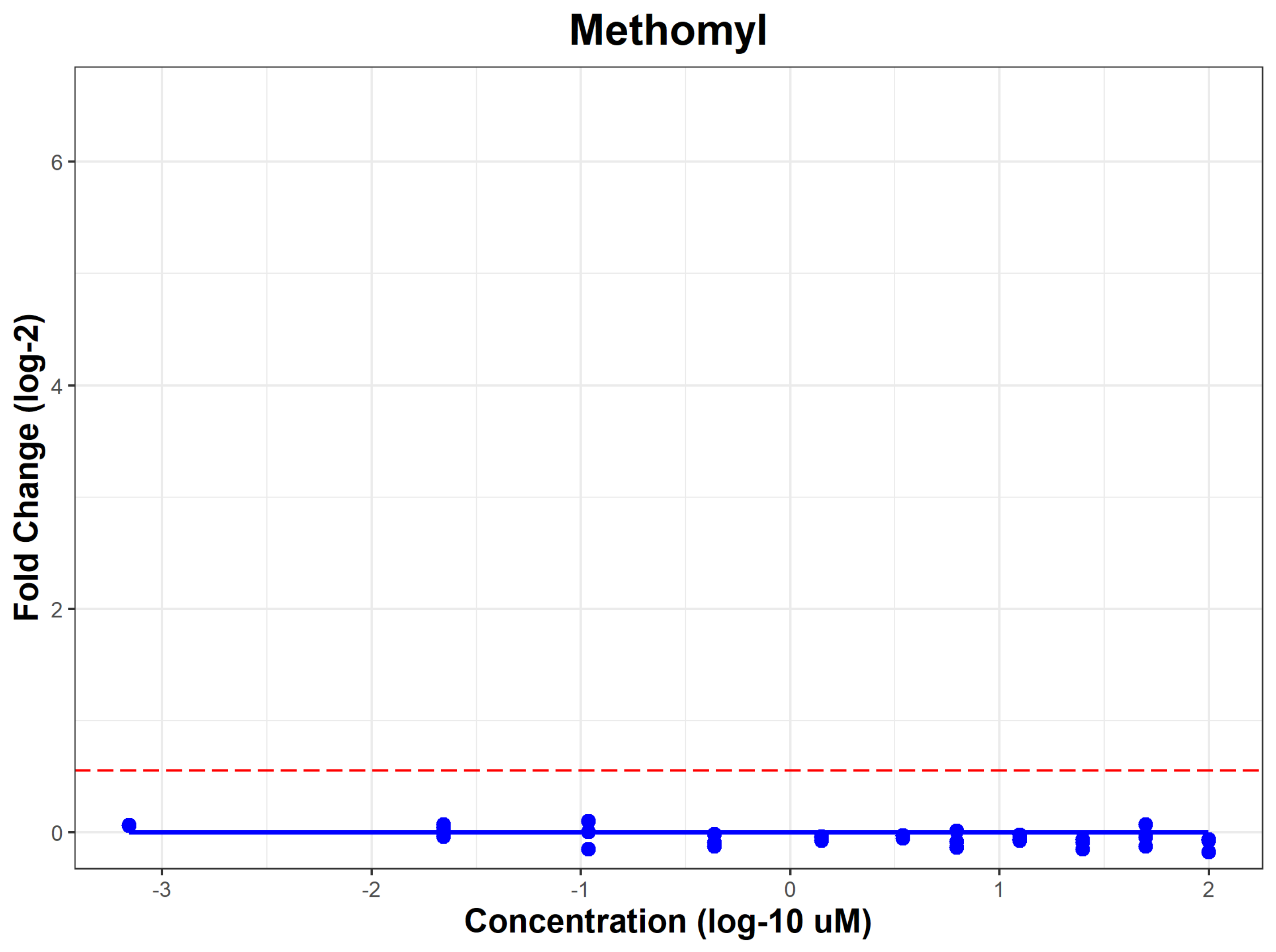

Methomyl
Fold Change (log-2)
6
4
2
0
-3
-2
-1
0
1
2
Concentration (log-10 uM)

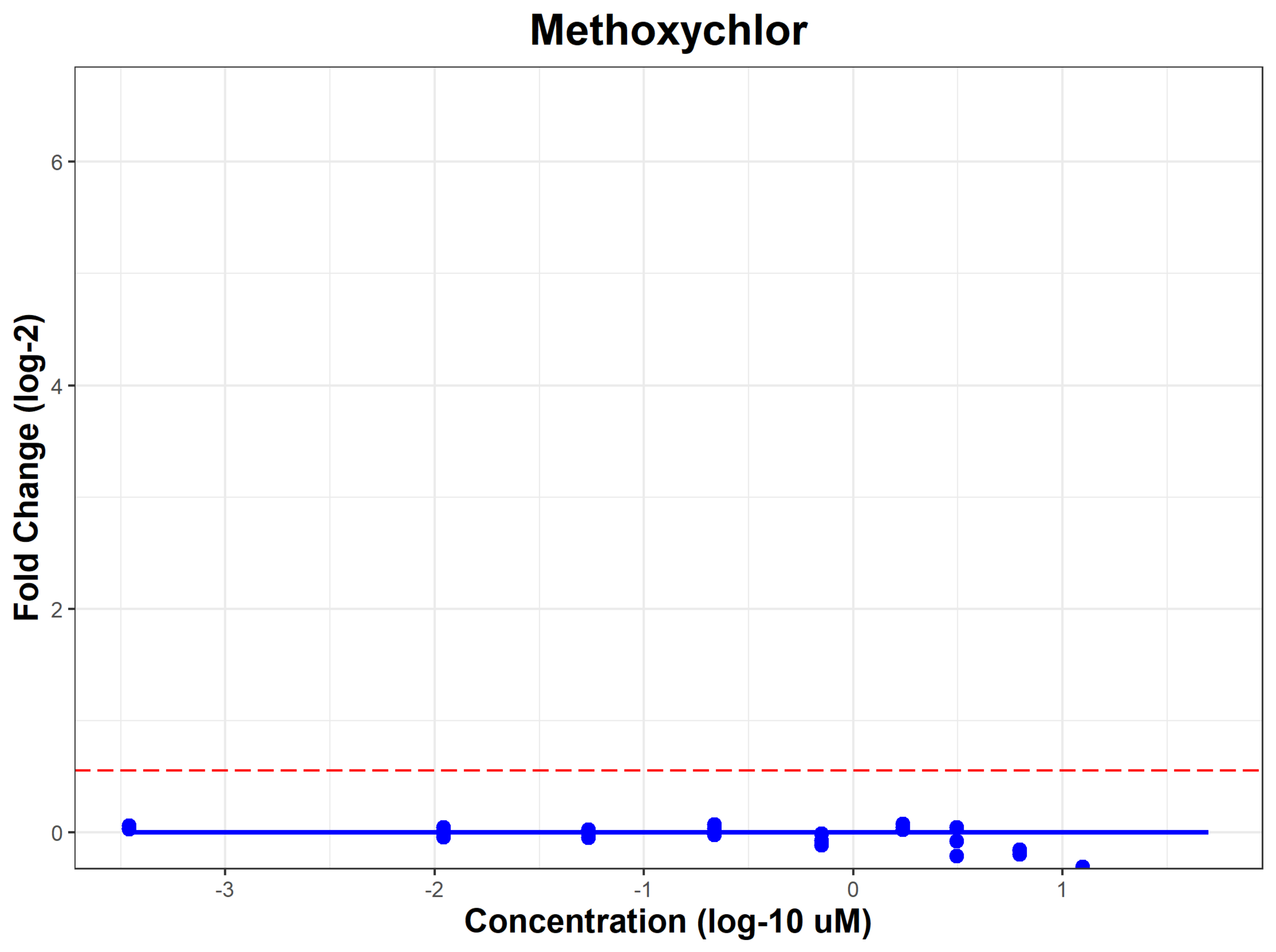

Methoxychlor
Fold Change (log-2)
Concentration (log-10 uM)
6
4
2
0
-3
-2
-1
0
1

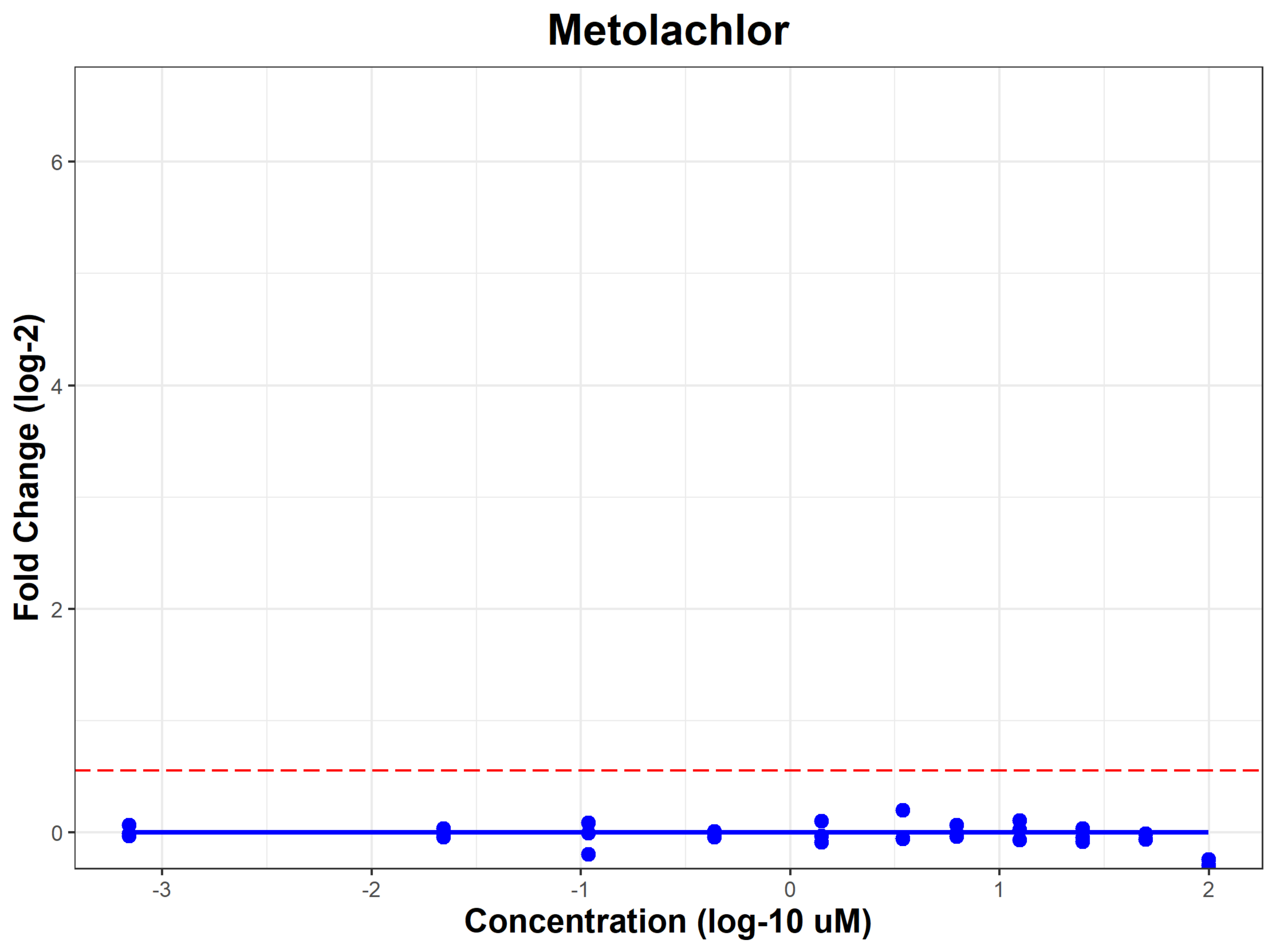

Metolachlor
Fold Change (log-2)
6
4
2
0
-3
-2
-1
0
1
2
Concentration (log-10 uM)

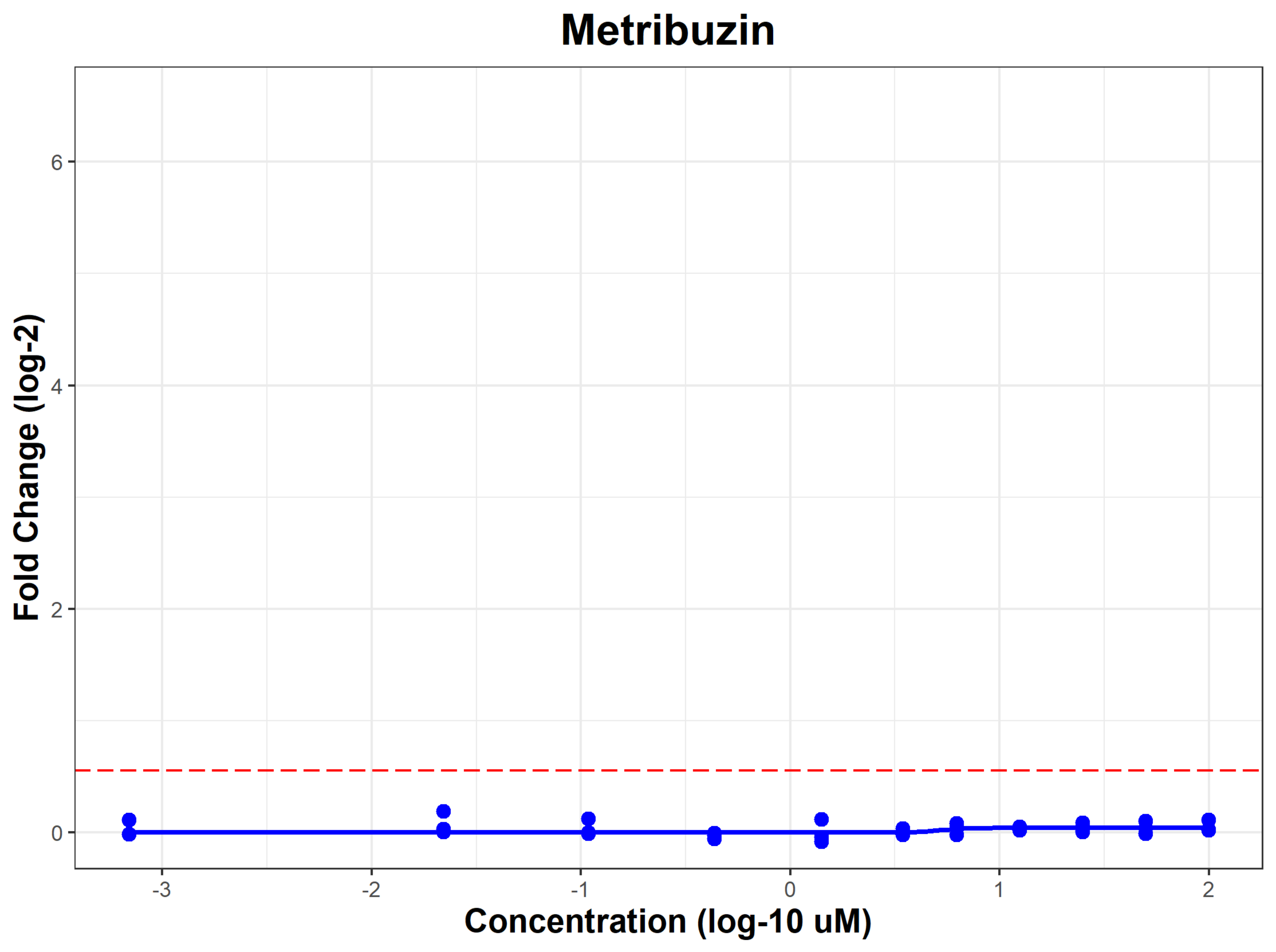

Metribuzin
Fold Change (log-2)
Concentration (log-10 uM)
6
4
2
0
-3
-2
-1
0
1
2

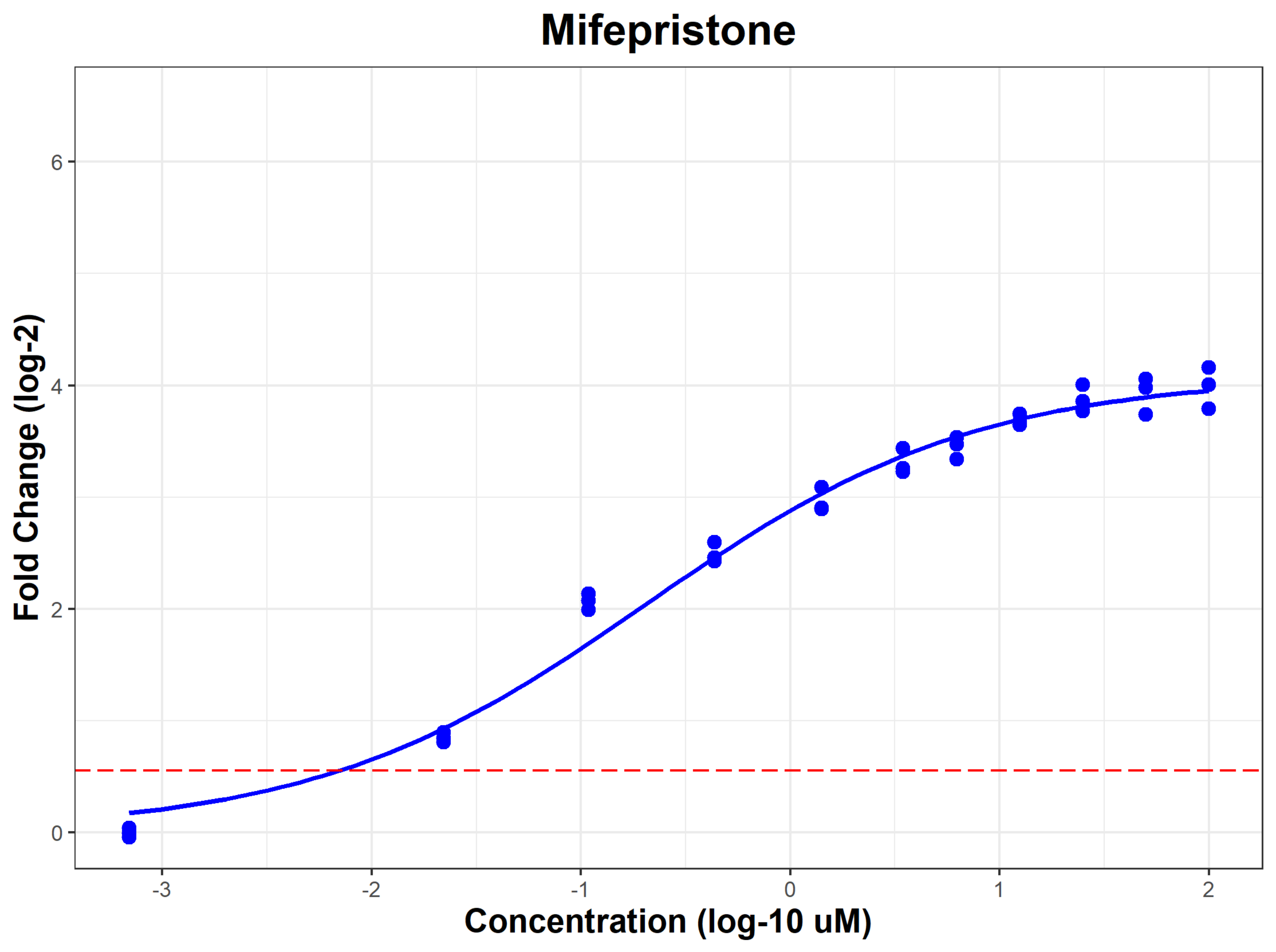

Mifepristone
Fold Change (log-2)
Concentration (log-10 uM)
6
4
2
0
-3
-2
-1
0
1
2

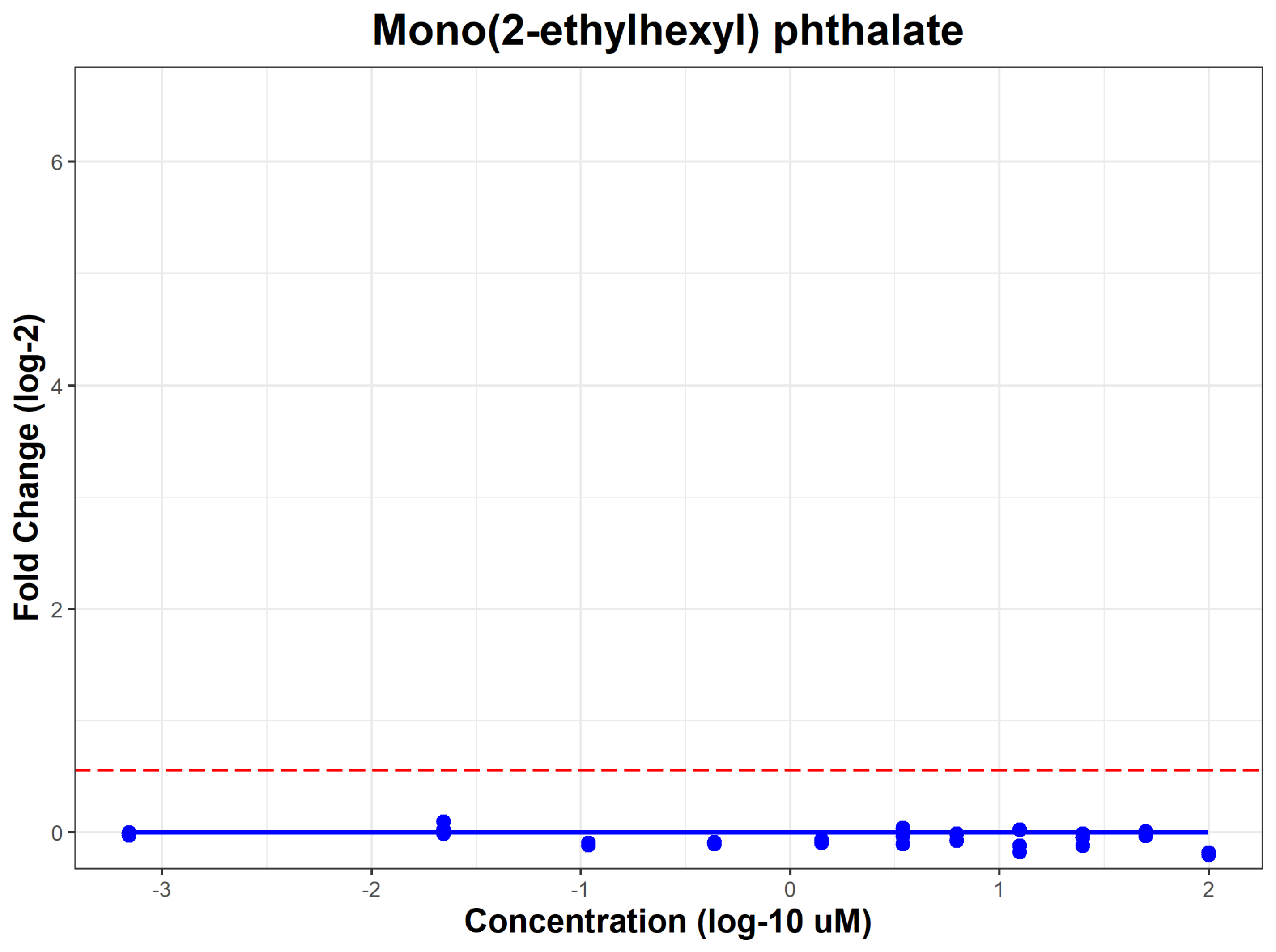

Mono(2-ethylhexyl) phthalate
Fold Change (log-2)
Concentration (log-10 uM)
6
4
2
0
-3
-2
-1
0
1
2

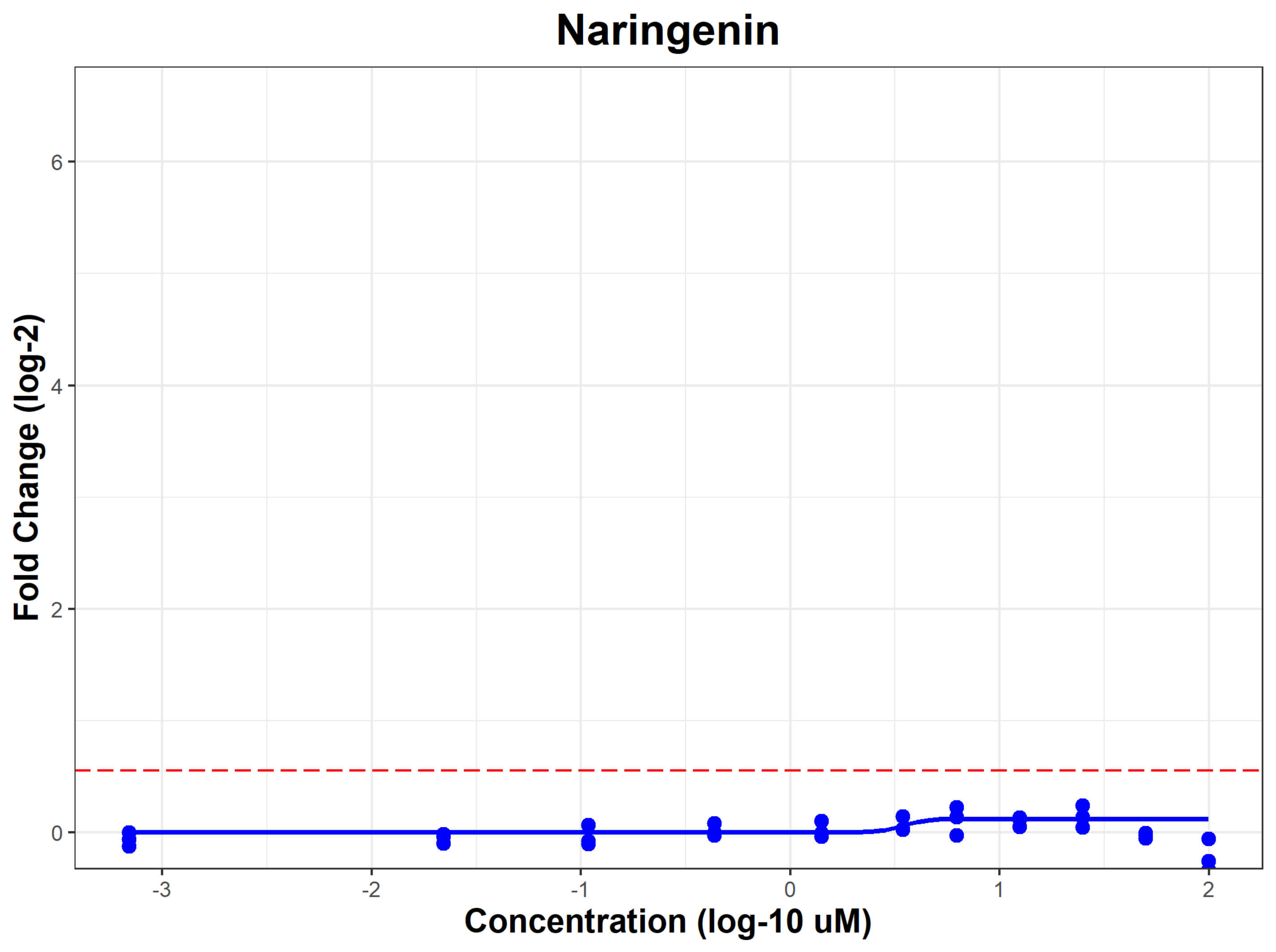

Naringenin
Fold Change (log-2)
Concentration (log-10 uM)
6
4
2
0
-3
-2
-1
0
1
2

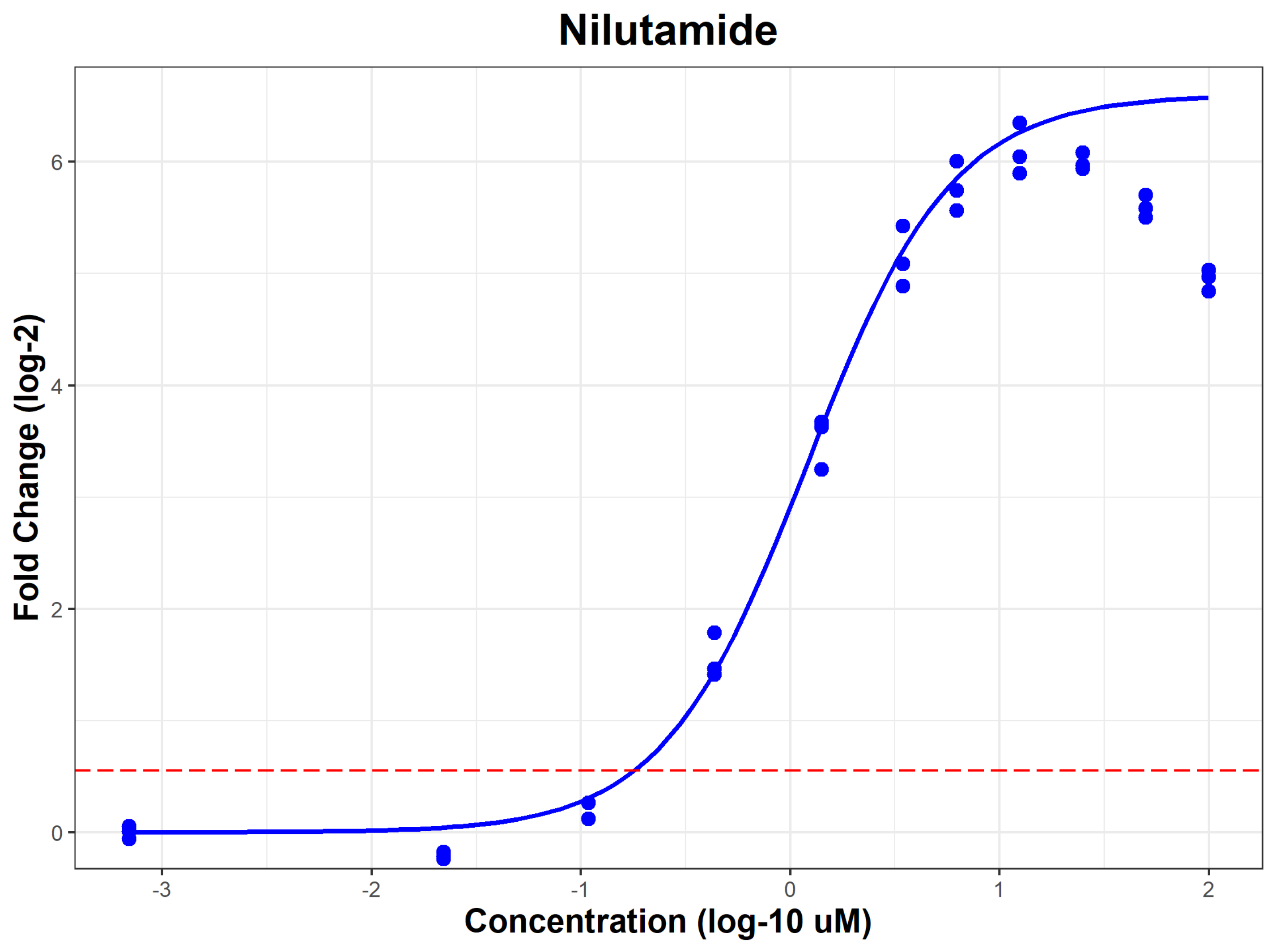

Nilutamide
Fold Change (log-2)
Concentration (log-10 uM)
0
2
4
6
-3
-2
-1
0
1
2

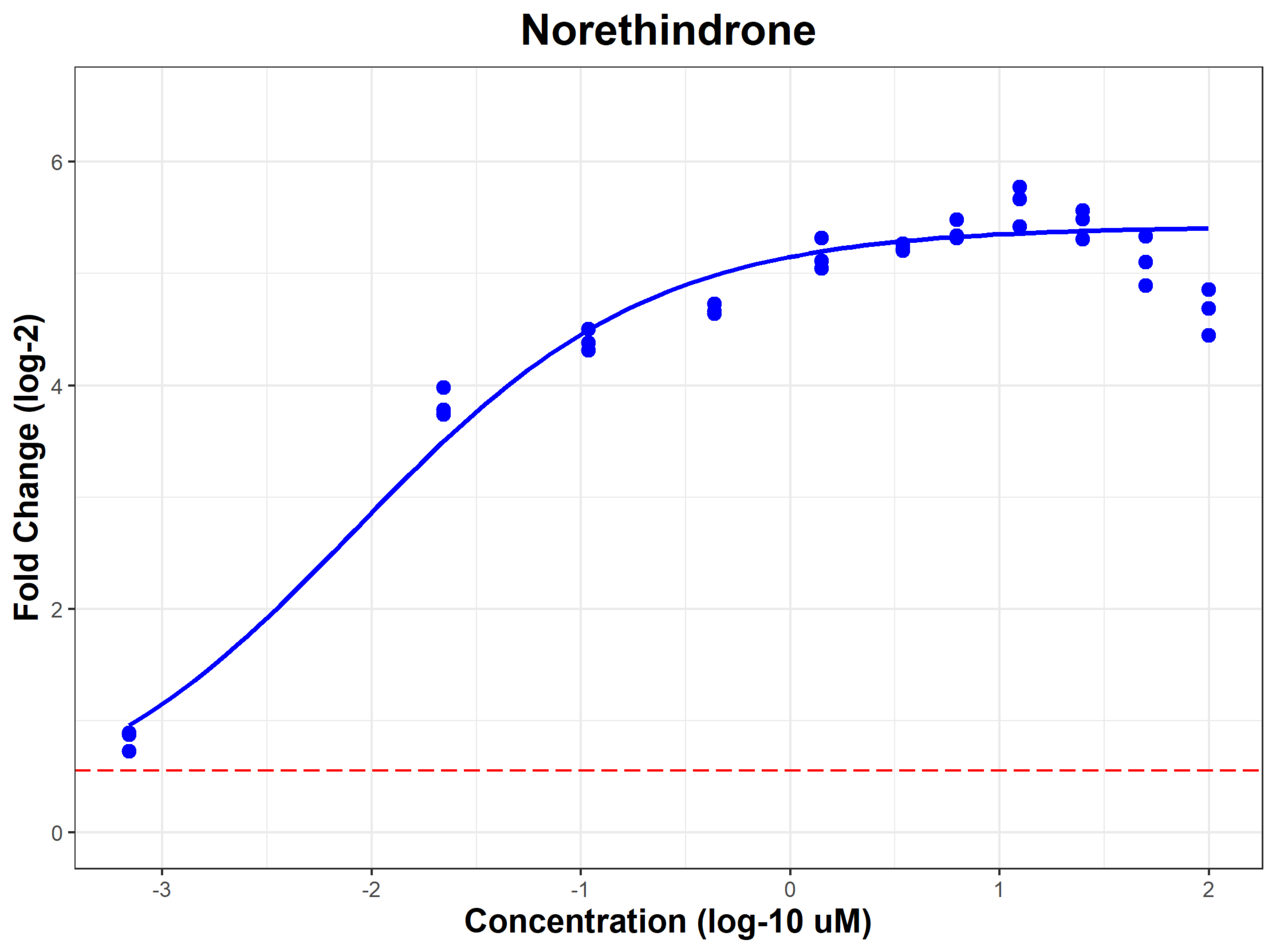

Norethindrone
Fold Change (log-2)
Concentration (log-10 uM)
0
2
4
6
-3
-2
-1
0
1
2

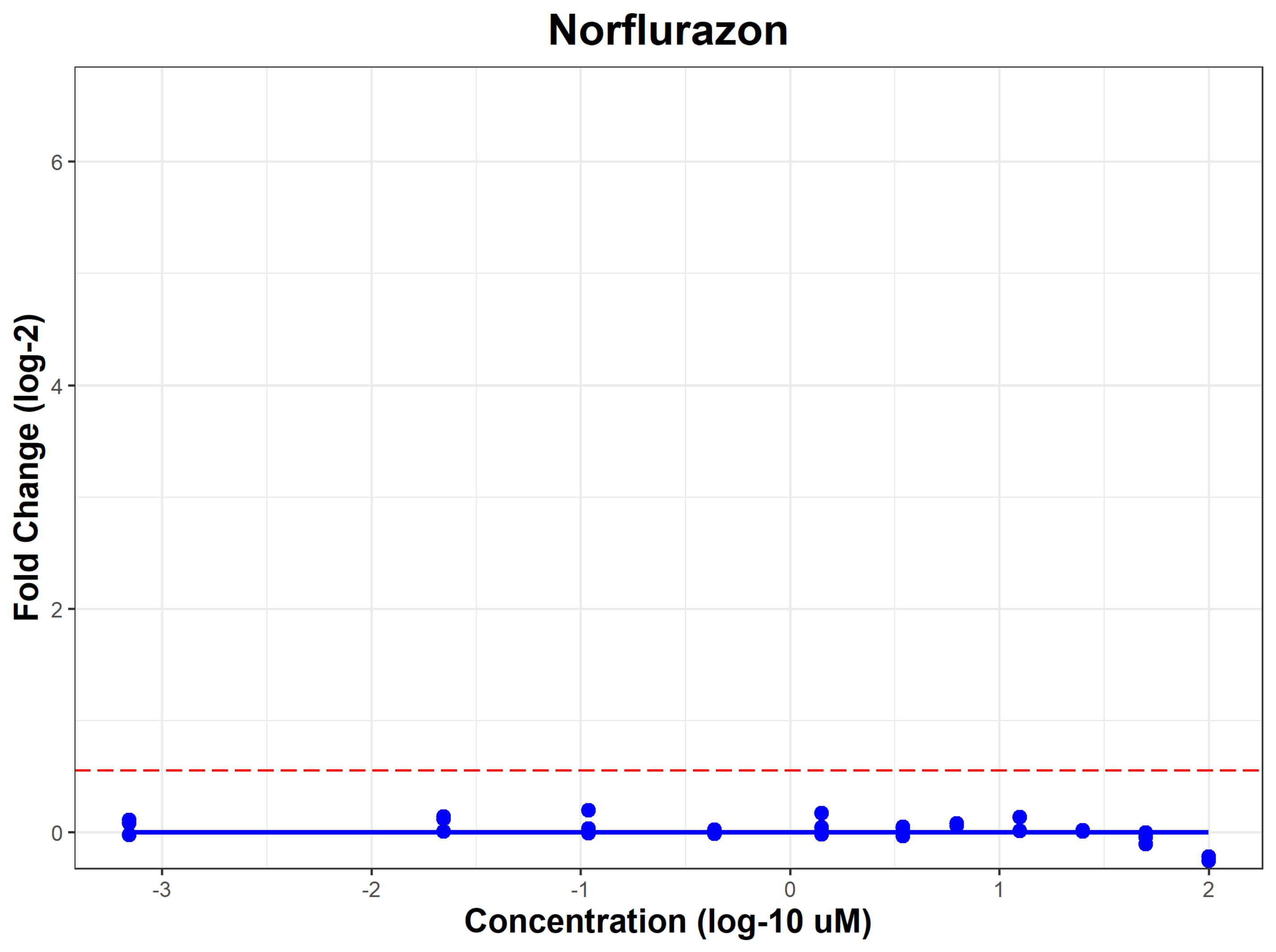
Norflurazon
Fold Change (log-2)
6
4
2
0
-3
-2
-1
0
1
2
Concentration (log-10 uM)

Octamethylcyclotetrasiloxane

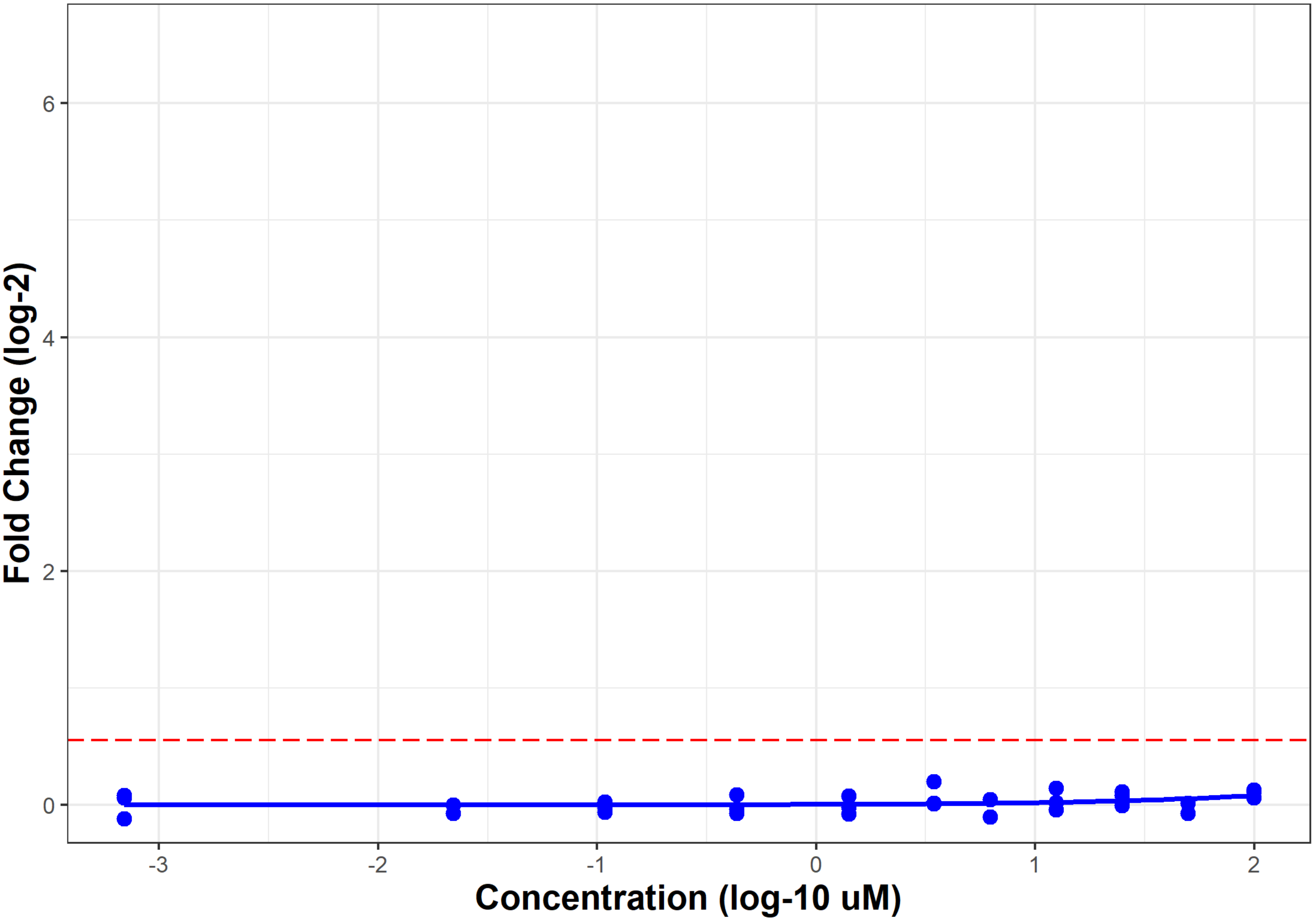

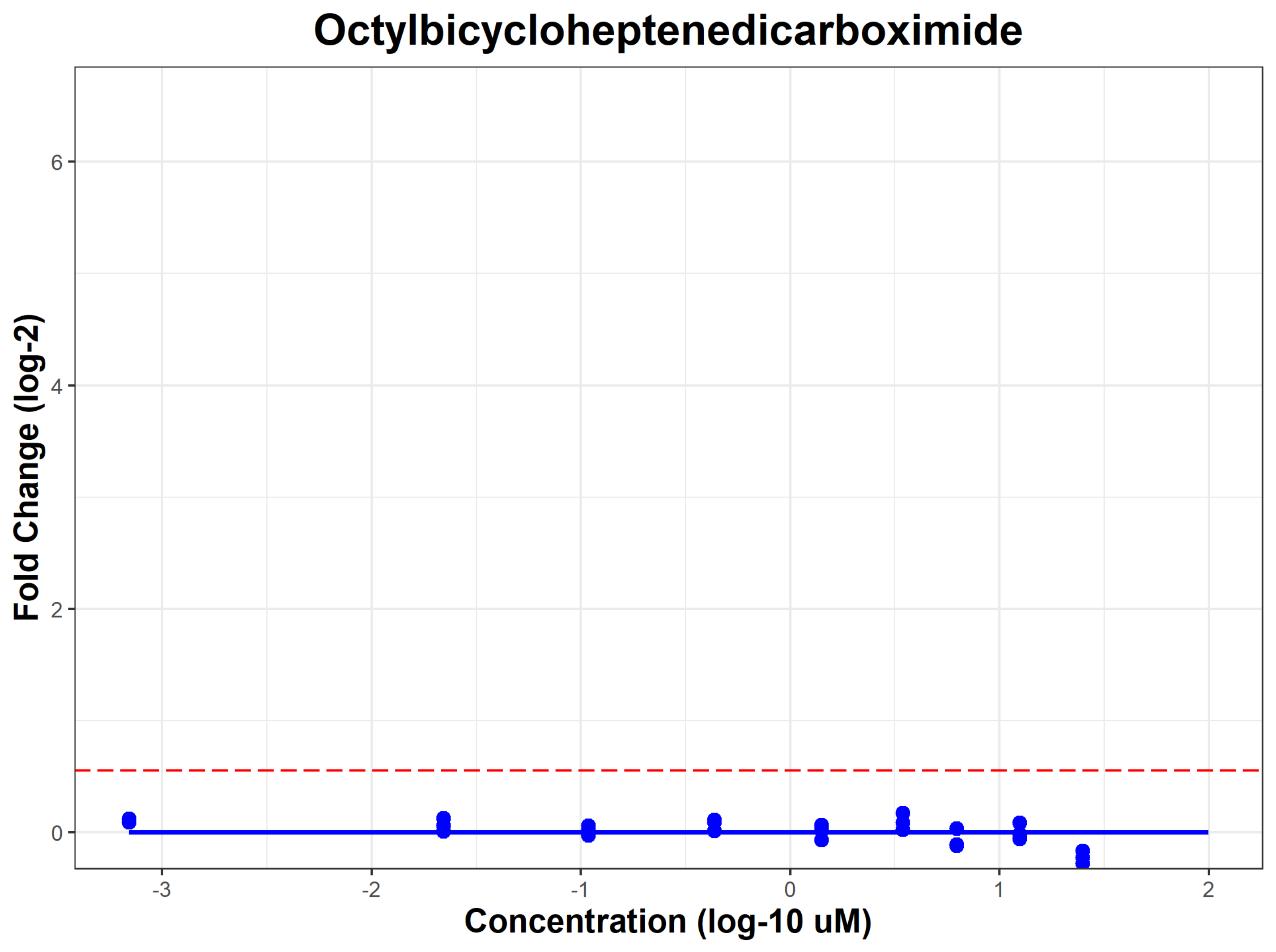

Octylbicycloheptenedicarboximide
Fold Change (log-2)
6
4
2
0
-3
-2
-1
0
1
2
Concentration (log-10 uM)

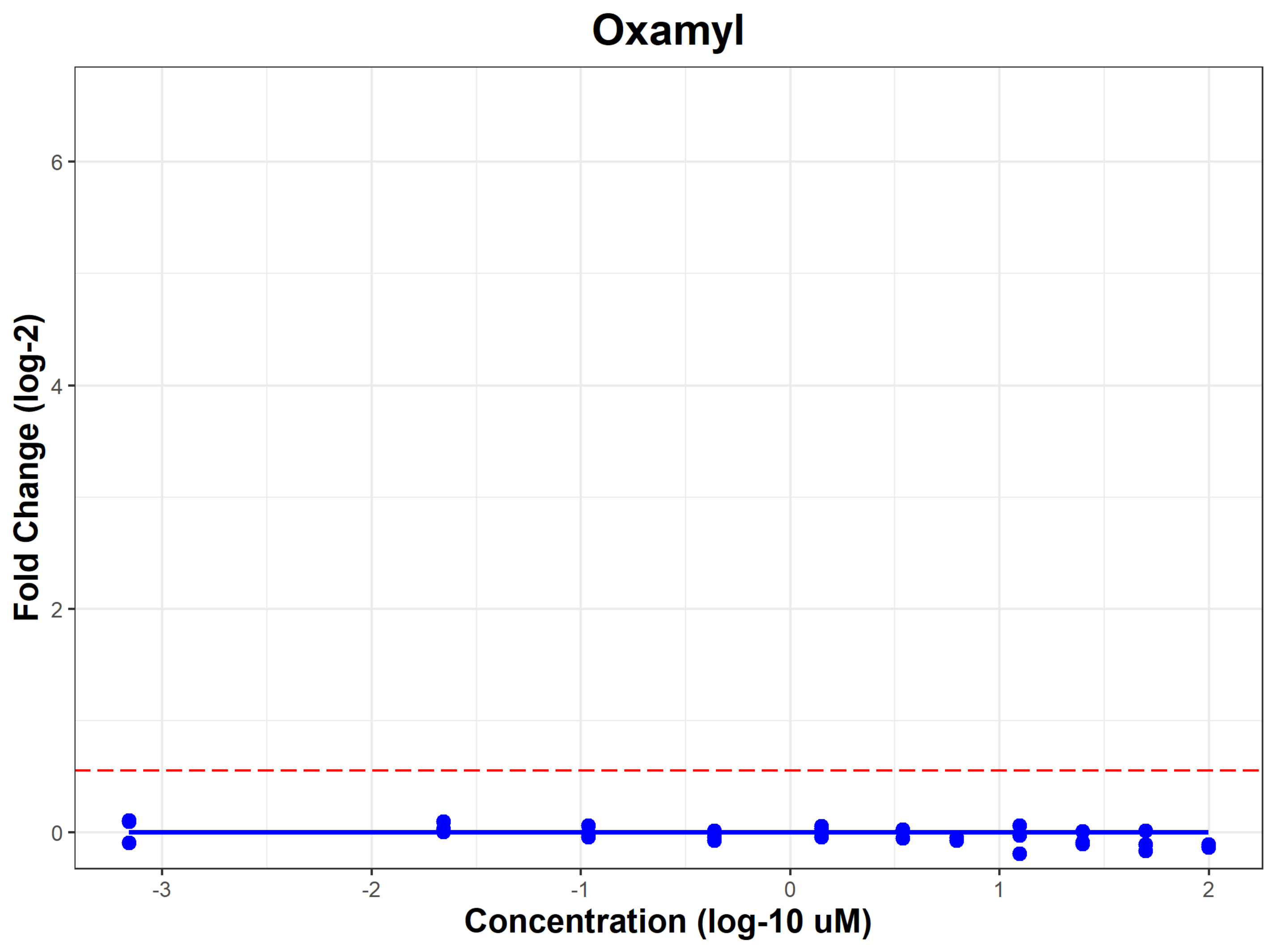

Oxamyl
Fold Change (log-2)
Concentration (log-10 uM)
6
4
2
0
-3
-2
-1
0
1
2

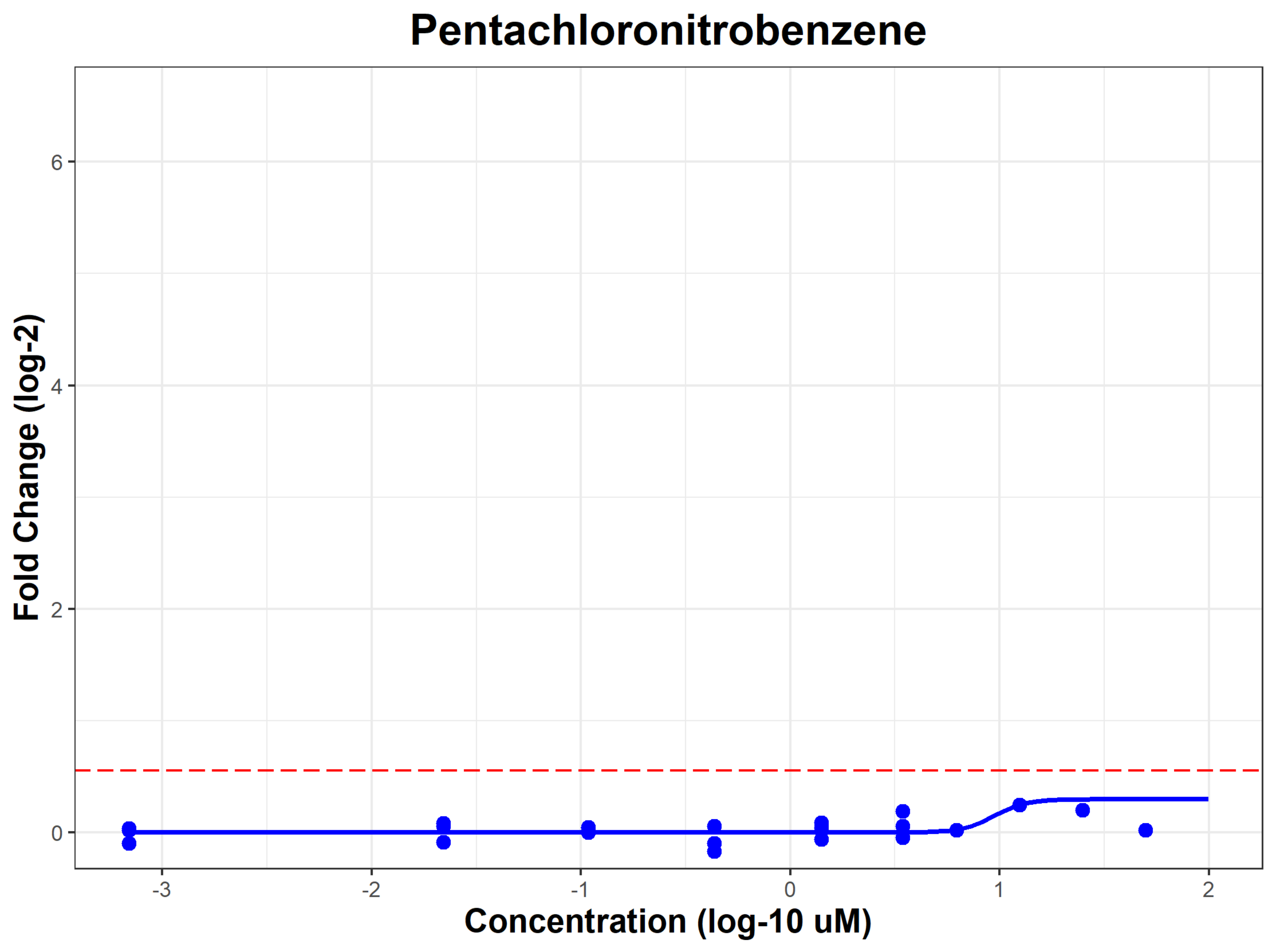

Pentachloronitrobenzene
Fold Change (log-2)
6
4
2
0
-3
-2
-1
0
1
2
Concentration (log-10 uM)

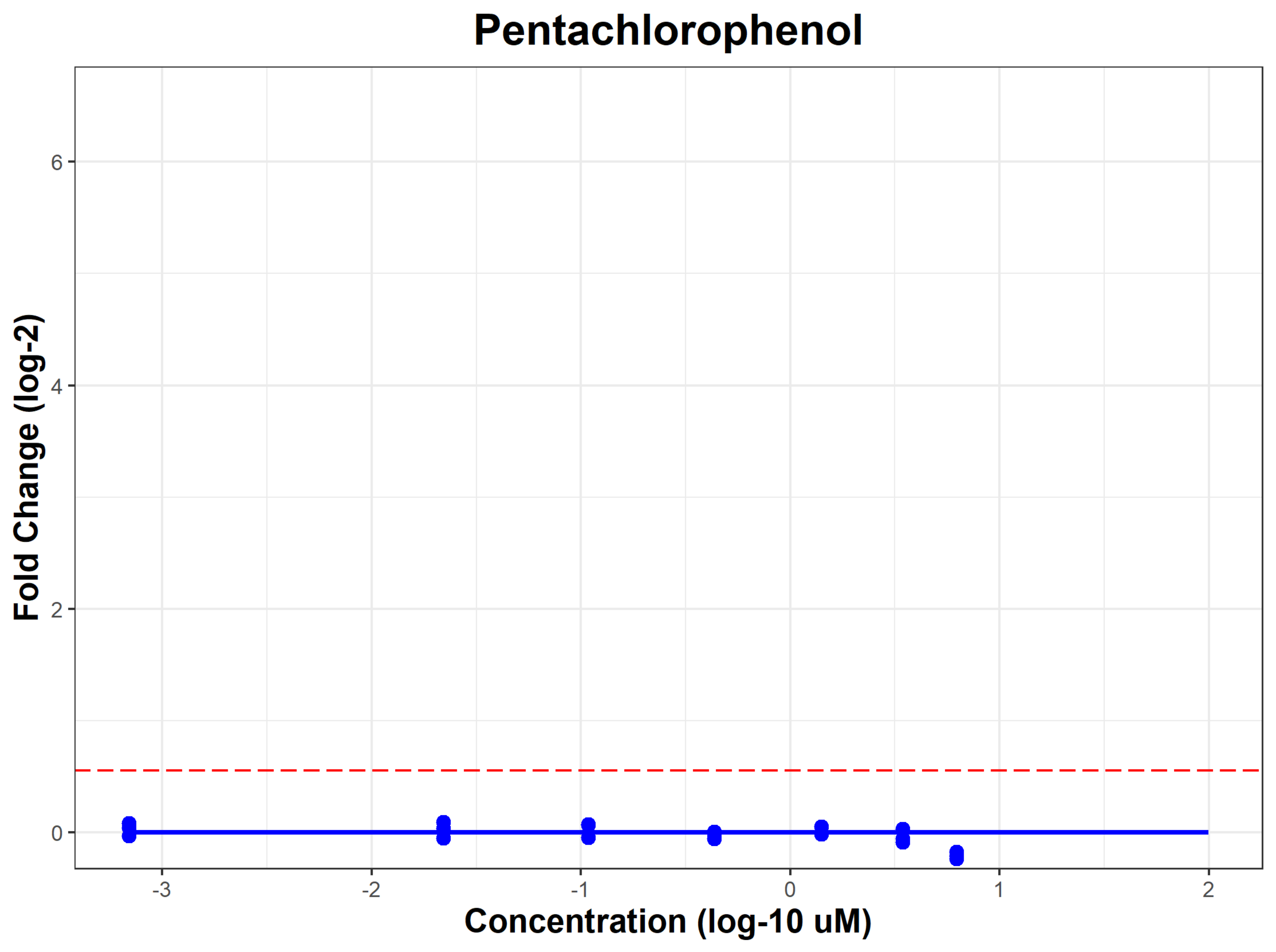
Pentachlorophenol
Fold Change (log-2)
6
4
2
0
-3
-2
-1
0
1
2
Concentration (log-10 uM)

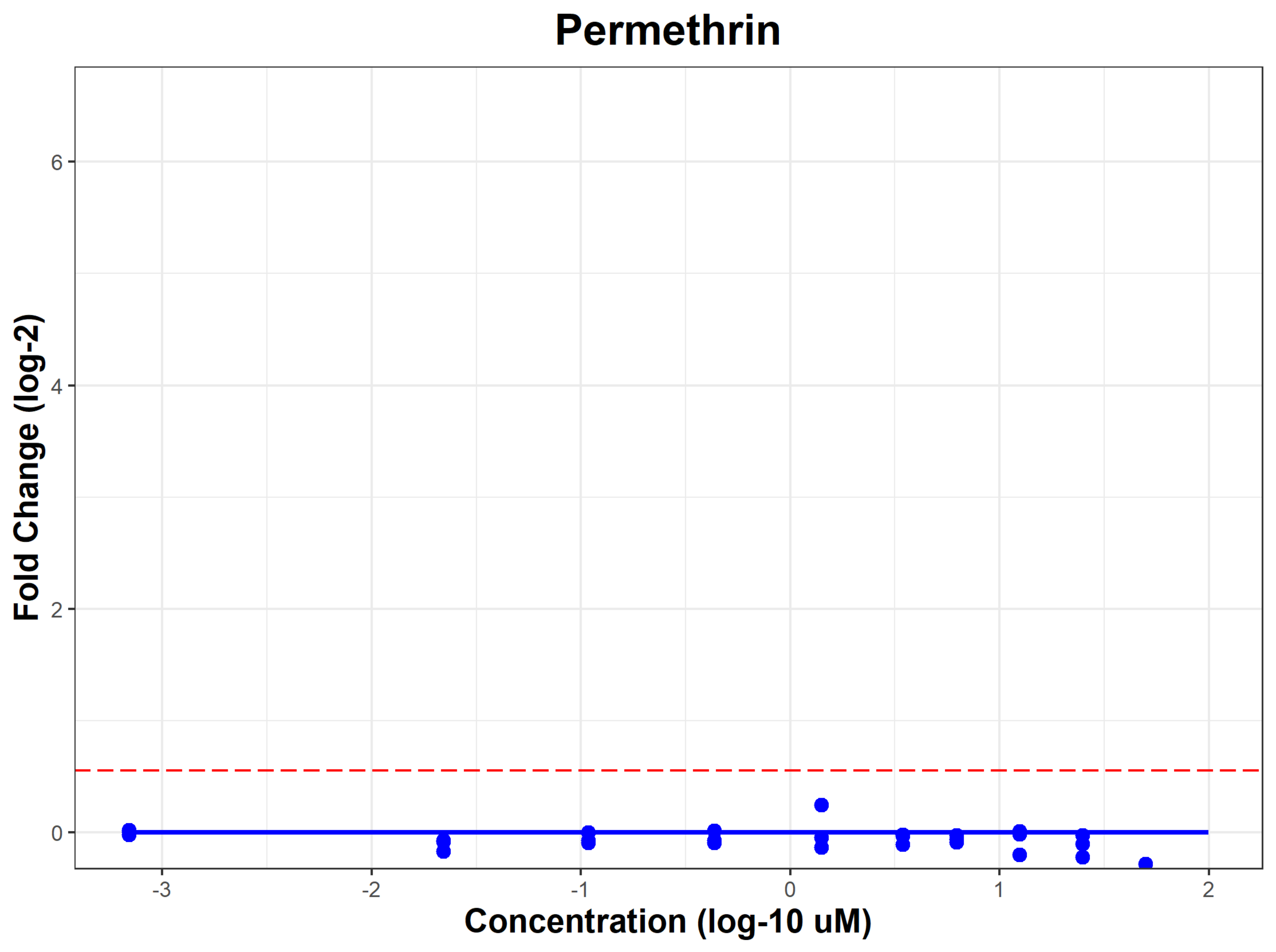

Permethrin
Fold Change (log-2)
0
2
4
6
-3
-2
-1
0
1
2
Concentration (log-10 uM)

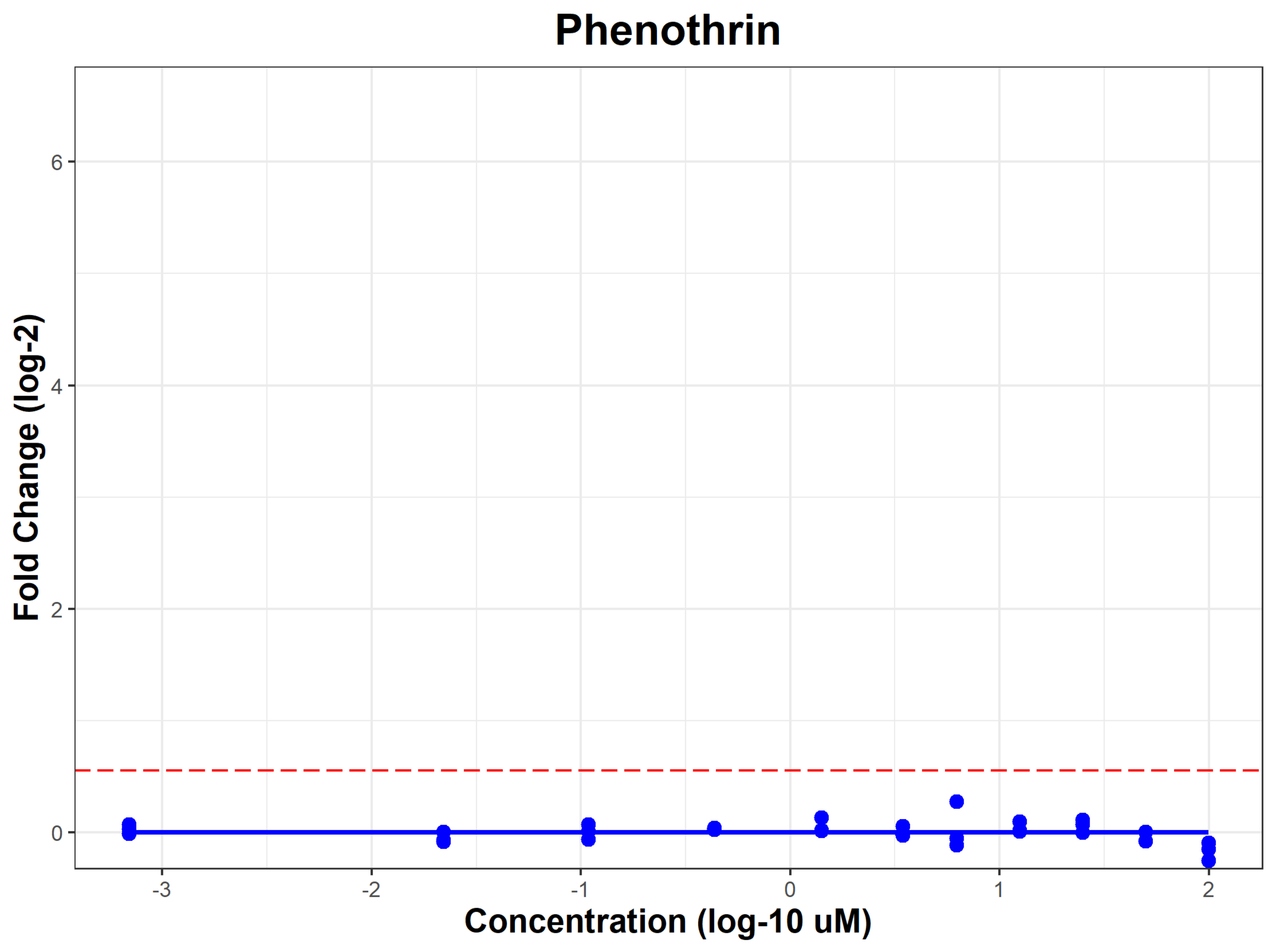

Phenothrin
Fold Change (log-2)
6
4
2
0
-3
-2
-1
0
1
2
Concentration (log-10 uM)

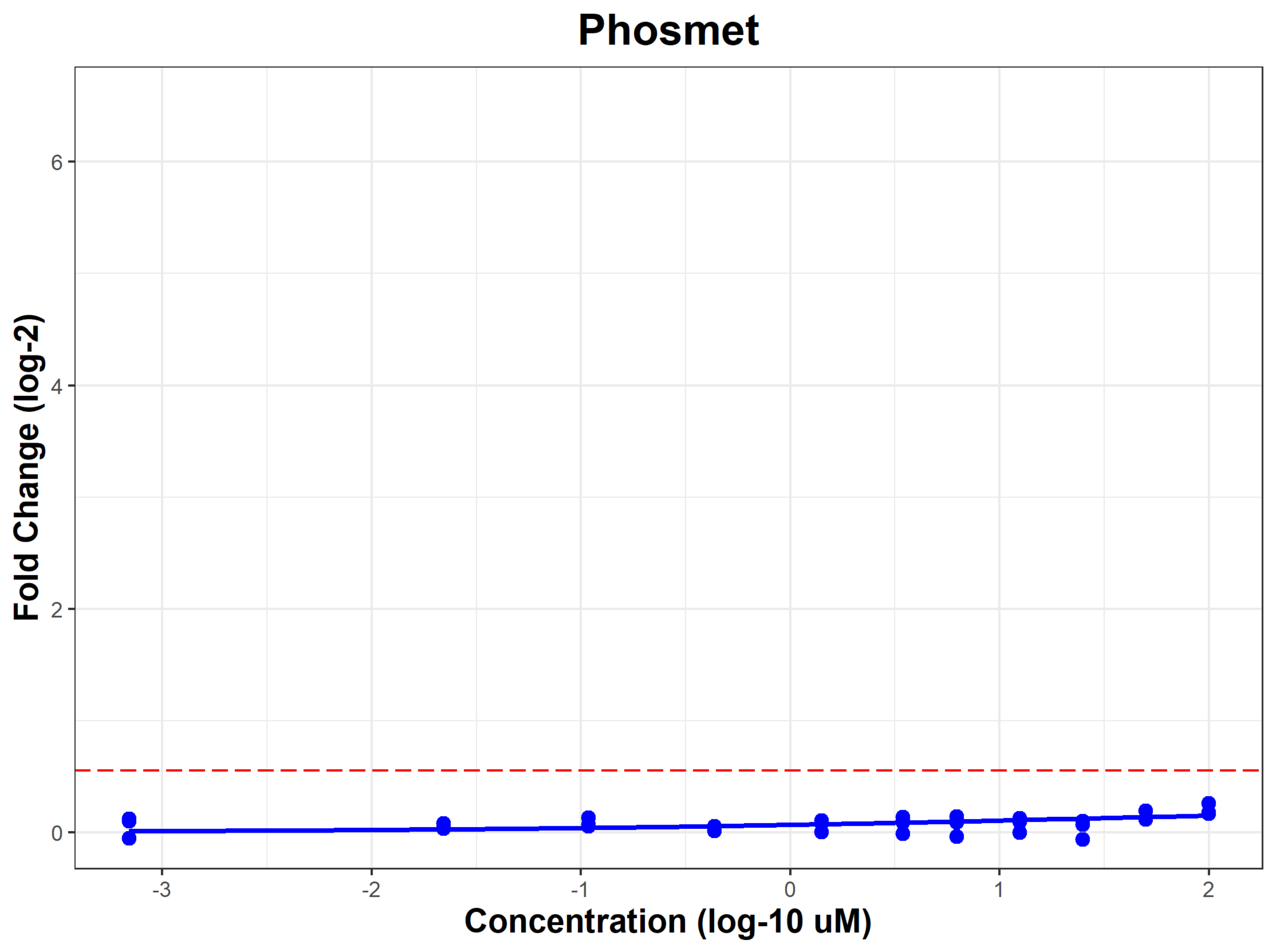

Phosmet
Fold Change (log-2)
6
4
2
0
-3
-2
-1
0
1
2
Concentration (log-10 uM)

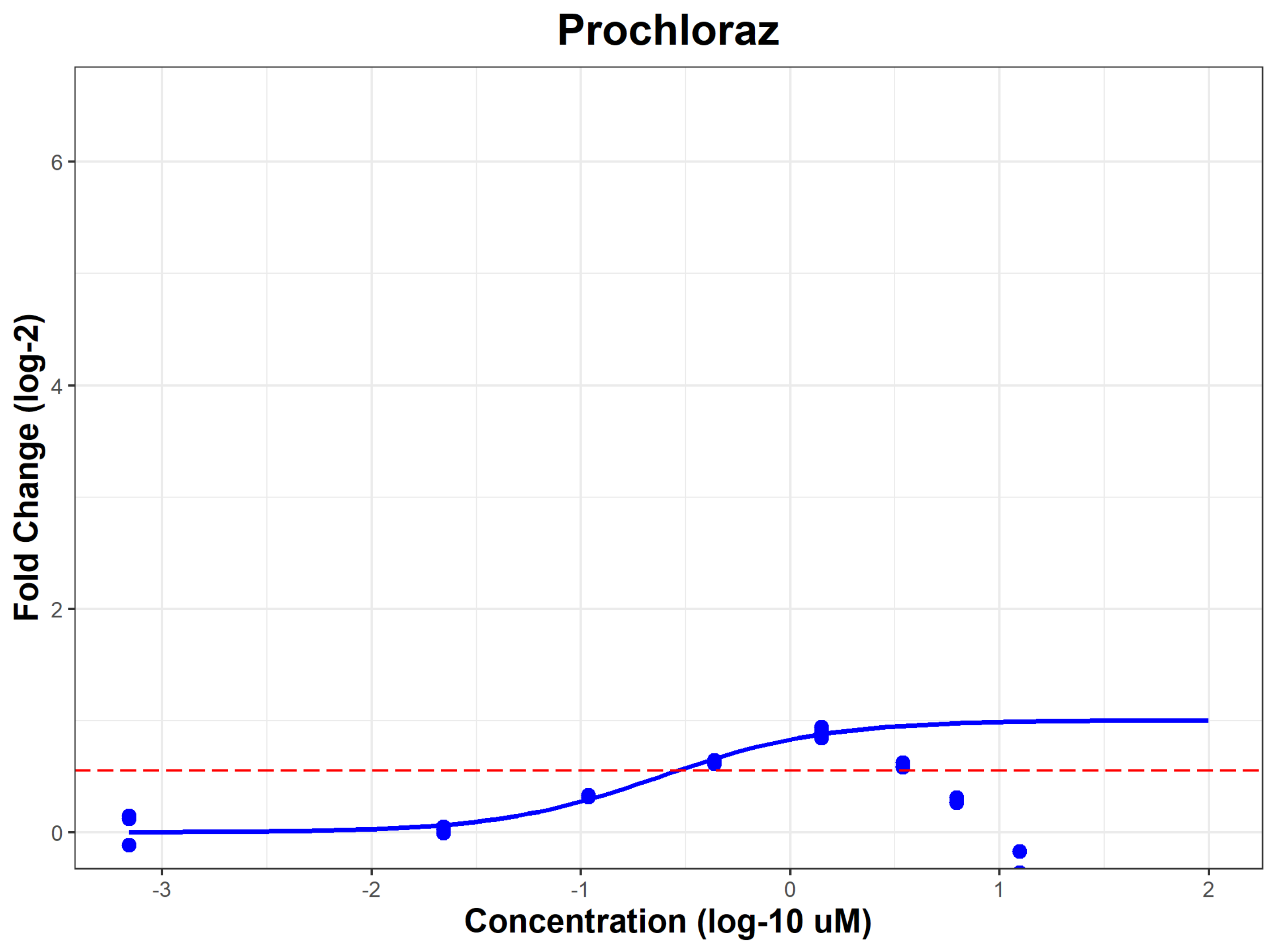

Prochloraz
Fold Change (log-2)
6
4
2
0
-3
-2
-1
0
1
2
Concentration (log-10 uM)

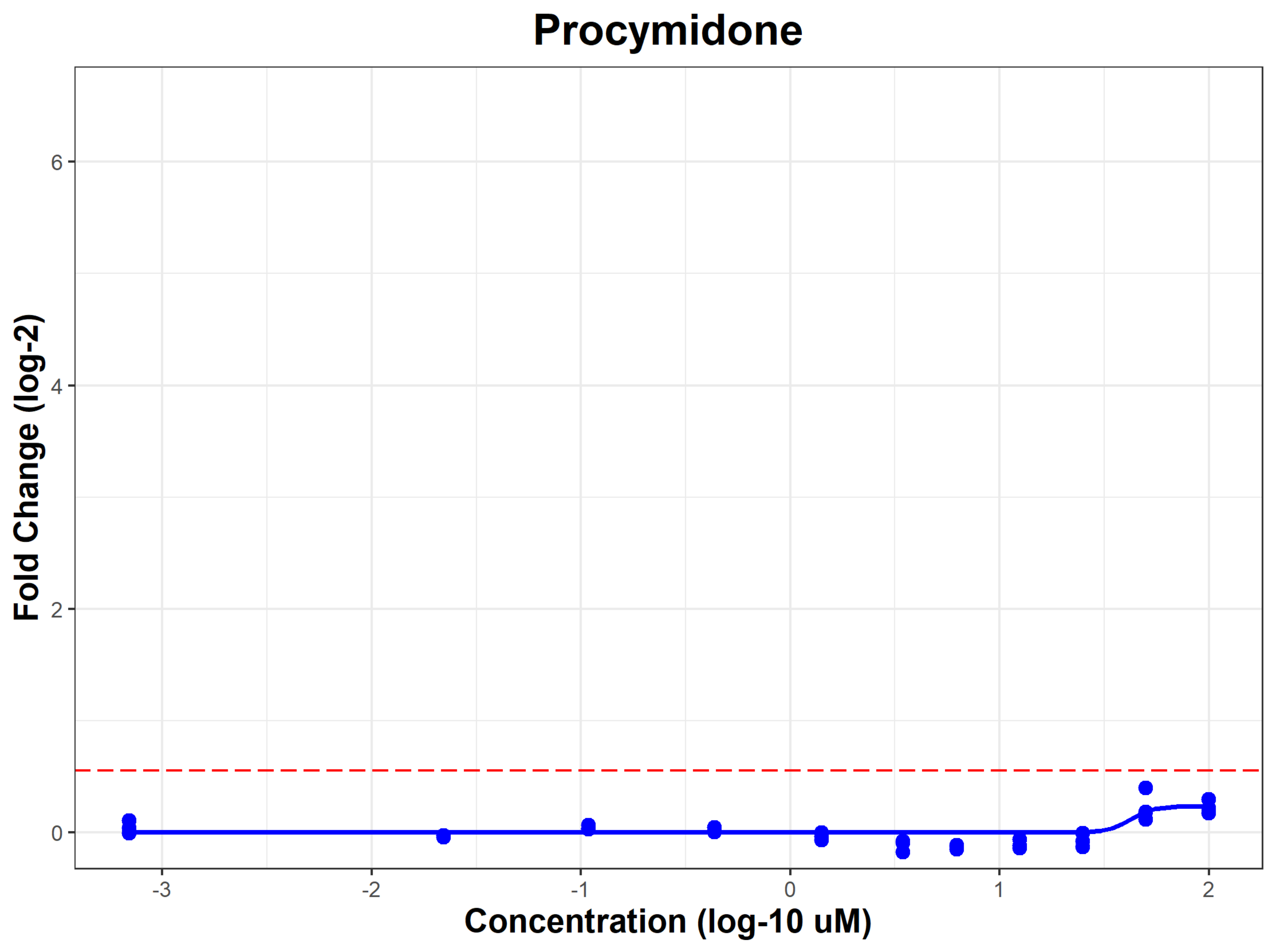

Procymidone
Fold Change (log-2)
6
4
2
0
-3
-2
-1
0
1
2
Concentration (log-10 uM)

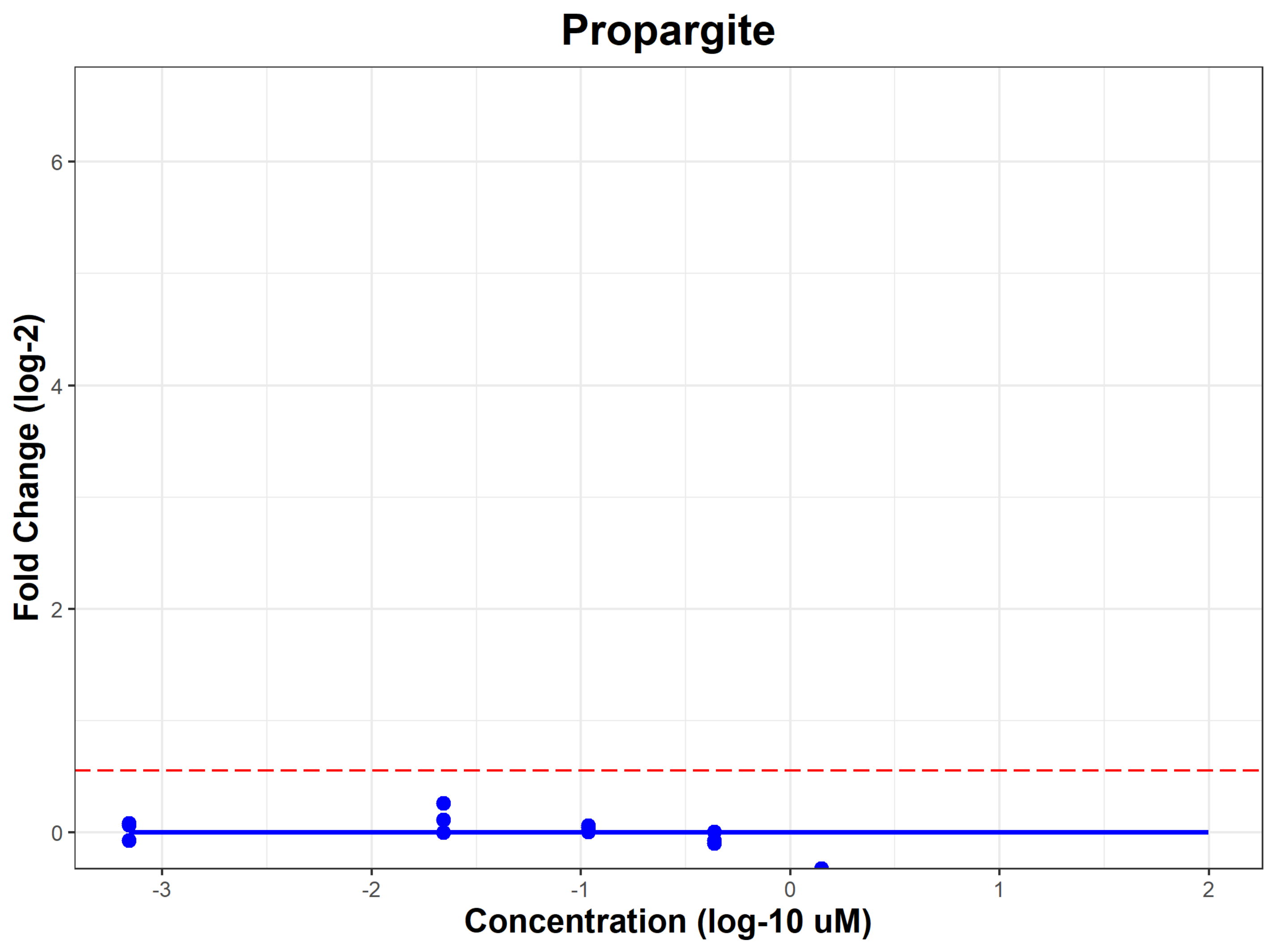

Propargite
Fold Change (log-2)
6
4
2
0
-3
-2
-1
0
1
2
Concentration (log-10 uM)

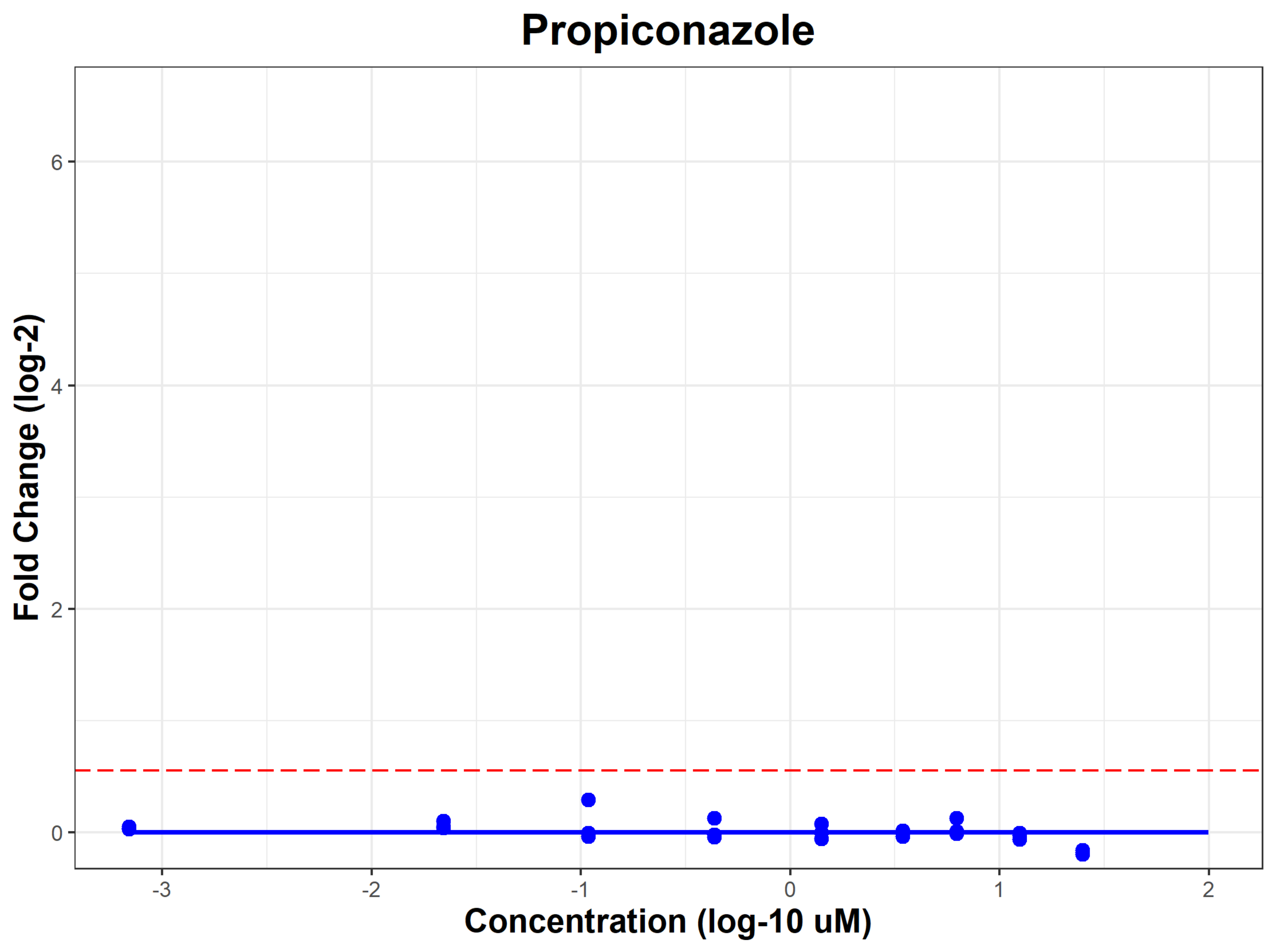
Propiconazole
Fold Change (log-2)
6
4
2
0
-3
-2
-1
0
1
2
Concentration (log-10 uM)

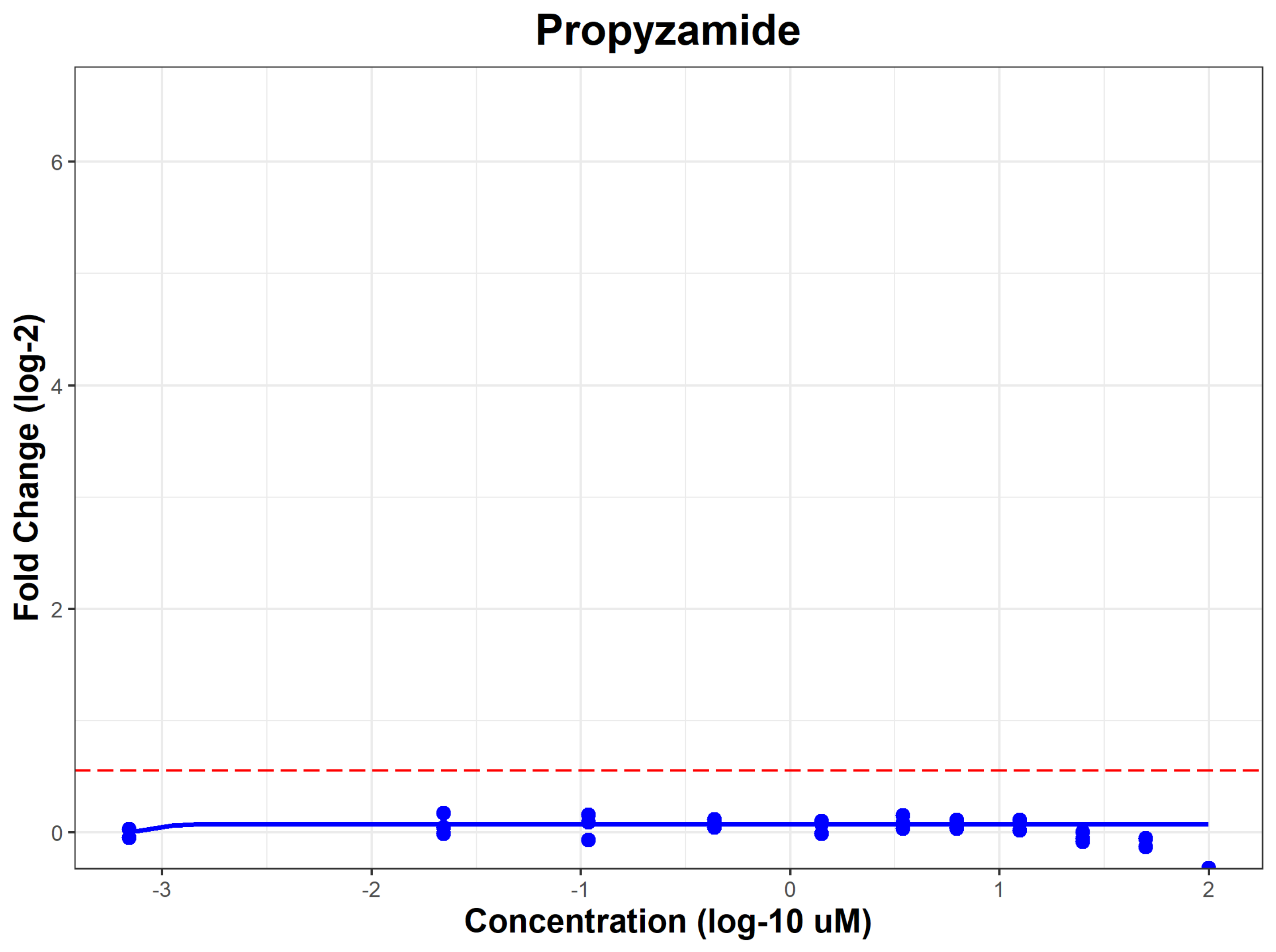
Propyzamide
Fold Change (log-2)
6
4
2
0
-3
-2
-1
0
1
2
Concentration (log-10 uM)

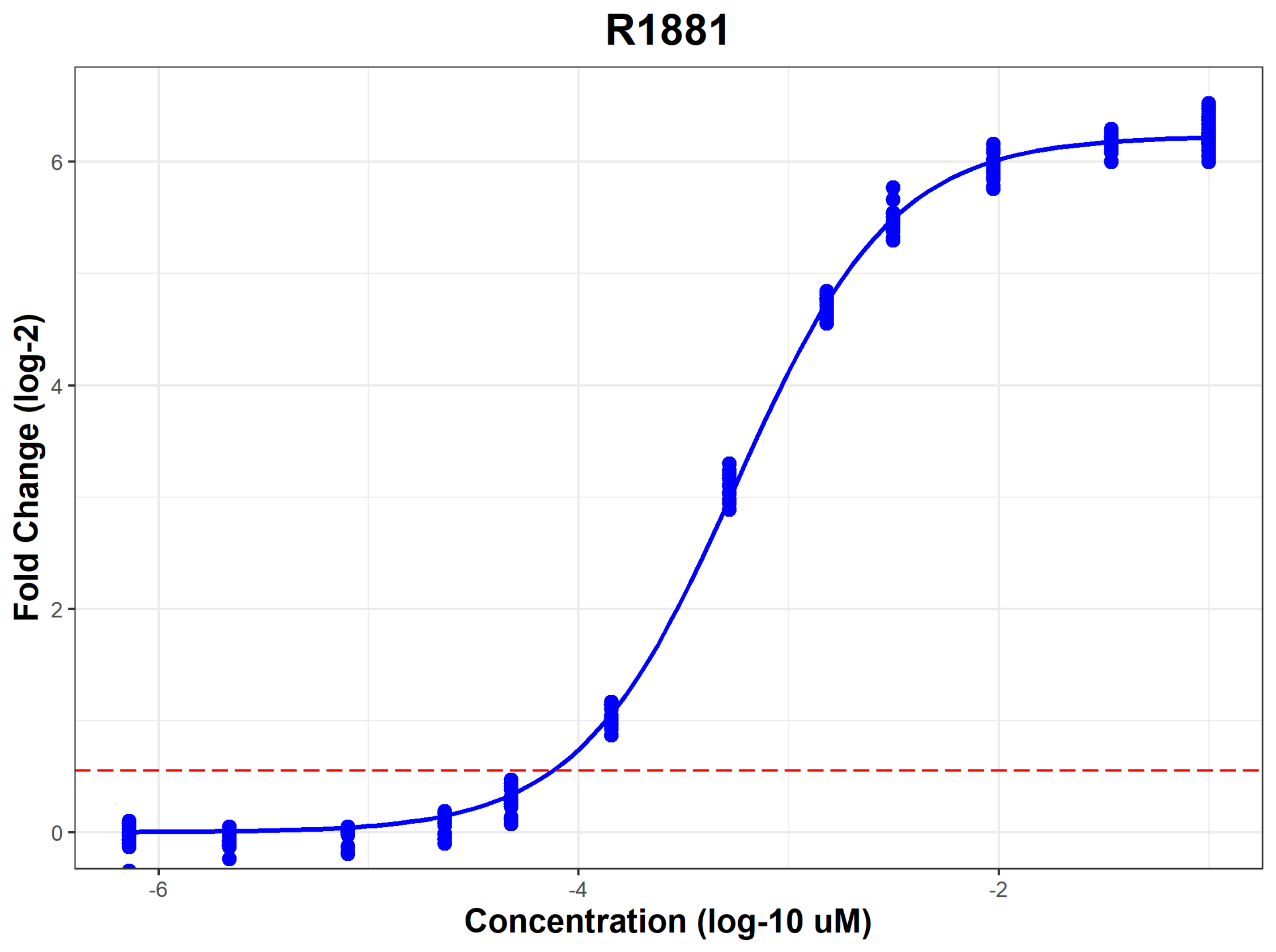
R1881
Fold Change (log-2)
Concentration (log-10 uM)
6
4
2
0
-6
-4
-2

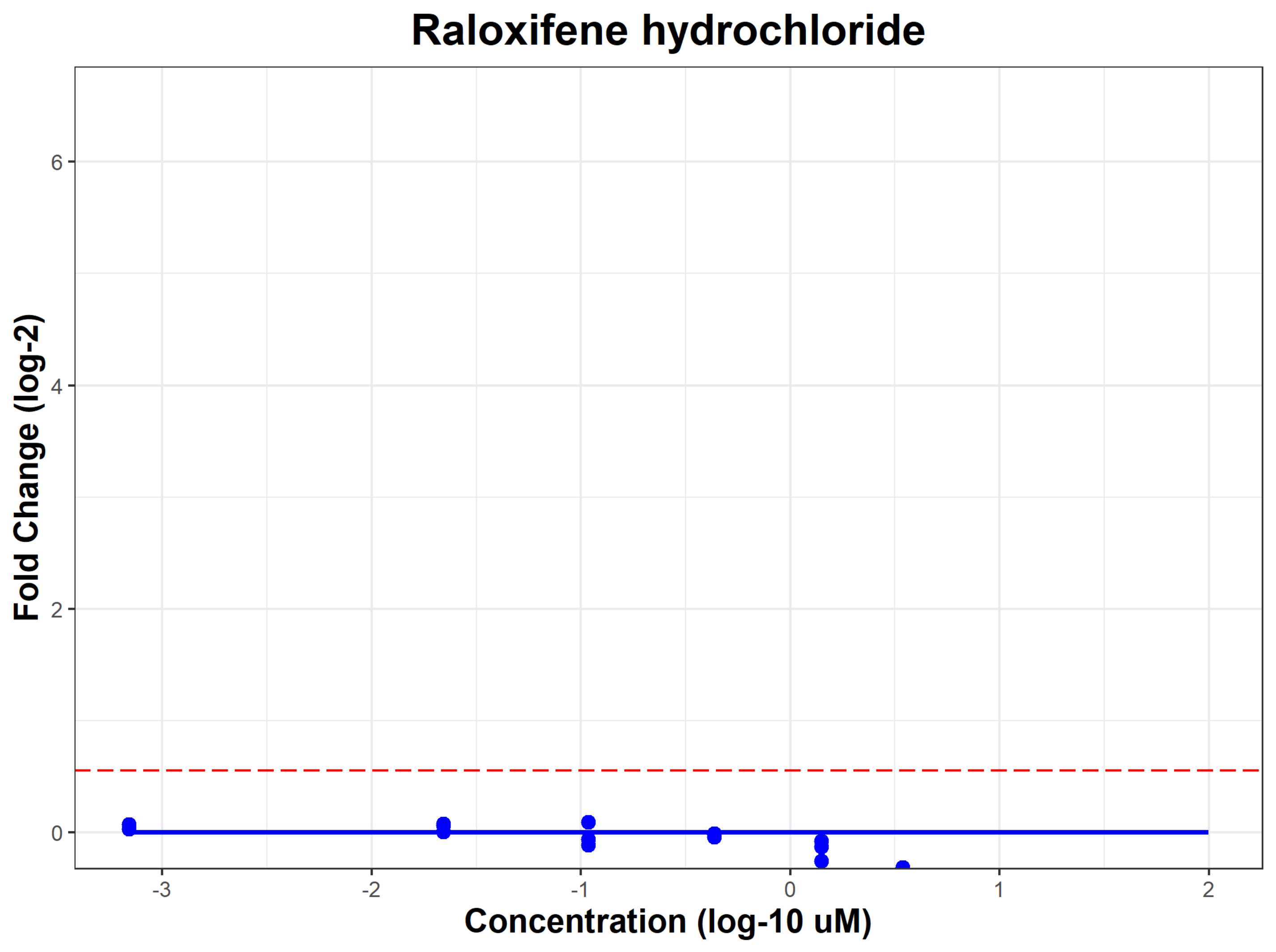
Raloxifene hydrochloride
Fold Change (log-2)
Concentration (log-10 uM)
6
4
2
0
-3
-2
-1
0
1
2

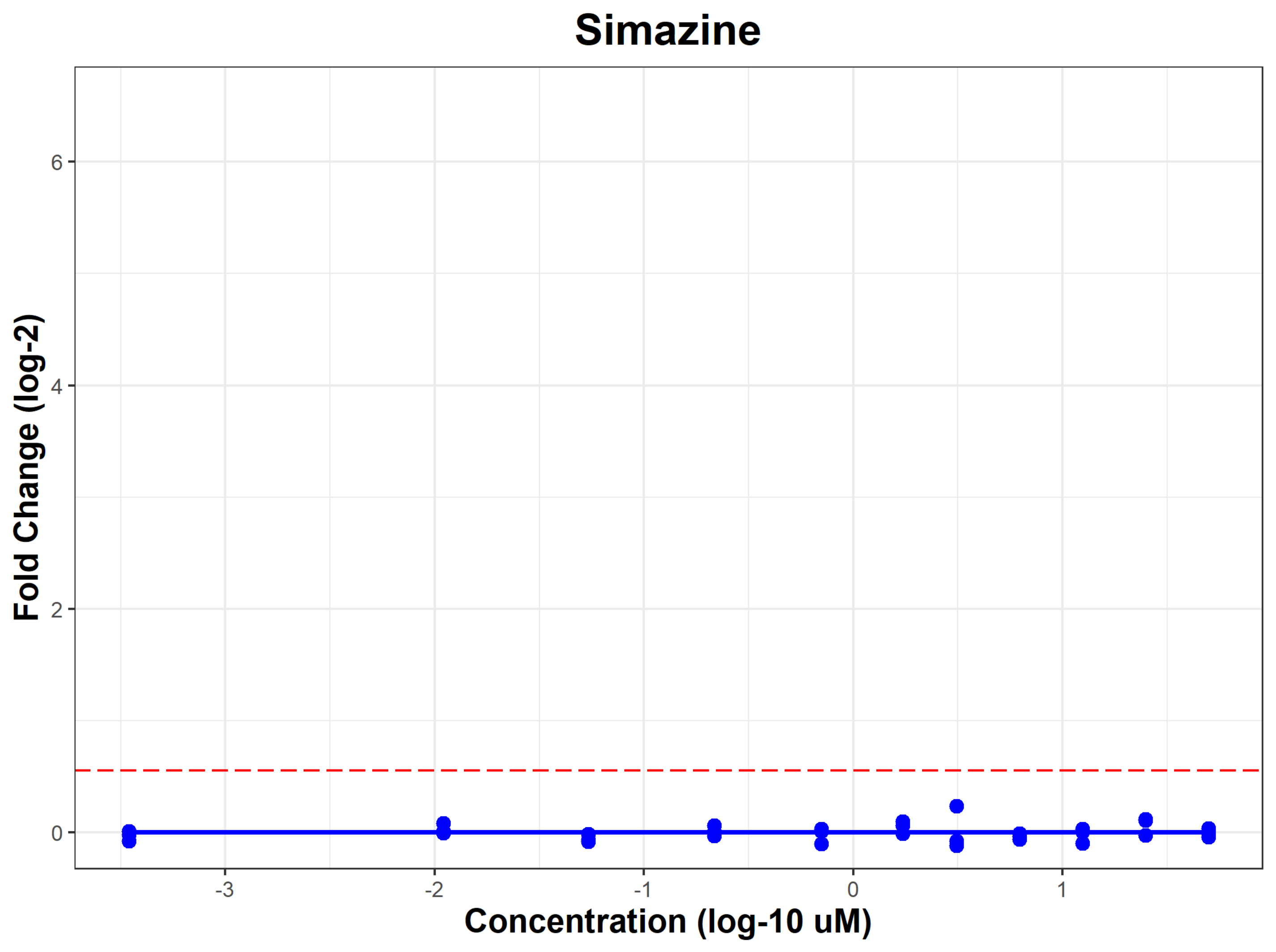

Simazine
Fold Change (log-2)
6
4
2
0
-3
-2
-1
0
1
Concentration (log-10 uM)

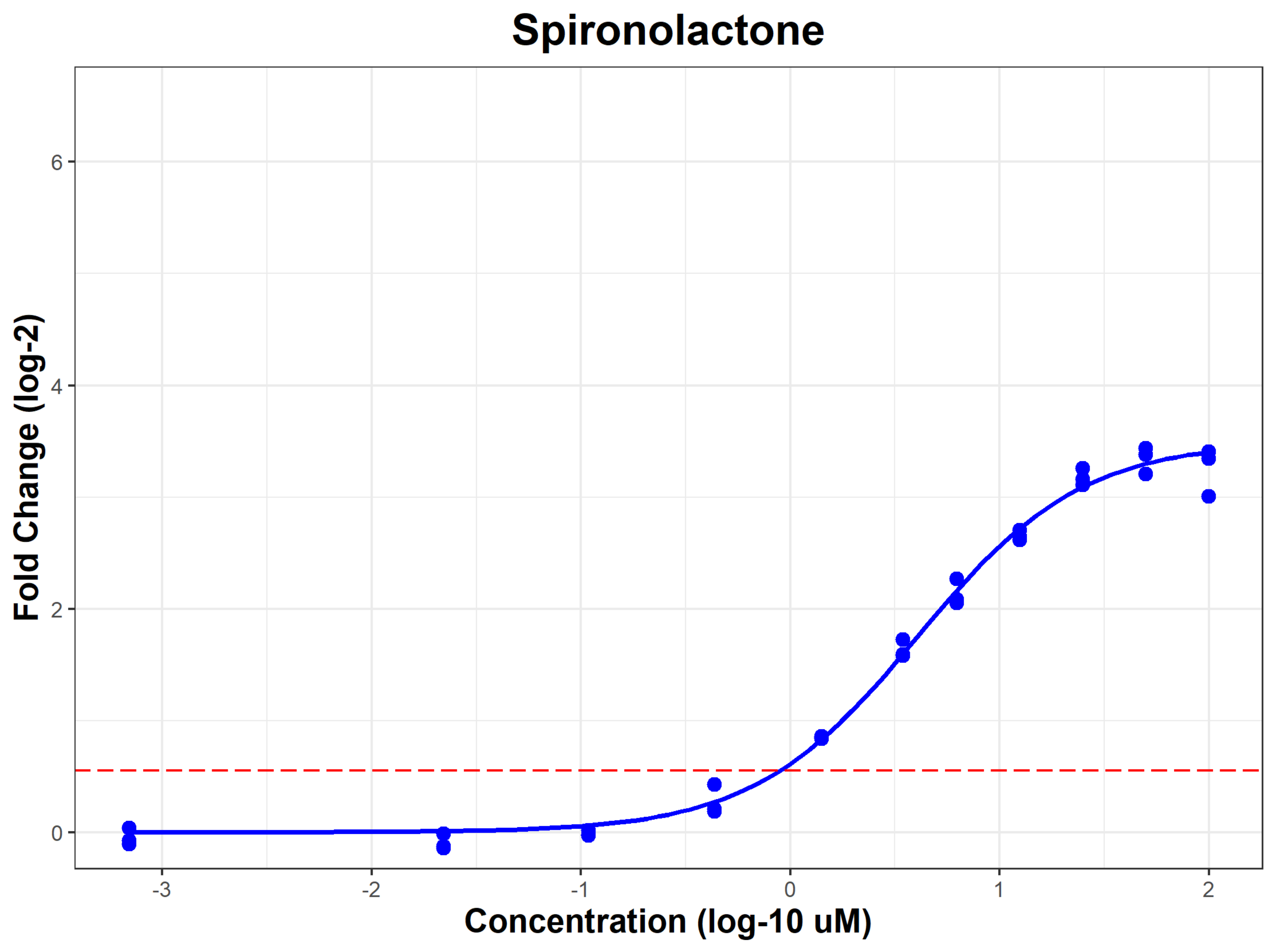

Spironolactone
Fold Change (log-2)
0
2
4
6
-3
-2
-1
0
1
2
Concentration (log-10 uM)

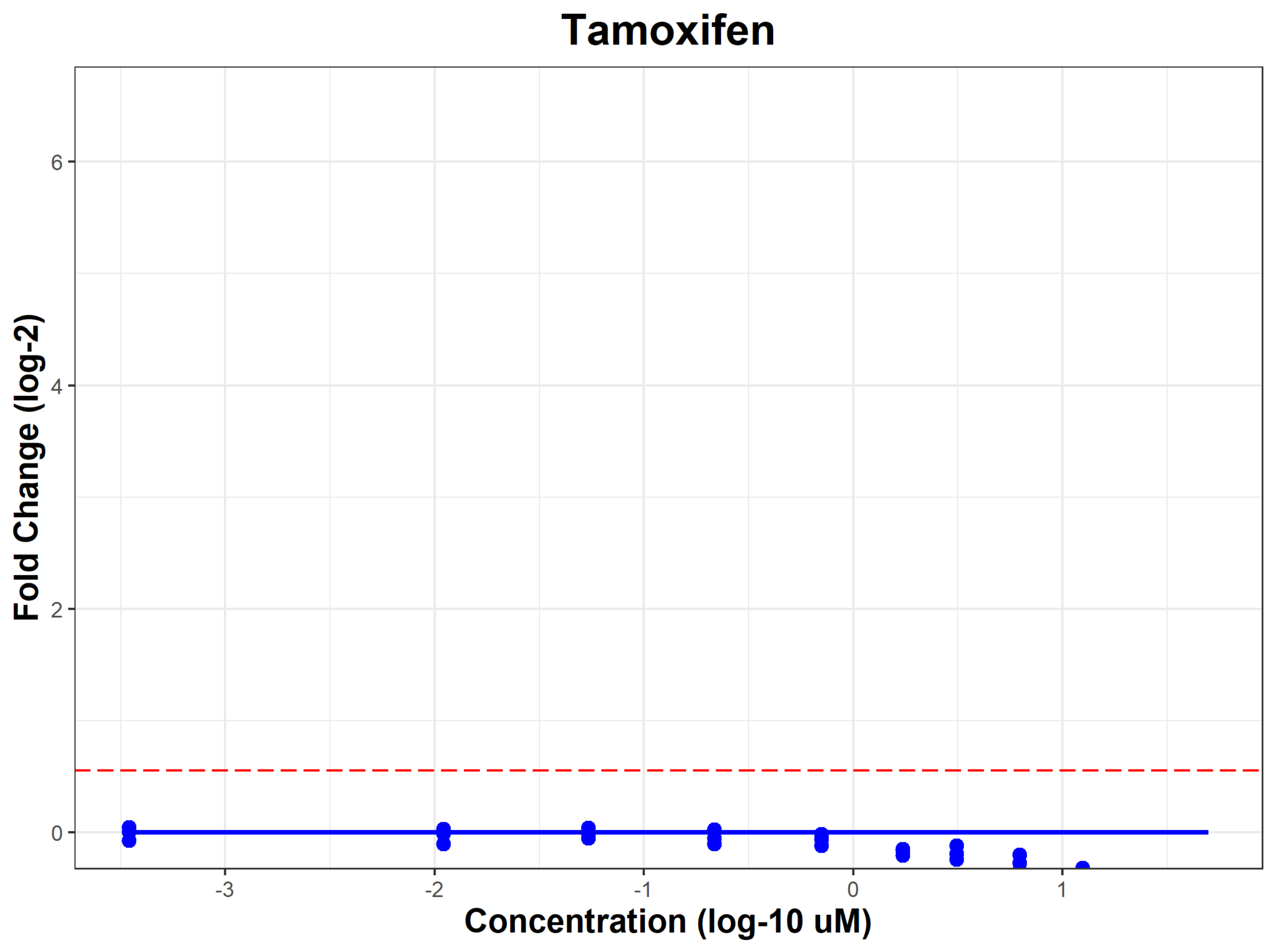

Tamoxifen
Fold Change (log-2)
6
4
2
0
-3
-2
-1
0
1
Concentration (log-10 uM)

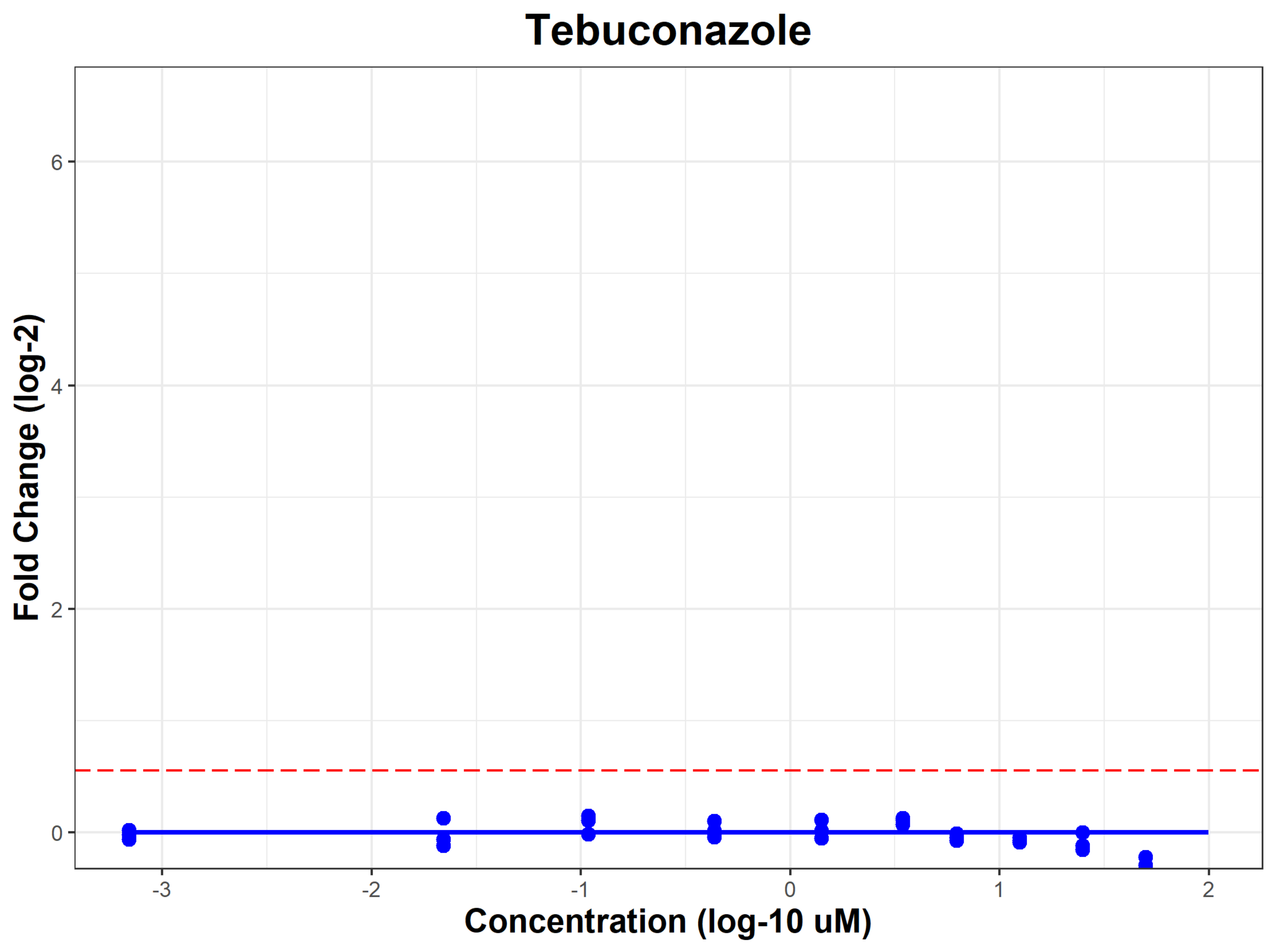
Tebuconazole
Fold Change (log-2)
6
4
2
0
-3
-2
-1
0
1
2
Concentration (log-10 uM)

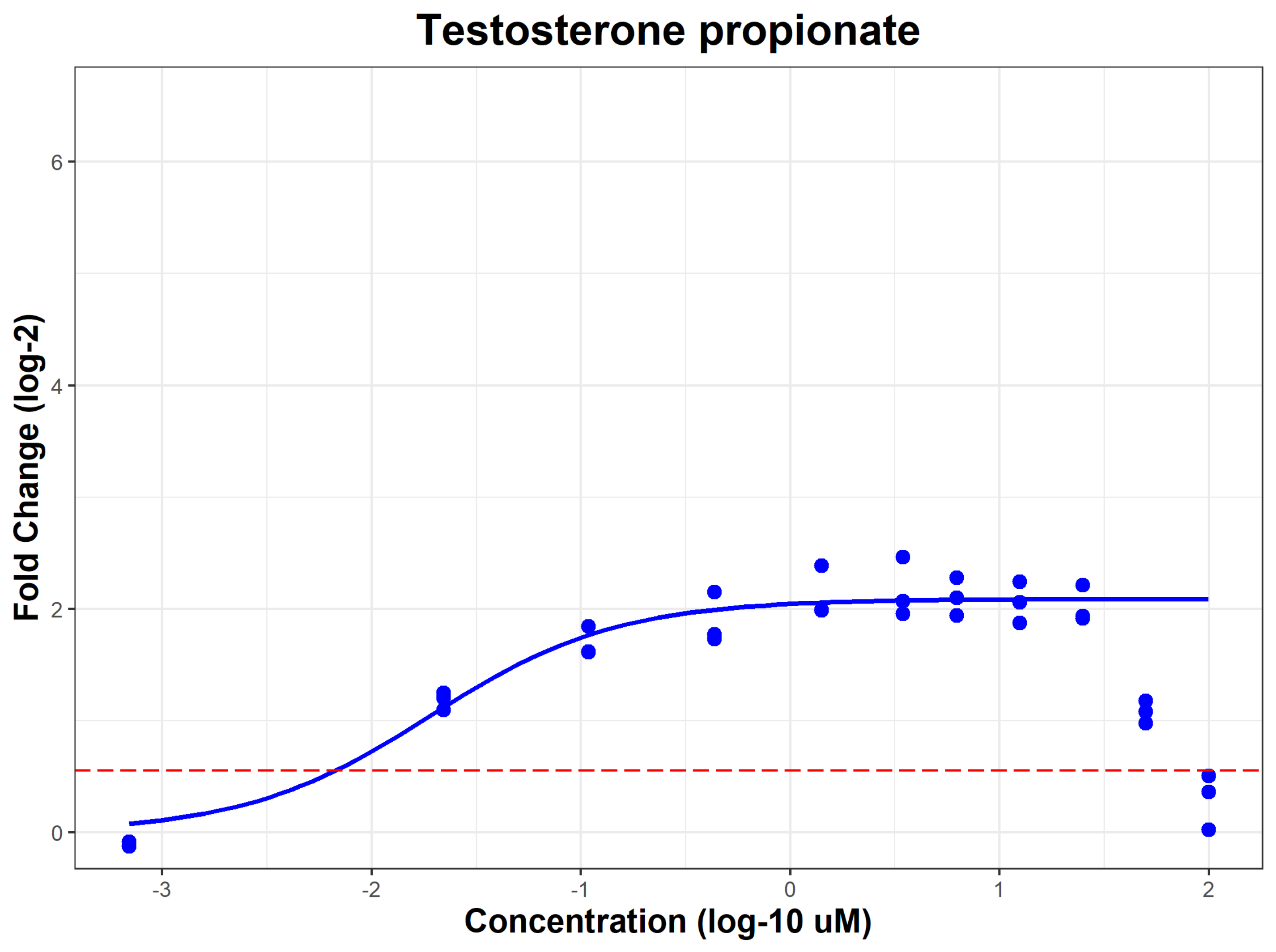

Testosterone propionate
Fold Change (log-2)
Concentration (log-10 uM)
6
4
2
0
-3
-2
-1
0
1
2

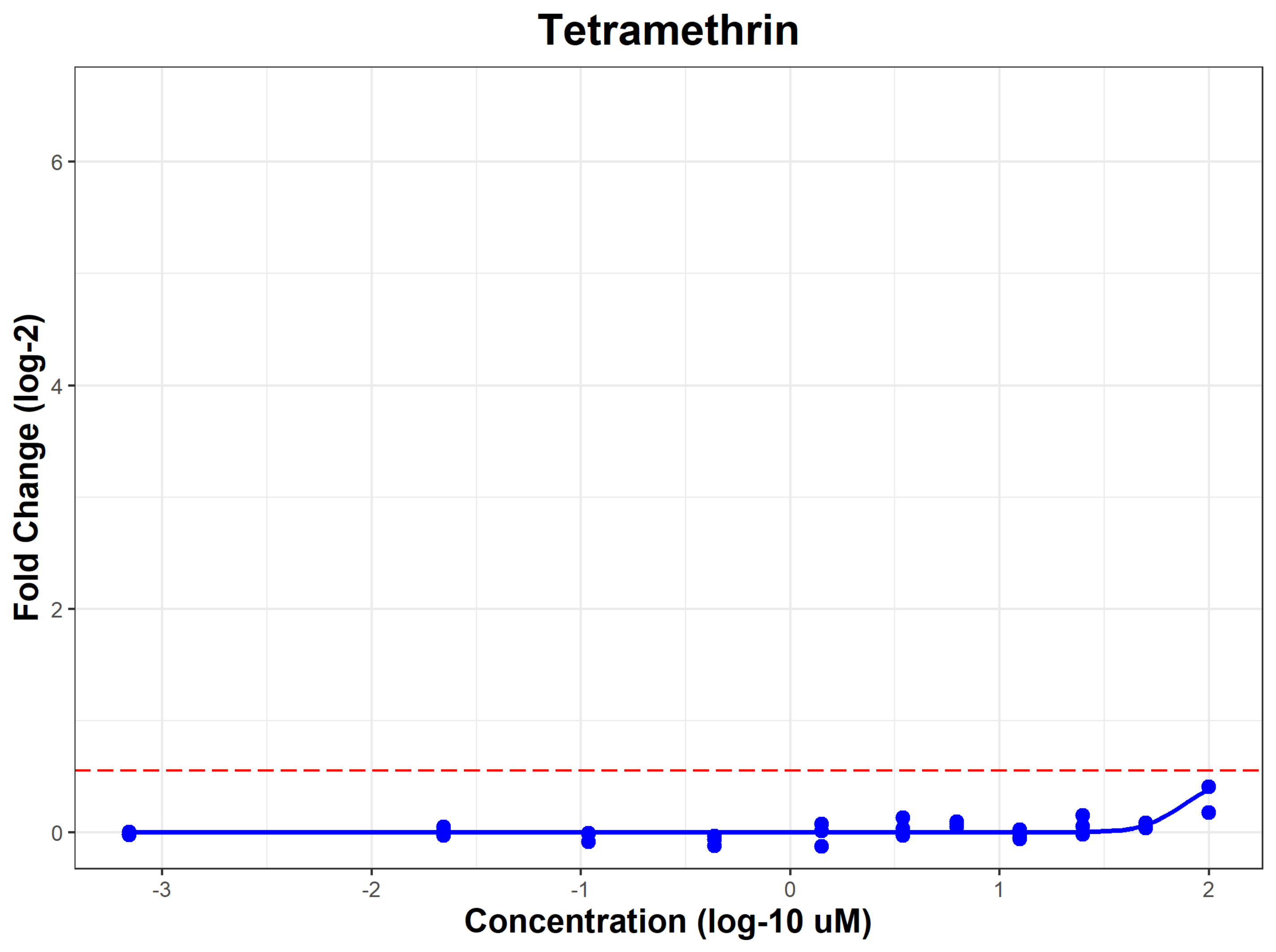

Tetramethrin
Fold Change (log-2)
6
4
2
0
-3
-2
-1
0
1
2
Concentration (log-10 uM)

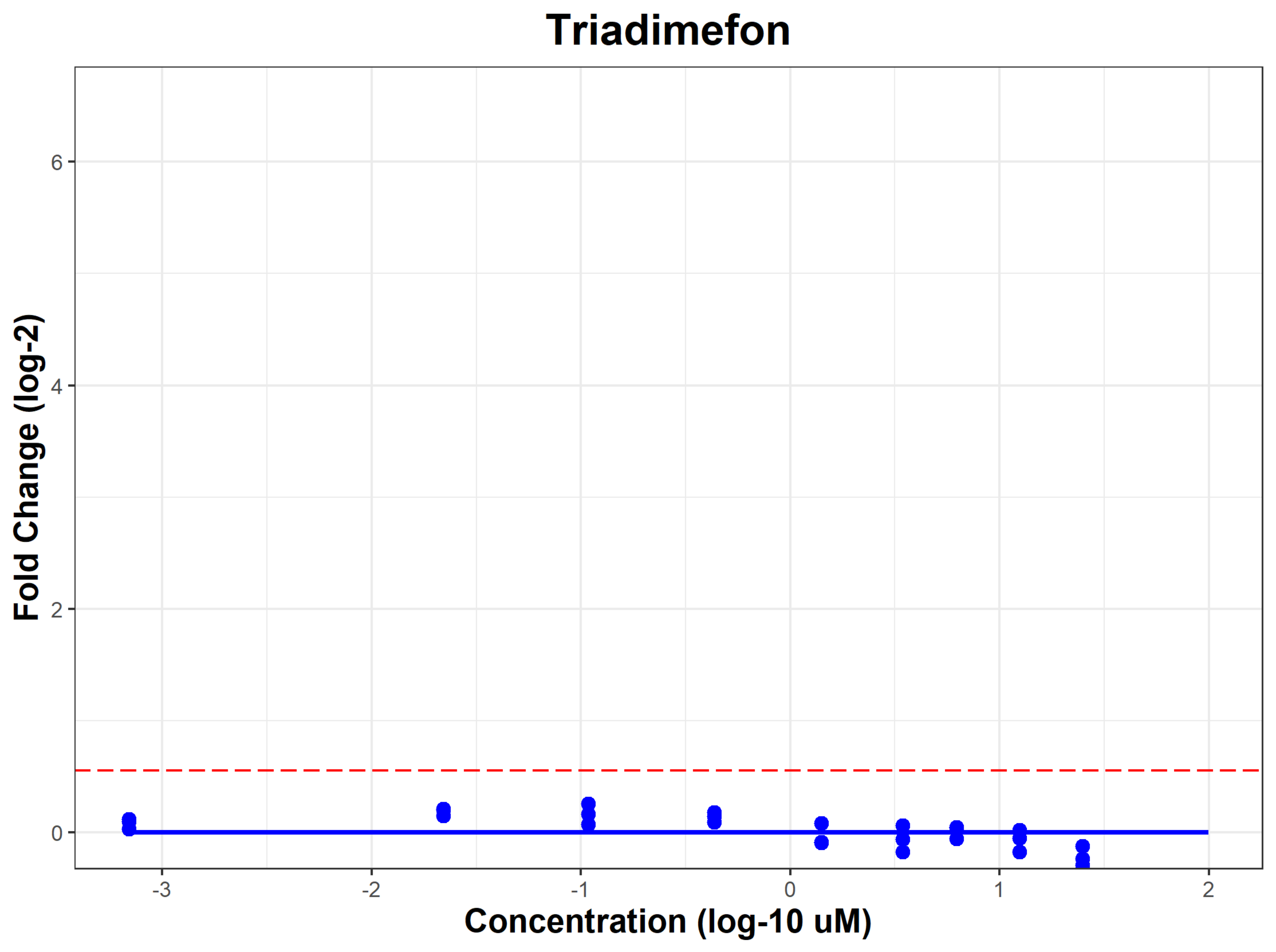
Triadimefon
Fold Change (log-2)
6
4
2
0
-3
-2
-1
0
1
2
Concentration (log-10 uM)

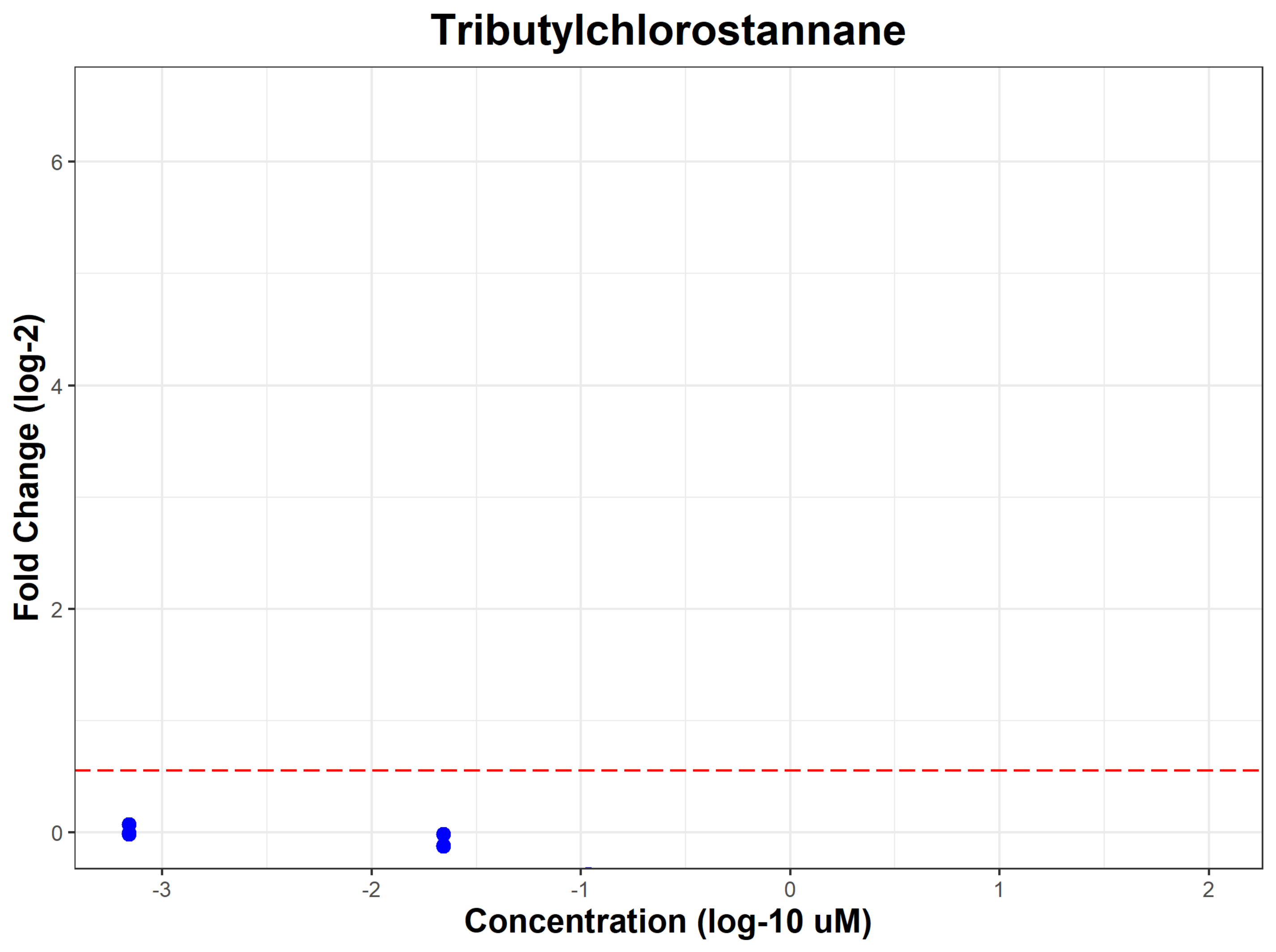
Tributylchlorostannane
Fold Change (log-2)
6
4
2
0
-3
-2
-1
0
1
2
Concentration (log-10 uM)

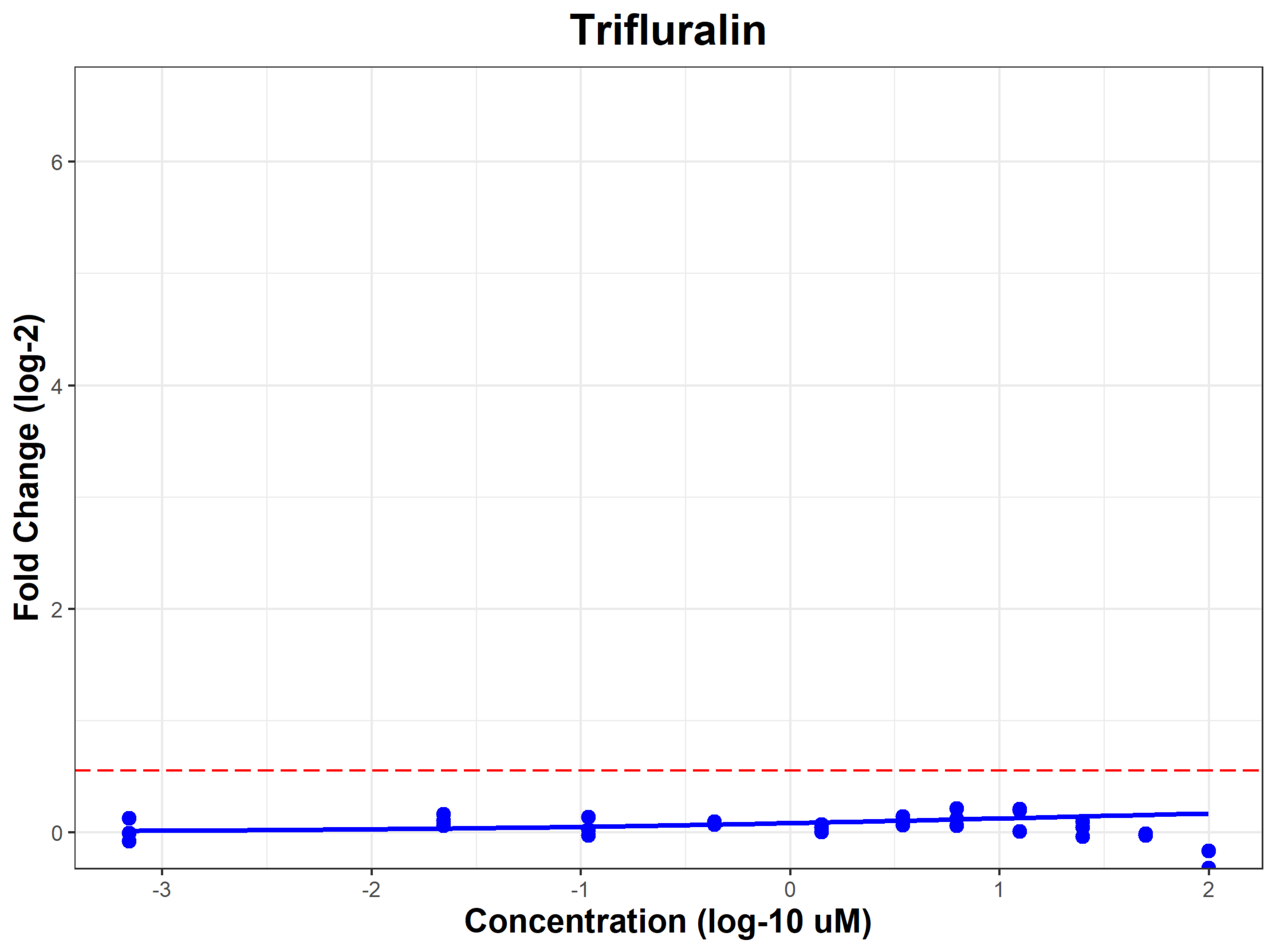

Trifluralin
Fold Change (log-2)
6
4
2
0
-3
-2
-1
0
1
2
Concentration (log-10 uM)

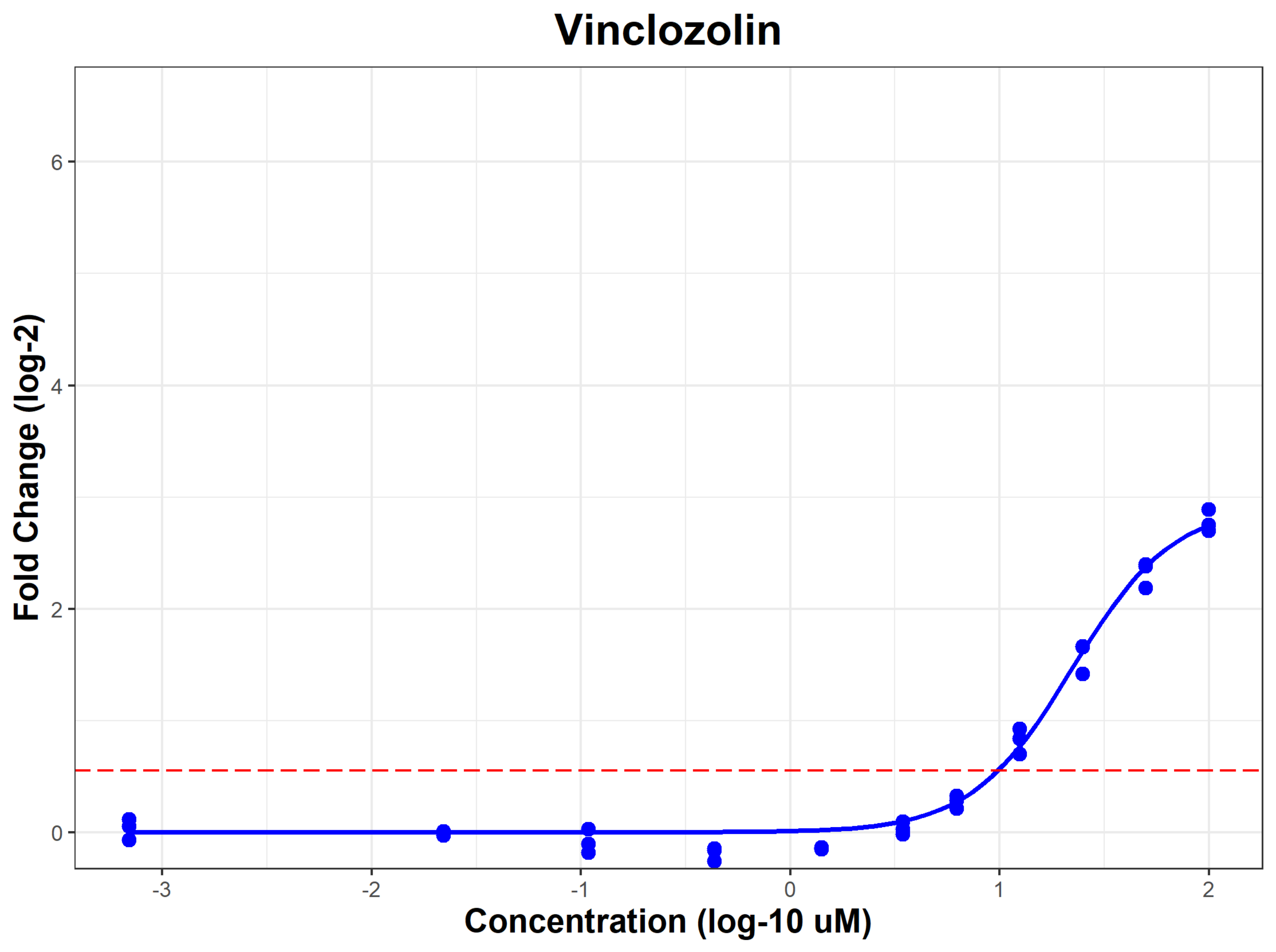
Vinclozolin
Fold Change (log-2)
0
2
4
6
-3
-2
-1
0
1
2
Concentration (log-10 uM)

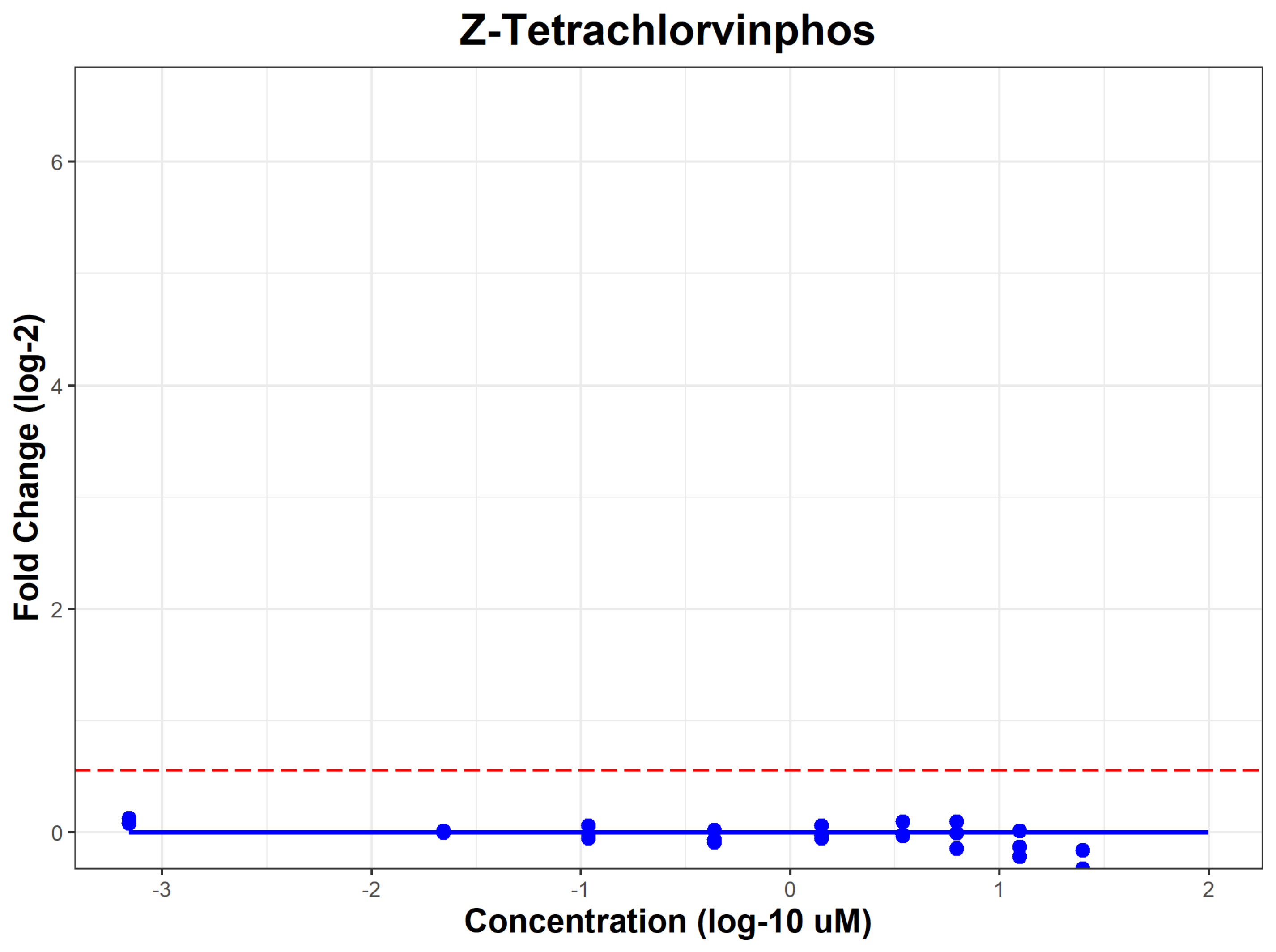

Z-Tetrachlorvinphos
Fold Change (log-2)
Concentration (log-10 uM)
6
4
2
0
-3
-2
-1
0
1
2

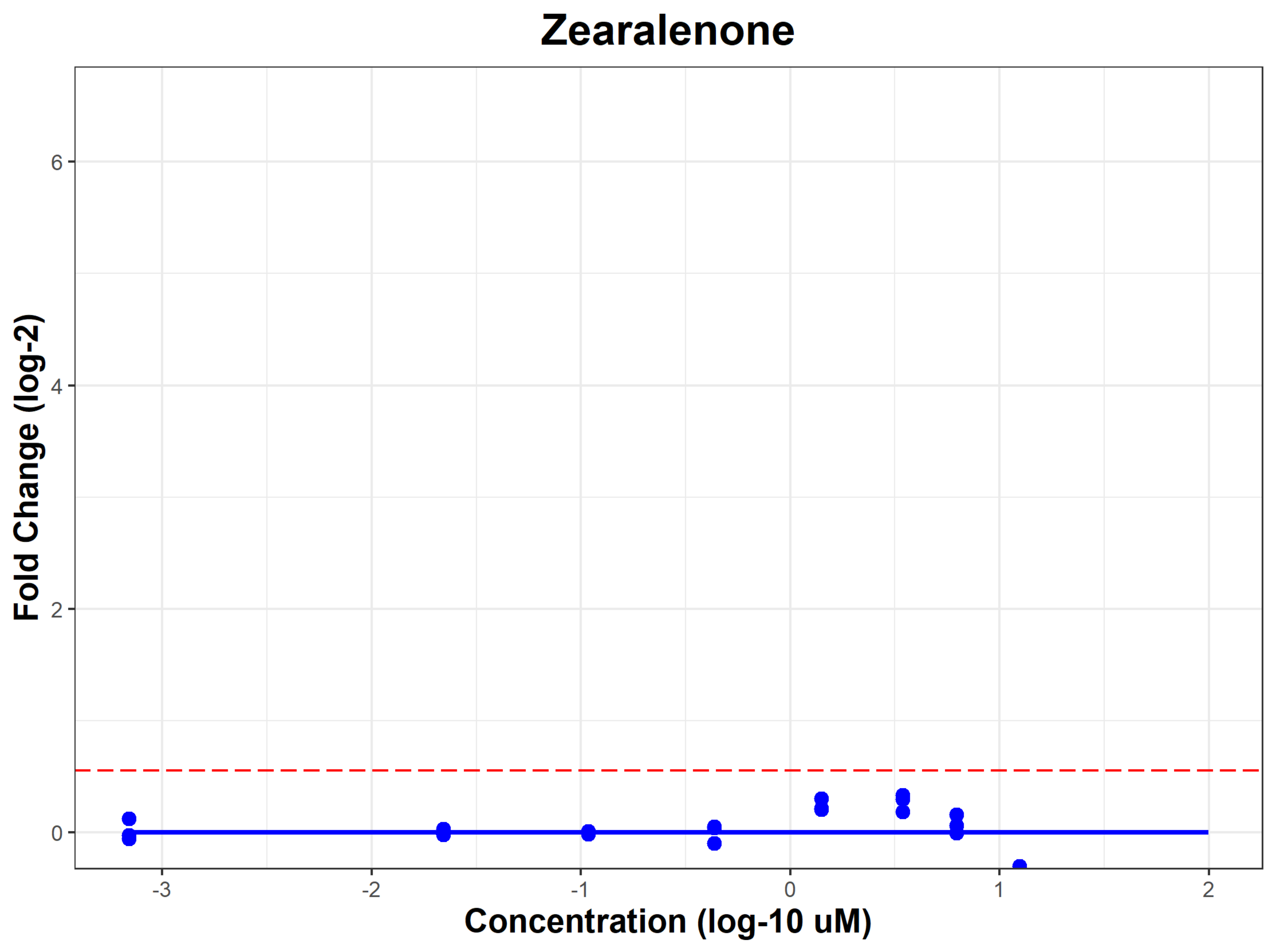

Zearalenone
Fold Change (log-2)
Concentration (log-10 uM)
6
4
2
0
-3
-2
-1
0
1
2

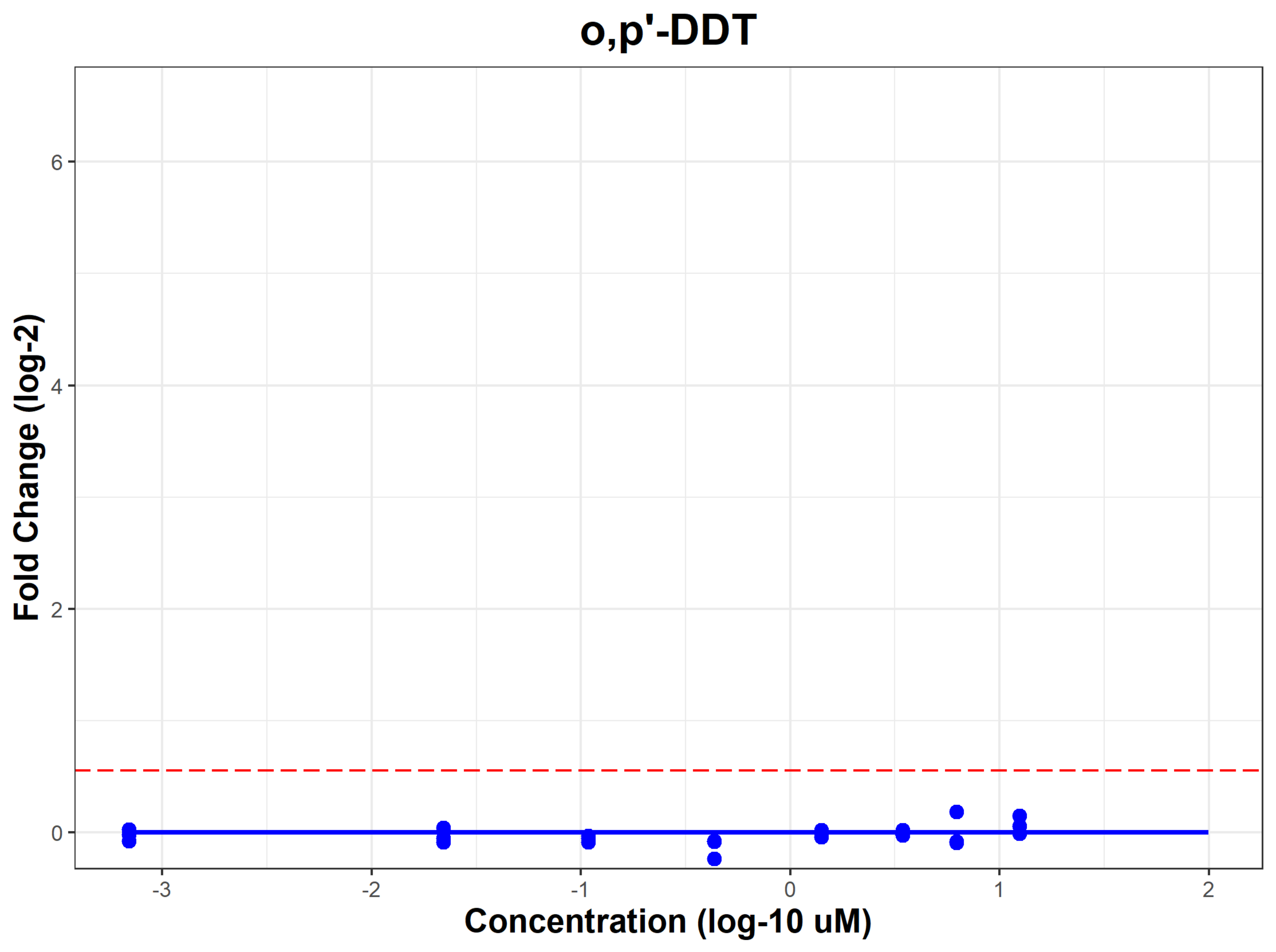

o,p'-DDT
Fold Change (log-2)
6
4
2
0
-3
-2
-1
0
1
2
Concentration (log-10 uM)

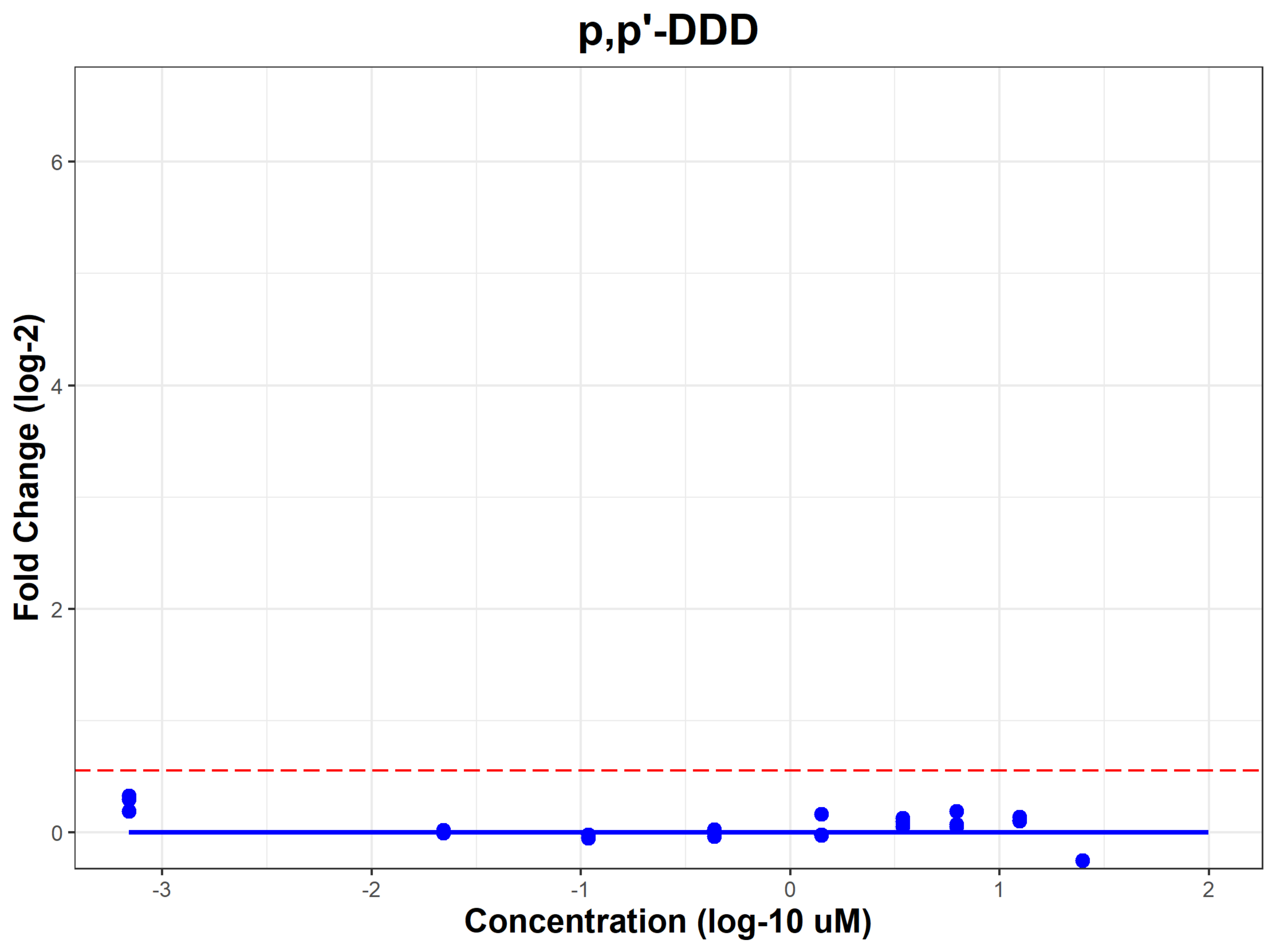

p,p'-DDD
Fold Change (log-2)
6
4
2
0
-3
-2
-1
0
1
2
Concentration (log-10 uM)

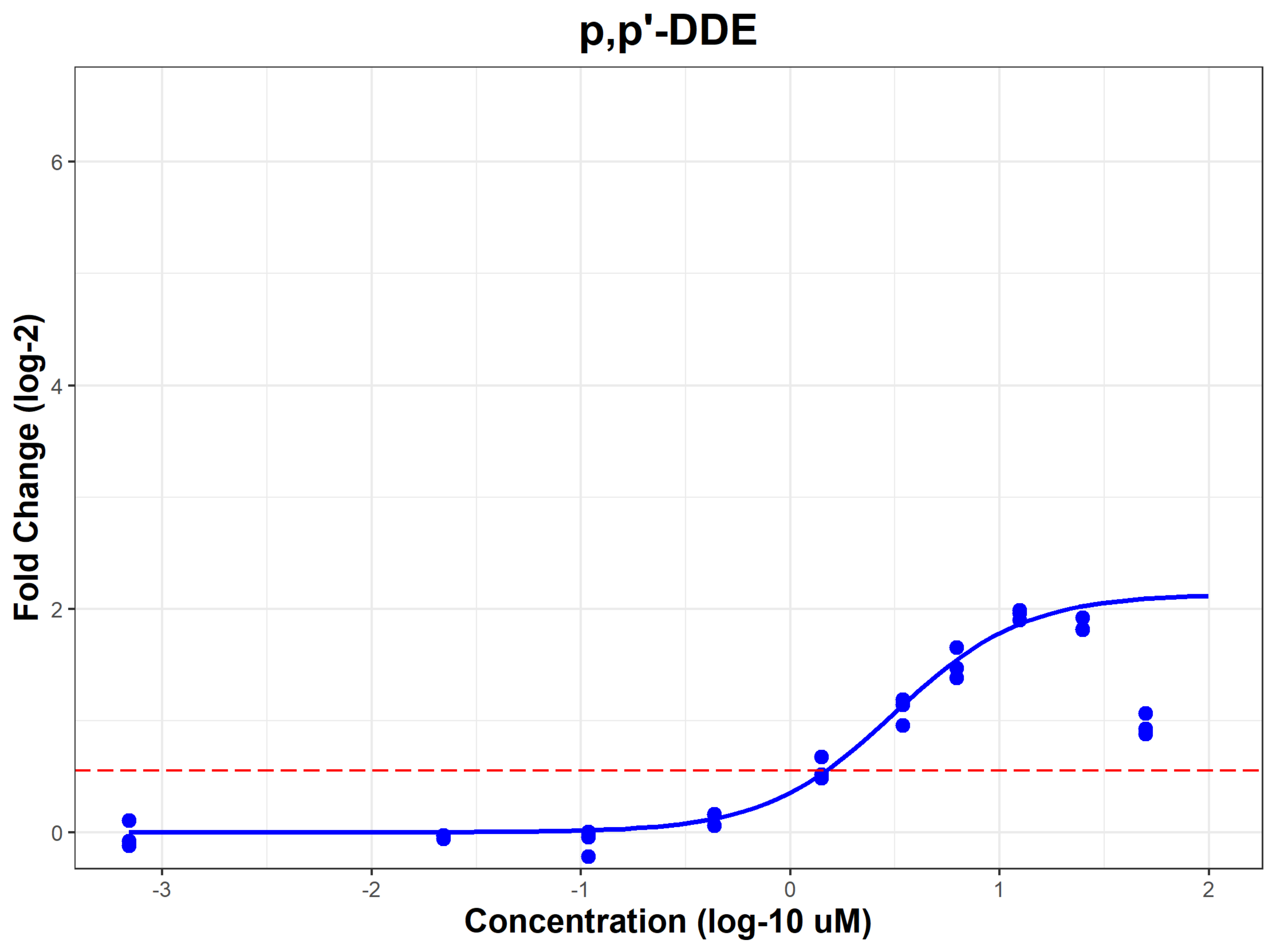
p,p'-DDE
Fold Change (log-2)
6
4
2
0
-3
-2
-1
0
1
2
Concentration (log-10 uM)

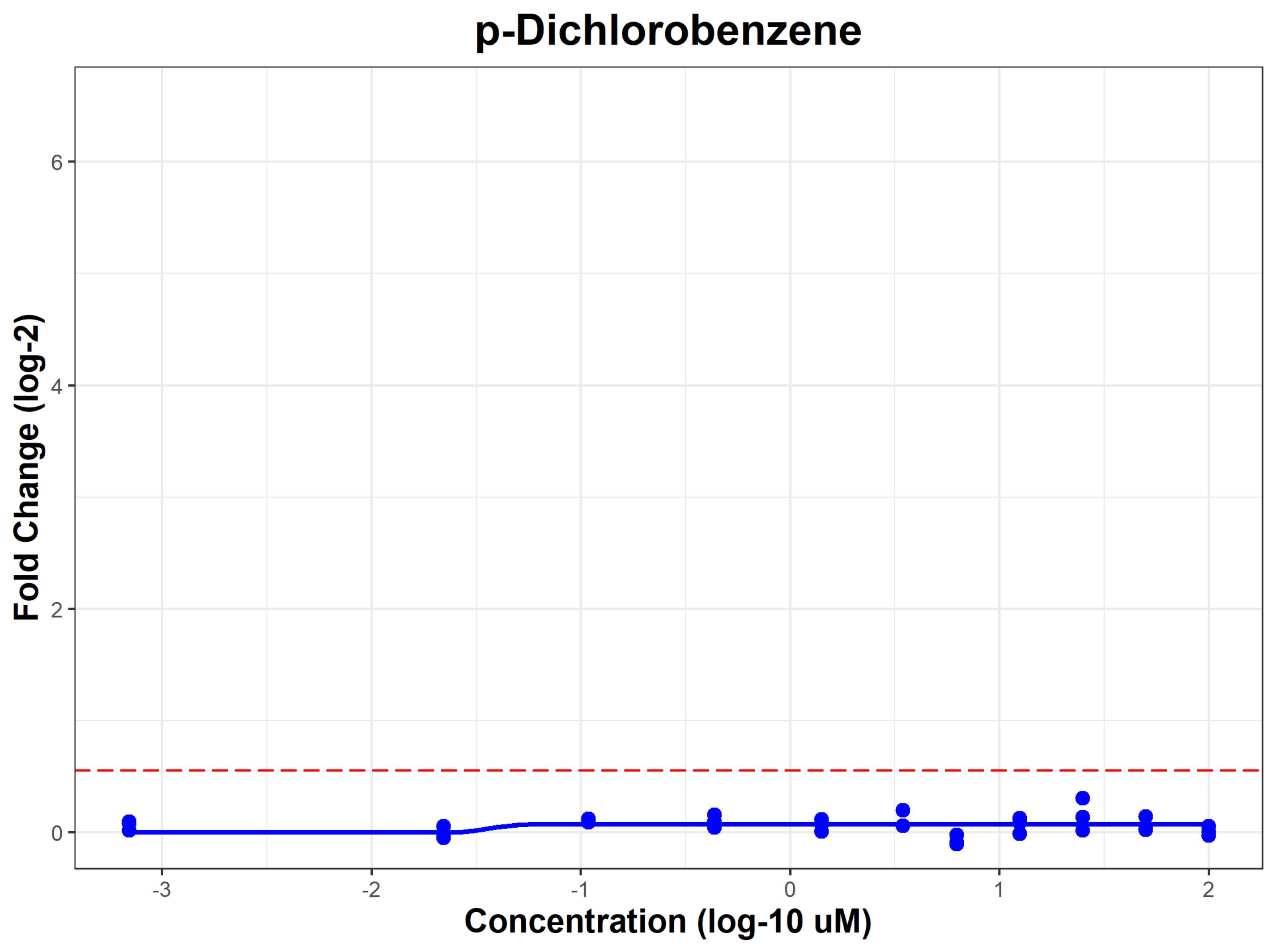
p-Dichlorobenzene
Fold Change (log-2)
6
4
2
0
-3
-2
-1
0
1
2
Concentration (log-10 uM)
